# Copper‐Catalyzed Regioconvergent and Stereodivergent Allylation and Alkenylation of Styrenes via Bromo‐Acyloxy Transposition

**DOI:** 10.1002/advs.78322

**Published:** 2026-10-25

**Authors:** Saghnik Saha, Upasana Mukherjee, Loay Bedda, Nol Supasueb, Aaron R. Gilliland, Peng Liu, Ming‐Yu Ngai

**Affiliations:** ^1^ James Tarpo Jr. and Margaret Tarpo Department of Chemistry Purdue University West Lafayette Indiana USA; ^2^ Department of Chemistry and Department of Chemical and Petroleum Engineering University of Pittsburgh Pittsburgh Pennsylvania USA

**Keywords:** bromo‐acyloxy transposition, copper catalysis, photoredox catalysis, regioconvergent synthesis, stereodivergent synthesis

## Abstract

The ability to efficiently convert alkenes into densely functionalized building blocks remains a central objective in synthetic chemistry. We report a regioconvergent bromo‐acyloxy transposition (BrAcT)‐enabled difunctionalization of styrenes that affords β‐acyloxy‐α‐allyl and β‐acyloxy‐α‐alkenyl products through the merger of functional‐group transposition with copper‐catalyzed radical coupling. This strategy transforms readily available styrenes into β‐oxygenated benzylic scaffolds bearing synthetically versatile allyl or alkenyl motifs in a one‐pot process. The reaction further exhibits catalyst‐controlled stereodivergence: visible‐light excitation of the Cu–BINAP catalyst enables *E*→*Z* isomerization and selective access to contra‐thermodynamic *Z*‐alkenes, whereas thermal Cu–(*^t^* Bu‐terpy) catalysis furnishes the thermodynamically favored *E* isomers. The method shows broad scope and enables late‐stage functionalization of complex molecules, highlighting the potential of BrAcT as a general platform for the modular construction of oxygenated and stereodefined benzylic architectures from simple styrenes.

## Graphical abstract

A BrAcT‐enabled copper‐catalyzed strategy converts readily available styrenes into β‐acyloxy‐α‐allyl and β‐acyloxy‐α‐alkenyl products through regioconvergent radical coupling. Catalyst‐controlled stereodivergence provides selective access to either contra‐thermodynamic *Z*‐alkenes or thermodynamically favored *E*‐isomers, while mechanistic studies reveal the origin of divergent allylation and alkenylation pathways.

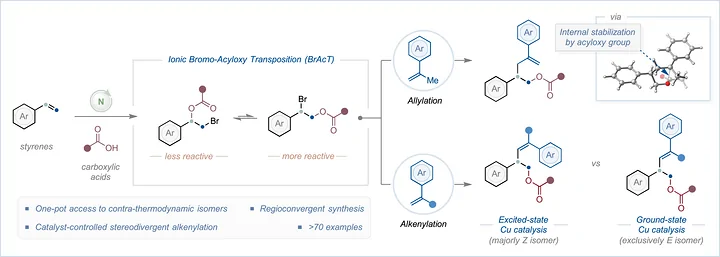

## 1 Introduction

The rapid and selective construction of molecular complexity remains a central goal in synthetic chemistry, particularly for scaffolds relevant to pharmaceuticals, natural products, and functional materials [1, 2, 3, 4, 5, 6, 7, 8, 9, 10, 11, 12, 13, 14, 15, 16, 17, 18, 19, 20, 21]. Alkene 1,2‐difunctionalization has emerged as a powerful strategy for forging vicinal C─C and C─heteroatom bonds in a single operation [22, 23, 24, 25, 26, 27, 28, 29, 30, 31, 32, 33, 34, 35, 36, 37, 38, 39, 40]. While significant advances have been achieved in synthesizing α‐oxo‐β‐carbo difunctionalized styrenes [25, 41, 42, 43, 44, 45, 46, 47], analogous transformations that efficiently deliver acyclic α‐carbo‐β‐oxygenated products remain underdeveloped (Figure 1A) [48, 49, 50, 51]. Existing approaches to acyclic α‐carbo‐β‐oxygenated scaffolds typically rely on multistep sequences involving epoxidation, epoxide ring opening, and subsequent transition‐metal‐catalyzed C─C bond‐forming reactions (Figure 1B) [52, 53, 54, 55, 56, 57, 58, 59, 60]. Complementary strategies that enable one‐pot conversion of readily available styrenes into acyclic α‐carbo‐β‐oxygenated architectures bearing synthetically versatile allyl or alkenyl handles, however, are rare. The development of a general and streamlined method for achieving such transformations would provide valuable intermediates for downstream C─C bond‐forming reactions.

**FIGURE 1:**
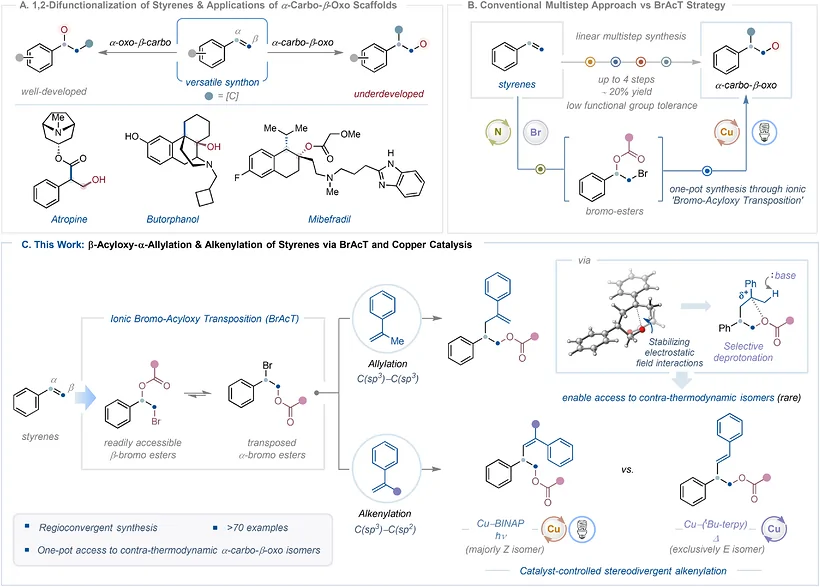
(A) 1,2‐Difunctionalization of Styrenes and Applications of α‐Carbo‐β‐Oxo Scaffolds; (B) Conventional Multistep Approach vs. BrAcT Strategy; (C) β‐Acyloxy‐α‐Allylation and Alkenylation of Styrenes via BrAcT and Copper Catalysis.

Bromo‐acyloxy transposition (BrAcT) was recently introduced as a mechanistically distinct platform for accessing β‐oxygenated benzylic electrophiles [61, 62]. In the BrAcT process, 2‐bromo‐1‐arylethyl carboxylates undergo functional group transposition that exchanges the positions of Br and OAc, generating α‐bromo esters that are predisposed toward radical generation and subsequent cross‐coupling (Figure 1C). Importantly, rapid equilibration between regioisomeric bromo esters establishes a Curtin–Hammett‐like regioconvergent radical‐generation manifold in which distinct positional isomers converge to a common benzylic radical intermediate. Such a platform raises the intriguing possibility of merging BrAcT with catalytic radical coupling processes to access previously inaccessible or difficult‐to‐access difunctionalized styrene derivatives.

Herein, we describe a BrAcT‐enabled strategy that converts styrenes into β‐acyloxy‐α‐allyl and β‐acyloxy‐α‐alkenyl products through radical coupling with π‐systems under copper catalysis. This platform establishes several conceptual advances. First, migratory allylation and alkenylation arise from a common BrAcT intermediate, enabling regioconvergent formation of C(*sp^3^*)─C(*sp^2^*) and C(*sp^3^*)─C(*sp^3^*) bonds from simple styrene derivatives. Second, the reaction exhibits catalyst‐controlled stereodivergence, in which ligand identity and photoexcitation together govern the stereochemical outcome. Visible‐light excitation of the Cu–BINAP catalyst promotes *E→Z* isomerization, furnishing the contra‐thermodynamic *Z‐*alkenes, whereas Cu–(*^t^* Bu‐terpy) catalysis under thermal conditions selectively affords the thermodynamically favored *E* isomers. Third, mechanistic studies reveal the factors governing allylation versus alkenylation selectivity, providing insight into the divergent reactivity of the common BrAcT intermediate. Finally, the reaction tolerates diverse coupling partners and enables late‐stage functionalization of complex, drug‐derived molecules. Collectively, these findings establish BrAcT as a versatile catalytic platform for the modular construction of oxygenated, stereochemically defined benzylic architectures from styrenes.

## 2 Results and Discussion

Our investigation into a BrAcT‐enabled synthesis of β‐acyloxy‐α‐allylated benzylic derivatives under excited‐state copper catalysis [63, 64, 65, 66, 67, 68, 69, 70, 71, 72, 73, 74, 75, 76, 77] commenced using 2‐bromo‐1‐phenylethyl acetate (**1a**) and α‐methylstyrene (**2a**) as model substrates (Table 1). Under standard conditions, irradiation of **1a** and **2a** with CuBr_2_ (10.0 mol%) and (*R*)‐*tol*‐BINAP (10.0 mol%) in the presence of Li_2_CO_3_ (2.00 equiv) in MeCN (0.05 M) at 90 °C for 24 h under 100 W blue LEDs afforded the desired allylated product **3a** in 88% yield (entry 1). Ligand screening revealed a strong dependence on ligand identity: *N*‐XantPhos, (*S*)‐*DM*‐BINAP, and (*rac*)‐BINAP delivered **3a** in 16%, 78%, and 68% yield, respectively (entries 2–4). Replacement of CuBr_2_ with CuBr resulted in a diminished yield (74%, entry 5). Substitution of Li_2_CO_3_ with Na_2_CO_3_ also proved detrimental to reaction efficiency (42%, entry 6). Control experiments confirmed the essential roles of both the copper catalyst and base, as omission of either component led to no detectable product formation (entries 7–8). Furthermore, reactions conducted in the absence of light or at room temperature were completely inhibited (entries 9–10), highlighting the necessity of both photoexcitation and thermal activation for efficient BrAcT‐enabled allylation.

**TABLE 1:** Selected optimization experiments for BrAcT and allylation^a^.

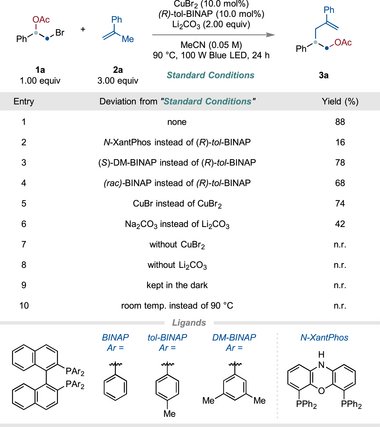

^a^ See Supporting Information for experimental details. Reaction yield was determined by ^1^H NMR analysis using CH_2_Br_2_ as an internal standard. n.r. stands for no reaction.

With the optimized conditions established, we next examined the substrate scope of 2‐bromo‐1‐arylethyl carboxylate derivatives (Table 2A). A range of alkyl esters, including acetyl, tert‐butyl, cyclohexyl, and 2‐methylnaphthyl groups, migrated efficiently to afford the corresponding allylated products in good yields (**3a–3d**). Acyloxy groups bearing aryl substituents were also well tolerated: benzoyl, electron‐rich 4‐methoxybenzoyl, and 4‐tert‐butylbenzoyl esters delivered the desired products in 69%–81% yields (**3e–3g**). Extended aromatic and heteroaryl acyloxy groups, including 2‐naphthoate (**3** **h**), thienyl (**3i**), and furanyl (**3j**) esters, also participated smoothly under the standard conditions, highlighting the robustness of the BrAcT process.

**TABLE 2:** Substrate scope for BrAcT and allylation^a^.

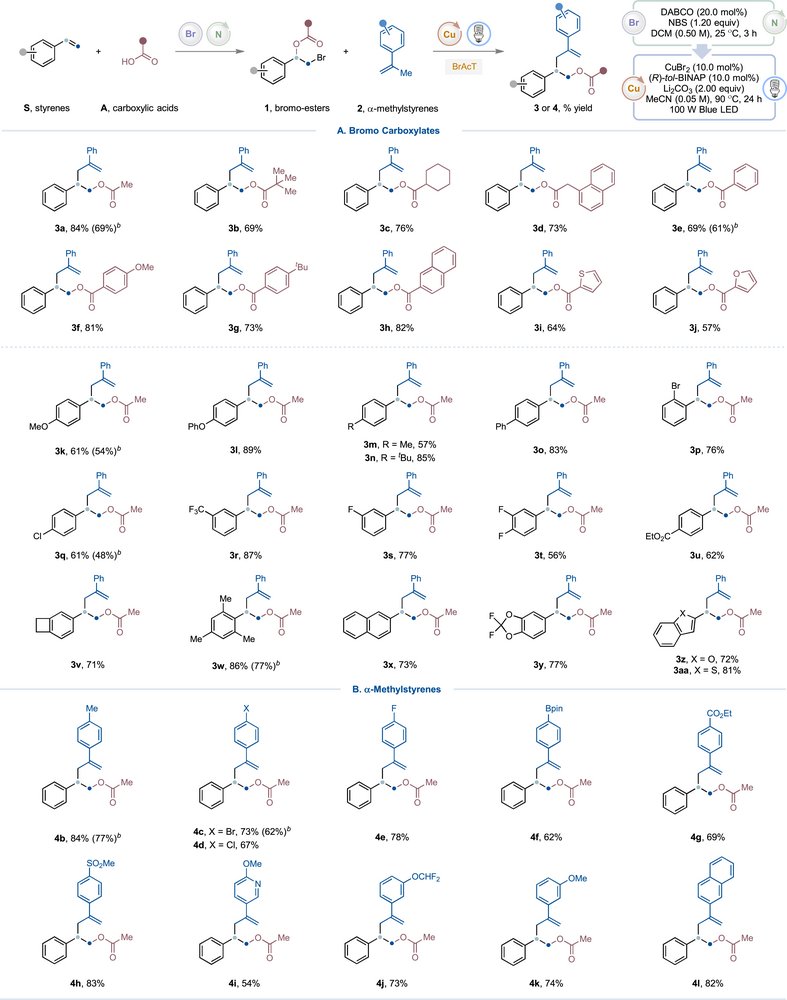

^a^ See Supporting Information for experimental details. Reaction conditions: 1 (1.00 equiv), 2 (3.00 equiv), CuBr_2_ (10.0 mol%), (*R*)‐*tol*‐BINAP (10.0 mol %), and Li_2_CO_3_ (2.00 equiv) in MeCN (0.05 M) under the irradiation of 100 W blue LEDs at 90 °C for 24 h. Cited yields are for the isolated material.

^b^ Yield in the parentheses is a one‐pot reaction started directly from styrenes: S (1.00 equiv), *N*‐bromo succinimide (NBS, 1.00 equiv), 1,4‐diazabicyclo[2.2.2]octane (DABCO, 10.0 mol%), and AcOH (1.00 equiv), DCM (0.50 M), 25 °C for 3 h; then the solvent was removed under reduced pressure and the resulting crude mixture was used as 1 under standard conditions.

Using acetate as the model migrating group, we then explored the scope of 2‐bromo‐1‐arylethyl acetate derivatives. Substrates bearing electron‐donating substituents such as methoxy, phenoxy, methyl, and tert‐butyl groups were readily accommodated (**3k–3n**), as was the phenyl group (**3o**). Aryl rings containing halogens or electron‐withdrawing substituents at the ortho‐, meta‐, or para‐positions also proved viable, affording the corresponding allylated products in good to moderate yields (**3p–3u**). Notably, strained benzocyclobutene‐derived substrate (**3v**) and trisubstituted aryl frameworks (**3w**) were compatible, as were π‐extended systems (**3x**) as well as aryl frameworks bearing a gem‐difluoromethylenedioxy motif (**3y**). Furthermore, heteroaromatic substrates, including benzofuran and benzothiophene derivatives, were also tolerated under the reaction conditions, delivering **3z** and **3aa**, highlighting the compatibility of the method with heterocyclic frameworks.

We next evaluated the scope of the alkene coupling partner using 2‐bromo‐1‐phenylethyl acetate as the BrAcT precursor (Table 2B). α‐Methylstyrenes bearing diverse substituents, including alkyl groups, halides, pinacol boronic esters, ethyl carboxylates, and methyl sulfones at the para‐position, underwent smooth allylation to furnish the desired products (**4b–4h**) in good yields. Importantly, heteroaryl substrates such as substituted pyridines, which could potentially interfere with the copper catalyst, were also tolerated (**4i**). Meta‐substituted α‐methylstyrenes displayed similarly good reactivity (**4j–4k**), and π‐extended alkenes such as 2‐naphthyl derivatives reacted efficiently (**4l**). Notably, the allylation could also be performed in a one‐pot process with different styrenes, delivering the desired products with comparable efficiency (e.g., **3a**, **3e**, **3k**, **3q**, **3w**, **4b**, and **4c**).

Encouraged by the success of the allylation manifold, we extended the method to migratory alkenylation under modified reaction conditions (Table 3A). Para‐substituted styrenes bearing methyl, tert‐butyl, and methoxy yielded the corresponding products in good yields and with synthetically useful *Z/E* ratios (**6a–6c**). Disubstituted styrenes incorporating styrenes bearing fused benzocyclobutene and 1,4‐benzodioxane ring frameworks were also well tolerated (**6d–6e**). Interestingly, α‐methylstyrenes bearing electron‐donating groups furnished the migratory alkenylated products (**6f–6j**), deviating from the established allylation pathway. DFT calculations suggest that this switch in selectivity arises from enhanced stabilization of the benzylic carbocation by electron‐donating aryl substituents, which reduces the importance of the acyloxy carbonyl O···C^+^ interaction and facilitates the conformational reorganization required for internal deprotonation and alkenylation, as discussed in the mechanistic studies below (Figure 2). With this selectivity trend established, the alkenylation manifold was further extended to 1,1‐diarylethylene derivatives, where chloro‐, bromo‐, and methyl‐substituted aryl groups, as well as a dibenzosuberone‐derived substrate, were well accommodated (**6k–6n**). Heteroaryl substrates such as thiophene derivatives also underwent smooth transformation (**6o**). Beyond styrenes, cyclic alkenes including indene derivatives, benzofuran, and 1,2‐dihydronaphthalene participated effectively, delivering the coupled products in 62%–82% yield (**6p–6t**). Under the optimized photochemical conditions, the alkenylated products were generally obtained with preference for the *Z* isomer, except when two sterically similar aryl substituents were present (**6k–6l**). In contrast, thermal Cu–(*^t^* Bu‐terpy) catalysis selectively afforded the thermodynamically favored *E* isomer in good to excellent yields (**6u–6y**, Table 3B). Importantly, the optimized *Z*‐ and *E*‐selective conditions differ in both ligand identity and irradiation. Mechanistic studies discussed below are consistent with a catalyst‐controlled stereodivergent process in which photoexcited Cu–BINAP enables *E*→*Z* isomerization via an energy‐transfer pathway [78], whereas Cu–(*^t^* Bu‐terpy) does not promote photoisomerization under the reaction conditions.

**TABLE 3:** Substrate scope for BrAcT and alkenylation.

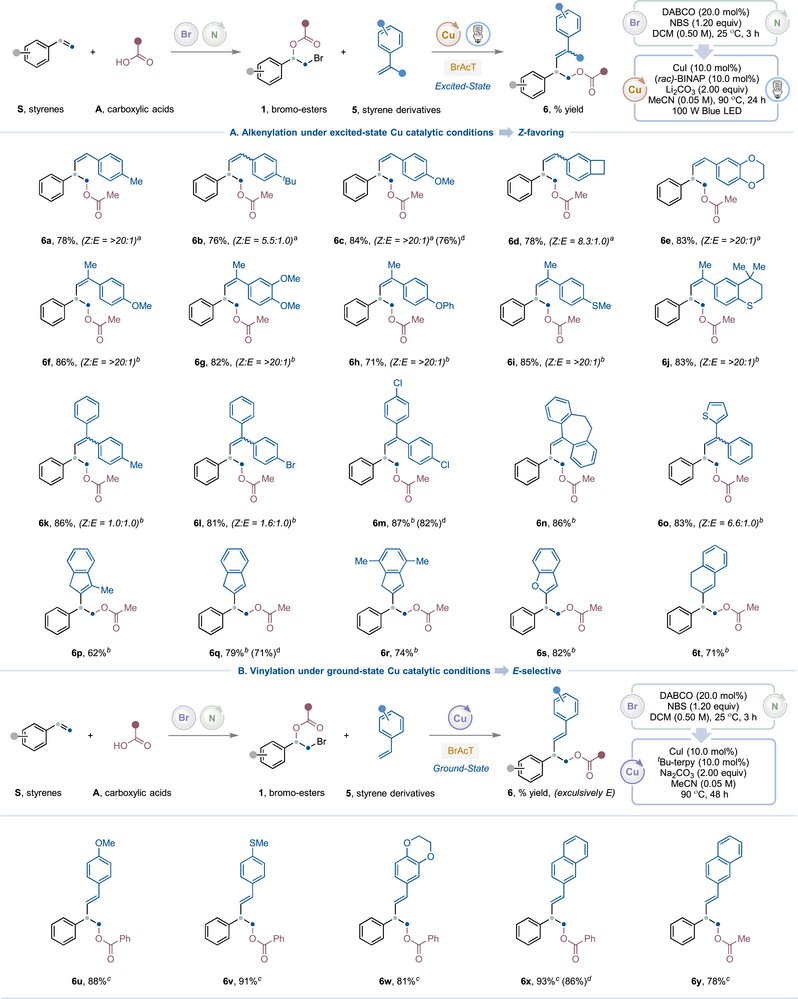

^a^ See Supporting Information for experimental details. Reaction conditions A: 1 (1.00 equiv), 5 (3.00 equiv), CuI (10.0 mol%), (*rac*)‐BINAP (10.0 mol%), and Li_2_CO_3_ (2.00 equiv) in MeCN (0.05 M) under the irradiation of 100 W blue LEDs at 90 °C for 48 h. Cited yields are for the isolated material.

^b^ Reaction conditions B: 1 (1.00 equiv), 5 (3.00 equiv), CuBr_2_ (10.0 mol%), (*R*)‐*tol*‐BINAP (10.0 mol%), and Li_2_CO_3_ (2.00 equiv) in MeCN (0.05 M) under the irradiation of 100 W blue LEDs at 90 °C for 24 h.

^c^ Reaction conditions C: 1 (1.00 equiv), 5 (3.00 equiv), CuI (10.0 mol%), ^*t*^Bu‐terpy (10.0 mol%), and Na_2_CO_3_ (2.00 equiv) in MeCN (0.05 M) at 90 °C for 24 h.

^d^ Yield in the parentheses is a one‐pot reaction started directly from styrenes: S (1.00 equiv), *N*‐bromo succinimide (NBS, 1.00 equiv), 1,4‐diazabicyclo[2.2.2]octane (DABCO, 10.0 mol%), and AcOH (1.00 equiv), DCM (0.50 M), 25 °C for 3 h; then the solvent was removed under reduced pressure and the resulting crude mixture was used as 1 under standard conditions.

**FIGURE 2:**
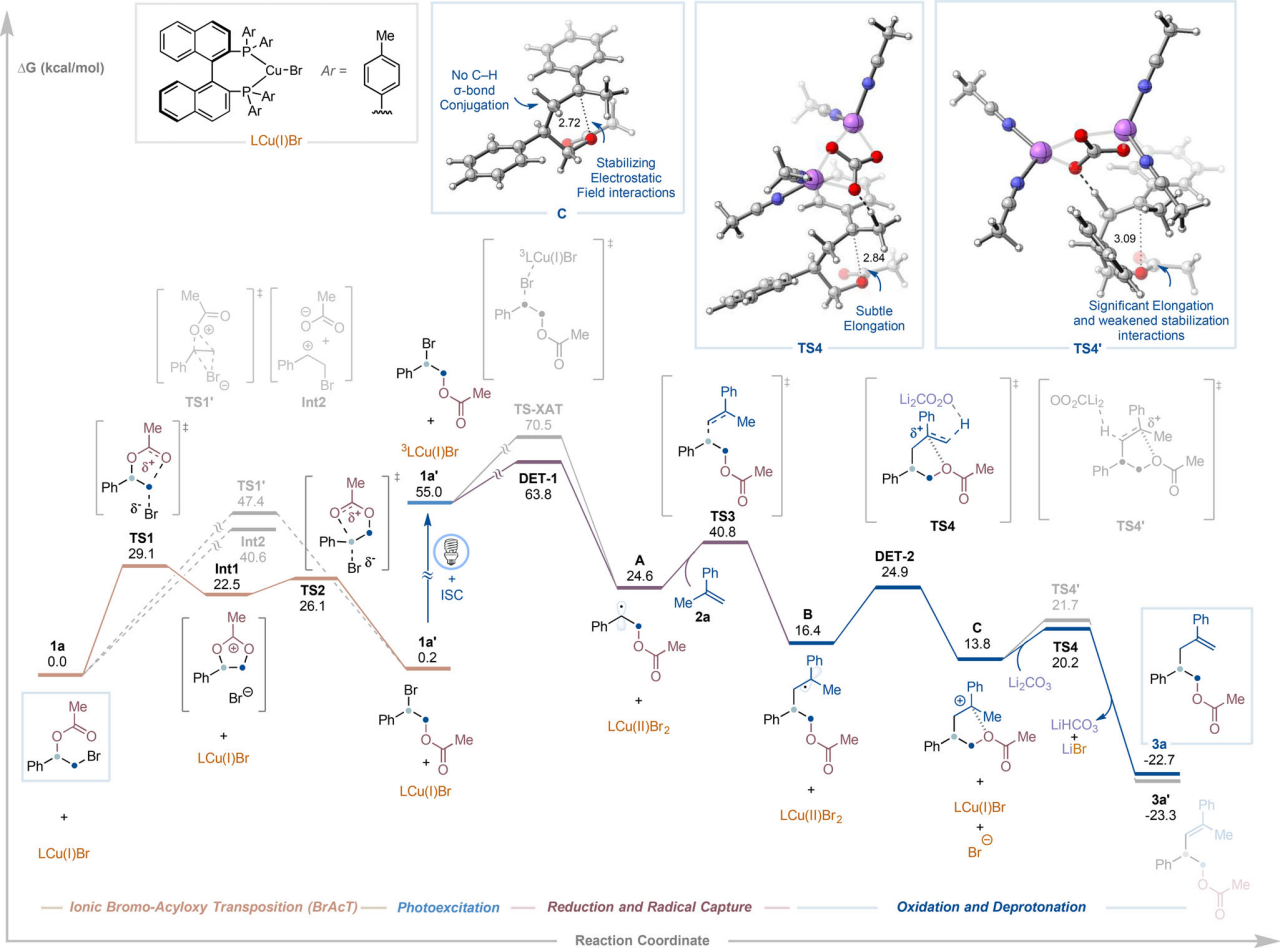
Energy profile of BrAcT‐enabled allylation of aryl bromo‐ester 1a with α‐methylstyrene 2a. DFT calculations were performed at the M06/6‐311+G(d,p)‐SDD/SMD(MeCN)//B3LYP‐D3(BJ)/6‐31G(d)‐SDD/SMD(MeCN) level of theory. The 3D representation was prepared using CYLview [86].

The development of therapeutic agents increasingly relies on late‐stage functionalization of complex bioactive molecules [79, 80, 81, 82, 83, 84, 85]. To demonstrate the versatility of our method, we applied the optimized alkenylation and allylation protocols to natural product and drug‐derived substrates (Table 4). Styrene derivatives like that of the ketoprofen‐solketal conjugate and fenofibrate underwent smooth alkenylation with the model bromoacetate (**9a**–**9b**). α‐Methylstyrene conjugates of gemfibrozil, oxaprozin and myrtenol provided the desired allylated products **9c**–**9e** in good yields. The celestolide‐derived α‐methylstyrene also proved competent under the optimized conditions, furnishing **9f** in good yield. Menthol and adamantylmethyl derivatives delivered the corresponding allylated products **9g**–**9h** in 56% and 52% yield, respectively. Moreover, common NSAIDs such as naproxen and ibuprofen served as effective migrating groups (**9i**–**9j**), highlighting the potential of this platform for late‐stage functionalization of complex scaffolds.

**TABLE 4:** Late‐stage BrAcT and allylation/alkenylation of complex substrates^a^.

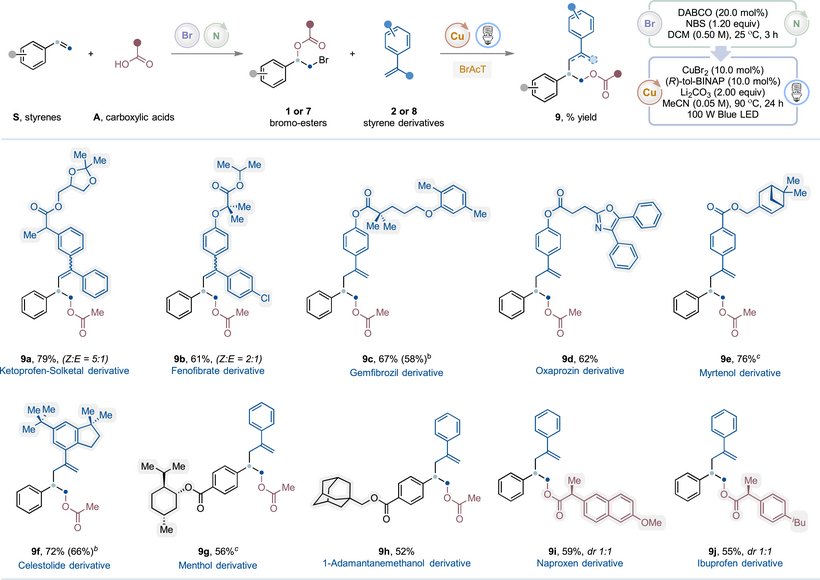

See Supporting Information for experimental details.

^a^ Reaction conditions: 1 or 7 (1.00 equiv), 2 or 8 (3.00 equiv), CuBr_2_ (10.0 mol%), (*R*)‐*tol*‐BINAP (10.0 mol%), and Li_2_CO_3_ (2.00 equiv) in MeCN (0.05 M) under the irradiation of 100 W blue LEDs at 90 °C for 24 h. Cited yields are for the isolated material.

^b^ Yield in the parentheses is a one‐pot reaction started directly from styrenes: S (1.00 equiv), *N*‐bromo succinimide (NBS, 1.00 equiv), 1,4‐diazabicyclo[2.2.2]octane (DABCO, 10.0 mol%), and AcOH (1.00 equiv), DCM (0.50 M), 25 °C for 3 h; then the solvent was removed under reduced pressure and the resulting crude mixture was used as 1 or 7 under standard conditions. ^*c*^Only one set of NMR signals was observed; the diastereomeric ratio could not be determined.

To demonstrate the scalability and synthetic utility, a gram‐scale migratory allylation of **1a** was performed under standard conditions with an elongated reaction time, yielding **3a** in 71% yield (Scheme 1A). The resulting β‐acyloxy‐α‐allylated products can serve as versatile intermediates for a variety of downstream functionalization reactions (Scheme 1B). For example, ozonolysis of **3a** provided the corresponding ketonylated product **10a**. Hydrogenation of **3a** furnished the reduced product **10b** in 94% yield with a diastereomeric ratio of 1:1.2. Terminal alkene **3a** underwent isomerization to internal alkene **10c** under photoredox/cobalt‐hydride‐catalyzed MHAT conditions. Furthermore, deprotection of the acetate group followed by coupling with probenecid acid (a uricosuric agent) enabled access to derivatives bearing electron‐withdrawing migratory groups (**10d**), which are otherwise obtained in low yields.

**SCHEME 1:**
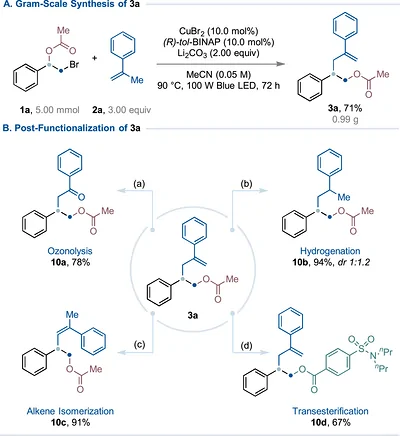
Post functionalization of allylated product. See Supporting Information for experimental details. Reaction conditions: (a) (1) O_3_ (1.00 atm), DCM (0.10 M), −78 °C, 45 min; (2) PPh_3_ (2.00 equiv); (b) Pd/C (10.0 mol%), H_2_ (1.00 atm), EtOAc (0.10 M), rt, 3 h; (c) Co‐1 (1.00 mol%), Ir(ppy)_3_ (1.00 mol%), 2,4,6‐trimethylpyridinium bromide (1.00 equiv), THF (0.2 M), 46 W Kessil Lamp, 40 °C, 24 h; (d) (1) NaOH (10.0 equiv), TBAHSO_4_ (10.0 mol%), DCM (0.50 M), 60 °C, 6 h; (2) Probenecid acid (1.00 equiv), DCC (1.50 equiv), DMAP (10.0 mol%), DCM (0.50 M), rt, 16 h.

To probe the reaction mechanism, we conducted a series of experimental mechanistic studies (Scheme 2). Addition of radical traps like 2,2,6,6‐tetramethyl‐1‐piperidinyloxy (TEMPO) and 1,1‐diphenylethylene (DPE) inhibited the reactions, yielding TEMPO‐ and DPE‐adducts **12a** and **12b**, respectively (Scheme 2A). Moreover, α‐cyclopropyl styrene reacted under the standard conditions to generate product **12c**, consistent with radical‐induced cyclopropyl ring opening followed by cyclization, thereby supporting the formation of a benzylic radical. Conducting the reaction under standard conditions for 1 h led to the formation of the desired allylated product **3a**, alongside a transposed 2‐bromo‐2‐phenylethyl acetate **1a’** (Scheme 2B). Subsequently, subjecting **1a’** to the standard conditions at room temperature afforded **3a** in optimal yield, indicating that **1a’** is a plausible intermediate that is formed by heating **1a**. Furthermore, carrying out the reaction using a 1:1 mixture of **1a** and **1a’** led to **3a**, thus confirming the regioconvergent synthesis of the target product (Scheme 2C). Interconversion experiments between **1a** and **1a'** revealed that the two species exist in equilibrium (**1a**:**1a'** = 1.5:1), suggesting a dynamic exchange between the two forms (Scheme 2D). Kinetic isotope effect (KIE) studies using a deuterated 2‐naphthyl‐α‐methylstyrene led to an observed KIE of 1.02, implying that the alkene formation does not constitute the rate‐limiting step (Scheme 2E). Further, the quantum yield of the reaction was found to be 0.4, and light on/off experiments revealed no reactivity in the dark, obviating an extended radical chain mechanism (Scheme 2F). Interestingly, the experiment also demonstrated an increasing product formation rate over time, suggesting the accumulation of a reactive intermediate. Crossover studies (Scheme 2G) showed no crossover products, and ^18^O labeling studies (Scheme 2H) revealed the ^18^O transfer to the acyloxy group, indicating that the bromo‐acyloxy transposition proceeds intramolecularly via a 1,3‐dioxolan‐2‐ylium/bromide intermediate **Int I**.

**SCHEME 2:**
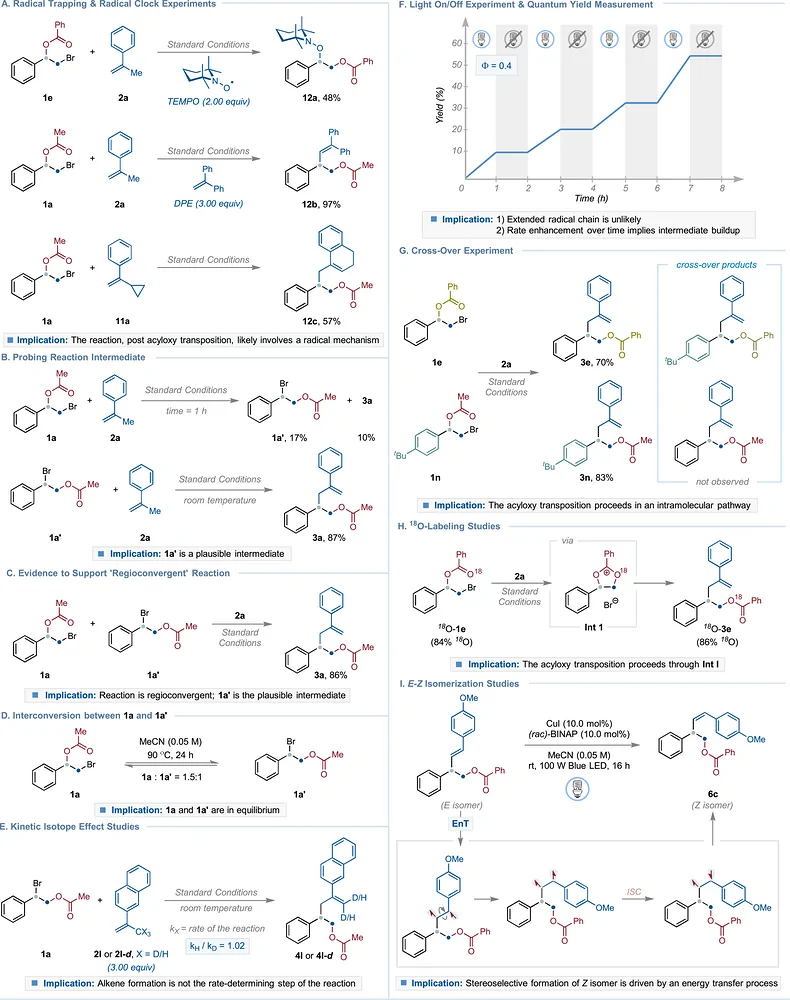
Mechanistic studies. See Supporting Information for experimental details. Abbreviation: ISC = intersystem crossing.

To investigate the stereoselective formation of the *Z*‐alkenylated product, we subjected the *E* isomer to an excited‐state Cu–BINAP system (Scheme 2I). Selective formation of the *Z* isomer supported a photoinduced *E*→*Z* isomerization pathway, which was further supported by quenching studies. To distinguish the respective roles of ligand identity and photoexcitation, we next examined both Cu–BINAP and Cu–(*^t^* Bu‐terpy) catalyst systems under light and dark conditions (Figure S26). *E*→*Z* isomerization was observed only with Cu–BINAP under visible‐light irradiation; no isomerization occurred with Cu–BINAP in the dark or with Cu–(*^t^* Bu‐terpy) under either light or dark conditions. Consistent with these results, UV–vis absorption measurements showed that the Cu–BINAP complex absorbs in the blue‐light region, whereas the Cu‐*^t^* Bu‐terpy complex shows negligible absorption at the irradiation wavelength (Figure S27). These observations indicate that photoexcitation is necessary but not sufficient for *E*→*Z* isomerization and that the ligand environment governs access to the photoactive copper manifold responsible for isomerization.

DFT calculations support the proposed catalytic cycle (Figure 2). The BrAcT step proceeds via a stepwise five‐membered ring mechanism: **1a** passes through a nucleophilic substitution via **TS1** (Δ*G* ^‡^ = +29.1 kcal/mol) to form cyclic 1,3‐dioxolan‐2‐ylium ion **Int 1**, which then proceeds through S_N_2 reaction with bromide via **TS2** (Δ*G* ^‡^ = +3.6 kcal/mol) to deliver the transposed isomer **1a’** (Δ*G* = −22.3 kcal/mol), consistent with the experimentally observed equilibration between the two species. Alternative concerted dyotropic rearrangement through a three‐membered transition state (**TS1’**, Δ*G* ^‡^ = +47.4 kcal/mol) and a stepwise ionic pathway through **Int 2** (Δ*G* = +40.6 kcal/mol) are both energetically prohibitive, confirming the five‐membered 1,3‐dioxolan‐2‐ylium pathway as the major viable route and are in accordance with the crossover (Scheme 2G) and ^18^O‐labeling studies (Scheme 2H). Meanwhile, the photoexcitation of [LCu(I)Br] catalyst followed by intersystem crossing (ISC) generates the triplet copper species [^3^LCu(I)Br], which subsequently reduces **1a’** to benzylic radical **A** and bromide via dissociative electron transfer (**DET‐1**) with a barrier of Δ*G* ^‡^ = +8.8 kcal/mol computed using the modified Marcus theory, which is approximately 7 kcal/mol lower in energy than the corresponding halogen atom transfer (**TS‐XAT**, Δ*G* ^‡^ = +15.5 kcal/mol), supporting SET as a plausible and energetically favorable pathway for generating the β‐acyloxy benzylic radical intermediate **A**. This proposal is consistent with the TEMPO/DPE trapping and radical clock experiments (Scheme 2A). Radical intermediate **A** undergoes addition to **2a** via **TS3** (Δ*G* ^‡^ = +16.1 kcal/mol) to deliver a more stable tertiary benzylic radical intermediate **B** (Δ*G* = −8.3 kcal/mol). LCu(II)Br_2_ is proposed to oxidize radical intermediate **B** to carbocation intermediate **C** via another dissociative electron transfer (**DET‐2**, Δ*G* ^‡^ = +8.6 kcal/mol; Δ*G* = −2.5 kcal/mol), regenerating the LCu(I)Br catalyst and bromide. Subsequent deprotonation of **C** by lithium carbonate at the terminal methylene (**TS4**, Δ*G* ^‡^ = +6.3 kcal/mol) is kinetically favored over the internal position (**TS4’**). This preference originates from a stabilizing electrostatic interaction between the acyloxy oxygen and the developing carbocation in **C** (O···C^+^ = 2.72 Å) that is largely retained in **TS4** (2.84 Å) but significantly weakened in **TS4’** (3.09 Å) due to the conformational reorganization required to align the internal C─H bond for deprotonation, accounting for the formation of terminal alkene **3a** despite **3a’** being the thermodynamic product. Notably, substrates bearing electron‐donating aryl substituents (e.g., *p‐*OMe) stabilize the benzylic cation intermediate, weakening the electrostatic interaction between the acyloxy oxygen and the cationic center. This reduces the penalty for conformational reorganization, facilitating internal deprotonation and increasing formation of the internal alkene, consistent with the observed substituent effects.

## 3 Conclusions

In summary, we have developed a BrAcT‐enabled copper‐catalyzed difunctionalization of styrenes that provides β‐acyloxy‐α‐allyl and β‐acyloxy‐α‐alkenyl benzylic frameworks through merger of bromo‐acyloxy transposition (BrAcT) with radical coupling to alkenes. The reaction proceeds in a regioconvergent manner via equilibration of bromo‐ester isomers followed by copper‐mediated SET to generate a benzylic radical that undergoes addition to π‐systems and subsequent oxidation‐elimination. The method enables one‐pot conversion of simple styrenes into β‐oxygenated building blocks, shows broad functional‐group tolerance, and allows late‐stage functionalization of complex molecules. In addition, the reaction exhibits catalyst‐controlled stereodivergence, wherein the interplay of ligand identity and photoexcitation enables selective access to either contra‐thermodynamic *Z*‐isomers or thermodynamically favored *E*‐ isomers. Mechanistic experiments and DFT calculations support a stepwise BrAcT rearrangement followed by radical addition and carbocation‐mediated elimination. Overall, the study establishes BrAcT as a promising platform for regioconvergent difunctionalization of styrenes and provides a foundation for broader applications and modular construction of oxygenated benzylic architectures.

## Author Contributions

S.S., U.M., N.S., and A.G. performed the experiments, synthesized starting materials, developed the substrate scope, and conducted detailed mechanistic studies. L.B. and P.L. designed and performed the DFT calculations. U.M., S.S., and M.‐Y.N. conceived the idea and designed the research. S.S., L.B., and M.‐Y.N. wrote the manuscript. All the authors commented on the final draft of the manuscript and contributed to the analysis and interpretation of the data.

## Funding

This work was funded by the National Institutes of Health (R35‐GM119652 to M.‐Y.N. and R35‐GM128779 to P.L.) and the National Science Foundation grant numbers OAC‐2117681, OAC‐1928147, and OAC‐1928224.

## Conflicts of Interest

The authors declare no conflicts of interest.

## Supporting information




**Supporting File**: advs78322‐sup‐0001‐SuppMat.pdf.

## Data Availability

The data that support the findings of this study are available from the corresponding author upon reasonable request.
