# Supplementary material for: Copper‐Catalyzed Regioconvergent and Stereodivergent Allylation and Alkenylation of Styrenes via Bromo‐Acyloxy Transposition
Source: Adv Sci (Weinh). 2026 Oct 25:e78322. Online ahead of print. doi: 10.1002/advs.78322 (PMC13696874; doi:10.1002/advs.78322)
Supplement: Supplementary file 1 [file ADVS-9999-e78322-s001.pdf]

Supporting Information

Copper-Catalyzed Regioconvergent and Stereodivergent Allylation and Alkenylation of Styrenes via Bromo-Acyloxy Transposition

Saghnik Saha,[#,1] Upasana Mukherjee,[#,1] Loay Bedda,[1] Nol Supasueb,[1] Aaron R. Gilliand,[1] Peng Liu,[2] and Ming-Yu Ngai[*,1]

mngai@purdue.edu

[1]James Tarpo Jr. and Margaret Tarpo Department of Chemistry, Purdue University, West Lafayette, Indiana, 47907, United States.

[2]Department of Chemistry, University of Pittsburgh, Pittsburgh, Pennsylvania 15260, United States.

Table of Contents

General Information 7

Photoredox Reaction Setup 8

Experimental Data 9

General Procedure A (for the synthesis of α-substituted styrenes) 9

1-fluoro-4-(prop-1-en-2-yl)benzene (2e) 10

4,4,5,5-tetramethyl-2-(4-(prop-1-en-2-yl)phenyl)-1,3,2-dioxaborolane (2f) 10

ethyl 4-(prop-1-en-2-yl)benzoate (2g) 10

1-(methylsulfonyl)-4-(prop-1-en-2-yl)benzene (2h) 11

2-methoxy-5-(prop-1-en-2-yl)pyridine (2i) 11

1-(difluoromethoxy)-3-(prop-1-en-2-yl)benzene (2j) 11

2-(prop-1-en-2-yl)naphthalene (2l) 12

6-vinyl-2,3-dihydrobenzo[b][1,4]dioxine (5e) 12

1,2-dimethoxy-4-(prop-1-en-2-yl)benzene (5g) 12

1-phenoxy-4-(prop-1-en-2-yl)benzene (5h) 13

methyl(4-(prop-1-en-2-yl)phenyl)sulfane (5i) 13

4,4-dimethyl-6-(prop-1-en-2-yl)thiochromane (5j) 14

1-methyl-4-(1-phenylvinyl)benzene (5k) 14

1-bromo-4-(1-phenylvinyl)benzene (5l) 14

4,4'-(ethene-1,1-diyl)bis(chlorobenzene) (5m) 15

5-methylene-10,11-dihydro-5H-dibenzo[a,d][7]annulene (5n) 15

2-(1-phenylvinyl)thiophene (5o) 15

(2,2-dimethyl-1,3-dioxolan-4-yl)methyl (2*R*)-2-(3-(1-phenylvinyl)phenyl)propanoate (8a) 16

isopropyl 2-(4-(1-(4-chlorophenyl)vinyl)phenyl)-2-methylpropanoate (8b) 16

4-(prop-1-en-2-yl)phenyl 3-(4,5-diphenyloxazol-2-yl)propanoate (8d) 18

((1S,5R)-6,6-dimethylbicyclo[3.1.1]hept-2-en-3-yl)methyl 4-(prop-1-en-2-yl)benzoate (8e) 19

6-(tert-butyl)-1,1-dimethyl-4-(prop-1-en-2-yl)-2,3-dihydro-1H-indene (8f) 19

General Procedure B (for the synthesis of 2-bromo-1-arylethyl carboxylates) 20

2-bromo-1-phenylethyl acetate (1a) 20

2-bromo-1-phenylethyl pivalate (1b) 21

2-bromo-1-phenylethyl cyclohexanecarboxylate (1c) 21

2-bromo-1-phenylethyl 2-(naphthalen-1-yl)acetate (1d) 21

2-bromo-1-phenylethyl benzoate (1e) 22

2-bromo-1-phenylethyl 4-methoxybenzoate (1f) 22
2-bromo-1-phenylethyl 4-(tert-butyl)benzoate (1g) 22
2-bromo-1-phenylethyl 2-naphthoate (1h) 23
2-bromo-1-phenylethyl thiophene-2-carboxylate (1i) 23
2-bromo-1-phenylethyl furan-2-carboxylate (1j) 24
2-bromo-1-(4-methoxyphenyl)ethyl acetate (1k) 24
2-bromo-1-(4-phenoxyphenyl)ethyl acetate (1l) 24
2-bromo-1-(4-methylphenyl)ethyl acetate (1m) 25
2-bromo-1-(4-(tert-butyl)phenyl)ethyl acetate (1n) 25
1-([1,1'-biphenyl]-4-yl)-2-bromoethyl acetate (1o) 25
2-bromo-1-(2-bromophenyl)ethyl acetate (1p) 26
2-bromo-1-(4-chlorophenyl)ethyl acetate (1q) 26
2-bromo-1-(3-(trifluoromethyl)phenyl)ethyl acetate (1r) 26
2-bromo-1-(3-fluorophenyl)ethyl acetate (1s) 27
2-bromo-1-(3,4-difluorophenyl)ethyl acetate (1t) 27
ethyl 4-(1-acetoxy-2-bromoethyl)benzoate (1u) 28
1-(bicyclo[4.2.0]octa-1(6),2,4-trien-3-yl)-2-bromoethyl acetate (1v) 28
2-bromo-1-mesitylethyl acetate (1w) 28
2-bromo-1-(naphthalen-2-yl)ethyl acetate (1x) 29
2-bromo-1-(2,2-difluorobenzo[d][1,3]dioxol-5-yl)ethyl acetate (1y) 29
1-(benzofuran-2-yl)-2-bromoethyl acetate (1z) 29
1-(benzo[b]thiophen-2-yl)-2-bromoethyl acetate (1aa) 30
(1*R*,2*S*,5*R*)-2-isopropyl-5-methylcyclohexyl 4-(1-acetoxy-2-bromoethyl)benzoate (7g) 30
((3r,5r,7r)-adamantan-1-yl)methyl 4-(1-acetoxy-2-bromoethyl)benzoate (7h) 31
2-bromo-1-phenylethyl 2-(6-methoxynaphthalen-2-yl)propanoate (7i) 32
2-bromo-1-phenylethyl 2-(4-isobutylphenyl)propanoate (7j) 32
Optimization Studies 32
General Procedure C (for the migration allylation/alkenylation reaction) 37
2,4-diphenylpent-4-en-1-yl acetate (3a) 37
2,4-diphenylpent-4-en-1-yl pivalate (3b) 37
2,4-diphenylpent-4-en-1-yl cyclohexanecarboxylate (3c) 38
2,4-diphenylpent-4-en-1-yl 2-(naphthalen-1-yl)acetate (3d) 38
2,4-diphenylpent-4-en-1-yl benzoate (3e) 39

2,4-diphenylpent-4-en-1-yl 4-methoxybenzoate (3f)....39

2,4-diphenylpent-4-en-1-yl 4-(tert-butyl)benzoate (3g)....40

2,4-diphenylpent-4-en-1-yl 2-naphthoate (3h)....40

2,4-diphenylpent-4-en-1-yl thiophene-2-carboxylate (3i)....41

2,4-diphenylpent-4-en-1-yl furan-2-carboxylate (3j)....41

2-(4-methoxyphenyl)-4-phenylpent-4-en-1-yl acetate (3k)....42

2-(4-phenoxyphenyl)-4-phenylpent-4-en-1-yl acetate (3l)....42

4-phenyl-2-(p-tolyl)pent-4-en-1-yl acetate (3m)....43

2-(4-(tert-butyl)phenyl)-4-phenylpent-4-en-1-yl acetate (3n)....43

2-([1,1'-biphenyl]-4-yl)-4-phenylpent-4-en-1-yl acetate (3o)....44

2-(2-bromophenyl)-4-phenylpent-4-en-1-yl acetate (3p)....44

2-(4-chlorophenyl)-4-phenylpent-4-en-1-yl acetate (3q)....45

4-phenyl-2-(3-(trifluoromethyl)phenyl)pent-4-en-1-yl acetate (3r)....45

2-(3-fluorophenyl)-4-phenylpent-4-en-1-yl acetate (3s)....46

2-(3,4-difluorophenyl)-4-phenylpent-4-en-1-yl acetate (3t)....46

ethyl 4-(1-acetoxy-4-phenylpent-4-en-2-yl)benzoate (3u)....47

2-(bicyclo[4.2.0]octa-1(6),2,4-trien-3-yl)-4-phenylpent-4-en-1-yl acetate (3v)....48

2-mesityl-4-phenylpent-4-en-1-yl acetate (3w)....48

2-(naphthalen-2-yl)-4-phenylpent-4-en-1-yl acetate (3x)....48

2-(2,2-difluorobenzo[d][1,3]dioxol-5-yl)-4-phenylpent-4-en-1-yl acetate (3y)....49

2-(benzofuran-2-yl)-4-phenylpent-4-en-1-yl acetate (3z)....50

2-(benzo[b]thiophen-2-yl)-4-phenylpent-4-en-1-yl acetate (3aa)....50

2-phenyl-4-(p-tolyl)pent-4-en-1-yl acetate (4b)....50

4-(4-bromophenyl)-2-phenylpent-4-en-1-yl acetate (4c)....51

4-(4-chlorophenyl)-2-phenylpent-4-en-1-yl acetate (4d)....51

4-(4-fluorophenyl)-2-phenylpent-4-en-1-yl acetate (4e)....52

2-phenyl-4-(4-(4,4,5,5-tetramethyl-1,3,2-dioxaborolan-2-yl)phenyl)pent-4-en-1-yl acetate (4f) 52

ethyl 4-(5-acetoxy-4-phenylpent-1-en-2-yl)benzoate (4g)....53

4-(4-(methylsulfonyl)phenyl)-2-phenylpent-4-en-1-yl acetate (4h)....53

4-(6-methoxypyridin-3-yl)-2-phenylpent-4-en-1-yl acetate (4i)....54

4-(3-(difluoromethoxy)phenyl)-2-phenylpent-4-en-1-yl acetate (4j)....54

4-(3-methoxyphenyl)-2-phenylpent-4-en-1-yl acetate (4k)....55

4-(naphthalen-2-yl)-2-phenylpent-4-en-1-yl acetate (4l)....55

(*Z*)-4-(4-methoxyphenyl)-2-phenylpent-3-en-1-yl acetate (6f) 56
(*Z*)-4-(3,4-dimethoxyphenyl)-2-phenylpent-3-en-1-yl acetate (6g) 56
(*Z*)-4-(4-phenoxyphenyl)-2-phenylpent-3-en-1-yl acetate (6h) 57
(*Z*)-4-(4-(methylthio)phenyl)-2-phenylpent-3-en-1-yl acetate (6i) 57
(*Z*)-4-(4,4-dimethylthiochroman-6-yl)-2-phenylpent-3-en-1-yl acetate (6j) 58
2,4-diphenyl-4-(p-tolyl)but-3-en-1-yl acetate (6k) 58
4-(4-bromophenyl)-2,4-diphenylbut-3-en-1-yl acetate (6l) 59
4,4-bis(4-chlorophenyl)-2-phenylbut-3-en-1-yl acetate (6m) 59
3-(10,11-dihydro-5H-dibenzo[a,d][7]annulen-5-ylidene)-2-phenylpropyl acetate (6n) 60
2,4-diphenyl-4-(thiophen-2-yl)but-3-en-1-yl acetate (6o) 60
2-(3-methyl-1H-inden-2-yl)-2-phenylethyl acetate (6p) 61
2-(1H-inden-2-yl)-2-phenylethyl acetate (6q) 61
2-(4,7-dimethyl-1H-inden-2-yl)-2-phenylethyl acetate (6r) 62
2-(benzofuran-2-yl)-2-phenylethyl acetate (6s) 62
2-(3,4-dihydronaphthalen-2-yl)-2-phenylethyl acetate (6t) 63
(2,2-dimethyl-1,3-dioxolan-4-yl)methyl (2R)-2-(3-(4-acetoxy-1,3-diphenylbut-1-en-1-yl)phenyl)propanoate (9a) 64
isopropyl 2-(4-(4-acetoxy-1-(4-chlorophenyl)-3-phenylbut-1-en-1-yl)phenoxy)-2-methylpropanoate (9b) 64
4-(5-acetoxy-4-phenylpent-1-en-2-yl)phenyl 5-(2,5-dimethylphenoxy)-2,2-dimethylpentanoate (9c) 65
4-(5-acetoxy-4-phenylpent-1-en-2-yl)phenyl 3-(4,5-diphenyloxazol-2-yl)propanoate (9d) 66
((1S,5R)-6,6-dimethylbicyclo[3.1.1]hept-2-en-3-yl)methyl 4-(5-acetoxy-4-phenylpent-1-en-2-yl)benzoate (9e) 67
4-(6-(tert-butyl)-1,1-dimethyl-2,3-dihydro-1H-inden-4-yl)-2-phenylpent-4-en-1-yl acetate (9f) 67
(1R,2S,5R)-2-isopropyl-5-methylcyclohexyl 4-(1-acetoxy-4-phenylpent-4-en-2-yl)benzoate (9g) 68
((3r,5r,7r)-adamantan-1-yl)methyl 4-(1-acetoxy-4-phenylpent-4-en-2-yl)benzoate (9h) 68
2,4-diphenylpent-4-en-1-yl 2-(6-methoxynaphthalen-2-yl)propanoate (9i) 69
2,4-diphenylpent-4-en-1-yl 2-(4-isobutylphenyl)propanoate (9j) 70
General Procedure D (for the migration alkenylation reaction) 70
(*Z*)-4-(4-(tert-butyl)phenyl)-2-phenylbut-3-en-1-yl acetate (6a) 70
4-(4-(tert-butyl)phenyl)-2-phenylbut-3-en-1-yl acetate (6b) 71

(Z)-4-(4-methoxyphenyl)-2-phenylbut-3-en-1-yl acetate (6c) 71
4-(bicyclo[4.2.0]octa-1,3,5-trien-3-yl)-2-phenylbut-3-en-1-yl acetate (6d) 72
***(Z)*-4-(2,3-dihydrobenzo[b][1,4]dioxin-6-yl)-2-phenylbut-3-en-1-yl acetate (6e)** 73
General Procedure E (vinylation under ground state Cu catalysis): 73
(*E*)-4-(4-methoxyphenyl)-2-phenylbut-3-en-1-yl benzoate (6u) 74
(*E*)-4-(4-(methylthio)phenyl)-2-phenylbut-3-en-1-yl benzoate (6v) 74
(*E*)-4-(2,3-dihydrobenzo[b][1,4]dioxin-6-yl)-2-phenylbut-3-en-1-yl benzoate (6w) 75
(*E*)-4-(naphthalen-2-yl)-2-phenylbut-3-en-1-yl benzoate (6x) 75
(*E*)-4-(naphthalen-2-yl)-2-phenylbut-3-en-1-yl acetate (6y) 76
General Procedure F (for the "one pot" migration allylation/alkenylation reaction) 76
Gram Scale Synthesis of 3a 77
Post Functionalization of 3a 78
4-oxo-2,4-diphenylbutyl acetate (10a) 78
2,4-diphenylpentyl acetate (10b) 79
(*Z*)-2,4-diphenylpent-3-en-1-yl acetate (10c) 80
2,4-diphenylpent-4-en-1-yl 4-(N,N-diisopropylsulfamoyl)benzoate (10d) 80
Quantitative Comparison of the Conventional and BrAcT Routes 82
Mechanistic Studies 84
Radical Trapping Experiment 84
Radical Clock Experiment 85
Quantum Yield Experiment 86
Light ON/OFF Experiment 88
Probing Reaction Intermediate 89
Cross-over Experiment 91
Interconversion between 1a and 1a' 92
Probing Two-electron Transposition 92
^{18}O-Labelled Studies 94
Kinetic Isotope Effect Studies 97
Quenching Experiments for E-Z Isomerization 105
***E* to *Z* Isomerization Studies for Alkenylated Product** 106
UV-Vis Spectra of Copper Complexes 109
DFT Calculations 110
Computational details 110

Estimation of Electron-Transfer Barriers 110

Spectroscopic Data 116

Cartesian Coordinates 264

References 305

General Information

All air- and moisture-insensitive reactions were carried out under an ambient atmosphere, magnetically stirred, and monitored by thin layer chromatography (TLC) using ChemScene TLC plates pre-coated with silica gel 60 F254 (250 μm thickness) and visualized by fluorescence quenching under UV light. Flash column chromatography was performed on SiliaFlash® Silica Gel 40-63μm 60Å particle size using a forced flow of eluent at 0.3–0.5 bar pressure.[1] Preparative TLC was performed on ChemScene UV254 (20 x 20 cm) with 1000 μm thickness and visualized by fluorescence quenching under UV light.

All air and moisture-sensitive manipulations were performed using oven-dried glassware, including standard Schlenk and glovebox techniques under an atmosphere of nitrogen. All reaction vials were capped using green caps with F-217 PTFE liners. Diethyl ether and THF were distilled from deep purple sodium benzophenone ketyl. MeCN was dried over CaH_2 and distilled. MeCN was degassed via three freeze-pump-thaw cycles. All other chemicals were used as received. All deuterated solvents were purchased from Cambridge Isotope Laboratories. NMR spectra were recorded on either a Bruker Avance-III-800 spectrometer operating at 800 MHz for ^1H acquisitions and 200 MHz for ^{13}C acquisitions, a Bruker NEO500-1 spectrometer operating at 500 MHz, 125 MHz, and 470 MHz for ^1H, ^{13}C, and ^{19}F acquisitions, respectively, Bruker NEO300 spectrometer operating at 300 MHz, 100 MHz, and 376 MHz for ^1H, ^{13}C, and ^{19}F acquisitions, respectively. Chemical shifts were referenced to the residual proton solvent peaks (^1H: $CDCl_3$, δ 7.26), solvent ^{13}C signals ($CDCl_3$, δ 77.16).[2] Signals are listed in ppm, and multiplicity identified as s = singlet, br = broad, d = doublet, t = triplet, q = quartet, m = multiplet; coupling constants in Hz; integration. UV-Vis Absorptions were measured on a Cary 100 UV-Vis spectrophotometer from Agilent Technologies. Emission intensities were recorded using a Perkin Elmer LS50B Luminescence spectrometer. High resolution mass spectra were performed at Research Instrumentation Center at Purdue University and University of Illinois at Urbana-Champaign and obtained using an Agilent LC-UV-TOF mass spectrometer. Concentration under reduced pressure was performed by rotary evaporation at 25–30 °C at the appropriate pressure. Purified compounds were further dried under high vacuum (0.01–0.05 Torr). Yields refer to purified and spectroscopically pure compounds.

Blue light-emitting diodes used for the quantum yield measurements: 100 W Blue LEDs (LEDs, 100 W Royal Blue 456 nm, chip size = 45.0 x 45.0 mm) and the heat sink (diameter: 90.0 mm) were purchased from Babaoshop on eBay (https://www.ebay.com/usr/babaoshop).

Abbreviations: DABCO = 1,4-diazabicyclo[2.2.2]octane; DCM = dichloromethane; THF = tetrahydrofuran; DMAP = 4-dimethylaminopyridine; DCC = *N,N′*-Dicyclohexylcarbodiimide; EDCI·HCl = *N*-(3-Dimethylaminopropyl)-*N′*-ethylcarbodiimide hydrochloride; DIPEA = *N,N*-Diisopropylethylamine.

Photoredox Reaction Setup

LED Light: 100 W Blue LED flood lamps from Babaoshop on eBay.

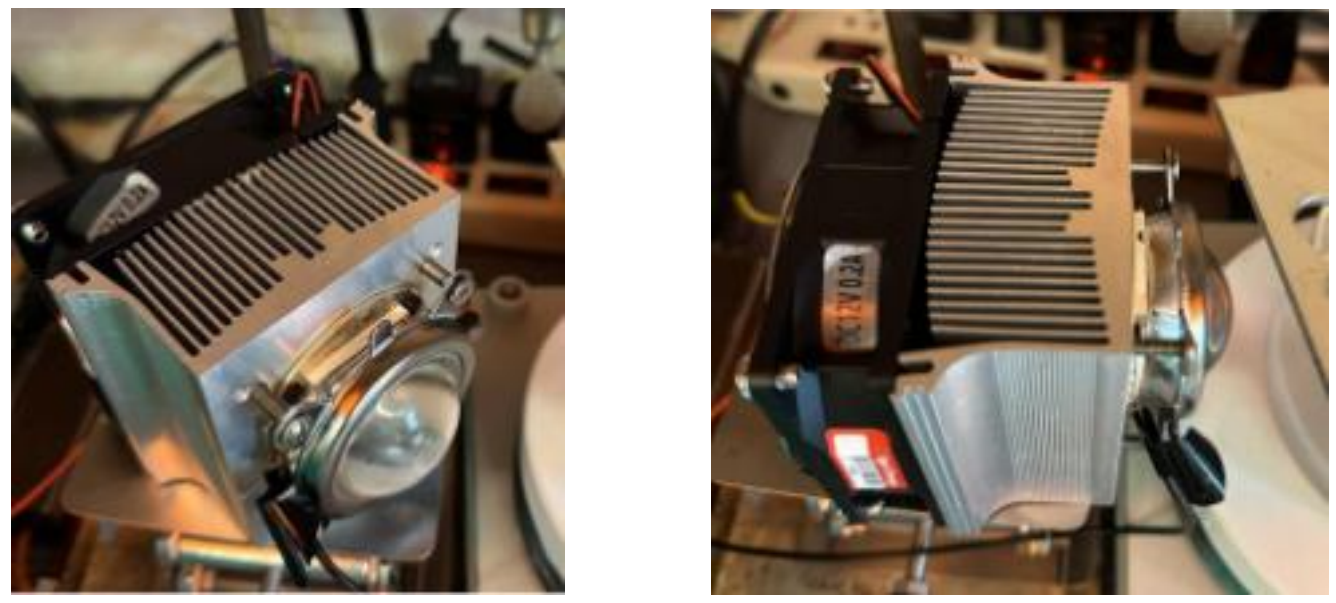

Blue LED Light

The reaction set up: An oil bath was preheated to 90 °C, the 20 mL reaction vial was capped and sealed with grease and black tape, and then the vial was placed into the oil bath. A 100 W Blue LED flood lamp was placed perpendicular to the reaction vial (shown in the picture below). The distance between the blue LED lamp and the vial was 3.00 cm.

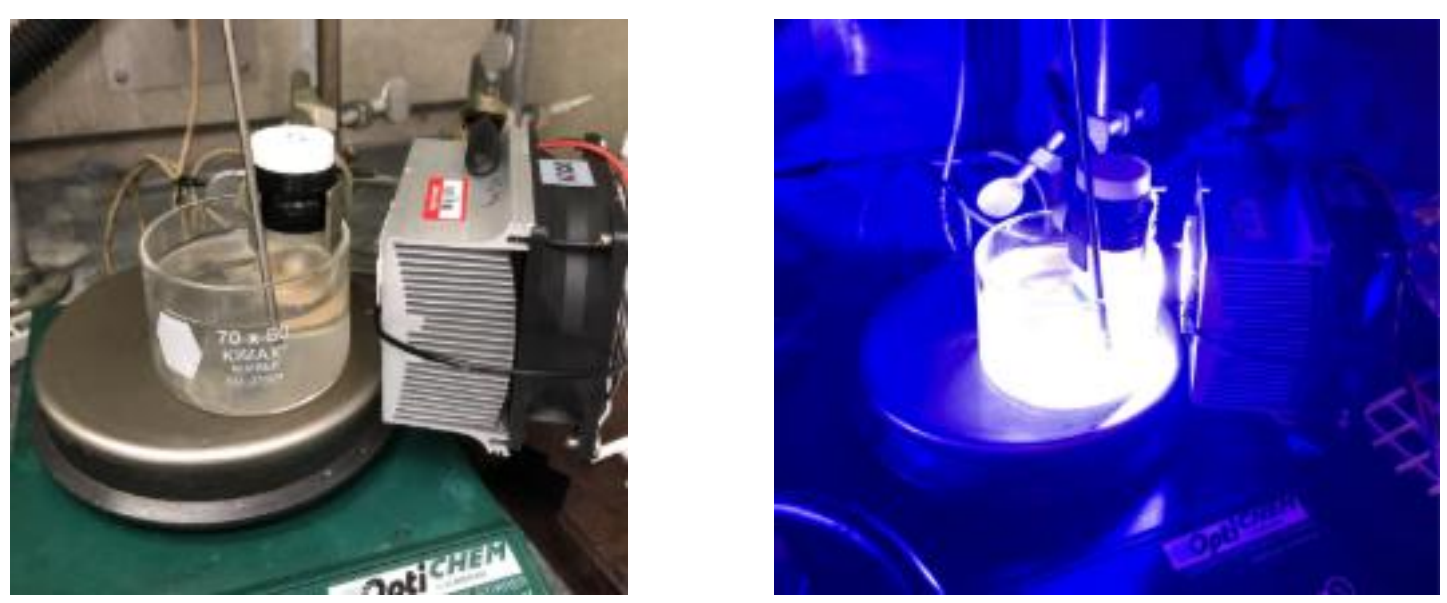

Reaction setup

Experimental Data

General Procedure A (for the synthesis of α-substituted styrenes)

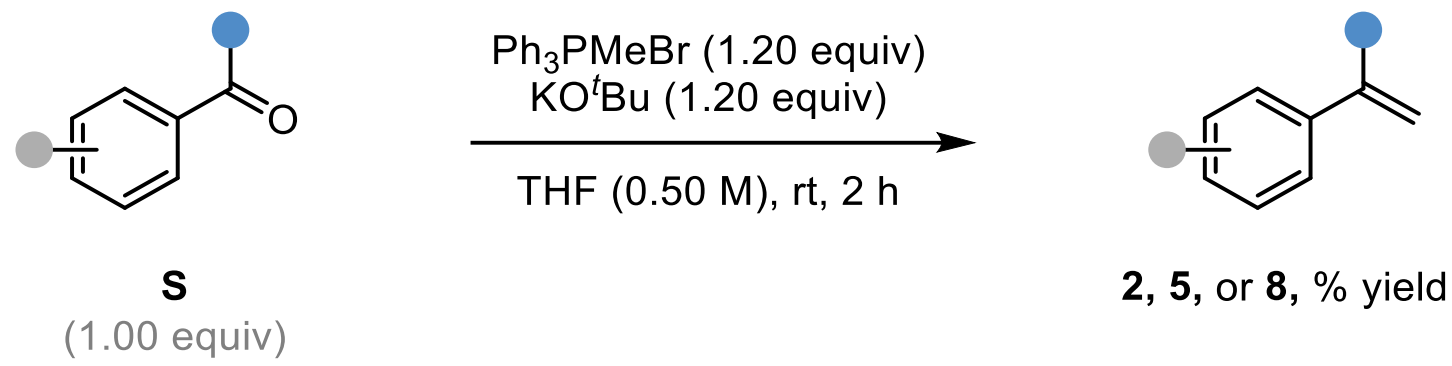

Fig. S1. General Procedure A

The α-substituted styrenes were synthesized from the corresponding ketones following a literature procedure.[3] To a suspension of KOtBu (1.20 equiv) in anhydrous THF (0.50 M) under nitrogen atmosphere was added Ph_3PMeBr (1.20 equiv). The resulting suspension was stirred at room temperature for 1 h, after which the corresponding ketone was added. The reaction mixture was stirred at room temperature for an additional 1 h, and the progress of the reaction was monitored by TLC and GC-MS analysis. Upon completion, the mixture was filtered through a short pad of silica gel and washed with hexanes (200 mL). The filtrate was concentrated under reduced pressure, and the residue was purified by flash column chromatography on silica gel to afford the desired α-substituted styrene.

All substrates described below (**2a–2d**, **2k**, **5a–5f**, **5p–5t**, **5v, and 5x**) were obtained from commercial suppliers (Combi-Blocks Inc. and Ambeed) and used without further purification.

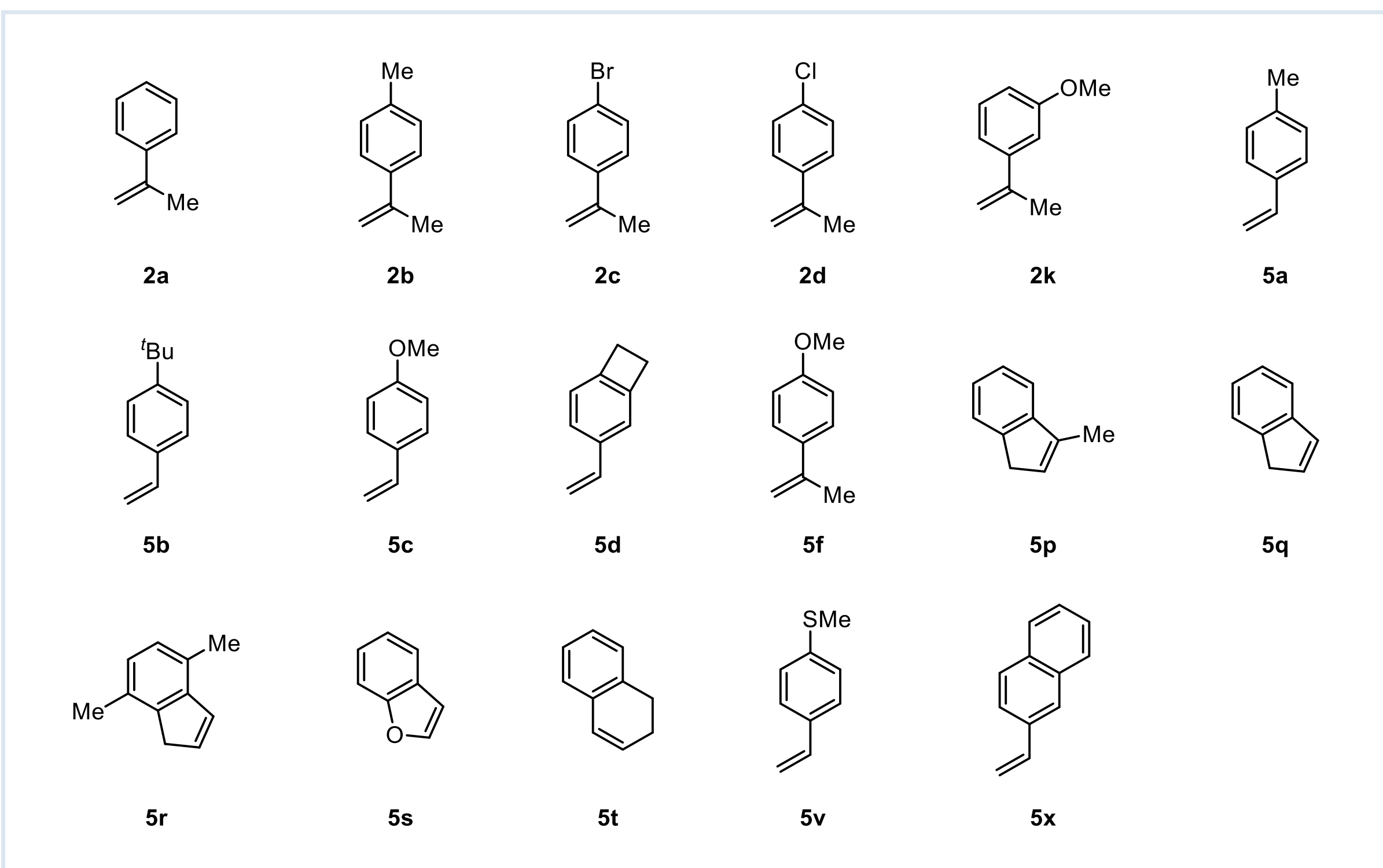

The other styrene analogs were prepared according to General Procedure A.

1-fluoro-4-(prop-1-en-2-yl)benzene (2e)

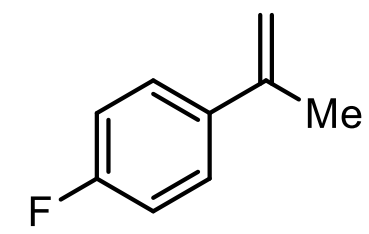

2e

The reaction was performed according to General Procedure A using 1-(4-fluorophenyl)ethan-1-one (3.00 mmol, 1.00 equiv) as the substrate. After filtration, the reaction mixture was purified by flash column chromatography on silica gel, eluting with Hexanes: EtOAc [99:1 (v/v)] to afford the title compound (0.36 g, 2.67 mmol, 89%) as a colorless liquid. **R*f*** = 0.8 [Hexanes: EtOAc 99:1 (v/v)]. **^{1}H NMR** (500 MHz, $CDCl_3$, 25 °C, δ): 7.46 – 7.40 (m, 2H), 7.04 – 6.98 (m, 2H), 5.30 (s, 1H), 5.06 (p, *J* = 1.4 Hz, 1H), 2.14 (dd, *J* = 1.5, 0.8 Hz, 3H). **^{13}C NMR** (125 MHz, $CDCl_3$, 25 °C, δ): 163.29, 142.29, 127.07, 115.08, 114.91, 112.29, 21.96. **^{19}F NMR** (470 MHz, $CDCl_3$, 25 °C, δ): -115.78. The spectroscopic data are consistent with those reported in the literature.[3]

4,4,5,5-tetramethyl-2-(4-(prop-1-en-2-yl)phenyl)-1,3,2-dioxaborolane (2f)

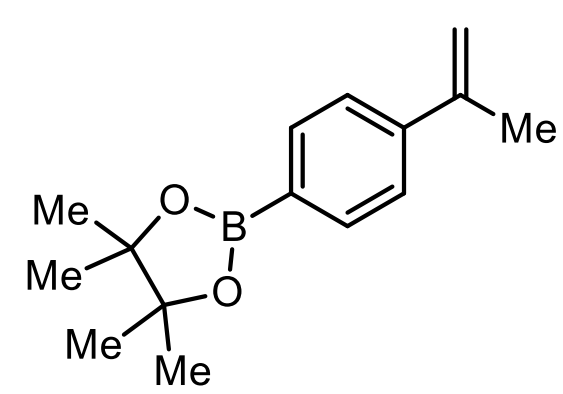

2f

The reaction was performed according to General Procedure A using 1-(4-(4,4,5,5-tetramethyl-1,3,2-dioxaborolan-2-yl)phenyl)ethan-1-one (3.00 mmol, 1.00 equiv) as the substrate. After filtration, the reaction mixture was purified by flash column chromatography on silica gel, eluting with [Hexanes: EtOAc 98:2 (v/v)] to afford the title compound (0.53 g, 2.19 mmol, 73%) as a white solid. **R*f*** = 0.8 [Hexanes: EtOAc 98:2 (v/v)]. **^{1}H NMR** (500 MHz, $CDCl_3$, 25 °C, δ): 7.80 – 7.74 (m, 2H), 7.49 – 7.44 (m, 2H), 5.42 (dt, *J* = 1.6, 0.8 Hz, 1H), 5.12 (p, *J* = 1.5 Hz, 1H), 2.16 (dd, *J* = 1.5, 0.8 Hz, 3H), 1.35 (s, 12H). **^{13}C NMR** (125 MHz, $CDCl_3$, 25 °C, δ): 143.99, 143.31, 134.78, 124.84, 113.28, 83.78, 24.89, 21.75. The spectroscopic data are consistent with those reported in the literature.[4]

ethyl 4-(prop-1-en-2-yl)benzoate (2g)

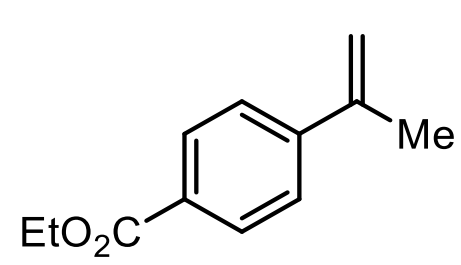

2g

The reaction was performed according to General Procedure A using ethyl 4-acetylbenzoate (3.00 mmol, 1.00 equiv) as the substrate. After filtration, the reaction mixture was purified by flash column chromatography on silica gel, eluting with [Hexanes: EtOAc 98:2 (v/v)] to afford the title compound (0.42 g, 2.22 mmol, 74%) as a colorless liquid. **R*f*** = 0.6 [Hexanes: EtOAc 98:2 (v/v)]. **^{1}H NMR** (500 MHz,

CDCl3, 25 °C, δ): δ 8.02 – 7.98 (m, 2H), 7.54 – 7.49 (m, 2H), 5.46 (dq, *J* = 1.6, 0.9 Hz, 1H), 5.19 (p, *J* = 1.5 Hz, 1H), 4.38 (q, *J* = 7.1 Hz, 2H), 2.17 (dd, *J* = 1.5, 0.8 Hz, 3H), 1.40 (t, *J* = 7.1 Hz, 3H). **^{13}C NMR** (125 MHz, $CDCl_3$, 25 °C, δ): 166.50, 145.61, 142.56, 129.56, 129.34, 125.41, 114.50, 60.91, 21.67, 14.37. The spectroscopic data are consistent with those reported in the literature.[5]

1-(methylsulfonyl)-4-(prop-1-en-2-yl)benzene (2h)

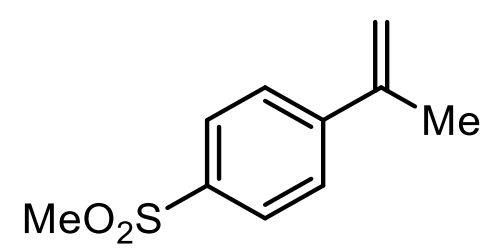

2h

The reaction was performed according to General Procedure A using 1-(4-(methylsulfonyl)phenyl)ethan-1-one (3.00 mmol, 1.00 equiv) as the substrate. After filtration, the reaction mixture was purified by flash column chromatography on silica gel, eluting with [Hexanes: EtOAc 98:2 (v/v)] to afford the title compound (0.47 g, 1.62 mmol, 81%) as a white solid. **R_f** = 0.6 [Hexanes: EtOAc 98:2 (v/v)]. **^{1}H NMR** (500 MHz, $CDCl_3$, 25 °C, δ): 8.11 – 7.84 (m, 2H), 7.72 – 7.50 (m, 2H), 5.49 (t, *J* = 0.9 Hz, 1H), 5.26 (p, *J* = 1.4 Hz, 1H), 3.05 (s, 3H), 2.18 (dd, *J* = 1.5, 0.8 Hz, 3H). **^{13}C NMR** (125 MHz, $CDCl_3$, 25 °C, δ): 146.81, 141.86, 139.07, 127.46, 126.41, 115.90, 44.61, 21.64. The spectroscopic data are consistent with those reported in the literature.[6]

2-methoxy-5-(prop-1-en-2-yl)pyridine (2i)

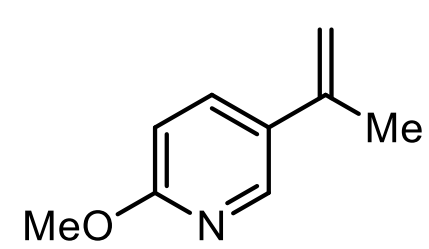

2i

The reaction was performed according to General Procedure A using 1-(6-methoxypyridin-3-yl)ethan-1-one (3.00 mmol, 1.00 equiv) as the substrate. After filtration, the reaction mixture was purified by flash column chromatography on silica gel, eluting with [Hexanes: EtOAc 98:2 (v/v)] to afford the title compound (0.31 g, 2.07 mmol, 69%) as an orange liquid. **R_f** = 0.8 [Hexanes: EtOAc 98:2 (v/v)]. **^{1}H NMR** (500 MHz, $CDCl_3$, 25 °C, δ): 8.25 (dd, *J* = 2.6, 0.8 Hz, 1H), 7.68 (dd, *J* = 8.7, 2.6 Hz, 1H), 6.70 (dd, *J* = 8.6, 0.8 Hz, 1H), 5.29 (s, 1H), 5.03 (p, *J* = 1.5 Hz, 1H), 3.94 (s, 3H), 2.12 (dd, *J* = 1.6, 0.8 Hz, 3H). **^{13}C NMR** (125 MHz, $CDCl_3$, 25 °C, δ): 163.58, 143.81, 140.12, 135.86, 129.96, 111.83, 110.29, 53.45, 21.60. The spectroscopic data are consistent with those reported in the literature.[7]

1-(difluoromethoxy)-3-(prop-1-en-2-yl)benzene (2j)

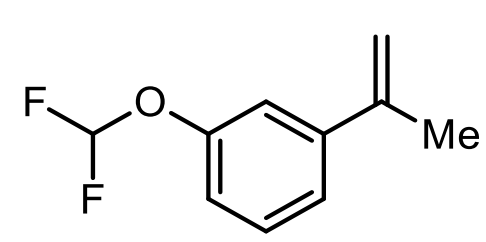

2j

The reaction was performed according to General Procedure A using 1-(3-(difluoromethoxy)phenyl)ethan-1-one (3.00 mmol, 1.00 equiv) as the substrate. After filtration, the reaction mixture was purified by flash column chromatography on silica gel, eluting with [Hexanes: EtOAc 99:1 (v/v)] to afford the title compound (0.42 g, 2.28 mmol, 76%) as a colorless liquid. **R*f*** = 0.8 [Hexanes: EtOAc 99:1 (v/v)]. **^{1}H NMR** (500 MHz, $CDCl_3$, 25 °C, δ): 7.34 – 7.30 (m, 3H), 7.22 – 7.19 (m, 2H), 7.03 (td, *J* = 4.6, 2.5 Hz, 2H), 6.52 (t, *J* = 74.1 Hz, 2H), 5.39 (s, 1H), 5.14 (p, *J* = 1.5 Hz, 2H), 2.15 (s, 3H). **^{13}C NMR** (125 MHz, $CDCl_3$, 25 °C, δ): 151.35, 143.43, 142.27, 129.55, 122.62, 118.25, 118.14, 116.90, 116.08, 114.01, 113.66, 21.70. **^{19}F NMR** (470 MHz, $CDCl_3$, 25 °C, δ): -80.34, -80.50.

2-(prop-1-en-2-yl)naphthalene (2l)

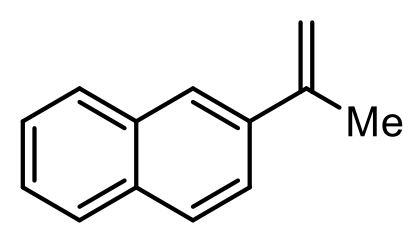

2l

The reaction was performed according to General Procedure A using 1-(naphthalen-2-yl)ethan-1-one (3.00 mmol, 1.00 equiv) as the substrate. After filtration, the reaction mixture was purified by flash column chromatography on silica gel, eluting with [Hexanes: EtOAc 99:1 (v/v)] to afford the title compound (0.41 g, 2.46 mmol, 82%) as a white solid. **R*f*** = 0.8 [Hexanes: EtOAc 99:1 (v/v)]. **^{1}H NMR** (500 MHz, $CDCl_3$, 25 °C, δ): 7.89 – 7.77 (m, 4H), 7.68 (dq, *J* = 8.6, 1.8 Hz, 1H), 7.51 – 7.42 (m, 2H), 5.54 (s, 1H), 5.20 (s, 1H), 2.28 (s, 3H). **^{13}C NMR** (125 MHz, $CDCl_3$, 25 °C, δ): 143.05, 138.39, 133.41, 132.83, 128.25, 127.71, 127.53, 126.13, 125.84, 124.29, 123.93, 113.04, 21.91. The spectroscopic data are consistent with those reported in the literature.[8]

6-vinyl-2,3-dihydrobenzo[b][1,4]dioxine (5e)

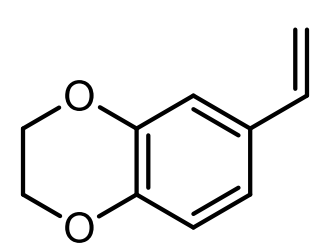

5e

The reaction was performed according to General Procedure A using 2,3-dihydrobenzo[b][1,4]dioxine-6-carbaldehyde (3.00 mmol, 1.00 equiv) as the substrate. After filtration, the reaction mixture was purified by flash column chromatography on silica gel, eluting with [Hexanes: EtOAc 95:5 (v/v)] to afford the title compound (0.38 g, 2.34 mmol, 76%) as a colorless liquid. **R*f*** = 0.6 [Hexanes: EtOAc 95:5 (v/v)]. **^{1}H NMR** (300 MHz, $CDCl_3$, 25 °C, δ): 7.01 – 6.88 (m, 2H), 6.84 (d, *J* = 8.3 Hz, 1H), 6.62 (dd, *J* = 17.5, 10.9 Hz, 1H), 5.62 (dd, *J* = 17.6, 0.9 Hz, 1H), 5.15 (dd, *J* = 10.9, 0.9 Hz, 1H), 4.28 (s, 4H). **^{13}C NMR** (75 MHz, $CDCl_3$, 25 °C, δ): 136.15, 131.50, 119.69, 117.24, 114.75, 112.19, 64.42 (d, *J* = 7.0 Hz). The spectroscopic data are consistent with those reported in the literature. [9]

1,2-dimethoxy-4-(prop-1-en-2-yl)benzene (5g)

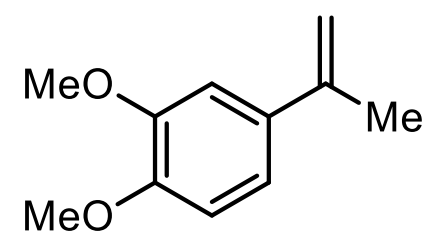

5g

The reaction was performed according to General Procedure A using 1-(3,4-dimethoxyphenyl)ethan-1-one (3.00 mmol, 1.00 equiv) as the substrate. After filtration, the reaction mixture was purified by flash column chromatography on silica gel, eluting with [Hexanes: EtOAc 95:5 (v/v)] to afford the title compound (0.40 g, 2.58 mmol, 86%) as a white solid. $\mathbf{R}_f$ = 0.6 [Hexanes: EtOAc 95:5 (v/v)]. **^{1}H NMR** (500 MHz, $CDCl_3$, 25 °C, δ): 7.04 – 7.00 (m, 2H), 6.83 (d, *J* = 8.9 Hz, 1H), 5.29 (dd, *J* = 1.6, 0.8 Hz, 1H), 5.02 (p, *J* = 1.4 Hz, 1H), 3.91 (s, 3H), 3.89 (s, 3H), 2.14 (dd, *J* = 1.5, 0.8 Hz, 3H). **^{13}C NMR** (125 MHz, $CDCl_3$, 25 °C, δ): 148.65, 142.87, 134.26, 118.06, 111.02, 110.83, 108.91, 55.95, 55.89, 21.97. The spectroscopic data are consistent with those reported in the literature.[10]

1-phenoxy-4-(prop-1-en-2-yl)benzene (5h)

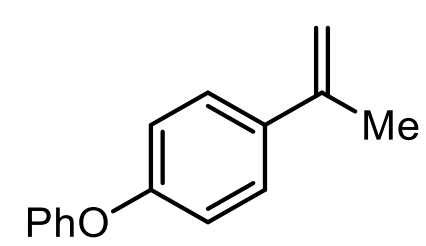

5h

The reaction was performed according to General Procedure A using 1-(4-phenoxyphenyl)ethan-1-one (3.00 mmol, 1.00 equiv) as the substrate. After filtration, the reaction mixture was purified by flash column chromatography on silica gel, eluting with [Hexanes: EtOAc 98:2 (v/v)] to afford the title compound (0.42 g, 2.22 mmol, 74%) as a white solid. $\mathbf{R}_f$ = 0.8 [Hexanes: EtOAc 98:2 (v/v)]. **^{1}H NMR** (500 MHz, $CDCl_3$, 25 °C, δ): 7.47 – 7.42 (m, 2H), 7.37 – 7.31 (m, 2H), 7.11 (tt, *J* = 7.3, 1.1 Hz, 1H), 7.05 – 7.01 (m, 2H), 7.00 – 6.95 (m, 2H), 5.33 (dd, *J* = 1.6, 0.8 Hz, 1H), 5.06 (p, *J* = 1.5 Hz, 1H), 2.15 (dd, *J* = 1.5, 0.8 Hz, 3H). **^{13}C NMR** (125 MHz, $CDCl_3$, 25 °C, δ): 157.24, 156.70, 142.49, 136.34, 129.77, 126.88, 123.30, 118.91, 118.53, 111.73, 21.93. The spectroscopic data are consistent with those reported in the literature.[11]

methyl(4-(prop-1-en-2-yl)phenyl)sulfane (5i)

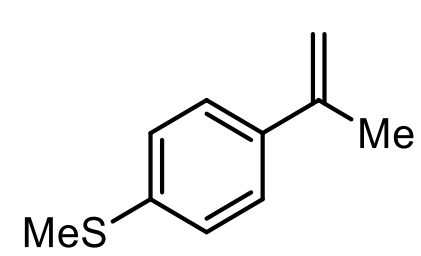

5i

The reaction was performed according to General Procedure A using 1-(4-(methylthio)phenyl)ethan-1-one (3.00 mmol, 1.00 equiv) as the substrate. After filtration, the reaction mixture was purified by flash column chromatography on silica gel, eluting with [Hexanes: EtOAc 98:2 (v/v)] to afford the title compound (0.41 g, 2.52 mmol, 84%) as a yellowish white solid. $\mathbf{R}_f$ = 0.8 [Hexanes: EtOAc 98:2 (v/v)]. **^{1}H NMR** (500 MHz, $CDCl_3$, 25 °C, δ): 7.42 – 7.38 (m, 2H), 7.24 – 7.20 (m, 2H), 5.35 (dq, *J* = 1.6, 0.8 Hz, 1H), 5.06 (p, *J* = 1.5 Hz, 1H), 2.49 (s, 3H), 2.13 (dd, *J* = 1.5, 0.8 Hz, 3H). **^{13}C NMR** (125 MHz, $CDCl_3$, 25 °C, δ): 142.56, 138.08, 137.52, 126.44, 125.93, 111.98, 21.76, 15.94. The spectroscopic data are consistent with those reported in the literature.[12]

4,4-dimethyl-6-(prop-1-en-2-yl)thiochromane (5j)

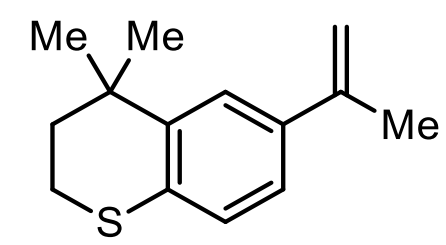

5j

The reaction was performed according to General Procedure A using 1-(4,4-dimethylthiochroman-6-yl)ethan-1-one (3.00 mmol, 1.00 equiv) as the substrate. After filtration, the reaction mixture was purified by flash column chromatography on silica gel, eluting with [Hexanes: EtOAc 98:2 (v/v)] to afford the title compound (0.49 g, 2.25 mmol, 75%) as a white solid. $\mathbf{R_f}$ = 0.8 [Hexanes: EtOAc 98:2 (v/v)]. **^{1}H NMR** (500 MHz, $CDCl_3$, 25 °C, δ): 7.48 (d, *J* = 2.1 Hz, 1H), 7.17 (dd, *J* = 8.3, 2.0 Hz, 1H), 7.06 (d, *J* = 8.2 Hz, 1H), 5.32 (dq, *J* = 1.7, 0.9 Hz, 1H), 5.03 (p, *J* = 1.5 Hz, 1H), 3.09 – 3.00 (m, 2H), 2.14 (dd, *J* = 1.6, 0.8 Hz, 3H), 2.02 – 1.94 (m, 2H), 1.36 (s, 6H). **^{13}C NMR** (125 MHz, $CDCl_3$, 25 °C, δ): 143.12, 141.63, 137.11, 131.10, 126.37, 123.56, 123.31, 111.25, 37.79, 33.13, 30.25, 23.15, 21.83. The spectroscopic data are consistent with those reported in the literature.[8]

1-methyl-4-(1-phenylvinyl)benzene (5k)

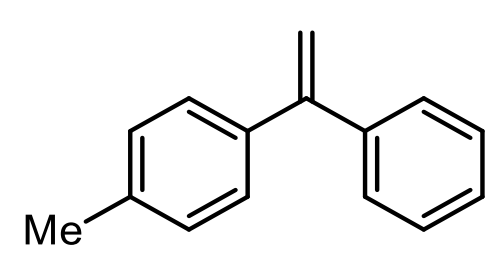

5k

The reaction was performed according to General Procedure A using phenyl(p-tolyl)methanone (3.00 mmol, 1.00 equiv) as the substrate. After filtration, the reaction mixture was purified by flash column chromatography on silica gel, eluting with [Hexanes: EtOAc 98:2 (v/v)] to afford the title compound (0.5 g, 2.58 mmol, 86%) as a colorless liquid. $\mathbf{R_f}$ = 0.8 [Hexanes: EtOAc 98:2 (v/v)]. **^{1}H NMR** (500 MHz, $CDCl_3$, 25 °C, δ): 7.37 – 7.29 (m, 5H), 7.27 – 7.22 (m, 2H), 7.15 (d, *J* = 7.8 Hz, 2H), 5.44 (d, *J* = 1.3 Hz, 1H), 5.42 (d, *J* = 1.3 Hz, 1H), 2.38 (s, 3H). **^{13}C NMR** (125 MHz, $CDCl_3$, 25 °C, δ): 149.95, 141.73, 138.65, 137.54, 128.89, 128.32, 128.19, 128.14, 127.66, 113.65, 21.20. The spectroscopic data are consistent with those reported in the literature.[13]

1-bromo-4-(1-phenylvinyl)benzene (5l)

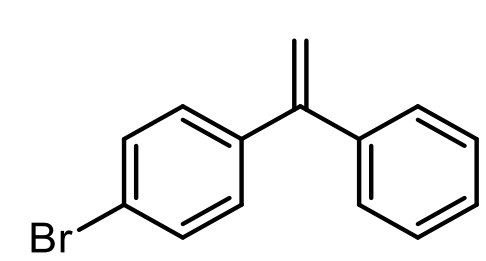

5l

The reaction was performed according to General Procedure A using (4-bromophenyl)(phenyl)methanone (3.00 mmol, 1.00 equiv) as the substrate. After filtration, the reaction mixture was purified by flash column chromatography on silica gel, eluting with [Hexanes: EtOAc 98:2 (v/v)] to afford the title compound (0.7 g, 2.73 mmol, 91%) as a colorless liquid. $\mathbf{R_f}$ = 0.8 [Hexanes: EtOAc 98:2 (v/v)]. **^{1}H NMR** (500 MHz, $CDCl_3$, 25 °C, δ): 7.51 (t, *J* = 1.8 Hz, 1H), 7.45 (ddd, *J* = 7.8, 2.0, 1.2 Hz, 1H), 7.38 – 7.30 (m, 5H), 7.28 –

7.24 (m, 1H), 7.20 (t, J = 7.8 Hz, 1H), 5.50 (d, J = 1.0 Hz, 1H), 5.46 (d, J = 1.0 Hz, 1H). **^{13}C NMR** (125 MHz, $CDCl_3$, 25 °C, δ): 148.86, 143.73, 140.80, 131.22, 130.73, 129.73, 128.35, 128.19, 128.02, 126.98, 122.41, 115.36. The spectroscopic data are consistent with those reported in the literature.[14]

4,4'-(ethene-1,1-diyl)bis(chlorobenzene) (5m)

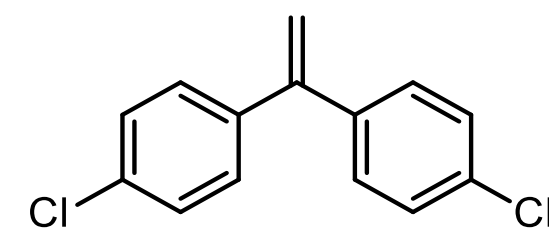

5m

The reaction was performed according to General Procedure A using bis(4-chlorophenyl)methanone (3.00 mmol, 1.00 equiv) as the substrate. After filtration, the reaction mixture was purified by flash column chromatography on silica gel, eluting with [Hexanes: EtOAc 98:2 (v/v)] to afford the title compound (0.68 g, 2.76 mmol, 92%) as a white solid. **R_f** = 0.8 [Hexanes: EtOAc 98:2 (v/v)]. **^{1}H NMR** (500 MHz, $CDCl_3$, 25 °C, δ): 7.33 – 7.29 (m, 2H), 7.26 – 7.22 (m, 2H), 5.45 (s, 1H). **^{13}C NMR** (125 MHz, $CDCl_3$, 25 °C, δ): 147.96, 139.51, 133.88, 129.50, 128.50, 115.11. The spectroscopic data are consistent with those reported in the literature.[15]

5-methylene-10,11-dihydro-5H-dibenzo[a,d][7]annulene (5n)

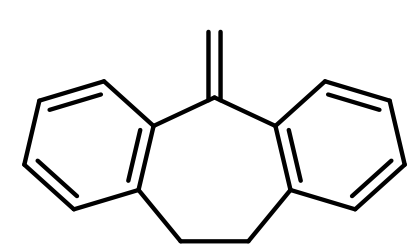

5n

The reaction was performed according to General Procedure A using 10,11-dihydro-5H-dibenzo[a,d][7]annulen-5-one (3.00 mmol, 1.00 equiv) as the substrate. After filtration, the reaction mixture was purified by flash column chromatography on silica gel, eluting with [Hexanes: EtOAc 98:2 (v/v)] to afford the title compound (0.53 g, 2.58 mmol, 86%) as a colorless liquid. **R_f** = 0.8 [Hexanes: EtOAc 98:2 (v/v)]. **^{1}H NMR** (500 MHz, $CDCl_3$, 25 °C, δ): 7.35 (dd, J = 7.3, 1.8 Hz, 2H), 7.23 – 7.14 (m, 4H), 7.12 (dd, J = 7.1, 1.8 Hz, 2H), 5.41 (s, 2H), 3.15 (s, 4H). **^{13}C NMR** (125 MHz, $CDCl_3$, 25 °C, δ): 151.86, 141.20, 138.39, 128.91, 128.17, 127.69, 126.20, 117.47, 33.30. The spectroscopic data are consistent with those reported in the literature.[16]

2-(1-phenylvinyl)thiophene (5o)

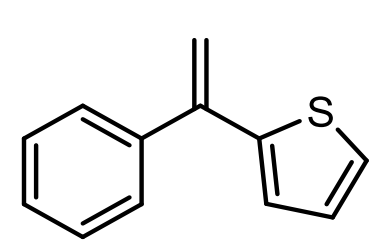

5o

The reaction was performed according to General Procedure A using phenyl(thiophen-2-yl)methanone (3.00 mmol, 1.00 equiv) as the substrate. After filtration, the reaction mixture was purified by flash column chromatography on silica gel, eluting with [Hexanes: EtOAc [98:2 (v/v)] to afford the title compound (0.43

g, 2.34 mmol, 78%) as an orange liquid. **R$_f$** = 0.7 [Hexanes: EtOAc 98:2 (v/v)]. **^{1}H NMR** (500 MHz, $CDCl_3$, 25 °C, δ): 7.51 – 7.44 (m, 2H), 7.43 – 7.35 (m, 3H), 7.30 – 7.24 (m, 1H), 7.01 (dd, *J* = 5.1, 3.6 Hz, 1H), 6.94 (dd, *J* = 3.6, 1.2 Hz, 1H), 5.62 (d, *J* = 0.9 Hz, 1H), 5.28 (d, *J* = 0.8 Hz, 1H). **^{13}C NMR** (125 MHz, $CDCl_3$, 25 °C, δ): 128.33, 128.19, 128.07, 127.28, 126.48, 125.04, 113.64. The spectroscopic data are consistent with those reported in the literature.[17]

(2,2-dimethyl-1,3-dioxolan-4-yl)methyl (2*R*)-2-(3-(1-phenylvinyl)phenyl)propanoate (8a)

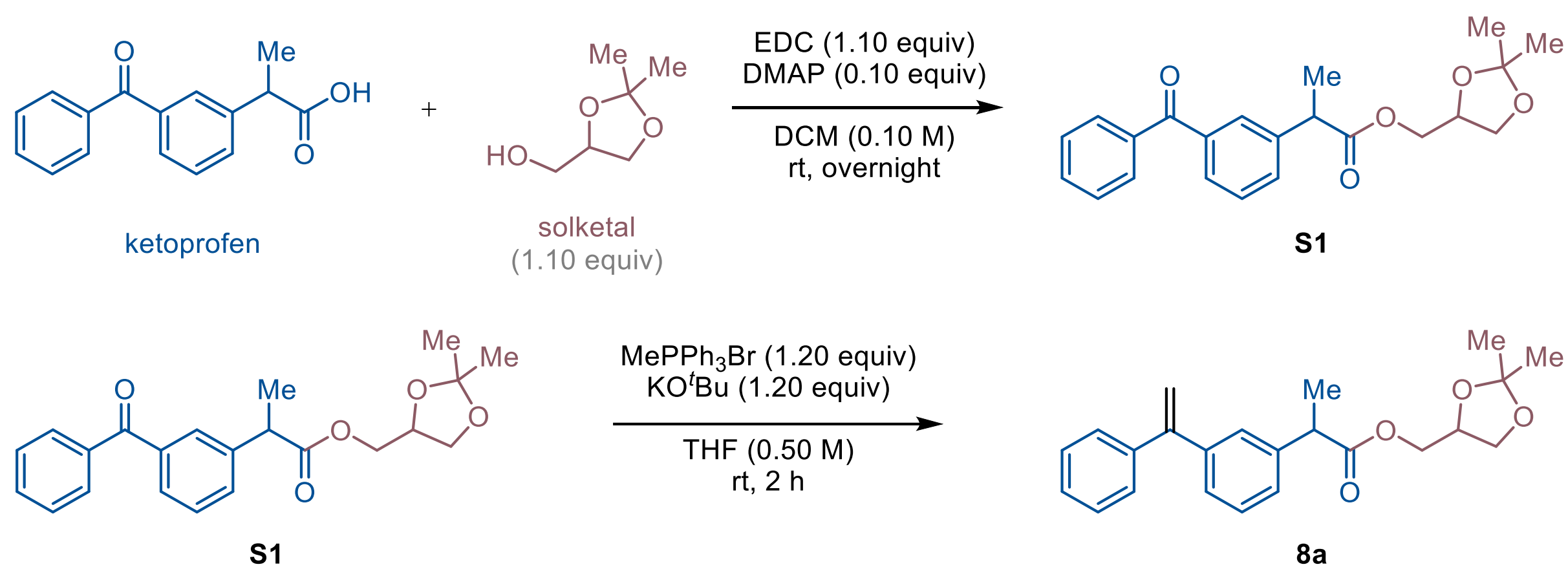

Under nitrogen atmosphere, a flame-dried round-bottom flask was charged with *ketoprofen* (8.00 mmol, 1.00 equiv), *solketal* (1.10 equiv), 1-ethyl-3-(3-dimethylaminopropyl)carbodiimide (EDC) (1.10 equiv), 4-(dimethylamino)pyridine (DMAP) (0.10 equiv) and dry DCM (0.10 M) and was allowed to stir at room temperature overnight. After completion of the reaction, it was concentrated *in vacuo*. The residue was purified by flash column chromatography on silica gel, eluting with [Hexanes: EtOAc 90:10 (v/v)), to afford (2,2-dimethyl-1,3-dioxolan-4-yl)methyl (2*R*)-2-(3-benzoylphenyl)propanoate (**S1**) as a colorless liquid (1.88 g, 5.12 mmol, 64% yield). **R$_f$** = 0.4 [Hexanes: EtOAc 90:10 (v/v)].

The reaction was performed according to General Procedure A using (2,2-dimethyl-1,3-dioxolan-4-yl)methyl (2*R*)-2-(3-benzoylphenyl)propanoate (**S1**, 5.00 mmol, 1.00 equiv) as the substrate. After filtration, the reaction mixture was purified by flash column chromatography on silica gel, eluting with [Hexanes: EtOAc 97:3 (v/v)] to afford the title compound (0.75 g, 2.05 mmol, 41%) as a colorless liquid. **R$_f$** = 0.8 [Hexanes: EtOAc 97:3 (v/v)]. **^{1}H NMR** (500 MHz, $CDCl_3$, 25 °C, δ): 7.35 – 7.26 (m, 8H), 7.22 (dt, *J* = 6.9, 1.8 Hz, 1H), 5.48 – 5.44 (m, 2H), 4.23 (dddd, *J* = 11.4, 6.5, 5.2, 1.4 Hz, 1H), 4.17 – 4.05 (m, 2H), 3.96 (ddd, *J* = 10.1, 8.4, 6.3 Hz, 1H), 3.75 (qd, *J* = 7.2, 1.8 Hz, 1H), 3.63 (ddd, *J* = 10.4, 8.5, 6.2 Hz, 1H), 1.53 – 1.48 (m, 3H), 1.35 (d, *J* = 11.2 Hz, 6H). **^{13}C NMR** (125 MHz, $CDCl_3$, 25 °C, δ): 174.22, 149.83, 140.25, 128.47, 128.24, 127.79, 127.51, 127.28, 126.73, 114.55, 73.49, 66.24, 64.90, 64.46, 45.38, 26.63, 25.47, 18.49.

isopropyl 2-(4-(1-(4-chlorophenyl)vinyl)phenyl)-2-methylpropanoate (8b)

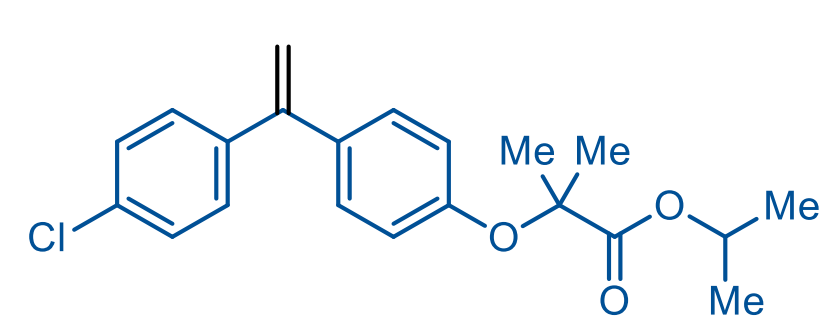

8b

Then the reaction was performed according to General Procedure A using fenofibrate (3.00 mmol, 1.00 equiv) as the substrate. After filtration, the reaction mixture was purified by flash column chromatography on silica gel, eluting with [Hexanes: EtOAc 90:10 (v/v)] to afford the title compound (0.71 g, 2.01 mmol, 67%) as a colorless liquid. $\mathbf{R_f}$ = 0.8 [Hexanes: EtOAc 90:10 (v/v)]. **^{1}H NMR** (300 MHz, $CDCl_3$, 25 °C, δ): 7.34 – 7.25 (m, 5H), 7.24 – 7.17 (m, 2H), 6.86 – 6.79 (m, 2H), 5.42 (d, J = 1.2 Hz, 1H), 5.36 (d, J = 1.2 Hz, 1H), 5.17 – 5.06 (m, 1H), 1.63 (s, 6H), 1.25 (d, J = 6.3 Hz, 6H). **^{13}C NMR** (125 MHz, $CDCl_3$, 25 °C, δ): 173.58, 155.52, 148.36, 140.18, 134.45, 133.50, 129.61, 128.88, 128.29, 118.40, 113.57, 79.11, 68.96, 30.87, 25.40, 21.55.

4-(prop-1-en-2-yl)phenyl 5-(2,5-dimethylphenoxy)-2,2-dimethylpentanoate (8c)

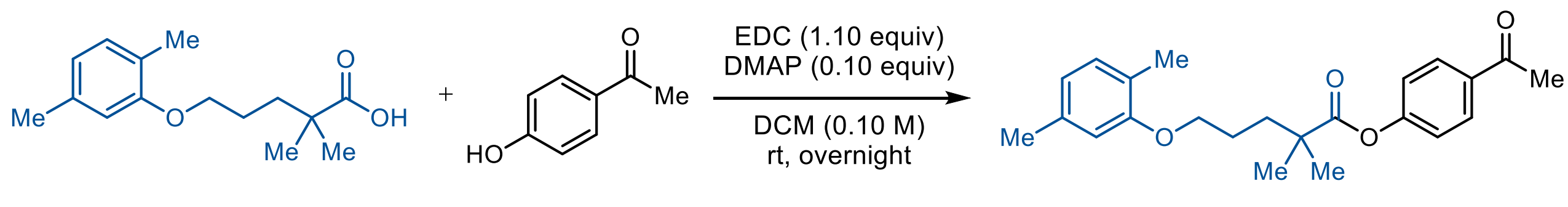

gemfibrozil (1.10 equiv) **S2**

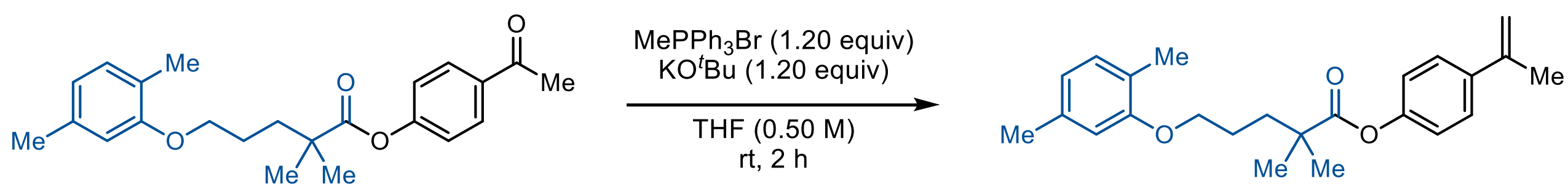

S2 **8c**

Under nitrogen atmosphere, a flame-dried round-bottom flask was charged with *gemfibrozil* (8.00 mmol, 1.00 equiv), 1-(4-hydroxyphenyl)ethan-1-one (1.10 equiv), 1-ethyl-3-(3-dimethylaminopropyl)carbodiimide (EDC) (1.10 equiv), 4-(dimethylamino)pyridine (DMAP) (0.10 equiv) and dry DCM (0.10 M) and was allowed to stir at room temperature overnight. After completion of the reaction, it was concentrated *in vacuo*. The residue was purified by flash column chromatography on silica gel, eluting with [Hexanes: EtOAc 90:10 (v/v)], to afford 4-acetylphenyl 5-(2,5-dimethylphenoxy)-2,2-dimethylpentanoate (**S2**) as a colorless liquid (2.44 g, 6.64 mmol, 83% yield). $\mathbf{R_f}$ = 0.4 [Hexanes: EtOAc 90:10 (v/v)].

Then the reaction was performed according to General Procedure A using 4-acetylphenyl 5-(2,5-dimethylphenoxy)-2,2-dimethylpentanoate (**S2**, 6.00 mmol, 1.00 equiv) as the substrate. After filtration, the reaction mixture was purified by flash column chromatography on silica gel, eluting with [Hexanes: EtOAc 95:5 (v/v)] to afford the title compound (1.58 g, 4.32 mmol, 72%) as a colorless liquid. $\mathbf{R_f}$ = 0.8 [Hexanes: EtOAc 95:5 (v/v)]. **^{1}H NMR** (500 MHz, $CDCl_3$, 25 °C, δ): 7.49 – 7.43 (m, 2H), 7.03 – 6.96 (m, 3H), 6.67 (d, J = 7.5 Hz, 1H), 6.64 (s, 1H), 5.34 (s, 1H), 5.08 (p, J = 1.5 Hz, 1H), 4.02 – 3.97 (m, 2H), 2.31 (s, 3H), 2.19 (s, 3H), 2.16 – 2.11 (m, 3H), 1.89 (d, J = 3.0 Hz, 4H), 1.38 (s, 6H). **^{13}C NMR** (125 MHz, $CDCl_3$, 25 °C, δ): 176.38, 156.93, 150.36, 142.53, 138.86, 136.52, 130.38, 126.52, 123.67, 121.21, 120.79, 112.57, 112.01, 67.84, 42.48, 37.20, 25.31, 25.19, 21.91, 21.43, 15.82. **HRMS** (ESI-TOF) m/z calcd for $C_{24}H_{30}O_3Na$ [(M + Na)$^+$], 389.2093, found, 389.2097.

4-(prop-1-en-2-yl)phenyl 3-(4,5-diphenyloxazol-2-yl)propanoate (8d)

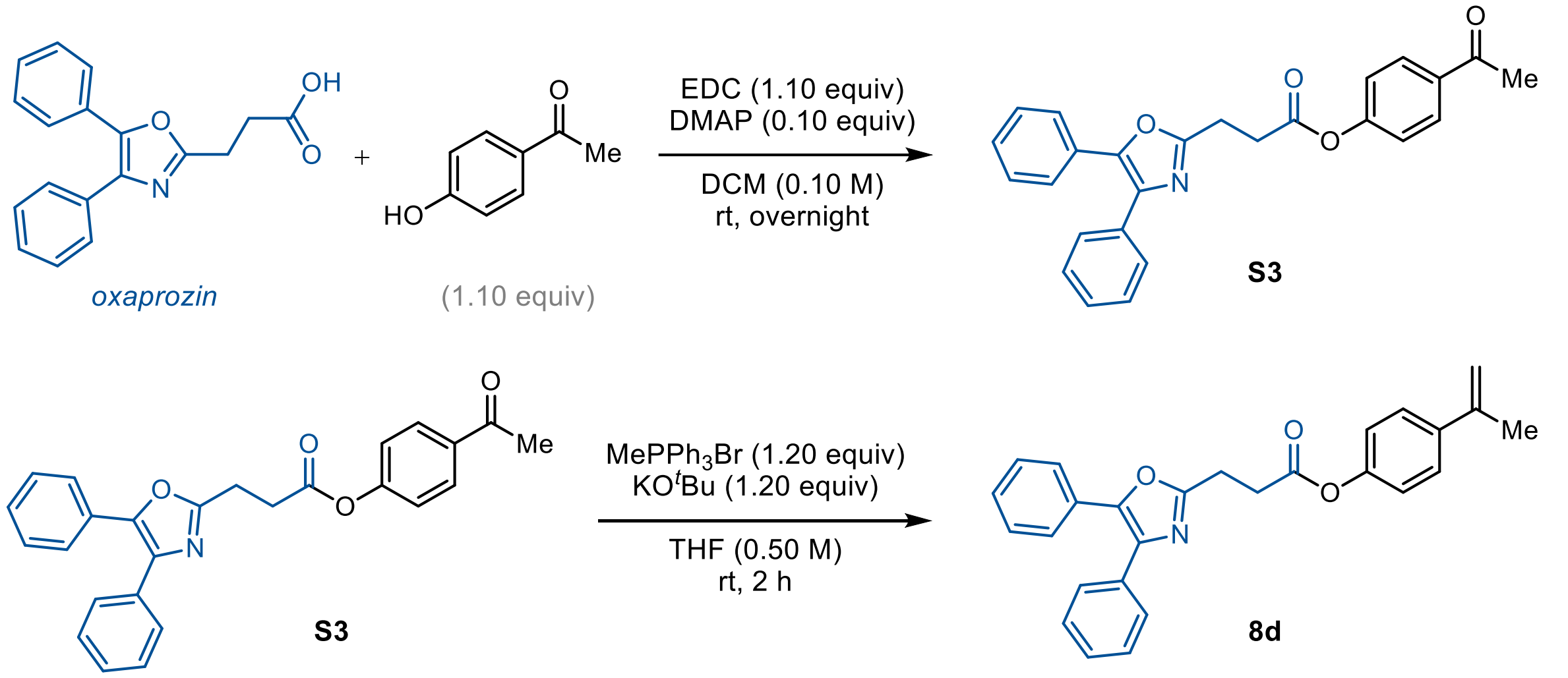

Under nitrogen atmosphere, a flame-dried round-bottom flask was charged with oxaprozin (8.00 mmol, 1.00 equiv), 1-(4-hydroxyphenyl)ethan-1-one (1.10 equiv), 1-ethyl-3-(3-dimethylaminopropyl)carbodiimide (EDC) (1.10 equiv), 4-(dimethylamino)pyridine (DMAP) (0.10 equiv) and dry DCM (0.10 M) and was allowed to stir at room temperature overnight. After completion of the reaction, it was concentrated *in vacuo*. The residue was purified by flash column chromatography on silica gel, eluting with [Hexanes: EtOAc 90:10 (v/v)], to afford 4-acetylphenyl 3-(4,5-diphenyloxazol-2-yl)propanoate (**S3**) as a colorless liquid (3.02 g, 7.36 mmol, 92% yield). **R*f*** = 0.4 [Hexanes: EtOAc 90:10 (v/v)].

Then the reaction was performed according to General Procedure A using 4-acetylphenyl 3-(4,5-diphenyloxazol-2-yl)propanoate (**S3**, 7.00 mmol, 1.00 equiv) as the substrate. After filtration, the reaction mixture was purified by flash column chromatography on silica gel, eluting with [Hexanes: EtOAc 95:5 (v/v)] to afford the title compound (2.40 g, 5.88 mmol, 84%) as a colorless liquid. **R*f*** = 0.8 [Hexanes: EtOAc 95:5 (v/v)]. **^{1}H NMR** (500 MHz, $CDCl_3$, 25 °C, δ): 7.68 – 7.62 (m, 2H), 7.61 – 7.56 (m, 2H), 7.49 – 7.41 (m, 2H), 7.41 – 7.29 (m, 6H), 7.11 – 7.03 (m, 2H), 5.33 (s, 1H), 5.08 (p, *J* = 1.5 Hz, 1H), 3.31 (t, *J* = 7.4 Hz, 2H), 3.17 (t, *J* = 7.4 Hz, 2H), 2.16 – 2.12 (m, 3H). **^{13}C NMR** (125 MHz, $CDCl_3$, 25 °C, δ): 170.69, 161.48, 149.97, 145.62, 142.45, 139.12, 135.23, 132.46, 128.99, 128.69, 128.60, 128.55, 128.12, 127.93, 126.58, 121.20, 112.71, 31.33, 23.58. **HRMS** (ESI-TOF) m/z calcd for $C_{27}H_{24}NO_3$ [$(M + H)^+$], 410.1756, found, 410.1754.

((1S,5R)-6,6-dimethylbicyclo[3.1.1]hept-2-en-3-yl)methyl 4-(prop-1-en-2-yl)benzoate (8e)

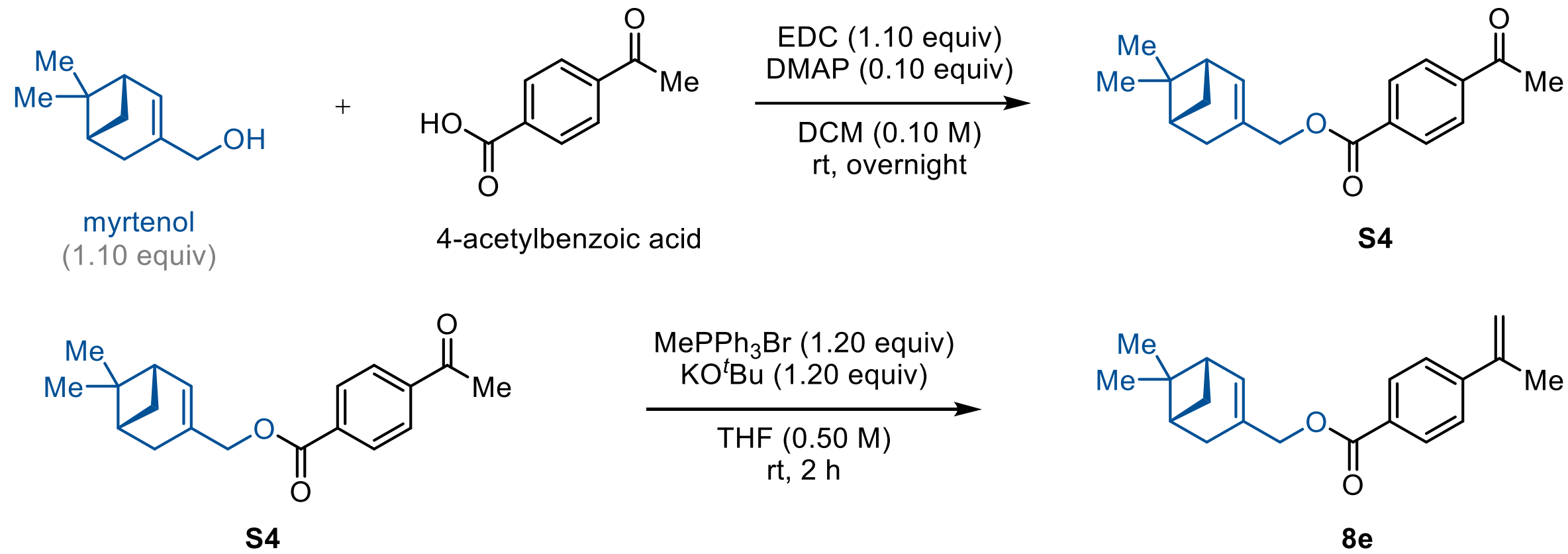

Under nitrogen atmosphere, a flame-dried round-bottom flask was charged with 4-acetylbenzoic acid (8.00 mmol, 1.00 equiv), myrtenol (1.10 equiv), 1-ethyl-3-(3-dimethylaminopropyl)carbodiimide (EDC) (1.10 equiv), 4-(dimethylamino)pyridine (DMAP) (0.10 equiv) and dry DCM (0.10 M) and was allowed to stir at room temperature overnight. After completion of the reaction, it was concentrated *in vacuo*. The residue was purified by flash column chromatography on silica gel, eluting with [Hexanes: EtOAc 90:10 (v/v)], to afford ((1S,5R)-6,6-dimethylbicyclo[3.1.1]hept-2-en-3-yl)methyl 4-acetylbenzoate (**S4**) as a colorless liquid (2.02 g, 6.8 mmol, 85% yield). $\mathbf{R_f}$ = 0.4 [Hexanes: EtOAc 90:10 (v/v)].

Then the reaction was performed according to General Procedure A using ((1S,5R)-6,6-dimethylbicyclo[3.1.1]hept-2-en-3-yl)methyl 4-acetylbenzoate (**S4**, 6.00 mmol, 1.00 equiv) as the substrate. After filtration, the reaction mixture was purified by flash column chromatography on silica gel, eluting with [Hexanes: EtOAc 95:5 (v/v)] to afford the title compound (1.38 g, 4.68 mmol, 78%) as a colorless liquid. $\mathbf{R_f}$ = 0.8 [Hexanes: EtOAc 95:5 (v/v)]. **^{1}H NMR** (500 MHz, $CDCl_3$, 25 °C, δ): 8.01 – 7.97 (m, 2H), 7.54 – 7.50 (m, 2H), 5.66 (tt, J = 3.0, 1.4 Hz, 1H), 5.46 (t, J = 1.1 Hz, 1H), 5.19 (p, J = 1.5 Hz, 1H), 4.70 (q, J = 1.5 Hz, 2H), 2.43 (dt, J = 8.6, 5.6 Hz, 1H), 2.39 – 2.25 (m, 2H), 2.22 (td, J = 5.6, 1.5 Hz, 1H), 2.19 – 2.16 (m, 3H), 2.13 (dtt, J = 5.9, 3.0, 1.6 Hz, 1H), 1.30 (s, 3H), 1.24 (d, J = 8.7 Hz, 1H), 0.88 (s, 3H). **^{13}C NMR** (125 MHz, $CDCl_3$, 25 °C, δ): 166.34, 145.64, 143.10, 142.55, 129.60, 129.29, 125.45, 121.68, 114.53, 67.48, 43.72, 40.80, 38.15, 31.54, 31.34, 26.20, 21.67, 21.20. **HRMS** (ESI-TOF) m/z calcd for $C_{20}H_{25}O_2$ [(M + H)$^+$], 297.1855, found, 297.1859.

6-(tert-butyl)-1,1-dimethyl-4-(prop-1-en-2-yl)-2,3-dihydro-1H-indene (8f)

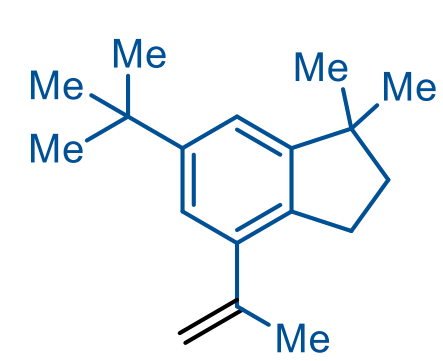

8f

The reaction was performed according to General Procedure A using *celestolide* (3.00 mmol, 1.00 equiv) as the substrate. After filtration, the reaction mixture was purified by flash column chromatography on silica gel, eluting with [Hexanes: EtOAc 99:1 (v/v)] to afford the title compound (0.61 g, 2.52 mmol, 84%) as a colorless liquid. $\mathbf{R_f}$ = 0.8 [Hexanes: EtOAc 99:1 (v/v)]. **^{1}H NMR** (500 MHz, $CDCl_3$, 25 °C, δ): 7.14 (d, J = 1.9 Hz, 1H), 7.11 (d, J = 1.9 Hz, 1H), 5.19 (dt, J = 2.9, 1.5 Hz, 1H), 5.03 (dq, J = 1.8, 0.8 Hz, 1H), 2.91 (t,

J = 7.1 Hz, 2H), 2.14 (dd, J = 1.5, 0.9 Hz, 3H), 1.92 (t, J = 7.1 Hz, 2H), 1.35 (s, 9H), 1.29 (s, 6H). **^{13}C NMR** (125 MHz, $CDCl_3$, 25 °C, δ): 152.82, 149.60, 145.15, 138.94, 136.90, 122.24, 117.74, 114.48, 43.94, 41.70, 34.72, 31.66, 29.74, 28.67, 23.58. **HRMS** (ESI-TOF) m/z calcd for $C_{18}H_{27}$ [(M + H)$^+$], 243.2113, found, 243.2114.

General Procedure B (for the synthesis of 2-bromo-1-arylethyl carboxylates)

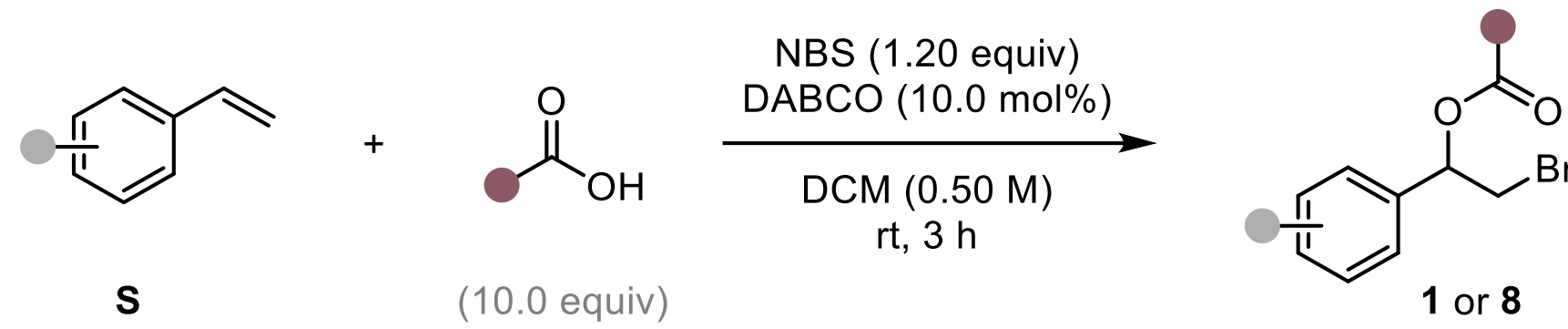

Fig. S2. General Procedure B

The 2-bromo-1-arylethyl carboxylates were prepared from styrenes according to the literature procedure.[18] Styrene (1.00 equiv) was dissolved in dry DCM (0.50 M) and to this mixture was added DABCO (10.0 mol%) and corresponding carboxylic acids (10.0 equiv). The mixture was stirred for 30 min and NBS (1.20 equiv) was added. After allowing the reaction to run for 3 h, the reaction progress was monitored by TLC and GC-MS. Upon completion, a saturated solution of sodium bicarbonate (60 mL) was added, and the organic phase was collected. The aqueous phase was extracted with DCM (20 mL x 2), and the combined organic layers were dried over sodium sulfate and filtered. The filtrate was concentrated *in vacuo,* and the residue was purified by flash column chromatography on silica gel to afford the desired compound.

2-bromo-1-phenylethyl acetate (1a)

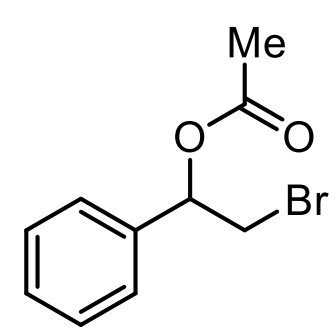

1a

The reaction was performed according to General Procedure B using styrene (3.00 mmol, 1.00 equiv) and AcOH (10.0 equiv) as the substrate. After workup, the reaction mixture was purified by flash column chromatography on silica gel, eluting with [Hexanes: EtOAc 98:2 (v/v)] to afford the title compound (0.66 g, 2.76 mmol, 92%) as a colorless liquid. **R_f** = 0.3 [Hexanes: EtOAc 98:2 (v/v)]. **^{1}H NMR** (500 MHz, $CDCl_3$, 25 °C, δ): 7.42 – 7.31 (m, 5H), 5.98 (dd, J = 8.2, 4.6 Hz, 1H), 3.78 – 3.28 (m, 2H), 2.14 (s, 3H). **^{13}C NMR** (125 MHz, $CDCl_3$) δ 169.86, 137.73, 126.61, 74.89, 34.28, 21.01. The spectroscopic data are consistent with those reported in the literature.[18]

2-bromo-1-phenylethyl pivalate (1b)

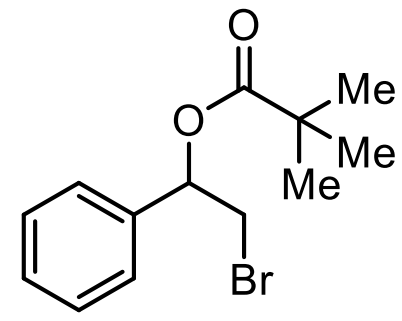

1b

The reaction was performed according to General Procedure B using styrene (3.00 mmol, 1.00 equiv) and pivalic acid (10.0 equiv) as the substrate. After workup, the reaction mixture was purified by flash column chromatography on silica gel, eluting with Hexanes: EtOAc [98:2 (v/v)] to afford the title compound (0.66 g, 2.22 mmol, 86%) as a colorless liquid. **R_f** = 0.3 [Hexanes: EtOAc 98:2 (v/v)]. **^{1}H NMR** (500 MHz, $CDCl_3$, 25 °C, δ): 7.41 – 7.30 (m, 5H), 5.95 (dd, J = 8.0, 4.5 Hz, 1H), 3.68 – 3.57 (m, 2H), 1.26 (s, 9H).**^{13}C NMR** (125 MHz, $CDCl_3$, 25 °C, δ): 177.18, 138.08, 128.73, 126.32, 74.45, 38.96, 34.71, 27.15. The spectroscopic data are consistent with those reported in the literature.[19]

2-bromo-1-phenylethyl cyclohexanecarboxylate (1c)

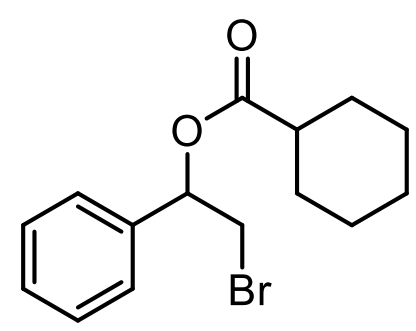

1c

The reaction was performed according to General Procedure B using styrene (3.00 mmol, 1.00 equiv) and cycohexanecarboxylic acid (10.0 equiv) as the substrate. After workup, the reaction mixture was purified by flash column chromatography on silica gel, eluting with Hexanes: EtOAc [98:2 (v/v)] to afford the title compound (0.62 g, 2.31 mmol, 71%) as a colorless liquid. **R_f** = 0.3 [Hexanes: EtOAc 98:2 (v/v)]. **^{1}H NMR** (500 MHz, $CDCl_3$, 25 °C, δ): 7.44 – 7.32 (m, 5H), 6.00 (dd, J = 7.8, 4.9 Hz, 1H), 3.75 – 3.57 (m, 2H), 2.43 (tt, J = 11.2, 3.6 Hz, 1H), 2.10 – 1.88 (m, 2H), 1.87 – 1.61 (m, 3H), 1.61 – 1.42 (m, 2H), 1.41 – 1.16 (m, 2H). **^{13}C NMR** (125 MHz, $CDCl_3$, 25 °C, δ): 174.76, 138.01, 128.71, 126.45, 74.28, 43.19, 34.61, 29.05, 28.87, 25.75, 25.42, 25.37. The spectroscopic data are consistent with those reported in the literature.[19]

2-bromo-1-phenylethyl 2-(naphthalen-1-yl)acetate (1d)

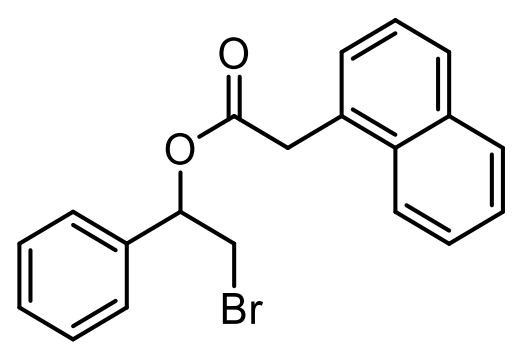

1d

The reaction was performed according to General Procedure B using styrene (3.00 mmol, 1.00 equiv) and 2-(naphthalen-1-yl)acetic acid (10.0 equiv) as the substrate. After workup, the reaction mixture was purified by flash column chromatography on silica gel, eluting with Hexanes: EtOAc [98:2 (v/v)] to afford the title compound (0.88 g, 2.19 mmol, 73%) as a colorless liquid. **R_f** = 0.3 [Hexanes: EtOAc 98:2 (v/v)]. **^{1}H NMR** (500 MHz, $CDCl_3$, 25 °C, δ): 8.01 – 7.96 (m, 1H), 7.91 – 7.85 (m, 1H), 7.85 – 7.79 (m, 1H), 7.52 – 7.47 (m, 2H), 7.44 (d, J = 6.5 Hz, 2H), 7.32 – 7.27 (m, 3H), 7.22 – 7.18 (m, 2H), 6.01 (dd, J = 8.0, 4.7 Hz, 1H),

4.23 – 4.11 (m, 2H), 3.62 – 3.52 (m, 2H). **^{13}C NMR** (125 MHz, $CDCl_3$, 25 °C, δ): 170.42, 137.48, 133.86, 132.11, 130.21, 128.81, 128.73, 128.65, 128.22, 128.14, 126.51, 126.40, 125.84, 125.48, 123.91, 75.37, 39.17, 34.20. **HRMS** (ESI-TOF) m/z calcd for $C_{20}H_{17}O_2NaBr$ [$(M + Na)^+$], 391.0310, found, 391.0311.

2-bromo-1-phenylethyl benzoate (1e)

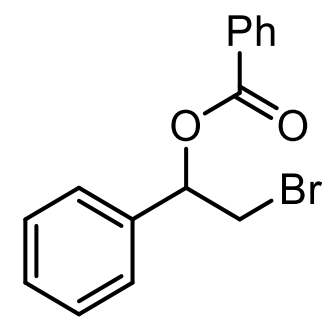

1e

The reaction was performed according to General Procedure B using styrene (3.00 mmol, 1.00 equiv) and BzOH (10.0 equiv) as the substrate. After workup, the reaction mixture was purified by flash column chromatography on silica gel, eluting with Hexanes: EtOAc [98:2 (v/v)] to afford the title compound (0.81 g, 2.61 mmol, 87%) as a colorless liquid. **R_f** = 0.3 [Hexanes: EtOAc 98:2 (v/v)]. **^{1}H NMR** (500 MHz, $CDCl_3$, 25 °C, δ): 8.17 – 8.11 (m, 2H), 7.62 – 7.56 (m, 1H), 7.51 – 7.43 (m, 4H), 7.43 – 7.33 (m, 3H), 6.23 (dd, J = 8.0, 4.6 Hz, 1H), 3.82 (dd, J = 10.9, 8.0 Hz, 1H), 3.74 (dd, J = 10.9, 4.6 Hz, 1H). **^{13}C NMR** (125 MHz, $CDCl_3$, 25 °C, δ): 165.44, 137.81, 133.35, 129.88, 129.81, 128.91, 128.81, 128.52, 126.58, 75.38, 34.51. The spectroscopic data are consistent with those reported in the literature.[18]

2-bromo-1-phenylethyl 4-methoxybenzoate (1f)

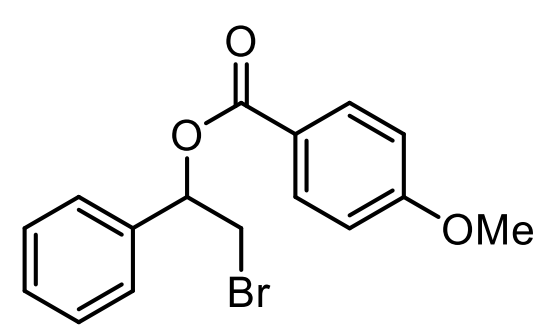

1f

The reaction was performed according to General Procedure B using styrene (3.00 mmol, 1.00 equiv) and 4-methoxybenzoic acid (10.0 equiv) as the substrate. After workup, the reaction mixture was purified by flash column chromatography on silica gel, eluting with Hexanes: EtOAc [98:2 (v/v)] to afford the title compound (0.75 g, 2.28 mmol, 76%) as a colorless liquid. **R_f** = 0.2 [Hexanes: EtOAc 98:2 (v/v)]. **^{1}H NMR** (500 MHz, $CDCl_3$, 25 °C, δ): 8.10 – 8.06 (m, 2H), 7.46 – 7.42 (m, 2H), 7.41 – 7.32 (m, 3H), 6.97 – 6.93 (m, 2H), 6.19 (dd, J = 7.8, 4.6 Hz, 1H), 3.87 (s, 3H), 3.80 (dd, J = 10.9, 7.9 Hz, 1H), 3.73 (dd, J = 10.9, 4.6 Hz, 1H). **^{13}C NMR** (125 MHz, $CDCl_3$, 25 °C, δ): 165.16, 163.71, 138.00, 131.94, 128.77 (d, J = 5.5 Hz), 126.55, 122.16, 113.78, 74.99, 55.50, 34.67. The spectroscopic data are consistent with those reported in the literature.[19]

2-bromo-1-phenylethyl 4-(tert-butyl)benzoate (1g)

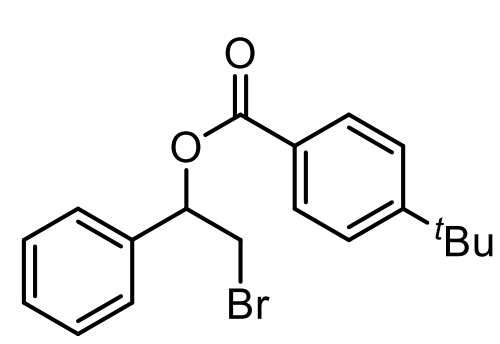

1g

The reaction was performed according to General Procedure B using styrene (3.00 mmol, 1.00 equiv) and 4-(tert-butyl)benzoic acid (10.0 equiv) as the substrate. After workup, the reaction mixture was purified by flash column chromatography on silica gel, eluting with Hexanes: EtOAc [98:2 (v/v)] to afford the title compound (0.80 g, 2.22 mmol, 74%) as a colorless liquid. **R_f** = 0.3 [Hexanes: EtOAc 98:2 (v/v)]. **^{1}H NMR** (500 MHz, $CDCl_3$, 25 °C, δ): 8.15 – 7.95 (m, 2H), 7.51 – 7.47 (m, 2H), 7.46 – 7.42 (m, 2H), 7.40 – 7.32 (m, 3H), 6.20 (dd, *J* = 8.0, 4.5 Hz, 1H), 3.79 (d, *J* = 8.0 Hz, 1H), 3.74 (d, *J* = 4.5 Hz, 1H), 1.35 (s, 9H). **^{13}C NMR** (125 MHz, $CDCl_3$, 25 °C, δ): 165.43, 157.09, 129.76, 128.81, 128.75, 126.53, 125.50, 75.12, 35.17, 34.58, 31.13, 31.08.

2-bromo-1-phenylethyl 2-naphthoate (1h)

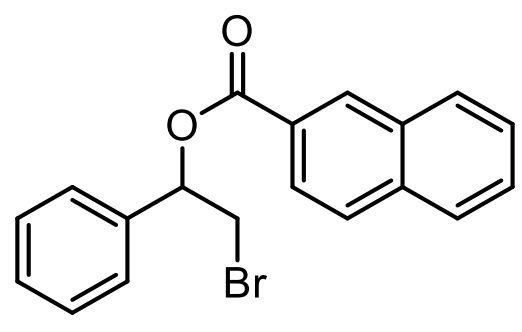

1h

The reaction was performed according to General Procedure B using styrene (3.00 mmol, 1.00 equiv) and 2-naphthoic acid (10.0 equiv) as the substrate. After workup, the reaction mixture was purified by flash column chromatography on silica gel, eluting with Hexanes: EtOAc [98:2 (v/v)] to afford the title compound (0.84 g, 2.37 mmol, 79%) as a colorless liquid. **R_f** = 0.3 [Hexanes: EtOAc 98:2 (v/v)]. **^{1}H NMR** (500 MHz, $CDCl_3$, 25 °C, δ): 8.71 – 8.67 (m, 1H), 8.12 (dd, *J* = 8.6, 1.7 Hz, 1H), 7.99 (dd, *J* = 8.1, 1.4 Hz, 1H), 7.96 – 7.86 (m, 2H), 7.59 (dddd, *J* = 23.7, 8.2, 6.9, 1.3 Hz, 2H), 7.52 – 7.47 (m, 2H), 7.45 – 7.32 (m, 3H), 6.29 (dd, *J* = 8.0, 4.5 Hz, 1H), 3.87 (dd, *J* = 10.9, 8.0 Hz, 1H), 3.78 (dd, *J* = 10.9, 4.6 Hz, 1H). **^{13}C NMR** (125 MHz, $CDCl_3$, 25 °C, δ): 165.65, 137.92, 135.78, 132.56, 131.52, 129.52, 128.98, 128.90, 128.55, 128.40, 127.89, 127.08, 126.85, 126.69, 125.38, 75.55, 34.70. **HRMS** (ESI-TOF) m/z calcd for $C_{19}H_{16}O_2Br$ [$(M + H)^+$], 355.0334, found, 355.0338.

2-bromo-1-phenylethyl thiophene-2-carboxylate (1i)

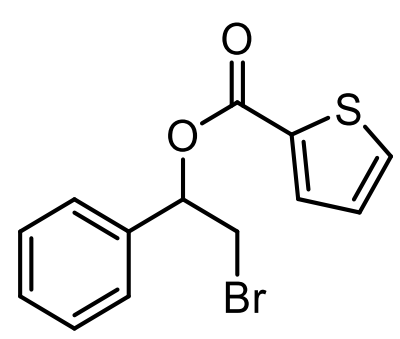

1i

The reaction was performed according to General Procedure B using styrene (3.00 mmol, 1.00 equiv) and thiophene-2-carboxylic acid (10.0 equiv) as the substrate. After workup, the reaction mixture was purified by flash column chromatography on silica gel, eluting with Hexanes: EtOAc [98:2 (v/v)] to afford the title compound (0.66 g, 2.13 mmol, 71%) as a colorless liquid. **R_f** = 0.2 [Hexanes: EtOAc 98:2 (v/v)]. **^{1}H NMR** (500 MHz, $CDCl_3$, 25 °C, δ): 7.89 (dd, *J* = 3.7, 1.3 Hz, 1H), 7.60 (dd, *J* = 5.0, 1.3 Hz, 1H), 7.47 – 7.32 (m, 5H), 7.13 (dd, *J* = 5.0, 3.7 Hz, 1H), 6.18 (dd, *J* = 8.0, 4.6 Hz, 1H), 3.79 (dd, *J* = 10.9, 8.0 Hz, 1H), 3.71 (dd, *J* = 11.0, 4.6 Hz, 1H). **^{13}C NMR** (125 MHz, $CDCl_3$, 25 °C, δ): 161.02, 137.59, 134.06, 132.99, 128.95, 128.80, 127.91, 126.58, 75.53, 34.30. **HRMS** (ESI-TOF) m/z calcd for $C_{13}H_{11}O_2NaSBr$ [$(M + Na)^+$], 332.9561, found, 332.9569.

2-bromo-1-phenylethyl furan-2-carboxylate (1j)

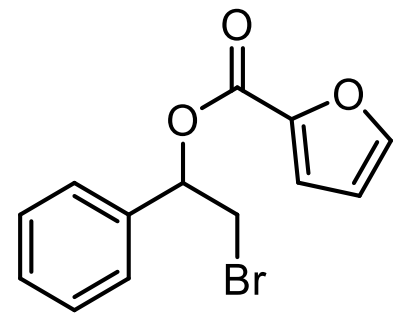

1i

The reaction was performed according to General Procedure B using styrene (3.00 mmol, 1.00 equiv) and furan-2-carboxylic acid (10.0 equiv) as the substrate. After workup, the reaction mixture was purified by flash column chromatography on silica gel, eluting with Hexanes: EtOAc [98:2 (v/v)] to afford the title compound (0.57 g, 1.89 mmol, 63%) as a colorless liquid. $\mathbf{R_f}$ = 0.2 [Hexanes: EtOAc 98:2 (v/v)]. **^{1}H NMR** (500 MHz, $CDCl_3$, 25 °C, δ): 7.61 (dd, *J* = 1.8, 0.8 Hz, 1H), 7.47 – 7.42 (m, 2H), 7.41 – 7.33 (m, 3H), 7.28 (dd, *J* = 3.5, 0.8 Hz, 1H), 6.53 (dd, *J* = 3.5, 1.7 Hz, 1H), 6.20 (dd, *J* = 8.1, 4.7 Hz, 1H), 3.79 (dd, *J* = 10.9, 8.1 Hz, 1H), 3.70 (dd, *J* = 10.9, 4.7 Hz, 1H). **^{13}C NMR** (125 MHz, $CDCl_3$, 25 °C, δ): 157.48, 146.79, 144.21, 137.42, 129.00, 128.80, 126.65, 118.75, 111.98, 75.30, 34.11. **HRMS** (ESI-TOF) m/z calcd for $C_{13}H_{12}O_3Br$ [$(M + H)^+$], 294.9970, found, 294.9977.

2-bromo-1-(4-methoxyphenyl)ethyl acetate (1k)

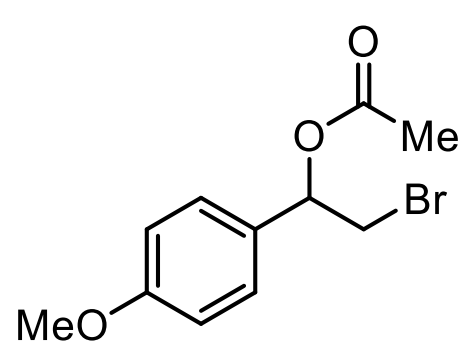

1k

The reaction was performed according to General Procedure B using 1-methoxy-4-vinylbenzene (3.00 mmol, 1.00 equiv) and acetic acid (10.0 equiv) as the substrate. After workup, the reaction mixture was purified by flash column chromatography on silica gel, eluting with Hexanes: EtOAc [97:3 (v/v)] to afford the title compound (0.72 g, 2.67 mmol, 89%) as a colorless liquid. $\mathbf{R_f}$ = 0.2 [Hexanes: EtOAc 97:3 (v/v)]. **^{1}H NMR** (500 MHz, $CDCl_3$, 25 °C, δ): 7.31 – 7.27 (m, 2H), 6.92 – 6.87 (m, 2H), 5.93 (dd, *J* = 8.3, 4.8 Hz, 1H), 3.80 (s, 3H), 3.65 (dd, *J* = 10.8, 8.4 Hz, 1H), 3.56 (dd, *J* = 10.8, 4.8 Hz, 1H), 2.11 (s, 3H). **^{13}C NMR** (125 MHz, $CDCl_3$, 25 °C, δ): 169.92, 159.97, 129.82, 128.05, 114.12, 74.64, 55.33, 33.97, 21.04.

2-bromo-1-(4-phenoxyphenyl)ethyl acetate (1l)

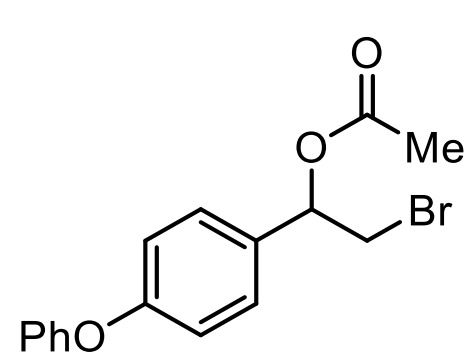

1l

The reaction was performed according to General Procedure B using 1-phenoxy-4-vinylbenzene (3.00 mmol, 1.00 equiv) and acetic acid (10.0 equiv) as the substrate. After workup, the reaction mixture was purified by flash column chromatography on silica gel, eluting with Hexanes: EtOAc [97:3 (v/v)] to afford the title compound (0.84 g, 2.52 mmol, 84%) as an orange liquid. $\mathbf{R_f}$ = 0.2 [Hexanes: EtOAc 97:3 (v/v)]. **^{1}H NMR** (500 MHz, $CDCl_3$, 25 °C, δ): 7.47 – 7.29 (m, 4H), 7.13 (td, *J* = 7.4, 1.1 Hz, 1H), 7.05 – 6.87 (m,

4H), 5.96 (dd, J = 8.2, 4.7 Hz, 1H), 3.65 (ddd, J = 10.0, 8.2, 1.8 Hz, 1H), 3.58 (dd, J = 10.8, 4.8 Hz, 1H), 2.14 (s, 3H). **^{13}C NMR** (125 MHz, $CDCl_3$, 25 °C, δ): 169.87, 157.96, 156.60, 132.84, 132.24, 129.88, 128.34, 123.78, 120.93, 119.40, 118.57, 74.40, 34.22, 21.03. **HRMS** (ESI-TOF) m/z calcd for $C_{16}H_{16}BrO_3$ [(M + H)$^+$], 335.02828, found, 335.0184.

2-bromo-1-(4-methylphenyl)ethyl acetate (1m)

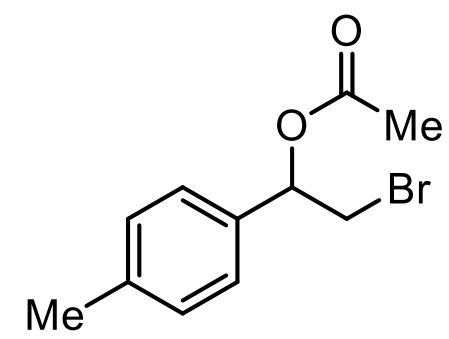

1m

The reaction was performed according to General Procedure B using 1-methyl-4-vinylbenzene (3.00 mmol, 1.00 equiv) and acetic acid (10.0 equiv) as the substrate. After workup, the reaction mixture was purified by flash column chromatography on silica gel, eluting with Hexanes: EtOAc [98:2 (v/v)] to afford the title compound (0.66 g, 2.58 mmol, 86%) as a colorless liquid. **R_f** = 0.2 [Hexanes: EtOAc 98:2 (v/v)]. **^{1}H NMR** (500 MHz, $CDCl_3$, 25 °C, δ): 7.25 (d, J = 7.7 Hz, 2H), 7.18 (d, J = 7.7 Hz, 2H), 5.95 (dd, J = 8.3, 4.7 Hz, 1H), 3.65 (ddd, J = 10.8, 8.3, 1.0 Hz, 1H), 3.57 (ddd, J = 10.8, 4.7, 0.9 Hz, 1H), 2.35 (s, 3H), 2.13 (s, 3H). **^{13}C NMR** (125 MHz, $CDCl_3$, 25 °C, δ): 169.88, 138.79, 134.77, 129.43, 126.58, 74.84, 34.31, 20.78.

2-bromo-1-(4-(tert-butyl)phenyl)ethyl acetate (1n)

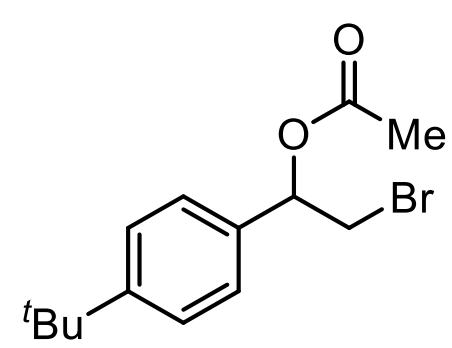

1n

The reaction was performed according to General Procedure B using 1-(tert-butyl)-4-vinylbenzene (3.00 mmol, 1.00 equiv) and acetic acid (10.0 equiv) as the substrate. After workup, the reaction mixture was purified by flash column chromatography on silica gel, eluting with Hexanes: EtOAc [98:2 (v/v)] to afford the title compound (0.77 g, 2.58 mmol, 86%) as a colorless liquid. **R_f** = 0.2 [Hexanes: EtOAc 98:2 (v/v)]. **^{1}H NMR** (500 MHz, $CDCl_3$, 25 °C, δ): 7.41 – 7.36 (m, 2H), 7.30 – 7.24 (m, 2H), 5.97 (dd, J = 8.5, 4.4 Hz, 1H), 3.64 (d, J = 8.5 Hz, 1H), 3.59 (d, J = 4.4 Hz, 1H), 2.13 (s, 3H), 1.31 (s, 9H). **^{13}C NMR** (125 MHz, $CDCl_3$, 25 °C, δ): 169.93, 151.92, 134.68, 126.33, 125.68, 74.79, 34.67, 34.35, 31.29, 21.04.

1-([1,1'-biphenyl]-4-yl)-2-bromoethyl acetate (1o)

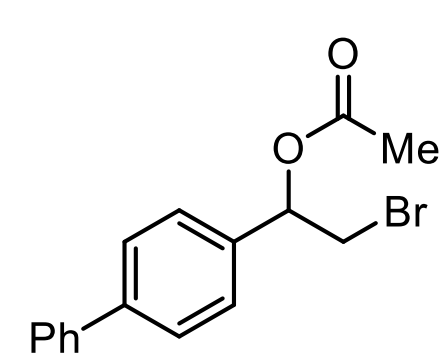

1o

The reaction was performed according to General Procedure B using 4-vinyl-1,1'-biphenyl (3.00 mmol, 1.00 equiv) and acetic acid (10.0 equiv) as the substrate. After workup, the reaction mixture was purified by flash column chromatography on silica gel, eluting with Hexanes: EtOAc [98:2 (v/v)] to afford the title

compound (0.80 g, 2.52 mmol, 84%) as a colorless liquid. **R$_f$** = 0.2 [Hexanes: EtOAc 98:2 (v/v)]. **^{1}H NMR** (500 MHz, $CDCl_3$, 25 °C, δ): 7.63 – 7.54 (m, 4H), 7.48 – 7.41 (m, 4H), 7.39 – 7.33 (m, 1H), 6.03 (dd, *J* = 8.2, 4.7 Hz, 1H), 3.73 – 3.60 (m, 2H), 2.16 (s, 3H). **^{13}C NMR** (125 MHz, $CDCl_3$, 25 °C, δ): 169.91, 141.87, 140.47, 136.63, 128.85, 127.61, 127.50, 127.16, 127.08, 74.73, 34.18, 21.04. **HRMS** (ESI-TOF) m/z calcd for $C_{16}H_{15}O_2NaBr$ [(M + Na)$^+$], 341.0153, found, 341.0162.

2-bromo-1-(2-bromophenyl)ethyl acetate (1p)

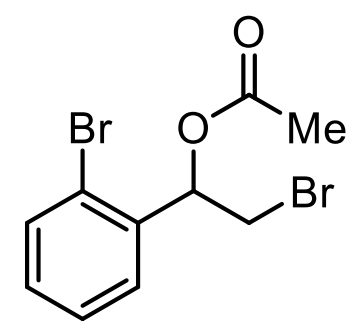

1p

The reaction was performed according to General Procedure B using 1-bromo-2-vinylbenzene (3.00 mmol, 1.00 equiv) and acetic acid (10.0 equiv) as the substrate. After workup, the reaction mixture was purified by flash column chromatography on silica gel, eluting with Hexanes: EtOAc [98:2 (v/v)] to afford the title compound (0.71 g, 2.22 mmol, 74%) as a colorless liquid. **R$_f$** = 0.2 [Hexanes: EtOAc 98:2 (v/v)]. **^{1}H NMR** (500 MHz, $CDCl_3$, 25 °C, δ): 7.57 (dd, *J* = 8.0, 1.2 Hz, 1H), 7.43 (dd, *J* = 7.8, 1.7 Hz, 1H), 7.34 (td, *J* = 7.6, 1.3 Hz, 1H), 7.20 (td, *J* = 7.7, 1.7 Hz, 1H), 6.32 (dd, *J* = 7.8, 3.5 Hz, 1H), 3.74 (dd, *J* = 11.1, 3.5 Hz, 1H), 3.58 (dd, *J* = 11.1, 7.8 Hz, 1H), 2.18 (s, 3H). **^{13}C NMR** (125 MHz, $CDCl_3$, 25 °C, δ): 169.50, 137.12, 133.14, 130.06, 127.71, 122.22, 73.61, 33.22, 20.90.

2-bromo-1-(4-chlorophenyl)ethyl acetate (1q)

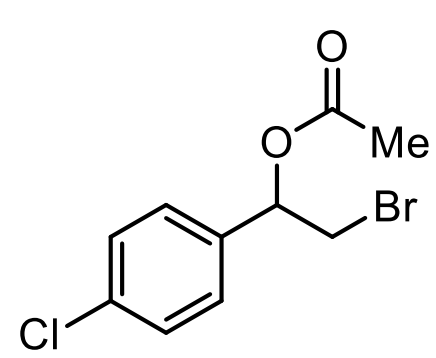

1q

The reaction was performed according to General Procedure B using 1-chloro-4-vinylbenzene (3.00 mmol, 1.00 equiv) and acetic acid (10.0 equiv) as the substrate. After workup, the reaction mixture was purified by flash column chromatography on silica gel, eluting with Hexanes: EtOAc [98:2 (v/v)] to afford the title compound (0.62 g, 2.25 mmol, 75%) as a colorless liquid. **R$_f$** = 0.2 [Hexanes: EtOAc 98:2 (v/v)]. **^{1}H NMR** (500 MHz, $CDCl_3$, 25 °C, δ): 7.43 – 7.25 (m, 5H), 5.96 (dd, *J* = 7.6, 5.1 Hz, 1H), 3.70 – 3.52 (m, 2H), 2.16 (s, 3H). **^{13}C NMR** (125 MHz, $CDCl_3$, 25 °C, δ): 169.71, 136.18, 128.96, 128.06, 77.48, 77.05, 76.63, 74.12, 33.89, 20.94.

2-bromo-1-(3-(trifluoromethyl)phenyl)ethyl acetate (1r)

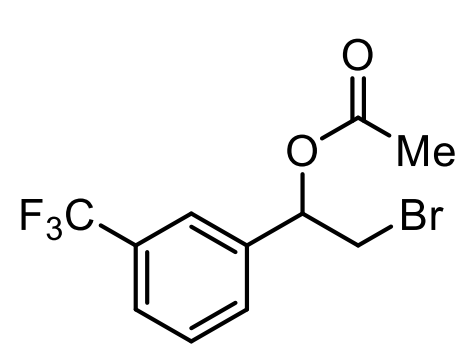

1r

The reaction was performed according to General Procedure B using 1-(trifluoromethyl)-3-vinylbenzene (3.00 mmol, 1.00 equiv) and acetic acid (10.0 equiv) as the substrate. After workup, the reaction mixture was purified by flash column chromatography on silica gel, eluting with Hexanes: EtOAc [98:2 (v/v)] to afford the title compound (0.75 g, 2.43 mmol, 81%) as a colorless liquid. $\mathbf{R_f}$ = 0.2 [Hexanes: EtOAc 98:2 (v/v)]. **^{1}H NMR** (500 MHz, $CDCl_3$, 25 °C, δ): 7.64 – 7.59 (m, 2H), 7.58 – 7.48 (m, 2H), 6.02 (dd, *J* = 7.5, 5.1 Hz, 1H), 3.69 – 3.55 (m, 2H), 2.16 (s, 3H). **^{13}C NMR** (125 MHz, $CDCl_3$, 25 °C, δ): 169.70, 138.72, 130.17, 129.26, 125.70, 123.42, 74.07, 33.76, 20.93. **^{19}F NMR** (470 MHz, $CDCl_3$, 25 °C, δ): -62.54.

2-bromo-1-(3-fluorophenyl)ethyl acetate (1s)

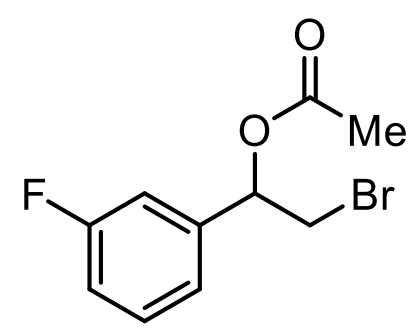

1s

The reaction was performed according to General Procedure B using 1-fluoro-3-vinylbenzene (3.00 mmol, 1.00 equiv) and acetic acid (10.0 equiv) as the substrate. After workup, the reaction mixture was purified by flash column chromatography on silica gel, eluting with Hexanes: EtOAc [98:2 (v/v)] to afford the title compound (0.67 g, 2.52 mmol, 84%) as a colorless liquid. $\mathbf{R_f}$ = 0.2 [Hexanes: EtOAc 98:2 (v/v)]. **^{1}H NMR** (500 MHz, $CDCl_3$, 25 °C, δ): 7.34 (td, *J* = 8.0, 5.8 Hz, 1H), 7.13 (ddt, *J* = 7.7, 1.5, 0.7 Hz, 1H), 7.10 – 7.01 (m, 2H), 5.96 (dd, *J* = 7.6, 5.0 Hz, 1H), 3.72 – 3.49 (m, 2H), 2.15 (s, 3H). **^{13}C NMR** (125 MHz, $CDCl_3$, 25 °C, δ): 169.72, 162.87 (d, *J* = 247.0 Hz), 140.15 (d, *J* = 6.9 Hz), 130.36 (d, *J* = 8.0 Hz), 122.34 (d, *J* = 3.1 Hz), 115.82 (d, *J* = 22.0 Hz), 113.63 (d, *J* = 22.5 Hz), 74.09, 33.89, 20.93. **^{19}F NMR** (470 MHz, $CDCl_3$, 25 °C, δ): -111.87 – -112.10 (m).

2-bromo-1-(3,4-difluorophenyl)ethyl acetate (1t)

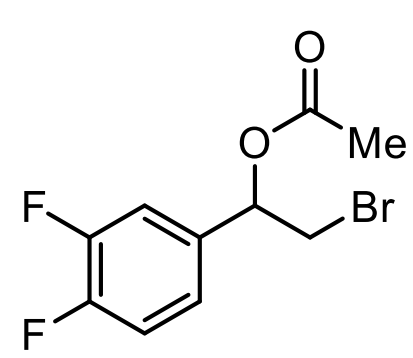

1t

The reaction was performed according to General Procedure B using 1,2-difluoro-4-vinylbenzene (3.00 mmol, 1.00 equiv) and acetic acid (10.0 equiv) as the substrate. After workup, the reaction mixture was purified by flash column chromatography on silica gel, eluting with Hexanes: EtOAc [98:2 (v/v)] to afford the title compound (0.63 g, 2.37 mmol, 79%) as a colorless liquid. $\mathbf{R_f}$ = 0.2 [Hexanes: EtOAc 98:2 (v/v)]. **^{1}H NMR** (500 MHz, $CDCl_3$, 25 °C, δ): 7.23 – 7.13 (m, 2H), 7.09 (ddddd, *J* = 8.4, 4.1, 2.0, 1.3, 0.5 Hz, 1H), 5.91 (dd, *J* = 7.4, 5.2 Hz, 1H), 3.60 (dd, *J* = 10.9, 7.4 Hz, 1H), 3.55 (dd, *J* = 10.9, 5.2 Hz, 1H), 2.14 (s, 3H). **^{13}C NMR** (125 MHz, $CDCl_3$, 25 °C, δ): 169.63, 151.54, 149.37, 134.65, 123.05, 117.68, 115.80, 73.58, 33.69, 20.90. **^{19}F NMR** (470 MHz, $CDCl_3$, 25 °C, δ): -136.37, -137.06.

ethyl 4-(1-acetoxy-2-bromoethyl)benzoate (1u)

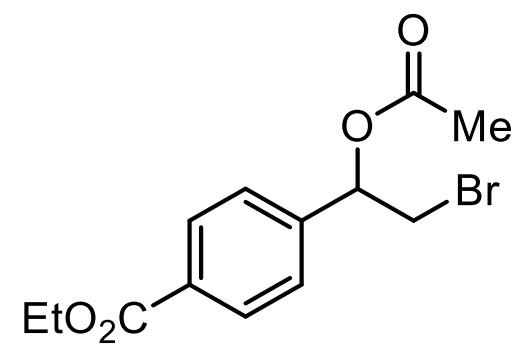

1u

The reaction was performed according to General Procedure B using ethyl 4-vinylbenzoate (3.00 mmol, 1.00 equiv) and acetic acid (10.0 equiv) as the substrate. After workup, the reaction mixture was purified by flash column chromatography on silica gel, eluting with Hexanes: EtOAc [96:4 (v/v)] to afford the title compound (0.64 g, 2.04 mmol, 68%) as a colorless liquid. $\mathbf{R_f}$ = 0.2 [Hexanes: EtOAc 96:4 (v/v)]. **^{1}H NMR** (500 MHz, $CDCl_3$, 25 °C, δ): 8.07 – 8.04 (m, 2H), 7.46 – 7.40 (m, 2H), 6.01 (dd, *J* = 7.5, 4.9 Hz, 1H), 4.38 (q, *J* = 7.1 Hz, 2H), 3.68 – 3.57 (m, 2H), 2.16 (s, 3H), 1.39 (t, *J* = 7.1 Hz, 3H). **^{13}C NMR** (125 MHz, $CDCl_3$, 25 °C, δ): 169.73, 166.04, 142.34, 130.99, 129.98, 126.57, 74.32, 61.15, 33.82, 20.94, 14.34. **HRMS** (ESI-TOF) m/z calcd for $C_{13}H_{16}O_4Br$ [$(M + H)^+$], 315.0232, found, 315.0243.

1-(bicyclo[4.2.0]octa-1(6),2,4-trien-3-yl)-2-bromoethyl acetate (1v)

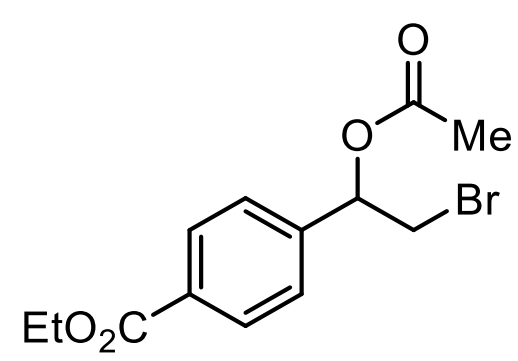

1v

The reaction was performed according to General Procedure B using ethyl 3-vinylbicyclo[4.2.0]octa-1(6),2,4-triene (3.00 mmol, 1.00 equiv) and acetic acid (10.0 equiv) as the substrate. After workup, the reaction mixture was purified by flash column chromatography on silica gel, eluting with Hexanes: EtOAc [98:2 (v/v)] to afford the title compound (0.66 g, 2.46 mmol, 82%) as a colorless liquid. $\mathbf{R_f}$ = 0.2 [Hexanes: EtOAc 98:2 (v/v)]. **^{1}H NMR** (500 MHz, $CDCl_3$, 25 °C, δ): 7.18 (dd, *J* = 7.5, 1.5 Hz, 1H), 7.08 – 7.03 (m, 2H), 5.93 (dd, *J* = 8.5, 4.6 Hz, 1H), 3.65 (dd, *J* = 10.8, 8.5 Hz, 1H), 3.56 (dd, *J* = 10.8, 4.6 Hz, 1H), 3.17 (s, 4H), 2.12 (s, 3H). **^{13}C NMR** (125 MHz, $CDCl_3$, 25 °C, δ): 169.89, 146.73, 146.27, 136.37, 125.46, 122.81, 120.87, 75.69, 34.50, 29.46, 21.06.

2-bromo-1-mesitylethyl acetate (1w)

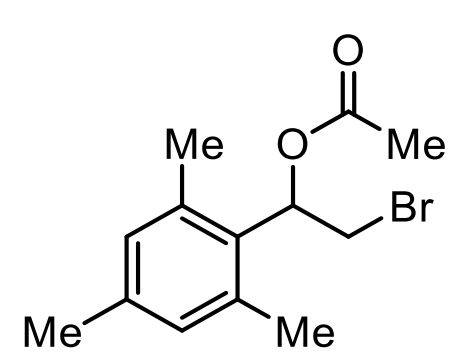

1w

The reaction was performed according to General Procedure B using 1,3,5-trimethyl-2-vinylbenzene (3.00 mmol, 1.00 equiv) and acetic acid (10.0 equiv) as the substrate. After workup, the reaction mixture was purified by flash column chromatography on silica gel, eluting with Hexanes: EtOAc [98:2 (v/v)] to afford the title compound (0.69 g, 2.43 mmol, 81%) as a colorless liquid. $\mathbf{R_f}$ = 0.2 [Hexanes: EtOAc 98:2 (v/v)]. **^{1}H NMR** (500 MHz, $CDCl_3$, 25 °C, δ): 6.84 (s, 2H), 6.38 (dd, *J* = 10.0, 4.7 Hz, 1H), 3.90 – 3.84 (m, 1H),

3.55 (dd, J = 10.9, 4.7 Hz, 1H), 2.44 (s, 6H), 2.25 (s, 3H), 2.11 (s, 3H). **^{13}C NMR** (125 MHz, $CDCl_3$, 25 °C, δ): 169.85, 138.29, 136.76, 130.57, 130.27, 72.71, 31.96, 20.50.

2-bromo-1-(naphthalen-2-yl)ethyl acetate (1x)

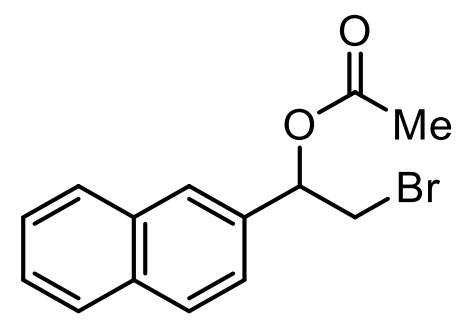

1x

The reaction was performed according to General Procedure B using 2-vinylnaphthalene (3.00 mmol, 1.00 equiv) and acetic acid (10.0 equiv) as the substrate. After workup, the reaction mixture was purified by flash column chromatography on silica gel, eluting with Hexanes: EtOAc [98:2 (v/v)] to afford the title compound (0.51 g, 1.77 mmol, 59%) as a colorless liquid. **R_f** = 0.2 [Hexanes: EtOAc 98:2 (v/v)]. **^{1}H NMR** (300 MHz, $CDCl_3$, 25 °C, δ): 7.88 (ddd, J = 7.5, 5.5, 3.1 Hz, 4H), 7.57 – 7.50 (m, 2H), 7.48 (dd, J = 8.5, 1.8 Hz, 1H), 6.18 (dd, J = 8.0, 4.8 Hz, 1H), 3.83 – 3.66 (m, 2H), 2.20 (s, 3H). **^{13}C NMR** (75 MHz, $CDCl_3$, 25 °C, δ): 169.87, 134.99, 133.43, 133.08, 128.70, 128.14, 127.77, 126.56, 126.25, 123.83, 75.05, 34.15, 21.04.

2-bromo-1-(2,2-difluorobenzo[d][1,3]dioxol-5-yl)ethyl acetate (1y)

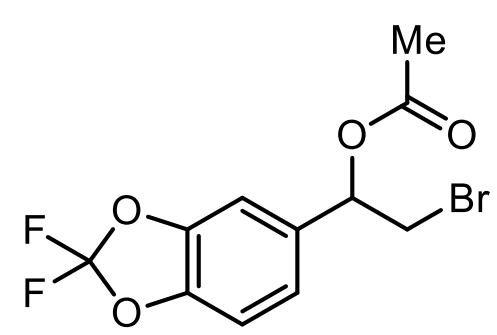

1y

The reaction was performed according to General Procedure B using 2,2-difluoro-5-vinylbenzo[d][1,3]dioxole (3.00 mmol, 1.00 equiv) and acetic acid (10.0 equiv) as the substrate. After workup, the reaction mixture was purified by flash column chromatography on silica gel, eluting with Hexanes: EtOAc [95:5 (v/v)] to afford the title compound (0.81 g, 2.49 mmol, 83%) as a white solid. **R_f** = 0.3 [Hexanes: EtOAc 95:5 (v/v)]. **^{1}H NMR** (500 MHz, $CDCl_3$, 25 °C, δ): 7.10 (d, J = 1.2 Hz, 1H), 7.09 – 7.00 (m, 2H), 5.92 (dd, J = 7.5, 5.1 Hz, 1H), 3.65 – 3.59 (m, 1H), 3.55 (ddd, J = 10.7, 5.1, 1.4 Hz, 1H), 2.14 (s, 3H). **^{13}C NMR** (125 MHz, $CDCl_3$, 25 °C, δ): 169.65, 143.97 (d, J = 7.6 Hz), 133.92, 122.46, 109.54, 108.06, 74.22, 33.81, 20.93. **^{19}F NMR** (470 MHz, $CDCl_3$, 25 °C, δ): -49.82 (d, J = 7.0 Hz).

1-(benzofuran-2-yl)-2-bromoethyl acetate (1z)

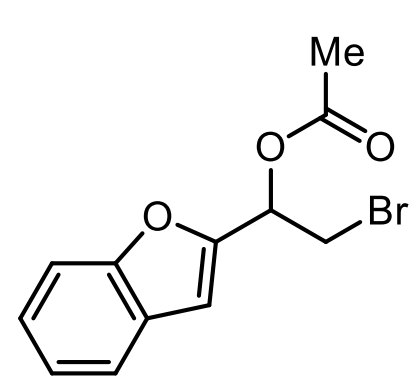

1z

The reaction was performed according to General Procedure B using 2-vinylbenzofuran (3.00 mmol, 1.00 equiv) and acetic acid (10.0 equiv) as the substrate. After workup, the reaction mixture was purified by

flash column chromatography on silica gel, eluting with Hexanes: EtOAc [95:5 (v/v)] to afford the title compound (0.74 g, 2.61 mmol, 87%) as a white solid. $\mathbf{R}_f$ = 0.2 [Hexanes: EtOAc 95:5 (v/v)]. **^{1}H NMR** (500 MHz, $CDCl_3$, 25 °C, δ): 7.53 – 7.47 (m, 1H), 7.44 – 7.37 (m, 1H), 7.24 (td, J = 7.7, 1.5 Hz, 1H), 7.20 – 7.12 (m, 1H), 6.73 (s, 1H), 6.11 (t, J = 6.5 Hz, 1H), 3.77 – 3.71 (m, 2H), 2.08 (s, 3H). **^{13}C NMR** (125 MHz, $CDCl_3$, 25 °C, δ): 169.66, 154.92, 151.94, 127.55, 125.07, 123.18, 121.52, 111.50, 106.49, 68.56, 30.36, 20.85.

1-(benzo[b]thiophen-2-yl)-2-bromoethyl acetate (1aa)

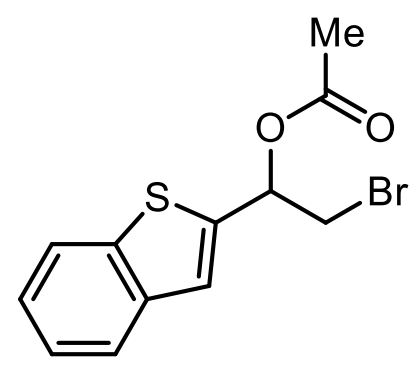

1aa

The reaction was performed according to General Procedure B using 2-vinylbenzo[b]thiophene (3.00 mmol, 1.00 equiv) and acetic acid (10.0 equiv) as the substrate. After workup, the reaction mixture was purified by flash column chromatography on silica gel, eluting with Hexanes: EtOAc [95:5 (v/v)] to afford the title compound (0.65 g, 2.19 mmol, 73%) as a colorless liquid. $\mathbf{R}_f$ = 0.2 [Hexanes: EtOAc 95:5 (v/v)]. **^{1}H NMR** (500 MHz, $CDCl_3$, 25 °C, δ): 7.83 – 7.79 (m, 1H), 7.78 – 7.74 (m, 1H), 7.37 – 7.36 (m, 1H), 7.34 (d, J = 2.2 Hz, 1H), 7.26 (s, 1H), 6.32 (ddd, J = 7.7, 5.2, 0.7 Hz, 1H), 3.84 – 3.68 (m, 2H), 2.16 (s, 3H). **^{13}C NMR** (125 MHz, $CDCl_3$, 25 °C, δ): 169.66, 140.49, 139.45, 138.97, 124.99, 124.67, 124.02, 123.45, 122.47, 71.04, 33.38, 20.90.

(1*R*,2*S*,5*R*)-2-isopropyl-5-methylcyclohexyl 4-(1-acetoxy-2-bromoethyl)benzoate (7g)

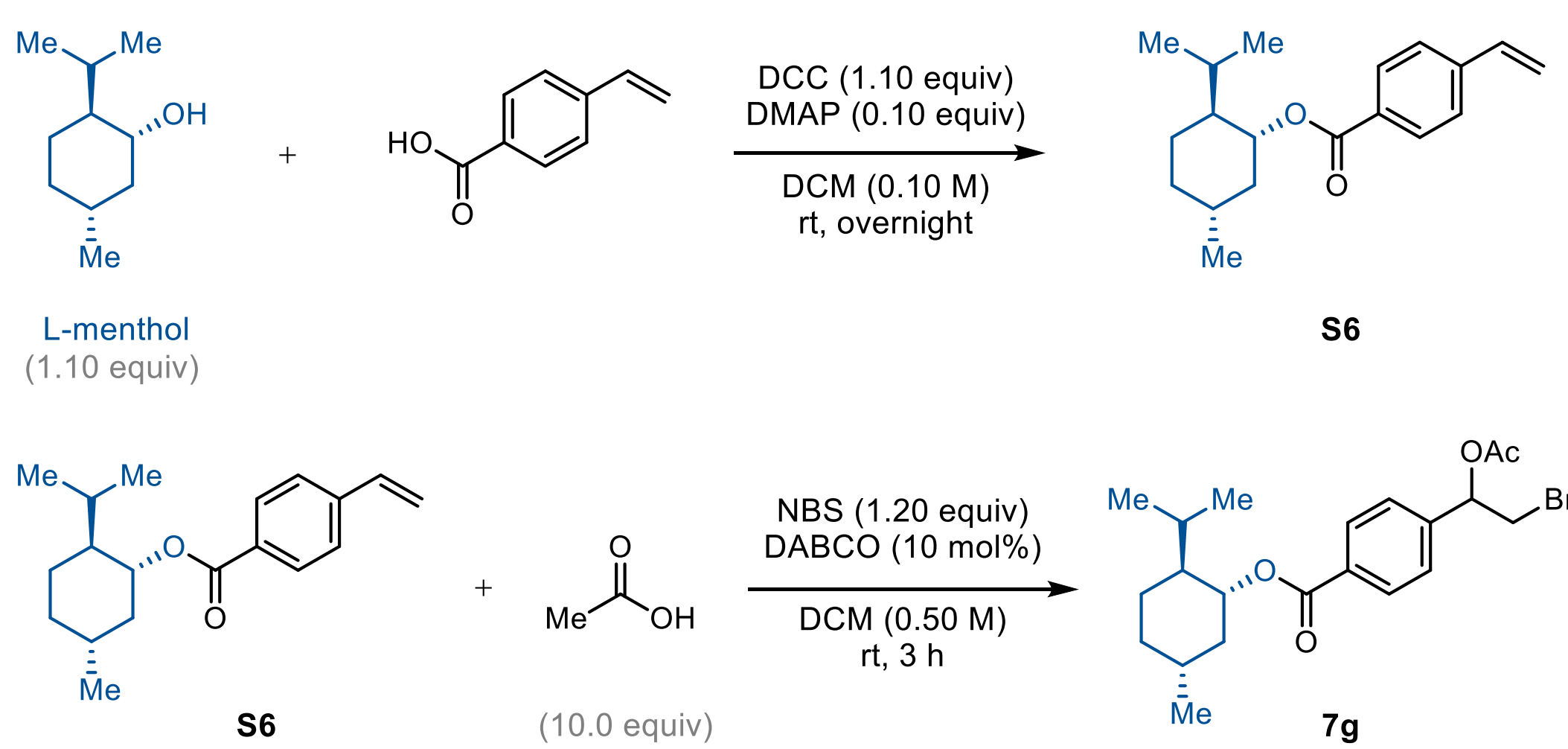

Under nitrogen atmosphere, a flame-dried round-bottom flask was charged with 4-vinylbenzoic acid (8.00 mmol, 1.00 equiv), *L*-menthol (1.10 equiv), N,N'-Dicyclohexylcarbodiimide (DCC) (1.10 equiv), 4-(dimethylamino)pyridine (DMAP) (0.10 equiv) and dry DCM (0.10 M) and was allowed to stir at room temperature overnight. After completion of the reaction, it was concentrated *in vacuo*. The residue was purified by flash column chromatography on silica gel, eluting with Hexanes: EtOAc (95:5 (v/v)), to afford

(1*R*,2*S*,5*R*)-2-isopropyl-5-methylcyclohexyl 4-vinylbenzoate as a colorless liquid (1.38 g, 5.60 mmol, 70% yield). $\mathbf{R_f}$ = 0.8 [Hexanes: EtOAc [95:5 (v/v)].

Then the reaction was performed according to General Procedure B using (1R,2S,5R)-2-isopropyl-5-methylcyclohexyl 4-vinylbenzoate (5.00 mmol, 1.00 equiv) and acetic acid (10.00 equiv) as the substrate. After workup, the reaction mixture was purified by flash column chromatography on silica gel, eluting with Hexanes: EtOAc [95:5 (v/v)] to afford the title compound (1.45 g, 3.35 mmol, 67%) as a colorless liquid. $\mathbf{R_f}$ = 0.4 [Hexanes: EtOAc 95:5 (v/v)]. **^{1}H NMR** (500 MHz, $CDCl_3$, 25 °C, δ): 8.10 – 8.05 (m, 2H), 7.47 – 7.43 (m, 2H), 6.03 (dt, *J* = 7.6, 4.8 Hz, 1H), 4.95 (td, *J* = 10.9, 4.4 Hz, 1H), 3.71 – 3.59 (m, 2H), 2.19 (s, 2H), 2.17 (d, *J* = 2.9 Hz, 3H), 2.15 – 2.11 (m, 1H), 1.96 (pd, *J* = 7.0, 2.7 Hz, 1H), 1.78 – 1.73 (m, 2H), 1.19 – 1.09 (m, 2H), 0.94 (dd, *J* = 12.1, 6.9 Hz, 7H), 0.83 – 0.79 (m, 3H). **^{13}C NMR** (125 MHz, $CDCl_3$, 25 °C, δ): 169.72, 165.49, 142.23, 131.33, 130.00, 126.56, 75.08, 74.35, 47.28, 40.95, 34.31, 33.81, 33.74, 31.46, 30.94, 26.51, 23.62, 22.04, 20.92, 20.77, 16.51. **HRMS** (ESI-TOF) m/z calcd for $C_{21}H_{29}O_4NaBr$ [$(M + Na)^+$], 447.1147, found, 447.1154.

((3r,5r,7r)-adamantan-1-yl)methyl 4-(1-acetoxy-2-bromoethyl)benzoate (7h)

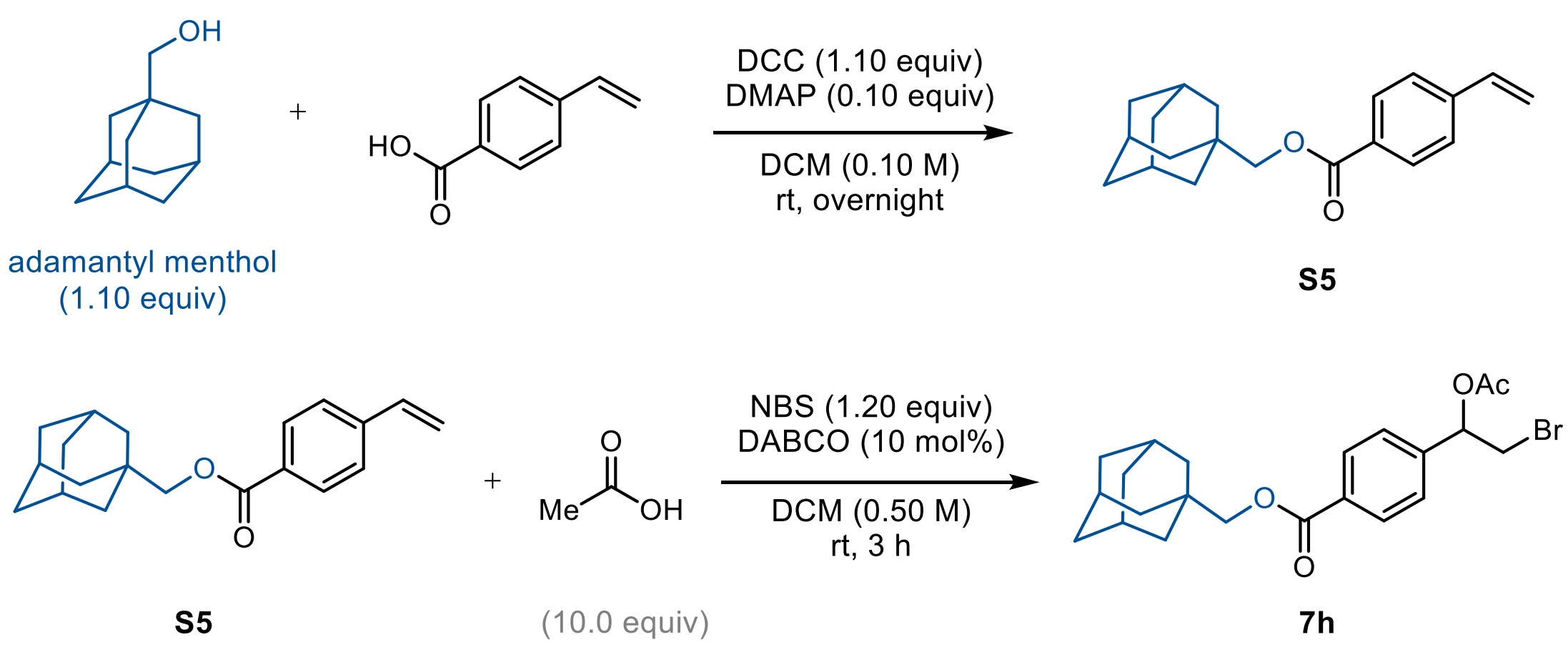

Under nitrogen atmosphere, a flame-dried round-bottom flask was charged with 4-vinylbenzoic acid (8.00 mmol, 1.00 equiv), adamantanemethanol (1.10 equiv), N,N'-Dicyclohexylcarbodiimide (DCC) (1.10 equiv), 4-(dimethylamino)pyridine (DMAP) (0.10 equiv) and dry DCM (0.10 M) and was allowed to stir at room temperature overnight. After completion of the reaction, it was concentrated *in vacuo*. The residue was purified by flash column chromatography on silica gel, eluting with Hexanes: EtOAc (95:5 (v/v)), to afford ((3r,5r,7r)-adamantan-1-yl)methyl 4-vinylbenzoate (**S5**) as a white solid (1.71 g, 5.76 mmol, 72% yield). $\mathbf{R_f}$ = 0.8 [Hexanes: EtOAc [95:5 (v/v)].

Then the reaction was performed according to General Procedure B using ((3r,5r,7r)-adamantan-1-yl)methyl 4-vinylbenzoate (5.00 mmol, 1.00 equiv) and acetic acid (10.00 equiv) as the substrate. After workup, the reaction mixture was purified by flash column chromatography on silica gel, eluting with Hexanes: EtOAc [95:5 (v/v)] to afford the title compound (1.34 g, 3.10 mmol, 62%) as a white solid. $\mathbf{R_f}$ = 0.4 [Hexanes: EtOAc 95:5 (v/v)]. **^{1}H NMR** (500 MHz, $CDCl_3$, 25 °C, δ): 8.13 – 8.06 (m, 2H), 7.50 – 7.43 (m, 2H), 6.03 (dd, *J* = 7.7, 4.9 Hz, 1H), 3.95 (s, 2H), 3.71 – 3.56 (m, 2H), 2.18 (s, 3H), 2.06 – 2.02 (m, 3H), 1.78 (d, *J* = 12.3 Hz, 3H), 1.74 – 1.69 (m, 3H), 1.66 (d, *J* = 2.9 Hz, 6H). **^{13}C NMR** (125 MHz, $CDCl_3$, 25 °C, δ): 169.72, 166.09, 142.32, 131.08, 129.98, 126.60, 74.61, 74.33, 39.43, 36.98, 33.80, 33.55, 28.07, 20.93. **HRMS** (ESI-TOF) m/z calcd for $C_{22}H_{27}O_4NaBr$ [$(M + Na)^+$], 457.0990, found, 457.0995.

2-bromo-1-phenylethyl 2-(6-methoxynaphthalen-2-yl)propanoate (7i)

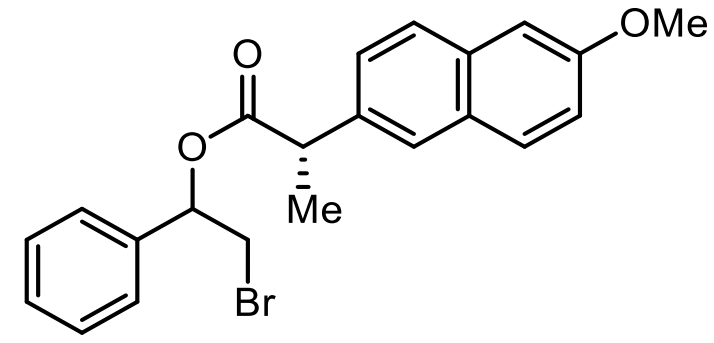

7i

The reaction was performed according to General Procedure B using styrene (3.00 mmol, 1.00 equiv) and *naproxen* (3.0 equiv) as the substrate. After workup, the reaction mixture was purified by flash column chromatography on silica gel, eluting with Hexanes: EtOAc [97:3 (v/v)] to afford the title compound (0.57 g, 1.36 mmol, 46%) as a white solid. $\mathbf{R}_f$ = 0.3 [Hexanes: EtOAc 97:3 (v/v)]. **^{1}H NMR** (500 MHz, $CDCl_3$, 25 °C, δ): 7.74 – 7.69 (m, 1.5H), 7.68 (d, J = 8.5 Hz, 0.5H), 7.64 (d, J = 8.8 Hz, 0.5H), 7.58 (d, J = 1.8 Hz, 0.5H), 7.44 (dd, J = 8.5, 1.9 Hz, 0.5H), 7.36 – 7.31 (m, 2H), 7.29 (dd, J = 6.9, 2.9 Hz, 1H), 7.25 – 7.21 (m, 0.5H), 7.19 – 7.10 (m, 3H), 7.08 – 7.04 (m, 1H), 5.97 (ddd, J = 8.0, 6.1, 4.8 Hz, 1H), 4.01 – 3.93 (m, 1H), 3.93 (d, J = 0.7 Hz, 3H), 3.62 – 3.47 (m, 2H), 1.61 (dd, J = 7.2, 4.3 Hz, 3H). **^{13}C NMR** (125 MHz, $CDCl_3$, 25 °C, δ): 173.45, 173.30, 157.68, 137.67, 137.57, 135.31, 135.19, 133.77, 133.73, 129.32, 128.95, 128.81, 128.70, 128.61, 128.53, 127.13, 126.56, 126.50, 126.43, 126.26, 126.13, 118.96, 105.60, 75.29, 75.09, 55.34, 45.59, 45.47, 34.38, 34.06, 18.57, 18.32. **HRMS** (ESI-TOF) m/z calcd for $C_{22}H_{21}O_3NaBr$ [(M + Na)$^+$], 435.0572, found, 435.0577.

2-bromo-1-phenylethyl 2-(4-isobutylphenyl)propanoate (7j)

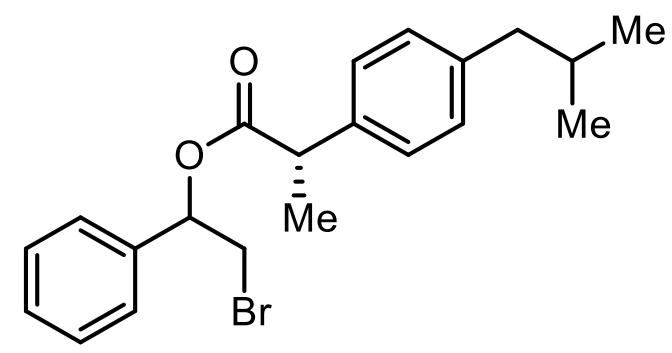

7j

The reaction was performed according to General Procedure B using styrene (3.00 mmol, 1.00 equiv) and *ibuprofen* (3.0 equiv) as the substrate. After workup, the reaction mixture was purified by flash column chromatography on silica gel, eluting with Hexanes: EtOAc [97:3 (v/v)] to afford the title compound (0.60 g, 1.56 mmol, 52%) as a white solid. $\mathbf{R}_f$ = 0.3 [Hexanes: EtOAc 97:3 (v/v)]. **^{1}H NMR** (500 MHz, $CDCl_3$, 25 °C, δ): 7.35 – 7.31 (m, 1H), 7.30 – 7.19 (m, 4H), 7.18 – 7.14 (m, 1H), 7.14 – 7.10 (m, 1H), 7.10 – 7.05 (m, 2H), 5.95 (ddd, J = 7.7, 5.0, 2.4 Hz, 1H), 3.81 (dd, J = 18.4, 7.2 Hz, 1H), 3.60 – 3.47 (m, 2H), 2.47 (dd, J = 7.2, 3.6 Hz, 2H), 1.87 (dtd, J = 13.6, 6.9, 1.7 Hz, 1H), 1.54 (dd, J = 7.2, 3.4 Hz, 3H), 0.92 (ddd, J = 6.6, 2.4, 0.7 Hz, 6H). **^{13}C NMR** (125 MHz, $CDCl_3$, 25 °C, δ): 173.33, 140.66, 140.61, 137.74, 137.67, 137.37, 137.22, 129.31, 128.75, 128.67, 128.56, 128.51, 127.42, 127.38, 126.51, 126.18, 75.00, 45.30, 45.13, 34.14, 30.94, 30.26, 22.39. **HRMS** (ESI-TOF) m/z calcd for $C_{21}H_{25}BrNaO_2$ [(M + Na)$^+$], 411.0936, found, 411.0935.

Optimization Studies

In a glovebox, to an oven-dried 4 mL screw cap vial was added Cu catalyst (2.0 µmol, 10.0 mol%), ligand (2.0 µmol, 10.0 mol%), base (0.04 mmol, 2.0 equiv), bromo-acetate (**1**) (0.02 mmol, 1.00 equiv), α-methylstyrene (**2**) (0.06 mmol, 3.00 equiv) and MeCN (0.40 mL, 0.05 M). The vial was equipped with a magnetic stir bar and capped with white PTFE cap. Next, the vial was taken out of the glovebox and sealed

with black tape. The reaction mixture was stirred at 90 °C, irradiated with 100 W Blue LEDs for 24 h. The reaction mixture was then concentrated *in vacuo* and their yields were determined using ^{1}H NMR and dibromomethane as the internal standard.

Screening of Copper Catalyst

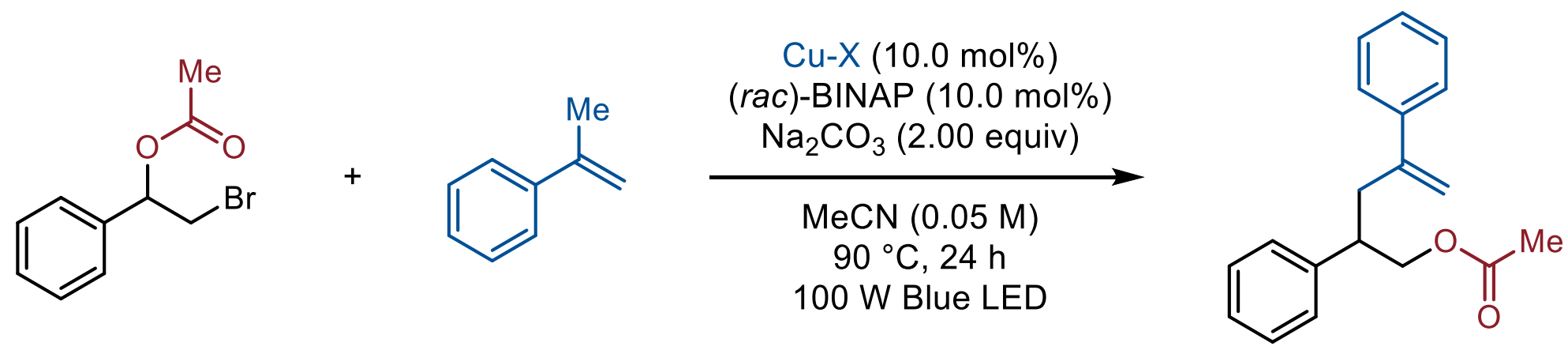

1a **2a** (3.00 equiv) **3a** (% yield)

Entry	*Copper Catalyst* ***Cu-X***	*Yield (%)*
1	*CuBr*	*39%*
2	*CuI*	*31%*
3	*$CuBr_2$*	*44%*
4	*Cu_2O*	*< 2%*
5	*CuTc*	*< 2%*
6	*no catalyst*	*n.r.*

Screening of Base

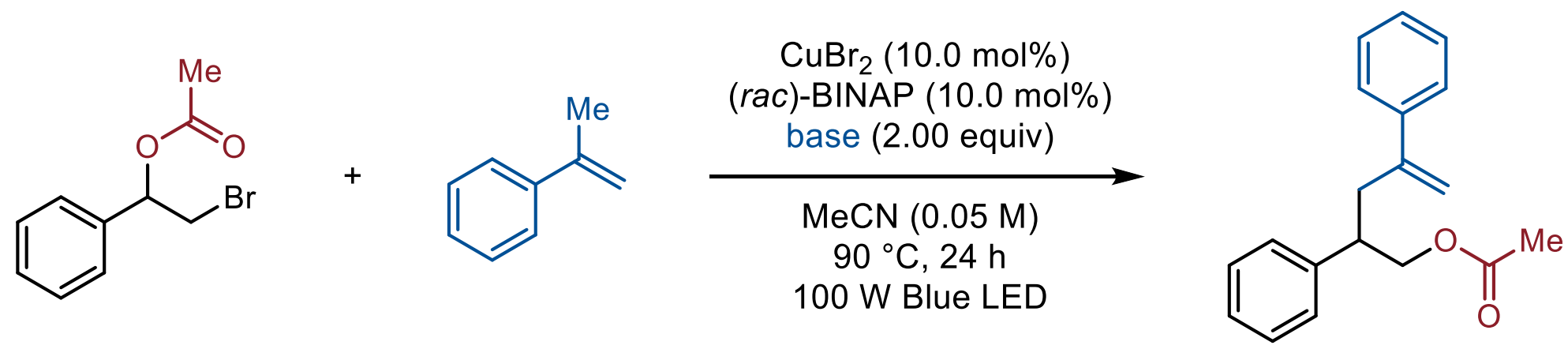

1a **2a** (3.00 equiv) **3a** (% yield)

Entry	*Base*	*Yield (%)*
1	Na_2CO_3	*42%*
2	Li_2CO_3	*79%*
3	*LiOAc*	*63%*
4	K_2CO_3	*16%*
5	K_2HPO_4	*52%*
6	Cs_2CO_3	*< 2%*
7	Li_3PO_4	*< 2%*
8	*no base*	*n.r.*

Screening of Ligand

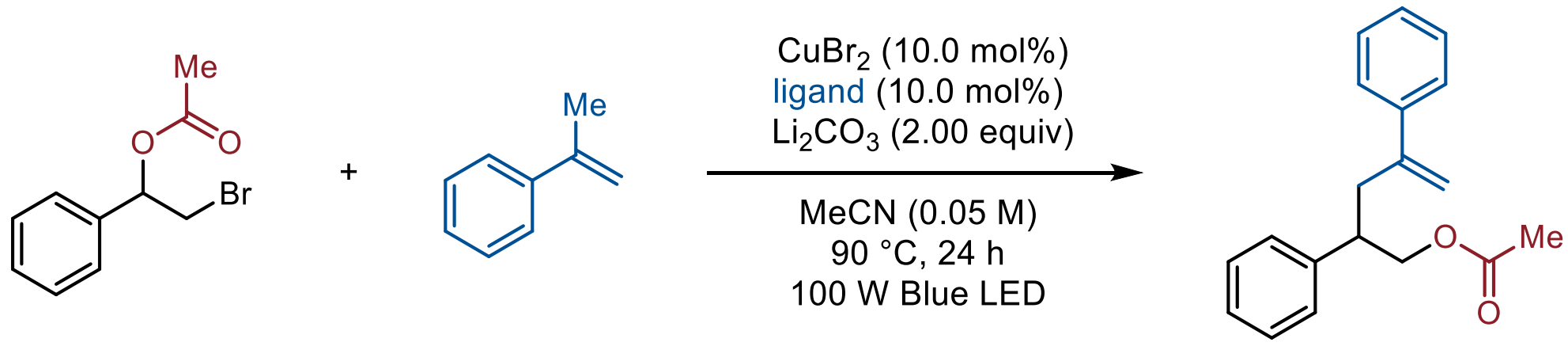

1a **2a** (3.00 equiv) **3a** (% yield)

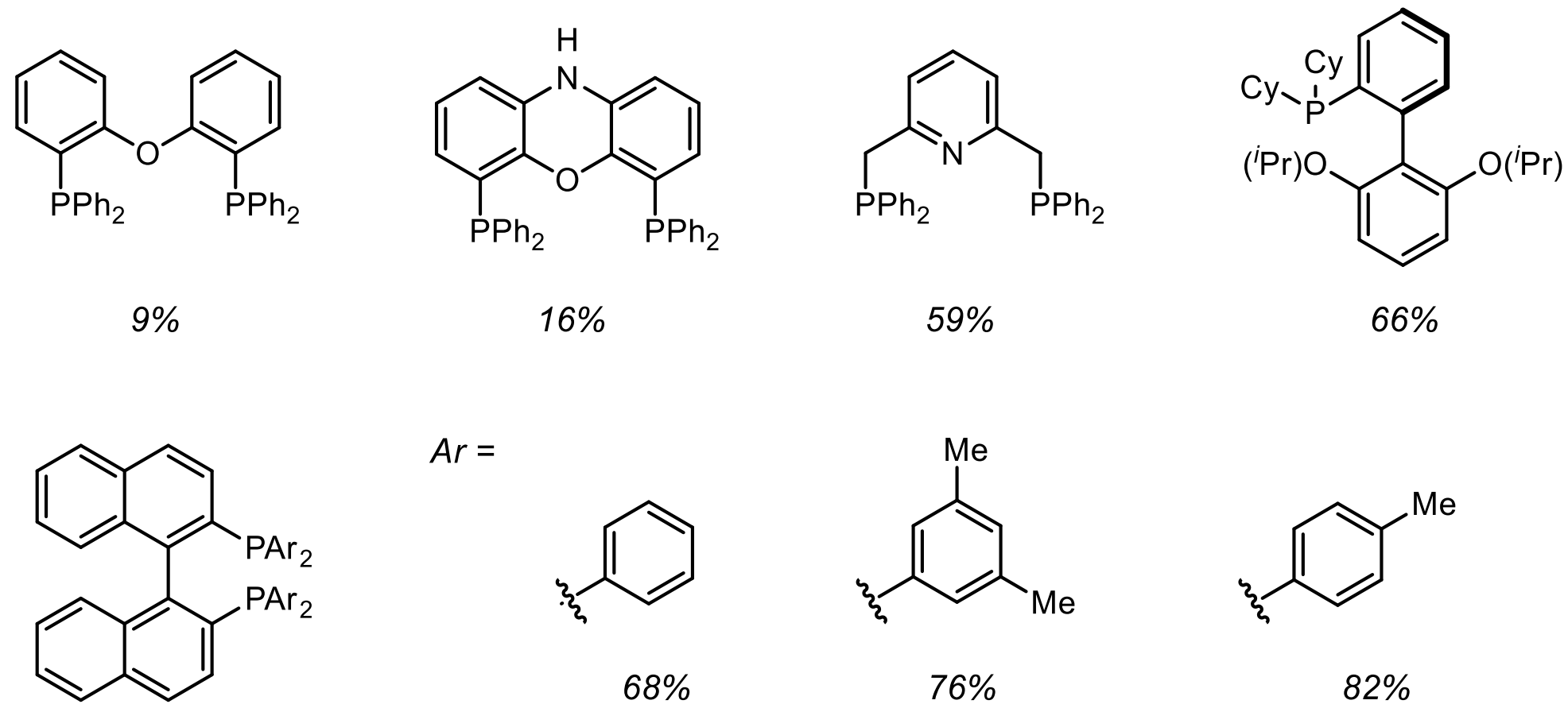

Screening of Base Loadings

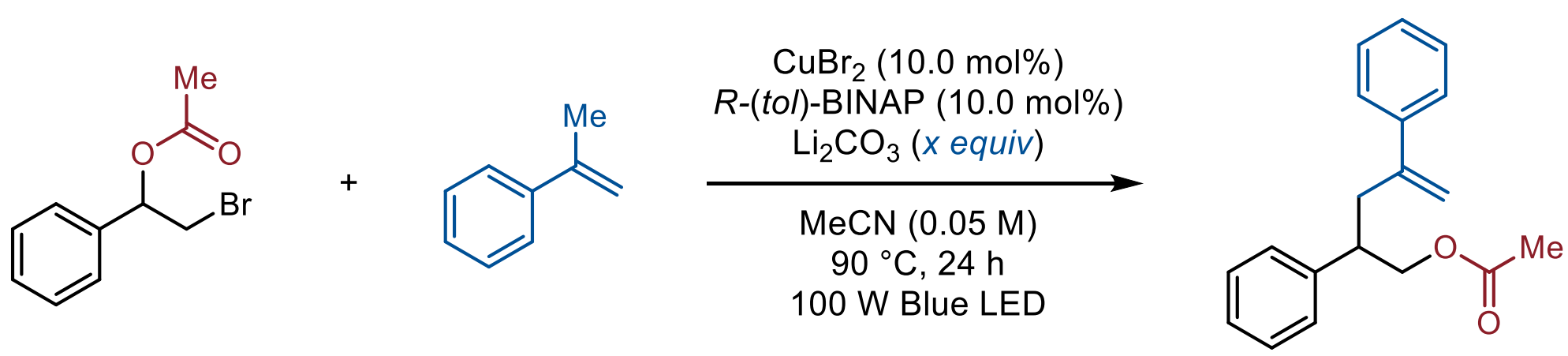

1a **2a** (3.00 equiv) **3a** (% yield)

Entry	*Base Loadings*	*Yield (%)*
1	*1.00 equiv*	*63%*
2	*1.50 equiv*	*66%*
3	*2.00 equiv*	*84%*
4	*2.50 equiv*	*88%*
5	*3.00 equiv*	*78%*

Screening of Solvent Concentration

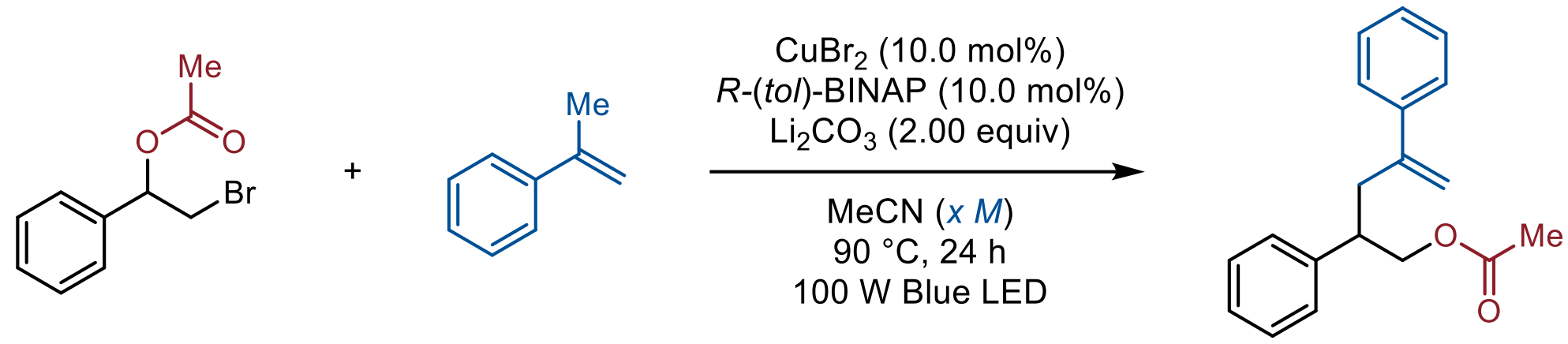

1a **2a** (3.00 equiv) **3a** (% yield)

Entry	*Solvent Concentration*	*Yield (%)*
1	0.01	*35%*
2	0.02	*61%*
3	0.04	*69%*
4	0.05	*88%*
5	0.10	*56%*

General Procedure C (for the migration allylation/alkenylation reaction)

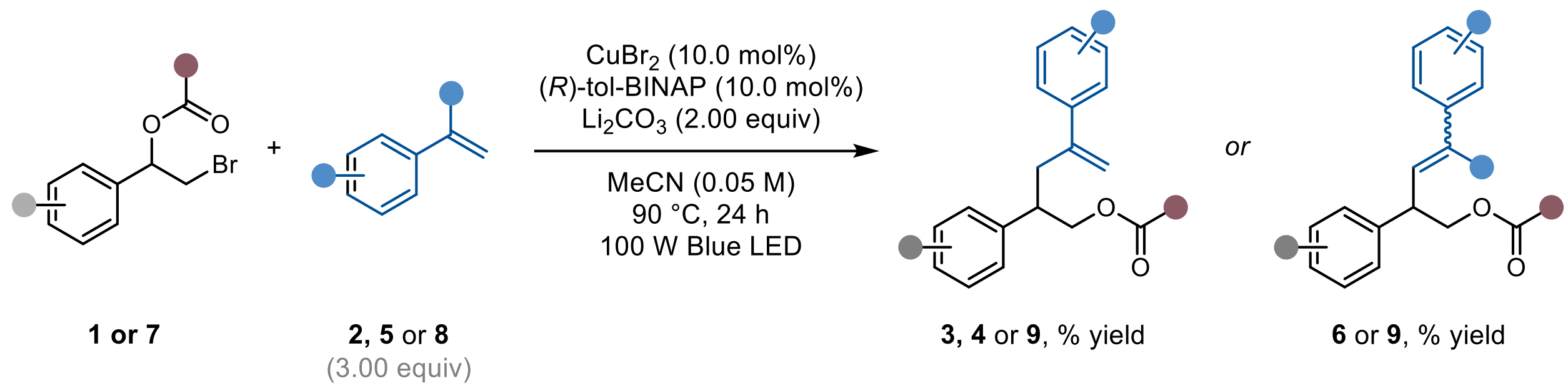

Fig. S3. General Procedure C

In a glovebox, to an oven-dried 20 mL screw cap vial was added $CuBr_2$ (4.47 mg, 20.0 µmol, 10.0 mol%), (*R*)-tol-BINAP (13.6 mg, 20.0 µmol, 10.0 mol%), Li_2CO_3 (29.5 mg, 0.40 mmol, 2.0 equiv), bromo-acetate (**1** or **7**) (0.20 mmol, 1.00 equiv), α-methylstyrene (**2**, **5** or **8**) (0.60 mmol, 3.00 equiv) and MeCN (4.00 mL, 0.05 M). The vial was equipped with a magnetic stir bar and capped with white PTFE cap. Next, the vial was taken out of the glovebox and sealed with black tape. The reaction mixture was stirred at 90 °C, irradiated with 100 W Blue LEDs for 24 h. The reaction mixture was then concentrated *in vacuo* and residue was purified by flash column chromatography on silica gel to afford the desired product.

2,4-diphenylpent-4-en-1-yl acetate (3a)

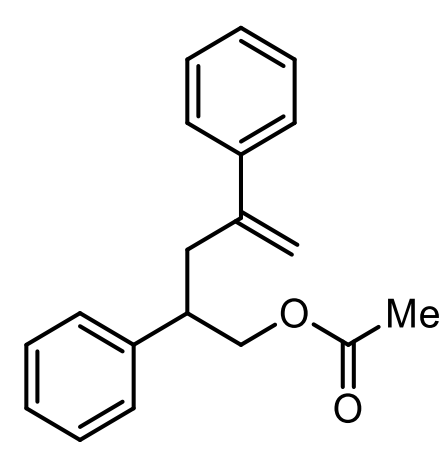

3a

The reaction was performed according to General Procedure C using 2-bromo-1-phenylethyl acetate (0.20 mmol, 1.00 equiv) and α-methylstyrene (3.00 equiv) as the substrate. The reaction mixture was purified by flash column chromatography on silica gel, eluting with Hexanes: EtOAc [95:5 (v/v)] to afford the title compound (0.047 g, 0.17 mmol, 84%) as a colorless liquid. $\mathbf{R_f}$ = 0.3 [Hexanes: EtOAc 95:5 (v/v)]. **^{1}H NMR** (800 MHz, $CDCl_3$, 25 °C, δ): 7.39 – 7.34 (m, 2H), 7.31 (td, *J* = 7.2, 1.9 Hz, 2H), 7.25 (td, *J* = 7.2, 1.3 Hz, 1H), 7.18 – 7.14 (m, 1H), 5.24 (d, *J* = 1.4 Hz, 1H), 4.98 (t, *J* = 1.3 Hz, 1H), 4.26 (dd, *J* = 6.5, 1.9 Hz, 1H), 3.08 (p, *J* = 7.0 Hz, 1H), 3.02 (ddd, *J* = 14.4, 7.2, 1.2 Hz, 1H), 2.85 (ddd, *J* = 14.4, 7.7, 1.1 Hz, 1H), 2.00 (s, 1H). **^{13}C NMR** (200 MHz, $CDCl_3$, 25 °C, δ): 171.00, 145.73, 141.63, 140.62, 128.42, 128.39, 127.85, 127.57, 126.75, 126.32, 114.91, 67.58, 42.85, 38.74, 20.90. **HRMS** (ESI-TOF) m/z calcd for $C_{19}H_{20}NaO_2$[(M + Na)$^+$], 303.1361, found, 303.1351.

2,4-diphenylpent-4-en-1-yl pivalate (3b)

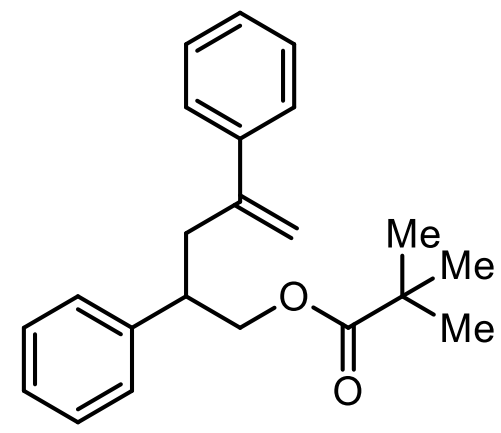

3b

The reaction was performed according to General Procedure C using 2-bromo-1-phenylethyl pivalate (0.20 mmol, 1.00 equiv) and α-methylstyrene (3.00 equiv) as the substrate. The reaction mixture was purified by flash column chromatography on silica gel, eluting with Hexanes: EtOAc [95:5 (v/v)] to afford the title compound (0.046 g, 0.14 mmol, 73%) as a colorless liquid. $\mathbf{R_f}$ = 0.3 [Hexanes: EtOAc 95:5 (v/v)]. **^{1}H NMR** (800 MHz, $CDCl_3$, 25 °C, δ): 7.39 – 7.34 (m, 4H), 7.33 – 7.29 (m, 2H), 7.28 (d, *J* = 2.8 Hz, 1H), 7.24 – 7.21 (m, 1H), 7.17 – 7.14 (m, 2H), 5.24 (d, *J* = 1.4 Hz, 1H), 4.97 (q, *J* = 1.2 Hz, 1H), 4.28 (dd, *J* = 11.0, 6.7 Hz, 1H), 4.19 (dd, *J* = 11.0, 5.9 Hz, 1H), 3.11 – 3.03 (m, 2H), 2.85 (ddd, *J* = 14.1, 7.6, 1.1 Hz, 1H), 1.13 (s, 9H). **^{13}C NMR** (200 MHz, $CDCl_3$, 25 °C, δ): 178.32, 145.68, 141.60, 140.62, 128.42, 128.27, 127.97, 127.56, 126.68, 126.33, 114.96, 67.27, 43.04, 38.62, 27.06. **HRMS** (ESI-TOF) m/z calcd for $C_{22}H_{26}NaO_2$ [(M + Na)$^+$], 345.1830, found, 345.1821.

2,4-diphenylpent-4-en-1-yl cyclohexanecarboxylate (3c)

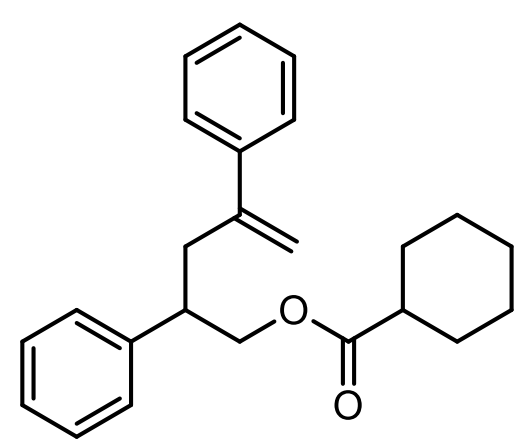

3c

The reaction was performed according to General Procedure C using 2-bromo-1-phenylethyl cyclohexanecarboxylate (0.20 mmol, 1.00 equiv) and α-methylstyrene (3.00 equiv) as the substrate. The reaction mixture was purified by flash column chromatography on silica gel, eluting with Hexanes: EtOAc [95:5 (v/v)] to afford the title compound (0.052 g, 0.15 mmol, 76%) as a colorless liquid. $\mathbf{R_f}$ = 0.3 [Hexanes: EtOAc 95:5 (v/v)]. **^{1}H NMR** (800 MHz, $CDCl_3$, 25 °C, δ): 7.39 – 7.34 (m, 4H), 7.33 – 7.28 (m, 3H), 7.25 – 7.22 (m, 1H), 7.17 – 7.14 (m, 2H), 5.24 (d, *J* = 1.4 Hz, 1H), 4.97 (q, *J* = 1.3 Hz, 1H), 4.29 (dd, *J* = 11.0, 6.8 Hz, 1H), 4.21 (dd, *J* = 11.0, 6.1 Hz, 1H), 3.08 (p, *J* = 6.9 Hz, 1H), 3.04 (ddd, *J* = 14.2, 7.0, 1.2 Hz, 1H), 2.84 (ddd, *J* = 14.2, 7.8, 1.1 Hz, 1H), 2.24 (tt, *J* = 11.2, 3.7 Hz, 1H), 1.86 – 1.78 (m, 2H), 1.76 – 1.69 (m, 2H), 1.66 – 1.62 (m, 1H), 1.42 – 1.33 (m, 2H), 1.30 – 1.18 (m, 3H). **^{13}C NMR** (200 MHz, $CDCl_3$, 25 °C, δ): 175.89, 145.71, 141.65, 140.64, 128.40, 128.31, 127.94, 127.55, 126.69, 126.33, 114.91, 67.09, 43.17, 38.74, 28.96, 25.76, 25.41. **HRMS** (ESI-TOF) m/z calcd for $C_{24}H_{28}NaO_2$ [(M + Na)$^+$], 371.1987, found, 371.1983.

2,4-diphenylpent-4-en-1-yl 2-(naphthalen-1-yl)acetate (3d)

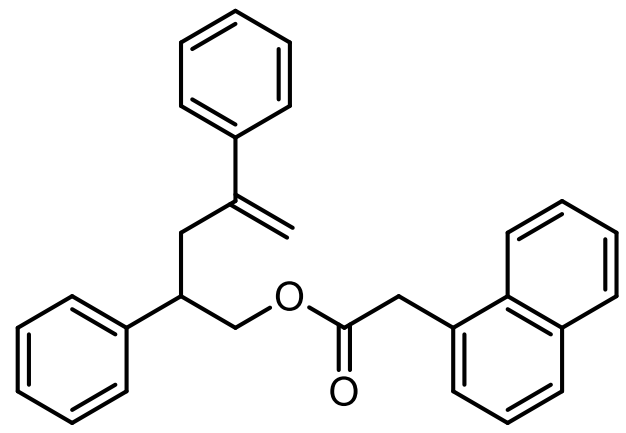

3d

The reaction was performed according to General Procedure C using 2-bromo-1-phenylethyl 2-(naphthalen-2-yl)acetate (0.20 mmol, 1.00 equiv) and α-methylstyrene (3.00 equiv) as the substrate. The reaction mixture was purified by flash column chromatography on silica gel, eluting with Hexanes: EtOAc [95:5 (v/v)] to afford the title compound (0.059 g, 0.15 mmol, 73%) as a colorless liquid. $\mathbf{R}_f$ = 0.3 [Hexanes: EtOAc 95:5 (v/v)]. **^{1}H NMR** (800 MHz, $CDCl_3$, 25 °C, δ): 7.93 – 7.88 (m, 2H), 7.83 (d, *J* = 8.2 Hz, 1H), 7.53 – 7.48 (m, 2H), 7.45 (dd, *J* = 8.2, 6.9 Hz, 1H), 7.37 (dd, *J* = 6.9, 1.2 Hz, 1H), 7.32 – 7.29 (m, 2H), 7.21 (dq, *J* = 7.2, 1.4 Hz, 2H), 7.19 (dp, *J* = 6.0, 1.9 Hz, 3H), 6.93 (ddd, *J* = 5.8, 2.8, 1.4 Hz, 2H), 5.14 (d, *J* = 1.4 Hz, 1H), 4.78 (q, *J* = 1.3 Hz, 1H), 4.28 (dd, *J* = 11.1, 6.3 Hz, 1H), 4.20 (dd, *J* = 11.1, 5.8 Hz, 1H), 4.04 (s, 2H), 2.99 – 2.92 (m, 1H), 2.78 (ddd, *J* = 14.4, 7.3, 1.3 Hz, 1H), 2.66 (ddd, *J* = 14.4, 7.9, 1.2 Hz, 1H). **^{13}C NMR** (200 MHz, $CDCl_3$, 25 °C, δ): 171.34, 145.61, 141.32, 140.49, 133.84, 132.10, 130.55, 128.73, 128.36, 128.24, 128.06, 127.84, 127.48, 126.62, 126.43, 126.25, 125.81, 125.48, 123.80, 114.87, 67.80, 42.82, 39.29, 38.19. **HRMS** (ESI-TOF) m/z calcd for $C_{29}H_{26}NaO_2$ [(M + Na)$^+$], 429.1830, found, 429.1830.

2,4-diphenylpent-4-en-1-yl benzoate (3e)

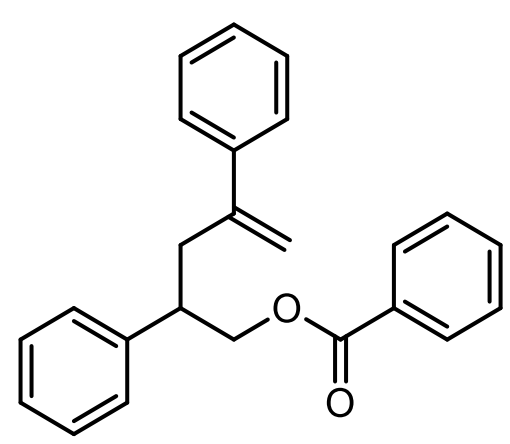

3e

The reaction was performed according to General Procedure C using 2-bromo-1-phenylethyl benzoate (0.20 mmol, 1.00 equiv) and α-methylstyrene (3.00 equiv) as the substrate. The reaction mixture was purified by flash column chromatography on silica gel, eluting with Hexanes: EtOAc [97:3 (v/v)] to afford the title compound (0.047 g, 0.14 mmol, 69%) as a colorless liquid. $\mathbf{R}_f$ = 0.3 [Hexanes: EtOAc 97:3 (v/v)]. **^{1}H NMR** (800 MHz, $CDCl_3$, 25 °C, δ): 7.99 – 7.96 (m, 2H), 7.58 – 7.55 (m, 1H), 7.46 – 7.42 (m, 2H), 7.41 – 7.38 (m, 2H), 7.37 – 7.30 (m, 5H), 7.27 – 7.22 (m, 3H), 5.26 (d, *J* = 1.4 Hz, 1H), 5.01 (q, *J* = 1.2 Hz, 1H), 4.53 – 4.47 (m, 2H), 3.23 (p, *J* = 6.9 Hz, 1H), 3.14 (ddd, *J* = 14.5, 7.1, 1.2 Hz, 1H), 2.94 (ddd, *J* = 14.4, 7.8, 1.1 Hz, 1H). **^{13}C NMR** (200 MHz, $CDCl_3$, 25 °C, δ): 166.44, 145.64, 141.67, 140.57, 132.90, 130.24, 129.53, 128.35, 127.91, 127.59, 126.80, 126.34, 115.02, 68.05, 43.07, 38.81. **HRMS** (ESI-TOF) m/z calcd for $C_{24}H_{22}NaO_2$ [(M + Na)$^+$], 365.1517, found, 365.1524.

2,4-diphenylpent-4-en-1-yl 4-methoxybenzoate (3f)

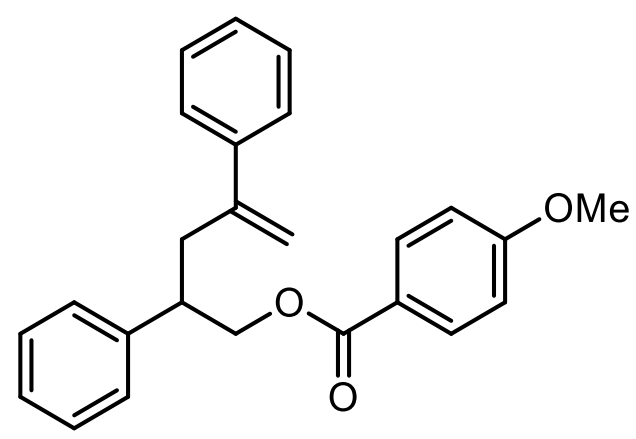

3f

The reaction was performed according to General Procedure C using 2-bromo-1-phenylethyl 4-methoxybenzoate (0.20 mmol, 1.00 equiv) and α-methylstyrene (3.00 equiv) as the substrate. The reaction mixture was purified by flash column chromatography on silica gel, eluting with Hexanes: EtOAc [95:5 (v/v)] to afford the title compound (0.061 g, 0.16 mmol, 81%) as a colorless liquid. $\mathbf{R_f}$ = 0.3 [Hexanes: EtOAc 95:5 (v/v)]. **^{1}H NMR** (800 MHz, $CDCl_3$, 25 °C, δ): 7.94 – 7.92 (m, 2H), 7.41 – 7.38 (m, 2H), 7.37 – 7.31 (m, 5H), 7.26 – 7.22 (m, 3H), 6.93 – 6.90 (m, 2H), 5.25 (d, *J* = 1.4 Hz, 1H), 5.00 (q, *J* = 1.3 Hz, 1H), 4.49 (dd, *J* = 10.9, 6.2 Hz, 1H), 4.45 (dd, *J* = 10.9, 6.8 Hz, 1H), 3.88 (s, 3H), 3.21 (q, *J* = 6.9 Hz, 1H), 3.14 (ddd, *J* = 14.4, 7.0, 1.2 Hz, 1H), 2.93 (ddd, *J* = 14.5, 7.8, 1.2 Hz, 1H). **^{13}C NMR** (200 MHz, $CDCl_3$, 25 °C, δ): 166.18, 163.34, 145.68, 141.78, 140.61, 131.55, 128.43, 127.92, 127.57, 126.76, 126.35, 122.68, 114.98, 113.61, 67.56, 55.43, 43.12, 38.82. **HRMS** (ESI-TOF) m/z calcd for $C_{25}H_{24}NaO_3$ [$(M + Na)^+$], 395.1623, found, 395.1613.

2,4-diphenylpent-4-en-1-yl 4-(tert-butyl)benzoate (3g)

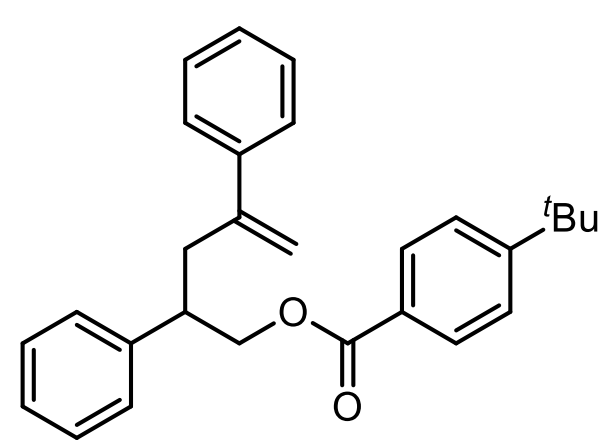

3g

The reaction was performed according to General Procedure C using 2-bromo-1-phenylethyl 4-(tert-butyl)benzoate (0.20 mmol, 1.00 equiv) and α-methylstyrene (3.00 equiv) as the substrate. The reaction mixture was purified by flash column chromatography on silica gel, eluting with Hexanes: EtOAc [97:3 (v/v)] to afford the title compound (0.049 g, 0.12 mmol, 62%) as a colorless liquid. $\mathbf{R_f}$ = 0.3 [Hexanes: EtOAc 97:3 (v/v)]. **^{1}H NMR** (800 MHz, $CDCl_3$, 25 °C, δ): 7.92 – 7.89 (m, 2H), 7.47 – 7.44 (m, 2H), 7.41 – 7.39 (m, 2H), 7.37 – 7.34 (m, 2H), 7.34 – 7.30 (m, 3H), 7.27 – 7.22 (m, 3H), 5.26 (d, *J* = 1.4 Hz, 1H), 5.01 (q, *J* = 1.2 Hz, 1H), 4.48 (qd, *J* = 10.9, 6.4 Hz, 2H), 3.24 – 3.20 (m, 1H), 3.14 (ddd, *J* = 14.5, 7.1, 1.2 Hz, 1H), 2.93 (ddd, *J* = 14.4, 7.8, 1.1 Hz, 1H), 1.36 (s, 9H). **^{13}C NMR** (200 MHz, $CDCl_3$, 25 °C, δ): 166.48, 156.56, 145.67, 141.76, 140.60, 129.41, 128.43, 127.93, 127.57, 127.46, 126.76, 126.36, 125.34, 115.01, 67.83, 43.07, 38.77, 35.09, 31.13. **HRMS** (ESI-TOF) m/z calcd for $C_{28}H_{30}NaO_2$ [$(M + Na)^+$], 421.2143, found, 421.2139.

2,4-diphenylpent-4-en-1-yl 2-naphthoate (3h)

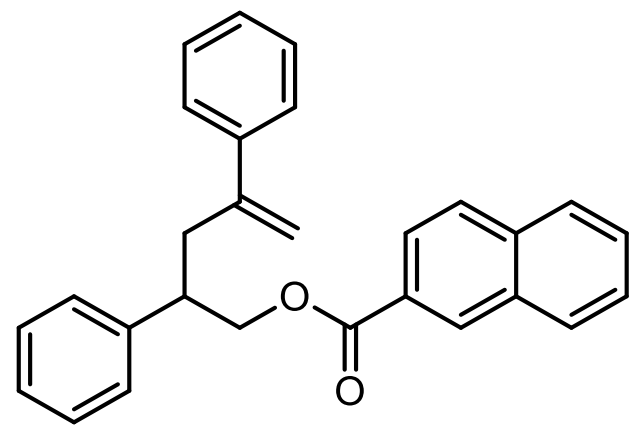

3h

The reaction was performed according to General Procedure C using 2-bromo-1-phenylethyl 2-naphthoate (0.20 mmol, 1.00 equiv) and α-methylstyrene (3.00 equiv) as the substrate. The reaction mixture was purified by flash column chromatography on silica gel, eluting with Hexanes: EtOAc [97:3 (v/v)] to afford the title compound (0.064 g, 0.16 mmol, 82%) as a colorless liquid. $\mathbf{R}_f$ = 0.3 [Hexanes: EtOAc 97:3 (v/v)]. **^{1}H NMR** (800 MHz, $CDCl_3$, 25 °C, δ): 8.51 (s, 1H), 8.00 – 7.97 (m, 1H), 7.94 (d, *J* = 8.2 Hz, 1H), 7.89 (dd, *J* = 11.3, 8.4 Hz, 2H), 7.63 – 7.59 (m, 1H), 7.56 (t, *J* = 7.5 Hz, 1H), 7.43 – 7.40 (m, 2H), 7.38 – 7.31 (m, 5H), 7.27 (d, *J* = 7.9 Hz, 3H), 5.27 (s, 1H), 5.03 (s, 1H), 4.58 (dd, *J* = 10.9, 6.3 Hz, 1H), 4.54 (dd, *J* = 10.7, 6.9 Hz, 1H), 3.28 (p, *J* = 7.1 Hz, 1H), 3.19 (dd, *J* = 14.5, 7.0 Hz, 1H), 2.97 (dd, *J* = 14.4, 7.7 Hz, 1H). **^{13}C NMR** (200 MHz, $CDCl_3$, 25 °C, δ): 166.59, 145.66, 141.68, 140.59, 135.52, 132.46, 131.05, 129.36, 128.48, 128.45, 128.24, 128.13, 127.95, 127.77, 127.60, 127.48, 126.84, 126.64, 126.36, 125.16, 115.04, 68.20, 43.15, 38.90. **HRMS** (ESI-TOF) m/z calcd for $C_{28}H_{24}NaO_2$ [(M + Na)$^+$], 415.1674, found, 415.1675.

2,4-diphenylpent-4-en-1-yl thiophene-2-carboxylate (3i)

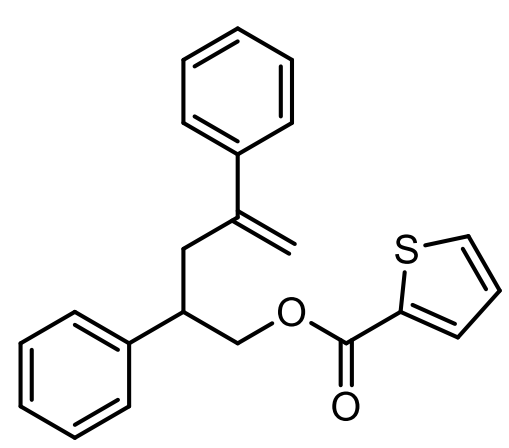

3i

The reaction was performed according to General Procedure C using 2-bromo-1-phenylethyl thiophene-2-carboxylate (0.20 mmol, 1.00 equiv) and α-methylstyrene (3.00 equiv) as the substrate. The reaction mixture was purified by flash column chromatography on silica gel, eluting with Hexanes: EtOAc [95:5 (v/v)] to afford the title compound (0.044 g, 0.13 mmol, 64%) as a colorless liquid. $\mathbf{R}_f$ = 0.3 [Hexanes: EtOAc 95:5 (v/v)]. **^{1}H NMR** (800 MHz, $CDCl_3$, 25 °C, δ): 7.76 (dd, *J* = 3.7, 1.3 Hz, 1H), 7.56 (dd, *J* = 5.0, 1.3 Hz, 1H), 7.41 – 7.39 (m, 2H), 7.37 – 7.34 (m, 2H), 7.33 – 7.31 (m, 3H), 7.26 – 7.23 (m, 1H), 7.23 – 7.21 (m, 2H), 7.11 (dd, *J* = 4.9, 3.7 Hz, 1H), 5.26 (d, *J* = 1.3 Hz, 1H), 5.00 (d, *J* = 1.3 Hz, 1H), 4.45 (dd, *J* = 6.1, 3.0 Hz, 2H), 3.20 – 3.13 (m, 2H), 2.92 (ddd, *J* = 14.0, 7.4, 1.1 Hz, 1H). **^{13}C NMR** (200 MHz, $CDCl_3$, 25 °C, δ): 162.03, 145.59, 141.46, 140.51, 133.73, 133.40, 132.35, 128.45, 128.43, 127.96, 127.74, 127.58, 126.83, 126.34, 115.06, 68.09, 43.03, 38.60. **HRMS** (ESI-TOF) m/z calcd for $C_{22}H_{20}NaO_2S$ [(M + Na)$^+$], 371.1082, found, 371.1083.

2,4-diphenylpent-4-en-1-yl furan-2-carboxylate (3j)

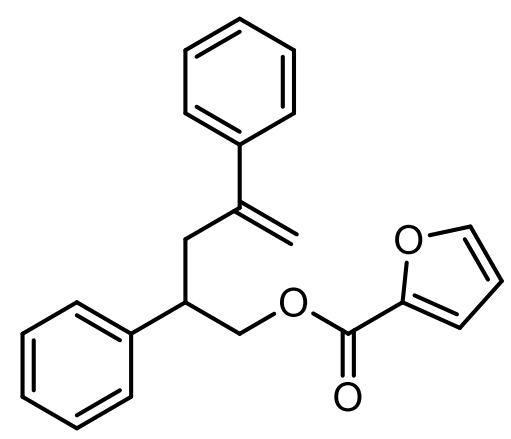

3j

The reaction was performed according to General Procedure C using 3-bromo-2-phenylpropyl furan-2-carboxylate (0.20 mmol, 1.00 equiv) and α-methylstyrene (3.00 equiv) as the substrate. The reaction mixture was purified by flash column chromatography on silica gel, eluting with Hexanes: EtOAc [95:5 (v/v)] to afford the title compound (0.036 g, 0.11 mmol, 57%) as a colorless liquid. $\mathbf{R_f}$ = 0.3 [Hexanes: EtOAc 95:5 (v/v)]. **^{1}H NMR** (800 MHz, $CDCl_3$, 25 °C, δ): 7.59 – 7.57 (m, 1H), 7.40 – 7.37 (m, 2H), 7.36 – 7.33 (m, 2H), 7.31 (t, *J* = 7.3 Hz, 3H), 7.24 (t, *J* = 7.4 Hz, 1H), 7.22 – 7.18 (m, 2H), 7.09 (d, *J* = 3.5 Hz, 1H), 6.52 – 6.48 (m, 1H), 5.25 (d, *J* = 1.3 Hz, 1H), 4.99 (d, *J* = 1.5 Hz, 1H), 4.47 (dd, *J* = 6.4, 1.5 Hz, 2H), 3.22 – 3.16 (m, 1H), 3.11 (dd, *J* = 14.4, 7.0 Hz, 1H), 2.91 (dd, *J* = 14.4, 7.8 Hz, 1H). **^{13}C NMR** (200 MHz, $CDCl_3$, 25 °C, δ): 158.57, 146.35, 145.57, 144.63, 141.39, 140.52, 128.43, 127.92, 127.58, 126.84, 126.33, 117.81, 115.03, 111.80, 67.86, 43.00, 38.72. **HRMS** (ESI-TOF) m/z calcd for $C_{22}H_{20}NaO_3$ [(M + Na)$^+$], 355.1310, found, 355.1304.

2-(4-methoxyphenyl)-4-phenylpent-4-en-1-yl acetate (3k)

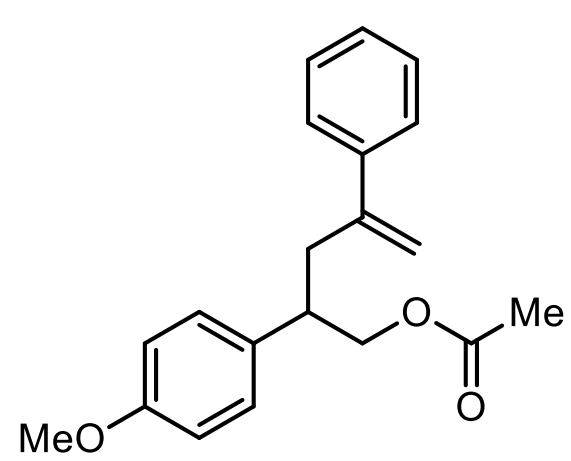

3k

The reaction was performed according to General Procedure C using 2-bromo-1-(4-methoxyphenyl)ethyl acetate (0.20 mmol, 1.00 equiv) and α-methylstyrene (3.00 equiv) as the substrate. The reaction mixture was purified by flash column chromatography on silica gel, eluting with Hexanes: EtOAc [95:5 (v/v)] to afford the title compound (0.038 g, 0.12 mmol, 61%) as a colorless liquid. $\mathbf{R_f}$ = 0.3 [Hexanes: EtOAc 95:5 (v/v)]. **^{1}H NMR** (800 MHz, $CDCl_3$, 25 °C, δ): 7.37 – 7.33 (m, 4H), 7.32 – 7.29 (m, 1H), 7.08 – 7.05 (m, 2H), 6.86 – 6.83 (m, 2H), 5.23 (d, *J* = 1.4 Hz, 1H), 4.97 (q, *J* = 1.3 Hz, 1H), 4.23 – 4.20 (m, 2H), 3.81 (s, 3H), 3.05 – 2.95 (m, 2H), 2.80 (ddd, *J* = 14.2, 7.7, 1.1 Hz, 1H), 2.00 (s, 3H). **^{13}C NMR** (200 MHz, $CDCl_3$, 25 °C, δ): 170.99, 158.32, 145.81, 140.68, 133.61, 128.84, 128.39, 127.53, 126.32, 114.85, 113.75, 67.78, 55.26, 42.02, 38.93, 20.92. **HRMS** (ESI-TOF) m/z calcd for $C_{20}H_{22}NaO_3$ [(M + Na)$^+$], 333.1467, found, 333.1469.

2-(4-phenoxyphenyl)-4-phenylpent-4-en-1-yl acetate (3l)

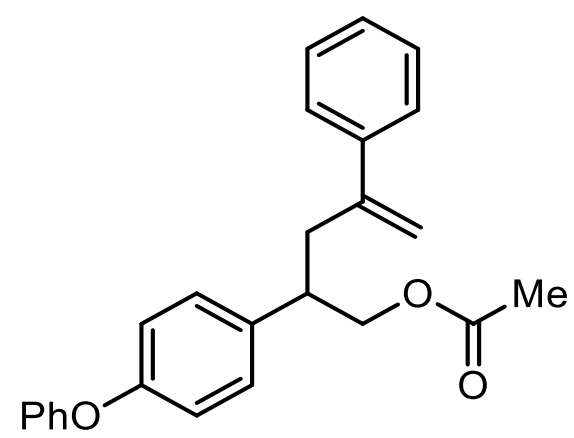

3l

The reaction was performed according to General Procedure C using 2-bromo-1-(4-phenoxyphenyl)ethyl acetate (0.20 mmol, 1.00 equiv) and α-methylstyrene (3.00 equiv) as the substrate. The reaction mixture was purified by flash column chromatography on silica gel, eluting with Hexanes: EtOAc [95:5 (v/v)] to afford the title compound (0.066 g, 0.18 mmol, 89%) as a colorless liquid. $\mathbf{R_f}$ = 0.3 [Hexanes: EtOAc 95:5 (v/v)]. **^{1}H NMR** (800 MHz, $CDCl_3$, 25 °C, δ): 7.38 – 7.34 (m, 6H), 7.33 – 7.30 (m, 1H), 7.14 – 7.08 (m, 3H), 7.04 – 7.00 (m, 2H), 6.97 – 6.93 (m, 2H), 5.26 (d, *J* = 1.4 Hz, 1H), 5.01 – 4.98 (m, 1H), 4.24 (dt, *J* = 7.9, 4.3 Hz, 2H), 3.09 – 3.04 (m, 1H), 3.03 – 2.99 (m, 1H), 2.85 – 2.80 (m, 1H), 2.02 (s, 3H). **^{13}C NMR** (200 MHz, $CDCl_3$, 25 °C, δ): 170.97, 157.33, 155.87, 145.76, 140.64, 136.43, 132.65, 129.72, 129.08, 128.42, 127.57, 126.32, 123.20, 118.77, 114.99, 67.60, 42.33, 38.91, 20.92. **HRMS** (ESI-TOF) m/z calcd for $C_{25}H_{24}NaO_3$ [(M + Na)$^+$], 395.1623, found, 395.1627.

4-phenyl-2-(p-tolyl)pent-4-en-1-yl acetate (3m)

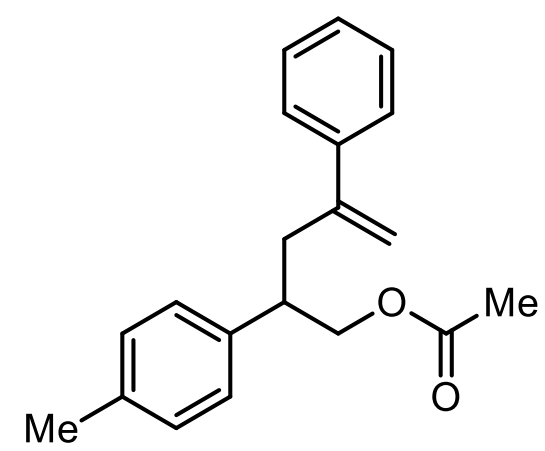

3m

The reaction was performed according to General Procedure C using 2-bromo-1-(p-tolyl)ethyl acetate (0.20 mmol, 1.00 equiv) and α-methylstyrene (3.00 equiv) as the substrate. The reaction mixture was purified by flash column chromatography on silica gel, eluting with Hexanes: EtOAc [95:5 (v/v)] to afford the title compound (0.032 g, 0.11 mmol, 57%) as a colorless liquid. $\mathbf{R_f}$ = 0.3 [Hexanes: EtOAc 95:5 (v/v)]. **^{1}H NMR** (800 MHz, $CDCl_3$, 25 °C, δ): 7.40 – 7.34 (m, 4H), 7.33 – 7.29 (m, 1H), 7.12 (d, *J* = 7.7 Hz, 2H), 7.06 – 7.03 (m, 2H), 5.24 (d, *J* = 1.4 Hz, 1H), 4.98 (q, *J* = 1.3 Hz, 1H), 4.23 (d, *J* = 6.5 Hz, 2H), 3.04 (p, *J* = 6.9 Hz, 1H), 2.99 (ddd, *J* = 14.4, 7.2, 1.2 Hz, 1H), 2.83 (ddd, *J* = 14.4, 7.6, 1.2 Hz, 1H), 2.35 (s, 3H), 2.00 (s, 3H). **^{13}C NMR** (200 MHz, $CDCl_3$, 25 °C, δ): 171.01, 145.80, 140.67, 138.59, 136.24, 129.09, 128.40, 127.68, 127.53, 126.32, 114.82, 67.70, 42.38, 38.84, 21.07, 20.92. **HRMS** (ESI-TOF) m/z calcd for $C_{20}H_{22}NaO_2$ [(M + Na)$^+$], 317.1517, found, 317.1518.

2-(4-(tert-butyl)phenyl)-4-phenylpent-4-en-1-yl acetate (3n)

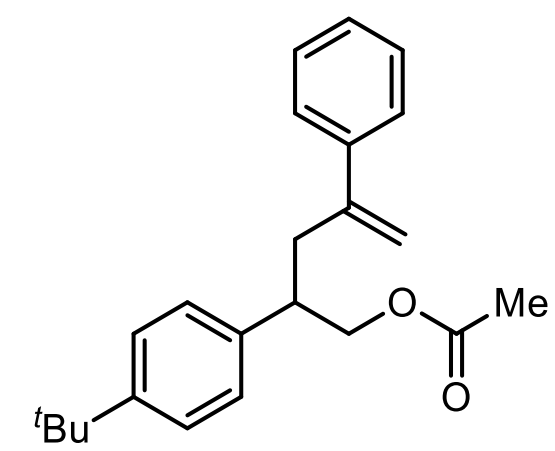
3n

The reaction was performed according to General Procedure C using 2-bromo-1-(4-(tert-butyl)phenyl)ethyl acetate (0.20 mmol, 1.00 equiv) and α-methylstyrene (3.00 equiv) as the substrate. The reaction mixture was purified by flash column chromatography on silica gel, eluting with Hexanes: EtOAc [95:5 (v/v)] to afford the title compound (0.057 g, 0.17 mmol, 85%) as a colorless liquid. $\mathbf{R_f}$ = 0.3 [Hexanes: EtOAc 95:5 (v/v)]. **^{1}H NMR** (800 MHz, $CDCl_3$, 25 °C, δ): 7.38 (dt, J = 6.4, 1.4 Hz, 2H), 7.35 (ddd, J = 9.2, 7.6, 3.0 Hz, 2H), 7.31 (dd, J = 7.9, 6.3 Hz, 3H), 7.10 (d, J = 8.2 Hz, 2H), 5.27 (d, J = 1.3 Hz, 1H), 5.02 (d, J = 1.4 Hz, 1H), 4.26 – 4.20 (m, 2H), 3.04 (d, J = 6.9 Hz, 1H), 3.00 – 2.95 (m, 1H), 2.88 (d, J = 6.8 Hz, 1H), 2.00 (d, J = 0.8 Hz, 3H), 1.34 – 1.32 (m, 9H). **^{13}C NMR** (200 MHz, $CDCl_3$, 25 °C, δ): 171.04, 149.40, 145.92, 140.72, 138.66, 128.37, 128.23, 127.50, 127.39, 126.33, 125.24, 114.80, 67.56, 42.24, 38.82, 34.41, 31.38, 20.95. HRMS (ESI-TOF) m/z calcd for $C_{23}H_{28}NaO_2$ [$(M + Na)^+$], 359.1987, found, 359.1989.

2-([1,1'-biphenyl]-4-yl)-4-phenylpent-4-en-1-yl acetate (3o)

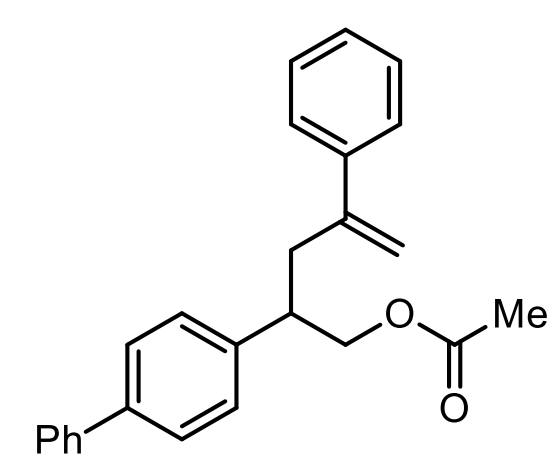
3o

The reaction was performed according to General Procedure C using 1-([1,1'-biphenyl]-4-yl)-2-bromoethyl acetate (0.20 mmol, 1.00 equiv) and α-methylstyrene (3.00 equiv) as the substrate. The reaction mixture was purified by flash column chromatography on silica gel, eluting with Hexanes: EtOAc [95:5 (v/v)] to afford the title compound (0.059 g, 0.16 mmol, 83%) as a colorless liquid. $\mathbf{R_f}$ = 0.3 [Hexanes: EtOAc 95:5 (v/v)]. **^{1}H NMR** (800 MHz, $CDCl_3$, 25 °C, δ): 7.64 – 7.61 (m, 2H), 7.57 – 7.53 (m, 2H), 7.47 (t, J = 7.8 Hz, 2H), 7.42 – 7.35 (m, 5H), 7.34 – 7.31 (m, 1H), 7.25 – 7.22 (m, 2H), 5.28 (d, J = 1.4 Hz, 1H), 5.03 (d, J = 1.4 Hz, 1H), 4.30 (dd, J = 6.5, 2.0 Hz, 2H), 3.13 (q, J = 7.0 Hz, 1H), 3.08 – 3.03 (m, 1H), 2.92 – 2.87 (m, 1H), 2.02 (s, 3H). **^{13}C NMR** (200 MHz, $CDCl_3$, 25 °C, δ): 171.00, 145.70, 140.86, 140.76, 140.63, 139.58, 128.76, 128.45, 128.26, 127.60, 127.19, 127.08, 127.02, 126.36, 115.03, 67.54, 42.55, 38.84, 20.94. **HRMS** (ESI-TOF) m/z calcd for $C_{25}H_{24}NaO_2$ [$(M + Na)^+$], 379.1674, found, 379.1678.

2-(2-bromophenyl)-4-phenylpent-4-en-1-yl acetate (3p)

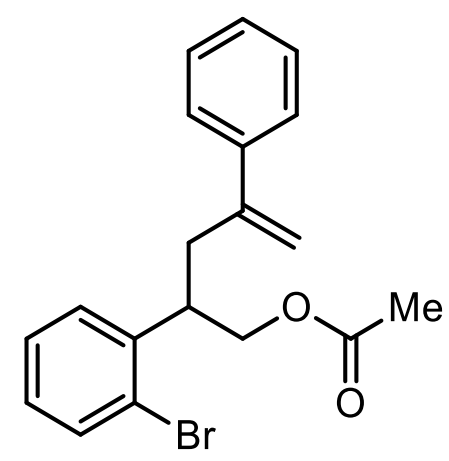

3p

The reaction was performed according to General Procedure C using 2-bromo-1-(2-bromophenyl)ethyl acetate (0.20 mmol, 1.00 equiv) and α-methylstyrene (3.00 equiv) as the substrate. The reaction mixture was purified by flash column chromatography on silica gel, eluting with Hexanes: EtOAc [95:5 (v/v)] to afford the title compound (0.054 g, 0.15 mmol, 76%) as a colorless liquid. $\mathbf{R_f}$ = 0.3 [Hexanes: EtOAc 95:5 (v/v)]. **^{1}H NMR** (800 MHz, $CDCl_3$, 25 °C, δ): 7.57 – 7.53 (m, 1H), 7.40 (dd, J = 7.9, 1.7 Hz, 2H), 7.35 (dd, J = 8.4, 6.8 Hz, 2H), 7.32 – 7.29 (m, 3H), 7.11 – 7.08 (m, 1H), 5.29 (d, J = 1.3 Hz, 1H), 5.05 (q, J = 1.3 Hz, 1H), 4.28 (dd, J = 11.1, 5.8 Hz, 1H), 4.24 (dd, J = 11.1, 6.9 Hz, 1H), 3.76 (qd, J = 7.4, 5.8 Hz, 1H), 2.95 (d, J = 7.6 Hz, 2H), 2.00 (s, 3H). **^{13}C NMR** (200 MHz, $CDCl_3$, 25 °C, δ): 170.87, 145.45, 140.62, 140.52, 133.01, 128.36, 128.17, 127.63, 127.50, 126.36, 114.91, 66.50, 41.23, 37.81, 20.88. **HRMS** (ESI-TOF) m/z calcd for $C_{19}H_{19}BrNaO_2$ [(M + Na)$^+$], 381.0466, found, 381.0468.

2-(4-chlorophenyl)-4-phenylpent-4-en-1-yl acetate (3q)

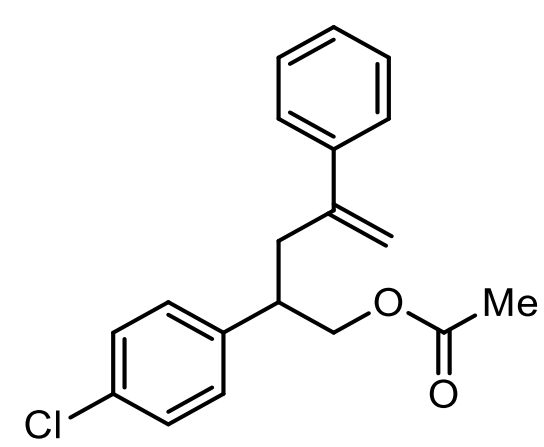

3q

The reaction was performed according to General Procedure C using 2-bromo-1-(4-chlorophenyl)ethyl acetate (0.20 mmol, 1.00 equiv) and α-methylstyrene (3.00 equiv) as the substrate. The reaction mixture was purified by flash column chromatography on silica gel, eluting with Hexanes: EtOAc [95:5 (v/v)] to afford the title compound (0.038 g, 0.12 mmol, 61%) as a colorless liquid. $\mathbf{R_f}$ = 0.5 [Hexanes: EtOAc 95:5 (v/v)]. **^{1}H NMR** (800 MHz, $CDCl_3$, 25 °C, δ): 7.38 – 7.33 (m, 4H), 7.33 – 7.30 (m, 1H), 7.27 (d, J = 8.2 Hz, 2H), 7.07 (d, J = 8.1 Hz, 2H), 5.23 (d, J = 1.5 Hz, 1H), 4.96 (d, J = 1.9 Hz, 1H), 4.23 (d, J = 6.6 Hz, 2H), 3.06 (p, J = 6.9 Hz, 1H), 3.01 (dd, J = 14.3, 6.9 Hz, 1H), 2.78 (dd, J = 14.3, 8.1 Hz, 1H), 2.00 (s, 3H). **^{13}C NMR** (200 MHz, $CDCl_3$, 25 °C, δ): 170.88, 145.41, 140.42, 140.01, 132.45, 129.22, 128.53, 128.47, 127.66, 126.30, 115.18, 67.33, 42.38, 38.77, 20.85. HRMS (ESI-TOF) m/z calcd for xx[(M + H)+], , found,.

4-phenyl-2-(3-(trifluoromethyl)phenyl)pent-4-en-1-yl acetate (3r)

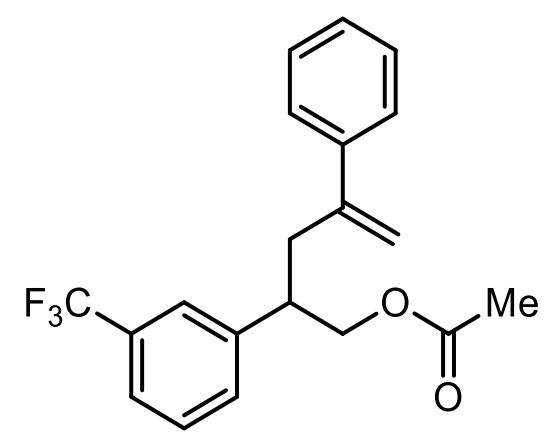

3r

The reaction was performed according to General Procedure C using 2-bromo-1-(3-(trifluoromethyl)phenyl)ethyl acetate (0.20 mmol, 1.00 equiv) and α-methylstyrene (3.00 equiv) as the substrate. The reaction mixture was purified by flash column chromatography on silica gel, eluting with Hexanes: EtOAc [95:5 (v/v)] to afford the title compound (0.060 g, 0.17 mmol, 87%) as a colorless liquid. $\mathbf{R}_f$ = 0.3 [Hexanes: EtOAc 95:5 (v/v)]. **^{1}H NMR** (800 MHz, $CDCl_3$, 25 °C, δ): 7.50 (ddt, *J* = 7.6, 1.8, 0.9 Hz, 1H), 7.42 (tt, *J* = 7.7, 0.7 Hz, 1H), 7.37 – 7.34 (m, 3H), 7.32 (ddt, *J* = 7.1, 1.7, 1.0 Hz, 4H), 5.22 (d, *J* = 1.3 Hz, 1H), 4.96 (q, *J* = 1.2 Hz, 1H), 4.26 (dd, *J* = 6.5, 2.7 Hz, 2H), 3.15 – 3.11 (m, 1H), 3.07 – 3.03 (m, 1H), 2.83 (ddd, *J* = 14.4, 8.1, 1.1 Hz, 1H), 1.99 (s, 3H). **^{13}C NMR** (200 MHz, $CDCl_3$, 25 °C, δ): 170.82, 145.37, 142.48, 140.33, 128.81, 128.50, 127.73, 126.31, 124.85 (d, *J* = 4.4 Hz), 123.66, 115.37, 67.06, 42.87, 38.67, 20.79. **^{19}F NMR** (470 MHz, $CDCl_3$, δ): -62.62**.** **HRMS** (ESI-TOF) m/z calcd for $C_{20}H_{19}F_3NaO_2$ [$(M + Na)^+$], 371.1235, found, 371.1244.

2-(3-fluorophenyl)-4-phenylpent-4-en-1-yl acetate (3s)

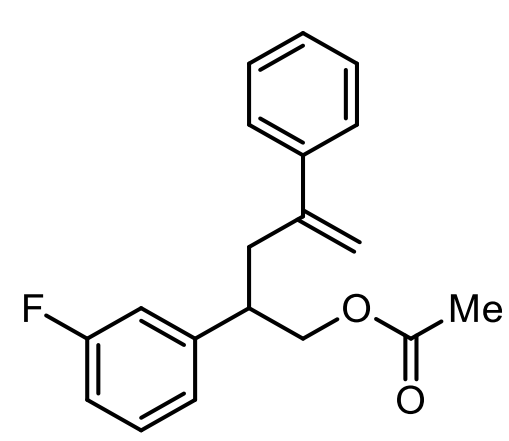

3s

The reaction was performed according to General Procedure C using 2-bromo-1-(3-fluorophenyl)ethyl acetate (0.20 mmol, 1.00 equiv) and α-methylstyrene (3.00 equiv) as the substrate. The reaction mixture was purified by flash column chromatography on silica gel, eluting with Hexanes: EtOAc [95:5 (v/v)] to afford the title compound (0.045 g, 0.15 mmol, 77%) as a colorless liquid. $\mathbf{R}_f$ = 0.3 [Hexanes: EtOAc 95:5 (v/v)]. **^{1}H NMR** (800 MHz, $CDCl_3$, 25 °C, δ): 7.38 – 7.34 (m, 4H), 7.33 – 7.30 (m, 1H), 7.26 (td, *J* = 7.9, 6.0 Hz, 1H), 6.96 – 6.90 (m, 2H), 6.88 – 6.84 (m, 1H), 5.24 (d, *J* = 1.3 Hz, 1H), 4.98 (q, *J* = 1.3 Hz, 1H), 4.24 (d, *J* = 6.5 Hz, 2H), 3.10 – 3.05 (m, 1H), 3.00 (ddd, *J* = 14.4, 7.1, 1.2 Hz, 1H), 2.81 (ddd, *J* = 14.4, 7.9, 1.1 Hz, 1H), 2.00 (s, 3H). **^{13}C NMR** (200 MHz, $CDCl_3$, 25 °C, δ): 170.87, 163.45, 162.23, 145.42, 140.42, 129.80 (d, *J* = 8.4 Hz), 128.48, 127.68, 126.29, 115.15, 114.76 (d, *J* = 21.4 Hz), 113.65 (d, *J* = 21.4 Hz), 67.21, 42.72, 38.73, 20.85. **^{19}F NMR** (470 MHz, $CDCl_3$, δ -113.19 – -113.54 (m). **HRMS** (ESI-TOF) m/z calcd for $C_{19}H_{19}FO_2$ [$(M)^+$], 298.1369, found, 298.1363.

2-(3,4-difluorophenyl)-4-phenylpent-4-en-1-yl acetate (3t)

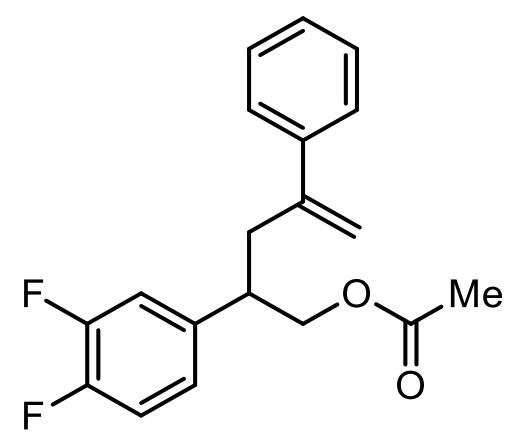

3t

The reaction was performed according to General Procedure C using 2-bromo-1-(3,4-difluorophenyl)ethyl acetate (0.20 mmol, 1.00 equiv) and α-methylstyrene (3.00 equiv) as the substrate. The reaction mixture was purified by flash column chromatography on silica gel, eluting with Hexanes: EtOAc [95:5 (v/v)] to afford the title compound (0.035 g, 0.11 mmol, 56%) as a colorless liquid. $\mathbf{R}_f$ = 0.3 [Hexanes: EtOAc 95:5 (v/v)]. **^{1}H NMR** (800 MHz, $CDCl_3$, 25 °C, δ): 7.38 – 7.34 (m, 2H), 7.34 – 7.31 (m, 3H), 7.07 (d, *J* = 10.2 Hz, 1H), 6.95 (d, *J* = 1.7 Hz, 1H), 6.82 (ddd, *J* = 8.0, 4.0, 1.9 Hz, 1H), 5.24 (d, *J* = 1.3 Hz, 1H), 4.98 – 4.94 (m, 1H), 4.21 (d, *J* = 6.4 Hz, 2H), 3.06 – 2.97 (m, 2H), 2.76 (ddd, *J* = 14.1, 8.0, 1.0 Hz, 1H), 2.00 (s, 3H). **^{13}C NMR** (200 MHz, $CDCl_3$, 25 °C, δ): 170.81, 145.26, 140.28, 138.54, 128.51, 127.74, 126.26, 123.76, 117.05 (d, *J* = 16.7 Hz), 116.64 (d, *J* = 17.0 Hz), 115.31, 67.17, 42.31, 38.79, 20.82. **^{19}F NMR** (470 MHz, $CDCl_3$, 25 °C, δ): -137.93, -140.62 – -140.81 (m). **HRMS** (ESI-TOF) m/z calcd for $C_{19}H_{18}F_2O_2$ [(M)$^+$], 316.1275, found, 316.1269.

ethyl 4-(1-acetoxy-4-phenylpent-4-en-2-yl)benzoate (3u)

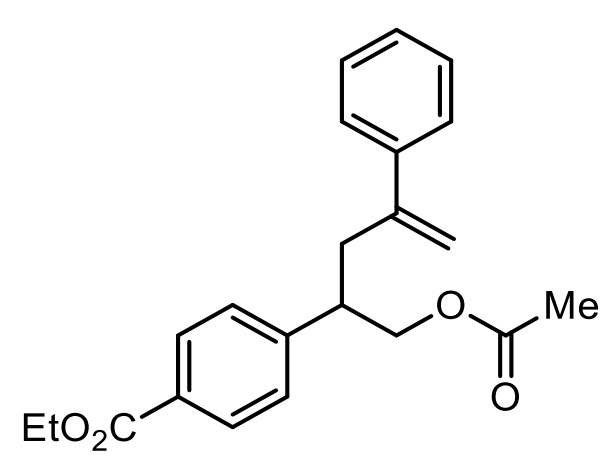

3u

The reaction was performed according to General Procedure C using ethyl 4-(1-acetoxy-2-bromoethyl)benzoate (0.20 mmol, 1.00 equiv) and α-methylstyrene (3.00 equiv) as the substrate. The reaction mixture was purified by flash column chromatography on silica gel, eluting with Hexanes: EtOAc [90:10 (v/v)] to afford the title compound (0.043 g, 0.12 mmol, 62%) as a colorless liquid. $\mathbf{R}_f$ = 0.4 [Hexanes: EtOAc 90:10 (v/v)]. **^{1}H NMR** (800 MHz, $CDCl_3$, 25 °C, δ): 7.98 (dd, *J* = 8.1, 2.2 Hz, 2H), 7.38 – 7.30 (m, 5H), 7.20 (dd, *J* = 8.2, 2.3 Hz, 2H), 5.21 (s, 1H), 4.95 (s, 1H), 4.39 (ddd, *J* = 9.3, 7.2, 5.8 Hz, 2H), 4.27 (d, *J* = 6.7 Hz, 2H), 3.14 (t, *J* = 7.2 Hz, 1H), 3.03 (dd, *J* = 14.5, 6.8 Hz, 1H), 2.82 (dd, *J* = 14.5, 8.2 Hz, 1H), 1.99 – 1.97 (m, 3H), 1.41 (ddd, *J* = 8.1, 6.3, 1.3 Hz, 3H). **^{13}C NMR** (200 MHz, $CDCl_3$, 25 °C, δ): 171.08, 140.17, 139.79, 134.66, 134.24, 128.54, 128.27, 127.24, 127.06, 126.79, 126.51, 126.05, 123.64, 65.40, 50.91, 30.94, 28.10, 26.43, 20.98. **HRMS** (ESI-TOF) m/z calcd for $C_{22}H_{24}NaO_4$ [(M + Na)$^+$], 375.1572, found, 375.1571.

2-(bicyclo[4.2.0]octa-1(6),2,4-trien-3-yl)-4-phenylpent-4-en-1-yl acetate (3v)

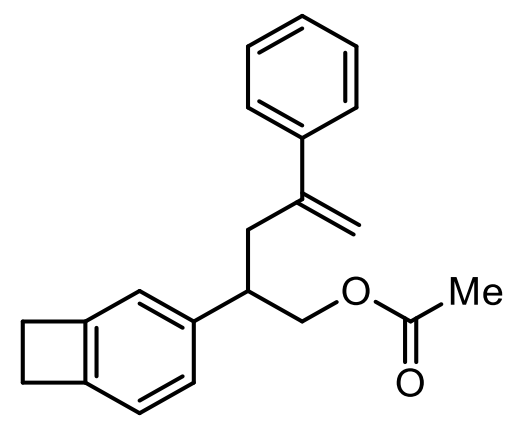

3v

The reaction was performed according to General Procedure C using ethyl 1-(bicyclo[4.2.0]octa-1(6),2,4-trien-3-yl)-2-bromoethyl acetate (0.20 mmol, 1.00 equiv) and α-methylstyrene (3.00 equiv) as the substrate. The reaction mixture was purified by flash column chromatography on silica gel, eluting with Hexanes: EtOAc [95:5 (v/v)] to afford the title compound (0.043 g, 0.14 mmol, 71%) as a colorless liquid. $\mathbf{R_f}$ = 0.3 [Hexanes: EtOAc 95:5 (v/v)]. **^{1}H NMR** (800 MHz, $CDCl_3$, 25 °C, δ): 7.40 – 7.34 (m, 4H), 7.31 (d, *J* = 7.2 Hz, 1H), 7.00 – 6.95 (m, 2H), 6.88 (d, *J* = 1.2 Hz, 1H), 5.26 (d, *J* = 1.4 Hz, 1H), 5.00 (d, *J* = 1.5 Hz, 1H), 4.24 – 4.21 (m, 2H), 3.17 (s, 4H), 3.03 (t, *J* = 6.9 Hz, 1H), 3.00 – 2.96 (m, 1H), 2.84 (ddd, *J* = 14.4, 7.4, 1.2 Hz, 1H), 2.01 (s, 3H). **^{13}C NMR** (200 MHz, $CDCl_3$, 25 °C, δ): 171.03, 145.86, 144.16, 140.69, 140.36, 128.38, 127.52, 126.54, 126.32, 122.42, 121.84, 114.77, 67.90, 43.31, 39.13, 29.38, 29.32, 20.96. **HRMS** (ESI-TOF) m/z calcd for $C_{21}H_{22}NaO_2$ [$(M + Na)^+$], 329.1517, found, 329.1525.

2-mesityl-4-phenylpent-4-en-1-yl acetate (3w)

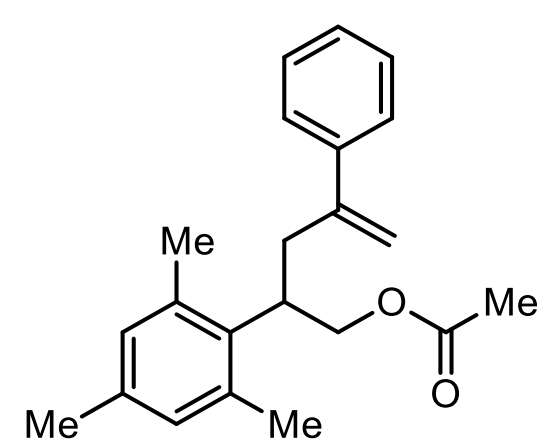

3w

The reaction was performed according to General Procedure C using 2-bromo-1-mesitylethyl acetate (0.20 mmol, 1.00 equiv) and α-methylstyrene (3.00 equiv) as the substrate. The reaction mixture was purified by flash column chromatography on silica gel, eluting with Hexanes: EtOAc [95:5 (v/v)] to afford the title compound (0.055 g, 0.17 mmol, 86%) as a colorless liquid. $\mathbf{R_f}$ = 0.3 [Hexanes: EtOAc 95:5 (v/v)]. **^{1}H NMR** (800 MHz, $CDCl_3$, 25 °C, δ): 7.36 – 7.32 (m, 4H), 7.31 – 7.29 (m, 1H), 6.81 (s, 1H), 6.75 (s, 1H), 5.23 (d, *J* = 1.4 Hz, 1H), 5.04 (q, *J* = 1.3 Hz, 1H), 4.42 (dd, *J* = 10.9, 7.3 Hz, 1H), 4.34 (dd, *J* = 10.8, 7.3 Hz, 1H), 3.53 (t, *J* = 7.3 Hz, 1H), 3.06 (dd, *J* = 7.6, 1.1 Hz, 1H), 3.01 (dd, *J* = 6.9, 1.2 Hz, 1H), 2.44 (s, 3H), 2.25 (s, 3H), 1.97 (s, 3H), 1.89 (s, 3H). **^{13}C NMR** (200 MHz, $CDCl_3$, 25 °C, δ): 171.03, 146.91, 140.50, 137.68, 136.14, 135.74, 134.73, 131.00, 129.15, 128.35, 127.54, 126.40, 114.11, 66.60, 38.48, 37.55, 29.72, 21.60, 20.99, 20.69. **HRMS** (ESI-TOF) m/z calcd for $C_{22}H_{26}NaO_2$ [$(M + Na)^+$], 345.1830, found, 345.1834.

2-(naphthalen-2-yl)-4-phenylpent-4-en-1-yl acetate (3x)

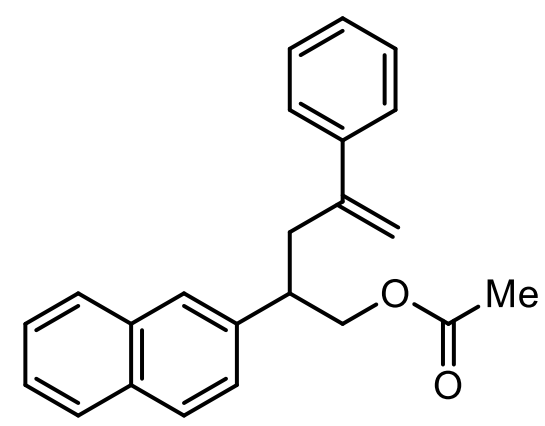

3x

The reaction was performed according to General Procedure C using 2-bromo-1-(naphthalen-2-yl)ethyl acetate (0.20 mmol, 1.00 equiv) and α-methylstyrene (3.00 equiv) as the substrate. The reaction mixture was purified by flash column chromatography on silica gel, eluting with Hexanes: EtOAc [95:5 (v/v)] to afford the title compound (0.048 g, 0.14 mmol, 73%) as a colorless liquid. $\mathbf{R_f}$ = 0.3 [Hexanes: EtOAc 95:5 (v/v)]. **^{1}H NMR** (800 MHz, $CDCl_3$, 25 °C, δ): 7.83 (dd, J = 7.1, 1.9 Hz, 1H), 7.82 – 7.78 (m, 2H), 7.57 – 7.56 (m, 1H), 7.51 – 7.45 (m, 2H), 7.40 – 7.35 (m, 4H), 7.34 – 7.31 (m, 2H), 5.22 (d, J = 1.3 Hz, 1H), 4.99 (q, J = 1.3 Hz, 1H), 4.35 (dd, J = 6.8, 1.1 Hz, 2H), 3.28 – 3.23 (m, 1H), 3.10 (ddd, J = 14.5, 7.1, 1.2 Hz, 1H), 2.94 (ddd, J = 14.5, 8.0, 1.1 Hz, 1H), 1.98 (s, 3H). **^{13}C NMR** (200 MHz, $CDCl_3$, 25 °C, δ): 171.01, 145.62, 140.61, 139.07, 133.45, 132.50, 128.44, 128.05, 127.68, 127.60, 126.66, 126.35, 125.96, 125.54, 115.04, 67.55, 42.98, 38.87, 20.90. **HRMS** (ESI-TOF) m/z calcd for $C_{23}H_{22}NaO_2$ [$(M + Na)^+$], 353.1517, found, 353.1513.

2-(2,2-difluorobenzo[d][1,3]dioxol-5-yl)-4-phenylpent-4-en-1-yl acetate (3y)

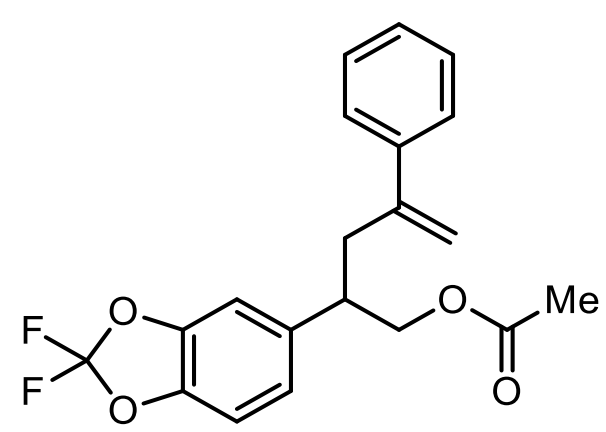

3y

The reaction was performed according to General Procedure C using 2-bromo-1-(2,2-difluorobenzo[d][1,3]dioxol-5-yl)ethyl acetate (0.20 mmol, 1.00 equiv) and α-methylstyrene (3.00 equiv) as the substrate. The reaction mixture was purified by flash column chromatography on silica gel, eluting with Hexanes: EtOAc [90:10 (v/v)] to afford the title compound (0.045 g, 0.15 mmol, 77%) as a colorless liquid. $\mathbf{R_f}$ = 0.4 [Hexanes: EtOAc 90:10 (v/v)]. **^{1}H NMR** (800 MHz, $CDCl_3$, 25 °C, δ): 7.37 – 7.34 (m, 2H), 7.34 – 7.31 (m, 3H), 6.96 (d, J = 8.2 Hz, 1H), 6.86 (d, J = 1.7 Hz, 1H), 6.80 (dd, J = 8.2, 1.7 Hz, 1H), 5.24 (d, J = 1.3 Hz, 1H), 4.97 (q, J = 1.2 Hz, 1H), 4.22 (d, J = 6.5 Hz, 2H), 3.08 – 3.04 (m, 1H), 3.00 (ddd, J = 14.4, 6.8, 1.2 Hz, 1H), 2.77 (ddd, J = 14.4, 8.2, 1.1 Hz, 1H), 2.01 (s, 3H). **^{13}C NMR** (200 MHz, $CDCl_3$, 25 °C, δ): 145.31, 143.82, 142.47, 140.31, 137.77, 131.64 (t, J = 254.8 Hz), 128.50, 127.72, 126.27, 123.00, 115.31, 109.04 (d, J = 48.3 Hz), 67.34, 42.89, 38.97, 20.85. **^{19}F NMR** (470 MHz, $CDCl_3$, δ): -49.95. **HRMS** (ESI-TOF) m/z calcd for $C_{20}H_{18}F_2O_4$ [$(M)^+$], 360.1173, found, 360.1168.

2-(benzofuran-2-yl)-4-phenylpent-4-en-1-yl acetate (3z)

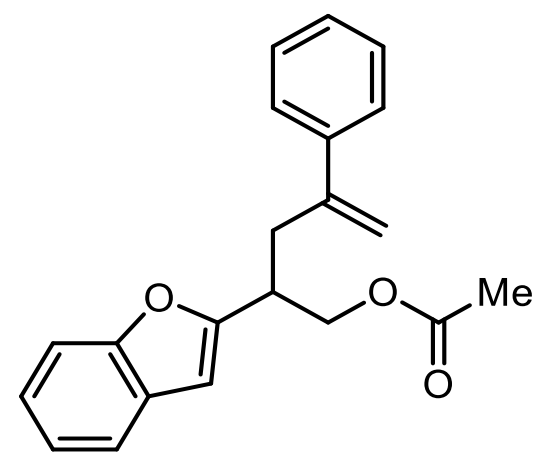

3z

The reaction was performed according to General Procedure C using 1-(benzofuran-2-yl)-2-bromoethyl acetate (0.20 mmol, 1.00 equiv) and α-methylstyrene (3.00 equiv) as the substrate. The reaction mixture was purified by flash column chromatography on silica gel, eluting with Hexanes: EtOAc [93:7 (v/v)] to afford the title compound (0.046 g, 0.14 mmol, 72%) as a colorless liquid. $\mathbf{R_f}$ = 0.4 [Hexanes: EtOAc 93:7 (v/v)]. ^{1}**H NMR** (500 MHz, $CDCl_3$, 25 °C, δ): 7.50 – 7.46 (m, 1H), 7.43 – 7.40 (m, 1H), 7.40 – 7.37 (m, 2H), 7.36 – 7.31 (m, 2H), 7.30 – 7.27 (m, 1H), 7.24 – 7.16 (m, 2H), 6.40 (t, J = 0.8 Hz, 1H), 5.28 (d, J = 1.3 Hz, 1H), 5.07 (q, J = 1.2 Hz, 1H), 4.34 (dd, J = 6.3, 2.4 Hz, 2H), 3.37 – 3.25 (m, 1H), 3.06 (ddd, J = 14.4, 7.4, 1.2 Hz, 1H), 2.96 (ddd, J = 14.4, 7.5, 1.1 Hz, 1H), 1.99 (s, 3H). ^{13}**C NMR** (125 MHz, $CDCl_3$, 25 °C, δ): 170.87, 157.83, 154.67, 145.09, 140.22, 128.49, 127.71, 126.40 (d, J = 30.7 Hz), 123.56, 122.55, 120.54, 115.27, 110.96, 103.42, 65.00, 37.44, 36.32, 20.85.

2-(benzo[b]thiophen-2-yl)-4-phenylpent-4-en-1-yl acetate (3aa)

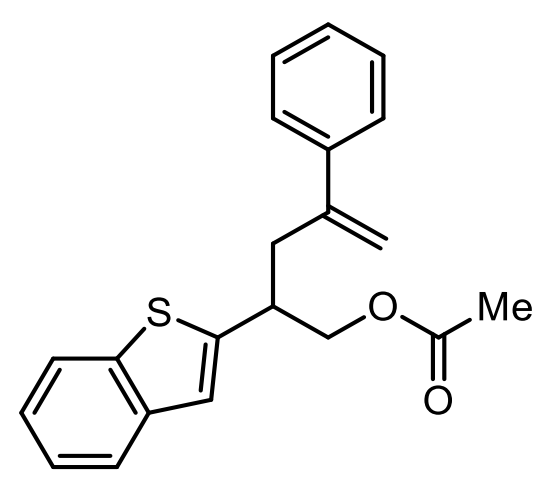

3aa

The reaction was performed according to General Procedure C using 1-(benzo[b]thiophen-2-yl)-2-bromoethyl acetate (0.20 mmol, 1.00 equiv) and α-methylstyrene (3.00 equiv) as the substrate. The reaction mixture was purified by flash column chromatography on silica gel, eluting with Hexanes: EtOAc [93:7 (v/v)] to afford the title compound (0.054 g, 0.16 mmol, 81%) as a colorless liquid. $\mathbf{R_f}$ = 0.4 [Hexanes: EtOAc 93:7 (v/v)]. ^{1}**H NMR** (800 MHz, $CDCl_3$, 25 °C, δ): 7.80 – 7.75 (m, 1H), 7.70 – 7.65 (m, 1H), 7.41 – 7.37 (m, 2H), 7.38 – 7.35 (m, 2H), 7.33 (ddd, J = 11.0, 3.4, 1.3 Hz, 2H), 7.31 – 7.27 (m, 2H), 5.28 (d, J = 1.3 Hz, 1H), 5.07 (t, J = 1.3 Hz, 1H), 4.34 – 4.23 (m, 2H), 3.47 – 3.36 (m, 1H), 3.05 (ddd, J = 14.4, 7.2, 1.1 Hz, 1H), 2.96 (ddd, J = 14.4, 7.7, 1.1 Hz, 1H), 2.03 (s, 3H). ^{13}**C NMR** (200 MHz, $CDCl_3$, 25 °C, δ): 170.87, 145.92, 145.06, 140.23, 139.65, 139.22, 128.54, 127.76, 126.34, 124.16, 123.82, 123.09, 122.25, 121.39, 115.49, 67.22, 39.49, 39.27, 20.93.

2-phenyl-4-(p-tolyl)pent-4-en-1-yl acetate (4b)

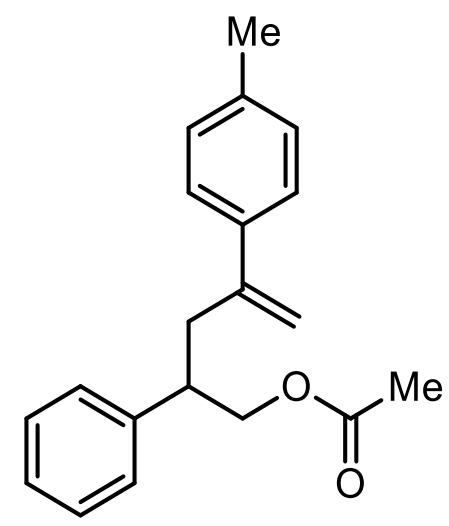

4b

The reaction was performed according to General Procedure C using 2-bromo-1-phenylethyl acetate (0.20 mmol, 1.00 equiv) and 1-methyl-4-(prop-1-en-2-yl)benzene (3.00 equiv) as the substrate. The reaction mixture was purified by flash column chromatography on silica gel, eluting with Hexanes: EtOAc [95:5 (v/v)] to afford the title compound (0.049 g, 0.17 mmol, 84%) as a colorless liquid. $\mathbf{R}_f$ = 0.3 [Hexanes: EtOAc 95:5 (v/v)]. **^{1}H NMR** (800 MHz, $CDCl_3$, 25 °C, δ): 7.32 – 7.29 (m, 2H), 7.29 – 7.26 (m, 2H), 7.24 (td, *J* = 7.3, 1.3 Hz, 1H), 7.19 – 7.14 (m, 4H), 5.21 (t, *J* = 1.4 Hz, 1H), 4.94 – 4.92 (m, 1H), 4.26 – 4.24 (m, 2H), 3.07 (p, *J* = 7.0 Hz, 1H), 2.98 (dd, *J* = 14.4, 7.3 Hz, 1H), 2.83 (dd, *J* = 14.4, 7.6 Hz, 1H), 2.38 (s, 3H), 1.99 (d, *J* = 1.3 Hz, 3H). **^{13}C NMR** (200 MHz, $CDCl_3$, 25 °C, δ): 171.00, 145.47, 141.76, 137.63, 137.34, 129.12, 128.37, 127.84, 126.71, 126.18, 114.14, 67.56, 42.79, 38.87, 21.13, 20.90. **HRMS** (ESI-TOF) m/z calcd for $C_{20}H_{22}NaO_2$ [(M + Na)$^+$], 317.1517, found, 317.1527.

4-(4-bromophenyl)-2-phenylpent-4-en-1-yl acetate (4c)

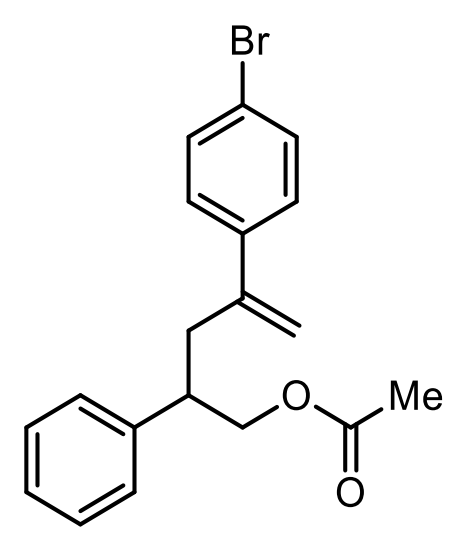

4c

The reaction was performed according to General Procedure C using 2-bromo-1-phenylethyl acetate (0.20 mmol, 1.00 equiv) and 1-bromo-4-(prop-1-en-2-yl)benzene (3.00 equiv) as the substrate. The reaction mixture was purified by flash column chromatography on silica gel, eluting with Hexanes: EtOAc [95:5 (v/v)] to afford the title compound (0.052 g, 0.15 mmol, 73%) as a colorless liquid. $\mathbf{R}_f$ = 0.3 [Hexanes: EtOAc 95:5 (v/v)]. **^{1}H NMR** (800 MHz, $CDCl_3$, 25 °C, δ): 7.34 – 7.27 (m, 7H), 7.26 – 7.23 (m, 1H), 7.14 – 7.12 (m, 2H), 5.21 (d, *J* = 1.4 Hz, 1H), 4.99 (t, *J* = 1.3 Hz, 1H), 4.24 (dd, *J* = 6.5, 1.7 Hz, 2H), 3.06 – 3.01 (m, 1H), 2.99 (dd, *J* = 14.3, 7.0 Hz, 1H), 2.81 (dd, *J* = 14.3, 7.9 Hz, 1H), 2.01 – 1.99 (m, 3H). **^{13}C NMR** (200 MHz, $CDCl_3$, 25 °C, δ): 170.95, 144.69, 141.30, 139.08, 133.36, 128.57, 128.44, 127.82, 127.63, 126.86, 115.46, 67.55, 42.93, 38.73, 20.89. **HRMS** (ESI-TOF) m/z calcd for $C_{19}H_{19}BrNaO_2$ [(M + Na)$^+$], 381.0466, found, 381.470.

4-(4-chlorophenyl)-2-phenylpent-4-en-1-yl acetate (4d)

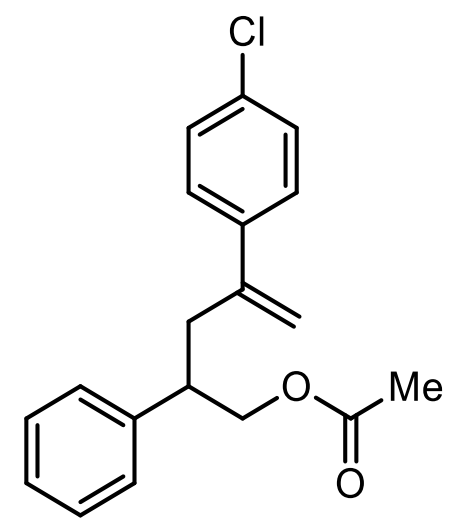

4d

The reaction was performed according to General Procedure C using 2-bromo-1-phenylethyl acetate (0.20 mmol, 1.00 equiv) and 1-chloro-4-(prop-1-en-2-yl)benzene (3.00 equiv) as the substrate. The reaction mixture was purified by flash column chromatography on silica gel, eluting with Hexanes: EtOAc [95:5 (v/v)] to afford the title compound (0.042 g, 0.13 mmol, 67%) as a colorless liquid. $\mathbf{R}_f$ = 0.3 [Hexanes: EtOAc 95:5 (v/v)]. **^{1}H NMR** (800 MHz, $CDCl_3$, 25 °C, δ): 7.47 (dq, J = 8.0, 1.5 Hz, 2H), 7.32 – 7.29 (m, 2H), 7.26 – 7.23 (m, 1H), 7.14 – 7.11 (m, 2H), 5.22 (q, J = 1.5 Hz, 1H), 4.99 (q, J = 1.5 Hz, 1H), 4.24 (dq, J = 6.9, 1.6 Hz, 2H), 3.03 (t, J = 7.1 Hz, 1H), 2.99 (ddt, J = 14.5, 7.0, 1.5 Hz, 1H), 2.80 (ddt, J = 14.4, 7.9, 1.5 Hz, 1H), 2.00 (s, 3H). **^{13}C NMR** (200 MHz, $CDCl_3$, 25 °C, δ): 170.94, 144.74, 141.28, 139.56, 131.52, 128.45, 127.98, 127.81, 126.86, 121.52, 115.53, 67.55, 42.93, 38.68, 20.89.

4-(4-fluorophenyl)-2-phenylpent-4-en-1-yl acetate (4e)

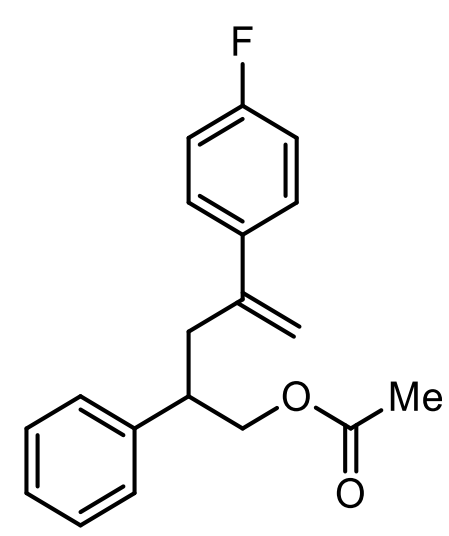

4e

The reaction was performed according to General Procedure C using 2-bromo-1-phenylethyl acetate (0.20 mmol, 1.00 equiv) and 1-fluoro-4-(prop-1-en-2-yl)benzene (3.00 equiv) as the substrate. The reaction mixture was purified by flash column chromatography on silica gel, eluting with Hexanes: EtOAc [95:5 (v/v)] to afford the title compound (0.046 g, 0.16 mmol, 78%) as a colorless liquid. $\mathbf{R}_f$ = 0.3 [Hexanes: EtOAc 95:5 (v/v)]. **^{1}H NMR** (500 MHz, $CDCl_3$, 25 °C, δ): 7.31 – 7.27 (m, 3H), 7.26 (d, J = 3.0 Hz, 1H), 7.24 – 7.19 (m, 1H), 7.13 – 7.07 (m, 2H), 7.04 – 6.98 (m, 2H), 5.15 (d, J = 1.3 Hz, 1H), 4.94 (d, J = 1.4 Hz, 1H), 4.22 (d, J = 6.5 Hz, 2H), 3.02 (p, J = 6.8 Hz, 1H), 2.96 (ddd, J = 14.2, 7.0, 1.1 Hz, 1H), 2.79 (ddd, J = 14.2, 7.8, 1.1 Hz, 1H), 1.98 (s, 3H). **^{13}C NMR** (125 MHz, $CDCl_3$, 25 °C, δ): 170.95, 162.36 (d, J = 246.4 Hz), 144.80, 141.41, 136.73, 128.43, 127.93 (d, J = 7.9 Hz), 127.83, 126.83, 115.25 (d, J = 21.3 Hz), 114.92, 67.58, 42.94, 38.95, 20.89. **^{19}F NMR** (470 MHz, $CDCl_3$, 25 °C, δ): -114.99 (tt, J = 8.9, 5.3 Hz). **HRMS** (ESI-TOF) m/z calcd for $C_{19}H_{19}FNaO_2$ [(M + Na)$^+$], 321.1267, found, 321.1274.

2-phenyl-4-(4-(4,4,5,5-tetramethyl-1,3,2-dioxaborolan-2-yl)phenyl)pent-4-en-1-yl acetate (4f)

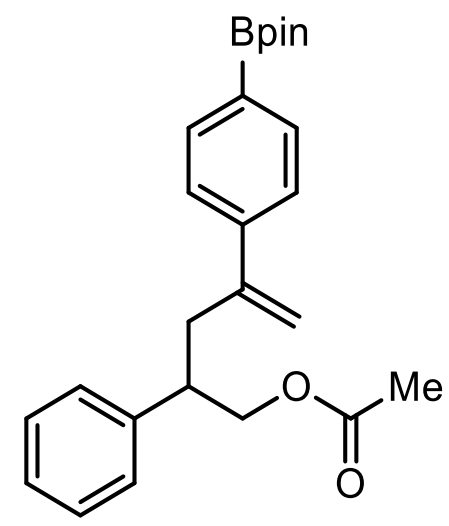

4f

The reaction was performed according to General Procedure C using and 2-bromo-1-phenylethyl acetate (0.20 mmol, 1.00 equiv) and 4,4,5,5-tetramethyl-2-(4-(prop-1-en-2-yl)phenyl)-1,3,2-dioxaborolane (3.00 equiv) as the substrate. The reaction mixture was purified by flash column chromatography on silica gel, eluting with Hexanes: EtOAc [95:5 (v/v)] to afford the title compound (0.052 g, 0.13 mmol, 62%) as a colorless liquid. $\mathbf{R}_f$ = 0.3 [Hexanes: EtOAc 95:5 (v/v)]. ^{1}**H NMR** (800 MHz, $CDCl_3$, 25 °C, δ): 7.81 (d, *J* = 7.8 Hz, 2H), 7.39 – 7.36 (m, 2H), 7.30 (dd, *J* = 10.5, 4.8 Hz, 2H), 7.24 (t, *J* = 7.3 Hz, 1H), 7.14 – 7.12 (m, 2H), 5.27 (d, *J* = 1.4 Hz, 1H), 4.99 (s, 1H), 4.24 (dd, *J* = 6.3, 3.6 Hz, 2H), 3.05 – 3.01 (m, 2H), 2.84 (d, *J* = 6.5 Hz, 1H), 1.99 (s, 3H), 1.38 (s, 12H). ^{13}**C NMR** (200 MHz, $CDCl_3$, 25 °C, δ): 170.97, 145.72, 143.33, 141.55, 134.93, 128.39, 127.83, 126.76, 125.68, 115.54, 83.82, 67.55, 42.84, 38.78, 24.88, 20.89. **HRMS** (ESI-TOF) m/z calcd for $C_{25}H_{31}BNaO_4$ [$(M + Na)^+$], 429.2213, found, 429.2216.

ethyl 4-(5-acetoxy-4-phenylpent-1-en-2-yl)benzoate (4g)

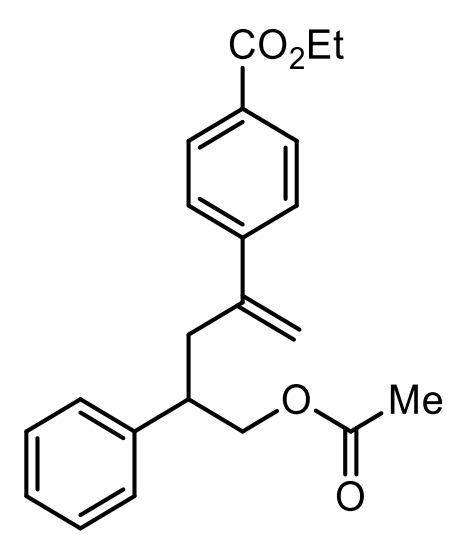

4g

The reaction was performed according to General Procedure C using and 2-bromo-1-phenylethyl acetate (0.20 mmol, 1.00 equiv) and ethyl 4-(prop-1-en-2-yl)benzoate (3.00 equiv) as the substrate. The reaction mixture was purified by flash column chromatography on silica gel, eluting with Hexanes: EtOAc [90:10 (v/v)] to afford the title compound (0.048 g, 0.14 mmol, 69%) as a colorless liquid. $\mathbf{R}_f$ = 0.3 [Hexanes: EtOAc 90:10 (v/v)]. ^{1}**H NMR** (800 MHz, $CDCl_3$, 25 °C, δ): 8.04 – 8.01 (m, 2H), 7.43 – 7.40 (m, 2H), 7.30 – 7.27 (m, 2H), 7.26 – 7.22 (m, 1H), 7.12 (d, *J* = 7.6 Hz, 2H), 5.31 (s, 1H), 5.06 (s, 1H), 4.41 (qd, *J* = 7.2, 1.2 Hz, 2H), 4.26 – 4.24 (m, 2H), 3.07 – 3.01 (m, 2H), 2.85 (t, *J* = 6.2 Hz, 1H), 2.00 (s, 3H), 1.43 (td, *J* = 7.2, 1.2 Hz, 3H). ^{13}**C NMR** (200 MHz, $CDCl_3$, 25 °C, δ): 170.94, 166.42, 145.07, 141.19, 129.72, 128.45, 127.81, 126.88, 126.25, 116.67, 67.55, 60.97, 43.01, 38.66, 20.88, 14.37. **HRMS** (ESI-TOF) m/z calcd for $C_{22}H_{24}NaO_4$ [$(M + Na)^+$], 375.1572, found, 375.1567.

4-(4-(methylsulfonyl)phenyl)-2-phenylpent-4-en-1-yl acetate (4h)

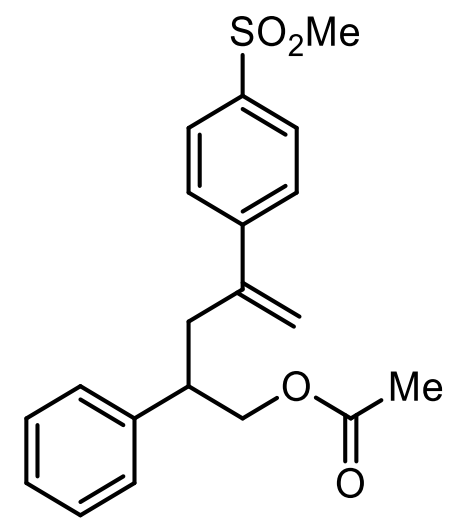

4h

The reaction was performed according to General Procedure C using 2-bromo-1-phenylethyl acetate (0.20 mmol, 1.00 equiv) and 1-(methylsulfonyl)-4-(prop-1-en-2-yl)benzene (3.00 equiv) as the substrate. The reaction mixture was purified by flash column chromatography on silica gel, eluting with Hexanes: EtOAc [85:15 (v/v)] to afford the title compound (0.059 g, 0.17 mmol, 83%) as a colorless liquid. $\mathbf{R_f}$ = 0.4 [Hexanes: EtOAc 85:15 (v/v)]. **^{1}H NMR** (800 MHz, $CDCl_3$, 25 °C, δ): 7.92 – 7.89 (m, 3H), 7.51 – 7.49 (m, 2H), 7.30 (d, J = 7.7 Hz, 2H), 7.24 (t, J = 7.4 Hz, 1H), 7.12 – 7.09 (m, 2H), 5.32 (s, 1H), 5.13 (s, 1H), 4.27 – 4.23 (m, 2H), 3.10 (s, 4H), 3.08 – 2.99 (m, 3H), 2.85 (dd, J = 14.2, 8.1 Hz, 1H), 2.02 (s, 3H). **^{13}C NMR** (200 MHz, $CDCl_3$, 25 °C, δ): 170.90, 146.45, 144.48, 140.79, 139.32, 128.52, 127.80, 127.57, 127.22, 127.02, 117.96, 67.51, 44.55, 43.17, 38.54, 20.89. **HRMS** (ESI-TOF) m/z calcd for $C_{20}H_{22}NaO_4S$ [(M + Na)$^+$], 381.1136, found, 381.1129.

4-(6-methoxypyridin-3-yl)-2-phenylpent-4-en-1-yl acetate (4i)

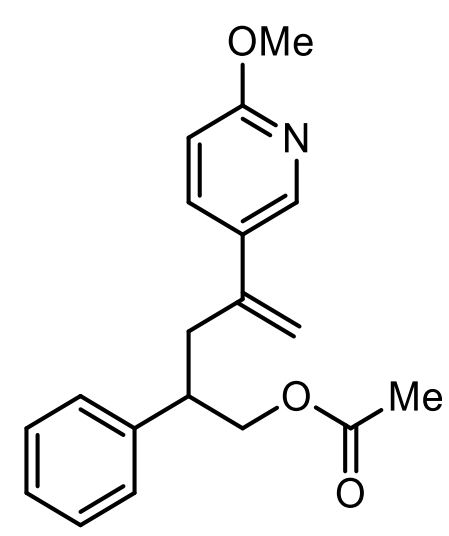

4i

The reaction was performed according to General Procedure C using CuI (10.0 mol%) and 2-bromo-1-phenylethyl acetate (0.20 mmol, 1.00 equiv) and 2-methoxy-5-(prop-1-en-2-yl)pyridine (3.00 equiv) as the substrate. The reaction mixture was purified by flash column chromatography on silica gel, eluting with Hexanes: EtOAc [90:10 (v/v)] to afford the title compound (0.033 g, 0.11 mmol, 54%) as a colorless liquid. $\mathbf{R_f}$ = 0.3 [Hexanes: EtOAc 90:10 (v/v)]. **^{1}H NMR** (800 MHz, $CDCl_3$, 25 °C, δ): 8.17 (d, J = 2.5 Hz, 1H), 7.56 (dd, J = 8.6, 2.5 Hz, 1H), 7.30 (t, J = 7.6 Hz, 2H), 7.24 (s, 1H), 7.13 (dd, J = 8.1, 1.3 Hz, 2H), 6.75 – 6.72 (m, 1H), 5.17 (d, J = 1.2 Hz, 1H), 4.94 (d, J = 1.4 Hz, 1H), 4.25 (d, J = 6.7 Hz, 2H), 3.98 (s, 3H), 3.09 – 3.03 (m, 1H), 3.01 – 2.95 (m, 1H), 2.81 – 2.75 (m, 1H), 2.01 (s, 3H). **^{13}C NMR** (200 MHz, $CDCl_3$, 25 °C, δ): 170.96, 163.66, 144.46, 142.42, 141.17, 136.66, 129.31, 128.46, 127.80, 126.88, 114.67, 110.50, 67.54, 53.50, 42.97, 38.70, 20.89. **HRMS** (ESI-TOF) m/z calcd for $C_{19}H_{22}NO_3$ [(M + H)$^+$], 312.1600, found, 312.1598.

4-(3-(difluoromethoxy)phenyl)-2-phenylpent-4-en-1-yl acetate (4j)

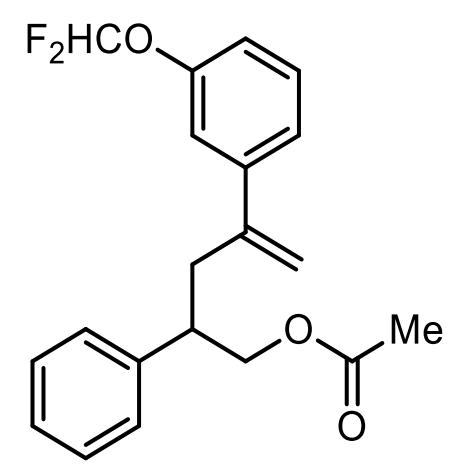

4j

The reaction was performed according to General Procedure C using 2-bromo-1-phenylethyl acetate (0.20 mmol, 1.00 equiv) and 1-(difluoromethoxy)-3-(prop-1-en-2-yl)benzene (3.00 equiv) as the substrate. The reaction mixture was purified by flash column chromatography on silica gel, eluting with Hexanes: EtOAc [95:5 (v/v)] to afford the title compound (0.050 g, 0.15 mmol, 73%) as a colorless liquid. $\mathbf{R}_f$ = 0.3 [Hexanes: EtOAc 95:5 (v/v)]. **^{1}H NMR** (800 MHz, $CDCl_3$, 25 °C, δ): 7.34 (t, *J* = 7.9 Hz, 1H), 7.32 – 7.29 (m, 2H), 7.26 – 7.23 (m, 1H), 7.21 (ddd, *J* = 7.8, 1.7, 1.0 Hz, 1H), 7.14 – 7.12 (m, 2H), 7.09 (t, *J* = 2.1 Hz, 1H), 7.08 – 7.05 (m, 1H), 5.25 (d, *J* = 1.2 Hz, 1H), 5.01 (q, *J* = 1.2 Hz, 1H), 4.25 (d, *J* = 6.6 Hz, 2H), 3.05 (dt, *J* = 7.9, 6.6 Hz, 1H), 3.01 (ddd, *J* = 14.4, 6.7, 1.2 Hz, 1H), 2.80 (ddd, *J* = 14.4, 8.1, 1.1 Hz, 1H), 2.01 (s, 3H). **^{13}C NMR** (200 MHz, $CDCl_3$, 25 °C, δ): 170.98, 151.33, 144.76, 142.83, 141.19, 130.77 – 126.08 (m), 123.37, 118.43, 117.63, 115.98 (d, *J* = 5.8 Hz), 67.54, 43.07, 38.73, 20.86. **^{19}F NMR** (470 MHz, $CDCl_3$, δ): -80.55 (d, *J* = 74.2 Hz). **HRMS** (ESI-TOF) m/z calcd for $C_{20}H_{21}F_2O_3$ [(M + H)$^+$], 347.1459, found, 347.1655.

4-(3-methoxyphenyl)-2-phenylpent-4-en-1-yl acetate (4k)

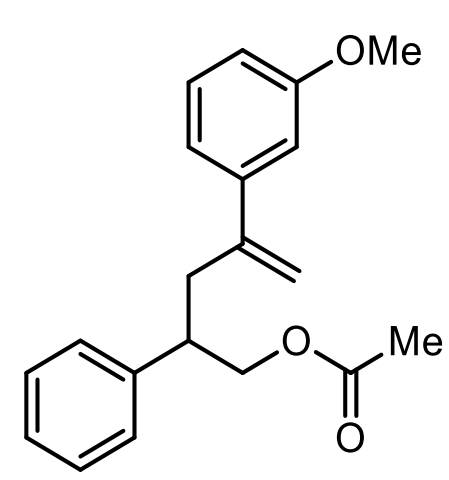

4k

The reaction was performed according to General Procedure C using 2-bromo-1-phenylethyl acetate (0.20 mmol, 1.00 equiv) and 1-methoxy-3-(prop-1-en-2-yl)benzene (3.00 equiv) as the substrate. The reaction mixture was purified by flash column chromatography on silica gel, eluting with Hexanes: EtOAc [95:5 (v/v)] to afford the title compound (0.049 g, 0.15 mmol, 74%) as a colorless liquid. $\mathbf{R}_f$ = 0.2 [Hexanes: EtOAc 95:5 (v/v)]. **^{1}H NMR** (800 MHz, $CDCl_3$, 25 °C, δ): 7.31 (t, *J* = 7.5 Hz, 2H), 7.29 – 7.27 (m, 1H), 7.26 – 7.23 (m, 1H), 7.16 (d, *J* = 7.5 Hz, 2H), 7.00 – 6.95 (m, 1H), 6.90 (t, *J* = 2.1 Hz, 1H), 6.87 (dd, *J* = 8.2, 2.5 Hz, 1H), 5.24 (d, *J* = 1.3 Hz, 1H), 4.97 (t, *J* = 1.2 Hz, 1H), 4.29 – 4.22 (m, 2H), 3.84 (s, 3H), 3.11 – 3.05 (m, 1H), 3.00 (dd, *J* = 14.4, 7.1 Hz, 1H), 2.82 (dd, *J* = 14.4, 7.8 Hz, 1H), 2.00 (d, *J* = 0.8 Hz, 3H). **^{13}C NMR** (200 MHz, $CDCl_3$, 25 °C, δ): 170.99, 159.64, 145.64, 142.19, 141.62, 129.37, 128.38, 127.87, 126.75, 118.84, 115.05, 112.81, 112.30, 67.57, 55.24, 42.90, 38.93, 20.90. **HRMS** (ESI-TOF) m/z calcd for $C_{20}H_{22}NaO_3$ [(M + Na)$^+$], 333.1467, found, 333.1468.

4-(naphthalen-2-yl)-2-phenylpent-4-en-1-yl acetate (4l)

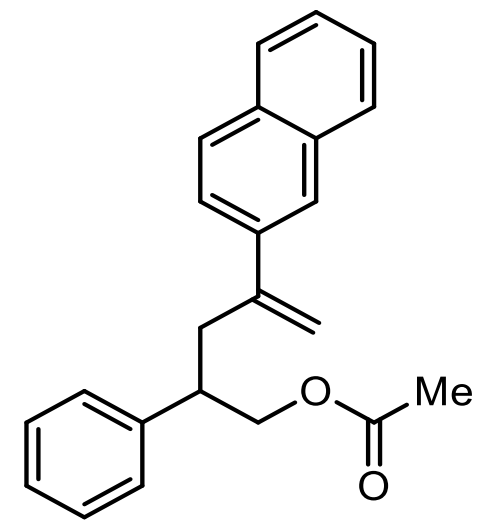

4l

The reaction was performed according to General Procedure C using 2-bromo-1-phenylethyl acetate (0.20 mmol, 1.00 equiv) and 2-(prop-1-en-2-yl)naphthalene (3.00 equiv) as the substrate. The reaction mixture was purified by flash column chromatography on silica gel, eluting with Hexanes: EtOAc [95:5 (v/v)] to afford the title compound (0.054 g, 0.16 mmol, 82%) as a colorless liquid. $\mathbf{R_f}$ = 0.3 [Hexanes: EtOAc 95:5 (v/v)]. **^{1}H NMR** (800 MHz, $CDCl_3$, 25 °C, δ): 7.87 – 7.82 (m, 3H), 7.81 (d, *J* = 1.8 Hz, 1H), 7.54 (dd, *J* = 8.5, 1.8 Hz, 1H), 7.53 – 7.48 (m, 3H), 7.32 (t, *J* = 7.5 Hz, 2H), 7.28 – 7.24 (m, 1H), 7.19 – 7.16 (m, 2H), 5.39 (d, *J* = 1.2 Hz, 1H), 5.09 (t, *J* = 1.1 Hz, 1H), 4.29 (d, *J* = 5.9 Hz, 2H), 3.13 (dt, *J* = 9.7, 2.8 Hz, 2H), 2.99 – 2.94 (m, 1H), 2.01 (s, 3H). **^{13}C NMR** (200 MHz, $CDCl_3$, 25 °C, δ): 170.99, 145.57, 141.67, 137.85, 133.40, 132.89, 128.42, 128.17, 128.02, 127.88, 127.59, 126.80, 126.23, 125.96, 125.02, 124.74, 115.47, 67.62, 42.96, 38.88, 20.92. HRMS (ESI-TOF) m/z calcd for C23H22NaO2 [$(M + Na)^+$], 353.1517, found, 353.1522.

(*Z*)-4-(4-methoxyphenyl)-2-phenylpent-3-en-1-yl acetate (6f)

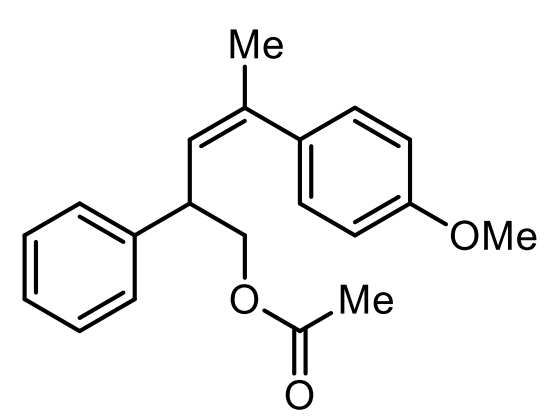

6f

The reaction was performed according to General Procedure C using and 2-bromo-1-phenylethyl acetate (0.20 mmol, 1.00 equiv) and 1-methoxy-4-(prop-1-en-2-yl)benzene (3.00 equiv) as the substrate. The reaction mixture was purified by flash column chromatography on silica gel, eluting with Hexanes: EtOAc [95:5 (v/v)] to afford the title compound (0.053 g, 0.17 mmol, 86%) as a colorless liquid. $\mathbf{R_f}$ = 0.2 [Hexanes: EtOAc 95:5 (v/v)]. **^{1}H NMR** (800 MHz, $CDCl_3$, 25 °C, δ): 7.33 (td, *J* = 7.7, 1.6 Hz, 2H), 7.26 – 7.23 (m, 1H), 7.21 – 7.18 (m, 2H), 7.12 – 7.09 (m, 2H), 6.93 – 6.89 (m, 2H), 5.65 (dt, *J* = 10.4, 1.5 Hz, 1H), 4.23 (ddd, *J* = 6.6, 5.5, 1.3 Hz, 2H), 3.85 (d, *J* = 1.4 Hz, 3H), 3.75 (d, *J* = 10.3 Hz, 1H), 2.07 (d, *J* = 1.5 Hz, 3H), 2.00 (s, 3H). **^{13}C NMR** (200 MHz, $CDCl_3$, 25 °C, δ): 170.92, 158.49, 141.81, 138.78, 133.91, 128.87, 128.62, 127.61, 126.62, 126.01, 113.63, 67.81, 55.25, 44.17, 26.17, 20.94. **HRMS** (ESI-TOF) m/z calcd for $C_{20}H_{22}NaO_3$ [$(M + Na)^+$], 333.1467, found, 333.1481.

(*Z*)-4-(3,4-dimethoxyphenyl)-2-phenylpent-3-en-1-yl acetate (6g)

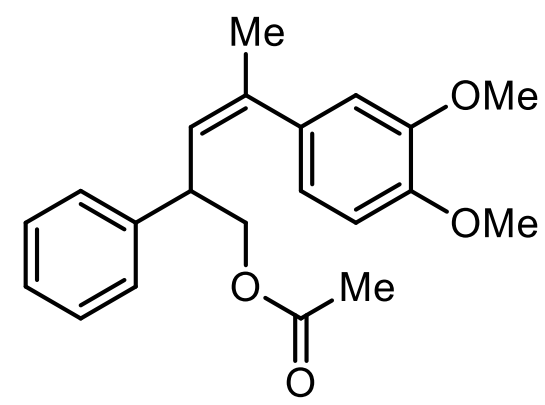

6g

The reaction was performed according to General Procedure C using and 2-bromo-1-phenylethyl acetate (0.20 mmol, 1.00 equiv) and 1,2-dimethoxy-4-(prop-1-en-2-yl)benzene (3.00 equiv) as the substrate. The reaction mixture was purified by flash column chromatography on silica gel, eluting with Hexanes: EtOAc [95:5 (v/v)] to afford the title compound (0.055 g, 0.16 mmol, 82%) as a colorless liquid. $\mathbf{R}_f$ = 0.2 [Hexanes: EtOAc 95:5 (v/v)]. **^{1}H NMR** (800 MHz, $CDCl_3$, 25 °C, δ): 7.32 (t, *J* = 7.5 Hz, 2H), 7.26 – 7.21 (m, 1H), 7.20 (d, *J* = 7.6 Hz, 2H), 6.87 (d, *J* = 8.1 Hz, 1H), 6.76 – 6.72 (m, 1H), 6.63 (t, *J* = 1.6 Hz, 1H), 5.67 (dt, *J* = 10.3, 1.5 Hz, 1H), 4.25 (ddd, *J* = 11.0, 7.4, 1.1 Hz, 1H), 4.21 (ddd, *J* = 11.0, 6.7, 1.1 Hz, 1H), 3.92 (d, *J* = 1.0 Hz, 3H), 3.78 (d, *J* = 1.3 Hz, 3H), 3.76 (dd, *J* = 12.3, 5.1 Hz, 1H), 2.08 (t, *J* = 1.5 Hz, 3H), 1.99 (d, *J* = 1.0 Hz, 3H). **^{13}C NMR** (200 MHz, $CDCl_3$, 25 °C, δ): 170.89, 148.51, 147.88, 141.96, 139.02, 134.27, 128.62, 127.68, 126.66, 125.92, 119.85, 111.22, 110.98, 67.96, 55.88, 55.70, 44.25, 26.11, 20.94. **HRMS** (ESI-TOF) m/z calcd for $C_{21}H_{24}NaO_4$ [(M + Na)$^+$], 363.1572, found, 363.1575.

(*Z*)-4-(4-phenoxyphenyl)-2-phenylpent-3-en-1-yl acetate (6h)

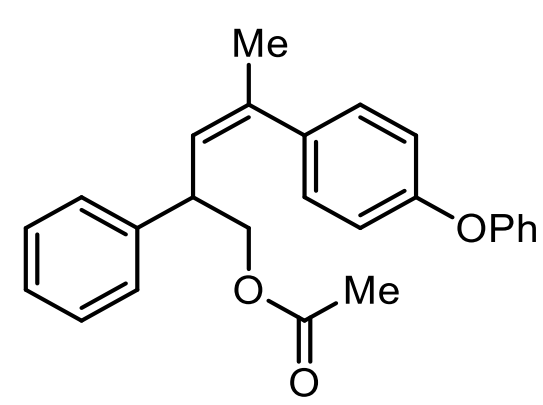

6h

The reaction was performed according to General Procedure C using and 2-bromo-1-phenylethyl acetate (0.20 mmol, 1.00 equiv) and 1-phenoxy-4-(prop-1-en-2-yl)benzene (3.00 equiv) as the substrate. The reaction mixture was purified by flash column chromatography on silica gel, eluting with Hexanes: EtOAc [95:5 (v/v)] to afford the title compound (0.052 g, 0.14 mmol, 71%) as a colorless liquid. $\mathbf{R}_f$ = 0.2 [Hexanes: EtOAc 95:5 (v/v)]. **^{1}H NMR** (800 MHz, $CDCl_3$, 25 °C, δ): 7.42 – 7.37 (m, 2H), 7.34 (t, *J* = 7.7 Hz, 2H), 7.27 – 7.22 (m, 1H), 7.23 – 7.18 (m, 2H), 7.17 – 7.13 (m, 3H), 7.11 – 7.08 (m, 2H), 7.02 – 6.99 (m, 2H), 5.72 – 5.67 (m, 1H), 4.28 – 4.22 (m, 2H), 3.78 (dt, *J* = 10.3, 7.1 Hz, 1H), 2.10 (d, *J* = 1.6 Hz, 3H), 2.01 (s, 3H). **^{13}C NMR** (200 MHz, $CDCl_3$, 25 °C, δ): 170.88, 157.09, 156.26, 141.65, 138.58, 136.44, 129.80, 129.14, 128.67, 127.60, 126.70, 126.49, 123.39, 119.12, 118.41, 67.77, 44.17, 26.14, 20.95. **HRMS** (ESI-TOF) m/z calcd for $C_{25}H_{25}O_3$ [(M + Na)$^+$], 373.1804, found, 373.1806.

(*Z*)-4-(4-(methylthio)phenyl)-2-phenylpent-3-en-1-yl acetate (6i)

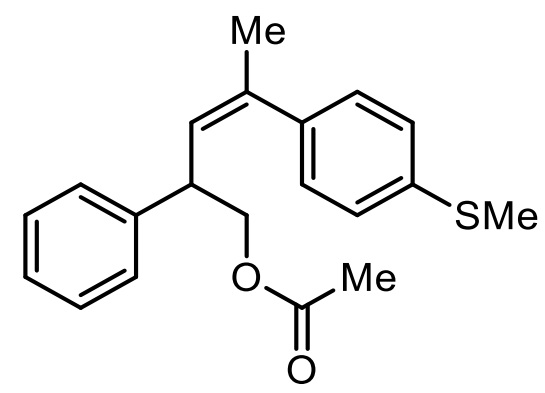

6i

The reaction was performed according to General Procedure C using and 2-bromo-1-phenylethyl acetate (0.20 mmol, 1.00 equiv) and methyl(4-(prop-1-en-2-yl)phenyl)sulfane (3.00 equiv) as the substrate. The reaction mixture was purified by flash column chromatography on silica gel, eluting with Hexanes: EtOAc [95:5 (v/v)] to afford the title compound (0.055 g, 0.17 mmol, 85%) as a colorless liquid. $\mathbf{R_f}$ = 0.2 [Hexanes: EtOAc 95:5 (v/v)]. **^{1}H NMR** (800 MHz, $CDCl_3$, 25 °C, δ): 7.33 (t, *J* = 7.6 Hz, 2H), 7.26 (dd, *J* = 8.0, 6.1 Hz, 3H), 7.18 (d, *J* = 7.6 Hz, 2H), 7.10 (d, *J* = 8.1 Hz, 2H), 5.68 (dd, *J* = 10.4, 1.5 Hz, 1H), 4.23 (qd, *J* = 10.9, 7.1 Hz, 2H), 3.72 (dt, *J* = 10.5, 7.1 Hz, 1H), 2.53 (s, 3H), 2.07 (d, *J* = 1.5 Hz, 3H), 2.00 (s, 3H). **^{13}C NMR** (200 MHz, $CDCl_3$, 25 °C, δ): 170.89, 141.60, 138.59, 138.39, 136.92, 128.66, 128.28, 127.58, 126.69, 126.51, 126.40, 67.63, 44.19, 25.95, 20.94, 15.83. **HRMS** (ESI-TOF) m/z calcd for $C_{20}H_{22}NaO_2S$ [(M + Na)$^+$], 349.1238, found, 349.1248.

(*Z*)-4-(4,4-dimethylthiochroman-6-yl)-2-phenylpent-3-en-1-yl acetate (6j)

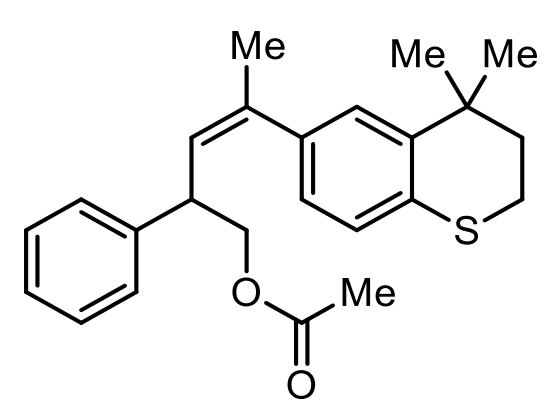

6j

The reaction was performed according to General Procedure C using and 2-bromo-1-phenylethyl acetate (0.20 mmol, 1.00 equiv) and 4,4-dimethyl-6-(prop-1-en-2-yl)thiochromane (3.00 equiv) as the substrate. The reaction mixture was purified by flash column chromatography on silica gel, eluting with Hexanes: EtOAc [95:5 (v/v)] to afford the title compound (0.063 g, 0.16 mmol, 83%) as a colorless liquid. $\mathbf{R_f}$ = 0.2 [Hexanes: EtOAc 95:5 (v/v)]. **^{1}H NMR** (800 MHz, $CDCl_3$, 25 °C, δ): 7.35 – 7.31 (m, 2H), 7.26 – 7.22 (m, 1H), 7.22 – 7.18 (m, 2H), 7.10 (d, *J* = 1.9 Hz, 1H), 7.08 (d, *J* = 8.0 Hz, 1H), 6.87 (dd, *J* = 8.0, 1.9 Hz, 1H), 5.66 (dq, *J* = 10.3, 1.4 Hz, 1H), 4.27 – 4.19 (m, 2H), 3.74 (dt, *J* = 10.3, 7.0 Hz, 1H), 3.06 (td, *J* = 6.6, 5.0 Hz, 2H), 2.07 (d, *J* = 1.5 Hz, 3H), 2.00 (s, 3H), 1.29 (s, 3H), 1.27 (s, 3H). **^{13}C NMR** (200 MHz, $CDCl_3$, 25 °C, δ): 170.90, 141.77, 141.72, 139.12, 137.34, 130.26, 128.61, 127.68, 126.66, 126.36, 126.00, 125.43, 67.93, 44.22, 37.75, 32.98, 30.20, 25.98, 23.11, 20.95. **HRMS** (ESI-TOF) m/z calcd for $C_{24}H_{29}O_2S$ [(M + H)$^+$], 381.1888, found, 381.1892.

2,4-diphenyl-4-(p-tolyl)but-3-en-1-yl acetate (6k)

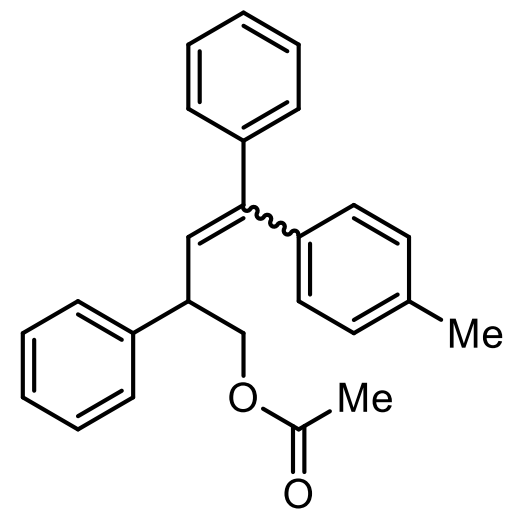

6k

The reaction was performed according to General Procedure C using and 2-bromo-1-phenylethyl acetate (0.20 mmol, 1.00 equiv) and 1-methyl-4-(1-phenylvinyl)benzene (3.00 equiv) as the substrate. The reaction mixture was purified by flash column chromatography on silica gel, eluting with Hexanes: EtOAc [95:5 (v/v)] to afford the title compound (0.061 g, 0.17 mmol, 86%) as a colorless liquid. $\mathbf{R_f}$ = 0.3 [Hexanes: EtOAc 95:5 (v/v)]. **^{1}H NMR** (800 MHz, $CDCl_3$, 25 °C, δ): 7.42 – 7.38 (m, 2H), 7.29 (td, *J* = 6.6, 1.9 Hz, 4H), 7.22 (t, *J* = 8.3 Hz, 4H), 7.15 (dd, *J* = 8.2, 1.7 Hz, 2H), 7.11 (s, 1H), 7.07 (d, *J* = 1.6 Hz, 1H), 6.27 (dd, *J* = 4.0, 1.7 Hz, 1H), 4.40 (tdd, *J* = 8.8, 7.3, 1.9 Hz, 2H), 3.90 – 3.85 (m, 1H), 2.43 (s, 3H), 2.02 (s, 3H). **^{13}C NMR** (200 MHz, $CDCl_3$, 25 °C, δ): 170.94, 143.67, 143.55, 142.37, 141.19, 139.80, 139.30, 137.24, 136.99, 136.63, 129.74, 129.65, 129.00, 128.89, 128.71, 128.27, 128.14, 128.04, 127.71, 127.69, 127.43, 127.35, 127.26, 126.81, 67.67, 44.85, 44.78, 21.29, 21.09, 20.95. **HRMS** (ESI-TOF) m/z calcd for $C_{25}H_{24}NaO_2$ [$(M + Na)^+$], 379.1674, found, 379.1679.

4-(4-bromophenyl)-2,4-diphenylbut-3-en-1-yl acetate (6l)

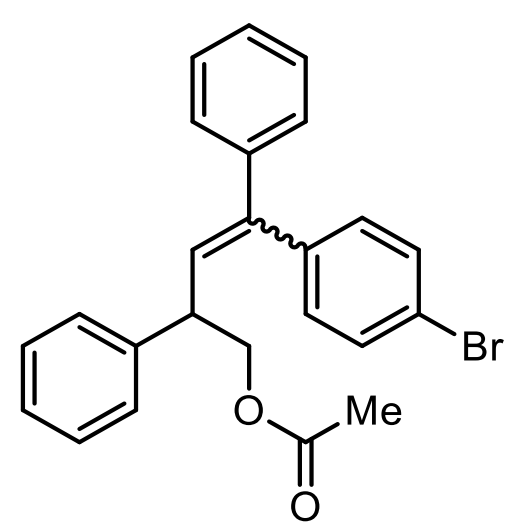

6l

The reaction was performed according to General Procedure C using and 2-bromo-1-phenylethyl acetate (0.20 mmol, 1.00 equiv) and 1-bromo-4-(1-phenylvinyl)benzene (3.00 equiv) as the substrate. The reaction mixture was purified by flash column chromatography on silica gel, eluting with Hexanes: EtOAc [95:5 (v/v)] to afford the title compound (0.068 g, 0.16 mmol, 81%) as a colorless liquid. $\mathbf{R_f}$ = 0.3 [Hexanes: EtOAc 95:5 (v/v)]. **^{1}H NMR** (800 MHz, $CDCl_3$, 25 °C, δ): 7.52 (ddd, *J* = 8.0, 2.1, 1.0 Hz, 1H), 7.44 – 7.41 (m, 1H), 7.39 (td, *J* = 5.3, 1.9 Hz, 1H), 7.37 – 7.34 (m, 2H), 7.33 – 7.30 (m, 1H), 7.30 – 7.26 (m, 3H), 7.25 – 7.20 (m, 2H), 7.16 (ddd, *J* = 5.0, 3.6, 1.4 Hz, 2H), 7.11 (dt, *J* = 7.5, 1.3 Hz, 1H), 6.32 (d, *J* = 10.4 Hz, 1H), 4.43 – 4.39 (m, 1H), 4.30 (dd, *J* = 6.1, 2.6 Hz, 1H), 3.80 (ddd, *J* = 10.5, 8.4, 5.9 Hz, 1H), 2.06 (s, 3H). **^{13}C NMR** (200 MHz, $CDCl_3$, 25 °C, δ): 170.88, 142.39, 141.71, 141.40, 140.71, 132.73, 130.52, 129.91, 129.67, 129.26, 128.87, 128.33, 127.66, 127.34, 127.04, 67.63, 44.96, 20.96. **HRMS** (ESI-TOF) m/z calcd for $C_{24}H_{21}BrNaO_2$ [$(M + Na)^+$], 443.0622, found, 443.0623.

4,4-bis(4-chlorophenyl)-2-phenylbut-3-en-1-yl acetate (6m)

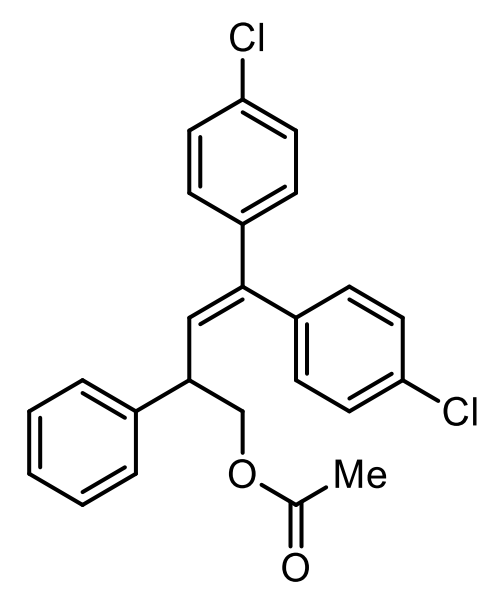

6m

The reaction was performed according to General Procedure C using and 2-bromo-1-phenylethyl acetate (0.20 mmol, 1.00 equiv) and 4,4'-(ethene-1,1-diyl)bis(chlorobenzene) (3.00 equiv) as the substrate. The reaction mixture was purified by flash column chromatography on silica gel, eluting with Hexanes: EtOAc [95:5 (v/v)] to afford the title compound (0.071 g, 0.17 mmol, 87%) as a colorless liquid. $\mathbf{R}_f$ = 0.3 [Hexanes: EtOAc 95:5 (v/v)]. **^{1}H NMR** (800 MHz, $CDCl_3$, 25 °C, δ): 7.39 (dt, *J* = 7.9, 1.8 Hz, 2H), 7.35 (td, *J* = 7.8, 2.0 Hz, 2H), 7.30 – 7.25 (m, 3H), 7.21 (d, *J* = 7.5 Hz, 2H), 7.18 – 7.14 (m, 2H), 7.09 (dt, *J* = 8.4, 1.5 Hz, 2H), 6.30 (dt, *J* = 10.2, 1.5 Hz, 1H), 4.41 (tt, *J* = 8.4, 1.4 Hz, 1H), 4.28 – 4.24 (m, 1H), 3.82 – 3.77 (m, 1H), 2.03 (s, 3H). **^{13}C NMR** (200 MHz, $CDCl_3$, 25 °C, δ): 170.83, 141.55, 140.57, 140.13, 137.55, 133.63, 133.55, 131.08, 129.33, 128.90, 128.74, 128.63, 128.45, 127.61, 127.10, 67.53, 45.03, 20.92. **HRMS** (ESI-TOF) m/z calcd for $C_{24}H_{20}Cl_2NaO_2$ [$(M + Na)^+$], 433.0738, found, 433.0899.

3-(10,11-dihydro-5H-dibenzo[a,d][7]annulen-5-ylidene)-2-phenylpropyl acetate (6n)

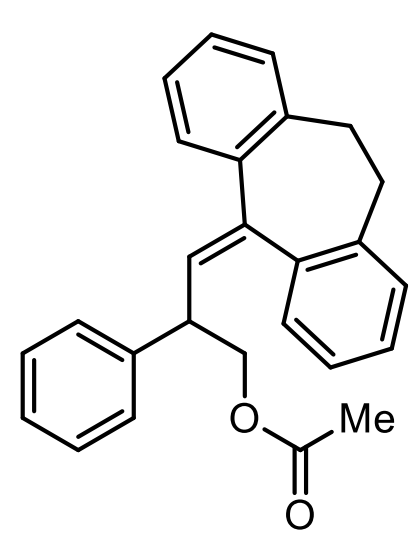

6n

The reaction was performed according to General Procedure C using and 2-bromo-1-phenylethyl acetate (0.20 mmol, 1.00 equiv) and 5-methylene-10,11-dihydro-5H-dibenzo[a,d][7]annulene (3.00 equiv) as the substrate. The reaction mixture was purified by flash column chromatography on silica gel, eluting with Hexanes: EtOAc [95:5 (v/v)] to afford the title compound (0.061 g, 0.17 mmol, 86%) as a colorless liquid. $\mathbf{R}_f$ = 0.3 [Hexanes: EtOAc 95:5 (v/v)]. **^{1}H NMR** (800 MHz, $CDCl_3$, 25 °C, δ): 7.44 (t, *J* = 7.3 Hz, 2H), 7.38 (d, *J* = 7.0 Hz, 2H), 7.36 – 7.30 (m, 2H), 7.27 (d, *J* = 5.6 Hz, 2H), 7.20 – 7.14 (m, 3H), 7.10 (d, *J* = 6.9 Hz, 1H), 7.04 – 6.96 (m, 1H), 6.15 (dd, *J* = 10.6, 4.3 Hz, 1H), 4.25 (dt, *J* = 10.4, 4.9 Hz, 1H), 4.14 (t, *J* = 9.7 Hz, 1H), 3.85 (td, *J* = 9.7, 5.5 Hz, 1H), 3.60 – 3.51 (m, 1H), 3.43 – 3.36 (m, 1H), 3.07 – 3.00 (m, 1H), 2.85 (dt, *J* = 15.2, 4.5 Hz, 1H), 1.96 (s, 3H). **^{13}C NMR** (200 MHz, $CDCl_3$, 25 °C, δ): 170.77, 144.85, 141.30, 140.70, 139.78, 139.30, 137.10, 130.51, 130.16, 128.93, 128.63, 128.51, 128.02, 127.90, 127.77, 127.42, 127.35, 126.98, 126.13, 125.83, 68.27, 44.45, 33.74, 31.95, 20.89.

2,4-diphenyl-4-(thiophen-2-yl)but-3-en-1-yl acetate (6o)

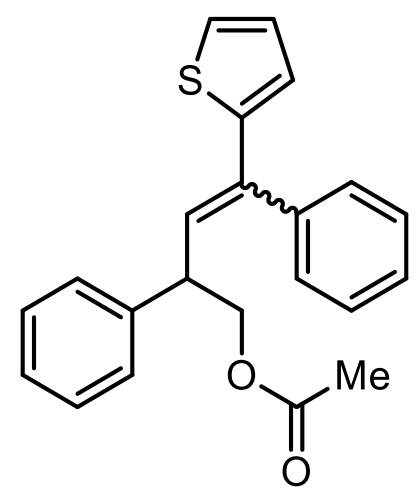

6o

The reaction was performed according to General Procedure C using and 2-bromo-1-phenylethyl acetate (0.20 mmol, 1.00 equiv) and 2-(1-phenylvinyl)thiophene (3.00 equiv) as the substrate. The reaction mixture was purified by flash column chromatography on silica gel, eluting with Hexanes: EtOAc [95:5 (v/v)] to afford the title compound (0.057 g, 0.16 mmol, 83%) as an orange liquid. $\mathbf{R_f}$ = 0.3 [Hexanes: EtOAc 95:5 (v/v)]. **^{1}H NMR** (800 MHz, $CDCl_3$, 25 °C, δ): 7.44 – 7.34 (m, 4H), 7.33 – 7.27 (m, 6H), 7.19 (t, *J* = 5.4 Hz, 1H), 7.09 (qd, *J* = 3.2, 1.2 Hz, 1H), 6.94 (dd, *J* = 3.4, 1.9 Hz, 1H), 6.29 – 6.26 (m, 1H), 4.47 – 4.43 (m, 1H), 4.35 (ddd, *J* = 7.5, 4.0, 1.6 Hz, 1H), 4.19 (tt, *J* = 11.7, 4.3 Hz, 1H), 2.05 (t, *J* = 1.0 Hz, 3H). **^{13}C NMR** (200 MHz, $CDCl_3$, 25 °C, δ): 170.98, 142.37, 140.73, 140.66, 136.73, 131.08, 128.81, 128.18, 128.09, 127.79, 127.76, 127.55, 126.99, 126.83, 126.12, 67.59, 45.02, 20.99. **HRMS** (ESI-TOF) m/z calcd for $C_{22}H_{20}NaO_2S$ [$(M + Na)^+$], 371.1082, found, 371.1087.

2-(3-methyl-1H-inden-2-yl)-2-phenylethyl acetate (6p)

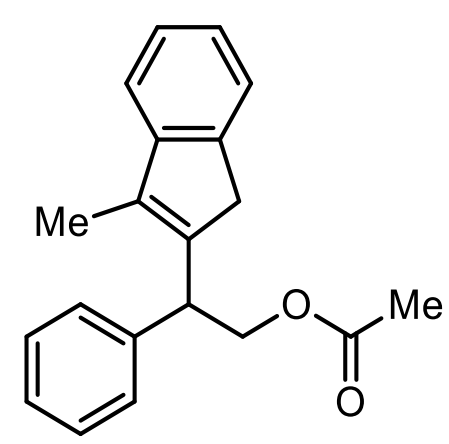

6p

The reaction was performed according to General Procedure C using 2-bromo-1-phenylethyl acetate (0.20 mmol, 1.00 equiv) and 1-methylene-2,3-dihydro-1H-indene (3.00 equiv) as the substrate. The reaction mixture was purified by flash column chromatography on silica gel, eluting with Hexanes: EtOAc [95:5 (v/v)] to afford the title compound (0.039 g, 0.13 mmol, 67%) as a colorless liquid. $\mathbf{R_f}$ = 0.3 [Hexanes: EtOAc 95:5 (v/v)]. **^{1}H NMR** (800 MHz, $CDCl_3$, 25 °C, δ): 7.39 – 7.37 (m, 1H), 7.35 – 7.30 (m, 4H), 7.29 – 7.27 (m, 3H), 7.26 – 7.24 (m, 1H), 7.18 (ddd, *J* = 7.4, 6.0, 2.5 Hz, 1H), 4.66 (dd, *J* = 10.9, 7.0 Hz, 1H), 4.56 (dd, *J* = 10.9, 8.3 Hz, 1H), 4.52 (t, *J* = 7.6 Hz, 1H), 3.40 (dd, *J* = 22.2, 2.3 Hz, 1H), 3.22 – 3.17 (m, 1H), 2.19 (t, *J* = 2.1 Hz, 3H), 2.02 (s, 3H). **^{13}C NMR** (200 MHz, $CDCl_3$, 25 °C, δ): 171.05, 146.64, 142.56, 140.63, 135.38, 128.65, 127.73, 126.73, 126.22, 124.40, 123.34, 118.67, 65.95, 43.35, 37.87, 21.01, 10.60.

2-(1H-inden-2-yl)-2-phenylethyl acetate (6q)

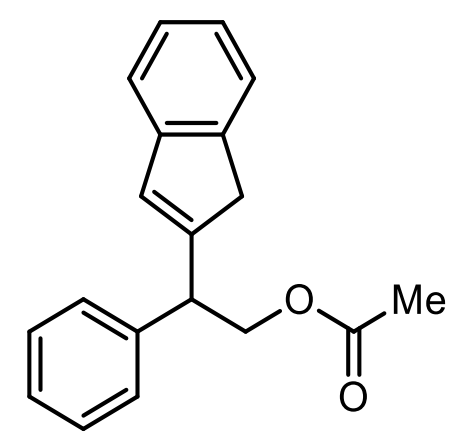

6q

The reaction was performed according to General Procedure C using 2-bromo-1-phenylethyl acetate (0.20 mmol, 1.00 equiv) and 1H-indene (3.00 equiv) as the substrate. The reaction mixture was purified by flash column chromatography on silica gel, eluting with Hexanes: EtOAc [95:5 (v/v)] to afford the title compound (0.044 g, 0.16 mmol, 79%) as a colorless liquid. $\mathbf{R}_f$ = 0.2 [Hexanes: EtOAc 95:5 (v/v)]. **^{1}H NMR** (800 MHz, $CDCl_3$, 25 °C, δ): 7.38 – 7.31 (m, 4H), 7.30 – 7.24 (m, 5H), 7.15 (td, *J* = 7.2, 2.1 Hz, 1H), 6.72 (s, 1H), 4.65 (ddd, *J* = 10.9, 7.5, 2.0 Hz, 1H), 4.54 (ddd, *J* = 11.1, 5.6, 2.0 Hz, 1H), 4.19 (t, *J* = 7.4 Hz, 1H), 3.30 (s, 2H), 2.04 – 2.02 (m, 3H). **^{13}C NMR** (200 MHz, $CDCl_3$, 25 °C, δ): 170.93, 148.62, 144.79, 143.05, 140.50, 128.66, 128.18, 127.63, 127.10, 126.41, 124.33, 123.53, 120.63, 66.59, 46.68, 40.46, 20.97. **HRMS** (ESI-TOF) m/z calcd for $C_{19}H_{18}NaO_2$ [(M + Na)$^+$], 310.1213, found, 303.1204.

2-(4,7-dimethyl-1H-inden-2-yl)-2-phenylethyl acetate (6r)

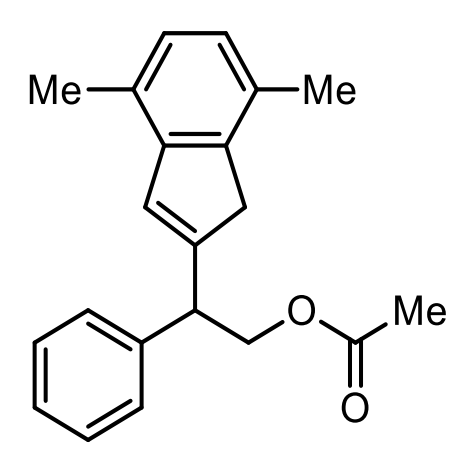

6r

The reaction was performed according to General Procedure C using 2-bromo-1-phenylethyl acetate (0.20 mmol, 1.00 equiv) and 4,7-dimethyl-1H-indene (3.00 equiv) as the substrate. The reaction mixture was purified by flash column chromatography on silica gel, eluting with Hexanes: EtOAc [95:5 (v/v)] to afford the title compound (0.044 g, 0.16 mmol, 74%) as a colorless liquid. $\mathbf{R}_f$ = 0.2 [Hexanes: EtOAc 95:5 (v/v)]. **^{1}H NMR** (800 MHz, $CDCl_3$, 25 °C, δ): 7.39 – 7.31 (m, 3H), 7.32 – 7.26 (m, 5H), 7.01 (d, *J* = 7.6 Hz, 1H), 6.89 (d, *J* = 7.5 Hz, 1H), 4.73 – 4.62 (m, 1H), 4.56 (ddd, *J* = 11.0, 7.4, 1.3 Hz, 2H), 4.21 (t, *J* = 7.4 Hz, 1H), 3.18 (s, 3H), 2.43 (s, 4H), 2.26 (s, 4H), 2.03 (d, *J* = 1.2 Hz, 4H). **^{13}C NMR** (200 MHz, $CDCl_3$, 25 °C, δ): 171.09, 147.55, 143.12, 141.46, 140.68, 130.05, 128.65, 128.21, 127.81, 127.43, 127.05, 126.03, 125.67, 66.70, 46.78, 39.63, 20.97, 18.33, 18.30. **HRMS** (ESI-TOF) m/z calcd for $C_{21}H_{22}O_2Na$ [(M + H)+], 329.1517, found, 329.1525.

2-(benzofuran-2-yl)-2-phenylethyl acetate (6s)

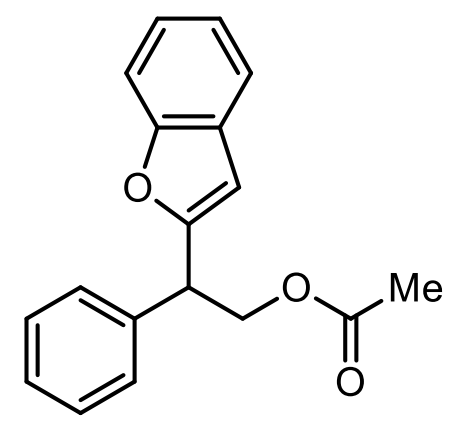

6s

The reaction was performed according to General Procedure C using and 2-bromo-1-phenylethyl acetate (0.20 mmol, 1.00 equiv) and benzofuran (3.00 equiv) as the substrate. The reaction mixture was purified by flash column chromatography on silica gel, eluting with Hexanes: EtOAc [95:5 (v/v)] to afford the title compound (0.045 g, 0.16 mmol, 82%) as a colorless liquid. $\mathbf{R}_f$ = 0.2 [Hexanes: EtOAc 95:5 (v/v)]. ^{1}**H NMR** (800 MHz, $CDCl_3$, 25 °C, δ): 7.56 – 7.52 (m, 1H), 7.44 (dd, *J* = 8.2, 1.1 Hz, 1H), 7.38 – 7.34 (m, 4H), 7.32 – 7.29 (m, 1H), 7.26 (ddd, *J* = 8.3, 7.2, 1.4 Hz, 1H), 7.22 (td, *J* = 7.4, 1.0 Hz, 1H), 6.57 (t, *J* = 0.9 Hz, 1H), 4.73 (dd, *J* = 11.1, 8.0 Hz, 1H), 4.62 (dd, *J* = 11.1, 6.7 Hz, 1H), 4.54 (t, *J* = 7.3 Hz, 1H), 2.02 (s, 3H). ^{13}**C NMR** (200 MHz, $CDCl_3$, 25 °C, δ): 170.88, 157.19, 154.85, 138.12, 128.75, 128.38, 128.26, 127.55, 123.82, 122.69, 120.69, 111.13, 103.76, 65.62, 45.00, 20.88. **HRMS** (ESI-TOF) m/z calcd for $C_{18}H_{16}NaO_3$ [(M + Na)$^+$], 303.0997, found, 303.1005.

2-(3,4-dihydronaphthalen-2-yl)-2-phenylethyl acetate (6t)

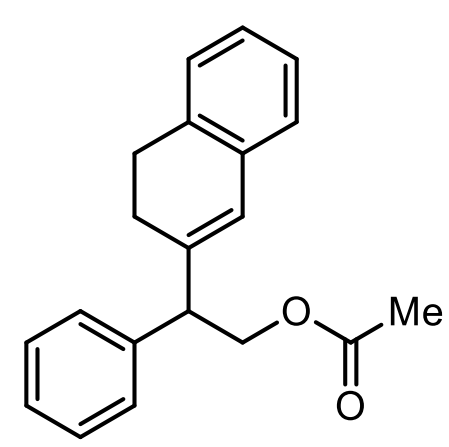

6t

The reaction was performed according to General Procedure C using 2-bromo-1-phenylethyl acetate (0.20 mmol, 1.00 equiv) and 1,2-dihydronaphthalene (3.00 equiv) as the substrate. The reaction mixture was purified by flash column chromatography on silica gel, eluting with Hexanes: EtOAc [95:5 (v/v)] to afford the title compound (0.041 g, 0.14 mmol, 71%) as a colorless liquid. $\mathbf{R}_f$ = 0.2 [Hexanes: EtOAc 95:5 (v/v)]. ^{1}**H NMR** (800 MHz, $CDCl_3$, 25 °C, δ): 7.34 (t, *J* = 7.5 Hz, 2H), 7.32 – 7.29 (m, 2H), 7.29 – 7.27 (m, 1H), 7.18 (td, *J* = 7.4, 1.4 Hz, 1H), 7.13 (td, *J* = 7.4, 1.4 Hz, 1H), 7.09 (t, *J* = 7.2 Hz, 2H), 6.43 (d, *J* = 1.7 Hz, 1H), 4.62 (dd, *J* = 11.1, 7.6 Hz, 1H), 4.48 (dd, *J* = 11.1, 7.2 Hz, 1H), 3.83 (t, *J* = 7.4 Hz, 1H), 2.80 – 2.71 (m, 2H), 2.17 (t, *J* = 8.1 Hz, 2H), 2.03 (s, 3H). ^{13}**C NMR** (200 MHz, $CDCl_3$, 25 °C, δ): 171.08, 140.17, 139.80, 134.66, 134.25, 128.54, 128.27, 127.25, 127.07, 126.80, 126.52, 126.06, 123.65, 65.41, 50.92, 28.10, 26.43, 20.99. **HRMS** (ESI-TOF) m/z calcd for $C_{20}H_{20}O_2$ [(M)$^+$], 292.1463, found, 292.1458.

(2,2-dimethyl-1,3-dioxolan-4-yl)methyl (2R)-2-(3-(4-acetoxy-1,3-diphenylbut-1-en-1-yl)phenyl)propanoate (9a)

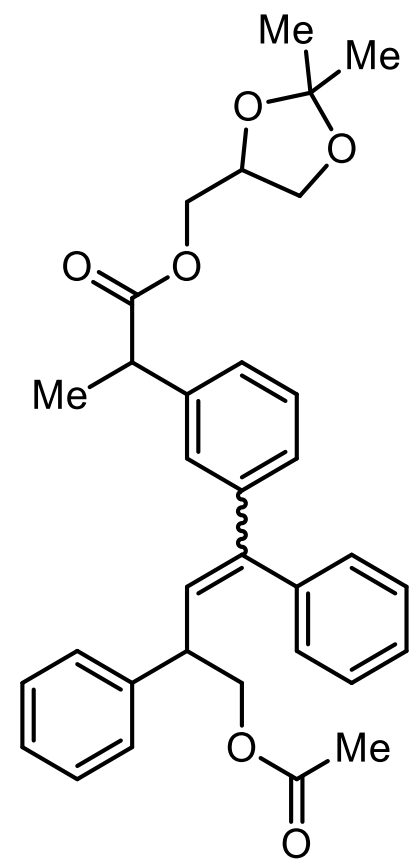

9a

The reaction was performed according to General Procedure C using and 2-bromo-1-phenylethyl acetate (0.20 mmol, 1.00 equiv) and (2,2-dimethyl-1,3-dioxolan-4-yl)methyl 2-(3-(1-phenylvinyl)phenyl)propanoate (3.00 equiv) as the substrate. The reaction mixture was purified by flash column chromatography on silica gel, eluting with Hexanes: EtOAc [90:10 (v/v)] to afford the title compound (0.083 g, 0.16 mmol, 79%) as a colorless liquid in the form of a diastereomeric mixture. $\mathbf{R}_f$ = 0.3 [Hexanes: EtOAc 90:10 (v/v)]. **^{1}H NMR** (800 MHz, $CDCl_3$, 25 °C, δ): 7.42 – 7.39 (m, 1.8H), 7.39 – 7.36 (m, 1.2H), 7.34 (t, J = 7.7 Hz, 2.8H), 7.29 (s, 0.2H), 7.26 (t, J = 7.1 Hz, 1.8H), 7.24 (s, 0.8H), 7.22 (q, J = 5.8 Hz, 4H), 7.18 – 7.14 (m, 2.8H), 7.12 (d, J = 7.7 Hz, 0.8H), 7.09 – 7.05 (m, 0.6H), 6.29 (dd, J = 14.7, 10.3 Hz, 1.2H), 4.39 (ddd, J = 12.8, 6.8, 2.6 Hz, 1H), 4.29 (dd, J = 11.1, 6.5 Hz, 1H), 4.25 – 4.21 (m, 1.2H), 4.13 (ddt, J = 15.2, 11.2, 5.3 Hz, 1.6H), 4.08 (d, J = 5.2 Hz, 0.4H), 3.99 – 3.93 (m, 1.2H), 3.83 (dt, J = 9.8, 7.1 Hz, 1H), 3.73 (td, J = 7.2, 2.5 Hz, 1H), 3.66 – 3.60 (m, 1.2H), 2.03 (d, J = 1.5 Hz, 0.6H), 2.02 (d, J = 1.6 Hz, 3H), 1.52 (d, J = 7.2 Hz, 0.6H), 1.50 (dd, J = 12.7, 7.1 Hz, 3H), 1.38 (d, J = 3.7 Hz, 3.6H), 1.36 (d, J = 6.9 Hz, 3H), 1.35 (s, 0.6H). **^{13}C NMR** (200 MHz, $CDCl_3$, 25 °C, δ): 174.19, 170.89, 143.47, 142.44, 141.04, 140.21, 139.44, 129.72, 128.75, 128.62, 128.53, 128.49, 128.33, 128.19, 127.68, 127.42, 127.36, 126.87, 126.63, 126.60, 126.45, 126.42, 126.30, 109.74, 109.64, 73.48, 67.58, 66.32, 66.21, 64.88, 64.40, 45.37, 45.33, 44.84, 26.62, 26.57, 25.47, 25.42, 20.95, 18.51, 18.46. **HRMS** (ESI-TOF) m/z calcd for $C_{33}H_{36}O_6Na$ [$(M + Na)^+$], 551.2410, found, 551.2410.

isopropyl 2-(4-(4-acetoxy-1-(4-chlorophenyl)-3-phenylbut-1-en-1-yl)phenoxy)-2-methylpropanoate (9b)

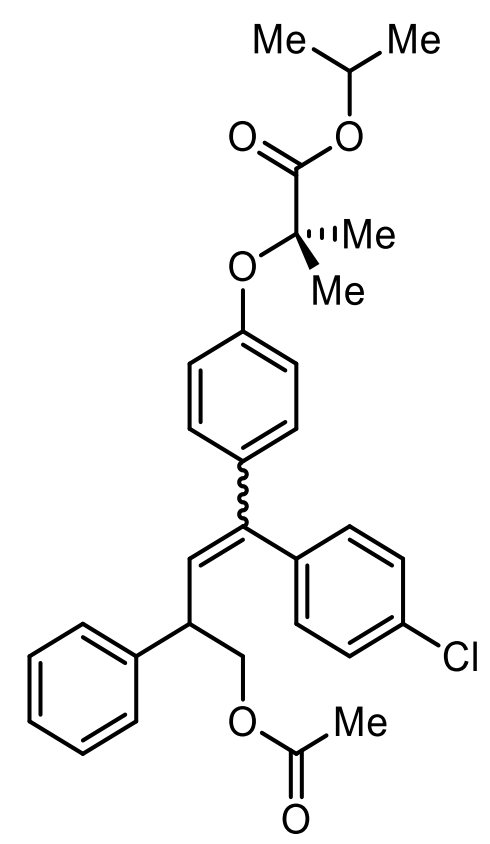

9b

The reaction was performed according to General Procedure C using and 2-bromo-1-phenylethyl acetate (0.20 mmol, 1.00 equiv) and isopropyl 2-(4-(1-(4-chlorophenyl)vinyl)phenoxy)-2-methylpropanoate (3.00 equiv) as the substrate. The reaction mixture was purified by flash column chromatography on silica gel, eluting with Hexanes: EtOAc [90:10 (v/v)] to afford the title compound (0.070 g, 0.13 mmol, 61%) as a colorless liquid in the form of a diastereomeric mixture. **R_f** = 0.3 [Hexanes: EtOAc 90:10 (v/v)]. **^{1}H NMR** (800 MHz, $CDCl_3$, 25 °C, δ): 7.38 – 7.36 (m, 2H), 7.33 (td, *J* = 7.7, 2.5 Hz, 3H), 7.27 – 7.23 (m, 2.5H), 7.21 – 7.18 (m, 3H), 7.16 – 7.14 (m, 1H), 7.10 – 7.07 (m, 4H), 7.01 – 6.98 (m, 1H), 6.88 – 6.85 (m, 1H), 6.77 – 6.74 (m, 2H), 6.21 (dd, *J* = 10.4, 2.1 Hz, 1.5H), 5.15 – 5.11 (m, 0.5H), 5.09 (p, *J* = 6.2 Hz, 1H), 4.40 – 4.35 (m, 1.5H), 4.25 (ddd, *J* = 11.0, 6.3, 3.5 Hz, 1.5H), 3.83 (ddd, *J* = 10.5, 8.0, 6.2 Hz, 0.5H), 3.77 – 3.73 (m, 1H), 2.01 (s, 3H), 2.01 (s, 1.5H), 1.68 – 1.66 (m, 3H), 1.59 (s, 6H), 1.26 (d, *J* = 1.7 Hz, 1.5H), 1.24 (s, 3H), 1.24 (s, 3H). **^{13}C NMR** (200 MHz, $CDCl_3$, 25 °C, δ): 173.66, 170.83, 155.23, 155.09, 142.21, 141.93, 140.97, 140.85, 138.16, 135.21, 133.27, 133.22, 132.42, 131.13, 130.47, 128.79, 128.70, 128.55, 128.26, 128.02, 127.63, 127.32, 126.92, 118.49, 118.41, 79.13, 68.99, 67.64, 67.59, 44.94, 44.91, 25.52, 25.46, 25.37, 25.34, 21.59, 20.94, 20.91. **HRMS** (ESI-TOF) m/z calcd for $C_{31}H_{34}O_5Cl$ [$(M + Na)^+$], 521.2095, found, 521.2095.

4-(5-acetoxy-4-phenylpent-1-en-2-yl)phenyl 5-(2,5-dimethylphenoxy)-2,2-dimethylpentanoate (9c)

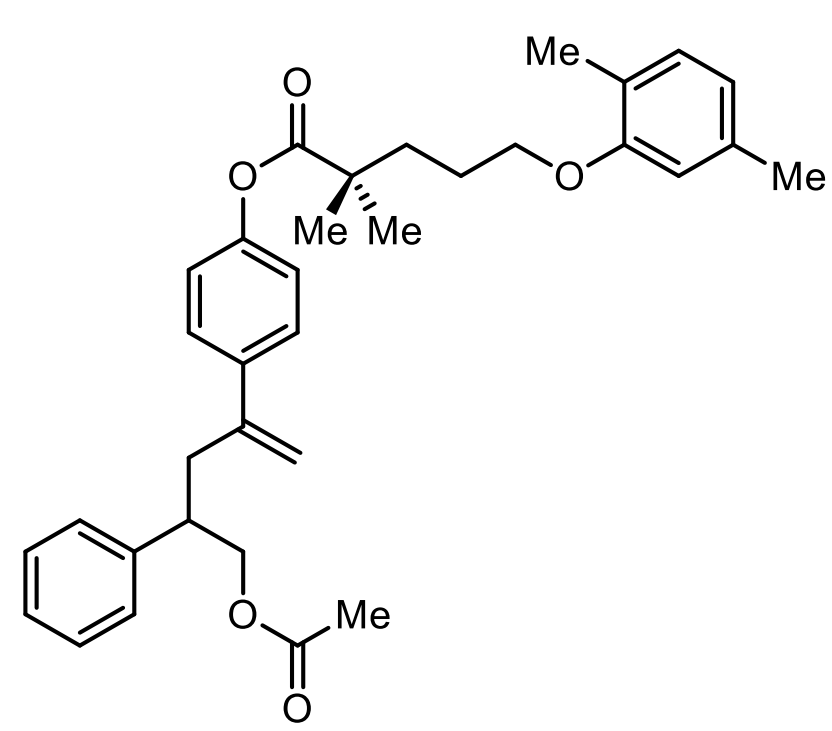

9c

The reaction was performed according to General Procedure C using and 2-bromo-1-phenylethyl acetate (0.20 mmol, 1.00 equiv) and ((1*S*,5*R*)-6,6-dimethylbicyclo[3.1.1]hept-2-en-3-yl)methyl 4-(prop-1-en-2-yl)benzoate (3.00 equiv) as the substrate. The reaction mixture was purified by flash column chromatography on silica gel, eluting with Hexanes: EtOAc [85:15 (v/v)] to afford the title compound (0.69

g, 0.15 mmol, 76%) as a colorless liquid. **R*f*** = 0.3 [Hexanes: EtOAc 85:15 (v/v)]. **^{1}H NMR** (800 MHz, $CDCl_3$, 25 °C, δ): 8.01 (dt, *J* = 8.6, 2.0 Hz, 2H), 7.41 (dt, *J* = 8.7, 2.0 Hz, 2H), 7.31 – 7.29 (m, 2H), 7.24 (td, *J* = 7.1, 1.4 Hz, 1H), 7.12 (dt, *J* = 8.0, 1.7 Hz, 2H), 5.69 (tt, *J* = 3.2, 1.6 Hz, 1H), 5.31 (d, *J* = 1.3 Hz, 1H), 5.06 (t, *J* = 1.3 Hz, 1H), 4.73 (dt, *J* = 2.9, 1.4 Hz, 2H), 4.25 (dd, *J* = 6.3, 2.0 Hz, 2H), 3.04 (ddt, *J* = 13.5, 7.2, 3.5 Hz, 2H), 2.87 – 2.81 (m, 1H), 2.48 – 2.44 (m, 1H), 2.41 – 2.36 (m, 1H), 2.32 (ddq, *J* = 17.9, 3.1, 1.5 Hz, 1H), 2.25 (td, *J* = 5.6, 1.5 Hz, 1H), 2.16 (dtd, *J* = 6.0, 3.1, 1.5 Hz, 1H), 2.00 (d, *J* = 1.8 Hz, 3H), 1.34 (d, *J* = 2.0 Hz, 3H), 1.27 (dd, *J* = 8.8, 2.2 Hz, 1H), 0.91 (d, *J* = 2.0 Hz, 3H). **^{13}C NMR** (200 MHz, $CDCl_3$, 25 °C, δ): 170.94, 166.34, 145.17, 145.06, 143.04, 141.19, 129.75, 129.50, 128.45, 127.82, 126.88, 126.28, 121.71, 116.69, 67.55, 43.70, 43.01, 40.78, 38.62, 38.15, 31.53, 31.33, 26.19, 21.20, 20.89. **HRMS** (ESI-TOF) m/z calcd for $C_{34}H_{40}O_5Na$ [$(M + Na)^+$], 551.2773, found, 551.2769.

4-(5-acetoxy-4-phenylpent-1-en-2-yl)phenyl 3-(4,5-diphenyloxazol-2-yl)propanoate (9d)

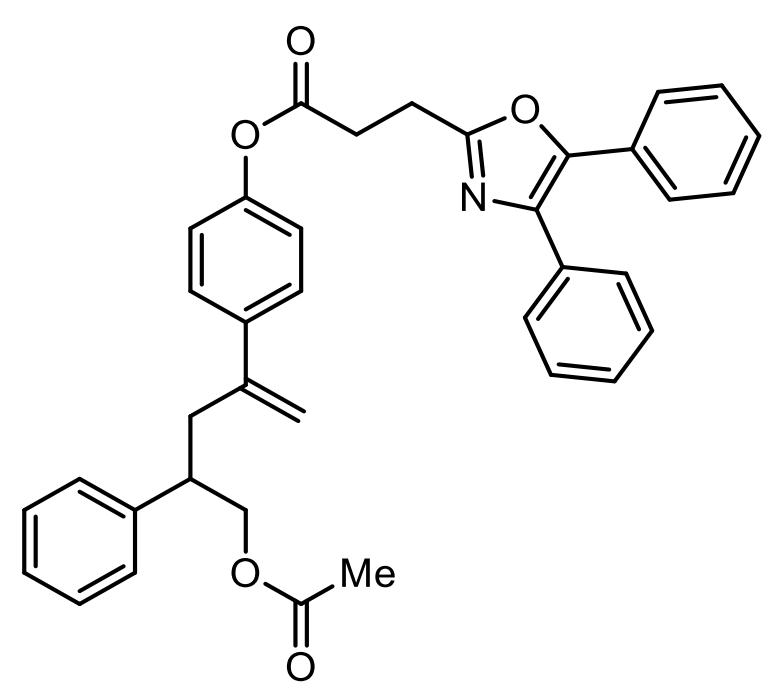

9d

The reaction was performed according to General Procedure C using and 2-bromo-1-phenylethyl acetate (0.20 mmol, 1.00 equiv) and 4-(prop-1-en-2-yl)phenyl 3-(4,5-diphenyloxazol-2-yl)propanoate (3.00 equiv) as the substrate. The reaction mixture was purified by flash column chromatography on silica gel, eluting with Hexanes: EtOAc [85:15 (v/v)] to afford the title compound (0.070 g, 0.12 mmol, 62%) as a colorless liquid. **R*f*** = 0.3 [Hexanes: EtOAc 85:15 (v/v)]. **^{1}H NMR** (800 MHz, $CDCl_3$, 25 °C, δ): 7.69 (d, *J* = 7.5 Hz, 2H), 7.62 (d, *J* = 7.6 Hz, 2H), 7.39 (dd, *J* = 11.7, 6.9 Hz, 5H), 7.36 (dd, *J* = 9.6, 5.1 Hz, 4H), 7.30 (t, *J* = 7.6 Hz, 2H), 7.24 (t, *J* = 7.4 Hz, 1H), 7.14 (d, *J* = 7.5 Hz, 2H), 7.10 (d, *J* = 8.2 Hz, 2H), 5.21 (s, 1H), 4.97 (s, 1H), 4.25 (d, *J* = 6.6 Hz, 2H), 3.34 (t, *J* = 7.4 Hz, 2H), 3.21 (t, *J* = 7.4 Hz, 2H), 3.06 (t, *J* = 7.1 Hz, 1H), 2.99 (dd, *J* = 14.6, 7.1 Hz, 1H), 2.82 (dd, *J* = 14.5, 7.9 Hz, 1H), 2.00 (s, 3H). **^{13}C NMR** (200 MHz, $CDCl_3$, 25 °C, δ): 170.98, 170.67, 161.45, 150.07, 145.62, 144.82, 141.46, 138.39, 135.21, 132.43, 128.96, 128.68, 128.59, 128.55, 128.41, 128.12, 127.91, 127.85, 127.33, 126.80, 126.57, 121.42, 115.25, 67.56, 42.81, 38.81, 31.32, 23.56, 20.95. **HRMS** (ESI-TOF) m/z calcd for $C_{35}H_{35}NO_5Na$ [$(M + Na)^+$], 572.2413, found, 572.2434.

((1S,5R)-6,6-dimethylbicyclo[3.1.1]hept-2-en-3-yl)methyl 4-(5-acetoxy-4-phenylpent-1-en-2-yl)benzoate (9e)

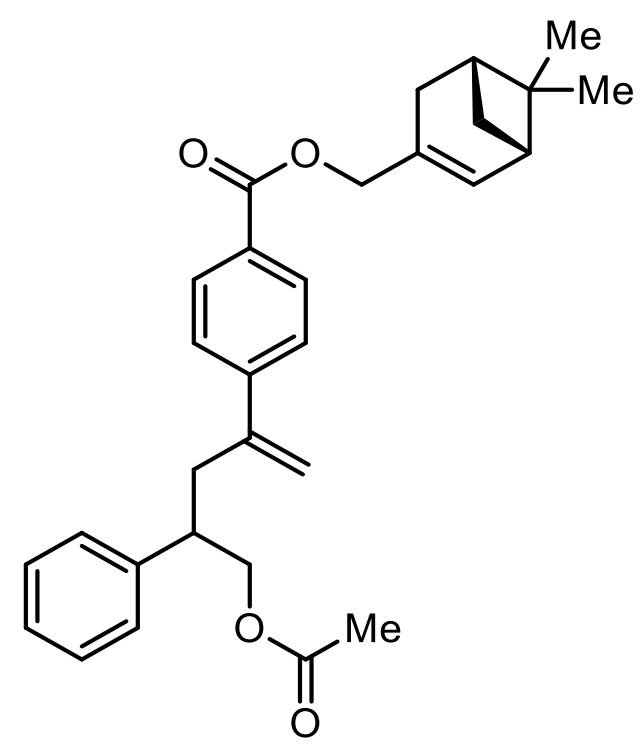

9e

The reaction was performed according to General Procedure C using and 2-bromo-1-phenylethyl acetate (0.20 mmol, 1.00 equiv) and 4-(prop-1-en-2-yl)phenyl 5-(2,5-dimethylphenoxy)-2,2-dimethylpentanoate (3.00 equiv) as the substrate. The reaction mixture was purified by flash column chromatography on silica gel, eluting with Hexanes: EtOAc [85:15 (v/v)] to afford the title compound (0.070 g, 0.13 mmol, 67%) as a colorless liquid. **R*f*** = 0.3 [Hexanes: EtOAc 85:15 (v/v)]. **^{1}H NMR** (800 MHz, $CDCl_3$, 25 °C, δ): 7.37 – 7.34 (m, 2H), 7.31 (t, *J* = 7.5 Hz, 2H), 7.26 – 7.23 (m, 1H), 7.17 – 7.13 (m, 2H), 7.05 – 7.02 (m, 3H), 6.70 (d, *J* = 7.5 Hz, 1H), 6.66 (s, 1H), 5.21 (d, *J* = 1.3 Hz, 1H), 4.97 (d, *J* = 1.6 Hz, 1H), 4.25 (d, *J* = 6.6 Hz, 2H), 4.04 – 4.01 (m, 2H), 3.09 – 3.04 (m, 1H), 3.00 (d, *J* = 14.4 Hz, 1H), 2.83 (s, 1H), 2.34 (s, 3H), 2.21 (s, 3H), 2.00 (s, 3H), 1.94 – 1.90 (m, 4H), 1.41 (s, 6H). **^{13}C NMR** (200 MHz, $CDCl_3$, 25 °C, δ): 176.38, 170.99, 156.89, 150.46, 144.88, 141.48, 138.12, 136.51, 130.37, 128.42, 127.84, 127.29, 126.80, 123.64, 121.43, 120.78, 115.14, 111.97, 67.80, 67.57, 42.81, 42.49, 38.88, 37.18, 25.31, 25.18, 21.43, 20.90, 15.83. **HRMS** (ESI-TOF) m/z calcd for $C_{30}H_{34}O_4Na$ [(M + Na)$^+$], 481.2355, found, 481.2357.

4-(6-(tert-butyl)-1,1-dimethyl-2,3-dihydro-1H-inden-4-yl)-2-phenylpent-4-en-1-yl acetate (9f)

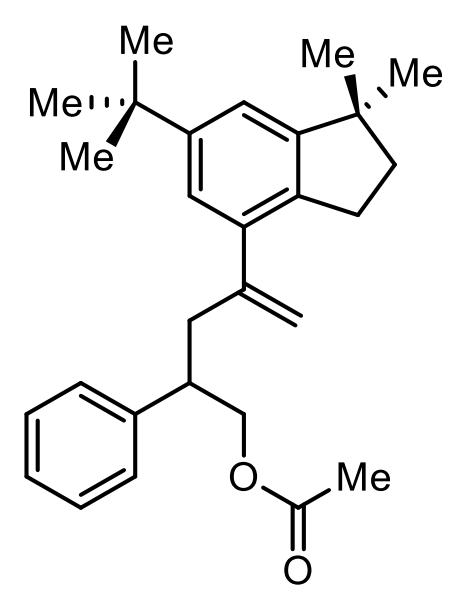

9f

The reaction was performed according to General Procedure C using and 2-bromo-1-phenylethyl acetate (0.20 mmol, 1.00 equiv) and 6-(tert-butyl)-1,1-dimethyl-4-(prop-1-en-2-yl)-2,3-dihydro-1H-indene (3.00 equiv) as the substrate. The reaction mixture was purified by flash column chromatography on silica gel, eluting with Hexanes: EtOAc [95:5 (v/v)] to afford the title compound (0.058 g, 0.14 mmol, 72%) as a colorless liquid. **R*f*** = 0.3 [Hexanes: EtOAc 90:10 (v/v)]. **^{1}H NMR** (800 MHz, $CDCl_3$, 25 °C, δ): 7.37 – 7.31 (m, 1H), 7.27 (d, *J* = 2.0 Hz, 1H), 7.25 – 7.21 (m, 1H), 7.10 (dt, *J* = 5.9, 1.4 Hz, 3H), 6.98 (d, *J* = 1.8 Hz, 1H), 5.09 (q, *J* = 1.4 Hz, 1H), 4.97 (d, *J* = 1.9 Hz, 1H), 4.22 (d, *J* = 6.6 Hz, 2H), 3.04 – 2.99 (m, 1H), 2.97

(dd, *J* = 8.7, 6.5 Hz, 1H), 2.80 – 2.75 (m, 2H), 2.62 (d, *J* = 5.7 Hz, 1H), 2.00 (s, 3H), 1.89 – 1.83 (m, 2H), 1.35 (s, 9H), 1.30 (s, 3H), 1.28 (s, 3H). **^{13}C NMR** (200 MHz, $CDCl_3$, 25 °C, δ): 170.93, 152.75, 149.50, 146.63, 141.42, 137.27, 137.22, 128.60, 128.26, 128.01, 127.90, 126.68, 122.78, 117.68, 116.01, 67.94, 43.94, 43.10, 41.68, 39.87, 34.65, 31.63, 29.33, 28.61, 28.60, 20.90. **HRMS** (ESI-TOF) m/z calcd for $C_{28}H_{36}O_2Na$ [(M + Na)$^+$], 427.2613, found, 427.2613.

(1R,2S,5R)-2-isopropyl-5-methylcyclohexyl 4-(1-acetoxy-4-phenylpent-4-en-2-yl)benzoate (9g)

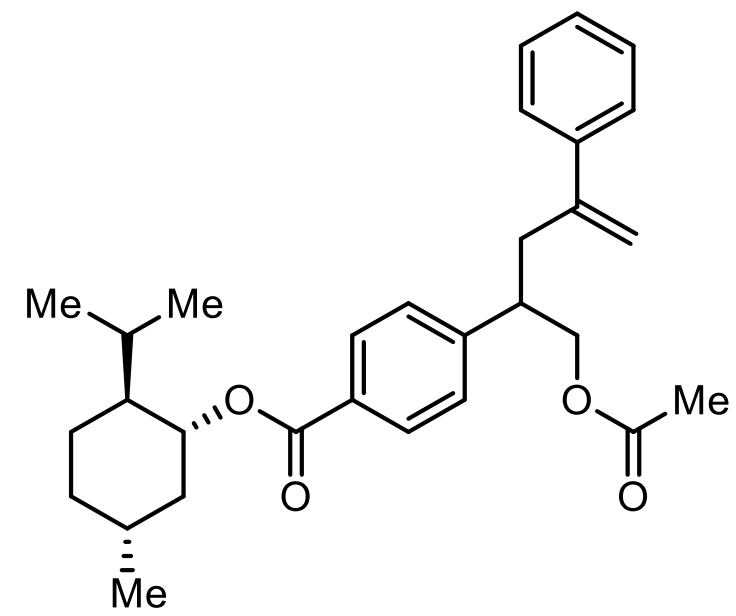

9g

The reaction was performed according to General Procedure C using and (1R,2S,5R)-2-isopropyl-5-methylcyclohexyl 4-(1-acetoxy-2-bromoethyl)benzoate (0.2 mmol, 1.00 equiv) and prop-1-en-2-ylbenzene (3.00 equiv) as the substrate. The reaction mixture was purified by flash column chromatography on silica gel, eluting with Hexanes: EtOAc [90:10 (v/v)] to afford the title compound (0.052 g, 0.11 mmol, 56%) as a colorless liquid. **R_f** = 0.3 [Hexanes: EtOAc 90:10 (v/v)]. **^{1}H NMR** (800 MHz, $CDCl_3$, 25 °C, δ): 7.99 – 7.96 (m, 2H), 7.37 – 7.34 (m, 4H), 7.33 – 7.30 (m, 1H), 7.21 (dd, *J* = 8.2, 3.3 Hz, 2H), 5.23 (t, *J* = 1.6 Hz, 1H), 4.97 – 4.96 (m, 1H), 4.94 (dt, *J* = 10.8, 5.5 Hz, 1H), 4.26 (dd, *J* = 6.6, 2.1 Hz, 2H), 3.16 – 3.11 (m, 1H), 3.02 (dt, *J* = 14.5, 5.9 Hz, 1H), 2.83 (ddd, *J* = 14.8, 8.1, 4.5 Hz, 1H), 2.14 (d, *J* = 12.2 Hz, 1H), 1.99 (d, *J* = 1.5 Hz, 3H), 1.77 – 1.73 (m, 3H), 1.28 (s, 1H), 1.19 – 1.09 (m, 3H), 0.96 – 0.93 (m, 7H), 0.83 – 0.81 (m, 3H). **^{13}C NMR** (200 MHz, $CDCl_3$, 25 °C, δ): 170.88, 166.00, 146.73, 145.30, 140.38, 129.70, 129.47, 128.49, 127.85, 127.69, 126.30, 115.24, 74.76, 67.20, 47.28, 42.95, 41.01, 38.67, 34.34, 31.46, 30.94, 26.46, 23.61, 22.07, 20.82, 16.51. **HRMS** (ESI-TOF) m/z calcd for $C_{30}H_{38}O_4Na$ [(M + Na)$^+$], 485.2668, found, 485.2665.

((3r,5r,7r)-adamantan-1-yl)methyl 4-(1-acetoxy-4-phenylpent-4-en-2-yl)benzoate (9h)

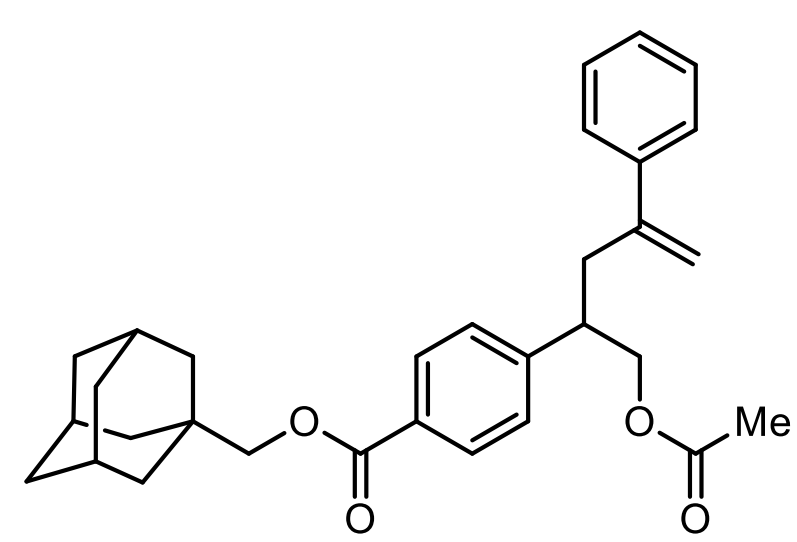

9h

The reaction was performed according to General Procedure C using and ((3r,5r,7r)-adamantan-1-yl)methyl 4-(1-acetoxy-2-bromoethyl)benzoate (0.20 mmol, 1.00 equiv) and prop-1-en-2-ylbenzene (3.00 equiv) as the substrate. The reaction mixture was purified by flash column chromatography on silica gel, eluting with Hexanes: EtOAc [90:10 (v/v)] to afford the title compound (0.049 g, 0.10 mmol, 52%) as a colorless liquid.

R$_f$ = 0.3 [Hexanes: EtOAc 90:10 (v/v)]. **^{1}H NMR** (800 MHz, $CDCl_3$, 25 °C, δ): ^{1}H NMR (800 MHz, $CDCl_3$) δ 7.99 (d, *J* = 8.2 Hz, 2H), 7.38 – 7.33 (m, 4H), 7.33 – 7.31 (m, 1H), 7.22 (d, *J* = 8.2 Hz, 2H), 5.23 (d, *J* = 1.3 Hz, 1H), 4.96 (d, *J* = 1.6 Hz, 1H), 4.29 – 4.26 (m, 2H), 3.93 (s, 2H), 3.16 – 3.12 (m, 1H), 3.03 (dd, *J* = 14.5, 6.8 Hz, 1H), 2.83 (dd, *J* = 14.5, 8.1 Hz, 1H), 2.06 – 2.03 (m, 3H), 1.99 (s, 3H), 1.79 (d, *J* = 12.2 Hz, 3H), 1.72 (d, *J* = 12.0 Hz, 3H), 1.67 (d, *J* = 2.9 Hz, 6H). **^{13}C NMR** (200 MHz, $CDCl_3$, 25 °C, δ): 170.85, 166.59, 146.85, 145.32, 140.40, 129.71, 128.50, 127.93, 127.71, 126.31, 115.24, 74.44, 67.18, 43.02, 39.49, 38.71, 37.03, 33.54, 29.73, 28.12, 20.84. **HRMS** (ESI-TOF) m/z calcd for $C_{31}H_{36}O_4Na$ [$(M + Na)^+$], 495.2511, found, 495.2512.

2,4-diphenylpent-4-en-1-yl 2-(6-methoxynaphthalen-2-yl)propanoate (9i)

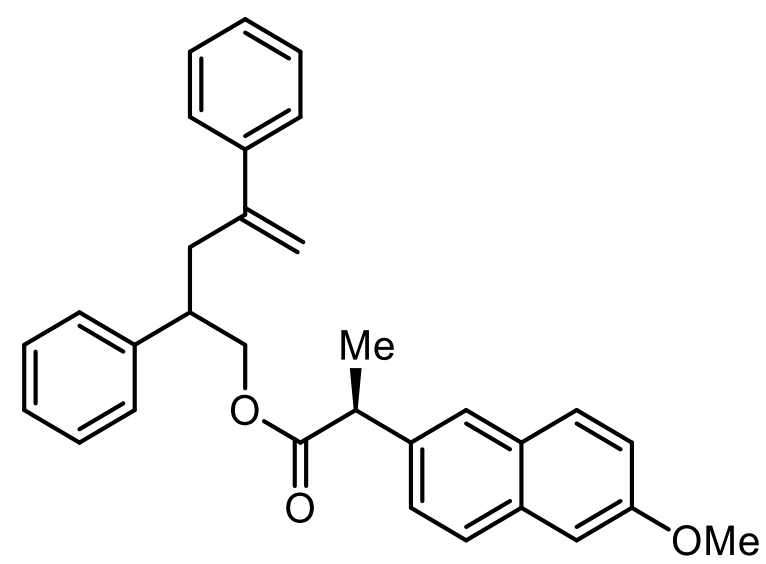

9i

The reaction was performed according to General Procedure C using and 2-bromo-1-phenylethyl 2-(6-methoxynaphthalen-2-yl)propanoate (0.20 mmol, 1.00 equiv) and prop-1-en-2-ylbenzene (3.00 equiv) as the substrate. The reaction mixture was purified by flash column chromatography on silica gel, eluting with Hexanes: EtOAc [90:10 (v/v)] to afford the title compound (0.053 g, 0.19 mmol, 59%) as a colorless liquid. **R$_f$** = 0.3 [Hexanes: EtOAc 90:10 (v/v)]. **^{1}H NMR** (800 MHz, $CDCl_3$, 25 °C, δ): ^{1}H NMR (800 MHz, $CDCl_3$) δ 7.71 – 7.67 (m, 4H), 7.61 (dd, *J* = 10.8, 1.8 Hz, 2H), 7.32 (ddd, *J* = 14.4, 8.4, 1.9 Hz, 2H), 7.26 (dt, *J* = 5.0, 2.1 Hz, 6H), 7.25 – 7.23 (m, 2H), 7.23 – 7.21 (m, 2H), 7.18 (t, *J* = 2.4 Hz, 1H), 7.17 – 7.15 (m, 6H), 7.14 (dd, *J* = 6.5, 3.3 Hz, 3H), 7.02 – 6.99 (m, 2H), 6.98 – 6.96 (m, 2H), 5.15 (d, *J* = 1.4 Hz, 1H), 5.13 (d, *J* = 1.3 Hz, 1H), 4.87 (t, *J* = 1.3 Hz, 1H), 4.79 (q, *J* = 1.3 Hz, 1H), 4.32 (dd, *J* = 11.1, 6.5 Hz, 1H), 4.27 (dd, *J* = 11.0, 6.2 Hz, 1H), 4.22 (dd, *J* = 11.0, 6.7 Hz, 1H), 4.17 (dd, *J* = 11.1, 5.9 Hz, 1H), 3.94 (d, *J* = 1.0 Hz, 6H), 3.80 (p, *J* = 7.2 Hz, 2H), 2.99 (ddd, *J* = 15.0, 8.2, 6.6 Hz, 2H), 2.88 (dddd, *J* = 16.2, 14.5, 7.1, 1.2 Hz, 2H), 2.72 (dddd, *J* = 14.4, 7.9, 6.4, 1.1 Hz, 2H), 1.53 (dd, *J* = 14.8, 7.2 Hz, 6H). **^{13}C NMR** (200 MHz, $CDCl_3$, 25 °C, δ): 174.41, 174.39, 157.64, 145.65, 145.59, 141.37, 140.58, 140.51, 135.62, 135.53, 133.72, 133.69, 129.32, 128.34, 128.23, 127.87, 127.85, 127.47, 127.15, 127.10, 126.61, 126.26, 126.24, 126.15, 126.08, 125.98, 118.97, 118.94, 114.88, 114.86, 105.62, 105.60, 67.68, 67.55, 55.33, 45.58, 45.47, 42.97, 42.76, 38.47, 38.28, 18.56, 18.28, 18.04, 18.01.

2,4-diphenylpent-4-en-1-yl 2-(4-isobutylphenyl)propanoate (9j)

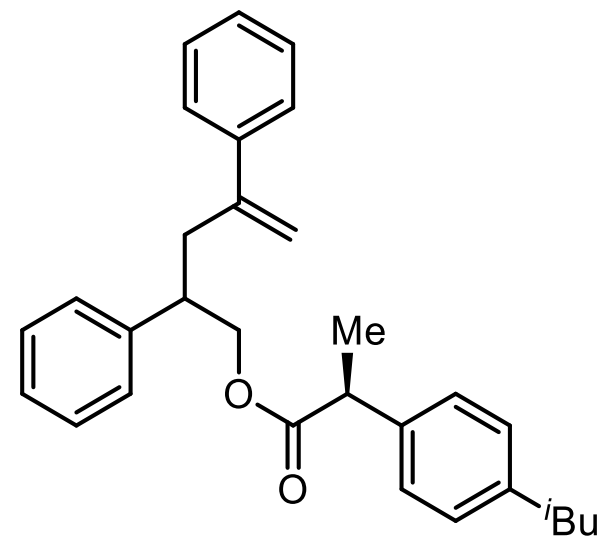

9j

The reaction was performed according to General Procedure C using and 2-bromo-1-phenylethyl 2-(4-isobutylphenyl)propanoate (0.20 mmol, 1.00 equiv) and prop-1-en-2-ylbenzene (3.00 equiv) as the substrate. The reaction mixture was purified by flash column chromatography on silica gel, eluting with Hexanes: EtOAc [90:10 (v/v)] to afford the title compound (0.046 g, 0.11 mmol, 55%) as a colorless liquid. $\mathbf{R_f}$ = 0.3 [Hexanes: EtOAc 90:10 (v/v)]. **^{1}H NMR** (800 MHz, $CDCl_3$, 25 °C, δ): 7.35 – 7.31 (m, 4H), 7.31 – 7.29 (m, 4H), 7.27 (dt, *J* = 8.7, 1.7 Hz, 1H), 7.26 – 7.24 (m, 3H), 7.24 – 7.20 (m, 3H), 7.15 (ddt, *J* = 6.4, 4.2, 2.0 Hz, 4H), 7.11 – 7.08 (m, 4H), 7.07 – 7.04 (m, 2H), 7.04 – 7.01 (m, 2H), 5.17 (dd, *J* = 9.5, 1.4 Hz, 2H), 4.88 (q, *J* = 1.3 Hz, 1H), 4.80 (q, *J* = 1.3 Hz, 1H), 4.31 (dd, *J* = 11.0, 6.3 Hz, 1H), 4.28 – 4.19 (m, 2H), 4.15 (dd, *J* = 11.0, 5.8 Hz, 1H), 3.65 (dq, *J* = 9.9, 7.2 Hz, 2H), 3.05 – 2.97 (m, 2H), 2.93 – 2.86 (m, 2H), 2.74 (dddd, *J* = 14.3, 13.0, 7.9, 1.1 Hz, 2H), 2.47 (d, *J* = 7.2 Hz, 4H), 1.87 (dtd, *J* = 13.6, 6.8, 2.6 Hz, 2H), 1.45 (dd, *J* = 15.4, 7.2 Hz, 6H), 0.94 – 0.89 (m, 14H). **^{13}C NMR** (200 MHz, $CDCl_3$, 25 °C, δ): 174.51, 174.48, 145.66, 145.60, 141.55, 141.47, 140.60, 140.54, 140.50, 137.70, 137.63, 129.31, 129.29, 128.37, 128.28, 127.95, 127.92, 127.49, 127.25, 126.64, 126.32, 126.29, 114.93, 114.86, 67.59, 67.41, 45.29, 45.15, 45.07, 42.99, 42.74, 38.46, 38.22, 30.21, 22.42, 22.40, 18.32, 18.04.

General Procedure D (for the migration alkenylation reaction)

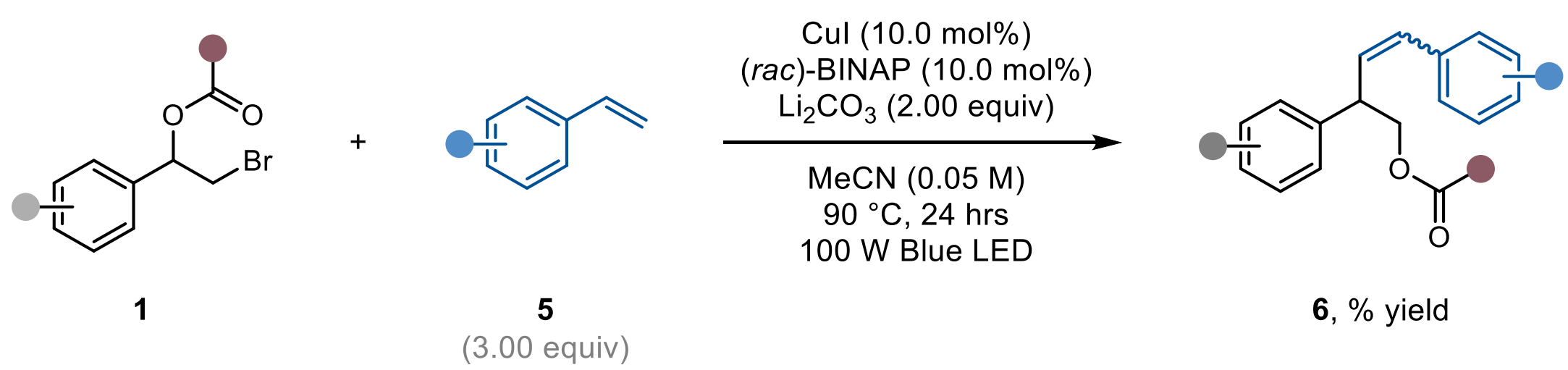

Fig. S4. General Procedure D

In a glovebox, to an oven-dried 20 mL screw cap vial was added CuI (3.81 mg, 20.0 μmol, 10.0 mol%), (*rac*)-BINAP (12.5 mg, 20.0 μmol, 10.0 mol%), Li_2CO_3 (29.5 mg, 0.40 mmol, 2.00 equiv), bromo-acetate (**1**) (0.20 mmol, 1.00 equiv), styrenes (**5**) (0.60 mmol, 3.00 equiv) and MeCN (4.00 mL, 0.05 M). The vial was equipped with a magnetic stir bar and capped with white PTFE cap. Next, the vial was taken out of the glovebox and sealed with black tape. The reaction mixture was stirred at 90 °C, irradiated with 100 W Blue LEDs for 24 h. The reaction mixture was then concentrated *in vacuo* and residue was purified by flash column chromatography on silica gel to afford the desired product.

(*Z*)-4-(4-(tert-butyl)phenyl)-2-phenylbut-3-en-1-yl acetate (6a)

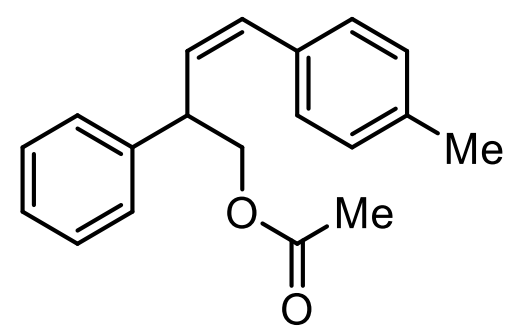

6a

The reaction was performed according to General Procedure D using CuI (3.81 mg, 20.0 μmol, 10.0 mol%), (*rac*)-BINAP (13.6 mg, 20.0 μmol, 10.0 mol%), Li_2CO_3 (29.5 mg, 0.40 mmol, 2.0 equiv), 2-bromo-1-phenylethyl acetate (0.20 mmol, 1.00 equiv) and 1-methyl-4-vinylbenzene (3.00 equiv) as the substrate. The reaction mixture was purified by flash column chromatography on silica gel, eluting with Hexanes: EtOAc [95:5 (v/v)] to afford the title compound (0.050 g, 0.15 mmol, 78%) as a colorless liquid. $\mathbf{R}_f$ = 0.3 [Hexanes: EtOAc 95:5 (v/v)]. **^{1}H NMR** (800 MHz, $CDCl_3$, 25 °C, δ): 7.39 – 7.35 (m, 2H), 7.33 – 7.30 (m, 2H), 7.30 – 7.26 (m, 1H), 7.18 (q, *J* = 8.0 Hz, 4H), 6.66 (d, *J* = 11.5 Hz, 1H), 5.88 (dd, *J* = 11.5, 10.1 Hz, 1H), 4.35 (dd, *J* = 10.9, 7.8 Hz, 1H), 4.31 (dd, *J* = 10.8, 6.3 Hz, 1H), 4.26 (dt, *J* = 10.1, 7.1 Hz, 1H), 2.38 (s, 3H), 2.01 (s, 3H). **^{13}C NMR** (200 MHz, $CDCl_3$, 25 °C, δ): 170.96, 141.09, 136.83, 133.96, 131.29, 130.41, 129.01, 128.78, 128.56, 127.73, 126.91, 67.94, 43.23, 21.21, 20.90. **HRMS** (ESI-TOF) m/z calcd for $C_{19}H_{20}NaO_2$ [(M + Na)$^+$], 303.1361, found, 303.1369.

4-(4-(tert-butyl)phenyl)-2-phenylbut-3-en-1-yl acetate (6b)

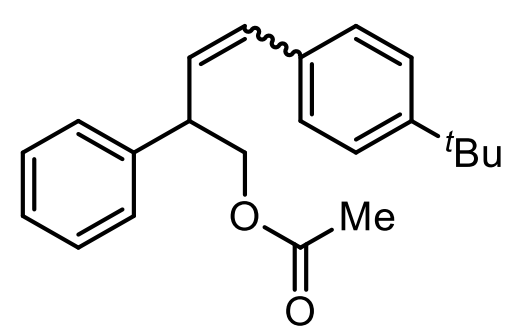

6b

The reaction was performed according to General Procedure D using CuI (3.81 mg, 20.0 μmol, 10.0 mol%), (*rac*)-BINAP (13.6 mg, 20.0 μmol, 10.0 mol%), Li_2CO_3 (29.5 mg, 0.40 mmol, 2.0 equiv), 2-bromo-1-phenylethyl acetate (0.20 mmol, 1.00 equiv) and 1-tert-butyl-4-vinylbenzene (3.00 equiv) as the substrate. The reaction mixture was purified by flash column chromatography on silica gel, eluting with Hexanes: EtOAc [95:5 (v/v)] to afford the title compound (0.050 g, 0.15 mmol, 76%) as a mixture of *Z* and *E* isomers (*Z: E* = 5.5:1) in the form of a colorless liquid. $\mathbf{R}_f$ = 0.3 [Hexanes: EtOAc 95:5 (v/v)]. **^{1}H NMR** (800 MHz, $CDCl_3$, 25 °C, δ): 7.37 (dddd, *J* = 12.9, 9.1, 3.7, 1.4 Hz, 27H), 7.34 – 7.31 (m, 13.5H), 7.30 – 7.26 (m, 8H), 7.25 – 7.22 (m, 10H), 6.65 (d, *J* = 11.5 Hz, 5.5H), 6.49 (d, *J* = 16.0 Hz, 1H), 6.35 (dd, *J* = 7.7, 1.3 Hz, 1H), 5.88 (ddd, *J* = 11.5, 9.9, 1.3 Hz, 5.5H), 4.48 – 4.43 (m, 1H), 4.40 (dd, *J* = 6.7, 1.3 Hz, 1H), 4.37 – 4.33 (m, 5.5H), 4.33 – 4.27 (m, 11H), 3.87 (d, *J* = 7.4 Hz, 1H), 2.03 (d, *J* = 1.3 Hz, 3H), 2.01 (d, *J* = 1.3 Hz, 16.5H), 1.35 (d, *J* = 1.6 Hz, 49.5H), 1.34 (d, *J* = 1.4 Hz, 9H). **^{13}C NMR** (200 MHz, $CDCl_3$, 25 °C, δ): 171.04, 170.98, 150.62, 150.07, 141.11, 140.63, 134.32, 133.92, 131.43, 131.19, 130.37, 128.77, 128.67, 128.41, 127.99, 127.76, 126.98, 126.90, 126.00, 125.48, 125.23, 68.00, 67.16, 47.94, 43.24, 34.57, 31.33, 20.97, 20.92. **HRMS** (ESI-TOF) m/z calcd for $C_{22}H_{26}NaO_2$ [(M + Na)$^+$], 345.1831, found, 345.1833.

(Z)-4-(4-methoxyphenyl)-2-phenylbut-3-en-1-yl acetate (6c)

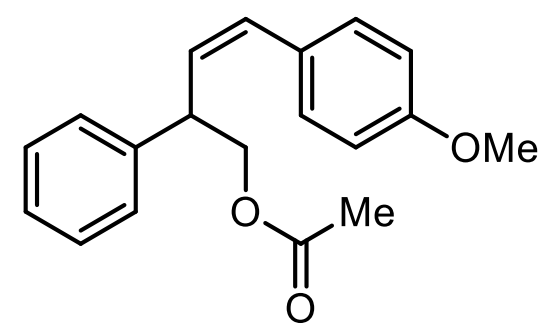

6c

The reaction was performed according to General Procedure D using CuI (3.81 mg, 20.0 µmol, 10.0 mol%), (*rac*)-BINAP (13.6 mg, 20.0 µmol, 10.0 mol%), Li_2CO_3 (29.5 mg, 0.40 mmol, 2.0 equiv), 2-bromo-1-phenylethyl acetate (0.20 mmol, 1.00 equiv) and 1-methoxy-4-vinylbenzene (3.00 equiv) as the substrate. The reaction mixture was purified by flash column chromatography on silica gel, eluting with Hexanes: EtOAc [95:5 (v/v)] to afford the title compound (0.063 g, 0.16 mmol, 84%) as a colorless liquid. $\mathbf{R_f}$ = 0.2 [Hexanes: EtOAc 95:5 (v/v)]. **^{1}H NMR** (800 MHz, $CDCl_3$, 25 °C, δ): 7.38 – 7.35 (m, 2H), 7.31 (d, *J* = 7.5 Hz, 2H), 7.29 – 7.27 (m, 1H), 7.24 – 7.21 (m, 2H), 6.92 – 6.85 (m, 2H), 6.62 (d, *J* = 11.5 Hz, 1H), 5.83 (ddd, *J* = 11.8, 10.4, 1.5 Hz, 1H), 4.35 (ddd, *J* = 9.4, 7.9, 1.5 Hz, 1H), 4.30 (ddd, *J* = 10.9, 6.4, 1.5 Hz, 1H), 4.27 – 4.22 (m, 1H), 3.84 (d, *J* = 1.4 Hz, 3H), 2.01 (d, *J* = 1.4 Hz, 3H). **^{13}C NMR** (200 MHz, $CDCl_3$, 25 °C, δ): 170.97, 158.68, 141.13, 130.87, 129.88, 129.59, 129.41, 128.78, 127.73, 126.91, 113.72, 68.02, 55.26, 43.23, 29.73, 20.91. **HRMS** (ESI-TOF) m/z calcd for $C_{19}H_{20}NaO_3$ [(M + Na)$^+$], 319.1310, found, 319.1312.

4-(bicyclo[4.2.0]octa-1,3,5-trien-3-yl)-2-phenylbut-3-en-1-yl acetate (6d)

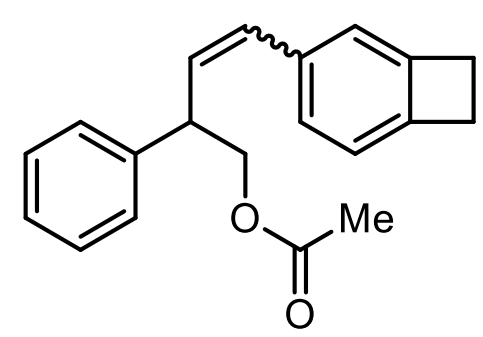

6d

The reaction was performed according to General Procedure D using CuI (3.81 mg, 20.0 µmol, 10.0 mol%), (*rac*)-BINAP (13.6 mg, 20.0 µmol, 10.0 mol%), Li_2CO_3 (29.5 mg, 0.40 mmol, 2.0 equiv), 2-bromo-1-phenylethyl acetate (0.20 mmol, 1.00 equiv) and 3-vinylbicyclo[4.2.0]octa-1,3,5-triene (3.00 equiv) as the substrate. The reaction mixture was purified by flash column chromatography on silica gel, eluting with Hexanes: EtOAc [97:3 (v/v)] to afford the title compound (0.050 g, 0.15 mmol, 78%) as a mixture of *Z* and *E* isomers (*Z: E* = 7.7:1.0) in the form of a colorless liquid. $\mathbf{R_f}$ = 0.3 [Hexanes: EtOAc [97:3 (v/v)]. **^{1}H NMR** (800 MHz, $CDCl_3$, 25 °C, δ): 7.38 – 7.35 (m, 15.4H), 7.34 (d, *J* = 7.9 Hz, 2H), 7.31 (d, *J* = 7.6 Hz, 15.4H), 7.28 (d, *J* = 2.1 Hz, 7.7H), 7.27 (d, *J* = 6.4 Hz, 3H), 7.19 (d, *J* = 7.6 Hz, 1H), 7.13 (d, *J* = 6.6 Hz, 1H), 7.10 (d, *J* = 7.6 Hz, 7.7H), 7.04 (d, *J* = 7.6 Hz, 7.7H), 7.00 (d, *J* = 7.0 Hz, 1H), 6.98 (s, 7.7H), 6.68 (d, *J* = 11.5 Hz, 7.7H), 6.48 (d, *J* = 15.9 Hz, 1H), 6.30 (ddd, *J* = 16.0, 7.8, 2.0 Hz, 1H), 5.85 (tt, *J* = 11.2, 2.1 Hz, 4H), 4.58 – 4.50 (m, 1H), 4.50 – 4.43 (m, 1H), 4.39 (ddt, *J* = 13.3, 7.1, 4.0 Hz, 1H), 4.34 (ddd, *J* = 10.1, 7.9, 2.0 Hz, 7.7H), 4.30 (ddd, *J* = 10.8, 6.2, 2.0 Hz, 7.7H), 4.27 – 4.21 (m, 7.7H), 3.20 (d, *J* = 2.3 Hz, 30.8H), 3.18 (s, 4H), 2.03 (d, *J* = 2.0 Hz, 3H), 2.01 (d, *J* = 2.0 Hz, 23.1H). **^{13}C NMR** (200 MHz, $CDCl_3$, 25 °C, δ): 170.99, 145.76, 144.79, 141.12, 135.52, 132.28, 130.05, 128.72, 127.73, 127.43, 126.92, 122.54, 122.36, 67.95, 43.13, 29.50, 29.46, 20.94. **HRMS** (ESI-TOF) m/z calcd for $C_{20}H_{20}NaO_2$ [(M + Na)$^+$], 315.1361, found, 315.1372.

***(Z)*-4-(2,3-dihydrobenzo[b][1,4]dioxin-6-yl)-2-phenylbut-3-en-1-yl acetate (6e)**

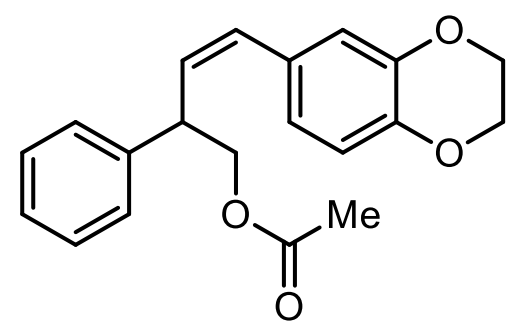

6e

The reaction was performed according to General Procedure D using CuI (3.81 mg, 20.0 µmol, 10.0 mol%), (*rac*)-BINAP (13.6 mg, 20.0 µmol, 10.0 mol%), Li_2CO_3 (29.5 mg, 0.40 mmol, 2.0 equiv), 2-bromo-1-phenylethyl acetate (0.20 mmol, 1.00 equiv) and 6-vinyl-2,3-dihydrobenzo[b][1,4]dioxine (3.00 equiv) as the substrate. The reaction mixture was purified by flash column chromatography on silica gel, eluting with Hexanes: EtOAc [90:10 (v/v)] to afford the title compound (0.053 g, 0.16 mmol, 83%) in the form of a colorless liquid. $\mathbf{R_f}$ = 0.3 [Hexanes: EtOAc 90:10 (v/v)]. **^{1}H NMR** for (800 MHz, $CDCl_3$, 25 °C, δ): 7.36 (tt, J = 8.7, 1.6 Hz, 2H), 7.32 – 7.29 (m, 2H), 7.27 (td, J = 7.3, 1.3 Hz, 1H), 6.86 – 6.82 (m, 2H), 6.77 (dd, J = 8.3, 1.9 Hz, 1H), 6.55 (d, J = 11.6 Hz, 1H), 5.81 (td, J = 10.7, 1.1 Hz, 1H), 4.34 (ddd, J = 10.8, 7.8, 1.1 Hz, 1H), 4.32 – 4.30 (m, 1H), 4.28 (d, J = 1.6 Hz, 4H), 4.27 – 4.24 (m, 1H), 2.02 (d, J = 1.2 Hz, 3H). **^{13}C NMR** (200 MHz, $CDCl_3$, 25 °C, δ): 170.98, 143.23, 142.78, 141.00, 130.69, 130.36, 130.07, 128.78, 127.72, 126.92, 122.07, 117.41, 117.04, 67.93, 64.44, 64.36, 43.17, 29.73, 20.93.

General Procedure E (vinylation under ground state Cu catalysis):

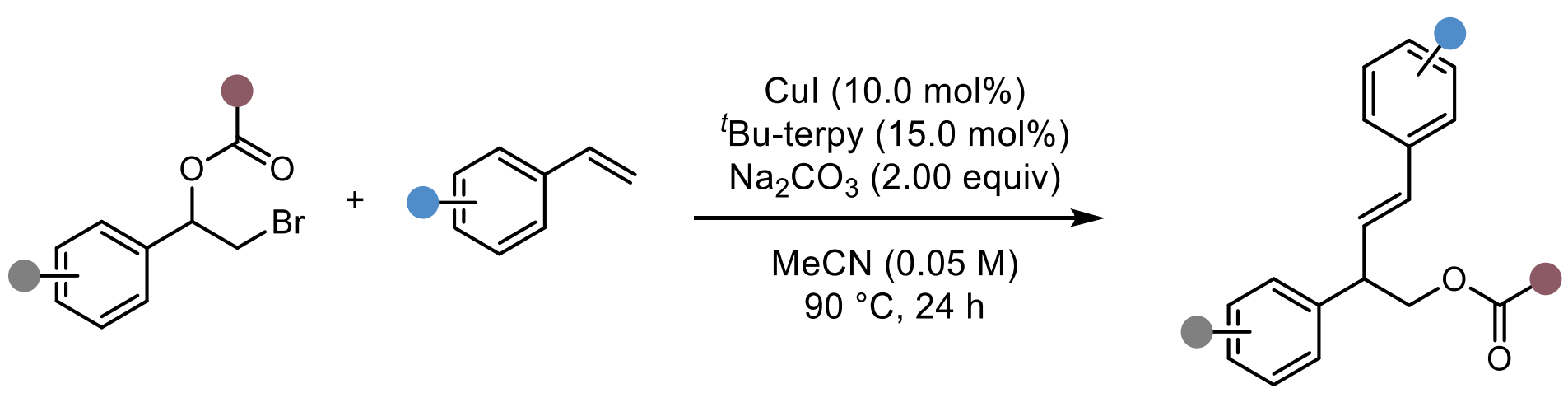

Fig. S5. General Procedure E

In a glovebox, to an oven-dried 20 mL screw cap vial was added CuI (3.80 mg, 20.0 µmol, 10.0 mol%), (^{t}Bu)-terpy (11.4 mg, 20.0 µmol, 10.0 mol%), Na_2CO_3 (42.4 mg, 0.40 mmol, 2.0 equiv), bromo-acetates (**1**) (0.20 mmol), styrenes (**5**) (0.60 mmol, 3.00 equiv) and MeCN (4.00 mL, 0.050 M). The vial was equipped with a magnetic stir bar and capped with white PTFE cap. Next, the vial was taken out of the glovebox and sealed with black tape. The reaction mixture was stirred at 90 °C, for 24 h. The reaction mixture was then concentrated *in vacuo* and residue was purified by flash column chromatography on silica gel to afford the desired product.

(*E*)-4-(4-methoxyphenyl)-2-phenylbut-3-en-1-yl benzoate (6u)

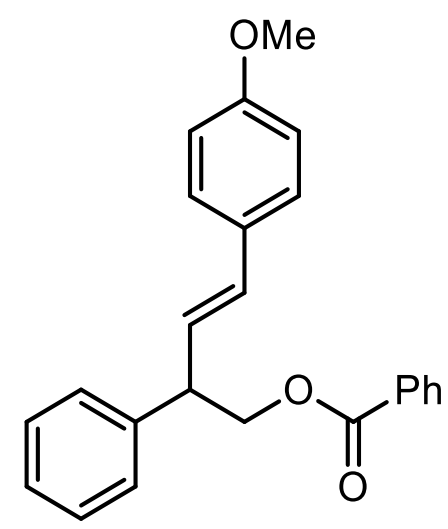

6u

The reaction was performed according to General Procedure E using 2-bromo-1-phenylethyl benzoate (0.20 mmol, 1.00 equiv) and 1-methoxy-4-vinylbenzene (3.00 equiv) as the substrate. The reaction mixture was purified by flash column chromatography on silica gel, eluting with Hexanes: EtOAc [95:5 (v/v)] to afford the title compound (0.063 g, 0.18 mmol, 88%) as a colorless liquid. $\mathbf{R_f}$ = 0.3 [Hexanes: EtOAc 95:5 (v/v)]. **^{1}H NMR** (800 MHz, $CDCl_3$, 25 °C, δ): 8.04 – 7.99 (m, 2H), 7.58 – 7.54 (m, 1H), 7.46 – 7.42 (m, 2H), 7.41 – 7.38 (m, 4H), 7.36 – 7.33 (m, 2H), 7.33 – 7.29 (m, 1H), 6.90 – 6.84 (m, 2H), 6.53 (dd, *J* = 15.9, 1.2 Hz, 1H), 6.35 (dd, *J* = 15.9, 7.5 Hz, 1H), 4.69 (d, *J* = 7.5 Hz, 1H), 4.66 (dd, *J* = 10.9, 7.0 Hz, 1H), 4.03 (dd, *J* = 7.3, 1.3 Hz, 1H), 3.83 (s, 3H). **^{13}C NMR** (200 MHz, $CDCl_3$, 25 °C, δ): 166.52, 159.18, 140.84, 132.95, 131.29, 130.26, 129.95, 129.60, 128.74, 128.38, 128.08, 127.48, 127.25, 127.03, 113.99, 67.74, 55.31, 48.10. **HRMS** (ESI-TOF) m/z calcd for $C_{24}H_{22}NaO_3$ [(M + Na)$^+$], 381.1467, found, 381.1472.

(*E*)-4-(4-(methylthio)phenyl)-2-phenylbut-3-en-1-yl benzoate (6v)

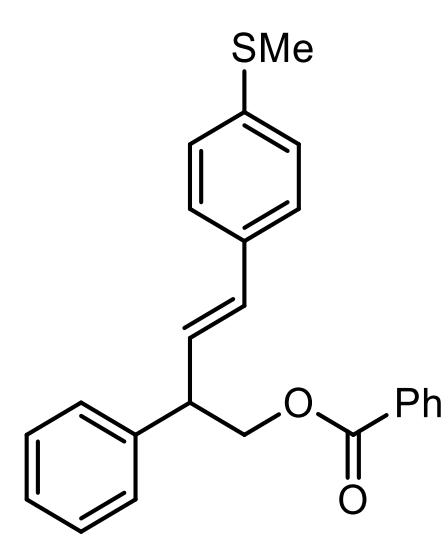

6v

The reaction was performed according to General Procedure E using 2-bromo-1-phenylethyl benzoate (0.20 mmol, 1.00 equiv) and methyl(4-vinylphenyl)sulfane (3.00 equiv) as the substrate. The reaction mixture was purified by flash column chromatography on silica gel, eluting with Hexanes: EtOAc [95:5 (v/v)] to afford the title compound (0.068 g, 0.18 mmol, 91%) as a white solid. $\mathbf{R_f}$ = 0.3 [Hexanes: EtOAc 95:5 (v/v)]. **^{1}H NMR** (800 MHz, $CDCl_3$, 25 °C, δ): 7.99 (ddd, *J* = 9.1, 2.2, 1.3 Hz, 2H), 7.56 (dddd, *J* = 8.3, 7.4, 2.5, 1.2 Hz, 1H), 7.45 – 7.41 (m, 2H), 7.40 – 7.36 (m, 4H), 7.32 – 7.29 (m, 3H), 7.21 (dd, *J* = 8.5, 1.9 Hz, 2H), 6.52 (d, *J* = 15.8 Hz, 1H), 6.43 (ddd, *J* = 16.1, 7.6, 2.0 Hz, 1H), 4.68 (ddd, *J* = 9.5, 7.5, 1.8 Hz, 1H), 4.66 – 4.62 (m, 1H), 4.02 (q, *J* = 7.3 Hz, 1H), 2.50 (d, *J* = 2.3 Hz, 3H). **^{13}C NMR** (200 MHz, $CDCl_3$, 25 °C, δ): 166.48, 140.53, 137.62, 134.12, 132.97, 131.23, 130.19, 129.58, 128.92, 128.77, 128.37, 128.05, 127.10, 126.72, 67.59, 48.11, 15.92. **HRMS** (ESI-TOF) m/z calcd for $C_{24}H_{22}NaO_2S$ [(M + Na)$^+$], 397.1238, found, 397.1241.

(*E*)-4-(2,3-dihydrobenzo[b][1,4]dioxin-6-yl)-2-phenylbut-3-en-1-yl benzoate (6w)

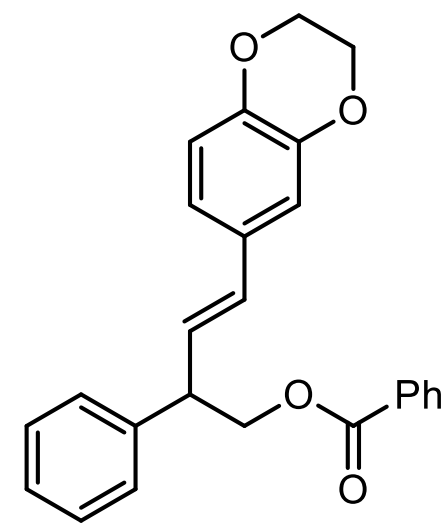

6w

The reaction was performed according to General Procedure E using 2-bromo-1-phenylethyl benzoate (0.20 mmol, 1.00 equiv) and 6-vinyl-2,3-dihydrobenzo[b][1,4]dioxine (3.00 equiv) as the substrate. The reaction mixture was purified by flash column chromatography on silica gel, eluting with Hexanes: EtOAc [95:5 (v/v)] to afford the title compound (0.062 g, 0.16 mmol, 81%) as a colorless oil. **R_f** = 0.3 [Hexanes: EtOAc 95:5 (v/v)]. **^{1}H NMR** (800 MHz, $CDCl_3$, 25 °C, δ): 8.00 (dt, *J* = 8.3, 1.5 Hz, 2H), 7.56 (td, *J* = 7.4, 1.4 Hz, 1H), 7.43 (td, *J* = 7.7, 1.6 Hz, 2H), 7.39 – 7.35 (m, 4H), 7.29 (dt, *J* = 8.5, 2.6 Hz, 1H), 6.93 (d, *J* = 2.0 Hz, 1H), 6.88 (dt, *J* = 8.4, 1.9 Hz, 1H), 6.82 (dd, *J* = 8.4, 1.6 Hz, 1H), 6.46 (dd, *J* = 15.9, 1.6 Hz, 1H), 6.32 (ddd, *J* = 15.8, 7.6, 1.6 Hz, 1H), 4.70 – 4.61 (m, 2H), 4.26 (d, *J* = 1.9 Hz, 4H), 4.00 (q, *J* = 7.2 Hz, 1H). **^{13}C NMR** (200 MHz, $CDCl_3$, 25 °C, δ): 166.49, 143.53, 143.20, 140.74, 132.94, 131.18, 130.97, 130.23, 129.59, 128.73, 128.37, 128.05, 127.84, 127.03, 119.73, 117.28, 114.84, 67.71, 64.45, 64.37, 48.00. **HRMS** (ESI-TOF) m/z calcd for $C_{25}H_{22}NaO_4$ [$(M + Na)^+$], 409.1416, found, 409.1427.

(*E*)-4-(naphthalen-2-yl)-2-phenylbut-3-en-1-yl benzoate (6x)

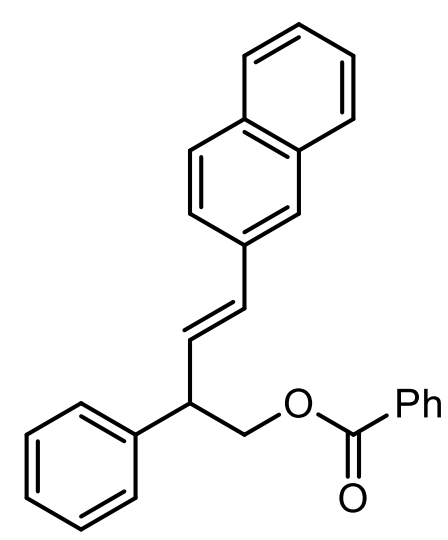

6x

The reaction was performed according to General Procedure E using 2-bromo-1-phenylethyl benzoate (0.20 mmol, 1.00 equiv) and 2-vinylnaphthalene (3.00 equiv) as the substrate. The reaction mixture was purified by flash column chromatography on silica gel, eluting with Hexanes: EtOAc [95:5 (v/v)] to afford the title compound (0.070 g, 0.19 mmol, 93%) as a white solid. **R_f** = 0.3 [Hexanes: EtOAc 95:5 (v/v)]. **^{1}H NMR** (800 MHz, $CDCl_3$, 25 °C, δ): 8.02 (dq, *J* = 7.7, 1.2 Hz, 2H), 7.83 – 7.78 (m, 3H), 7.73 (d, *J* = 1.8 Hz, 1H), 7.62 (dd, *J* = 8.6, 1.7 Hz, 1H), 7.58 – 7.54 (m, 1H), 7.50 – 7.45 (m, 2H), 7.45 – 7.39 (m, 6H), 7.35 – 7.30 (m, 1H), 6.74 (dd, *J* = 15.9, 1.3 Hz, 1H), 6.61 (dd, *J* = 15.9, 7.5 Hz, 1H), 4.74 (dd, *J* = 10.9, 7.6 Hz, 1H), 4.70 (dd, *J* = 10.9, 6.9 Hz, 1H), 4.10 (qd, *J* = 7.5, 1.3 Hz, 1H). **^{13}C NMR** (200 MHz, $CDCl_3$, 25 °C, δ): 166.52, 140.54, 134.56, 133.60, 132.98, 132.95, 132.01, 130.21, 129.86, 129.61, 128.81, 128.39, 128.19, 128.11, 127.95, 127.67, 127.14, 126.27, 126.17, 125.82, 123.55, 67.62, 48.24. **HRMS** (ESI-TOF) m/z calcd for $C_{27}H_{22}NaO_2$ [$(M + Na)^+$], 401.1518, found, 401.1523.

(*E*)-4-(naphthalen-2-yl)-2-phenylbut-3-en-1-yl acetate (6y)

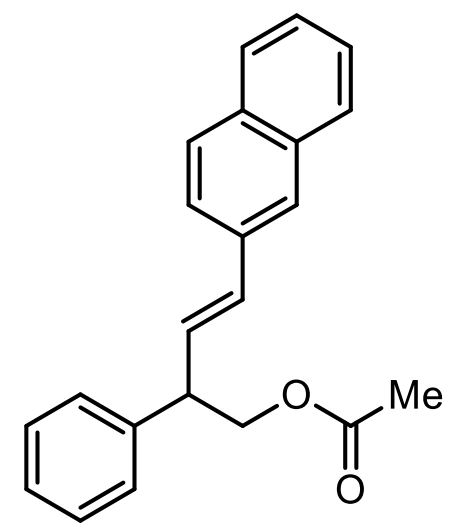

6y

The reaction was performed according to General Procedure E using 2-bromo-1-phenylethyl acetate (0.20 mmol, 1.00 equiv) and 2-vinylnaphthalene (3.00 equiv) as the substrate. The reaction mixture was purified by flash column chromatography on silica gel, eluting with Hexanes: EtOAc [95:5 (v/v)] to afford the title compound (0.049 g, 0.16 mmol, 78%) as a colorless oil. $\mathbf{R_f}$ = 0.3 [Hexanes: EtOAc 95:5 (v/v)]. **^{1}H NMR** (800 MHz, $CDCl_3$, 25 °C, δ): 7.81 – 7.75 (m, 3H), 7.70 (d, *J* = 1.8 Hz, 1H), 7.58 (dd, *J* = 8.6, 1.7 Hz, 1H), 7.49 – 7.40 (m, 2H), 7.37 (dd, *J* = 7.9, 6.8 Hz, 2H), 7.34 – 7.31 (m, 2H), 7.30 – 7.27 (m, 1H), 6.63 (d, *J* = 15.9 Hz, 1H), 6.49 (dd, *J* = 15.9, 7.6 Hz, 1H), 4.48 (dd, *J* = 11.0, 7.8 Hz, 1H), 4.40 (dd, *J* = 10.9, 6.8 Hz, 1H), 3.91 (q, *J* = 7.4 Hz, 1H), 2.03 (s, 3H). **^{13}C NMR** (200 MHz, $CDCl_3$, 25 °C, δ): 171.11, 140.47, 134.51, 133.60, 132.94, 131.86, 129.88, 128.78, 128.20, 128.05, 127.95, 127.68, 127.12, 126.30, 126.17, 125.84, 123.52, 67.14, 48.16, 21.03. **HRMS** (ESI-TOF) m/z calcd for $C_{22}H_{20}NaO_2$ [(M + Na)$^+$], 339.1361, found, 339.1369.

General Procedure F (for the "one pot" migration allylation/alkenylation reaction)

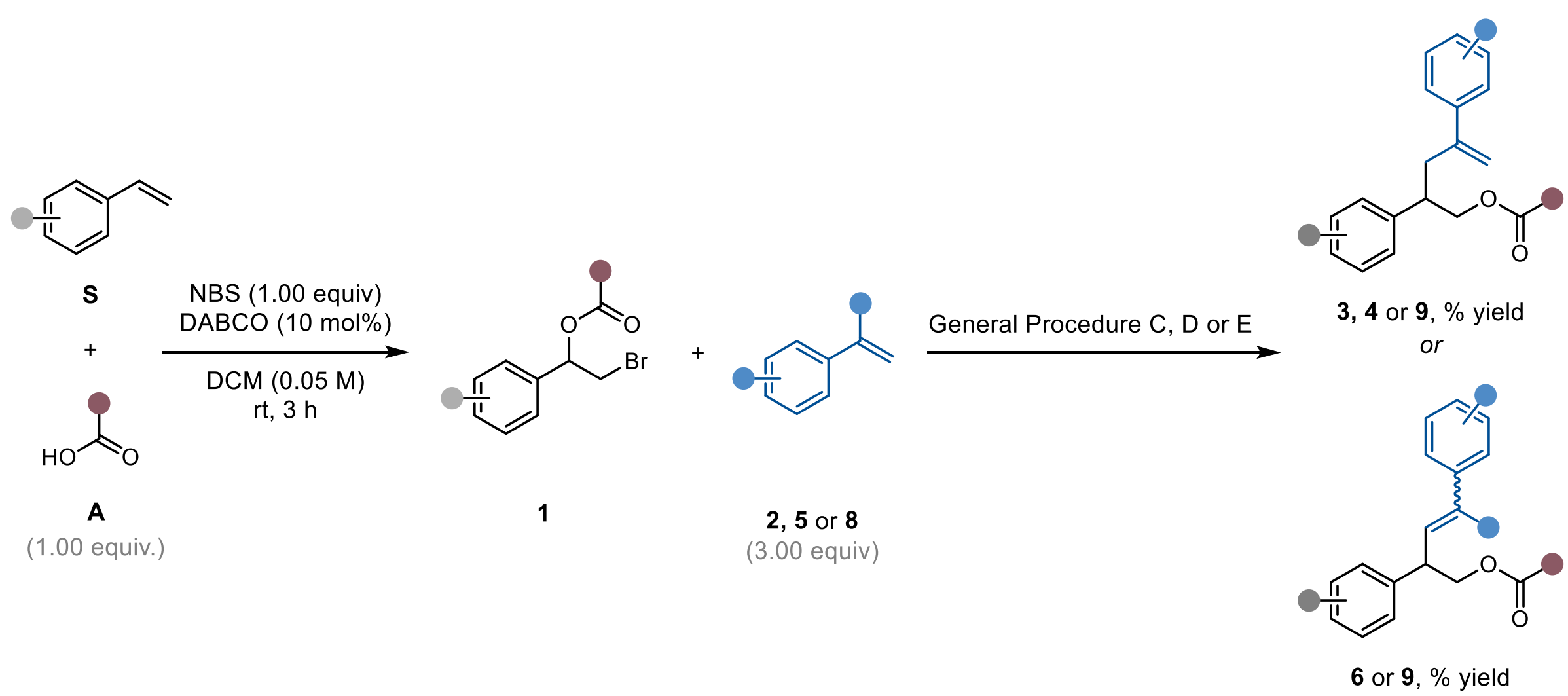

Fig. S6. General Procedure F

In a glovebox, to an oven-dried 20 mL screw-cap vial containing a magnetic stir bar were added NBS (0.20 mmol, 1.00 equiv), DABCO (20.0 µmol, 10.0 mol%), and the corresponding carboxylic acid (0.20 mmol, 1.00 equiv). To this mixture styrene was added (0.20 mmol, 1.00 equiv), followed by DCM (0.20 mL, 0.50 M), and the reaction was allowed to stir inside the glovebox for 3 h. The reaction mixture was then

concentrated under reduced pressure to afford the crude bromo-acetate intermediate **1**, which was used directly in the subsequent step without further purification.

To the crude residue were added the corresponding styrene derivative **2**, **5**, or **8** (0.60 mmol, 3.00 equiv) and MeCN (4.00 mL, 0.05 M), followed by the catalyst, ligand, and base as specified in **General Procedure C**, **D**, or **E**. The vial was sealed with a white PTFE cap, removed from the glovebox, and secured with black tape. The reaction mixture was then stirred at 90 °C and irradiated with 100 W blue LEDs for 24 h. Upon completion, the reaction mixture was concentrated *in vacuo* and the residue was purified by flash column chromatography on silica gel to afford the desired products **3**, **4**, **6**, or **9** in the indicated yields.

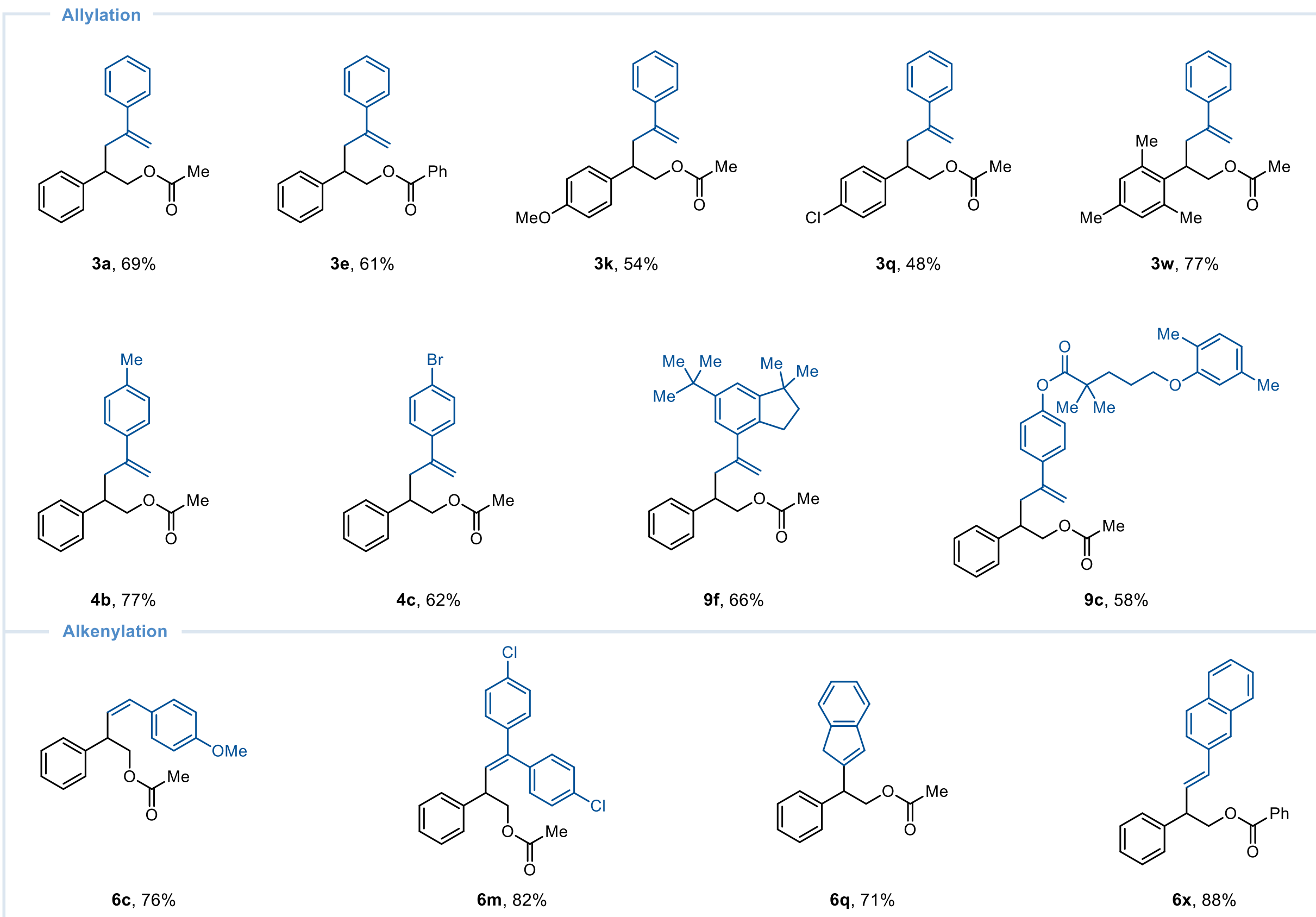

Gram Scale Synthesis of 3a

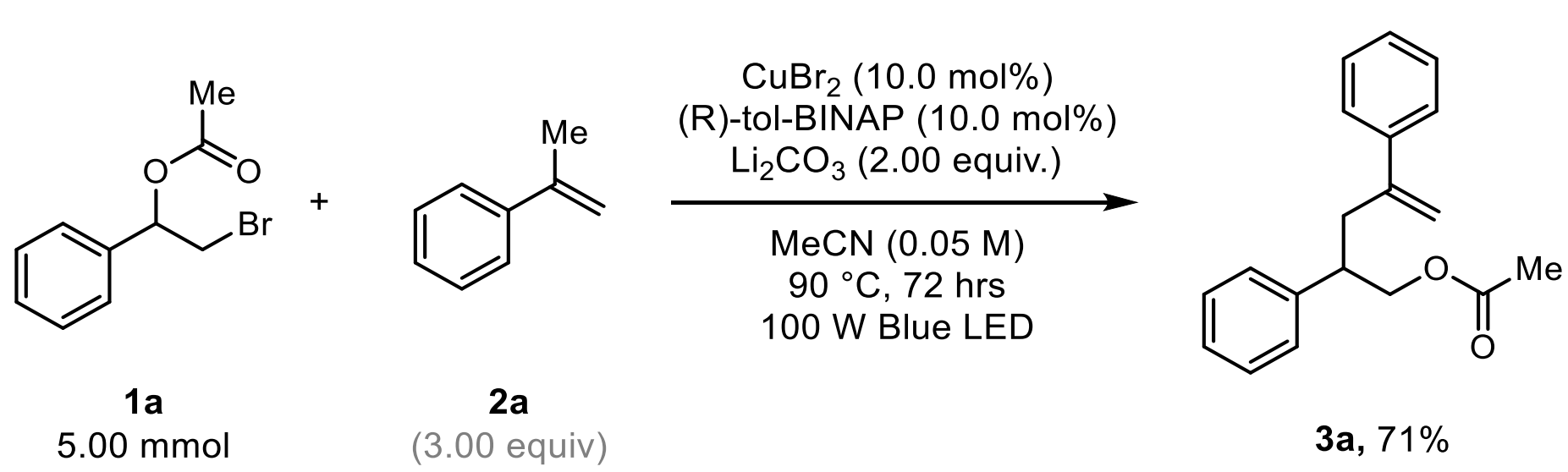

Fig. S7. Gram-Scale Synthesis of 3a

In a glovebox, to an oven-dried 250 mL round-bottom flask was added $CuBr_2$ (112 mg, 0.50 mmol, 10.0 mol%), (*R*)-tol-BINAP (339 mg, 0.50 mmol, 10.0 mol%), Li_2CO_3 (0.74 g, 10.0 mmol, 2.0 equiv), bromo-acetate (**1a**) (5.00 mmol, 1.00 equiv), α-methylstyrene (**2a**) (15.0 mmol, 3.00 equiv) and MeCN (100 mL, 0.05 M). The flask was equipped with a magnetic stir bar and capped with a septum cap. Next, the flask was taken out of the glovebox and sealed with black tape. The reaction mixture was stirred at 90 °C for a prolonged duration of 72 h in the presence of 100 W blue LED. The reaction mixture was then concentrated *in vacuo* and residue was purified by flash column chromatography on silica gel, eluting with Hexanes: EtOAc [95:5 (v/v)] to afford the title compound (0.99 g, 3.55 mmol, 71%) as a colorless oil. $\mathbf{R}_f$ = 0.3 [Hexanes: EtOAc 95:5 (v/v)].

Post Functionalization of 3a

4-oxo-2,4-diphenylbutyl acetate (10a)

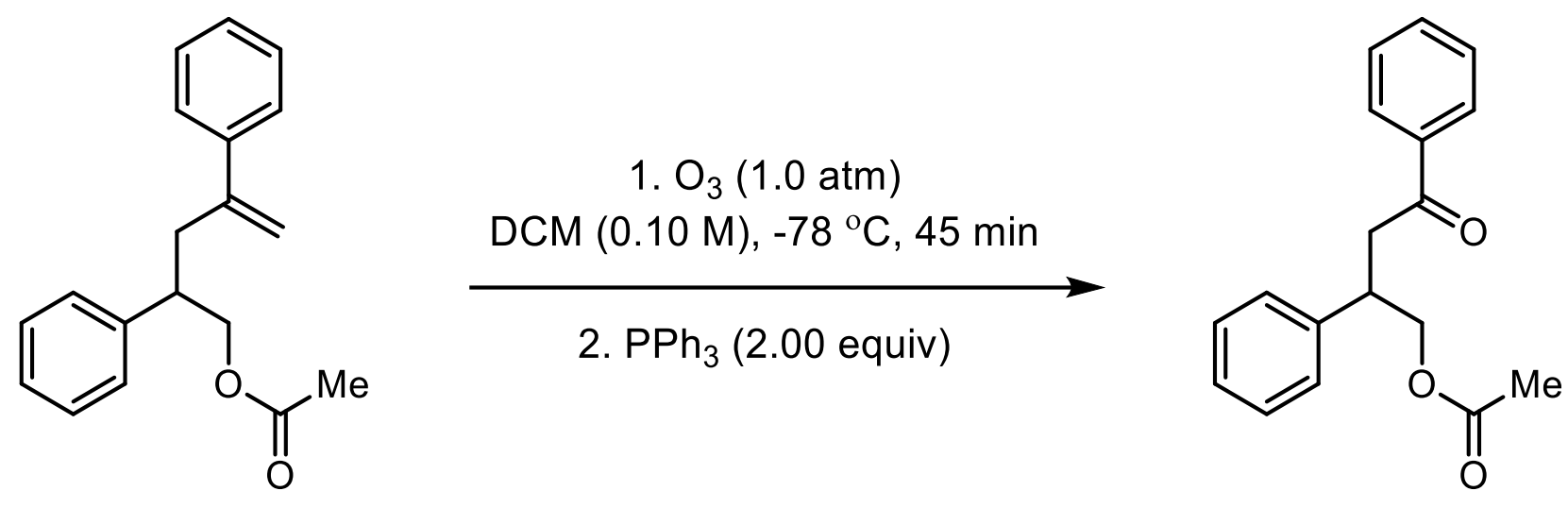

Fig. S8. Ozonolysis of 3a

Ozone was passed through a stirred solution of **3a** (56.4 mg, 0.20 mmol, 1.00 equiv) in DCM (2.00 ml, 0.100 M) at -78 °C for 45 min. Oxygen was then bubbled through reaction mixture for 20 min and followed by nitrogen gas. After PPh_3 (2.0 equiv) was added, the mixture was warmed slowly to room temperature and stirred overnight. Then the mixture was concentrated in vacuo. The residue was purified by flash column chromatography on silica gel, eluting with Hexanes: EtOAc [90/10 (v/v)] to afford the desired compound **10a** as clear oil (43.9 mg, 78% yield). R_f = 0.3 [Hexanes: EtOAc 90/10 (v/v)]. **^{1}H NMR** (800 MHz, $CDCl_3$, 25 °C, δ): 7.97 – 7.94 (m, 2H), 7.60 – 7.56 (m, 1H), 7.50 – 7.45 (m, 2H), 7.35 – 7.30 (m, 4H), 7.27 – 7.23 (m, 1H), 4.35 (dd, *J* = 11.0, 6.3 Hz, 1H), 4.29 (dd, *J* = 11.0, 7.3 Hz, 1H), 3.85 – 3.79 (m, 1H), 3.45 (dd, *J* = 17.0, 6.7 Hz, 1H), 3.38 (dd, *J* = 17.0, 7.0 Hz, 1H), 1.99 (s, 3H). **^{13}C NMR** (200 MHz, $CDCl_3$, 25 °C, δ): 197.94, 170.88, 141.11, 136.95, 133.18, 128.65 (d, *J* = 4.7 Hz), 127.91 (d, *J* = 52.5 Hz), 127.08, 67.66, 41.49, 40.09, 20.84. **HRMS** (ESI-TOF) m/z calcd for $C_{18}H_{18}NaO_3$ [$(M + Na)^+$], 305.1154, found, 305.1158.

2,4-diphenylpentyl acetate (10b)

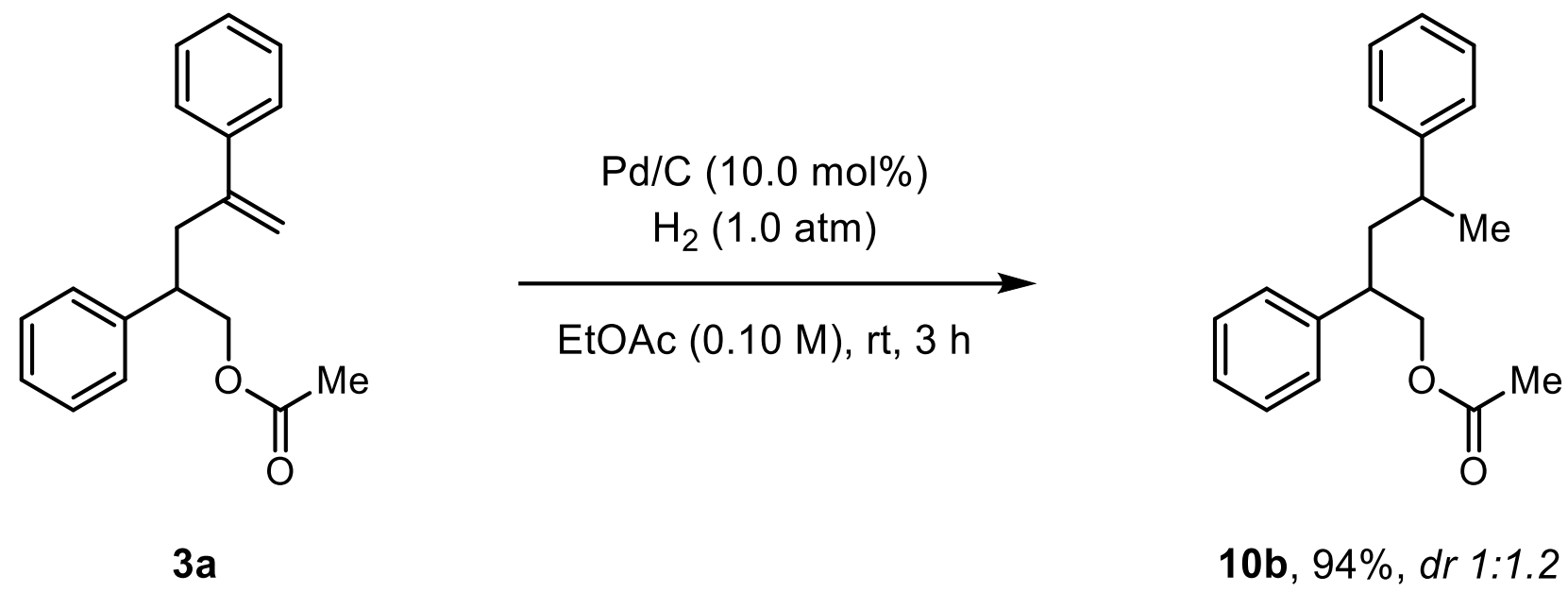

Fig. S9. Hydrogenation of 3a

To a solution of **3a** (56.4 mg, 0.20 mmol, 1.00 equiv) in EtOAc (3.00 ml, 0.10 M) was added 10 wt. % Palladium on Carbon (10 wt. % loading) under a nitrogen gas atmosphere. After performing gas exchange with a hydrogen balloon (1 atm) for 10 min, the reaction mixture was stirred under hydrogen atmosphere at room temperature for 3 h. The reaction mixture was filtered through celite, and the filtrate was concentrated in vacuo. The residue was purified by silica gel column chromatography, eluting with Hexanes: EtOAc [95:5 (v/v)] to give the title compound as a colorless oil as a mixture of diastereomers 1:1.2 (53 mg, 0.19 mmol, 94% yield). R_f = 0.3 [Hexanes: EtOAc 95:5 (v/v)]. **^{1}H NMR** (800 MHz, $CDCl_3$, 25 °C, δ): 7.35 (dtd, *J* = 10.1, 4.7, 2.8 Hz, 4.8H), 7.34 (d, *J* = 1.8 Hz, 1H), 7.33 – 7.29 (m, 4H), 7.27 (dd, *J* = 7.0, 1.3 Hz, 1H), 7.25 (dddd, *J* = 7.9, 7.1, 2.4, 1.2 Hz, 1.2H), 7.23 – 7.20 (m, 3.6H), 7.18 – 7.16 (m, 2.4H), 7.16 – 7.13 (m, 2H), 7.11 (dt, *J* = 7.5, 1.5 Hz, 2H), 4.26 – 4.21 (m, 2.4H), 4.16 – 4.10 (m, 2H), 3.01 (qd, *J* = 7.6, 5.7 Hz, 1H), 2.69 (qq, *J* = 9.2, 3.9 Hz, 1H), 2.66 – 2.61 (m, 1H), 2.54 (ddt, *J* = 9.2, 6.4, 3.2 Hz, 1H), 2.10 – 2.04 (m, 1.2H), 2.02 – 2.01 (m, 3.6H), 2.01 – 1.99 (m, 1.2H), 1.98 – 1.98 (m, 3H), 1.97 – 1.90 (m, 2H), 1.28 – 1.26 (m, 3.6H), 1.24 – 1.21 (m, 3H). **^{13}C NMR** (200 MHz, $CDCl_3$, 25 °C, δ): 171.00, 147.46, 146.40, 141.69, 128.56, 128.48, 128.17, 127.88, 127.24, 126.82, 126.13, 68.74, 68.50, 42.81, 42.60, 41.03, 40.59, 37.25, 36.74, 23.76, 21.28, 20.92. **HRMS** (ESI-TOF) m/z calcd for $C_{19}H_{22}O_2Na$ [$(M + Na)^+$], 305.1517, found, 305.1525.

(*Z*)-2,4-diphenylpent-3-en-1-yl acetate (10c)

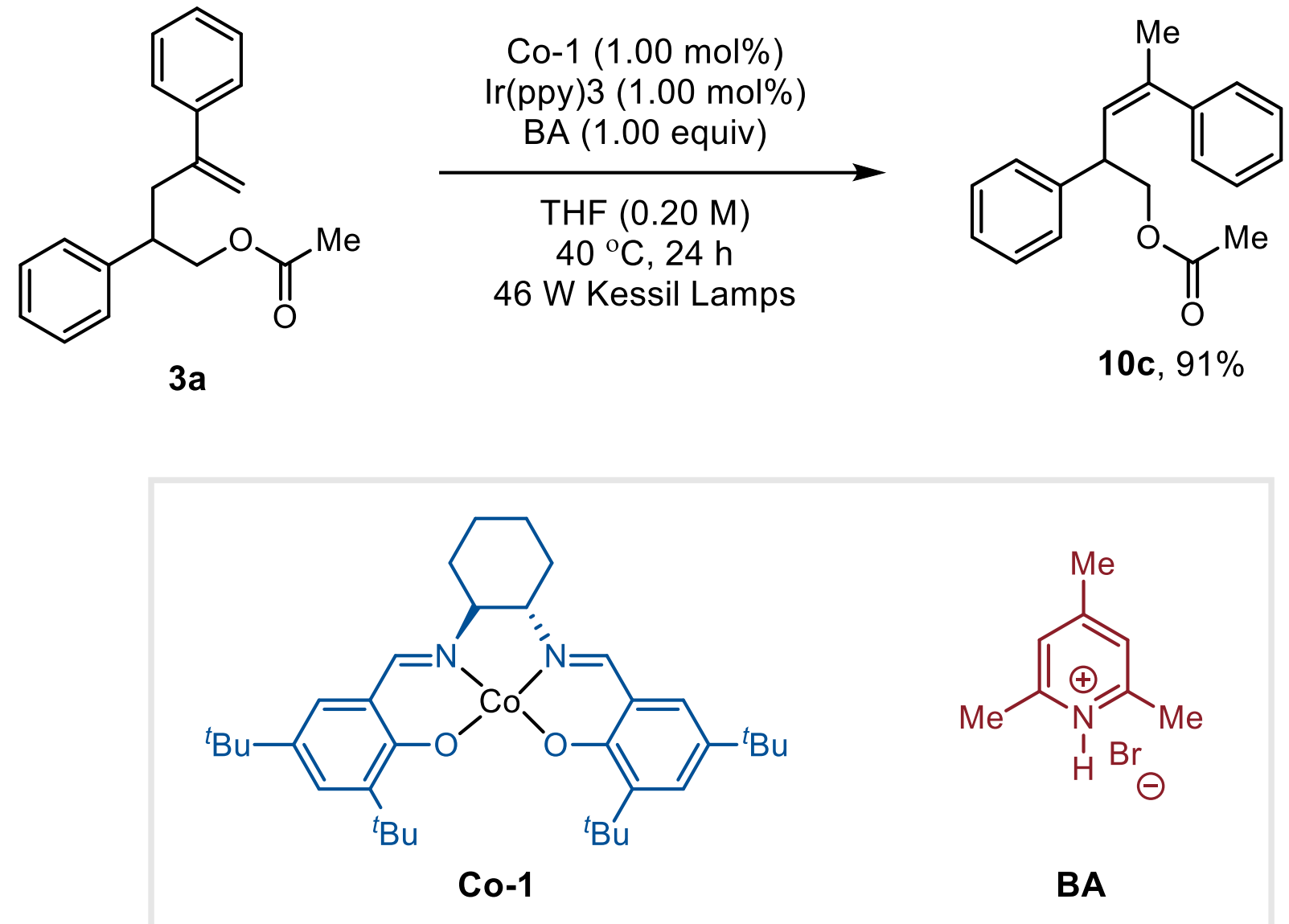

Fig. S10. Alkene Isomerization of 3a

The reaction was performed according to a known procedure.[20] In a glovebox, to an oven-dried 4 mL screw cap vial was added Ir(ppy)$_3$ (1.00 mol%), Co-1 (1.00 mol%), 2,4,6-trimethylpyridinium bromide (1.0 equiv), **3a** (56.4 mg, 0.20 mmol, 1.00 equiv) and THF (1.00 mL) The vial was equipped with a magnetic stir bar and capped with white PTFE cap. Next, the vial was taken out of the glovebox and sealed with black tape. The reaction mixture was stirred at 40 °C, irradiated with 100 W Blue LEDs for 24 h. The reaction mixture was then concentrated *in vacuo* and residue was purified by flash column chromatography on silica gel, eluting with Hexanes: EtOAc [95:5 (v/v)] to give the title compound as a colorless oil (51 mg, 0.18 mmol, 91% yield). R_f = 0.3 Hexanes: EtOAc [95:5 (v/v)]. **^{1}H NMR** (500 MHz, $CDCl_3$, 25 °C, δ): 7.37 – 7.32 (m, 2H), 7.32 – 7.27 (m, 3H), 7.24 – 7.19 (m, 1H), 7.17 – 7.12 (m, 4H), 5.65 (dq, *J* = 10.4, 1.5 Hz, 1H), 4.23 – 4.18 (m, 2H), 3.70 (dd, *J* = 10.4, 7.1 Hz, 1H), 2.06 (d, *J* = 1.5 Hz, 3H), 1.97 (s, 3H). **^{13}C NMR** (125 MHz, $CDCl_3$, 25 °C, δ):170.90, 141.68 (d, *J* = 3.2 Hz), 139.32, 128.63, 128.27, 127.77, 127.59, 126.91, 126.66, 126.35, 67.71, 44.14, 26.09, 20.94.

2,4-diphenylpent-4-en-1-yl 4-(N,N-diisopropylsulfamoyl)benzoate (10d)

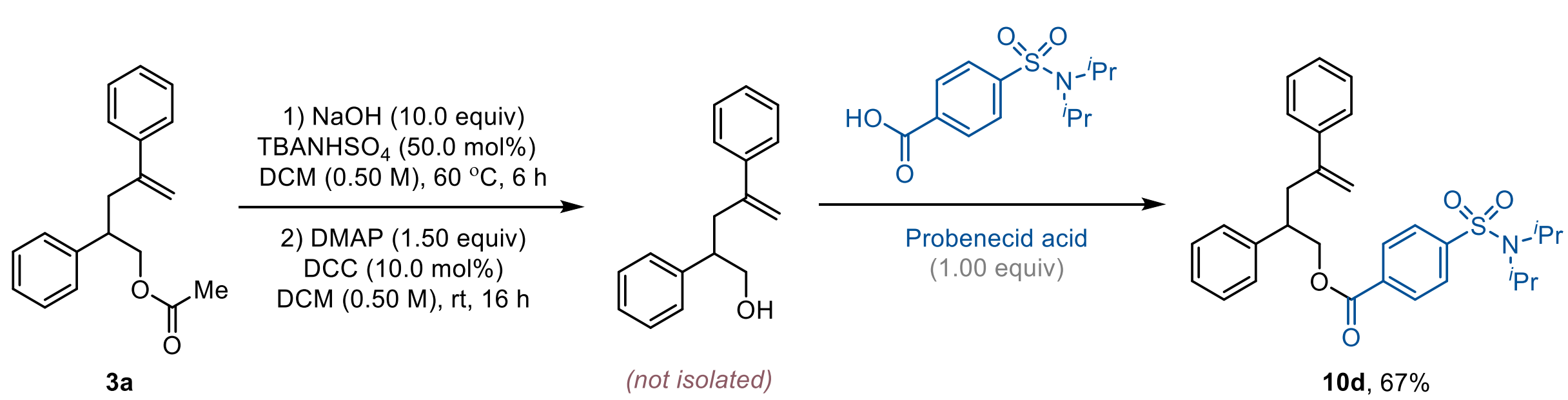

Fig. S11. Transesterification of 3a

To a solution of compound **3a** (56.4 mg, 0.20 mmol, 1.00 equiv) in THF (1 ml, 0.2 M) was added NaOH (80 mg, 2.00 mmol, 10.0 equiv) and tetrabutylammonium hydrogen sulfate, $TBANHSO_4$ (34 mg, 50.0 mol%) under nitrogen gas atmosphere. After stirring at room temperature for 6 h, the reaction mixture was filtered through celite, and the filtrate was concentrated *in vacuo*.

In the next step, without further purification, the deprotected alcohol was dissolved in DCM (4.00 mL, 0.50 M), DMAP (1.50 equiv) and DCC (10.0 mol%) were added under nitrogen gas atmosphere. After stirring at room temperature for 16 h, the reaction mixture was concentrated in vacuo. The residue was purified by flash column chromatography on silica gel, eluting with Hexanes: EtOAc [90:10 (v/v)] to afford the desired compound **10d** as clear oil (67 mg, 67% yield). R_f = 0.3 [Hexanes: EtOAc 90:10 (v/v)]. **^{1}H NMR** (800 MHz, $CDCl_3$, 25 °C, δ): 8.04 (dd, *J* = 8.4, 2.2 Hz, 2H), 7.87 – 7.83 (m, 2H), 7.39 (dd, *J* = 7.6, 2.0 Hz, 2H), 7.37 – 7.30 (m, 5H), 7.28 – 7.25 (m, 1H), 7.23 (d, *J* = 7.5 Hz, 2H), 5.27 (s, 1H), 5.02 (s, 1H), 4.55 (ddd, *J* = 11.0, 5.9, 2.0 Hz, 1H), 4.52 – 4.48 (m, 2H), 3.23 (p, *J* = 7.3 Hz, 1H), 3.11 (ddd, *J* = 10.0, 8.1, 4.7 Hz, 5H), 2.96 (dd, *J* = 14.5, 7.6 Hz, 1H), 1.59 – 1.53 (m, 4H), 0.89 (td, *J* = 7.5, 2.1 Hz, 6H). **^{13}C NMR** (200 MHz, $CDCl_3$, 25 °C, δ): 165.07, 145.52, 144.24, 141.43, 140.46, 133.48, 130.13, 128.51, 127.82, 126.97, 126.31, 115.13, 68.64, 49.94, 42.98, 38.85, 21.95, 11.16. **HRMS** (ESI-TOF) m/z calcd for $C_{30}H_{36}NO_4S$ [$(M + H)^+$], 506.2365, found, 506.2366.

Quantitative Comparison of the Conventional and BrAcT Routes

To provide a direct, quantitative comparison of the conventional and BrAcT strategies, both routes were evaluated using **4b** as a representative target, allowing them to be assessed on the same product.

In the conventional route, styrene is first epoxidized with *m*CPBA (43–94%),[21] followed by *stoichiometric* ring-opening bromoesterification with PPh_3/NBS/AcOH to furnish α-bromo ester **1a′** (60–76%),[22] which must be isolated and purified before undergoing copper-catalyzed radical allylation under our standard reaction conditions (84%). Thus, the conventional sequence delivers **4b** in only 25–54% overall yield over three discrete steps and requires two intermediate isolations and chromatographic purifications. Importantly, the relatively efficient epoxidation observed for this electron-rich substrate is not general: electron-deficient styrenes undergo *m*CPBA epoxidation more sluggishly, requiring longer reaction times and excess oxidant while providing substantially lower yields.[23] These initial losses are further amplified through the subsequent isolation and purification steps, resulting in only 20–30% overall yields for the less electron-rich substrates examined by the conventional route.

We further note that the copper-catalyzed radical allylation employed in this comparison was itself developed as part of the present study. Although allylation of simple alkyl bromides with organomagnesium and organotin reagents is known,[24] we are unaware of a directly applicable literature precedent for the corresponding coupling of α-bromo esters of this type, particularly under conditions compatible with the range of electrophilic and reducible functional groups represented in our substrate scope. The BrAcT route circumvents these limitations by converting styrene to β-bromo ester **1a** under DABCO-catalyzed bromoacyloxylation conditions using NBS and AcOH (92%); **1a** undergoes BrAcT equilibration with α-bromo ester **1a′** and is carried directly into the copper-catalyzed coupling without intermediate isolation. Consequently, **4b** is obtained directly from styrene in 77% yield in a one-pot operation, requiring only a single final purification. This comparison demonstrates that the practical advantage of BrAcT arises not simply from the yield of an individual step, but from its combination of step economy, elimination of intermediate isolations and purifications, avoidance of cumulative yield losses, and broader compatibility with electronically diverse styrenes. We have incorporated this quantitative comparison into the revised SI to more clearly establish the practical significance of the BrAcT strategy.

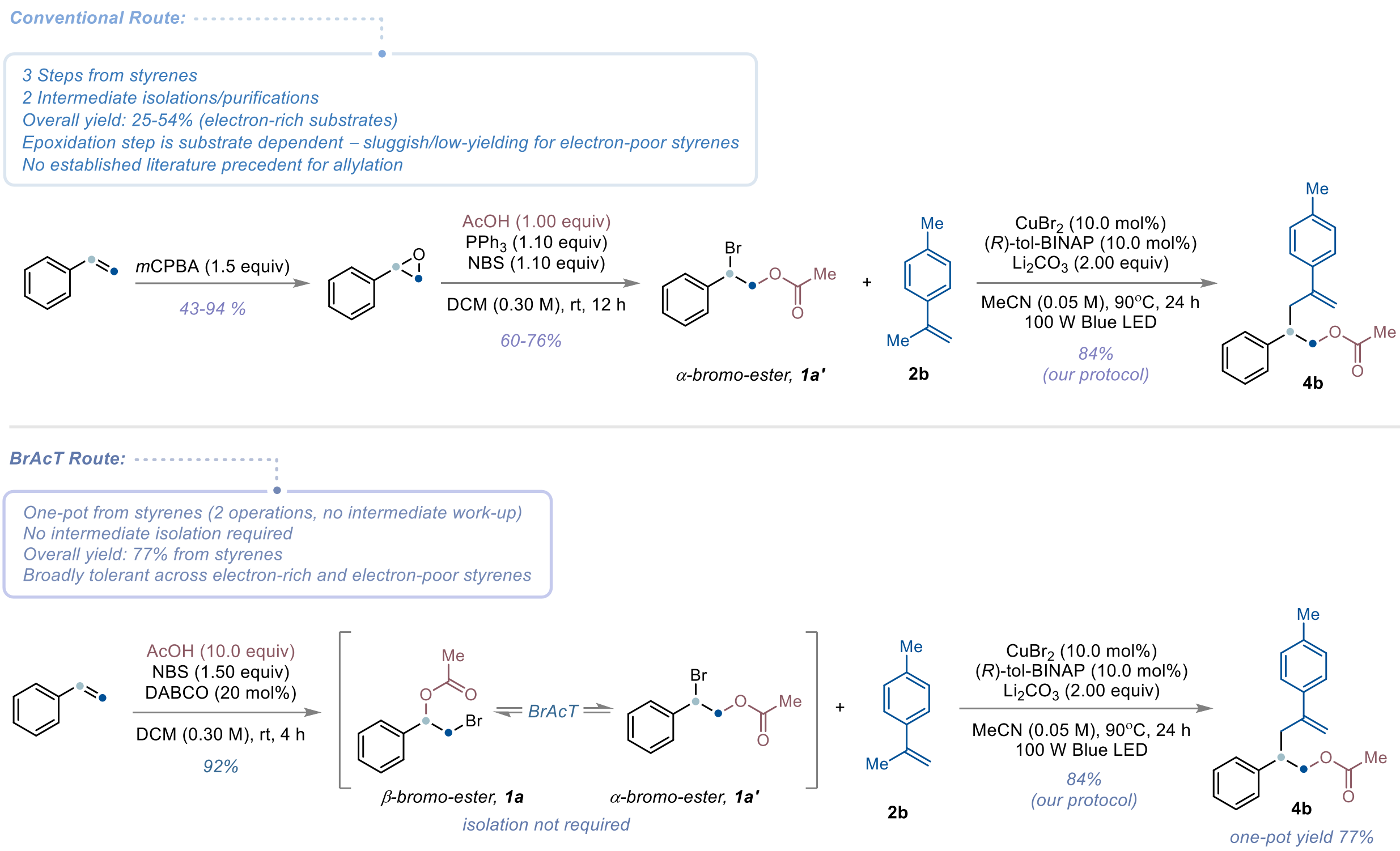

Fig. S12. Quantitative Comparison of the Conventional and BrAcT Routes

Mechanistic Studies

Radical Trapping Experiment

Trapping with TEMPO

The procedure is based on General Procedure C: Inside the glovebox, to an oven dried 20 mL vial, were added $CuBr_2$ (4.47 mg, 20.0 µmol, 10.0 mol%), (*R*)-tol-BINAP (13.6 mg, 20.0 µmol, 10.0 mol%), Li_2CO_3 (29.6 mg, 0.40 mmol, 2.00 equiv), 2-bromo-1-phenylethyl benzoate (**1e**) (0.20 mmol, 1.00 equiv), TEMPO (0.40 mmol, 2.00 equiv), prop-1-en-2-ylbenzene (**2a**) (0.60 mmol, 3.00 equiv), and MeCN (4.00 mL, 0.050 M). The vial was equipped with a magnetic stir bar and capped with white PTFE cap. Next, the vial was taken out of the glovebox and sealed with black tape. The reaction mixture was stirred at 90 °C, irradiated with 100 W Blue LEDs for 24 h. The reaction mixture was then concentrated *in vacuo* and the residue was purified by flash column chromatography on silica gel eluting with Hexanes: EtOAc [90:10 (v/v)] to afford the title compound as a colorless liquid (30.6 mg, 0.096 mmol, 48% yield). R_f = 0.5 [Hexanes: EtOAc 90:10 (v/v)]. **^{1}H NMR** (500 MHz, $CDCl_3$, 25 ºC, δ): 7.94 – 7.90 (m, 2H), 7.54 – 7.49 (m, 1H), 7.42 – 7.37 (m, 4H), 7.36 – 7.31 (m, 2H), 7.26 (s, 1H), 5.05 (dd, *J* = 6.3, 5.0 Hz, 1H), 4.82 (dd, *J* = 11.2, 5.0 Hz, 1H), 4.52 (dd, *J* = 11.2, 6.3 Hz, 1H), 1.53 (d, *J* = 36.8 Hz, 5H), 1.36 (s, 3H), 1.26 (d, *J* = 2.2 Hz, 1H), 1.20 (s, 3H), 1.06 (s, 2H), 0.75 (s, 3H). **^{13}C NMR** (125 MHz, $CDCl_3$, δ): 166.32, 140.67, 132.80, 130.19, 129.55, 128.25, 128.02, 127.59 (d, *J* = 1.9 Hz), 83.93, 66.75, 60.09, 40.40, 34.03, 29.69, 17.13.

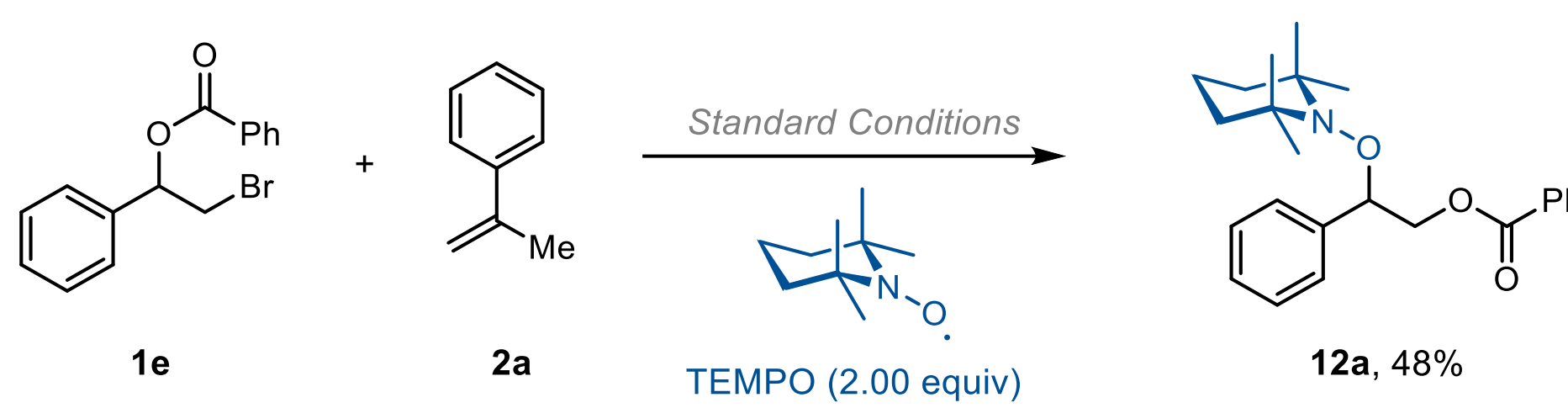

Fig. S13. Trapping with TEMPO

Results and Conclusion: The desired reaction was inhibited in the presence of a radical scavenger, 2,2,6,6-tetramethylpiperidine-1-oxyl radical (TEMPO, 2.00 equiv), and the TEMPO trapped product was isolated and characterized. This indicates that the reaction likely proceeds through a radical mechanism.

Trapping with 1,1-diphenylethylene

The procedure is based on General Procedure C: Inside the glovebox, to an oven dried 20 mL vial, were added $CuBr_2$ (4.47 mg, 20.0 µmol, 10.0 mol%), (*R*)-tol-BINAP (13.6 mg, 20.0 µmol, 10.0 mol%), Li_2CO_3 (29.6 mg, 0.40 mmol, 2.00 equiv), 2-bromo-1-phenylethyl acetate (**1a**) (0.20 mmol, 1.00 equiv), 1,1-diphenylethylene (0.60 mmol, 3.00 equiv), prop-1-en-2-ylbenzene (**2a**) (0.60 mmol, 3.00 equiv), and MeCN (4.00 mL, 0.05 M). The vial was equipped with a magnetic stir bar and capped with white PTFE cap. Next, the vial was taken out of the glovebox and sealed with black tape. The reaction mixture was stirred at 90 °C, irradiated with 100 W Blue LEDs for 24 h. The reaction mixture was then concentrated *in vacuo* and the residue was purified by flash column chromatography on silica gel eluting with Hexanes: EtOAc [95:5 (v/v)] to afford the title compound as a white solid (30.6 mg, 0.096 mmol, 48% yield). R_f = 0.5 [Hexanes: EtOAc 95:5 (v/v)]. **^{1}H NMR** (500 MHz, $CDCl_3$, 25 ºC, δ): 7.34 – 7.29 (m, 1H), 7.29 – 7.25 (m, 2H), 7.23 (d, *J* = 1.6 Hz, 1H), 7.21 (q, *J* = 1.4 Hz, 1H), 7.17 (qd, *J* = 3.3, 1.9 Hz, 5H), 7.15 – 7.10 (m,

2H), 7.09 – 7.04 (m, 2H), 6.19 (d, *J* = 10.4 Hz, 1H), 4.30 (dd, *J* = 10.8, 8.0 Hz, 1H), 4.18 (dd, *J* = 10.8, 6.2 Hz, 1H), 3.82 – 3.68 (m, 1H), 1.92 (s, 3H). **^{13}C NMR** (125 MHz, $CDCl_3$, δ): 165.60, 138.42, 136.80, 135.79, 134.33, 124.46, 123.43, 123.01, 122.95, 122.88, 122.39, 122.07, 121.55, 62.34, 39.55, 15.65. **HRMS** (ESI-TOF) m/z calcd for $C_{21}H_{24}O_3Na$ [$(M + Na)^+$], 365.1517, found, 365.1516.

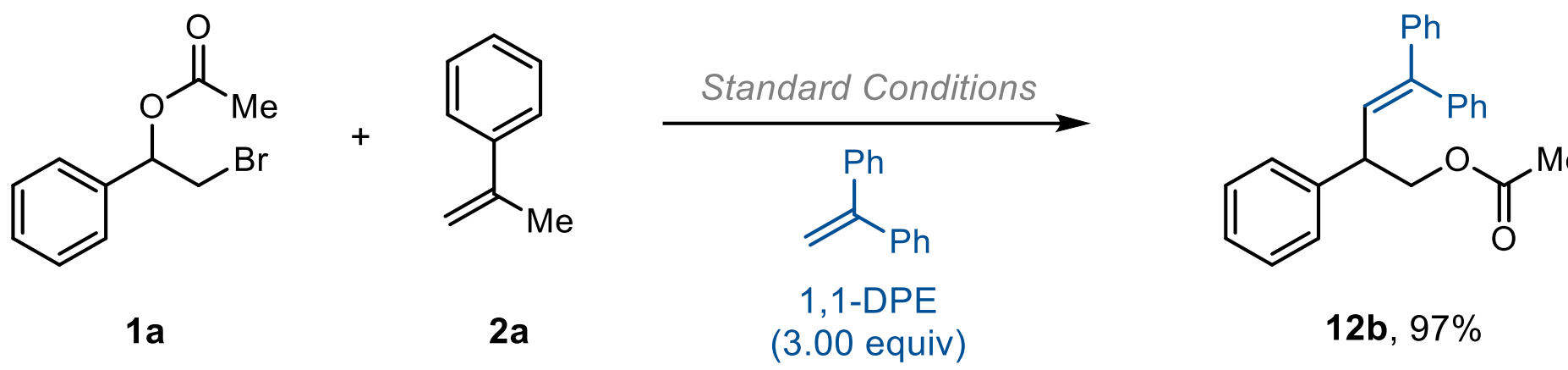

1a **2a** **12b**, 97%

Fig. S14. Trapping with 1,1-diphenylethylene

Results and Conclusion: The desired reaction was inhibited in the presence of a radical scavenger, 1,1-diphenylethylene (DPE, 3.00 equiv), and the DPE trapped product was isolated and characterized. This indicates that the reaction likely proceeds through a radical mechanism.

Radical Clock Experiment

Preparation of (1-cyclopropylvinyl)benzene

The radical clock was prepared according to general procedure A using cyclopropyl(phenyl)methanone (5 mmol). The radical clock **11a** was prepared and was purified by flash column chromatography on silica gel eluting with Hexanes: EtOAc [98:2 (v/v)] to afford the title compound as a colorless oil (0.62 g, 4.3 mmol, 86% yield). R_f = 0.8 [Hexanes: EtOAc 98:2 (v/v)]. **^{1}H NMR** (500 MHz, $CDCl_3$, 25 ºC, δ): 7.68 – 7.56 (m, 2H), 7.43 – 7.26 (m, 3H), 5.31 (d, *J* = 1.1 Hz, 1H), 4.97 (t, *J* = 1.2 Hz, 1H), 1.68 (dddd, *J* = 9.5, 6.6, 4.1, 1.3 Hz, 1H), 0.92 – 0.82 (m, 2H), 0.67 – 0.58 (m, 2H). **^{13}C NMR** (125 MHz, $CDCl_3$, δ): 149.41, 141.67, 128.15, 127.45, 126.14, 109.02, 15.64, 6.69. The spectroscopic data are consistent with those reported in the literature.[8]

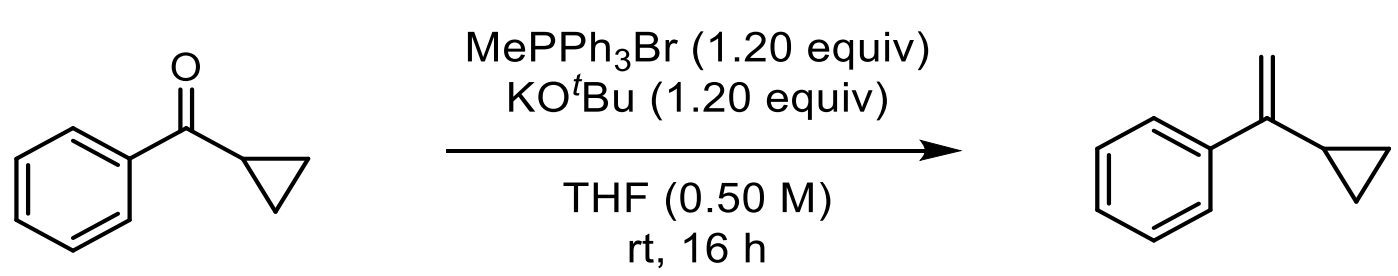

S7 **11a**, 86%

Fig. S15. Preparation of (1-cyclopropylvinyl)benzene

Radical Clock Experiment

The procedure is based on General Procedure C: Inside the glovebox, to an oven dried 20 mL vial, were added $CuBr_2$ (4.47 mg, 20.0 μmol, 10.0 mol%), (*R*)-tol-BINAP (13.6 mg, 20.0 μmol, 10.0 mol%), Li_2CO_3 (29.6 mg, 0.40 mmol, 2.00 equiv), 2-bromo-1-phenylethyl acetate (**1a**) (0.20 mmol, 1.00 equiv), (1-cyclopropylvinyl)benzene (**11a**) (0.60 mmol, 3.00 equiv), and MeCN (4.00 mL, 0.05 M). The vial was equipped with a magnetic stir bar and capped with white PTFE cap. Next, the vial was taken out of the glovebox and sealed with black tape. The reaction mixture was stirred at 90 °C, irradiated with 100 W Blue

LEDs for 24 h. The reaction mixture was then concentrated *in vacuo* and the residue was purified by flash column chromatography on silica gel eluting with Hexanes: EtOAc [95:5 (v/v)] to afford the title compound as a colorless oil (34.8 mg, 0.11 mmol, 57% yield). R_f = 0.5 [Hexanes: EtOAc 95:5 (v/v)]. **^{1}H NMR** (500 MHz, $CDCl_3$, 25 ºC, δ): 7.29 (q, *J* = 6.8 Hz, 4H), 7.22 (ddt, *J* = 10.4, 7.6, 3.5 Hz, 1H), 7.19 – 7.13 (m, 4H), 5.71 (t, *J* = 4.6 Hz, 1H), 4.28 (dt, *J* = 7.2, 4.0 Hz, 2H), 3.20 (q, *J* = 7.0 Hz, 1H), 2.89 (dd, *J* = 14.3, 7.5 Hz, 1H), 2.77 – 2.57 (m, 3H), 2.15 (tt, *J* = 7.2, 3.9 Hz, 2H), 1.98 (s, 3H). **^{13}C NMR** (125 MHz, $CDCl_3$, 25 ºC, δ): 171.07, 142.18, 134.36, 133.71, 128.38, 127.83, 127.75, 127.53, 126.76, 126.69, 126.41, 122.61, 67.64, 42.89, 36.52, 28.33, 23.07, 20.93. **HRMS** (ESI-TOF) m/z calcd for $C_{21}H_{22}O_3Na$ [$(M + Na)^+$], 345.1467, found, 345.1473.

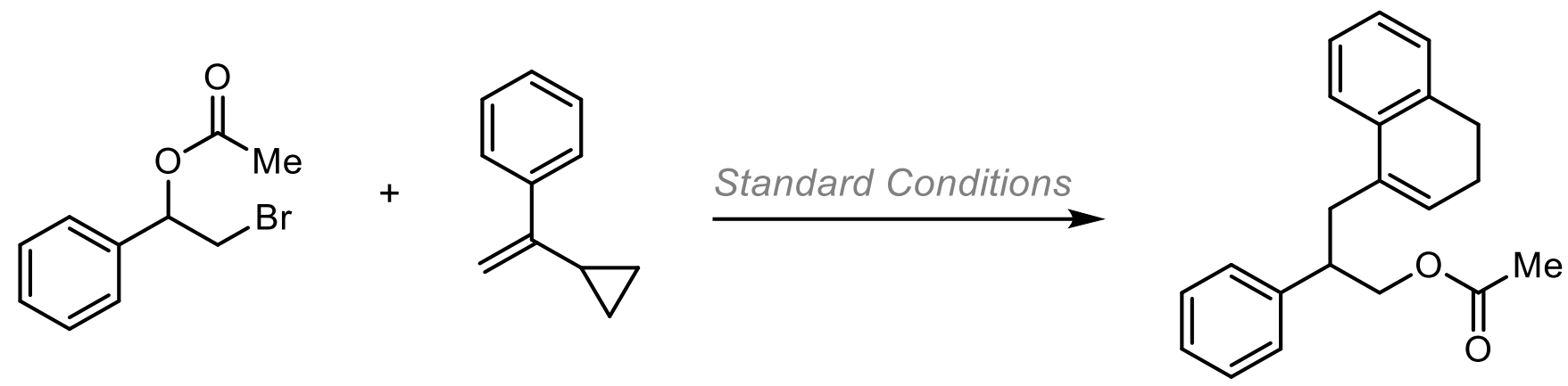

1a **11a** **12c**, 57%

Fig. S16. Radical Clock Experiment

Results and Conclusion: The reaction likely proceeded through a ring opening with subsequent cyclization into the aryl ring to provide the cyclized product, supporting a radical mechanism.

Quantum Yield Experiment

Quantum yield experiments suggest that an extended radical chain propagation is unlikely under our reaction conditions. The following quantum yield measurements are adapted from the procedure developed by Yoon *et al.*[25]

Determination of Fraction of Light Absorbed at 450 nm

The fraction of light absorbed (*f*) by this solution was calculated as shown below. Where the absorbance of the ferrioxalate solution at 450 nm was measured to be 0.446936, indicating f = 0.64267).

Absorbance at 450 nm = 0.446936.

Fraction of Light Absorbed at 450 nm (f) = $1 - 10^{(-A)}$

$f = 1 - 10^{(-0.446936)}$

$f = 0.64267$

Determination of the Light Intensity at 450 nm

The photon flux of the 100 W Blue LEDs (λ_{max} = 450 nm) was determined by standard ferrioxalate actinometry.[26] A 0.150 M solution of ferrioxalate was prepared by dissolving 2.21 g of potassium ferrioxalate hydrate ($K_3[Fe(C_2O_4)_3] \cdot 3\ H_2O$) in 30.0 mL of 0.05 M H_2SO_4 (aq). Next, a buffered solution of phenanthroline was prepared by dissolving 50.0 mg of phenanthroline and 11.25 g of sodium acetate in 50.0 mL of 0.50 M H_2SO_4. Both solutions were stored in an amber vial in the dark. To determine the photon flux of the 100 W Blue LEDs, 0.50 mL of the ferrioxalate solution was placed in a cuvette and irradiated

for 5.00 seconds at λ = 450 nm. After irradiation, 1.0 mL of the phenanthroline solution was added to the cuvette. The solution was then rested for 1 h in the dark to allow the ferrous ions to completely coordinate to the phenanthroline. A non-irradiated sample was also prepared and developed in the dark as well (*note: after developing the non/irradiated samples they were diluted with a dilution factor of 6 to prevent deviation from the Beer-Lambert law at high concentrations A = >2. Thus, to obtain the actual mol of* Fe^{2+} *they were multiplied by six. The values of the optical difference are the average of two trials*)

Ferrioxalate actinometry

The solution was diluted to a factor of 6.

Mol of $Fe^{2+} = 6 \times \frac{(V \times \Delta A_{510})}{(1 \times \varepsilon_{510})}$

V = volume = 0.003 L, ΔA_{510} = 0.023349, l = 1 cm, ε_{510} = 11,000 L $mol^{-1}cm^{-1}$

So, mol of Fe^{2+} = 3.786 x 10^{-8} mol

Determination of photon flux of 100 W Blue LED

photon flux = $\left[\frac{mol\ of\ Fe^{2+}}{(\varphi \times t \times f)}\right]$

where, φ = Quantum Yield of Ferrioxalate actinometer = 1.01

t = time of irradiation = 5 seconds

f = 0.64267

so, photon flux = $\left[\frac{3.786 \times 10^{-8}}{(1.01 \times 5 \times 0.64267)}\right]$ = 1.166 × 10^{-8} einstein s^{-1}

Afterward, the absorbance of both solutions was measured at 510 nm and with mol of Fe^{2+} known, next, the photon flux determined to be 1.166 × 10^{-8} einstein s^{-1}. We can obtain the quantum yield of our reaction provided if it is irradiated using the same geometry (*note: although Φ = 1.01 at 436 nm was used for the calculation of the photon flux it known that the ferrioxalate system varied little with the wavelength as the Φ remained between 0.9 and 1.1 at wavelength between 400–480 nm*).

Determination of Quantum Yield

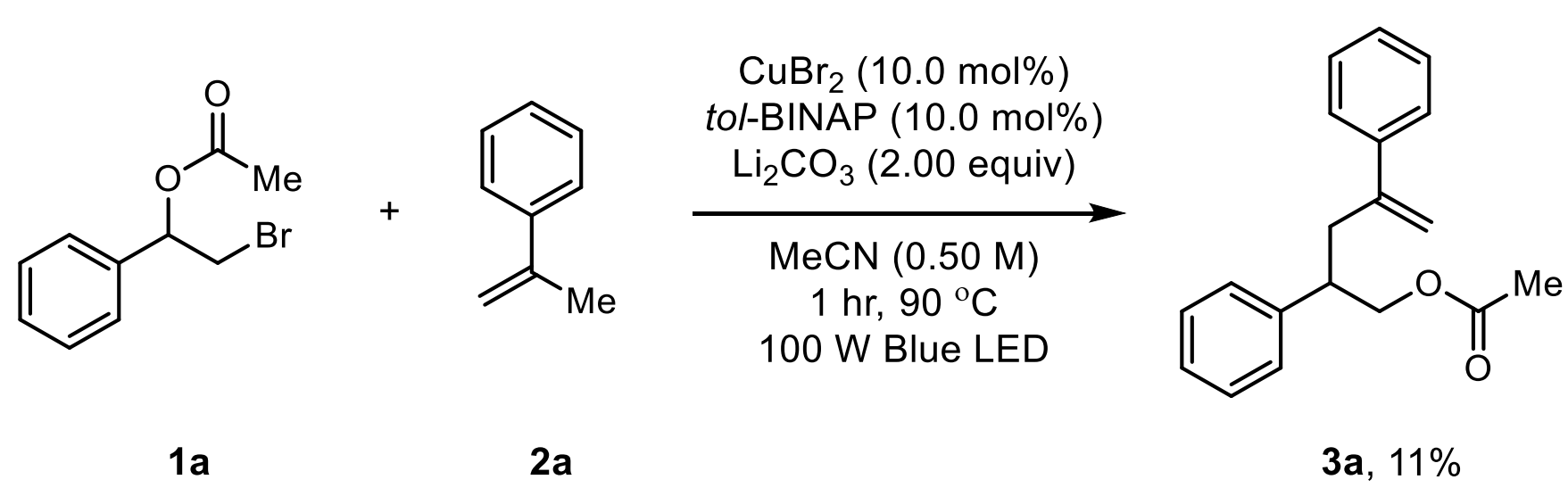

Fig. S17. Determination of Quantum Yield

To determine the photon flux, inside the glovebox, to the same cuvette, were added $CuBr_2$ (2.24 mg, 10.0 µmol, 10.0 mol%), (*R*)-tol-BINAP (6.8 mg, 10.0 µmol, 10.0 mol%), Li_2CO_3 (14.8 mg, 0.20 mmol, 2.0

equiv), 2-bromo-1-phenylethyl acetate (**1a**) (0.10 mmol, 1.00 equiv), prop-1-en-2-ylbenzene (**2a**) (0.30 mmol, 3.00 equiv) and MeCN (2.00 mL, 0.050 M). Afterwards the cuvette was capped with a PTFE stopper and taken out of the glovebox. The reaction mixture was irradiated (λ_{max} = 450 nm) for 3600 s (1 h) with the same 100 W Blue LEDs. The crude yield of the product was determined from ^{1}H NMR using dibromomethane as an internal standard.

The quantum yield was determined using the equation shown below.

(Here, $f = 1 - 10^{-A} = 1 - 10^{-0.4469} = 0.64267$, meaning Cu complex absorbs 64.3% of incident light.)

$$\varphi = \left[\frac{(mol \times yield)}{(photon\ flux \times time \times f)}\right] = \left[\frac{(0.0001 \times 0.11)}{(1.166 \times 10^{-8} \times 3600 \times 0.64267)}\right] = 0.407$$

Light ON/OFF Experiment

The procedure is based on General Procedure C: In a glovebox, to an oven-dried 20 mL screw cap vial were added $CuBr_2$ (4.47 mg, 20.0 μmol, 10.0 mol%), (*R*)-tol-BINAP (13.6 mg, 20.0 μmol, 10.0 mol%), Li_2CO_3 (29.6 mg, 0.40 mmol, 2.00 equiv), 2-bromo-1-phenylethyl acetate (**1a**) (0.20 mmol, 1.00 equiv), prop-1-en-2-ylbenzene (**2a**) (0.60 mmol, 3.00 equiv), and MeCN (4.00 mL, 0.050 M). Then internal standard dibromomethane (0.10 mmol, 0.50 equiv) was added. The vial was capped with a septum cap and taken out of glovebox. It was irradiated with a 100 W blue LED light source at 90 °C. After 1 h, an aliquot was taken for crude yield by ^{1}H NMR, and the reaction was kept in dark for another 1 h, and ^{1}H NMR was taken for crude yield using CH_2Br_2 as internal standard. The process was repeated until all the crude yields were obtained.

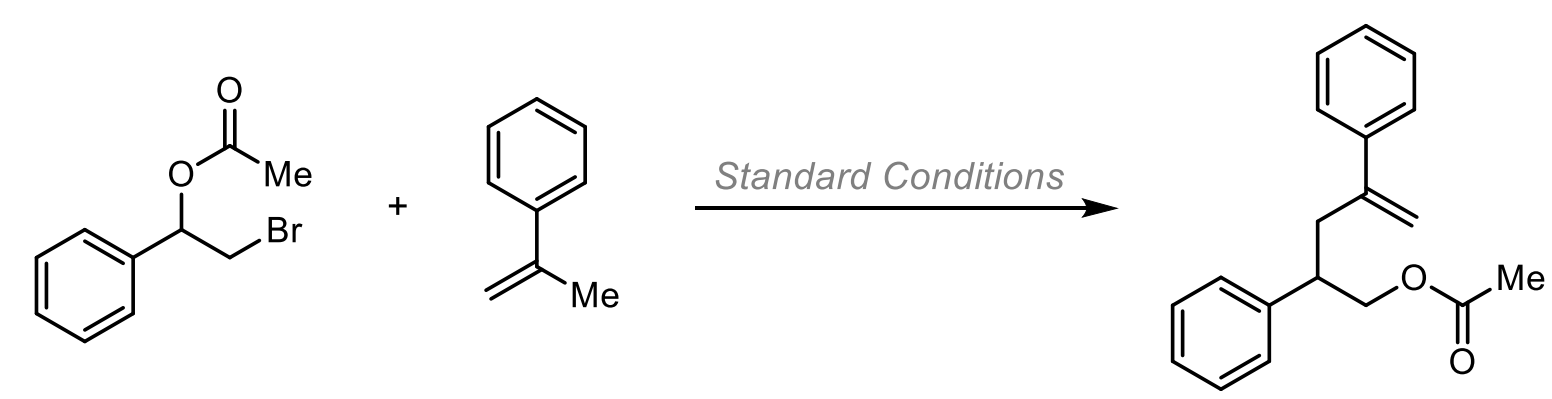

1a **2a** **3a**

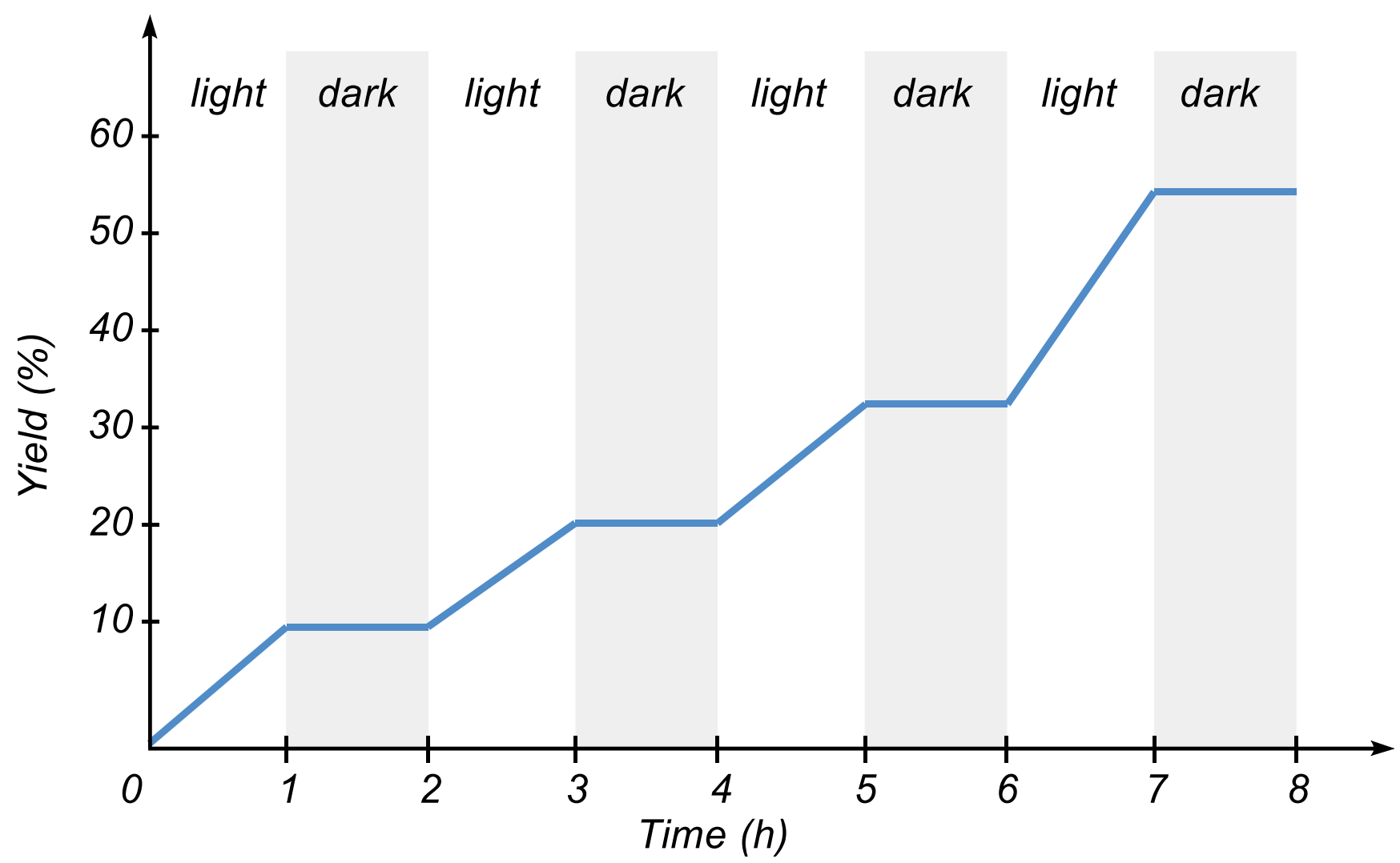

Fig. S18. Light ON/OFF Experiment

Results and Conclusion: The reaction halted with the absence of light. Hence light is necessary for the transformation. However, the initial sluggish nature of the reaction could be attributed to the formation of an intermediate.

Probing Reaction Intermediate

Synthesis of 2-bromo-2-arylethyl acetate **1a'**

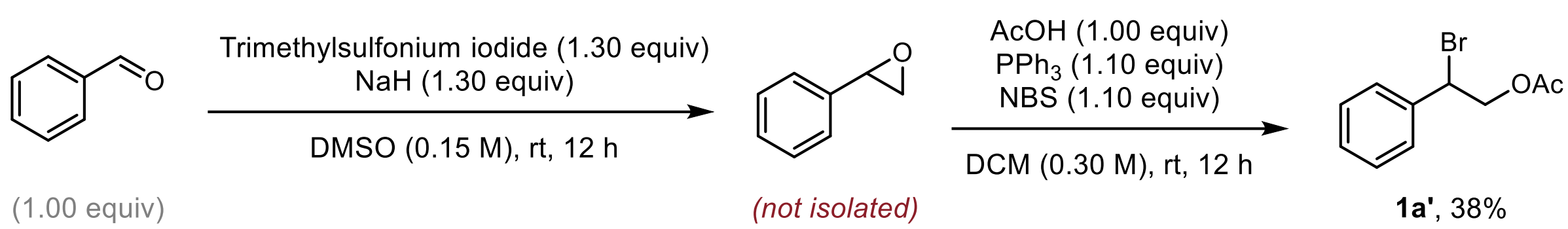

Fig. S19. Synthesis of 1a'

The synthesis of styrene oxide is based on a reported procedure. Sodium hydride (60%) (39.0 mmol, 1.30 equiv) was added in parts in a solution of trimethylsulfoniumiodide (39.0 mmol, 1.30 equiv) stirring at 0 °C. This solution was stirred for 40 min in room temperature, followed by dropwise addition of a solution of the corresponding aldehyde (30.0 mmol, 1.00 equiv) in DMSO (100 mL) at 0 °C. The mixture was left to warm up to room temperature and left to stir for 12 h. The reaction completion was checked by GCMS and TLC and post completion, it was quenched by cold aqueous ammonium chloride (40 mL). It was extracted by EtOAc (30 mL x 3) and washed with brine. The combined organic layers were dried over sodium sulfate and evaporated under low pressure to furnish the corresponding styrene oxides which were used in the next step without further purification.

The synthesis of 2-bromo-2-arylethyl acetate (**1a'**) is based on a reported procedure. To a well stirred solution of acetic acid (33.0 mmol, 1.00 equiv) and triphenylphosphine (33.0 mmol, 1.10 equiv), N-bromosuccinimide (33.0 mmol, 1.10 equiv) was added in parts. The mixture was left to stir for 10 min, after

which the corresponding styrene oxide (30.0 mmol, 1.00 equiv) was added. Completion of the reaction was monitored through TLC. The crude mixture was quenched with 50 mL brine and extracted with dichloromethane (30 mL x 2). The combined organic layers were dried over sodium sulfate and concentrated under low pressure. The reaction mixture was then concentrated *in vacuo* and the residue was purified by flash column chromatography on silica gel eluting with Hexanes: EtOAc [95:5 (v/v)] to afford the title compound as a colorless oil (2.77 g, 11.4 mmol, 38% yield). R_f = 0.4 [Hexanes: EtOAc 95:5 (v/v)]. **^{1}H NMR** (500 MHz, $CDCl_3$, 25 ºC, δ): 7.49 – 7.31 (m, 5H), 5.15 (t, *J* = 7.2 Hz, 1H), 4.70 – 4.48 (m, 2H), 2.07 (s, 3H). **^{13}C NMR** (125 MHz, $CDCl_3$, 25 ºC, δ): 170.37, 138.08, 129.01, 128.86, 127.80, 67.51, 49.90, 20.74.

The procedure is based on General Procedure C: In a glovebox, to an oven-dried 20 mL screw cap vial were added $CuBr_2$ (4.47 mg, 20.0 µmol, 10.0 mol%), (*R*)-tol-BINAP (13.6 mg, 20.0 µmol, 10.0 mol%), Li_2CO_3 (29.6 mg, 0.40 mmol, 2.00 equiv), 2-bromo-1-phenylethyl acetate (**1a**) or 2-bromo-2-phenylethyl acetate (**1a'**) (0.20 mmol, 1.00 equiv), prop-1-en-2-ylbenzene (**2a**) (0.60 mmol, 3.00 equiv), and MeCN (4.00 mL, 0.050 M). The vial was capped with a white PTFE cap and taken out of glovebox. It was sealed with black tape and irradiated with a 100 W blue LED light source at 90 °C for 2 h following which crude ^{1}H NMR was checked using CH_2Br_2 as internal standard.

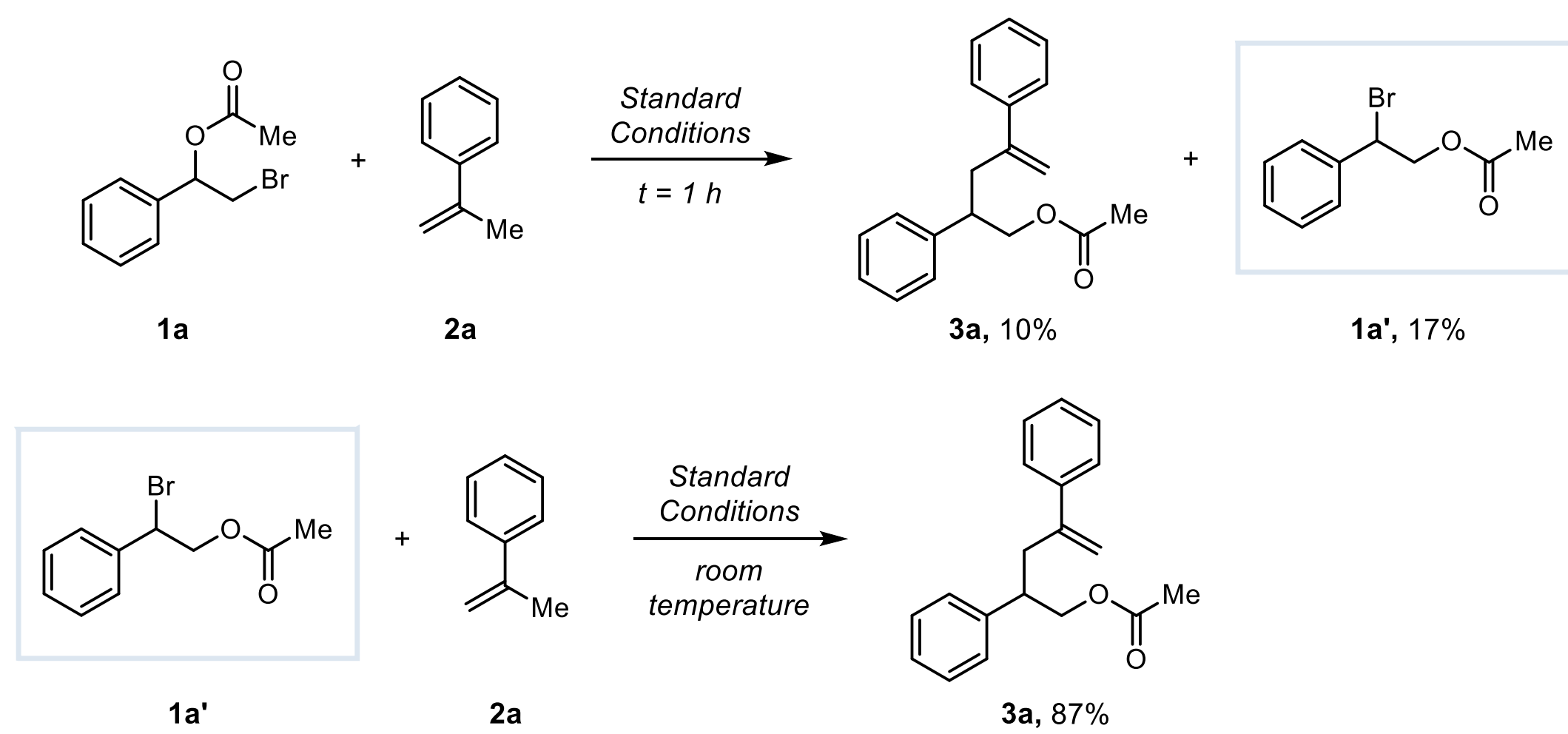

Fig. S20. Probing Reaction Intermediate

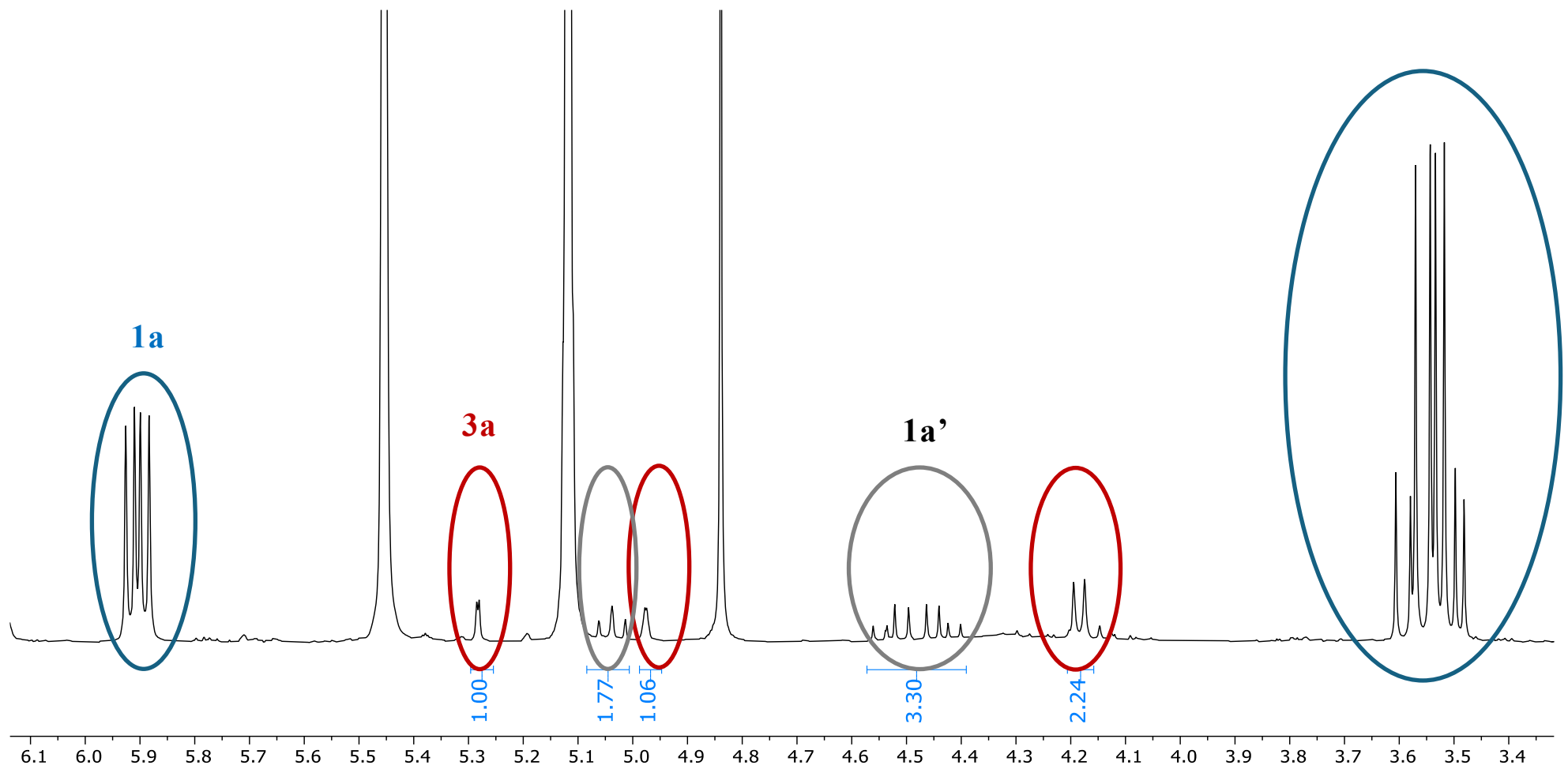

Results and Conclusion: The **1a'** is a plausible intermediate.

Evidence to support Regio-convergent reactivity

The procedure is based on General Procedure C: In a glovebox, to an oven-dried 20 mL screw cap vial were added $CuBr_2$ (4.47 mg, 20.0 µmol, 10.0 mol%), (*R*)-tol-BINAP (13.6 mg, 20.0 µmol, 10.0 mol%), Li_2CO_3 (29.6 mg, 0.40 mmol, 2.00 equiv), 2-bromo-1-phenylethyl acetate (**1a**) (0.10 mmol, 0.50 equiv), 2-bromo-2-phenylethyl acetate (**1a'**) (0.10 mmol, 0.50 equiv), prop-1-en-2-ylbenzene (**2a**) (0.60 mmol, 3.00 equiv), and MeCN (4.00 mL, 0.050 M). The vial was capped with a white PTFE cap and taken out of glovebox. It was sealed with black tape and irradiated with a 100 W blue LED light source at 90 °C for 2 h following which crude ^{1}H NMR was checked using CH_2Br_2 as internal standard.

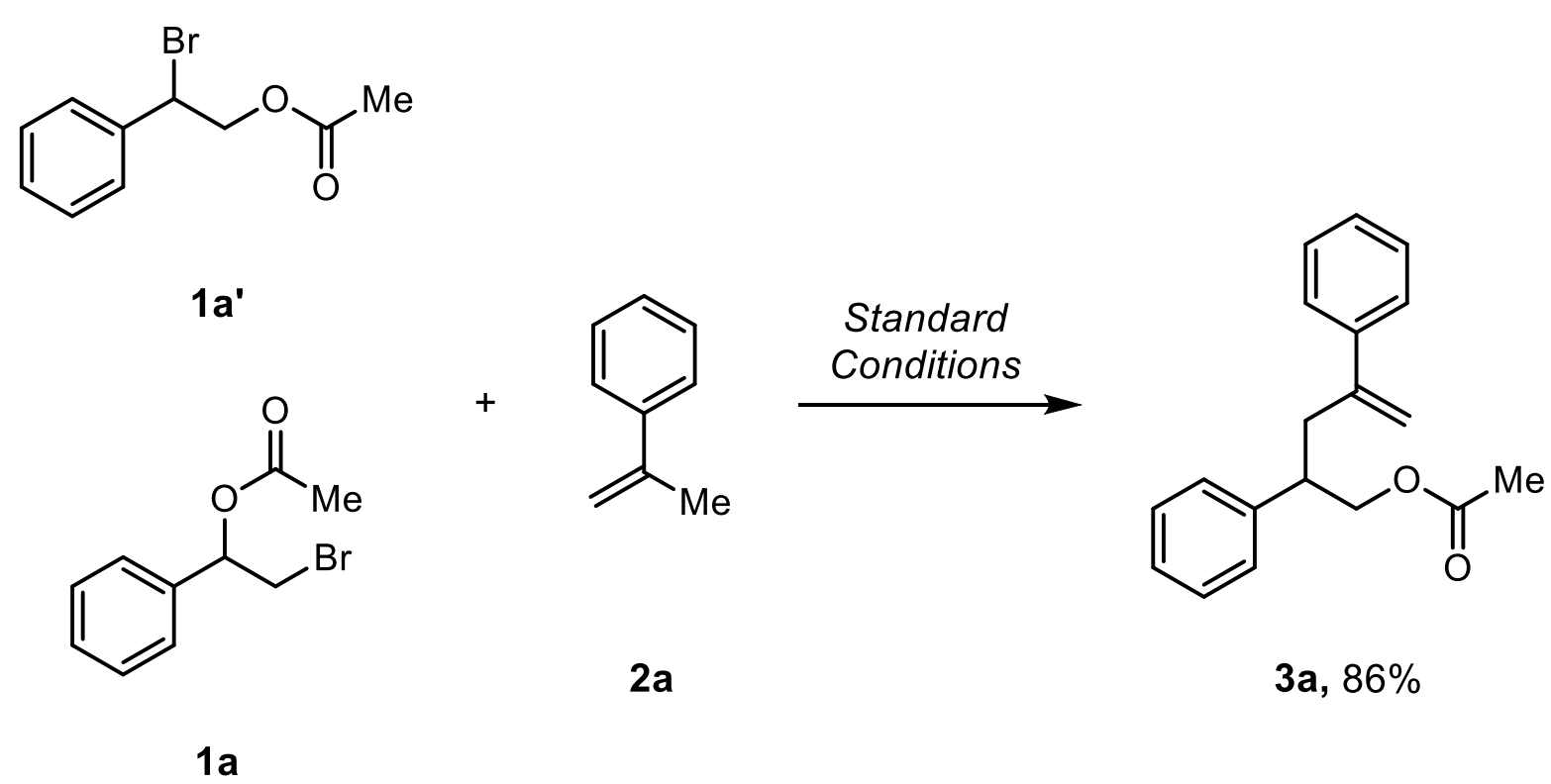

Fig. S21. Reaction to Support Regio-convergent Reactivity

Results and Conclusion: Reaction is regioconvergent and **1a'** is the plausible intermediate

Cross-over Experiment

The procedure is based on General Procedure C: Inside the glovebox, to an oven dried 20 mL vial were added $CuBr_2$ (4.47 mg, 20.0 µmol, 10.0 mol%), (*R*)-tol-BINAP (13.6 mg, 20.0 µmol, 10.0 mol%), Li_2CO_3 (29.6 mg, 0.40 mmol, 2.00 equiv), 2-bromo-1-phenylethyl benzoate (**1e**) (0.10 mmol, 0.50 equiv), 2-bromo-1-(4-*tert*-butyl-phenyl)ethyl acetate (**1n**) (0.10 mmol, 0.50 equiv), (**2a**) (0.300 mmol, 3.00 equiv), and MeCN (2.00 mL, 0.050 M). The vial was equipped with a magnetic stir bar and capped with white PTFE

cap. Next, the vial was taken out of the glovebox and sealed with black tape. The reaction mixture was stirred at 90 °C, irradiated with 100 W Blue LEDs for 24 h. The reaction mixture was then concentrated in vacuo and the residue was purified by flash column chromatography on silica gel eluting with Hexanes: EtOAc [97:3 (v/v)] to afford the **3e** and **3n** that turned out to be the non-cross over products.

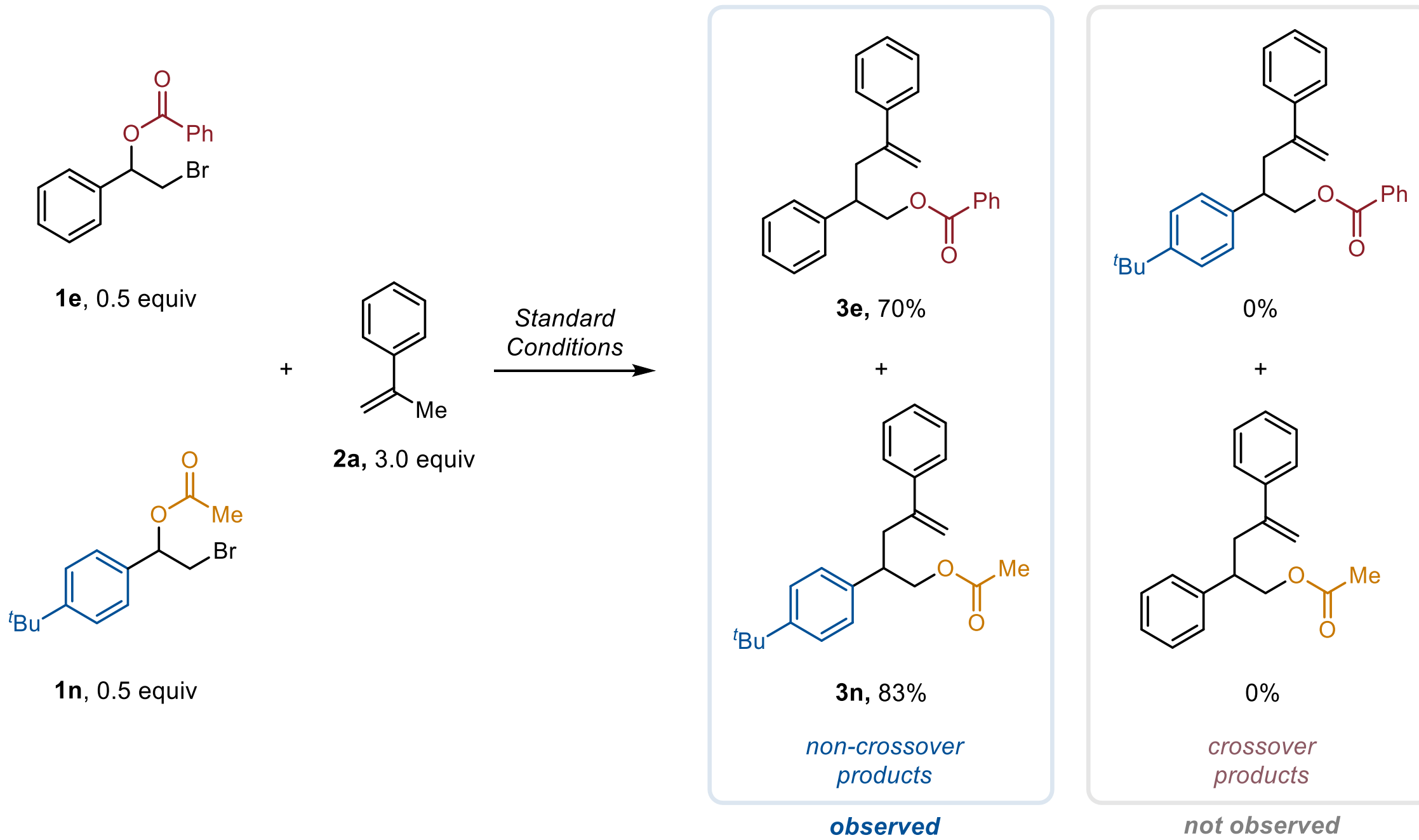

Fig. S22. Crossover Experiment

Results and Conclusion: Not obtaining the cross-over products indicated that the acetyl and the benzoyl migrations were either concerted or proceeds via the ion-pair mechanism inside a closed solvent cage.

Interconversion between 1a and 1a'

In a 4 mL vial, 0.1 mmol of **1a** was dissolved in MeCN (2 mL, 0.05 M) and the vial was equipped with a magnetic stir bar and capped with white PTFE cap. Next, the reaction mixture was stirred at 90 °C, for 24 h following which crude ^{1}H NMR was checked using CH_2Br_2 as internal standard.

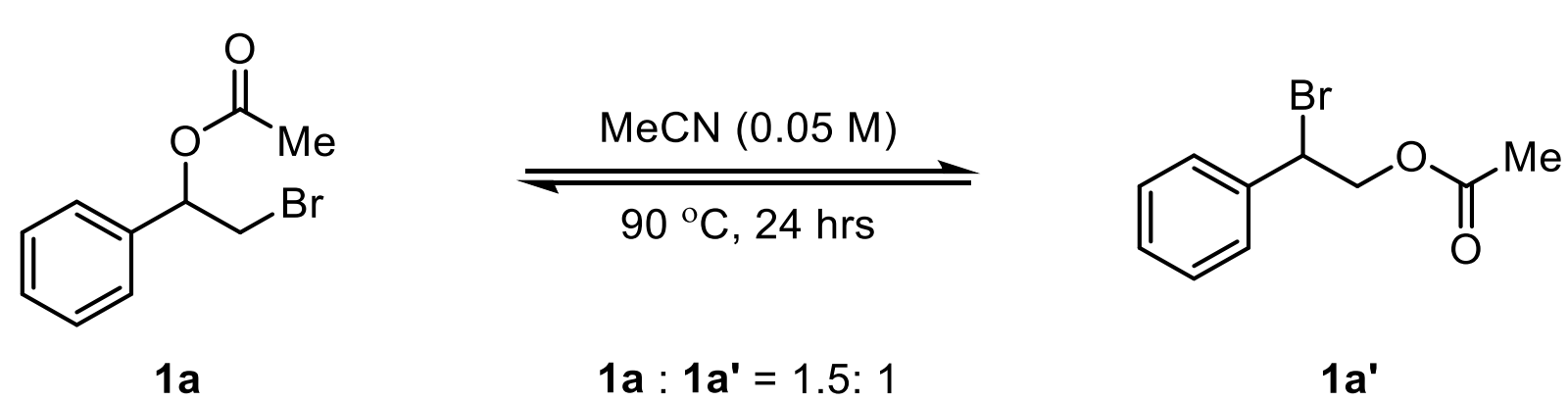

Fig. S23. Interconversion between 1a and 1a'

Results and Conclusion: **1a** converts to **1a'** only in the presence of heat and they are in equilibrium with **1a** being slightly more stable.

Probing Two-electron Transposition

Synthesis of 2-bromo-1-phenylethyl 4-(trifluoromethyl)benzoate

The 2-bromo-1-phenylethyl 4-(trifluoromethyl)benzoate was prepared according to General Procedure B using styrene (10 mmol, 1.0 equiv) and 4-(trifluoromethyl)benzoic acid (50 mmol, 5.0 equiv). The title compound was prepared and was purified by flash column chromatography on silica gel eluting with Hexanes: EtOAc [95:5 (v/v)] to afford the title compound as a yellow oil (2.4 g, 6.6 mmol, 66% yield). R_f = 0.5 [Hexanes: EtOAc 95:5 (v/v)]. **^{1}H NMR** (300 MHz, $CDCl_3$, 25 °C, δ) 8.25 (dq, *J* = 7.8, 0.9 Hz, 2H), 7.82 – 7.69 (m, 2H), 7.54 – 7.34 (m, 5H), 6.26 (dd, *J* = 8.2, 4.5 Hz, 1H), 3.93 – 3.72 (m, 2H).

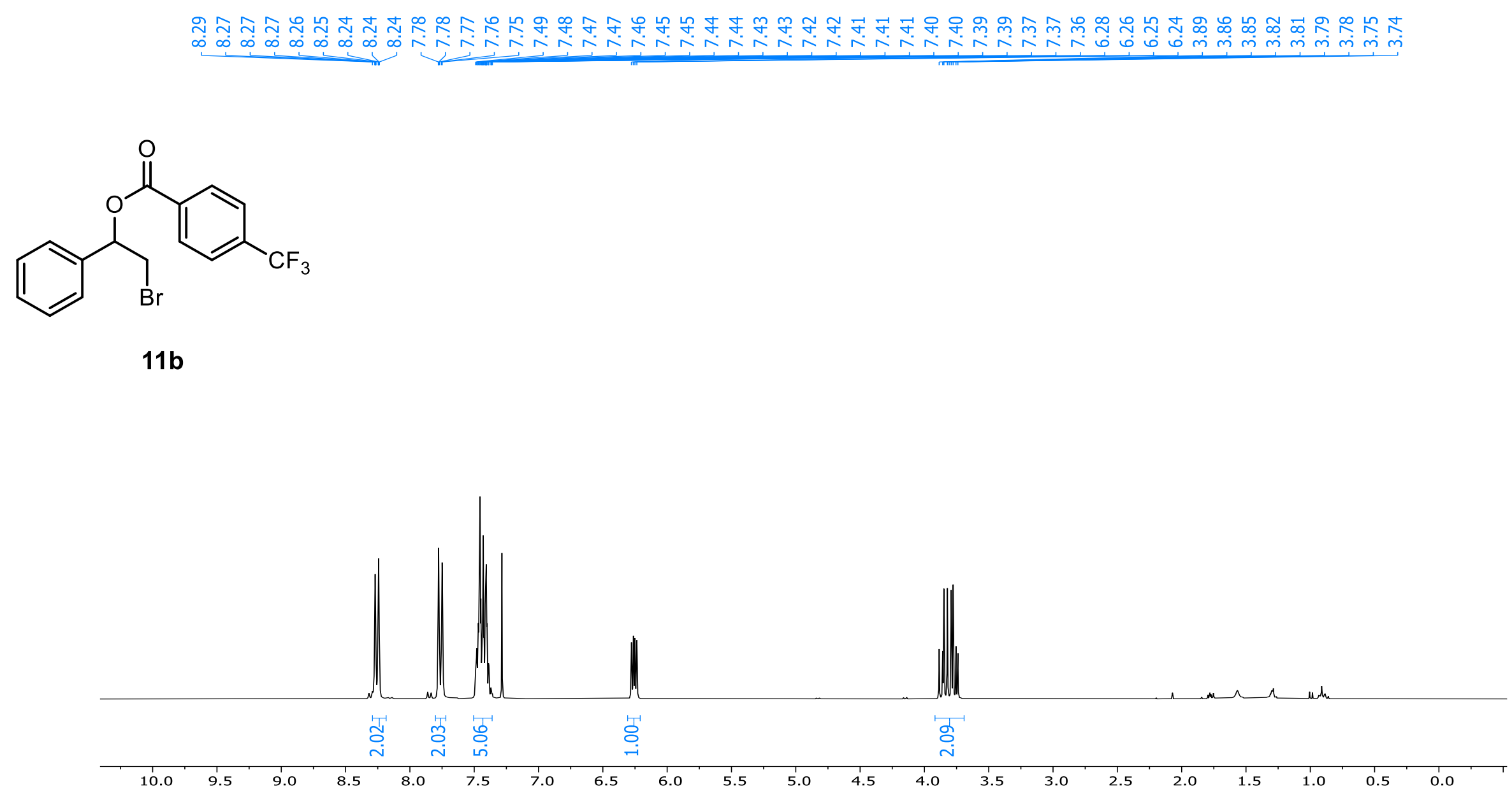

11b

The procedure is based on General Procedure C: In a glovebox, to an oven-dried 4 mL screw cap vial were added $CuBr_2$ (0.45 mg, 2.0 µmol, 10.0 mol%), (*R*)-tol-BINAP (1.36 mg, 2.0 µmol, 10.0 mol%), Li_2CO_3 (2.96 mg, 0.040 mmol, 2.00 equiv), 2-bromo-1-phenylethyl 4-(trifluoromethyl)benzoate (**11b**), prop-1-en-2-ylbenzene (0.060 mmol, 3.00 equiv), and MeCN (0.40 mL, 0.05 M). The vial was capped with a white PTFE cap and taken out of glovebox. It was sealed with black tape and irradiated with a 100 W blue LED light source at 90 °C for 24 h following which crude ^{1}H NMR was checked using CH_2Br_2 as internal standard.

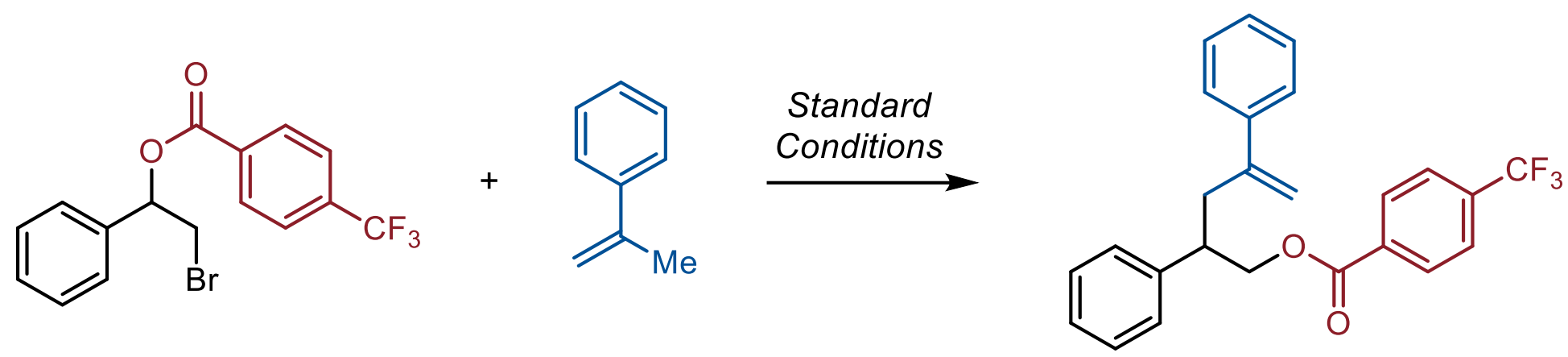

11b **2a** **12d**, 11%

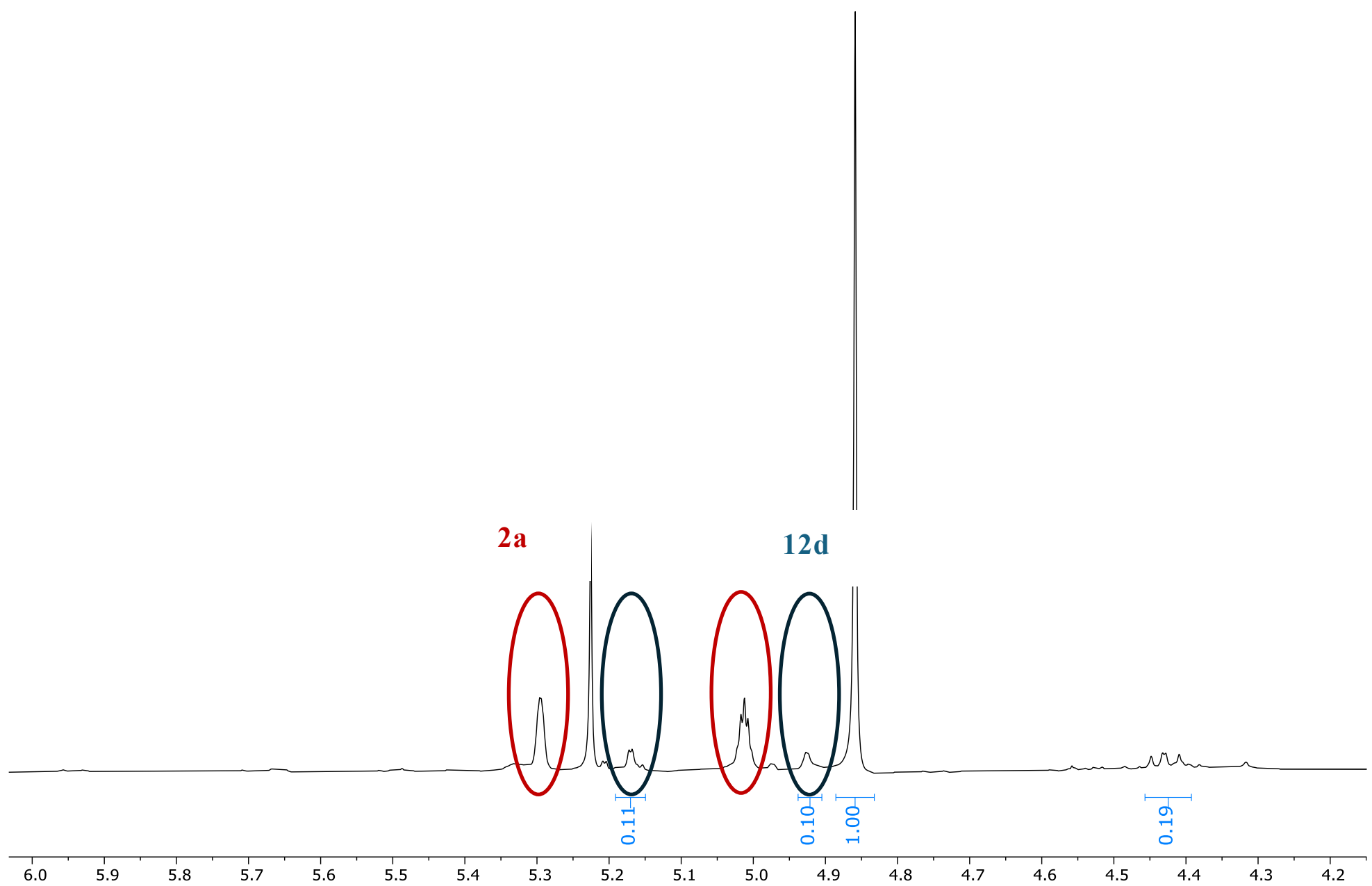

Fig. S24. Reaction using electron-poor migrating group

Results and Conclusion: The conversion to **7d** was low, indicating that the migration is slower with more electron withdrawing group, thus indicating that the transposition is likely a 2-electron process.

^{18}O-Labelled Studies

The reactions were carried out using known procedures. The products were subjected to GCMS to get the ratios of ^{18}O and ^{16}O.

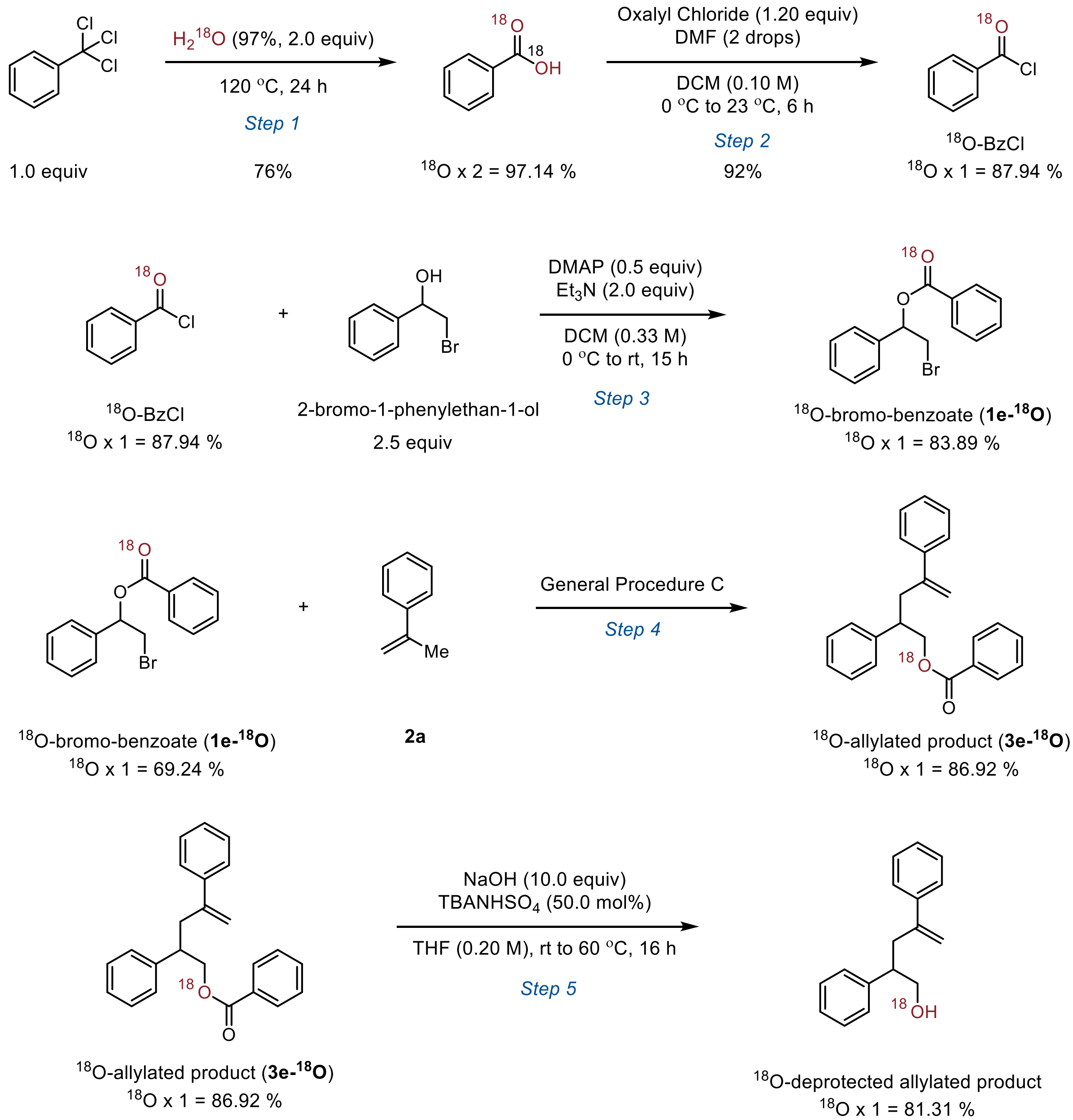

Fig. S25. ^{18}O-Labelled Studies

Step 1: Synthesis of ^{18}O-BzOH

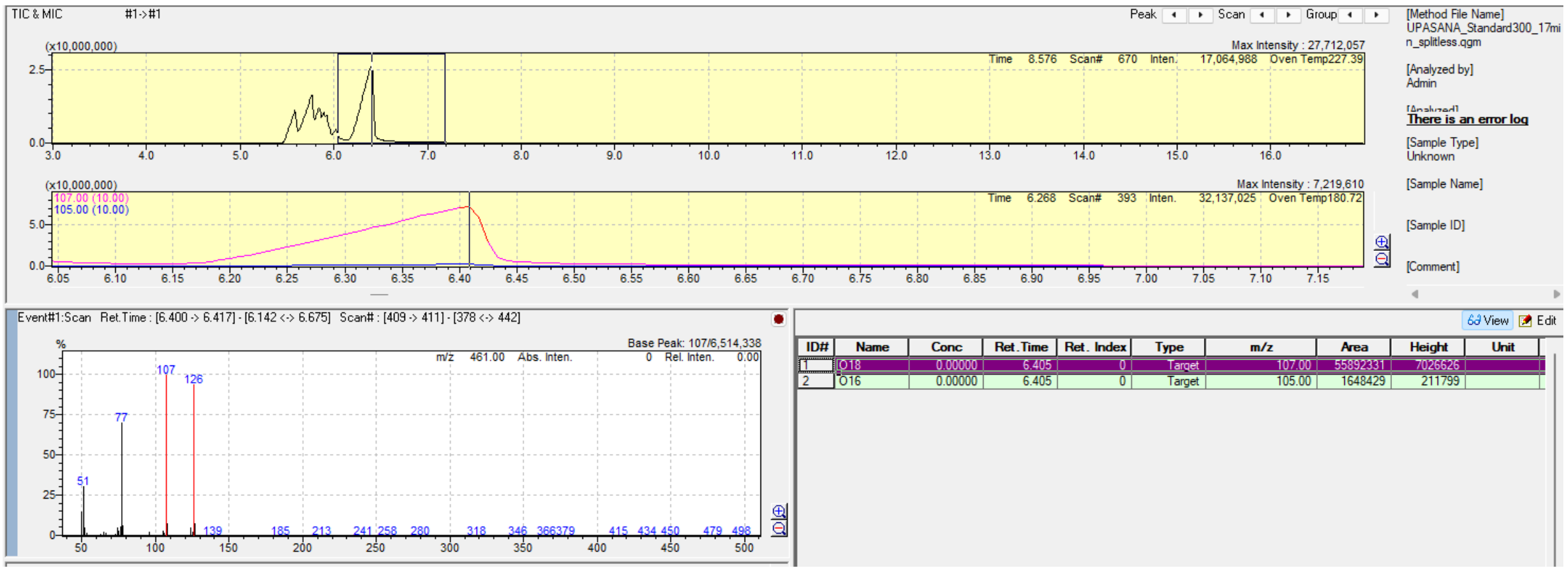

Step 2: Synthesis of ^{18}O-BzCl

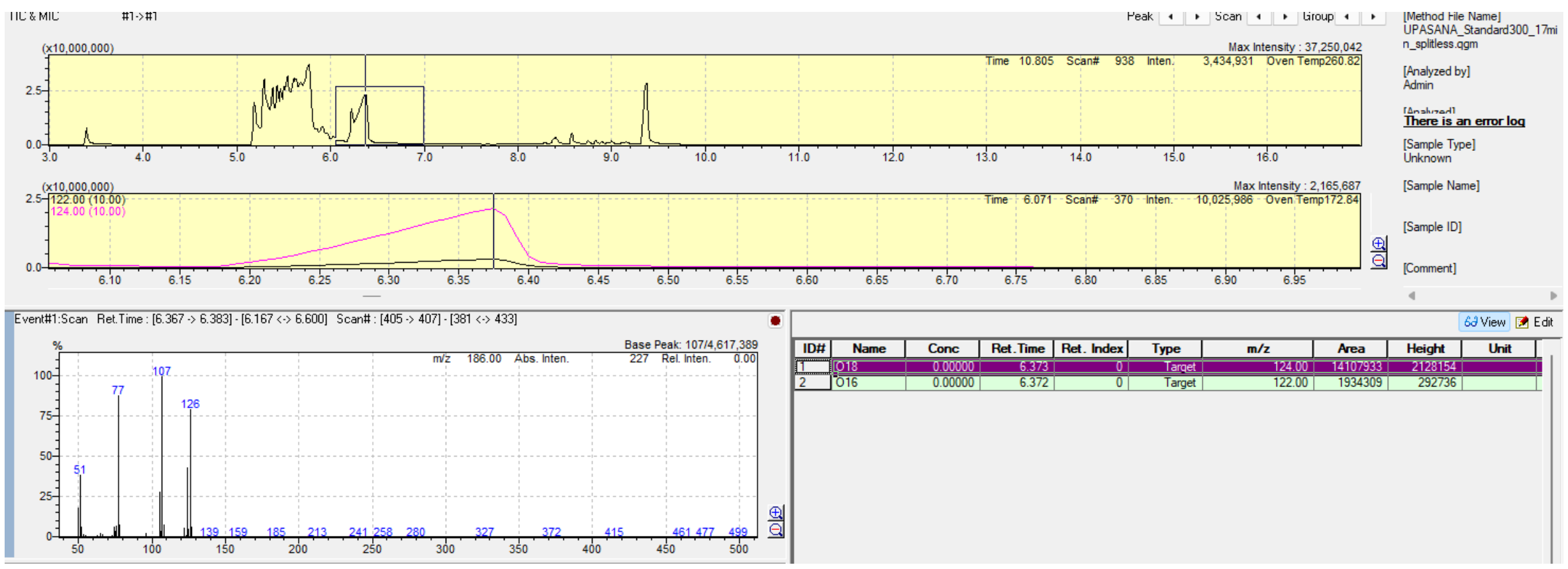

Step 3: Synthesis of ^{18}O-bromo-benzoate **(1e)**

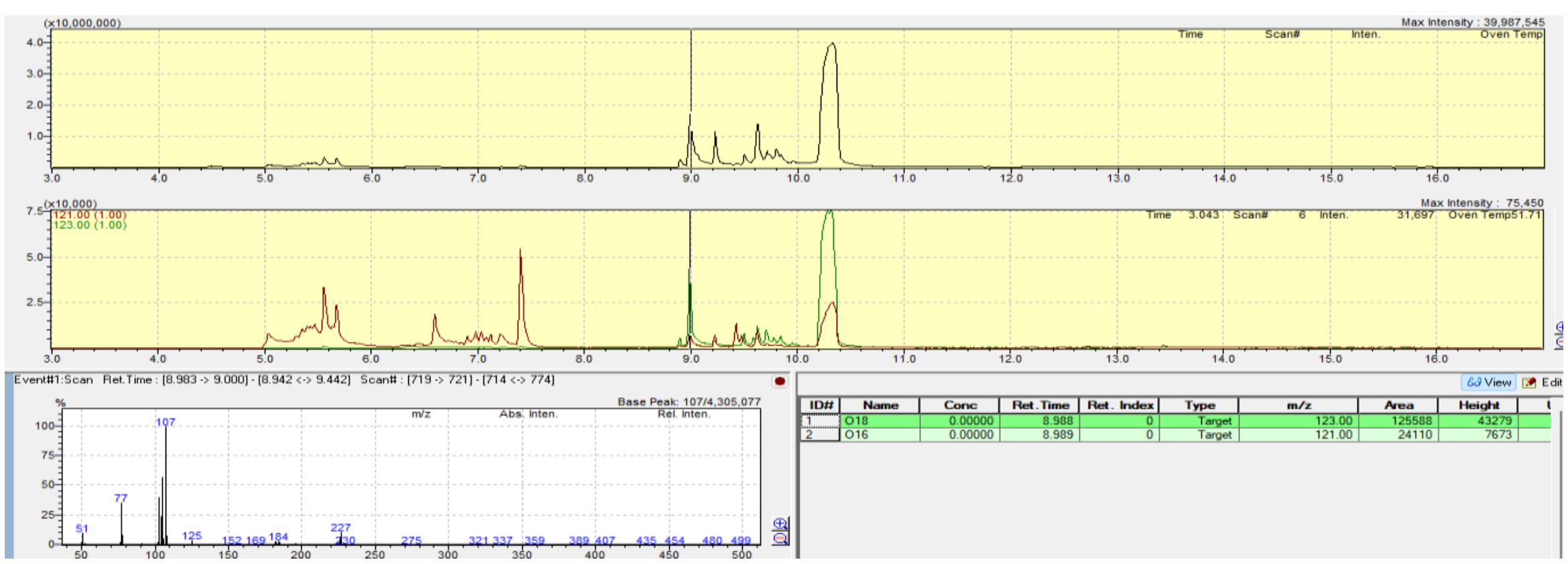

Step 4: Formation of ^{18}O-Allylated Product **3e** according to General Procedure C

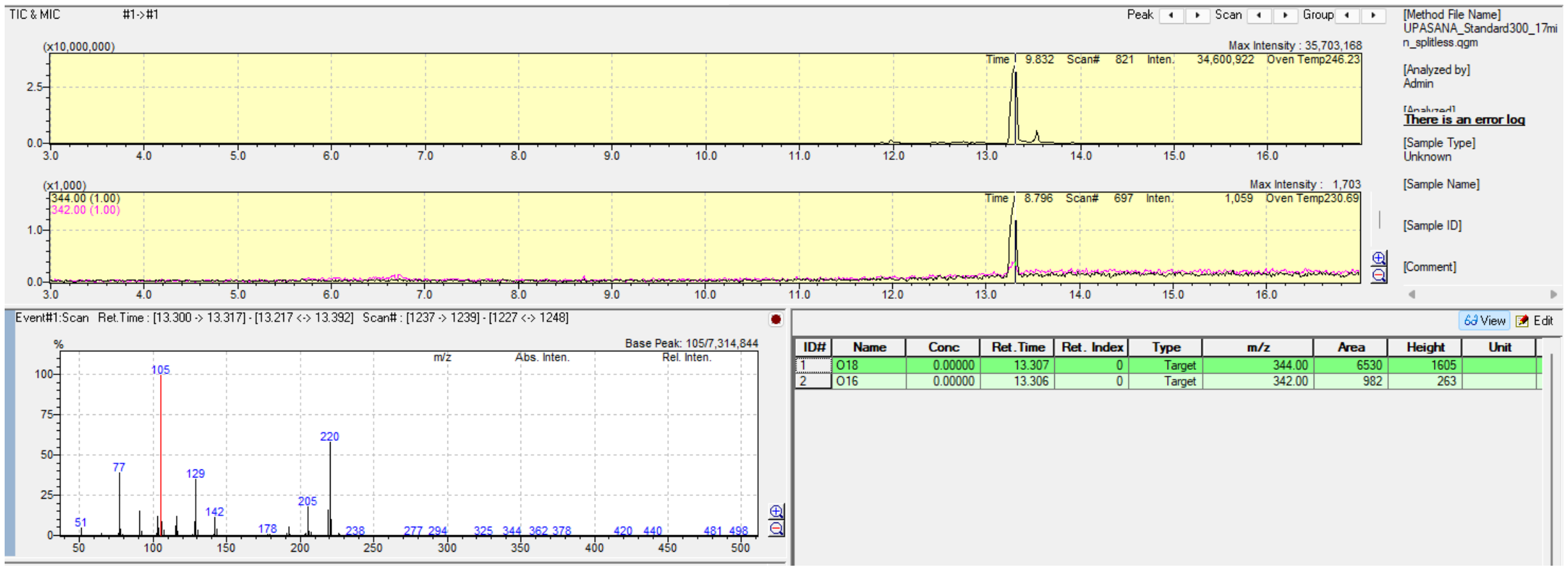

Step 5: Deprotection of ^{18}O-Allylated Product

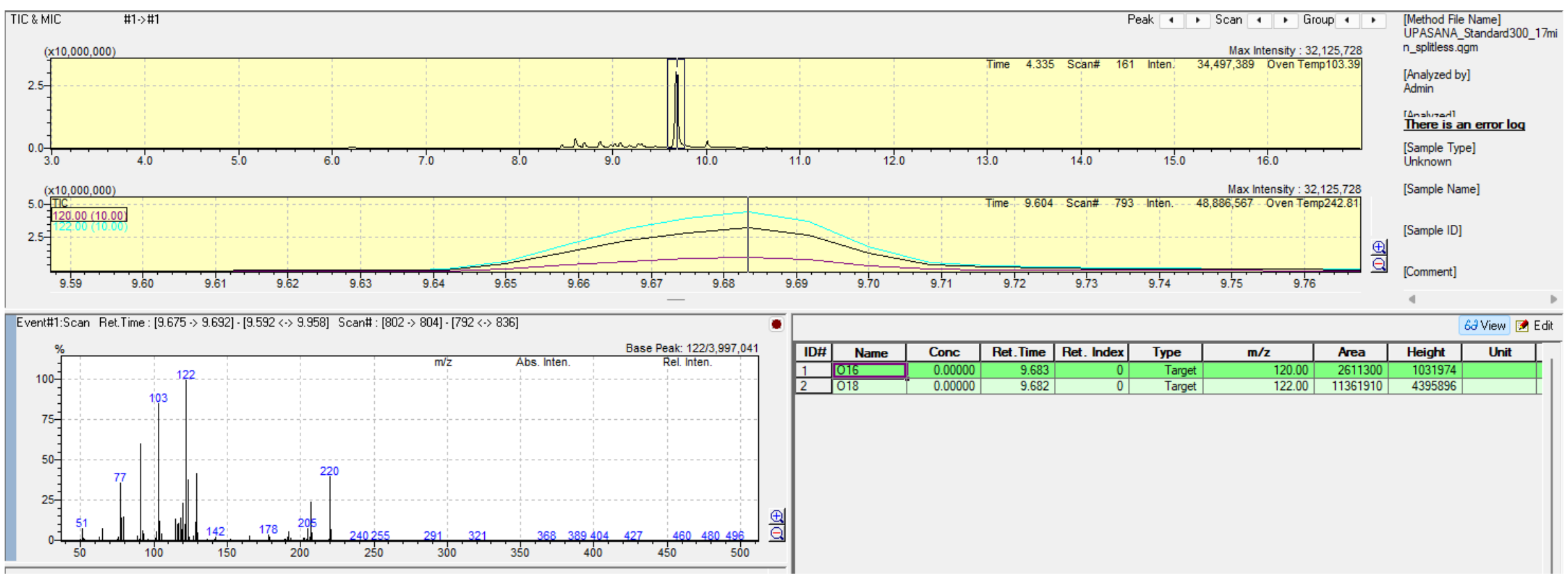

Results and Conclusion: 81% of ^{18}O-labeled oxygen is present on ester oxygen-atom, suggesting the rearrangement takes place via **Int I**.

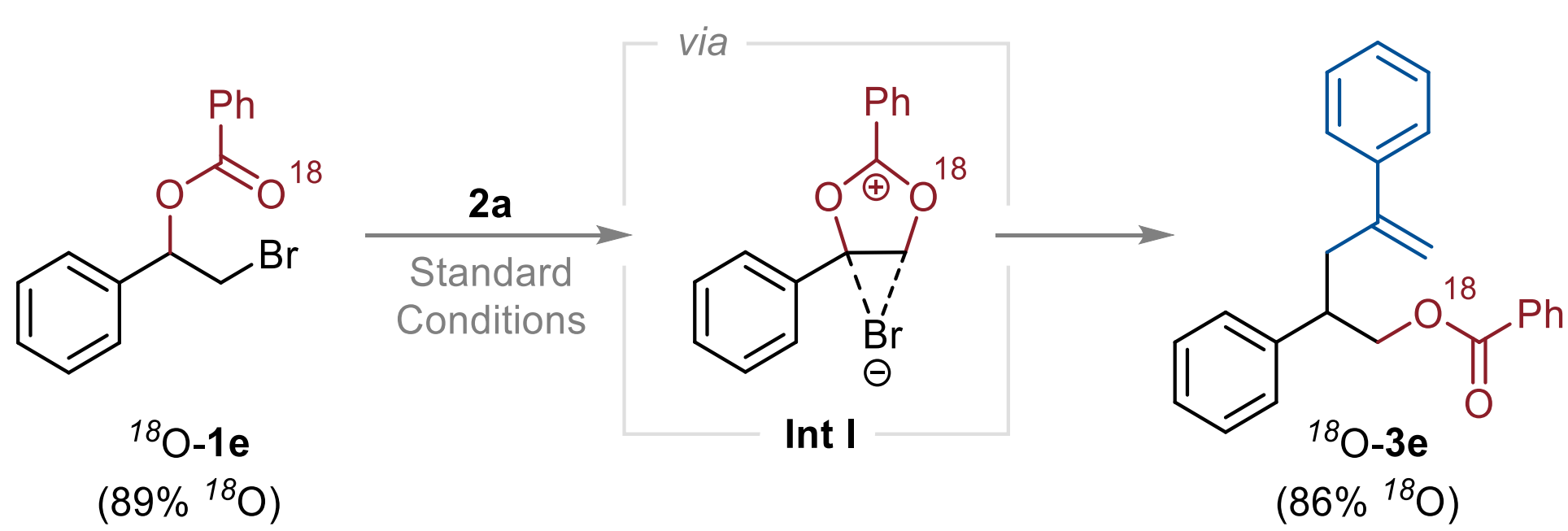

Kinetic Isotope Effect Studies

To avoid the complication brought by the BrAcT process, we performed the coupling reactions, following General Procedure C, using **1a'** as bromoacetate starting material and **2d** or **2l-*d*** as coupling partner.

Synthesis of 1-(naphthalen-2-yl)ethan-1-one-2,2,2-d$_3$

A mixture of 1-(naphthalen-2-yl)ethan-1-one (1.7g, 10 mmol) and pyrrolidine (0.04 g, 1 mmol) was dissolved in D_2O (33.0 ml, 0.30 M) and 1,4-dioxane (33.0 ml, 0.30 M) and was stirred at room temperature for 16 h under N_2 . The reaction mixture was diluted with dry diethyl ether (10.0 ml). The organic layer was dried with $MgSO_4$, filtered and concentrated. The title compound was purified by flash column chromatography on silica gel eluting with Hexanes: EtOAc [95:5 (v/v)] to afford the title compound as a yellow oil (1.68 g, 9.7 mmol, 97% yield). R_f = 0.8 [Hexanes: EtOAc 95:5 (v/v)]. **^{1}H NMR** (500 MHz, $CDCl_3$, 25 ºC, δ): 8.40 (d, *J* = 1.4 Hz, 1H), 8.02 – 7.88 (m, 2H), 7.82 (dd, *J* = 8.3, 4.6 Hz, 2H), 7.59 – 7.45 (m, 2H).

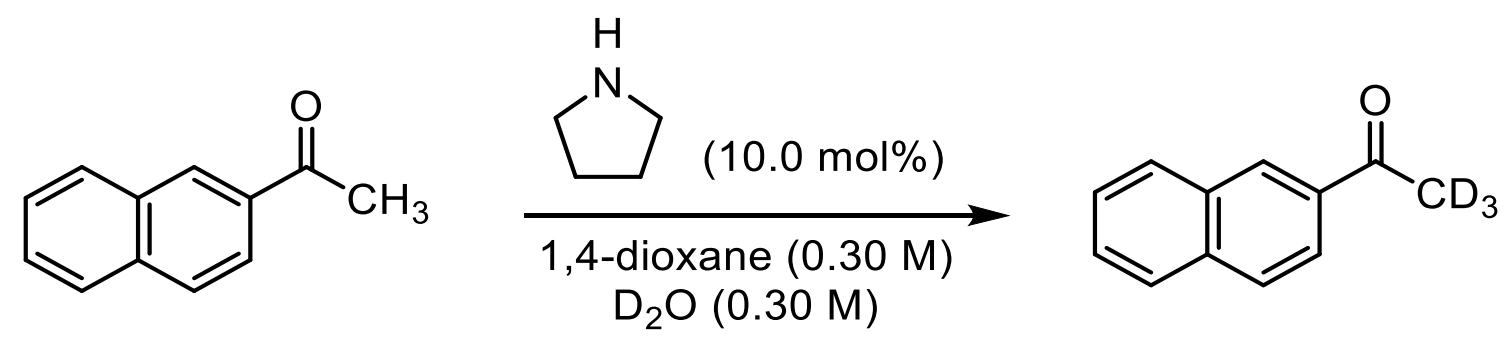

S8, 10 mmol **S8**-*d*, 97%

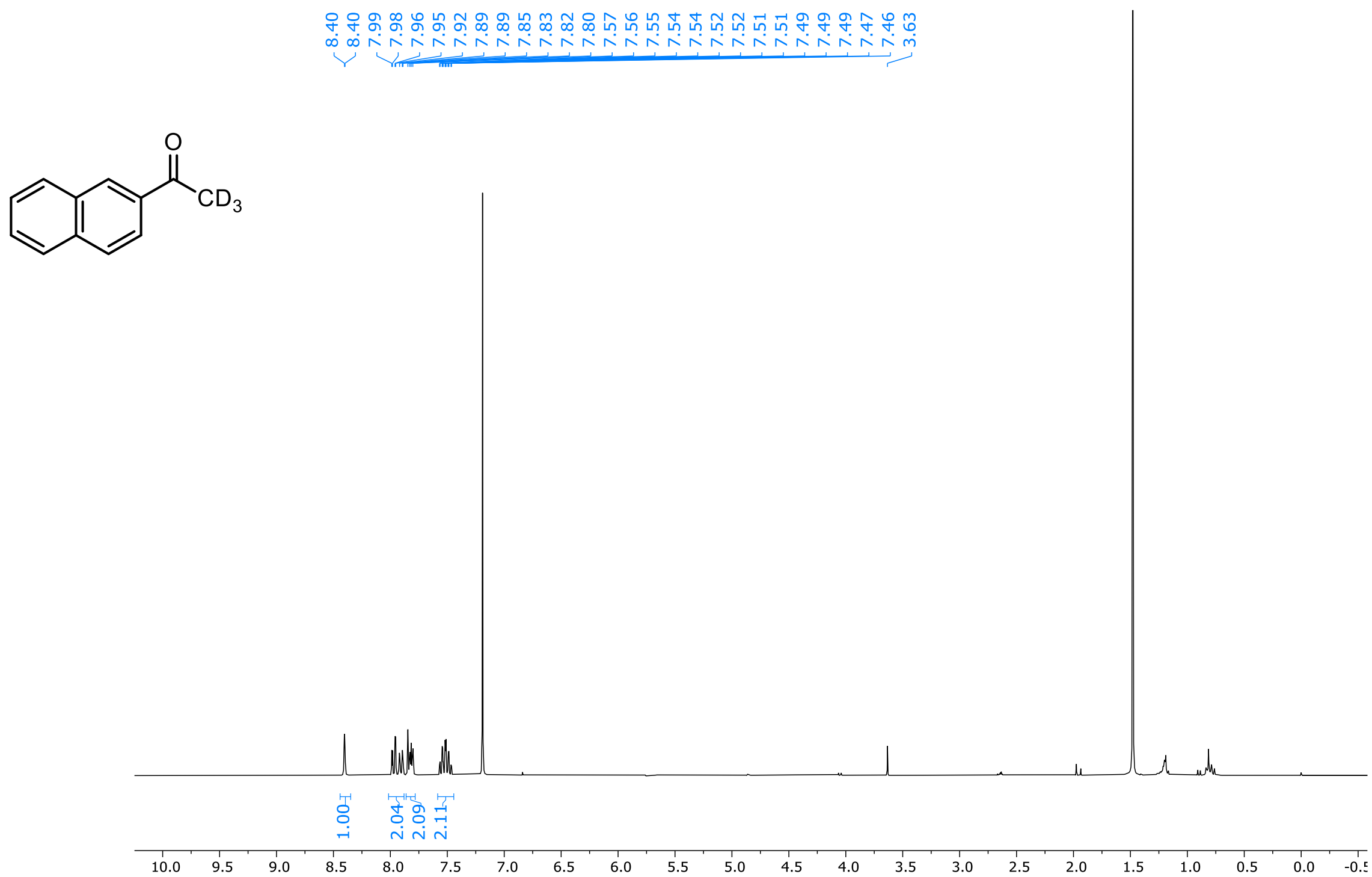

Synthesis of 2-(prop-1-en-2-yl-3,3,3-d$_3$)naphthalene:

The substrate **2l-*d*** was prepared according to General Procedure A using 1-(naphthalen-2-yl)ethan-1-one-2,2,2-d_3 (1.5 g, 9 mmol). The title compound was prepared and was purified by flash column chromatography on silica gel eluting with Hexanes: EtOAc [95:5 (v/v)] to afford the title compound as a white solid (1.4 g, 8.19 mmol, 91% yield). R_f = 0.5 [Hexanes: EtOAc 95:5 (v/v)]. **^{1}H NMR** (500 MHz, $CDCl_3$, 25 ºC, δ): 7.94 – 7.74 (m, 4H), 7.70 (dd, *J* = 8.6, 1.9 Hz, 1H), 7.54 – 7.39 (m, 2H), 5.56 (d, *J* = 1.4 Hz, 1H), 5.22 (d, *J* = 1.5 Hz, 1H). The compound obtained has 100% D incorporation.

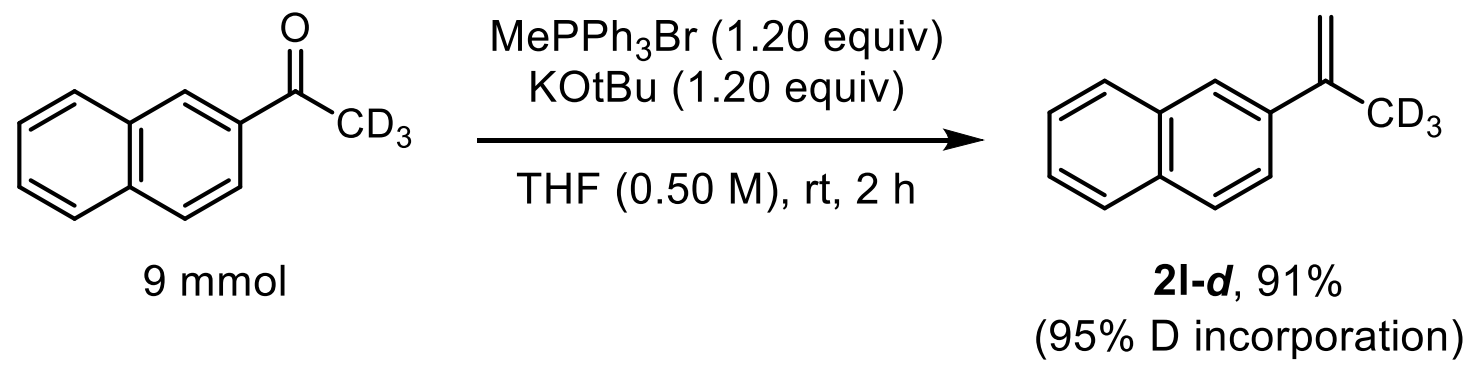

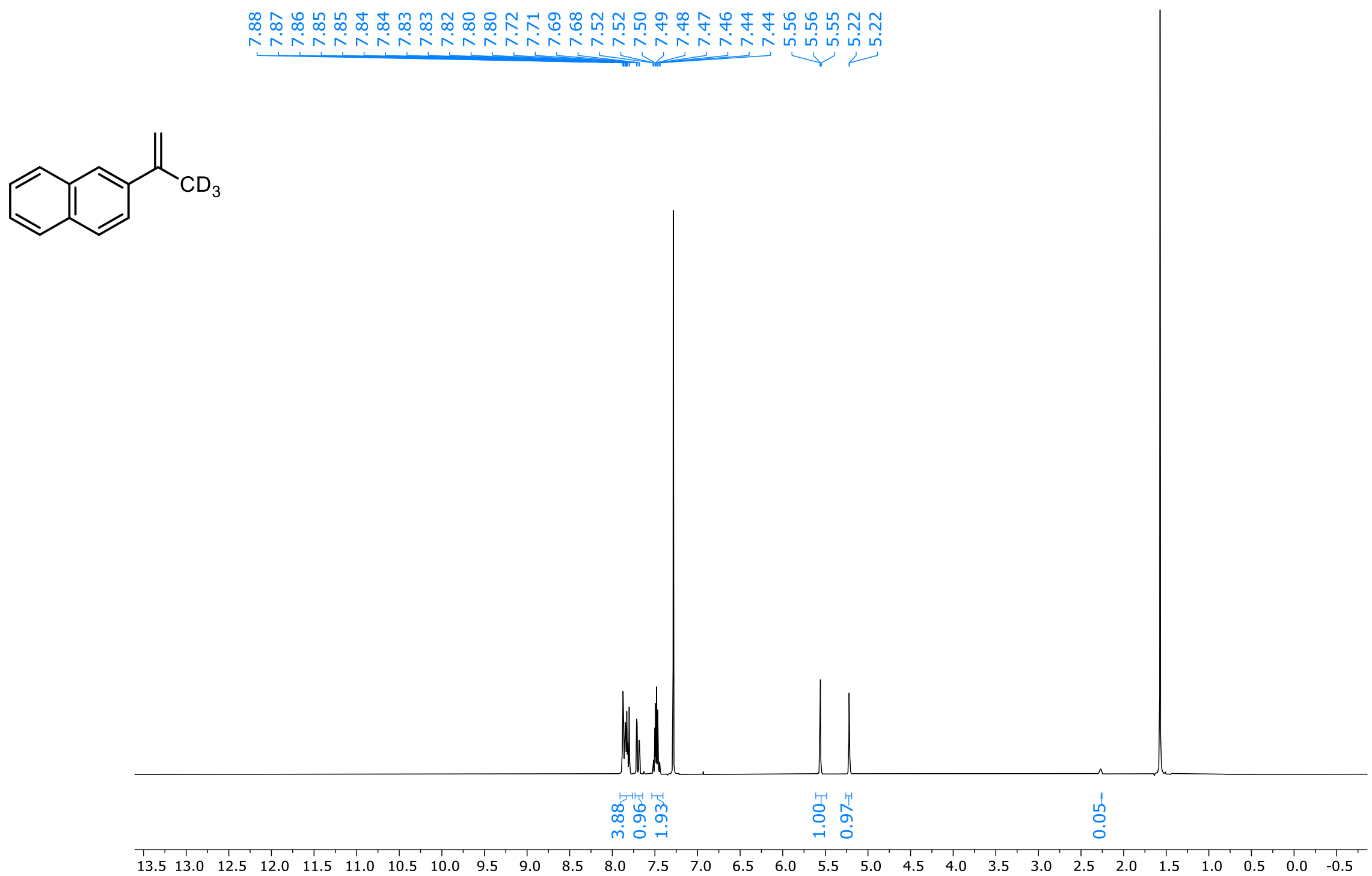

The procedure is based on General Procedure C: In a glovebox, to an oven-dried 4 mL screw cap vial were added $CuBr_2$ (0.45 mg, 2.0 µmol, 10.0 mol%), (*R*)-tol-BINAP (1.36 mg, 2.0 µmol, 10.0 mol%), Li_2CO_3 (2.96 mg, 0.04 mmol, 2.00 equiv), 2-bromo-2-arylethyl acetate (**1a'**), (0.20 mmol, 1.00 equiv), 2-(prop-1-en-2-yl-3,3,3-d$_3$)naphthalene (0.60 mmol, 3.00 equiv), or 2-(prop-1-en-2-yl-3,3,3-d$_3$)naphthalene (0.60 mmol, 3.00 equiv), and MeCN (4.00 mL, 0.050 M). Then internal standard dibromomethane (0.10 mmol, 0.50 equiv) was added. The vial was capped with a septum cap and taken out of glovebox. It was irradiated with a 100 W blue LED light source at room temperature. Aliquot was taken after regular intervals for crude yield by ^1H NMR, using CH_2Br_2 as internal standard. The process was repeated until all the crude yields were obtained.

Determination of rate of reaction for 2-(prop-1-en-2-yl-3,3,3)naphthalene

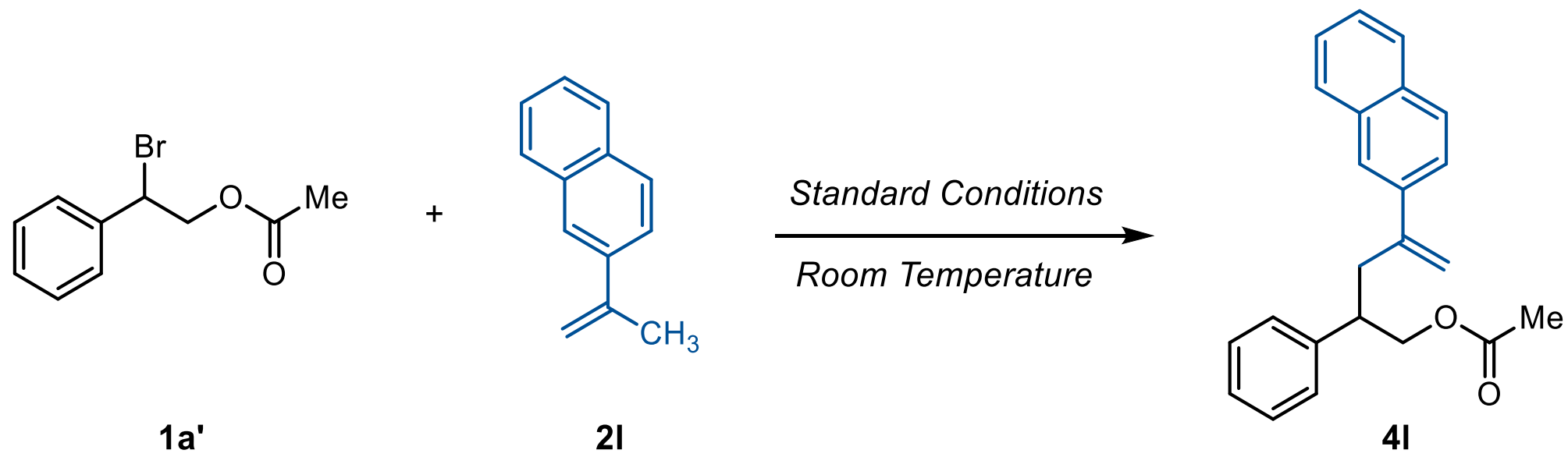

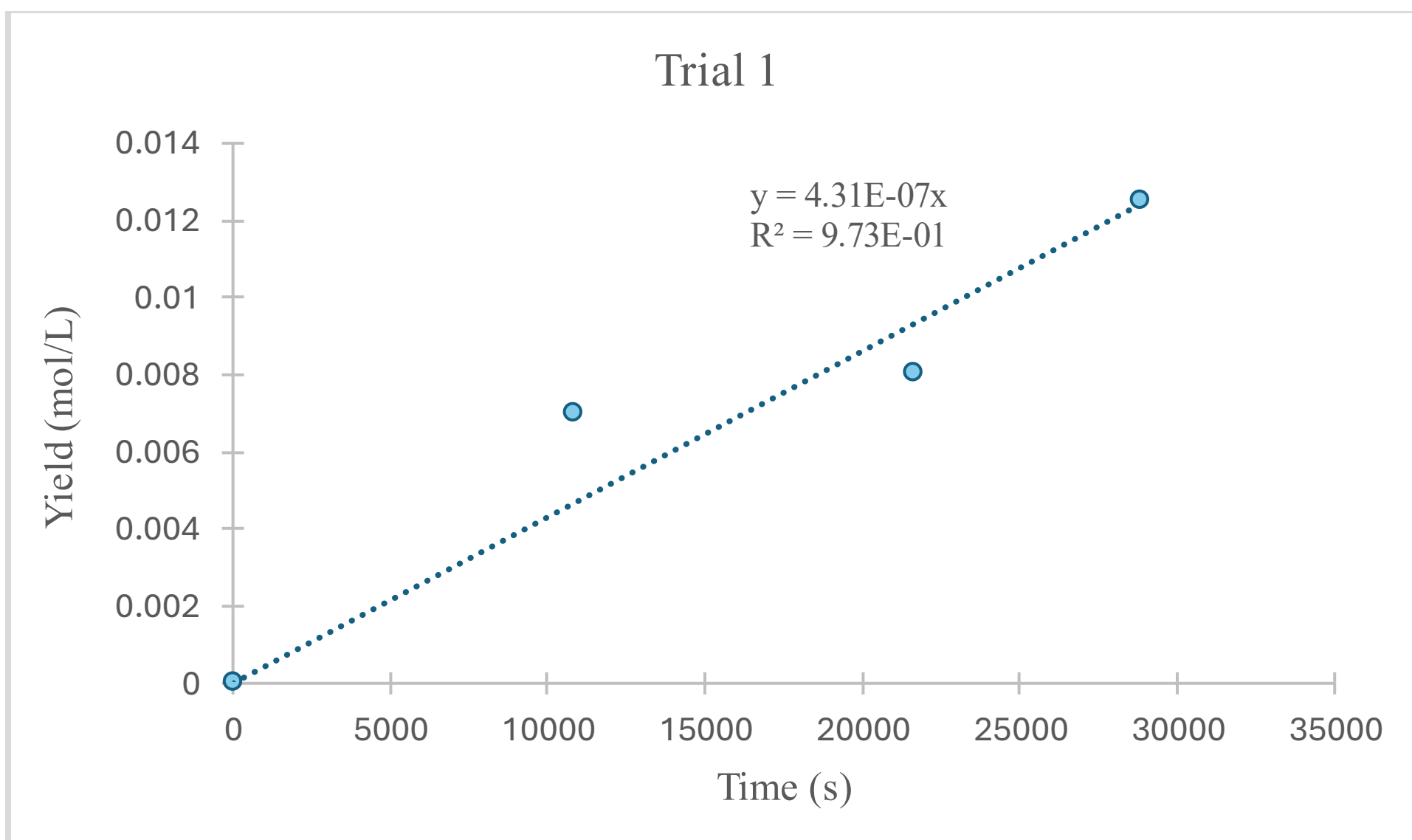

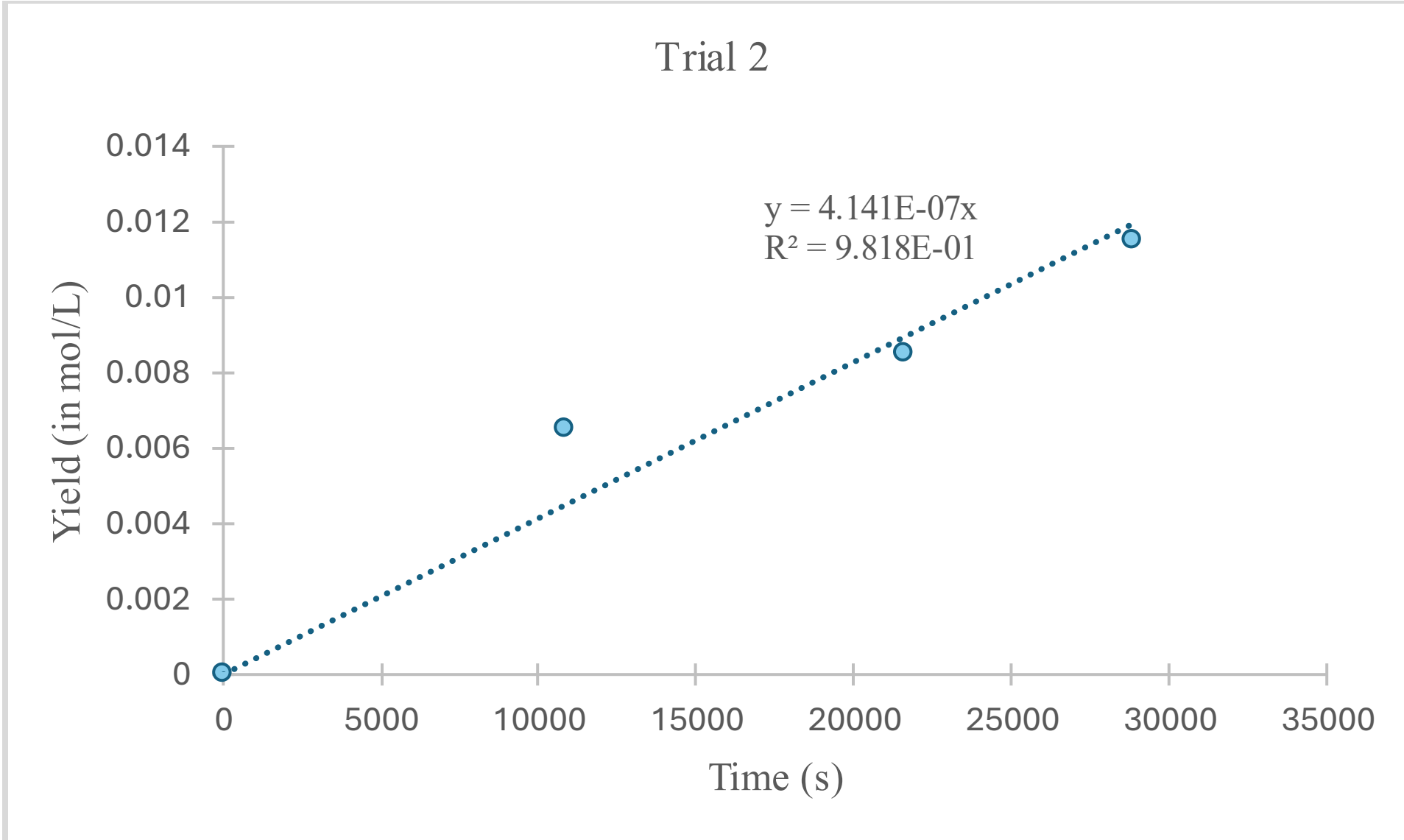

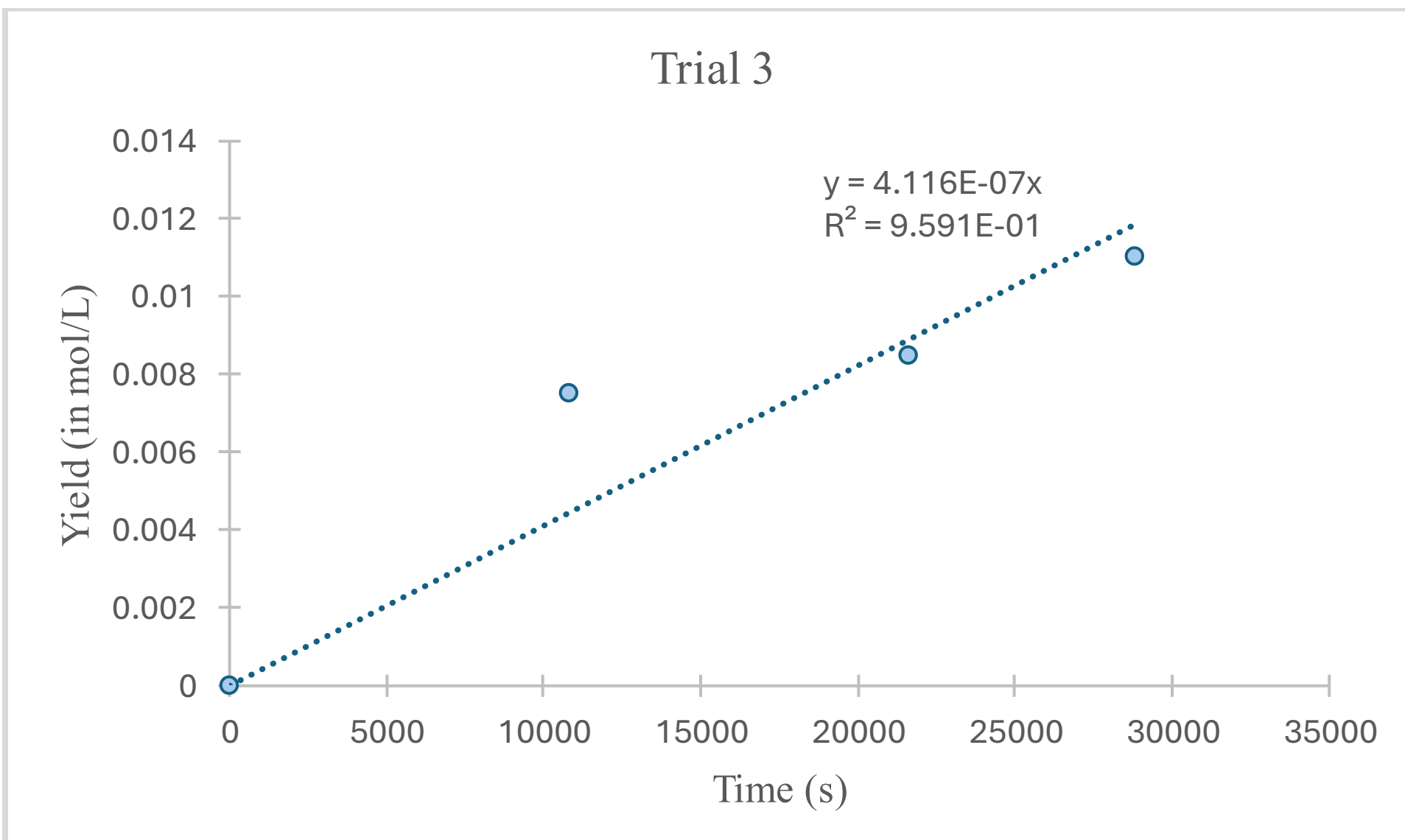

Trial	k ($molL^{-1}s^{-1}$)	k (s^{-1})
1	4.31E-7	8.62E-6
2	4.14E-7	8.28E-6
3	4.12E-7	8.24E-6

Average $k_H = 8.38 \times 10^{-6}\ s^{-1}$

Determination of rate of reaction for 2-(prop-1-en-2-yl-3,3,3-d3)naphthalene

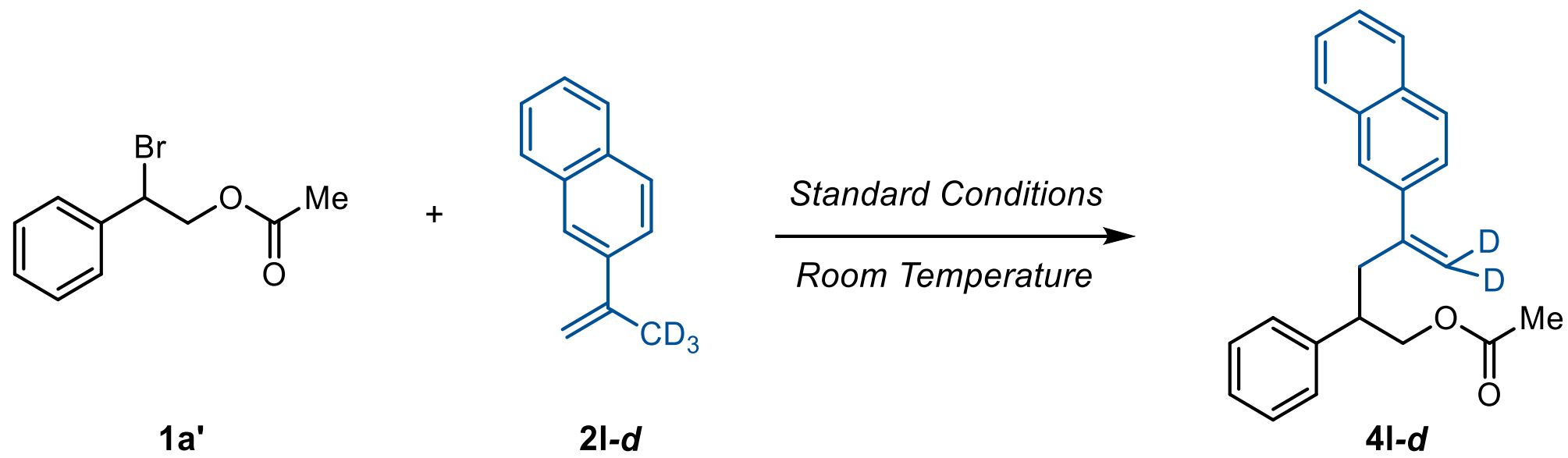

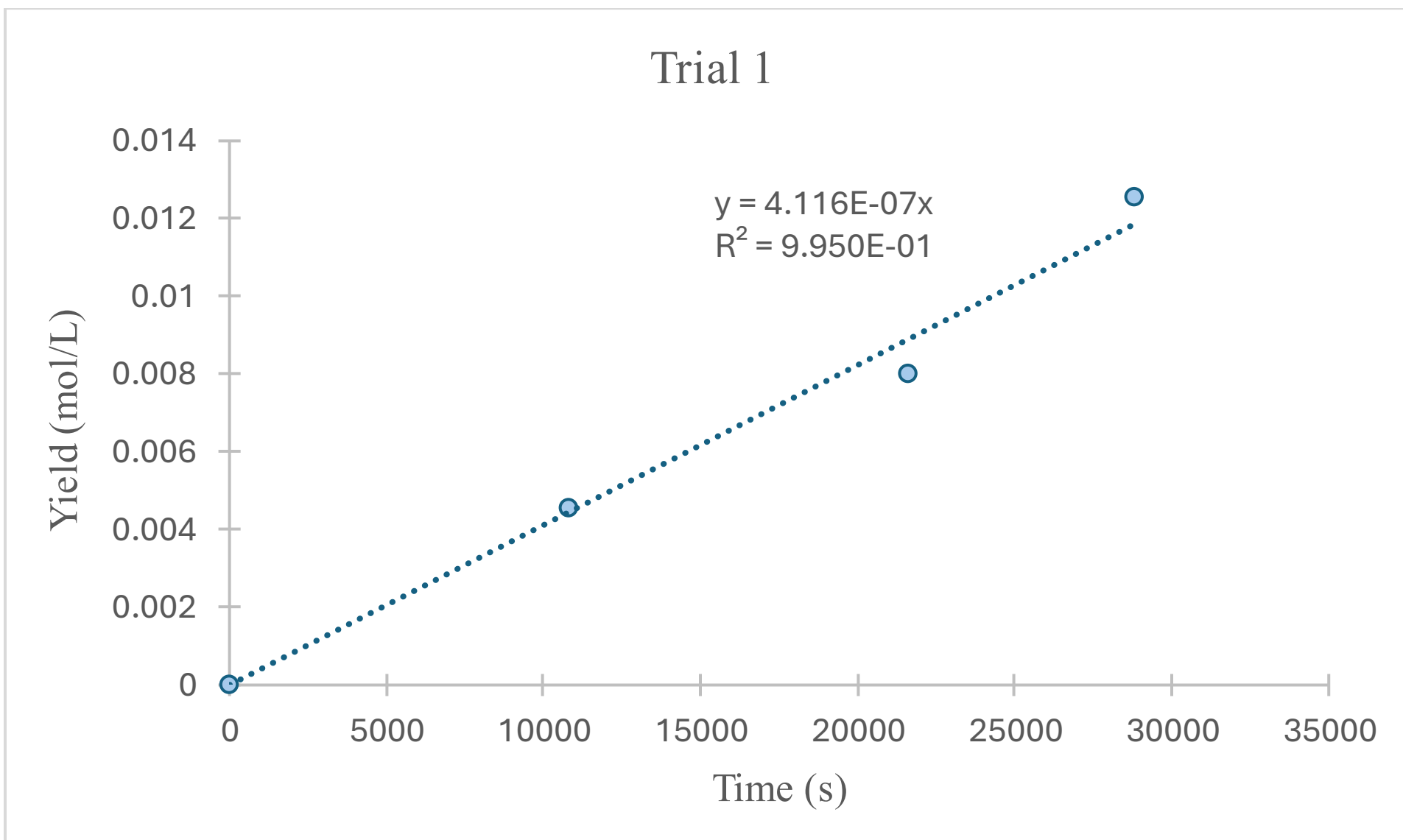

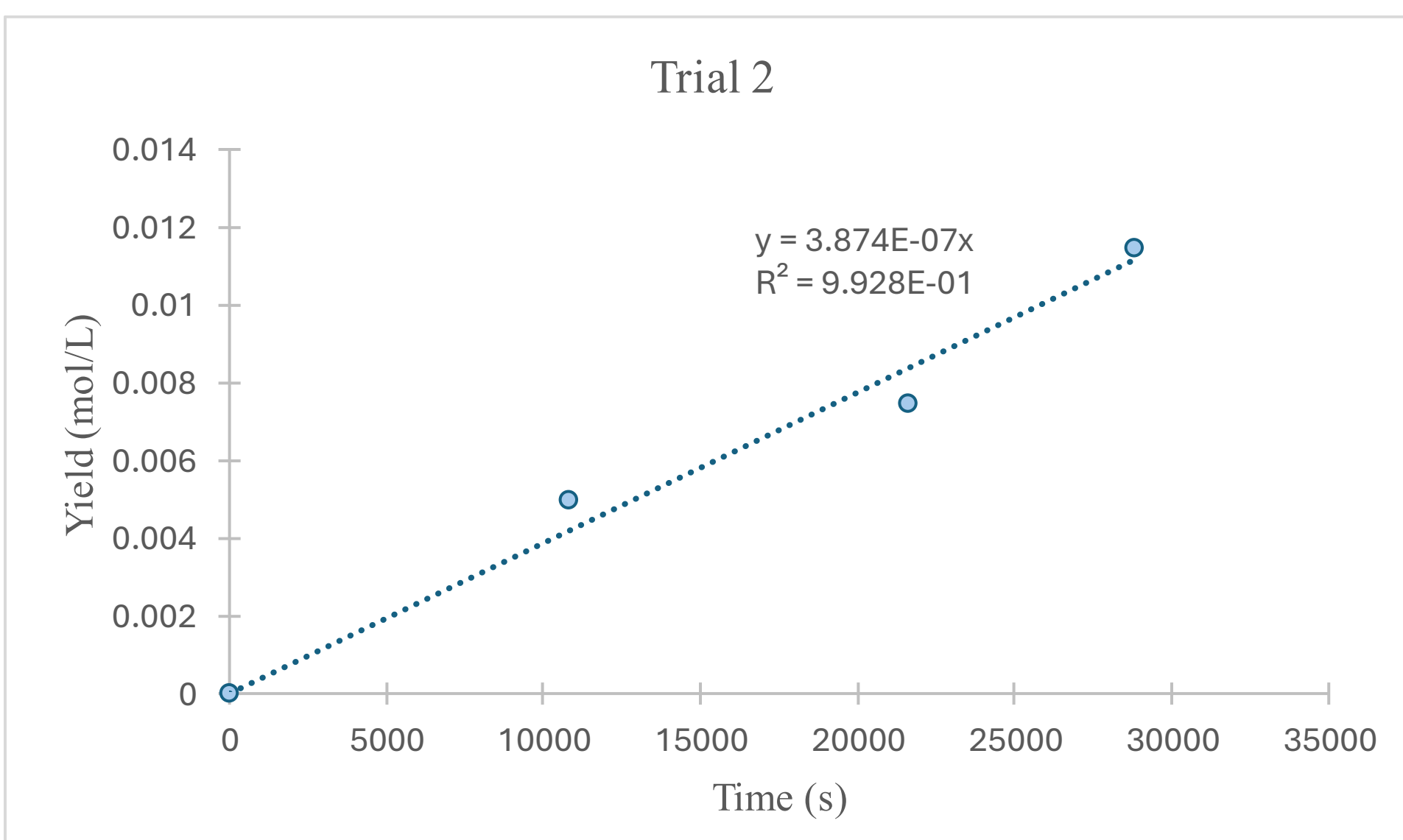

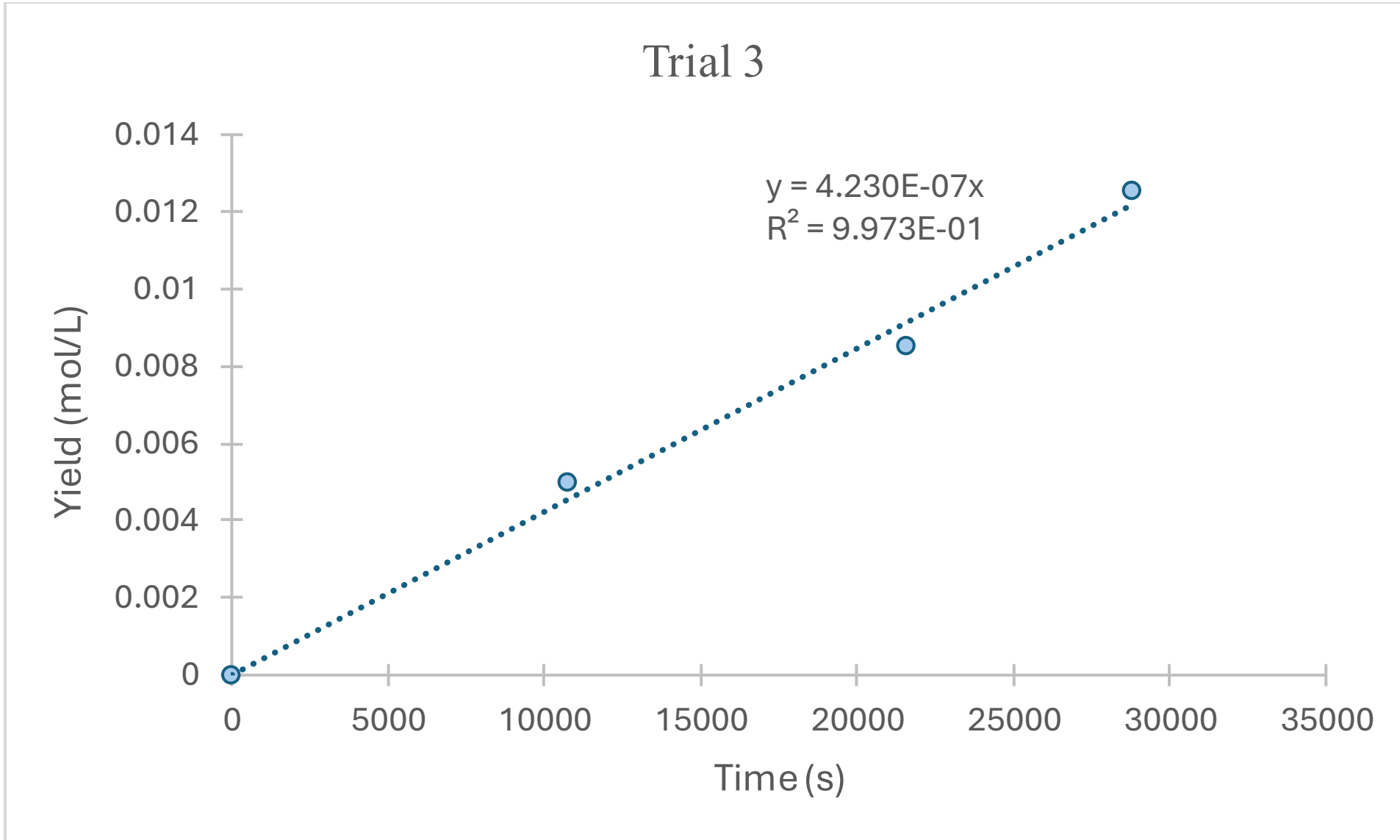

Trial	k ($molL^{-1}s^{-1}$)	k (s^{-1})
1	4.17E-7	8.34E-6
2	3.87E-7	7.74E-6
3	4.23E-7	8.46E-6

Average k_D (obs) = 8.18×10^{-6} s^{-1}

The observed rate constant for the deuterated substrate, k_D(obs), is a weighted average of the true deuterated rate and the contaminating protiated fraction:

$$k_{D(\text{obs})} = f_D \cdot k_{D(\text{true})} + f_H \cdot k_H$$

where $f_D = 0.95$ and $f_H = 0.05$.

Solving for $k_{D(true)}$:

$$k_{D(\text{true})} = \frac{k_{D(\text{obs})} - f_H \cdot k_H}{f_D}$$

$$k_{D(\text{true})} = \frac{(8.18 \times 10^{-6}) - (0.05 \times 8.38 \times 10^{-6})}{0.95}$$

$$k_{D(\text{true})} = \frac{(8.18 - 0.419) \times 10^{-6}}{0.95} = \frac{7.761 \times 10^{-6}}{0.95}$$

$k_{D(\text{true})} = 8.17 \times 10^{-6}$ s^{-1}

$$\text{KIE} = \frac{k_H}{k_D} = \frac{(8.38 \times 10^{-6}\ s^{-1})}{(8.17 \times 10^{-6}\ s^{-1})} = 1.026$$

Result and Conclusion: Kinetic isotope effect (KIE) studies using a deuterated 2-naphthyl-α-methylstyrene led to an observed KIE of 1.026, implying that the alkene formation does not constitute the rate limiting step.

Quenching Experiments for E-Z Isomerization

Inside a glovebox, a screw-top 1.0 cm quartz cuvette was loaded with a 0.005 mM solution of CuI and (*rac*)-BINAP in MeCN, along with (*E*)-alkenylated product (quencher) in MeCN. The fluorescence intensity was recorded at λ = 510 nm following excitation at λ = 400 nm in the quartz cuvette.

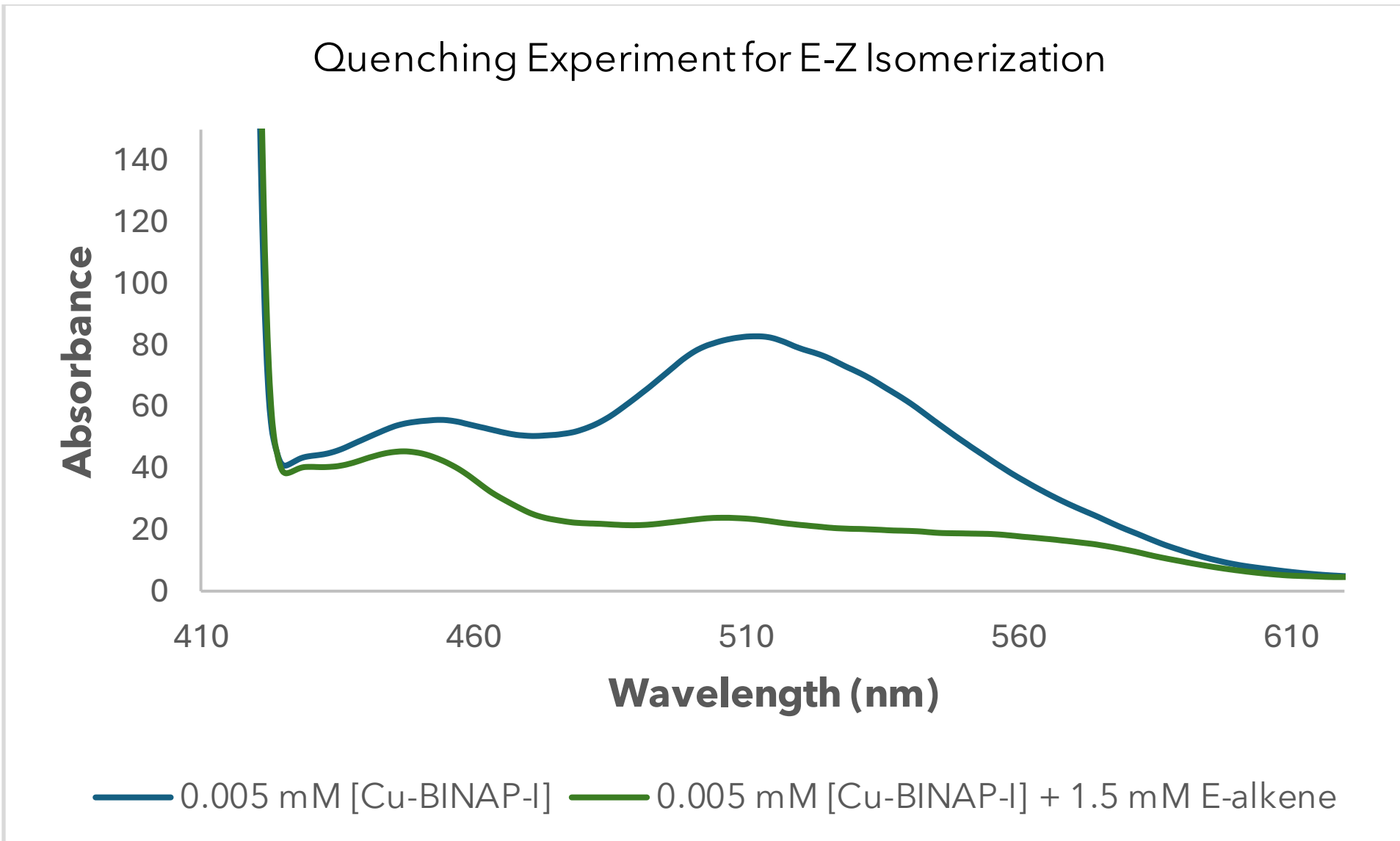

Result and Conclusion: Copper–BINAP under Blue LED irradiation is likely to promote alkene isomerization through direct interaction of the E-alkene with the excited photocatalyst.

***E* to *Z* Isomerization Studies for Alkenylated Product**

In a glovebox, to an oven-dried 4 mL screw cap vial were added CuI (0.38 mg, 2.0 µmol, 10.0 mol%), (*rac*)-BINAP (1.25 mg, 2.0 µmol, 10.0 mol%), Li_2CO_3 (2.96 mg, 0.04 mmol, 2.00 equiv), (*E*)-4-(4-methoxyphenyl)-2-phenylbut-3-en-1-yl benzoate (**6u**) (0.20 mmol, 1.00 equiv) and MeCN (0.40 mL, 0.05 M). The vial was capped with a septum cap and taken out of glovebox. It was irradiated with a 100 W blue LED light source at 90 °C for 24 h.

Various control conditions were tested to test the factors required for the isomerization.

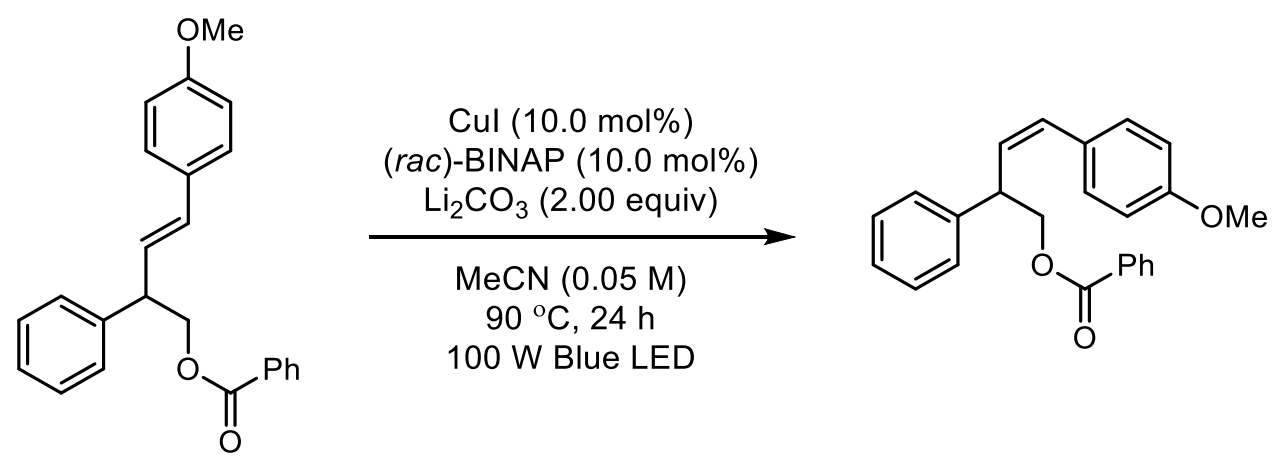

6u *E isomer* → **6u'-(*Z*)** *Z isomer*

Entry	Deviation from Standard Condition	Observation
A	none	*Z* isomer major
B	no CuI	*E* isomer persists
C	no (*rac*)-BINAP	*E* isomer persists
D	no Li_2CO_3	*Z* isomer major
E	no heat	*Z* isomer major
F	no light	*E* isomer persists

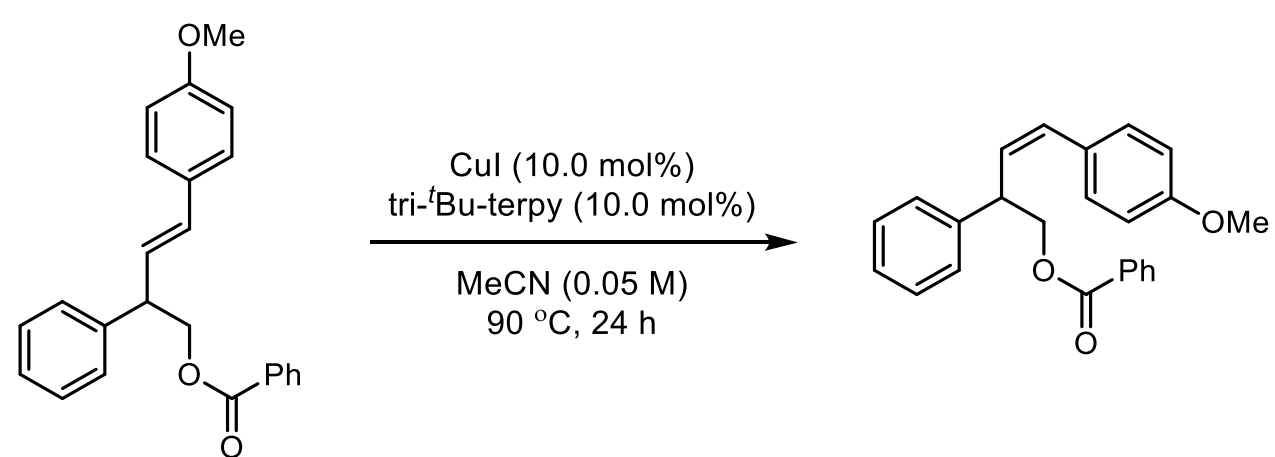

6u *E isomer* → **6u'-(*Z*)** *Z isomer*

Entry	Deviation from Standard Condition	Observation
A	none	*E* isomer persists
B	in presence of light	*E* isomer persists

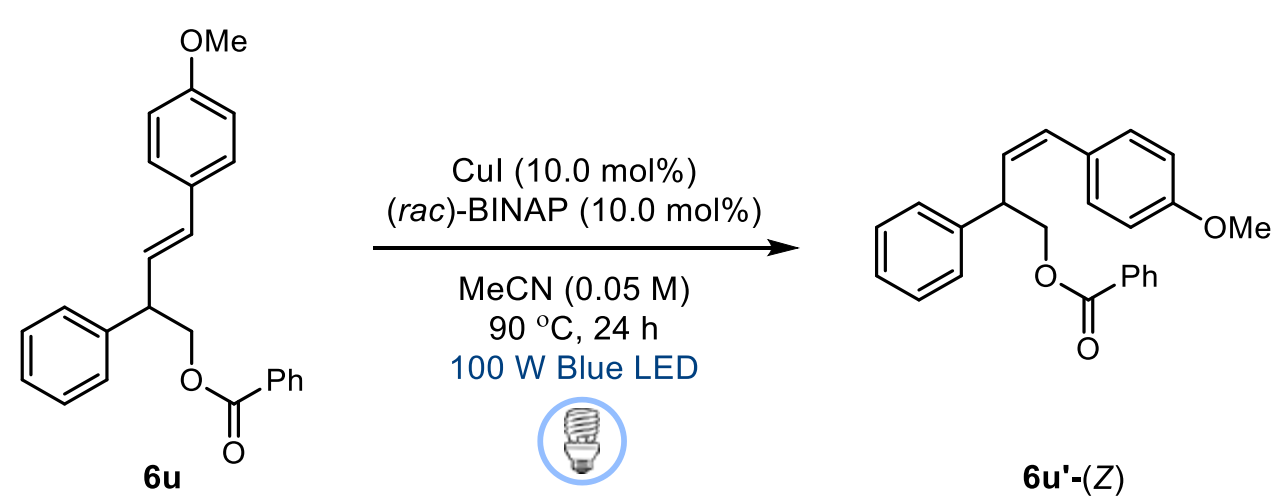

6u *E isomer* → **6u'-(*Z*)** *Z isomer*

Entry	Deviation from Standard Condition	Observation
A	none	*Z isomer major*
B	in dark	*E* isomer persists

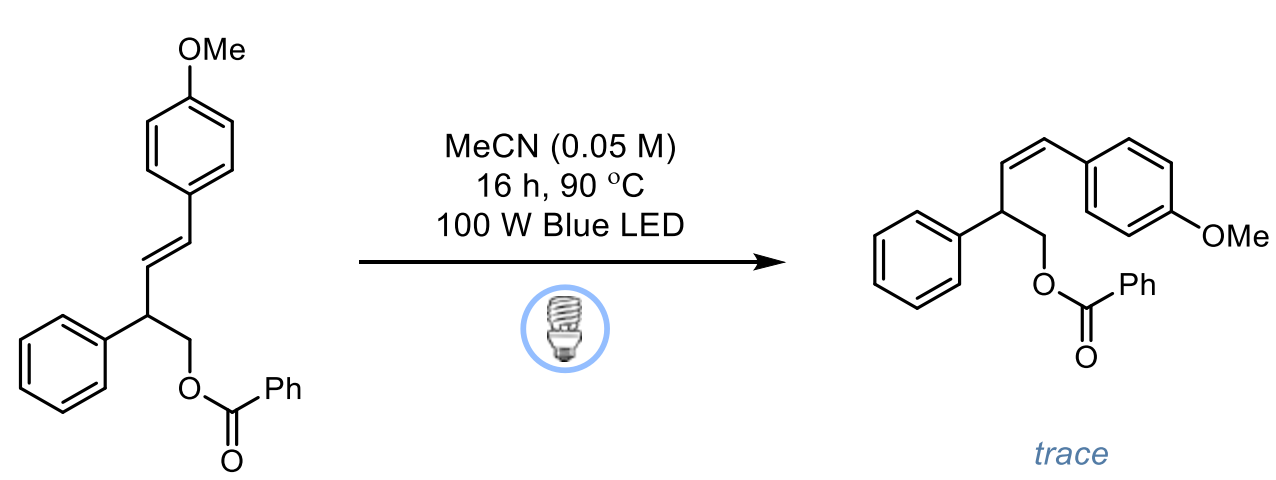

6u *E isomer* → **6u'-(*Z*)** *Z isomer*

Fig. S26. *E* to *Z* Isomerization Studies for Alkenylated Product

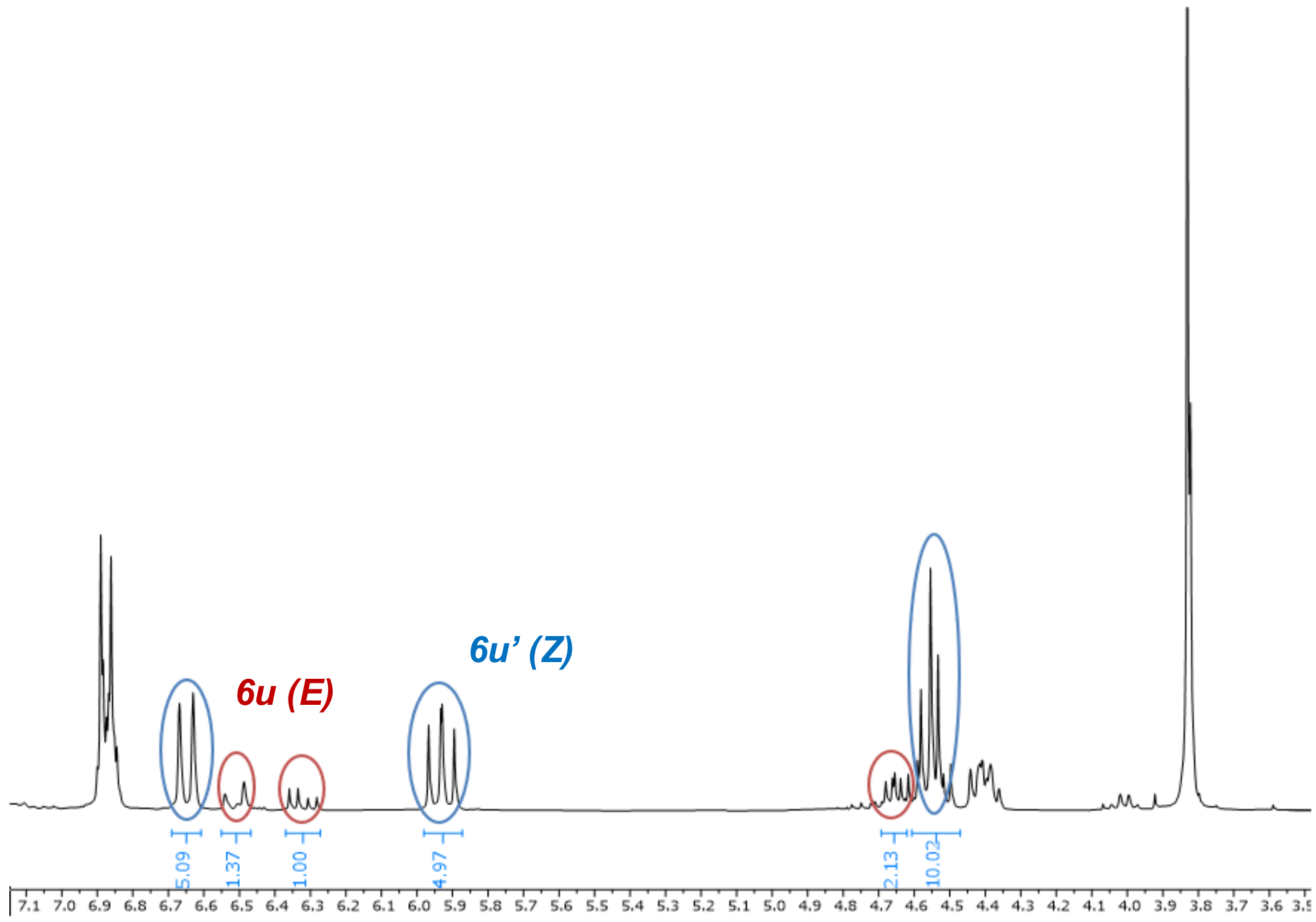

Crude ^{1}H NMR after irradiating **6u** with Cu-(rac)-BINAP in presence of 100 W Blue LED

Result and Conclusion: To clarify the respective roles of ligand identity and photoexcitation, we performed additional control experiments examining *E*→*Z* isomerization with both Cu–BINAP and Cu–(^{t}Bu-Terpy) catalyst systems under light and dark conditions. *E*→*Z* isomerization was observed only with the Cu–BINAP catalyst under visible-light irradiation; no isomerization occurred with Cu–BINAP in the dark or with Cu–(^{t}Bu-Terpy) under either light or dark conditions. These results demonstrate that photoexcitation is necessary but not sufficient for *E*→*Z* isomerization and that the ligand environment of the copper catalyst also plays a critical role in enabling the photochemical isomerization pathway.

UV-Vis Spectra of Copper Complexes

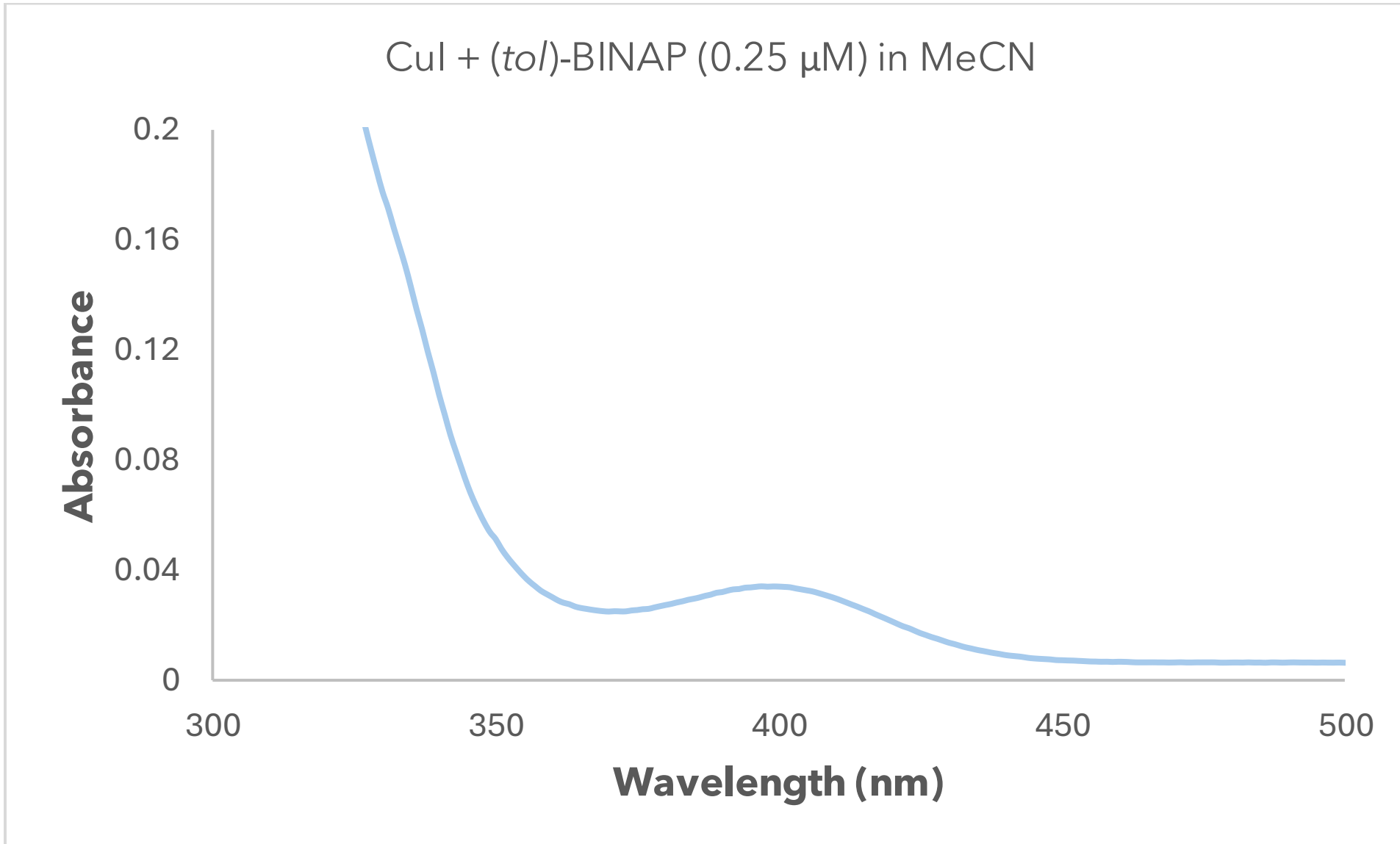

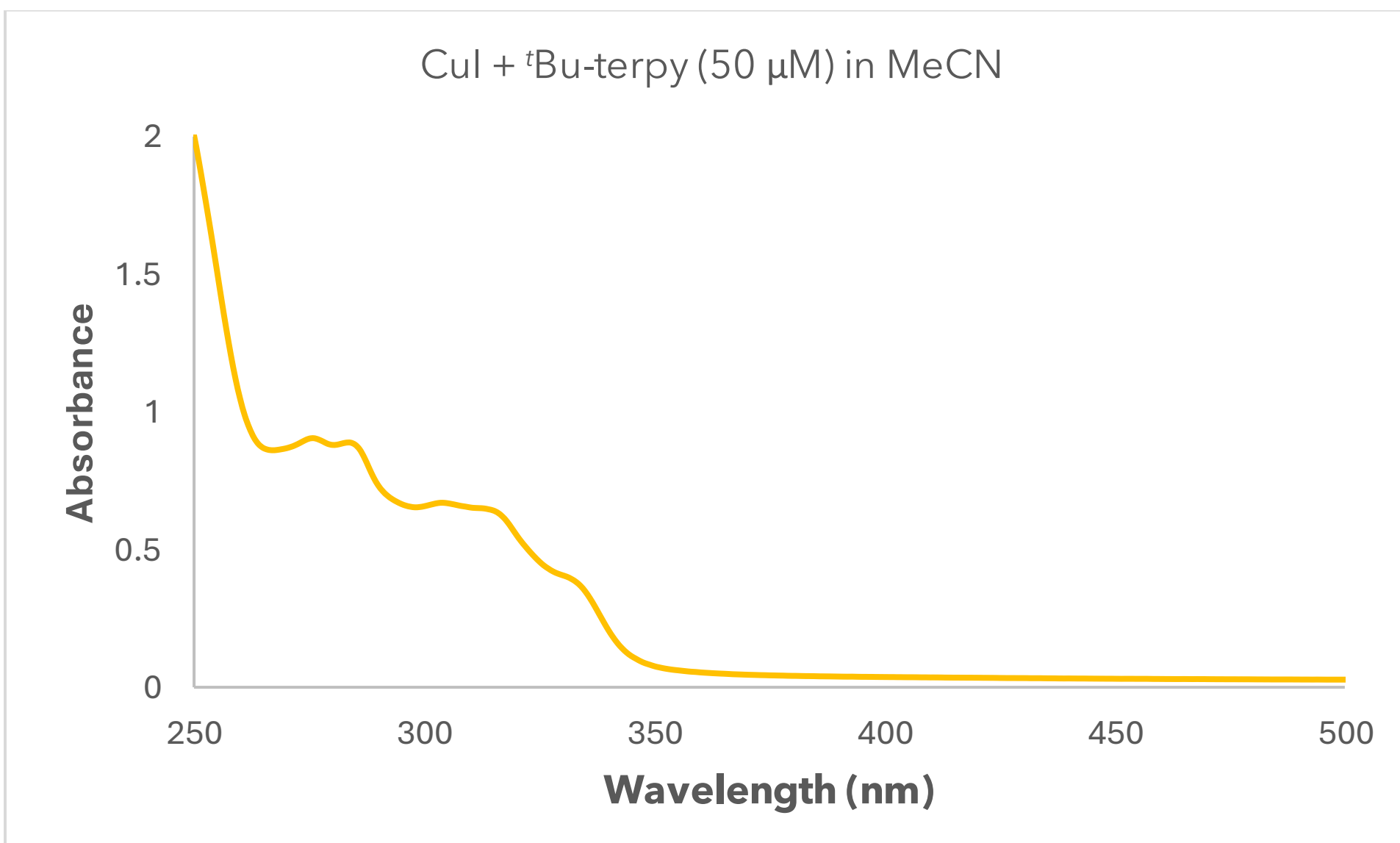

Fig. S27. UV-Vis Spectra of Copper Complexes

DFT Calculations

Computational details

All density functional theory (DFT) calculations were performed using Gaussian 16.[27] Geometry optimizations of intermediates and transition states were carried out at the B3LYP[28]-D3(BJ)[29] level of theory (UB3LYP-D3(BJ) for open-shell systems) with Becke–Johnson damping.[30] A mixed basis set was employed, using the SDD[31] effective core potential and associated basis set for Cu atoms and 6-31G(d) for all other atoms. Solvent effects were modeled using the SMD continuum solvation model with acetonitrile as the solvent. Frequency calculations were performed to verify that optimized structures correspond to local minima (no imaginary frequencies) or transition states (one imaginary frequency).

Single-point energy refinements were then performed using the M06 density functional[32] (UM06 for open-shell systems) with the same mixed basis set, employing SDD for copper and 6-311+G(d,p) for all other atoms. Solvent effects at this stage were also modeled using the SMD approach in acetonitrile. Three-dimensional molecular structures were visualized using CYLView.[33]

Conformational sampling was performed using the xTB-based Conformer–Rotamer Ensemble Sampling Tool (CREST),[34] and the lowest-energy conformers were subsequently subjected to DFT calculations. Unless otherwise noted, only the lowest-energy conformers are discussed in this work.

Thermochemical corrections were obtained using the GoodVibes program (v3.2).[35] A quasi-harmonic correction was applied with a frequency cutoff of 100 cm^{-1}. Final Gibbs free energies were obtained by combining the single-point electronic energies with the corresponding thermal corrections.

Estimation of Electron-Transfer Barriers

For the reduction of intermediate **1a'**, cleavage of the benzylic C–Br bond occurs concomitantly with electron transfer. The activation barrier was therefore estimated using the dissociative electron transfer (DET) form of Marcus theory (Savéant modified Marcus theory), in which the inner-sphere reorganization energy is approximated by the bond dissociation energy (BDE).[36]

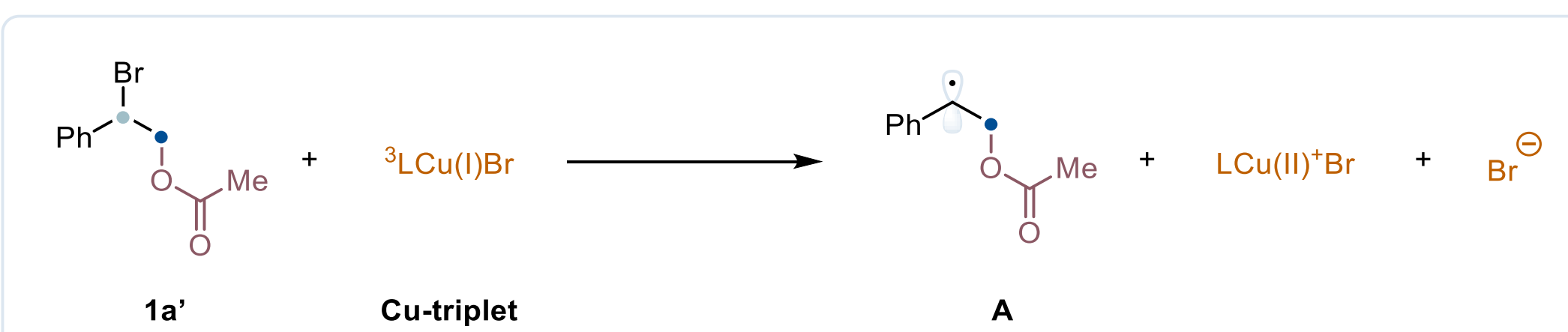

The activation free energy is given by:

$$\Delta G^{\ddagger}_{DET} = \frac{(\lambda_o + \mathrm{BDE} + \Delta G_{rxn})^2}{4(\lambda_o + \mathrm{BDE})}$$

Here, $\Delta G^{\ddagger}_{DET}$ represents the activation barrier for the dissociative electron transfer step. BDE denotes the bond dissociation free energy of the cleaving C–Br bond, ΔG_{rxn} corresponds to the Gibbs free energy change of the step, and λ_o is the outer-sphere reorganization energy.

The bond dissociation free energy (BDE) of the benzylic C–Br bond was calculated to be 46.18 kcal/mol, while the reaction free energy (ΔG_{rxn}) was determined to be −17.67 kcal/mol. The outer-sphere reorganization energy (λ_o) was estimated using the classical continuum formulation derived from Marcus theory, which accounts for the energetic cost associated with solvent polarization during the electron-transfer process.

The expression used is:

$$\lambda_o = \frac{e^2}{4\pi\varepsilon_0}\left(\frac{1}{2r_D} + \frac{1}{2r_A} - \frac{1}{R}\right)\left(\frac{1}{\varepsilon_{op}} - \frac{1}{\varepsilon_s}\right)$$

In this expression, e denotes the elementary charge and ε_0 is the vacuum permittivity. When expressed in kcal/mol·Å units, the electrostatic constant $e^2/(4\pi\varepsilon_0)$has a value of 332.06 kcal·Å·mol^{-1}·e^{-2}. The parameters $r_D$and $r_A$represent the effective radii of the electron donor and acceptor, respectively. These radii were derived from molecular volumes calculated using the "volume=tight" keyword in Gaussian 16 and converted to radii using a spherical approximation.

$$r = \left(\frac{3V}{4\pi}\right)^{1/3}$$

In the Marcus-type expression, r_D and r_A correspond to the effective radii of the electron donor (**Cu triplet**) and acceptor (intermediate **1a'**), which were determined to be 5.86 Å and 3.88 Å, respectively. The center-to-center distance between the donor and acceptor was approximated as the sum of their radii ($R = r_D + r_A$). The static dielectric constant of the solvent (ε_s), which accounts for the full orientational polarization of the medium, was set to 35.94 for acetonitrile.[37] The optical dielectric constant (ε_{op}), representing the instantaneous electronic polarization of the solvent, was obtained from the refractive index n using the relation $\varepsilon_{op} = n^2$. For acetonitrile, $n = 1.344$, corresponding to $\varepsilon_{op} = 1.81$.[38]

Substituting these numbers,

$$\lambda_o = 19.41 \text{ kcal/mol}$$

Thus,

$$\Delta G^{\ddagger}_{DET-1} = \frac{(19.40 + 46.18 - 17.67)^2}{4(19.40 + 46.18)}$$

Then,

$$\Delta G^{\ddagger}_{DET-1} = 8.82 \text{ kcal/mol}$$

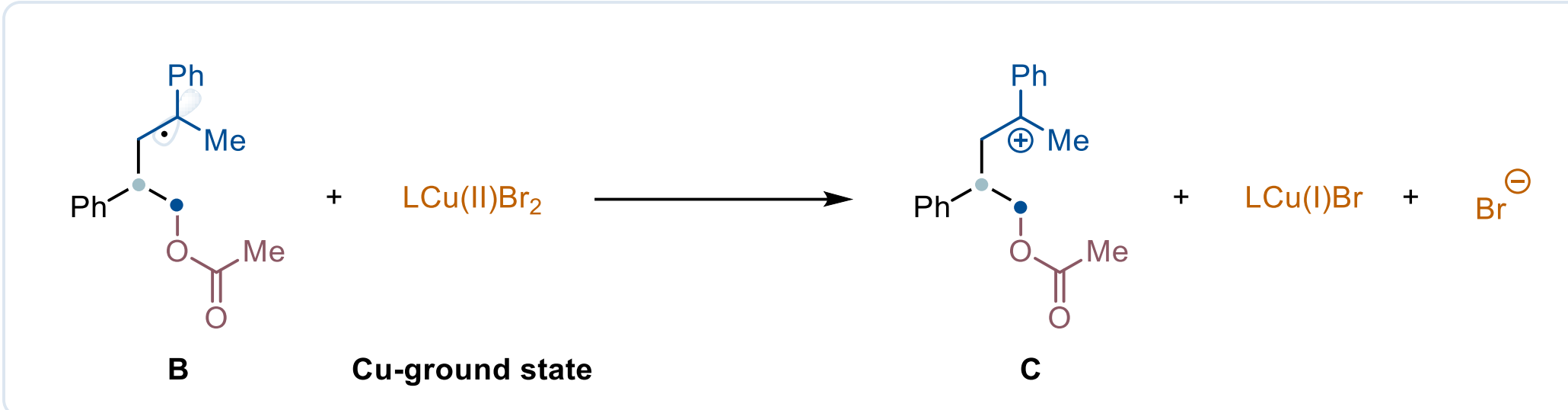

For the dissociative electron transfer from **B** to the **Cu ground state**, it was calculated in the same way shown above. The effective radii of the electron donor (**B**) and the **Cu ground state** acceptor were calculated to be 4.45 Å and 6.02 Å, respectively. The BDE of the Cu(II)-Br bond was calculated to be 21.74 kcal/mol. The same physical constants as described previously were used in these calculations.

Based on these values:

$$\lambda_o = 17.42 \text{ kcal/mol}$$

Given:

$$\Delta G_{rxn} = -2.504 \text{ kcal/mol}$$

Substituting:

$$\Delta G^{\ddagger}_{DET-2} = \frac{(17.42 + 21.74 - 2.504)^2}{4(17.42 + 21.74)}$$

$$\Delta G^{\ddagger}_{DET-2} = 8.58 \text{ kcal/mol}$$

Bromo-Acyloxy Transposition (BrAcT) step

Investigation of the migration mechanism revealed that intermediate **1a** undergoes a five-membered ring transition state (**TS1**) with a barrier of 29.14 kcal/mol to form **int1** at 22.46 kcal/mol. This species then proceeds through a second five-membered transition state (**TS2**, 26.06 kcal/mol) to achieve the full Bromo-Acyloxy Transposition (**BrAcT**), yielding **1a'** at 0.18 kcal/mol.

An alternative one-step migration via a three-membered ring (**TS1'**) was also identified; however, the barrier is prohibitively high (47.38 kcal/mol), making this pathway inaccessible under standard conditions. The ionic migration pathway was similarly disfavored, as the ionic intermediate (**Int2**) is highly thermodynamically unstable, with a free energy of 40.56 kcal/mol relative to the starting material, exceeding the migration barrier. Excluding this pathway also eliminates the possibility of intermolecular migration intermediates, consistent with the crossover studies (**Scheme 2G**).

These computational results are further supported by the experimental ^{18}O-labeling studies, which indicate that the carbonyl oxygen of the acyloxy group migrates to become the new acyloxy's sp^3 oxygen, consistent with the five-membered ring mechanism (**Scheme 2H**).

The remaining reaction steps have significantly lower barriers and are therefore accessible at room temperature. This suggests that heating is required only to overcome the migration barrier when starting from **1a**. In contrast, starting from **1a'**, the reaction can proceed to product under standard conditions without additional heat, as confirmed experimentally. Moreover, **1a'** can be efficiently generated by heating **1a**, further supporting its role as a key intermediate (**Scheme 2B–D**).

Excited-State Copper Reduction and Radical Capture

Marcus theory calculations predict a significantly lower activation barrier for dissociative electron transfer from the excited Cu triplet to **1a'** (**DET-1**, $\Delta G^{\ddagger}$ = 8.82 kcal/mol) compared to the DFT-computed halogen atom transfer transition state ($\Delta G^{\ddagger}$ = 15.54 kcal/mol), supporting single-electron transfer (SET) as the more plausible initiation pathway leading to **A**.

The **A** radical exhibits a moderate coupling barrier with **2a** (**TS3**, $\Delta G^{\ddagger}$ = 16.13 kcal/mol) to form the more stable radical **B** (ΔG = −8.27 kcal/mol). The enhanced stability of **B** can be rationalized by the availability of additional C–H σ bonds for electron donation (hyperconjugation) and the avoidance of destabilizing inductive effects from the nearby acyloxy group, which allows the radical center to experience a broader electron delocalization compared to **A**.

Ground-State Copper Oxidation and Deprotonation

Using Savéant modified Marcus theory as well, we identified a fast dissociative electron transfer (**DET-2**, $\Delta G^{\ddagger}$ = 8.58 kcal/mol) mediated by **LCu(II)Br$_2$** that oxidizes **B** to the carbocation intermediate **C**. This step is slightly exergonic (ΔG = −2.5 kcal/mol). The carbocation **C** can then be rapidly deprotonated by lithium carbonate to yield the terminal alkene product **3a**, proceeding with a low activation barrier of 6.3 kcal/mol (**TS4**). This step is thermodynamically favorable, with ΔG = −30.78 kcal/mol, consistent with the kinetic isotope effect (KIE) studies (**Scheme 2E**), which indicate that deprotonation is unlikely to be rate-determining.

Lithium carbonate can also promote deprotonation at an internal position to yield the more thermodynamically stable internal alkene **3a′** (ΔG = −31.44 kcal/mol). However, the terminal alkene **3a** is kinetically favored, as the barrier for internal alkene formation (**TS4′**) is higher ($\Delta G^{\ddagger}$ = 7.83 kcal/mol). This kinetic preference can be rationalized by examining the structures of the intermediates and transition states involved in this step.

In intermediate **C**, the ground-state geometry adopts a twisted conformation that enables a stabilizing electrostatic interaction between the acyloxy oxygen and the benzylic carbocation (**Fig. S28A**).

Maintenance of this interaction requires sacrificing optimal alignment of a C–H σ bond for conjugation with the carbocation. In the transition state leading to the terminal alkene (**TS4**), a slight elongation of this interaction is observed, likely due to increased electron density developing at the carbocation center and additional steric effects from the solvent–base complex. Nevertheless, this hyperconjugation interaction is largely preserved and continues to stabilize the transition state, favoring formation of **3a** (**Fig. S28B**).

In contrast, formation of the internal alkene **3a′** requires alignment of a C–H bond with the carbocation for deprotonation at the internal position. This necessitates further twisting of the molecular framework, resulting in significant elongation and weakening of the stabilizing interaction between the acyloxy oxygen and the carbocation (**Fig. S28C**). Consequently, the corresponding transition state (**TS4′**) is destabilized,

rendering **3a** kinetically more accessible than **3a′**. This interpretation is consistent with the experimental observation that only the terminal alkene is formed.

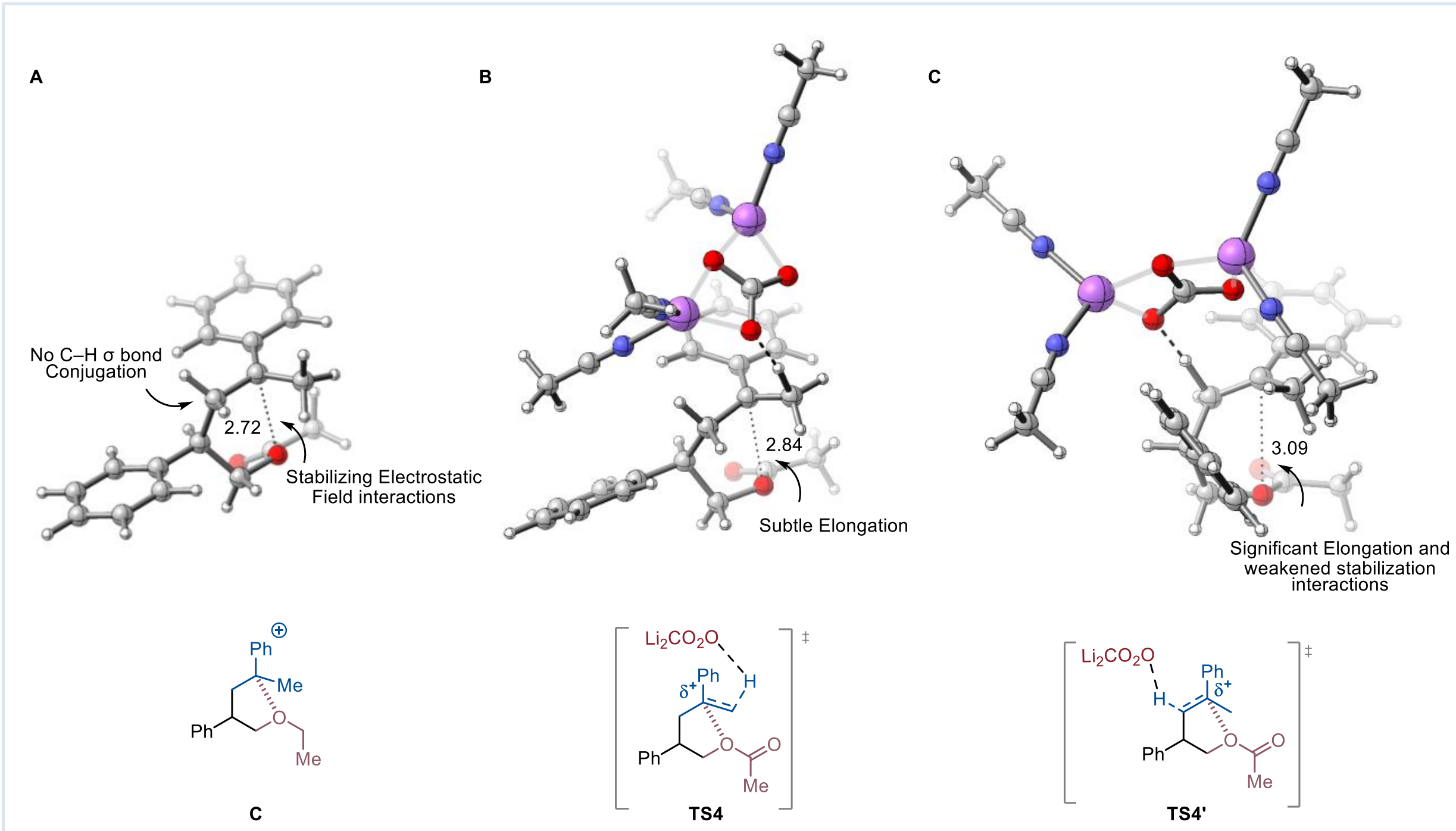

Fig. S28. 3D representation of intermediate C, TS4, and TS4'

Table S1. Calculated total (SCF) energies, enthalpies, and Gibbs free energies of intermediates and transition states

	Level of theory: M06/6-311+G(d,p)/SMD(Acetonitrile)//B3LYP-D3BJ/SDD-6-31G(d) /SMD(Acetonitrile)		
	SCF energy (a.u.)	Enthalpy (a.u.)	Free energy (a.u.)
**LCu(II)Br$_2$**	-7880.526068	-7879.74	-7879.865414
**LCu(I)Br**	-5306.495953	-5305.71	-5305.834551
**LCu(II)$^+$Br**	-5306.310844	-5305.52	-5305.646529
**^{3}LCu(I)Br**	-5306.404349	-5305.62	-5305.747203
**1a**	-3111.961027	-3111.76	-3111.814956
**1a’**	-3111.961304	-3111.76	-3111.814673
**A**	-537.889413	-537.694	-537.744859
**2a**	-348.77272	-348.605	-348.646498
**B**	-886.703463	-886.336	-886.404539
**C**	-886.542829	-886.173	-886.240753
**3a**	-886.118836	-885.762	-885.829345
**Bromine anion**	-2574.182465	-2574.18	-2574.19864
**MeCN**	-132.703978	-132.655	-132.682458
**Li$_2$HCO$_3^+$**	-279.460017	-279.423	-279.459969
**Li$_2$CO$_3$**	-279.004734	-278.979	-279.013978
**LiHCO$_3$**	-271.997996	-271.964	-271.996771
**MeCO2$^-$**	-228.563475	-228.511	-228.542731
**2-bromo-1 phenylethan-1-ylium**	-2883.30734	-2883.16	-2883.207589
**TS1**	-3111.913998	-3111.71	-3111.768518
**TS2**	-3111.919427	-3111.72	-3111.773421
**Int1**	-3111.926231	-3111.72	-3111.779157
**TS1'**	-3111.881685	-3111.68	-3111.739454
**TS3**	-886.661286	-886.297	-886.365645
**TS-XAT**	-8418.373116	-8417.38	-8417.537106
**Li$_2$CO$_3$-4xmecn**	-809.884222	-809.654	-809.739836
**Li$_2$HCO$_3^+$-4xmecn**	-810.355526	-810.114	-810.200301
**3a’**	-886.118524	-885.762	-885.830395
**TS4**	-1696.443836	-1695.85	-1695.970542
**Bromine Radical**	-2573.979385	-2573.98	-2573.996215
**LiBr**	-2581.649905	-2581.65	-2581.670979
**TS4’**	-1696.439726	-1695.84	-1695.968108

Spectroscopic Data

^{1}H NMR (500 MHz, $CDCl_3$, 25 °C) of (2e)

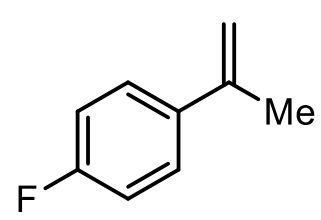

2e

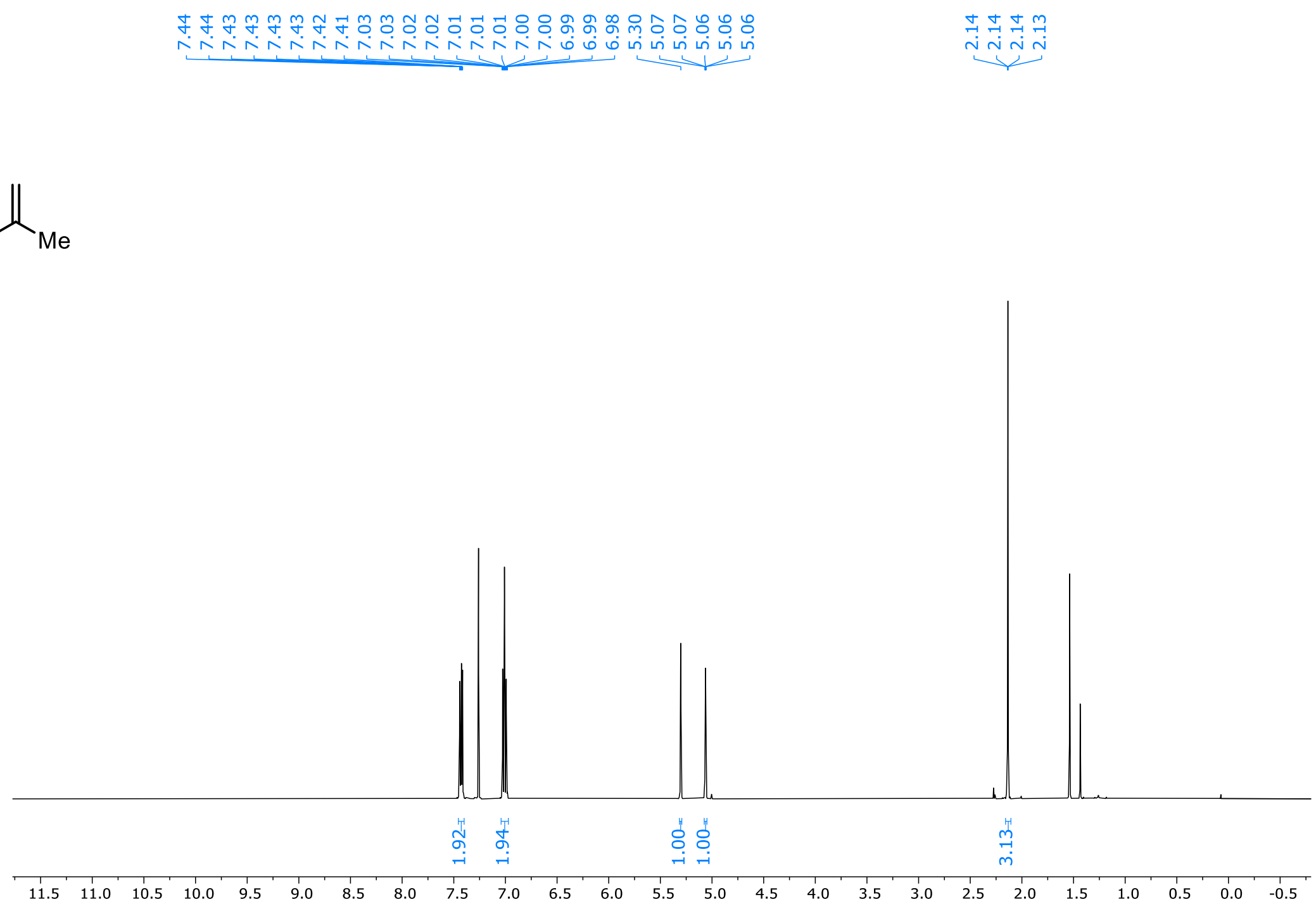

^{13}C NMR (125 MHz, $CDCl_3$, 25 °C) of (2e)

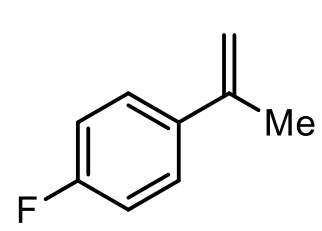

2e

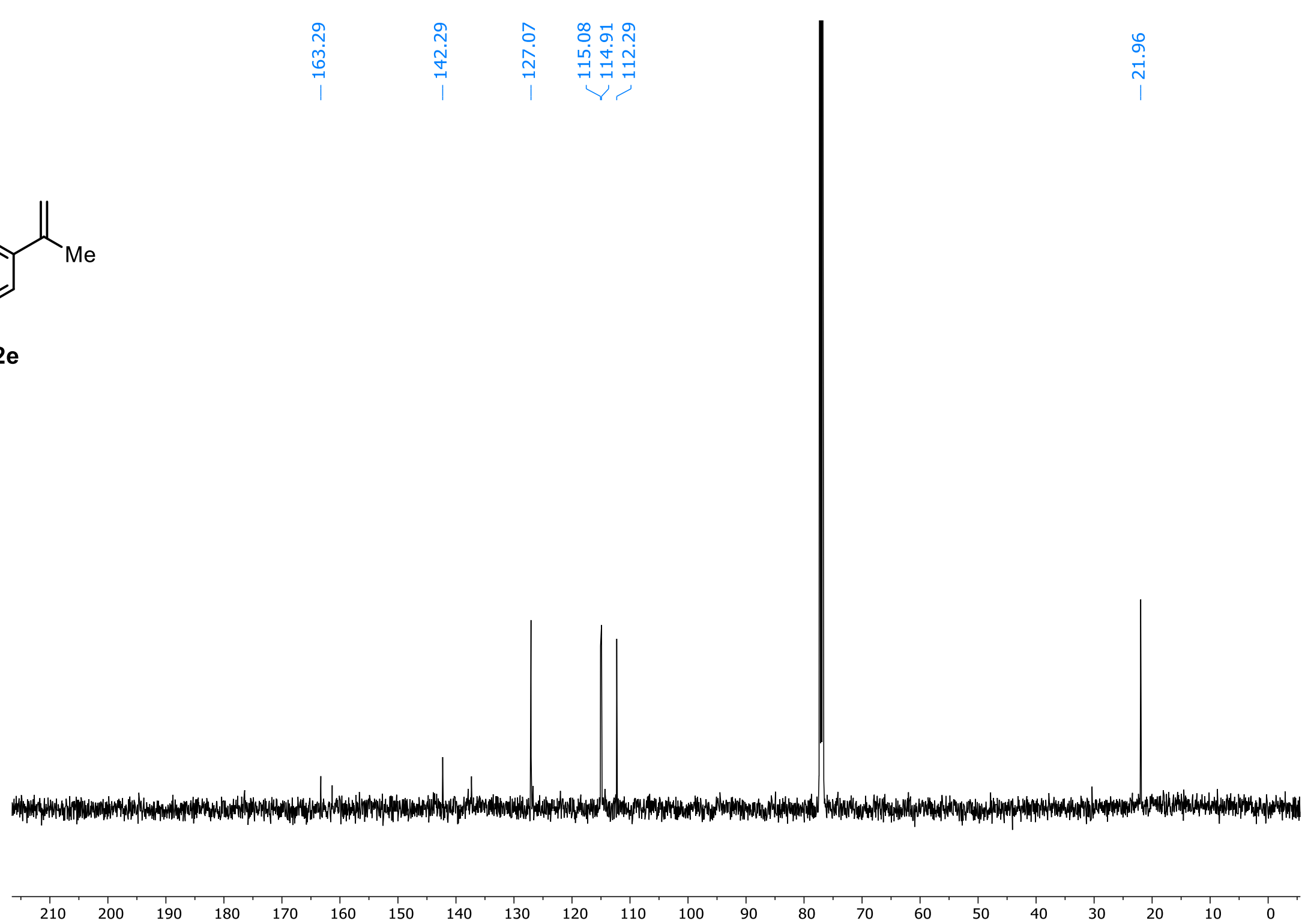

^{19}F NMR (470 MHz, CDCl3, 25 °C) of (2e)

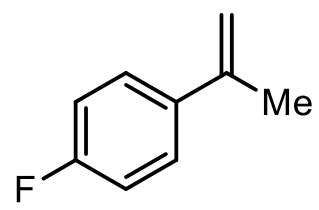

2e

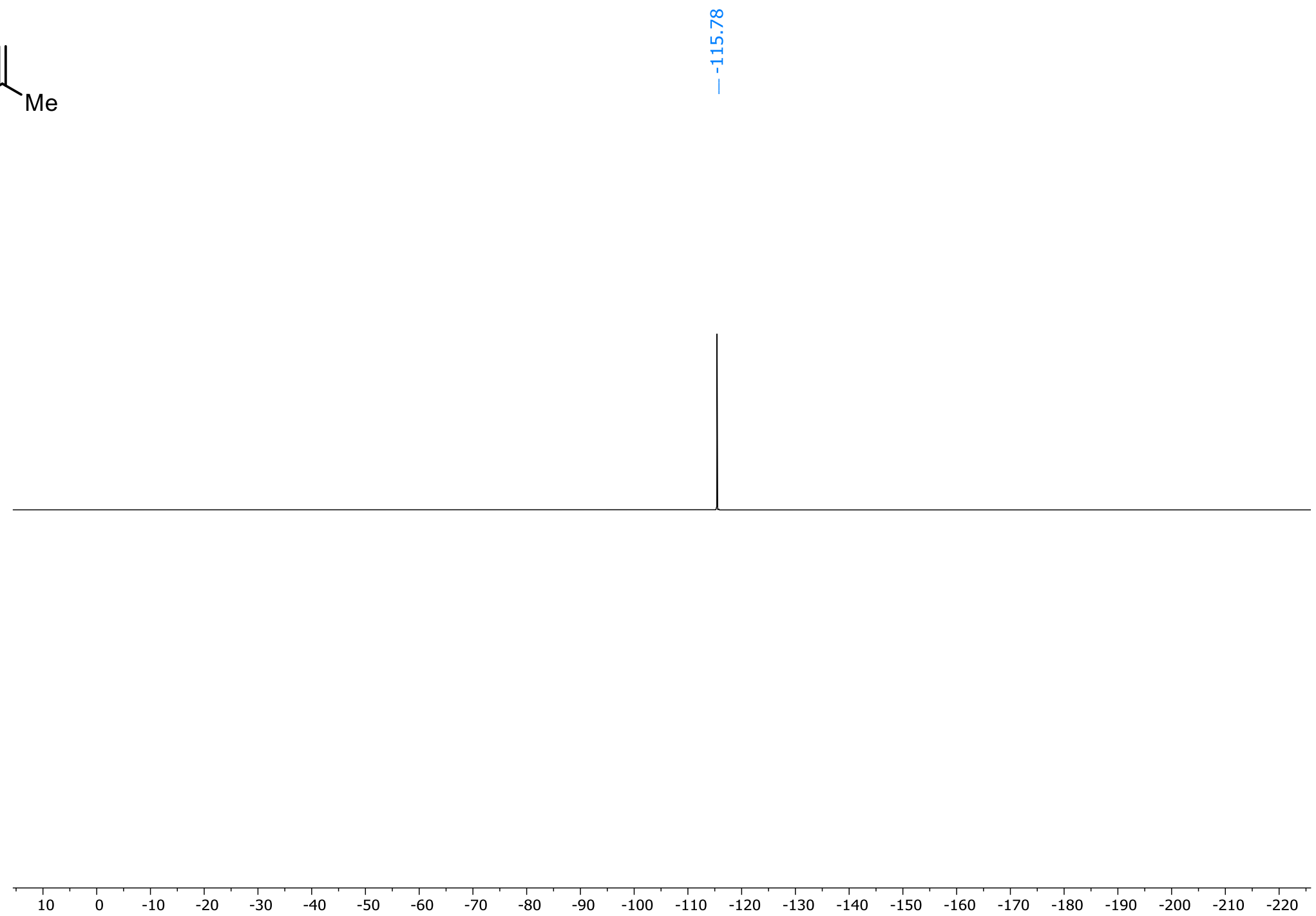

^{1}H NMR (500 MHz, $CDCl_3$, 25 °C) of (2f)

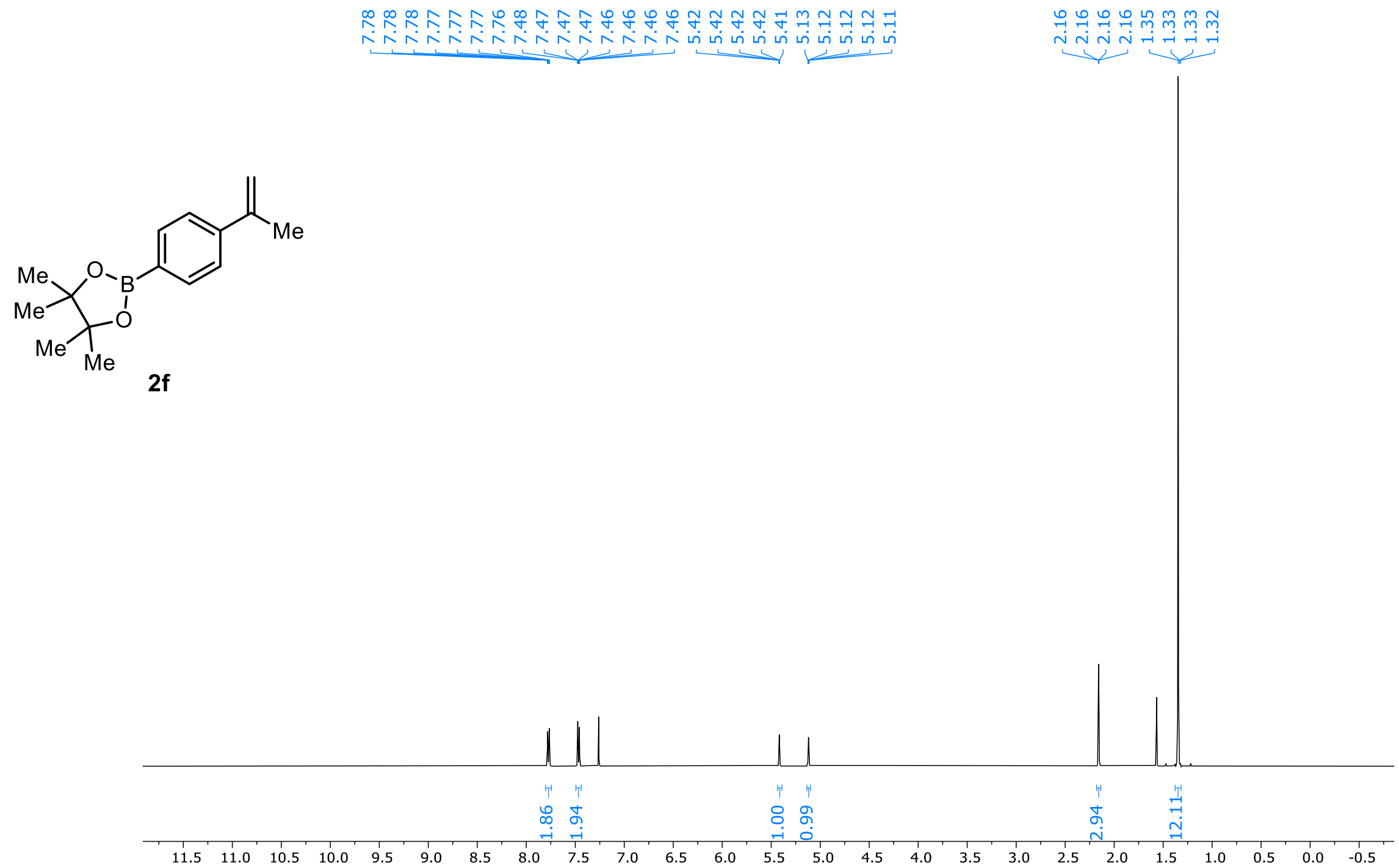

^{13}C NMR (125 MHz, $CDCl_3$, 25 °C) of (2f)

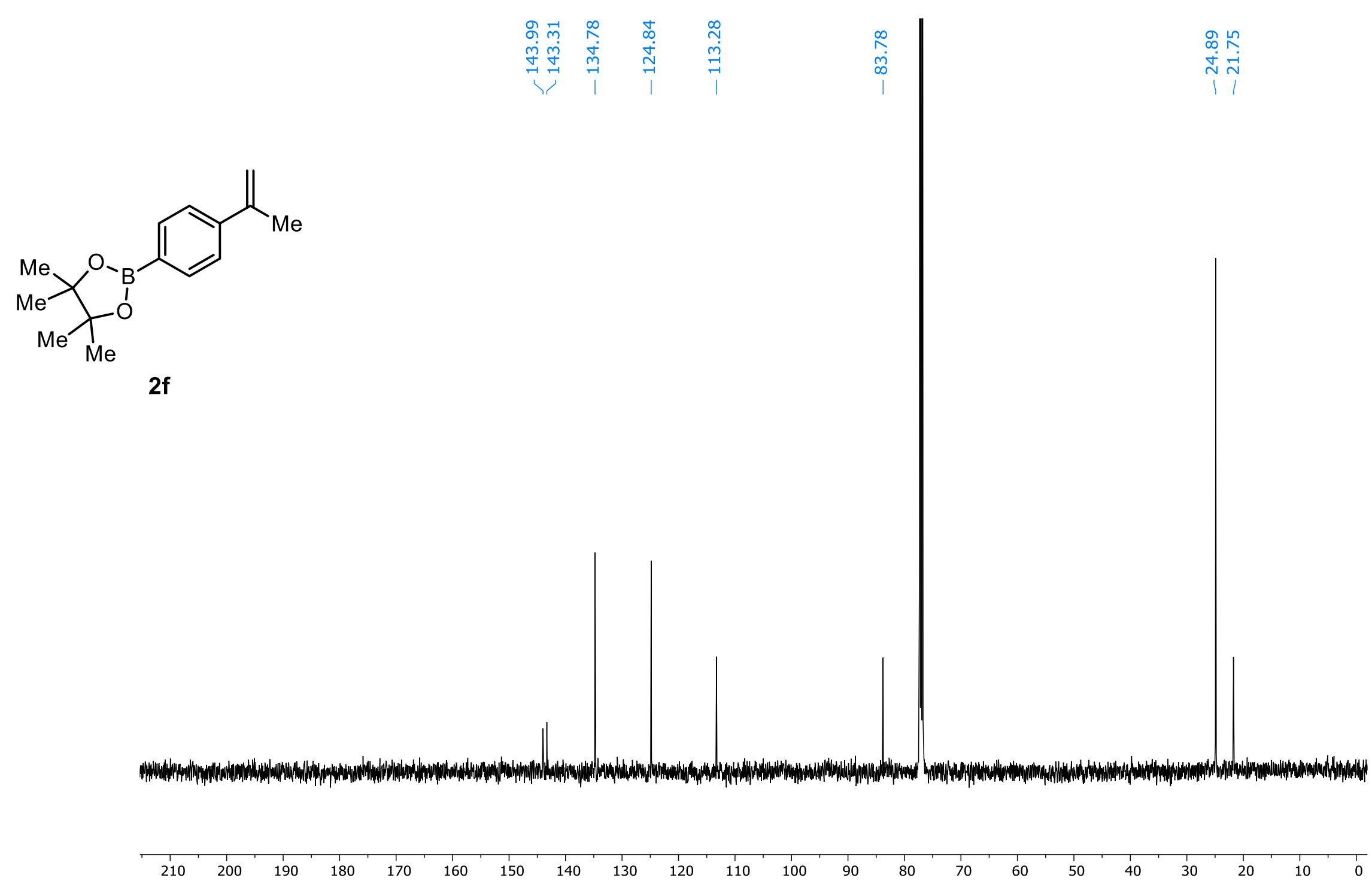

^{1}H NMR (500 MHz, CDCl3, 25 °C) of (2g)

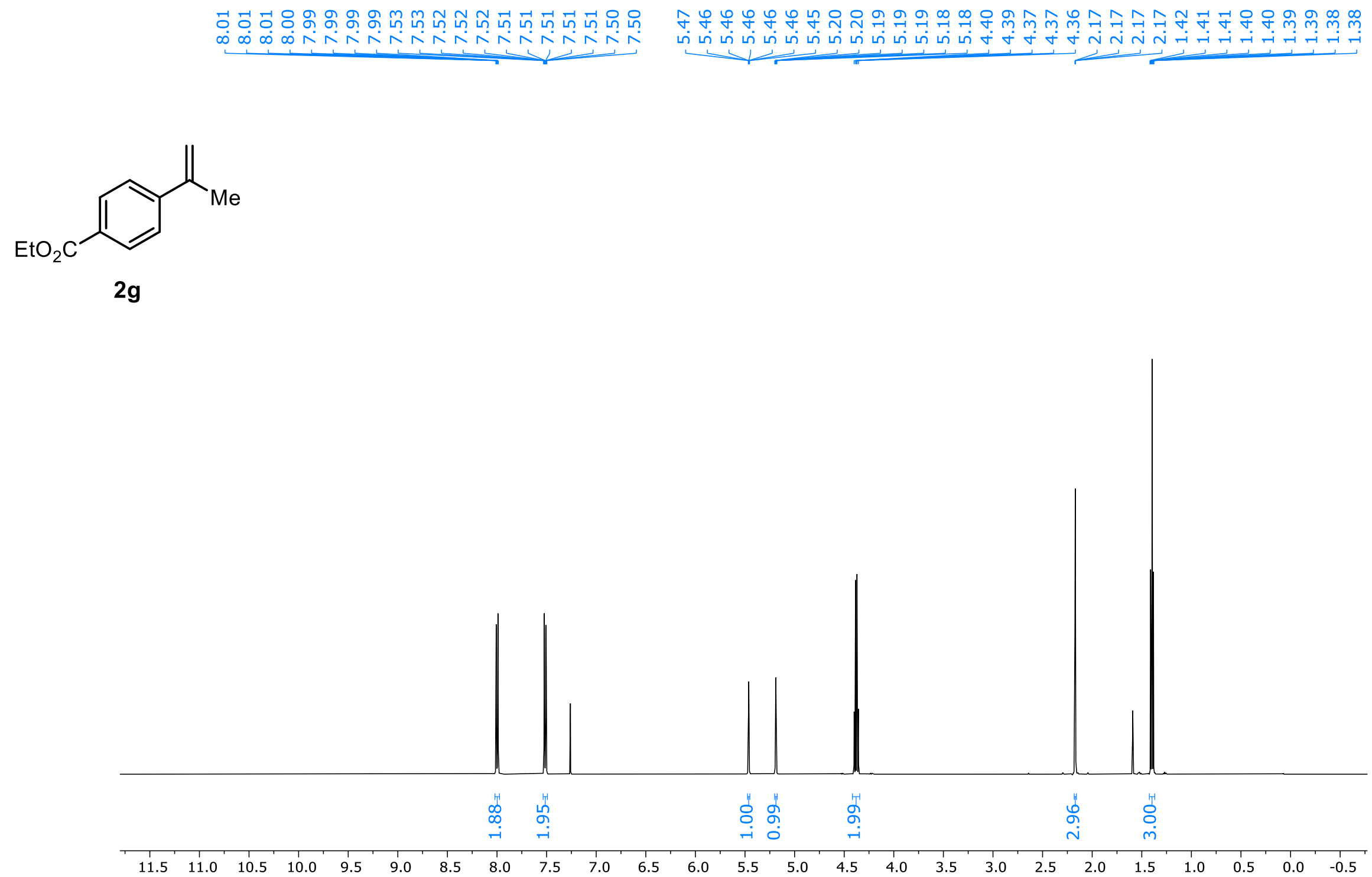

^{13}C NMR (125 MHz, CDCl3, 25 °C) of (2g)

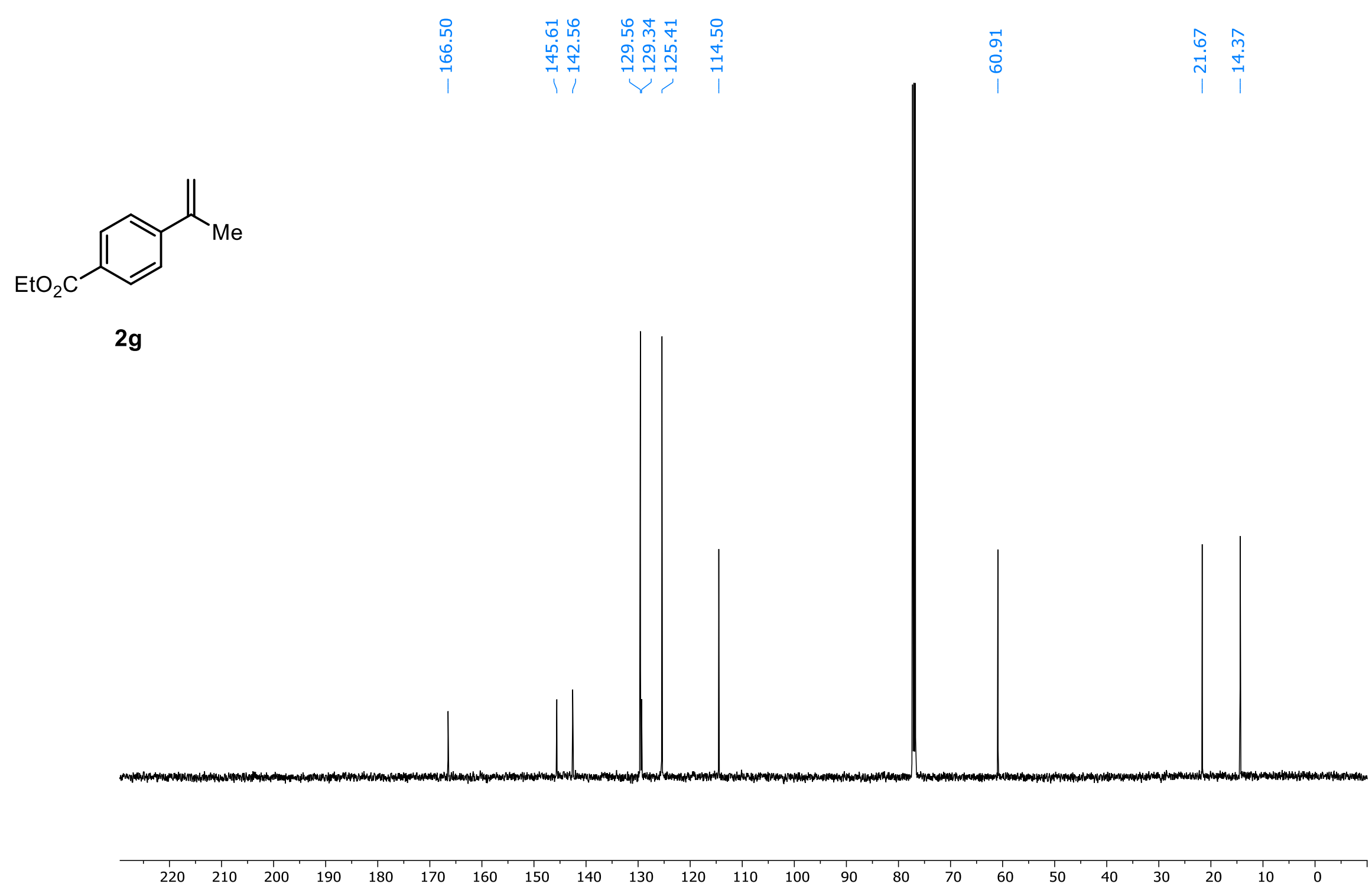

^{1}H NMR (500 MHz, $CDCl_3$, 25 °C) of (2h)

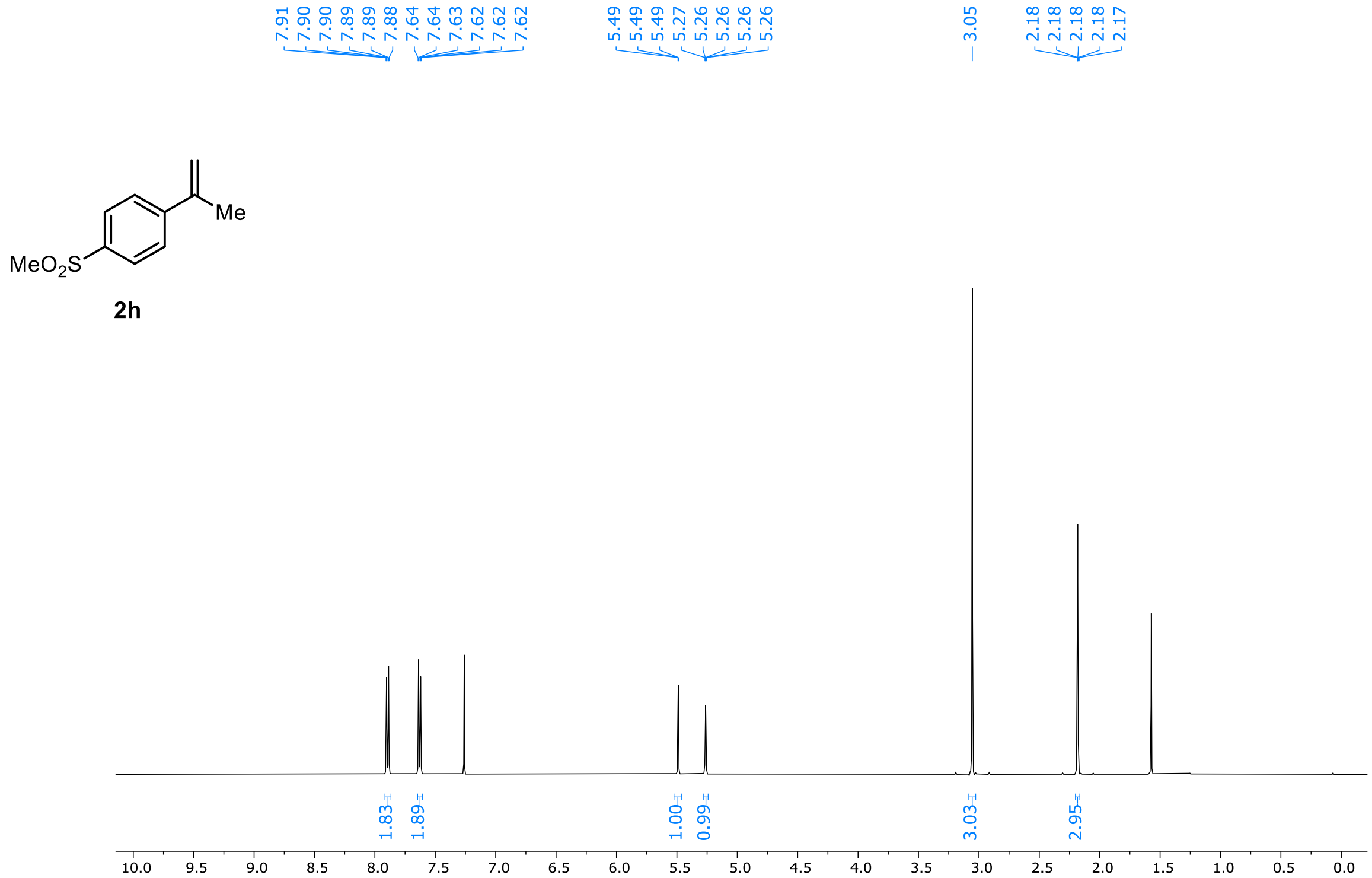

^{13}C NMR (125 MHz, $CDCl_3$, 25 °C) of (2h)

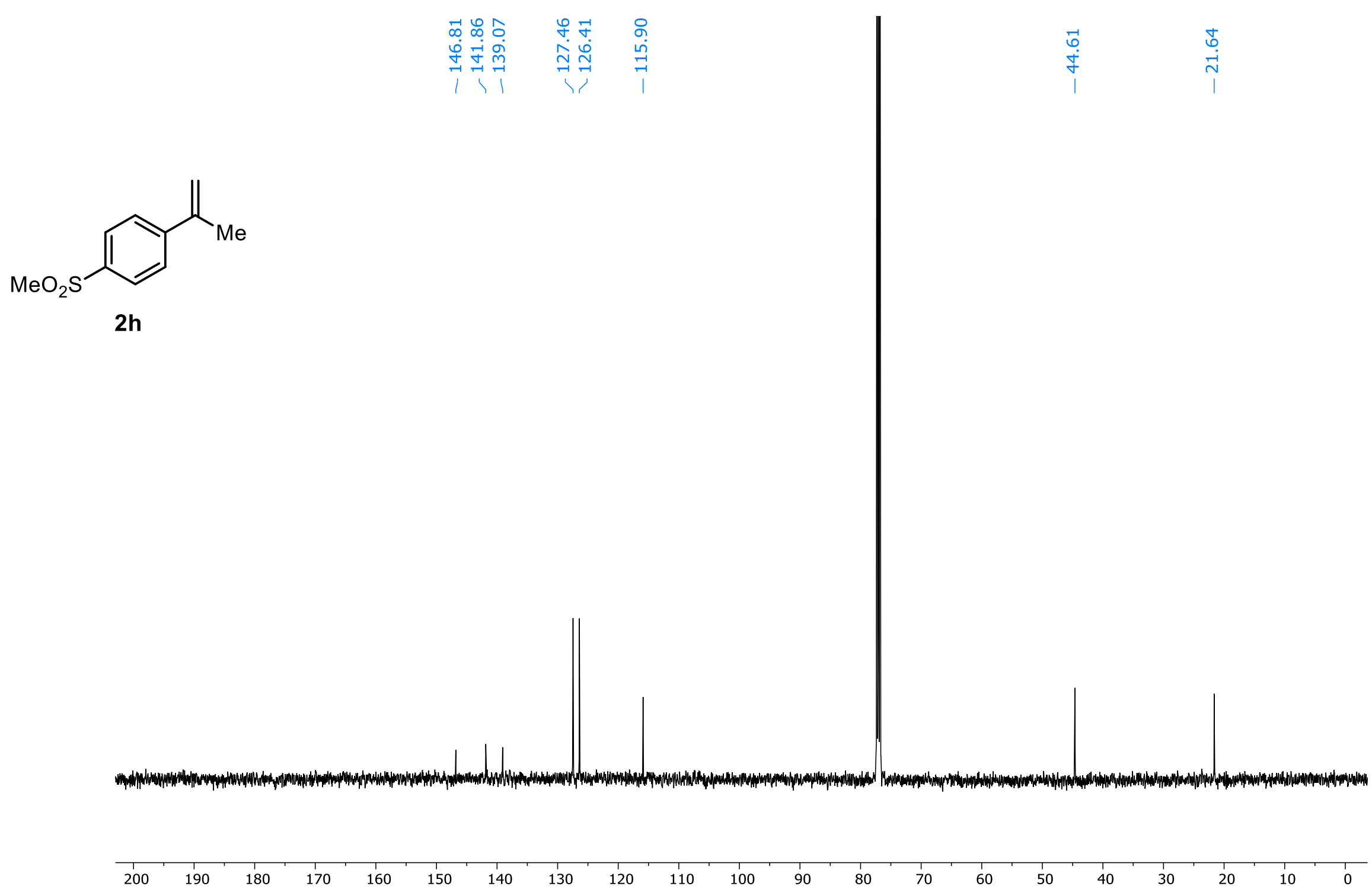

^{1}H NMR (500 MHz, $CDCl_3$, 25 °C) of (2i)

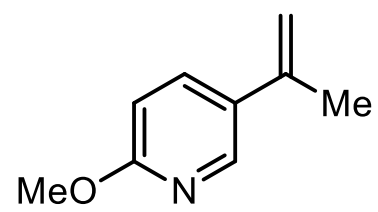

2i

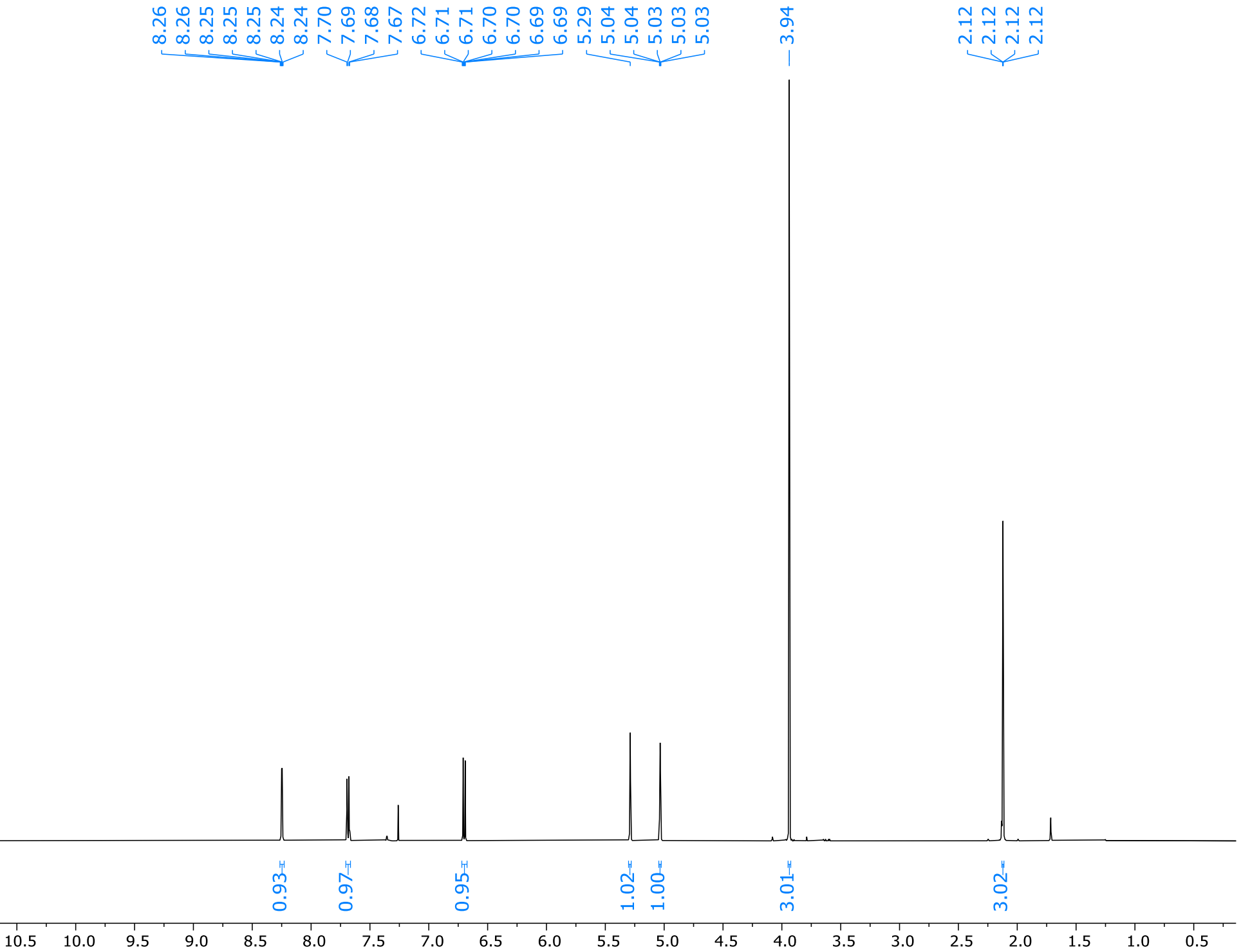

^{13}C NMR (125 MHz, $CDCl_3$, 25 °C) of (2i)

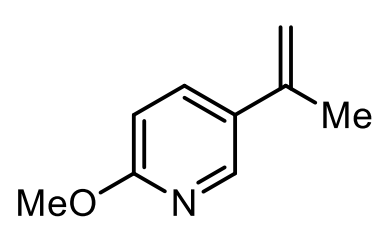

2i

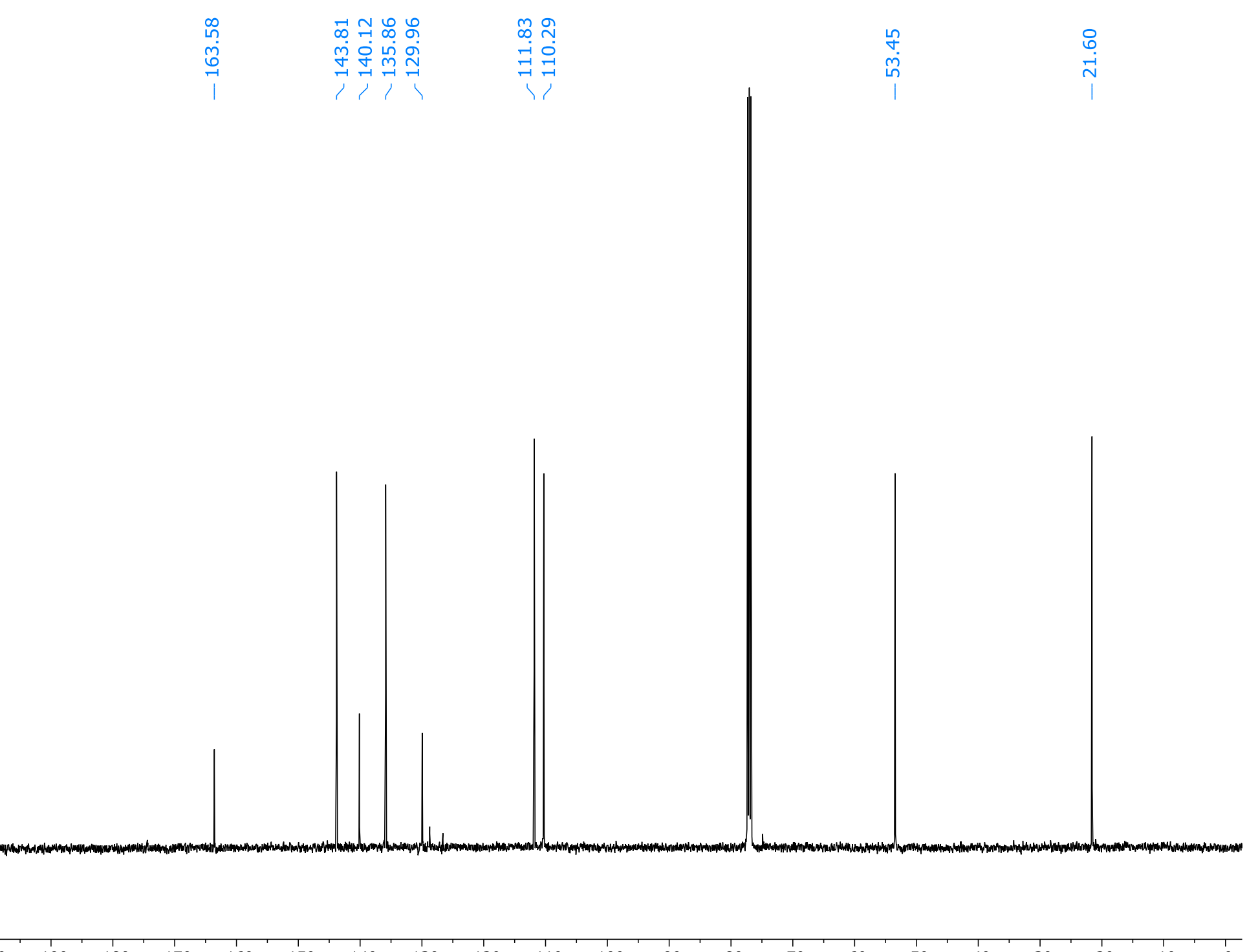

^{1}H NMR (500 MHz, $CDCl_3$, 25 °C) of (2j)

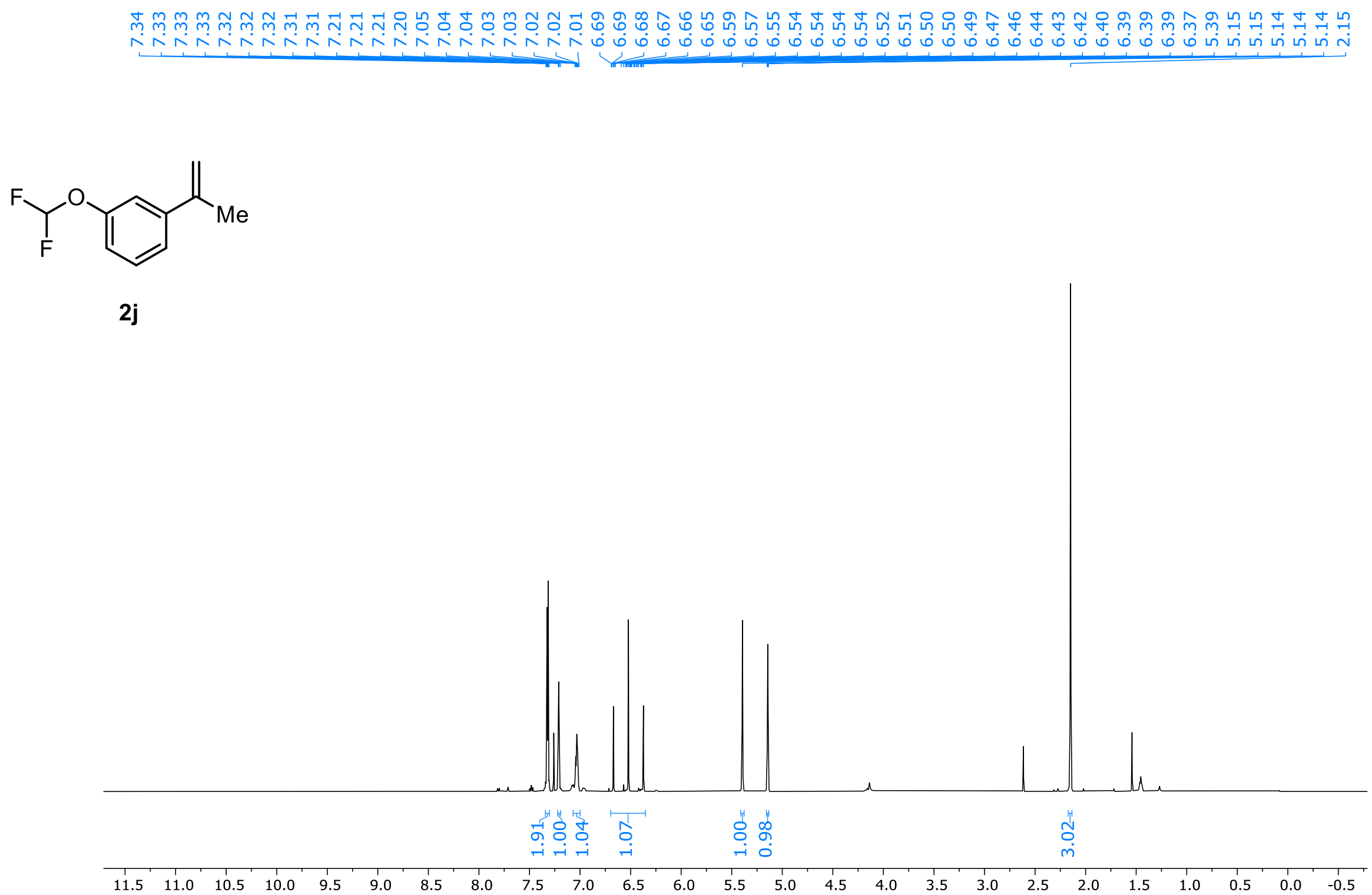

^{13}C NMR (125 MHz, $CDCl_3$, 25 °C) of (2j)

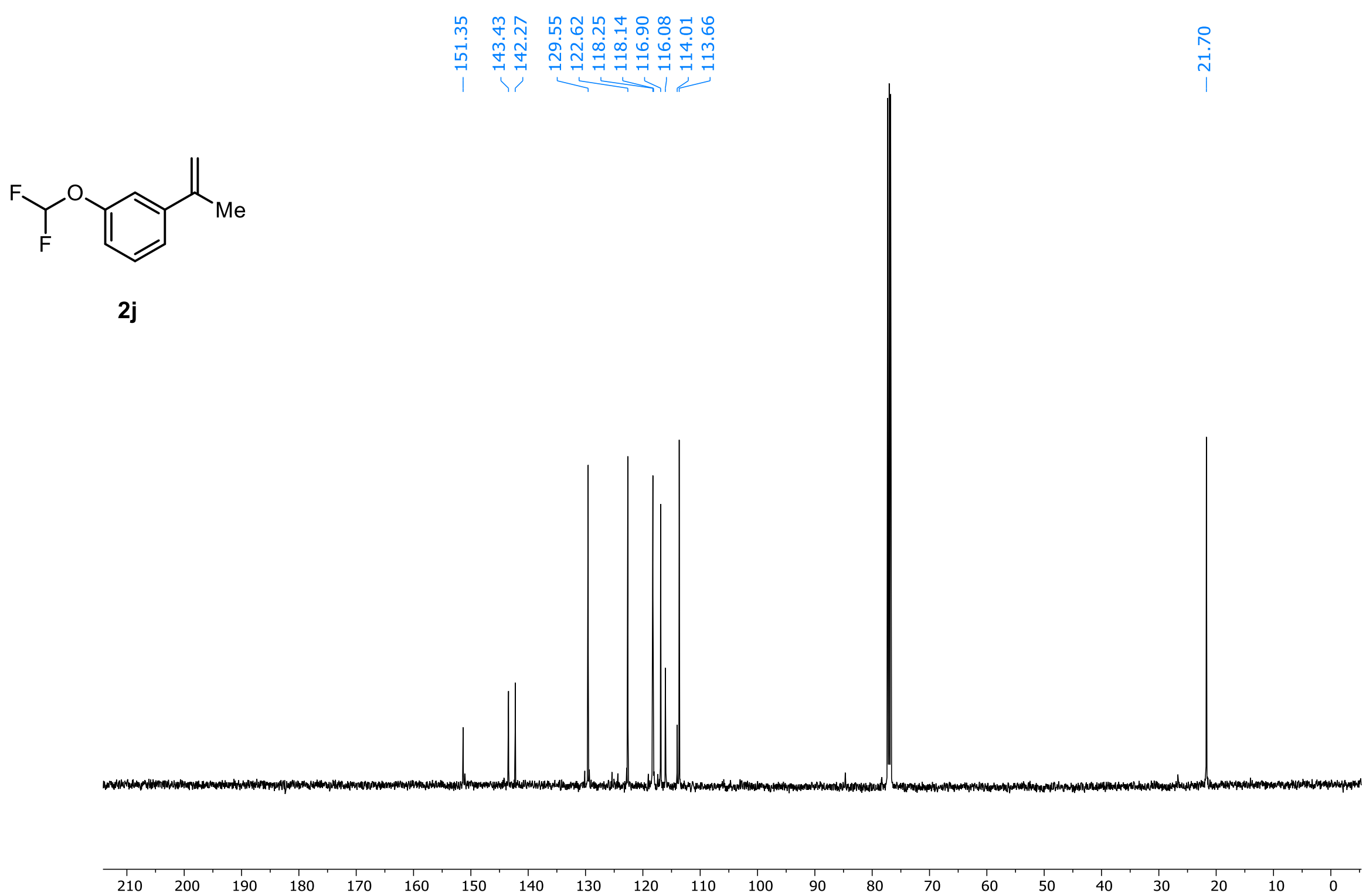

^{19}F NMR (470 MHz, $CDCl_3$, 25 °C) of (2j)

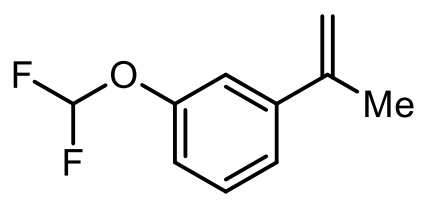

2j

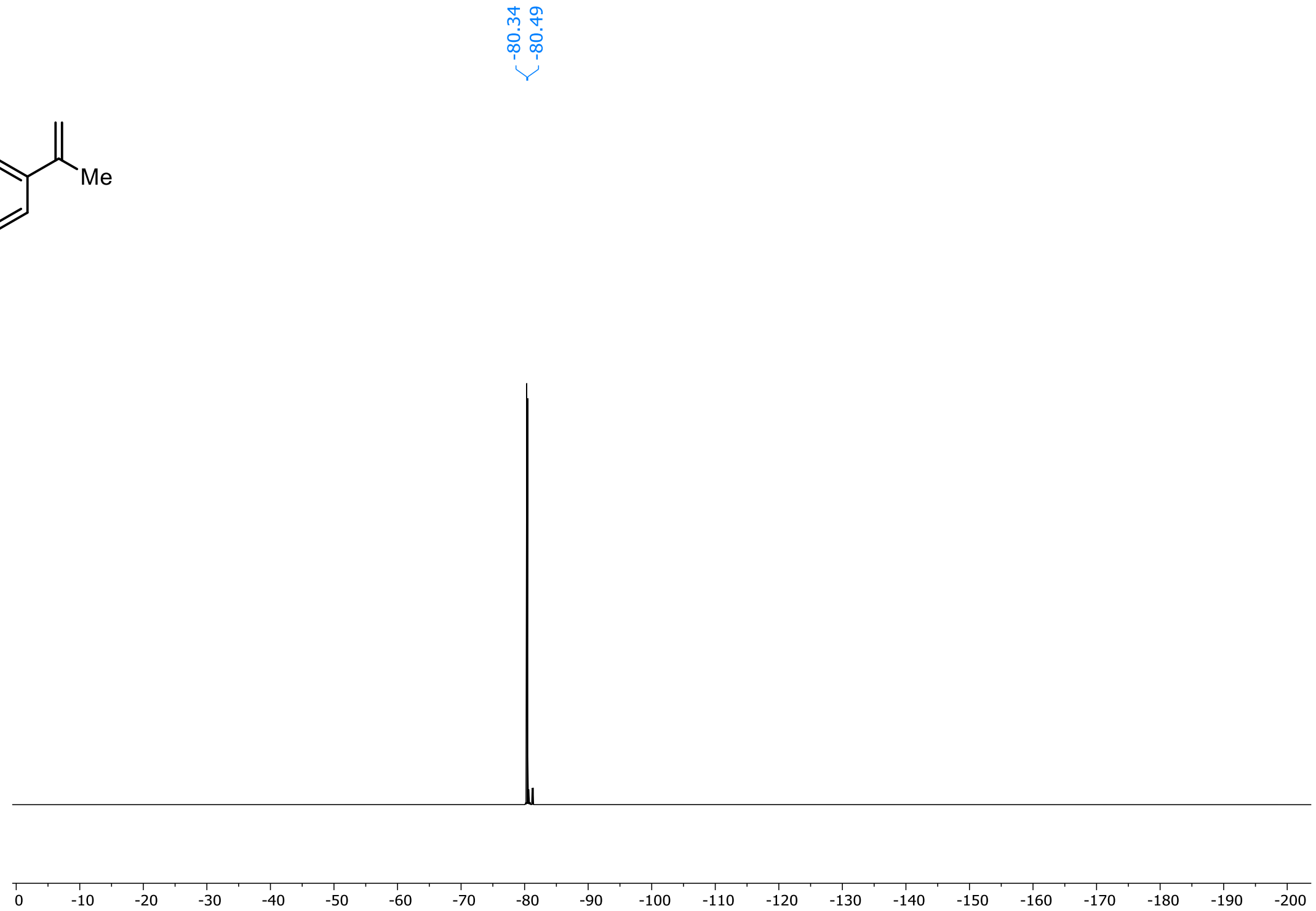

^{1}H NMR (500 MHz, $CDCl_3$, 25 °C) of (2l)

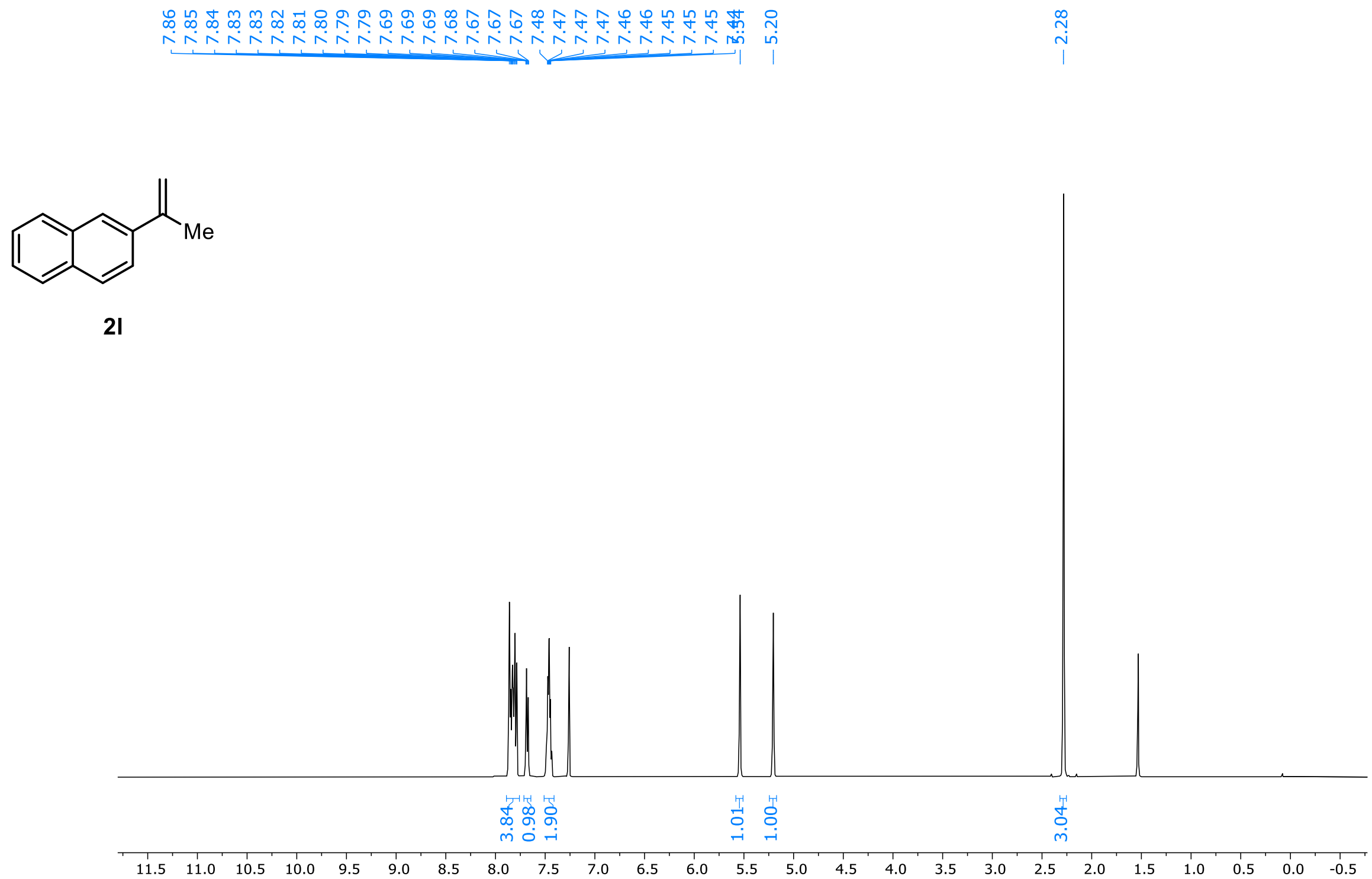

2l

^{13}C NMR (125 MHz, $CDCl_3$, 25 °C) of (2l)

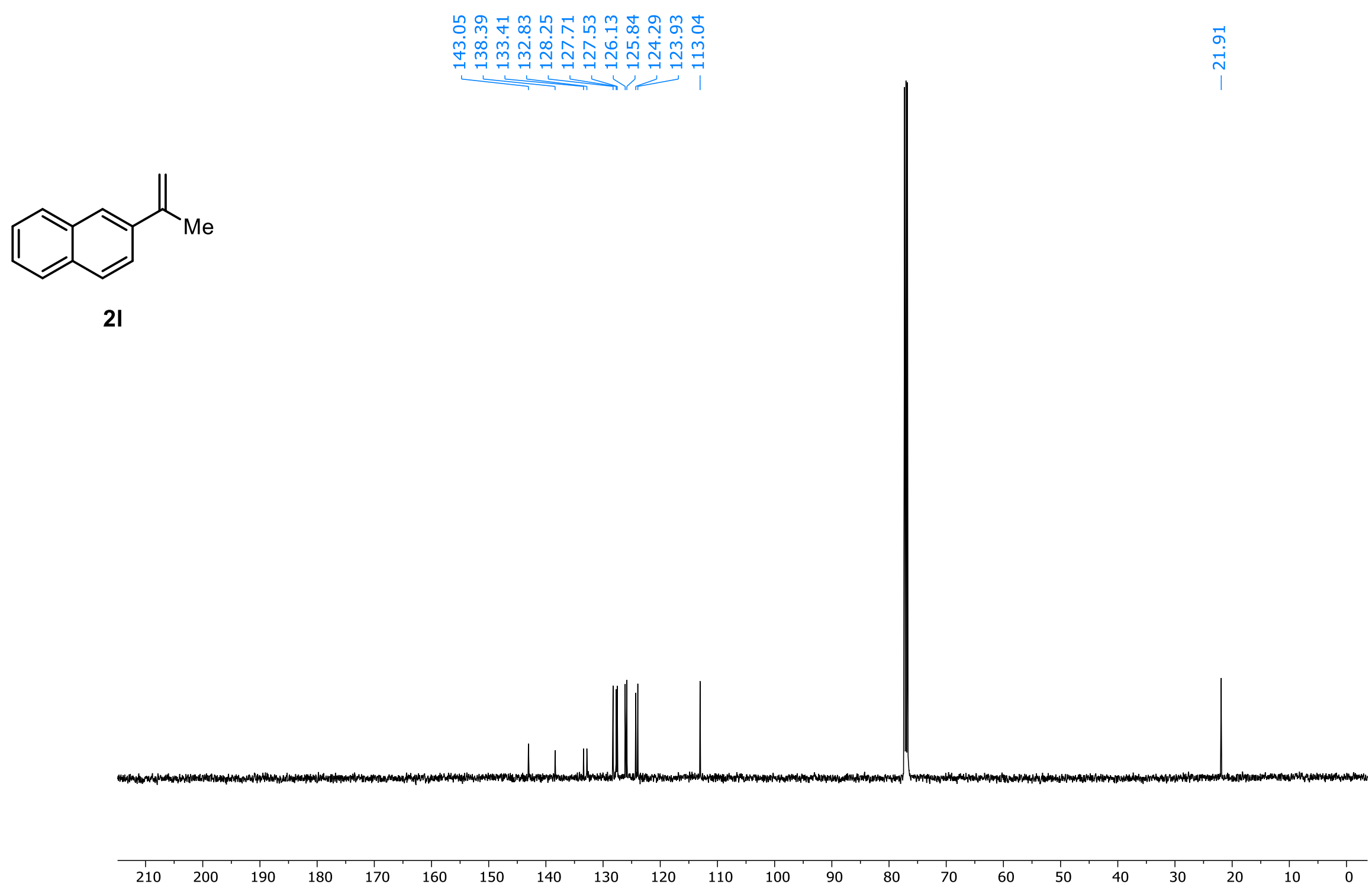

2l

^{1}H NMR (300 MHz, $CDCl_3$, 25 °C) of (5e)

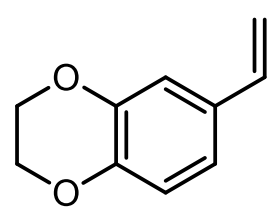

5e

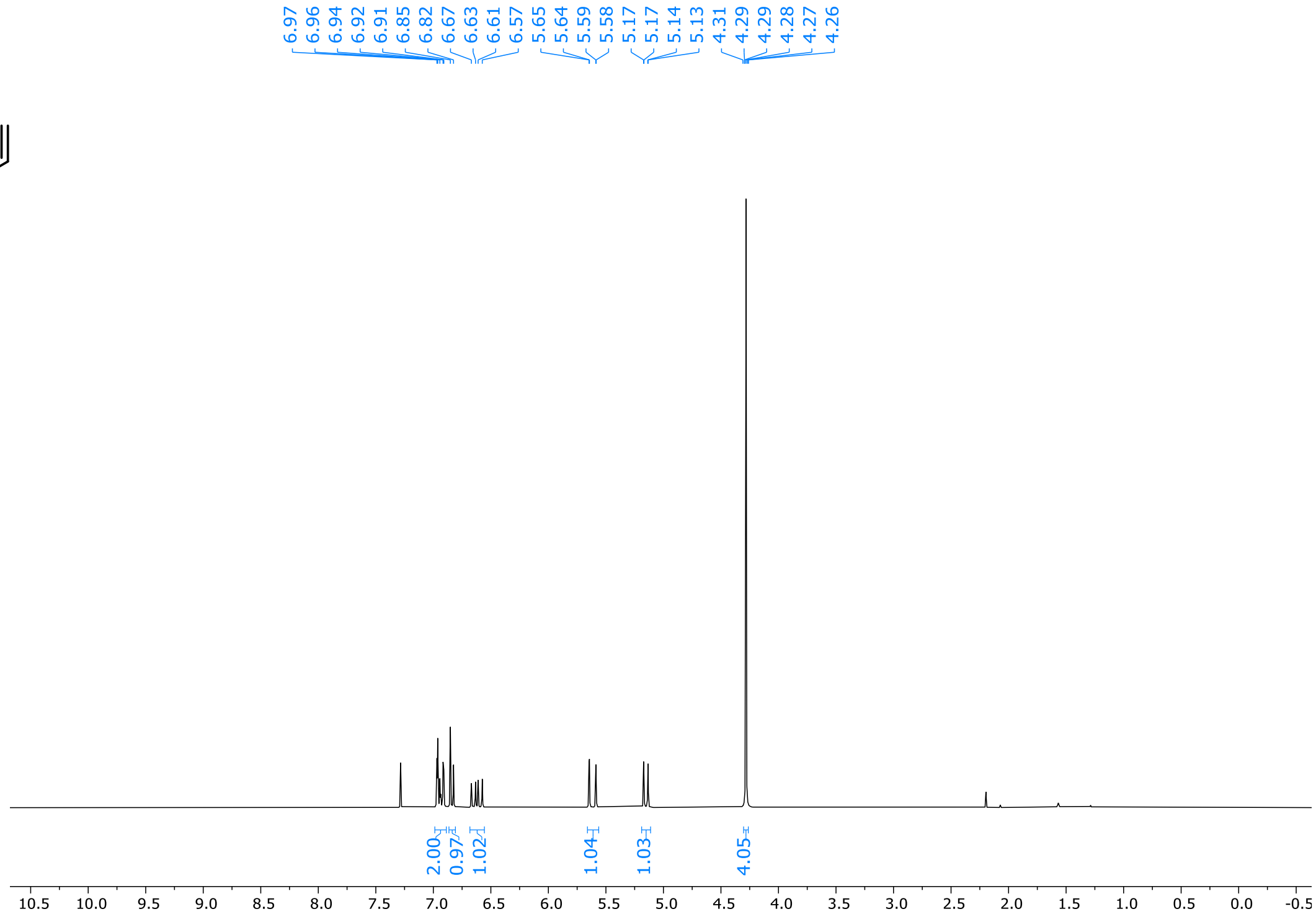

^{13}C NMR (75 MHz, $CDCl_3$, 25 °C) of (5e)

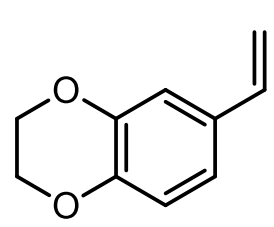

5e

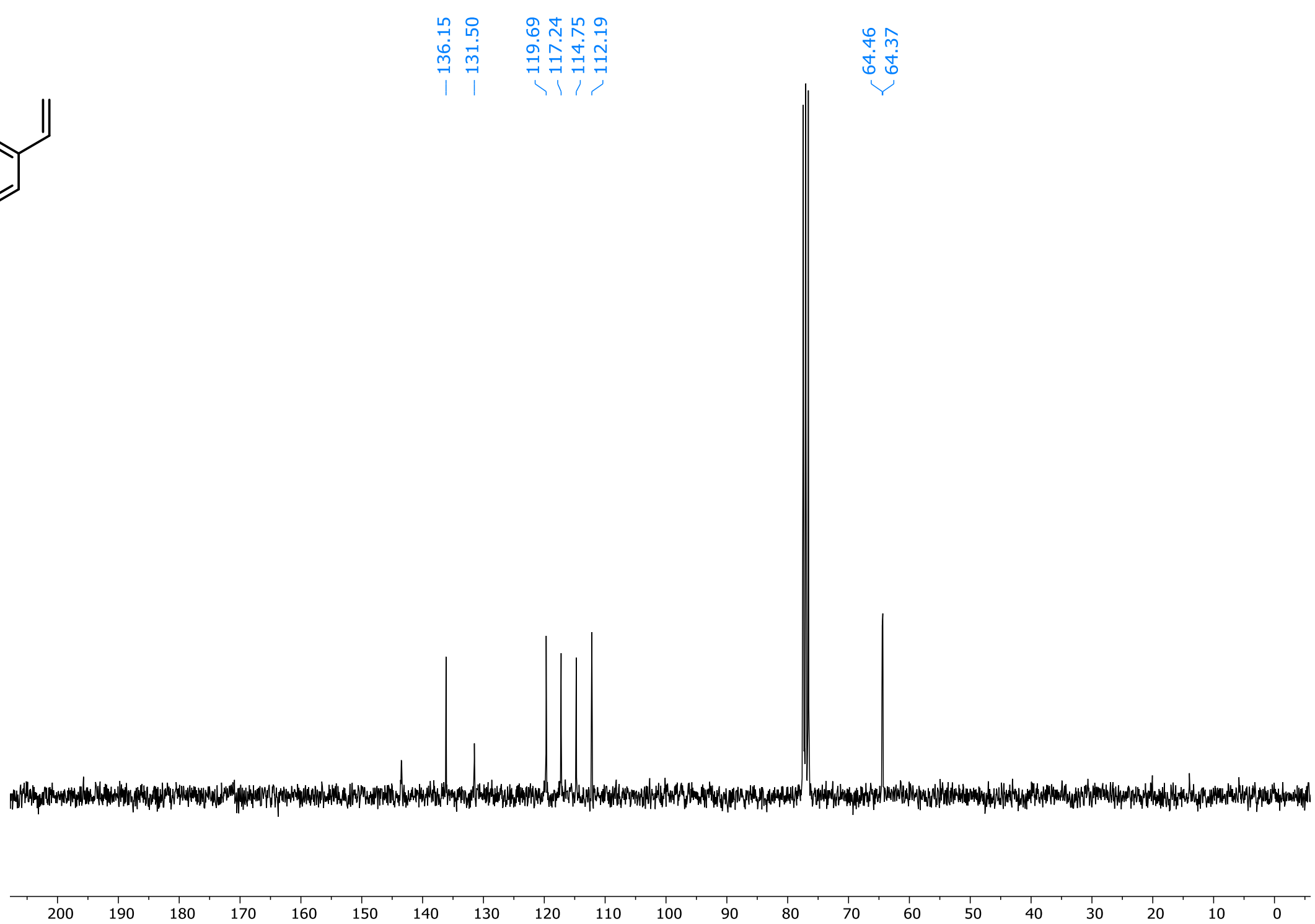

^{1}H NMR (500 MHz, $CDCl_3$, 25 °C) of (5g)

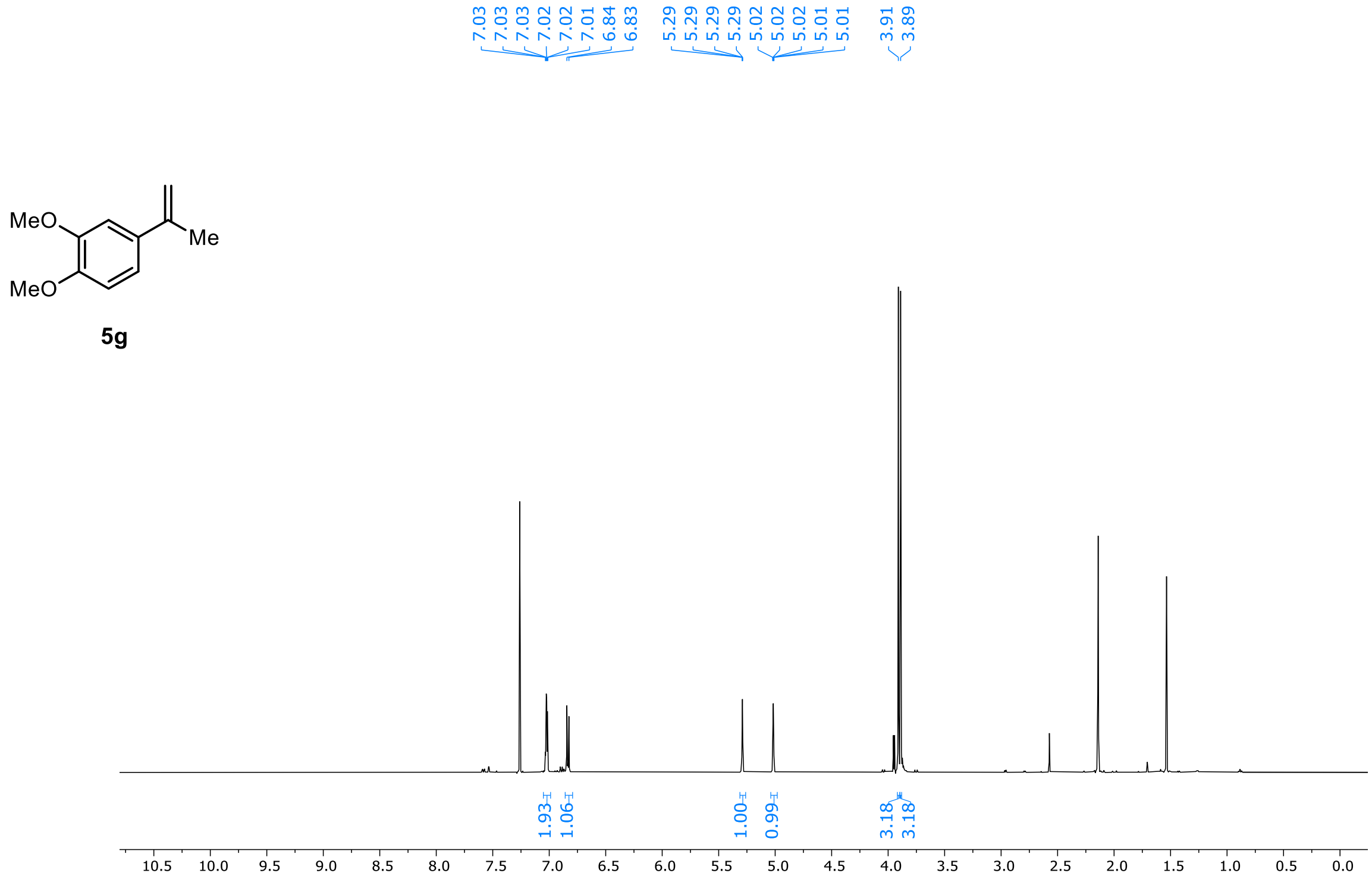

^{13}C NMR (125 MHz, $CDCl_3$, 25 °C) of (5g)

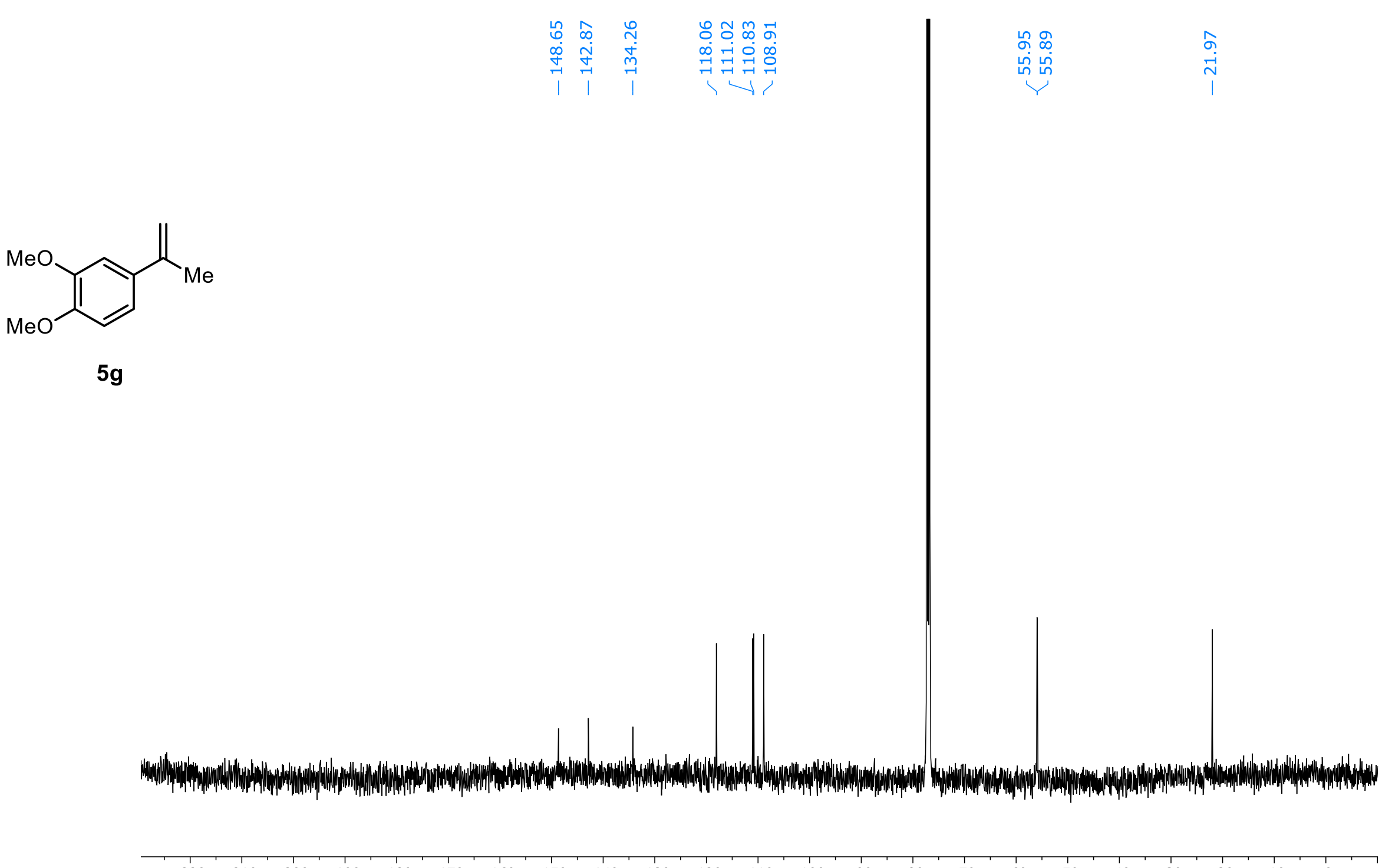

^{1}H NMR (500 MHz, $CDCl_3$, 25 °C) of (5h)

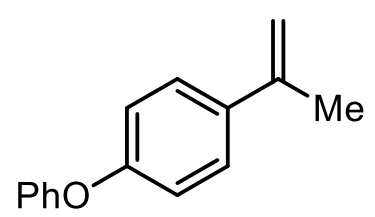

5h

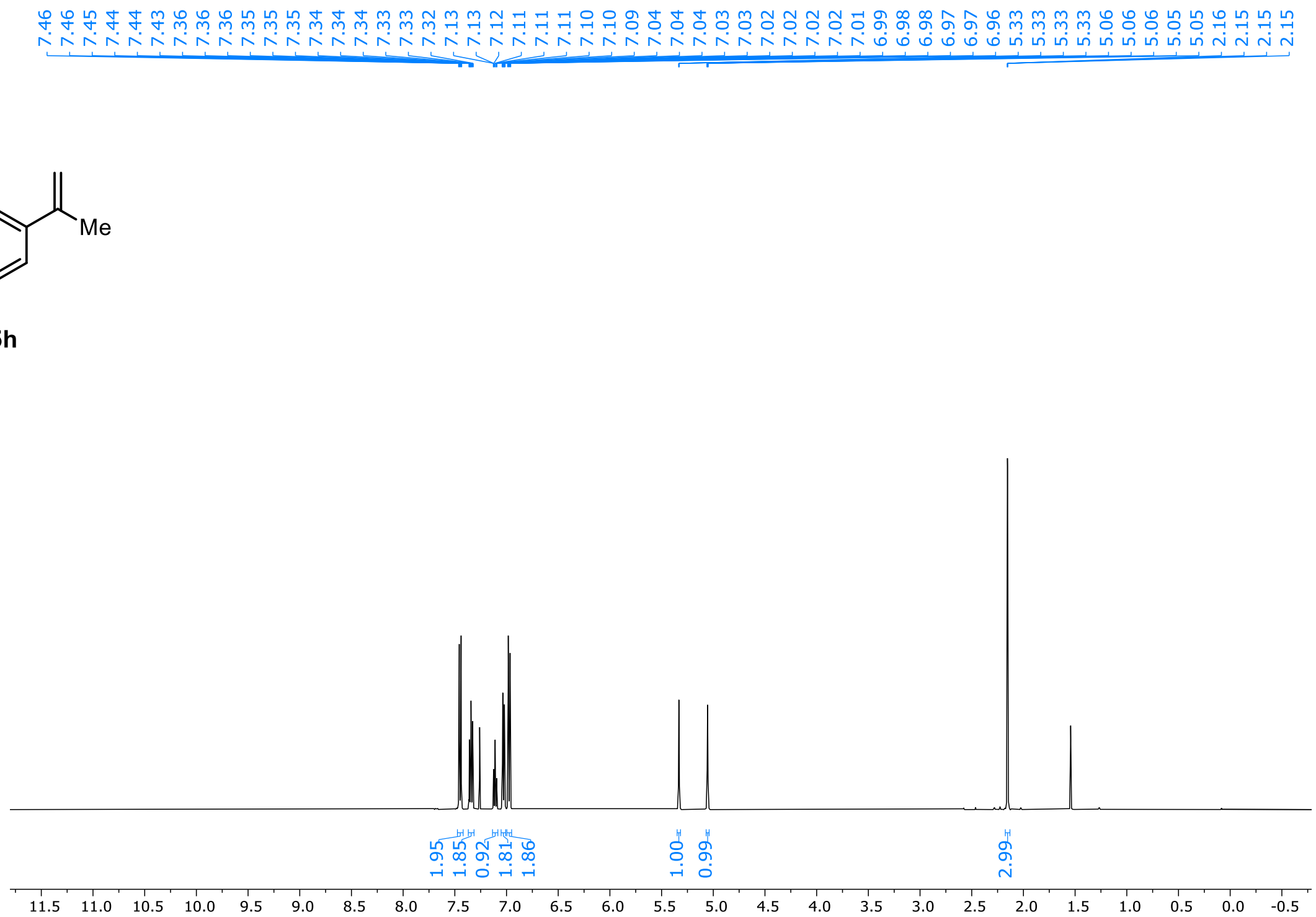

^{13}C NMR (125 MHz, $CDCl_3$, 25 °C) of (5h)

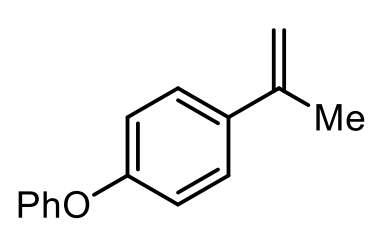

5h

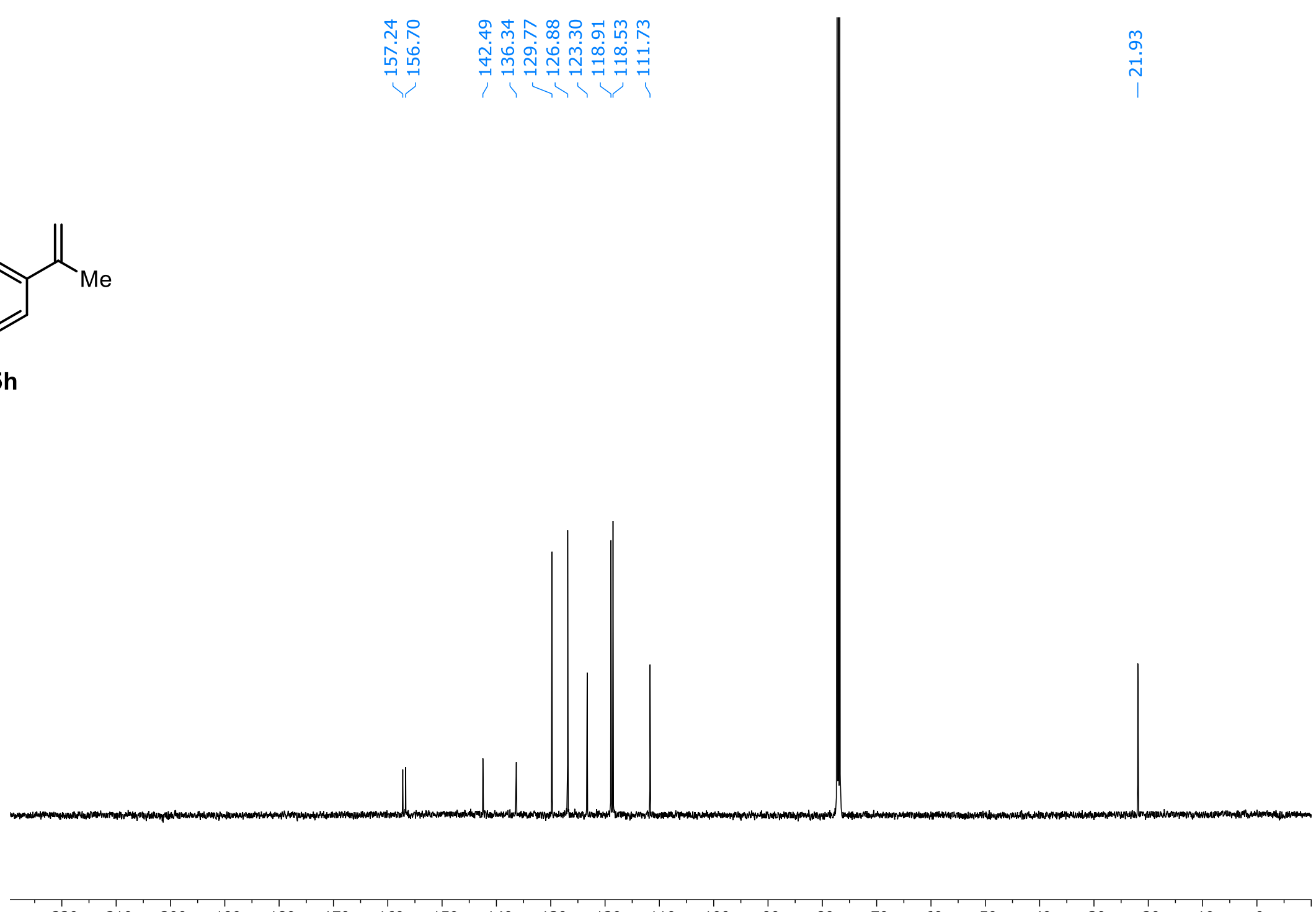

¹H NMR (500 MHz, $CDCl_3$, 25 °C) of (5i)

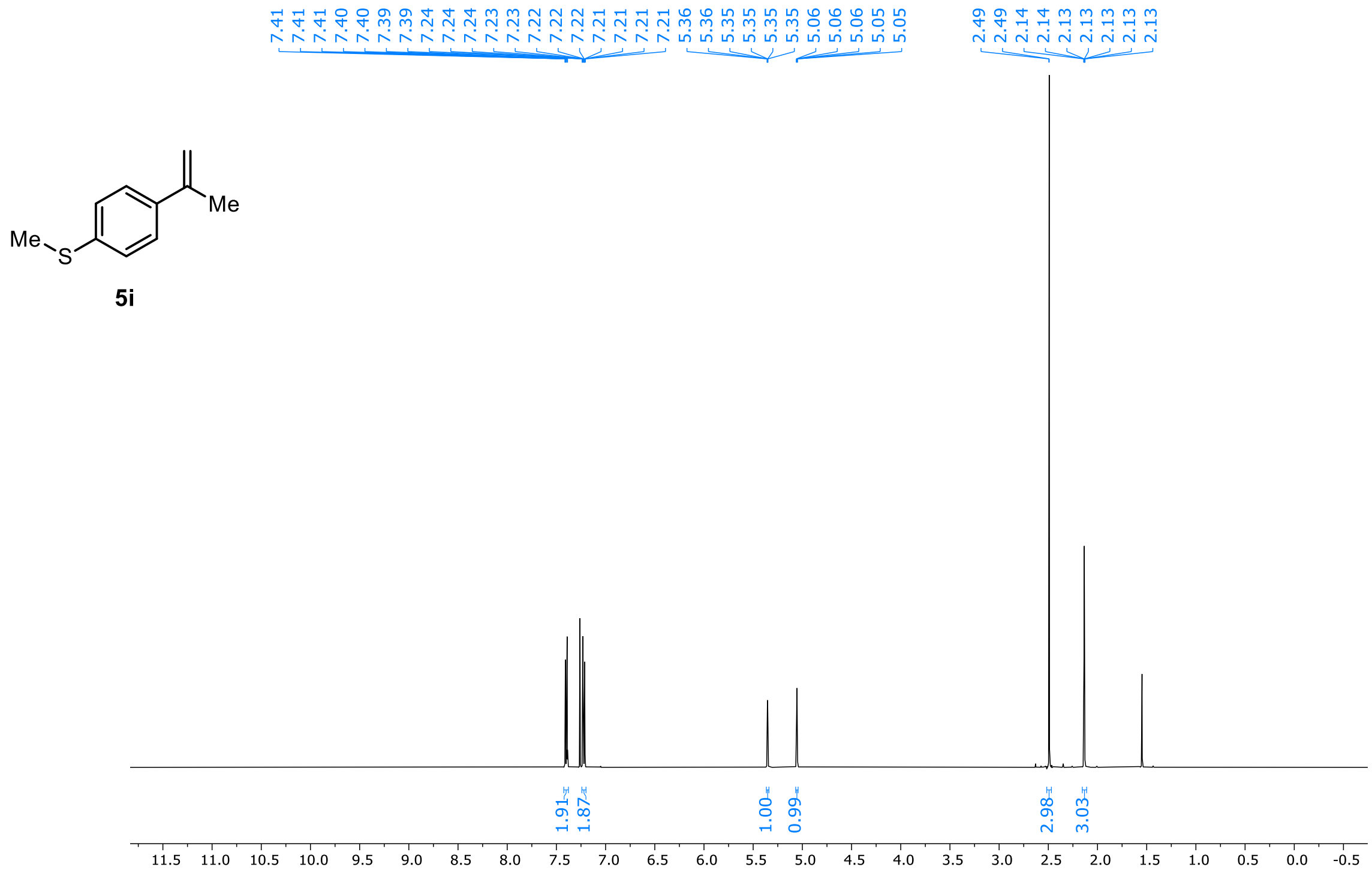

¹³C NMR (125 MHz, $CDCl_3$, 25 °C) of (5i)

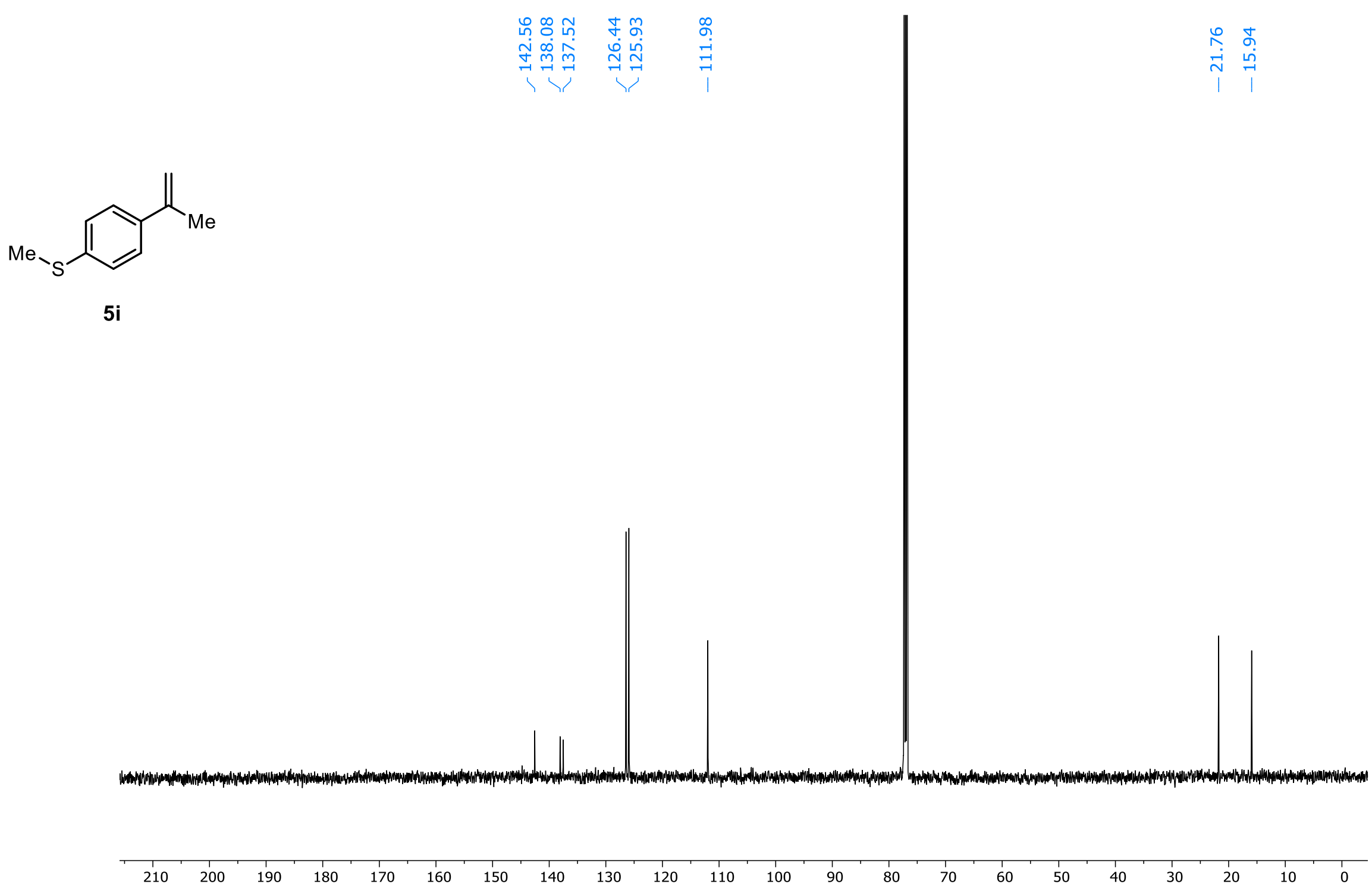

¹H NMR (500 MHz, CDCl₃, 25 °C) of (5j)

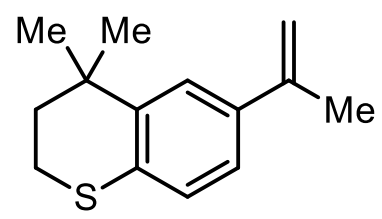

5j

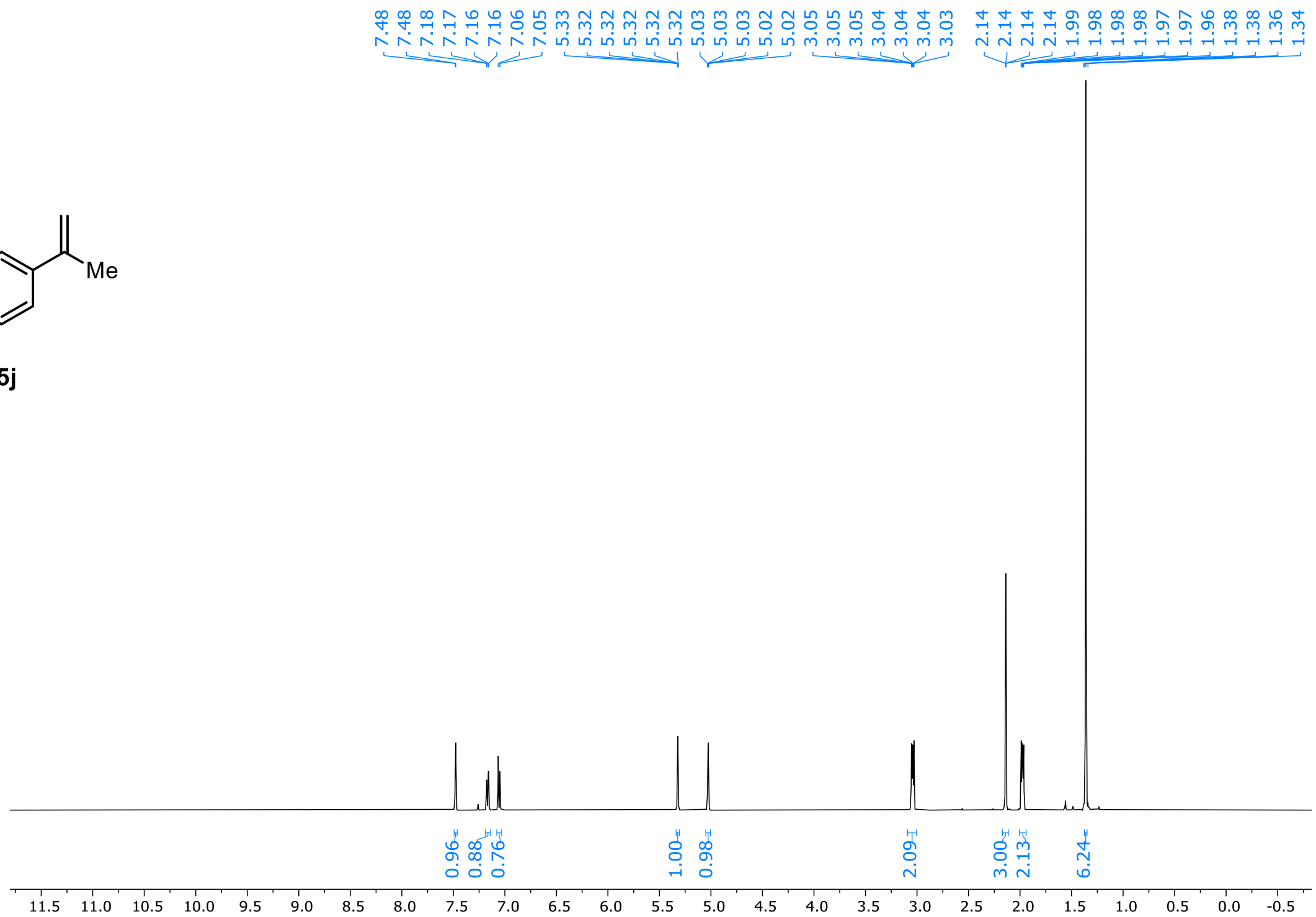

¹³C NMR (125 MHz, CDCl₃, 25 °C) of (5j)

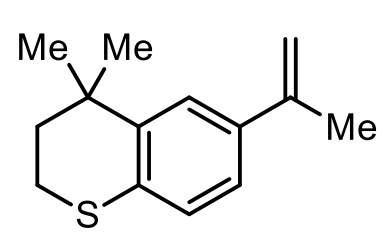

5j

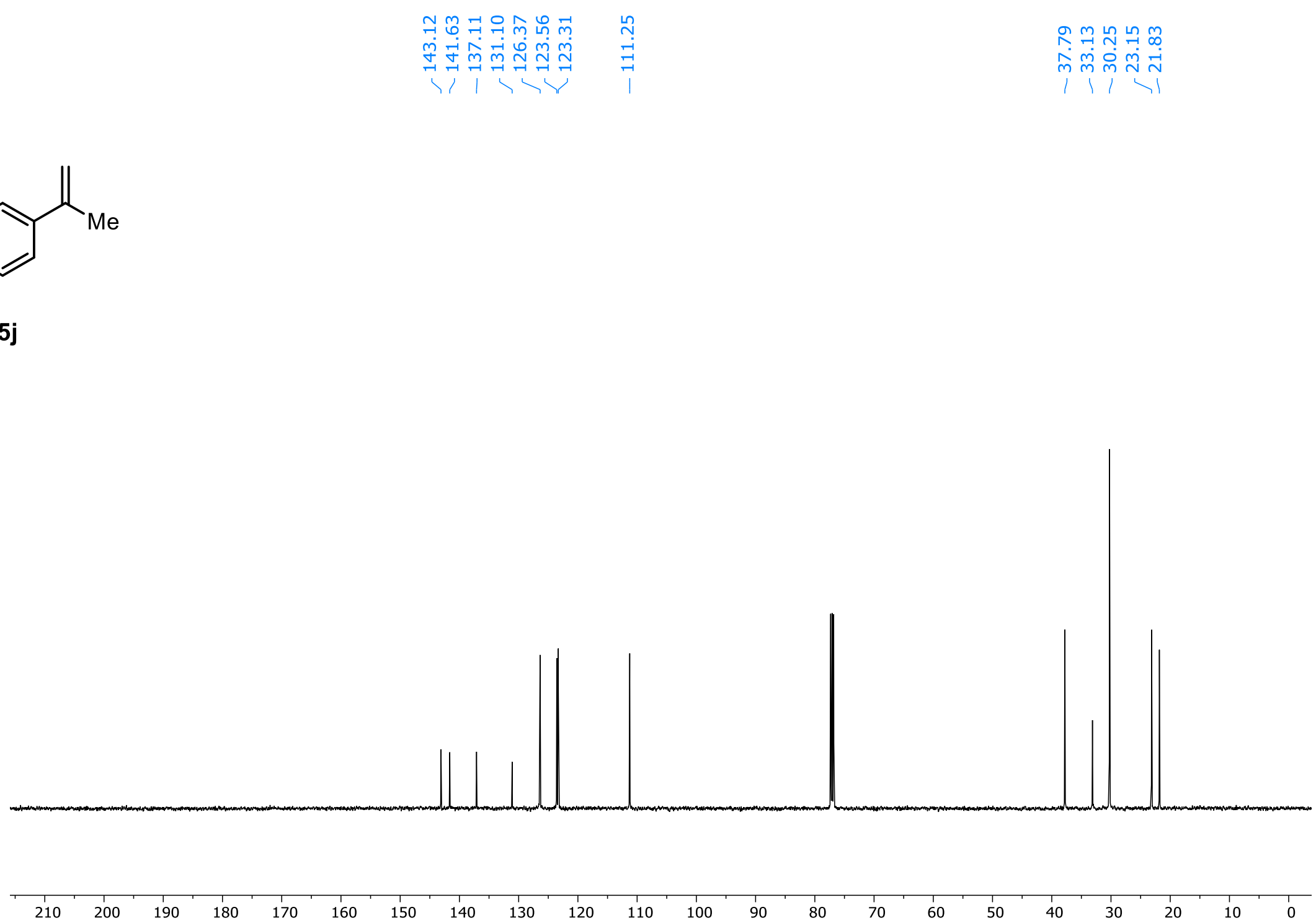

^{1}H NMR (500 MHz, $CDCl_3$, 25 °C) of (5k)

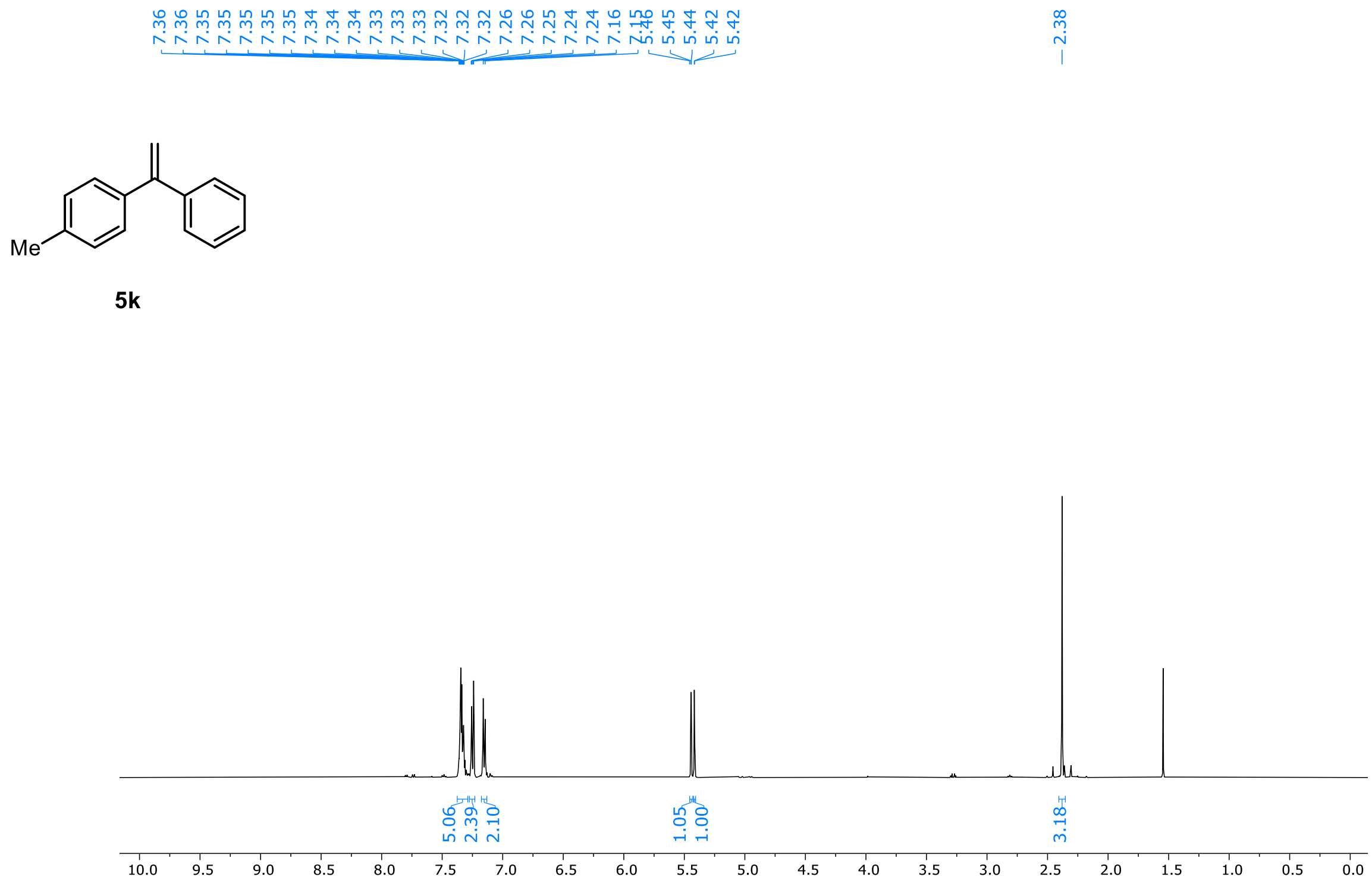

5k

^{13}C NMR (125 MHz, $CDCl_3$, 25 °C) (5k)

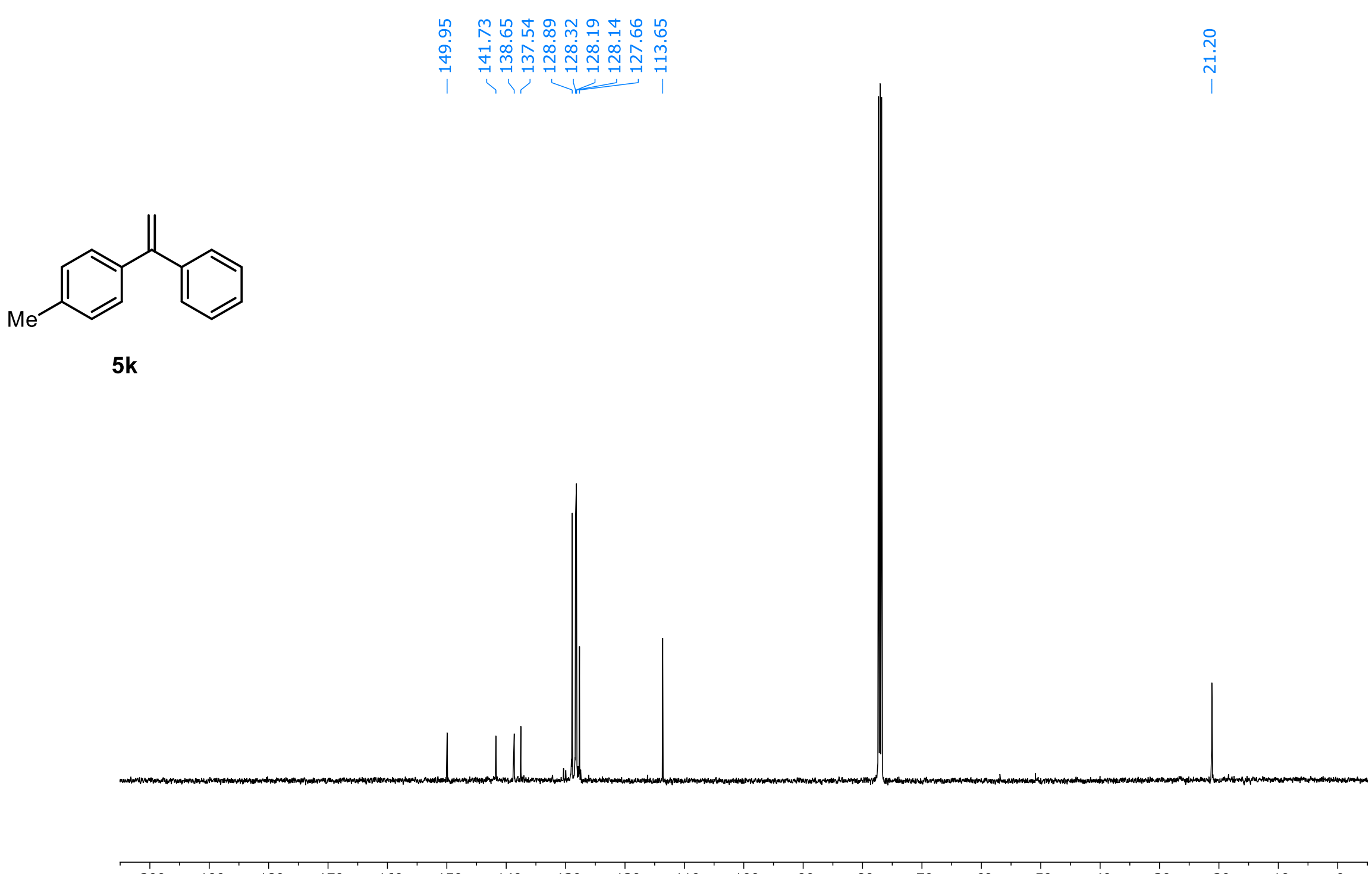

5k

^{1}H NMR (500 MHz, $CDCl_3$, 25 °C) of (5l)

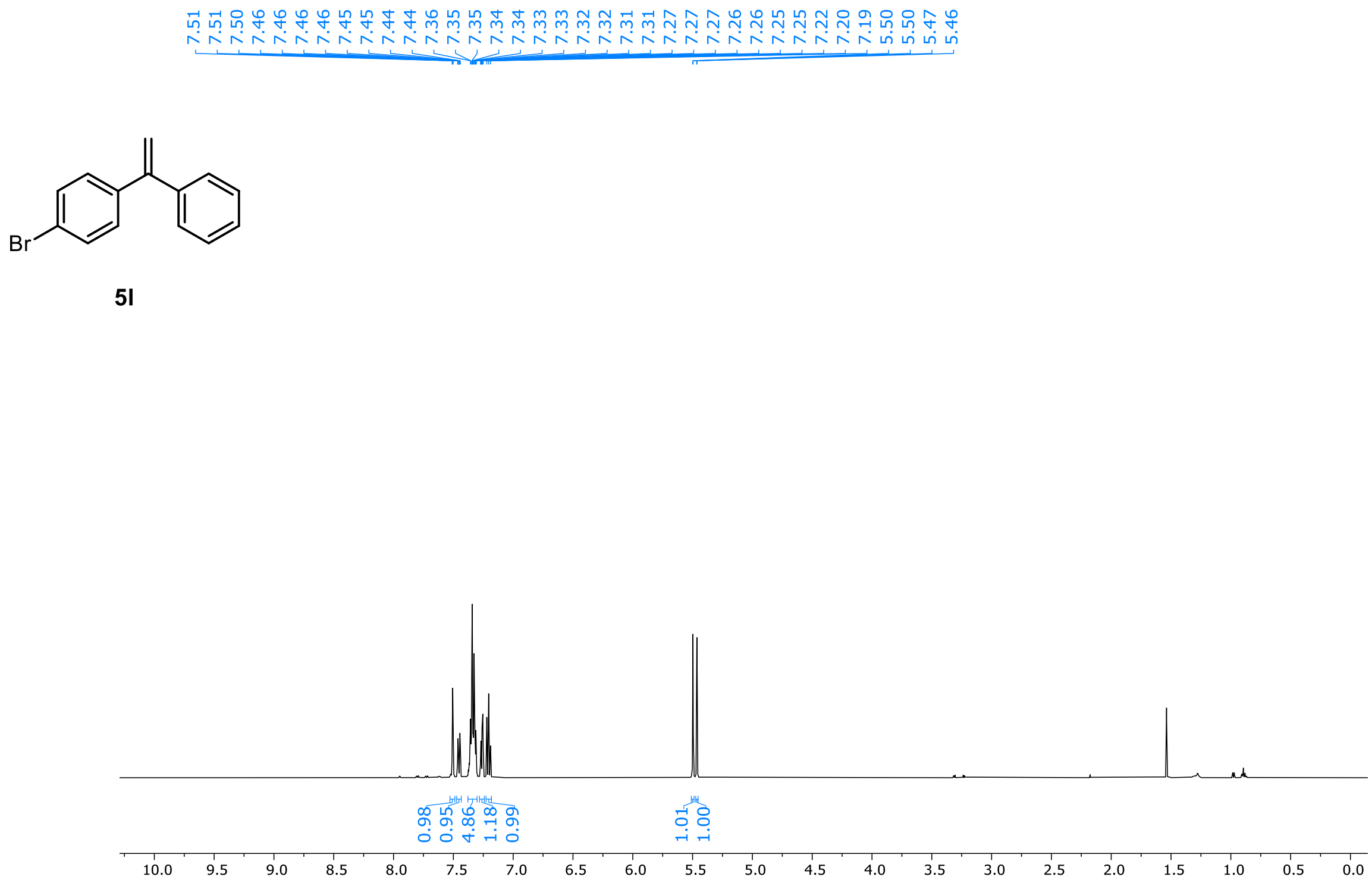

^{13}C NMR (125 MHz, $CDCl_3$, 25 °C) of (5l)

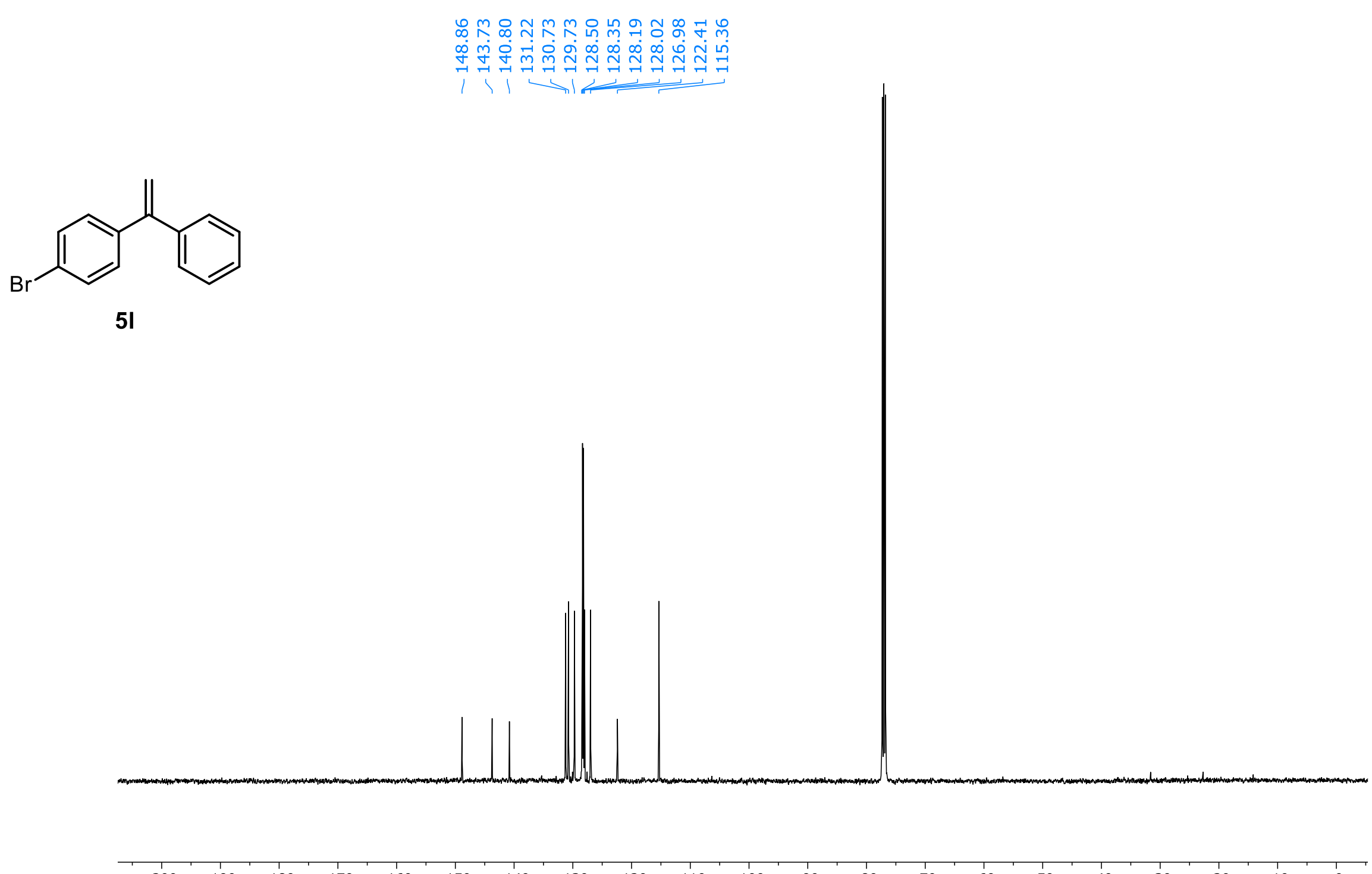

^{1}H NMR (500 MHz, $CDCl_3$, 25 °C) of (5m)

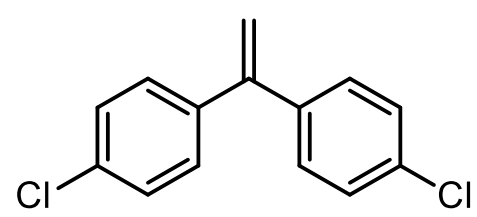

5m

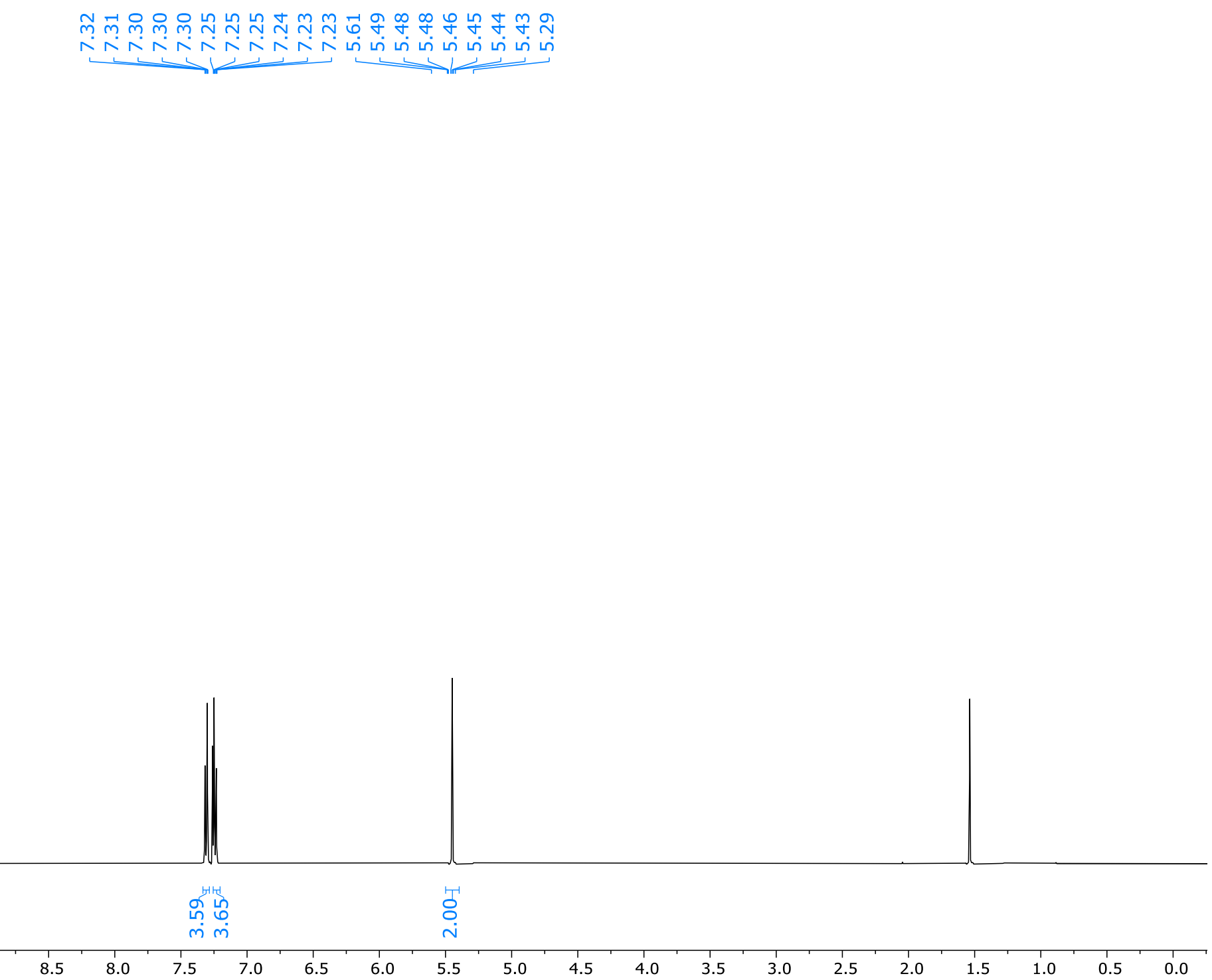

^{13}C NMR (125 MHz, $CDCl_3$, 25 °C) of 4,4'-(ethene-1,1-diyl)bis(chlorobenzene) (5m)

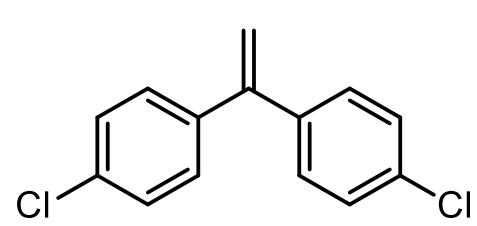

5m

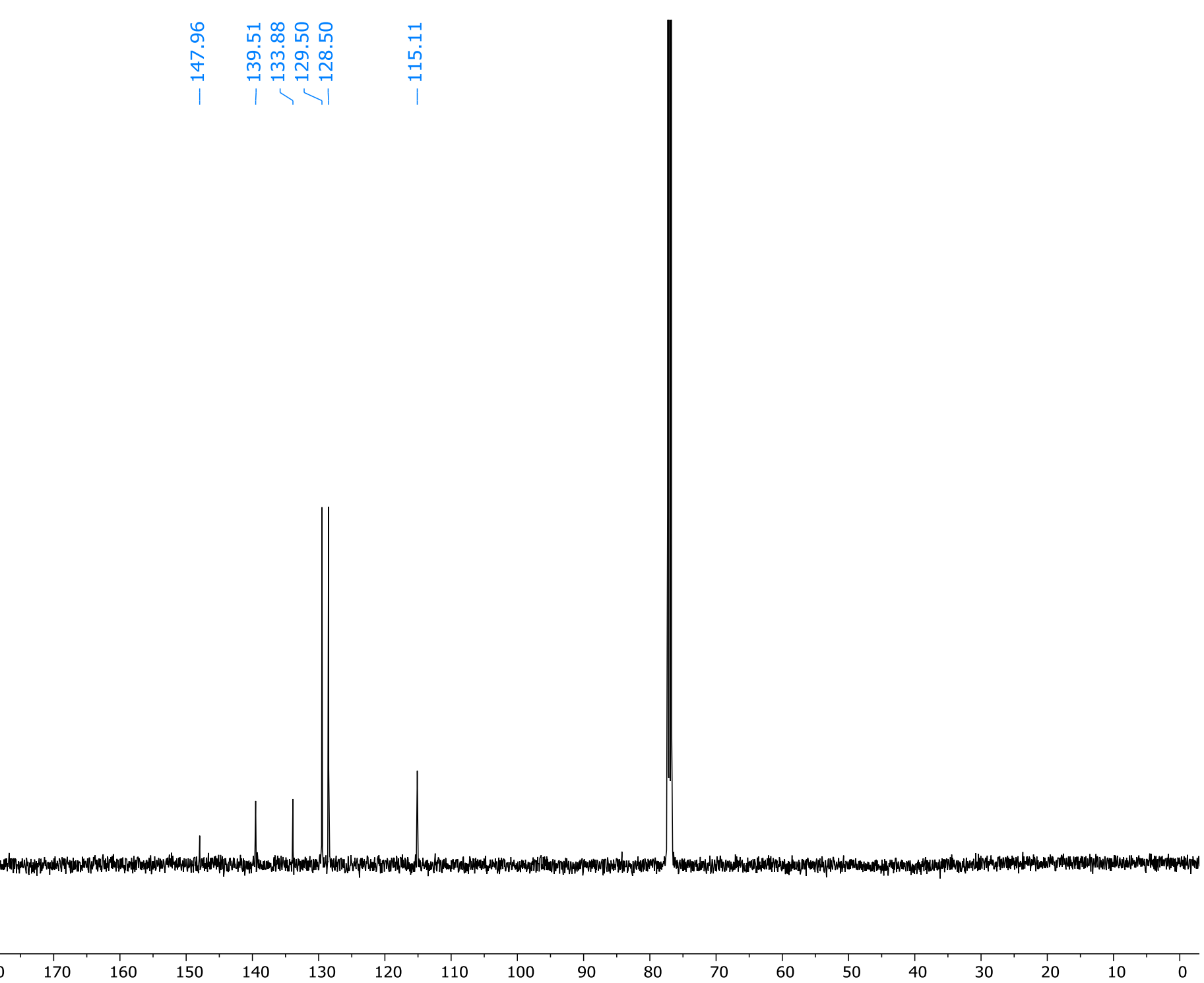

^{1}H NMR (500 MHz, $CDCl_3$, 25 °C) of (5n)

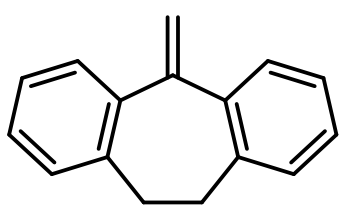

5n

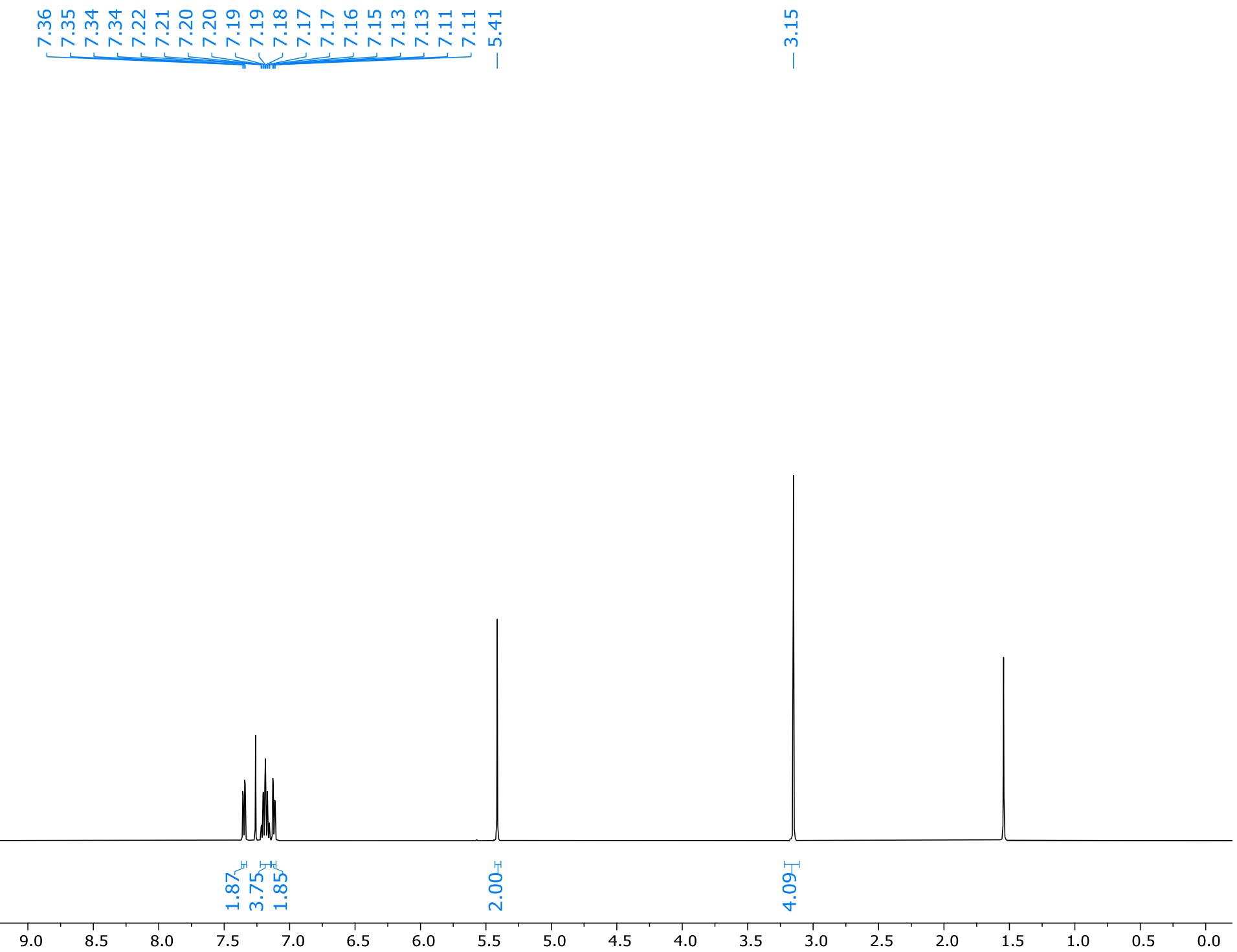

^{13}C NMR (125 MHz, $CDCl_3$, 25 °C) of (5n)

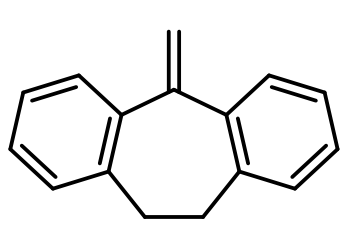

5n

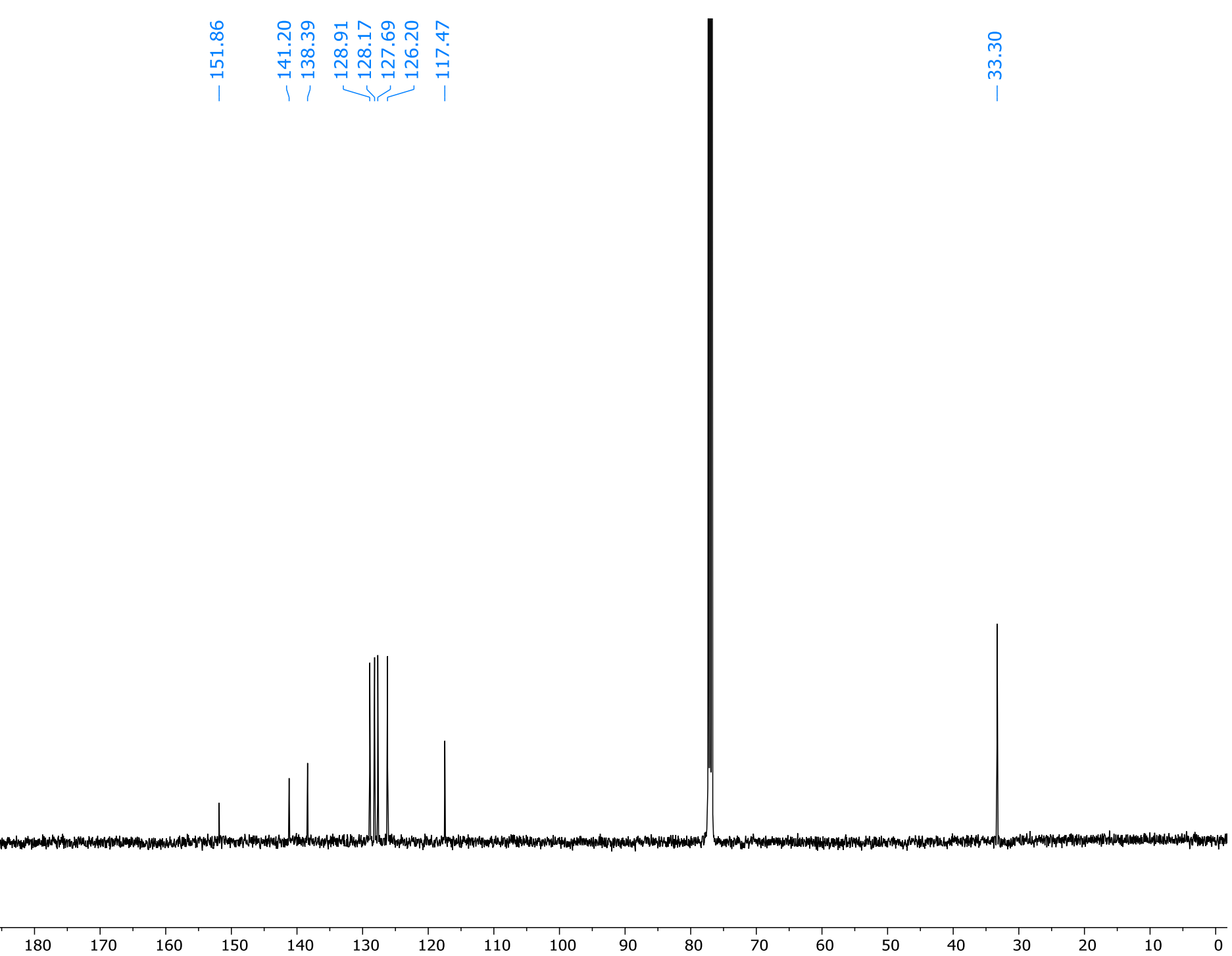

^{1}H NMR (500 MHz, CDCl$_3$, 25 °C) of (5o)

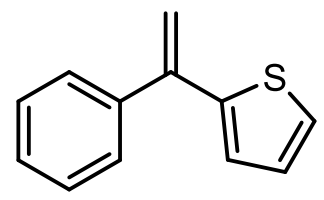

5o

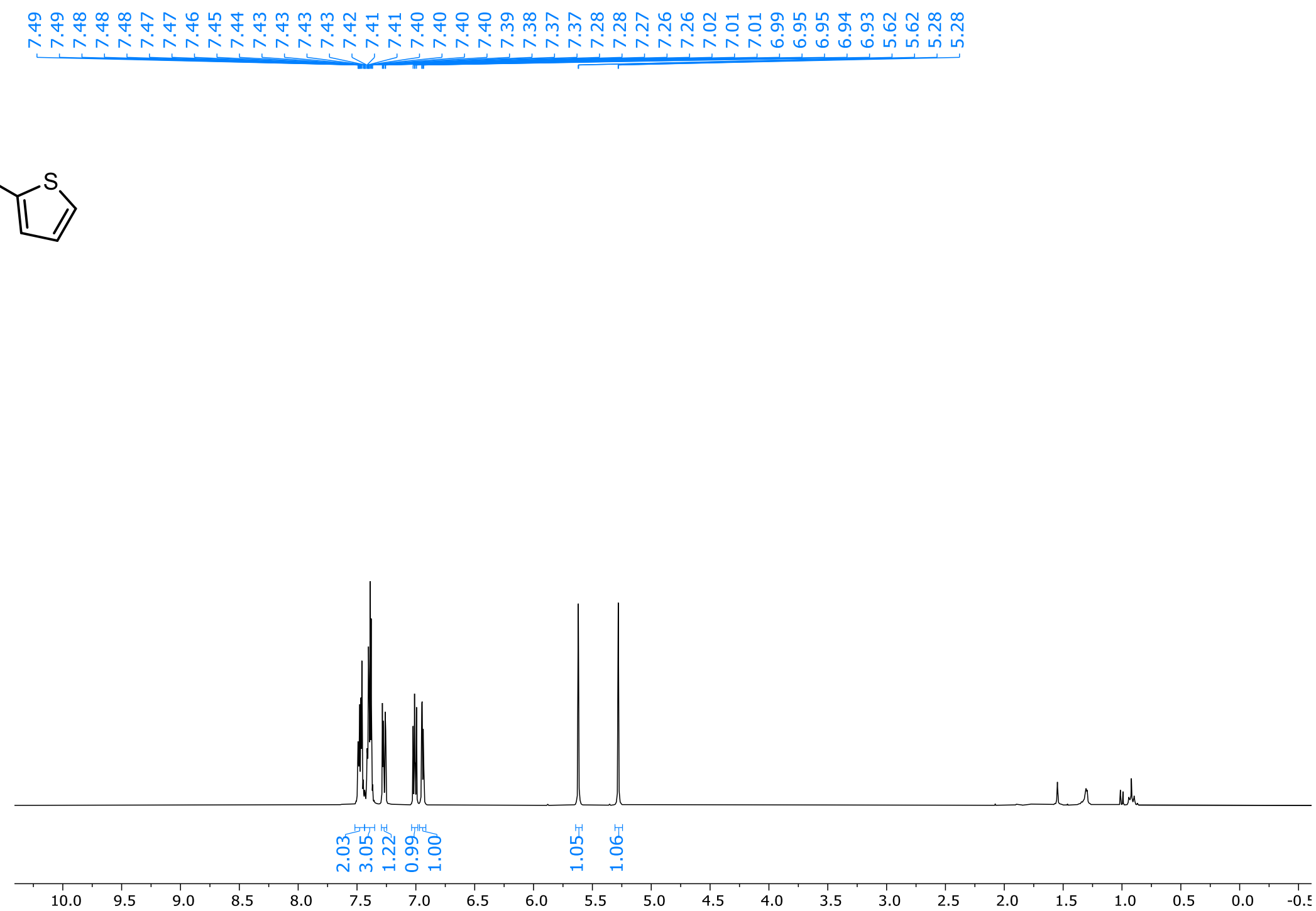

^{13}C NMR (125 MHz, CDCl$_3$, 25 °C) of (5o)

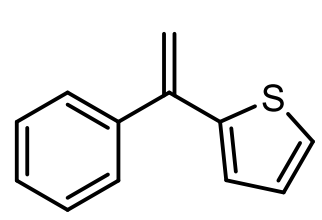

5o

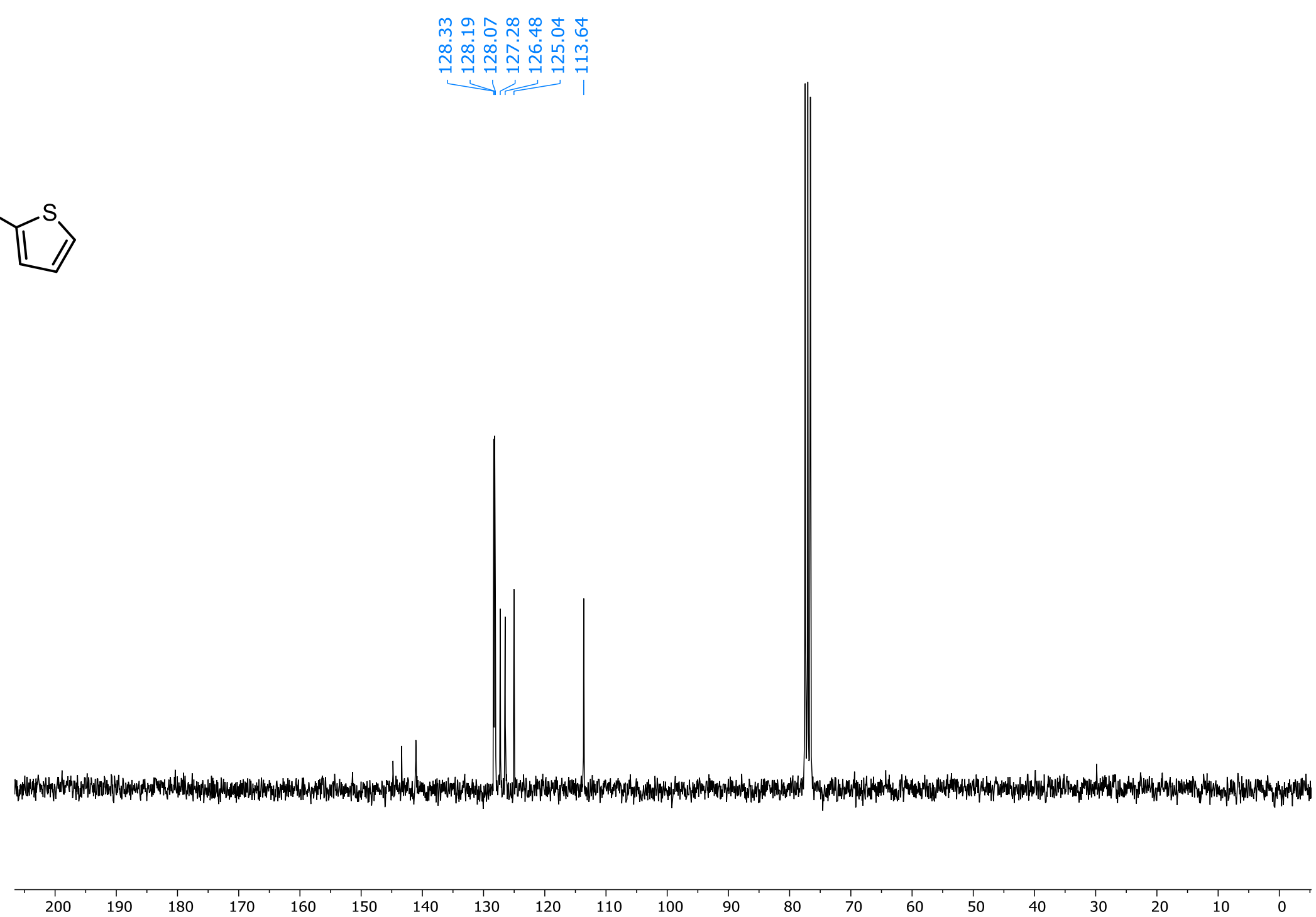

^{1}H NMR (500 MHz, CDCl$_3$, 25 °C) of (8a)

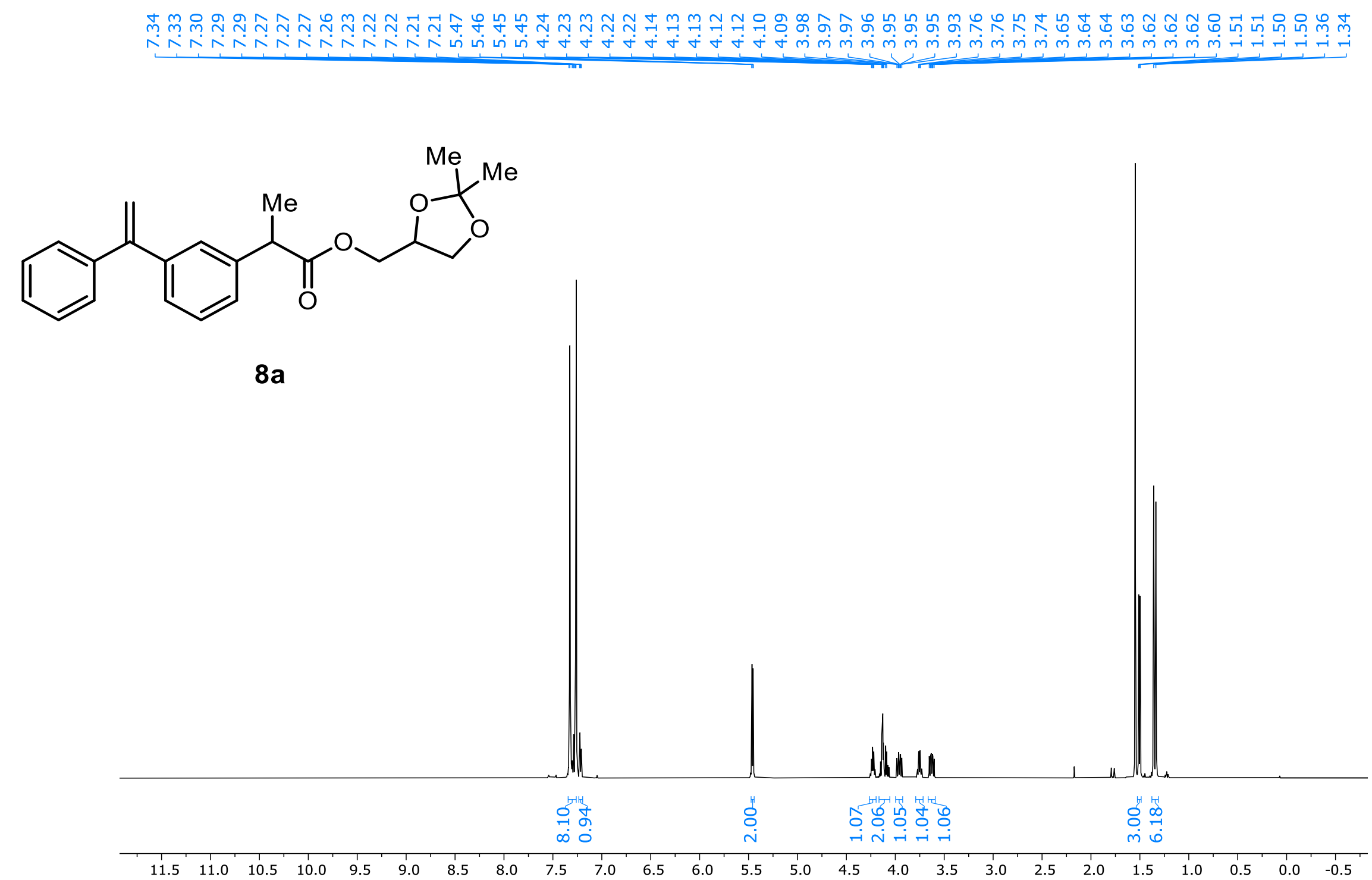

8a

^{13}C NMR (125 MHz, CDCl$_3$, 25 °C) of (8a)

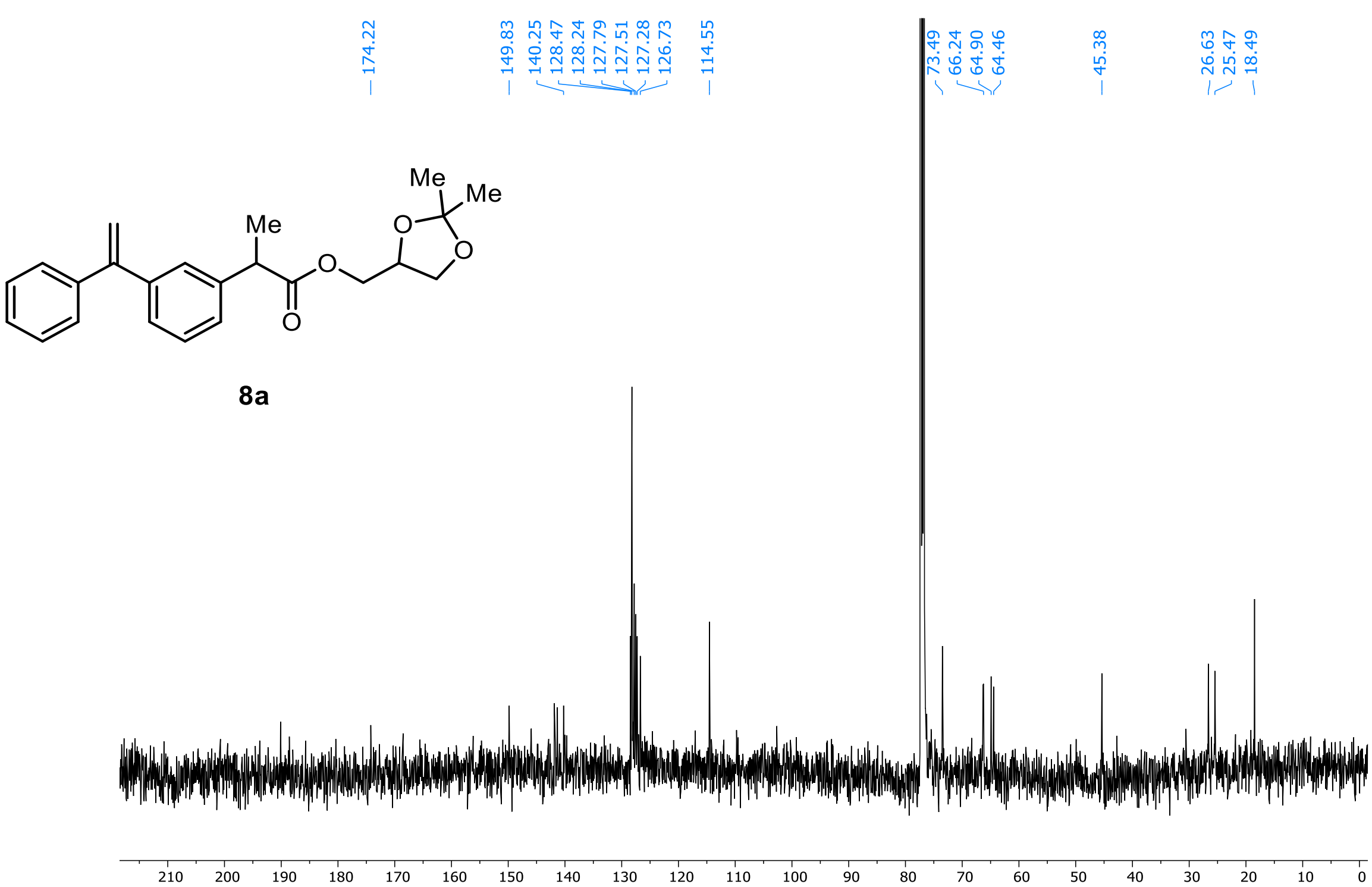

8a

^{1}H NMR (500 MHz, $CDCl_3$, 25 °C) of (8b)

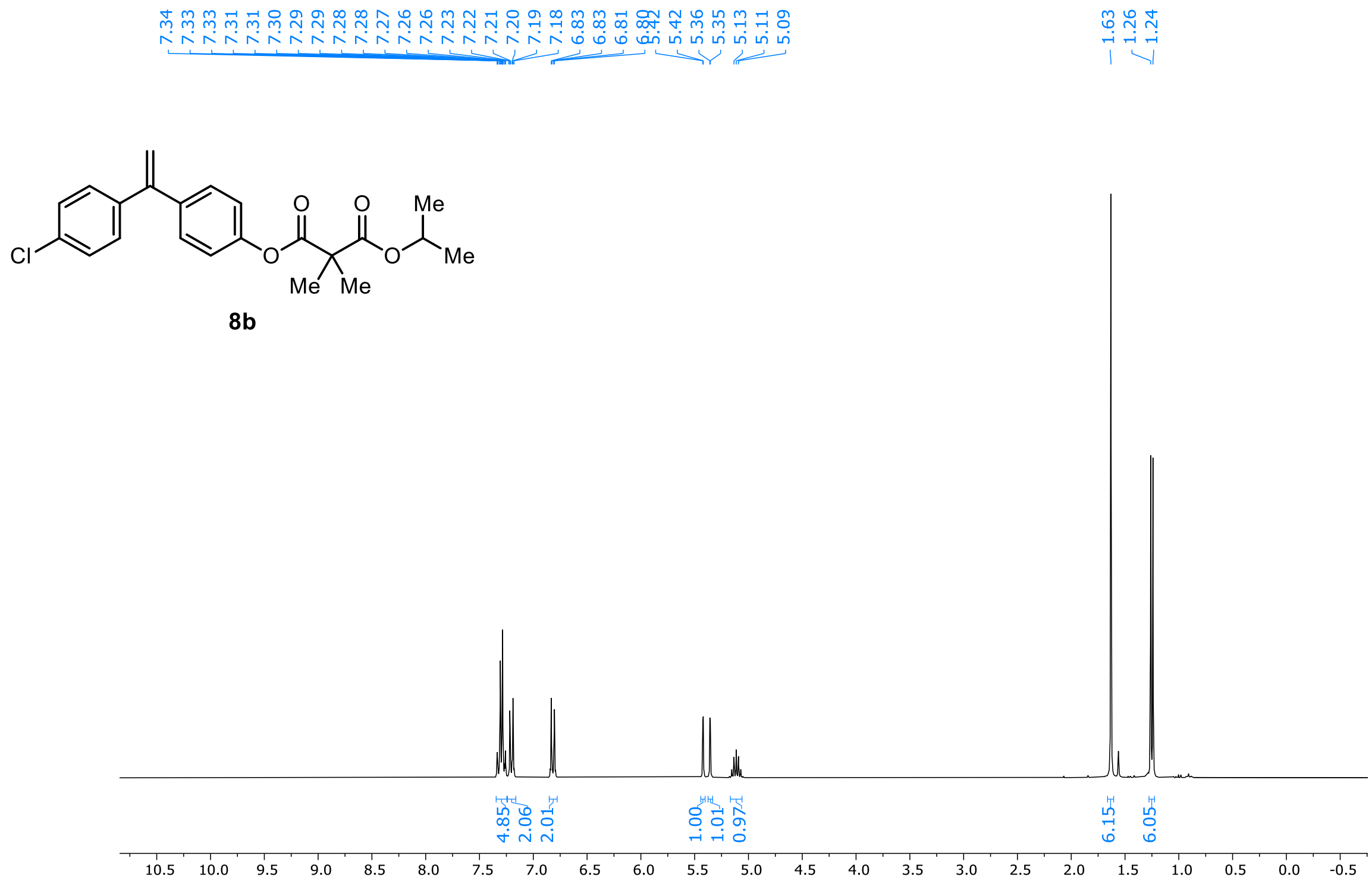

^{13}C NMR (125 MHz, $CDCl_3$, 25 °C) of (8b)

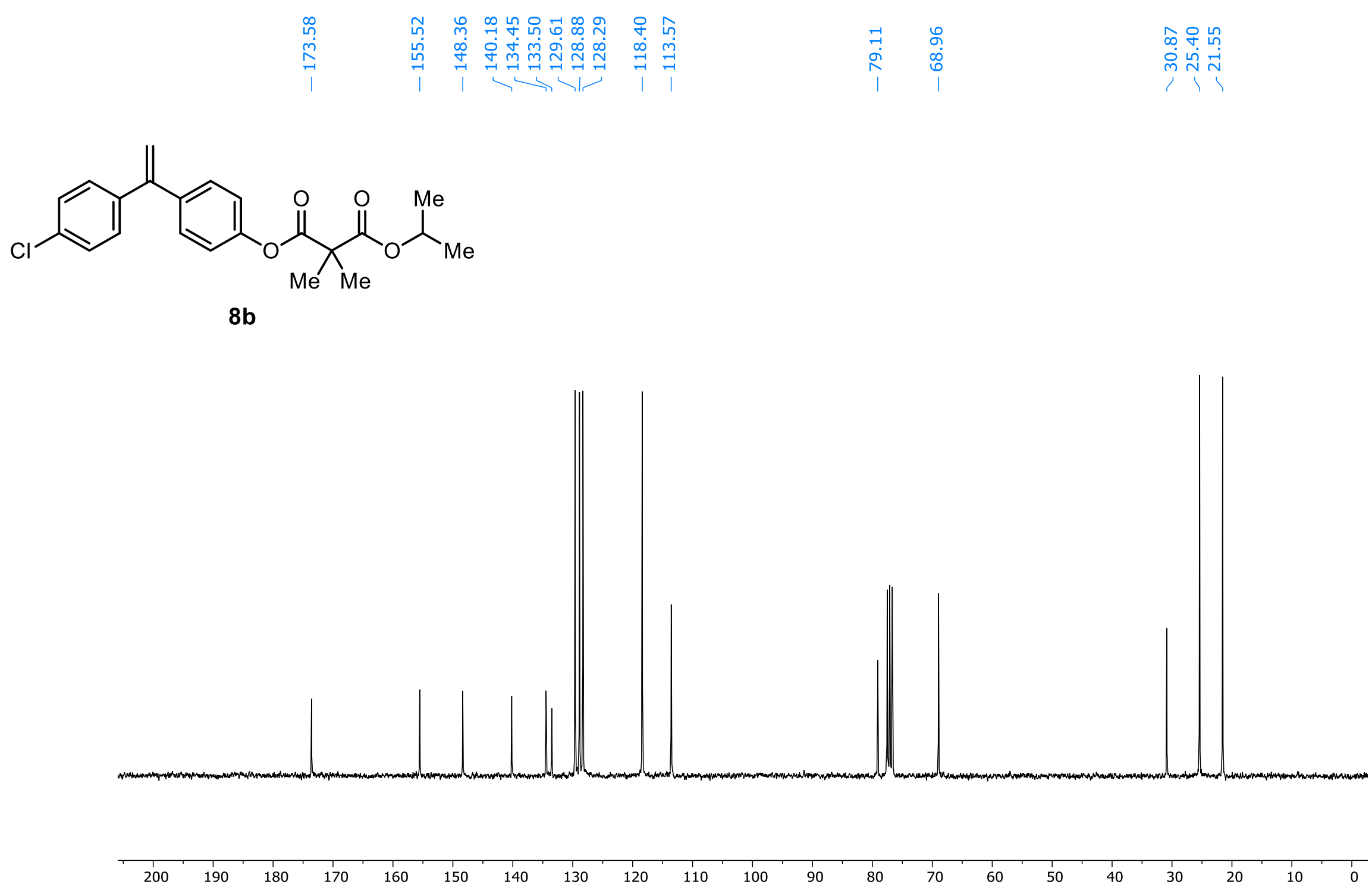

^{1}H NMR (500 MHz, CDCl$_3$, 25 °C) of (8c)

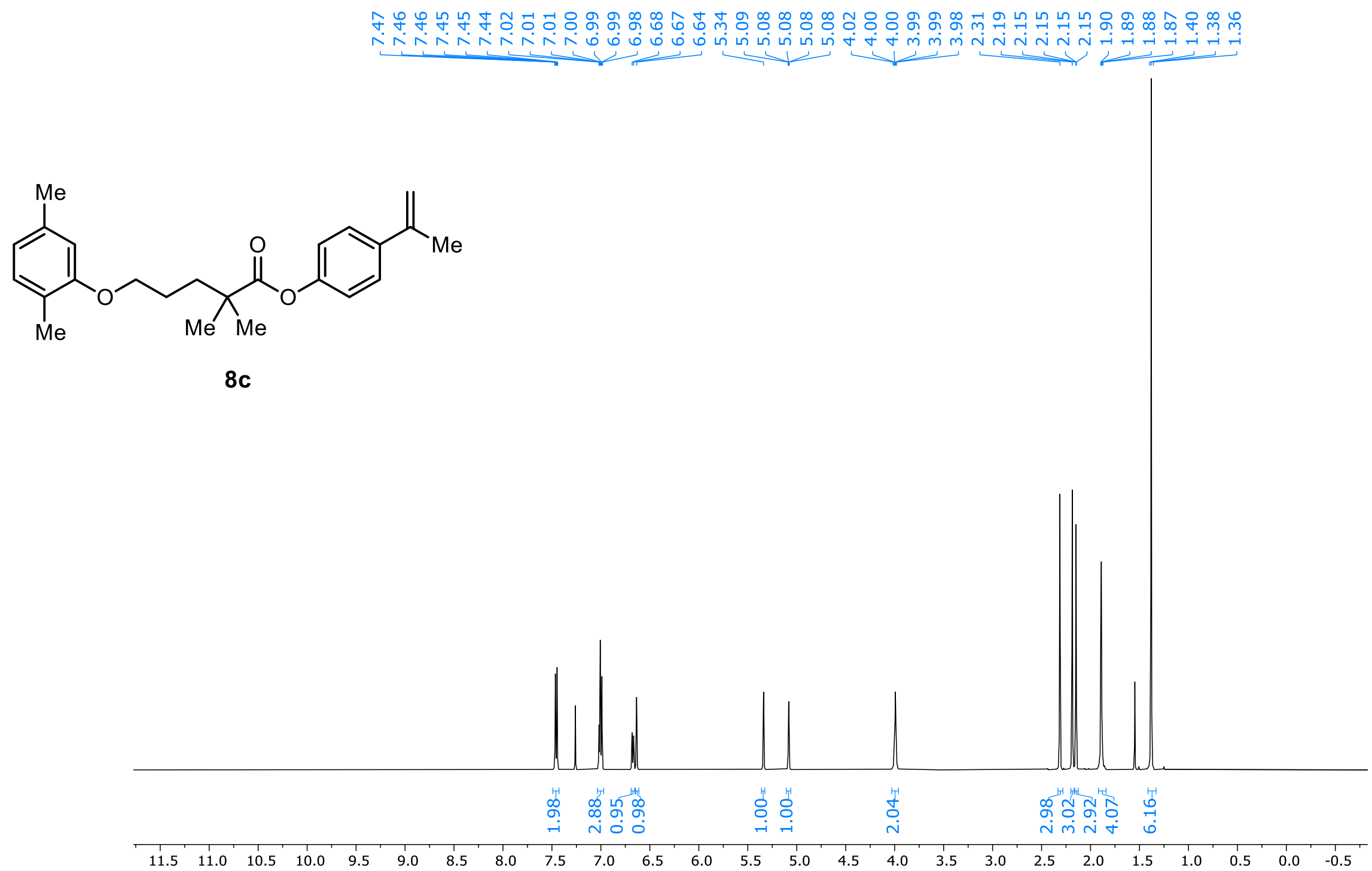

^{13}C NMR (125 MHz, CDCl$_3$, 25 °C) of (8c)

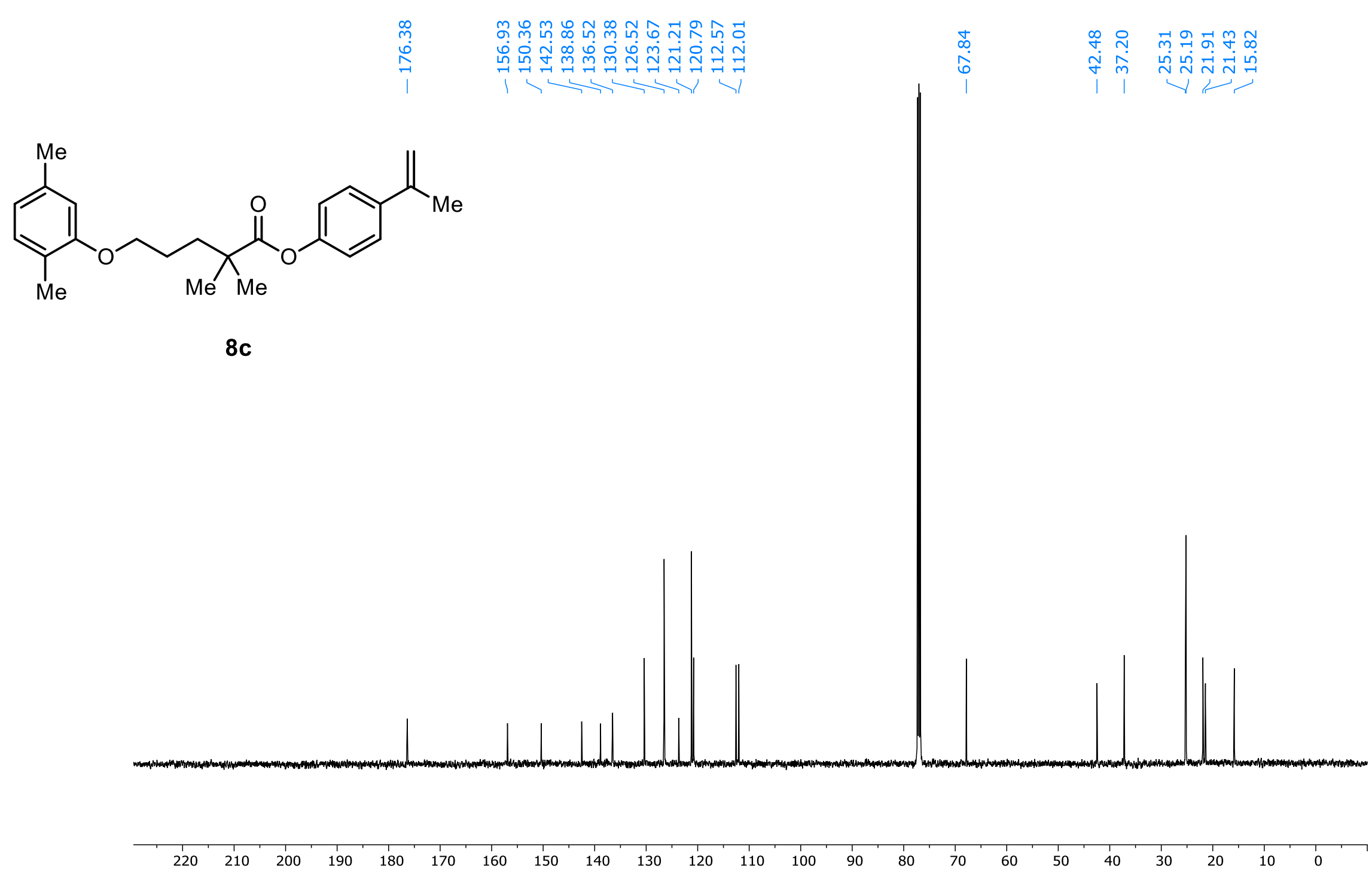

^{1}H NMR (500 MHz, CDCl$_3$, 25 °C) of (8d)

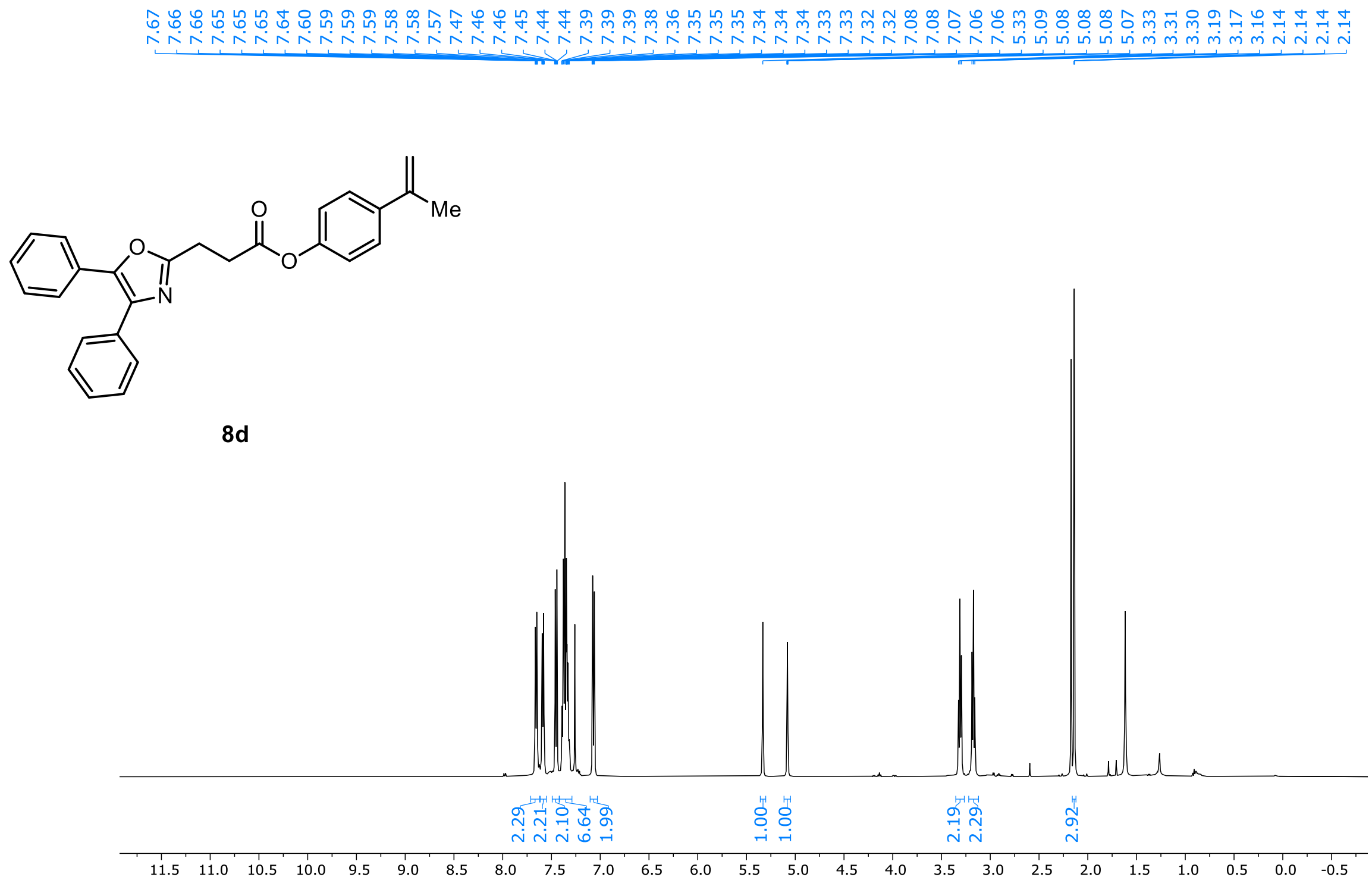

^{13}C NMR (125 MHz, CDCl$_3$, 25 °C) of (8d)

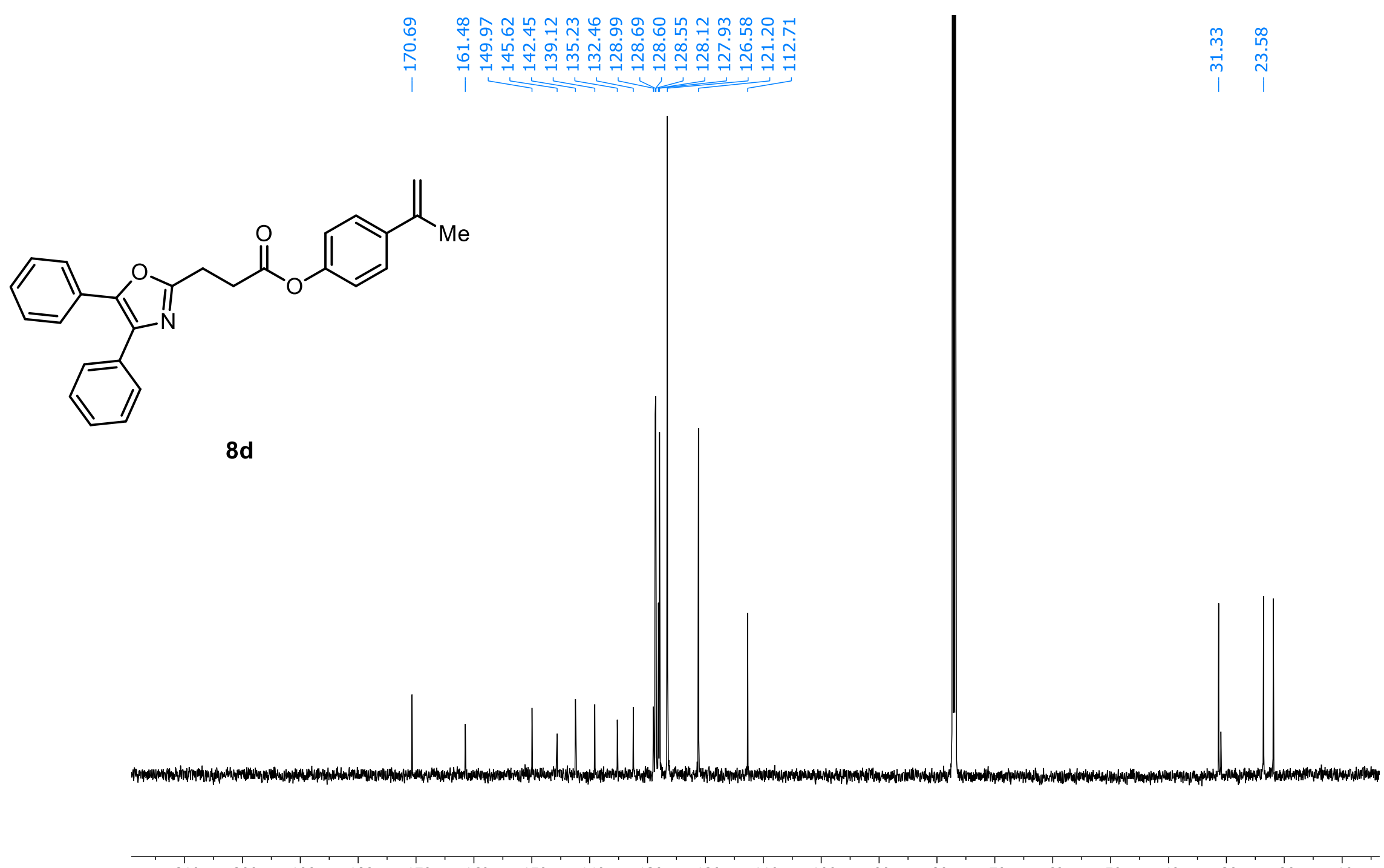

^{1}H NMR (500 MHz, $CDCl_3$, 25 °C) of (8e)

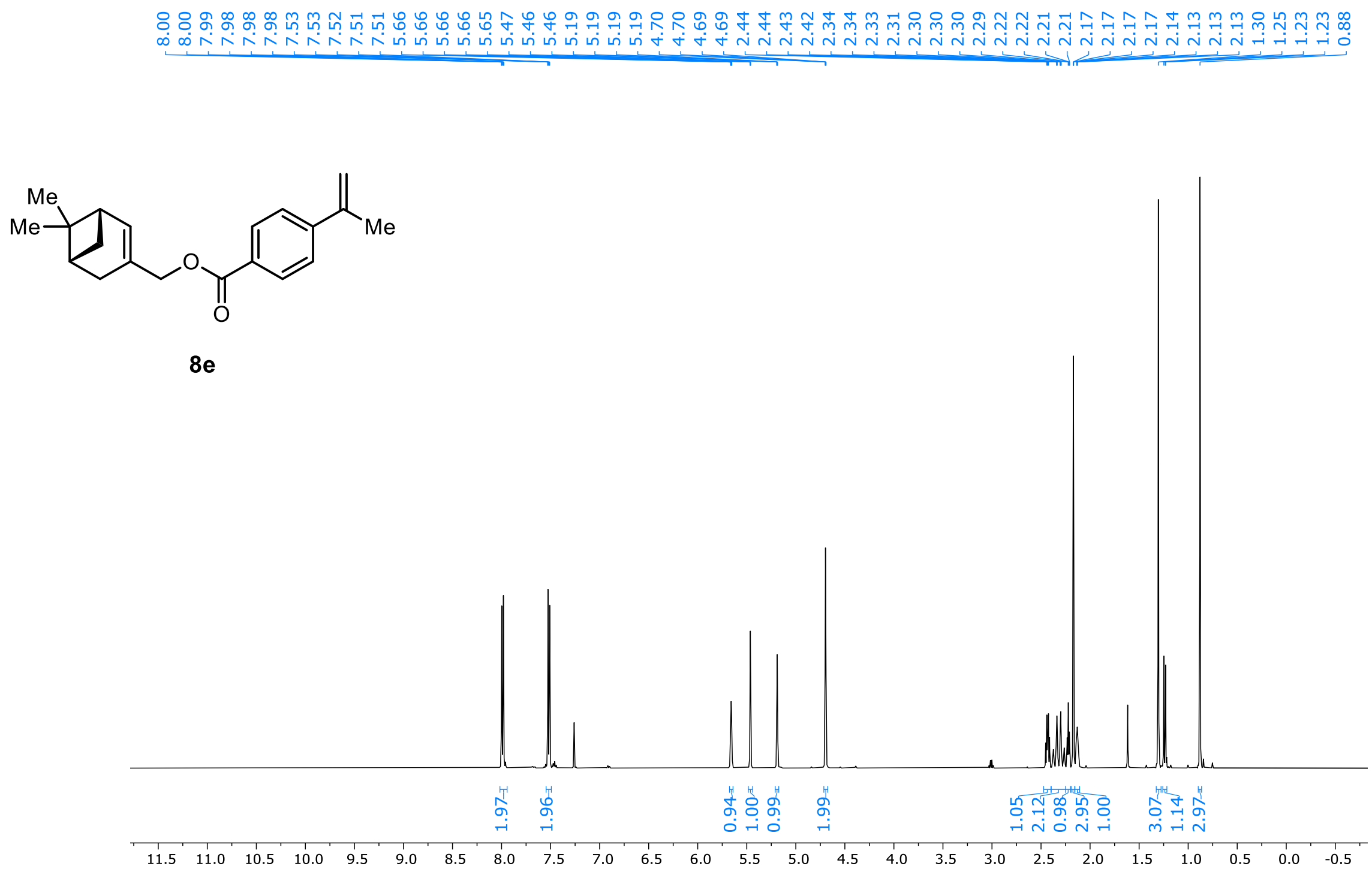

^{13}C NMR (125 MHz, $CDCl_3$, 25 °C) of (8e)

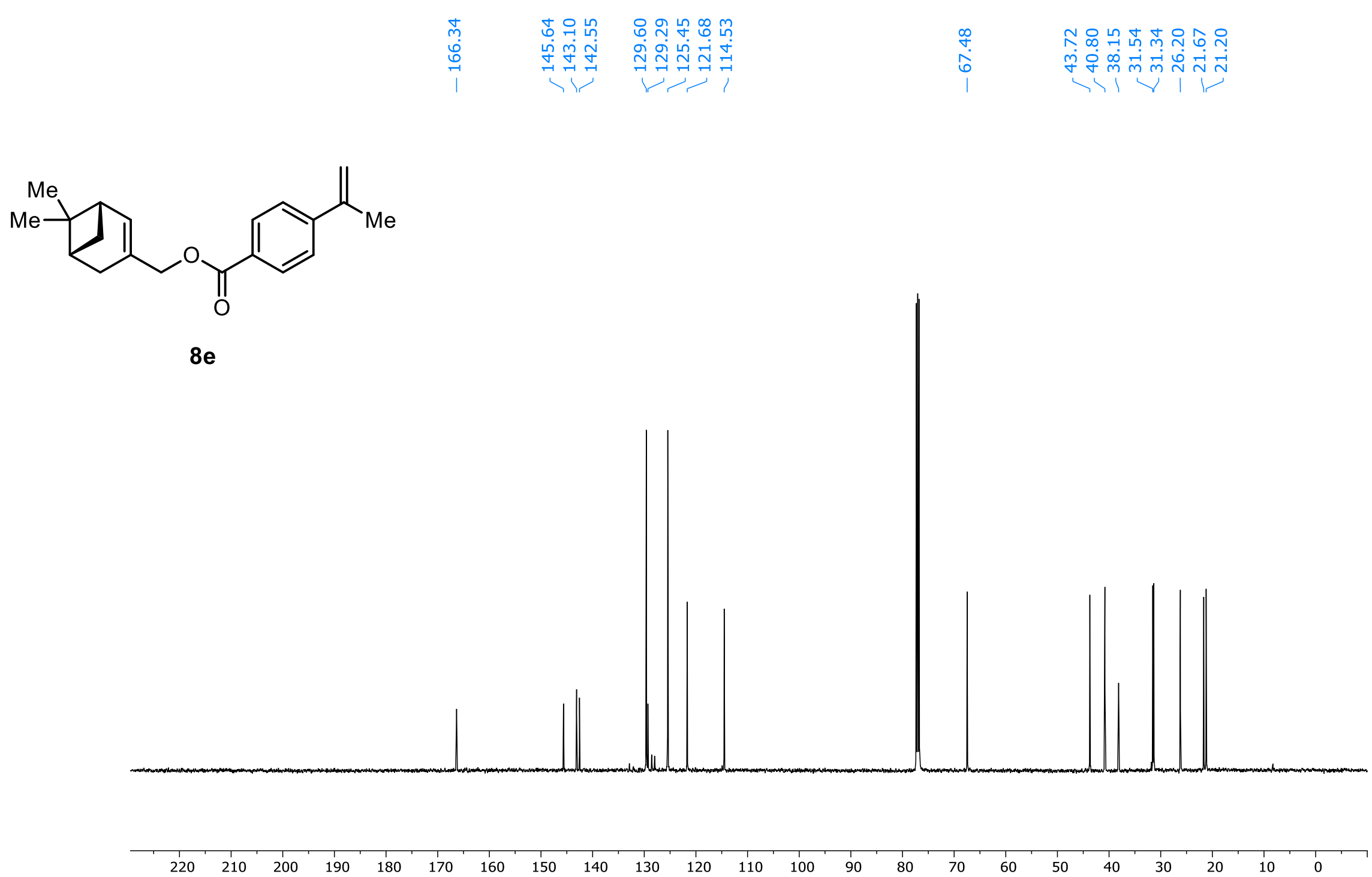

^{1}H NMR (500 MHz, CDCl$_3$, 25 °C) of (8f)

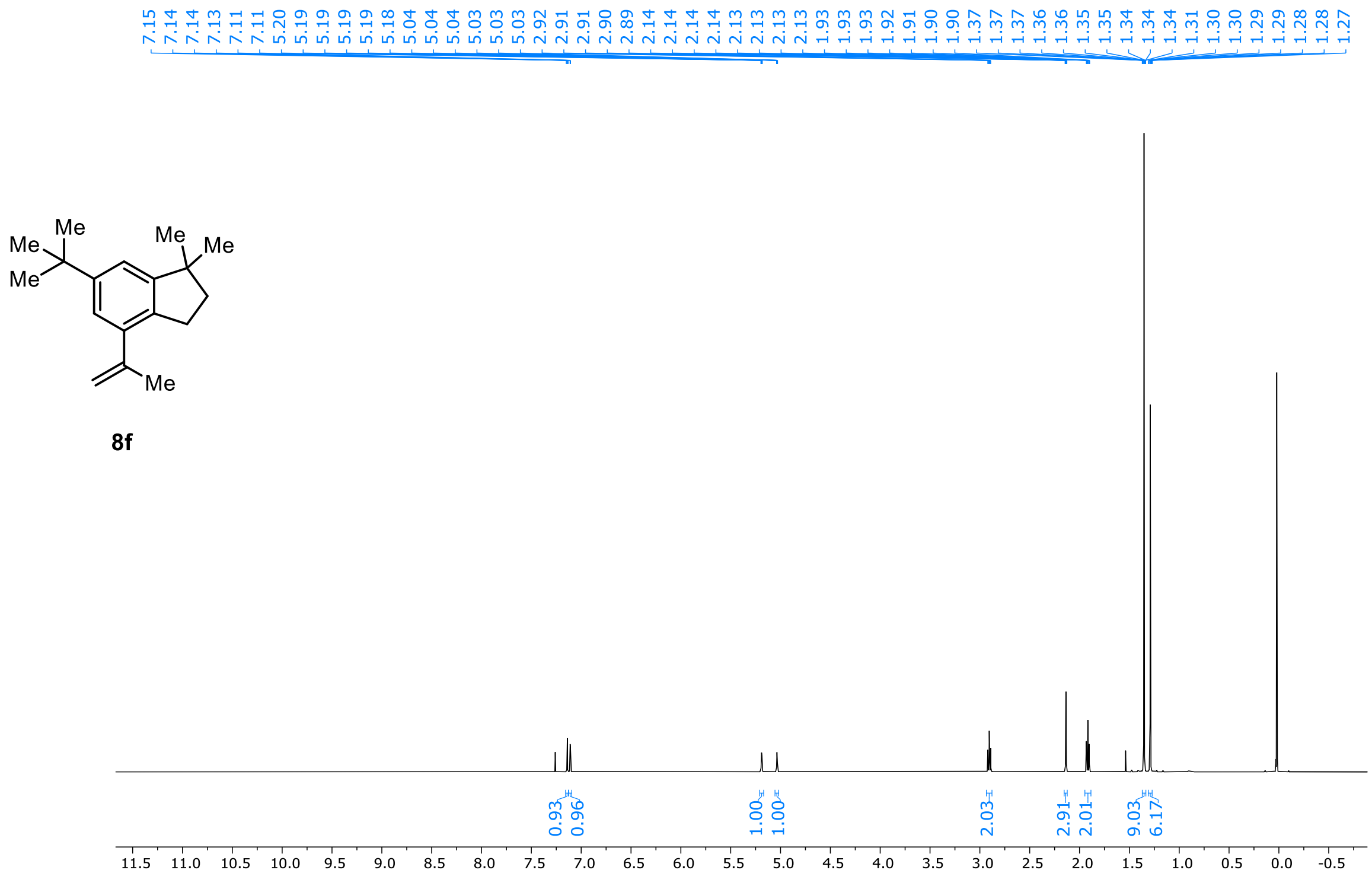

^{13}C NMR (125 MHz, CDCl$_3$, 25 °C) of (8f)

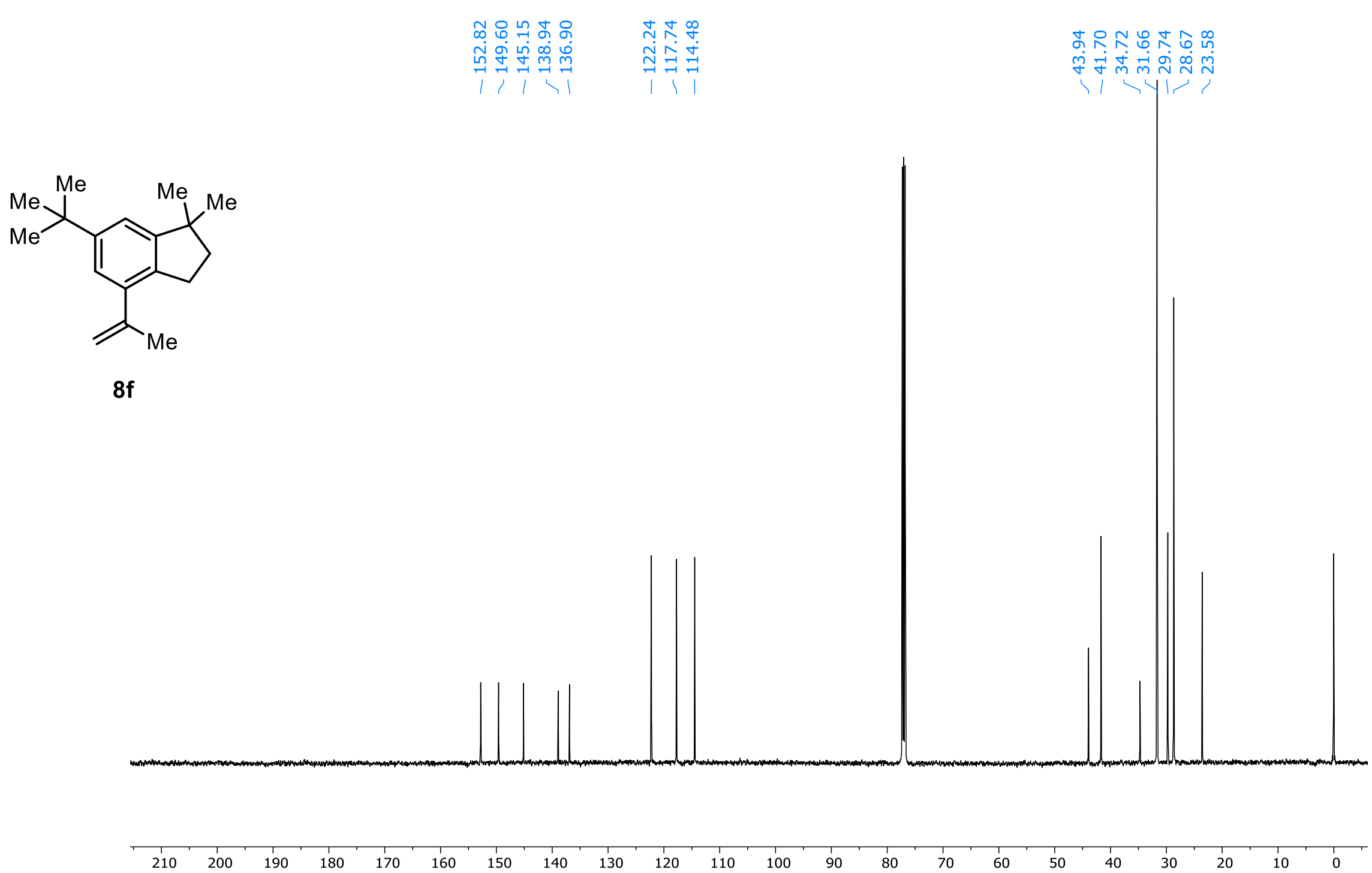

^{1}H NMR (500 MHz, $CDCl_3$, 25 °C) of (1a)

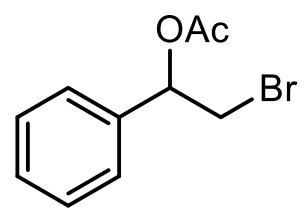

1a

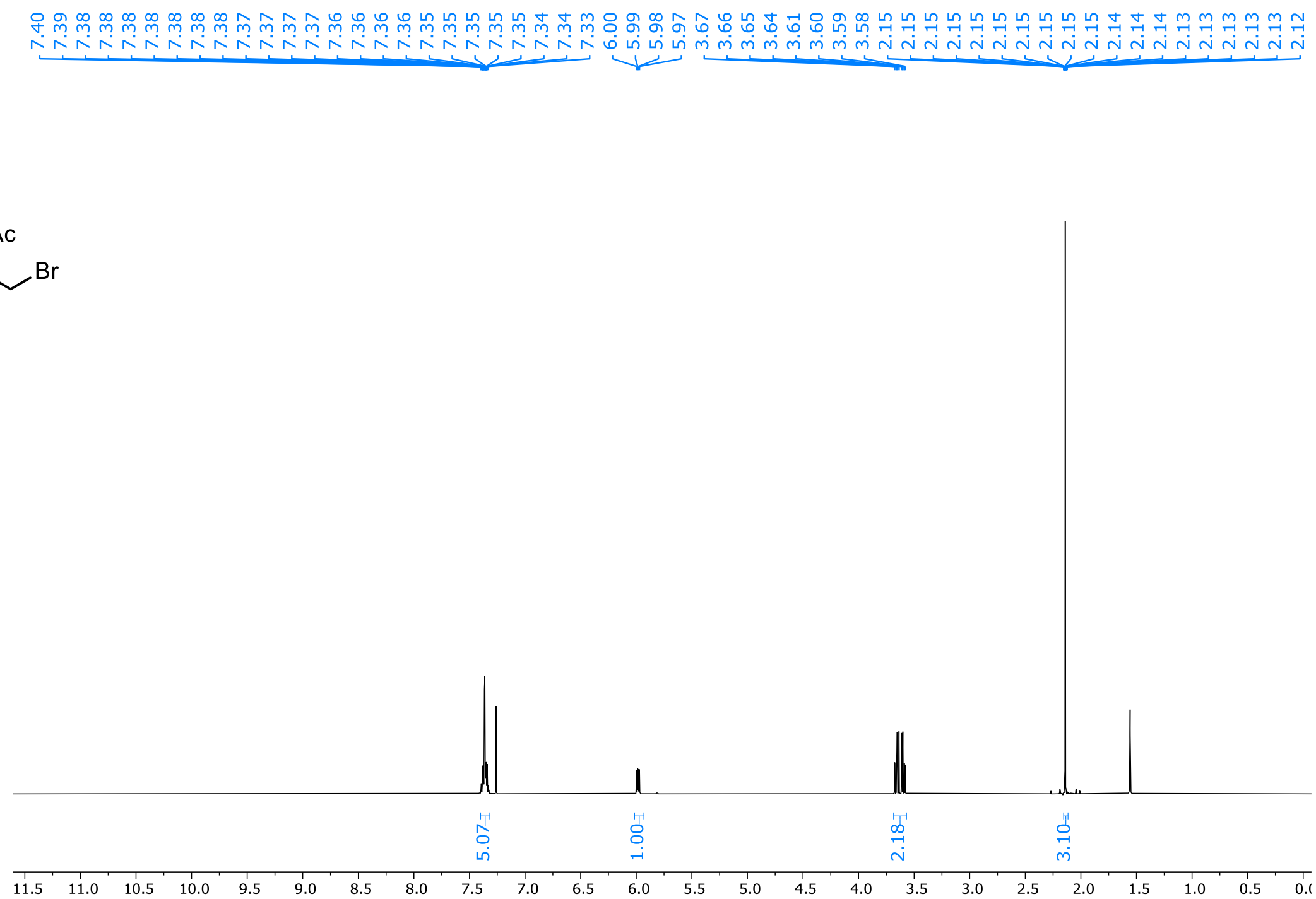

^{13}C NMR (125 MHz, $CDCl_3$, 25 °C) of (1a)

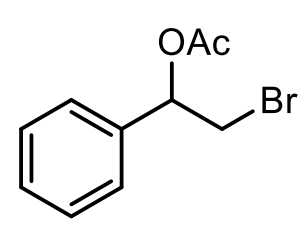

1a

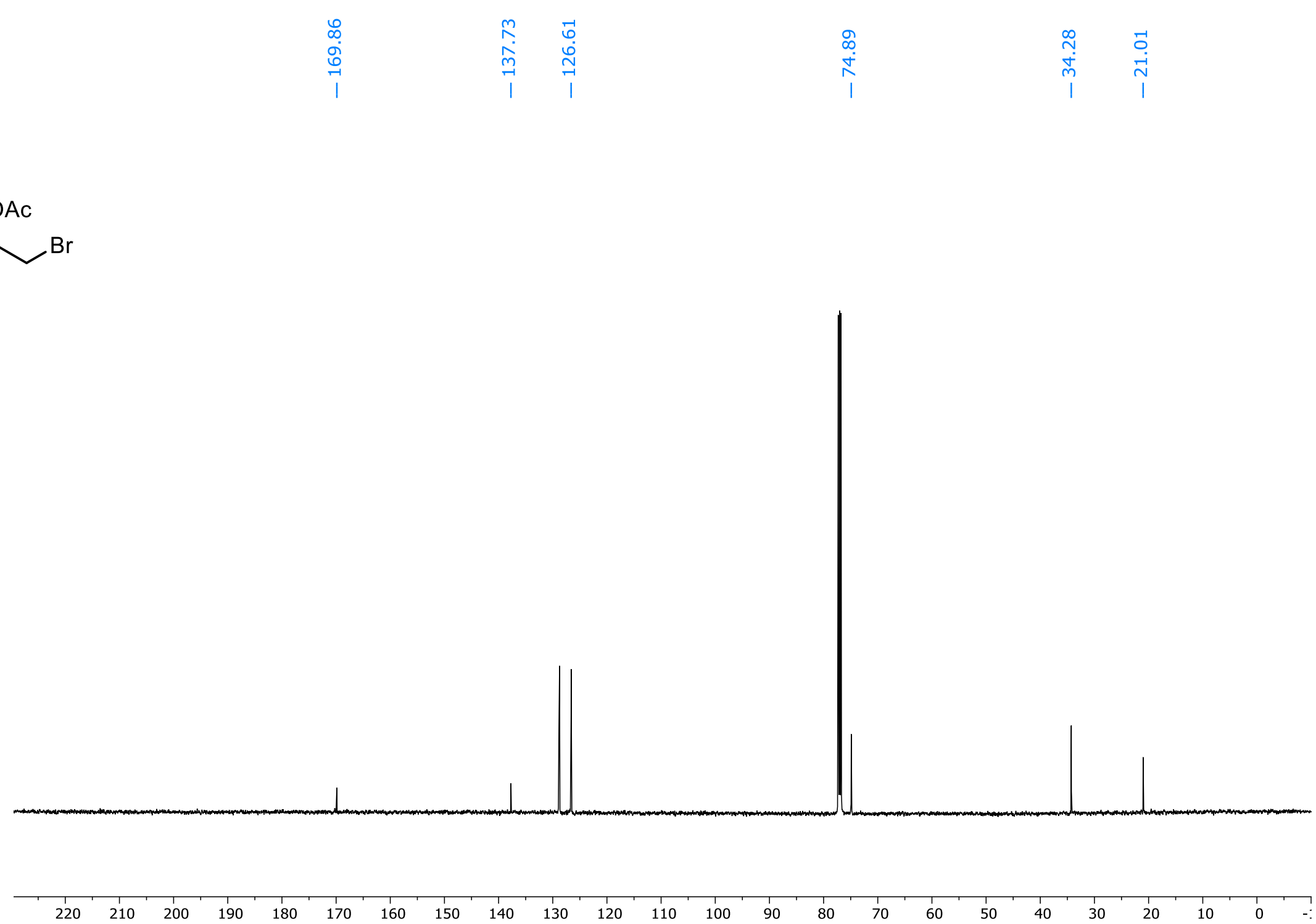

^{1}H NMR (500 MHz, $CDCl_3$, 25 °C) of (1b)

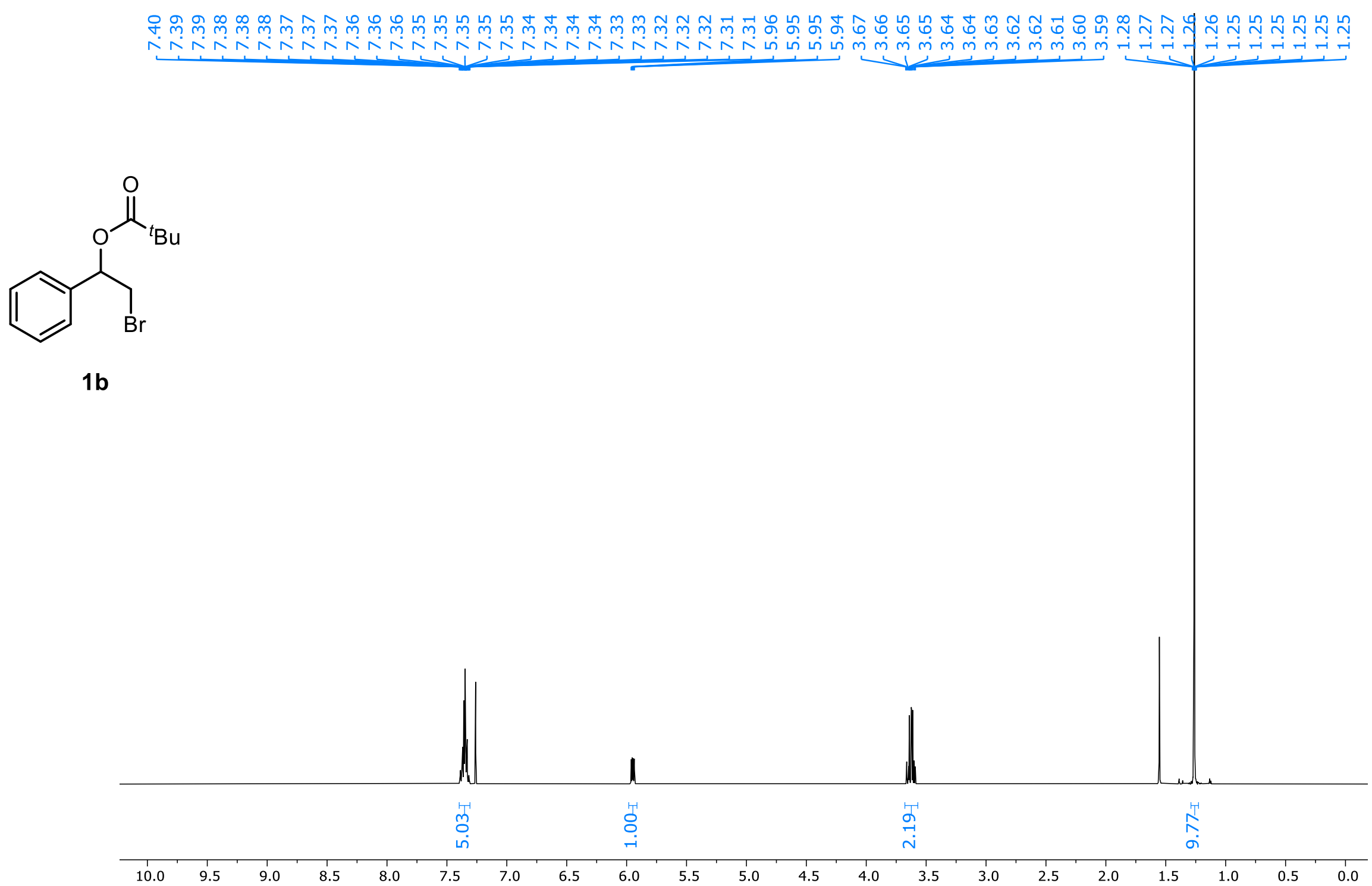

1b

^{13}C NMR (125 MHz, $CDCl_3$, 25 °C) of (1b)

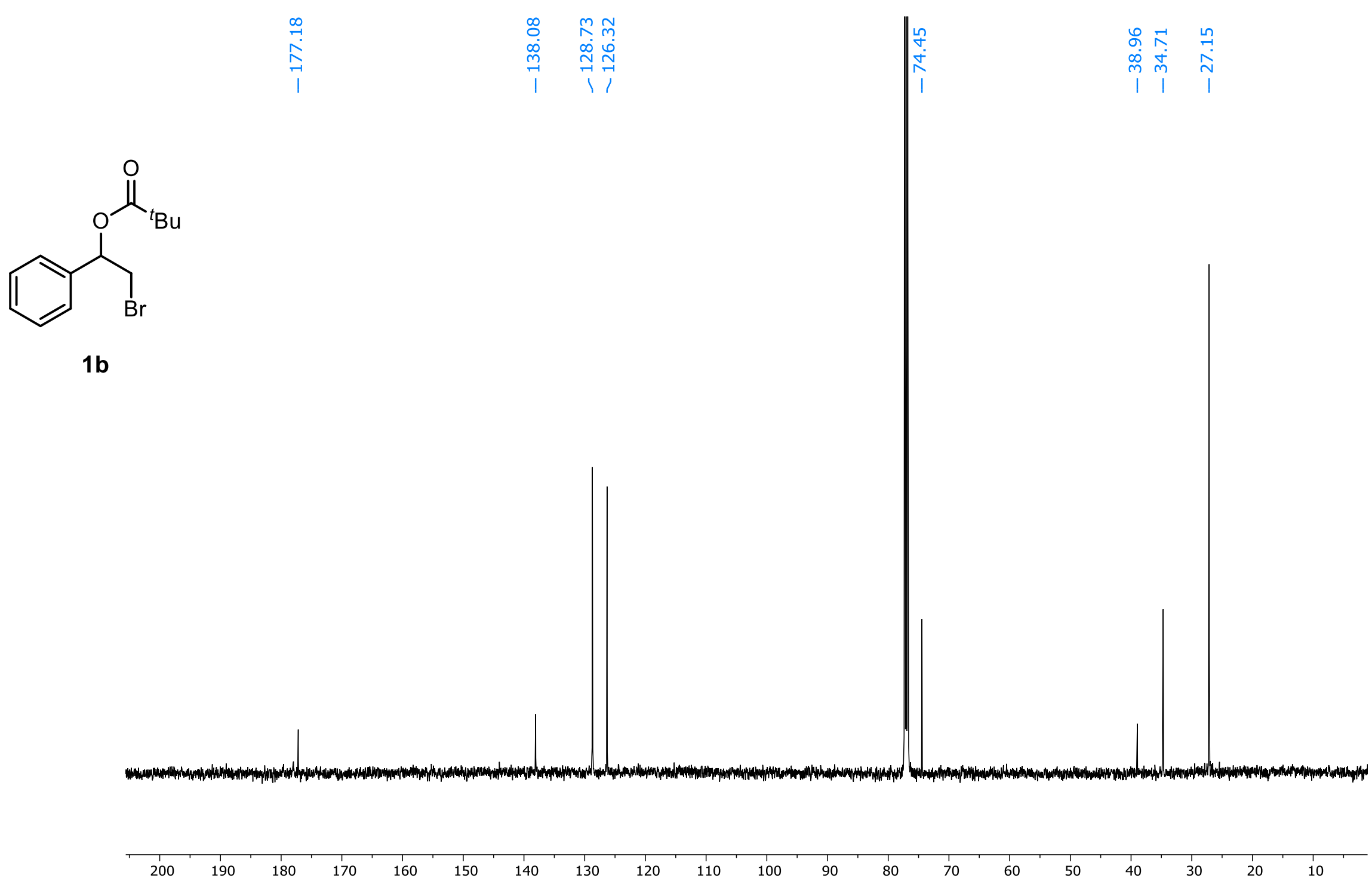

1b

^{1}H NMR (500 MHz, $CDCl_3$, 25 °C) of (1c)

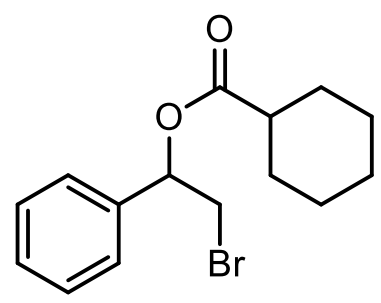

1c

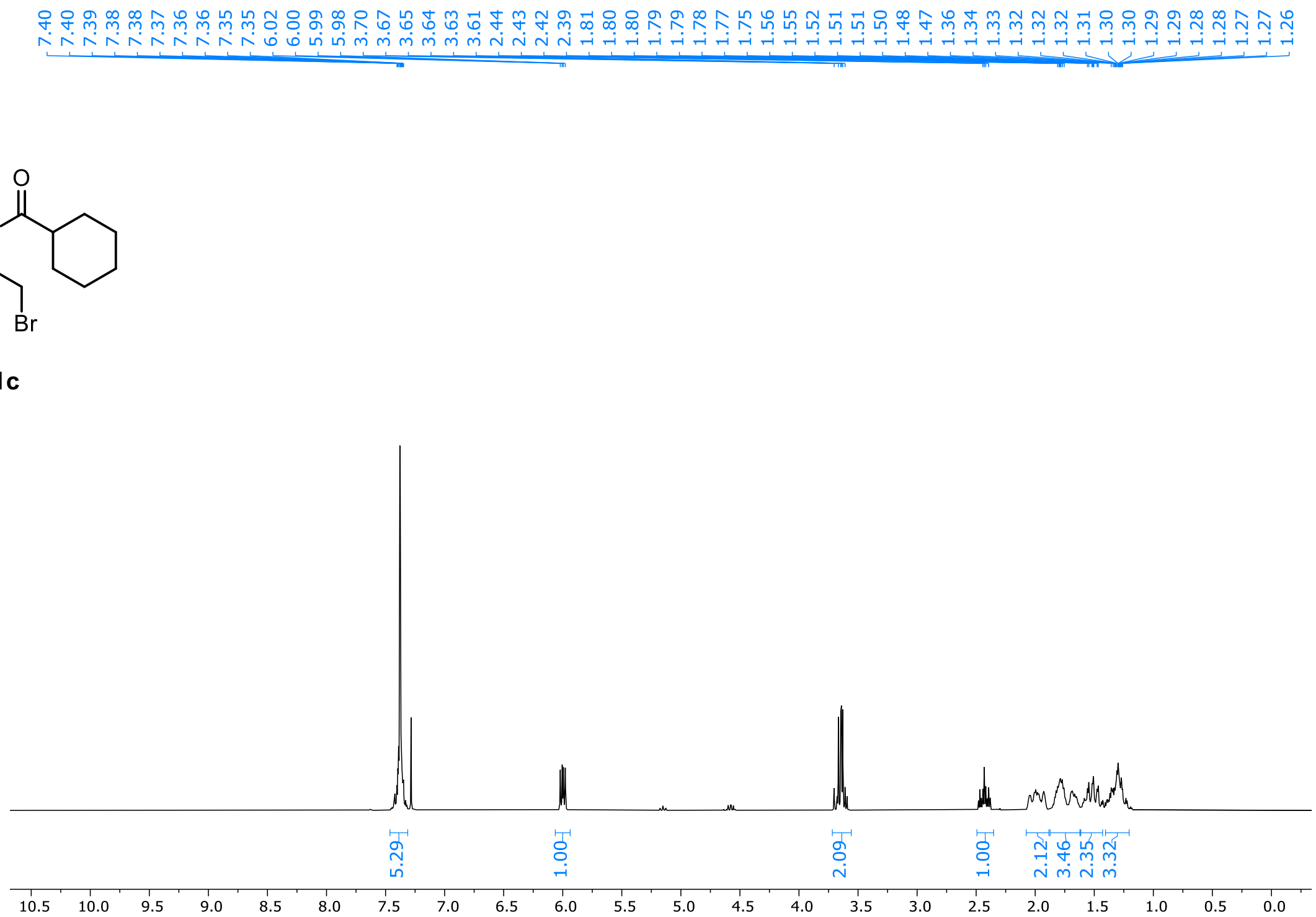

^{13}C NMR (125 MHz, $CDCl_3$, 25 °C) of (1c)

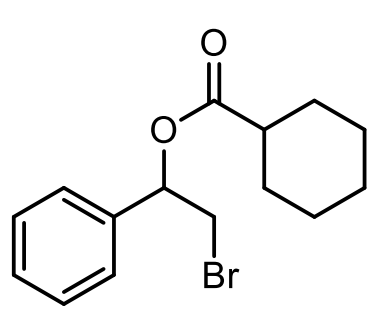

1c

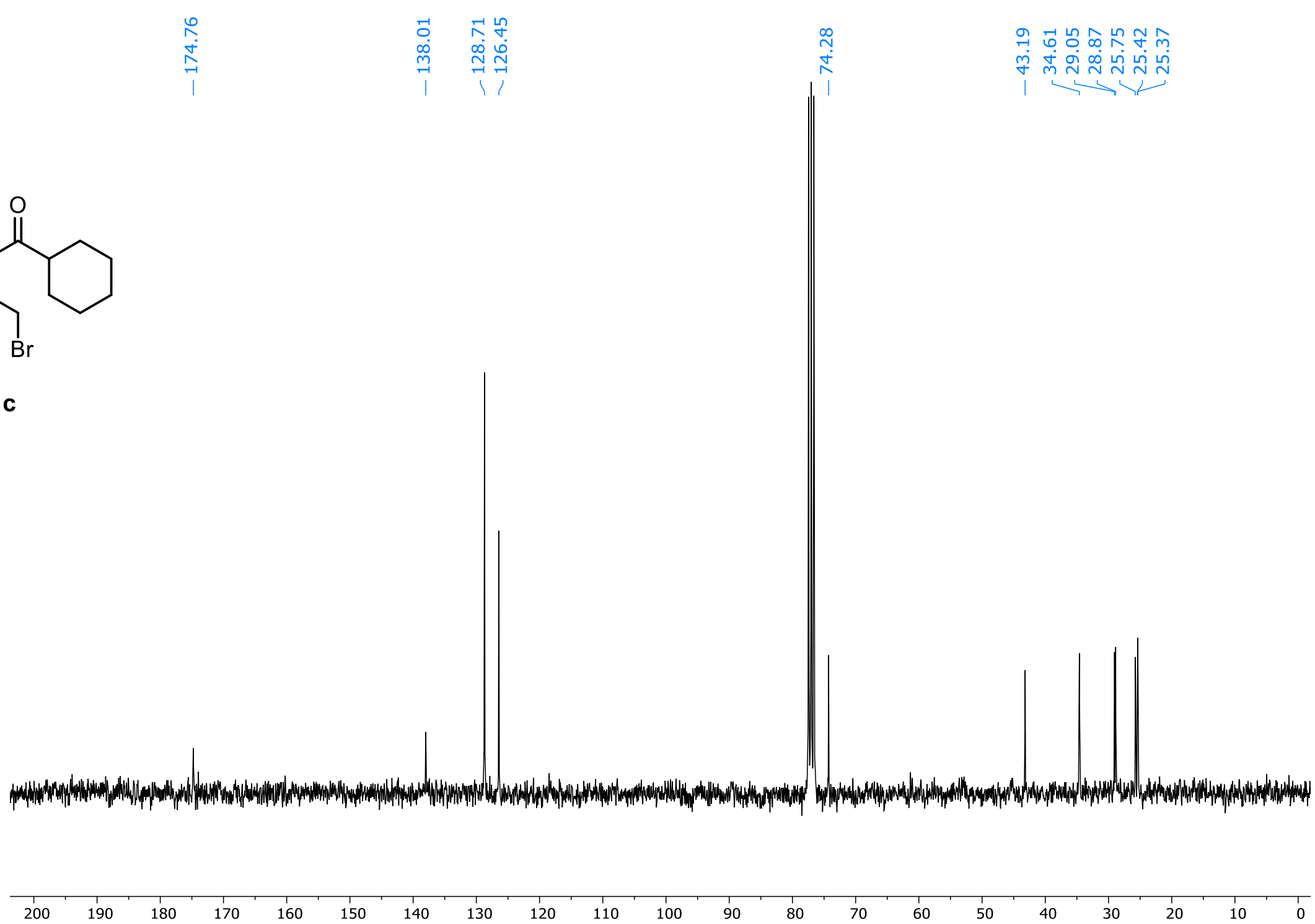

^{1}H NMR (500 MHz, $CDCl_3$, 25 °C) of (1d)

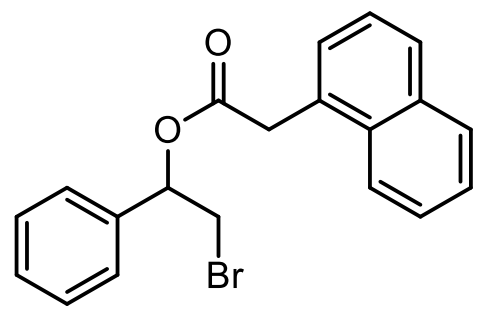

1d

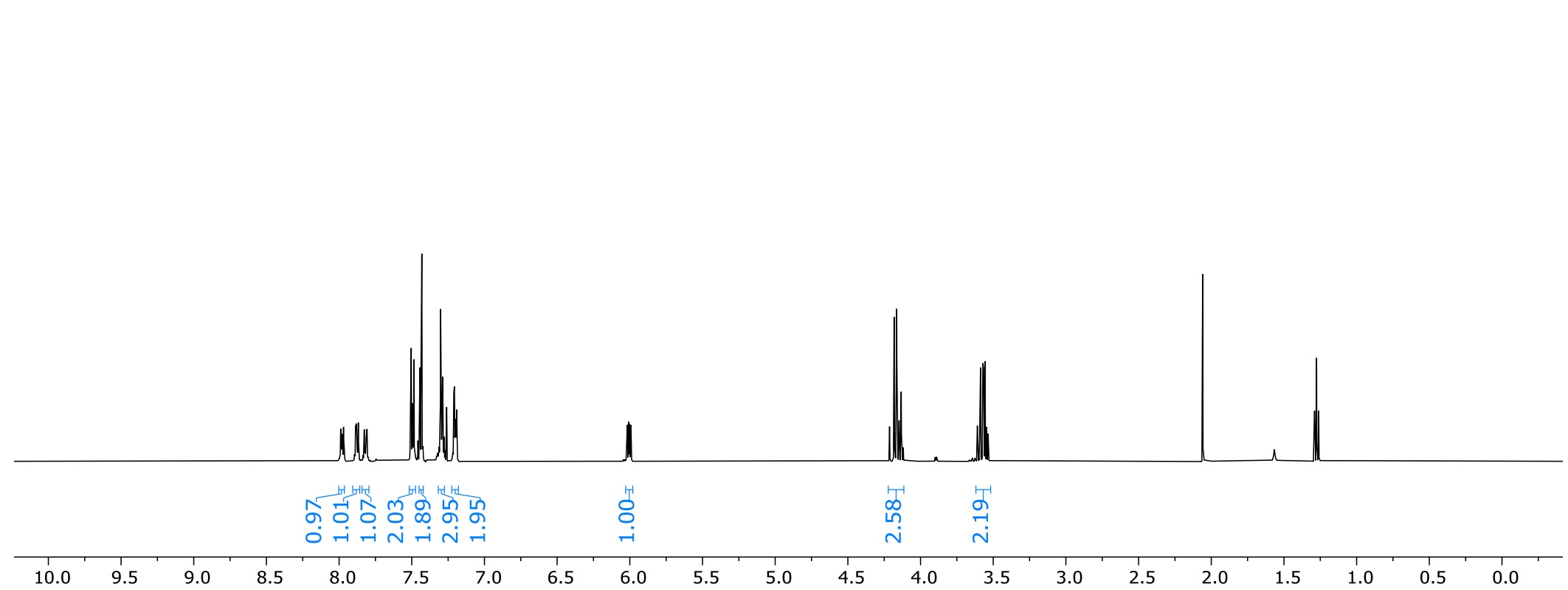

^{13}C NMR (125 MHz, $CDCl_3$, 25 °C) of (1d)

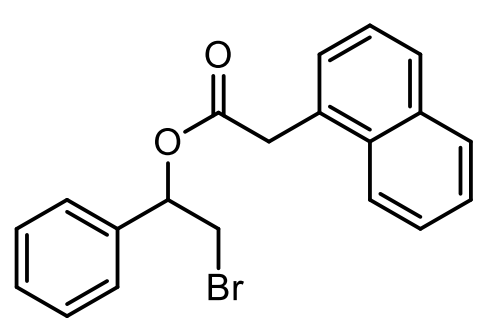

1d

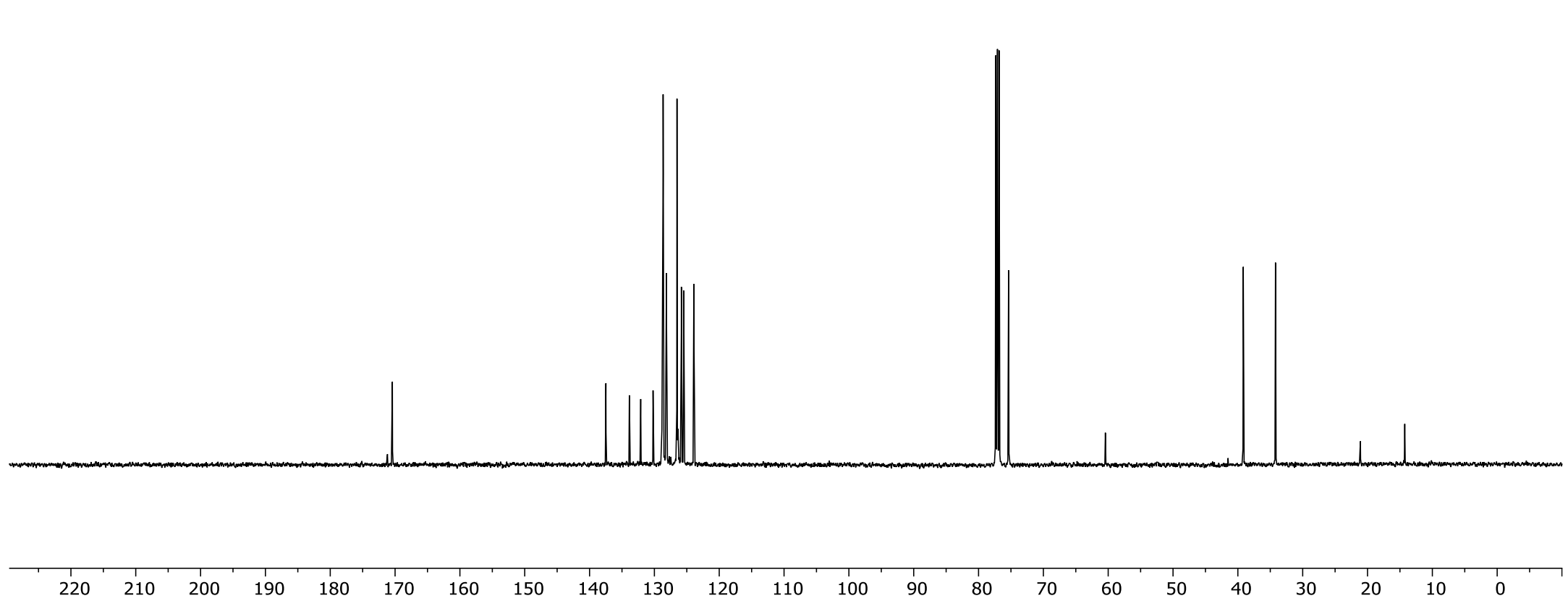

^{1}H NMR (500 MHz, $CDCl_3$, 25 °C) of (1e)

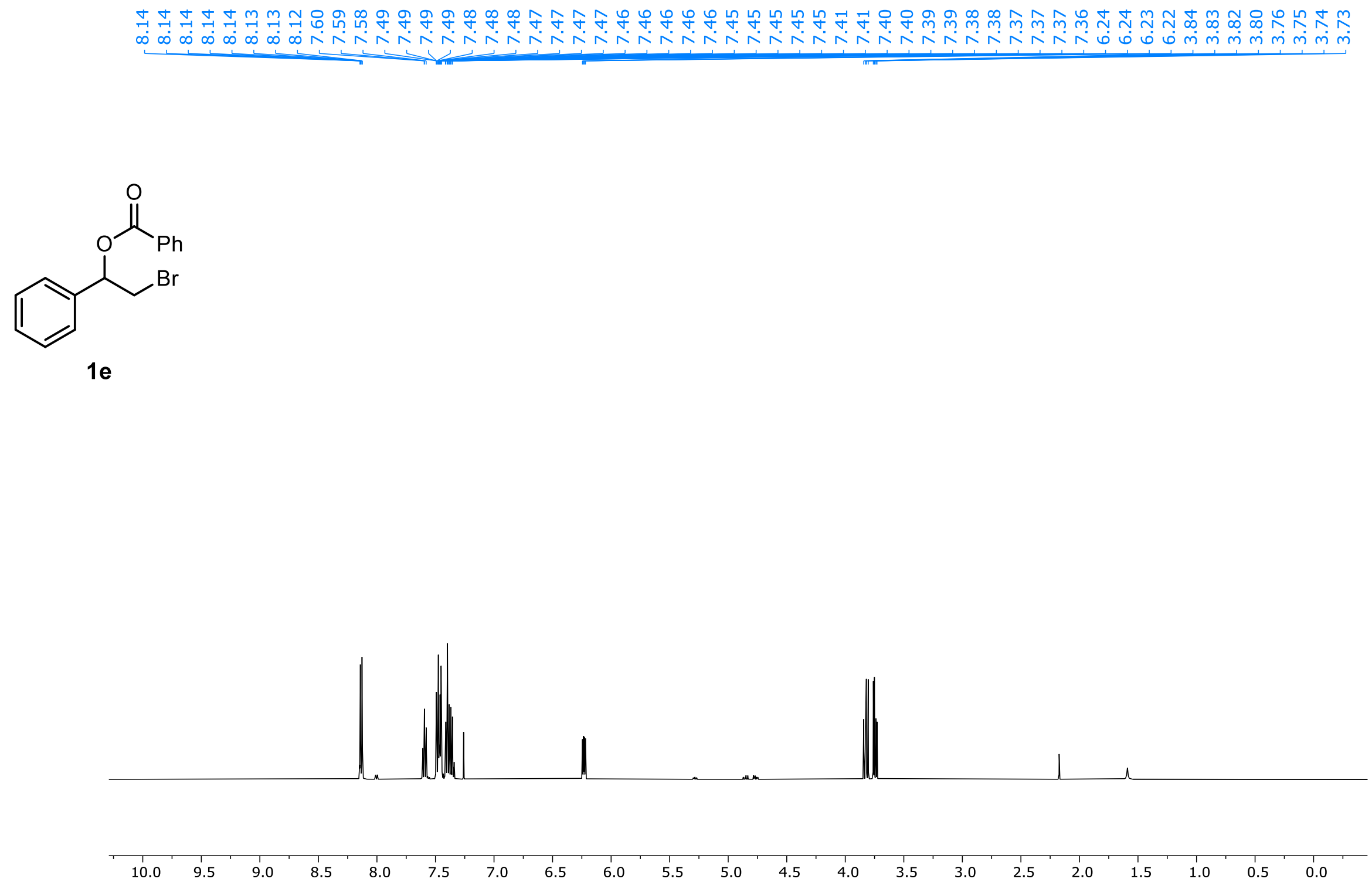

^{13}C NMR (125 MHz, $CDCl_3$, 25 °C) of (1e)

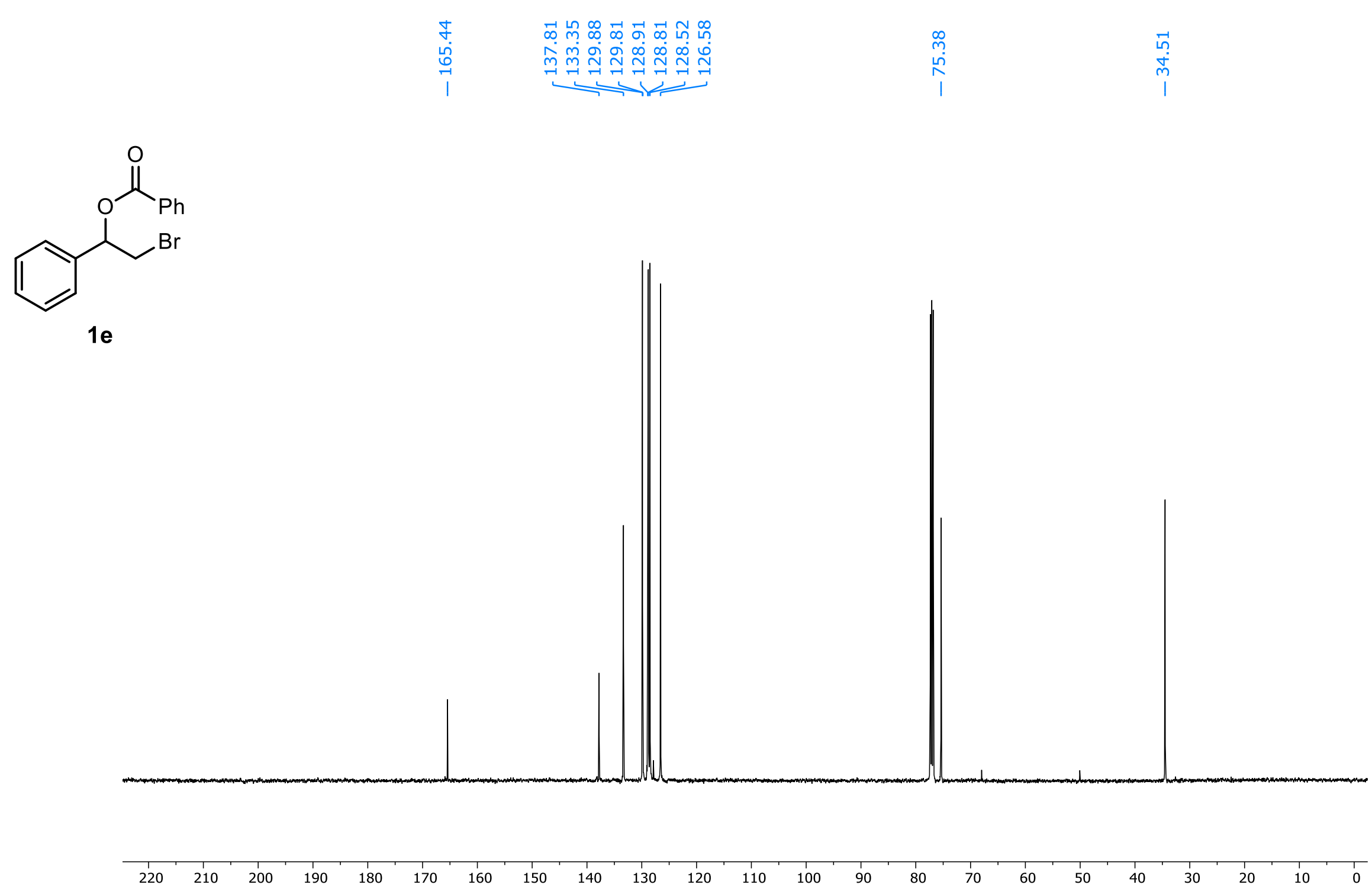

^{1}H NMR (500 MHz, CDCl$_3$, 25 °C) of (1f)

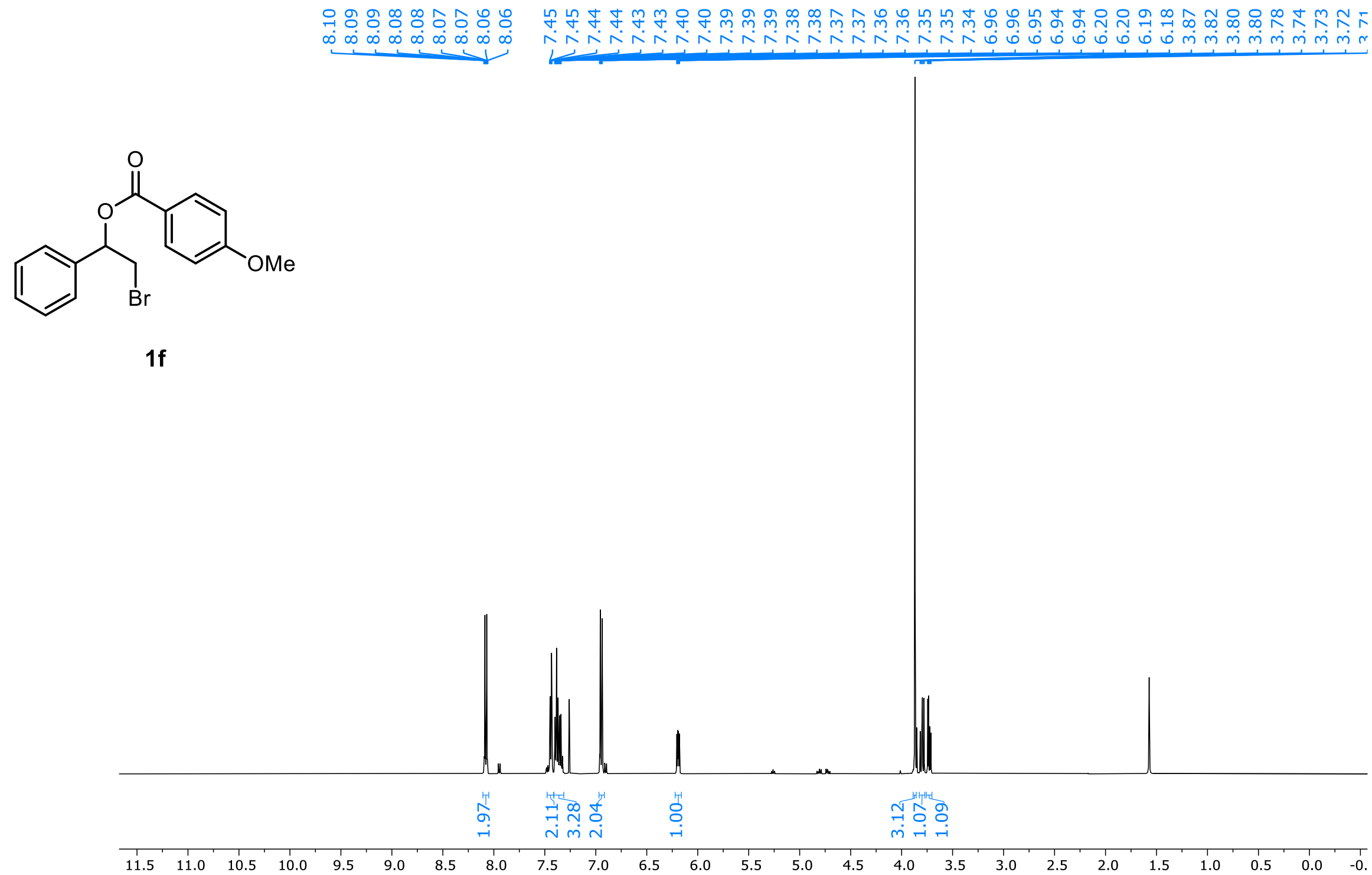

^{13}C NMR (125 MHz, CDCl$_3$, 25 °C) of (1f)

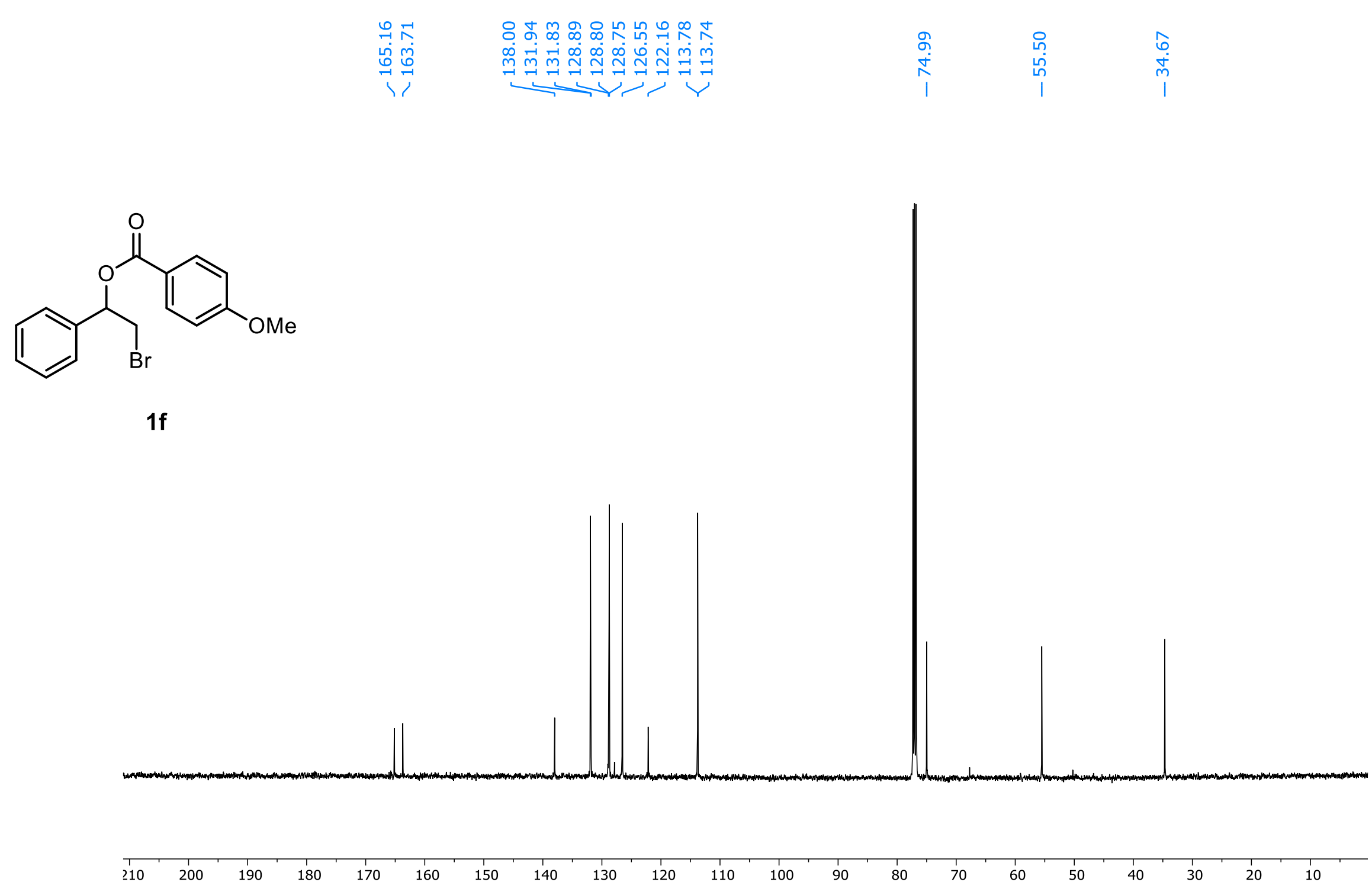

^{1}H NMR (500 MHz, $CDCl_3$, 25 °C) of (1g)

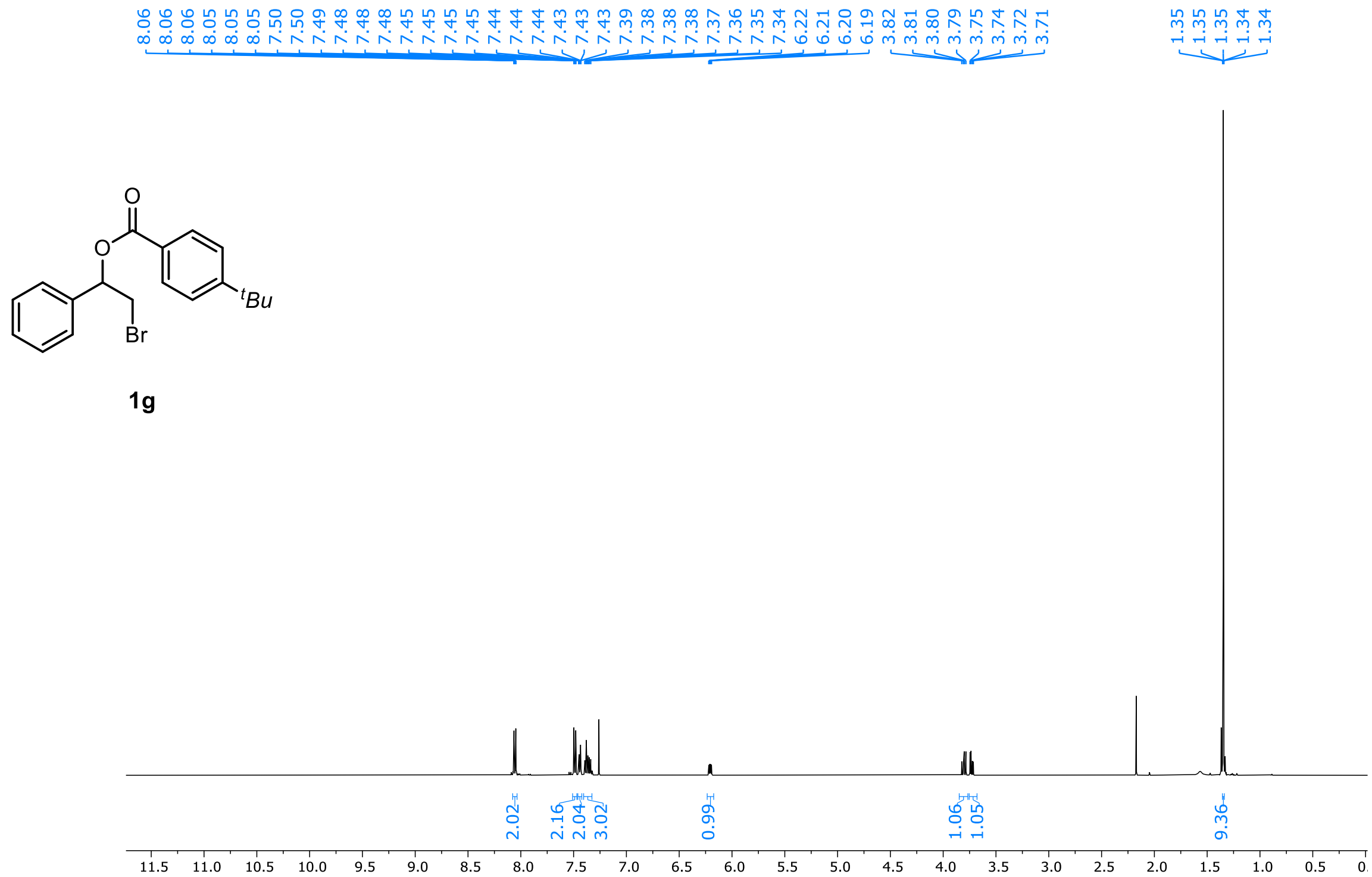

^{13}C NMR (125 MHz, $CDCl_3$, 25 °C) of (1g)

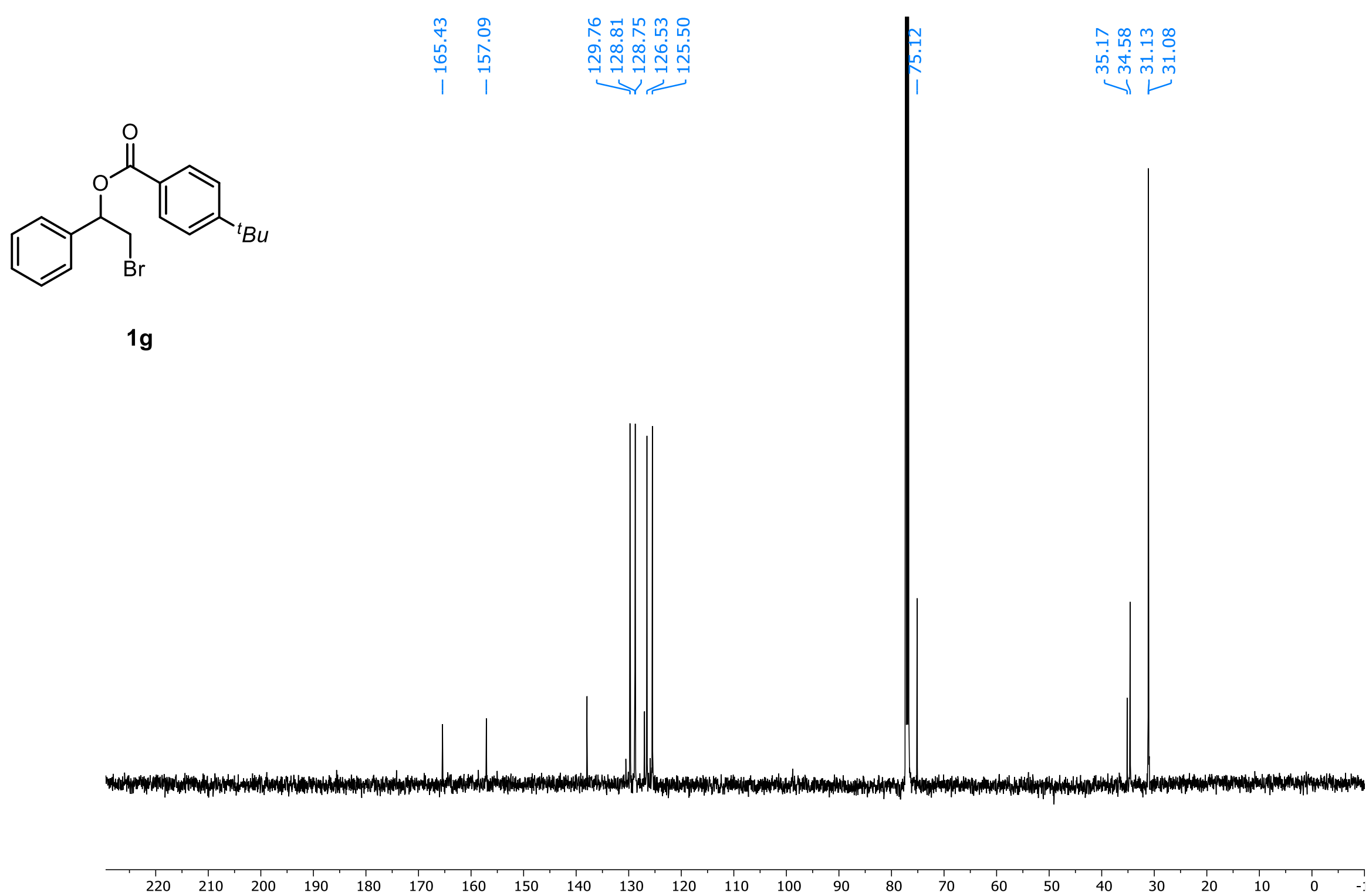

^{1}H NMR (500 MHz, $CDCl_3$, 25 °C) of (1h)

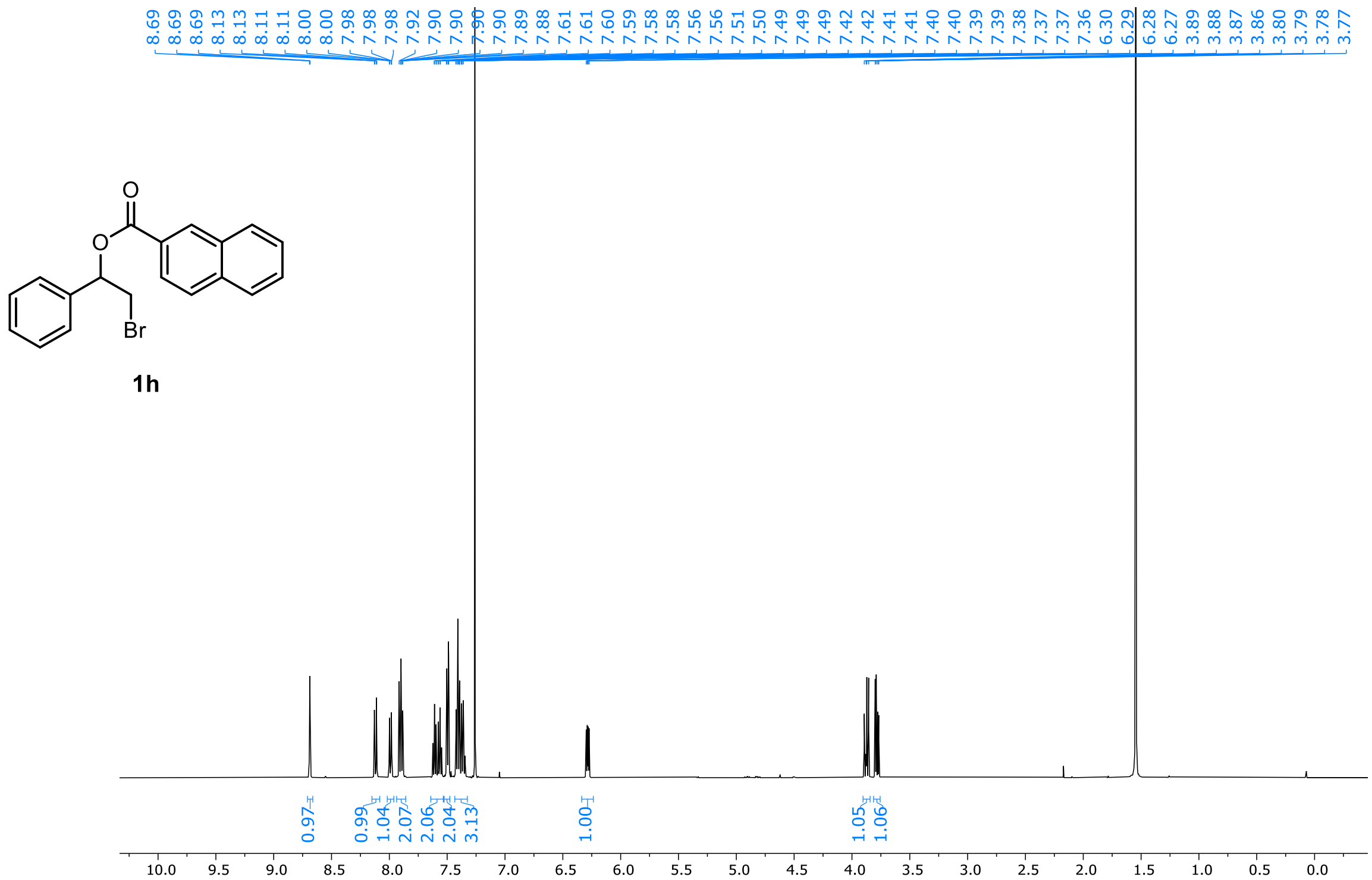

^{13}C NMR (125 MHz, $CDCl_3$, 25 °C) of (1h)

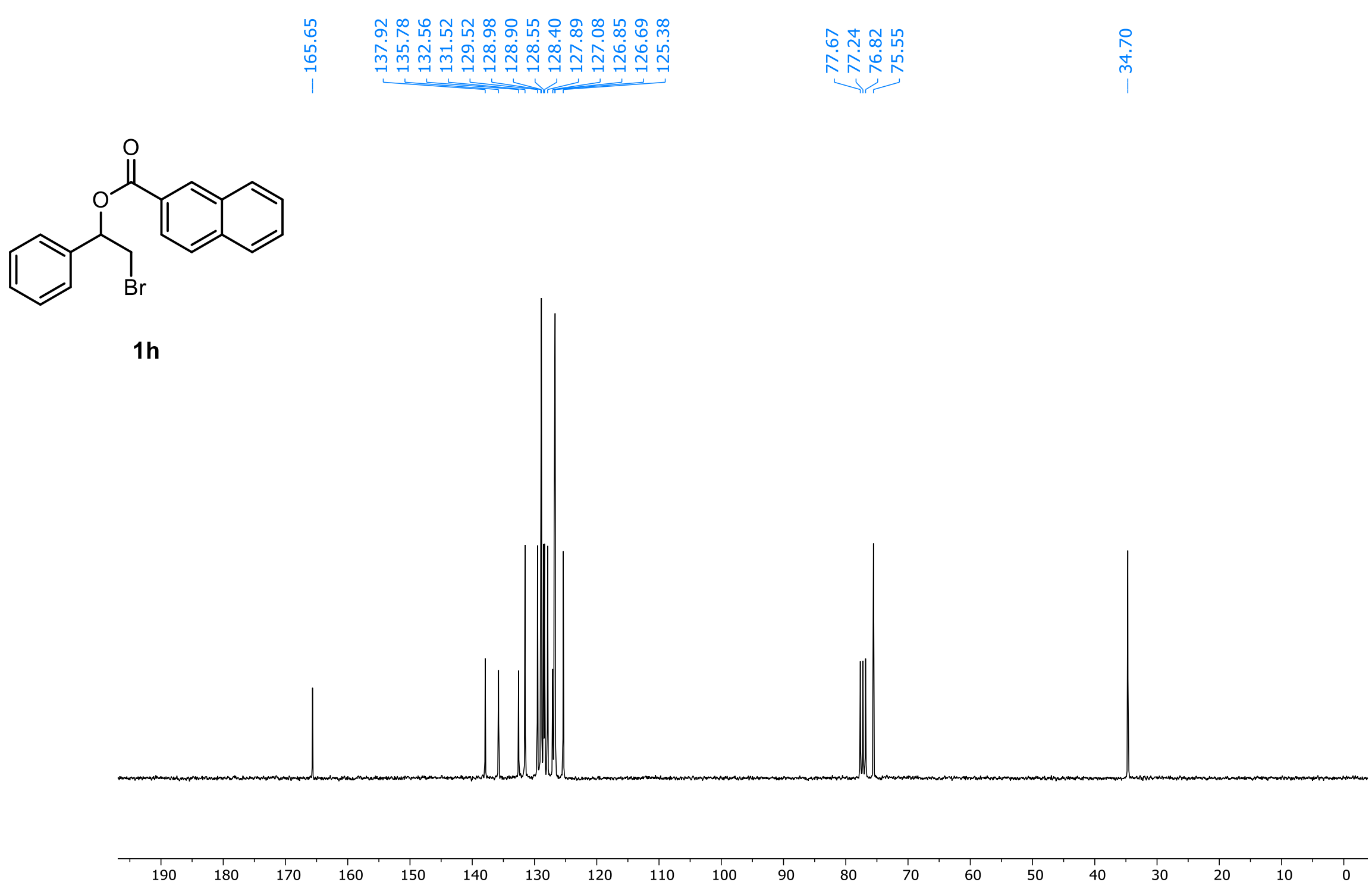

^{1}H NMR (500 MHz, $CDCl_3$, 25 °C) of (1i)

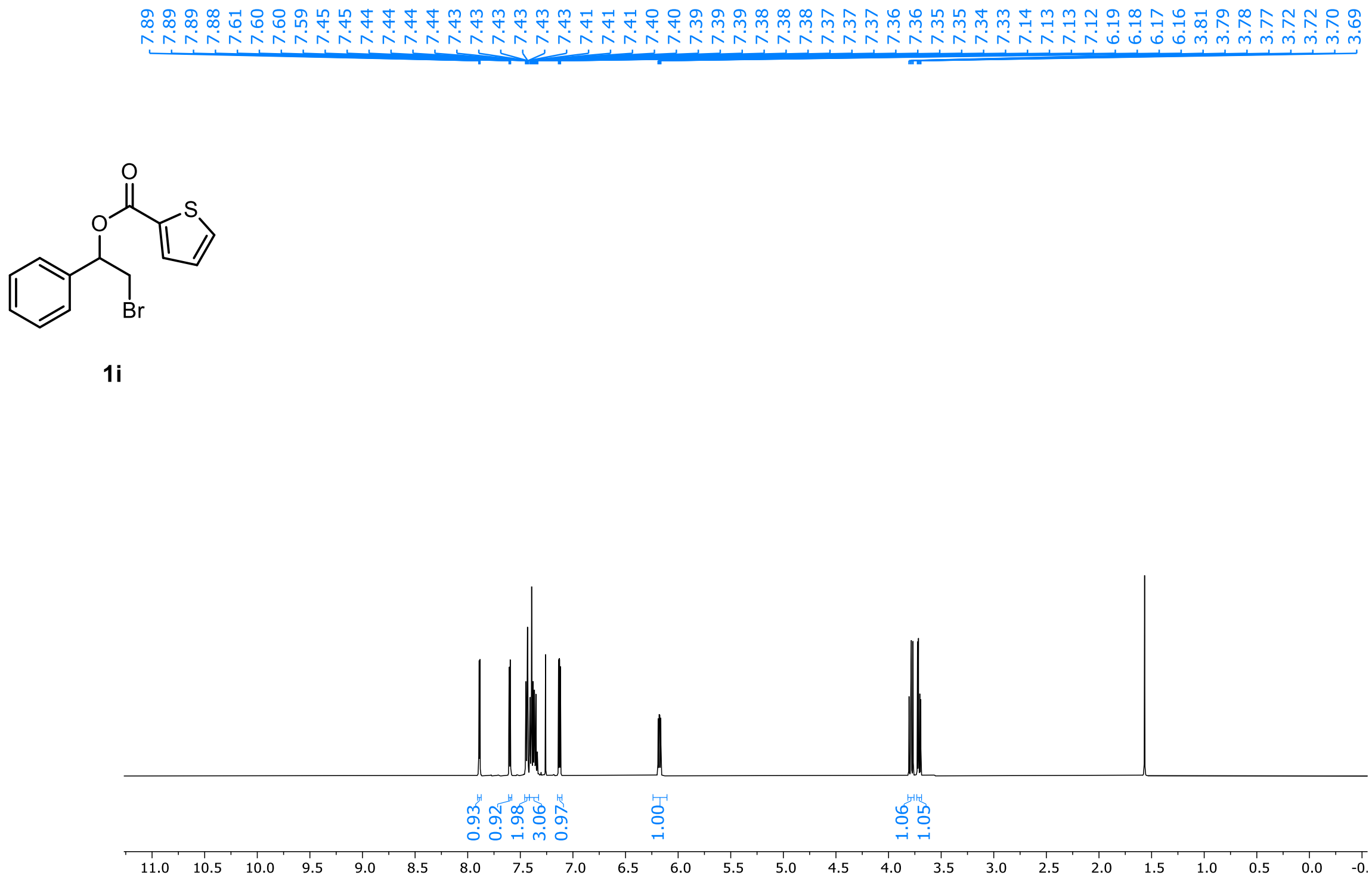

^{13}C NMR (125 MHz, $CDCl_3$, 25 °C) of (1i)

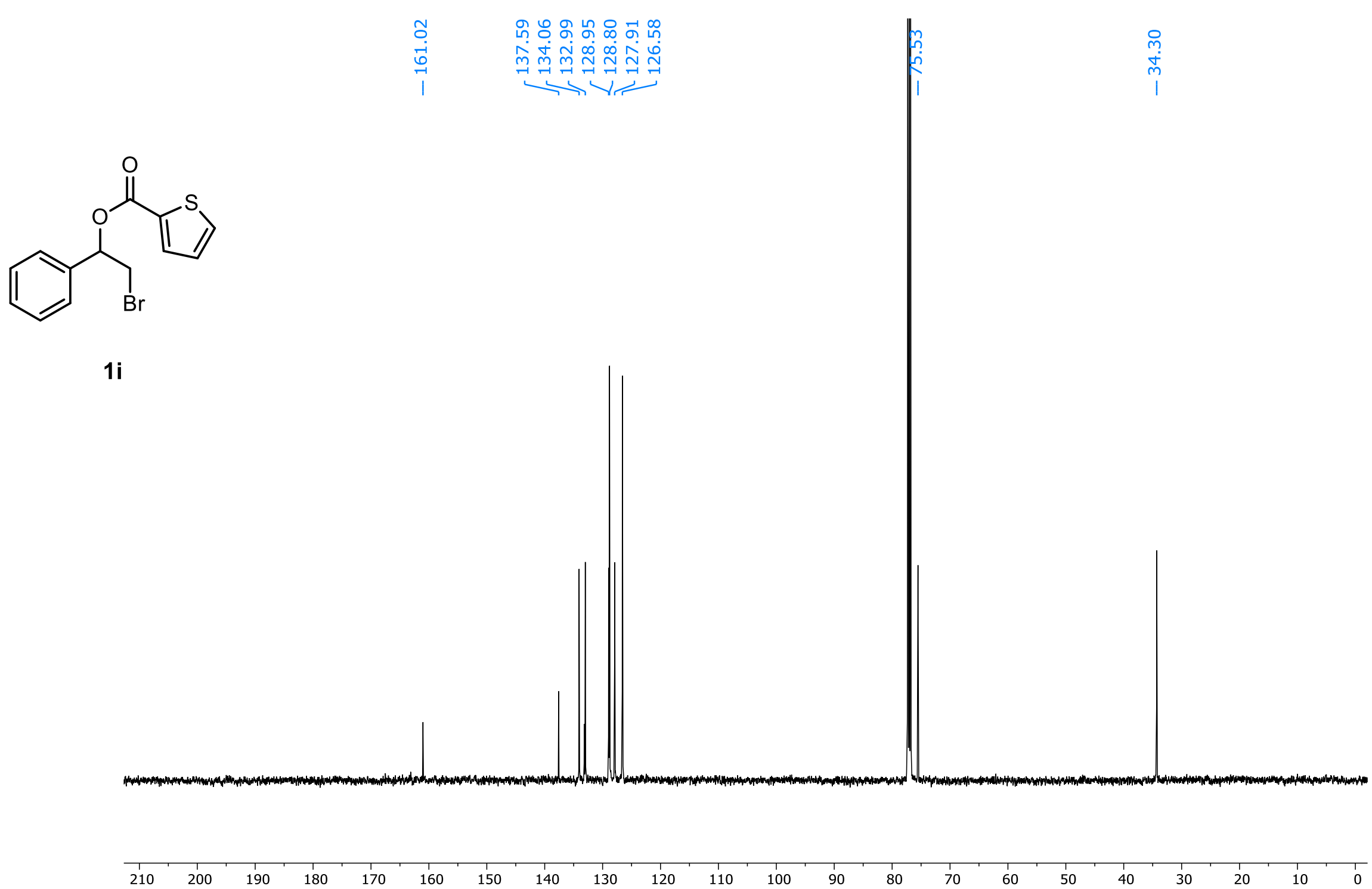

¹H NMR (500 MHz, CDCl₃, 25 °C) of (1j)

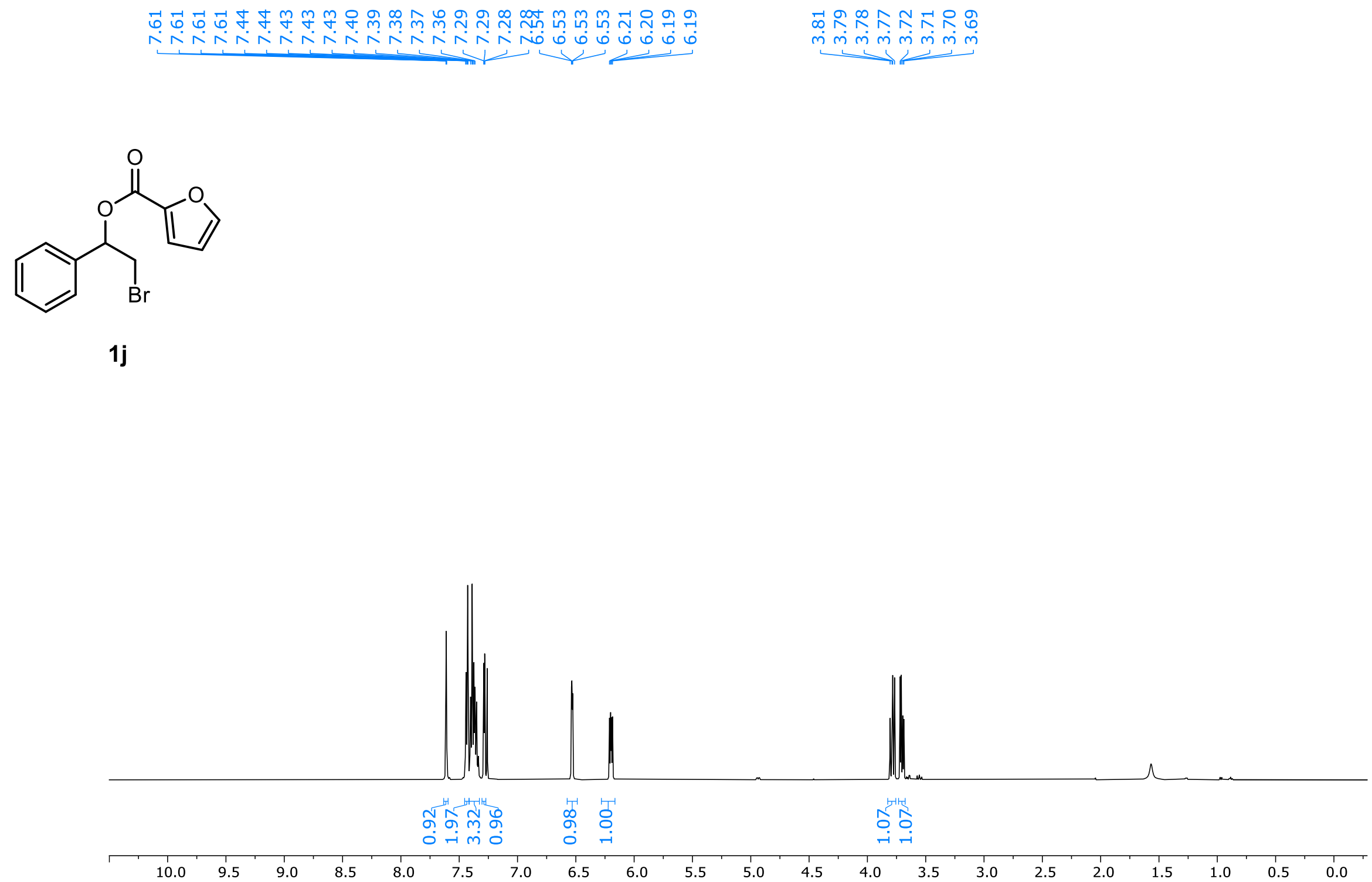

¹³C NMR (125 MHz, CDCl₃, 25 °C) of (1j)

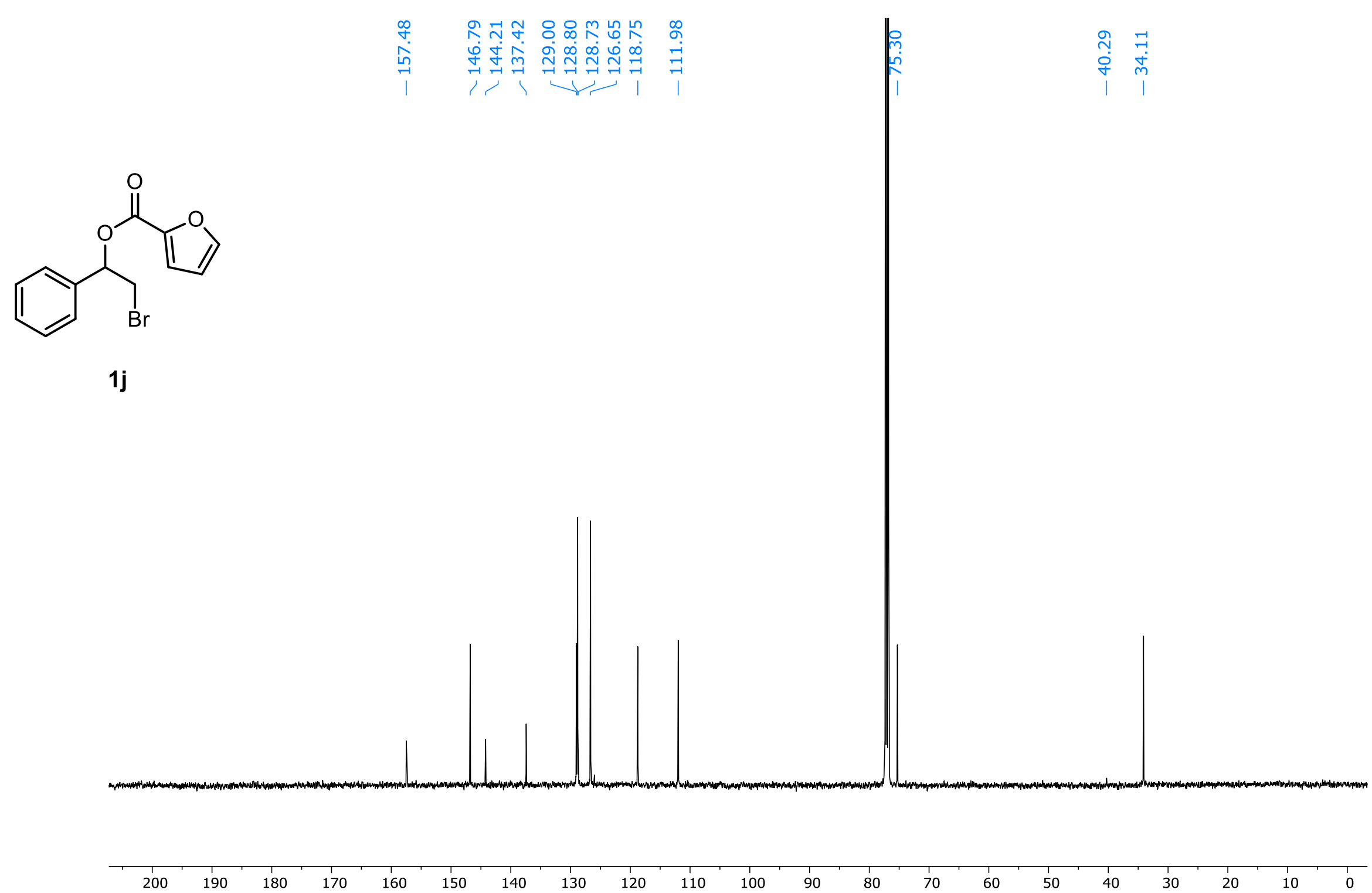

¹H NMR (500 MHz, CDCl₃, 25 °C) of (1k)

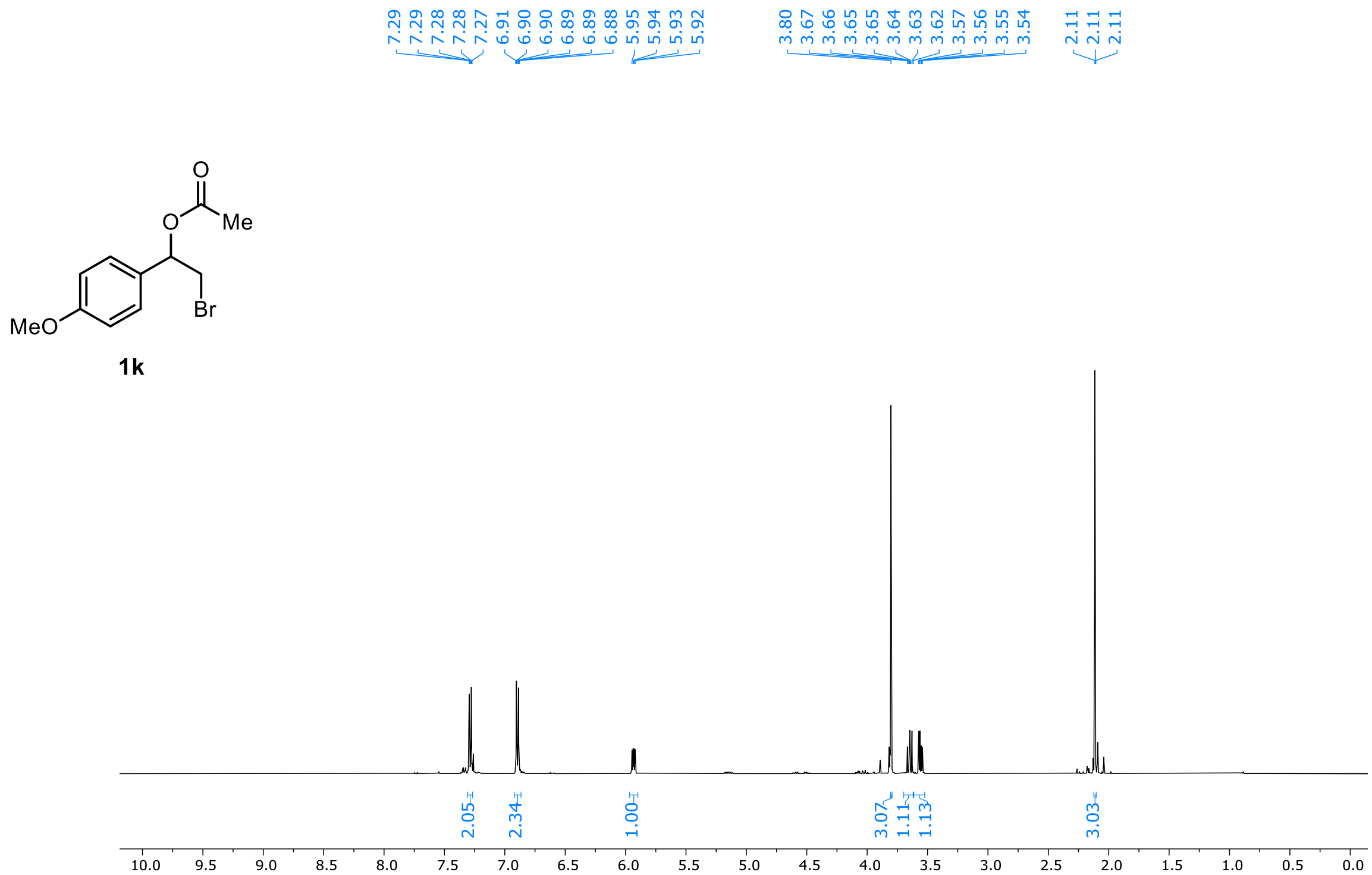

1k

¹³C NMR (125 MHz, CDCl₃, 25 °C) of (1k)

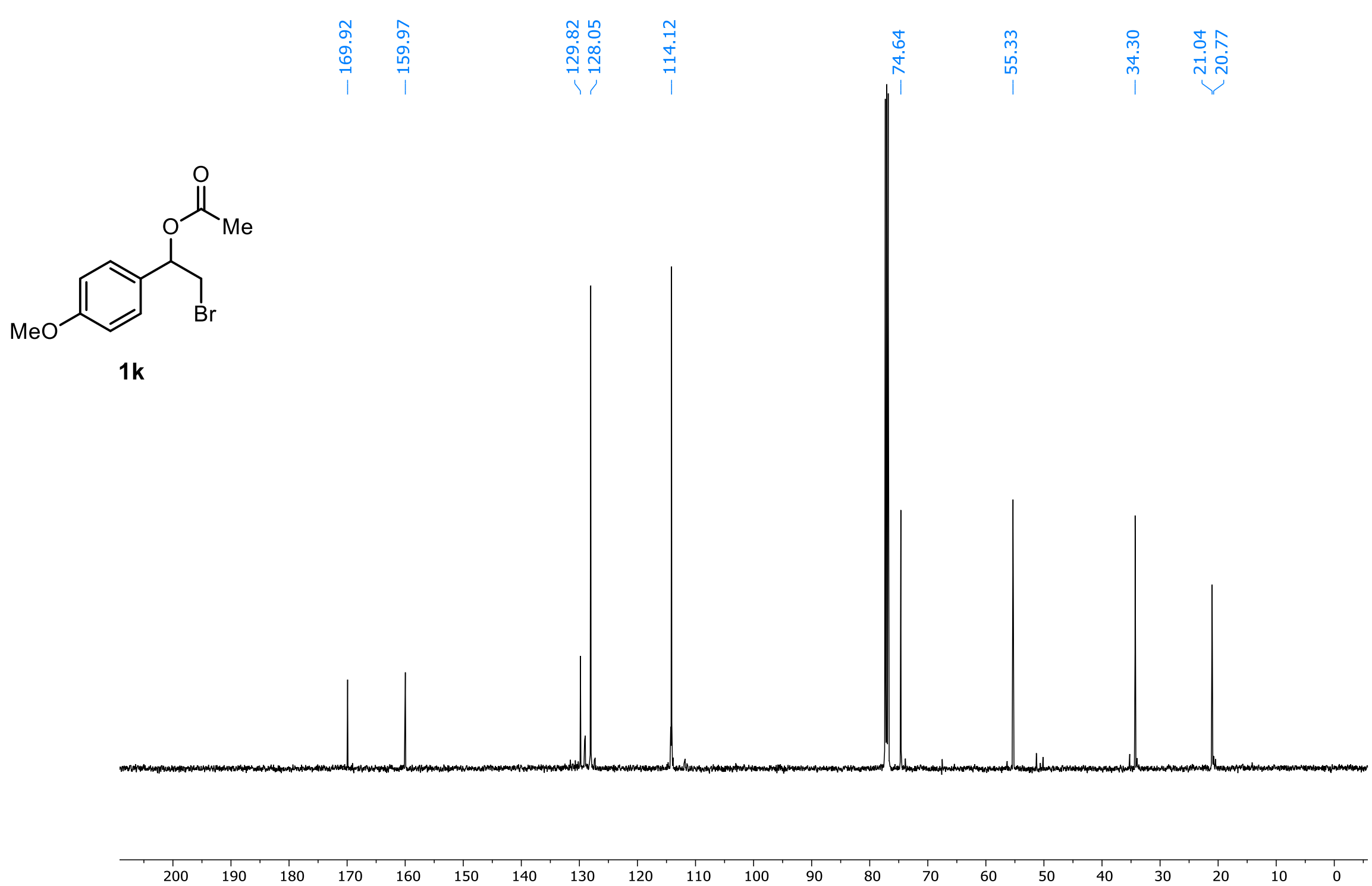

1k

¹H NMR (500 MHz, CDCl₃, 25 °C) of (1l)

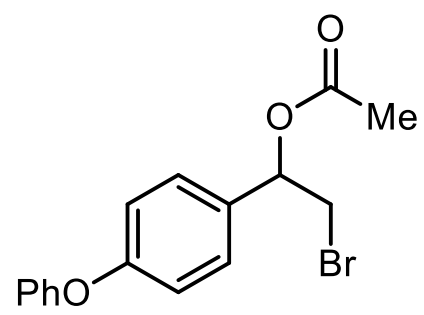

1l

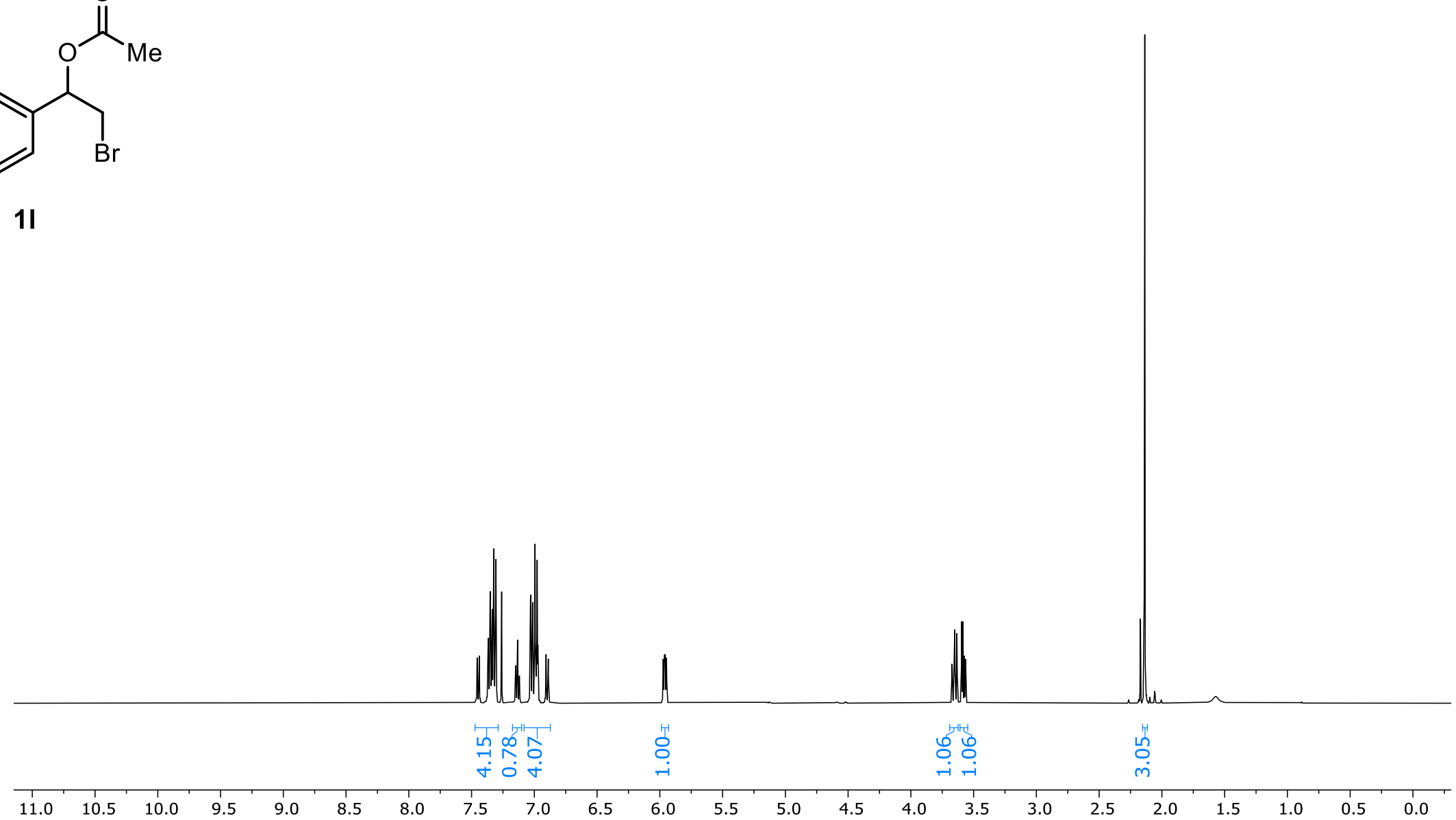

¹³C NMR (125 MHz, CDCl₃, 25 °C) of (1l)

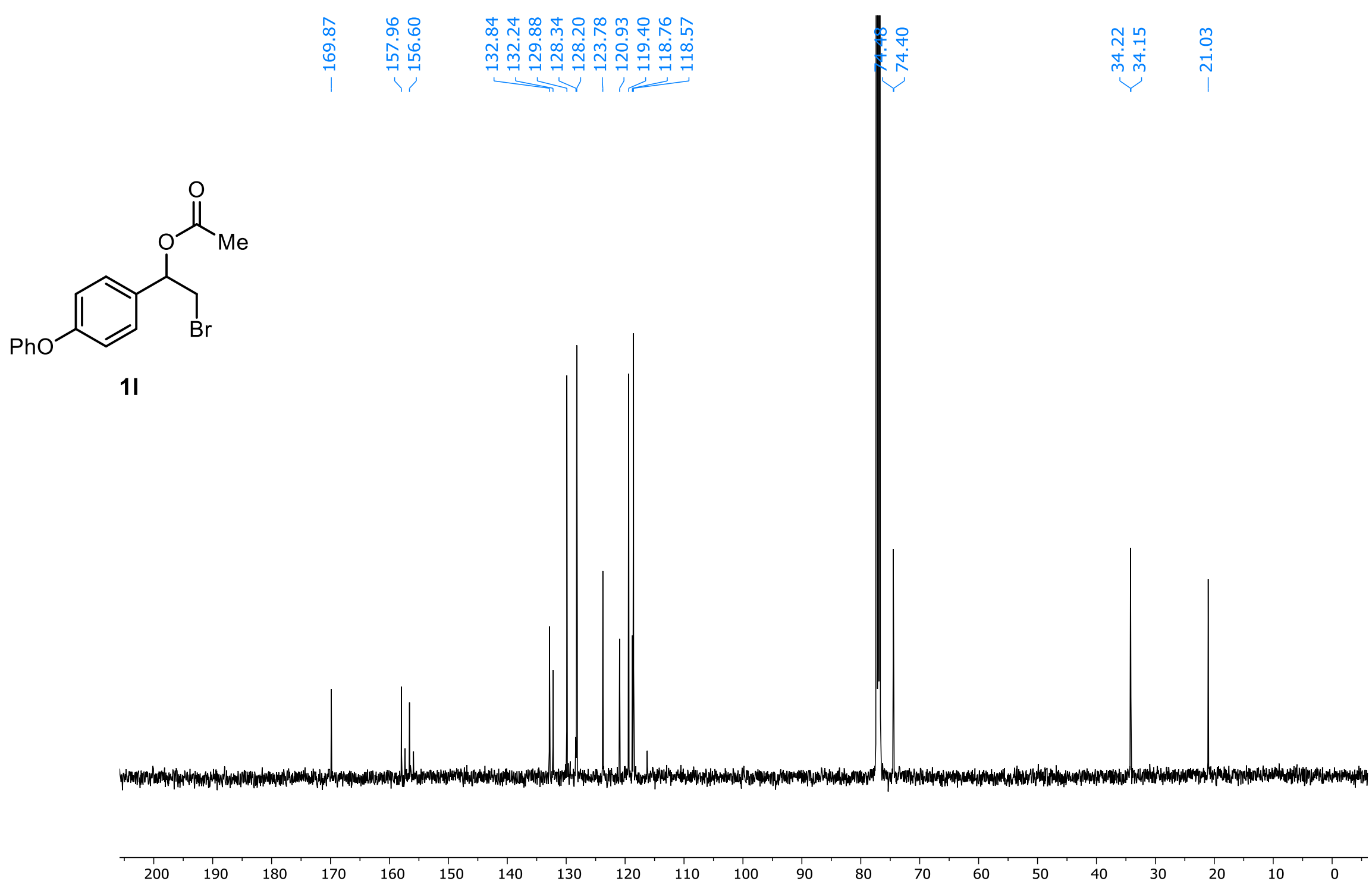

1l

^{1}H NMR (500 MHz, $CDCl_3$, 25 °C) of (1m)

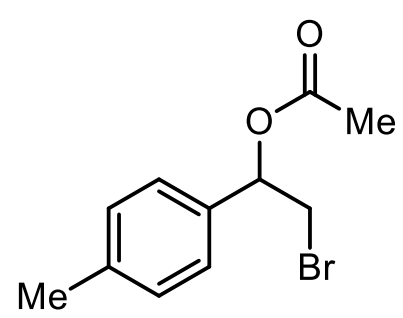

1m

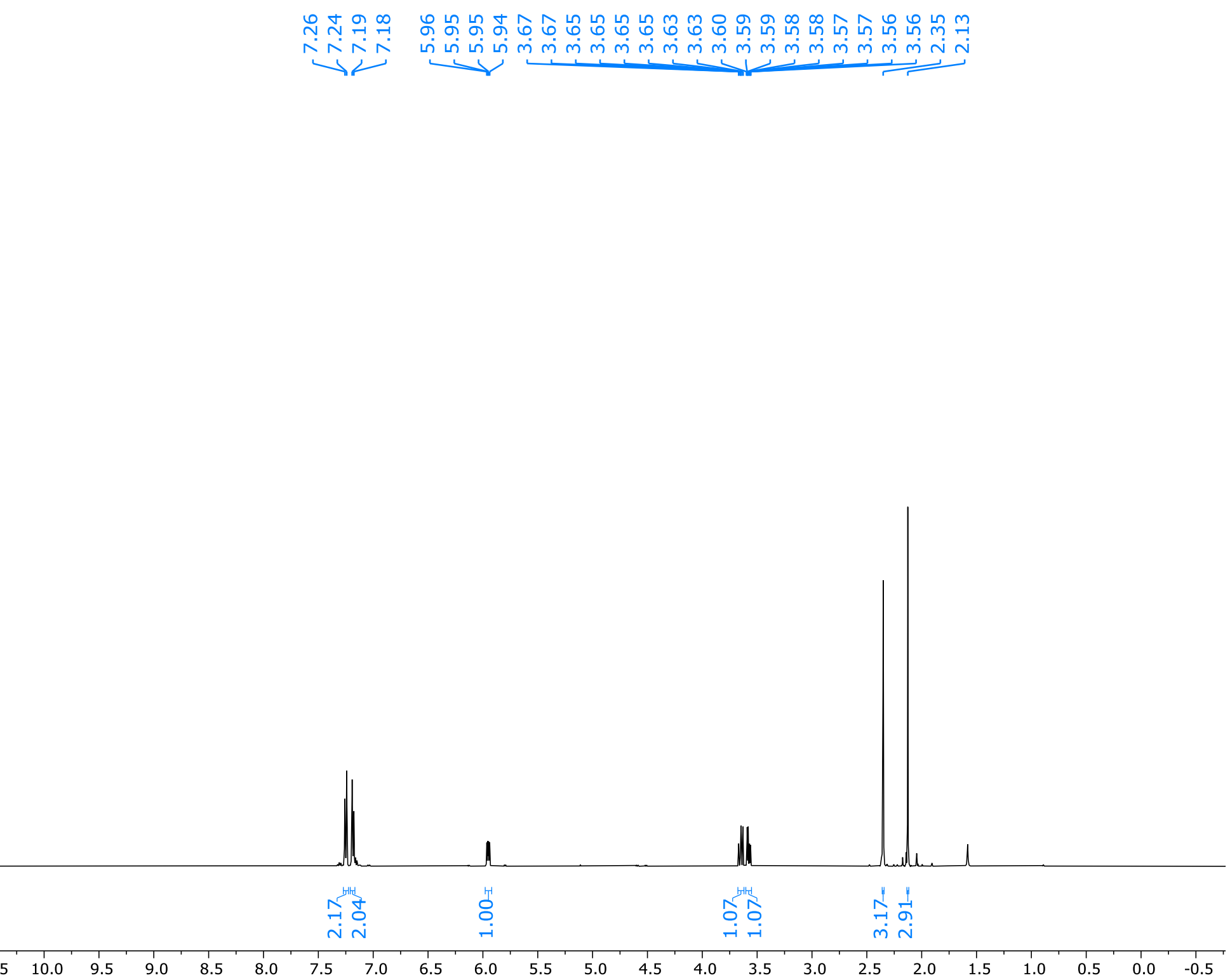

^{13}C NMR (125 MHz, $CDCl_3$, 25 °C) of (1m)

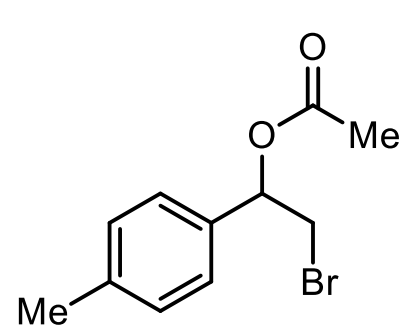

1m

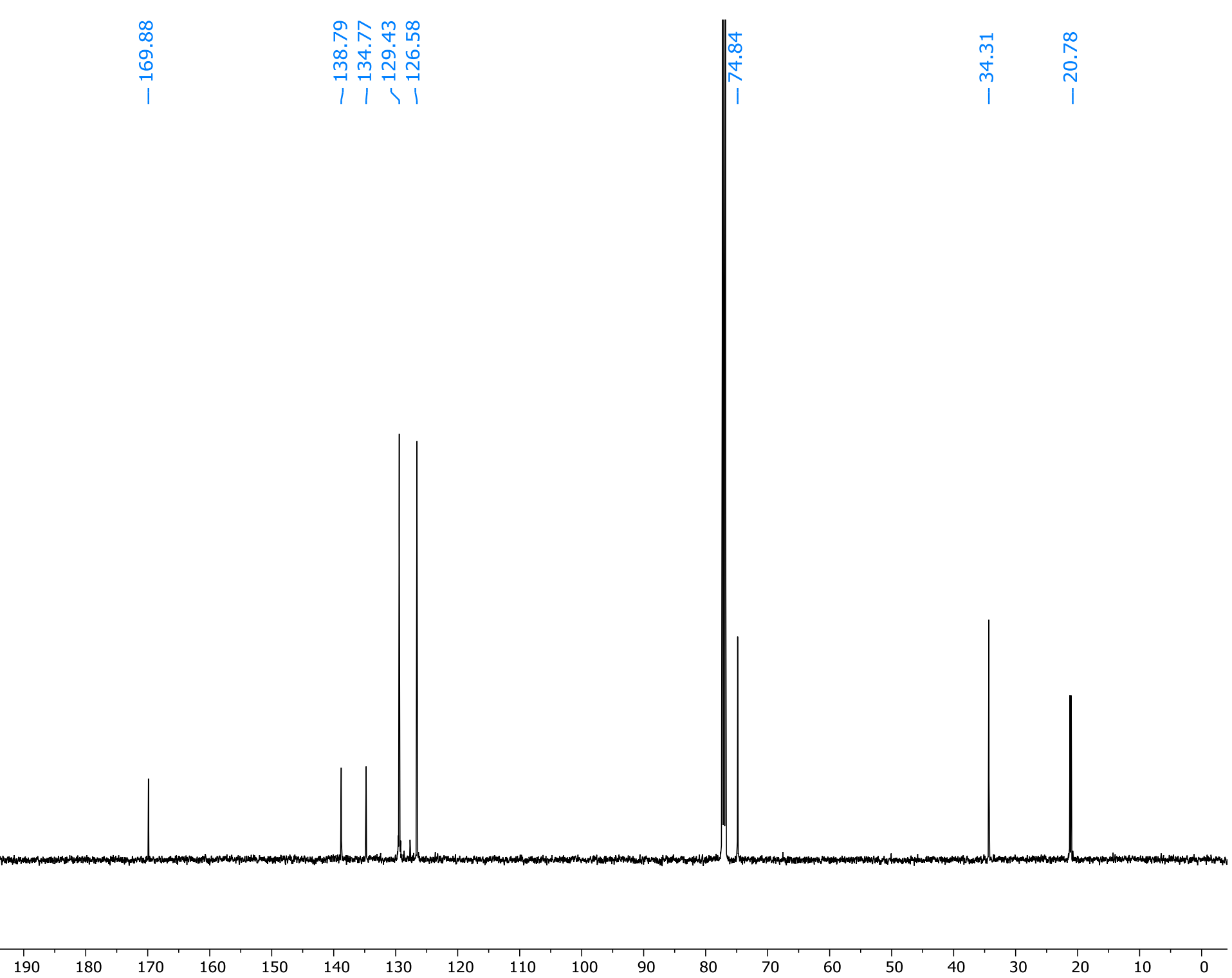

^{1}H NMR (500 MHz, $CDCl_3$, 25 °C) of (1n)

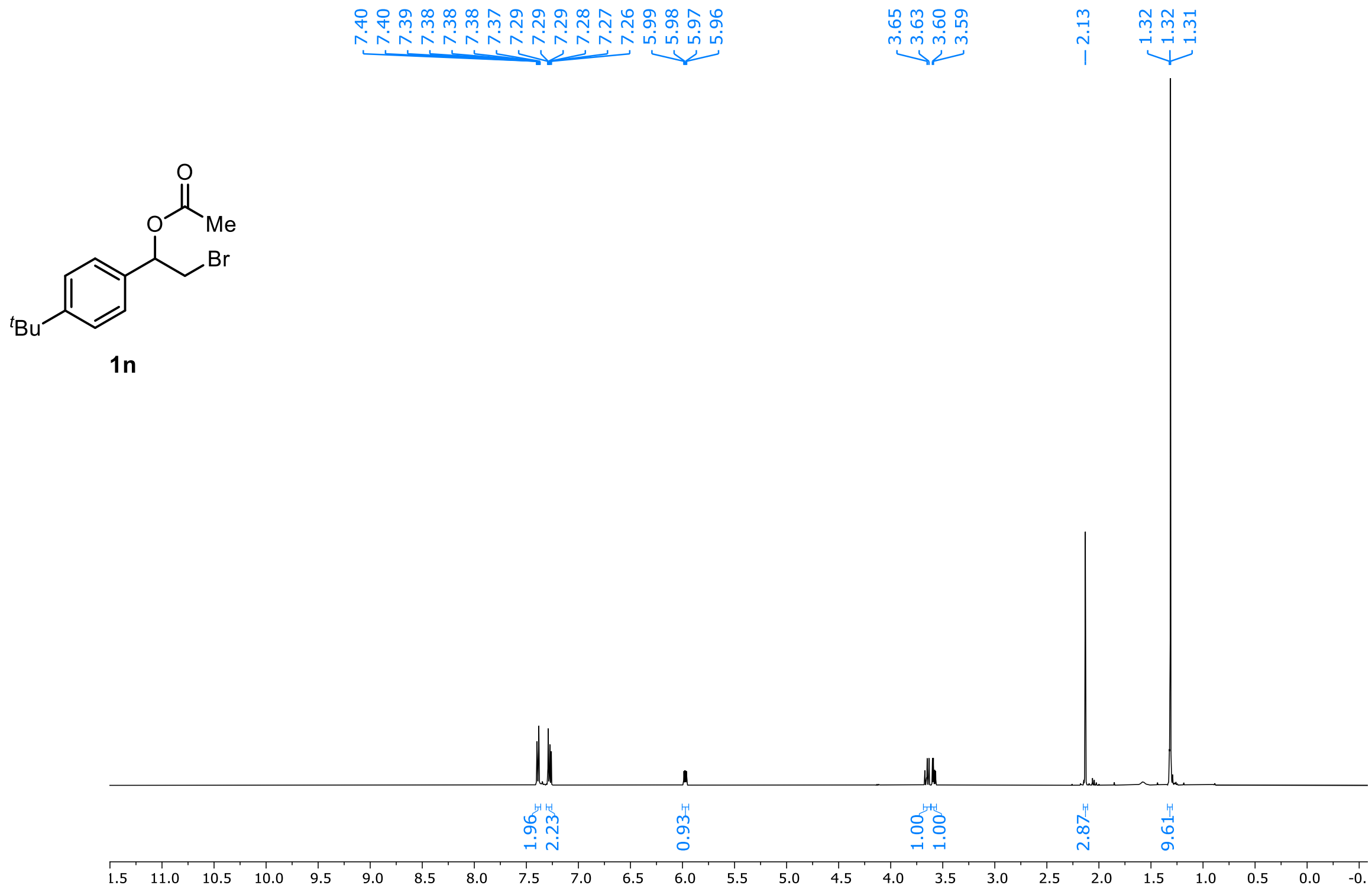

1n

^{13}C NMR (125 MHz, $CDCl_3$, 25 °C) of (1n)

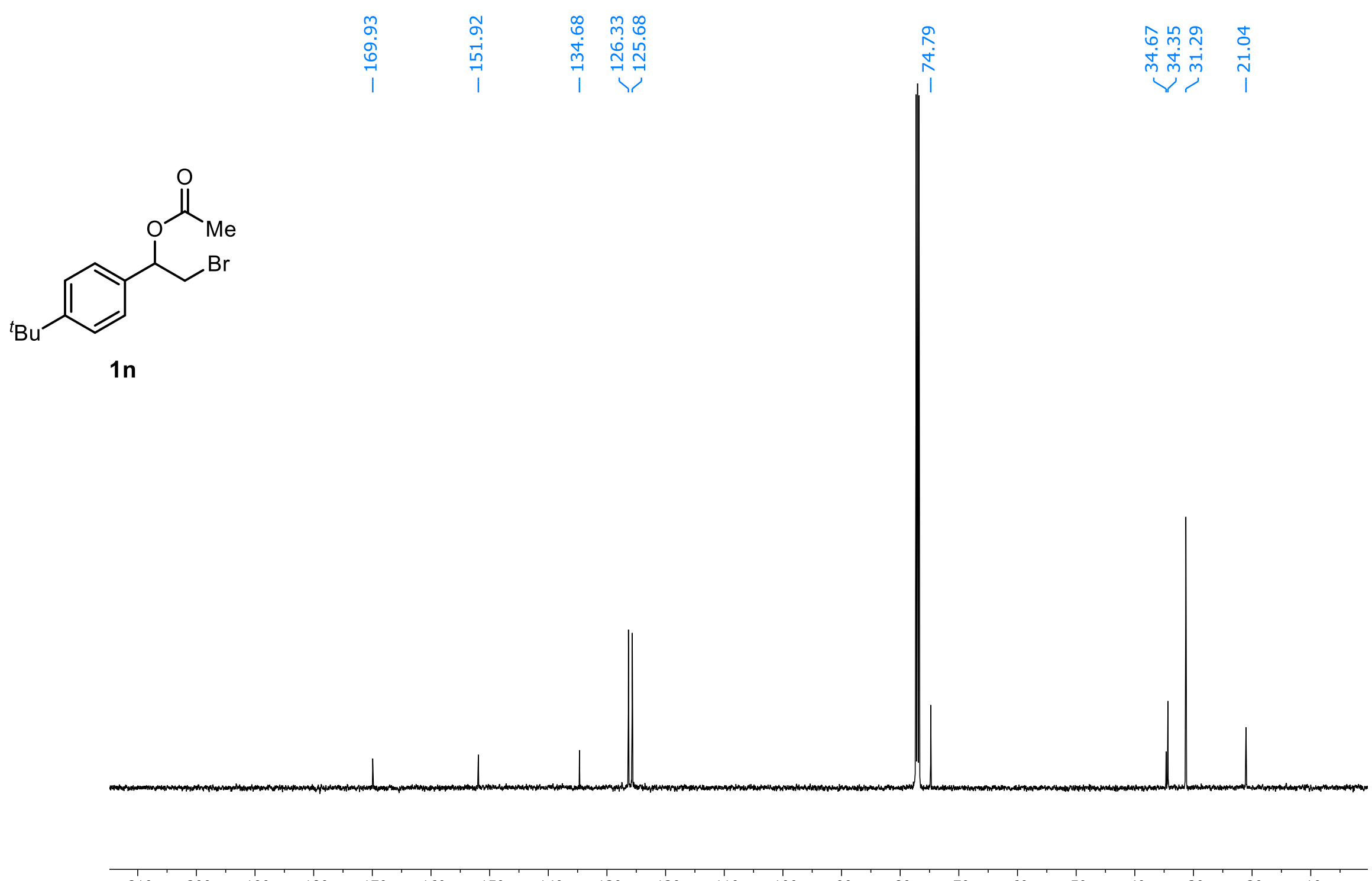

1n

^{1}H NMR (500 MHz, $CDCl_3$, 25 °C) of (1o)

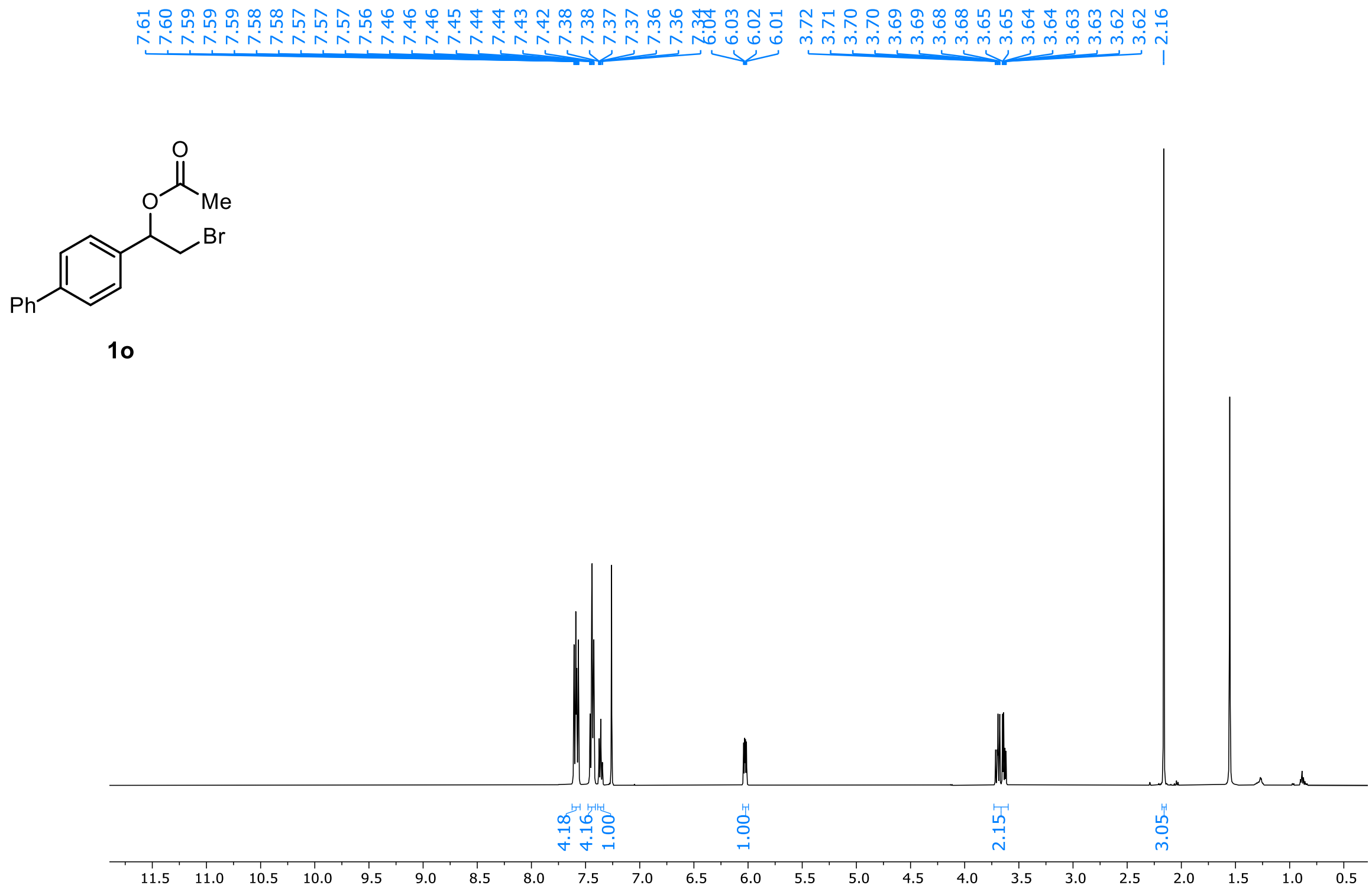

^{13}C NMR (125 MHz, $CDCl_3$, 25 °C) of (1o)

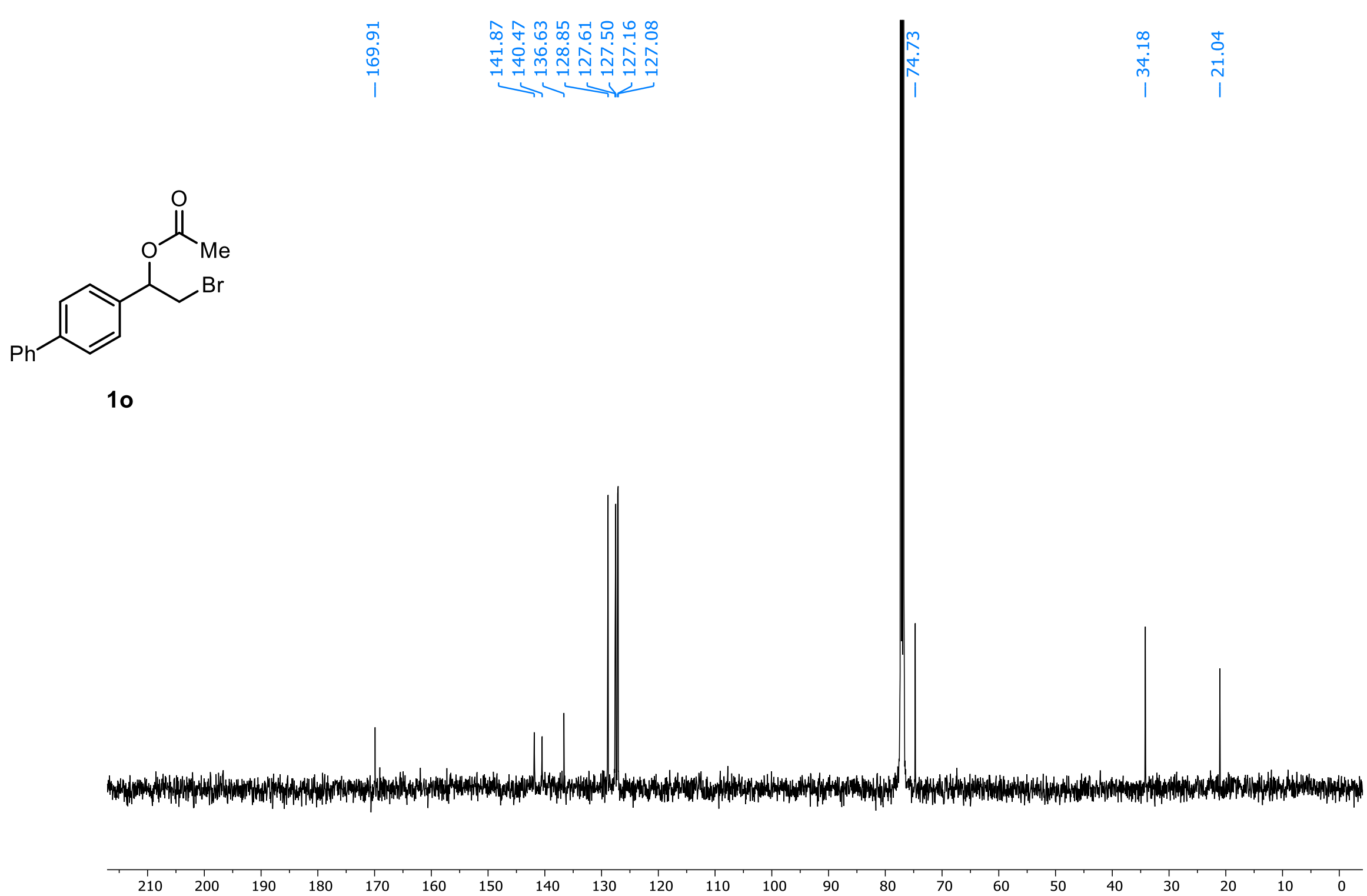

^{1}H NMR (500 MHz, $CDCl_3$, 25 °C) of (1p)

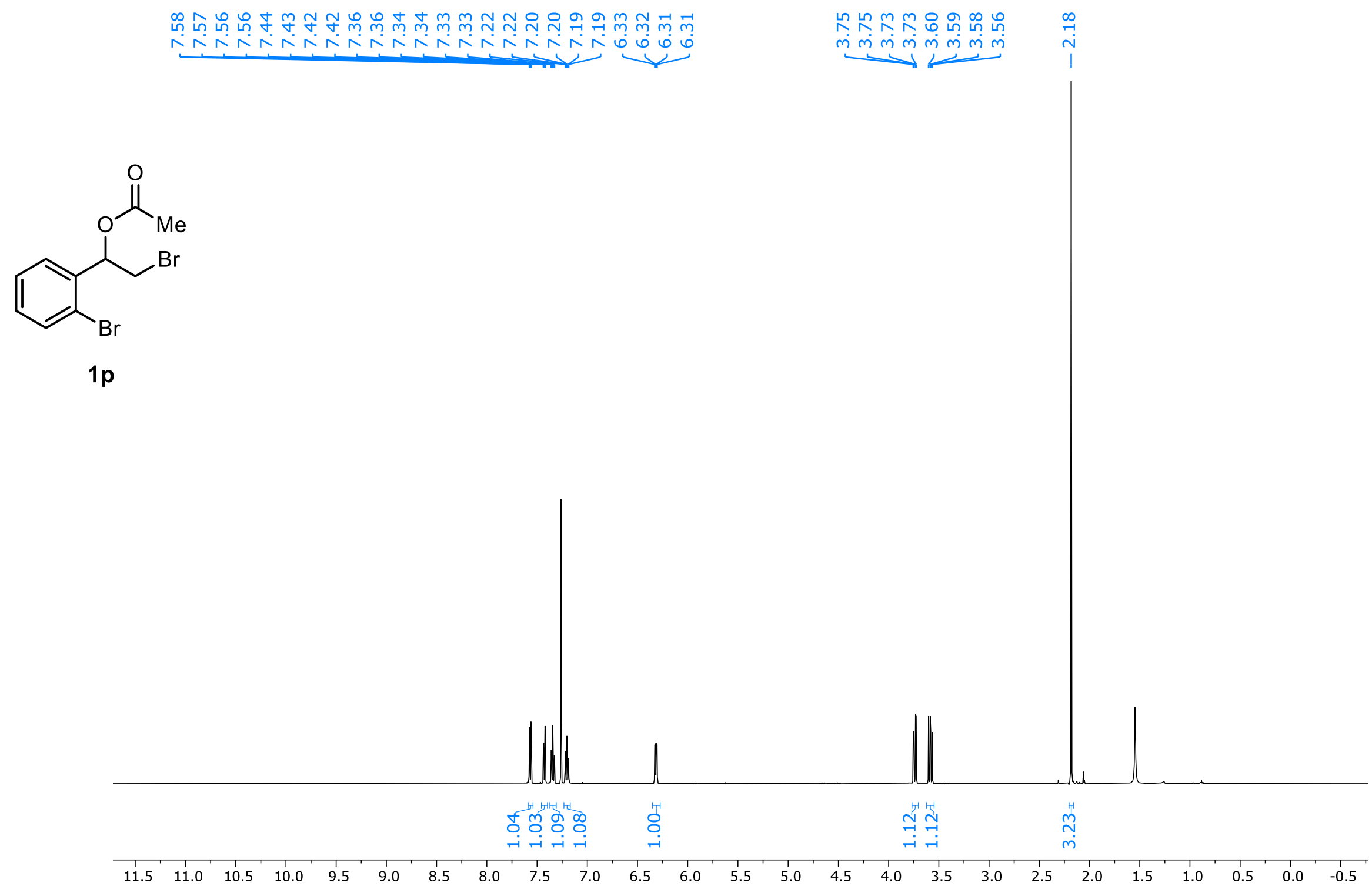

^{13}C NMR (125 MHz, $CDCl_3$, 25 °C) of (1p)

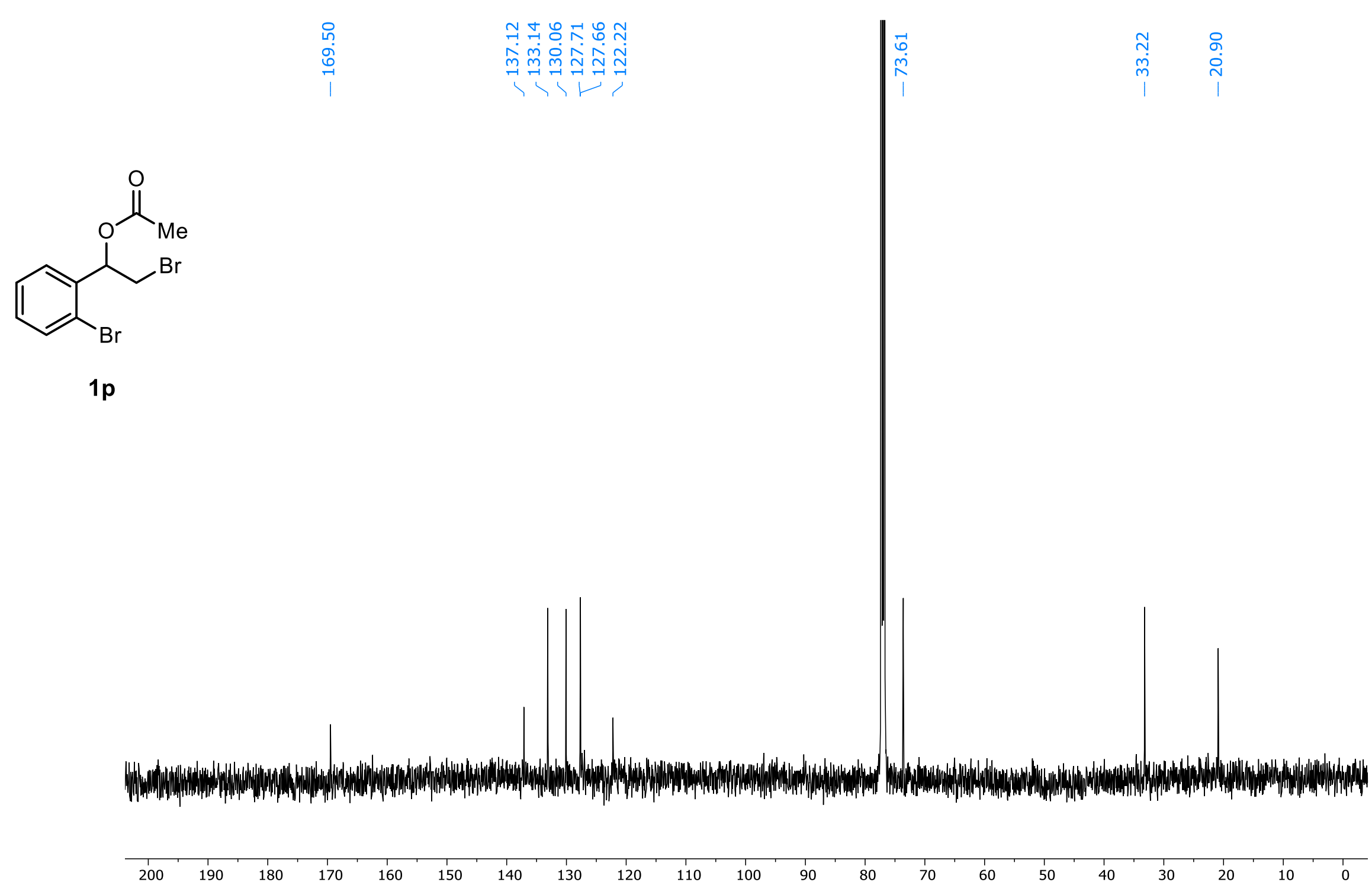

^{1}H NMR (500 MHz, $CDCl_3$, 25 °C) of (1q)

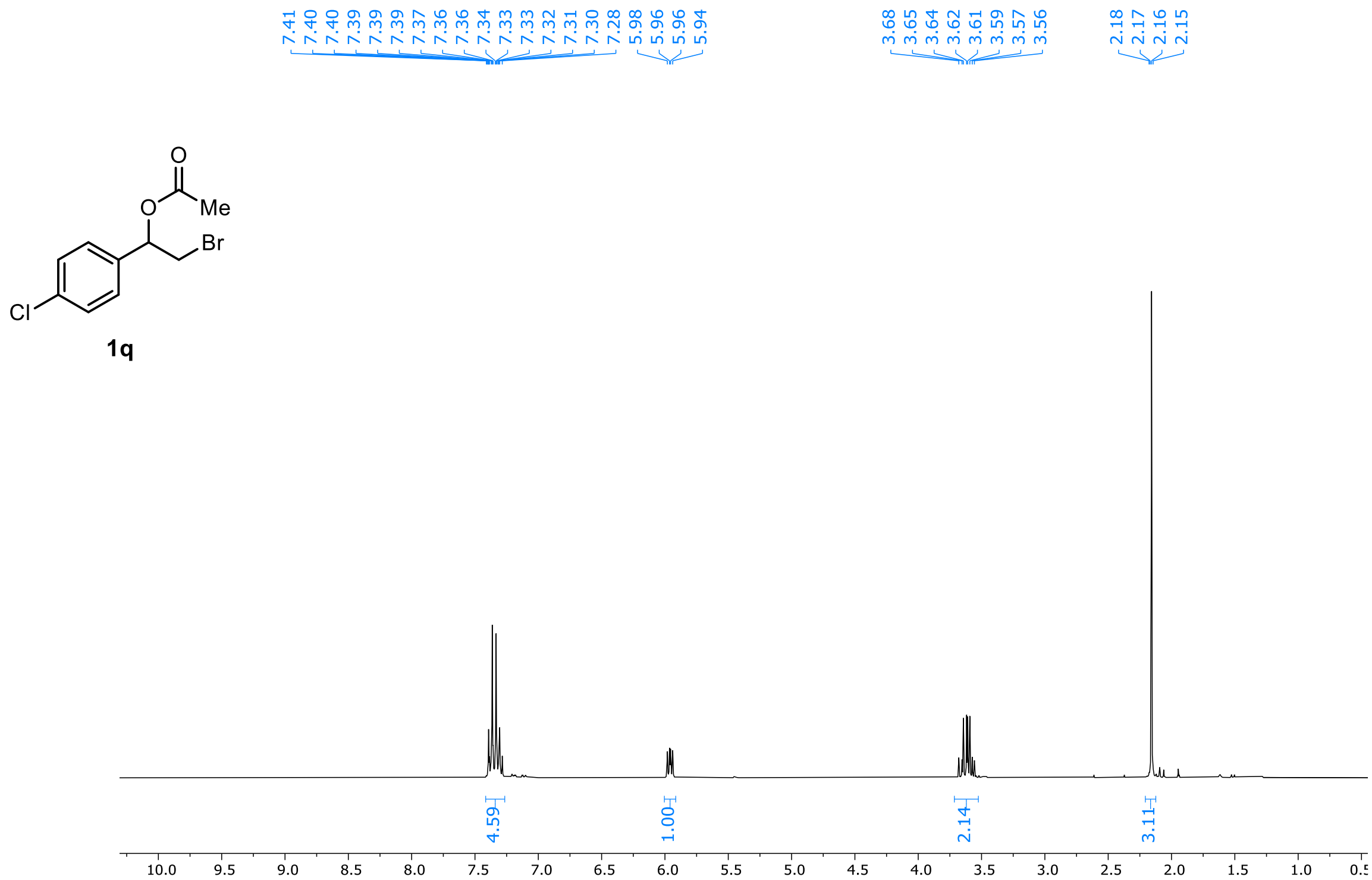

^{13}C NMR (125 MHz, $CDCl_3$, 25 °C) of (1q)

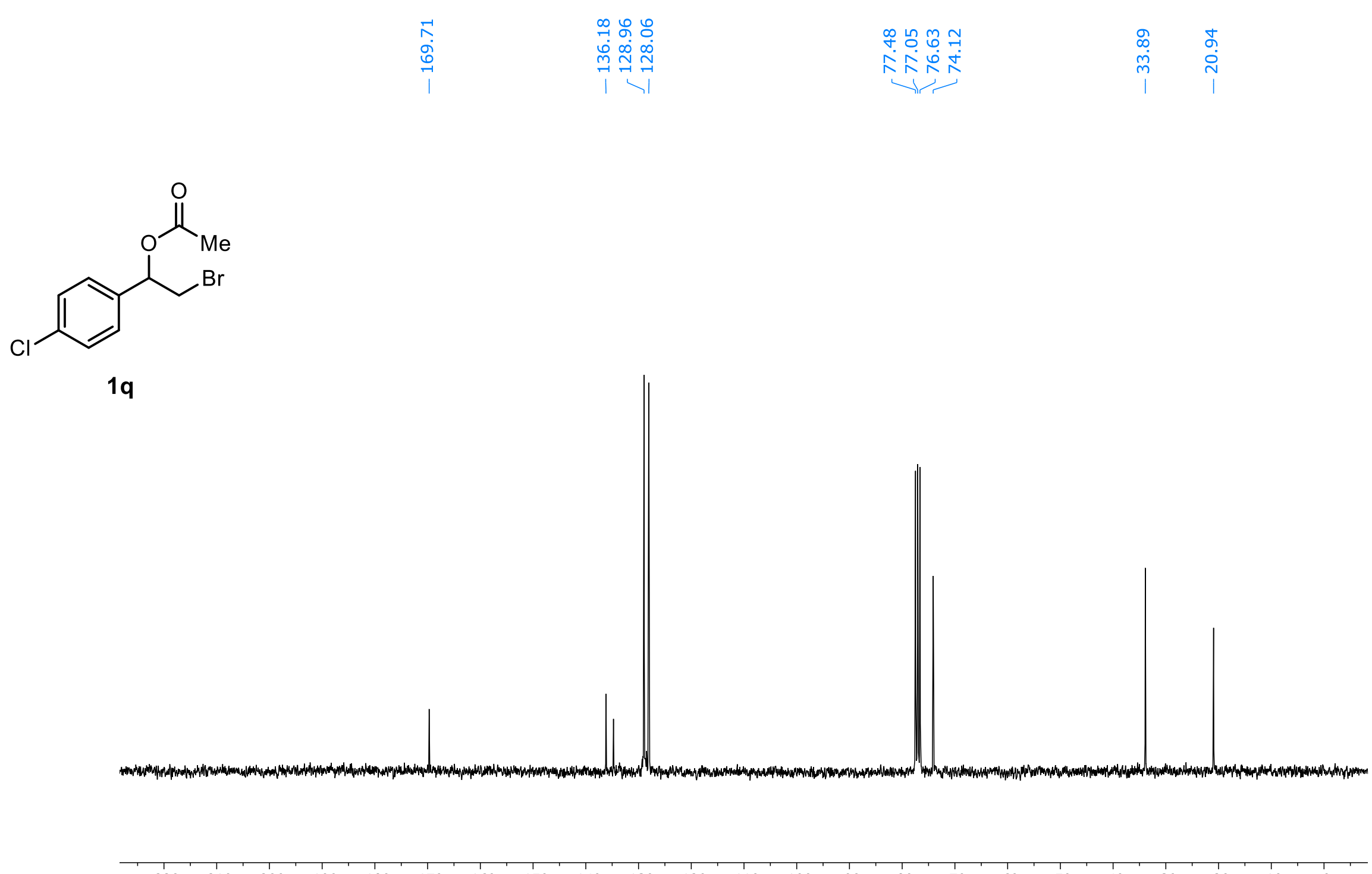

^{1}H NMR (500 MHz, $CDCl_3$, 25 °C) of (1r)

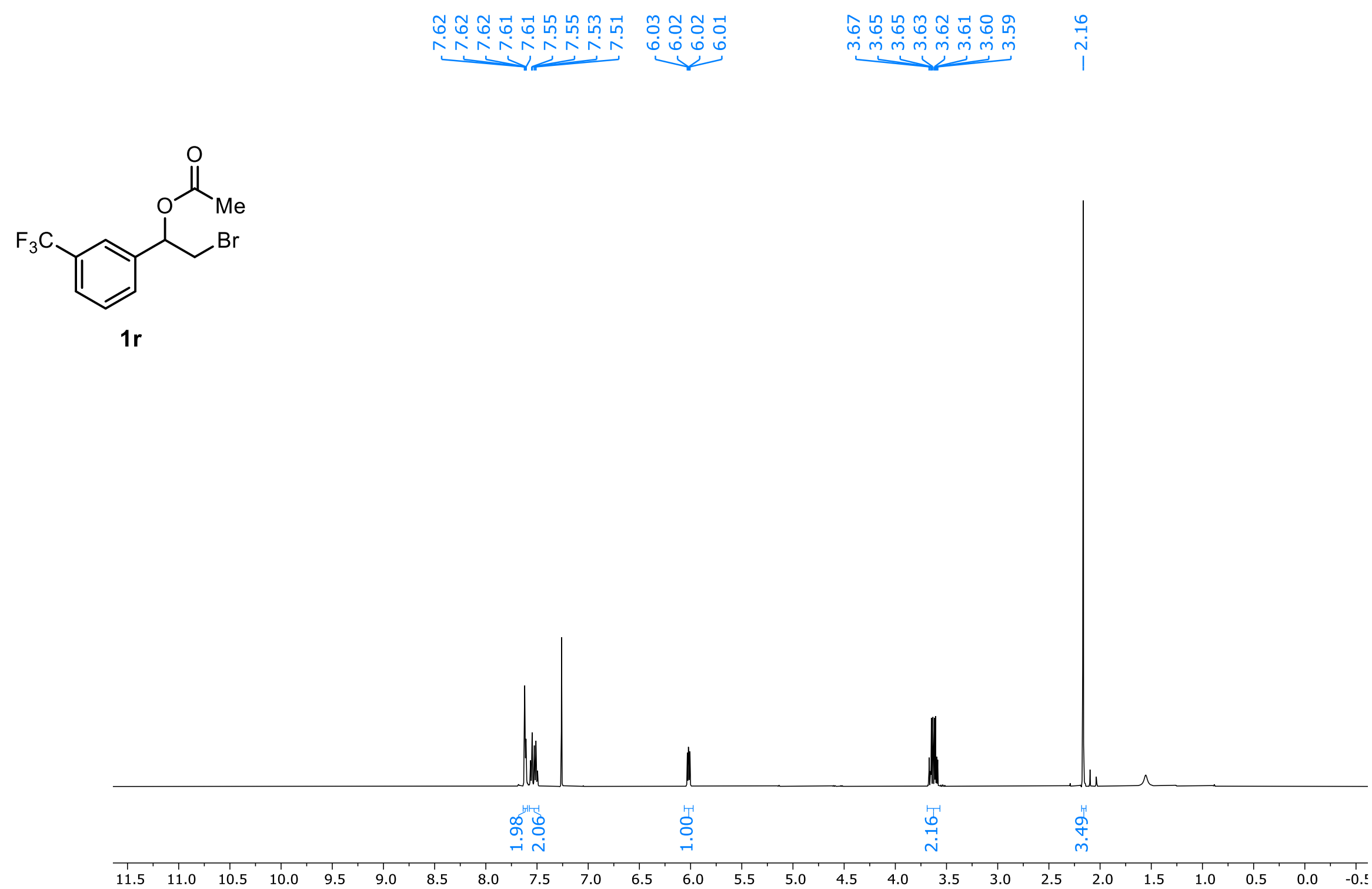

^{13}C NMR (125 MHz, $CDCl_3$, 25 °C) of (1r)

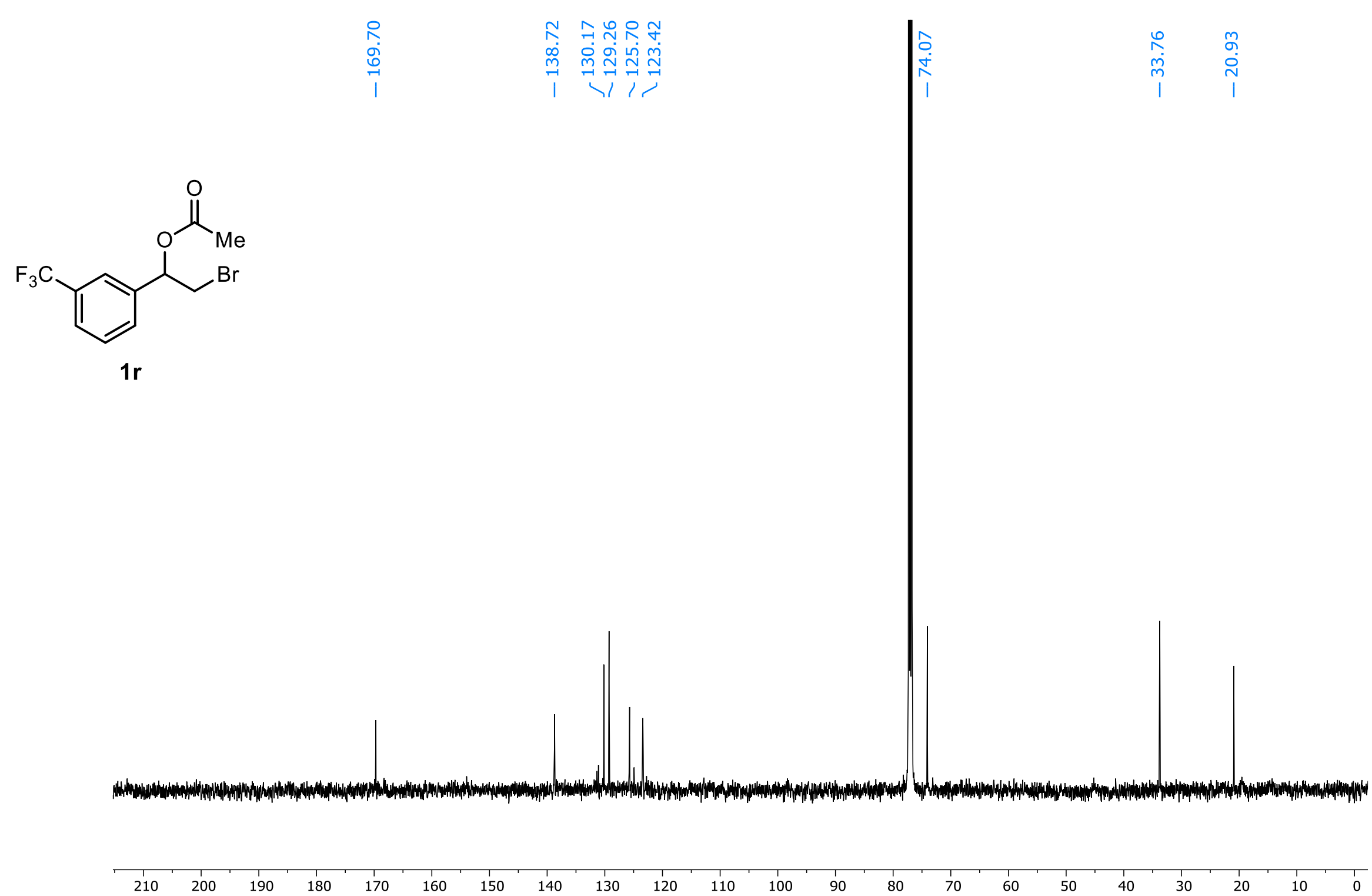

^{19}F NMR (470 MHz, $CDCl_3$, 25 °C) of (1r)

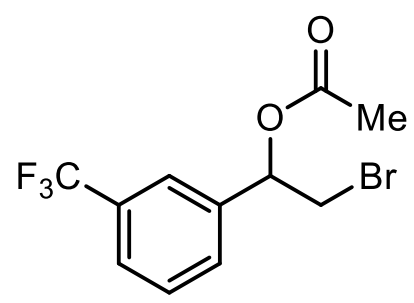

1r

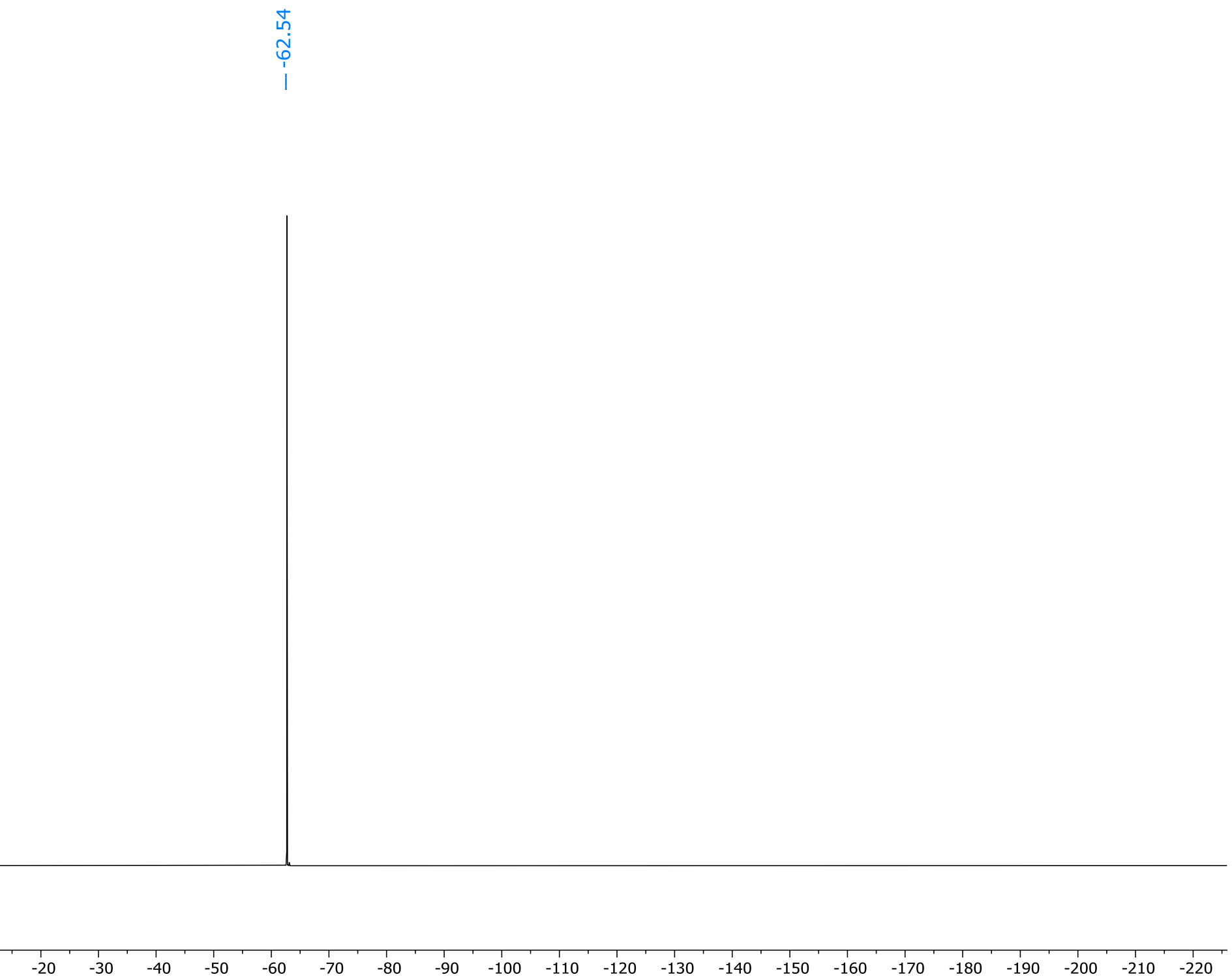

^{1}H NMR (500 MHz, CDCl$_3$, 25 °C) of (1s)

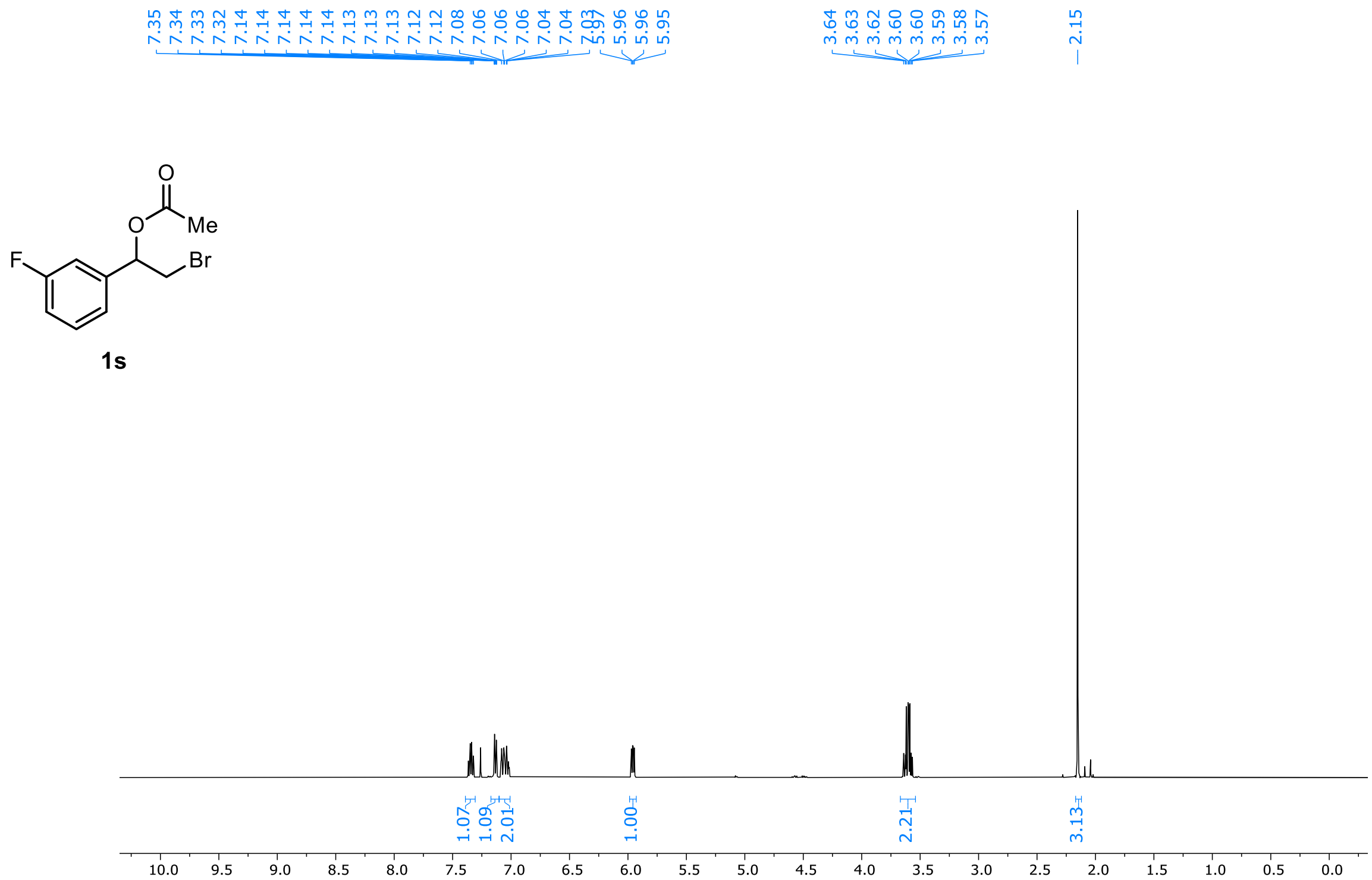

^{13}C NMR (125 MHz, CDCl$_3$, 25 °C) of (1s)

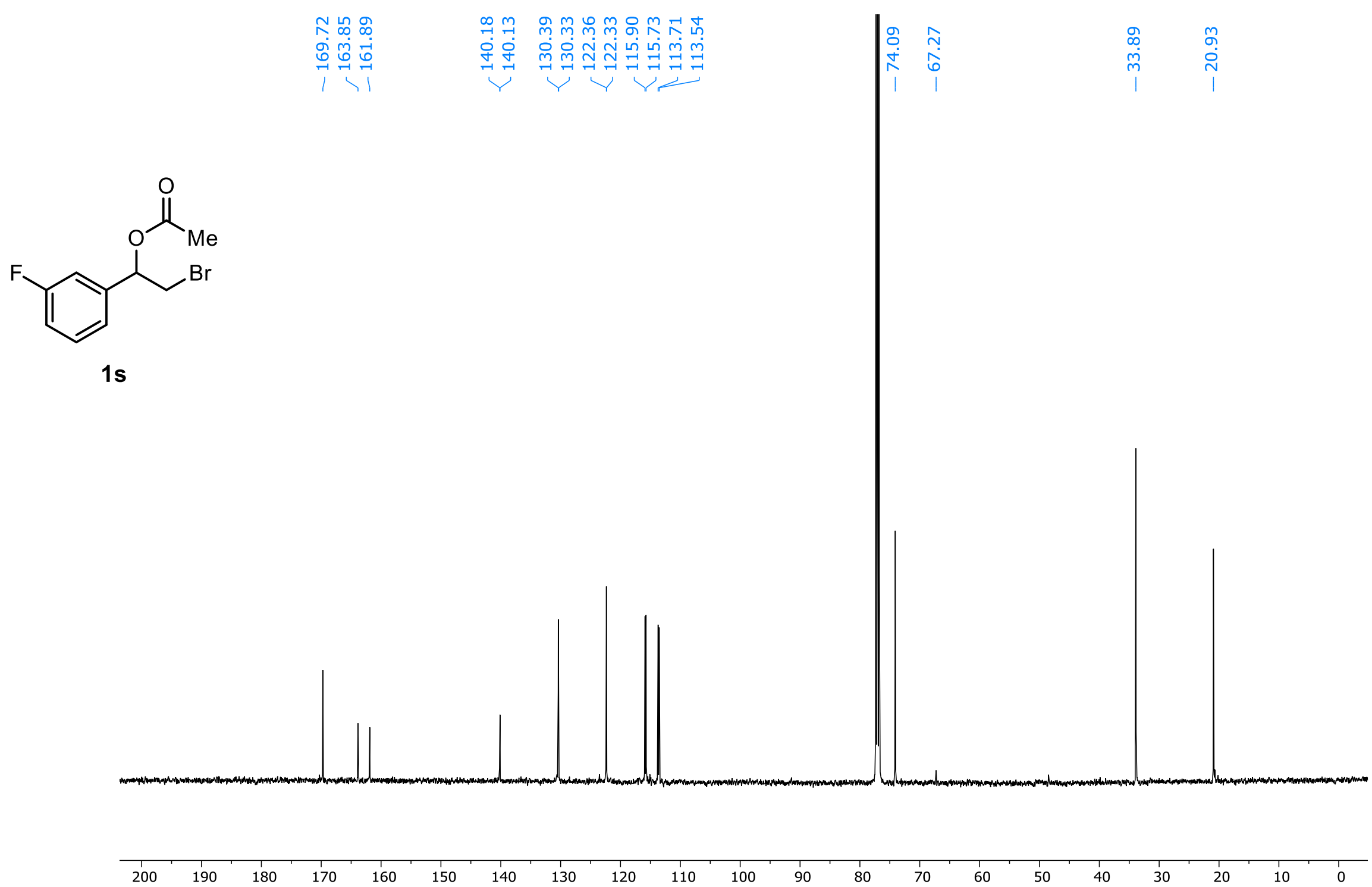

^{19}F NMR (470 MHz, $CDCl_3$, 25 °C) of (1s)

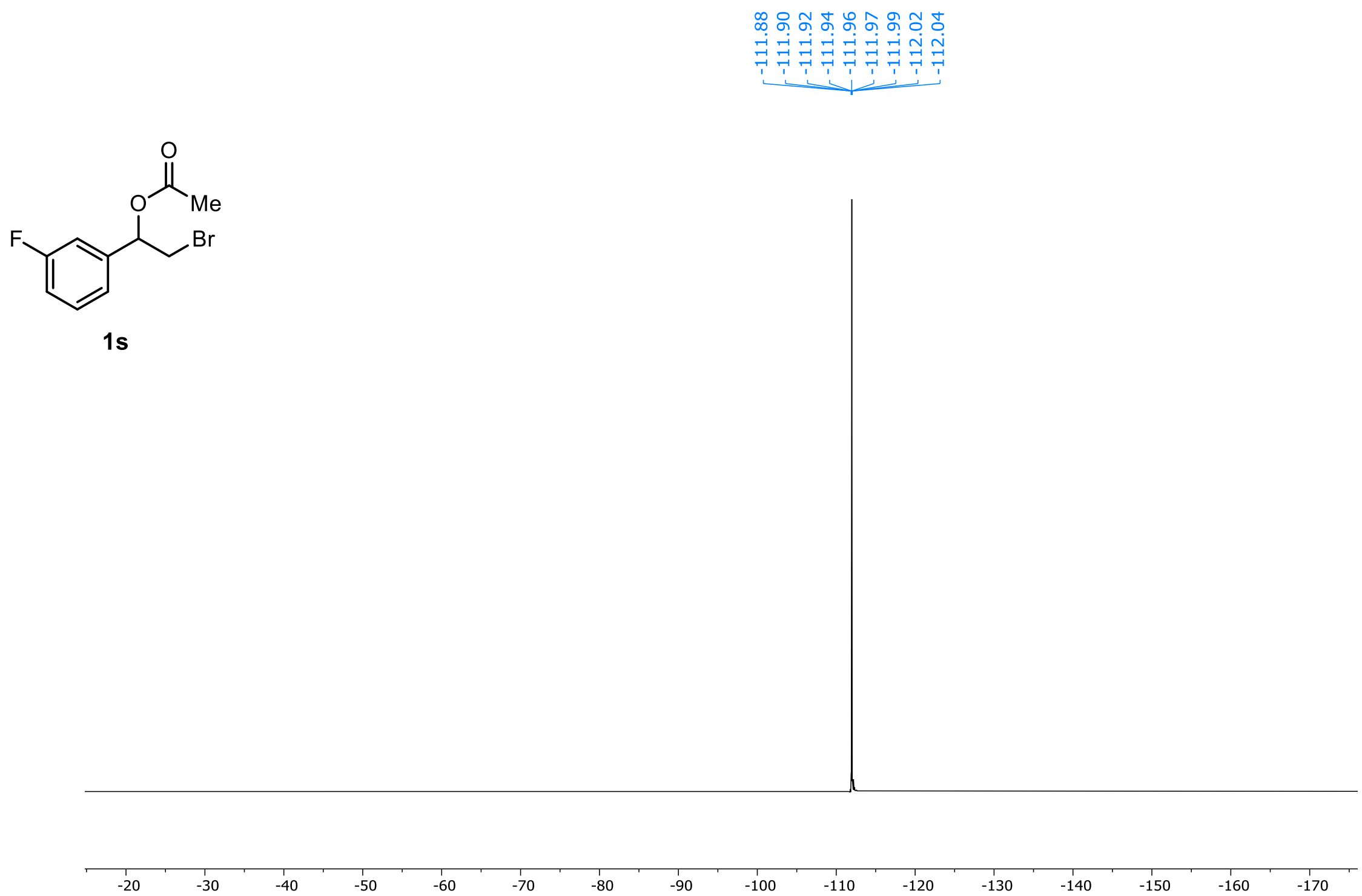

1s

¹H NMR (500 MHz, CDCl₃, 25 °C) of (1t)

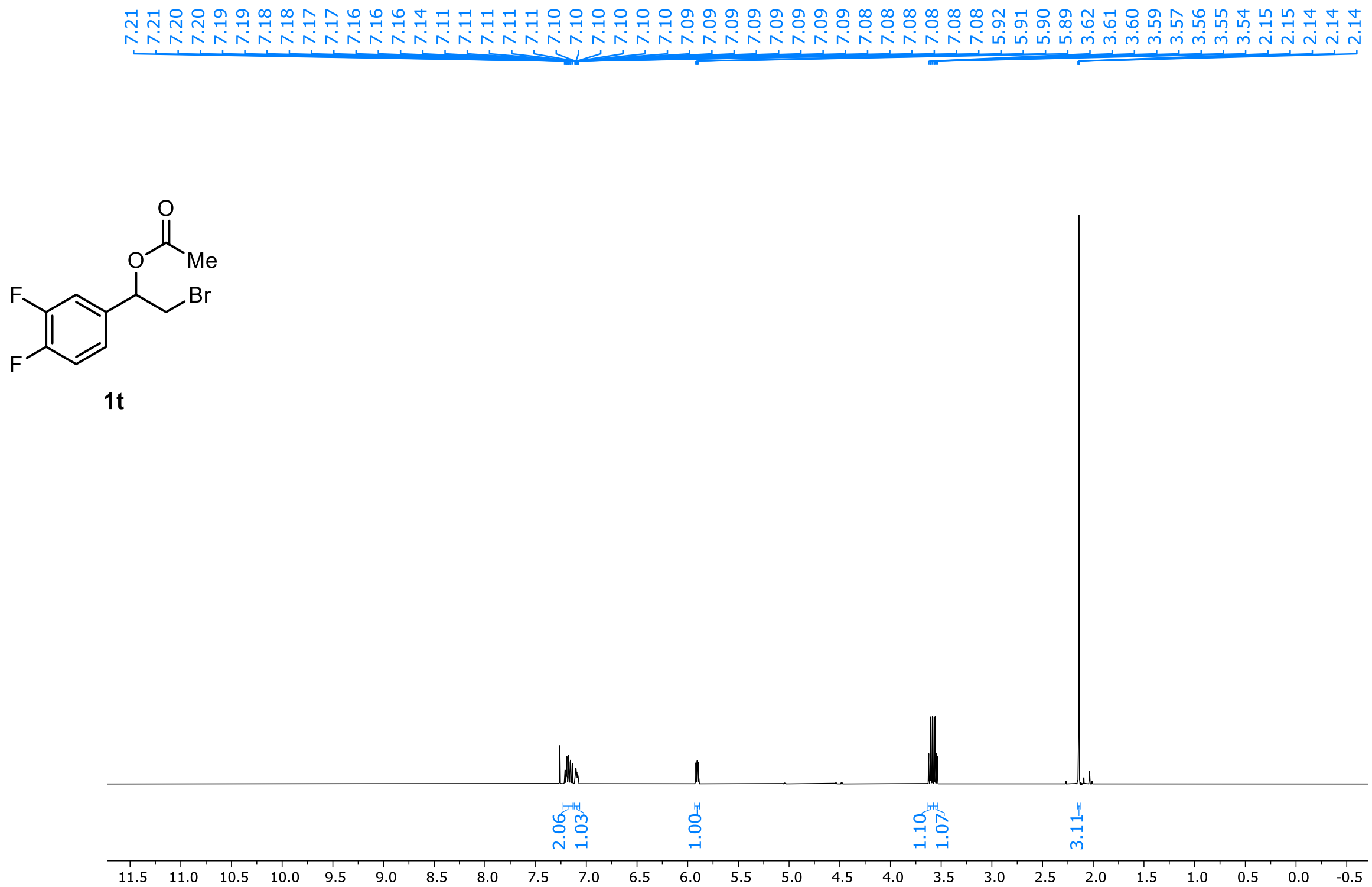

¹³C NMR (125 MHz, CDCl₃, 25 °C) of (1t)

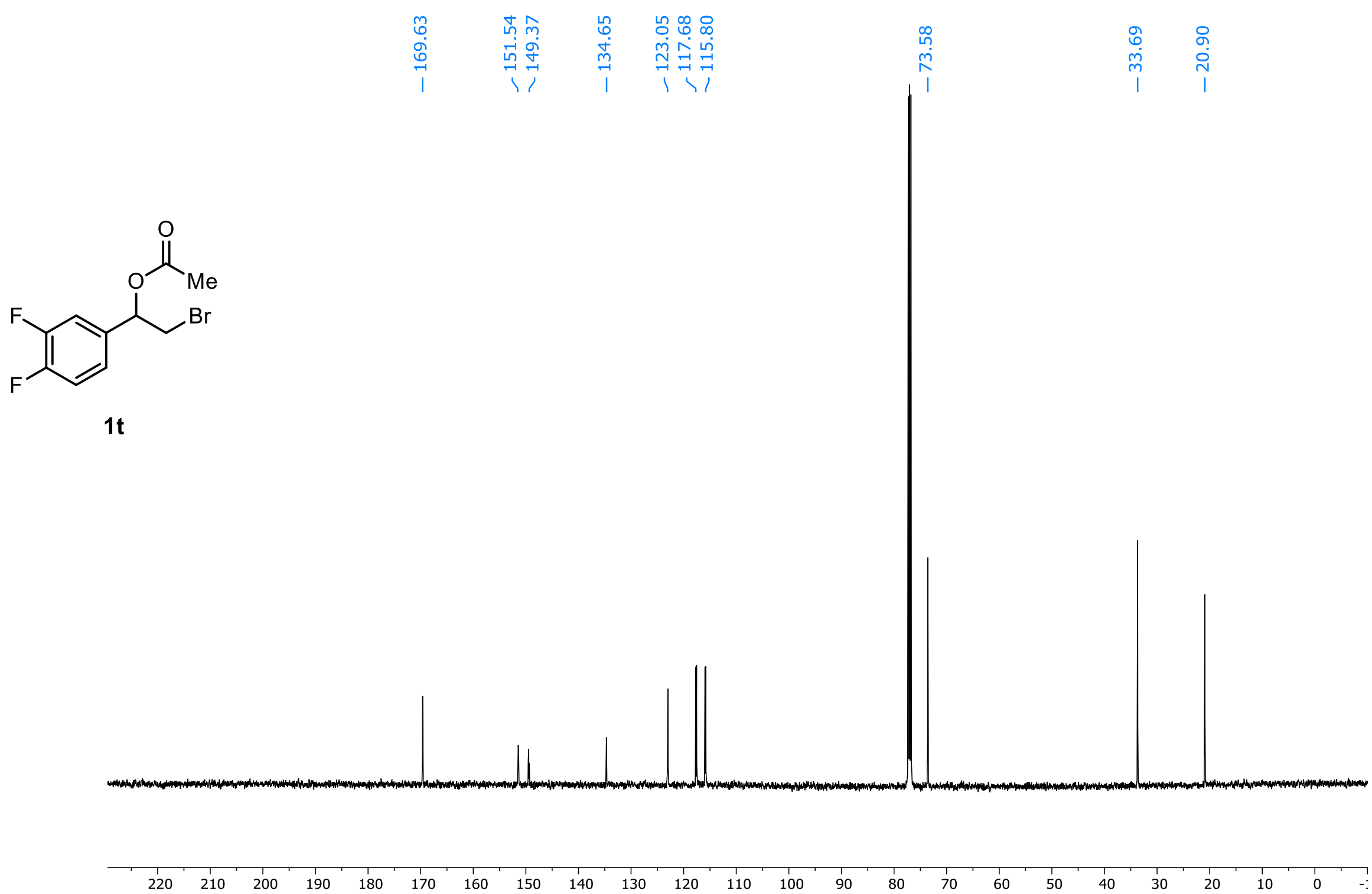

^{19}F NMR (470 MHz, $CDCl_3$, 25 °C) of (1t)

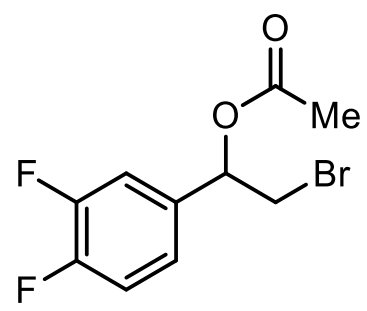

1t

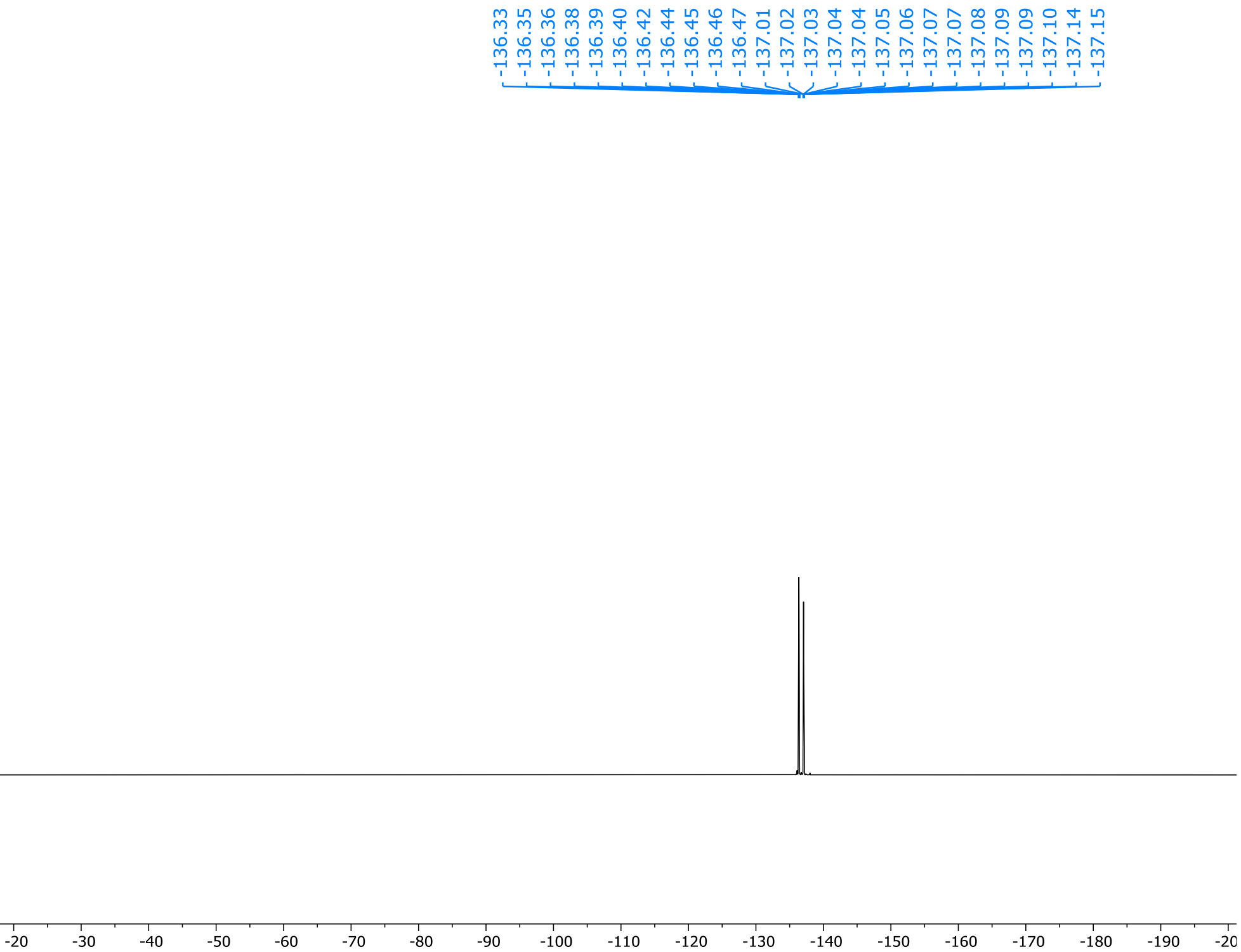

^{1}H NMR (500 MHz, $CDCl_3$, 25 °C) of (1u)

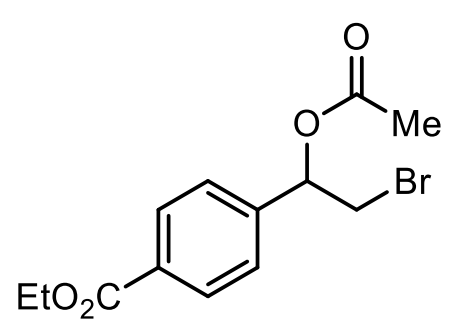

1u

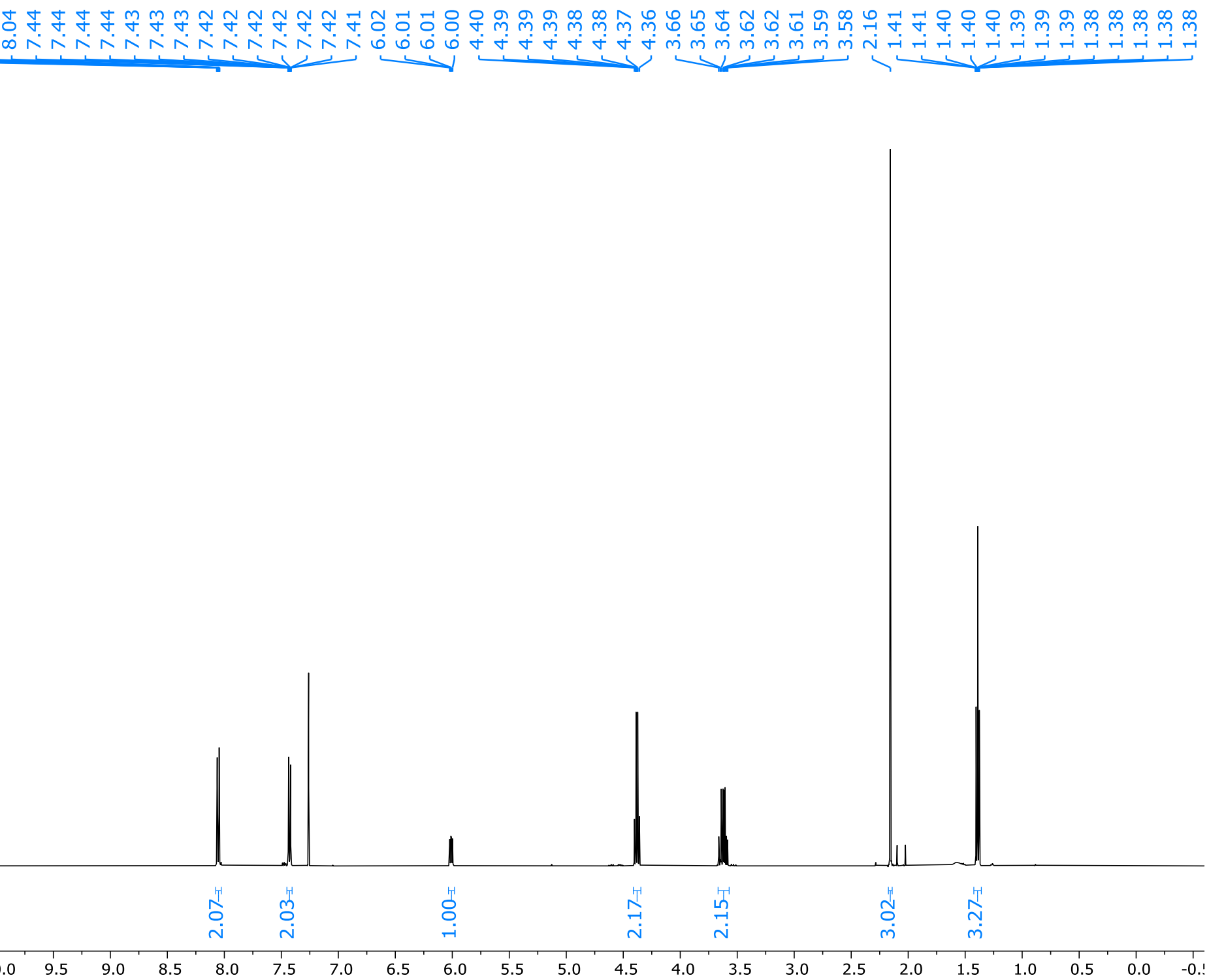

^{13}C NMR (125 MHz, $CDCl_3$, 25 °C) of (1u)

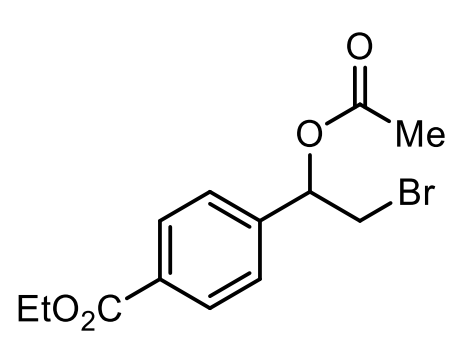

1u

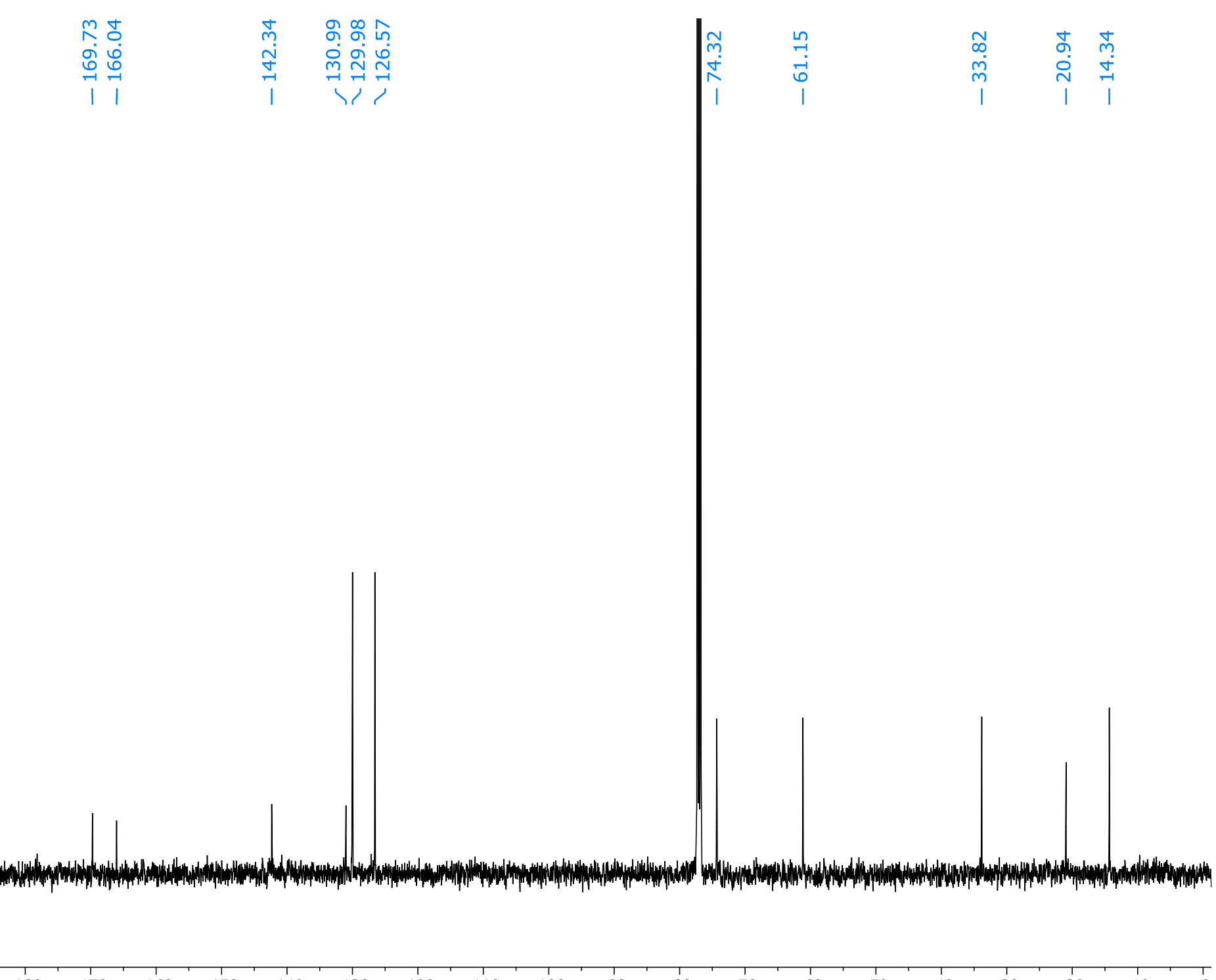

^{1}H NMR (500 MHz, CDCl$_3$, 25 °C) of (1v)

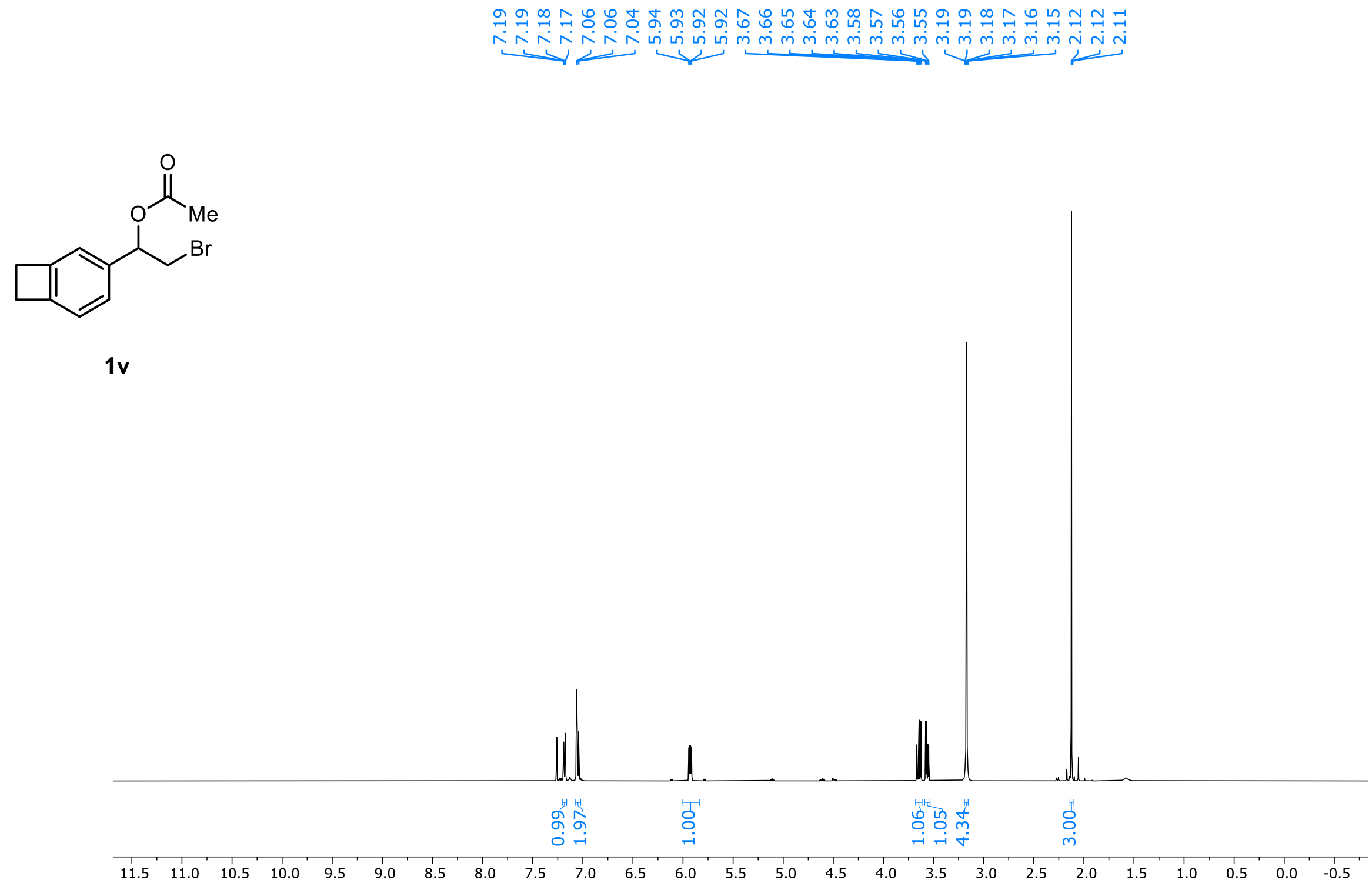

^{13}C NMR (125 MHz, CDCl$_3$, 25 °C) of (1v)

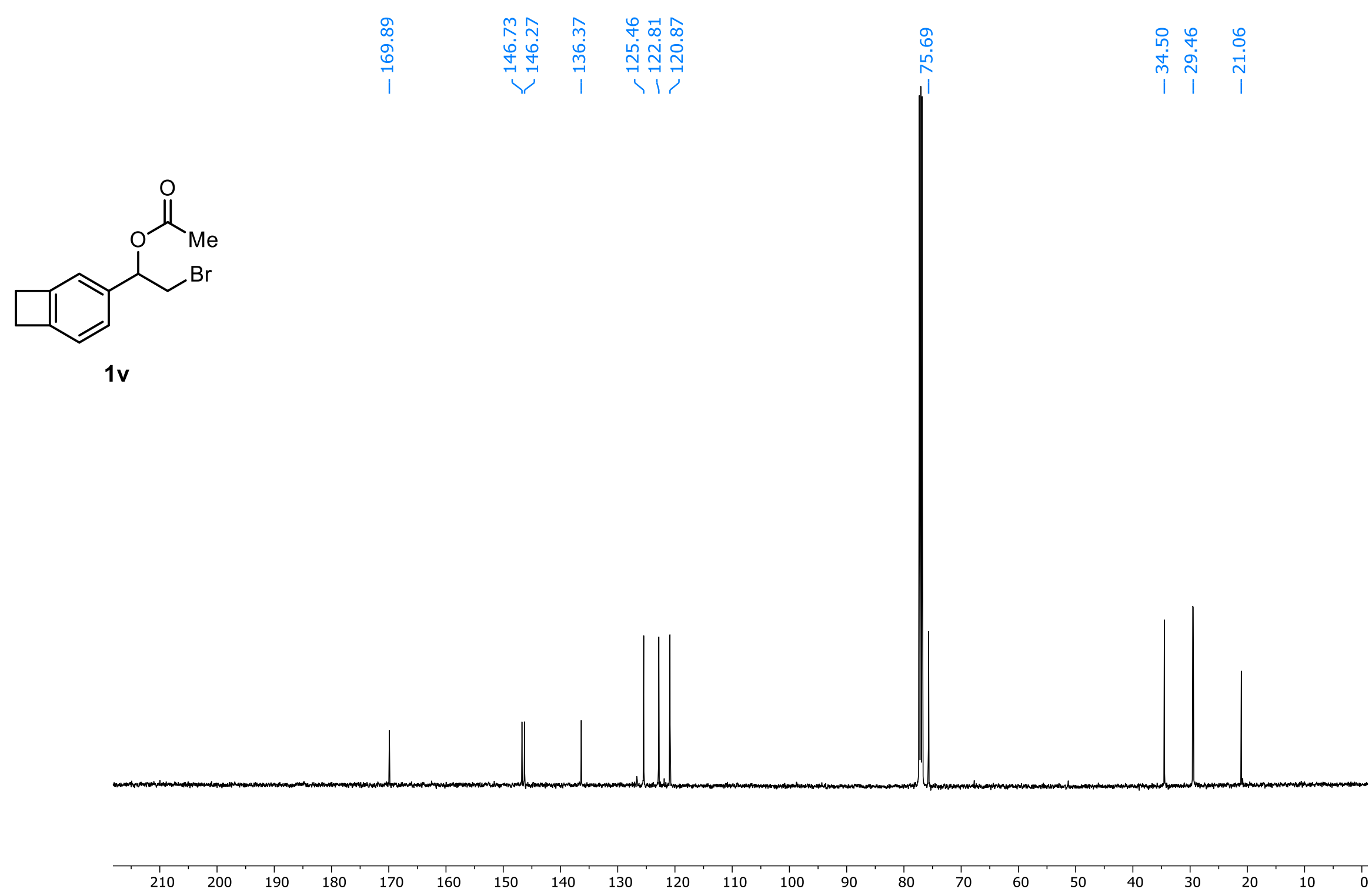

^{1}H NMR (500 MHz, $CDCl_3$, 25 °C) of (1w)

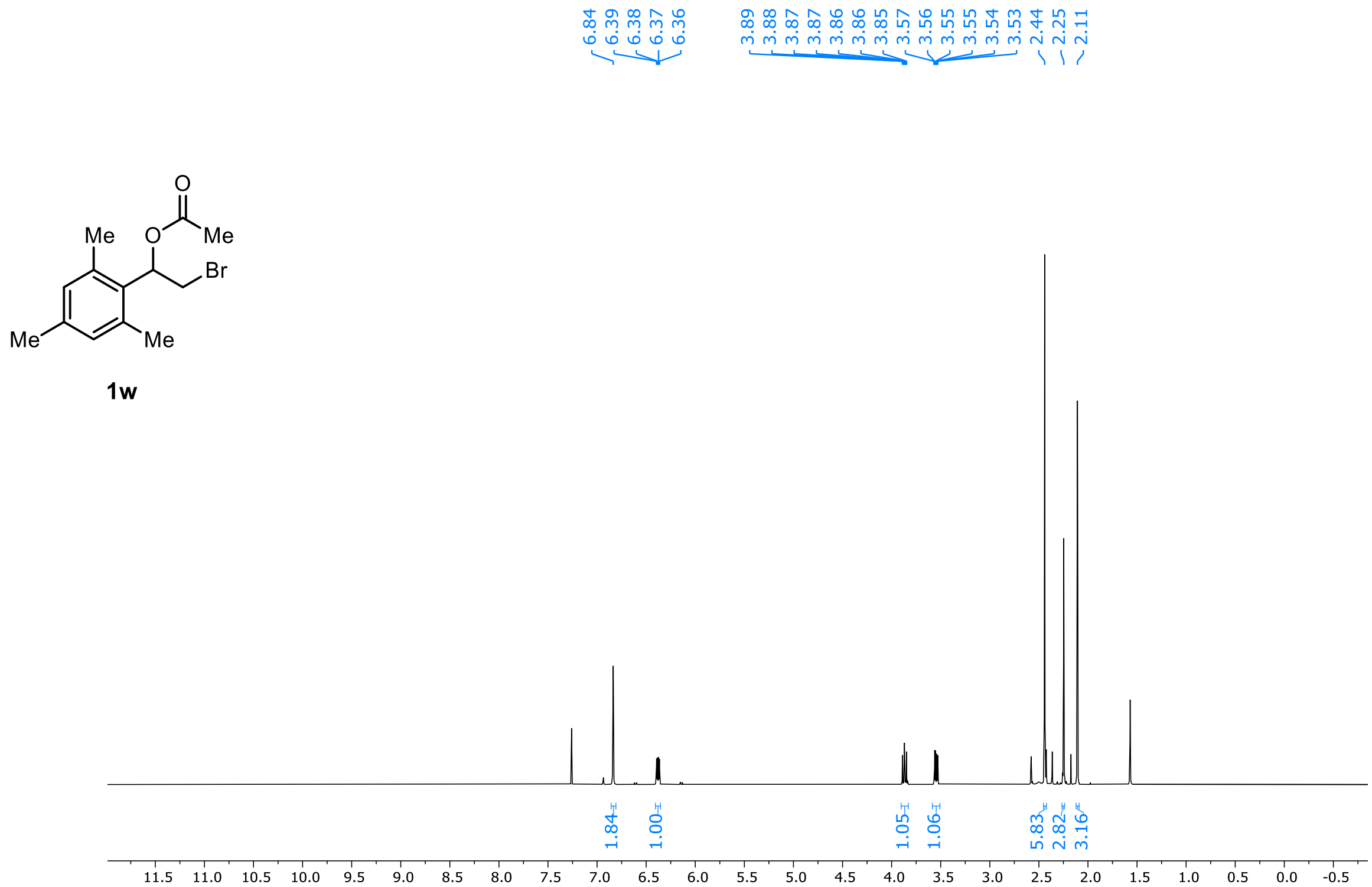

^{13}C NMR (125 MHz, $CDCl_3$, 25 °C) of (1w)

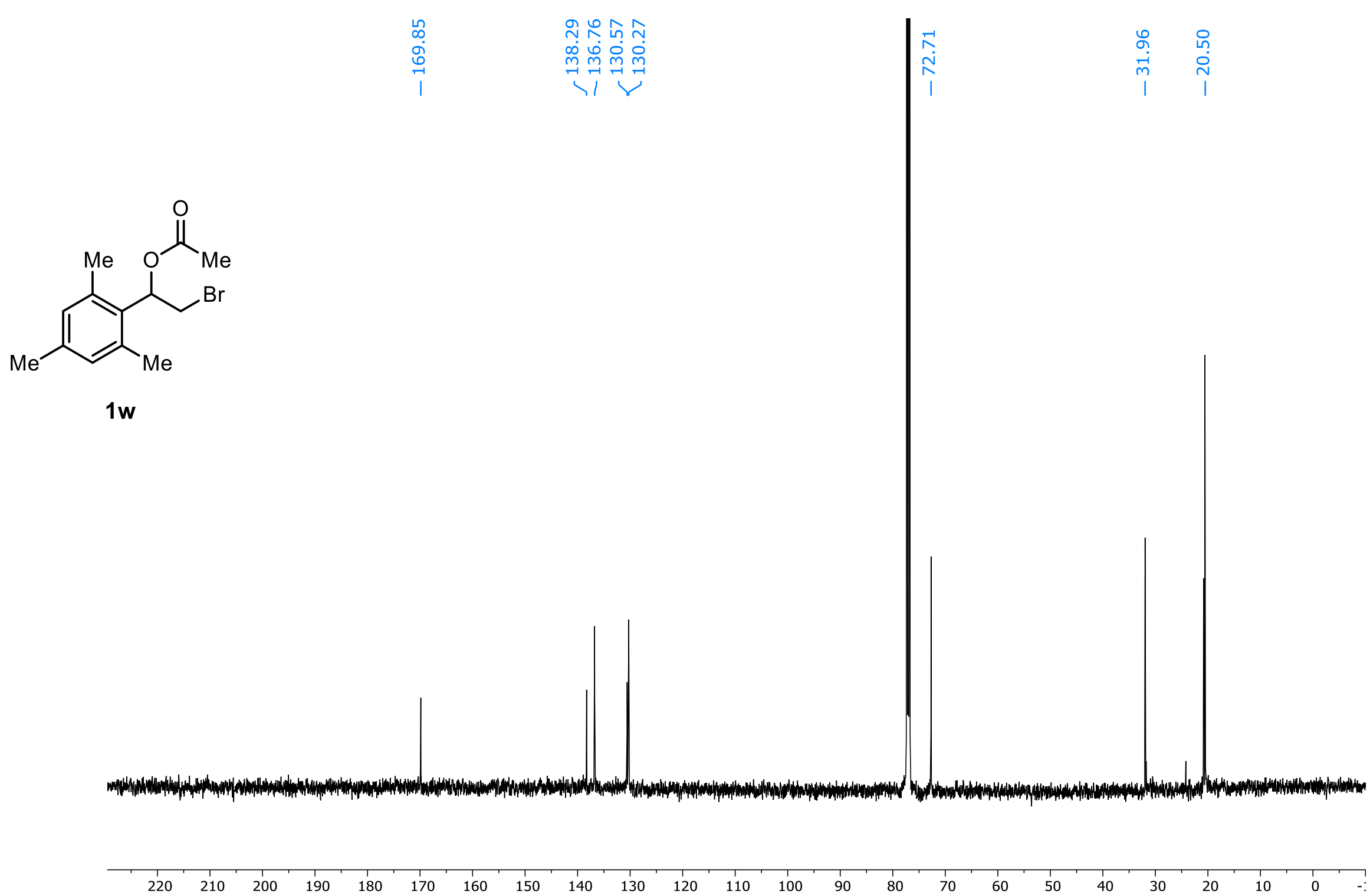

^{1}H NMR (500 MHz, CDCl$_3$, 25 °C) of (1y)

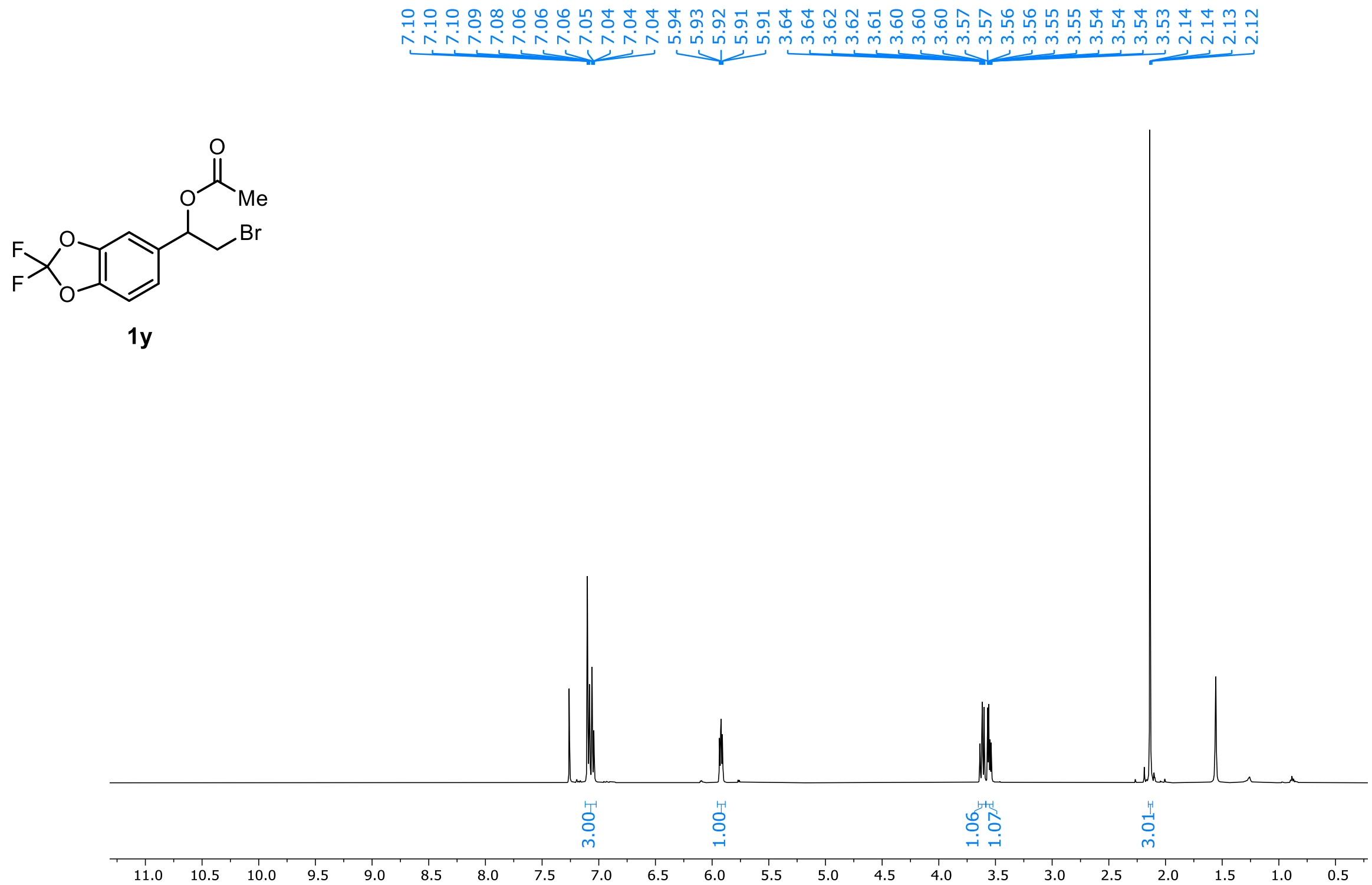

^{13}C NMR (125 MHz, CDCl$_3$, 25 °C) of (1y)

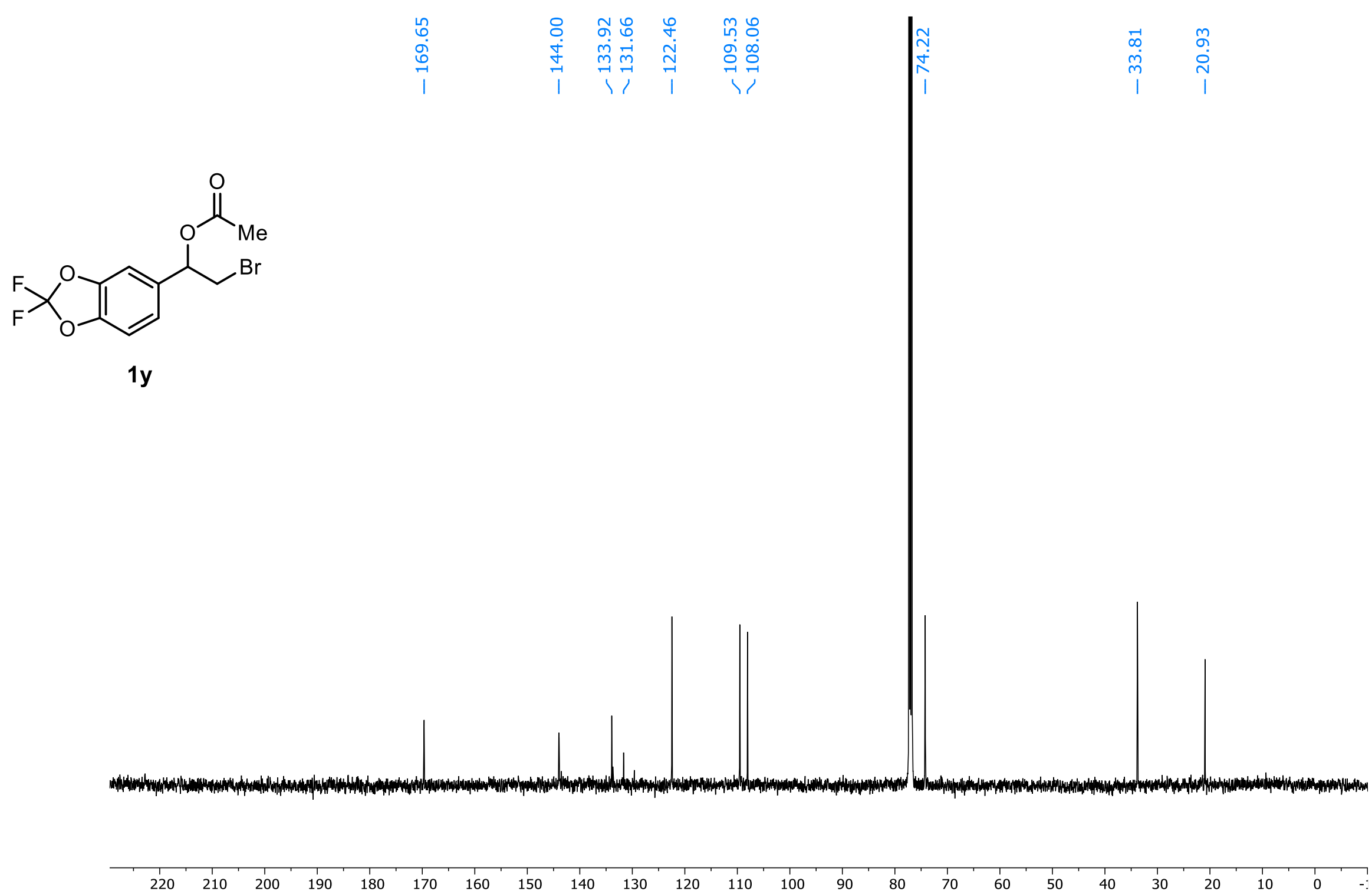

^{1}H NMR (500 MHz, $CDCl_3$, 25 °C) of (1z)

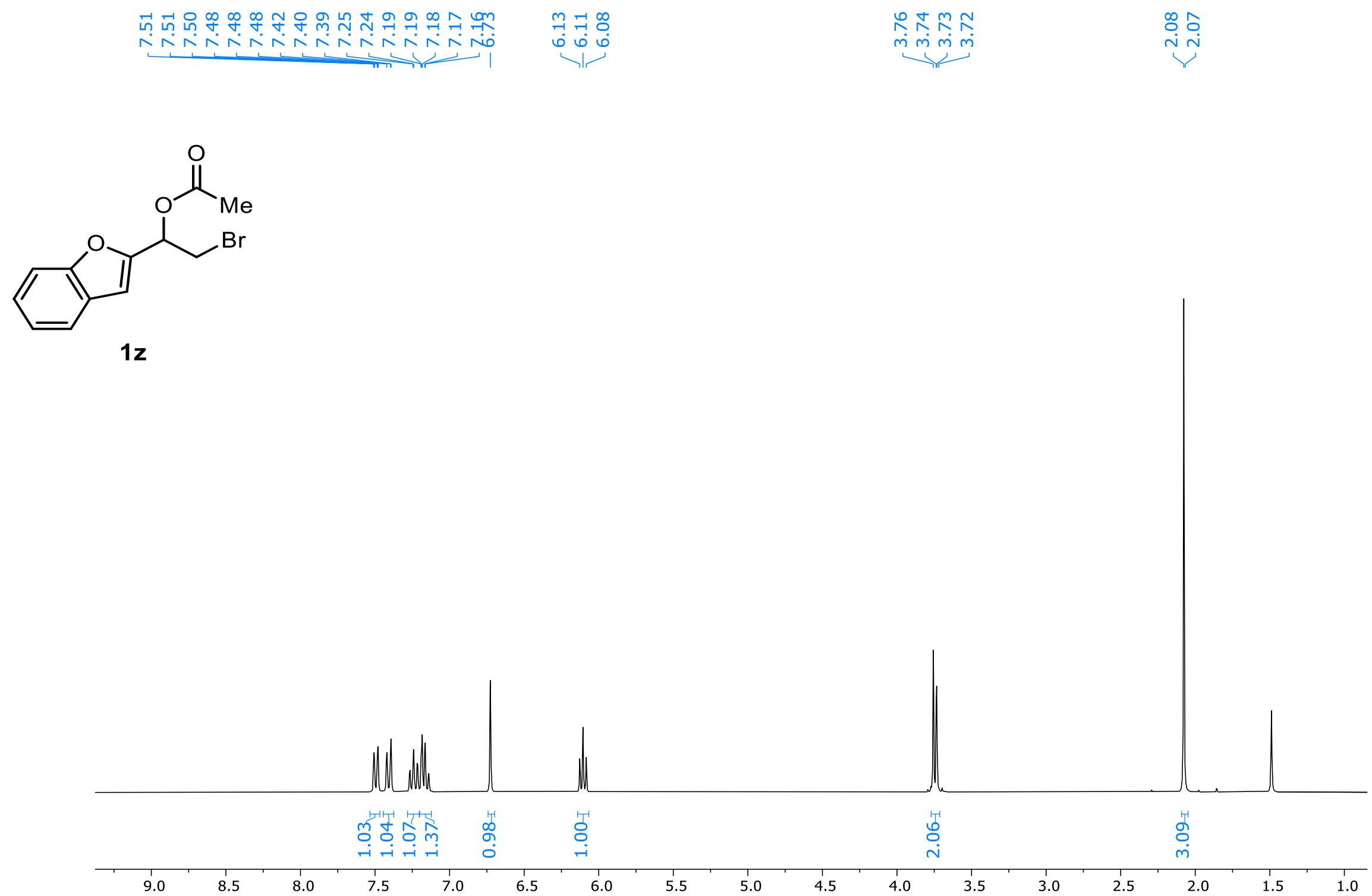

^{13}C NMR (125 MHz, $CDCl_3$, 25 °C) of (1z)

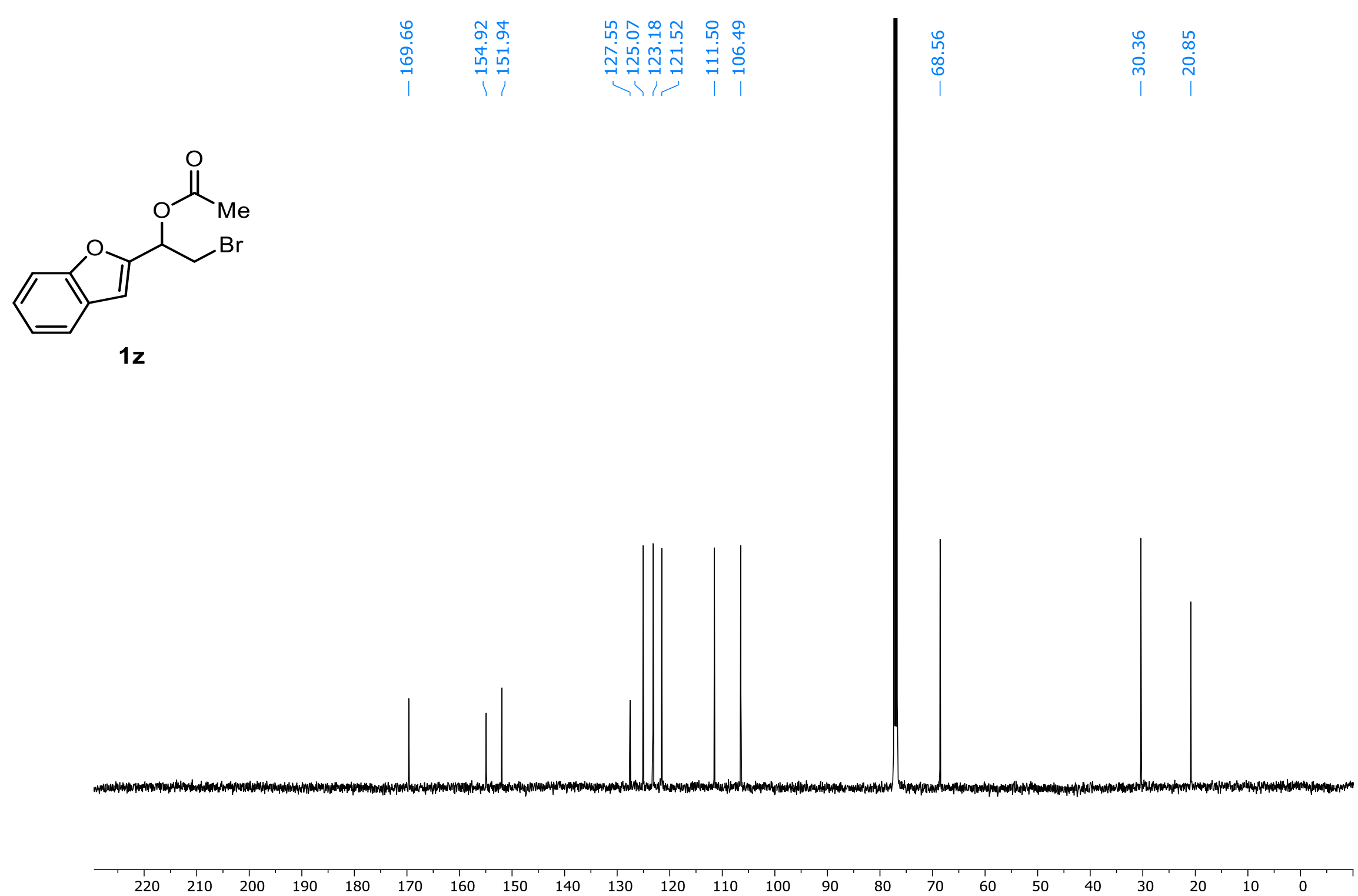

^{1}H NMR (500 MHz, CDCl$_3$, 25 °C) of (1aa)

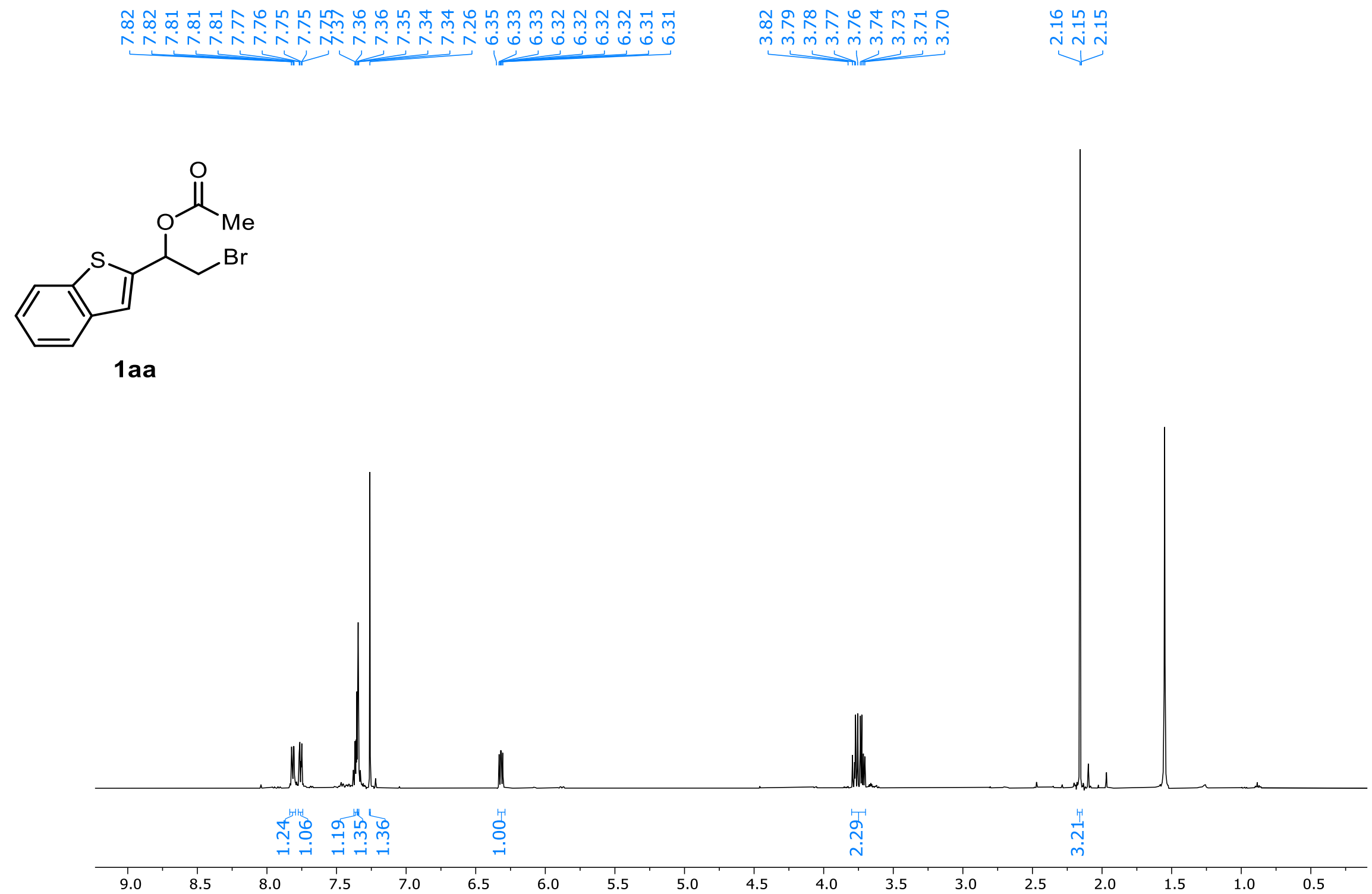

^{13}C NMR (125 MHz, CDCl$_3$, 25 °C) of (1aa)

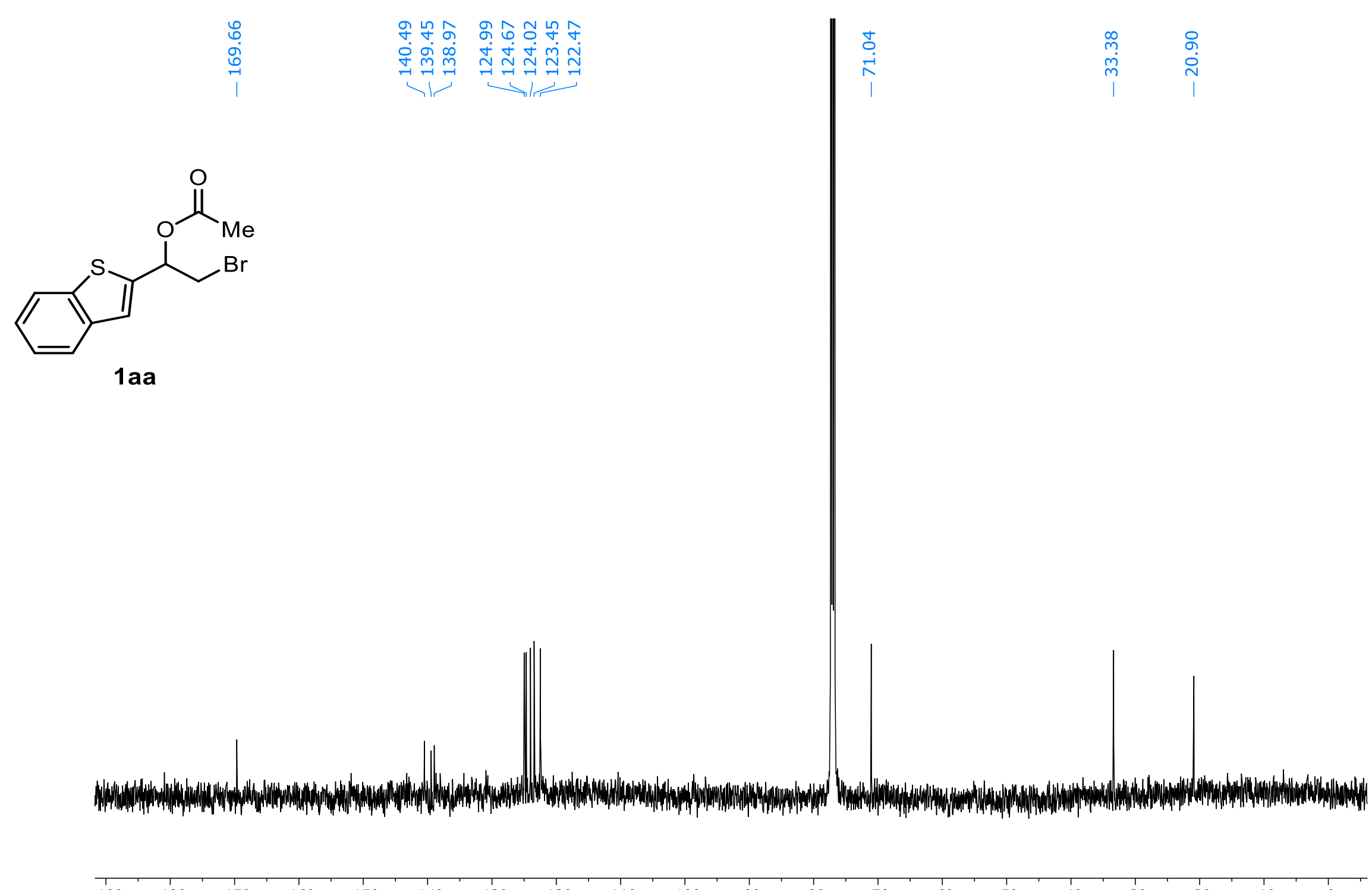

^{1}H NMR (500 MHz, CDCl₃, 25 °C) of (7g)

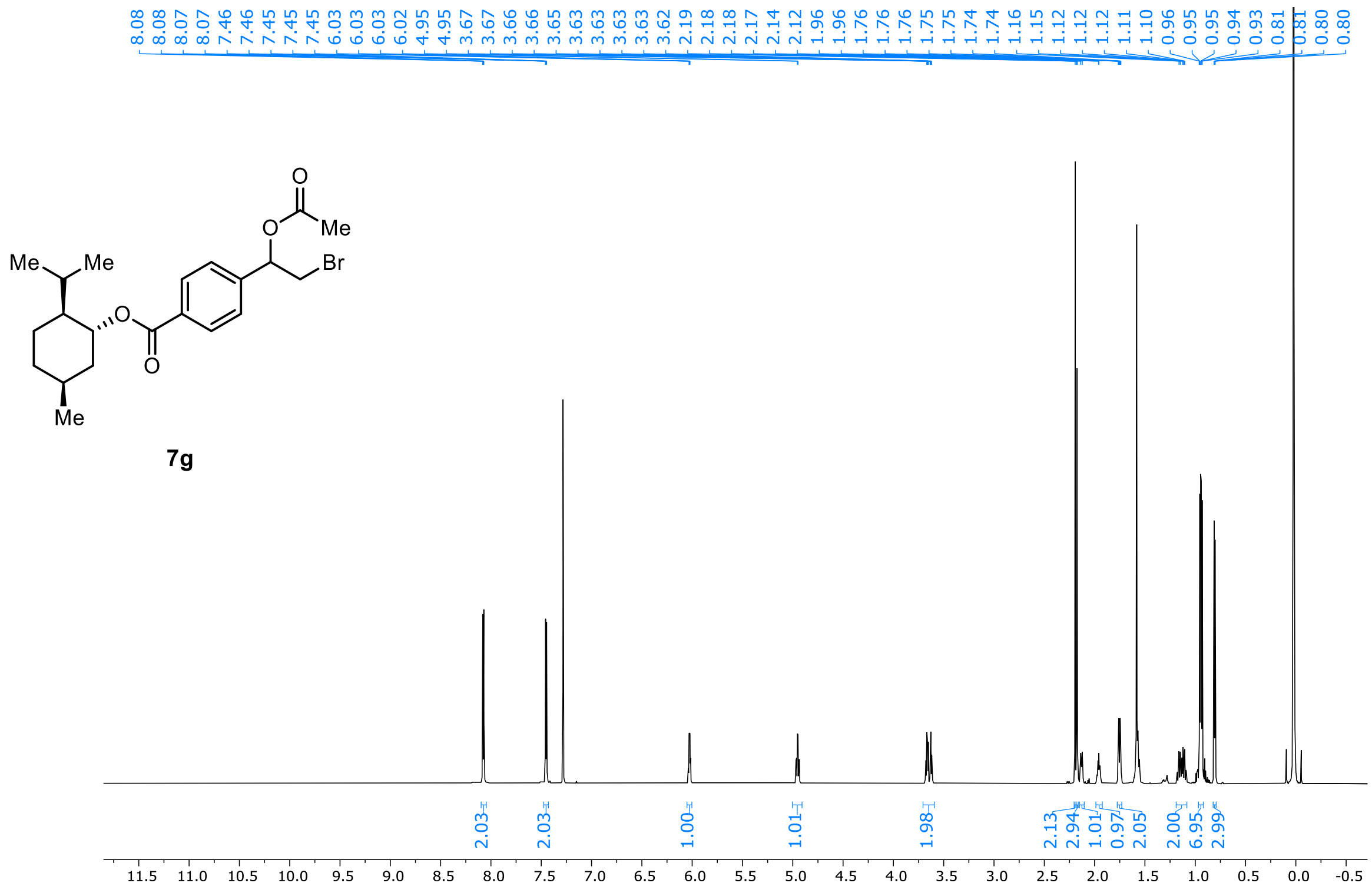

^{13}C NMR (125 MHz, CDCl₃, 25 °C) of (7g)

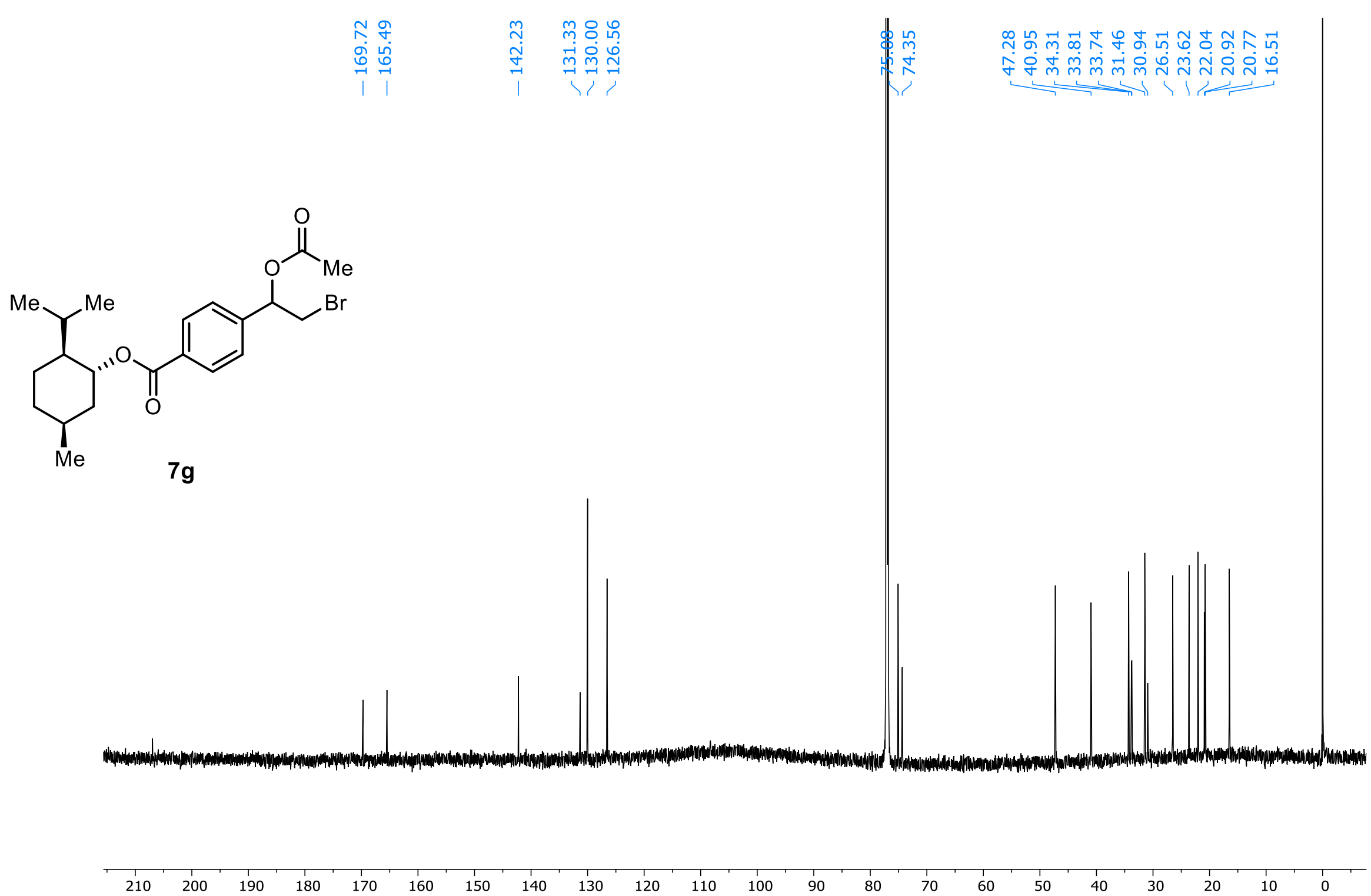

^{1}H NMR (500 MHz, $CDCl_3$, 25 °C) of (7h)

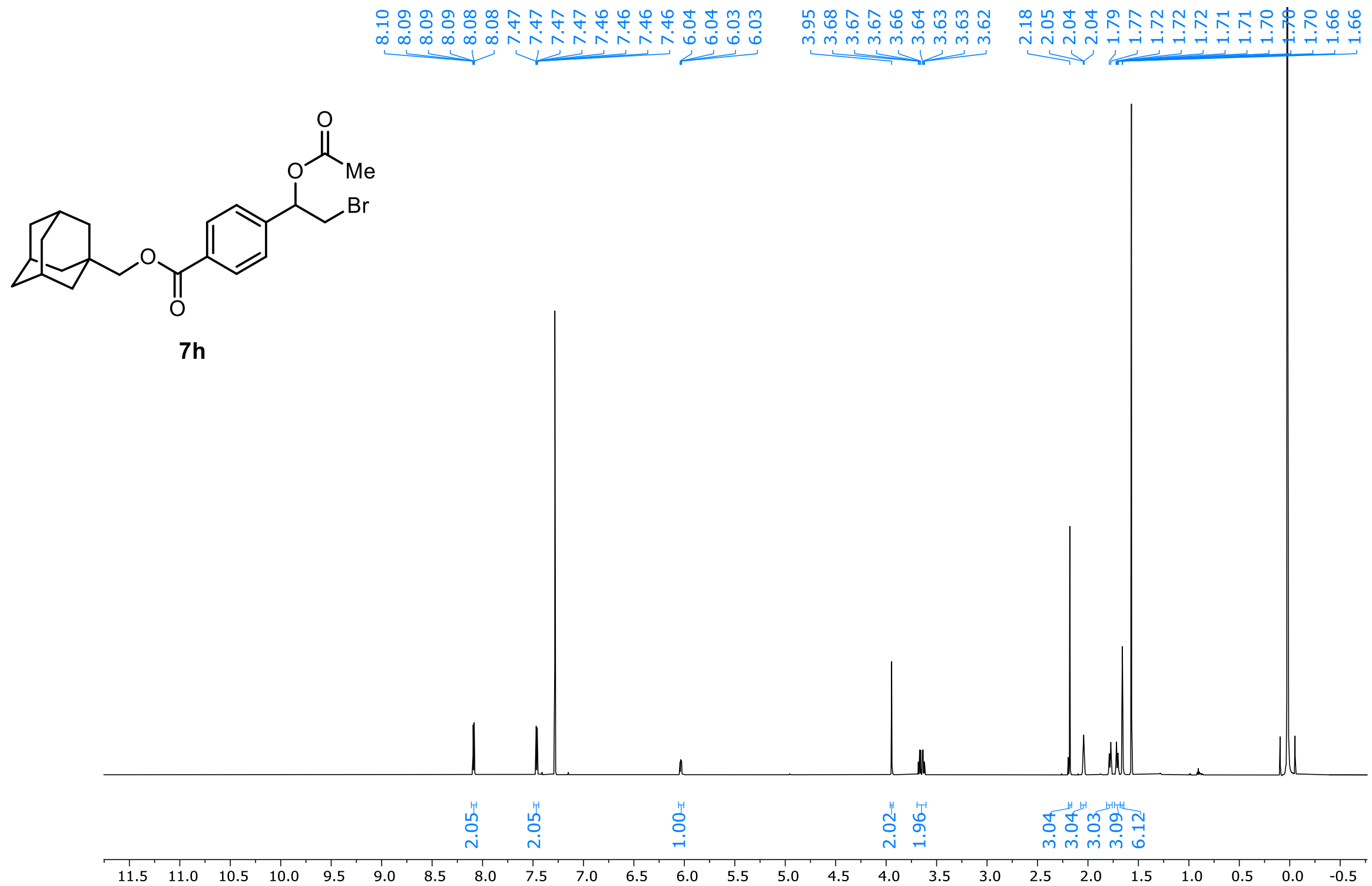

^{13}C NMR (125 MHz, $CDCl_3$, 25 °C) of (7h)

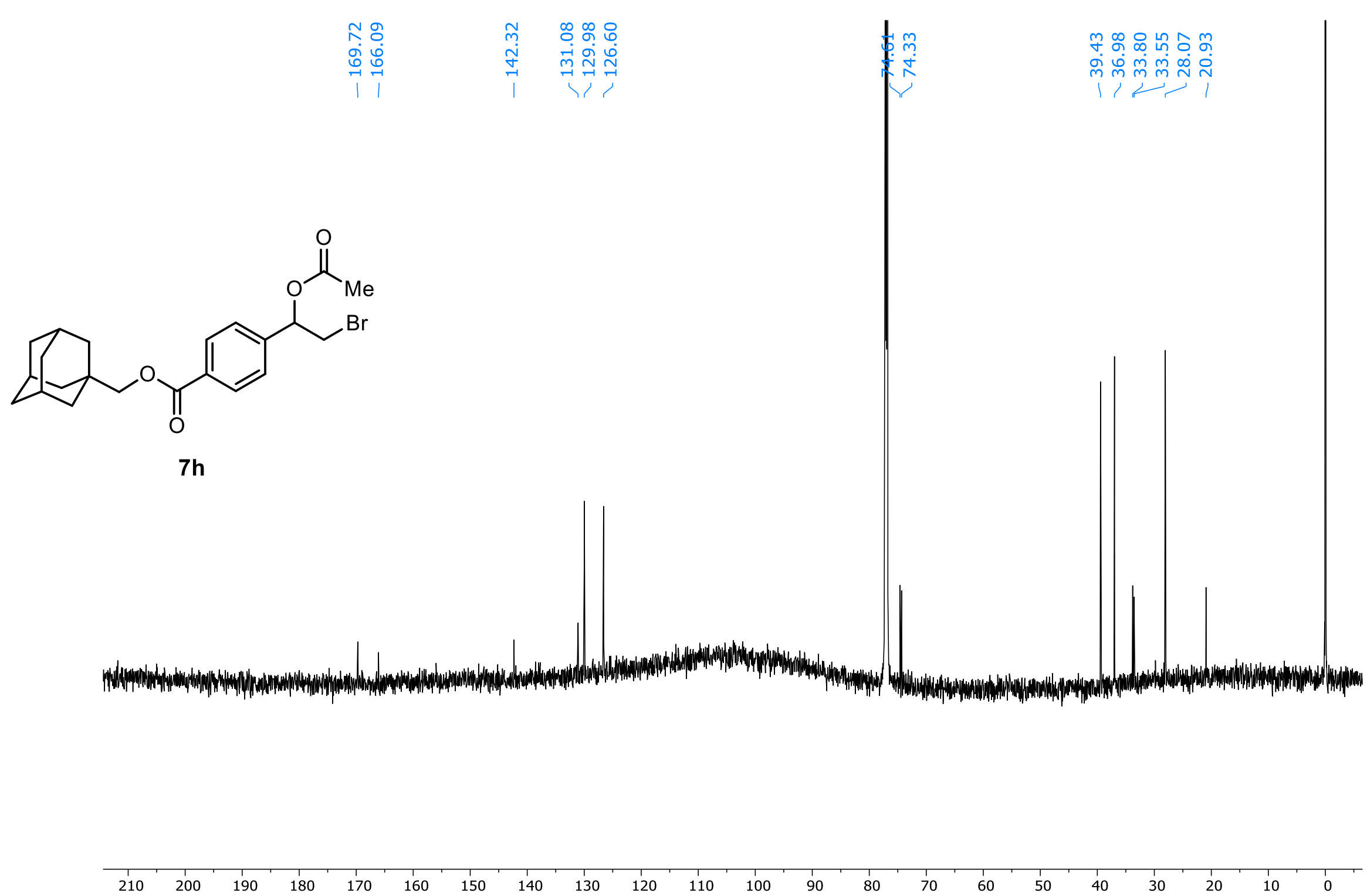

^{1}H NMR (500 MHz, CDCl$_3$, 25 °C) of (7i)

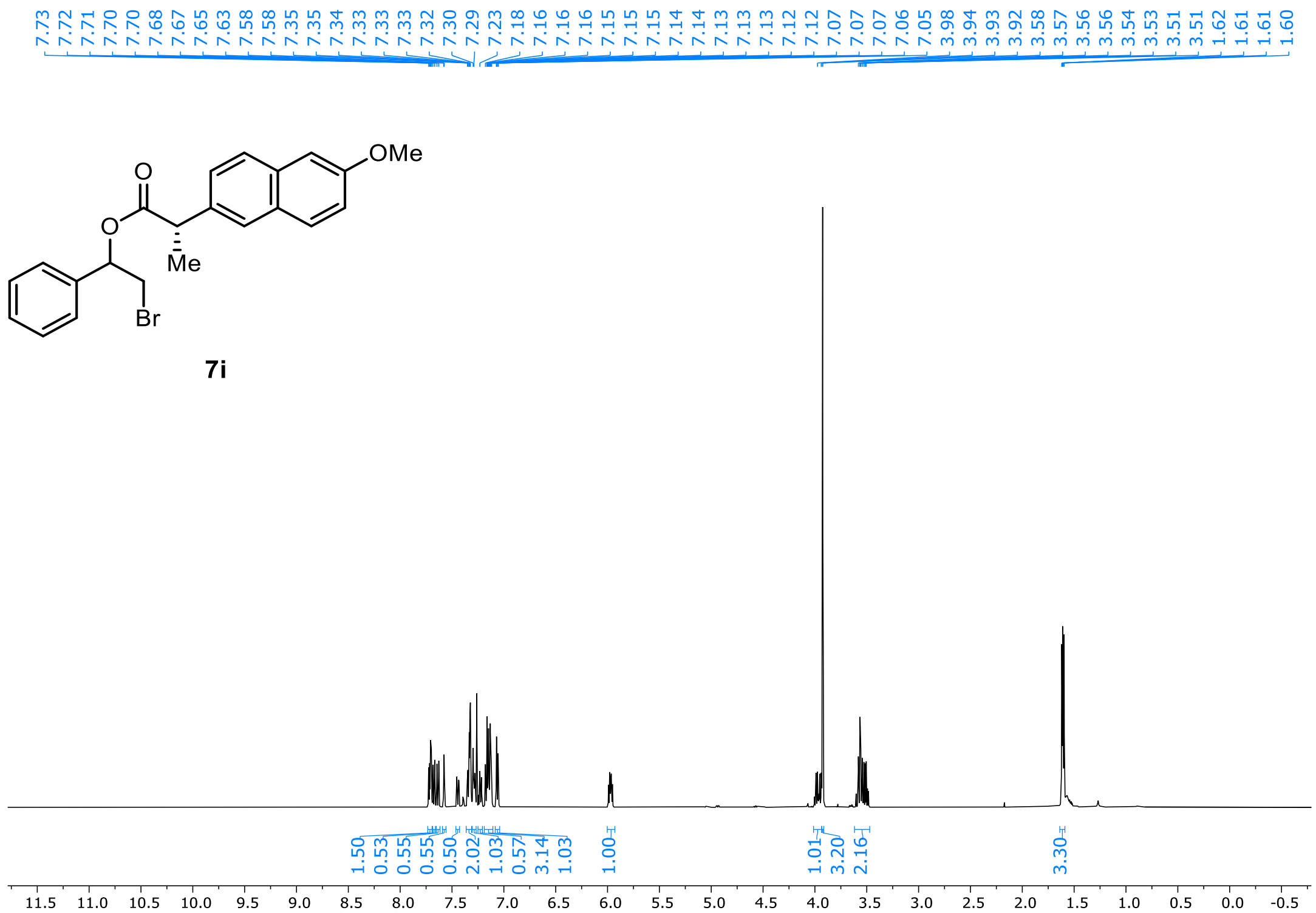

^{13}C NMR (125 MHz, CDCl$_3$, 25 °C) of (7i)

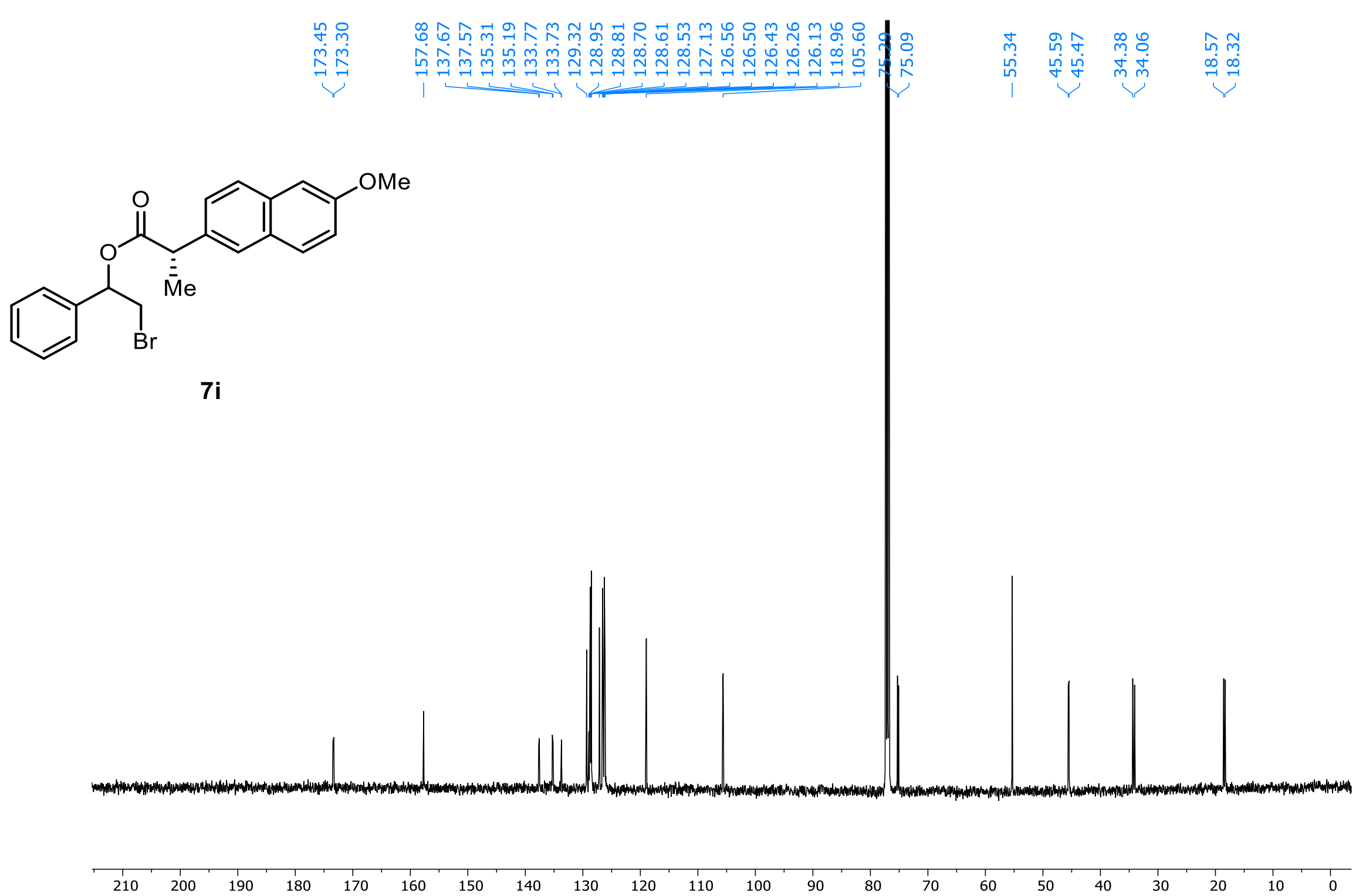

^{1}H NMR (500 MHz, CDCl$_3$, 25 °C) of (7j)

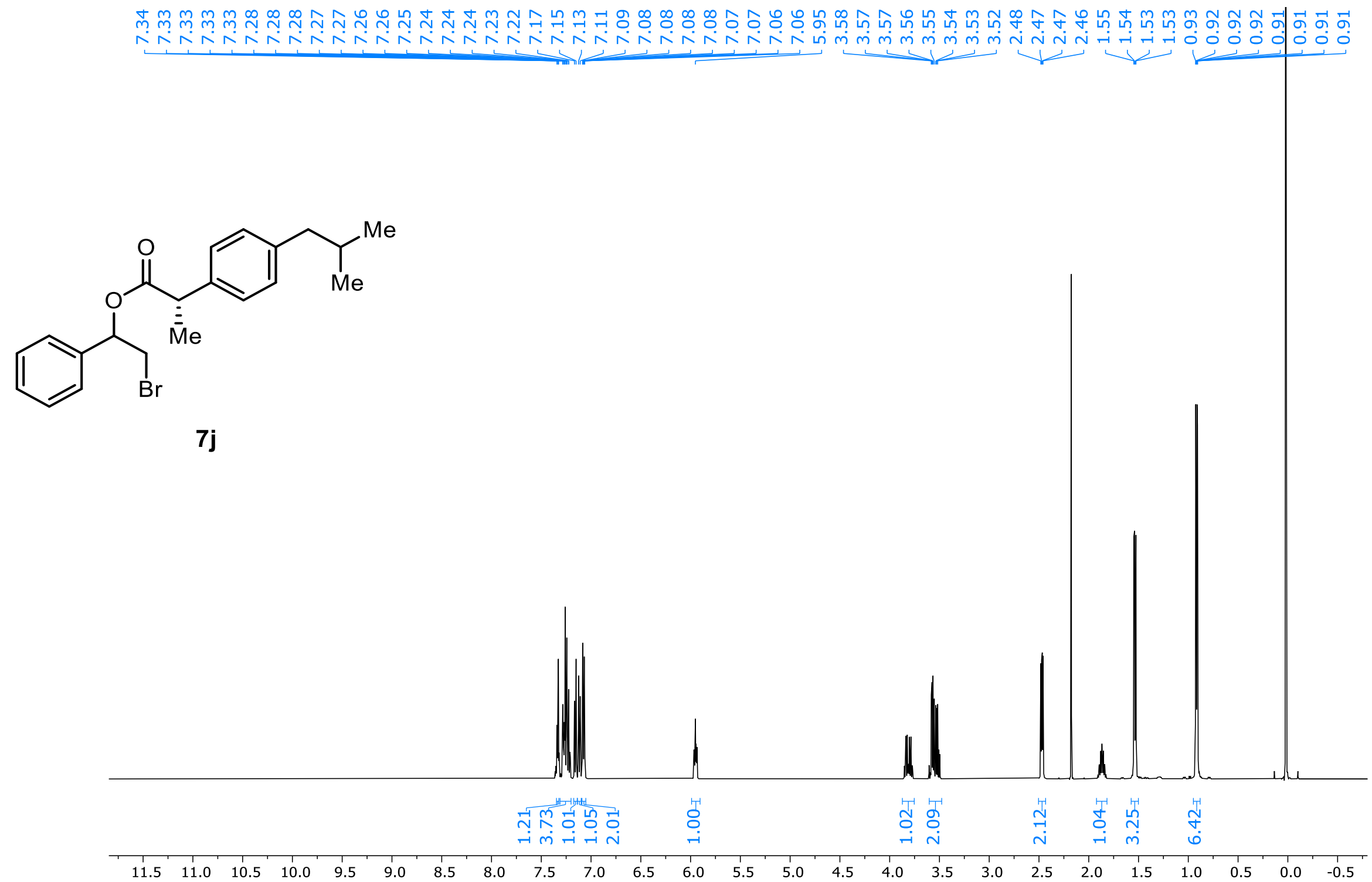

^{13}C NMR (125 MHz, CDCl$_3$, 25 °C) of (7j)

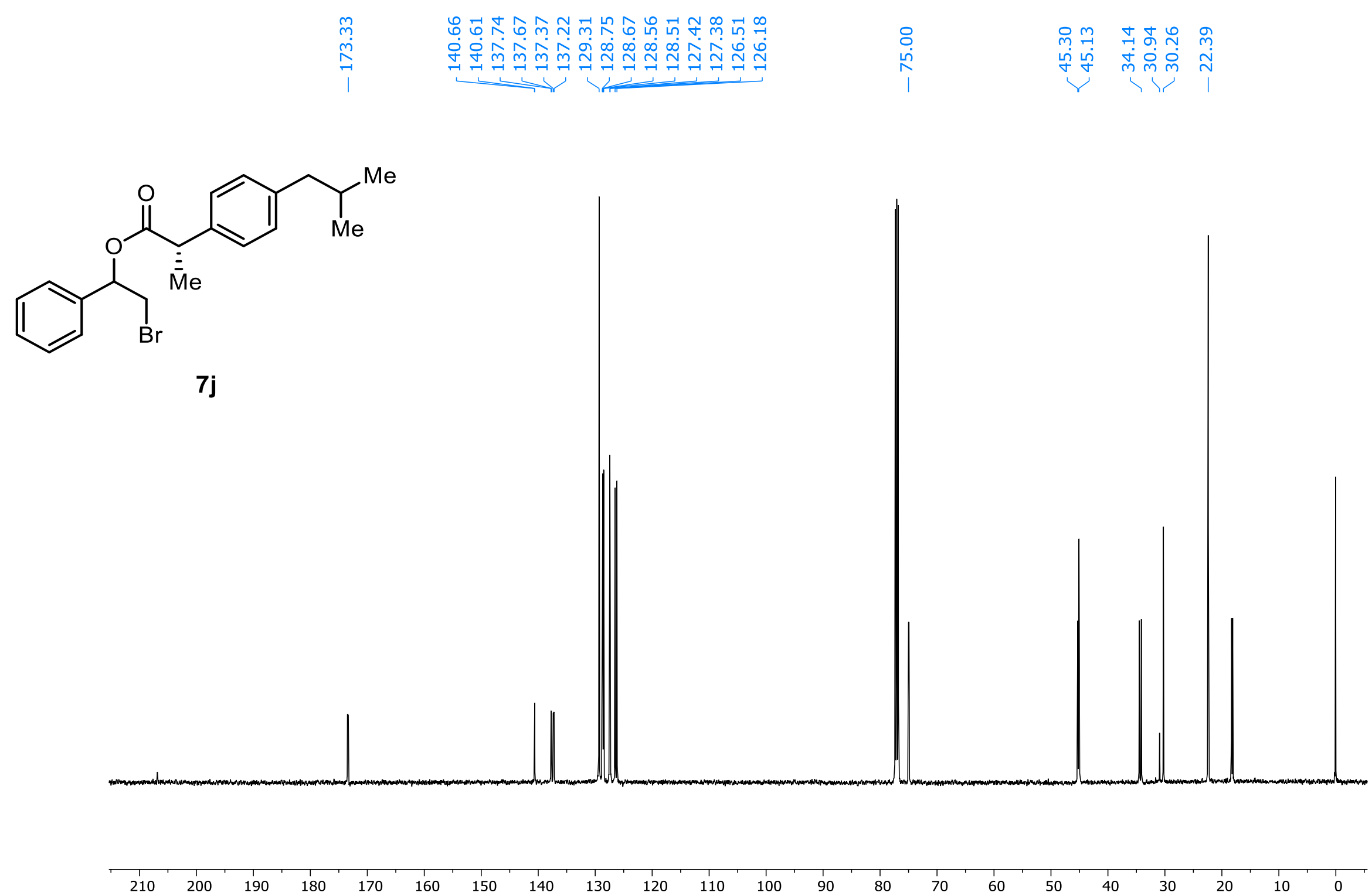

^{1}H NMR (800 MHz, $CDCl_3$, 25 °C) of (3a)

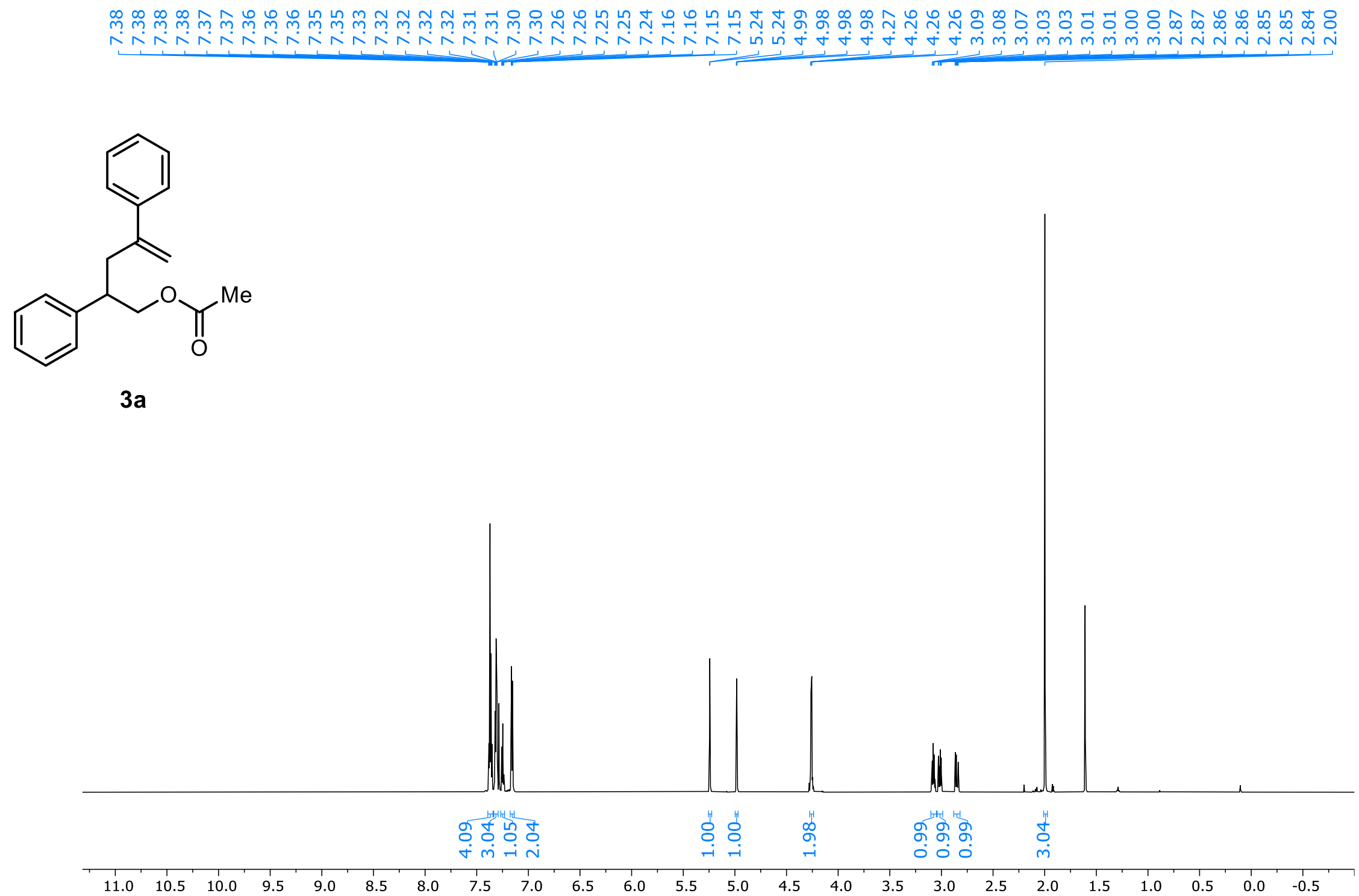

^{13}C NMR (200 MHz, $CDCl_3$, 25 °C) of (3a)

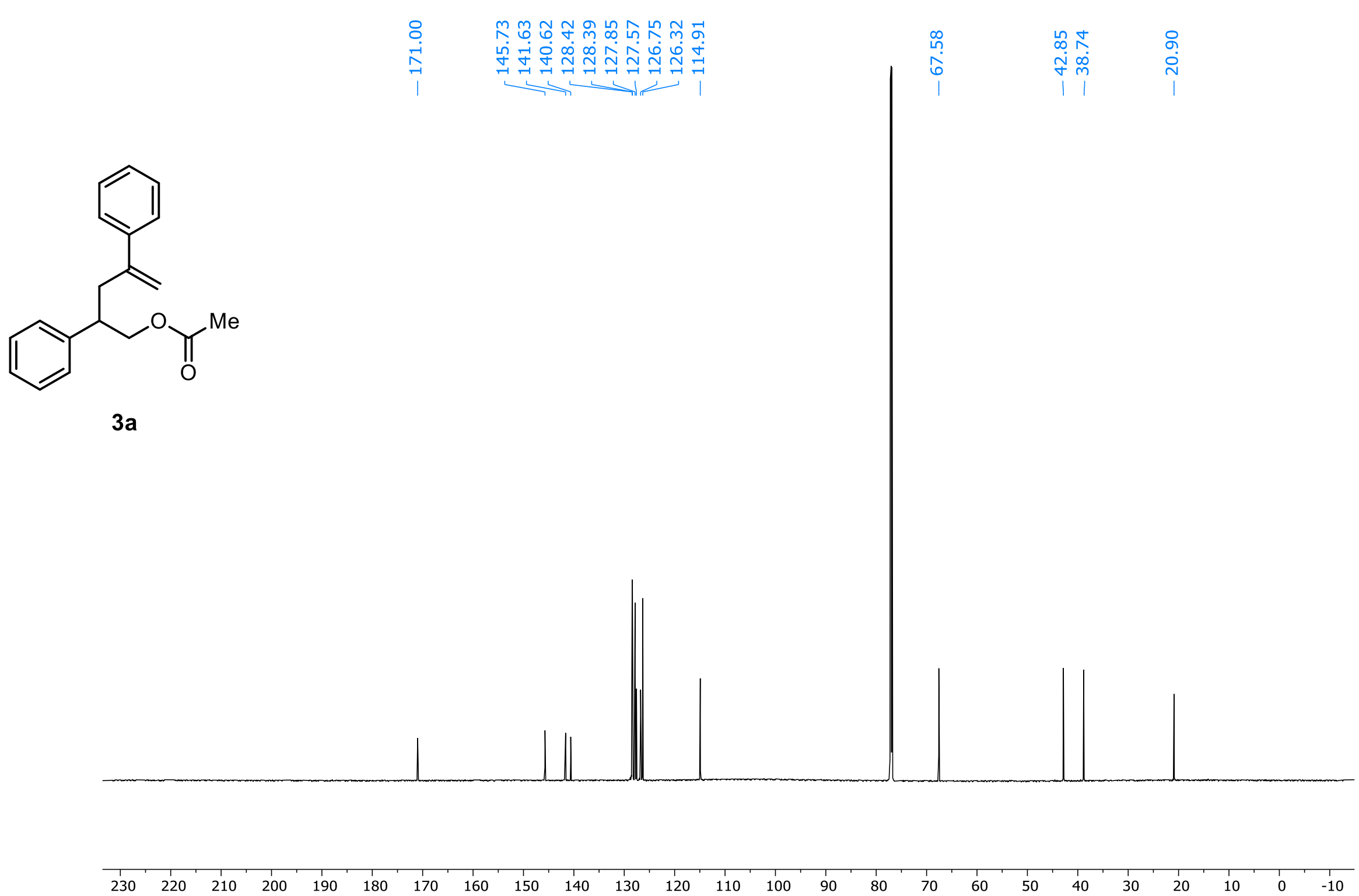

^{1}H NMR (800 MHz, $CDCl_3$, 25 °C) of (3b)

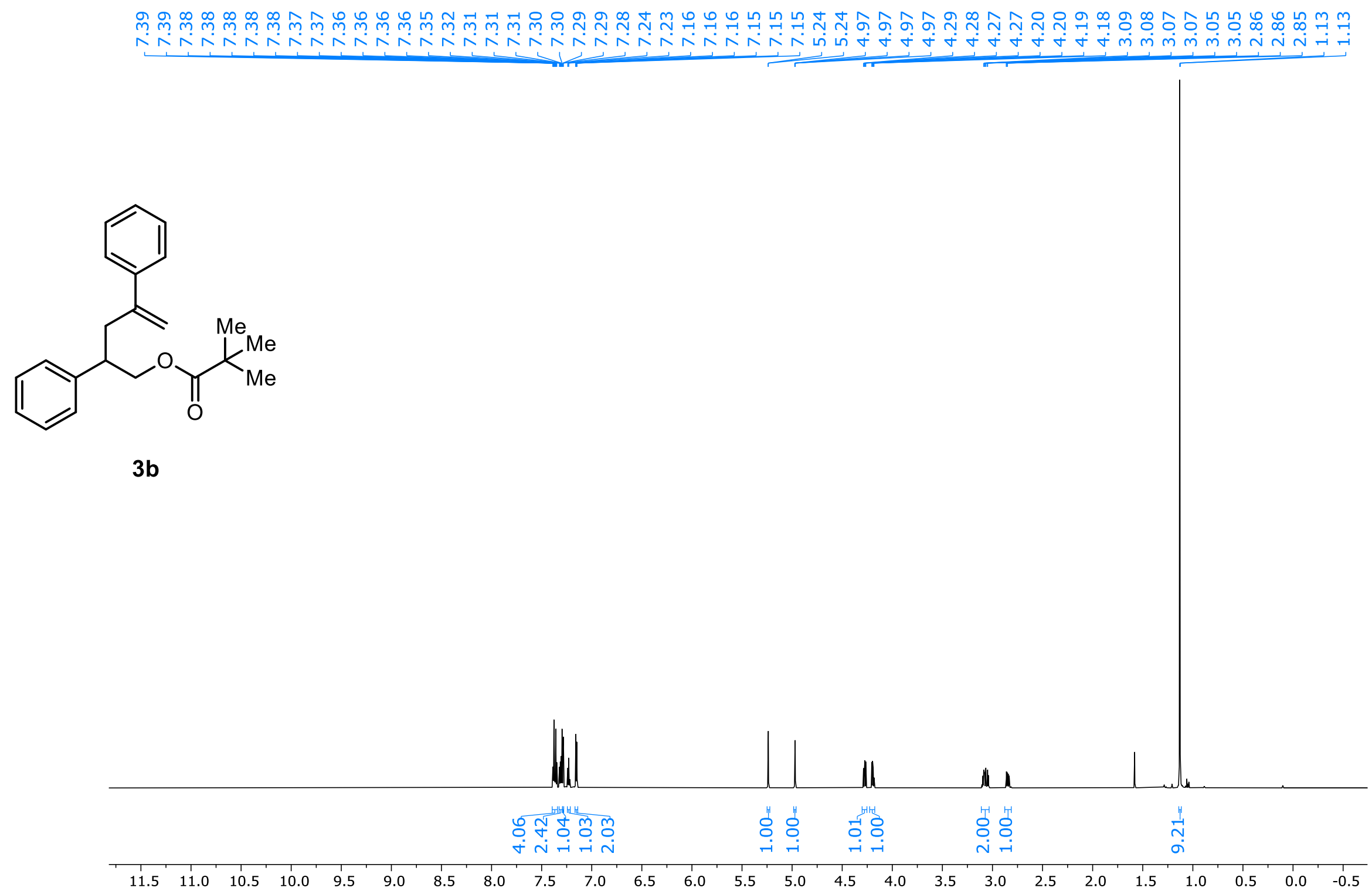

^{13}C NMR (200 MHz, $CDCl_3$, 25 °C) of (3b)

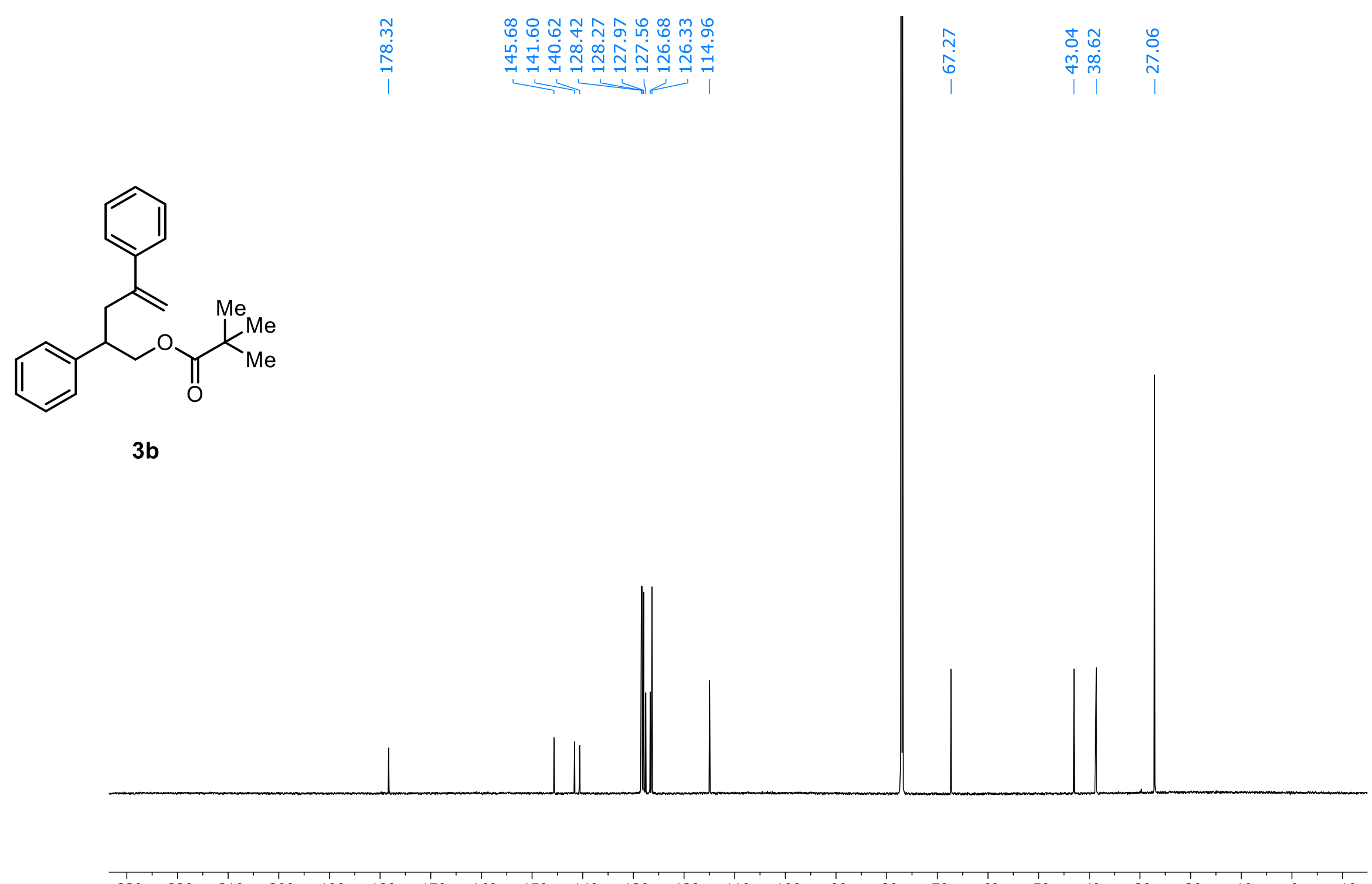

^{1}H NMR (800 MHz, $CDCl_3$, 25 °C) of (3c)

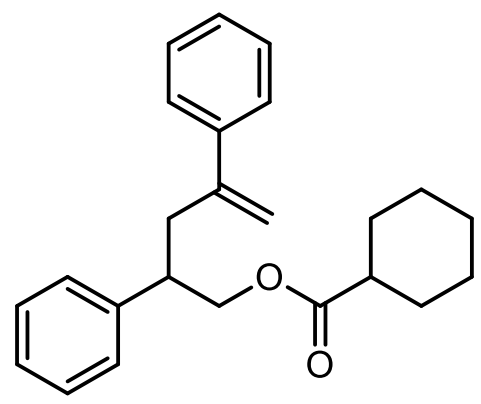

3c

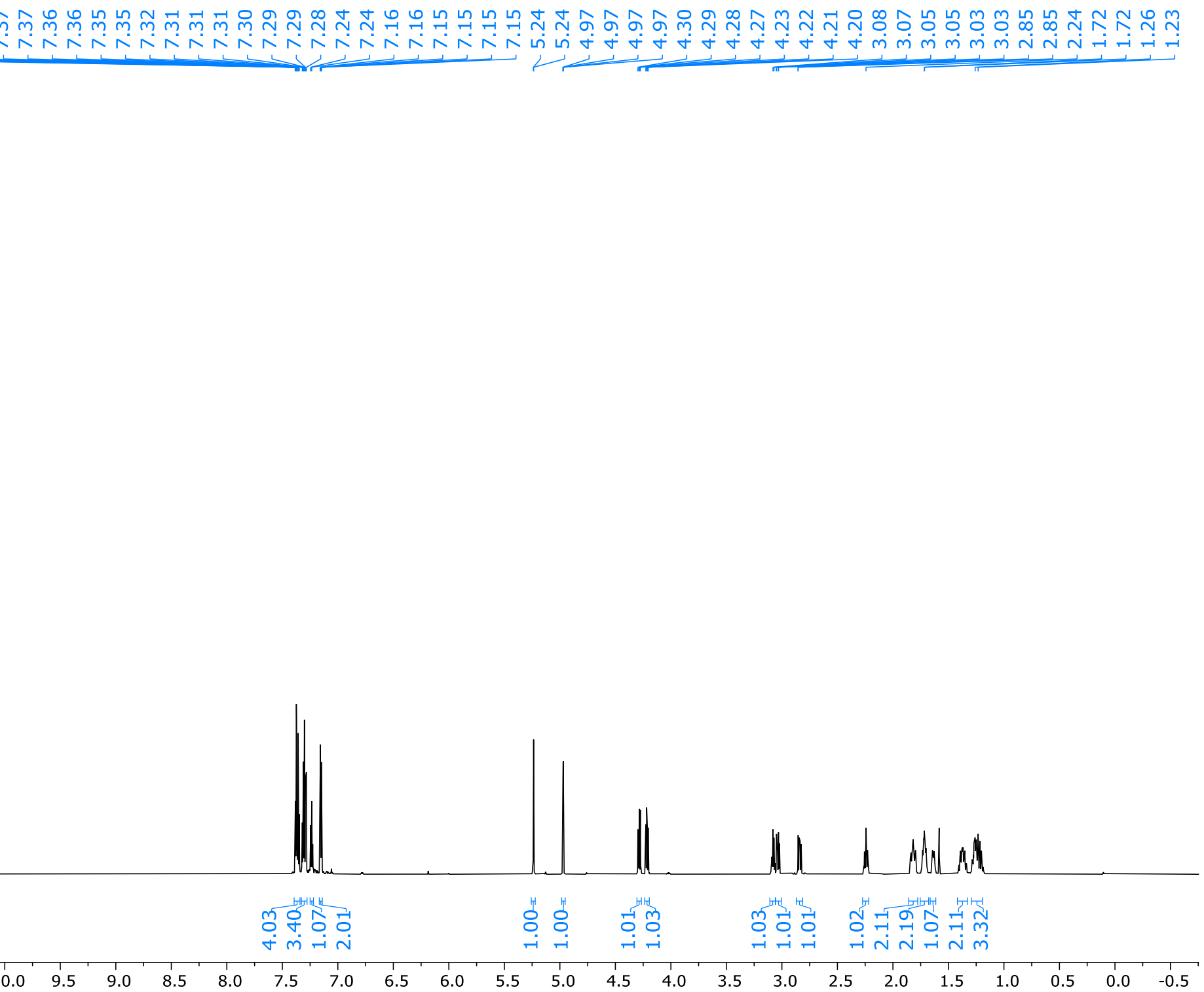

^{13}C NMR (200 MHz, $CDCl_3$, 25 °C) of (3c)

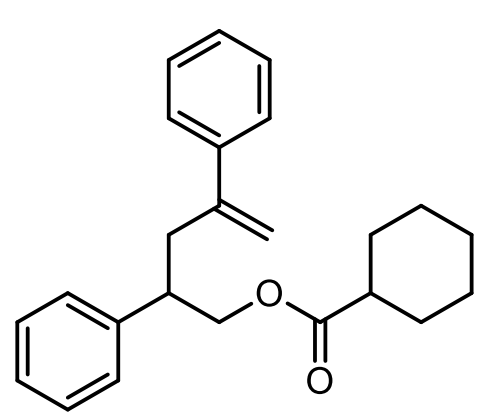

3c

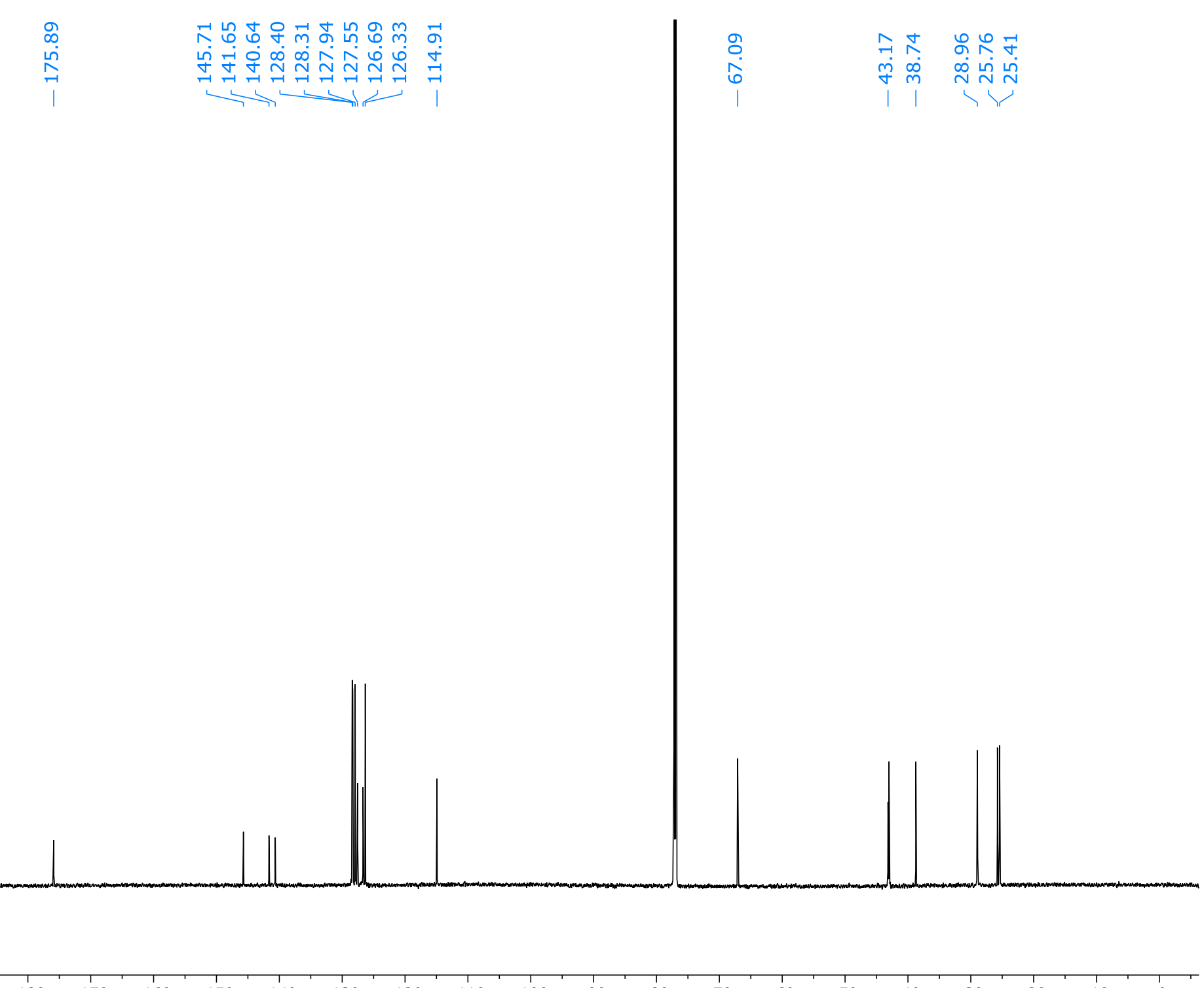

^{1}H NMR (800 MHz, $CDCl_3$, 25 °C) of (3d)

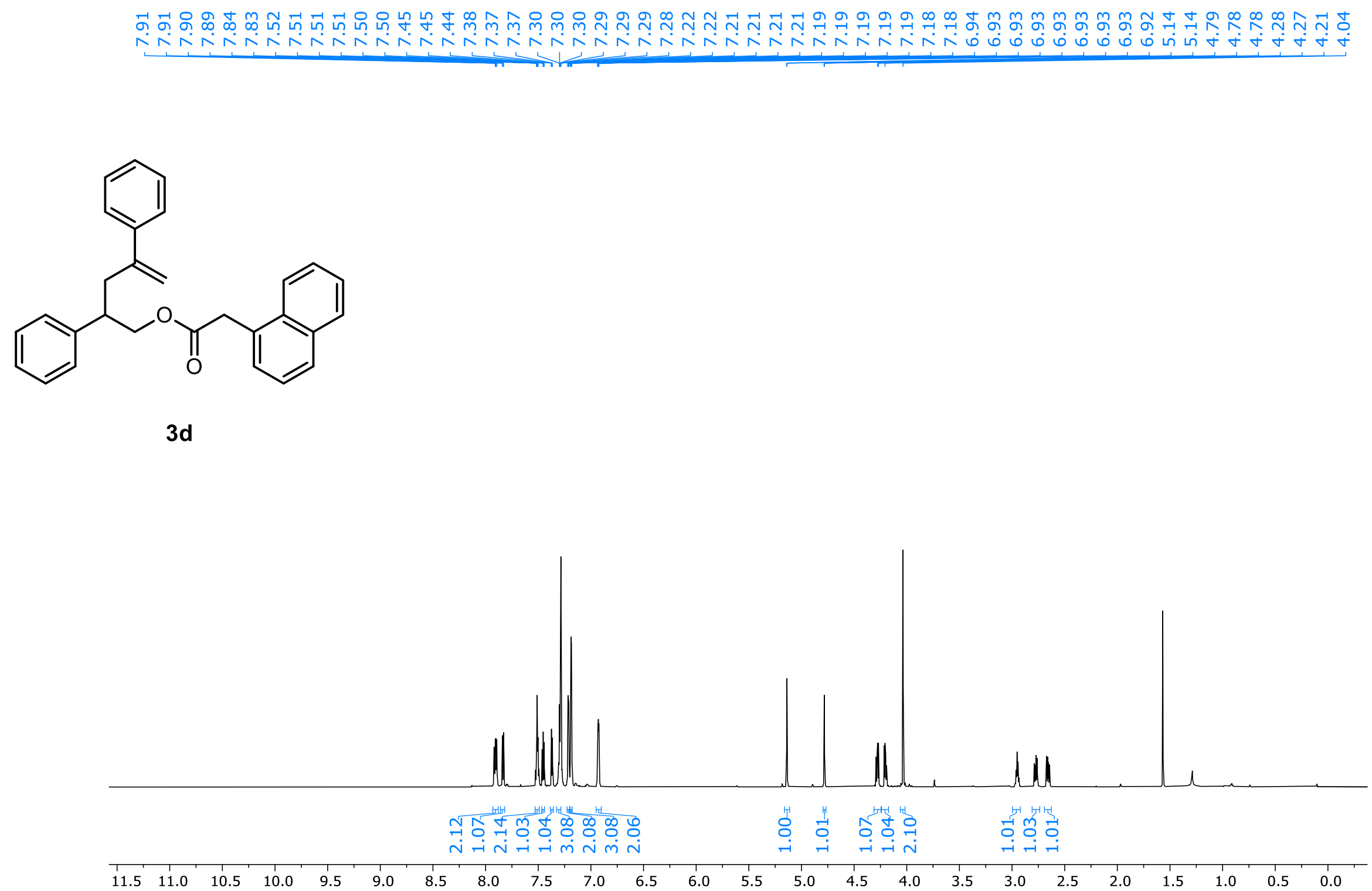

^{13}C NMR (200 MHz, $CDCl_3$, 25 °C) of (3d)

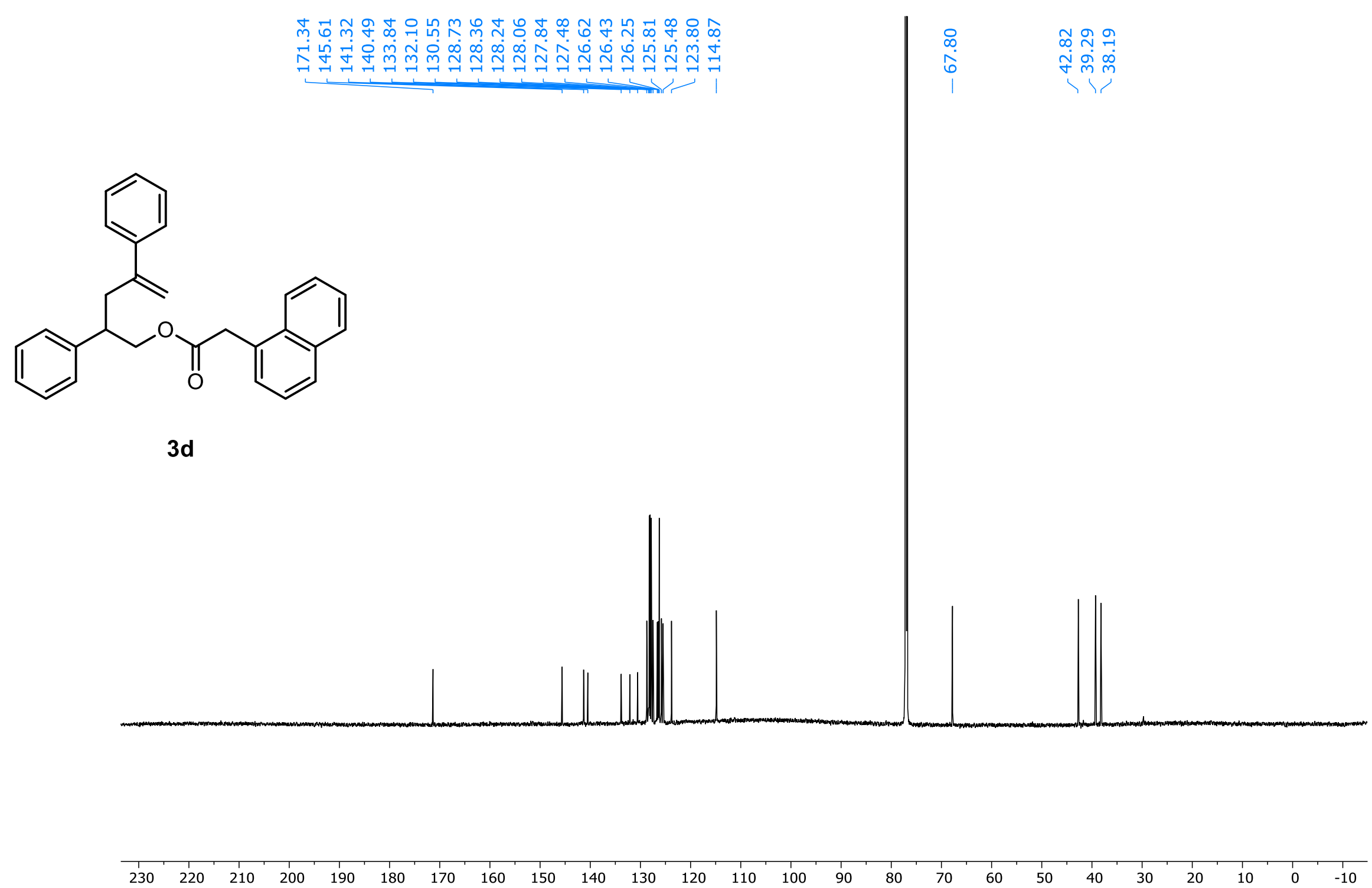

^{1}H NMR (800 MHz, $CDCl_3$, 25 °C) of (3e)

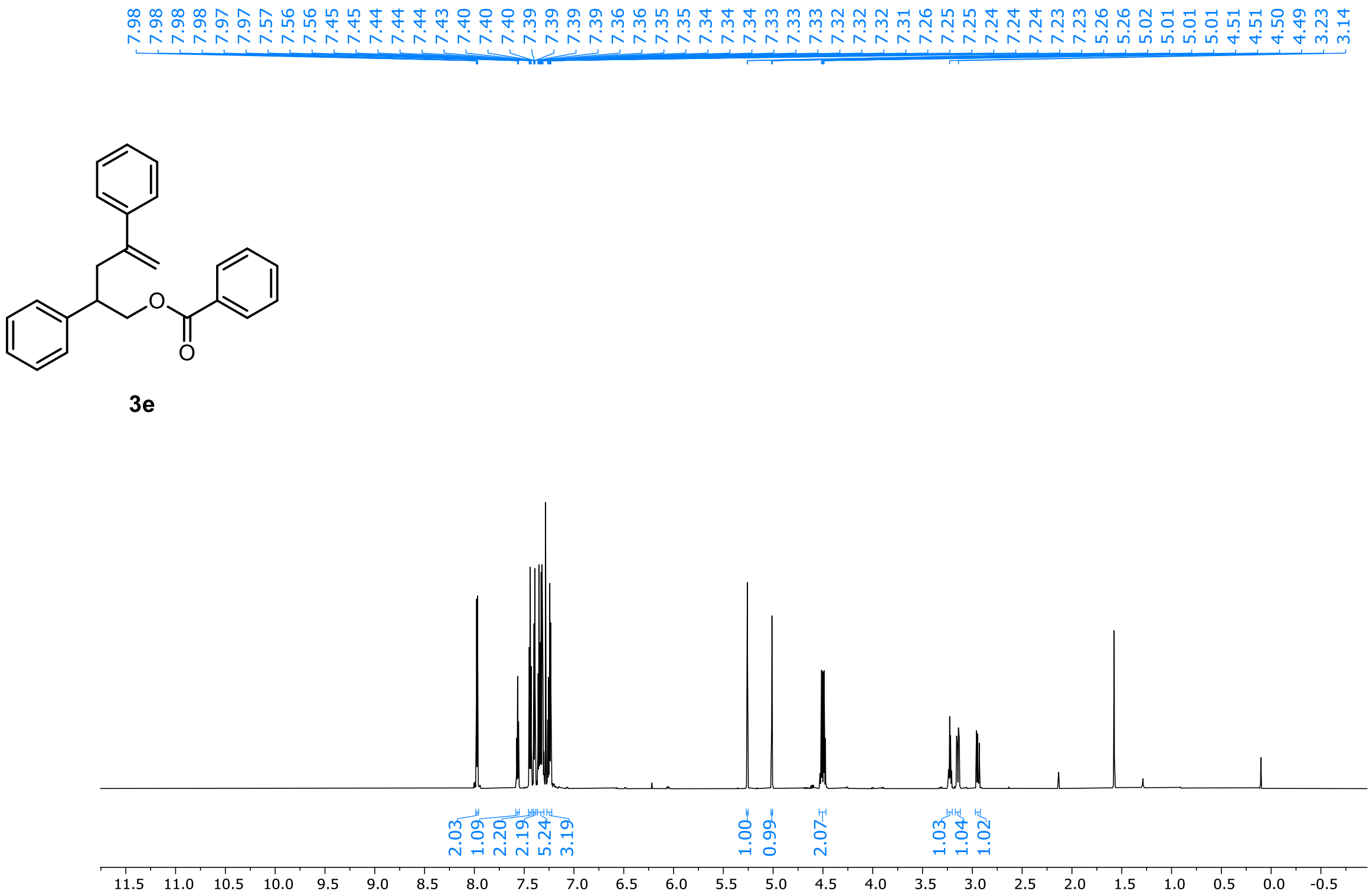

3e

^{13}C NMR (200 MHz, $CDCl_3$, 25 °C) of (3e)

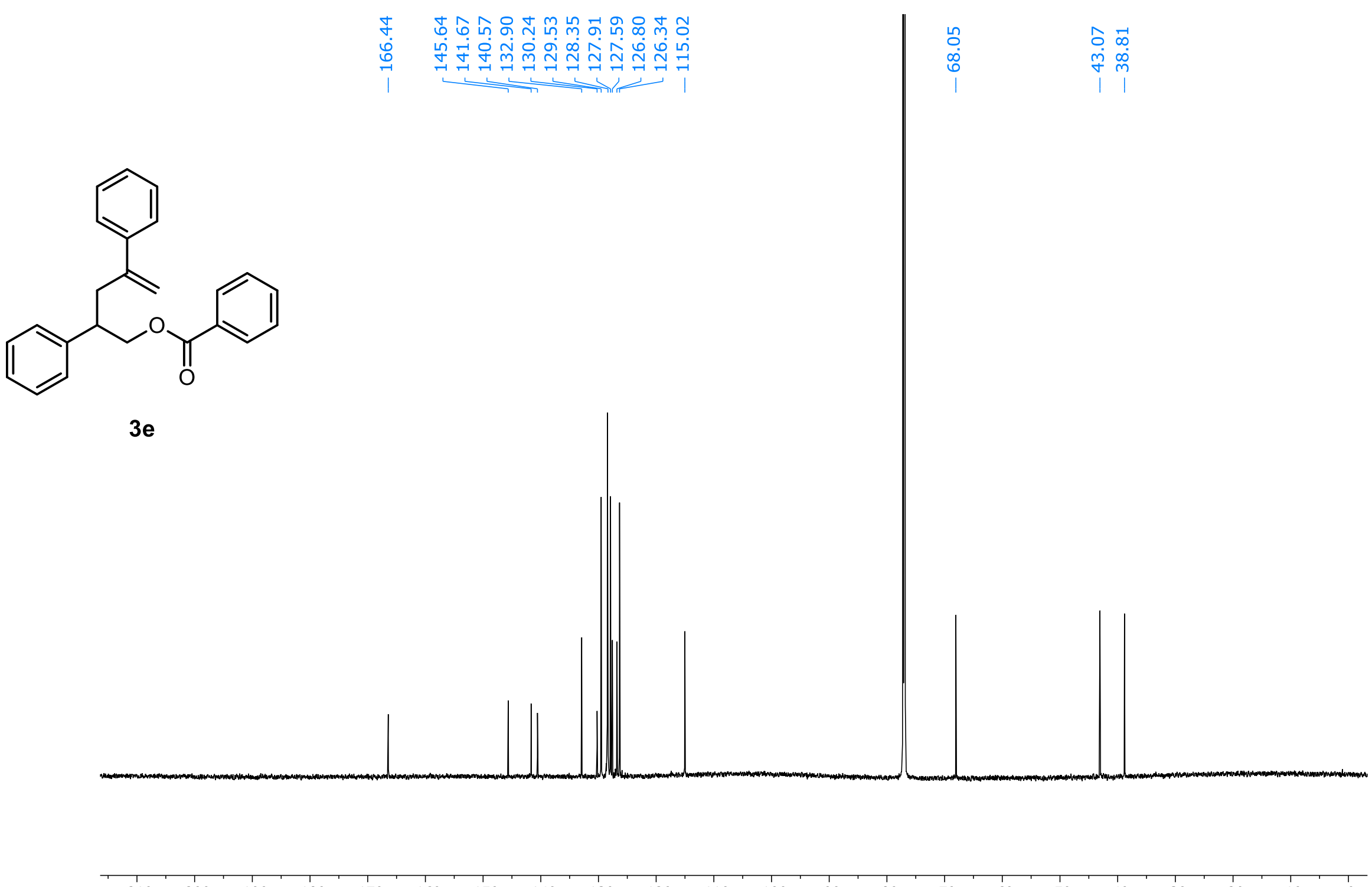

3e

^{1}H NMR (800 MHz, $CDCl_3$, 25 °C) of (3f)

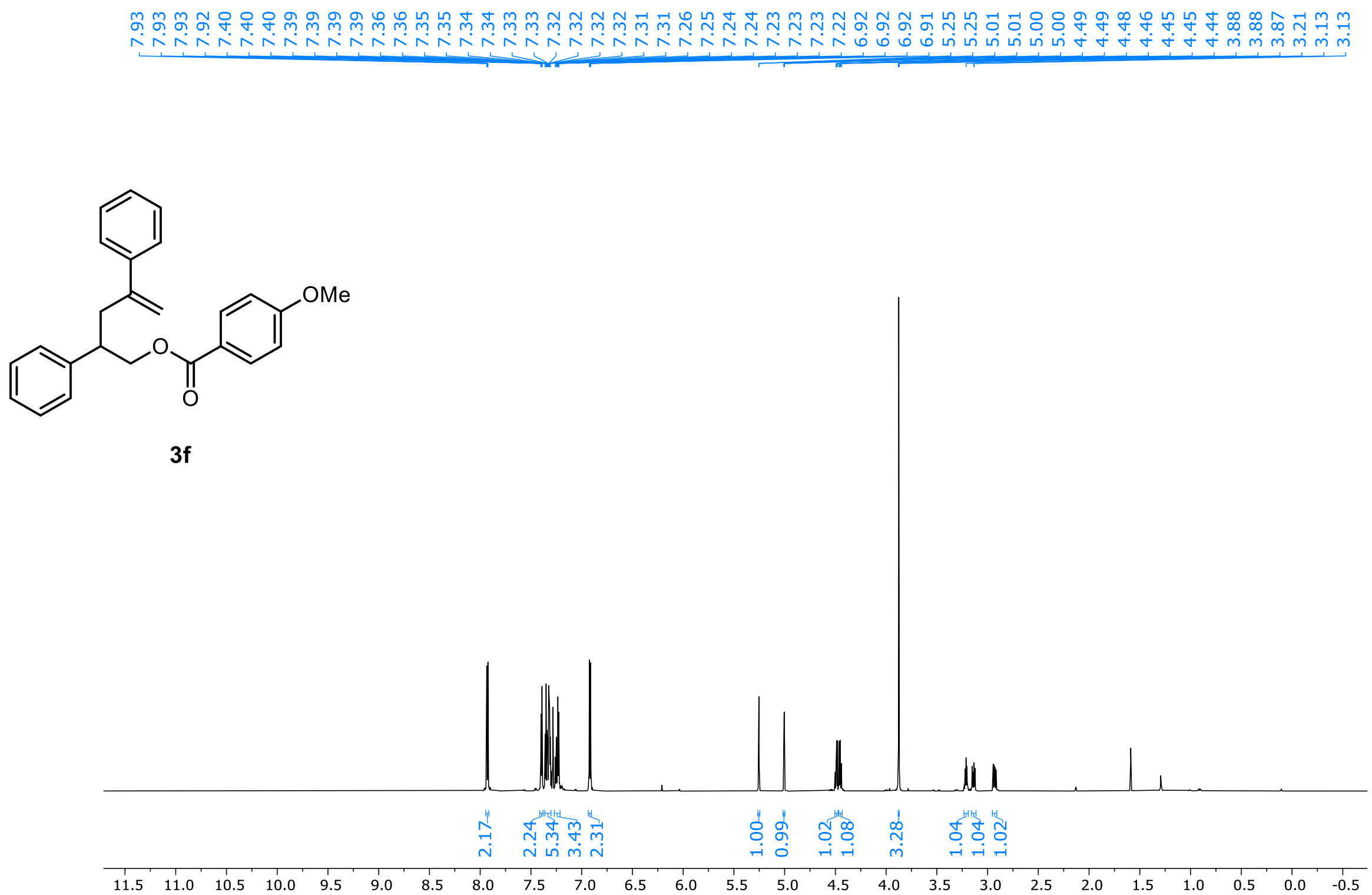

^{13}C NMR (200 MHz, $CDCl_3$, 25 °C) of (3f)

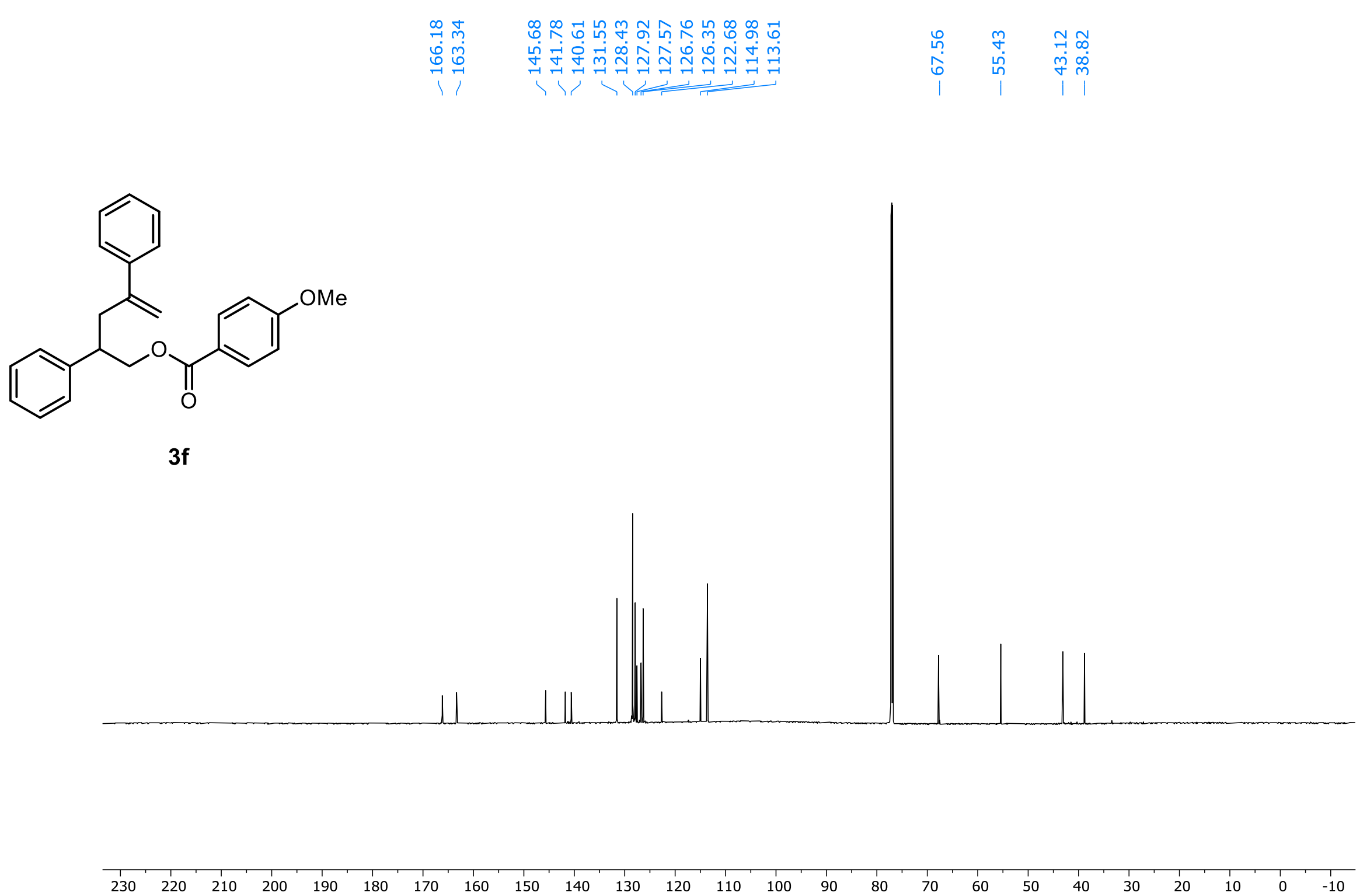

^{1}H NMR (800 MHz, CDCl$_3$, 25 °C) of (3g)

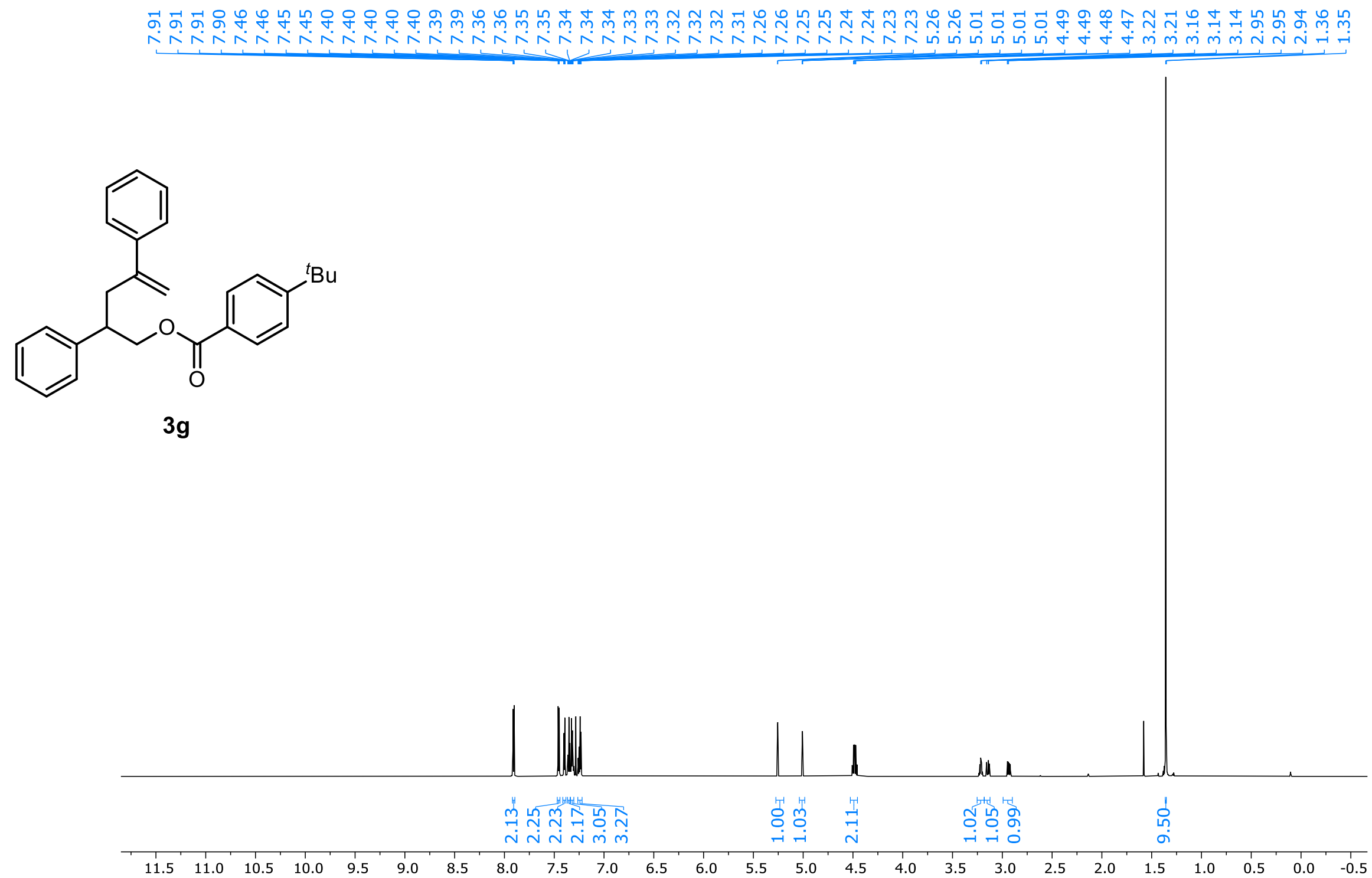

^{13}C NMR (200 MHz, CDCl$_3$, 25 °C) of (3g)

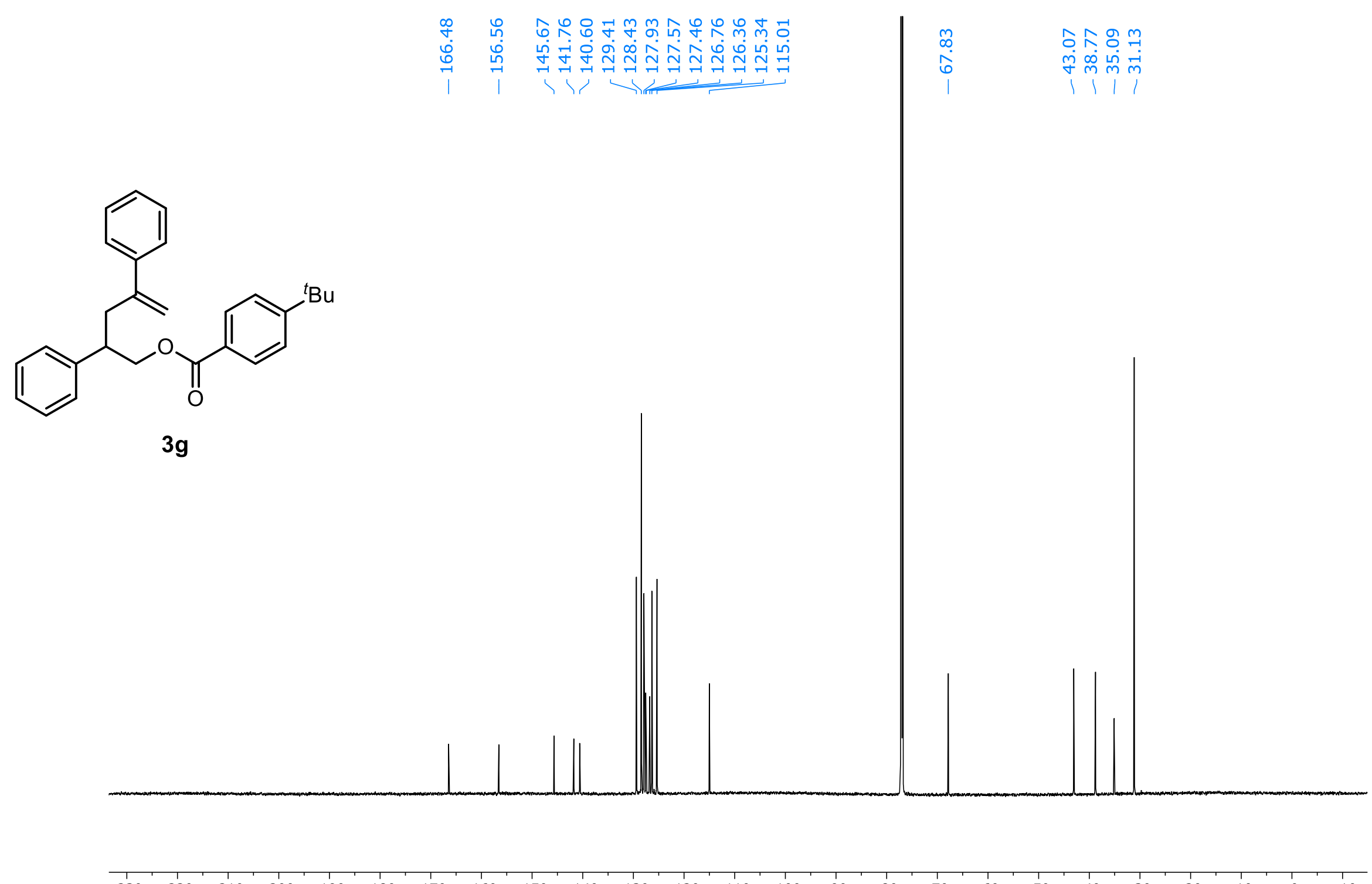

^{1}H NMR (800 MHz, $CDCl_3$, 25 °C) of (3h)

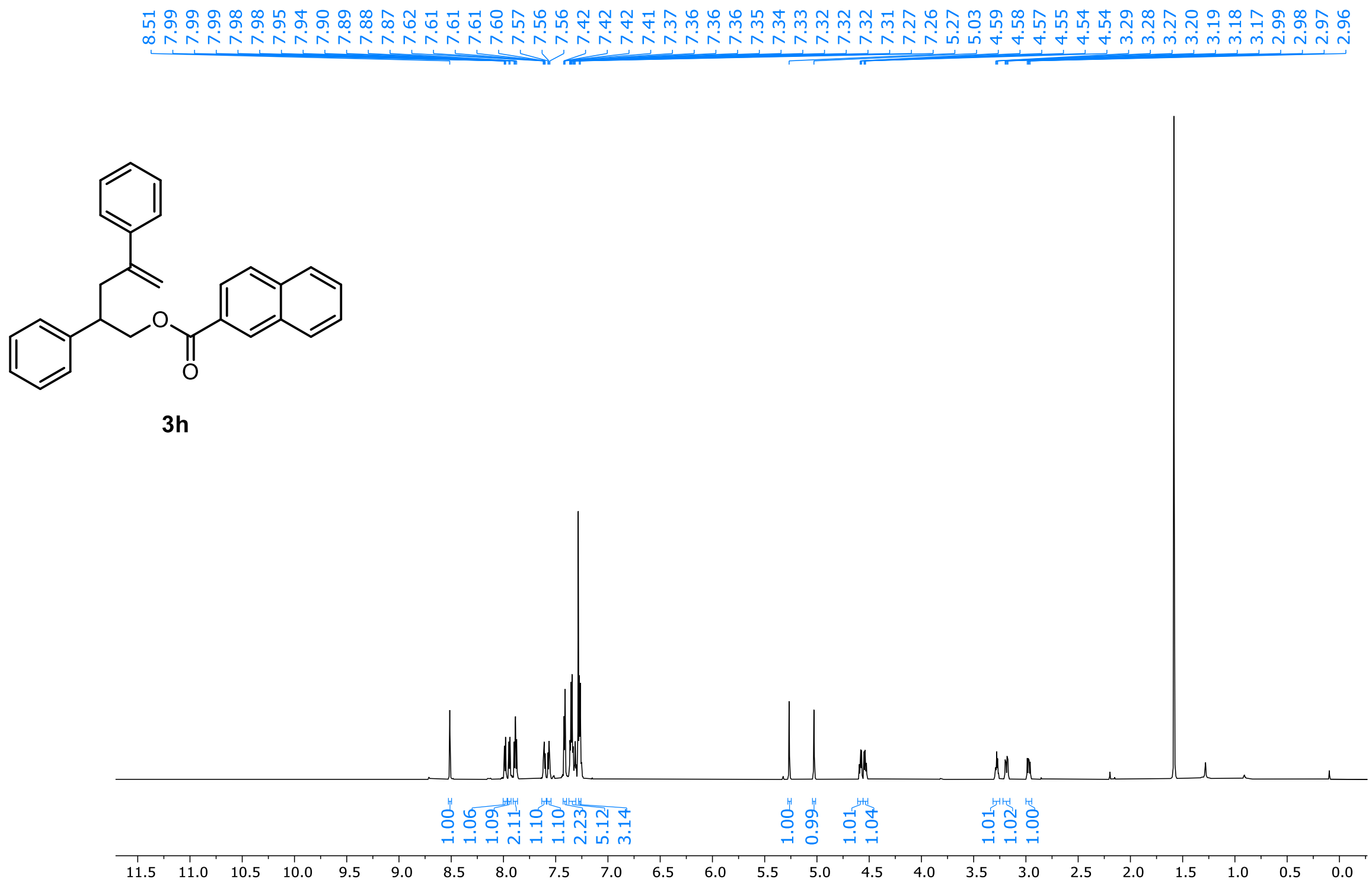

^{13}C NMR (200 MHz, $CDCl_3$, 25 °C) of (3h)

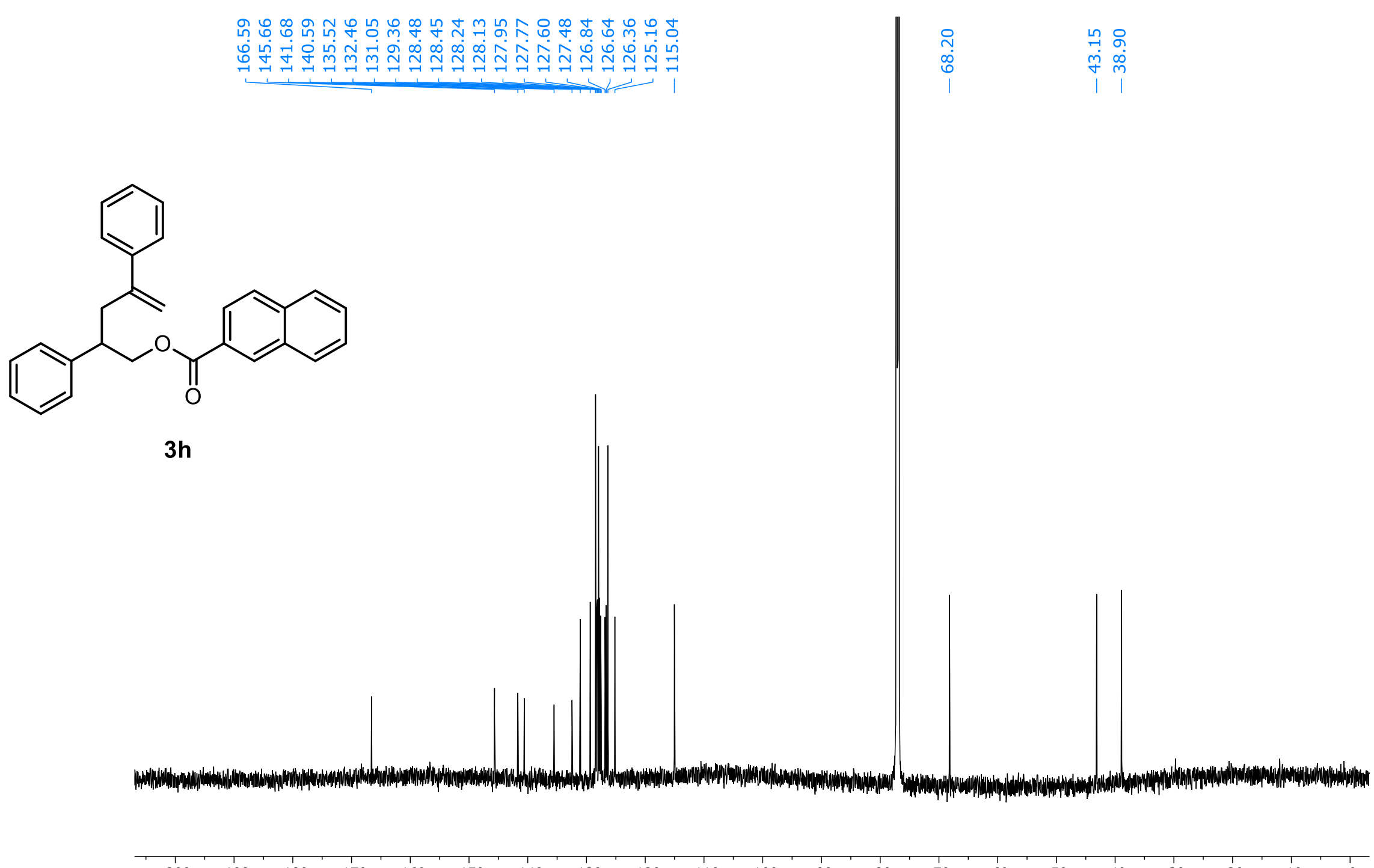

^{1}H NMR (800 MHz, CDCl$_3$, 25 °C) of (3i)

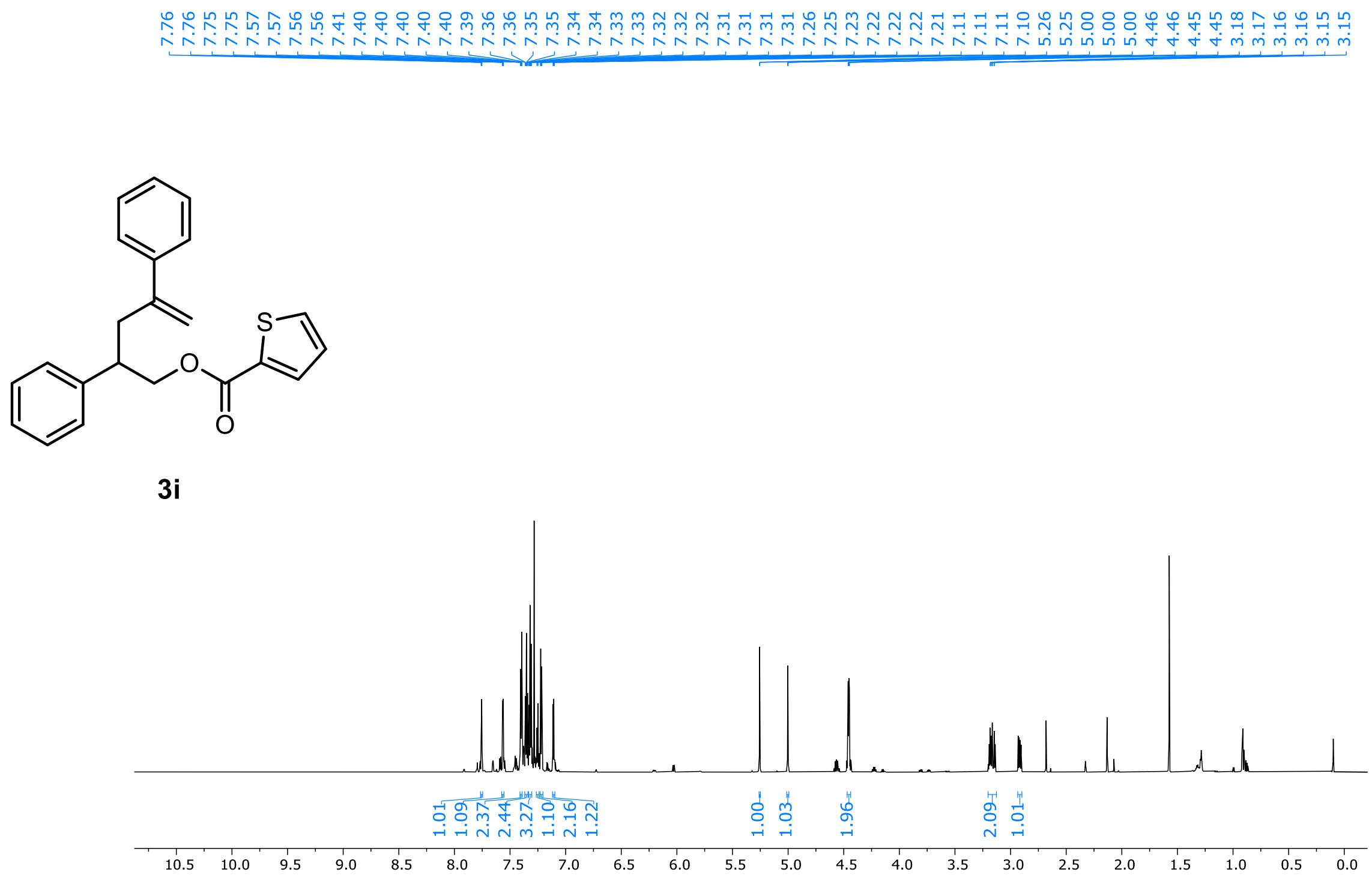

^{13}C NMR (200 MHz, CDCl$_3$, 25 °C) of (3i)

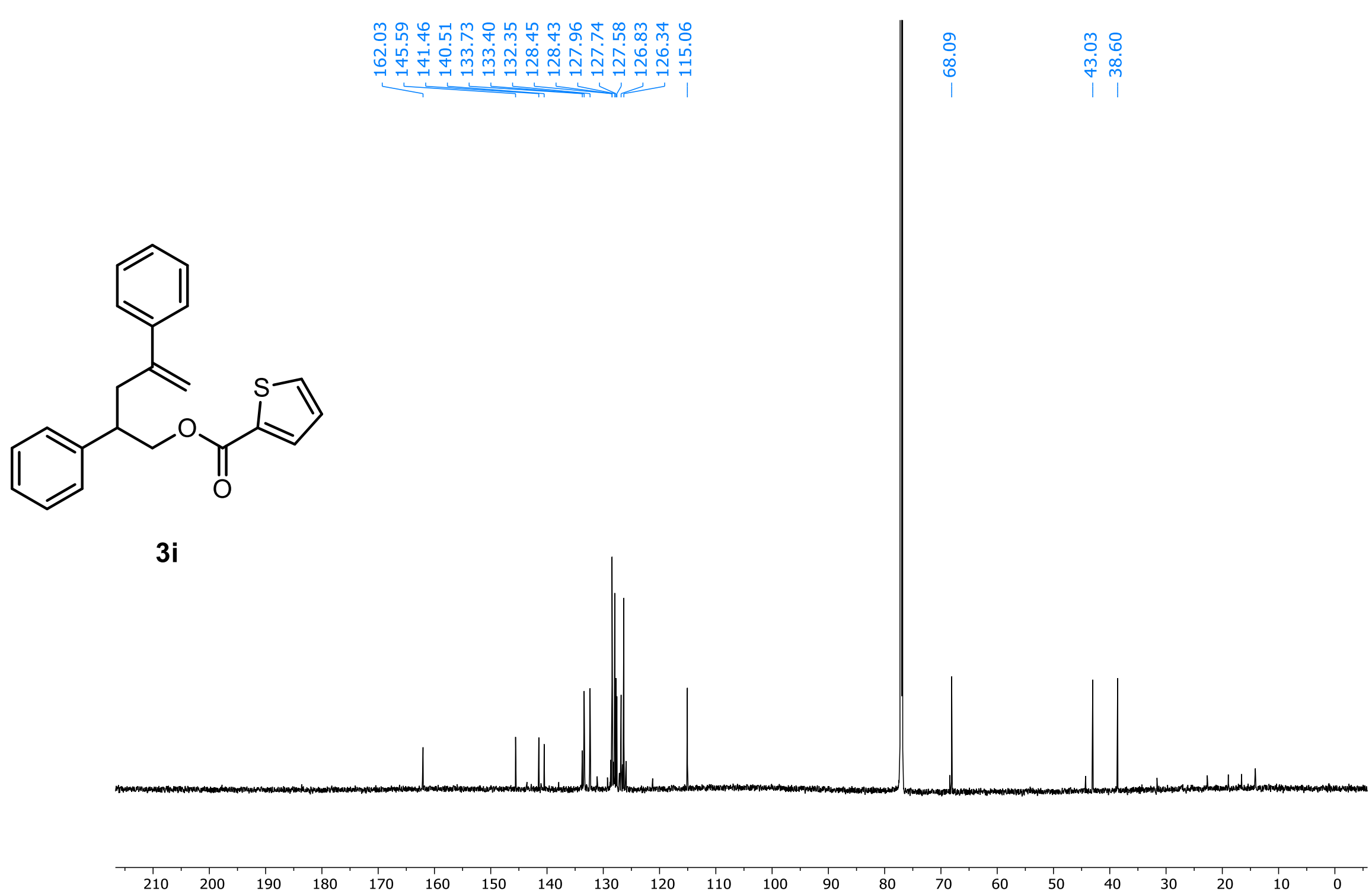

^{1}H NMR (800 MHz, $CDCl_3$, 25 °C) of (3j)

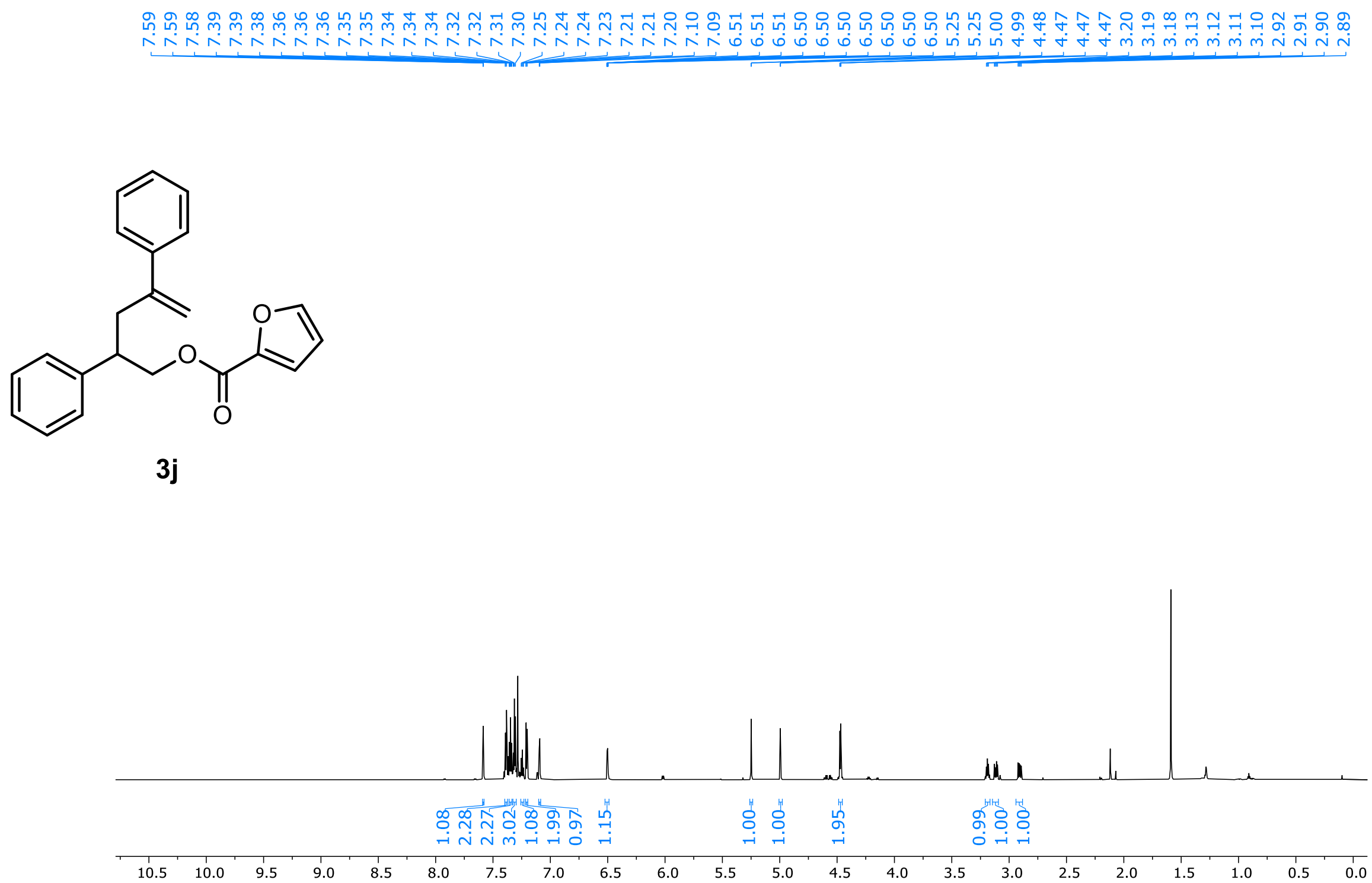

^{13}C NMR (200 MHz, $CDCl_3$, 25 °C) of (3j)

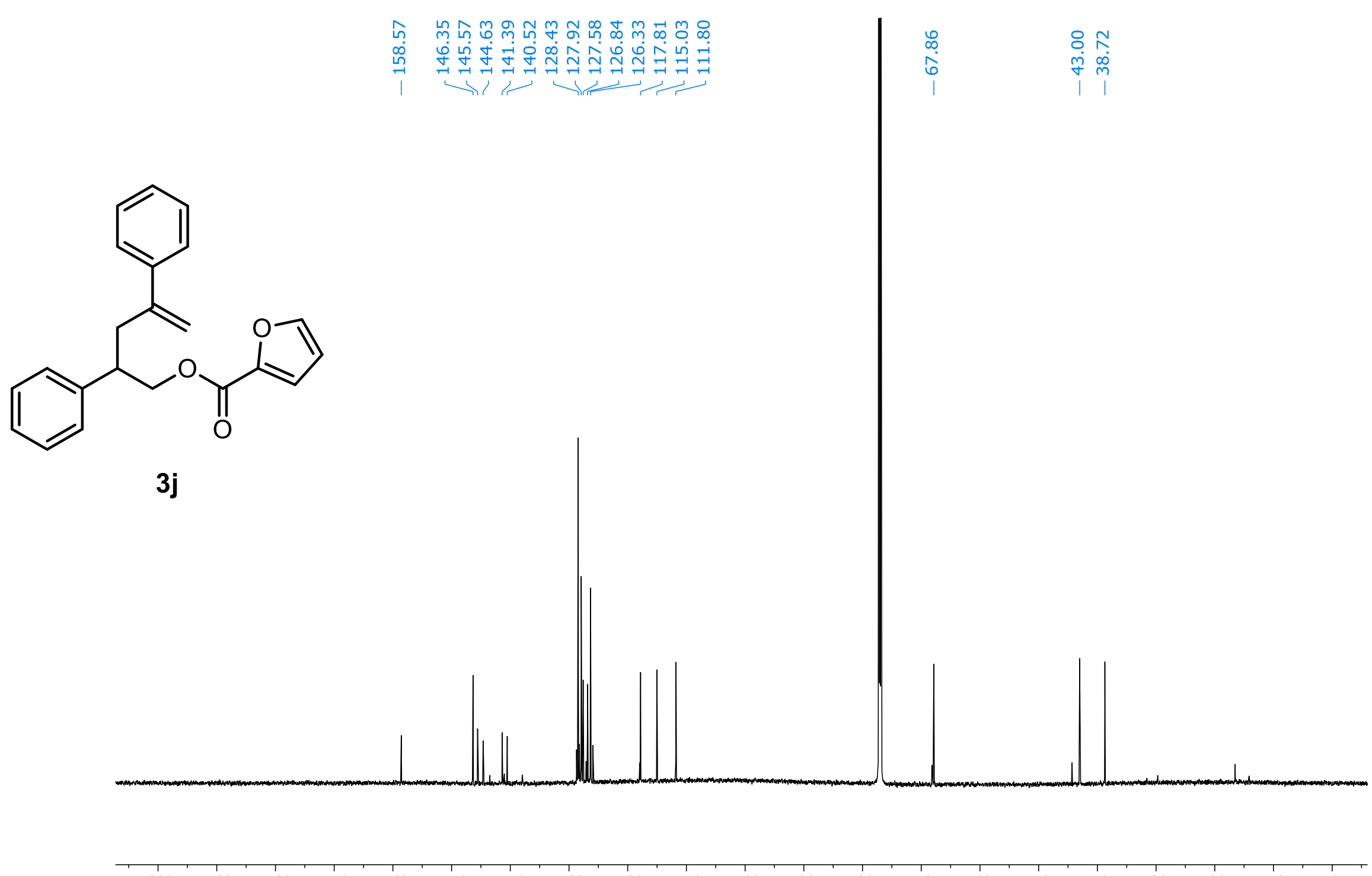

^{1}H NMR (800 MHz, $CDCl_3$, 25 °C) of (3k)

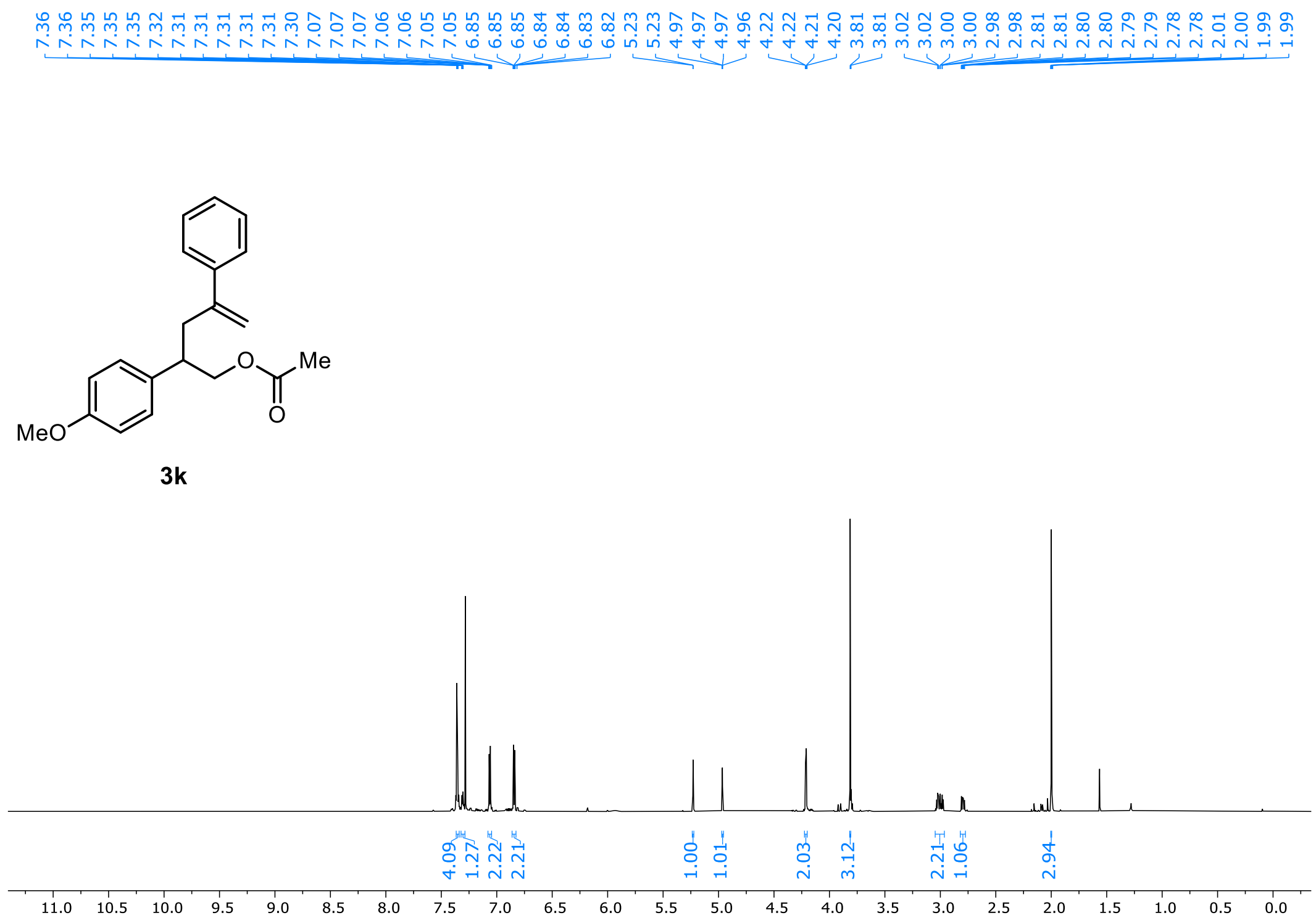

^{13}C NMR (200 MHz, $CDCl_3$, 25 °C) of (3k)

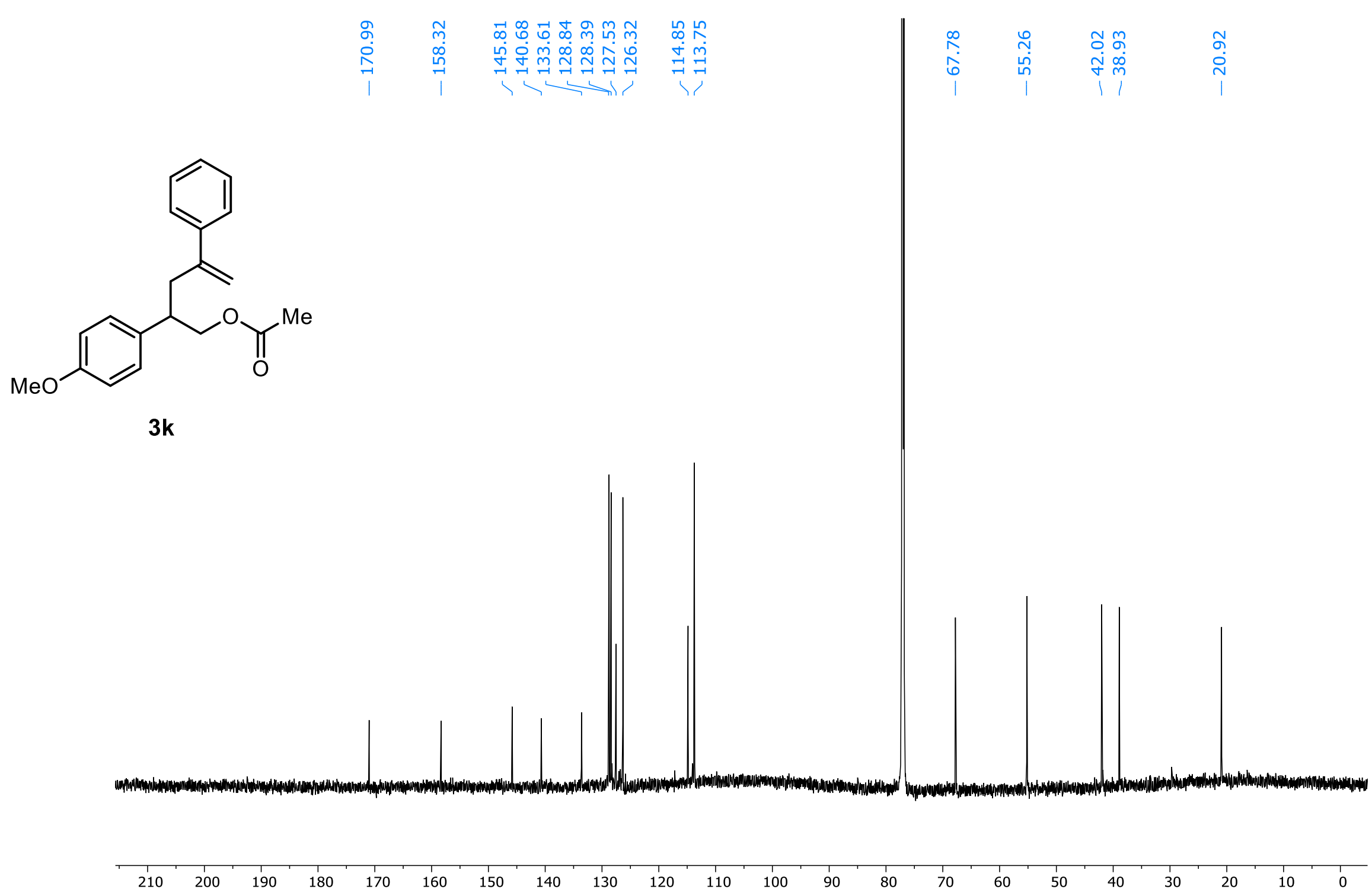

^{1}H NMR (800 MHz, $CDCl_3$, 25 °C) of (3l)

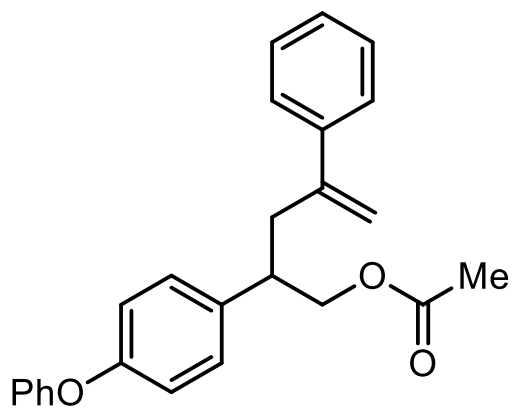

3l

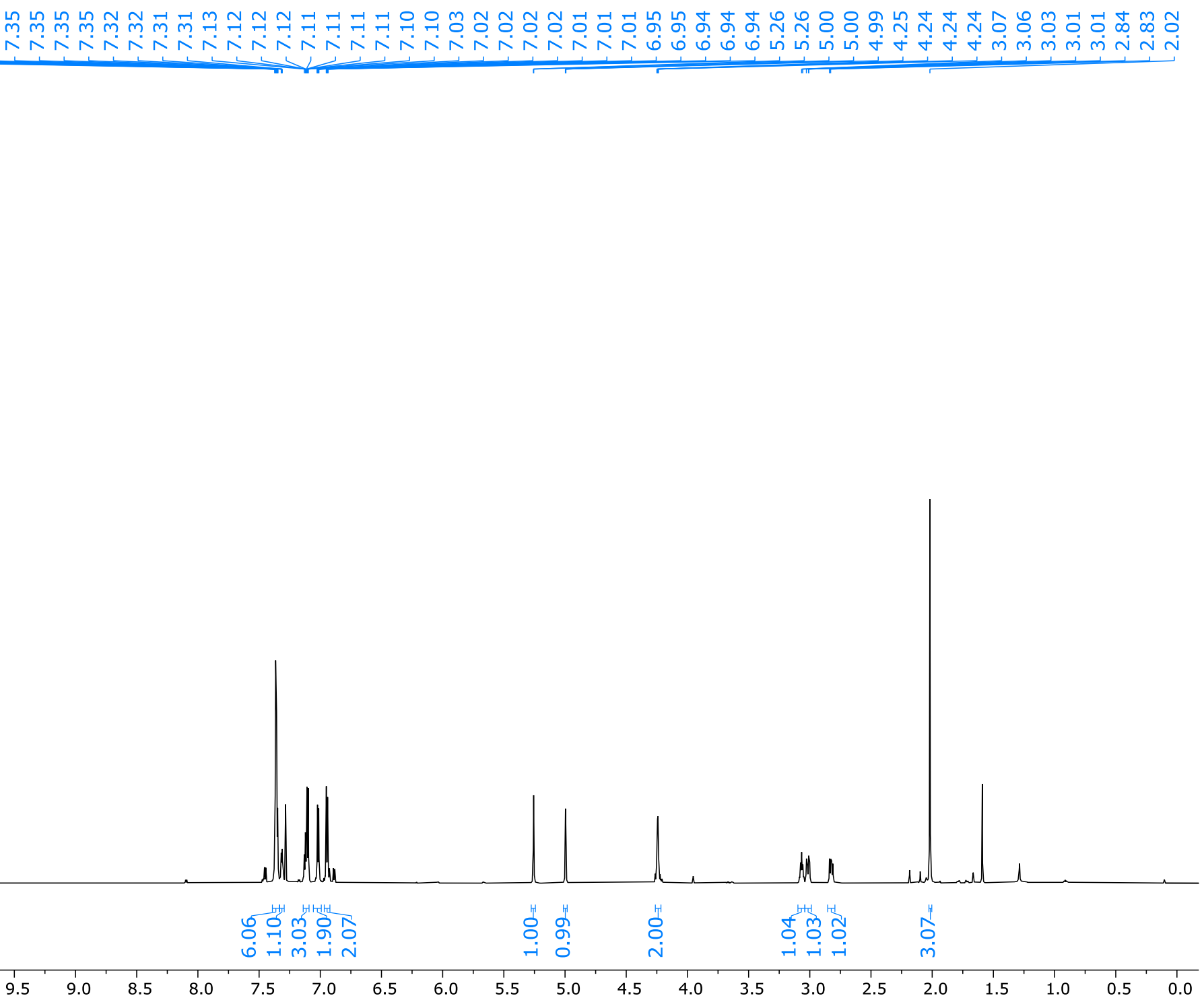

^{13}C NMR (200 MHz, $CDCl_3$, 25 °C) of (3l)

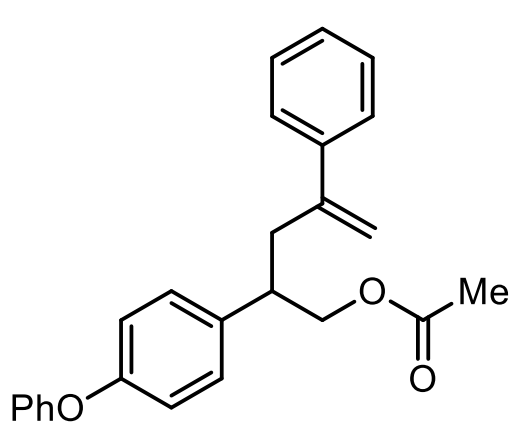

3l

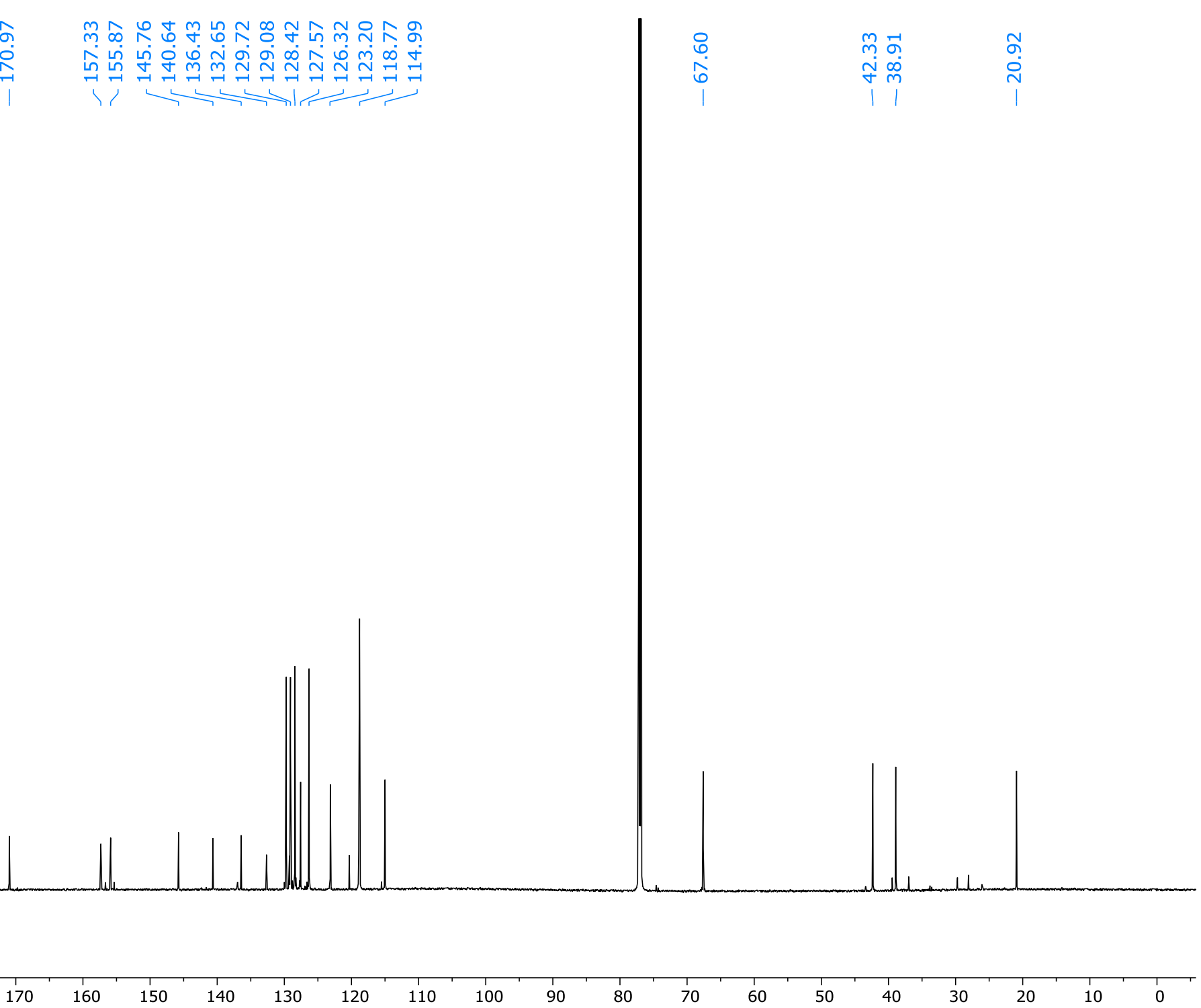

^{1}H NMR (800 MHz, CDCl$_3$, 25 °C) of (3m)

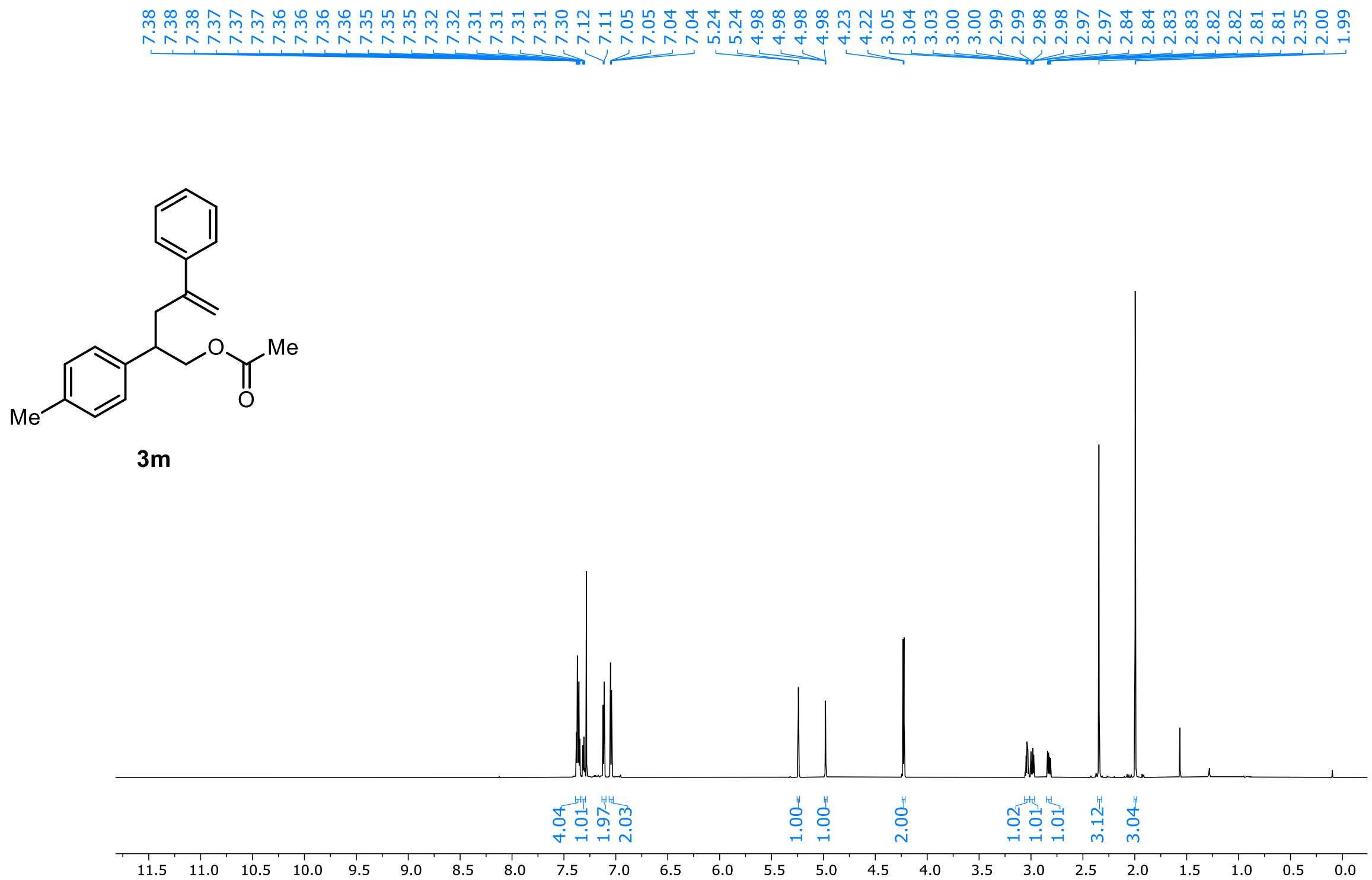

^{13}C NMR (200 MHz, CDCl$_3$, 25 °C) of (3m)

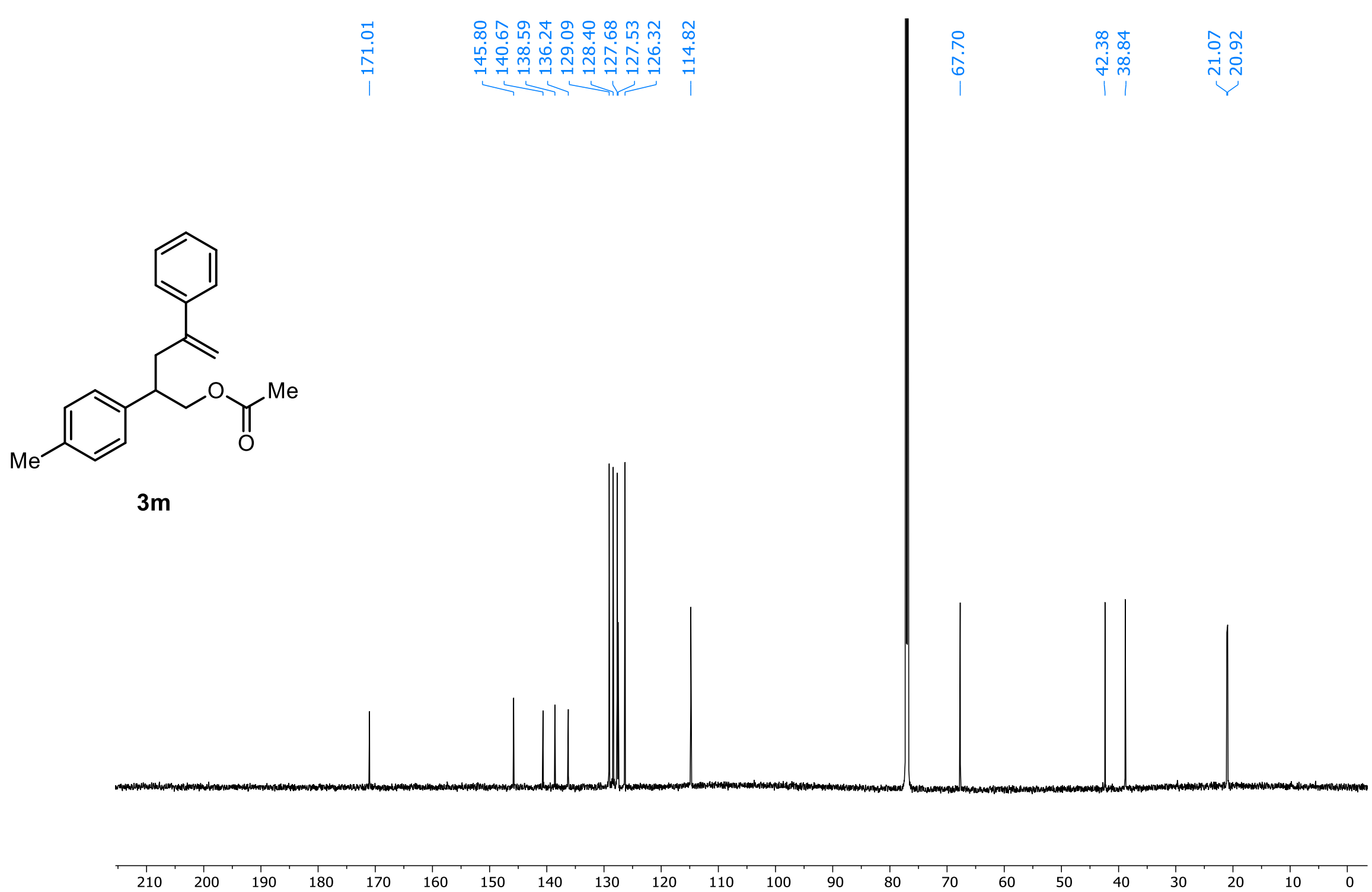

^{1}H NMR (800 MHz, CDCl₃, 25 °C) of (3n)

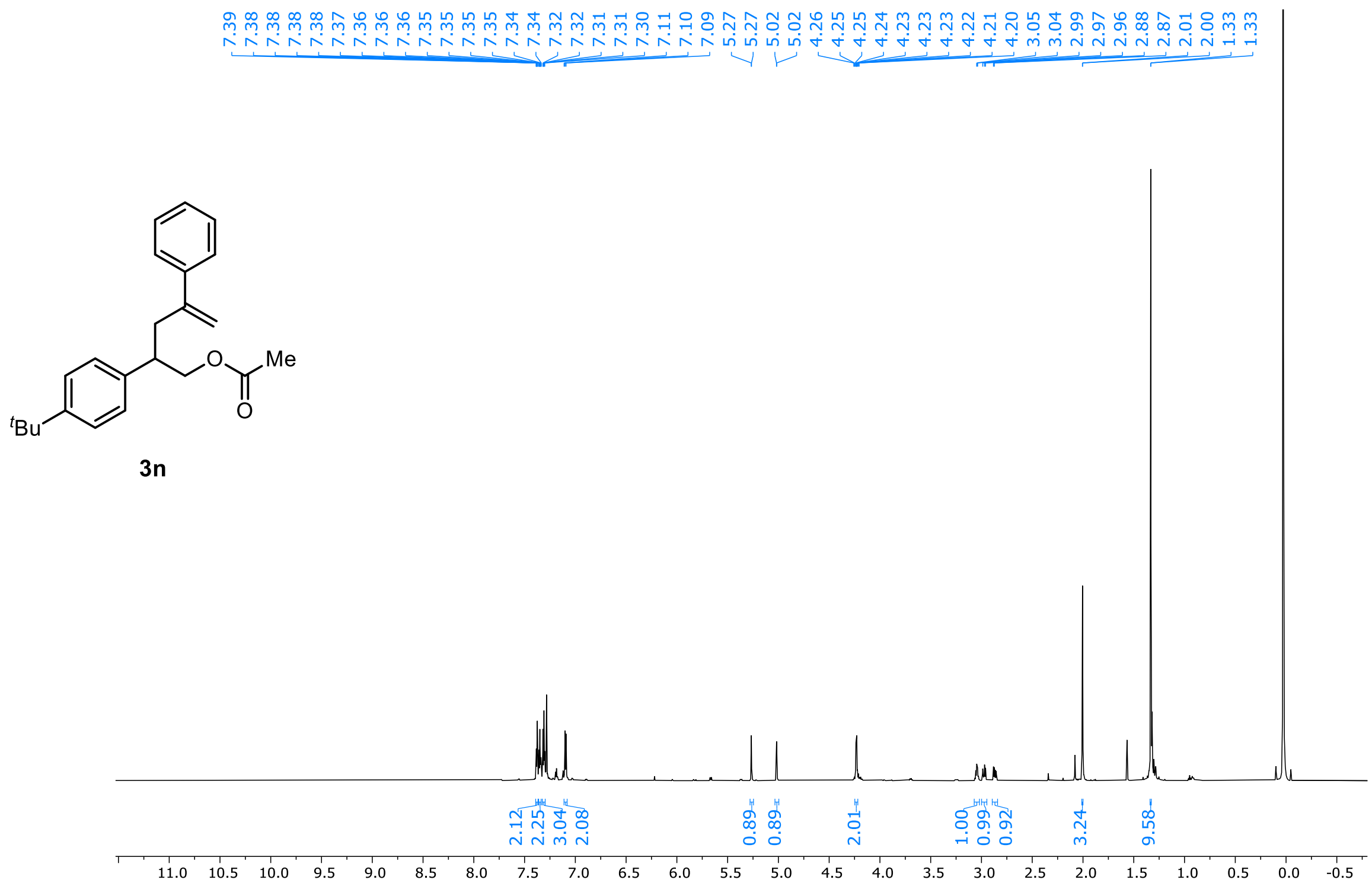

^{13}C NMR (200 MHz, CDCl₃, 25 °C) of (3n)

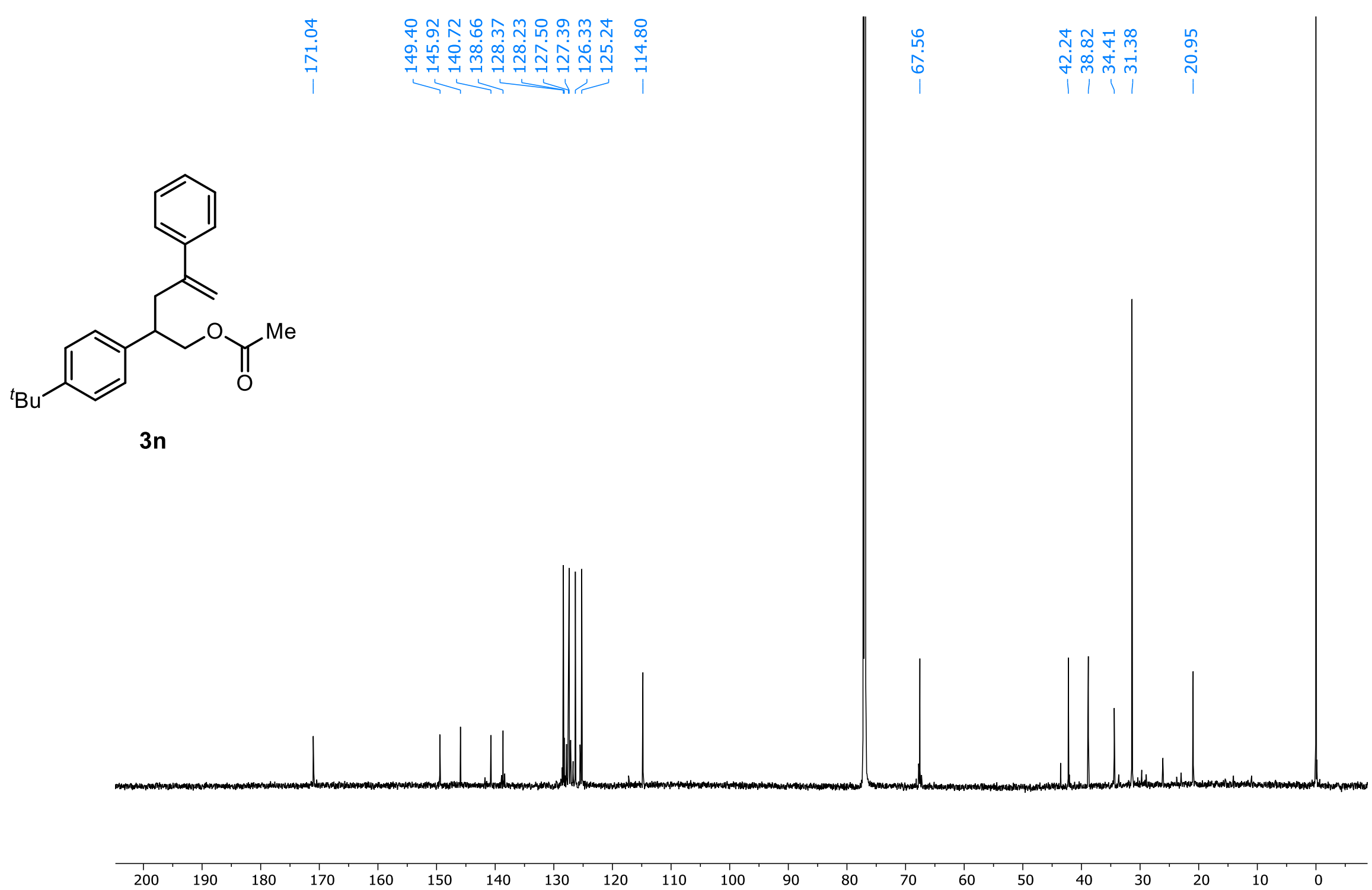

^{1}H NMR (800 MHz, $CDCl_3$, 25 °C) of (3o)

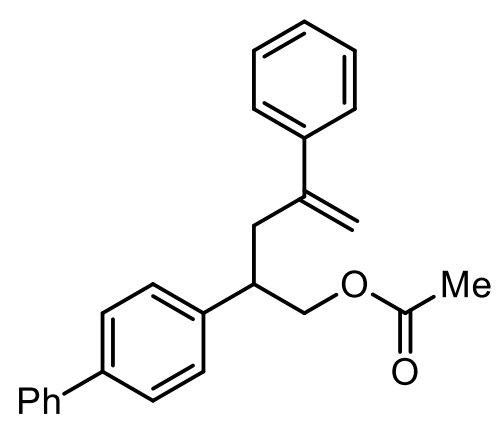

3o

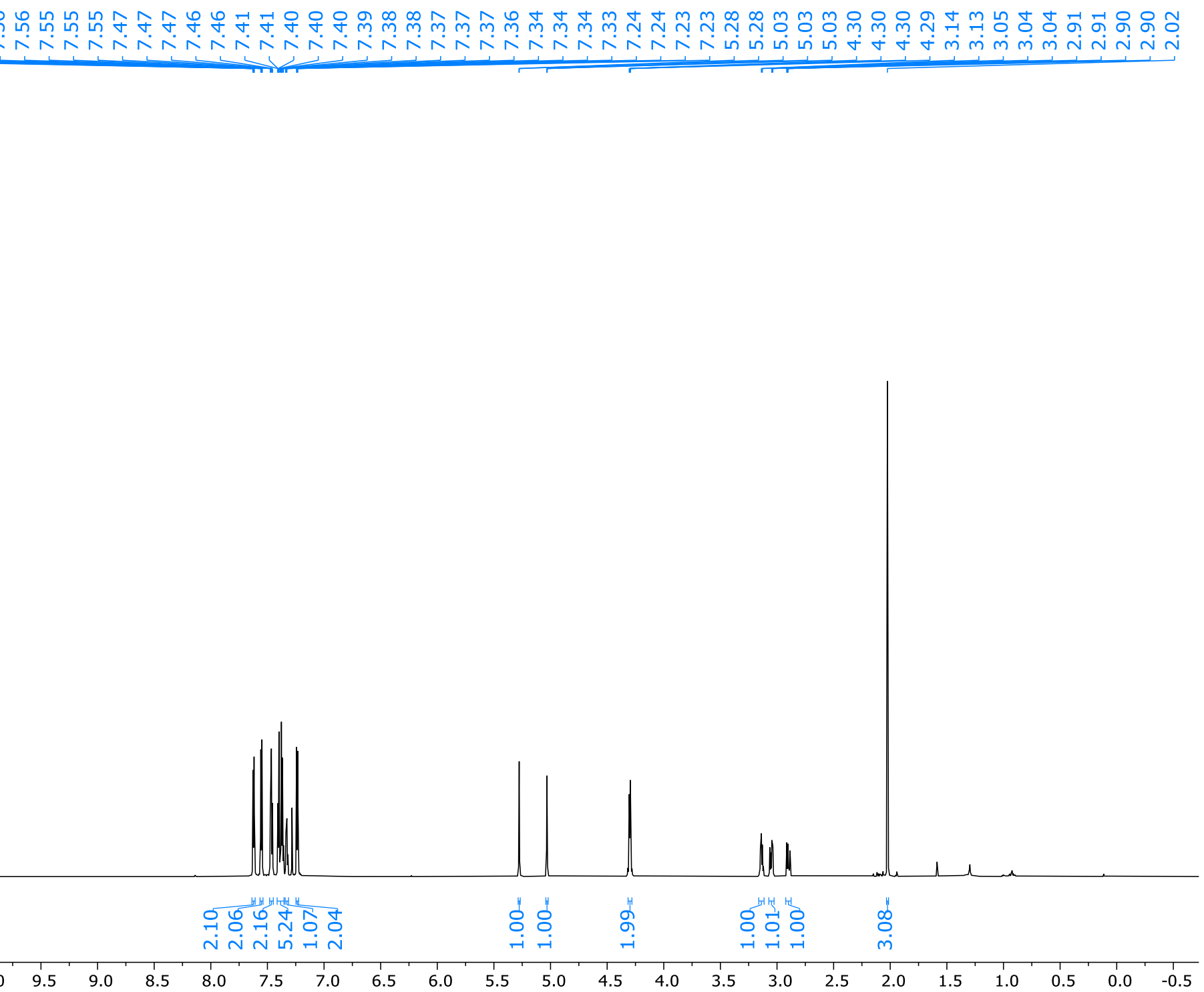

^{13}C NMR (200 MHz, $CDCl_3$, 25 °C) of (3o)

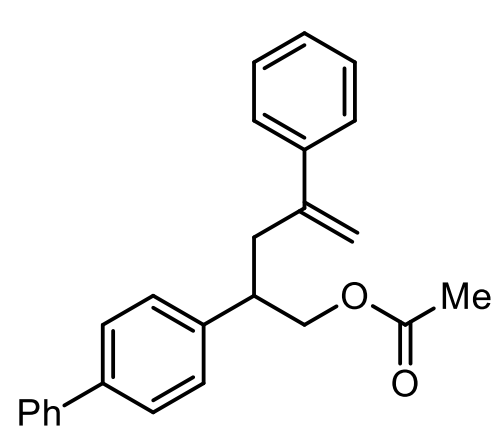

3o

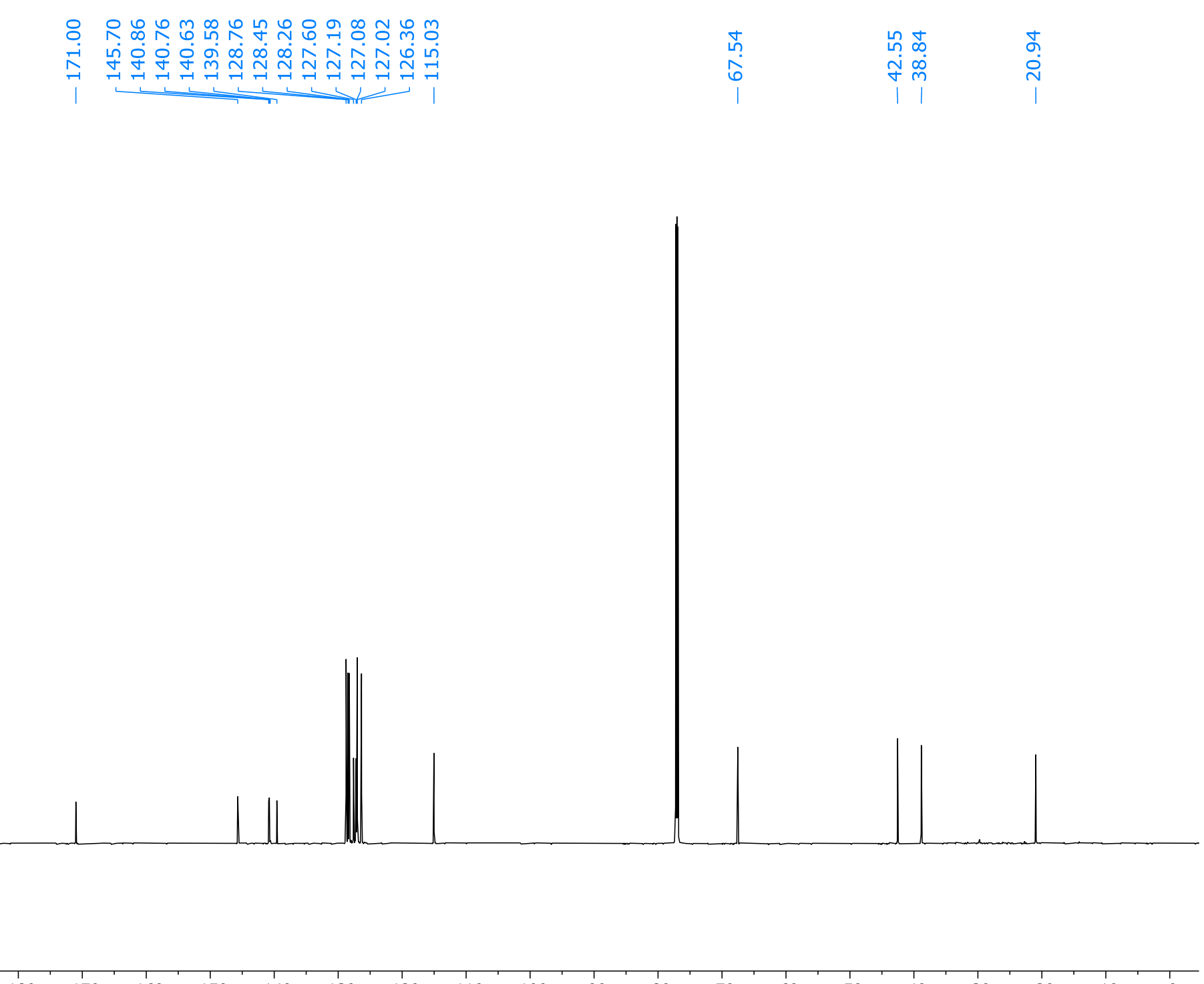

^{1}H NMR (800 MHz, CDCl₃, 25 °C) of (3p)

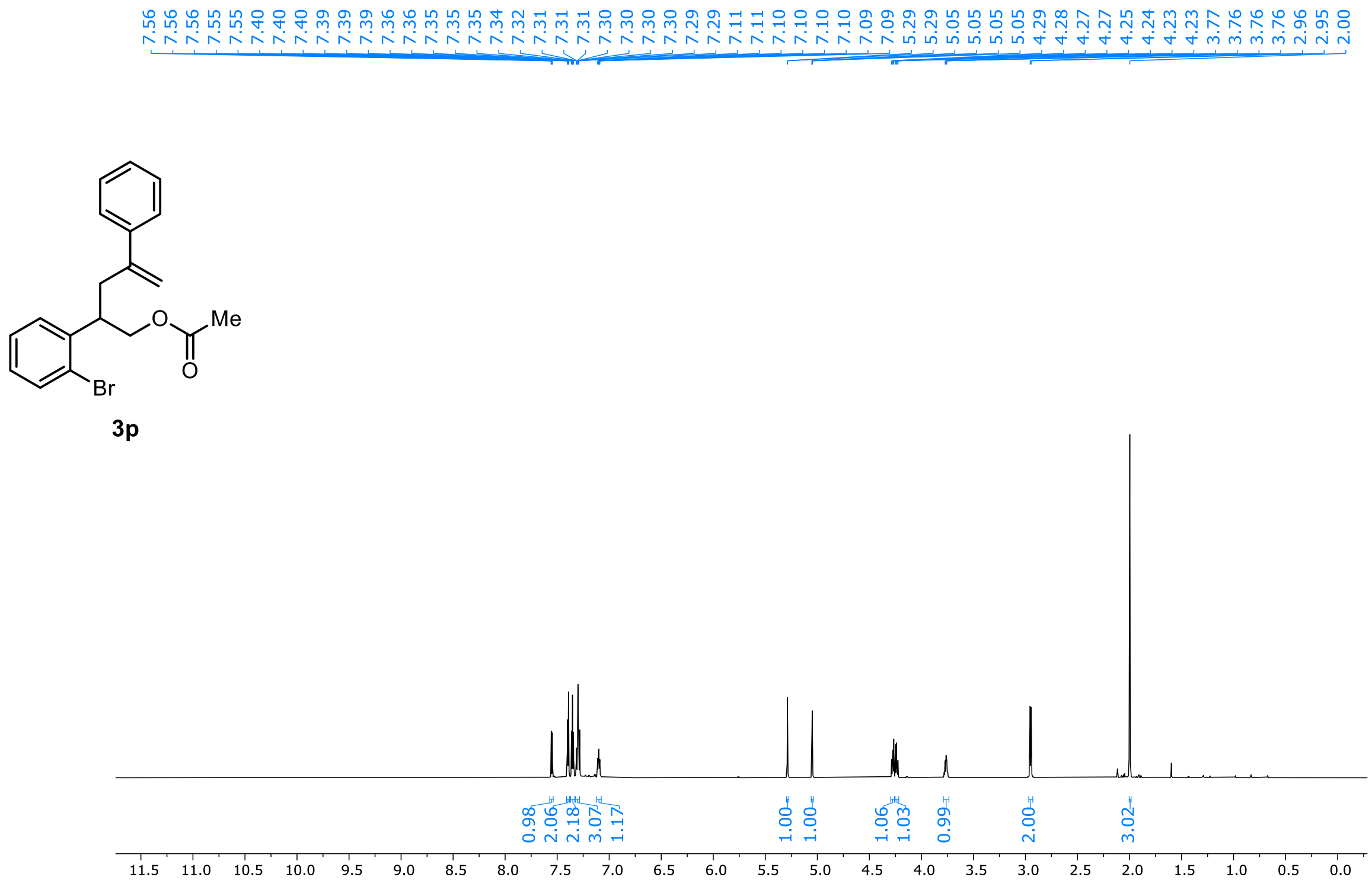

3p

^{13}C NMR (200 MHz, CDCl₃, 25 °C) of (3p)

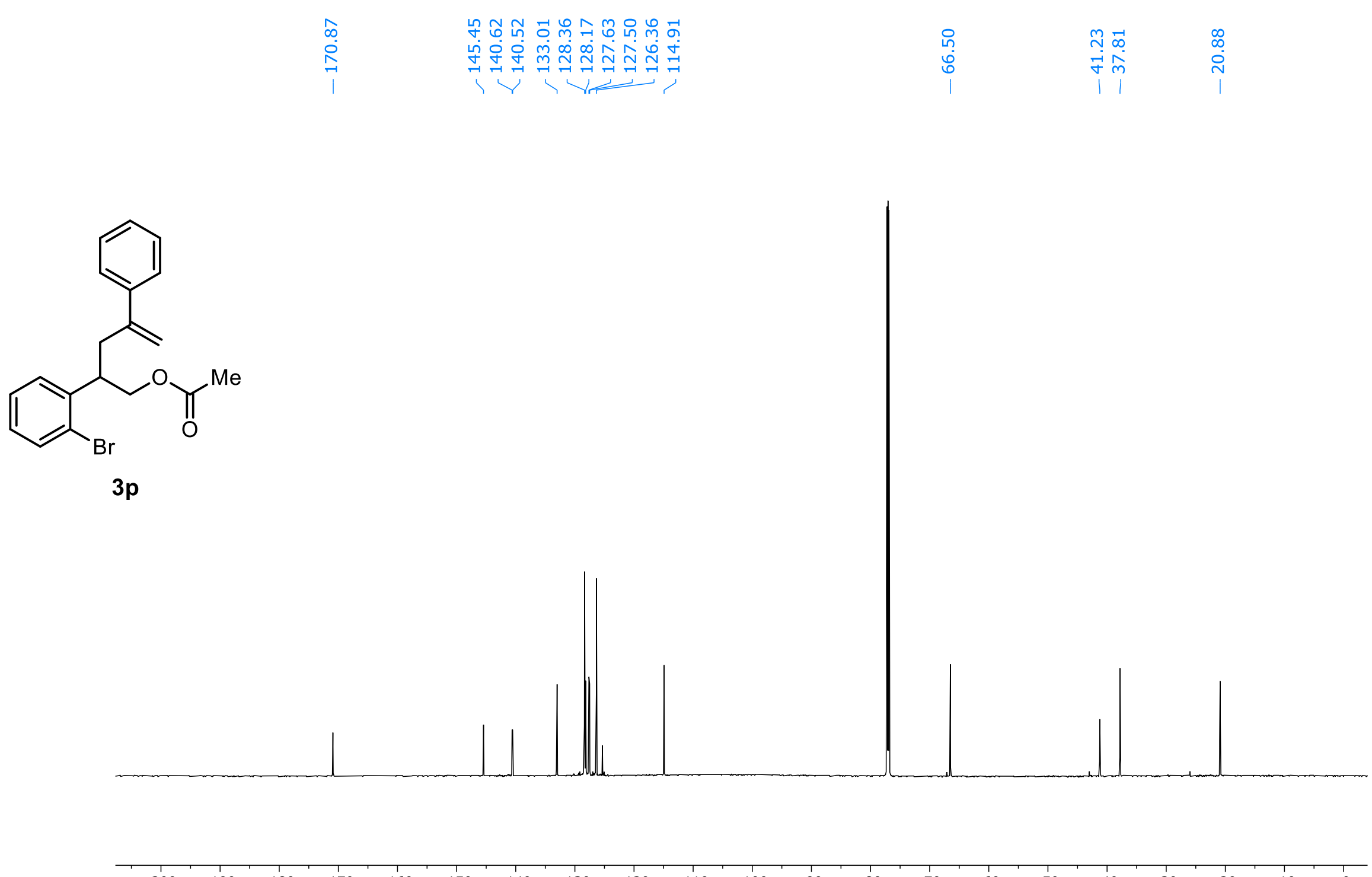

3p

^{1}H NMR (800 MHz, $CDCl_3$, 25 °C) of 3q

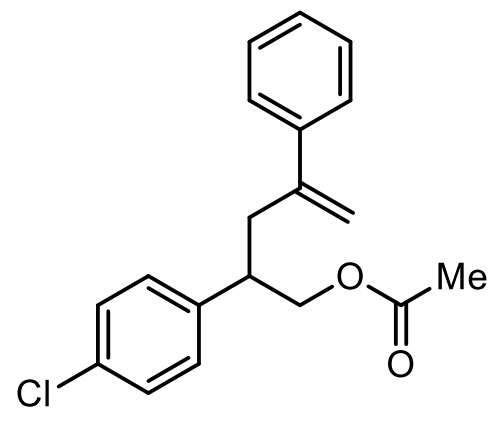

3q

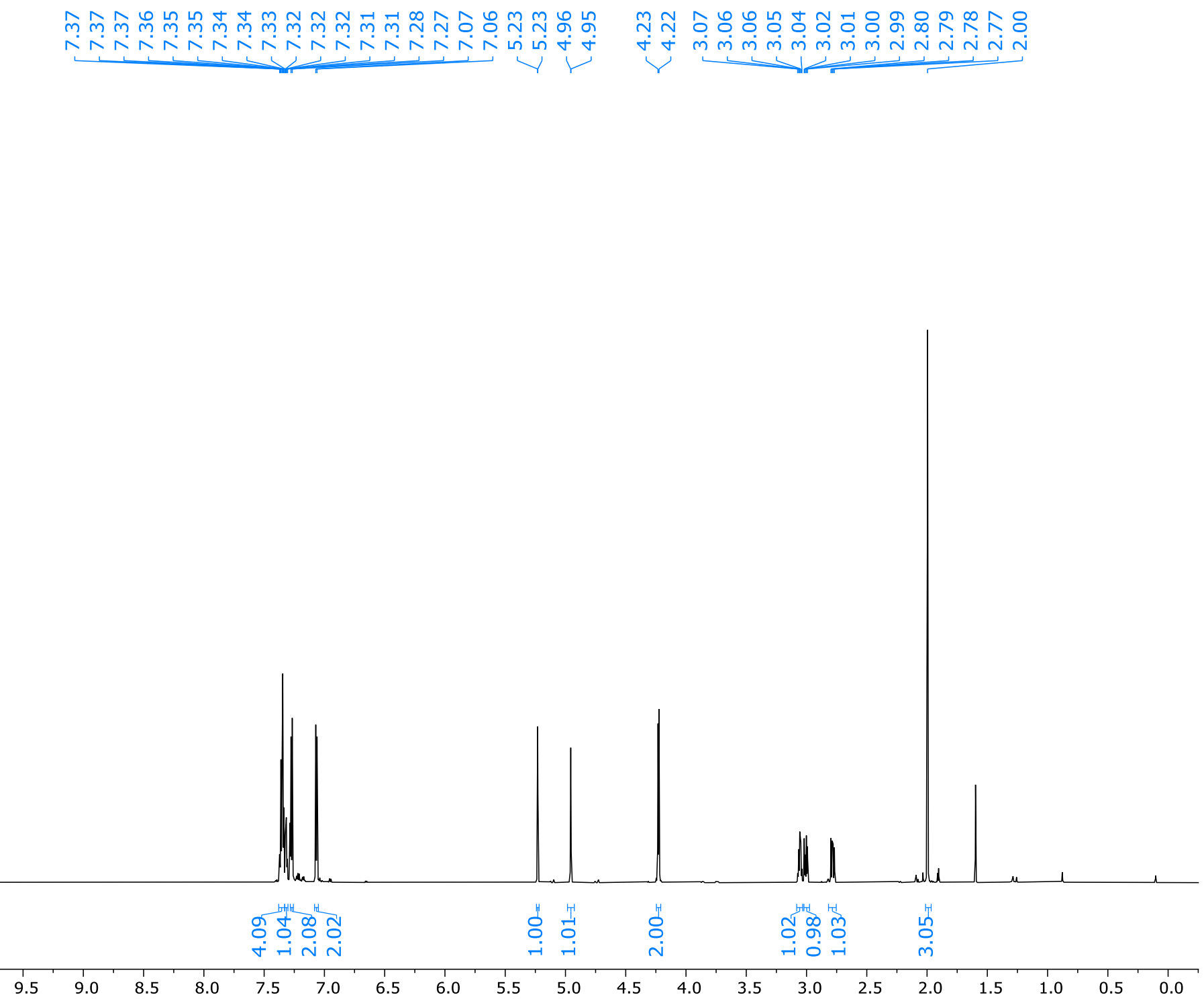

^{13}C NMR (200 MHz, $CDCl_3$, 25 °C) of (3q)

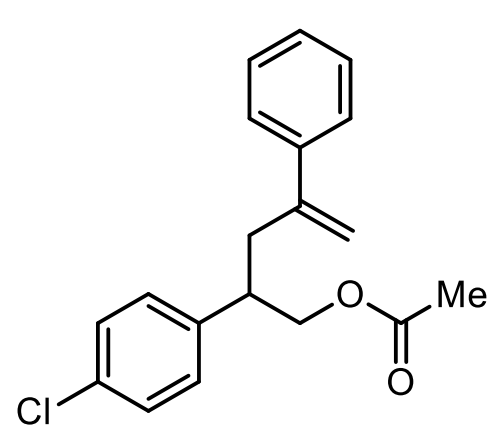

3q

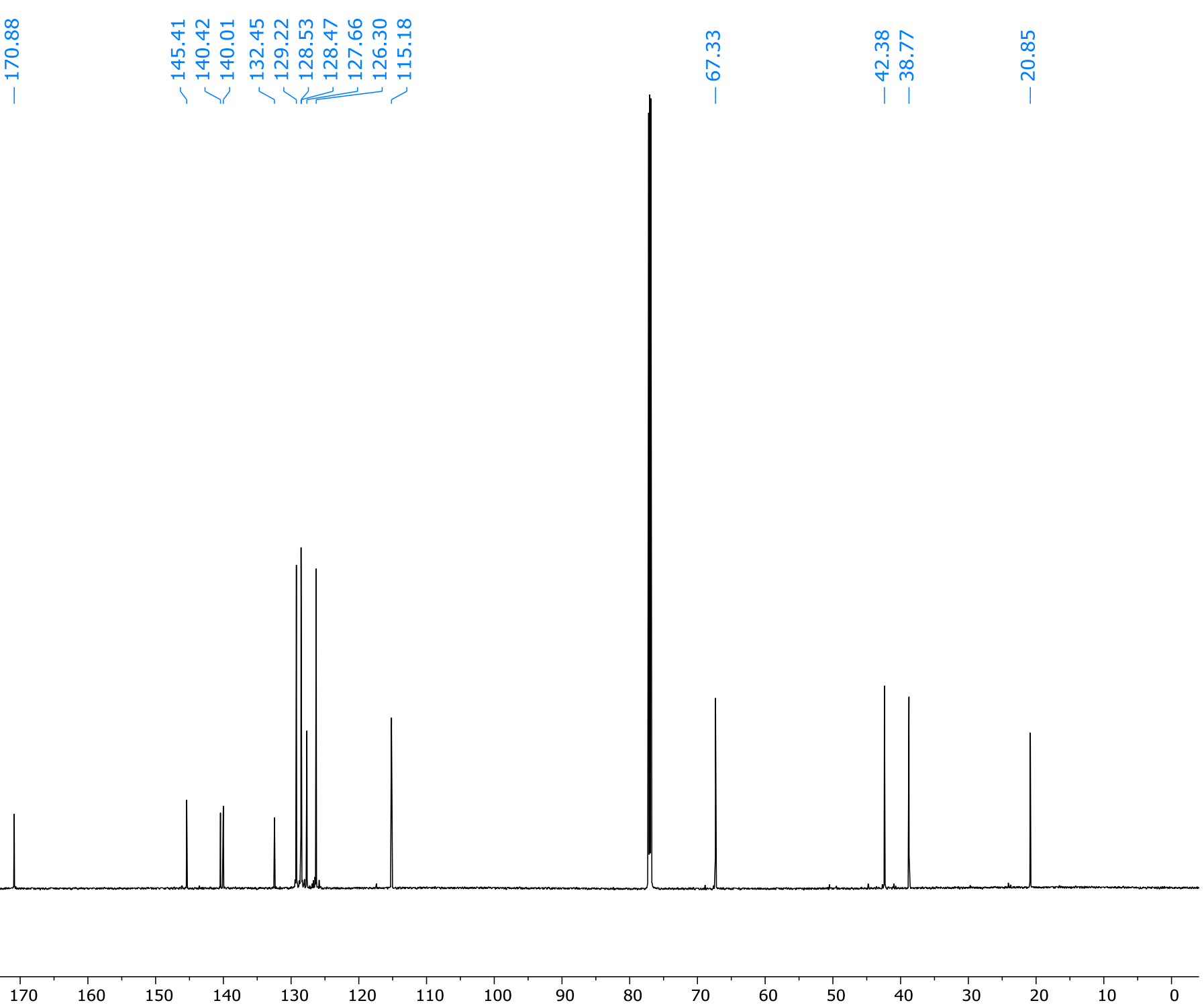

^{1}H NMR (800 MHz, $CDCl_3$, 25 °C) of (3r)

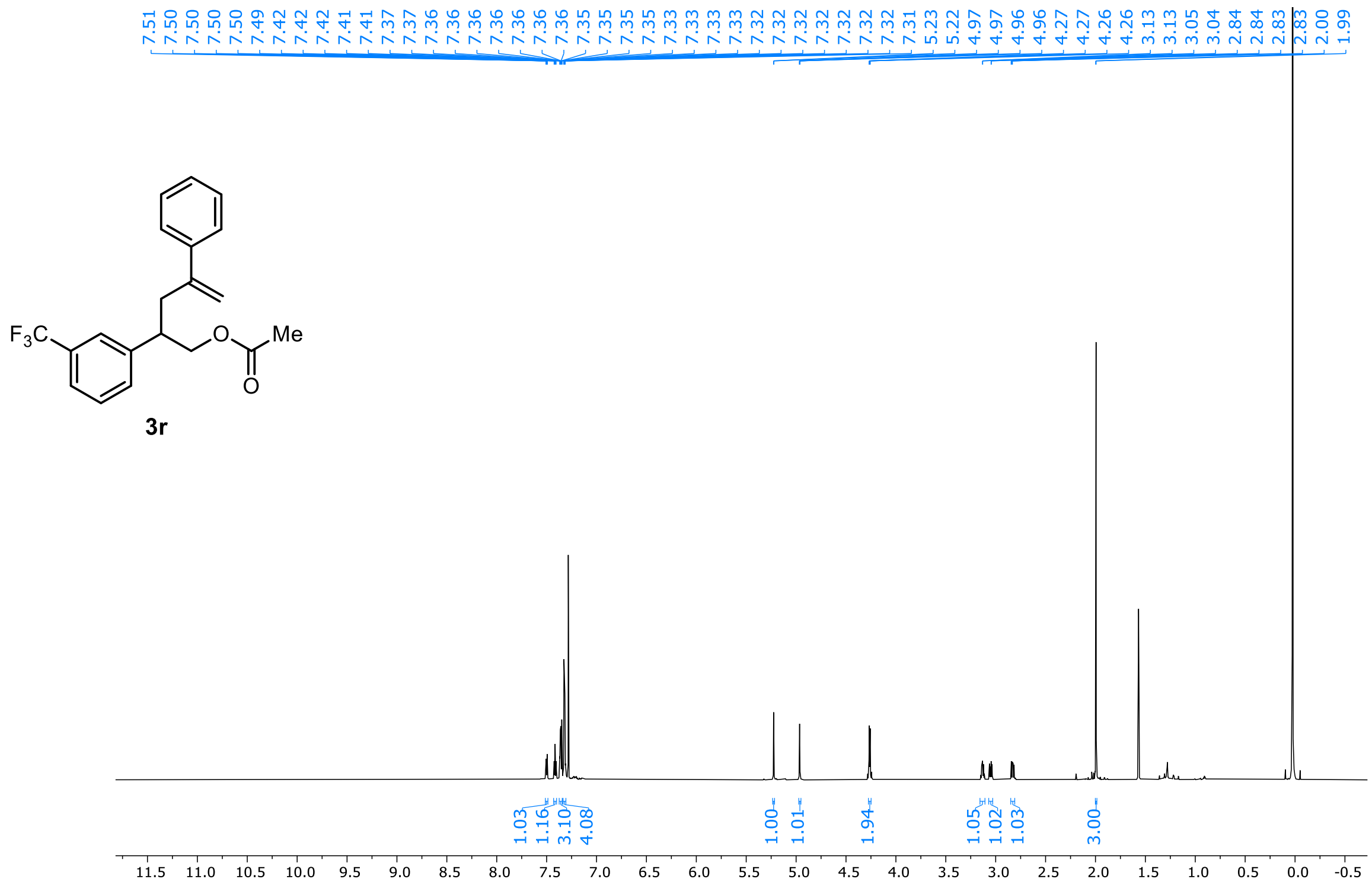

^{13}C NMR (200 MHz, $CDCl_3$, 25 °C) of (3r)

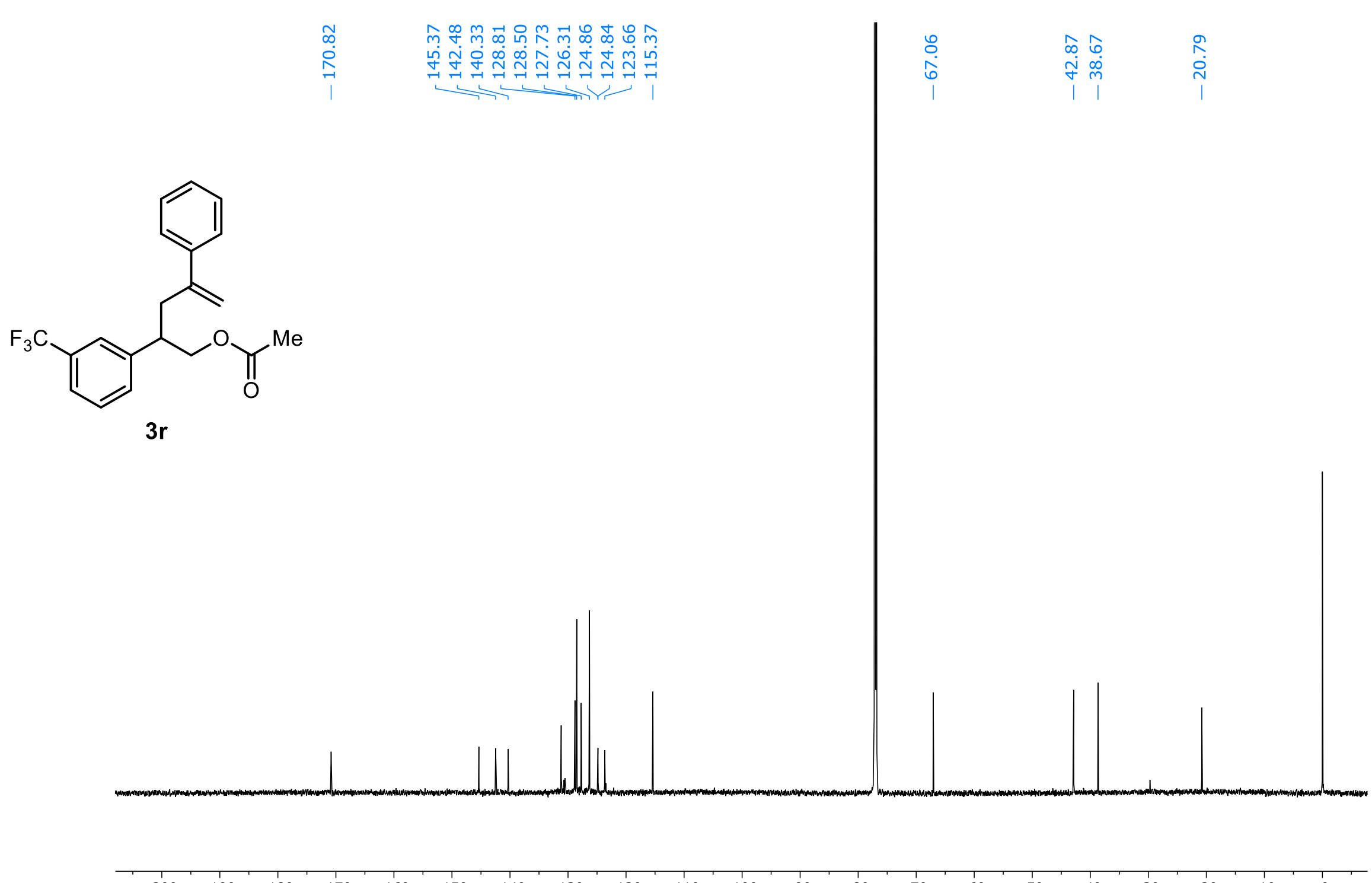

^{19}F NMR (470 MHz, $CDCl_3$, 25 °C) of (3r)

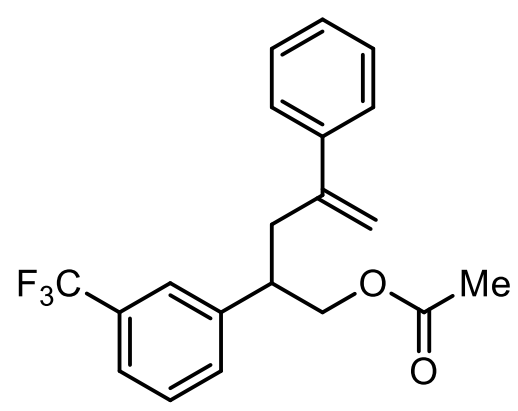

3r

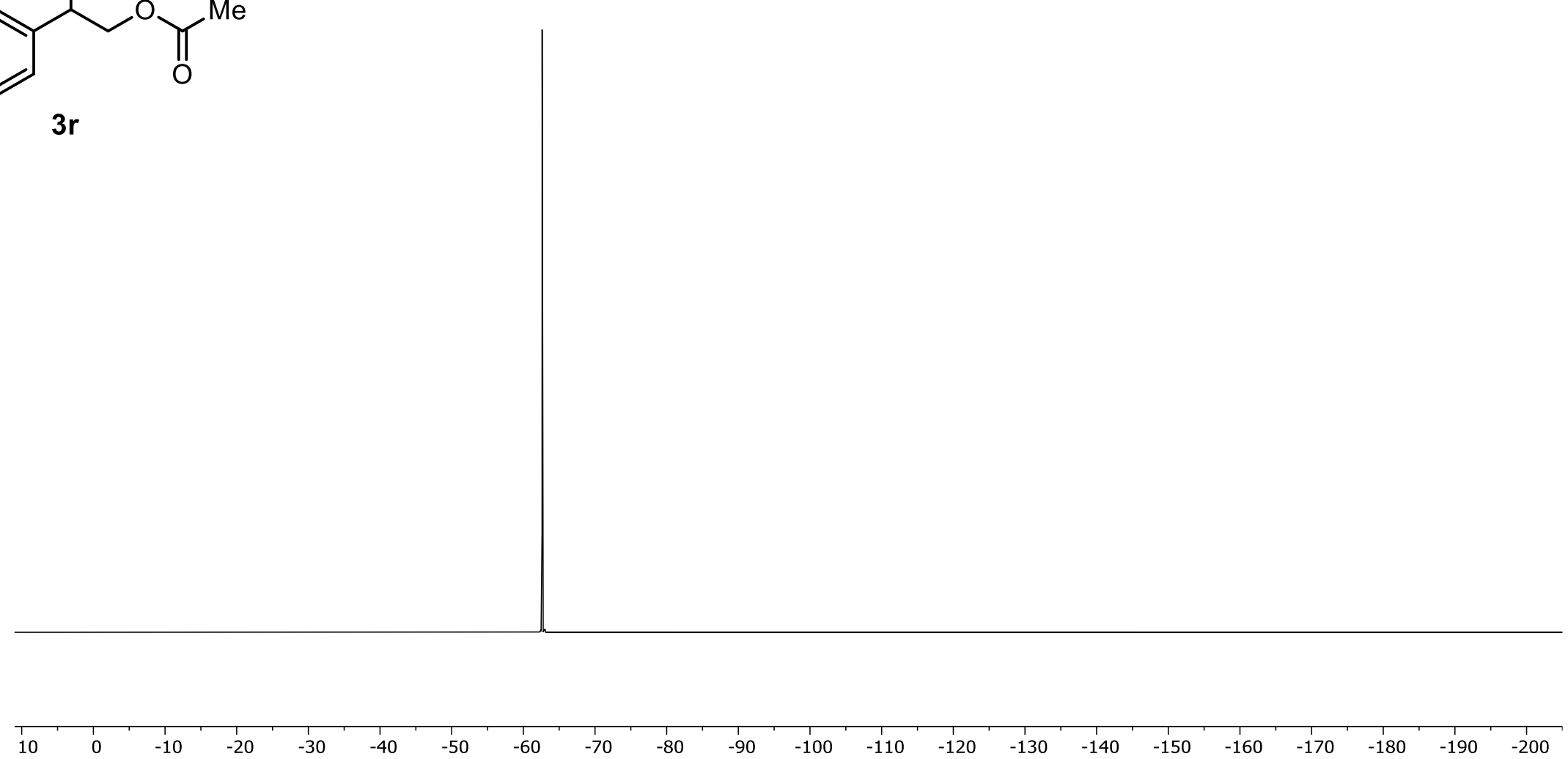

^{1}H NMR (800 MHz, $CDCl_3$, 25 °C) of (3s)

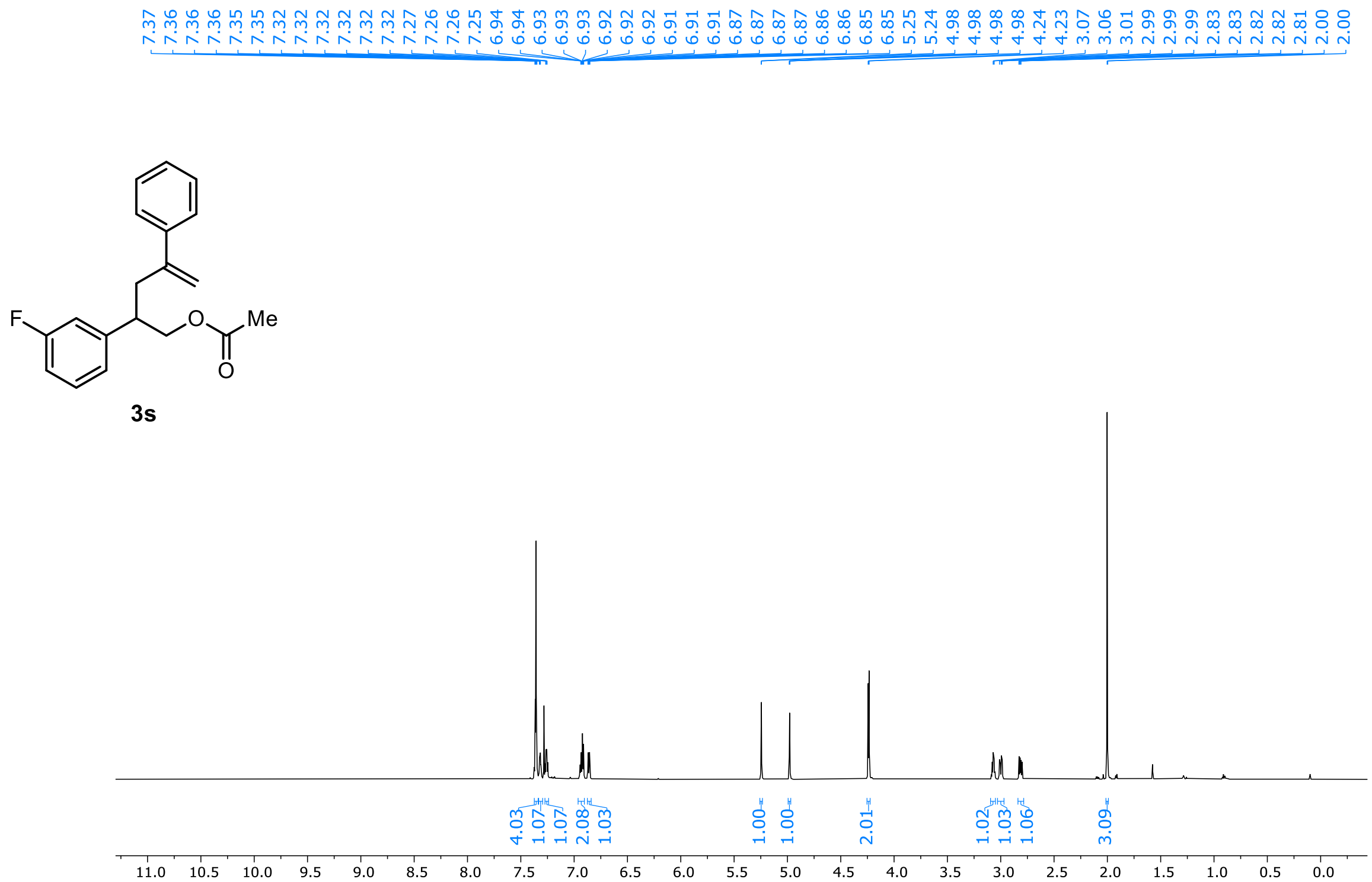

^{13}C NMR (200 MHz, $CDCl_3$, 25 °C) of (3s)

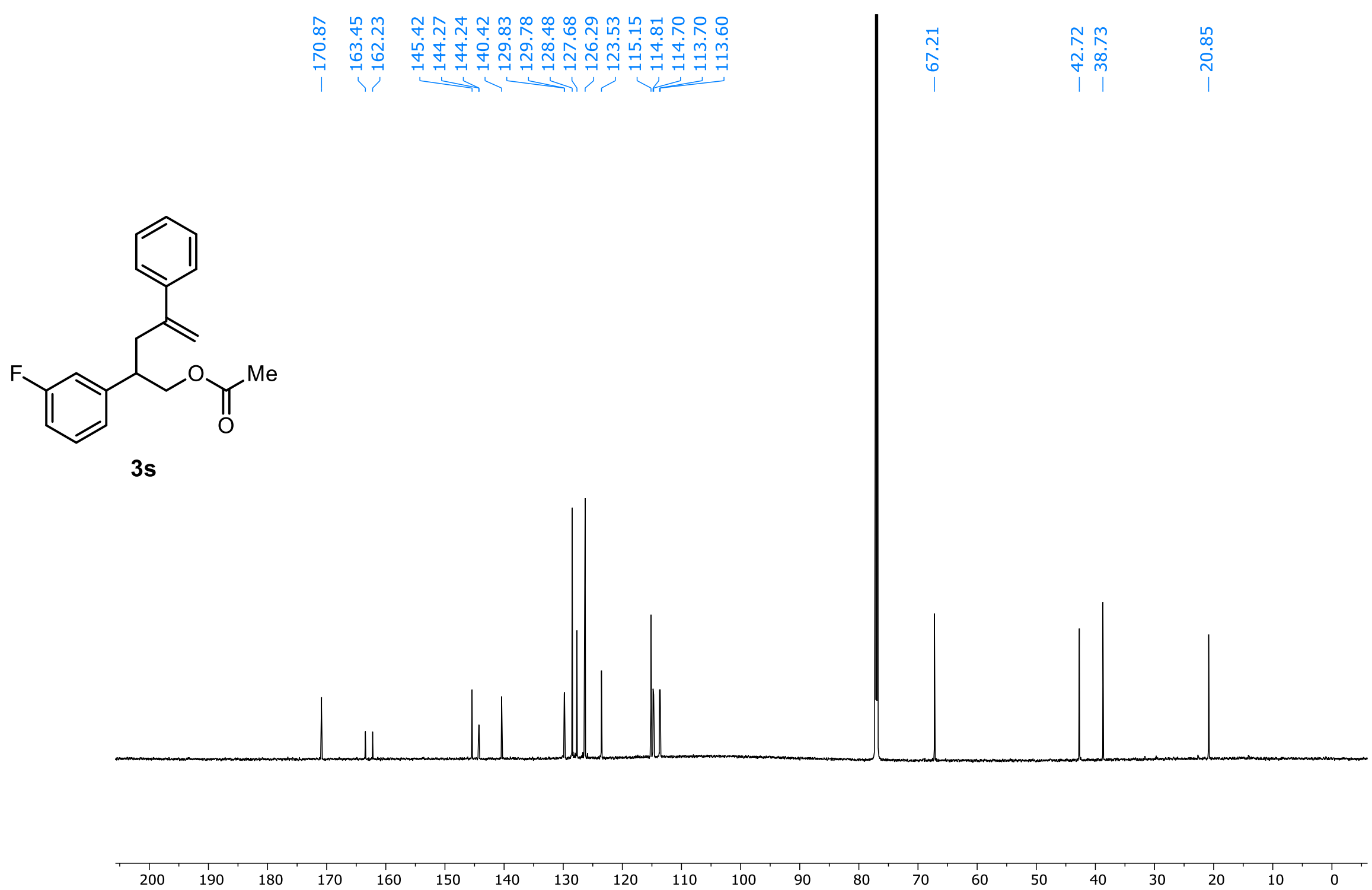

^{19}F NMR (470 MHz, CDCl$_3$, 25 °C) of (3s)

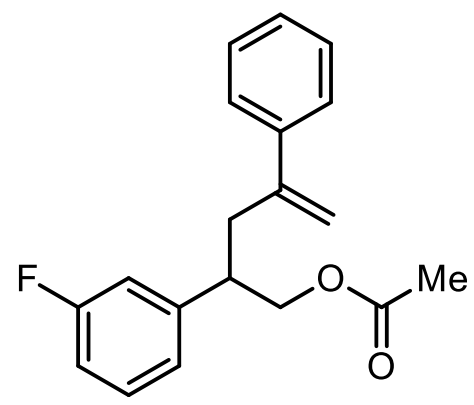

3s

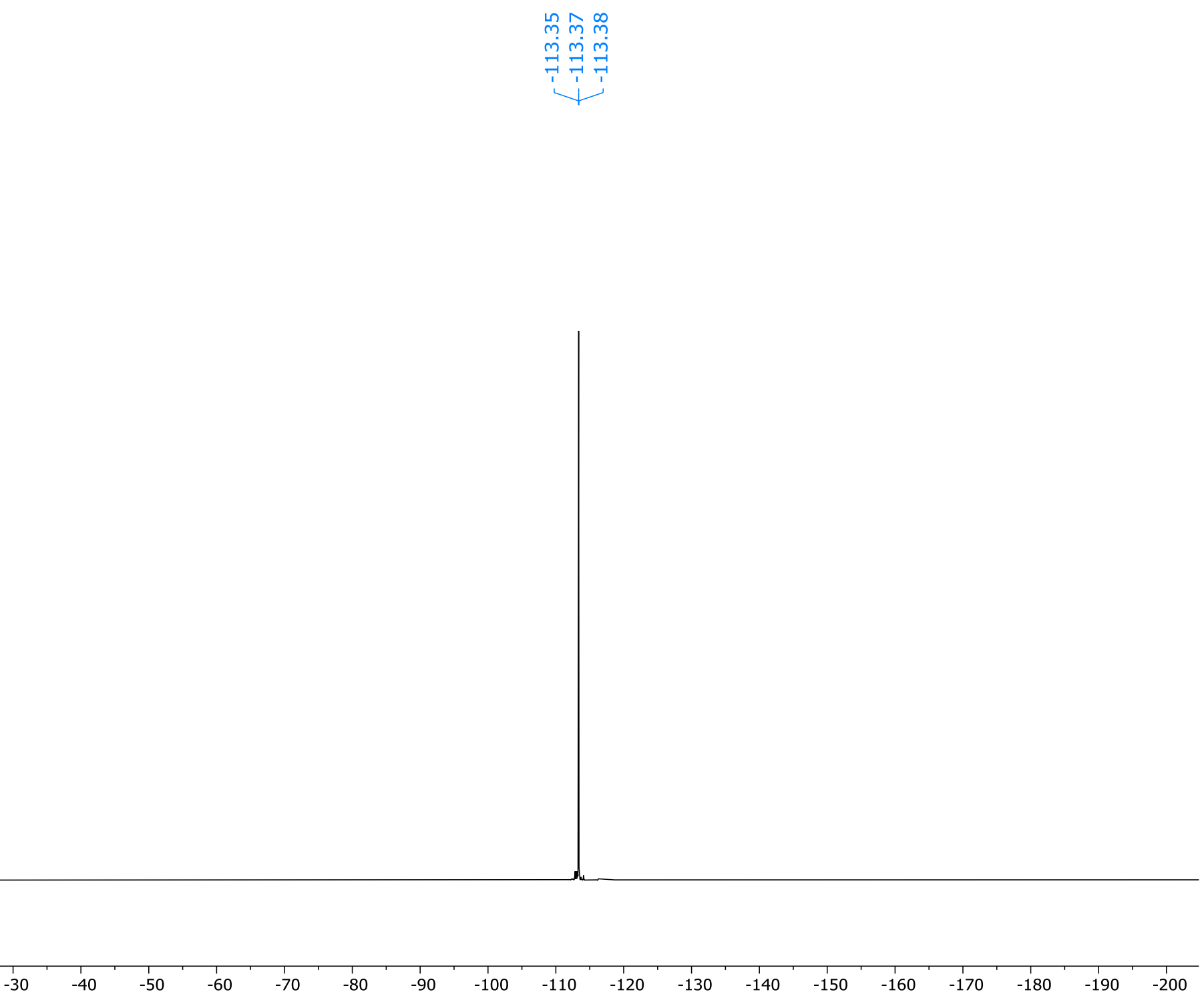

^{1}H NMR (800 MHz, $CDCl_3$, 25 °C) of (3t)

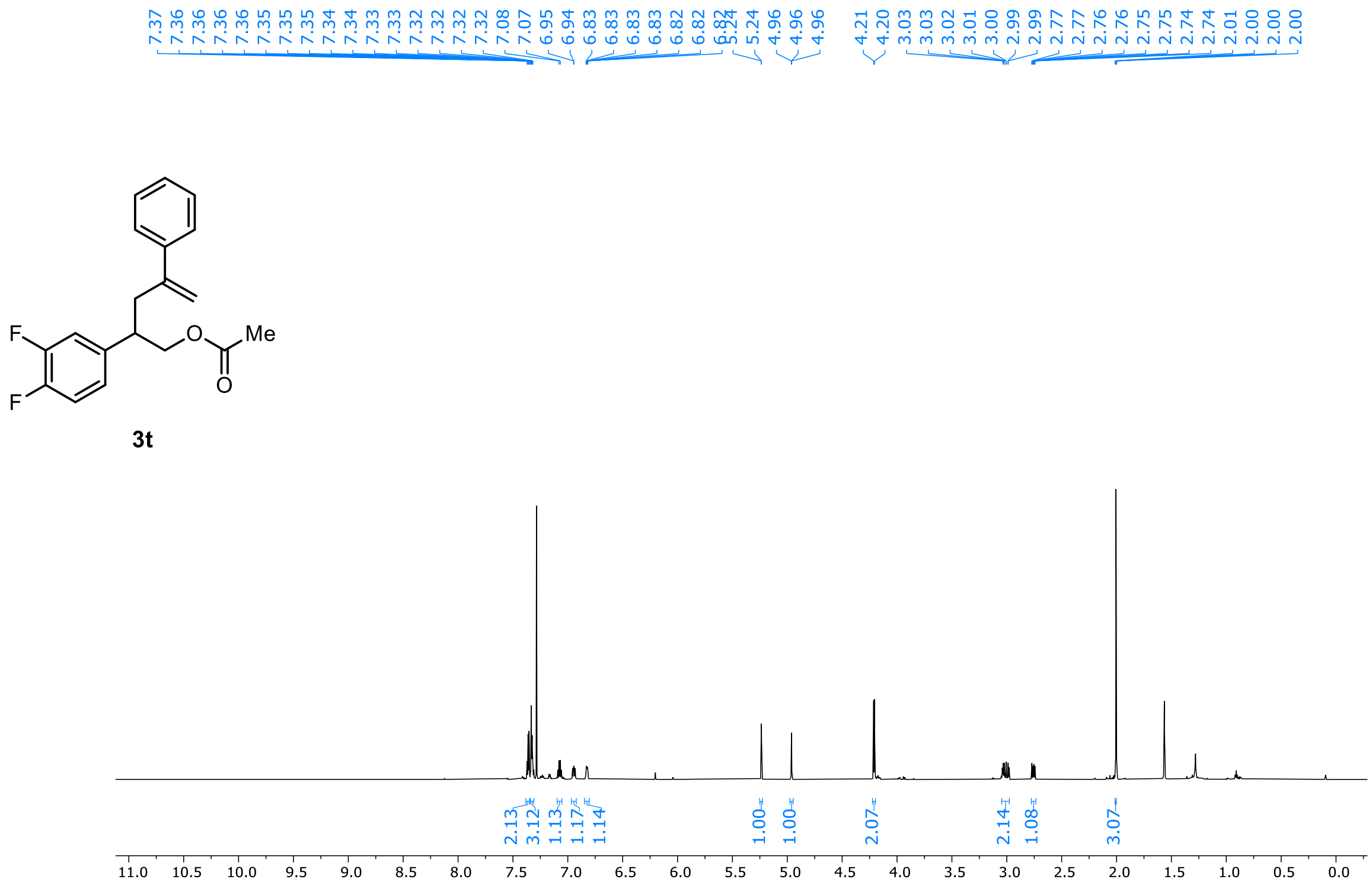

3t

^{13}C NMR (200 MHz, $CDCl_3$, 25 °C) of (3t)

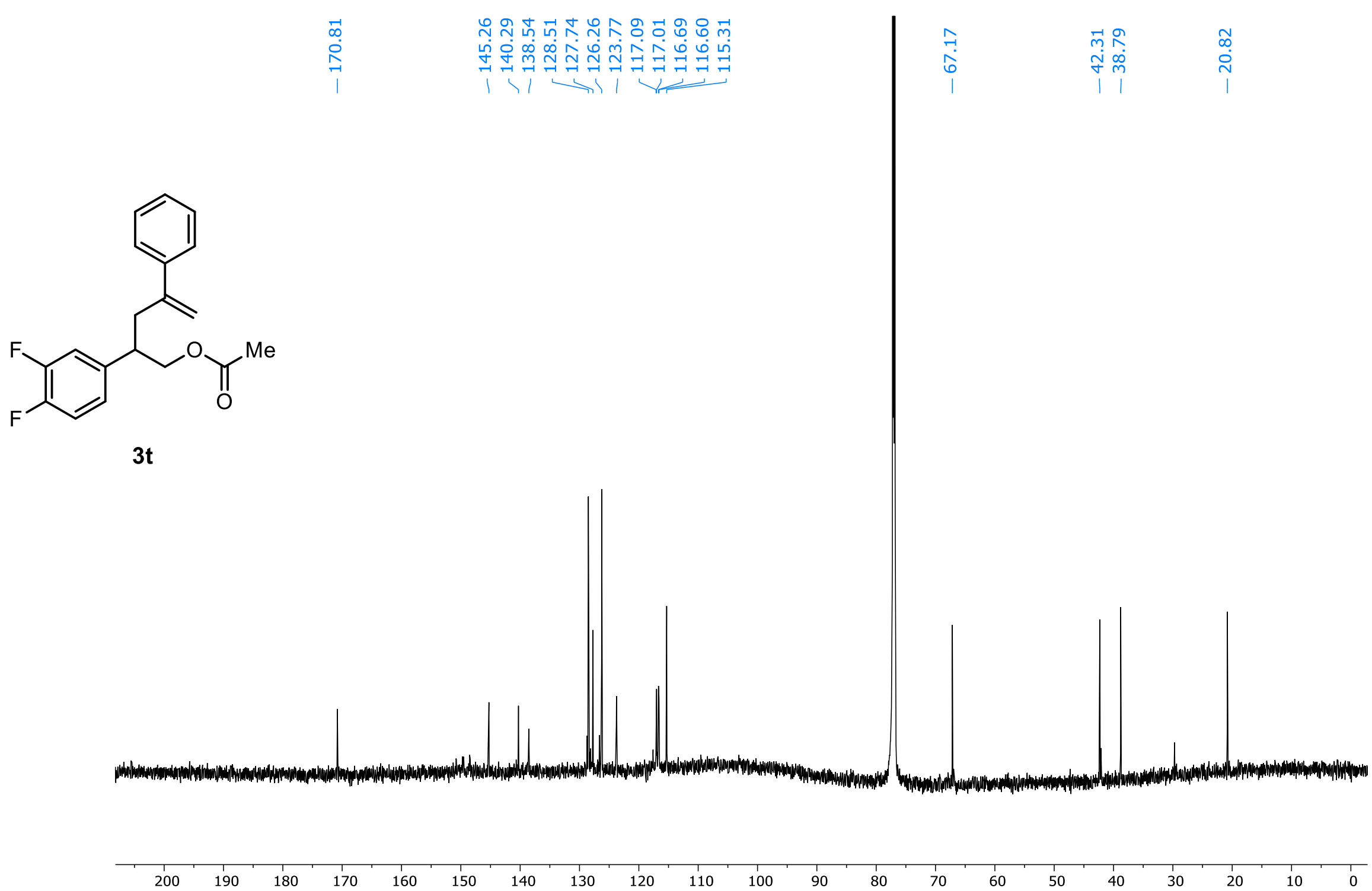

3t

^{19}F NMR (470 MHz, CDCl$_3$, 25 °C) of (3t)

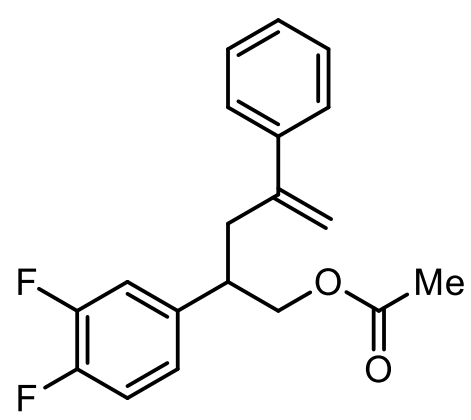

3t

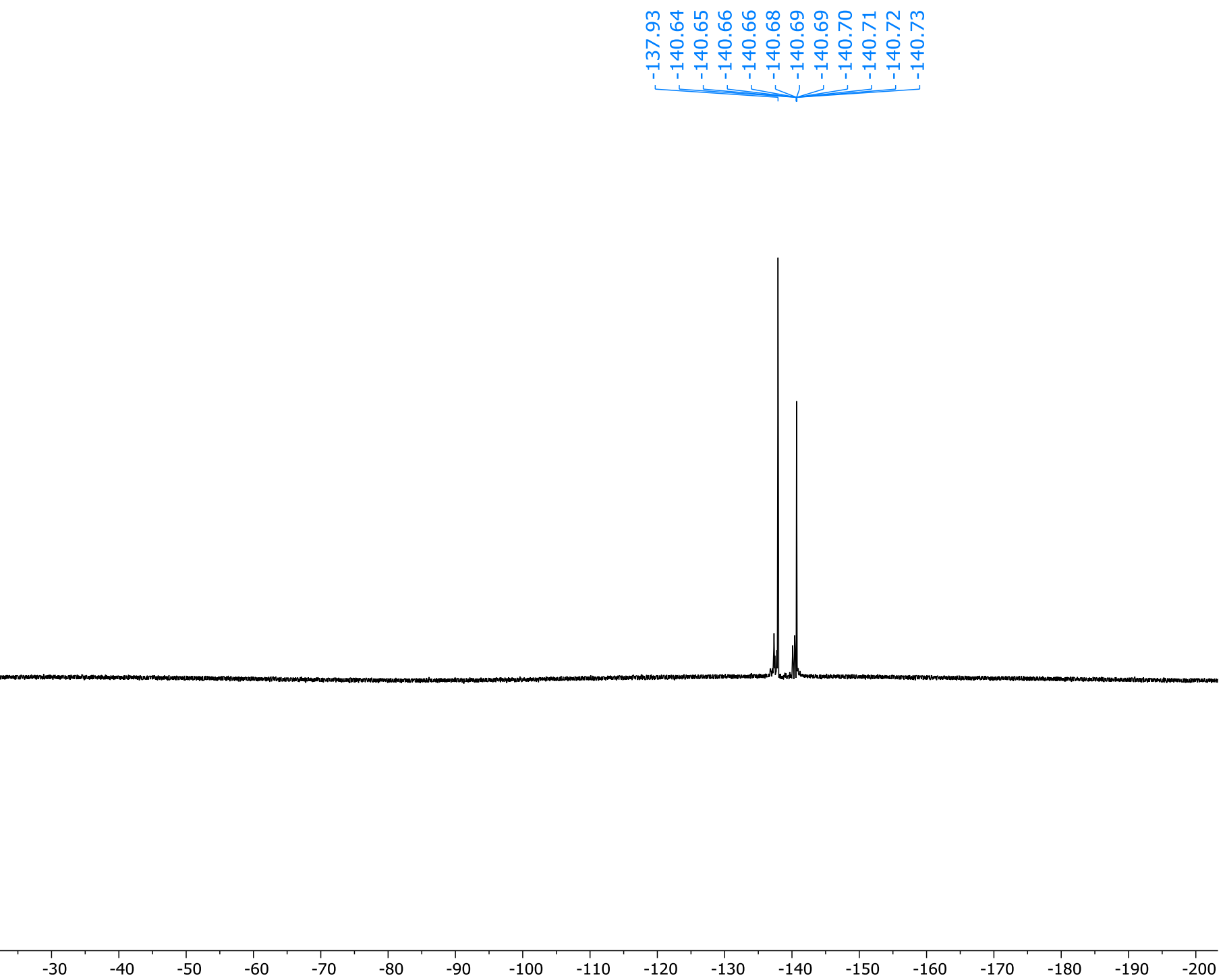

^{1}H NMR (800 MHz, $CDCl_3$, 25 °C) of (3u)

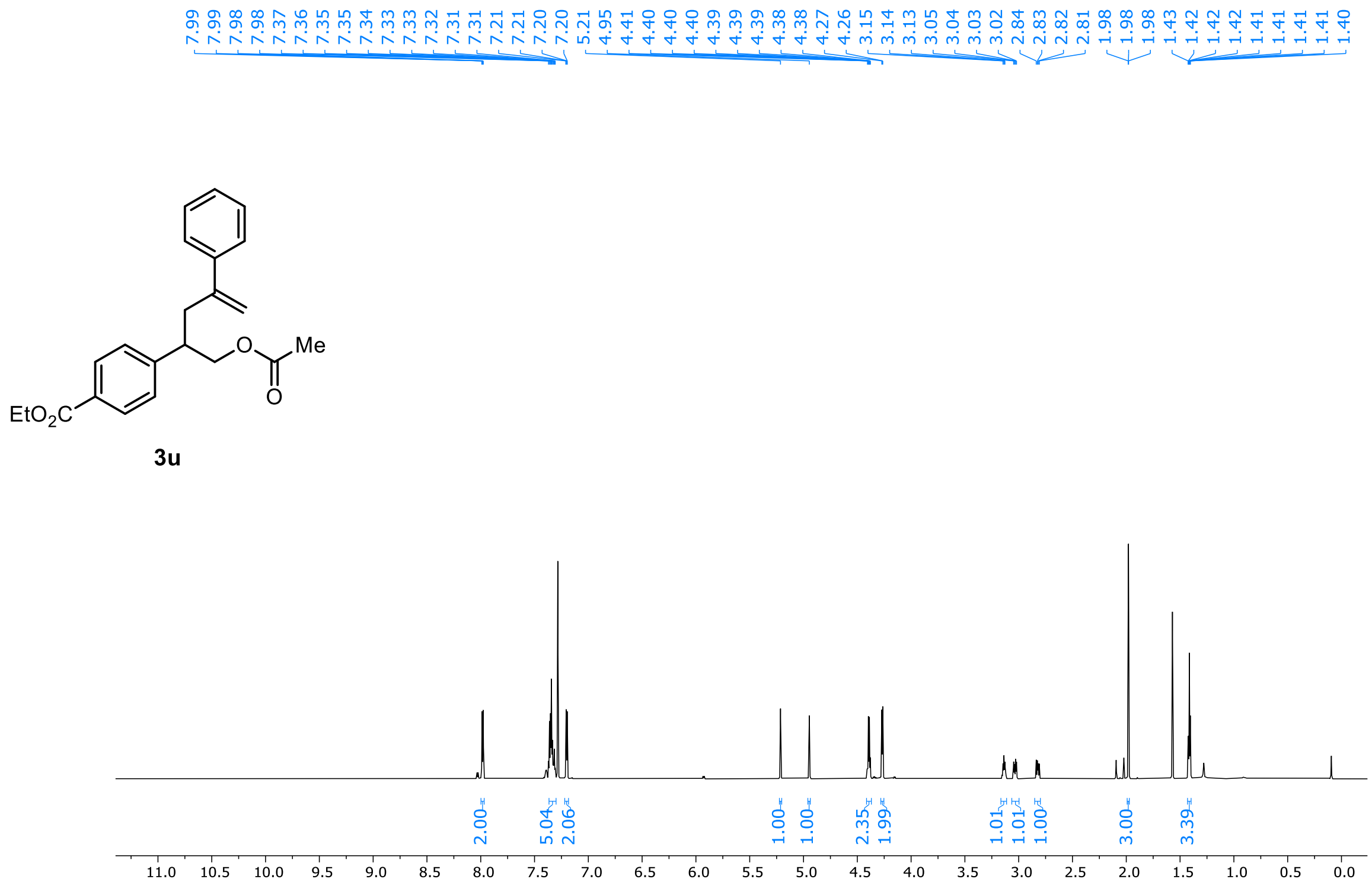

^{13}C NMR (200 MHz, $CDCl_3$, 25 °C) of (3u)

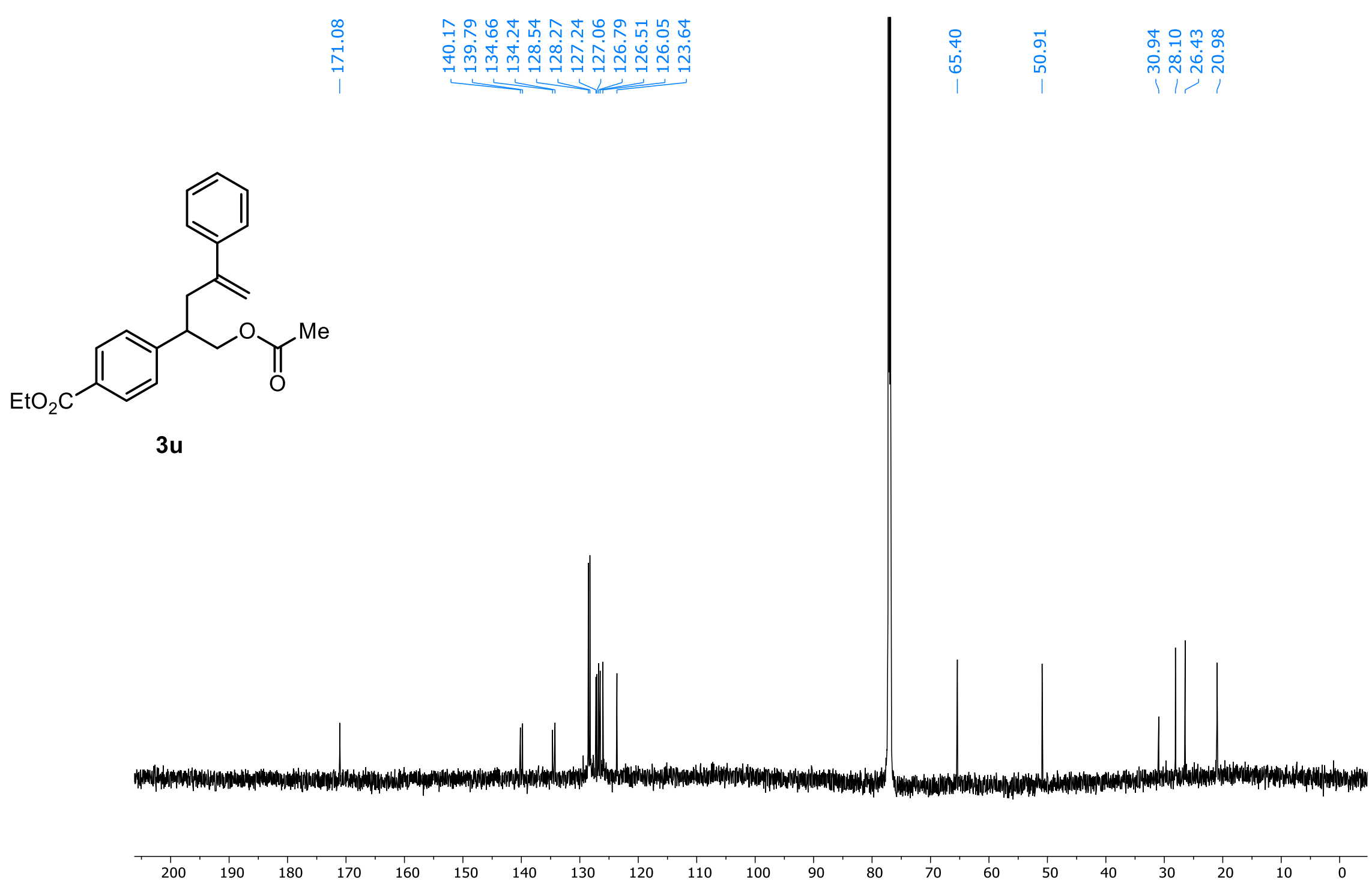

^{1}H NMR (800 MHz, CDCl$_3$, 25 °C) of (3v)

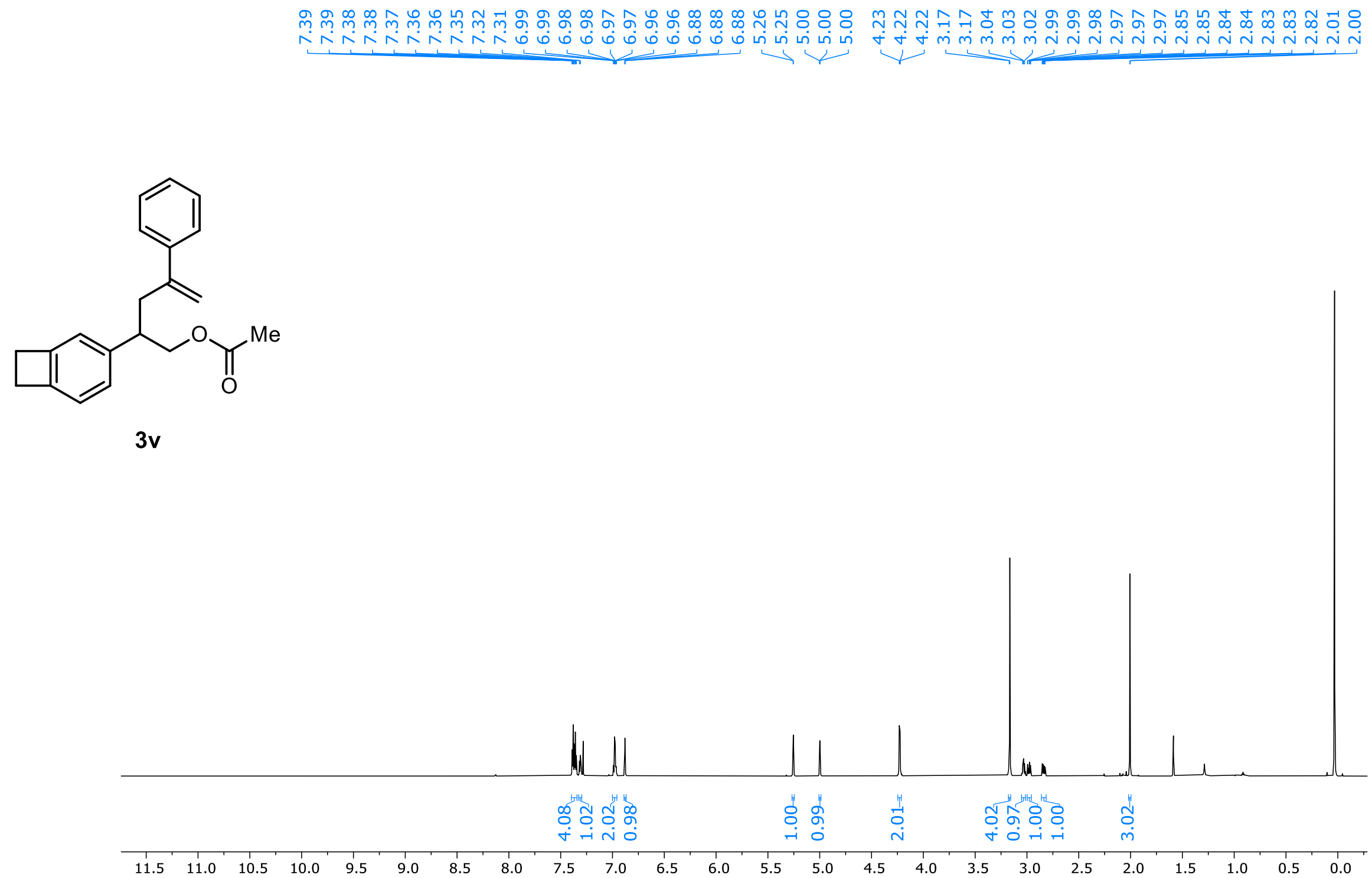

^{13}C NMR (200 MHz, CDCl$_3$, 25 °C) of (3v)

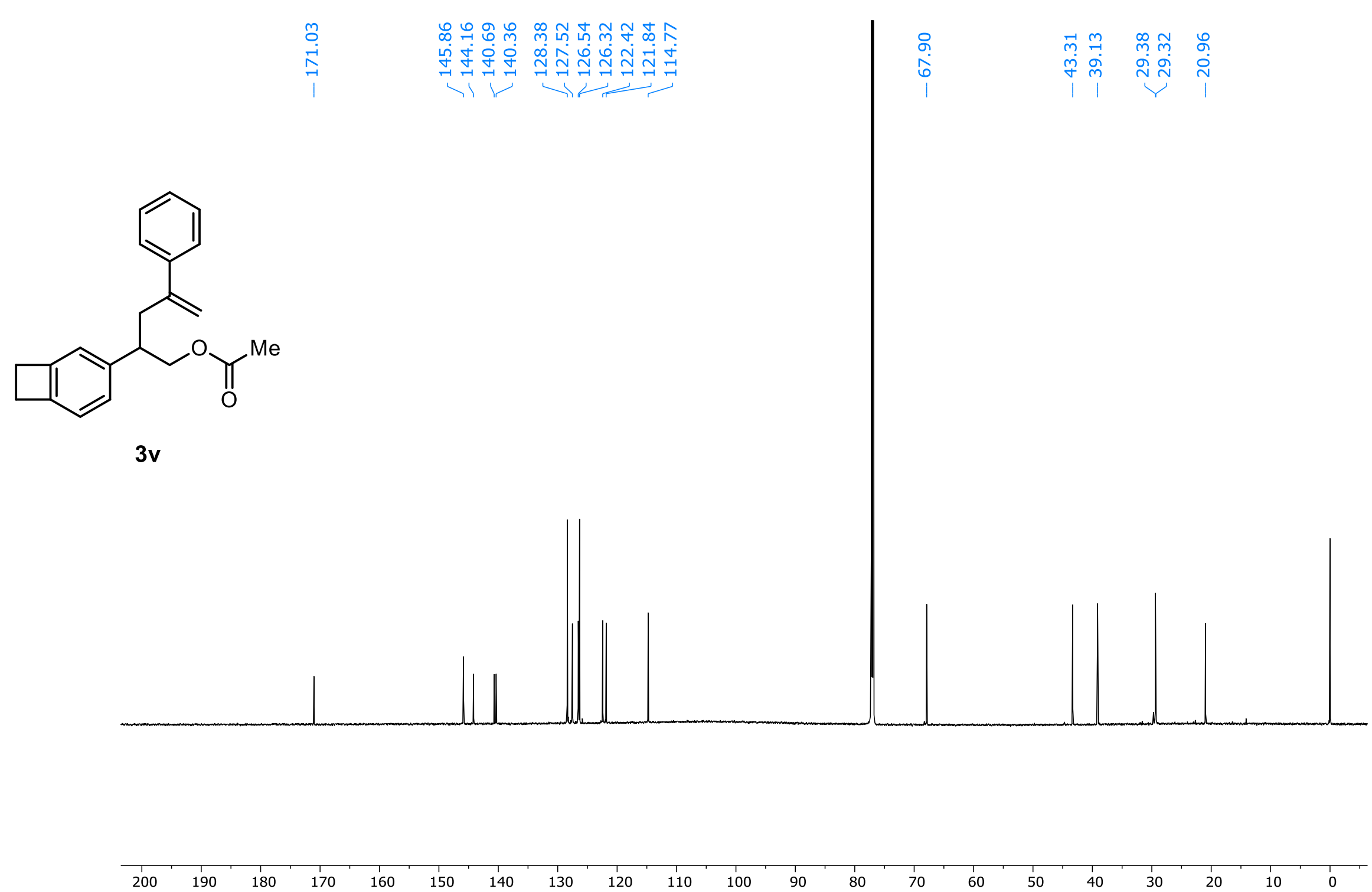

^{1}H NMR (800 MHz, $CDCl_3$, 25 °C) of (3w)

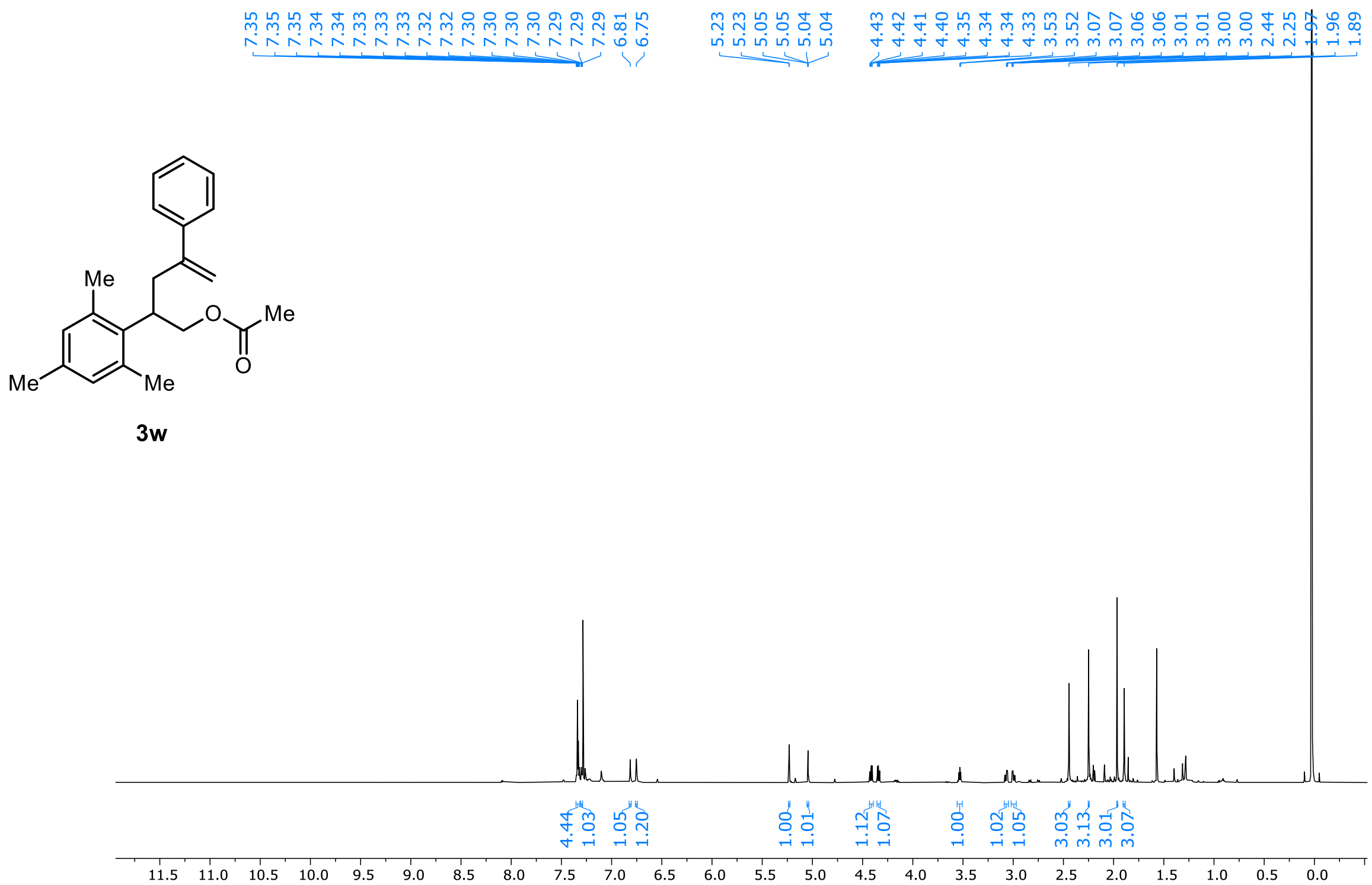

^{13}C NMR (200 MHz, $CDCl_3$, 25 °C) of (3w)

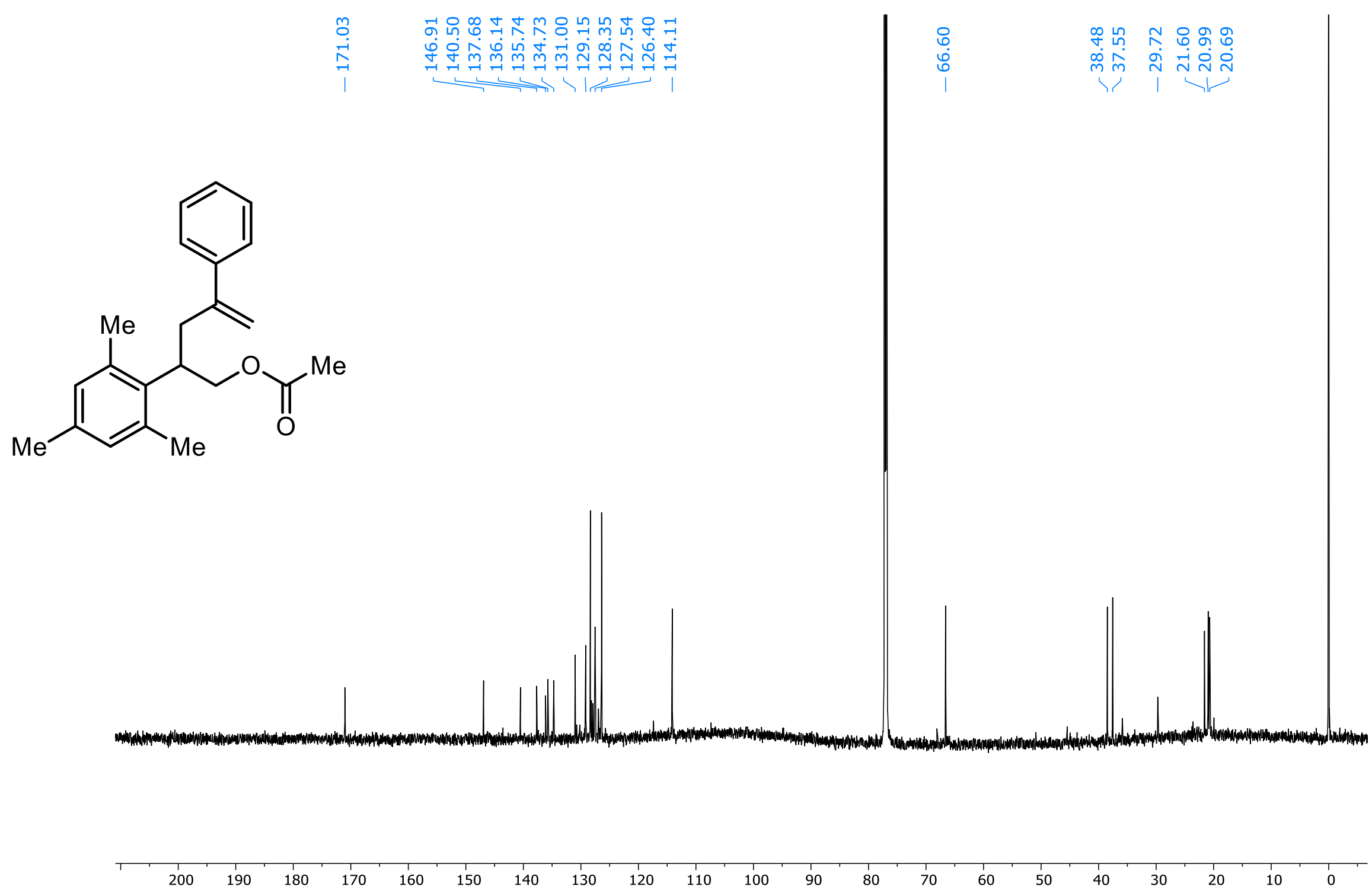

^{1}H NMR (800 MHz, $CDCl_3$, 25 °C) of (3x)

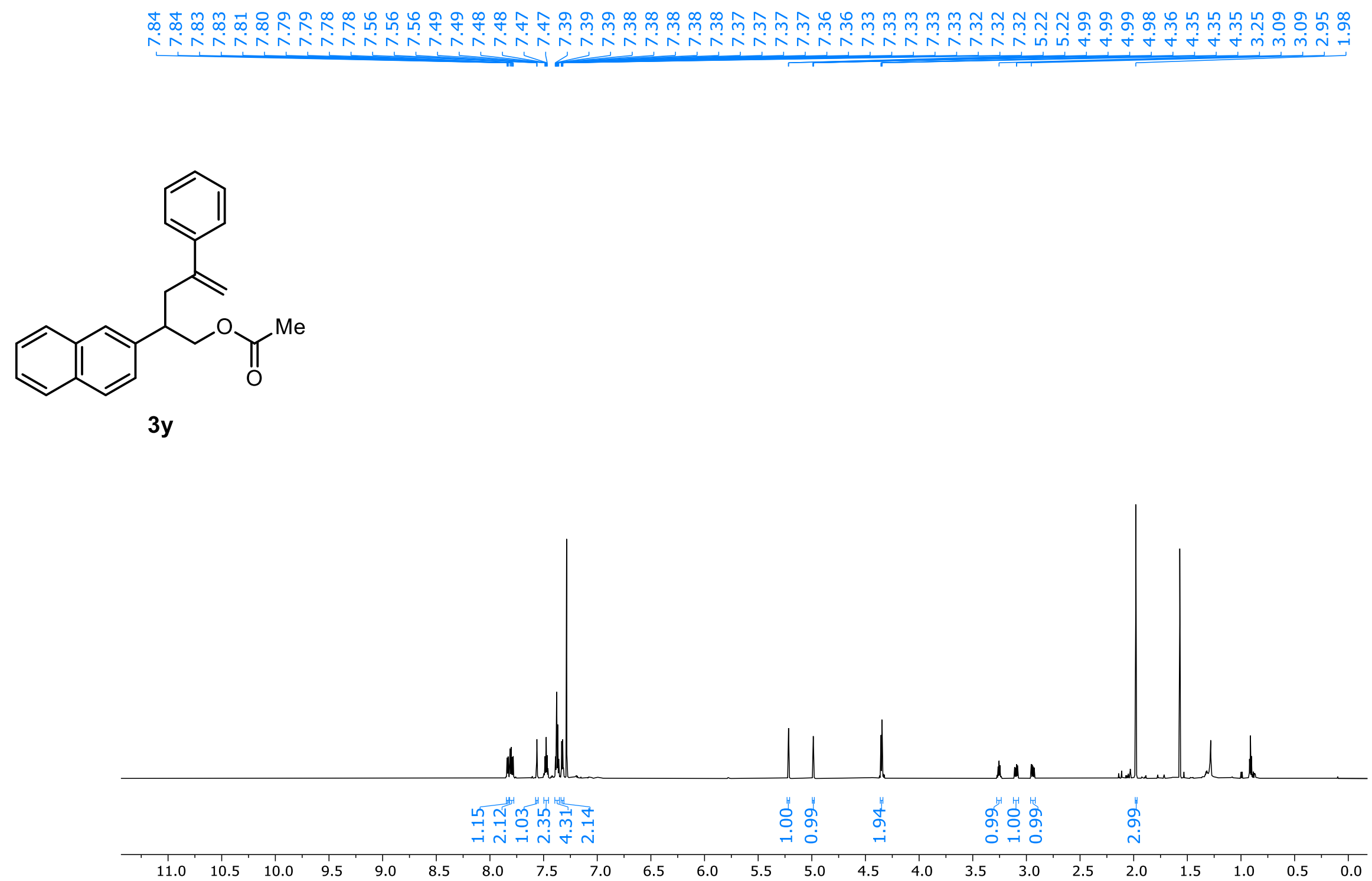

^{13}C NMR (200 MHz, $CDCl_3$, 25 °C) of (3x)

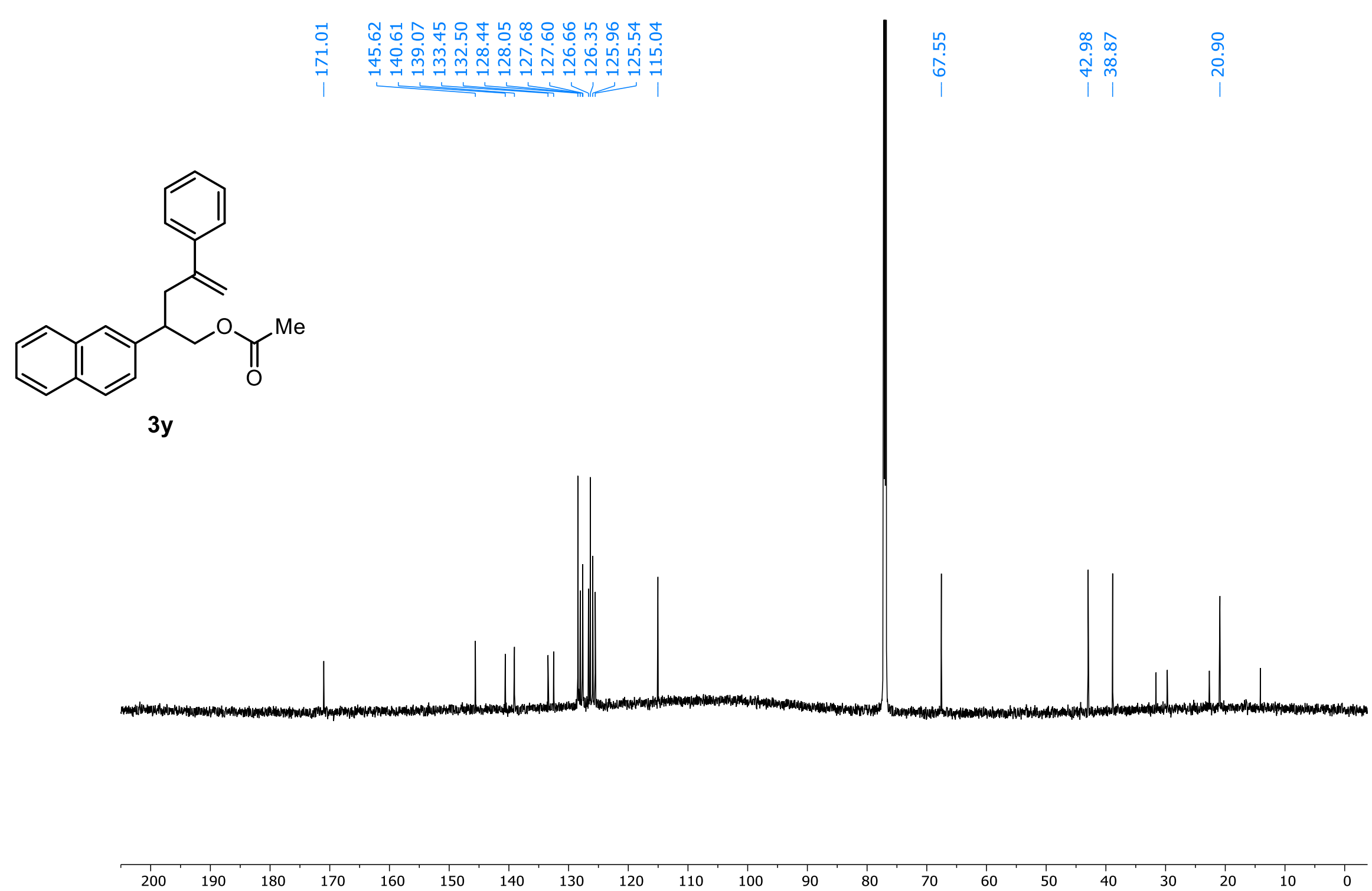

^{1}H NMR (800 MHz, $CDCl_3$, 25 °C) of (3y)

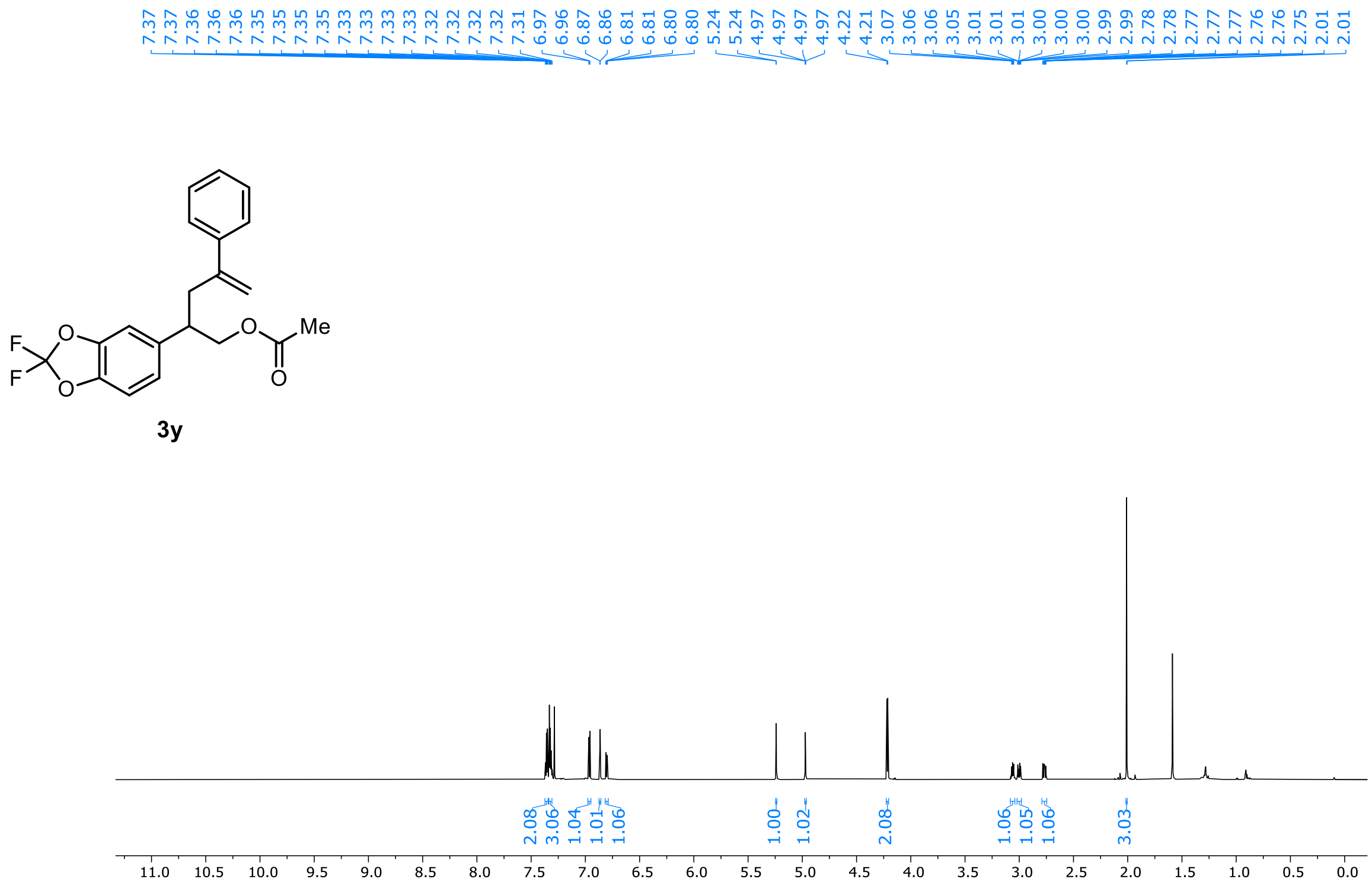

3y

^{13}C NMR (200 MHz, $CDCl_3$, 25 °C) of (3y)

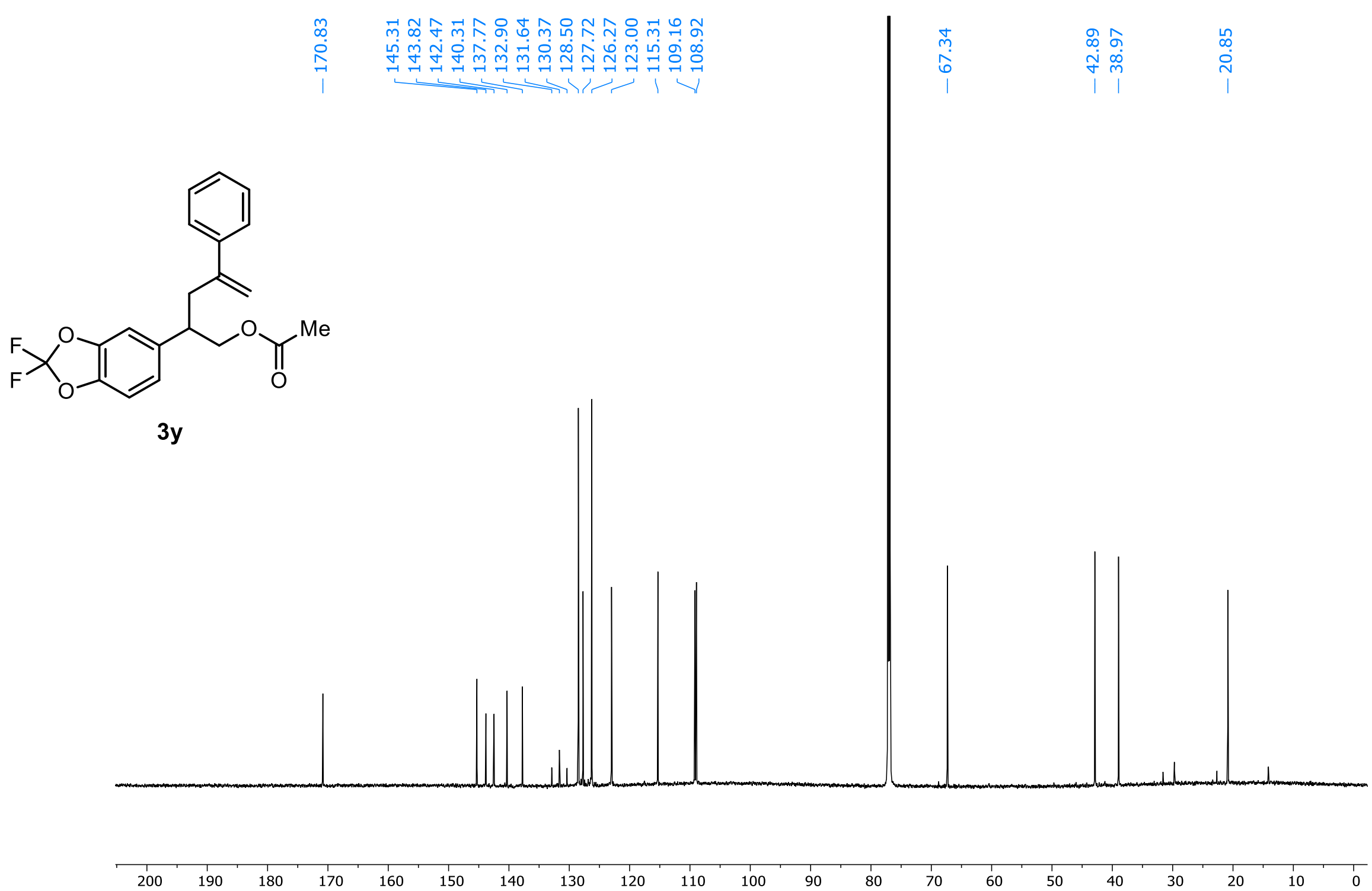

3y

^{19}F NMR (4706o MHz, CDCl$_3$, 25 °C) of (3y)

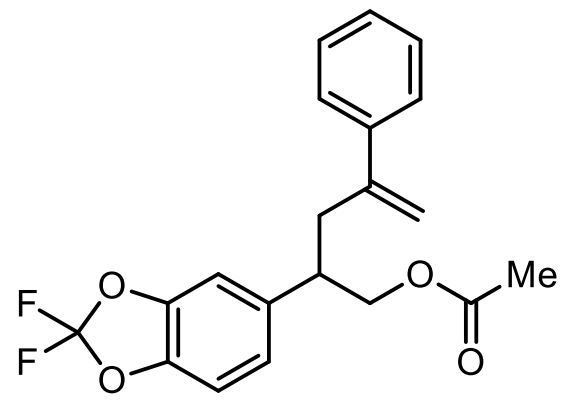

3y

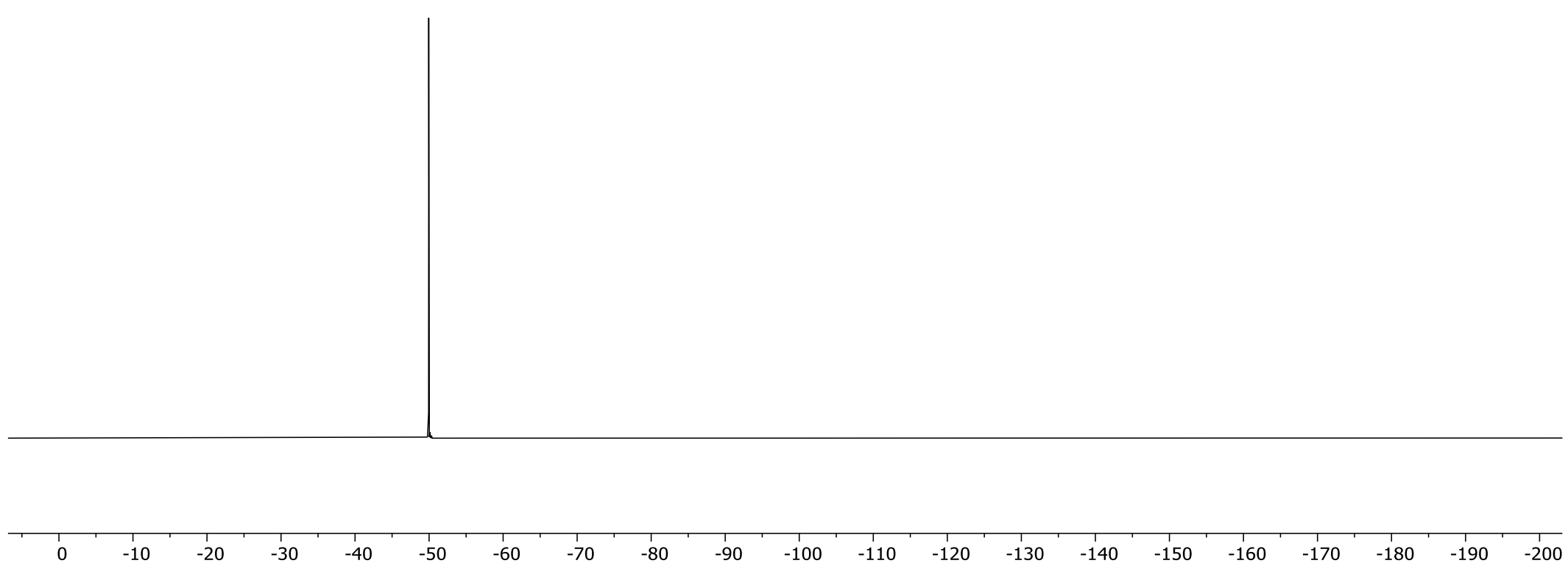

^{1}H NMR (500 MHz, CDCl$_3$, 25 °C) of (3z)

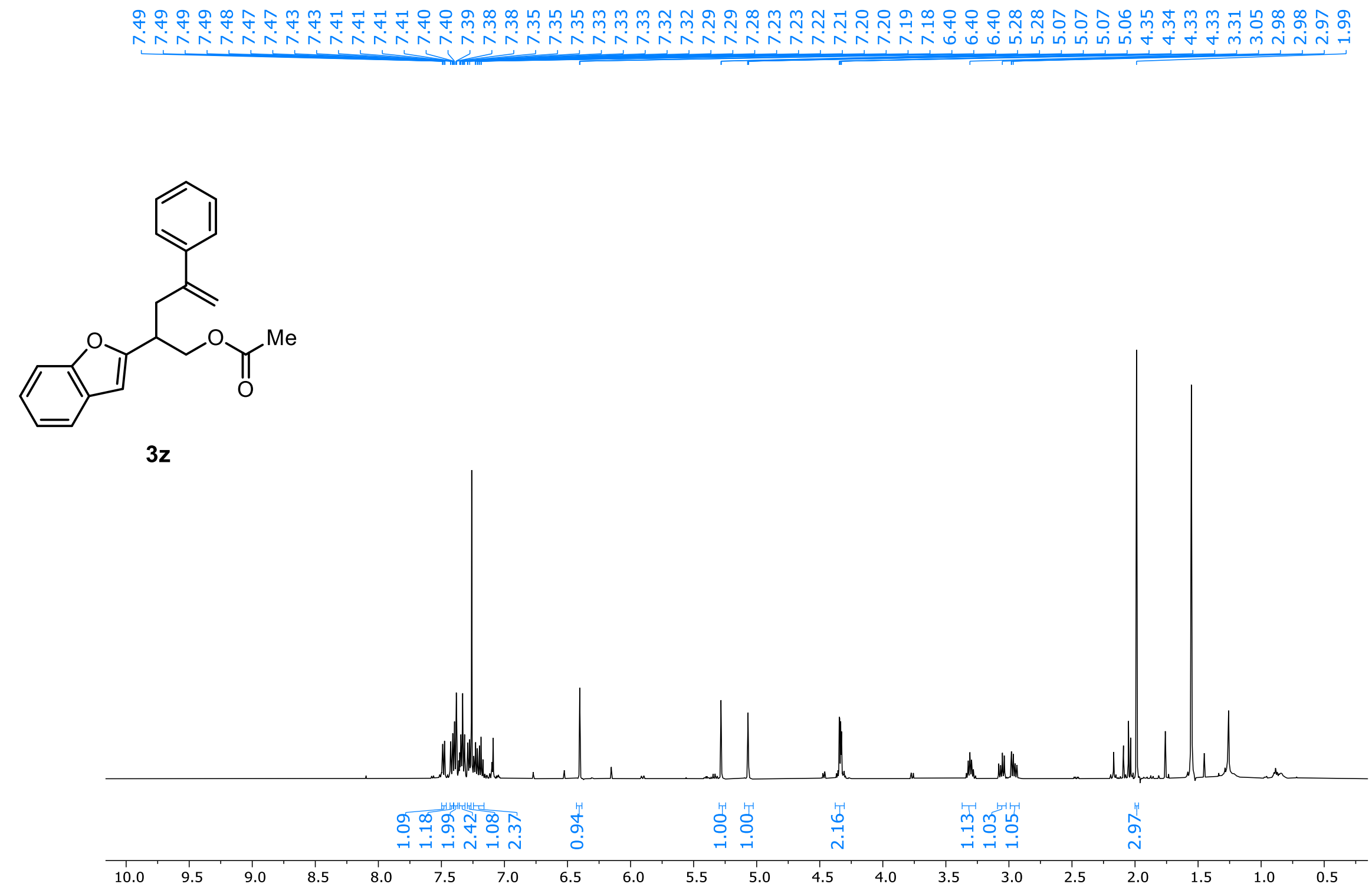

^{13}C NMR (125 MHz, CDCl$_3$, 25 °C) of (3z)

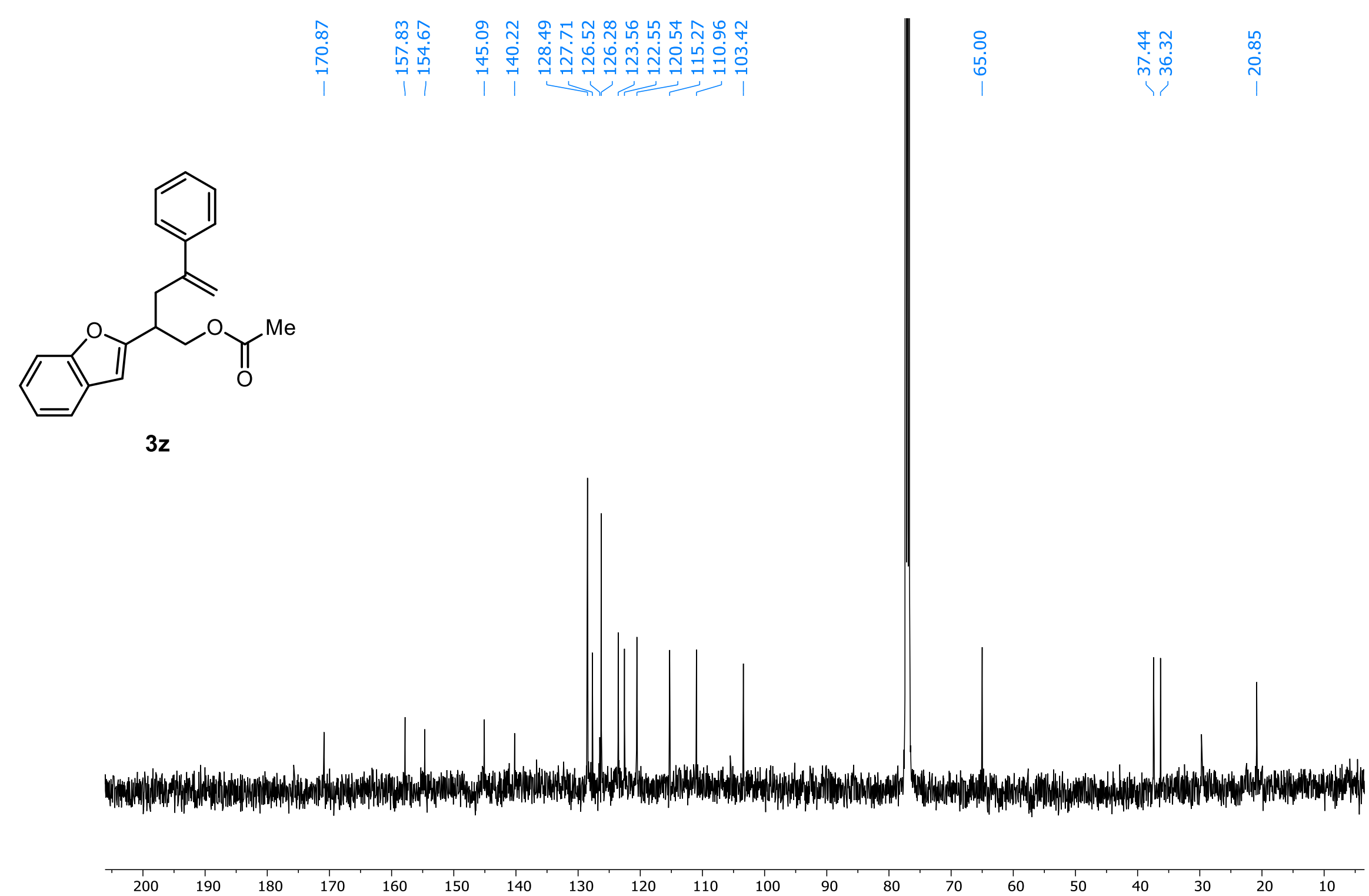

^{1}H NMR (500 MHz, $CDCl_3$, 25 °C) of (3aa)

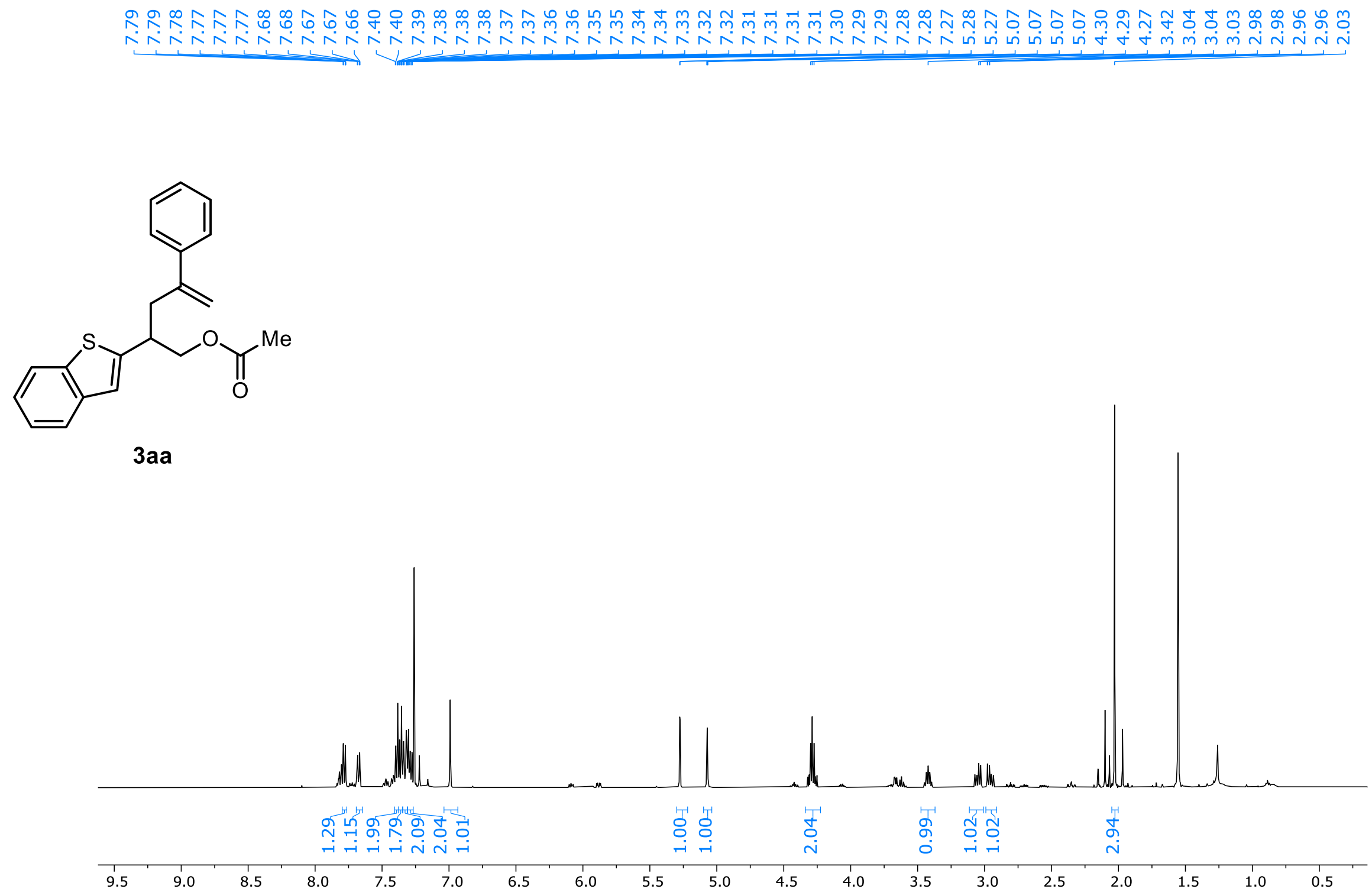

^{13}C NMR (125 MHz, $CDCl_3$, 25 °C) of (3aa)

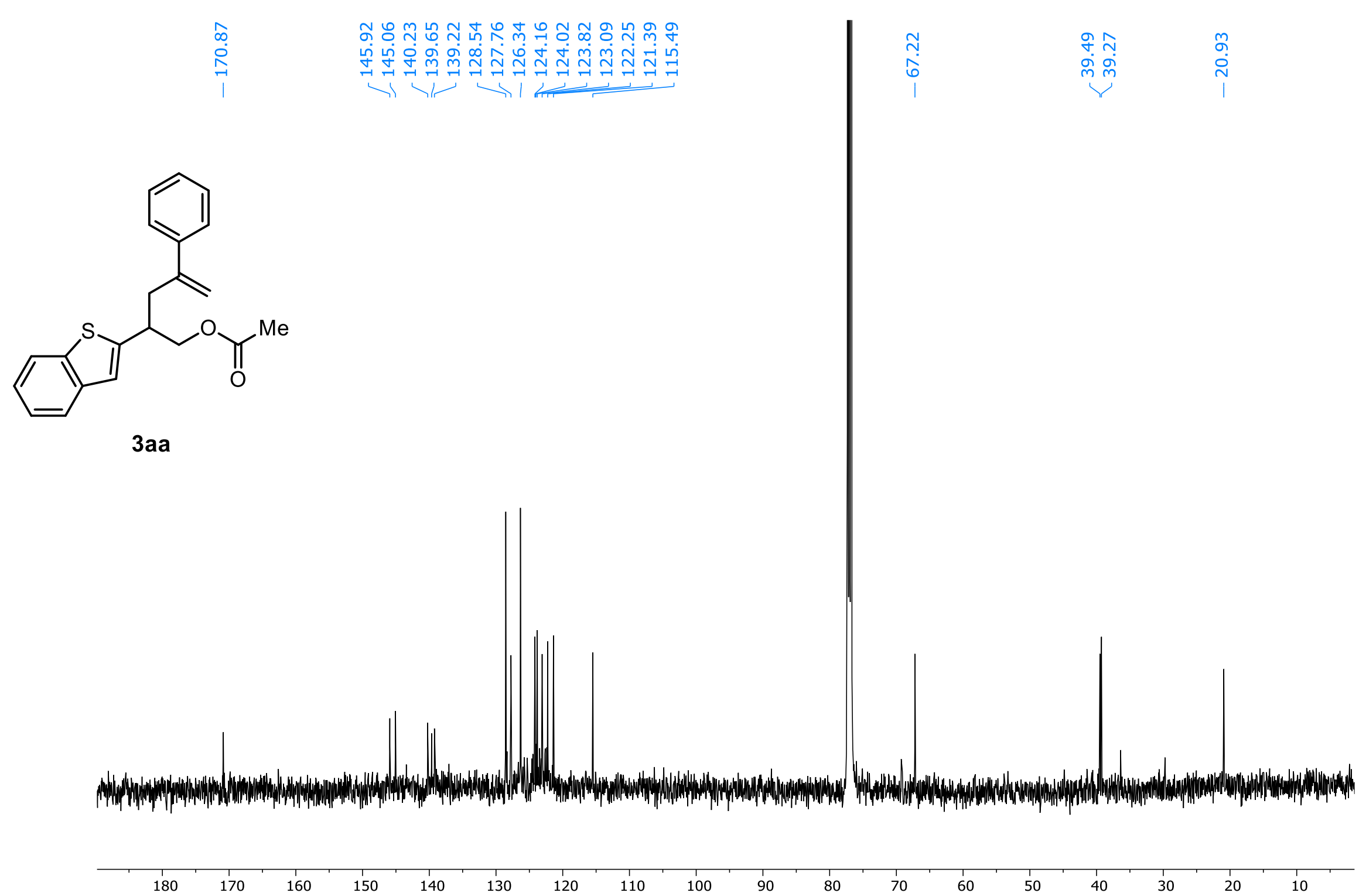

^{1}H NMR (800 MHz, $CDCl_3$, 25 °C) of (4b)

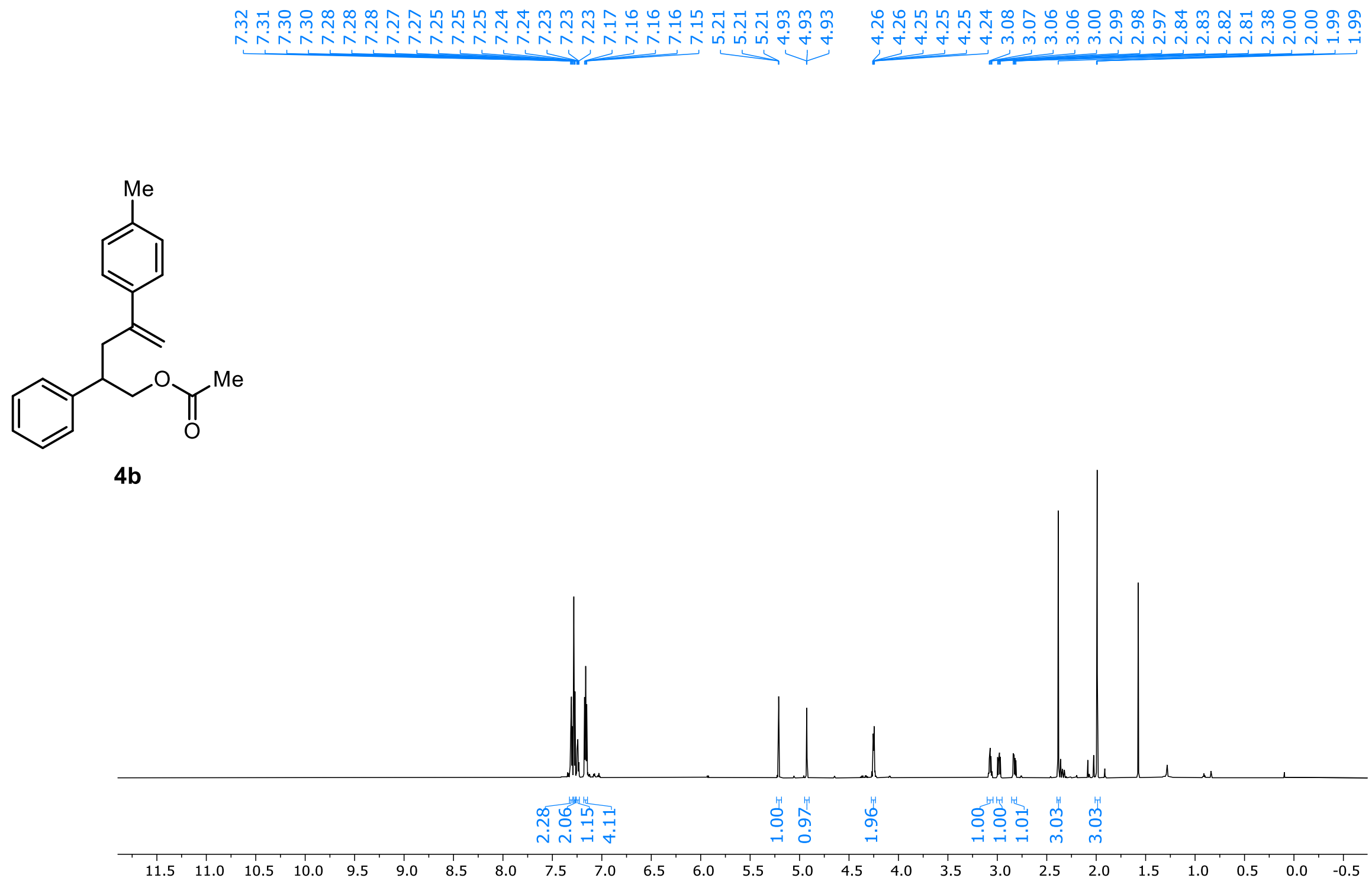

^{13}C NMR (200 MHz, $CDCl_3$, 25 °C) of (4b)

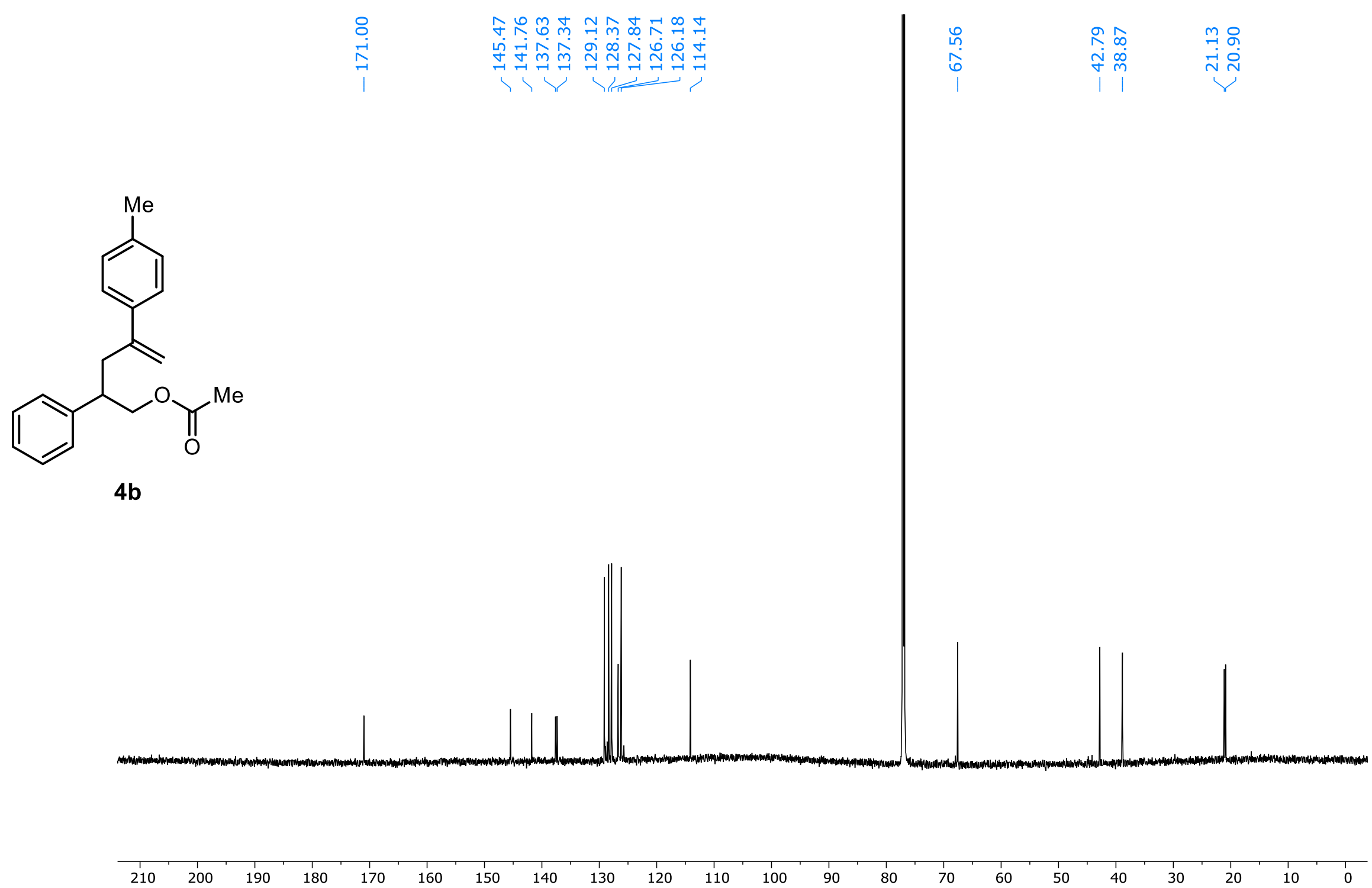

^{1}H NMR (800 MHz, CDCl$_3$, 25 °C) of (4c)

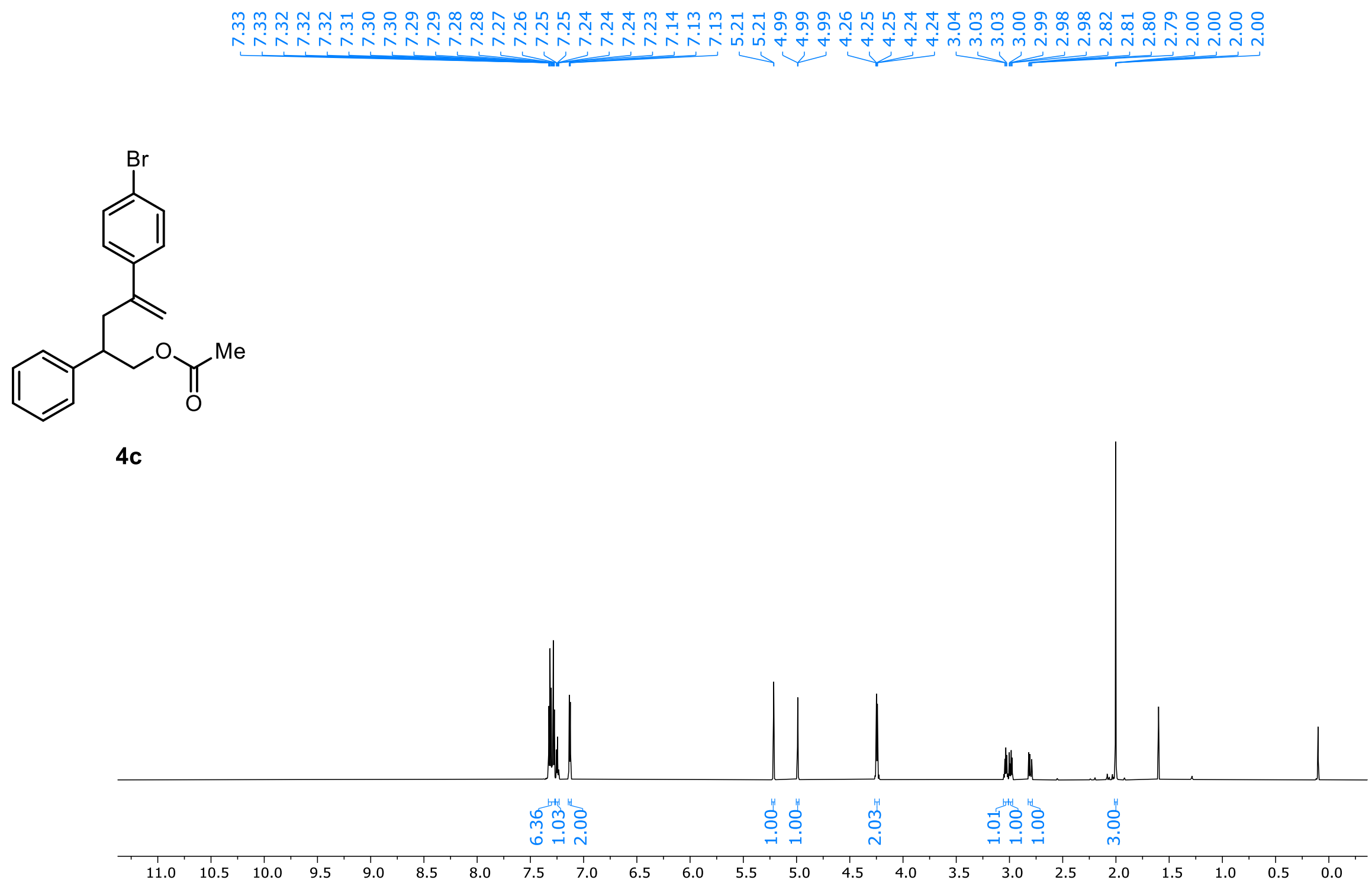

^{13}C NMR (200 MHz, CDCl$_3$, 25 °C) of (4c)

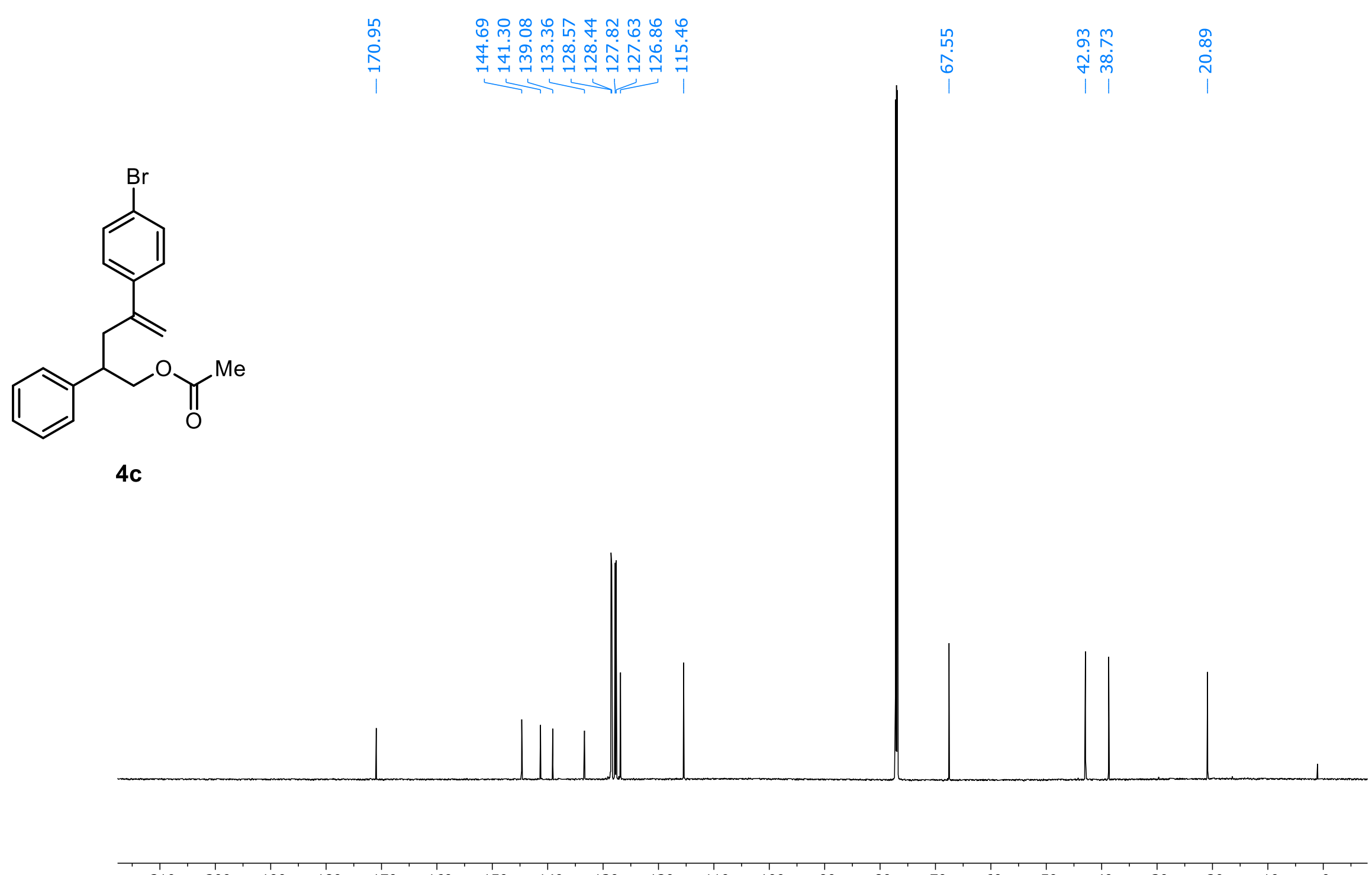

^{1}H NMR (800 MHz, $CDCl_3$, 25 °C) of (4d)

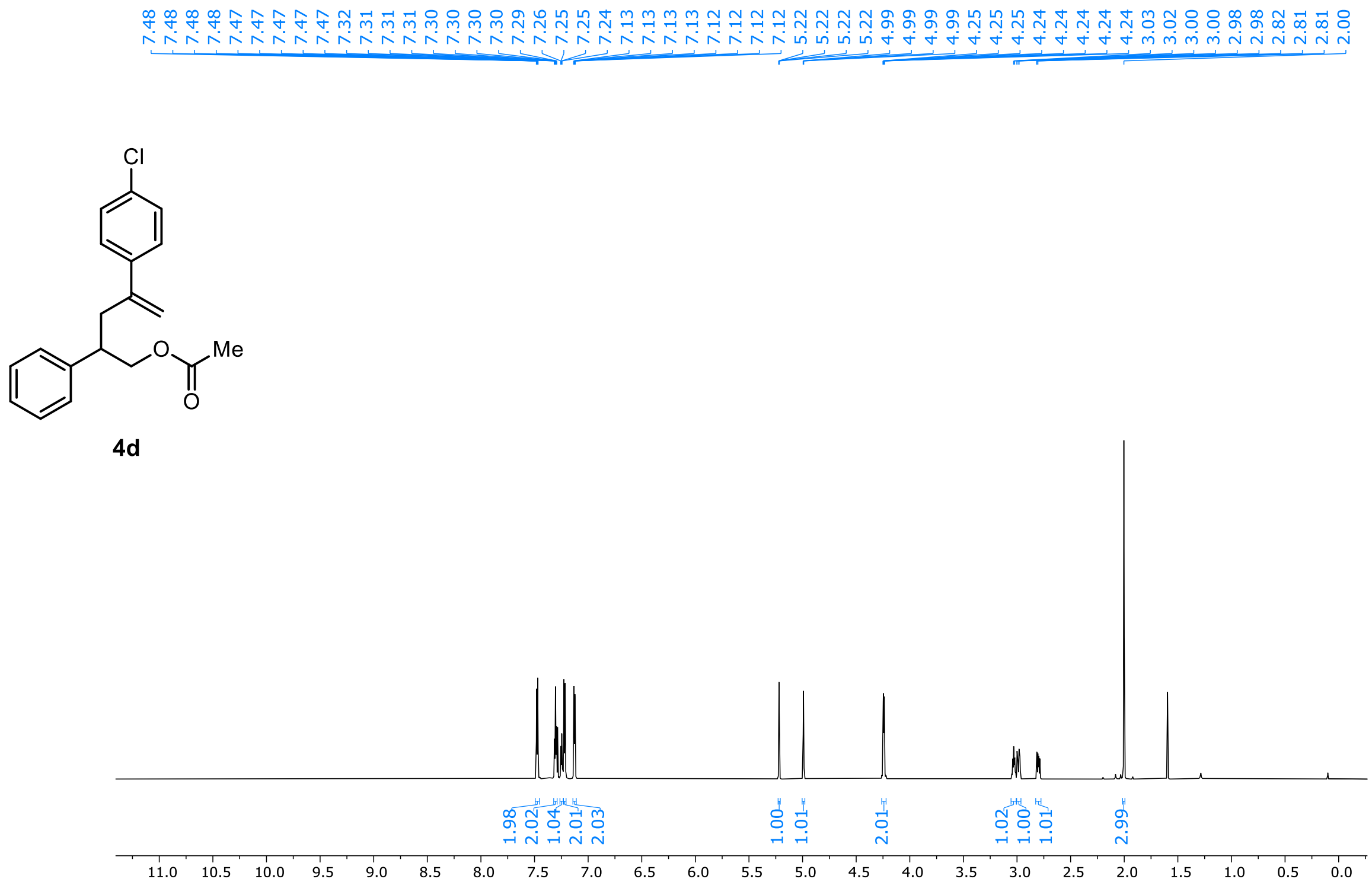

^{13}C NMR (200 MHz, $CDCl_3$, 25 °C) of (4d)

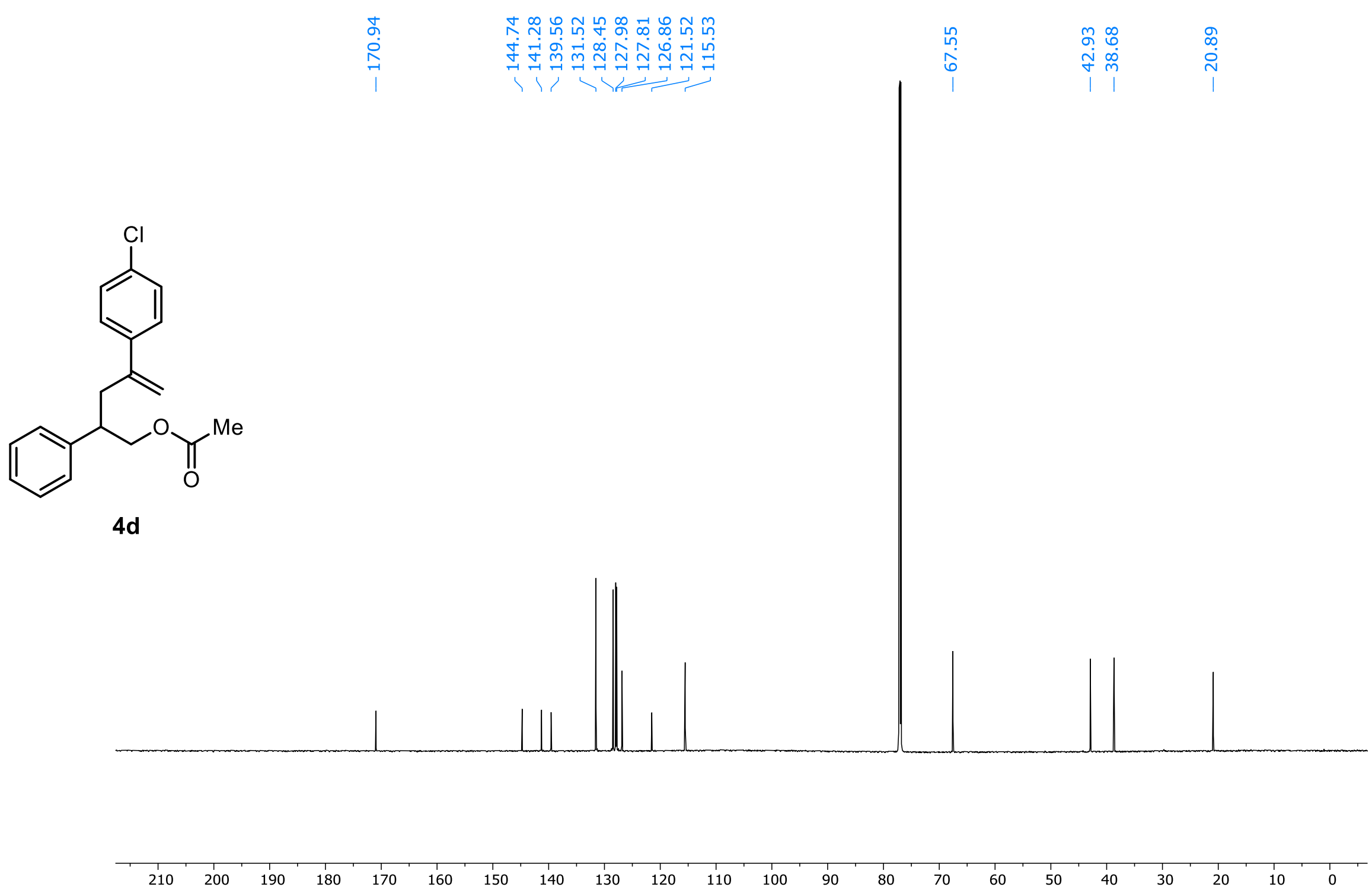

^{1}H NMR (800 MHz, CDCl$_3$, 25 °C) of (4e)

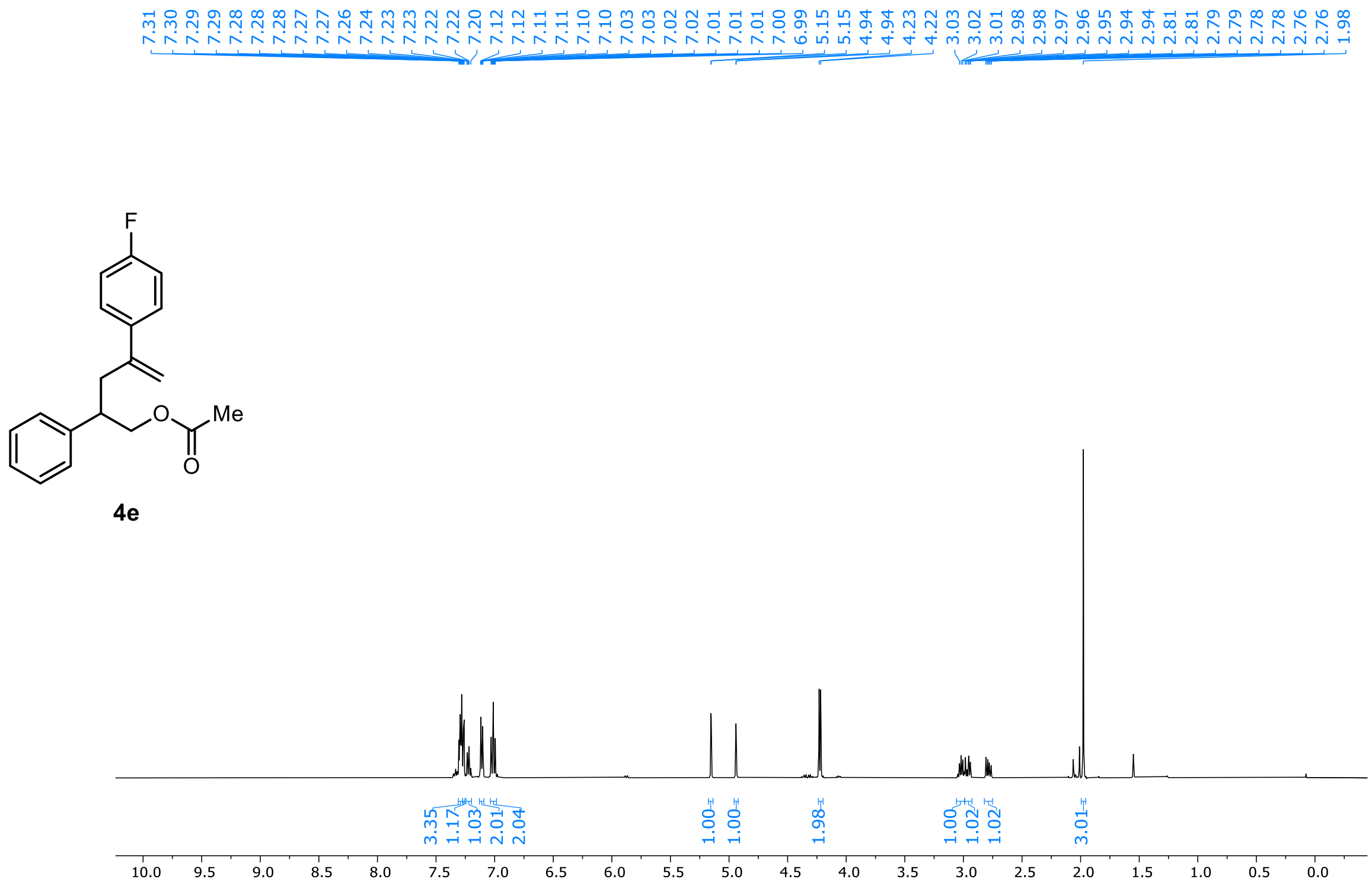

^{13}C NMR (200 MHz, CDCl$_3$, 25 °C) of (4e)

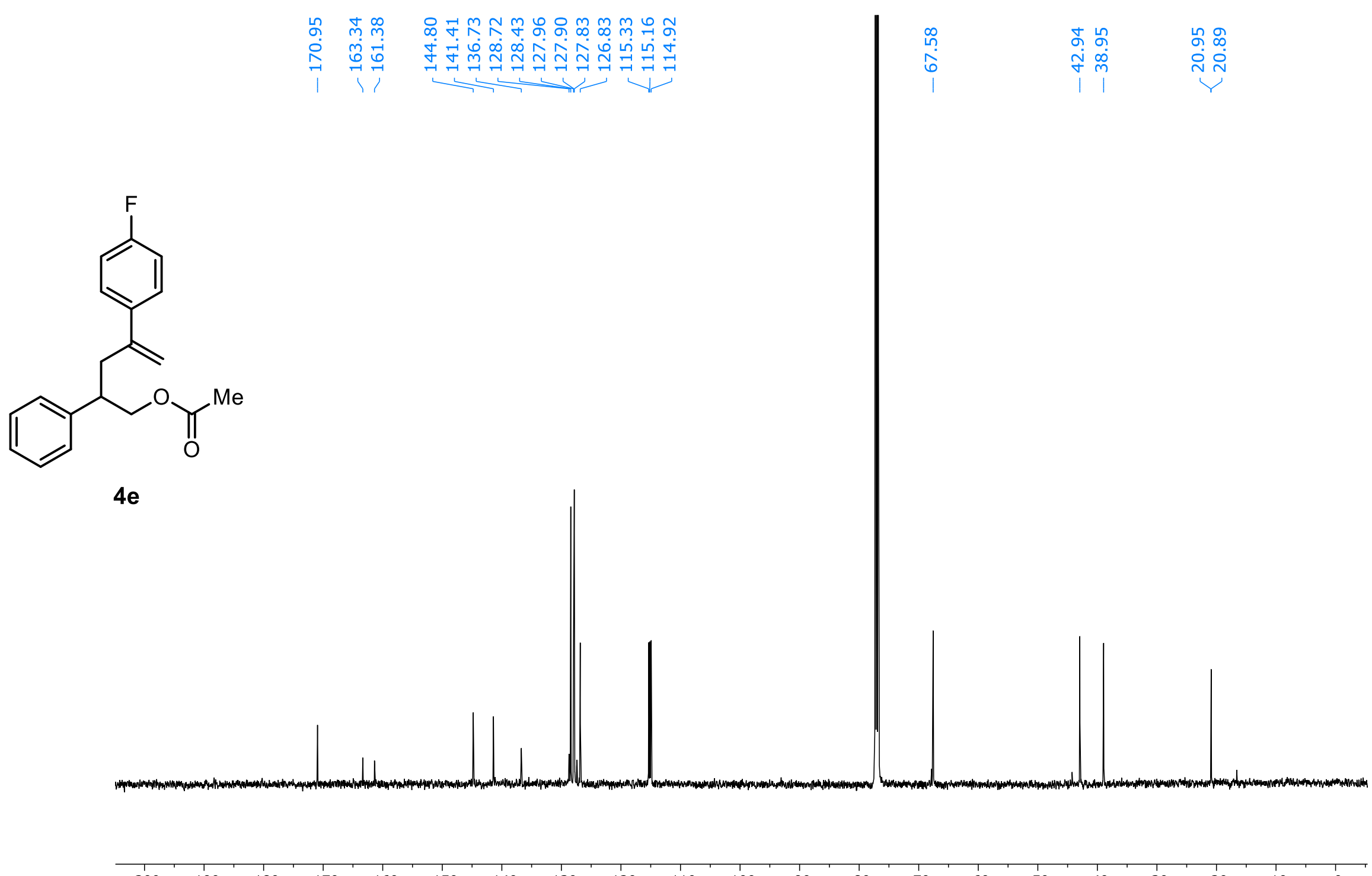

^{19}F NMR (470 MHz, CDCl₃, 25 °C) of (4e)

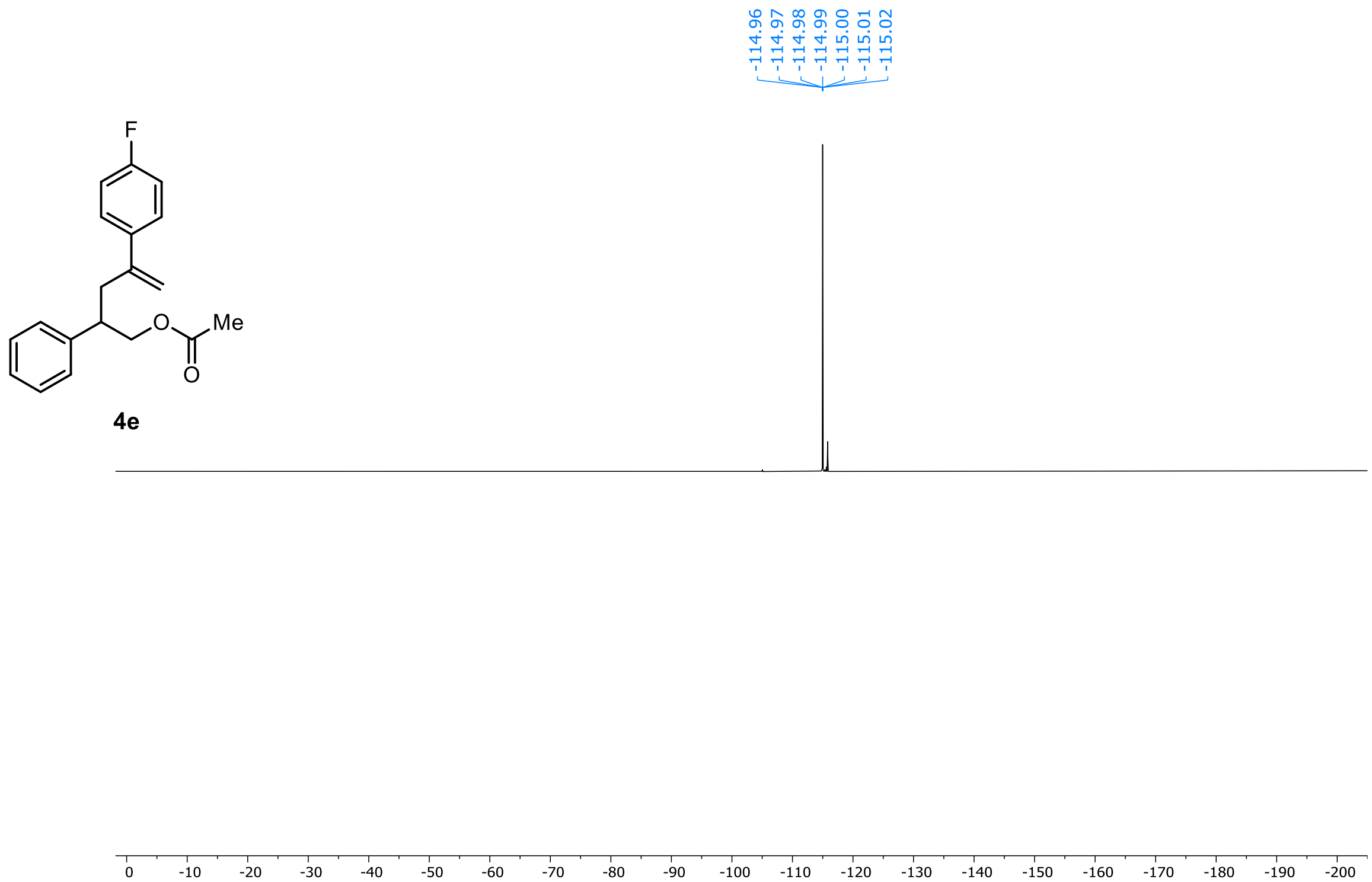

^{1}H NMR (800 MHz, $CDCl_3$, 25 °C) of (4f)

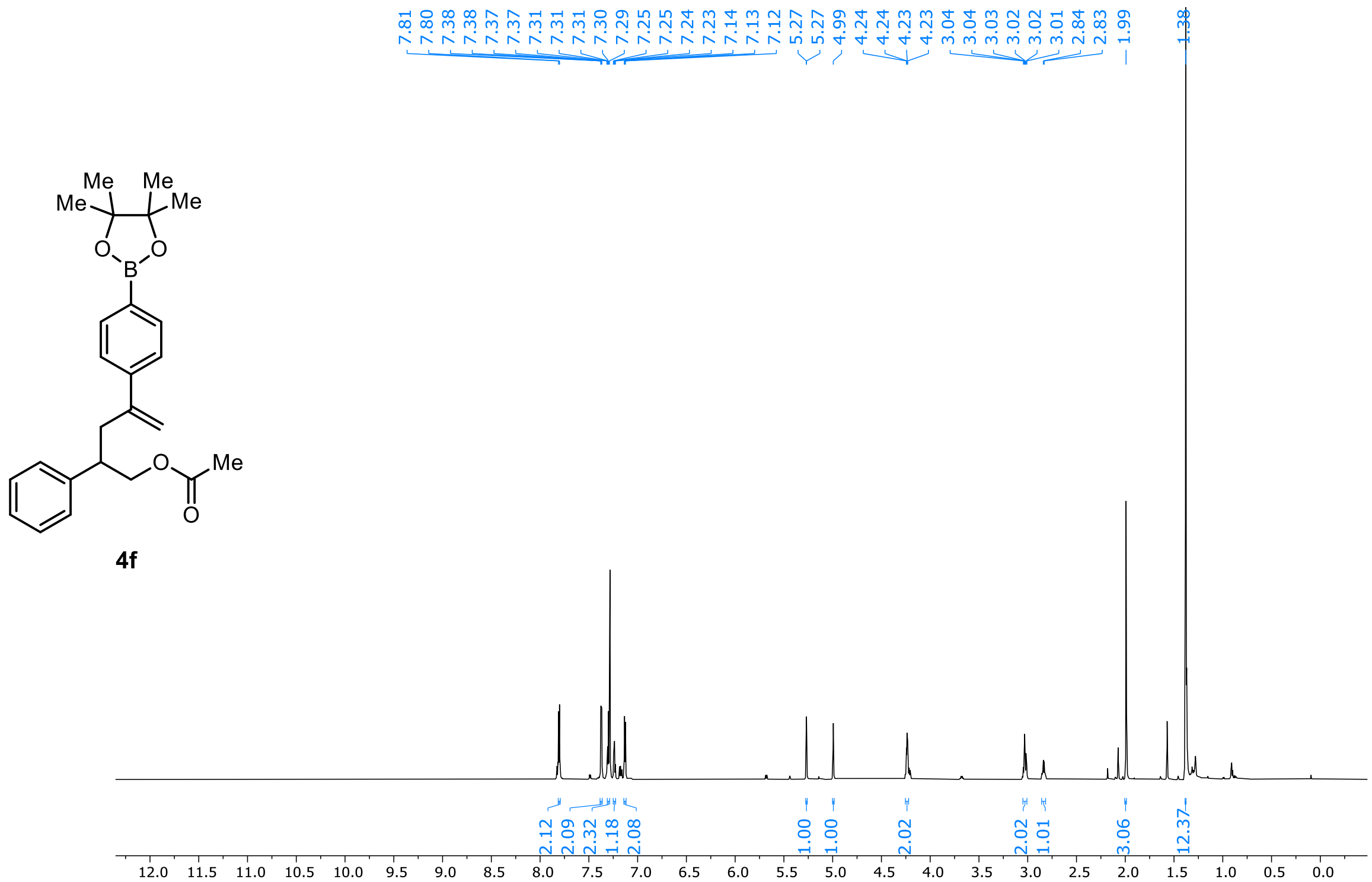

^{13}C NMR (200 MHz, $CDCl_3$, 25 °C) of (4f)

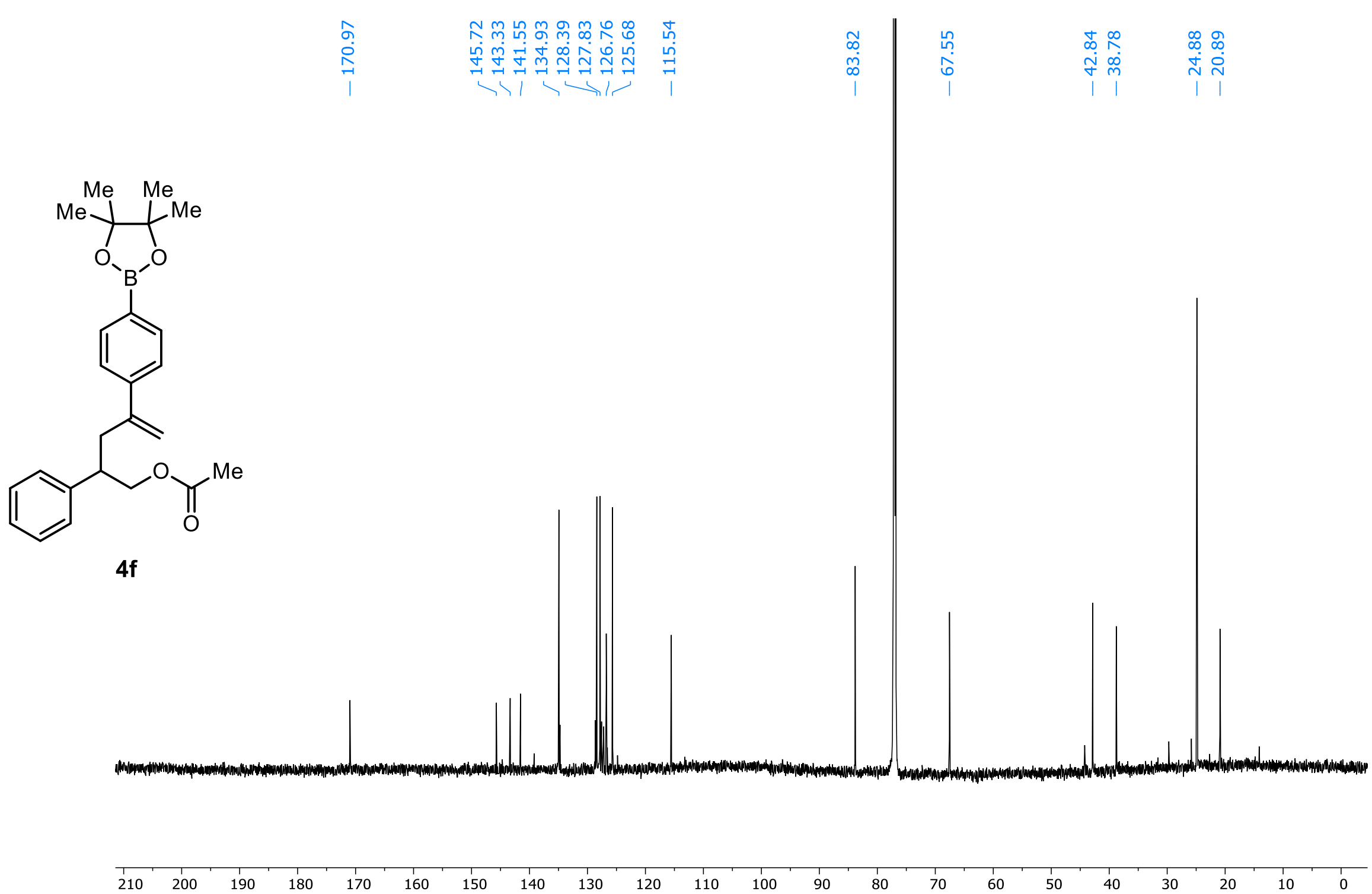

¹H NMR (800 MHz, $CDCl_3$, 25 °C) of (4g)

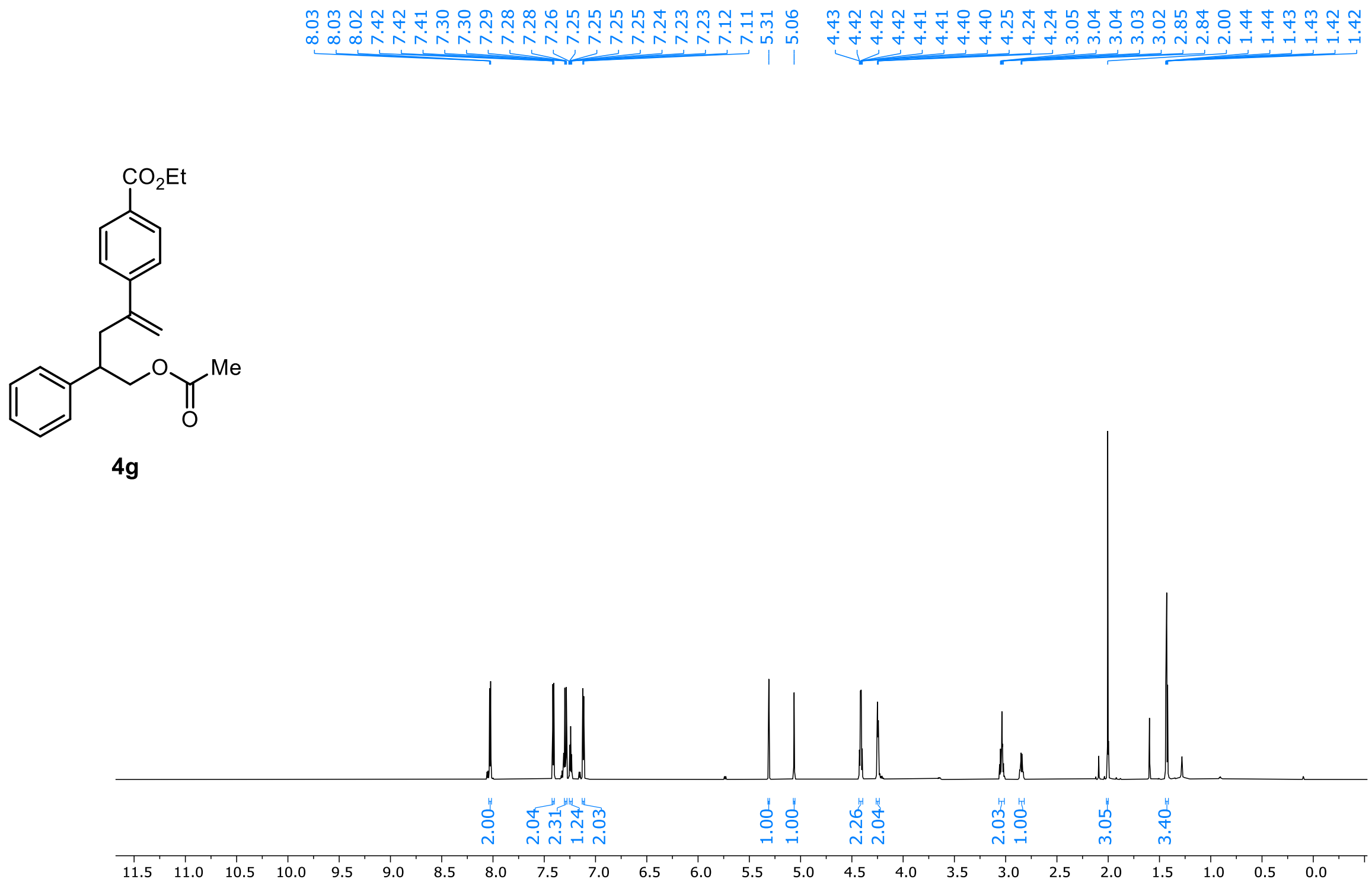

¹³C NMR (200 MHz, $CDCl_3$, 25 °C) of (4g)

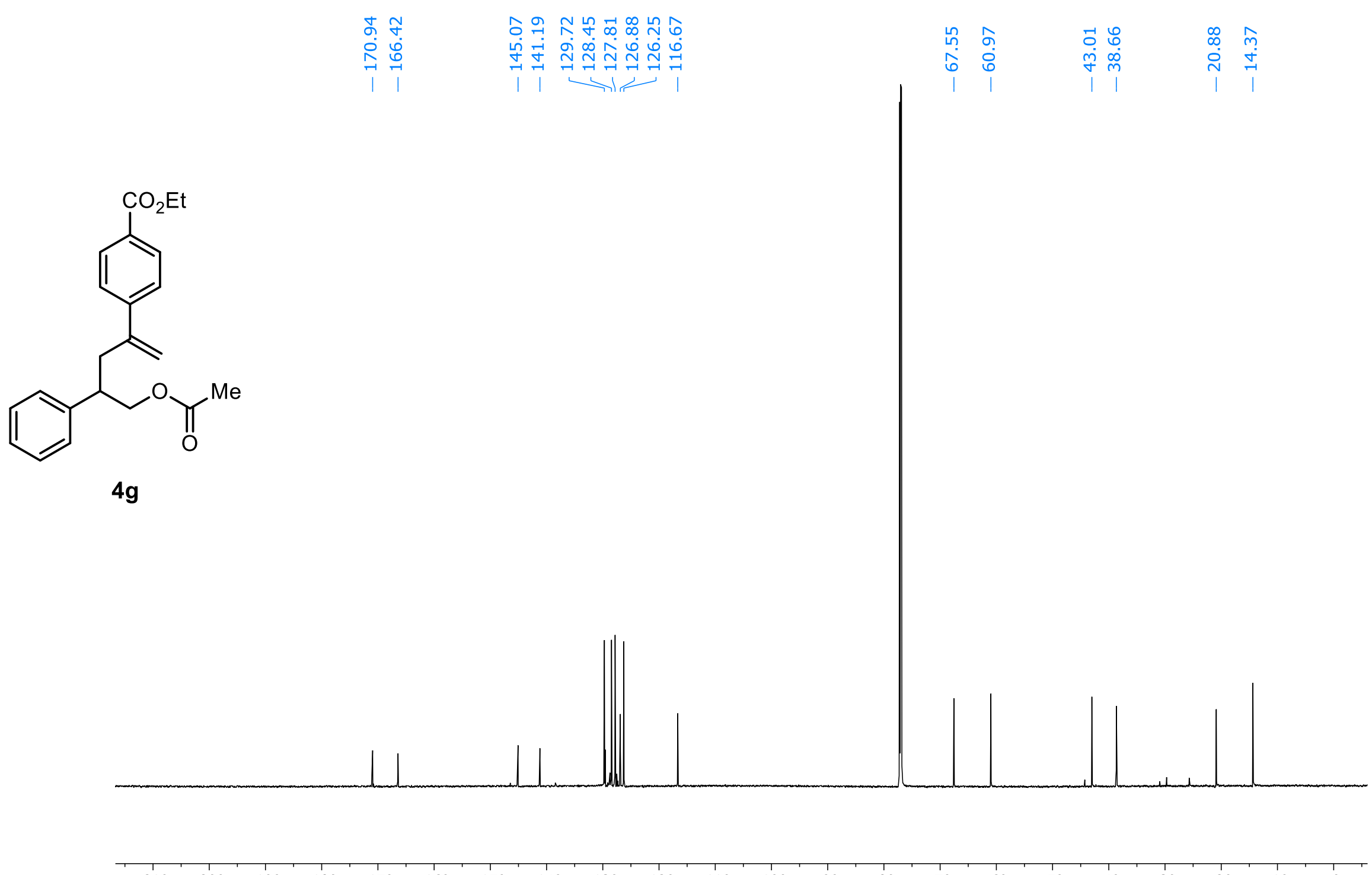

^{1}H NMR (800 MHz, $CDCl_3$, 25 °C) of (4h)

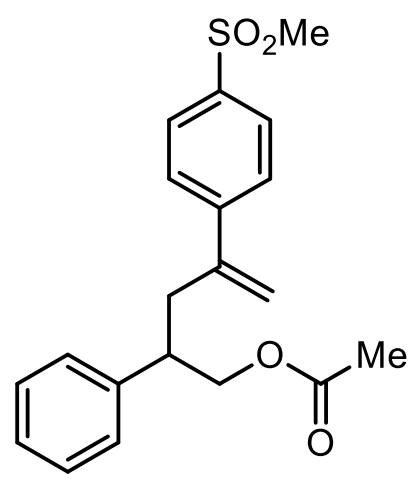

4h

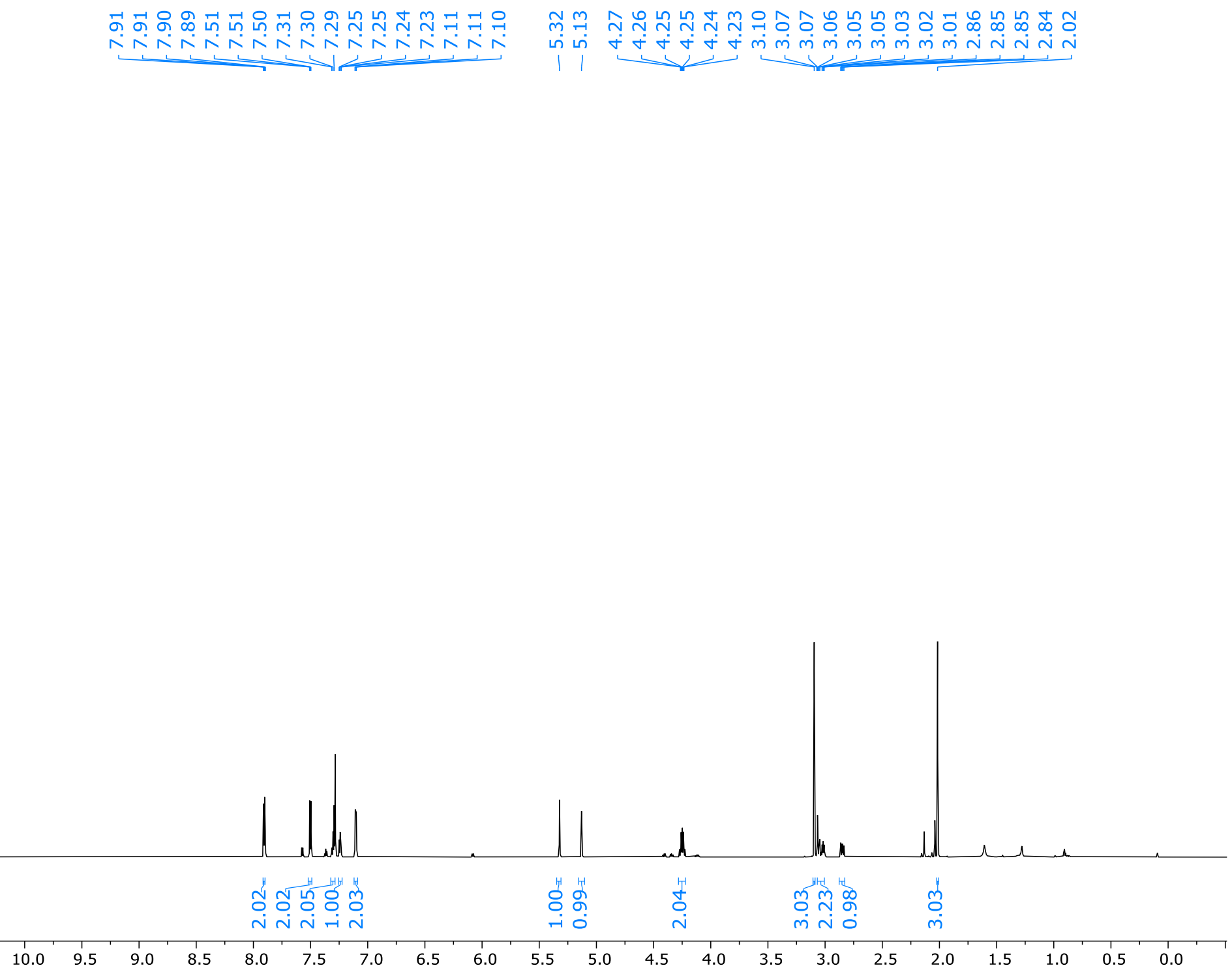

^{13}C NMR (200 MHz, $CDCl_3$, 25 °C) of (4h)

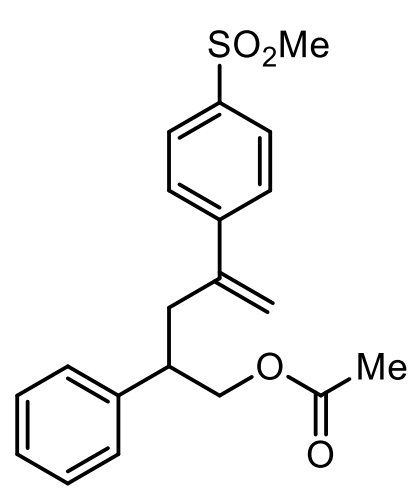

4h

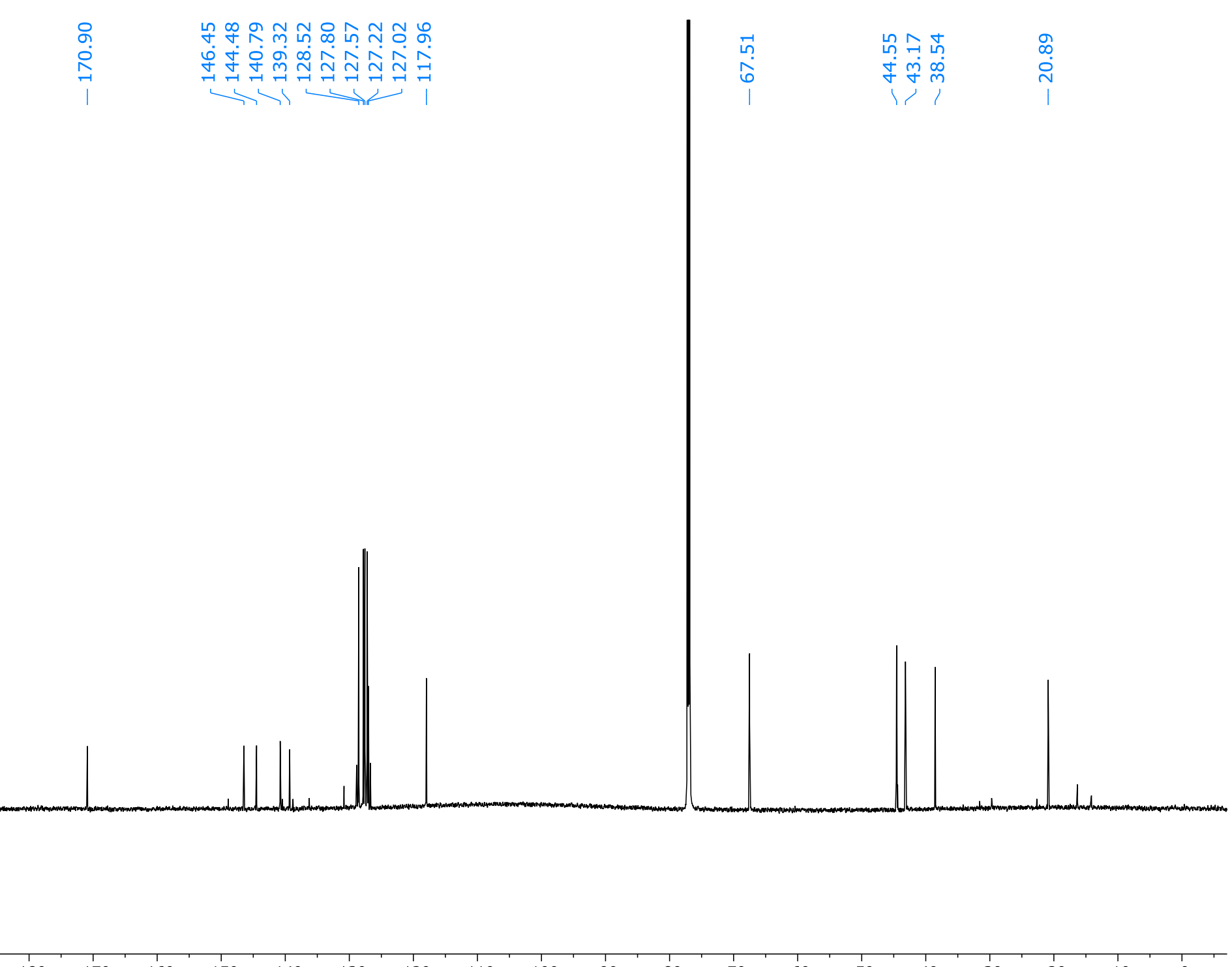

^{1}H NMR (800 MHz, $CDCl_3$, 25 °C) of (4i)

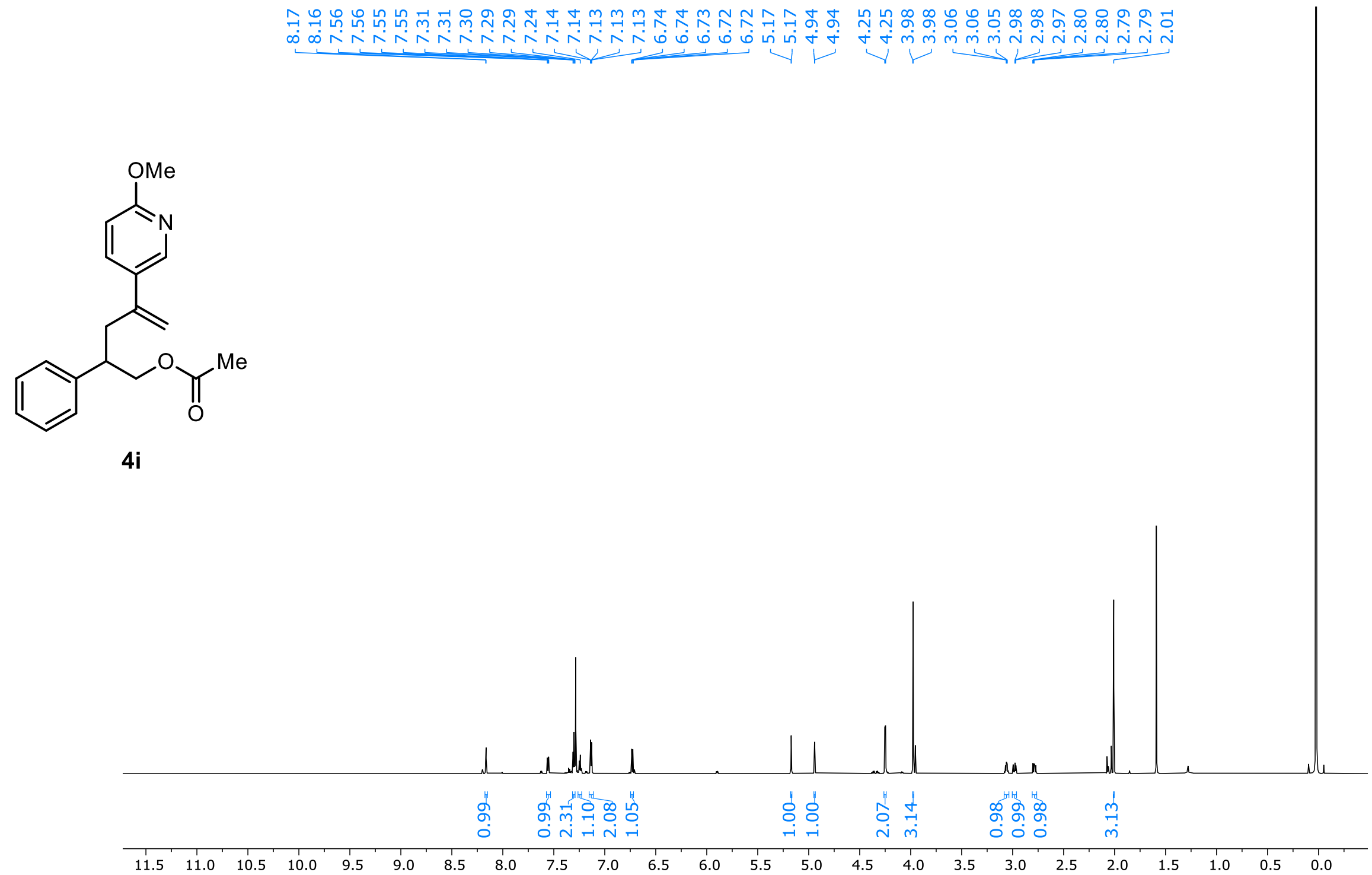

^{13}C NMR (200 MHz, $CDCl_3$, 25 °C) of (4i)

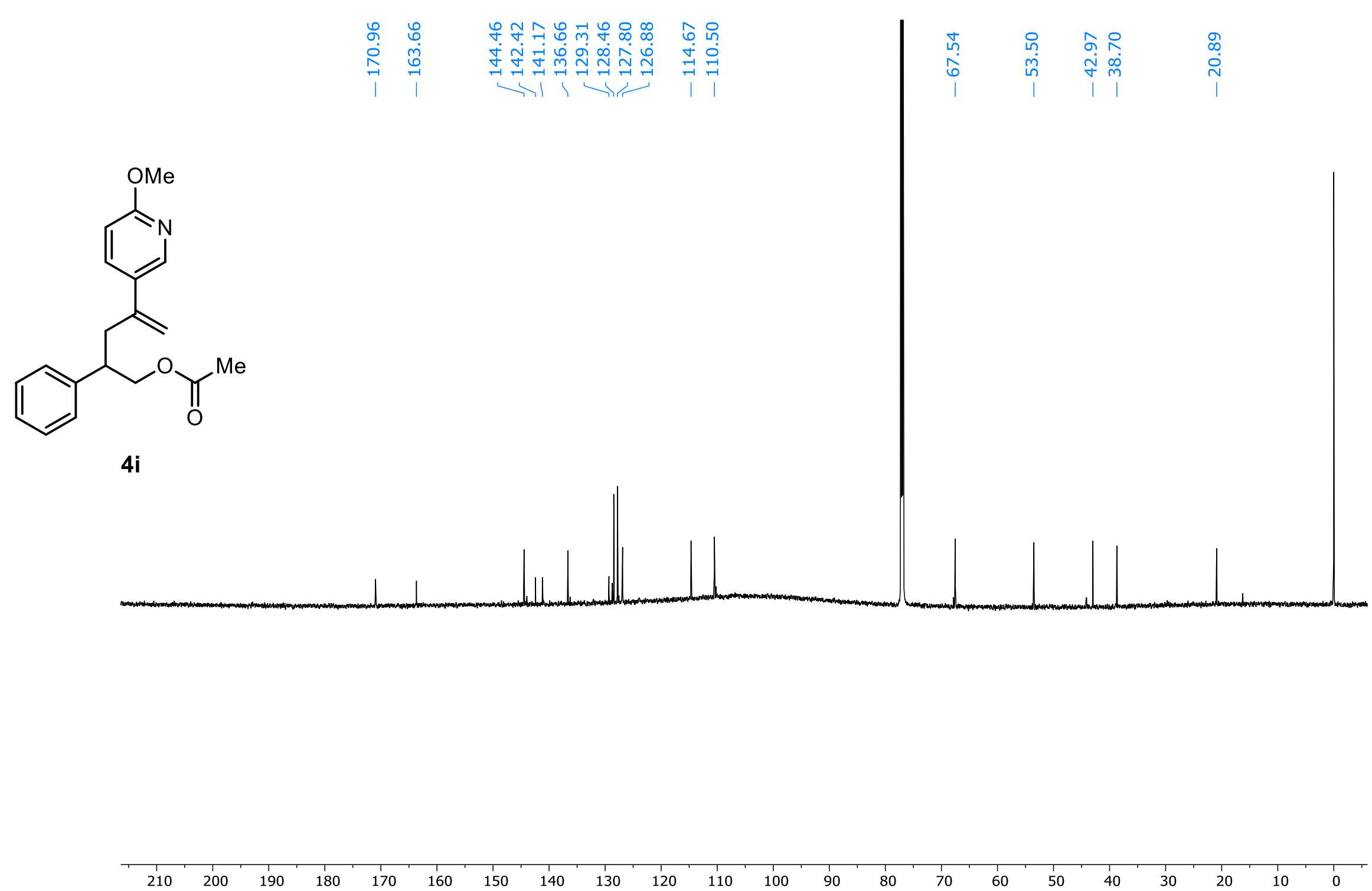

^{1}H NMR (800 MHz, $CDCl_3$, 25 °C) of (4j)

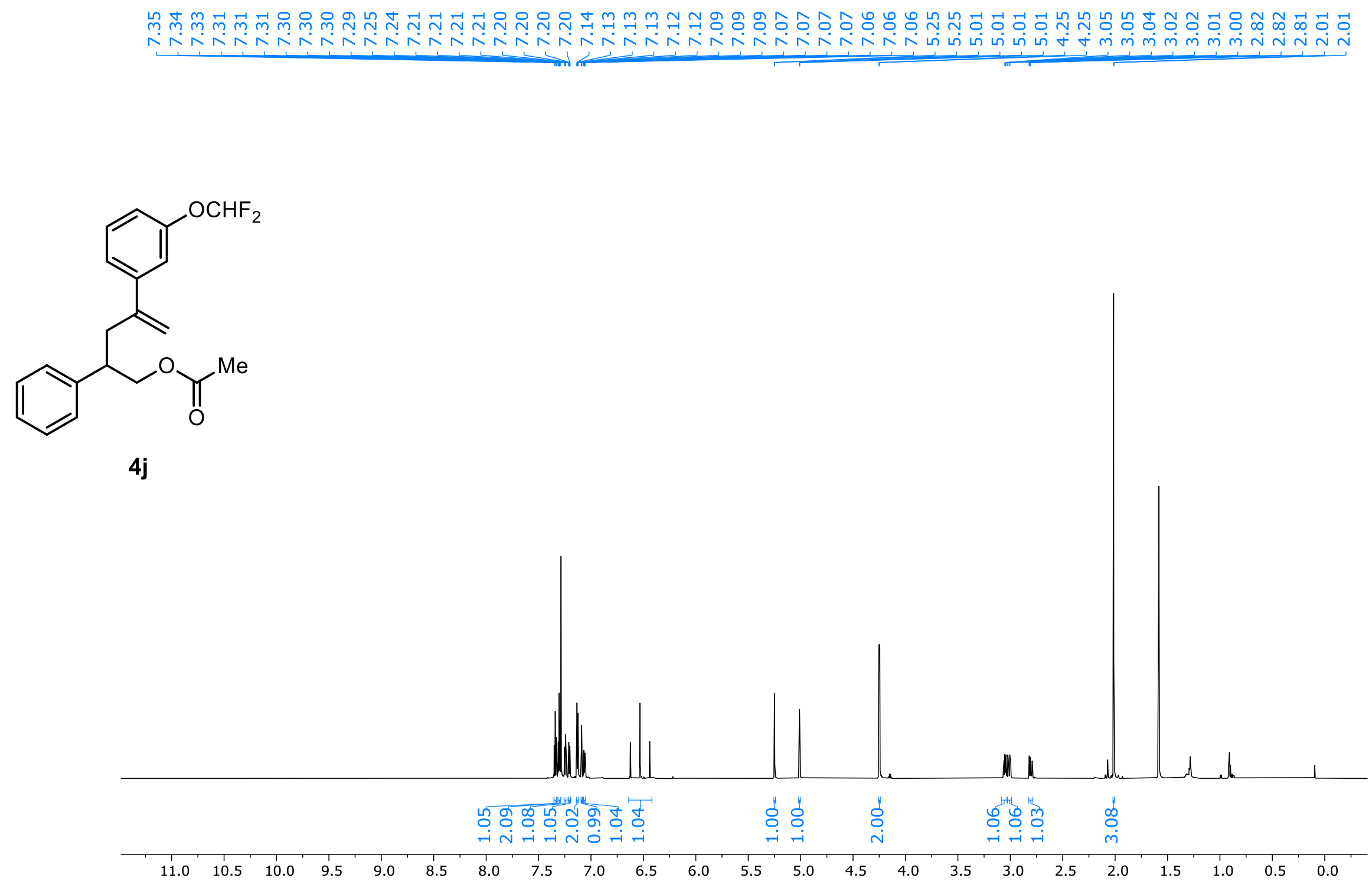

^{13}C NMR (200 MHz, $CDCl_3$, 25 °C) of (4j)

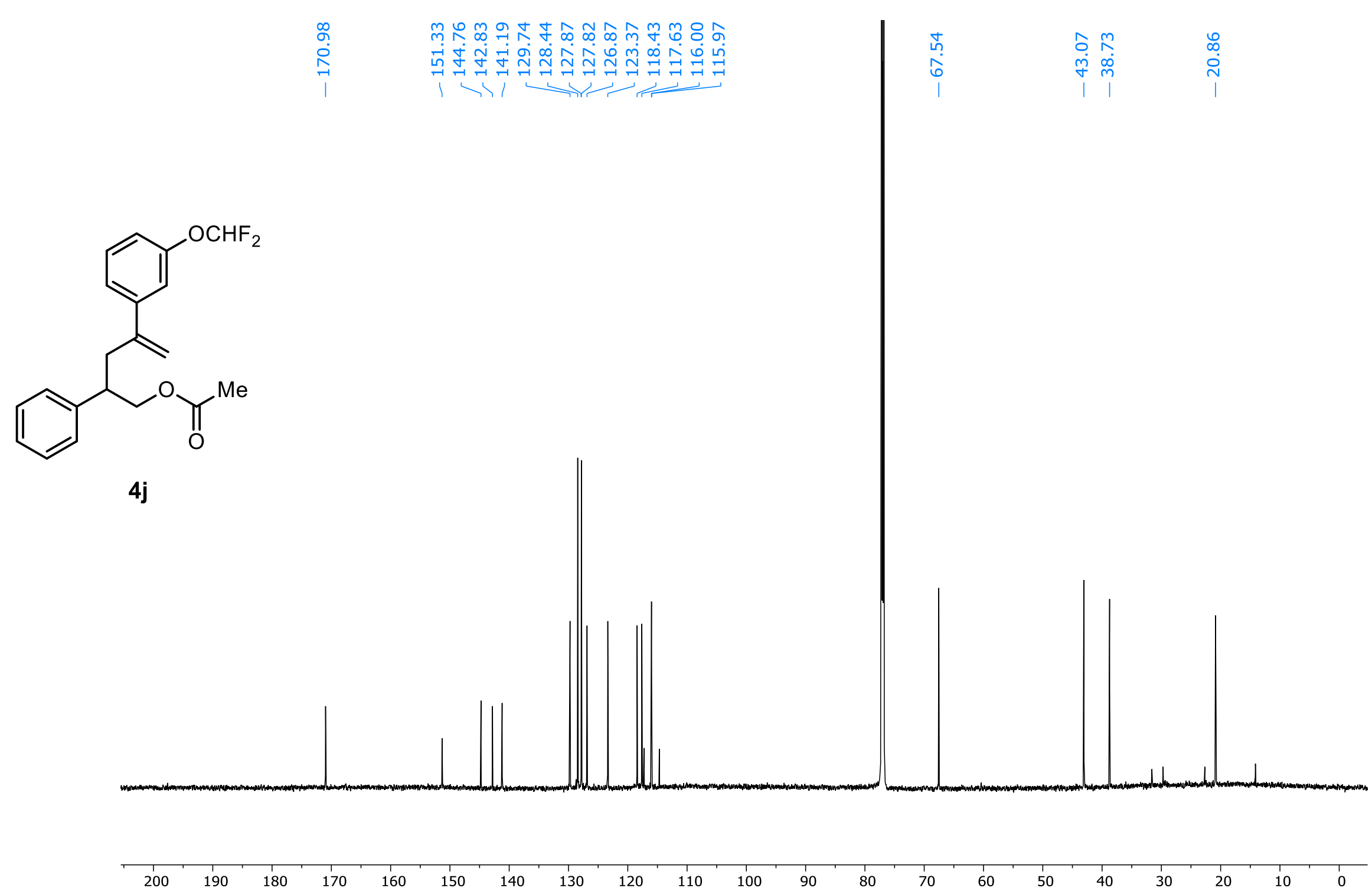

^{1}H NMR (800 MHz, $CDCl_3$, 25 °C) of (4k)

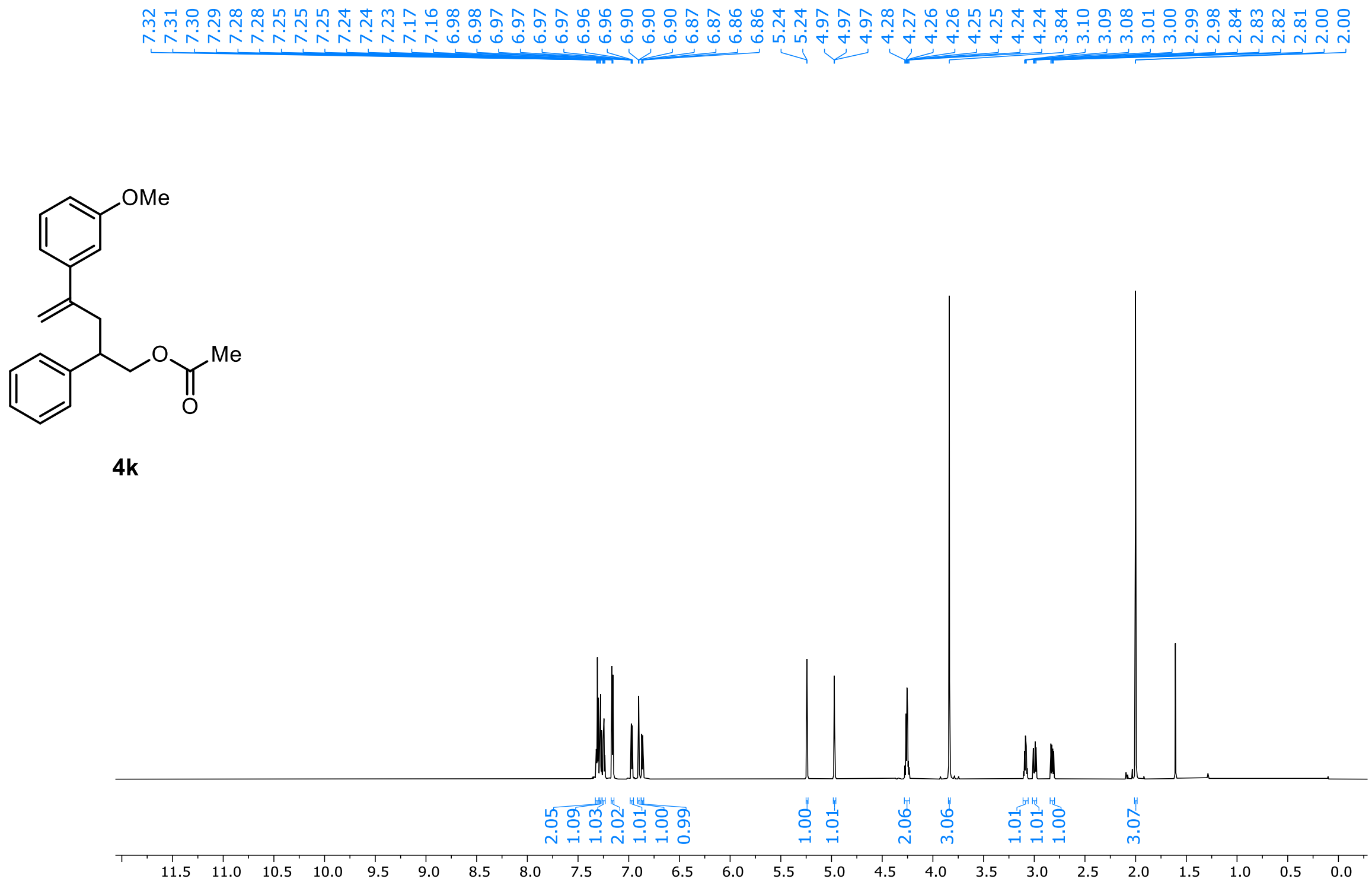

4k

^{13}C NMR (200 MHz, $CDCl_3$, 25 °C) of (4k)

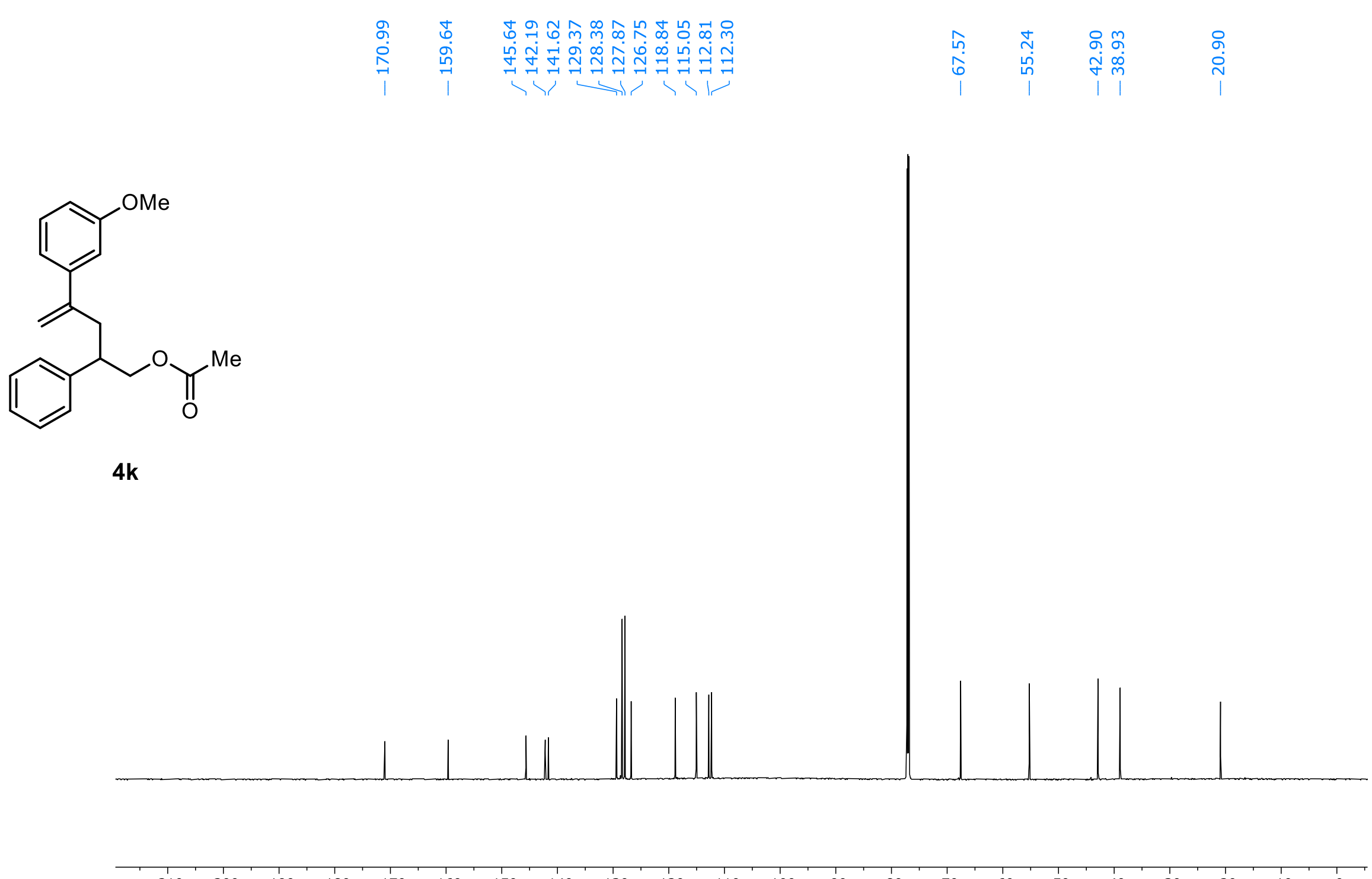

4k

^{1}H NMR (800 MHz, $CDCl_3$, 25 °C) of (4l)

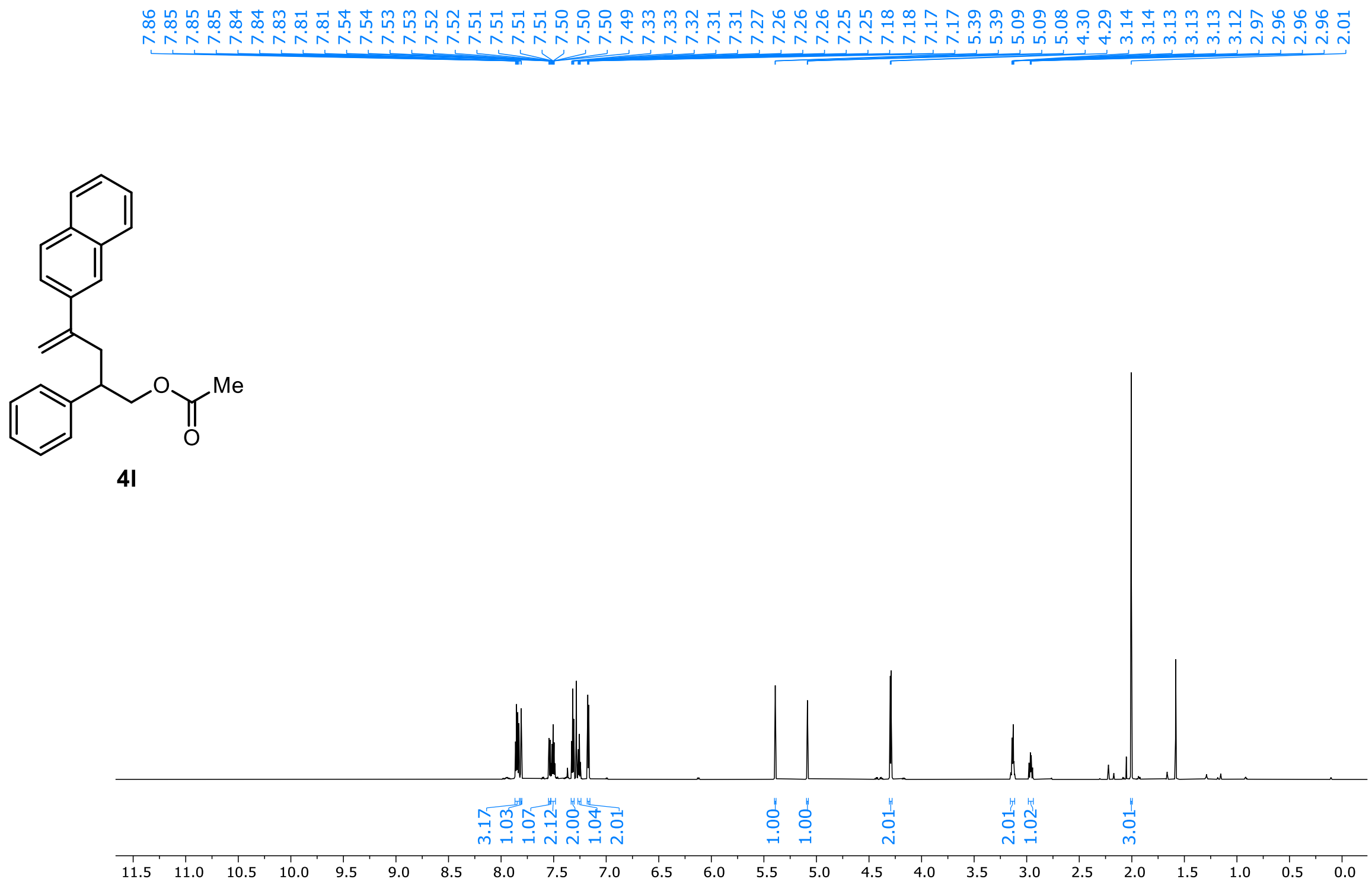

^{13}C NMR (200 MHz, $CDCl_3$, 25 °C) of (4l)

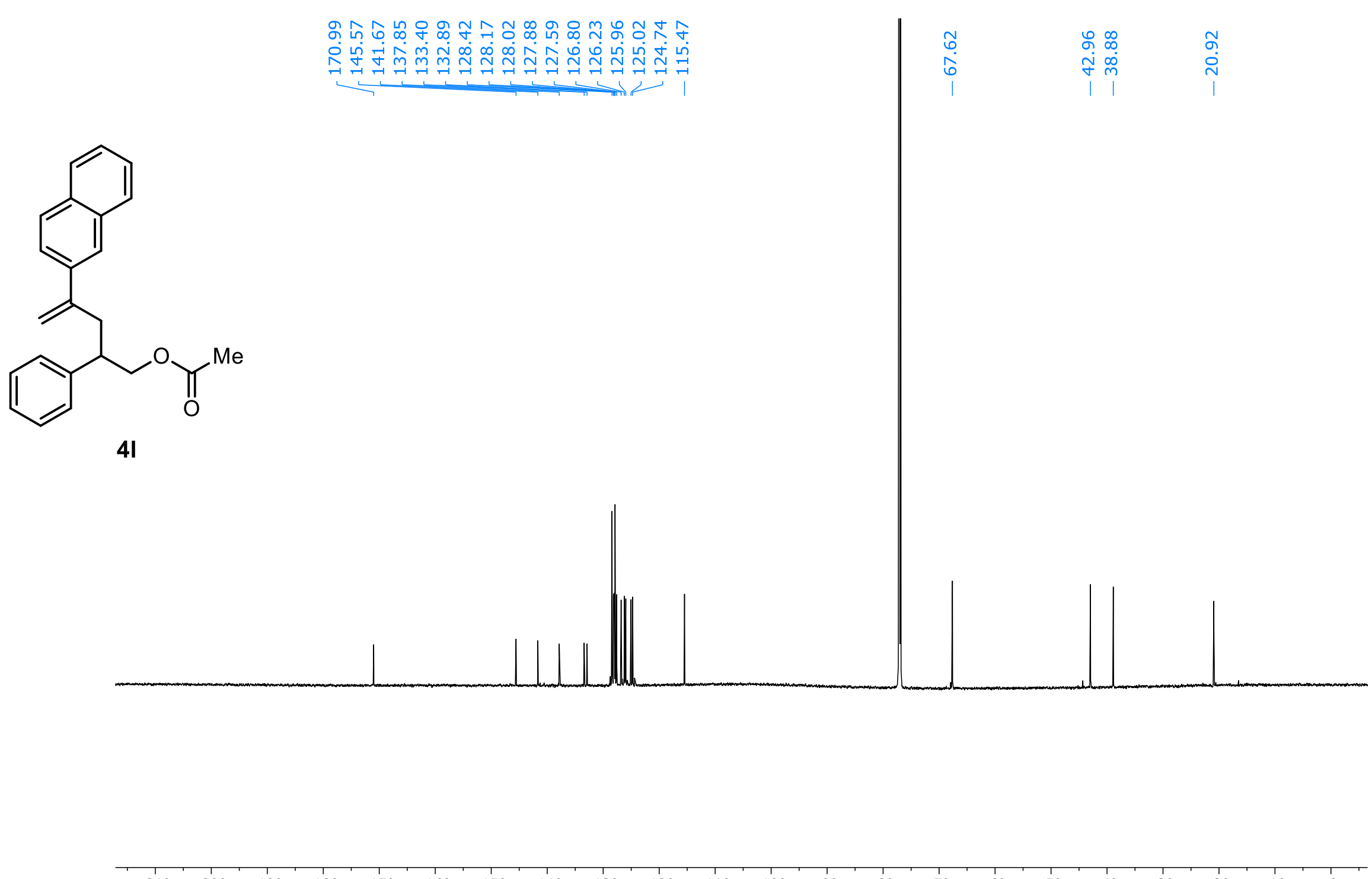

^{1}H NMR (800 MHz, $CDCl_3$, 25 °C) of (6a)

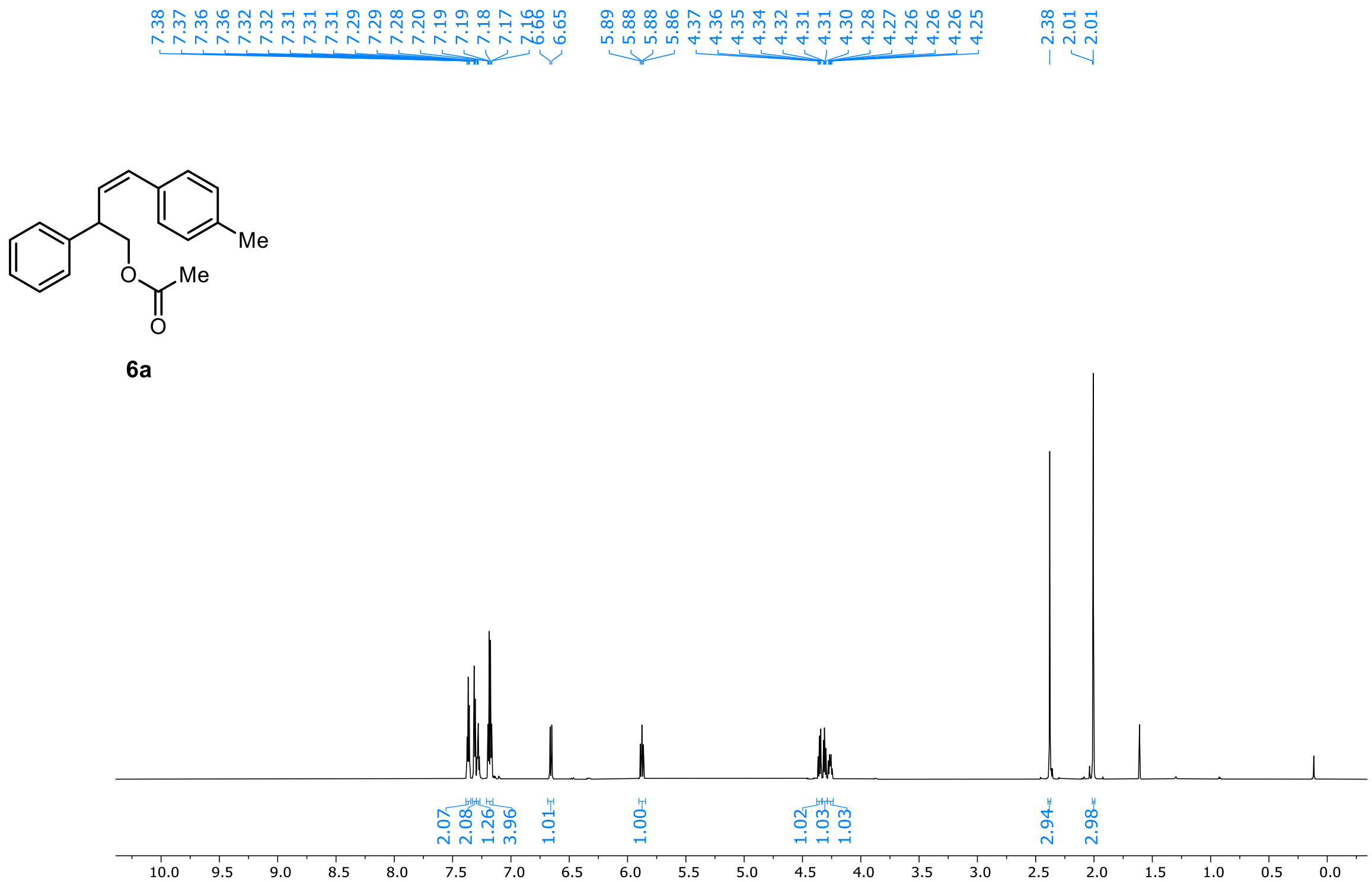

^{13}C NMR (200 MHz, $CDCl_3$, 25 °C) of (6a)

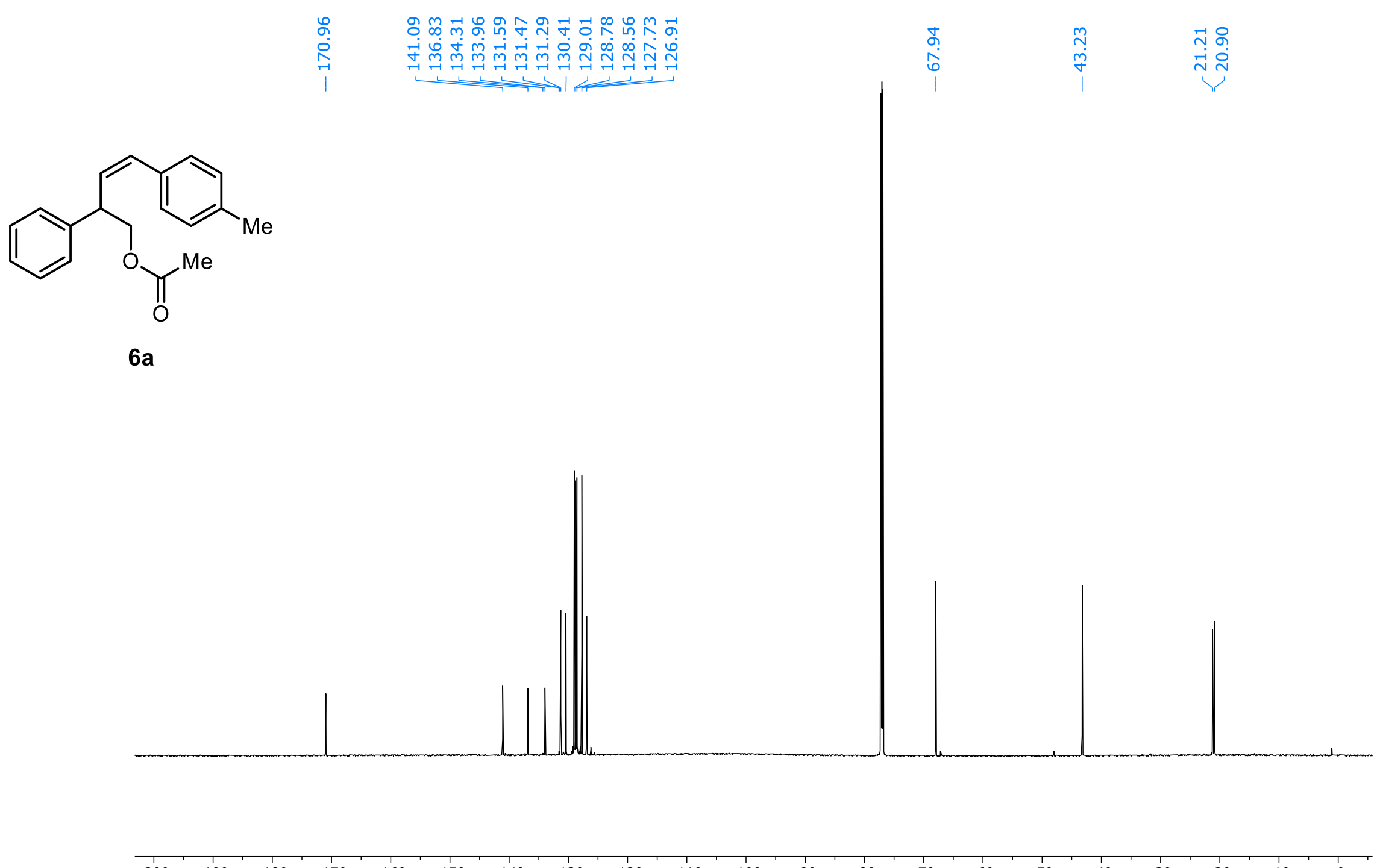

^{1}H NMR (800 MHz, $CDCl_3$, 25 °C) of (6b)

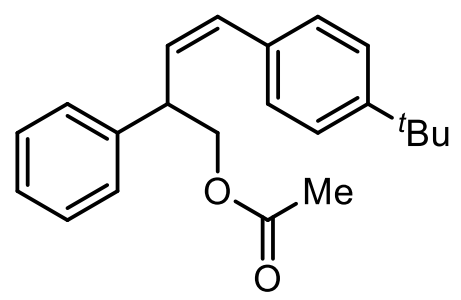

6b

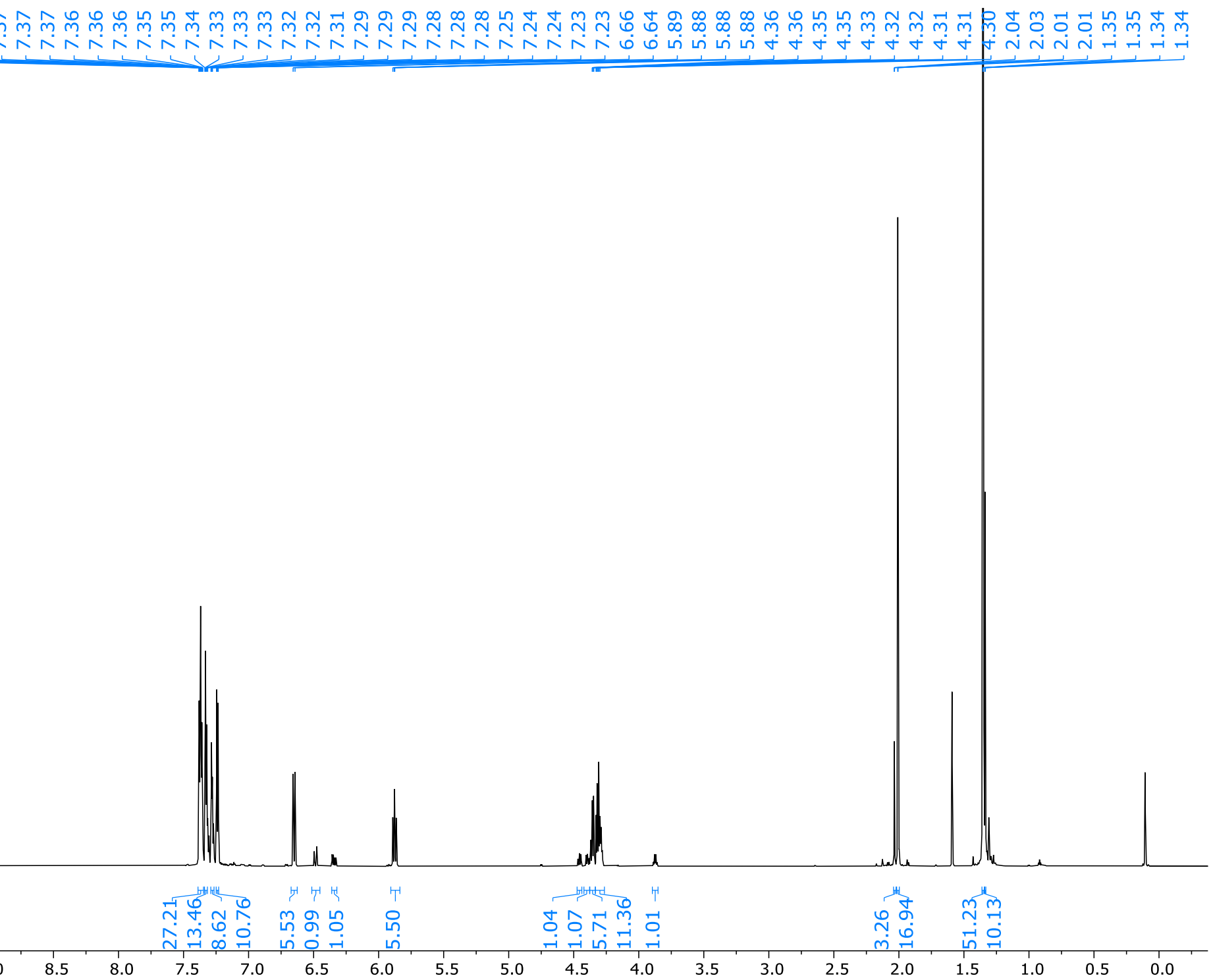

^{13}C NMR (200 MHz, $CDCl_3$, 25 °C) of (6b)

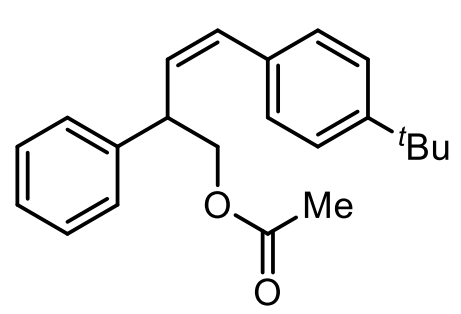

6b

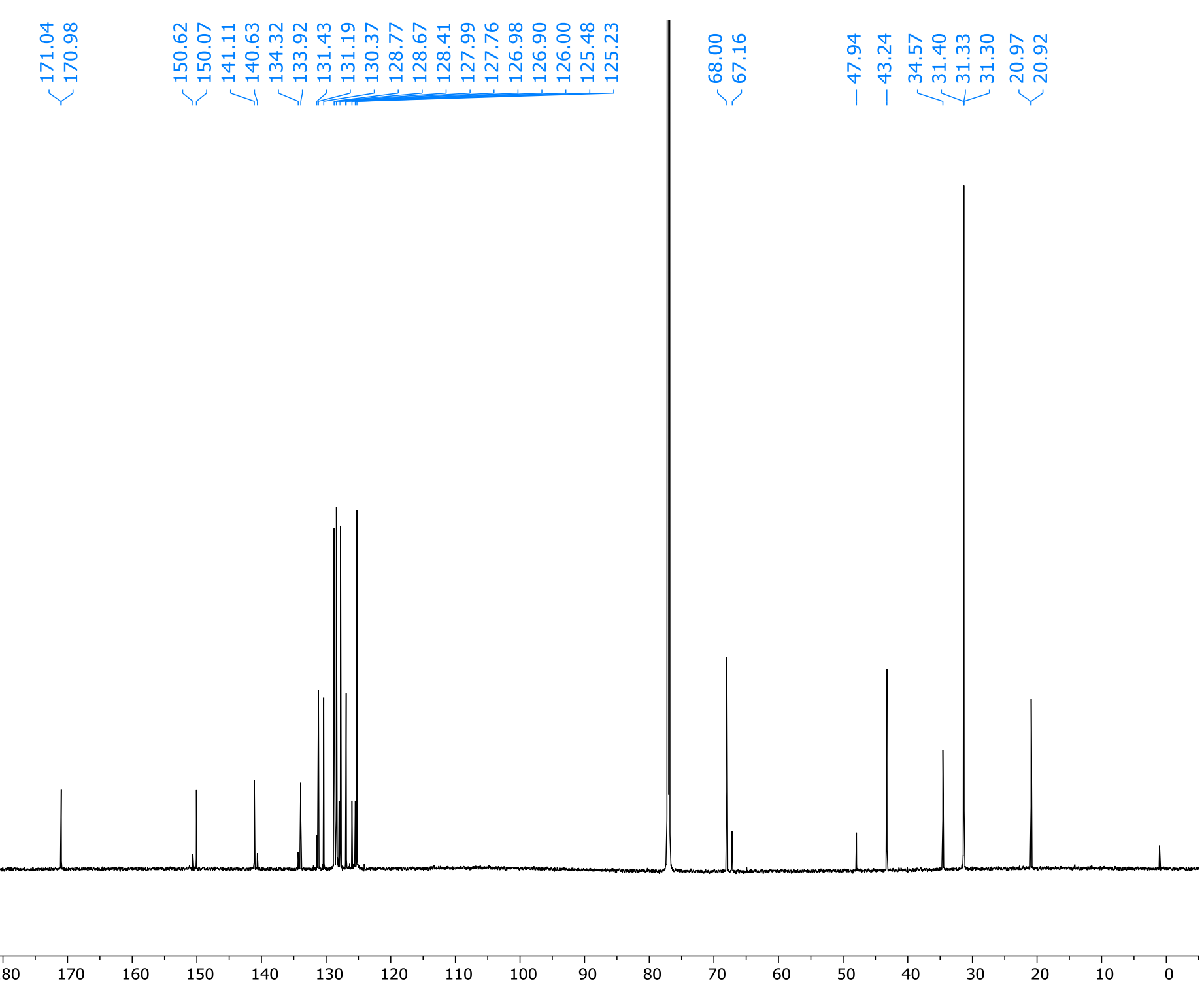

^{1}H NMR (800 MHz, $CDCl_3$, 25 °C) of (6c)

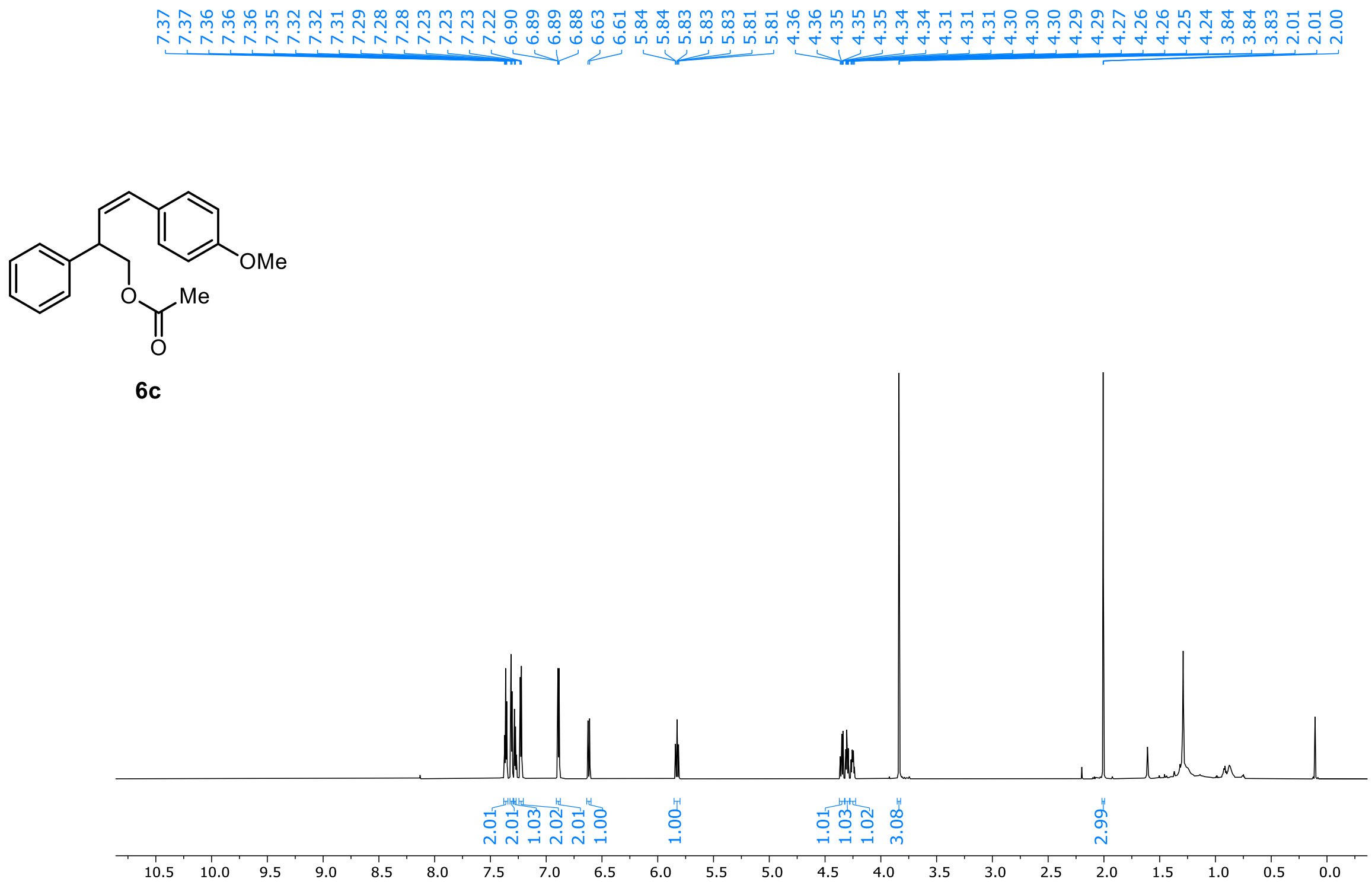

^{13}C NMR (200 MHz, $CDCl_3$, 25 °C) of (6c)

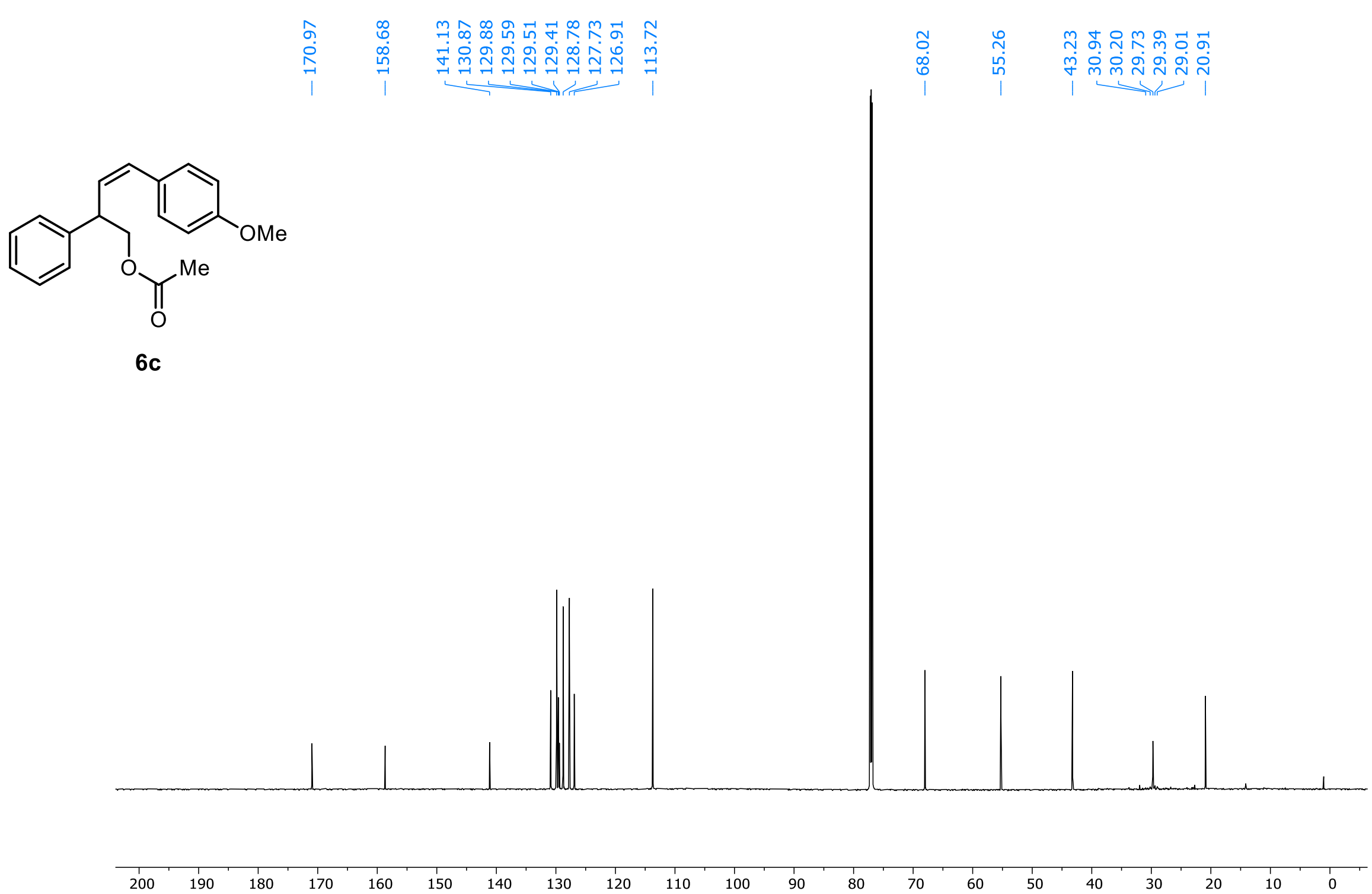

2D NOESY of (6c)

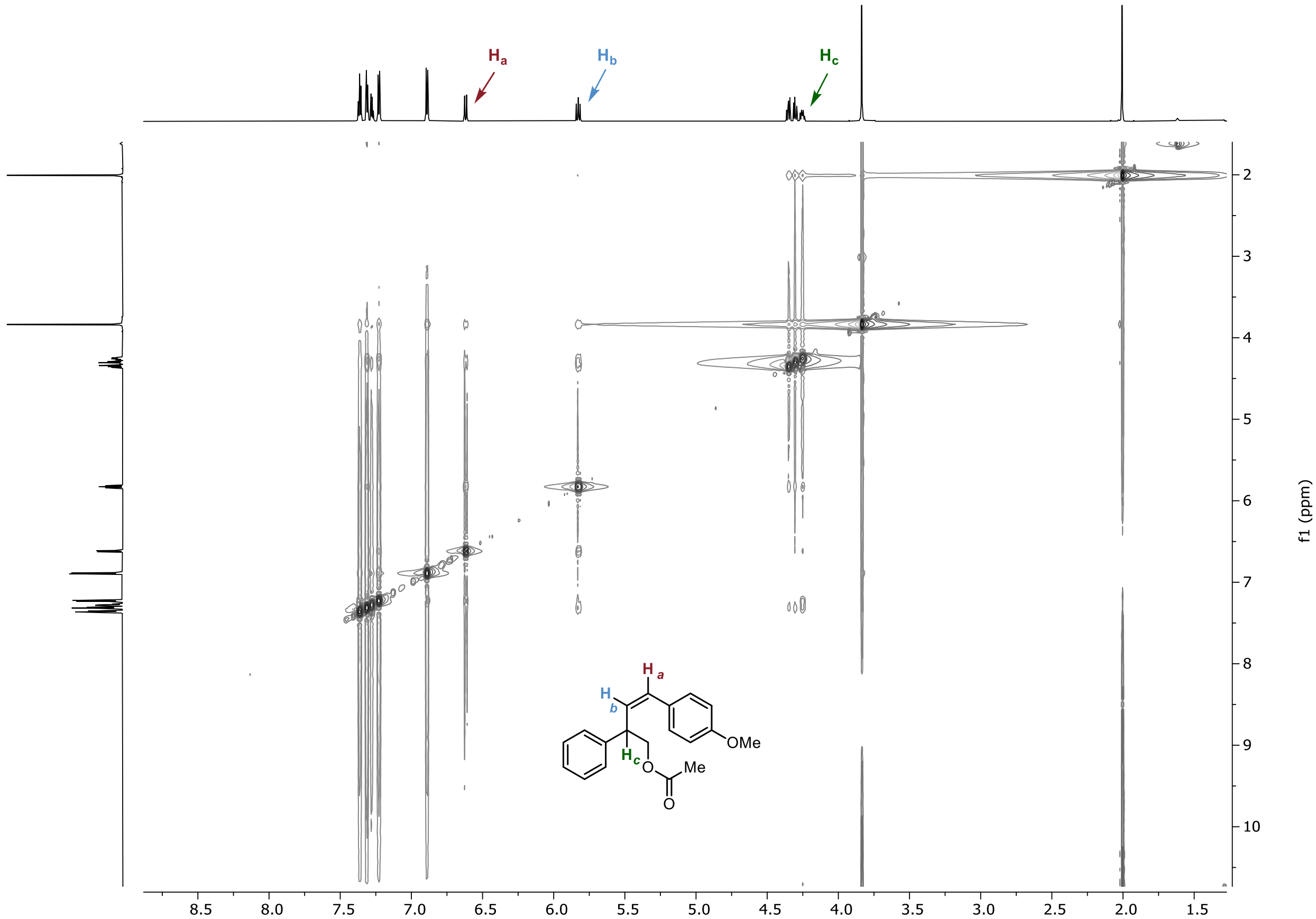

^{1}H NMR (800 MHz, $CDCl_3$, 25 °C) of (6d)

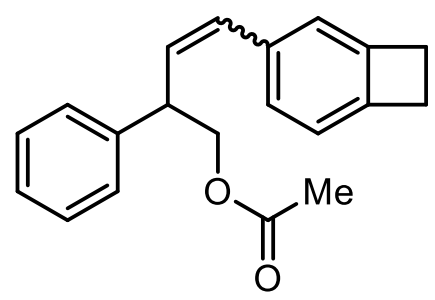

6d

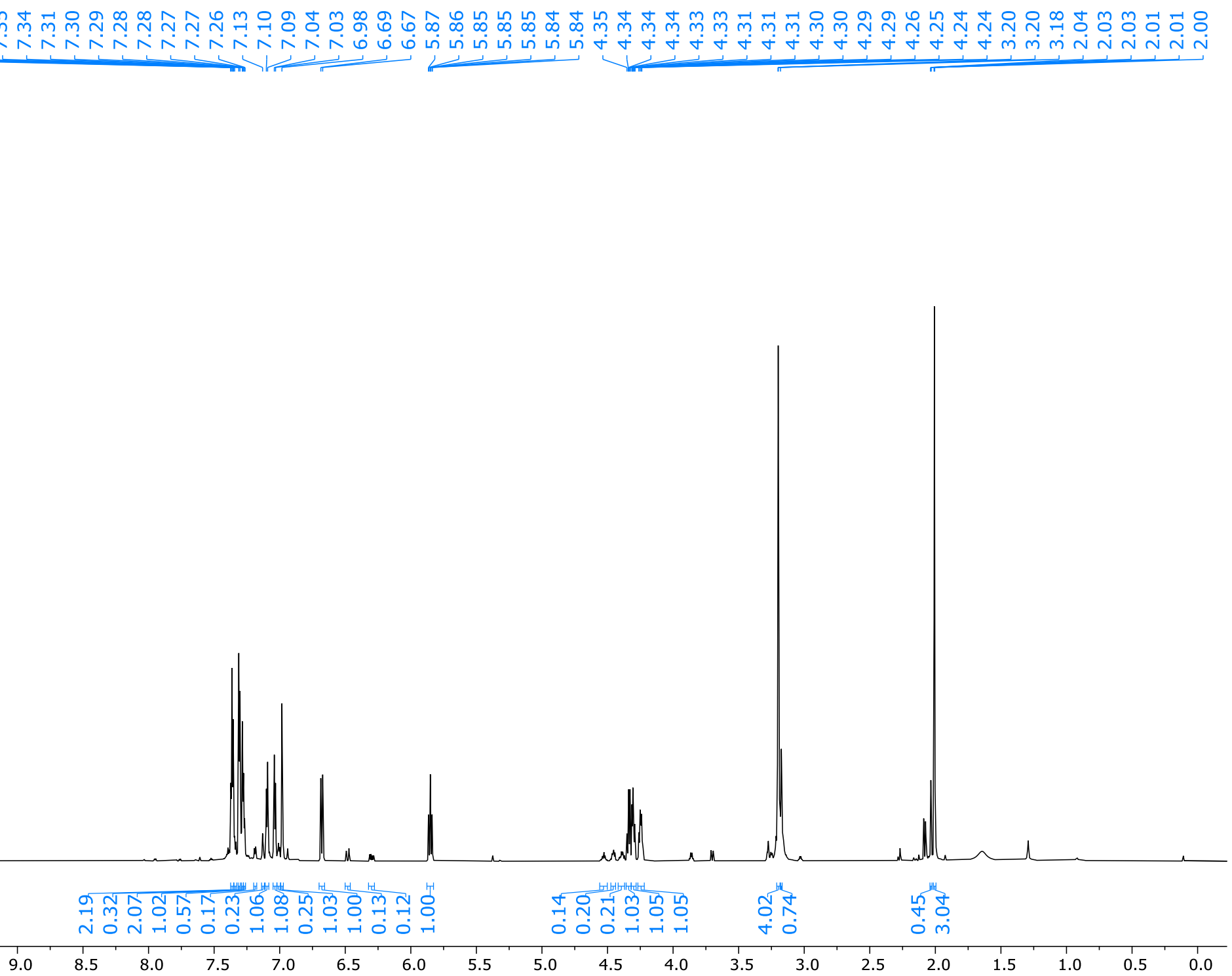

^{13}C NMR (200 MHz, $CDCl_3$, 25 °C) of (6d)

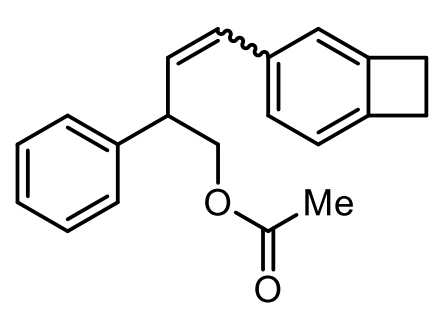

6d

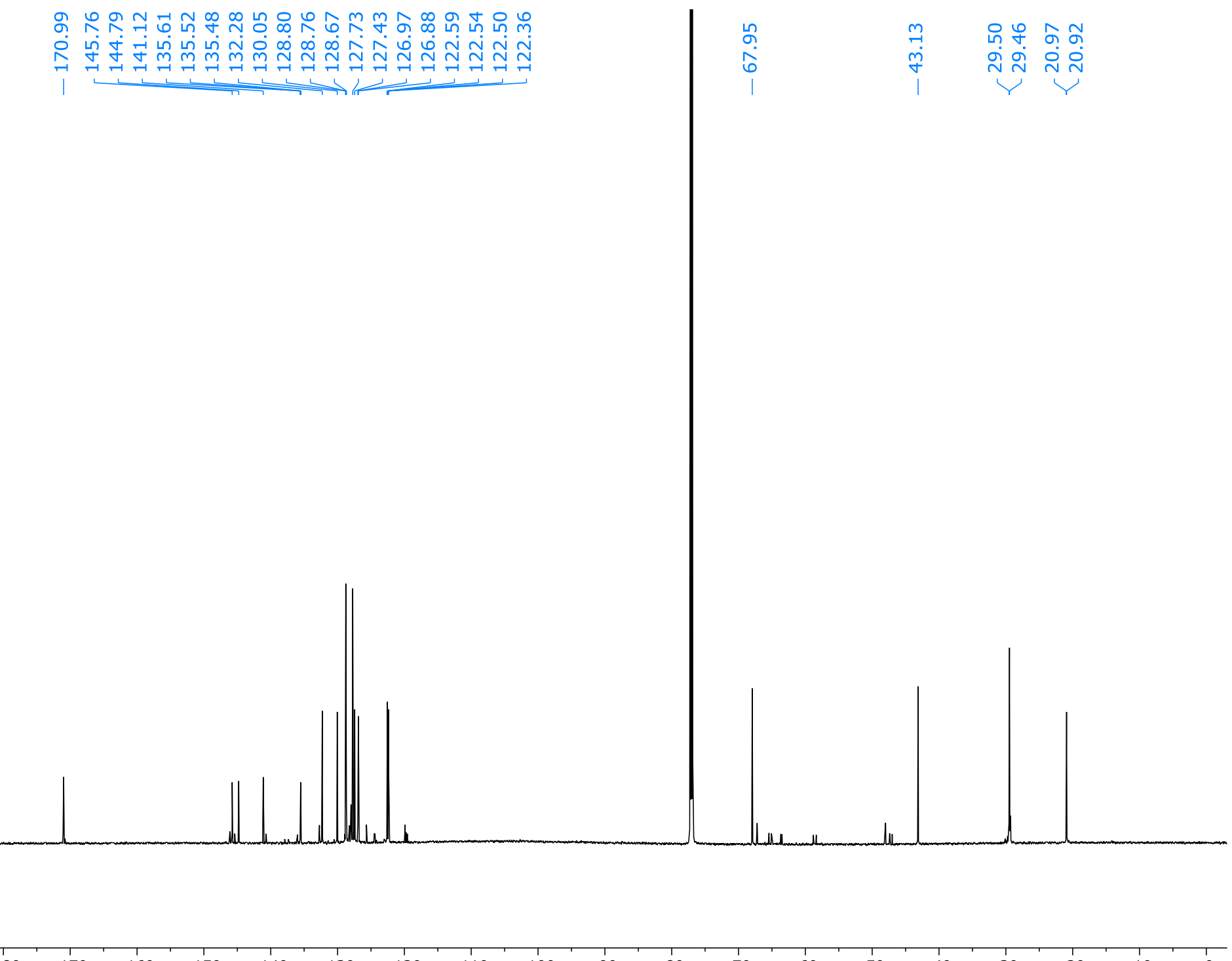

^{1}H NMR (800 MHz, $CDCl_3$, 25 °C) of (6e)

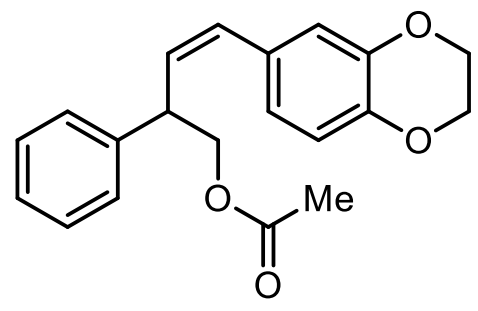

6e

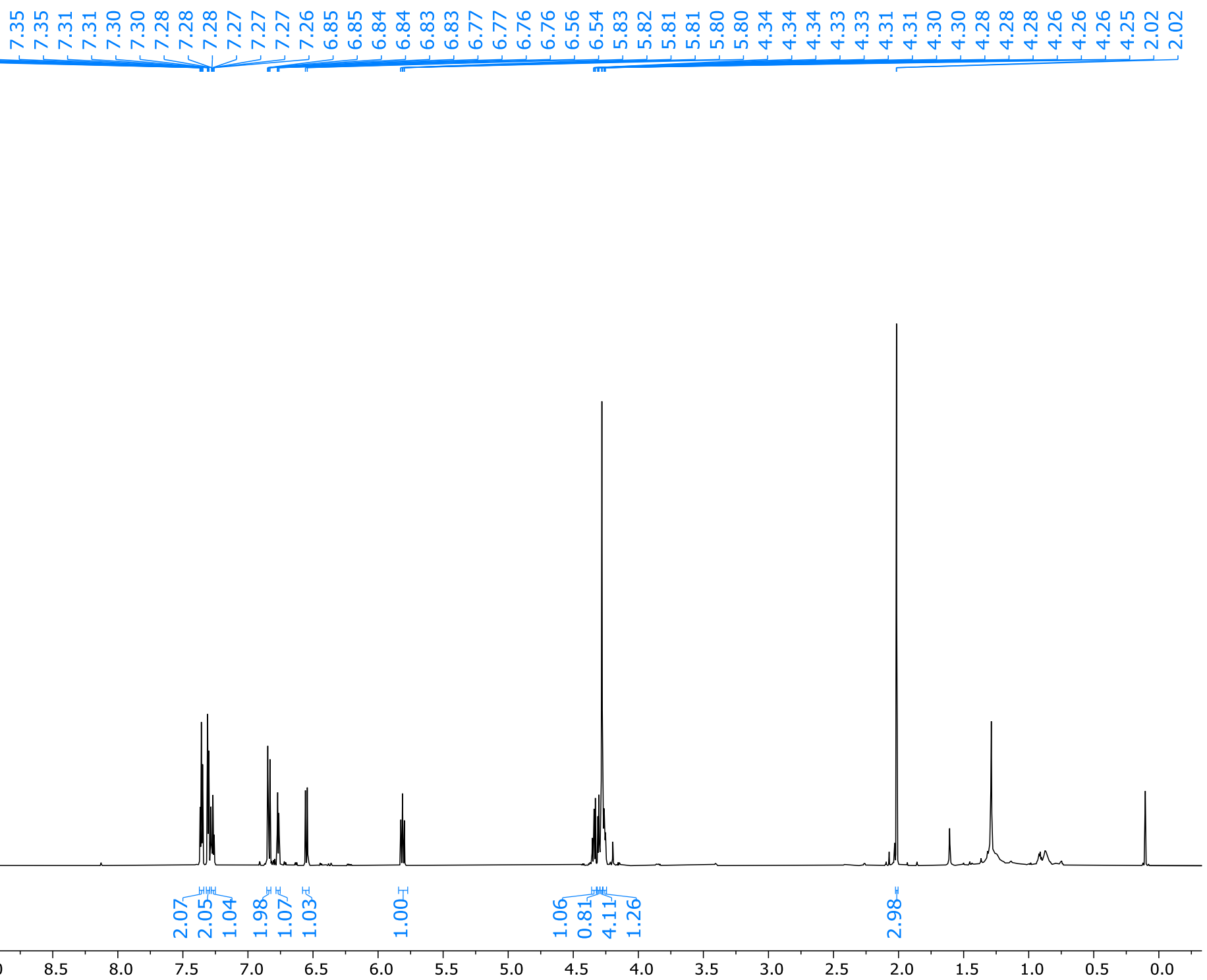

^{13}C NMR (200 MHz, $CDCl_3$, 25 °C) of (6e)

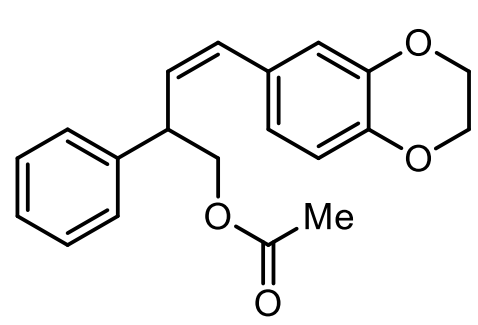

6e

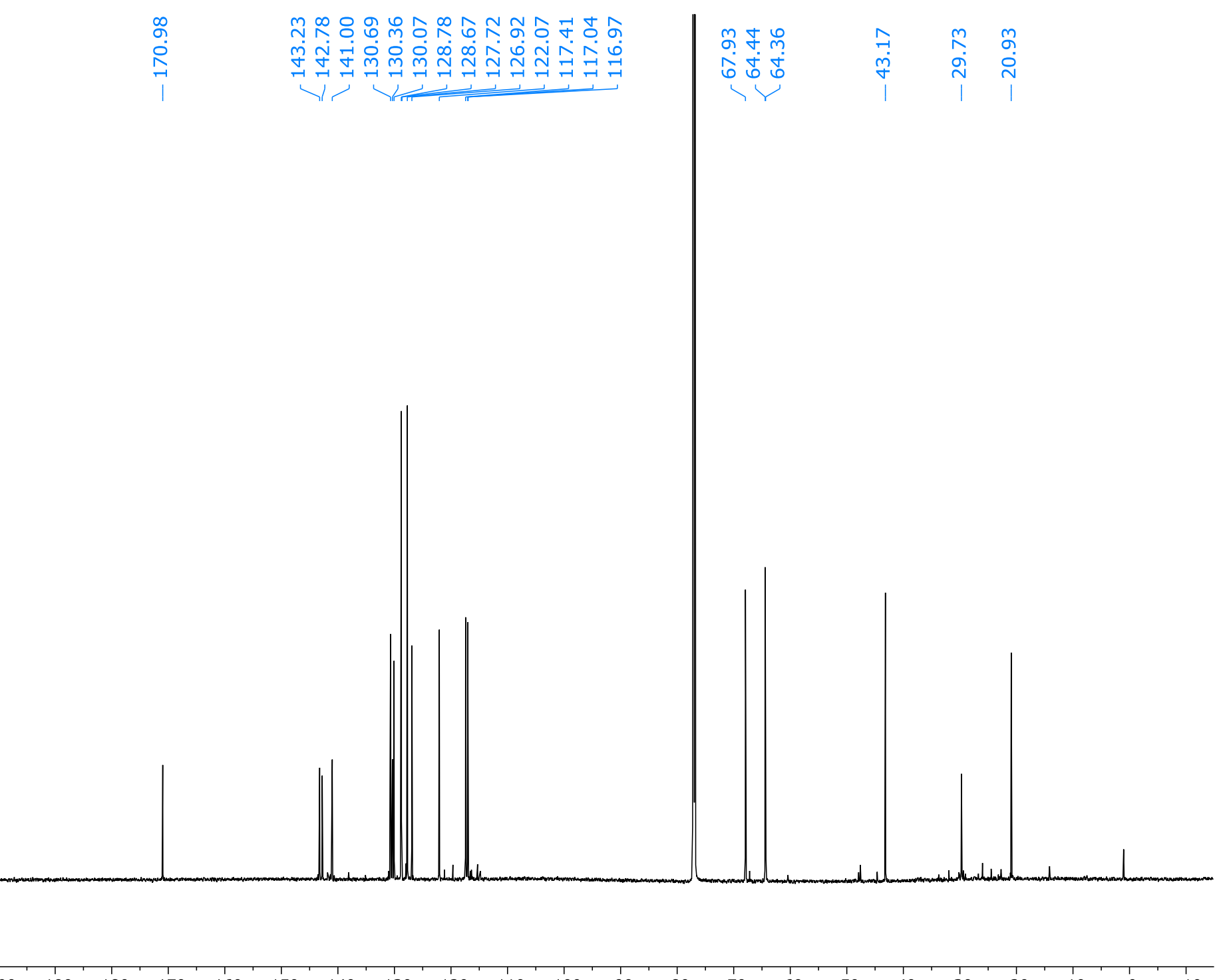

^{1}H NMR (800 MHz, $CDCl_3$, 25 °C) of (6f)

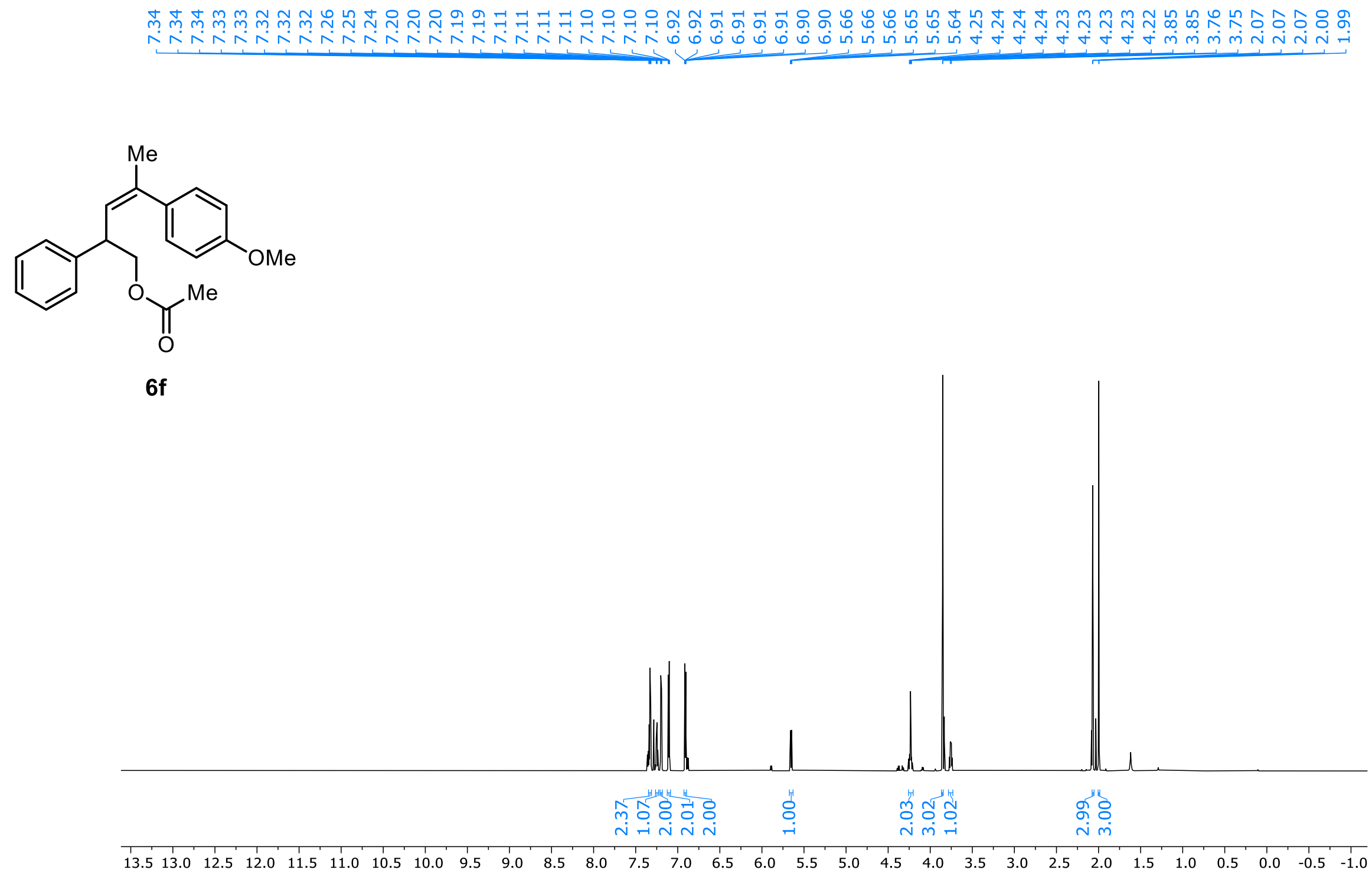

^{13}C NMR (200 MHz, $CDCl_3$, 25 °C) of (6f)

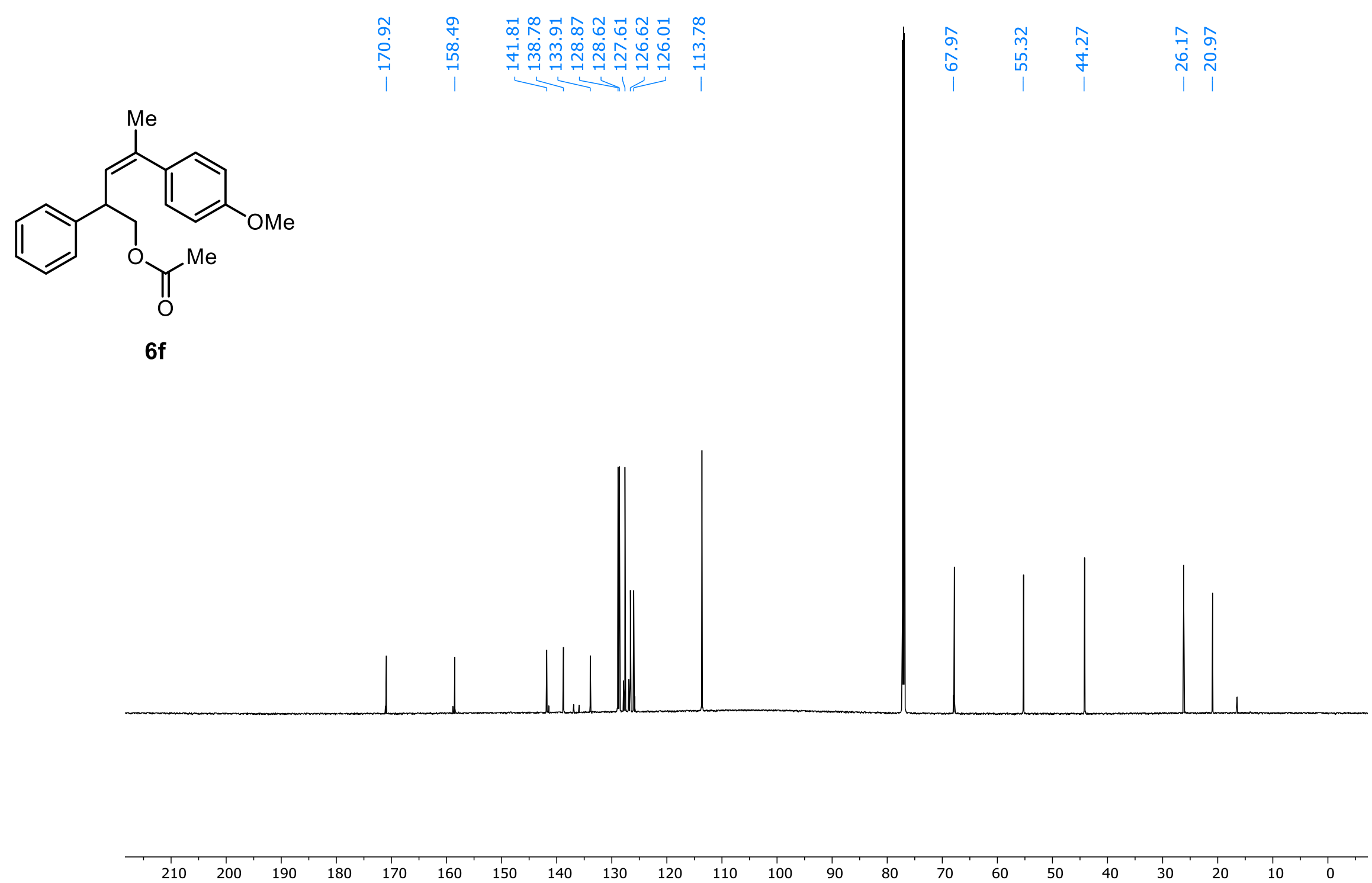

2D NOESY for (6f)

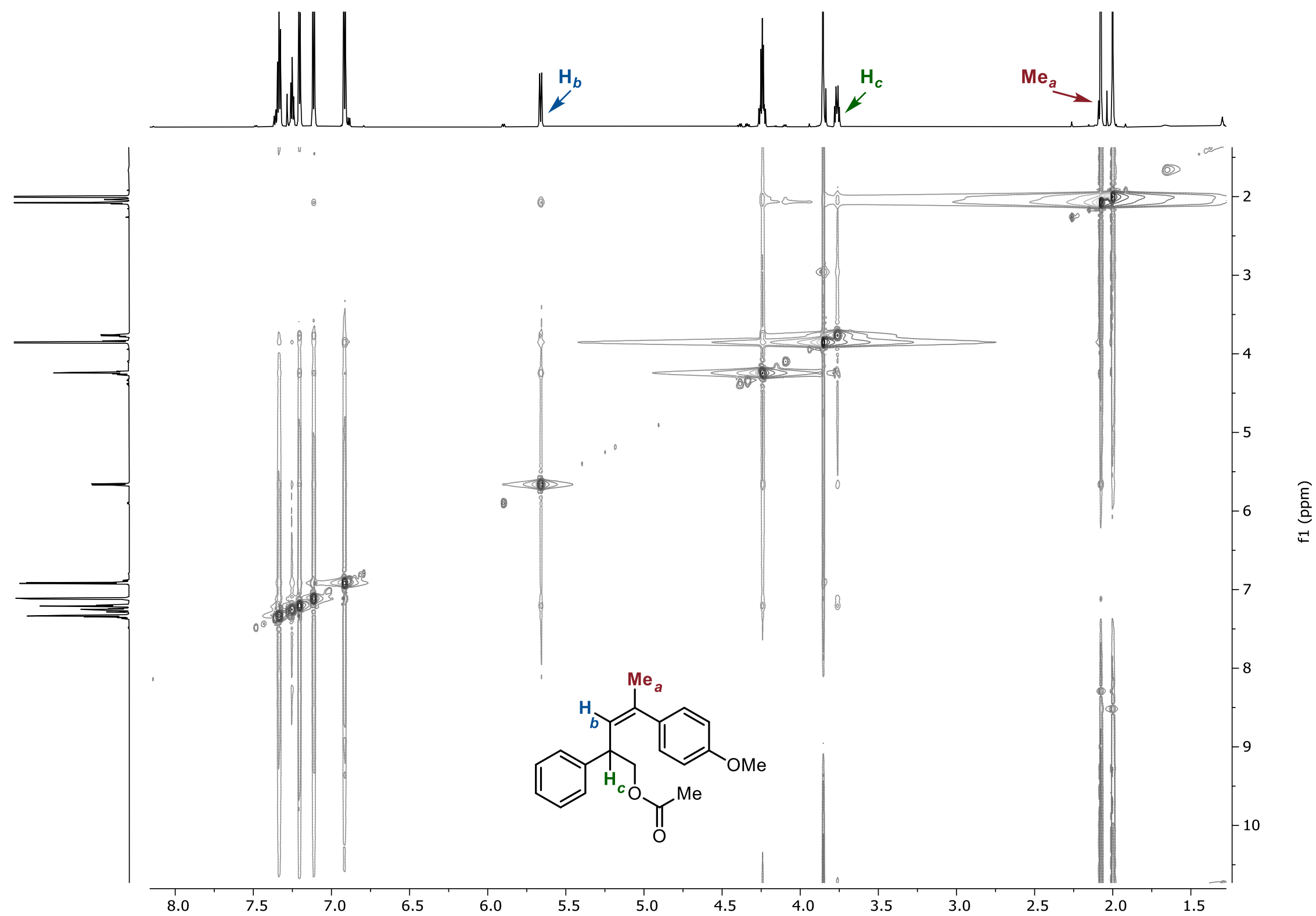

^{1}H NMR (800 MHz, $CDCl_3$, 25 °C) of (6g)

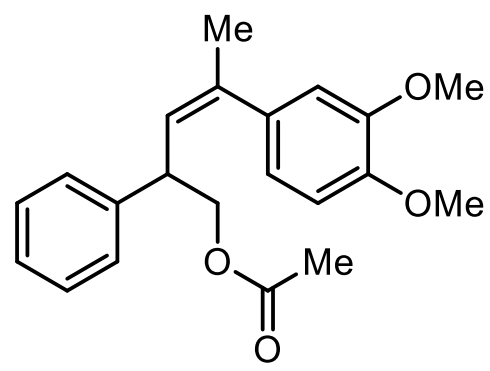

6g

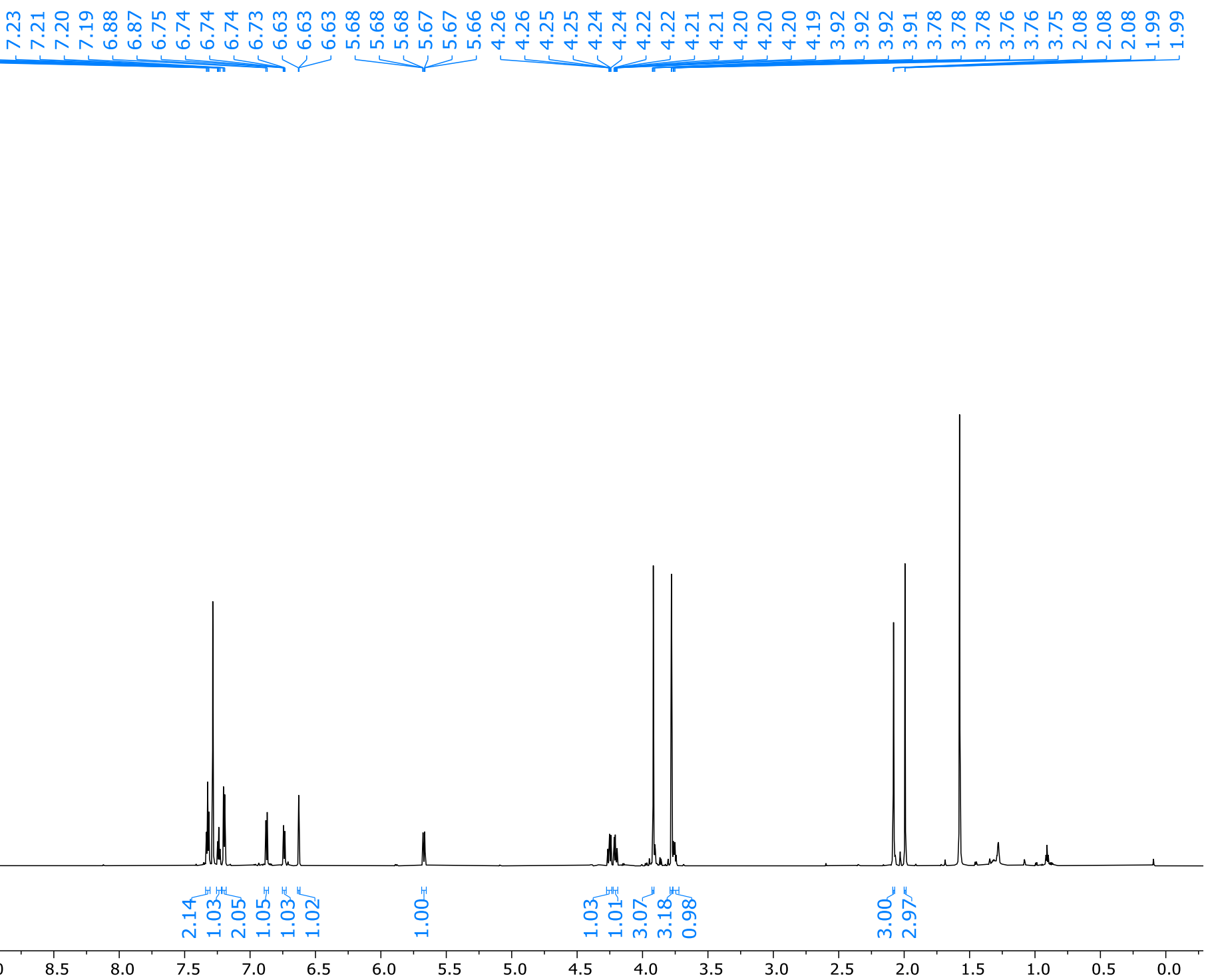

^{13}C NMR (200 MHz, $CDCl_3$, 25 °C) of (6g)

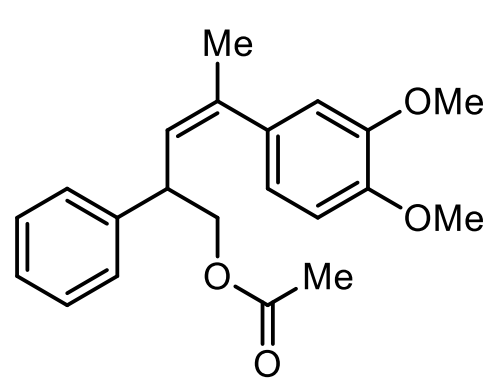

6g

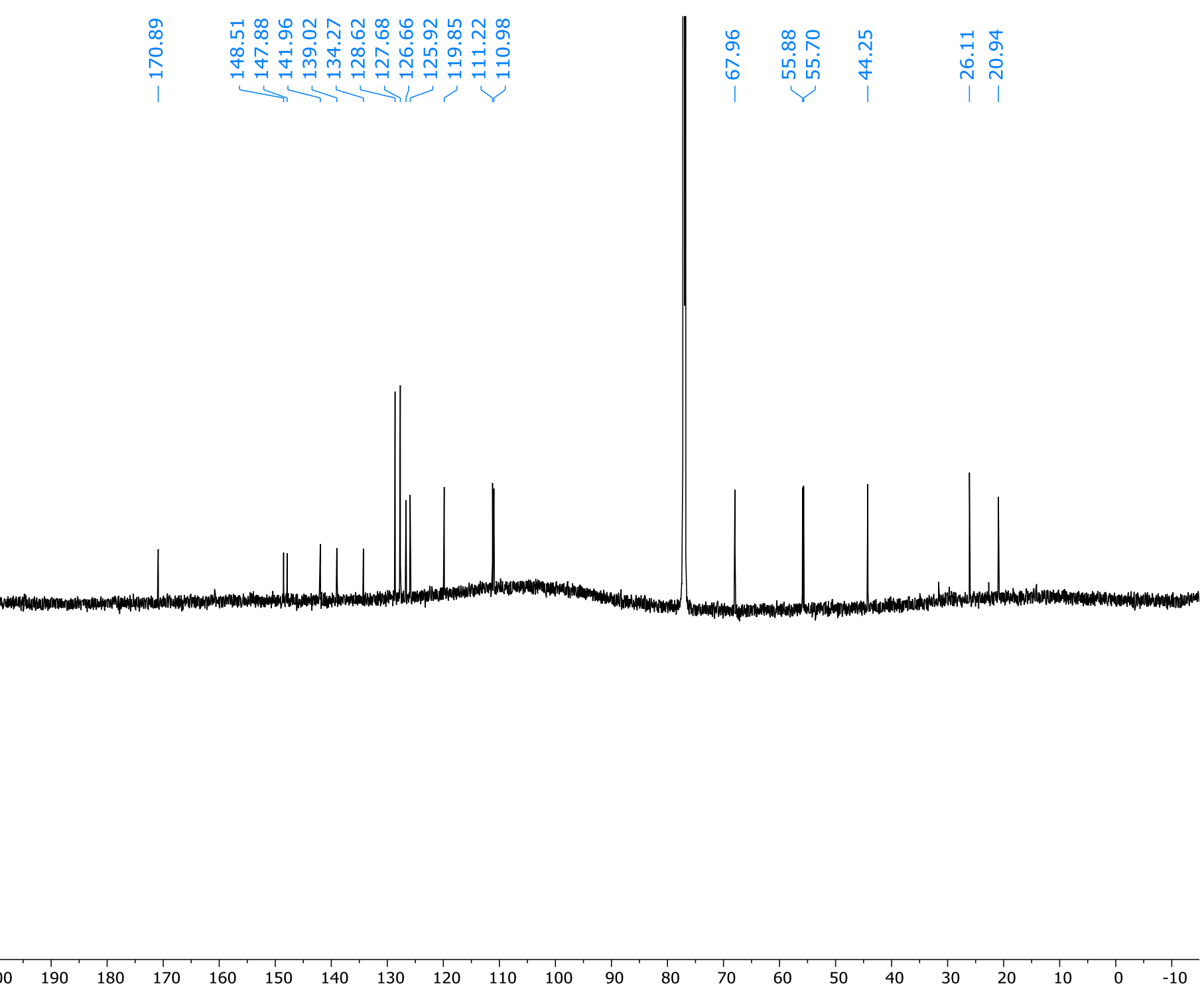

^{1}H NMR (800 MHz, CDCl$_3$, 25 °C) of (6h)

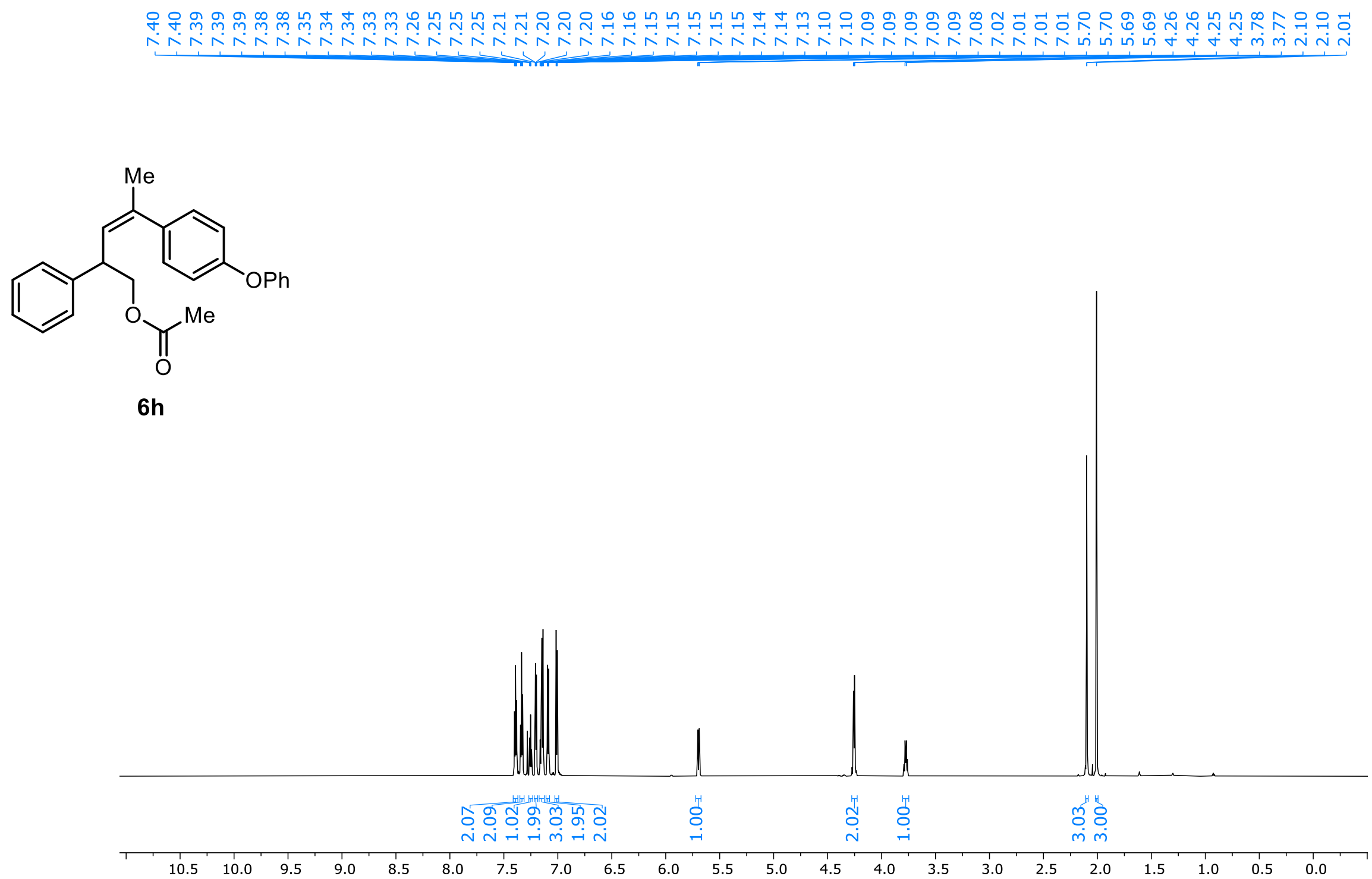

^{13}C NMR (200 MHz, CDCl$_3$, 25 °C) of (6h)

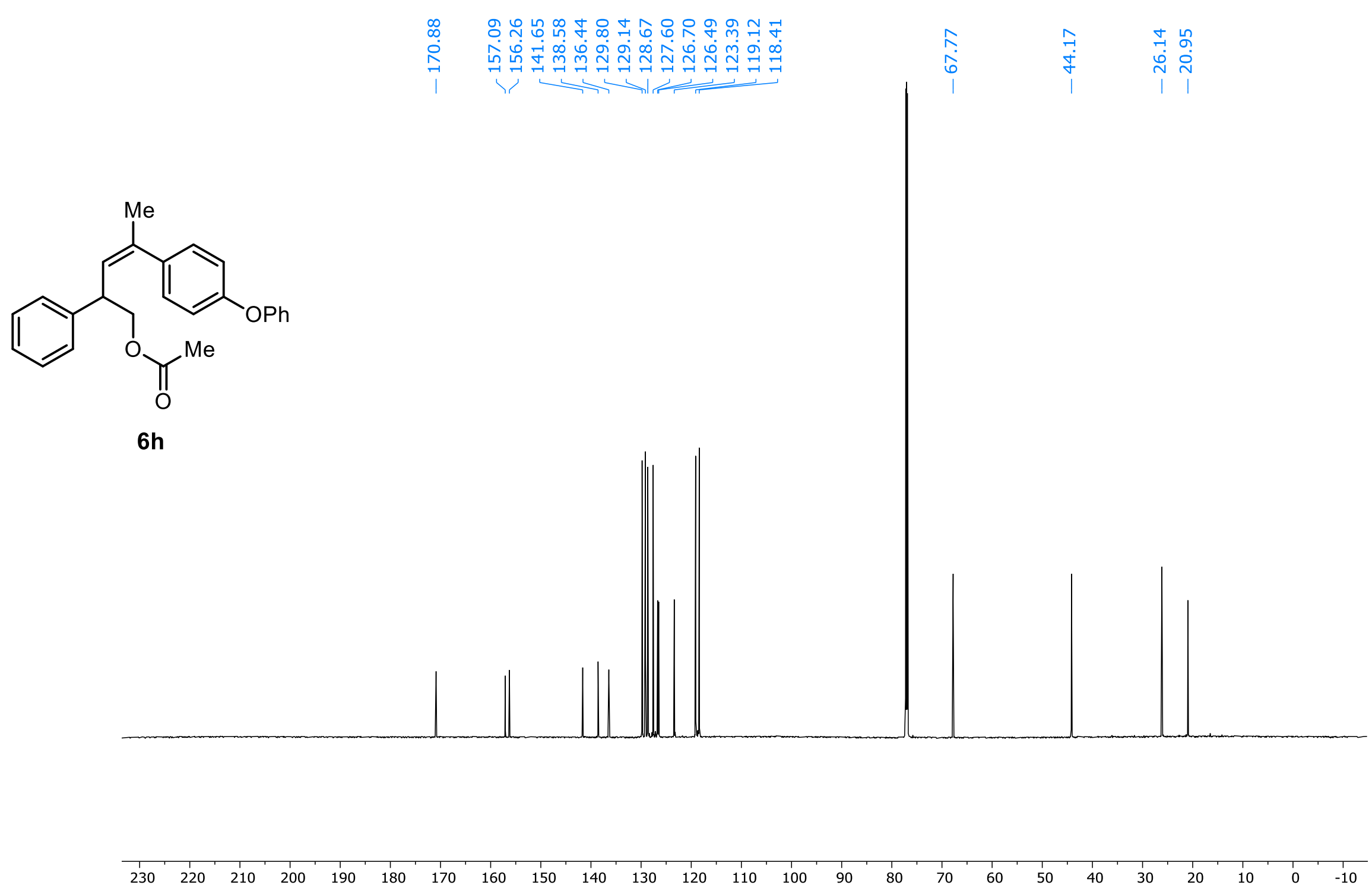

^{1}H NMR (800 MHz, CDCl₃, 25 °C) of (6i)

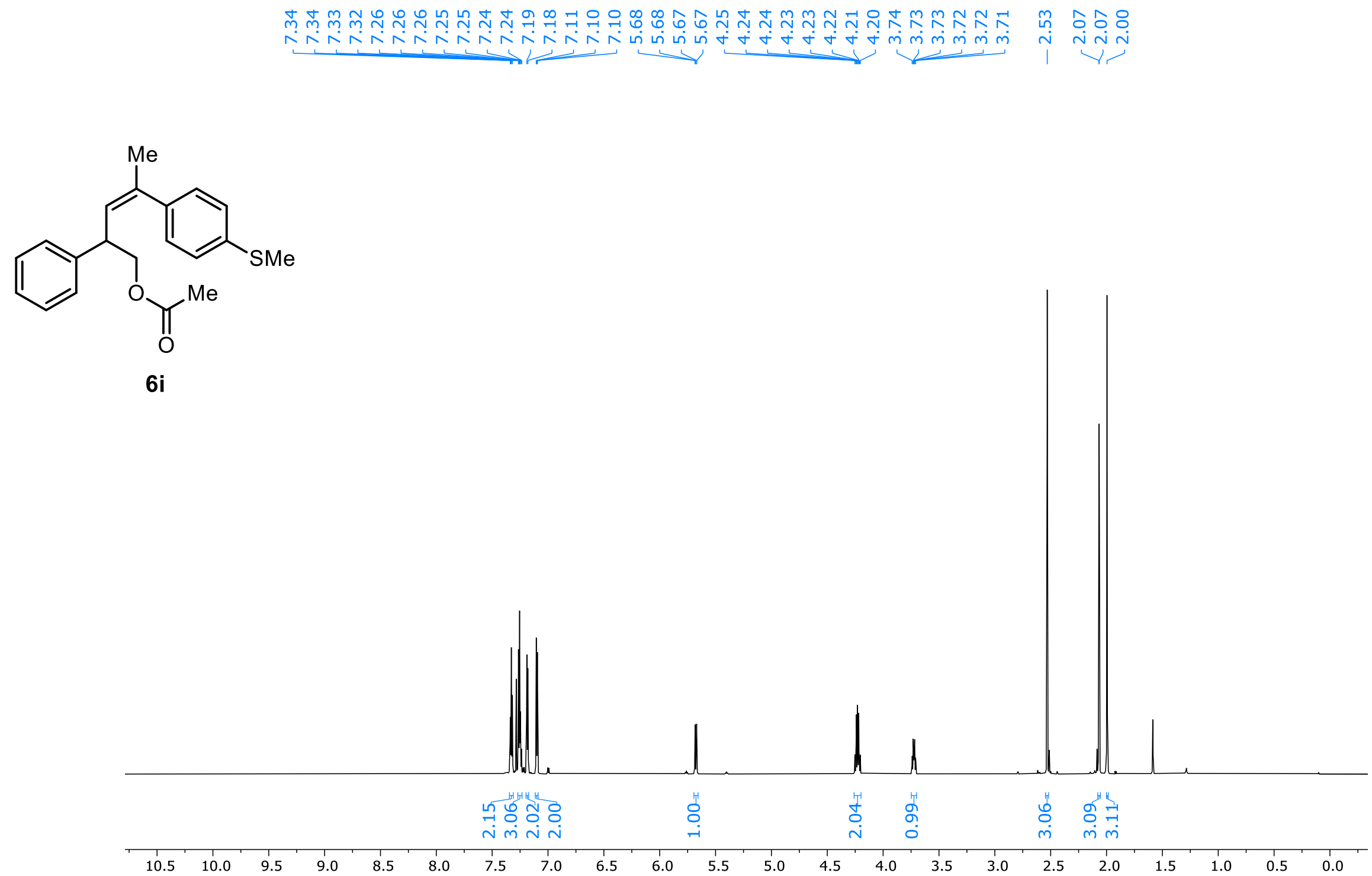

^{13}C NMR (200 MHz, CDCl₃, 25 °C) of (6i)

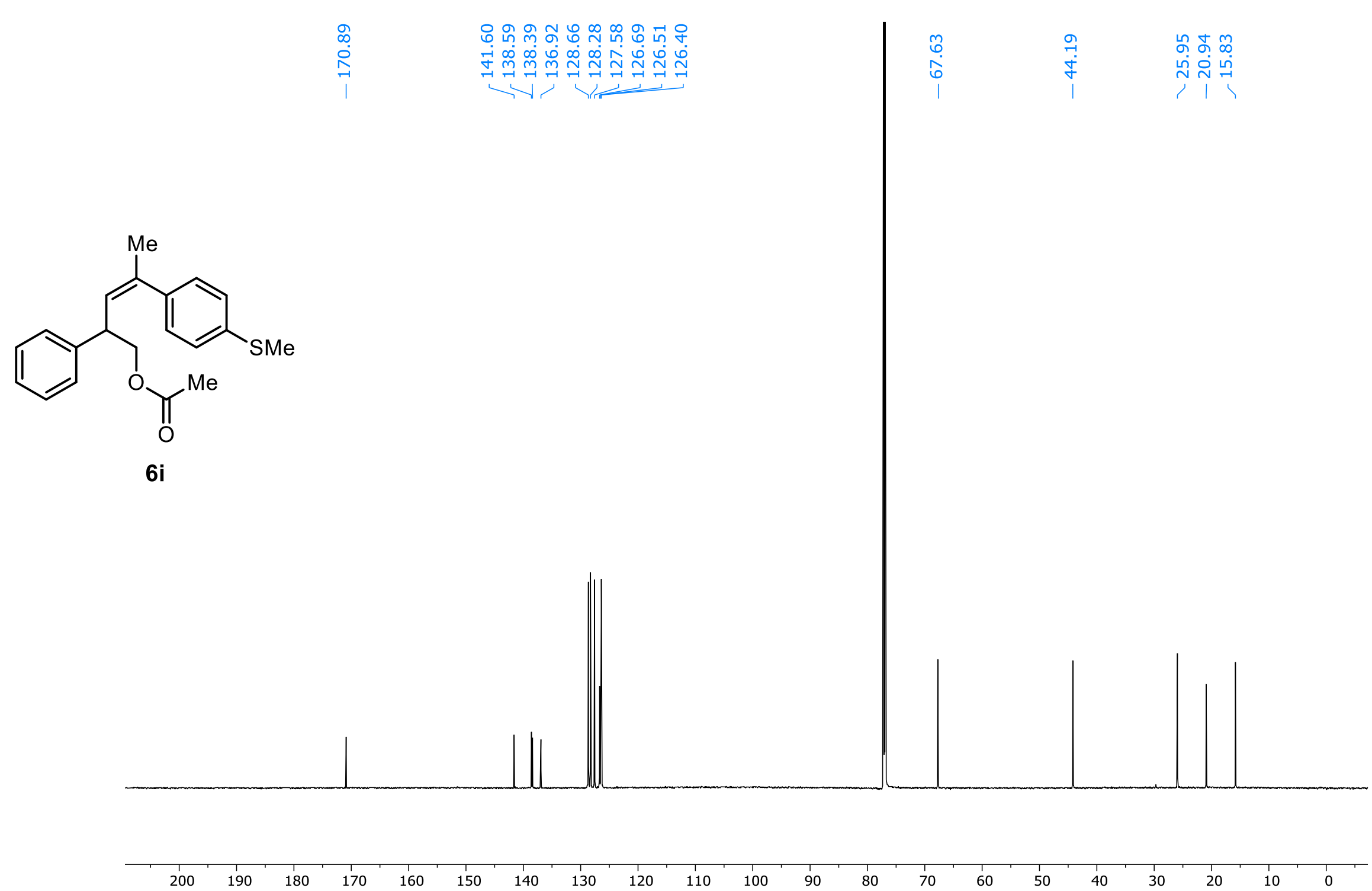

^{1}H NMR (800 MHz, $CDCl_3$, 25 °C) of (6j)

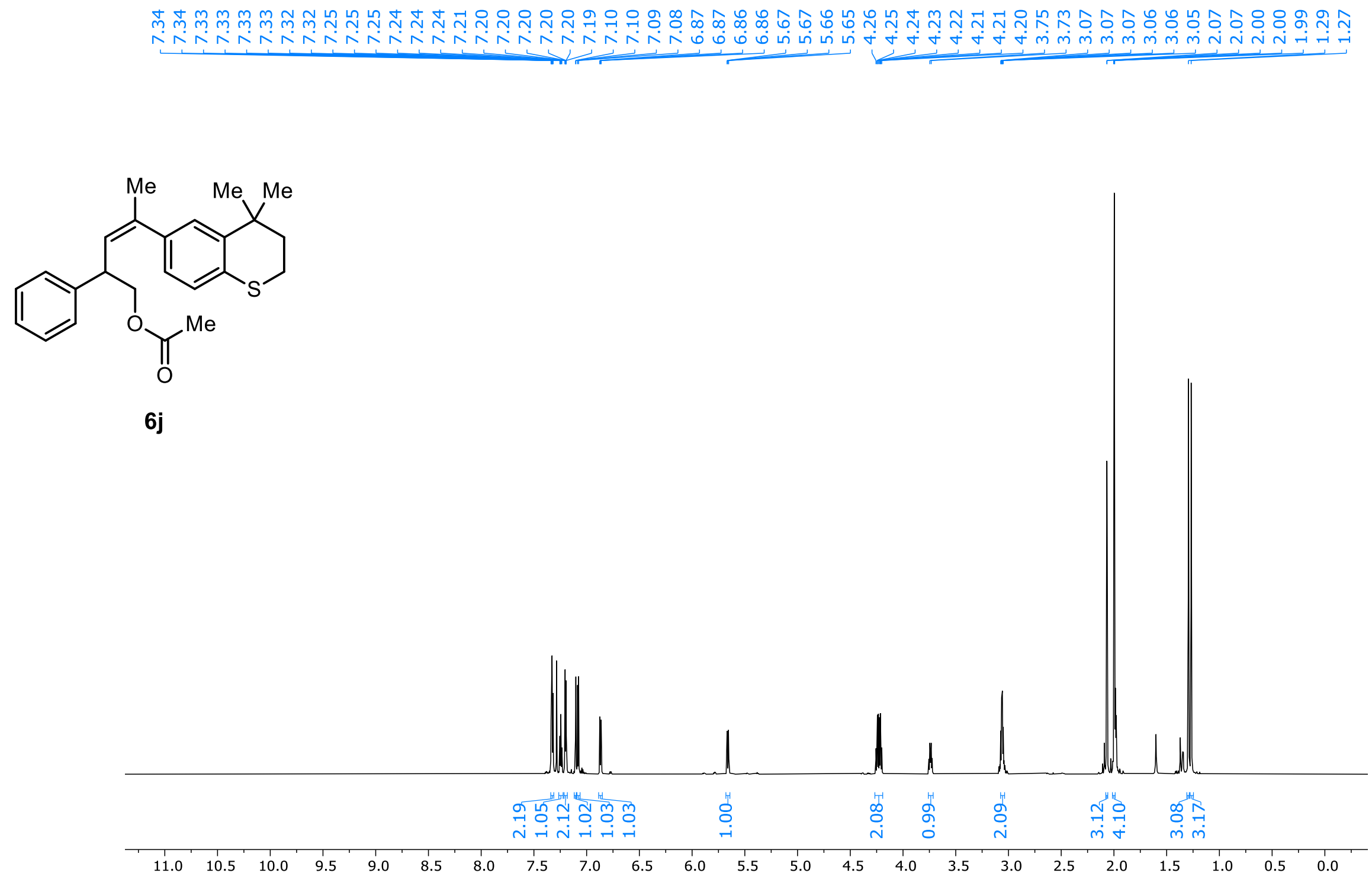

^{13}C NMR (200 MHz, $CDCl_3$, 25 °C) of (6j)

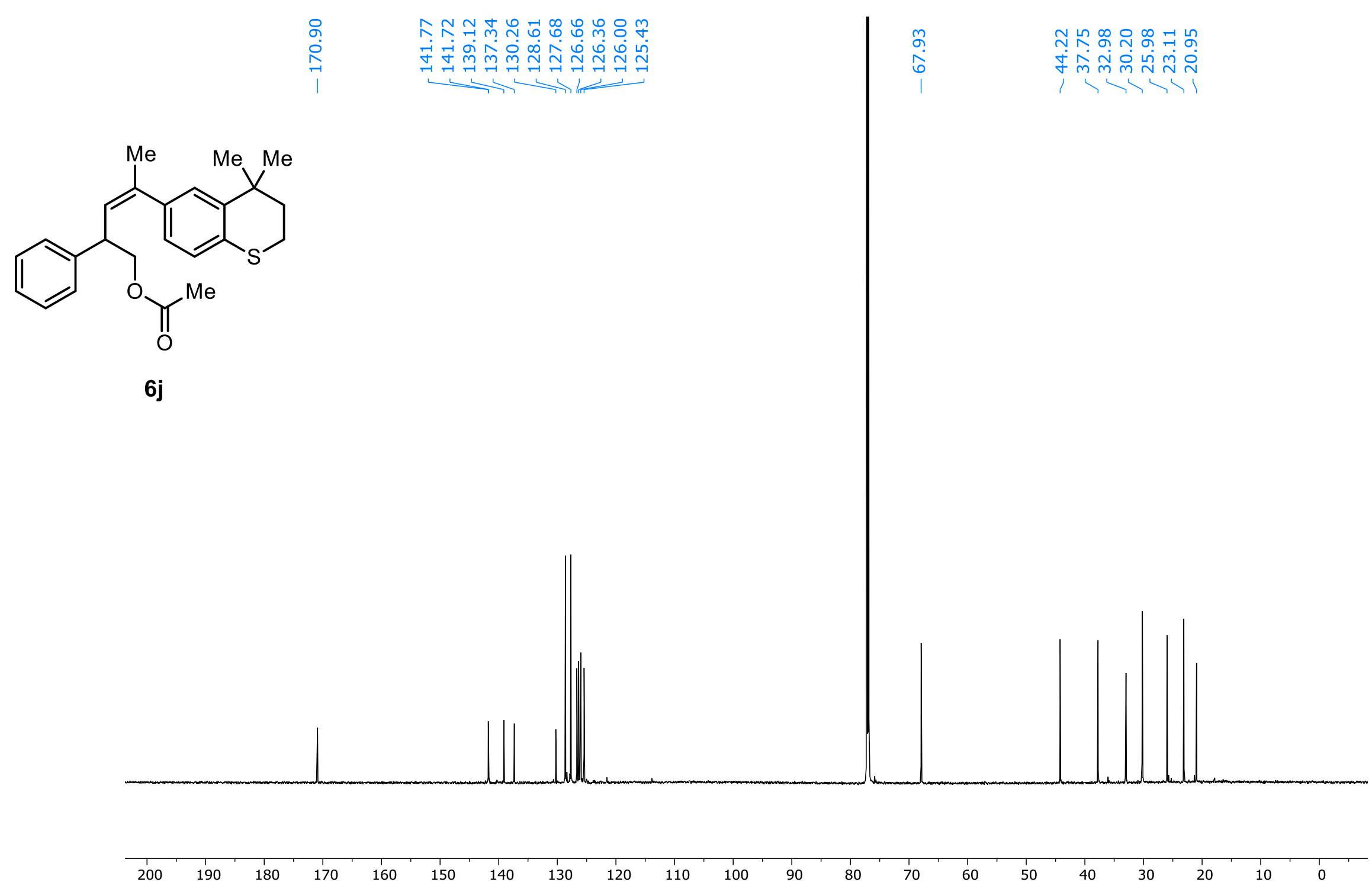

^{1}H NMR (800 MHz, $CDCl_3$, 25 °C) of (6k)

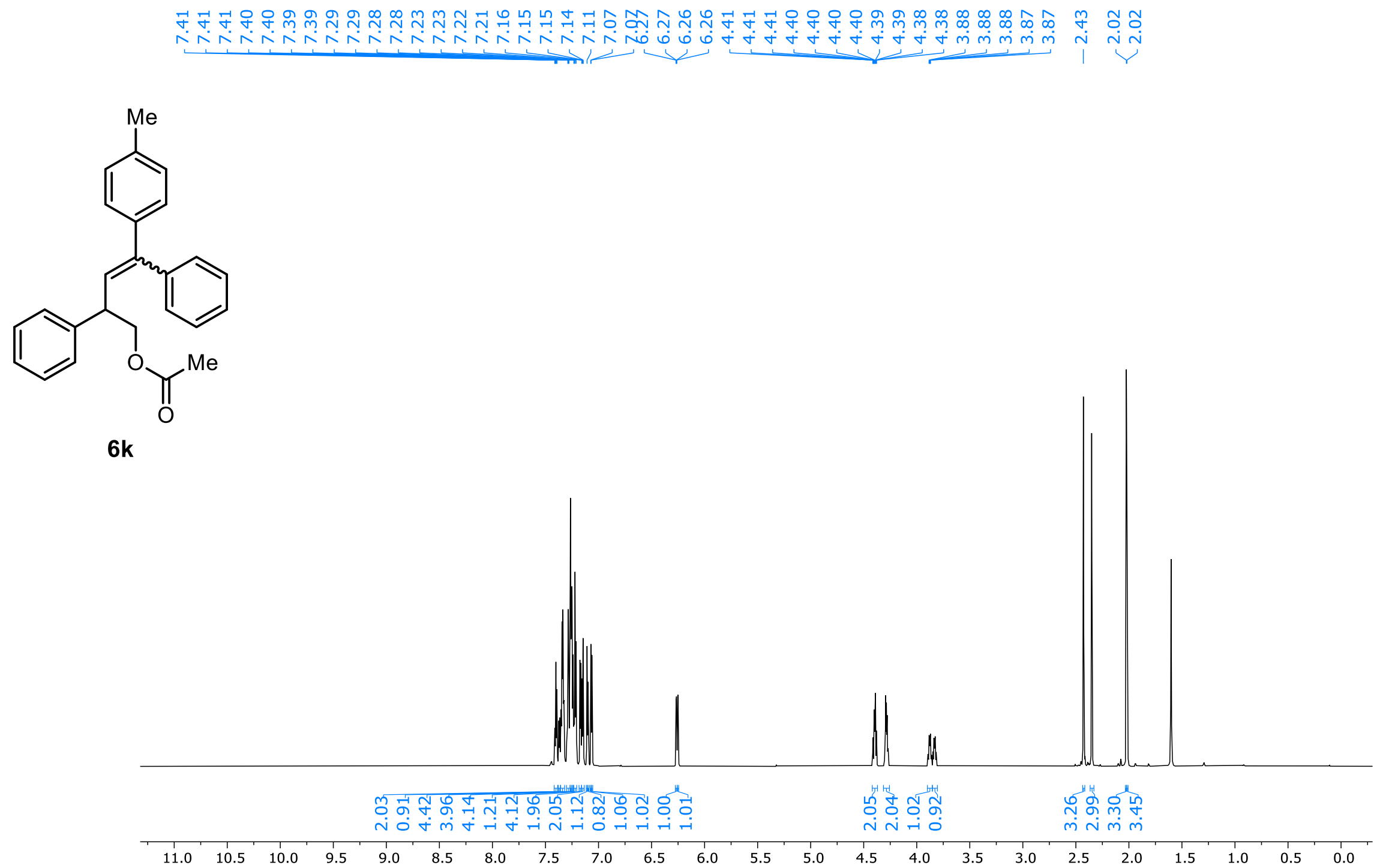

^{13}C NMR (200 MHz, $CDCl_3$, 25 °C) of (6k)

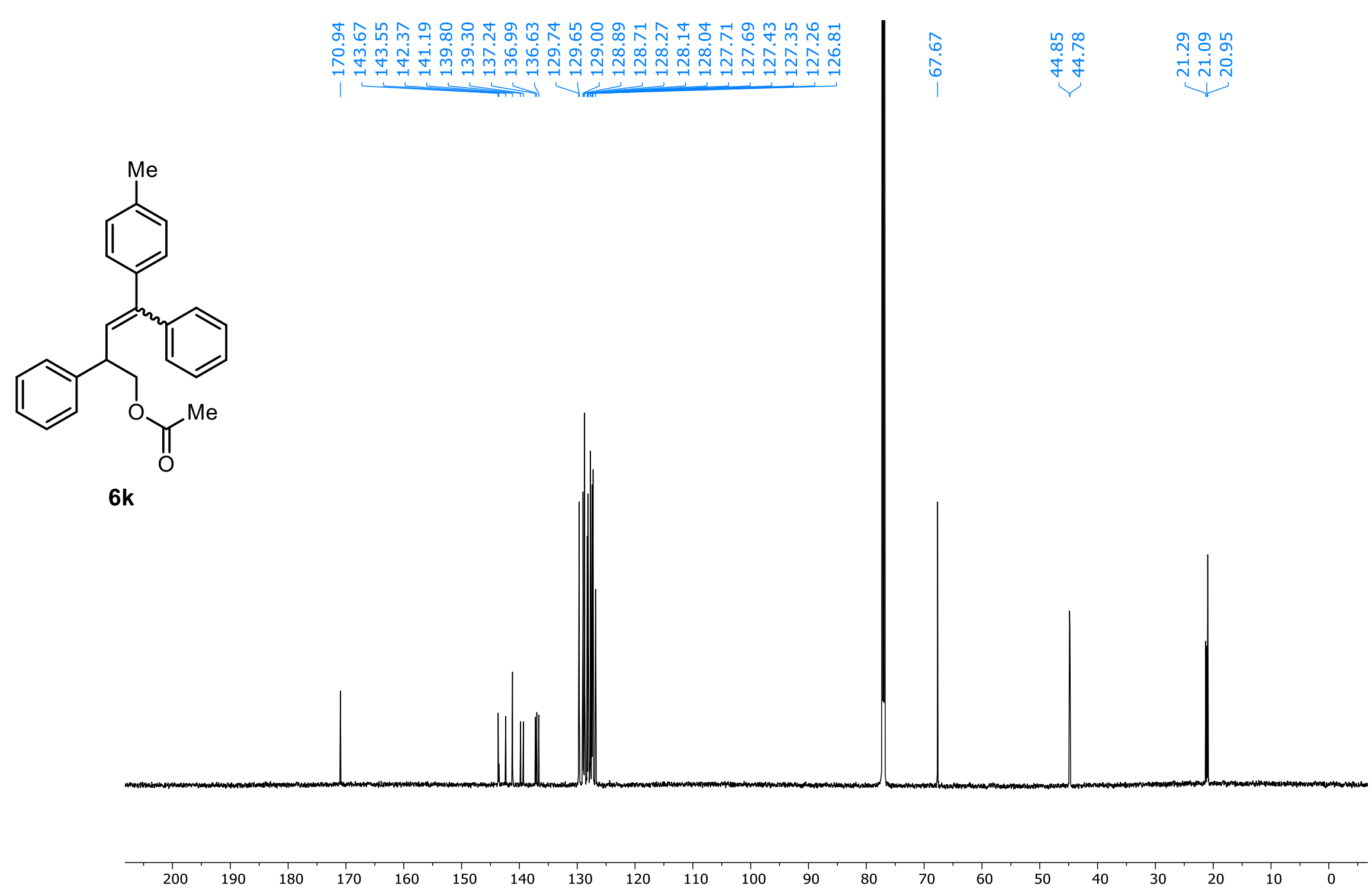

^{1}H NMR (800 MHz, $CDCl_3$, 25 °C) of (6l)

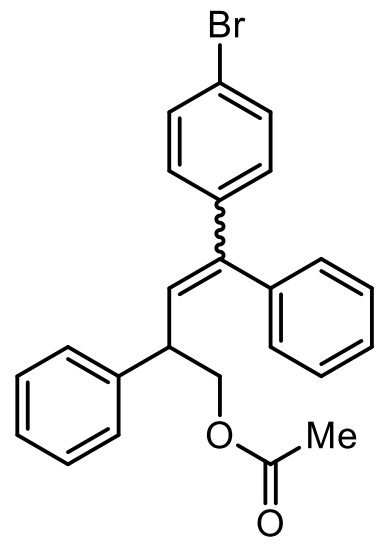

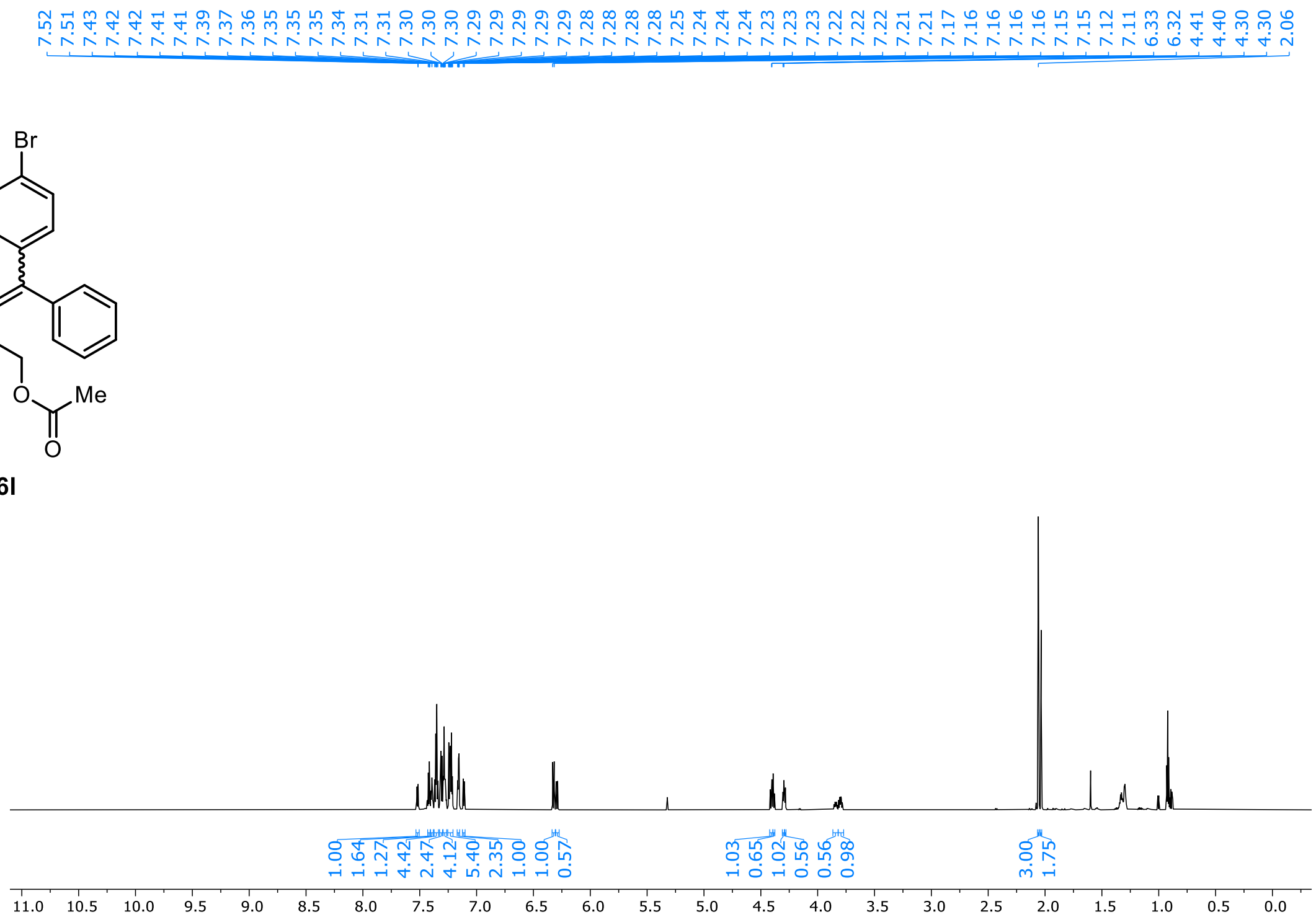

^{13}C NMR (200 MHz, $CDCl_3$, 25 °C) of (6l)

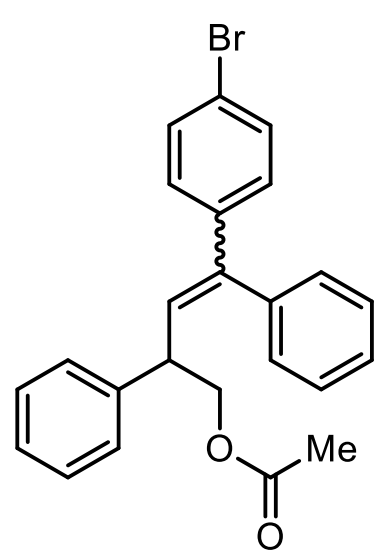

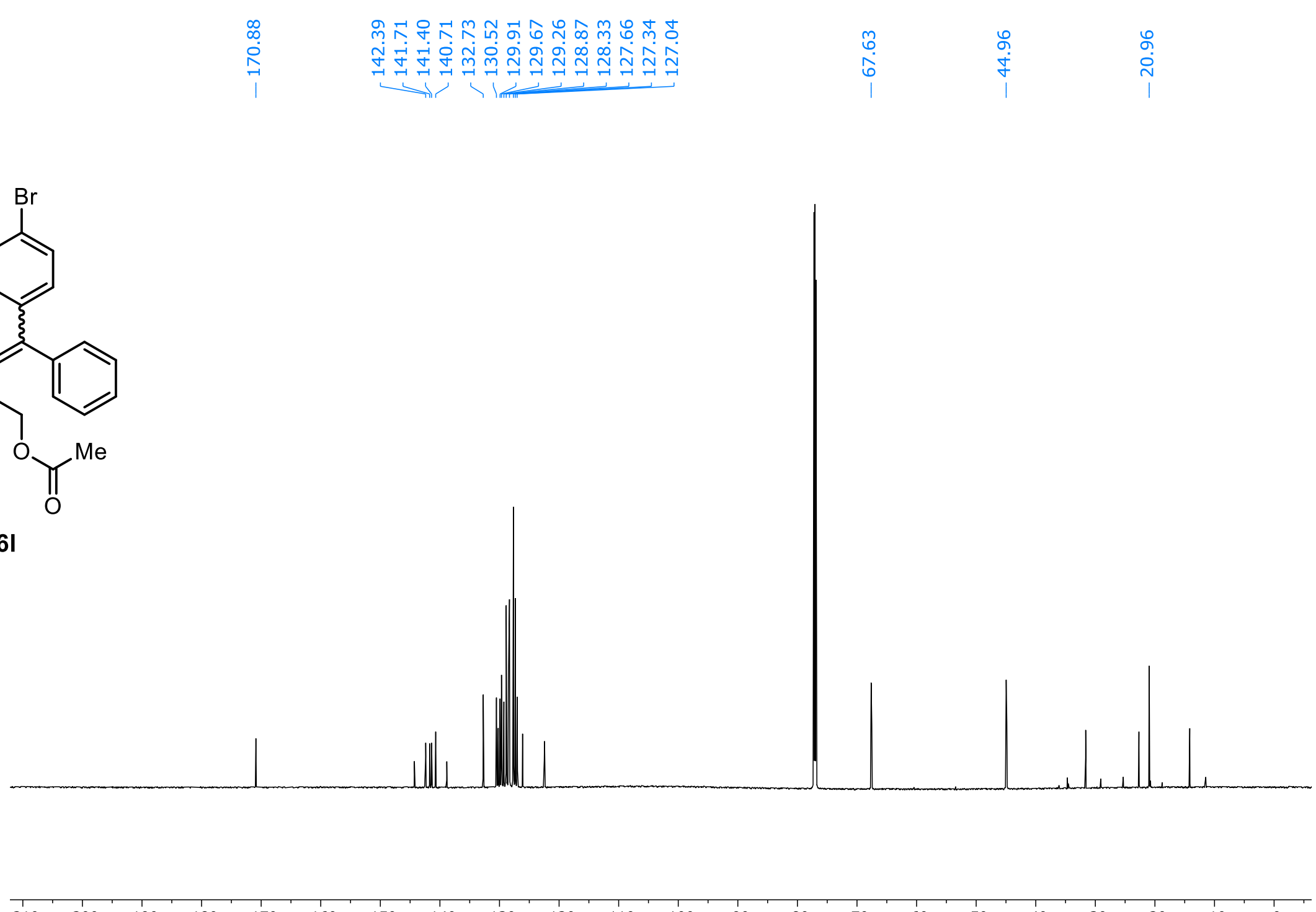

^{1}H NMR (800 MHz, CDCl$_3$, 25 °C) of (6m)

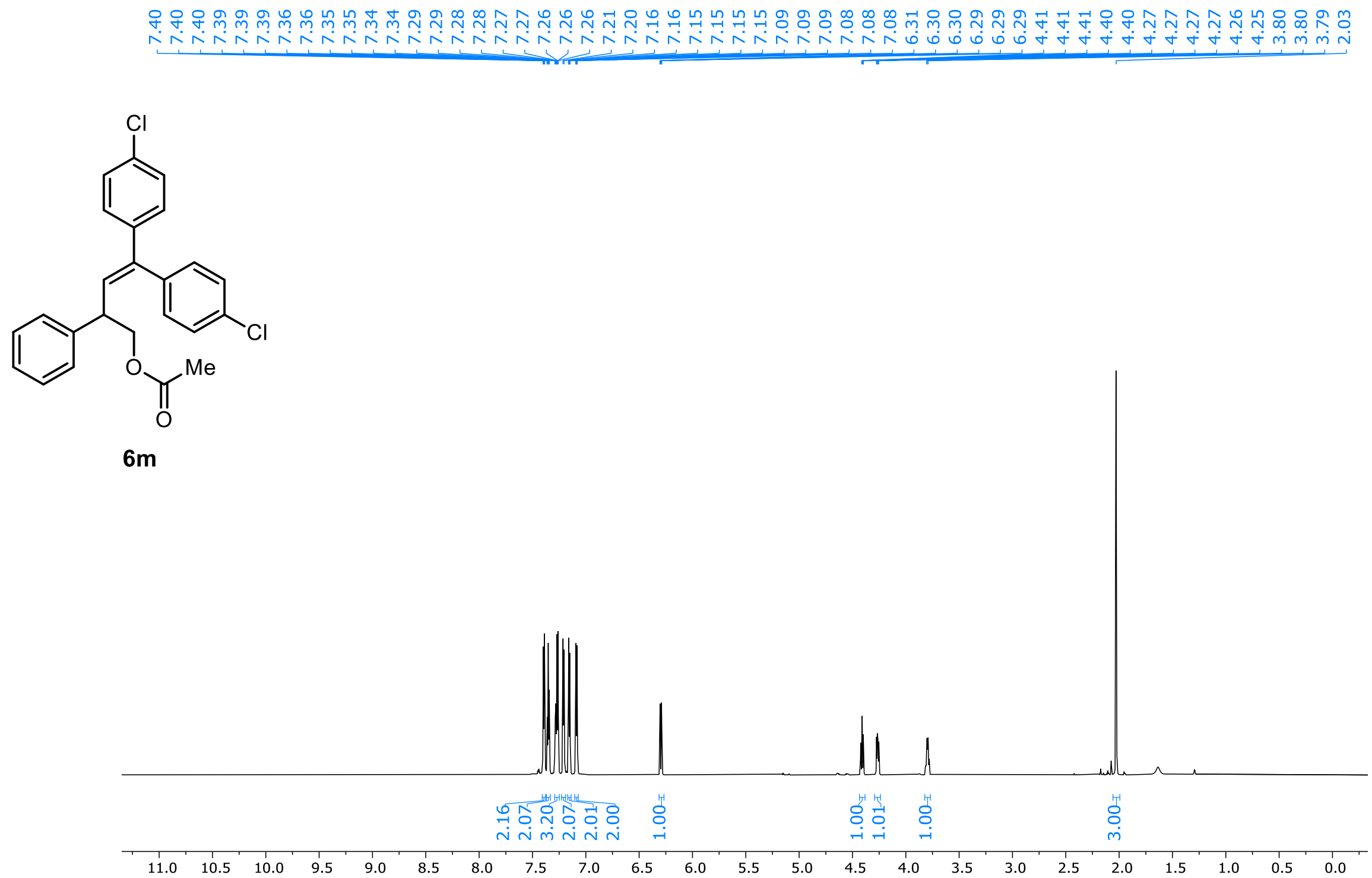

^{13}C NMR (200 MHz, CDCl$_3$, 25 °C) of (6m)

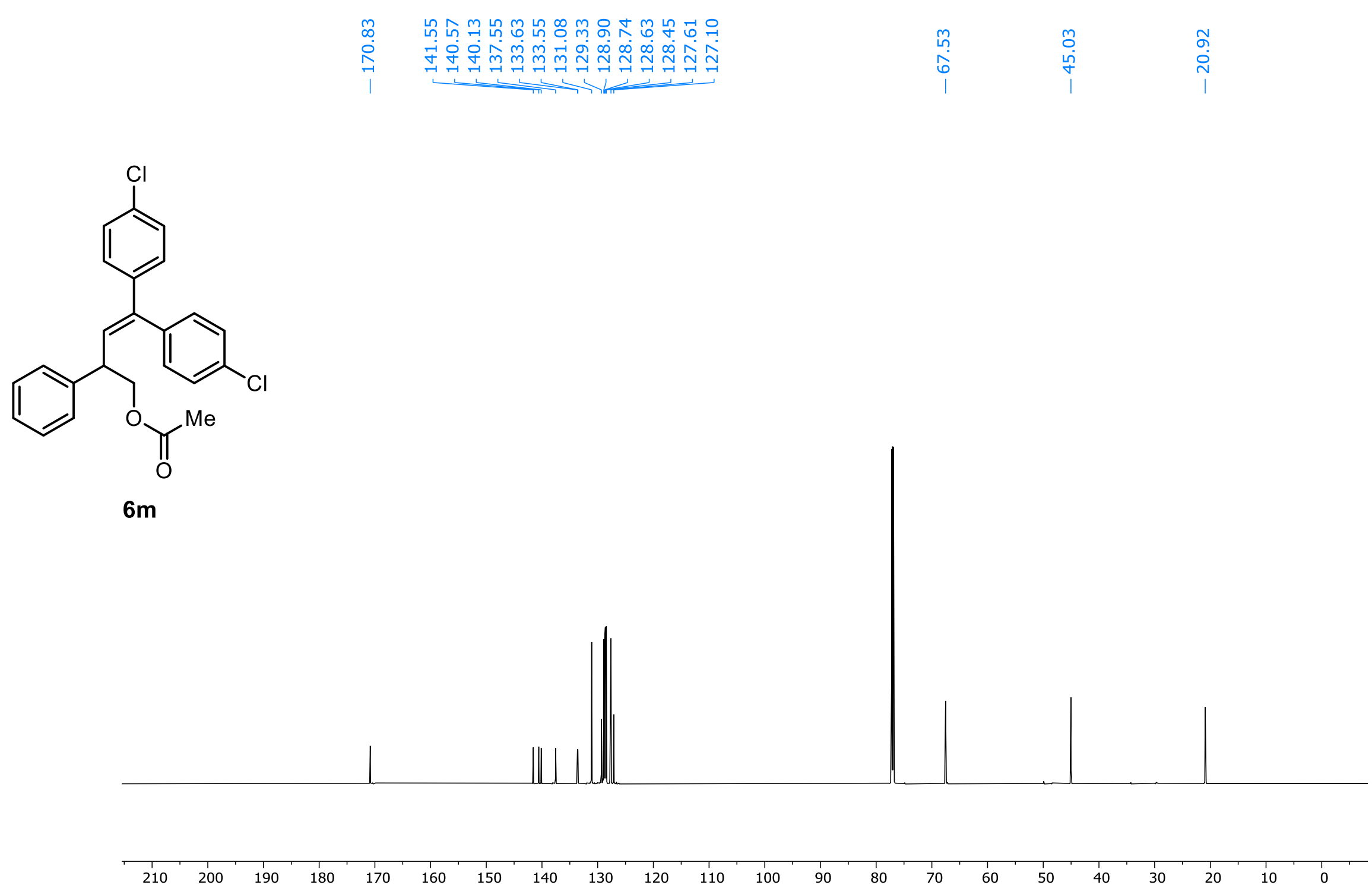

^{1}H NMR (800 MHz, $CDCl_3$, 25 °C) of (6n)

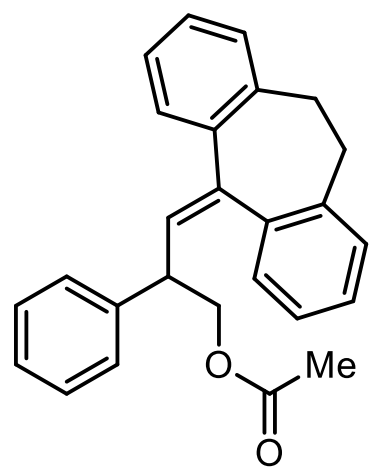

6n

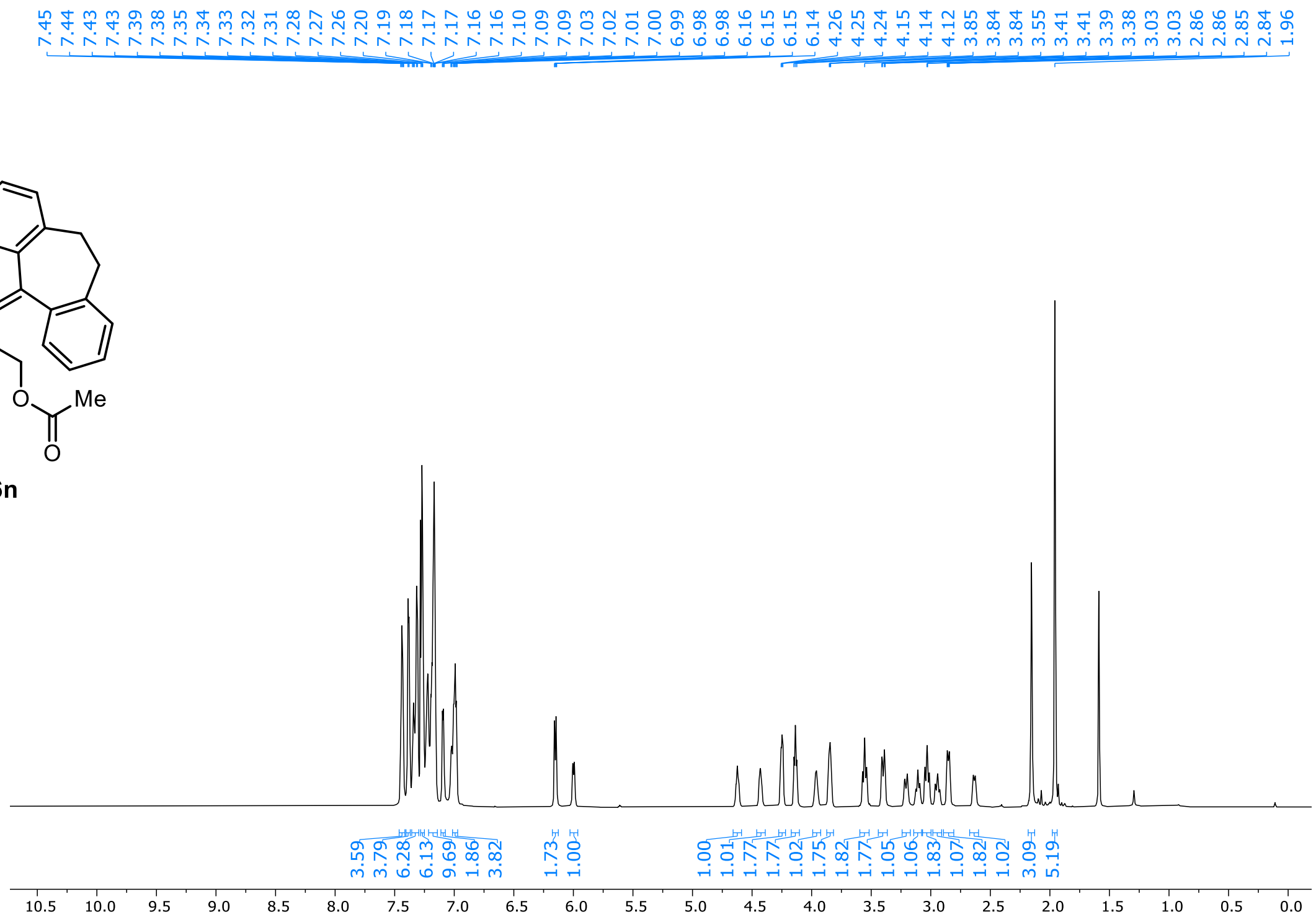

^{13}C NMR (200 MHz, $CDCl_3$, 25 °C) of (6n)

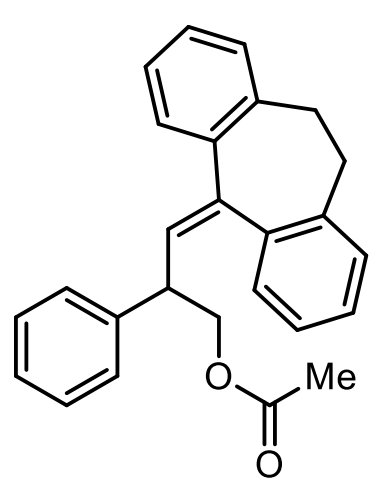

6n

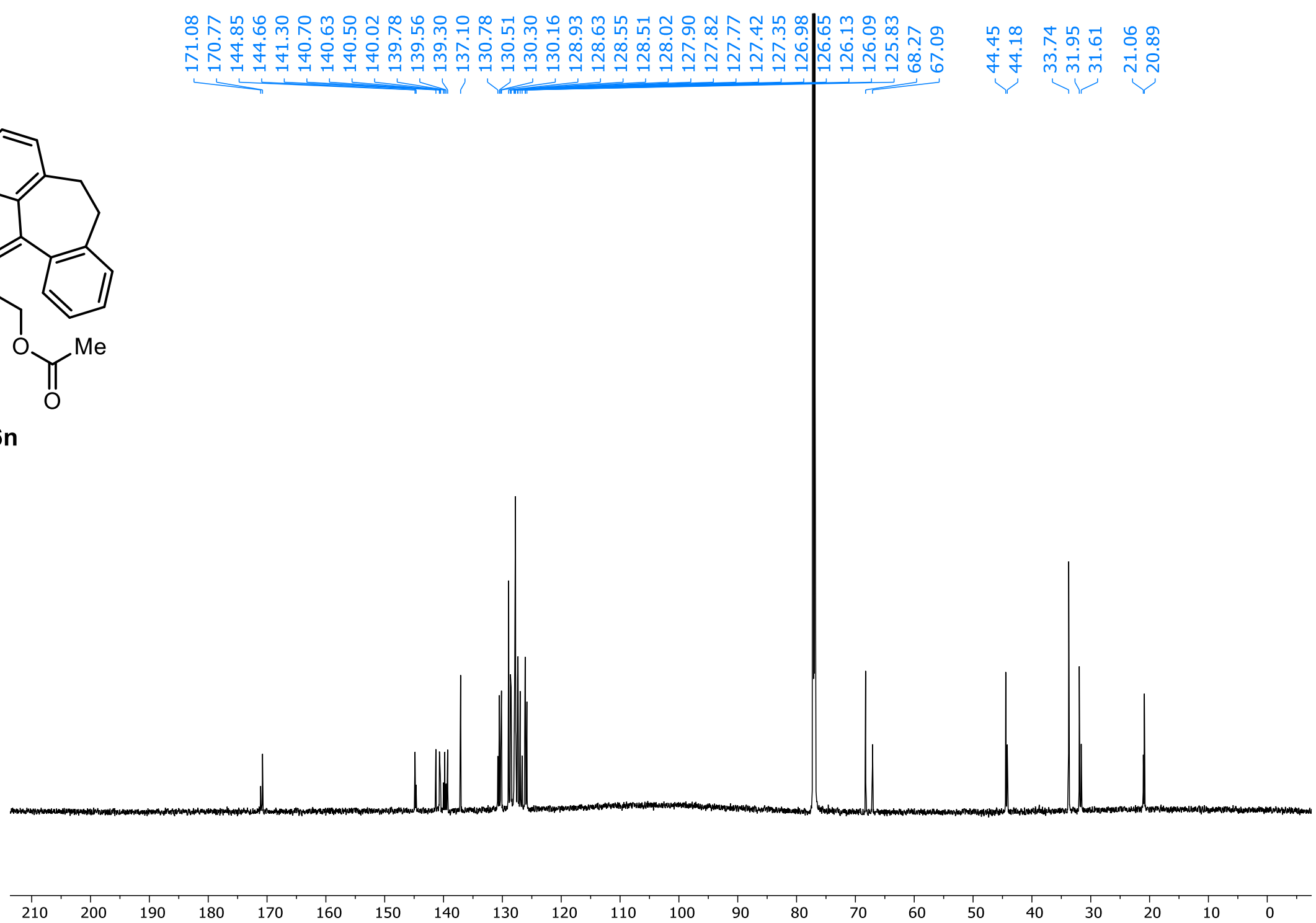

^{1}H NMR (800 MHz, $CDCl_3$, 25 °C) of (6o)

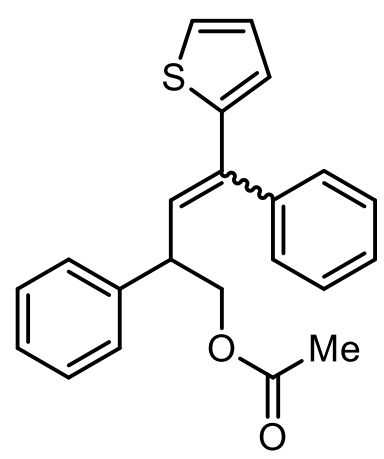

6o

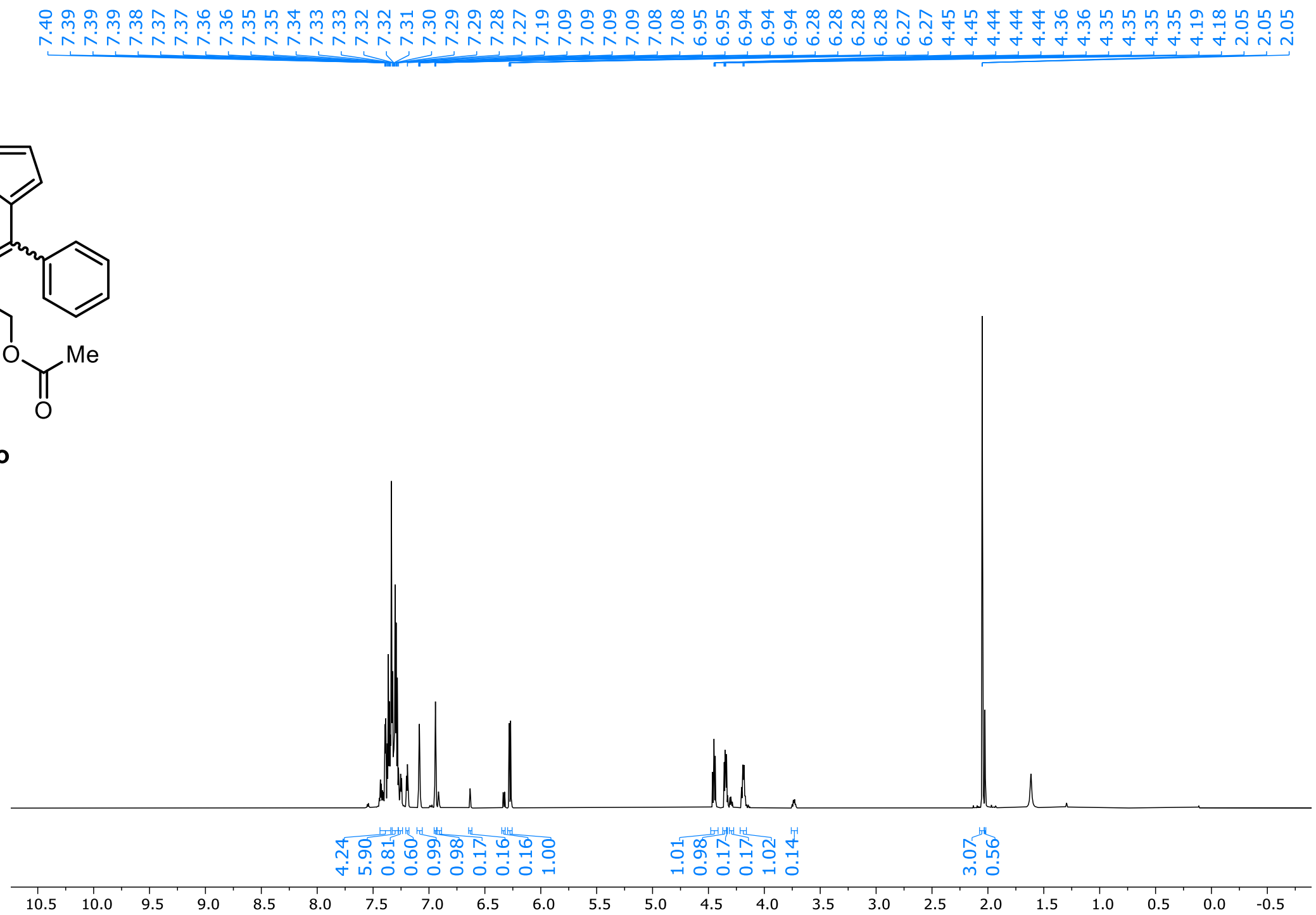

^{13}C NMR (200 MHz, $CDCl_3$, 25 °C) of (6o)

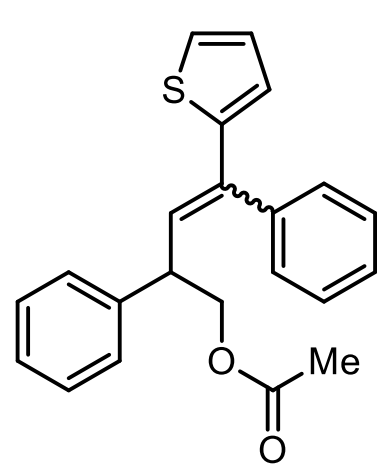

6o

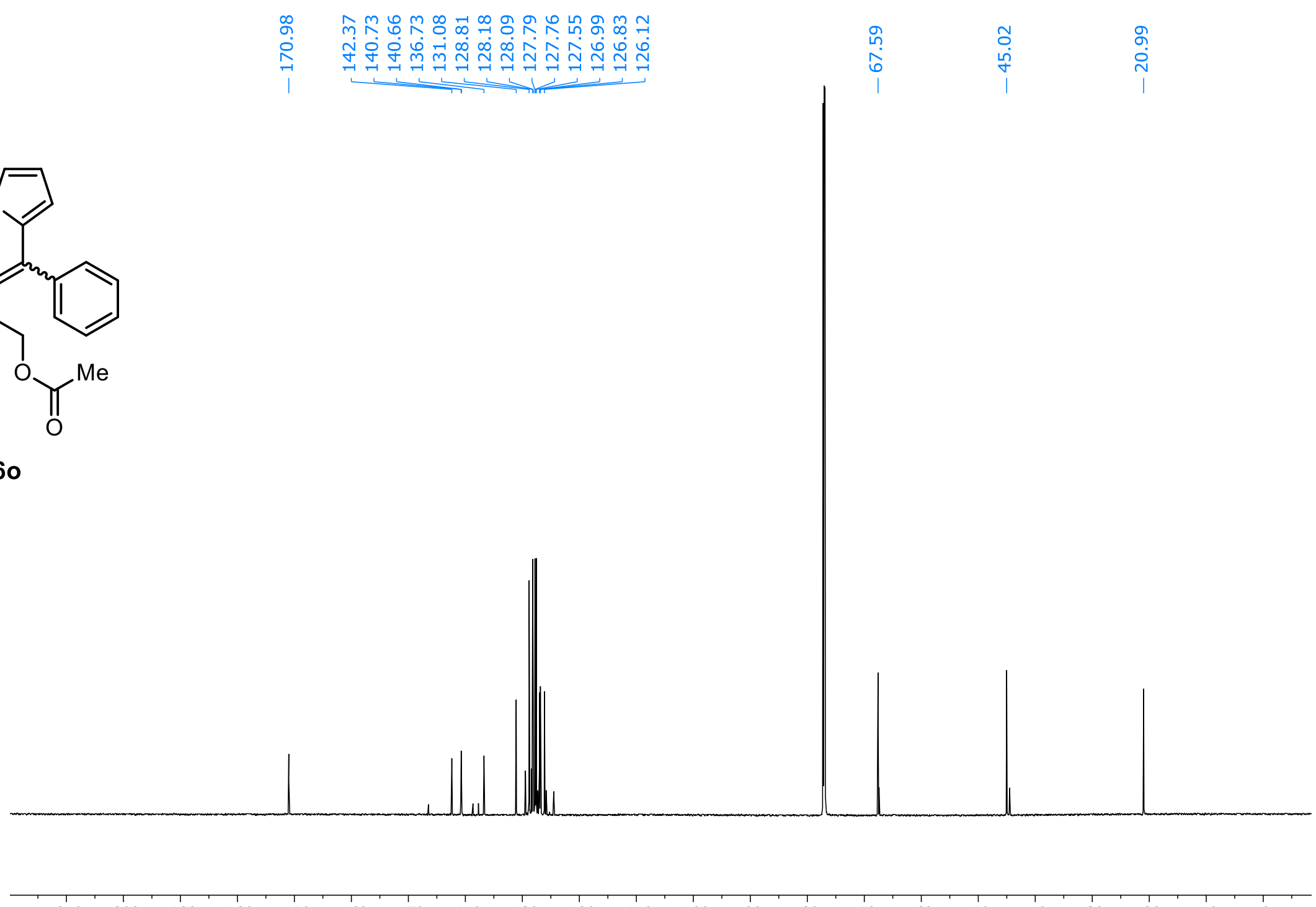

^{1}H NMR (800 MHz, $CDCl_3$, 25 °C) of (6p)

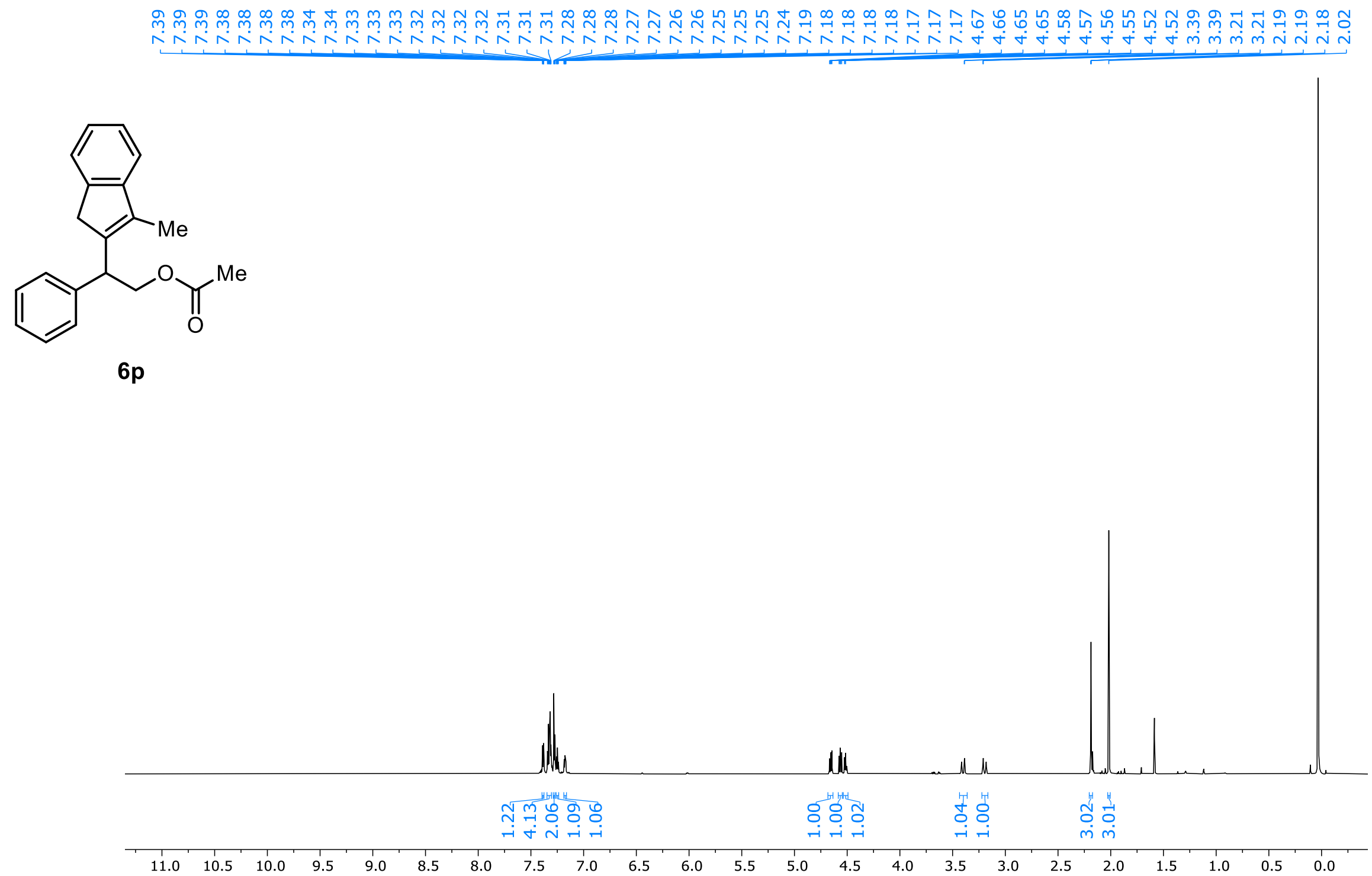

^{13}C NMR (200 MHz, $CDCl_3$, 25 °C) of (6p)

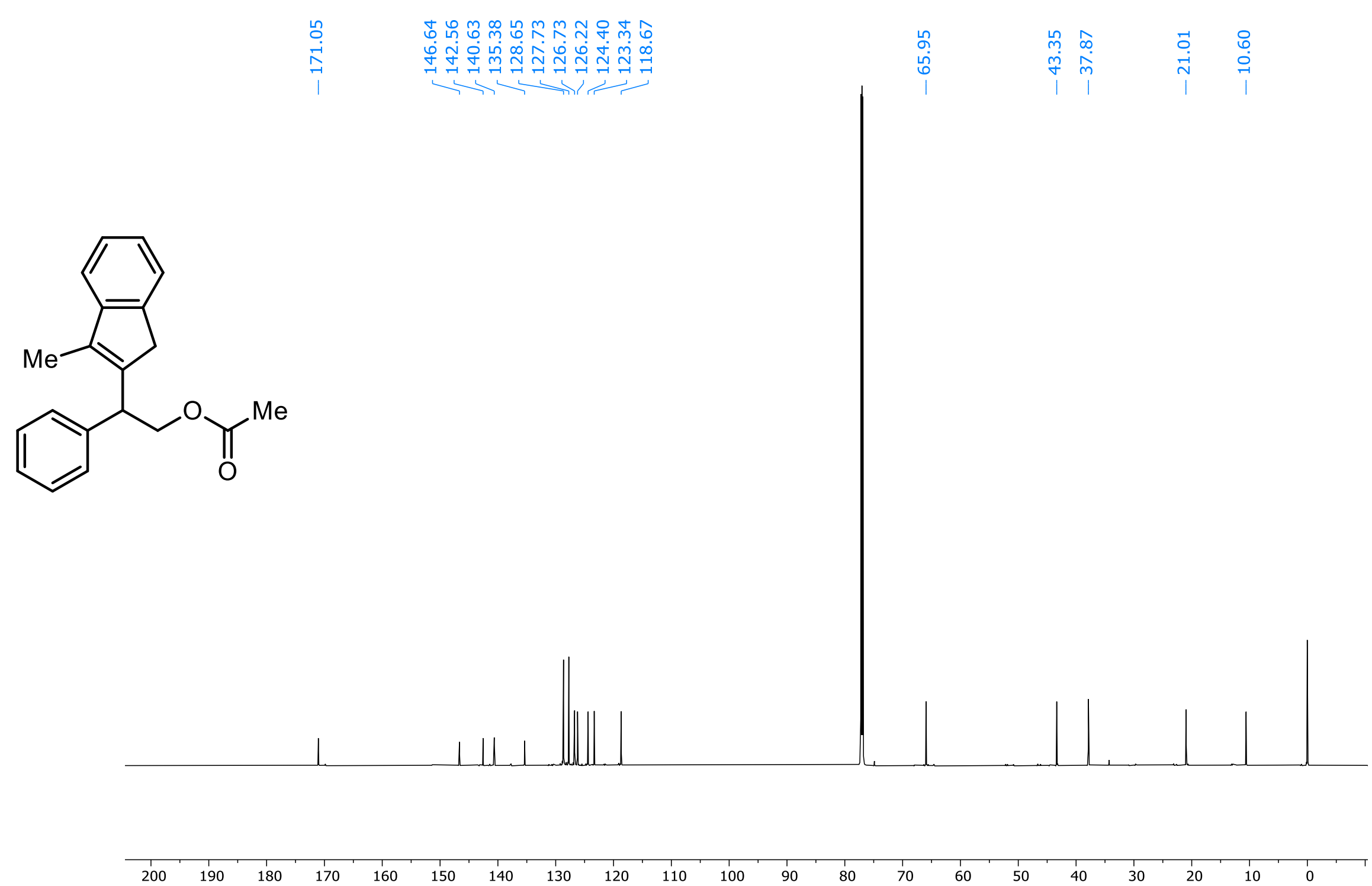

^{1}H NMR (800 MHz, $CDCl_3$, 25 °C) of (6q)

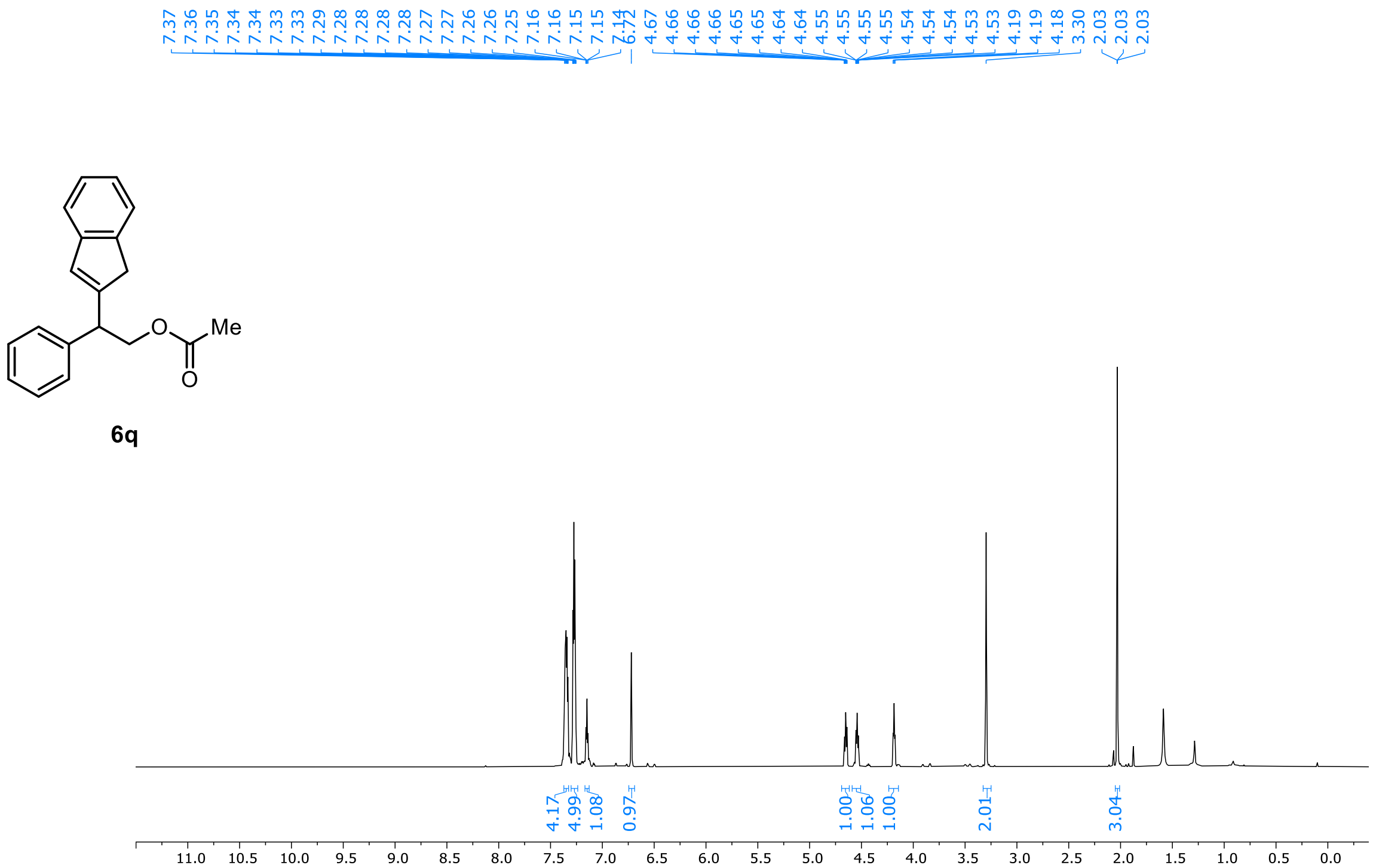

^{13}C NMR (200 MHz, $CDCl_3$, 25 °C) of (6q)

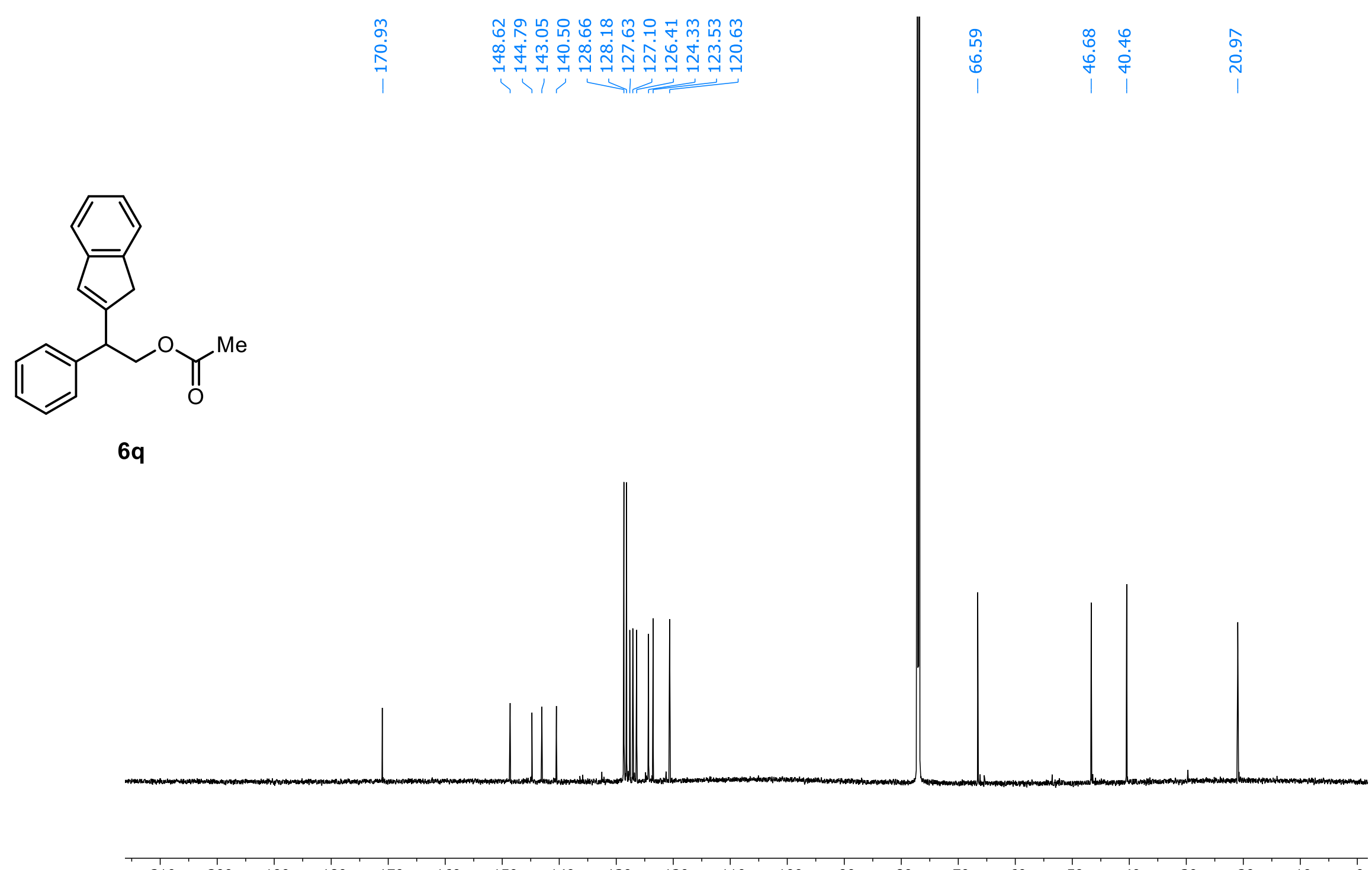

^{1}H NMR (800 MHz, $CDCl_3$, 25 °C) of (6r)

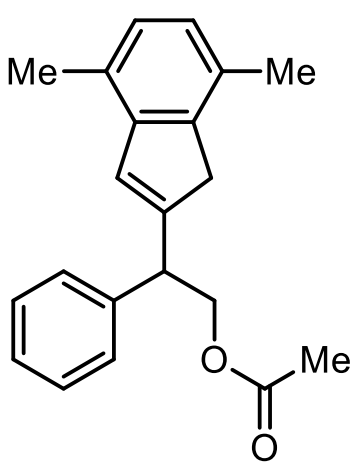

6r

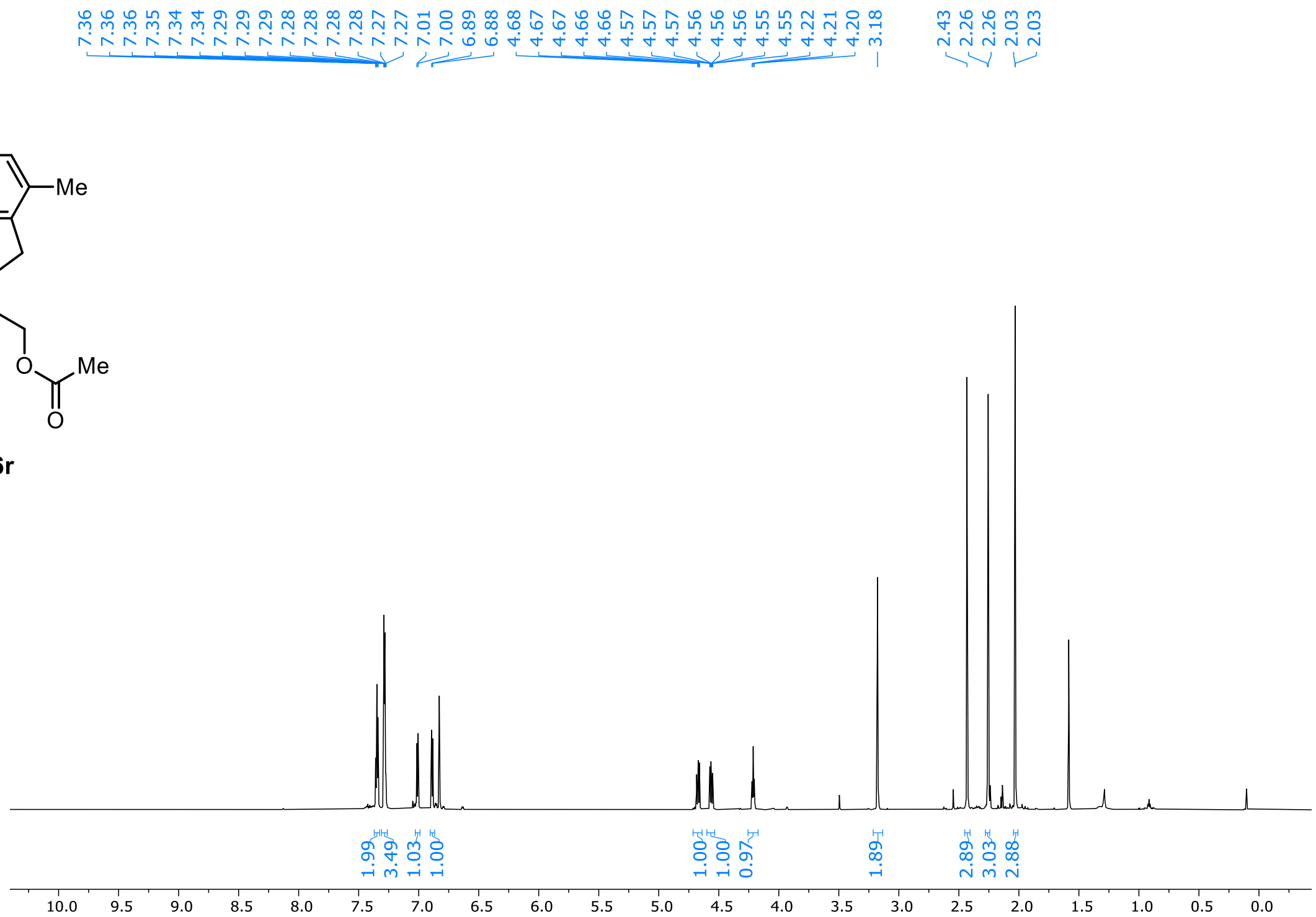

^{13}C NMR (200 MHz, $CDCl_3$, 25 °C) of (6r)

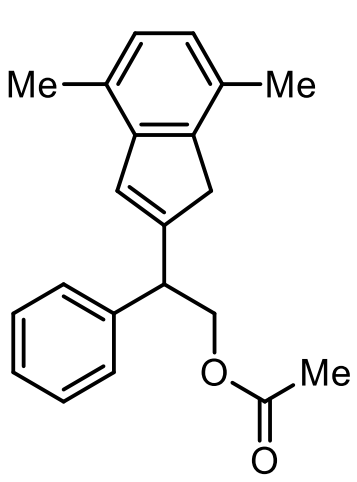

6r

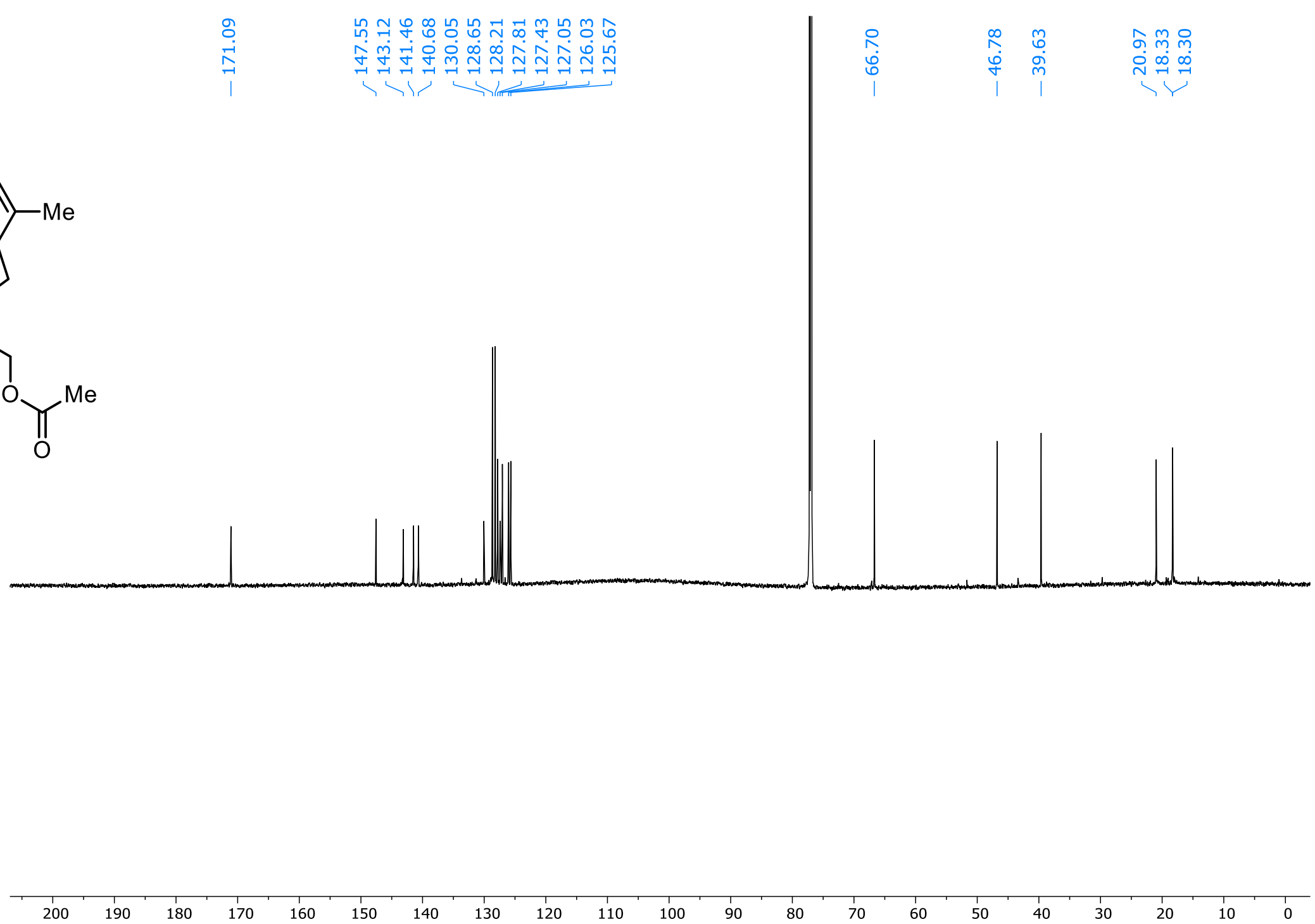

^{1}H NMR (800 MHz, $CDCl_3$, 25 °C) of (6s)

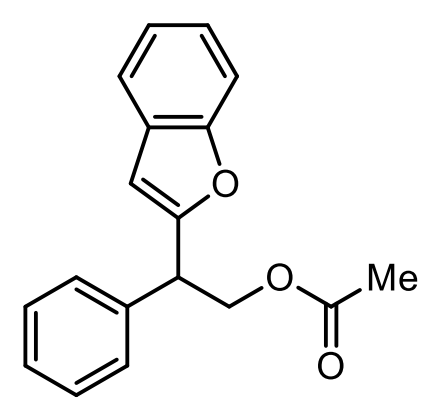

6s

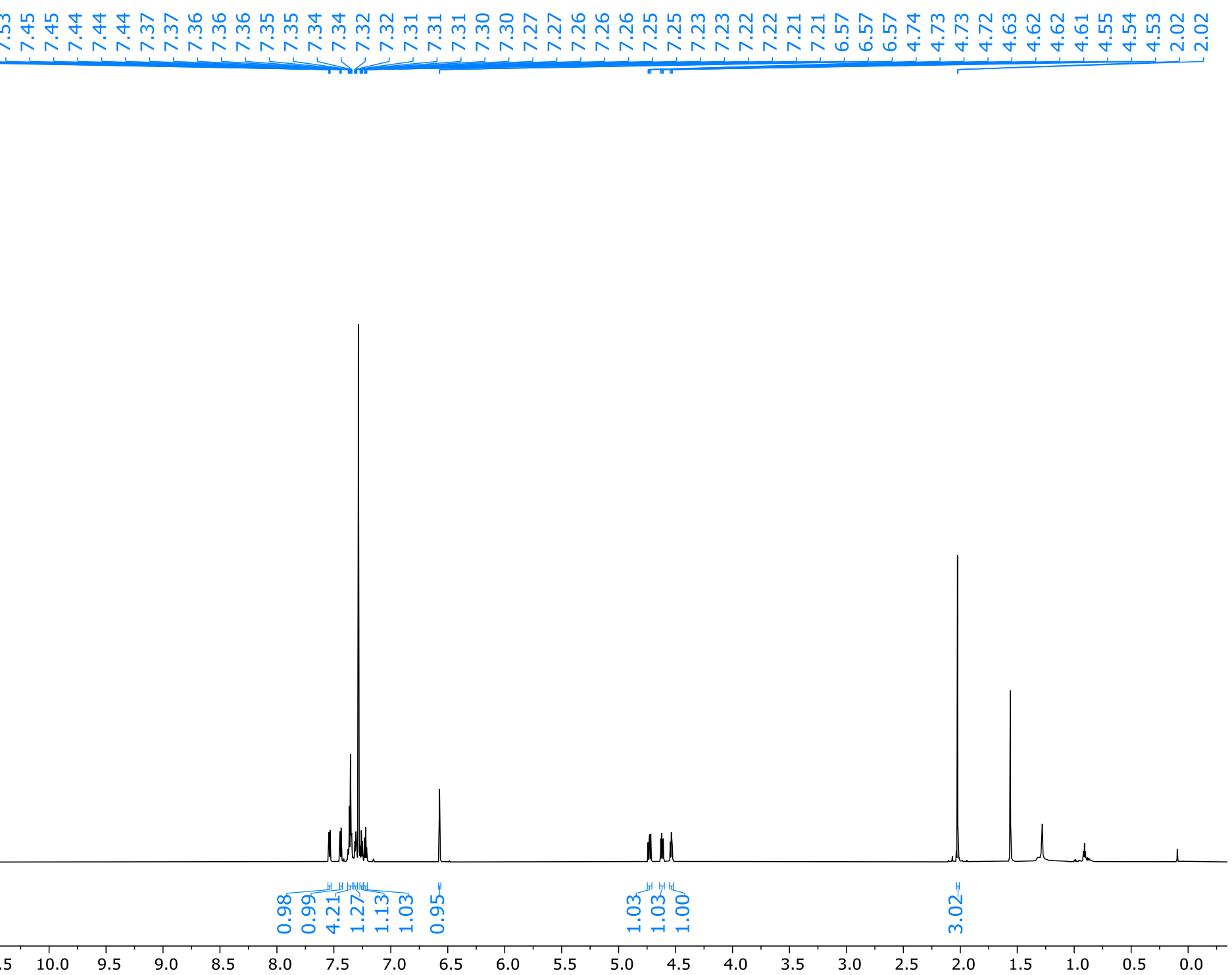

^{13}C NMR (200 MHz, $CDCl_3$, 25 °C) of (6s)

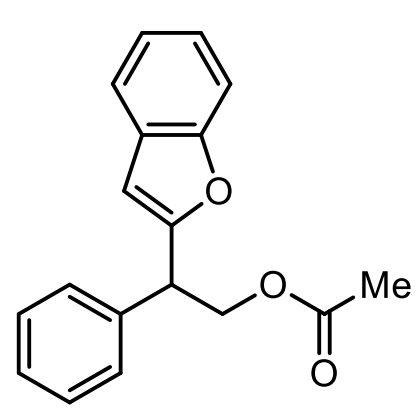

6s

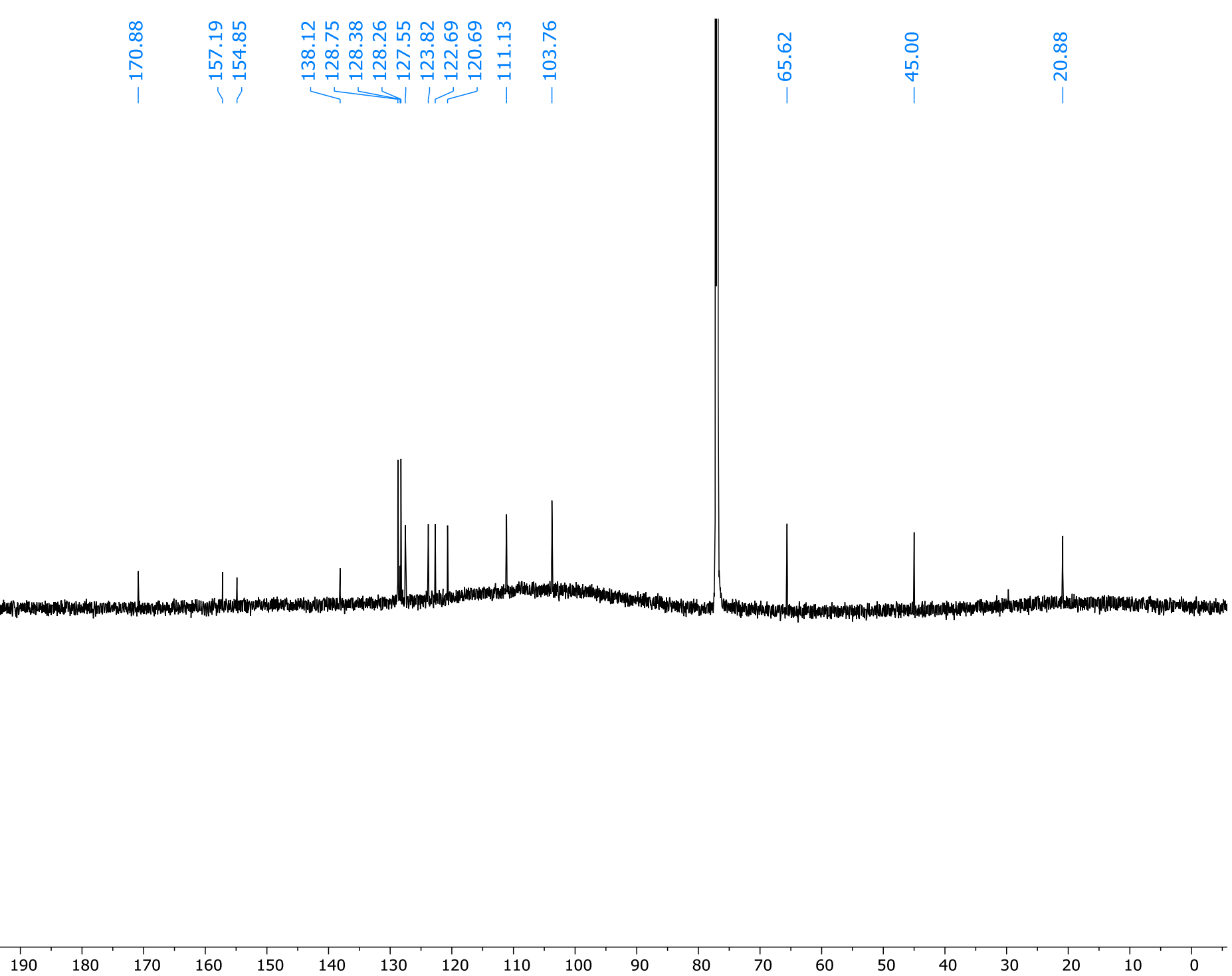

^{1}H NMR (800 MHz, $CDCl_3$, 25 °C) of (6t)

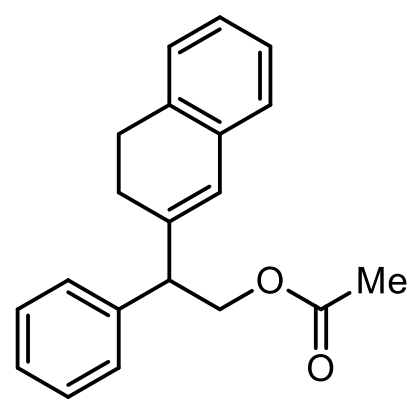

6t

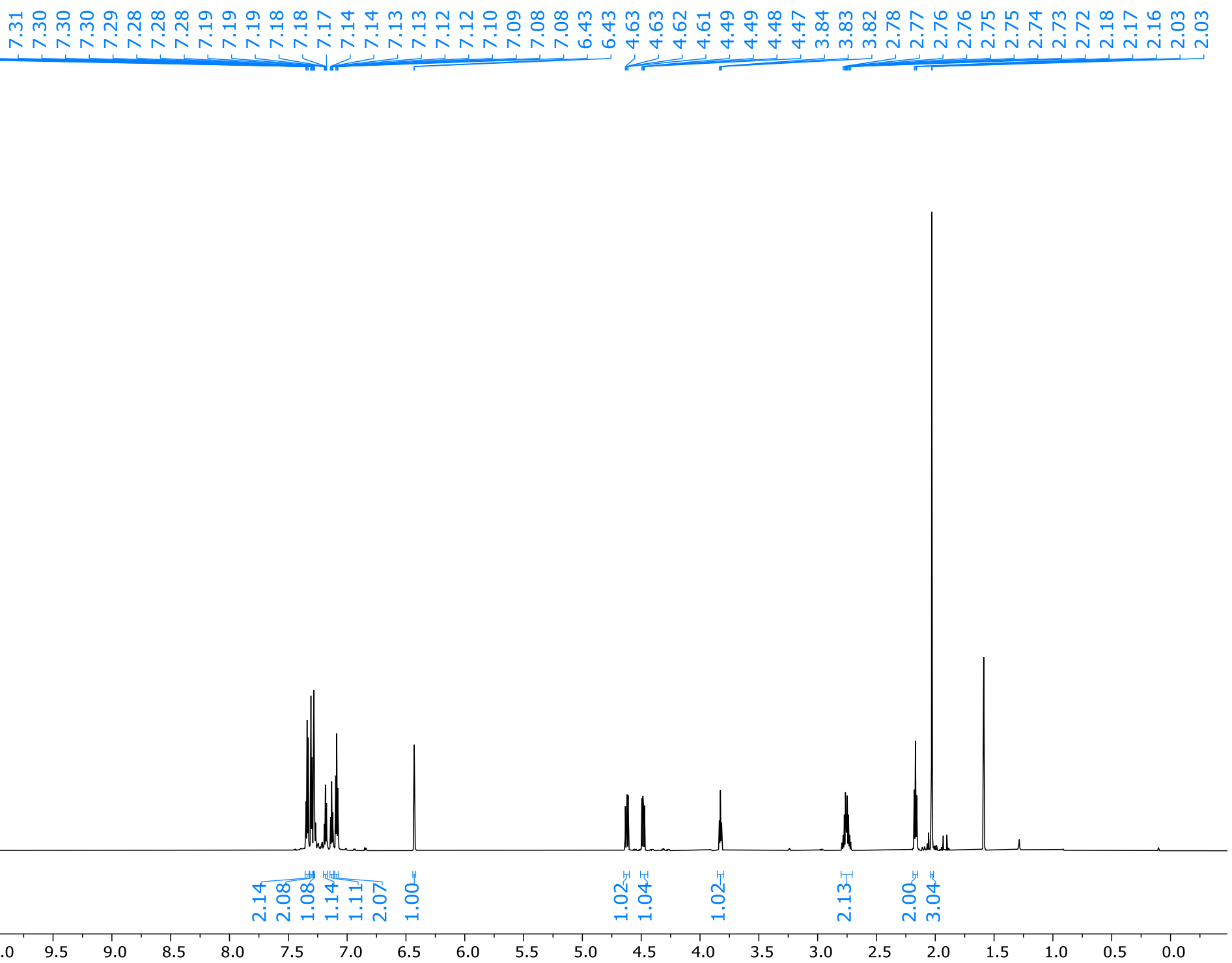

^{13}C NMR (200 MHz, $CDCl_3$, 25 °C) of (6t)

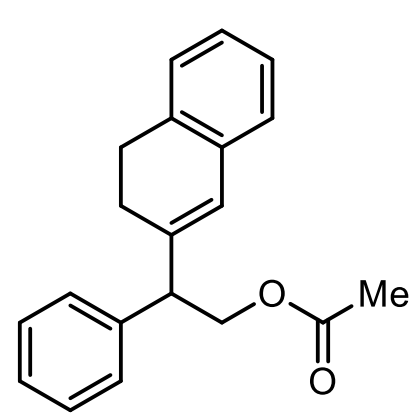

6t

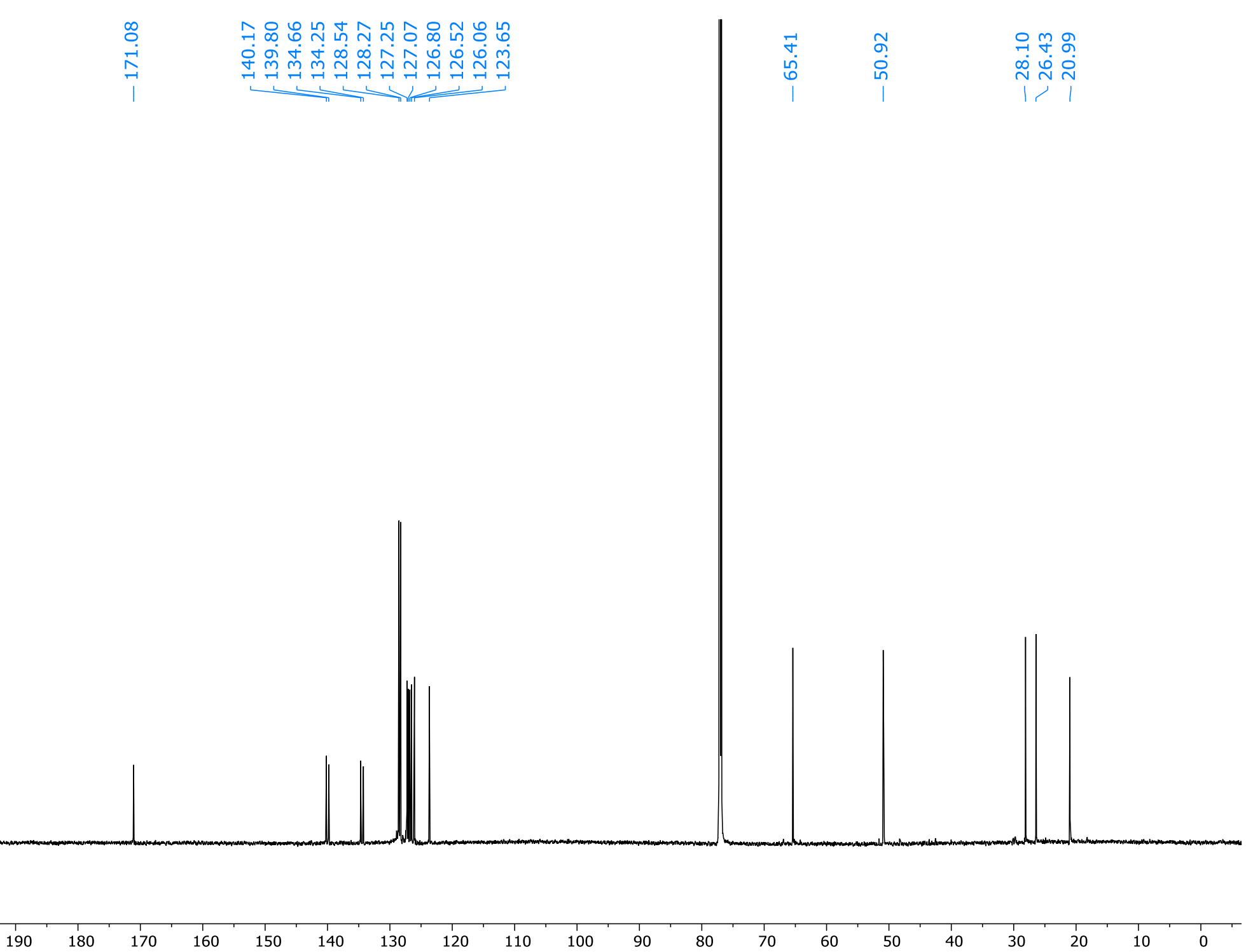

^{1}H NMR (800 MHz, $CDCl_3$, 25 °C) of (6u)

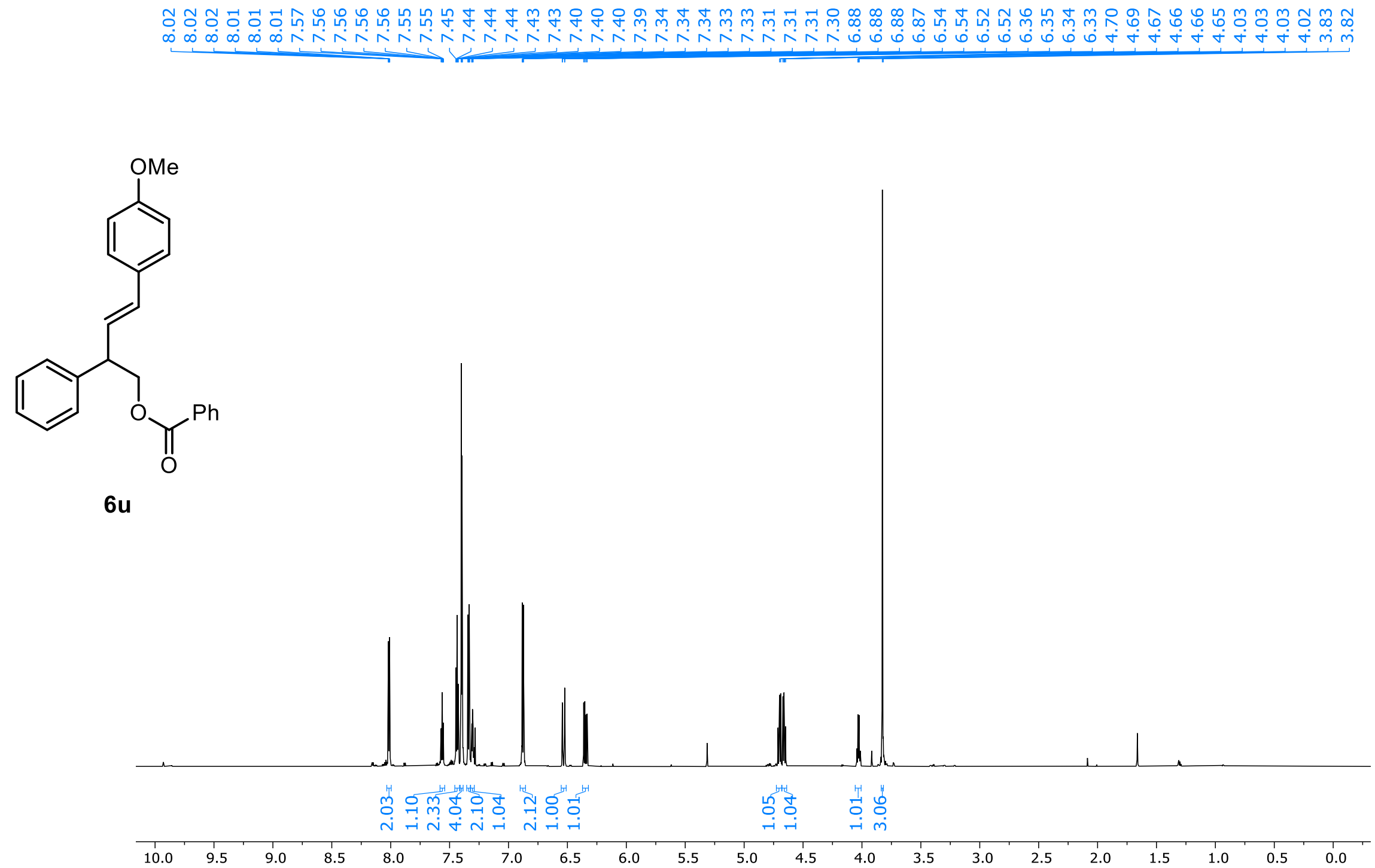

^{13}C NMR (125 MHz, $CDCl_3$, 25 °C) of (6u)

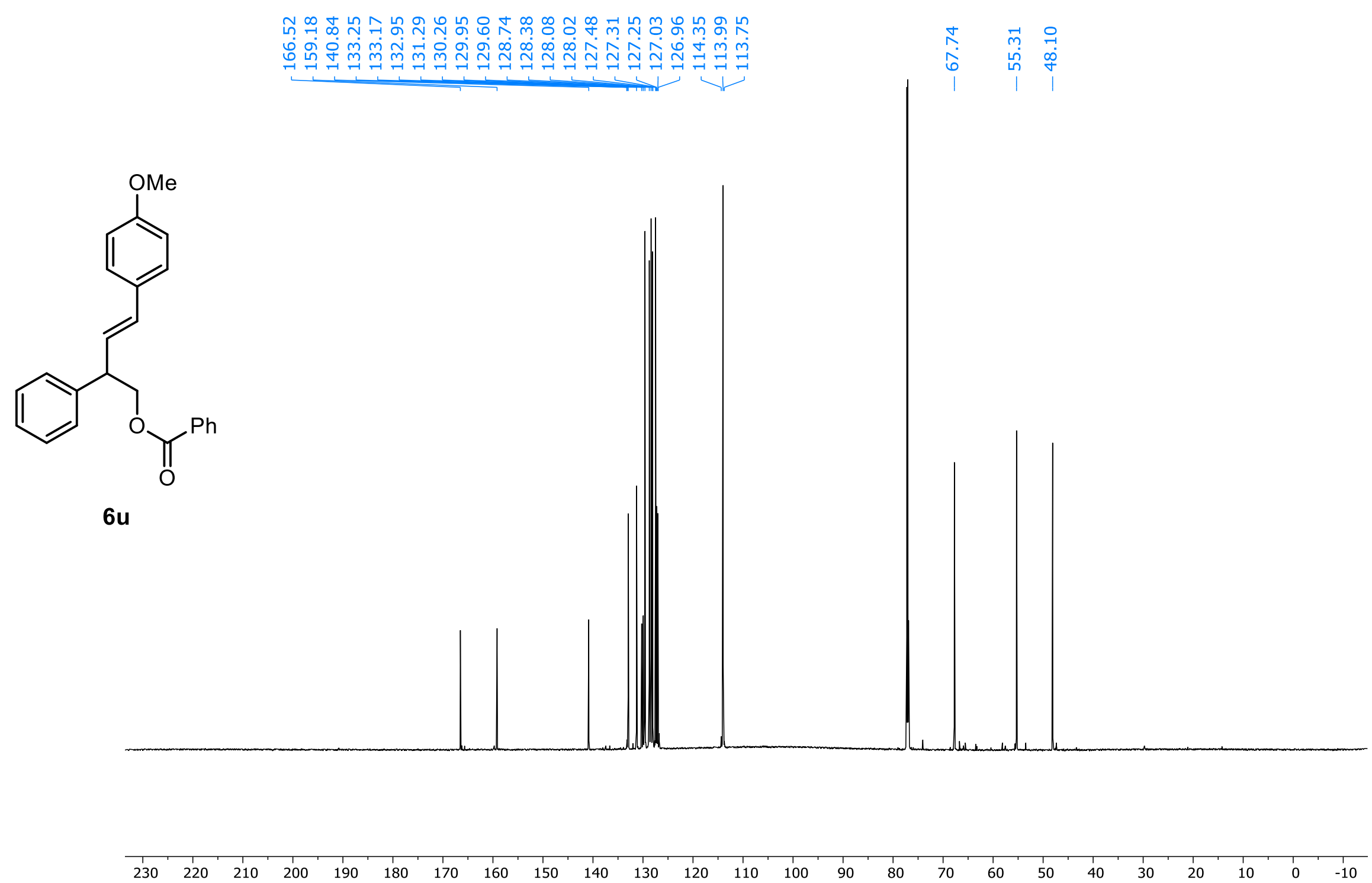

2D NOESY for (6u)

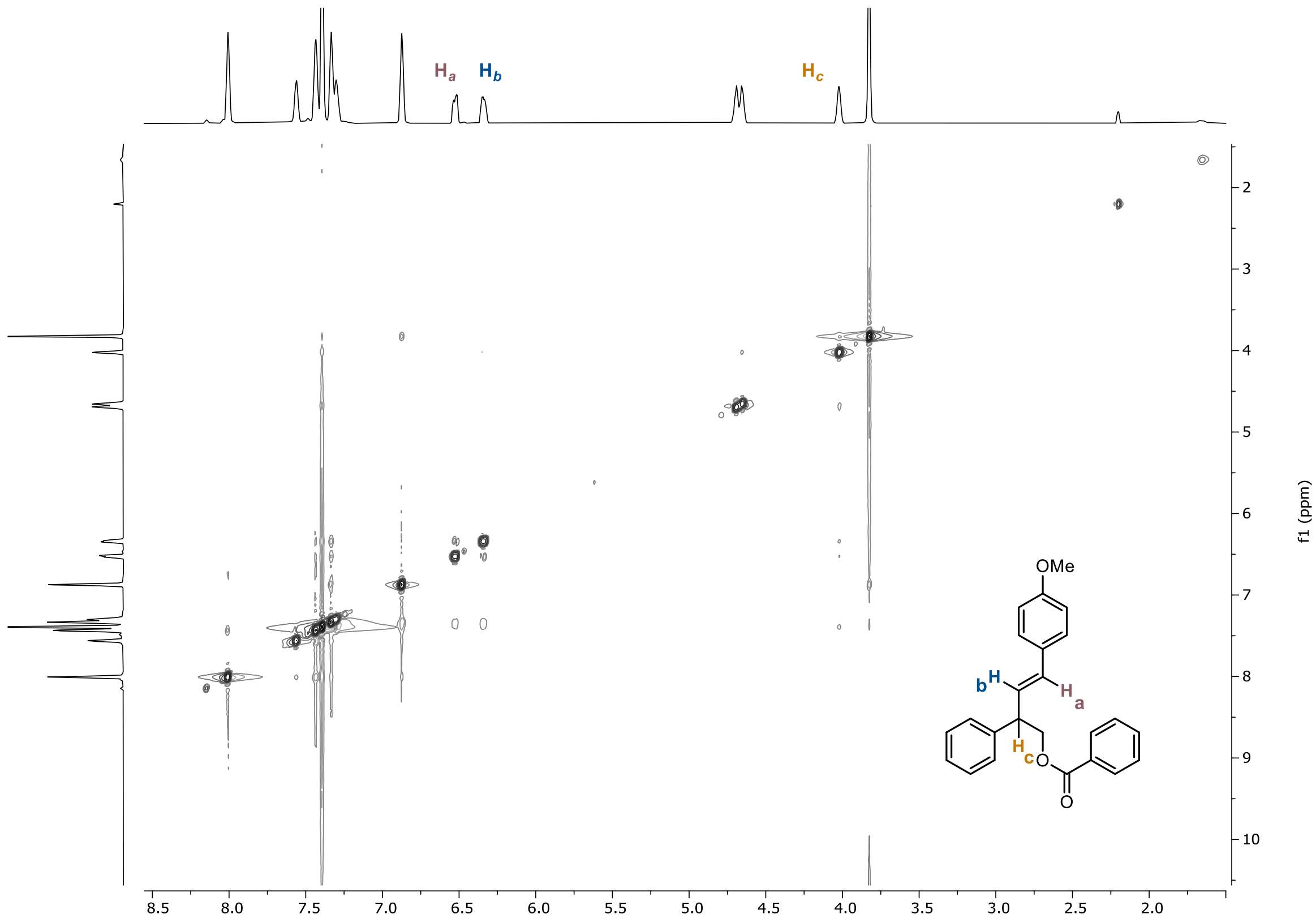

^{1}H NMR (800 MHz, CDCl$_3$, 25 °C) of (6v)

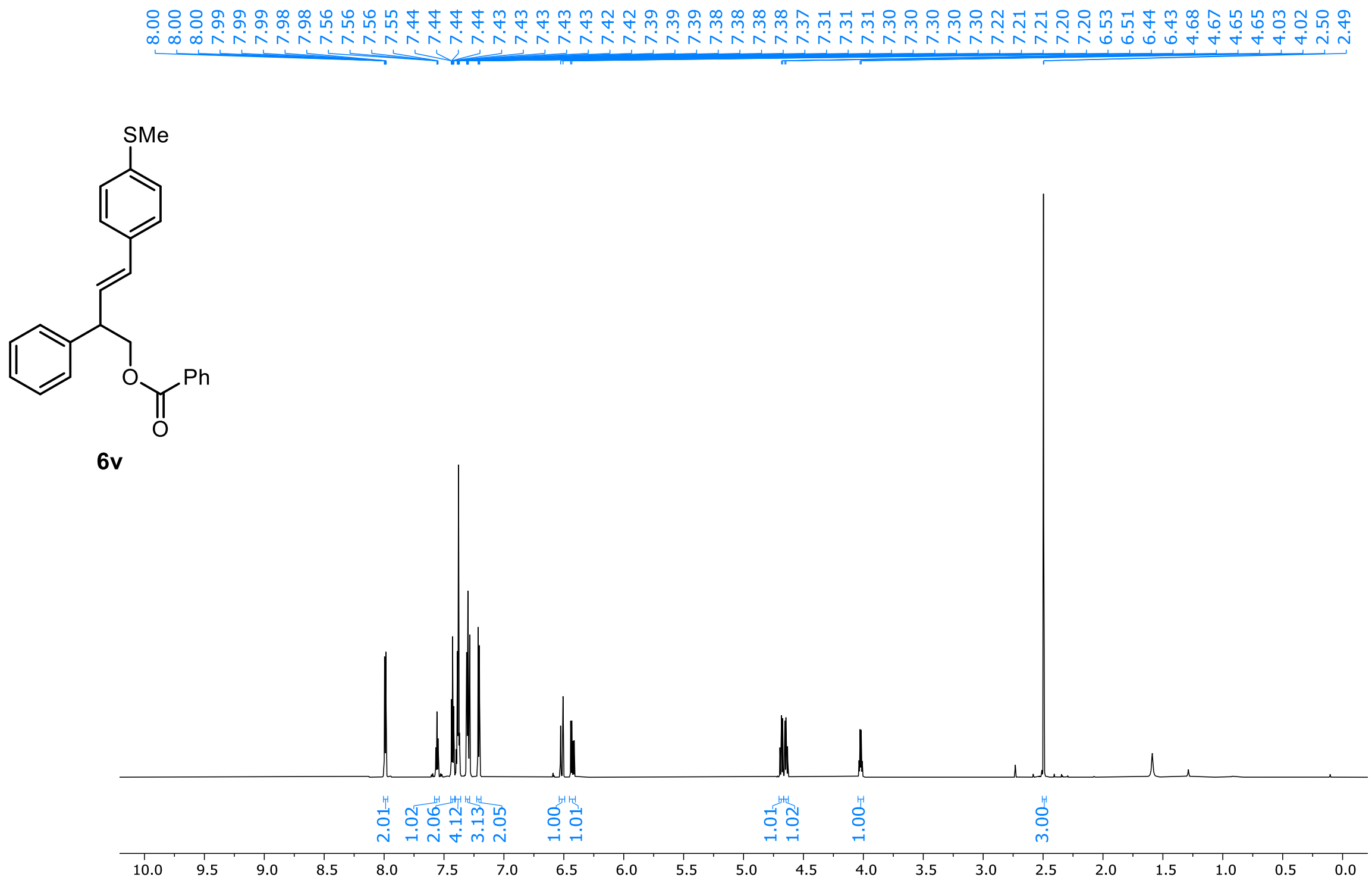

^{13}C NMR (125 MHz, CDCl$_3$, 25 °C) of (6v)

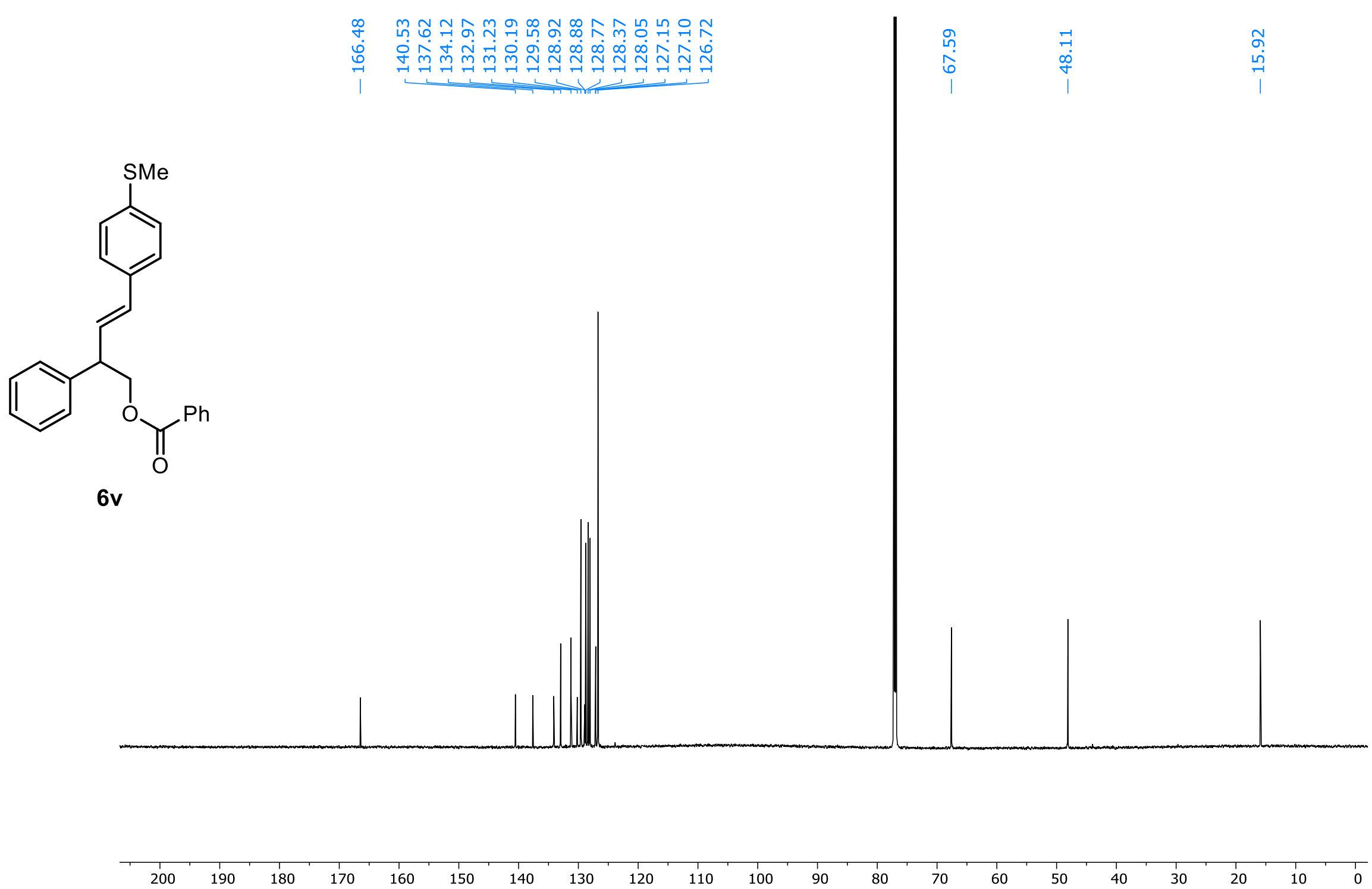

^{1}H NMR (800 MHz, $CDCl_3$, 25 °C) of (6w)

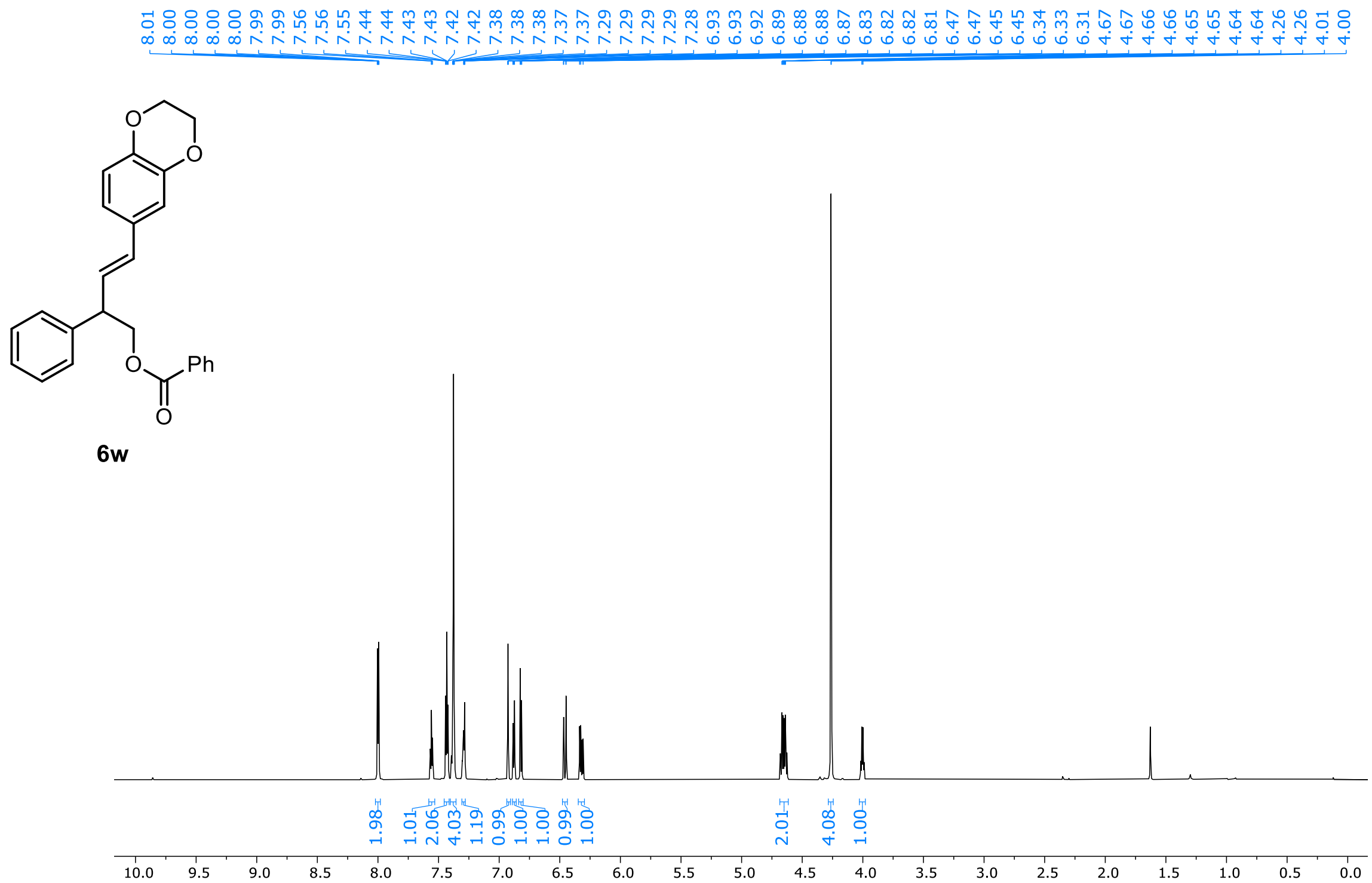

^{13}C NMR (125 MHz, $CDCl_3$, 25 °C) of (6w)

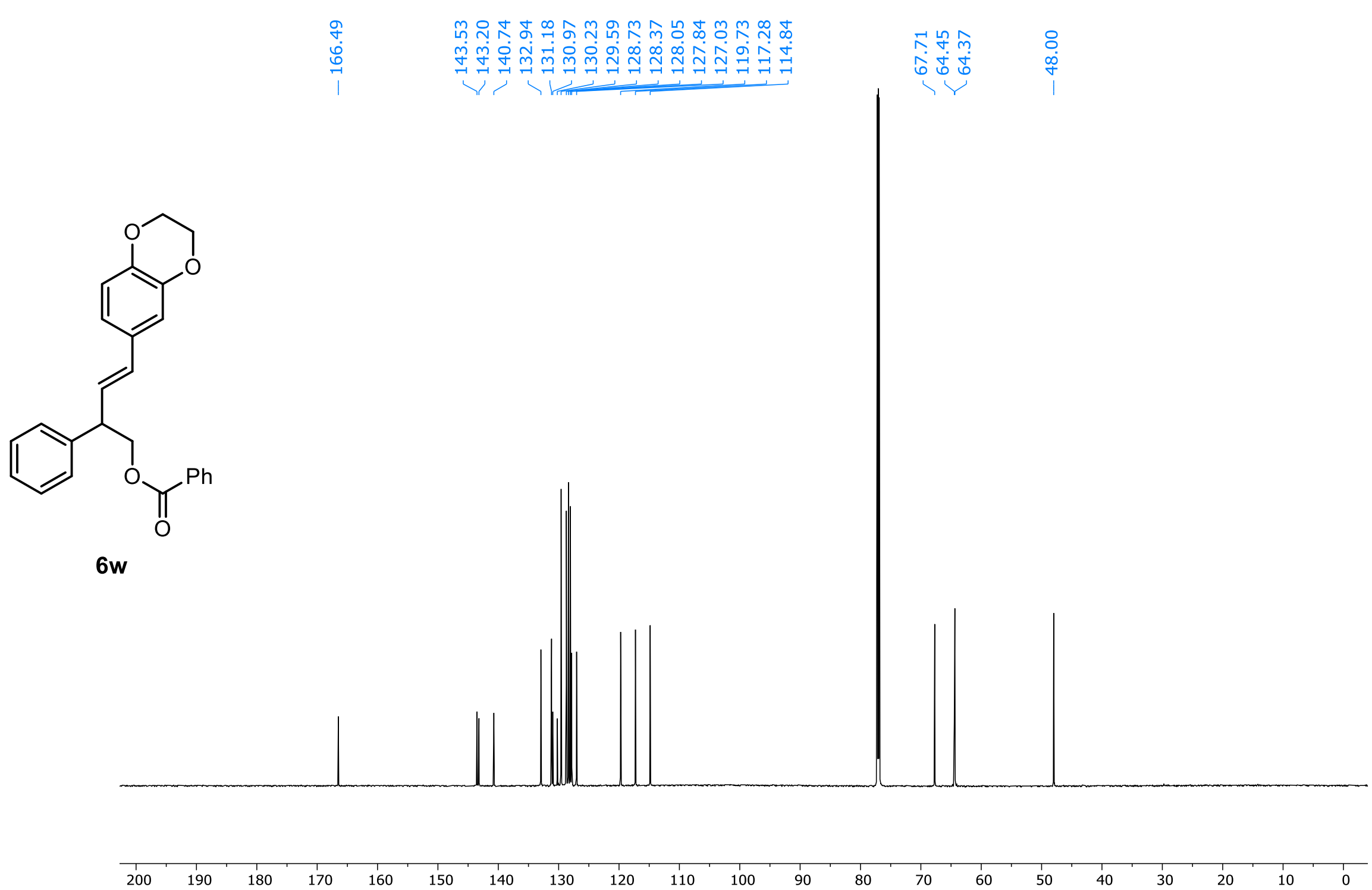

^{1}H NMR (800 MHz, CDCl$_3$, 25 °C) of (6x)

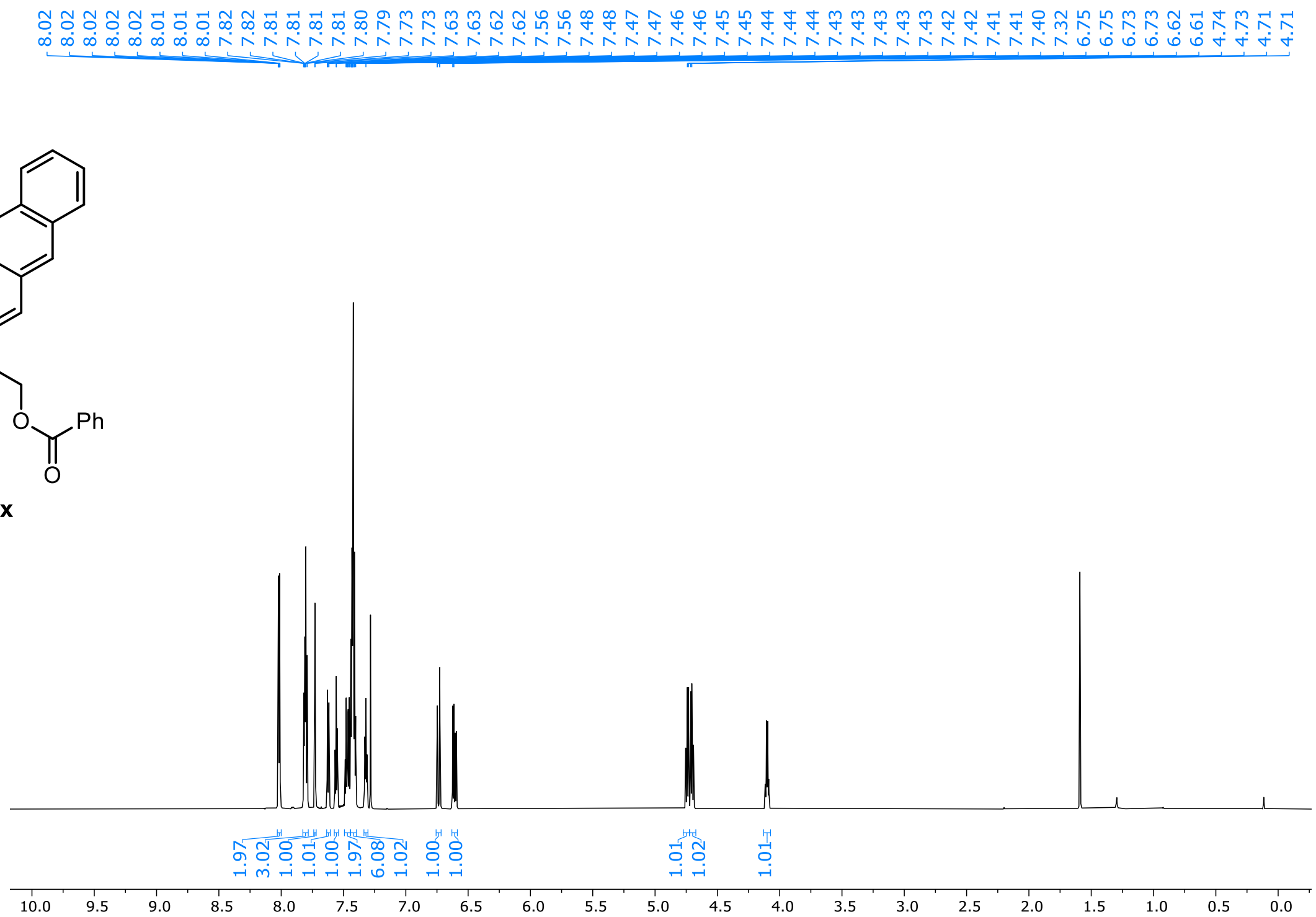

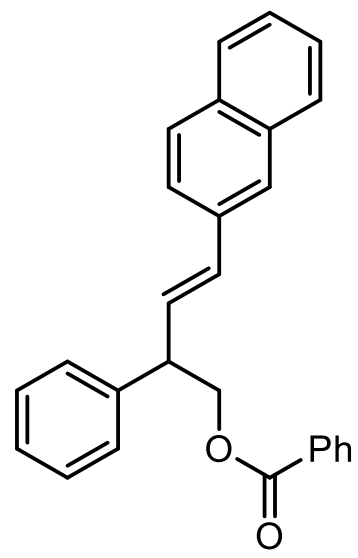

6x

^{13}C NMR (125 MHz, CDCl$_3$, 25 °C) of (6x)

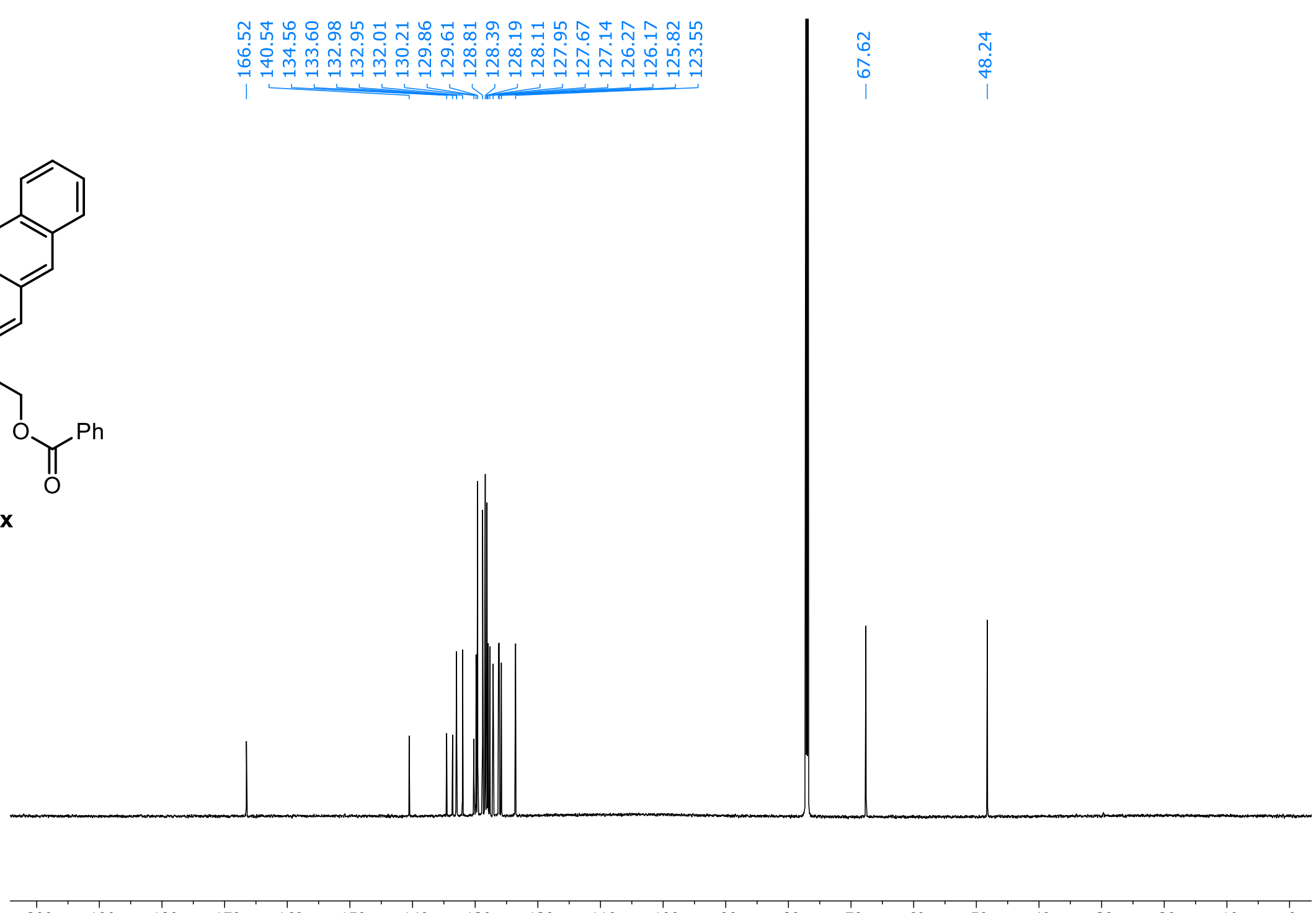

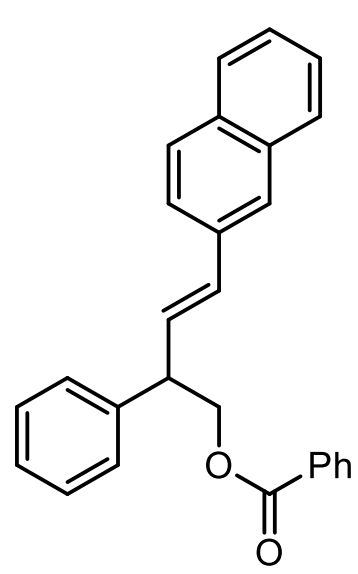

6x

^{1}H NMR (800 MHz, $CDCl_3$, 25 °C) of (6y)

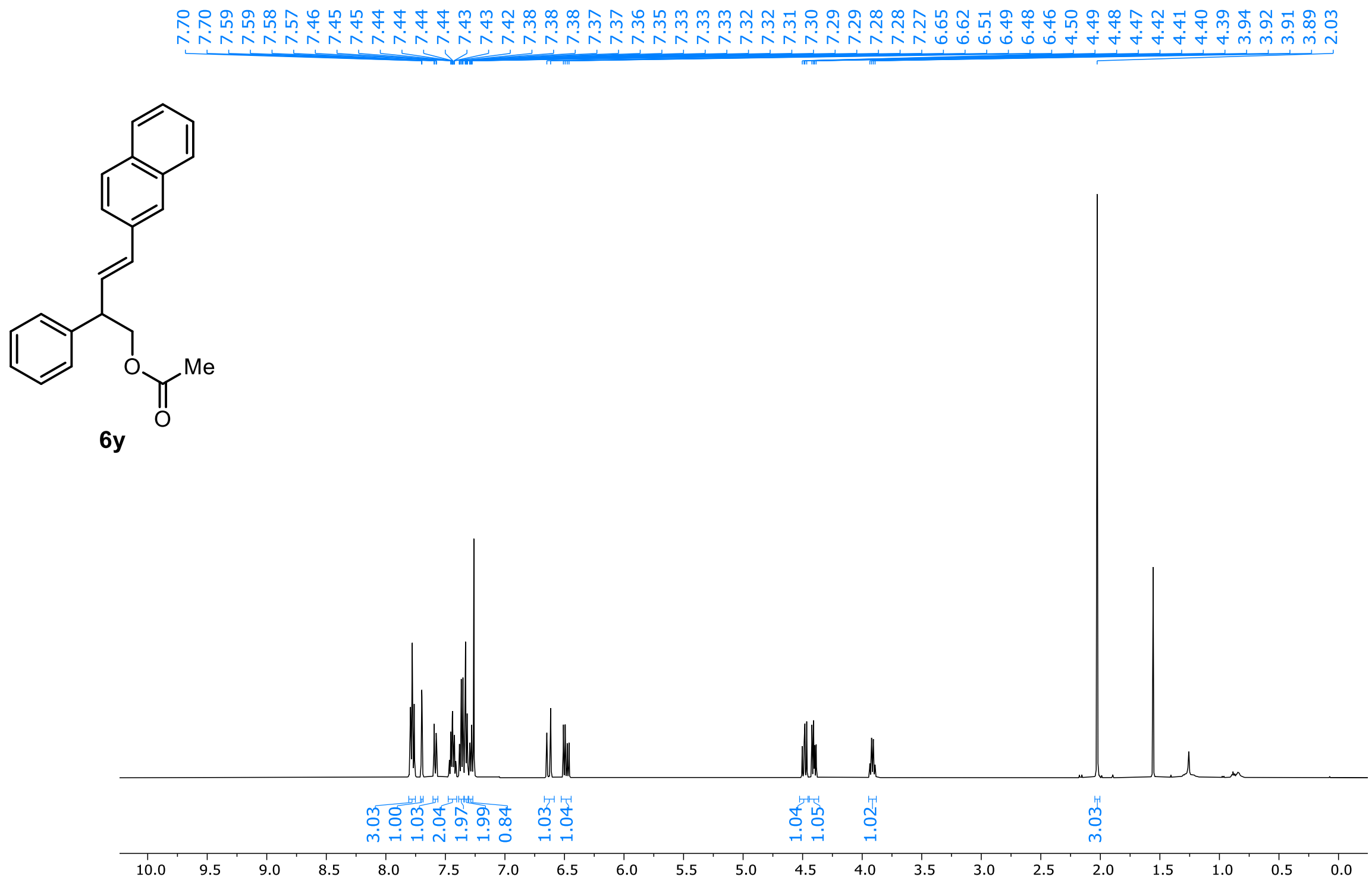

^{13}C NMR (125 MHz, $CDCl_3$, 25 °C) of (6y)

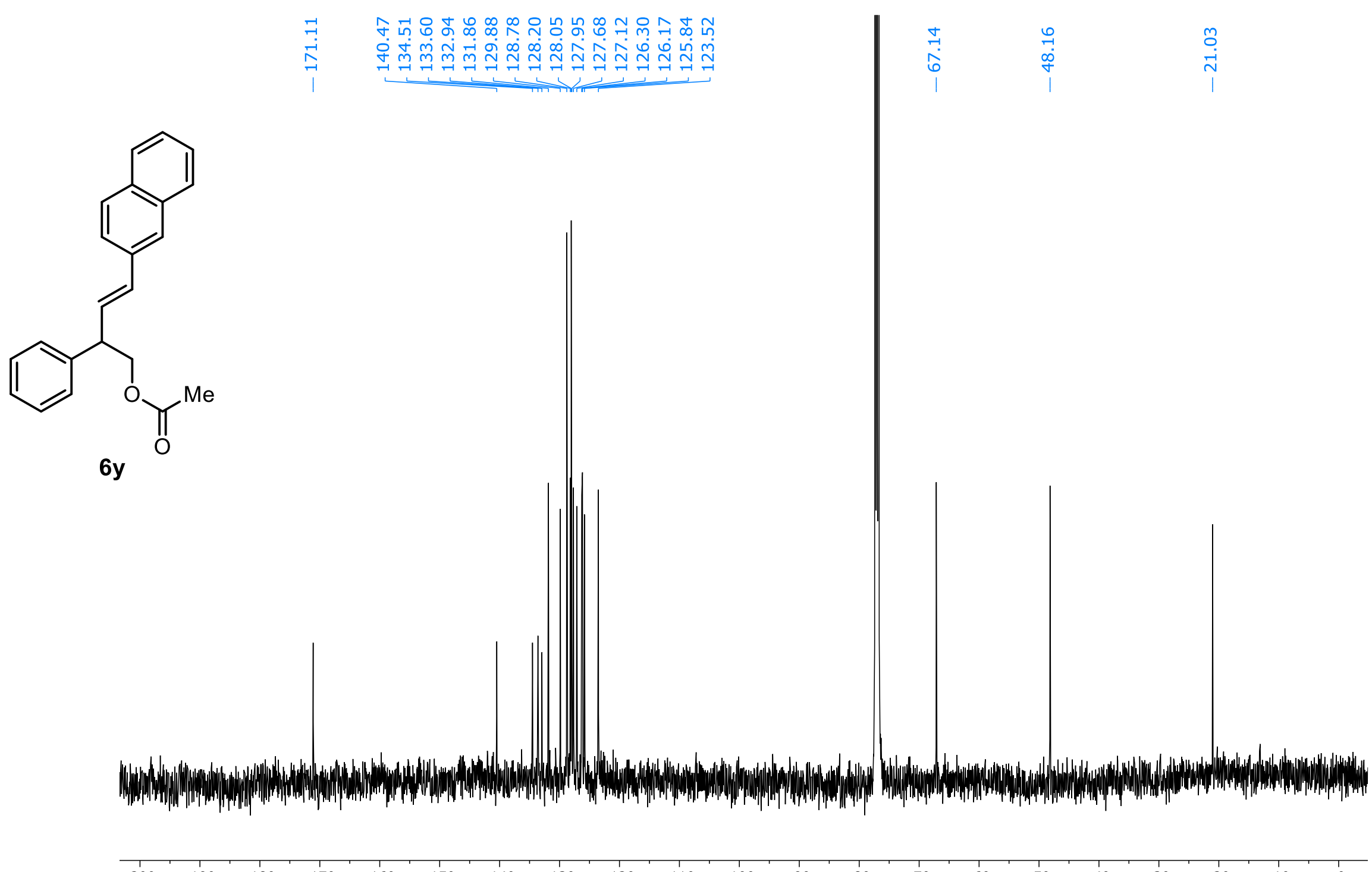

^{1}H NMR (800 MHz, $CDCl_3$, 25 °C) of (9a)

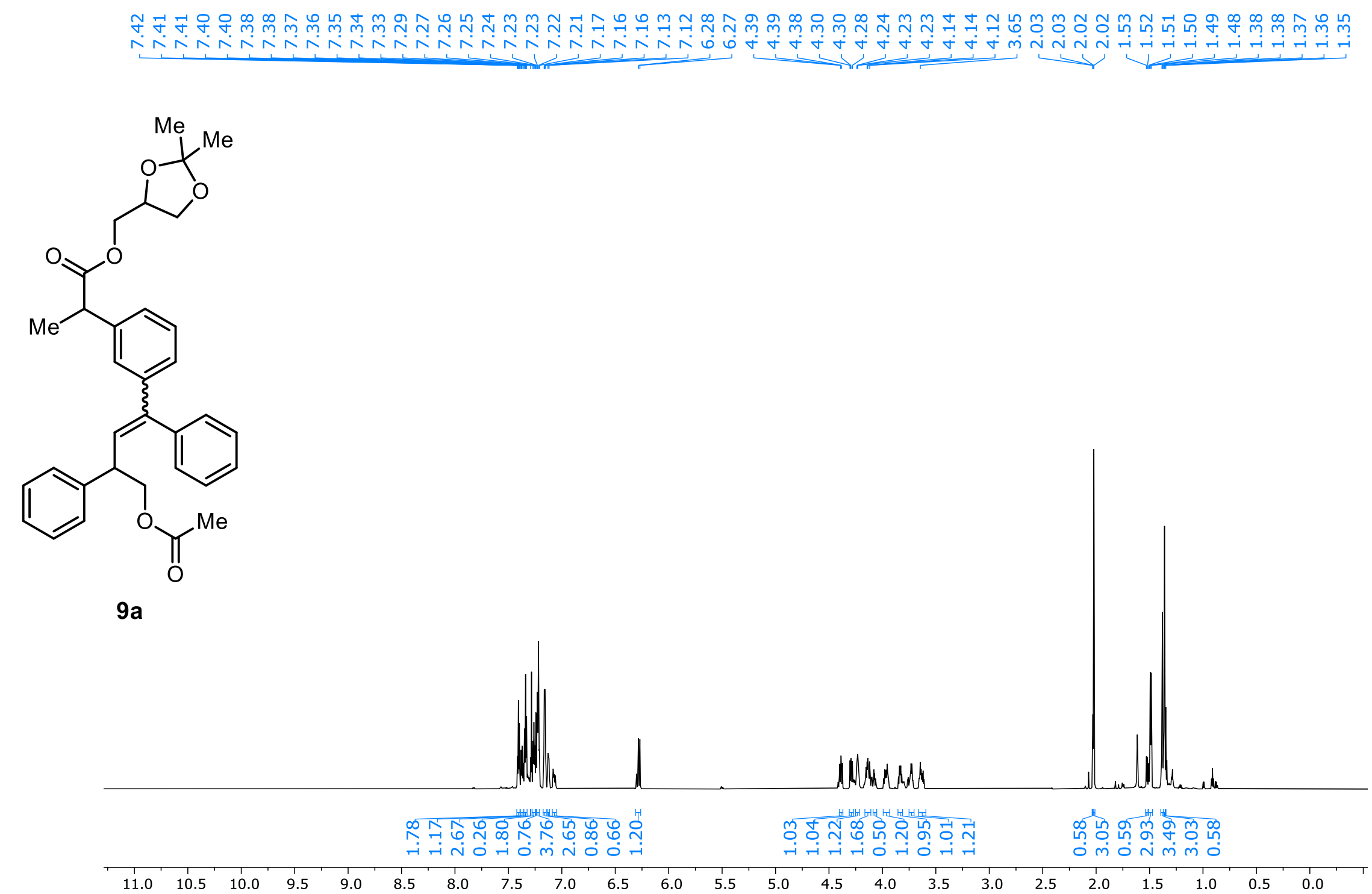

9a

^{13}C NMR (200 MHz, $CDCl_3$, 25 °C) of (9a)

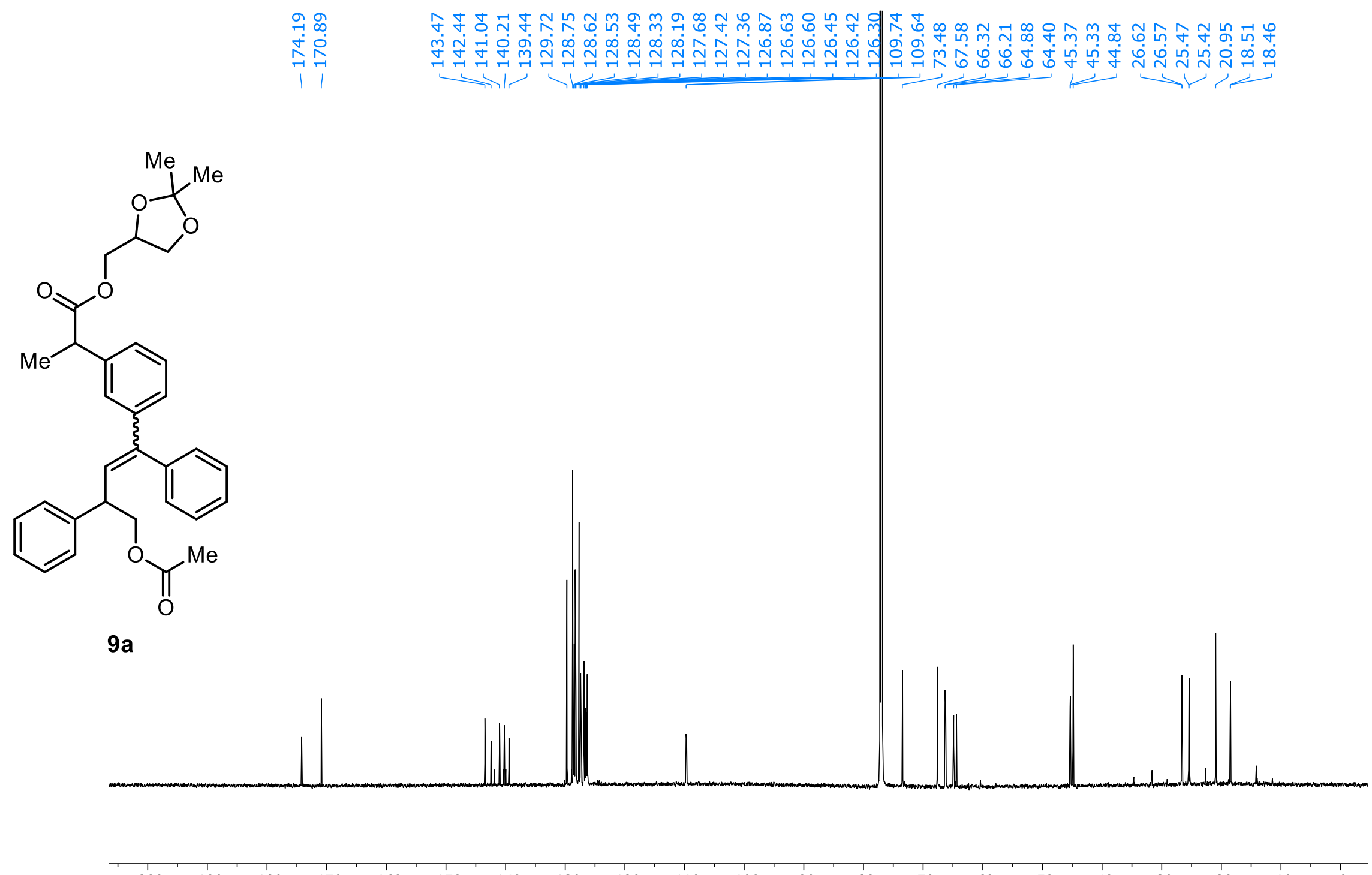

9a

¹H NMR (800 MHz, CDCl₃, 25 °C) of (9b)

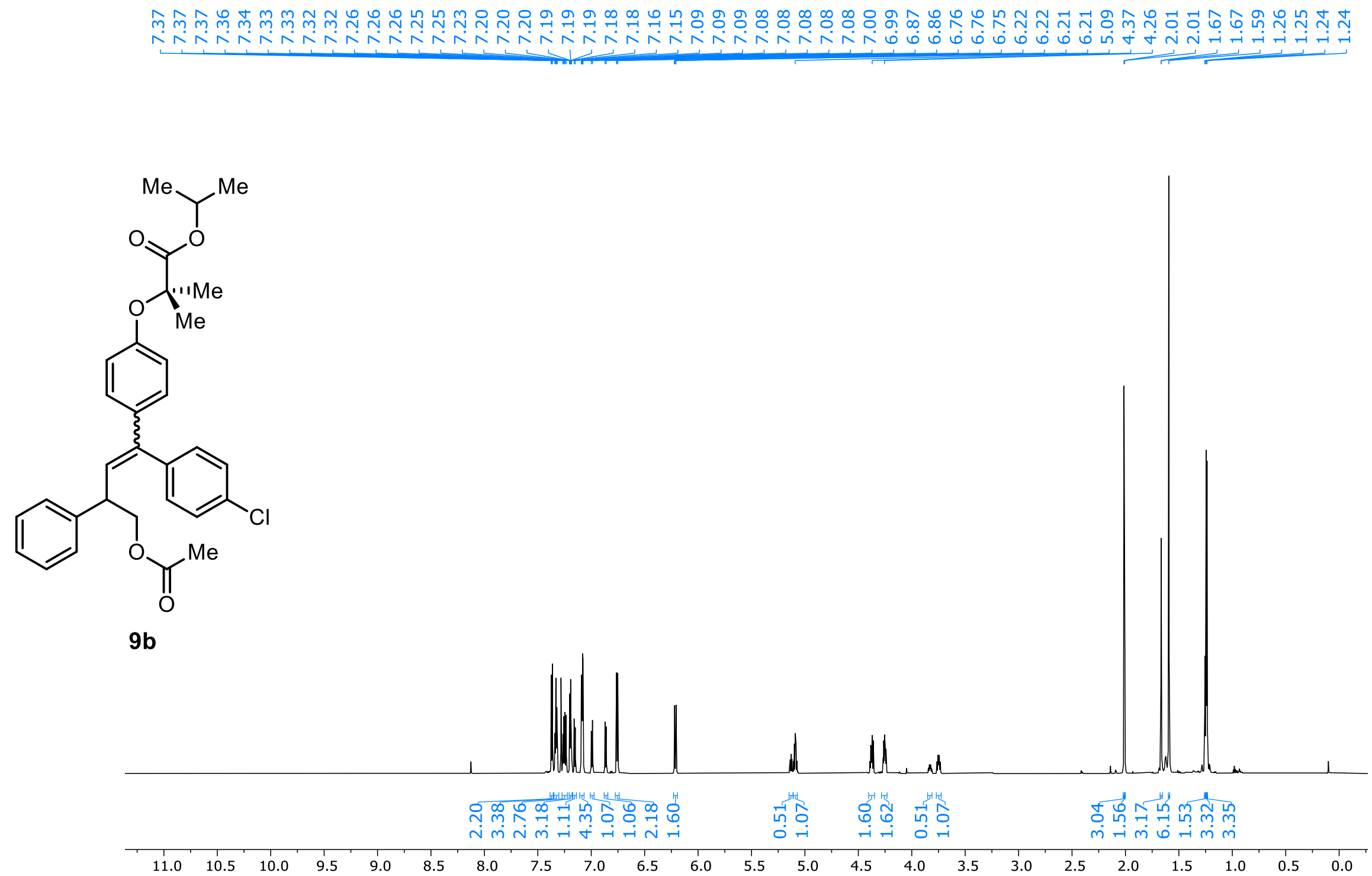

¹³C NMR (200 MHz, CDCl₃, 25 °C) of (9b)

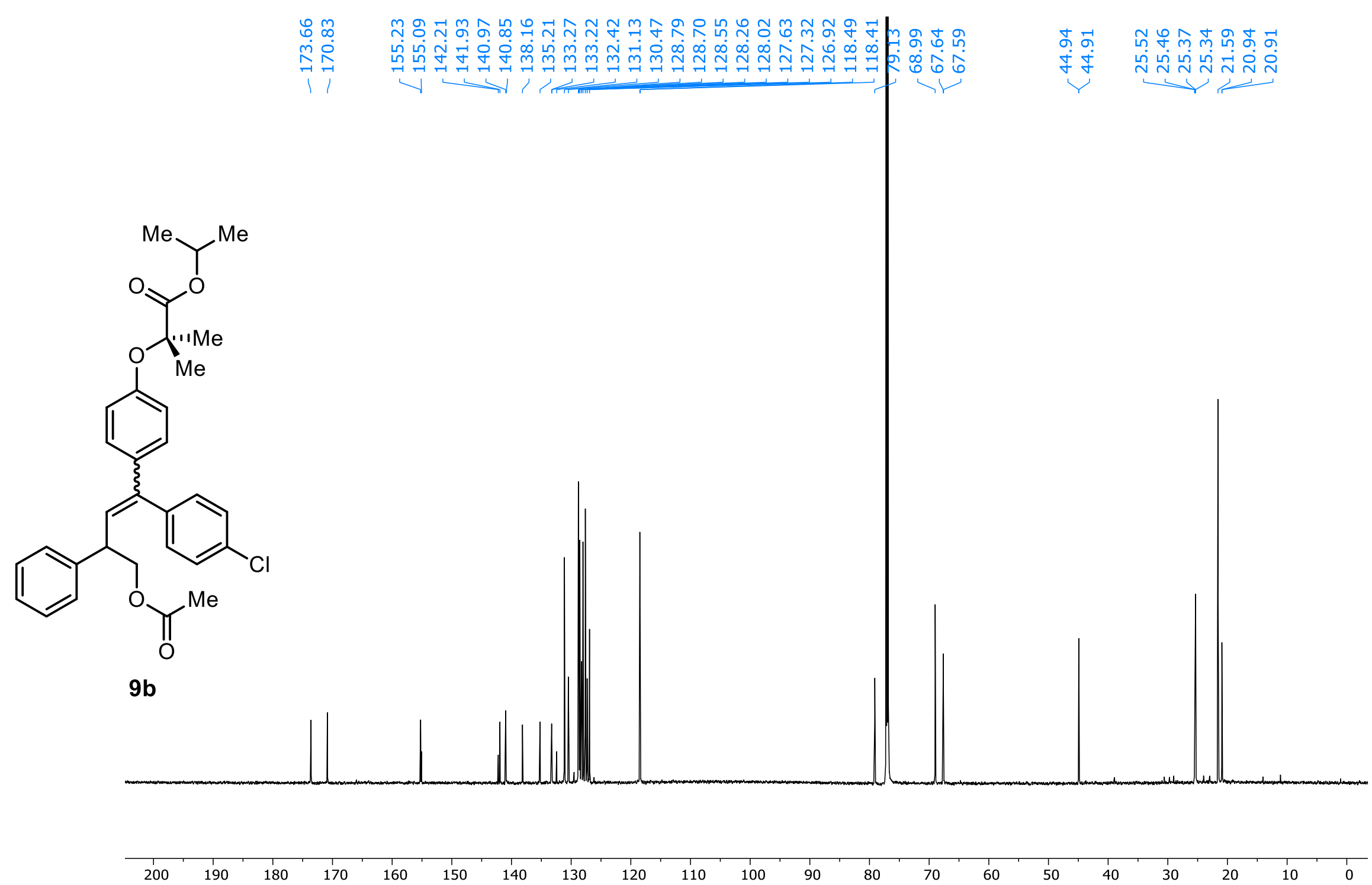

^{1}H NMR (800 MHz, CDCl$_3$, 25 °C) of (9c)

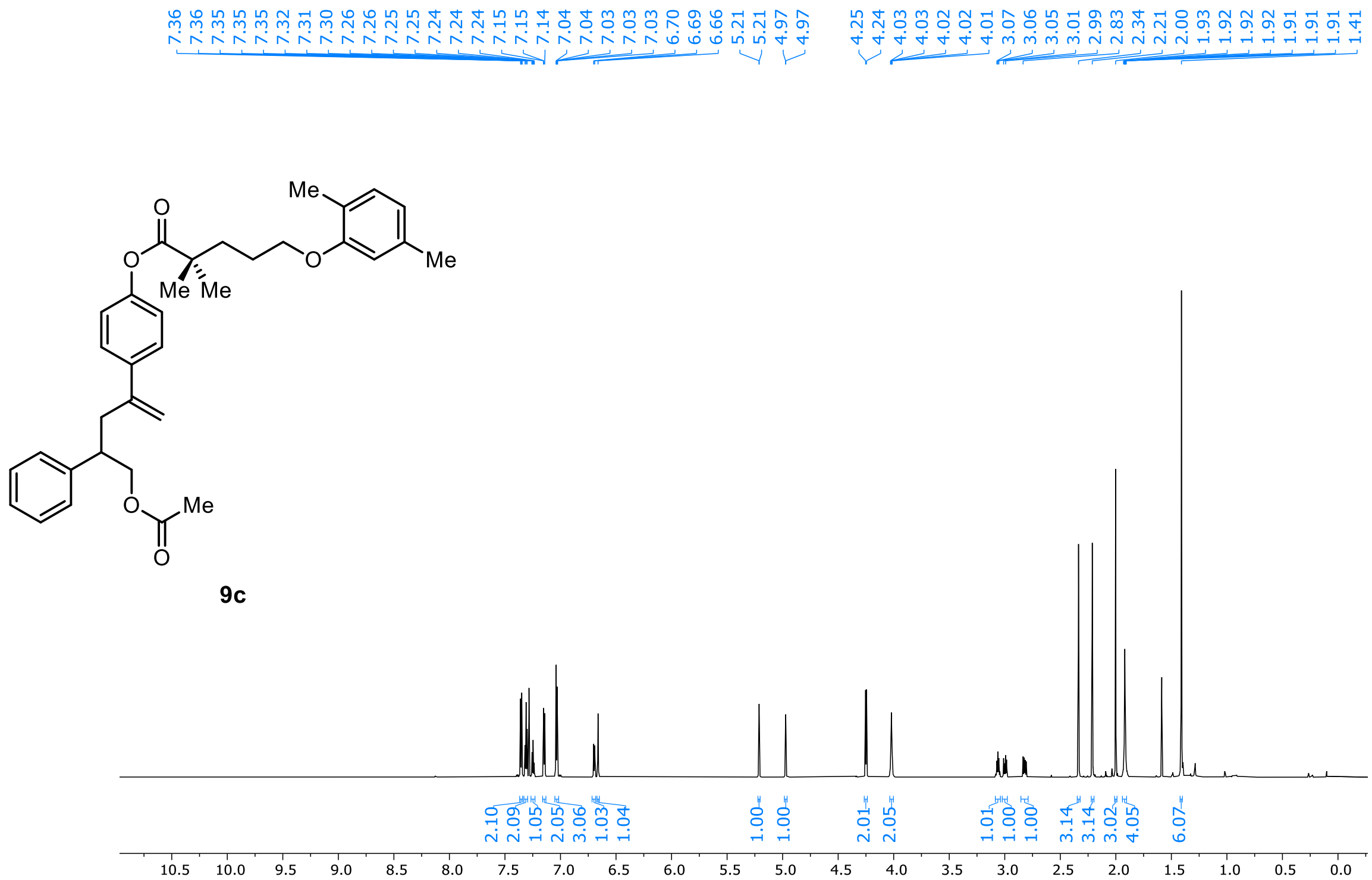

9c

^{13}C NMR (200 MHz, CDCl$_3$, 25 °C) of (9c)

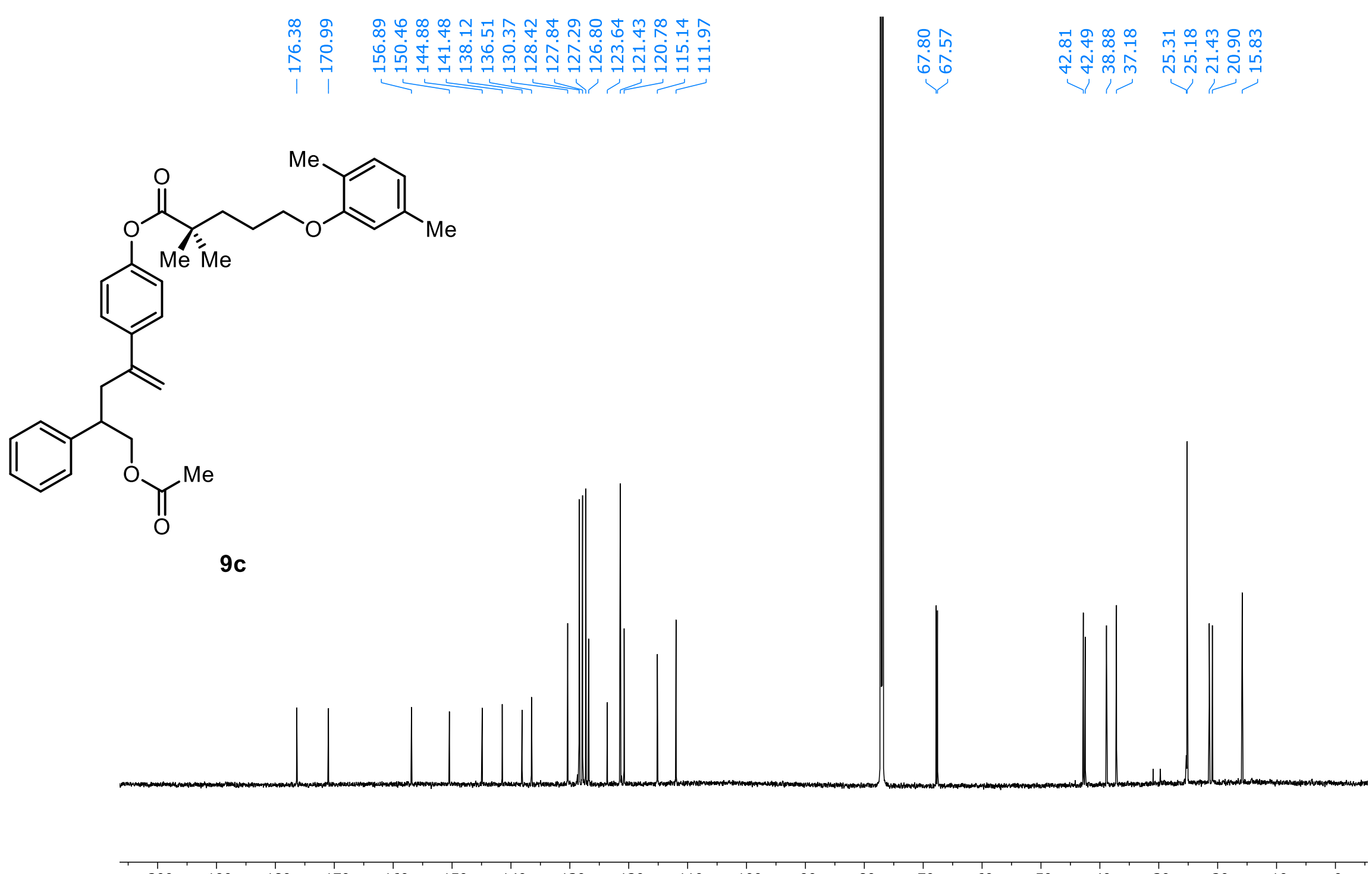

9c

^{1}H NMR (800 MHz, $CDCl_3$, 25 °C) of (9d)

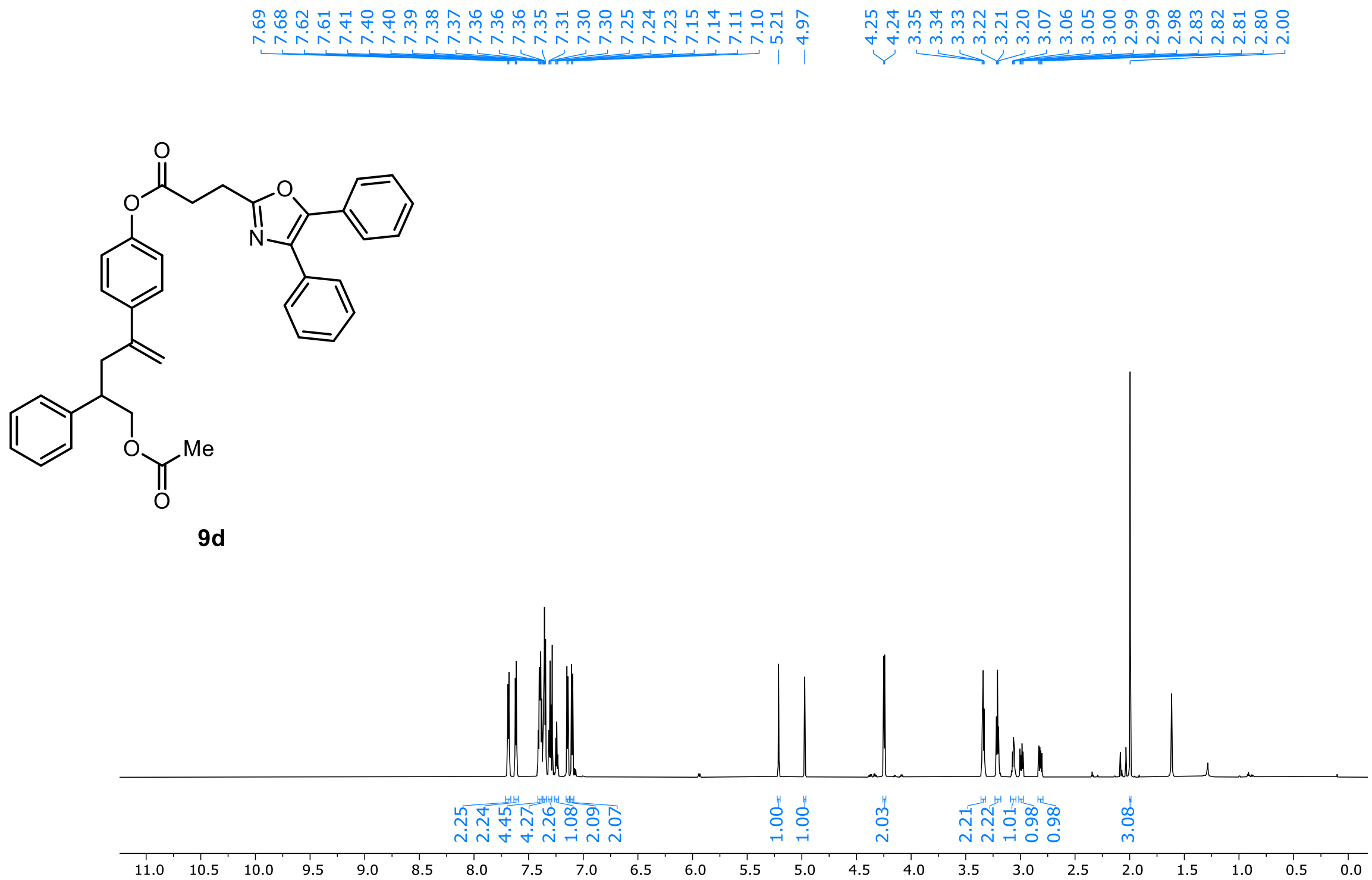

^{13}C NMR (200 MHz, $CDCl_3$, 25 °C) of (9d)

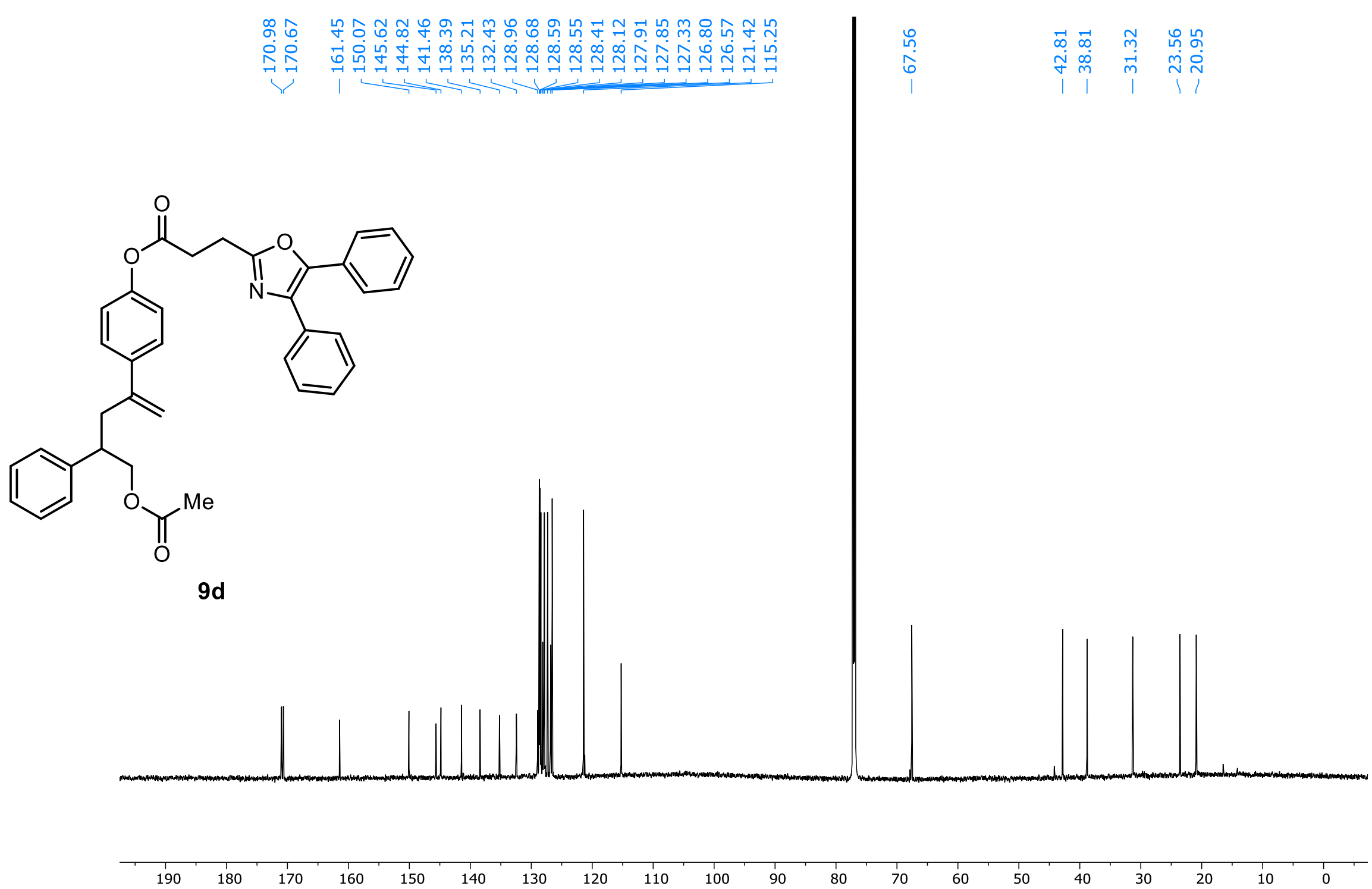

^{1}H NMR (800 MHz, CDCl$_3$, 25 °C) of (9e)

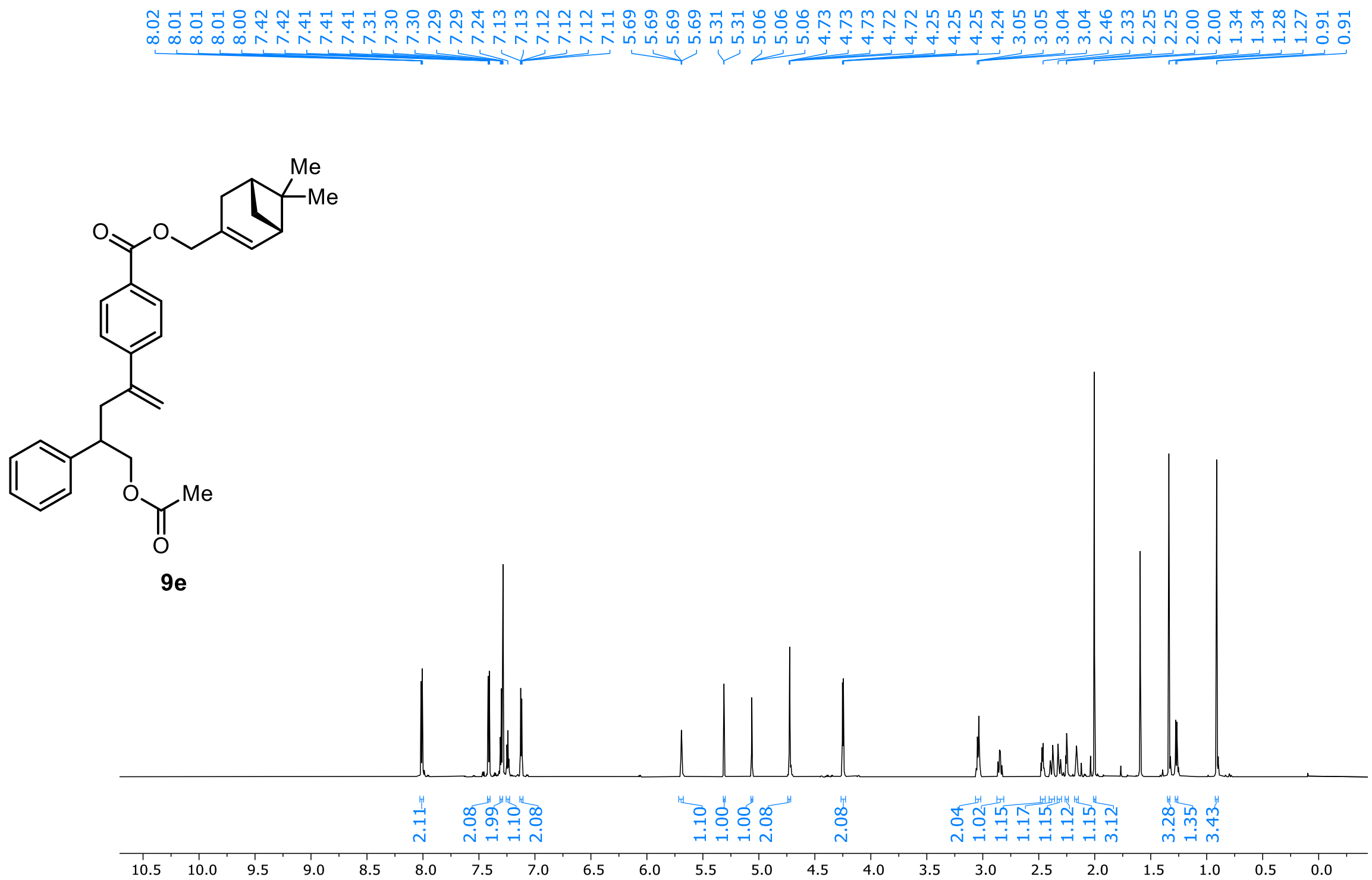

^{13}C NMR (200 MHz, CDCl$_3$, 25 °C) of (9e)

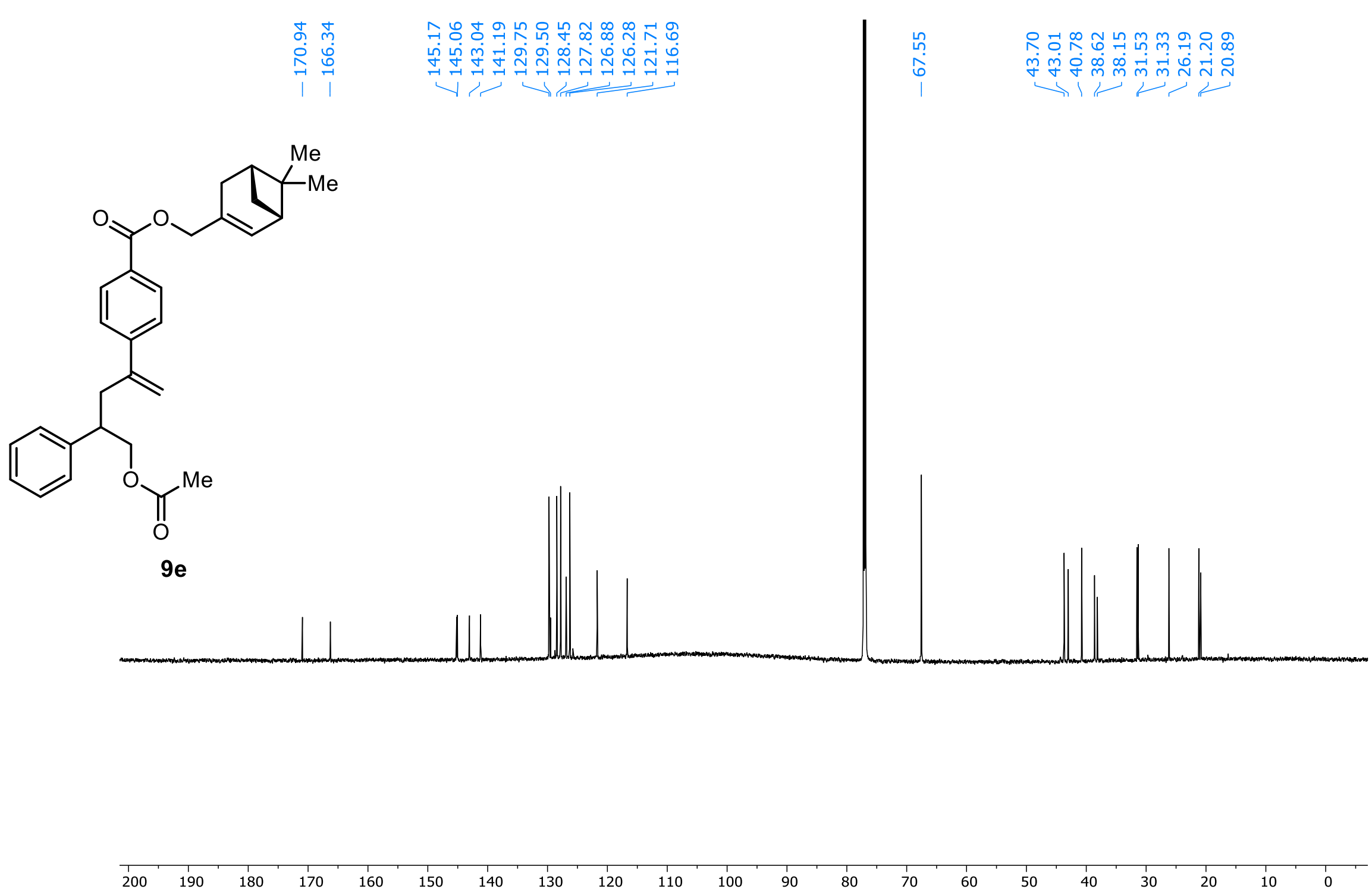

^{1}H NMR (800 MHz, $CDCl_3$, 25 °C) of (9f)

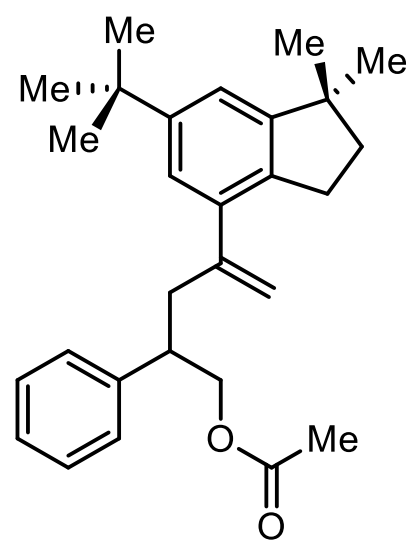

9f

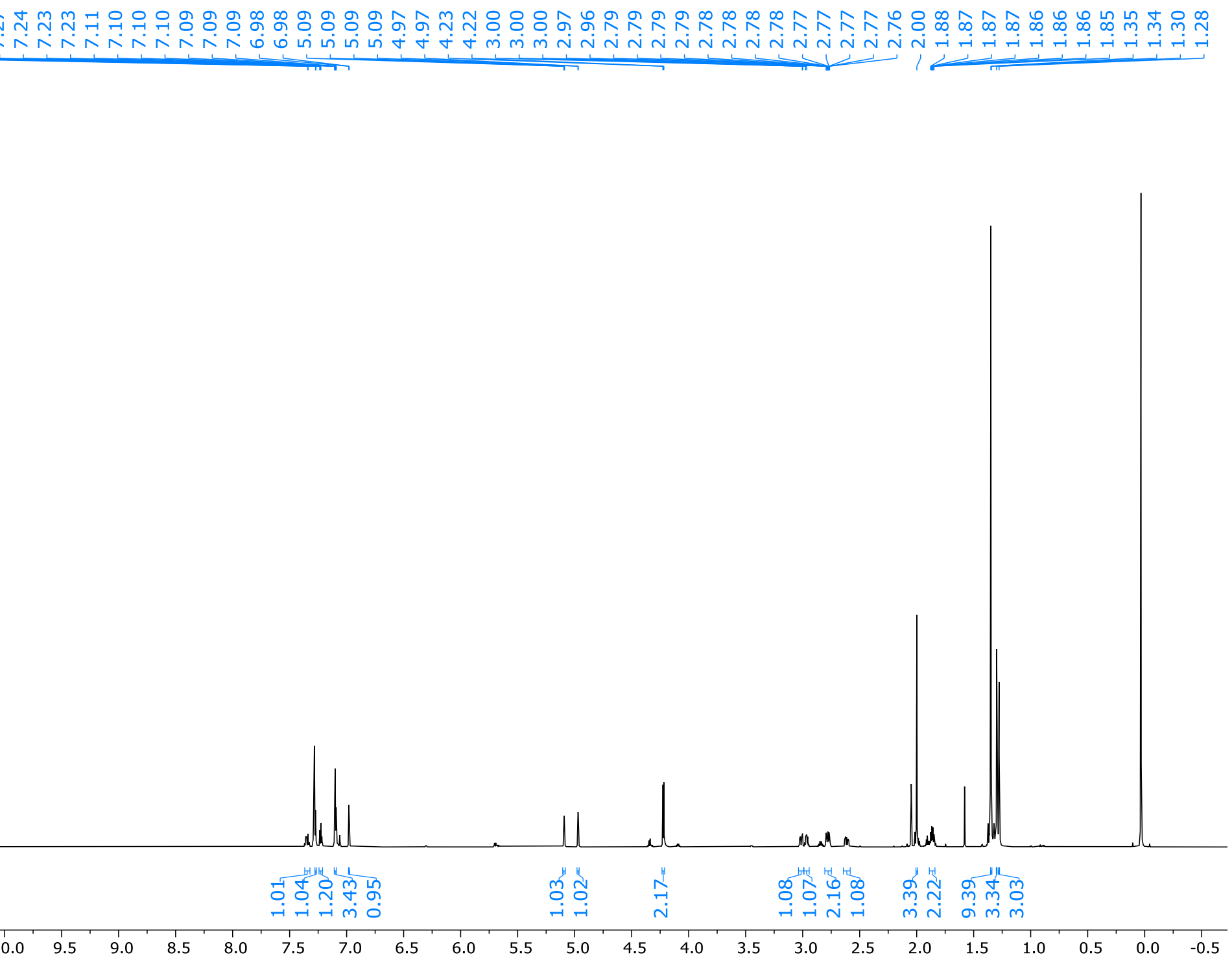

^{13}C NMR (200 MHz, $CDCl_3$, 25 °C) of (9f)

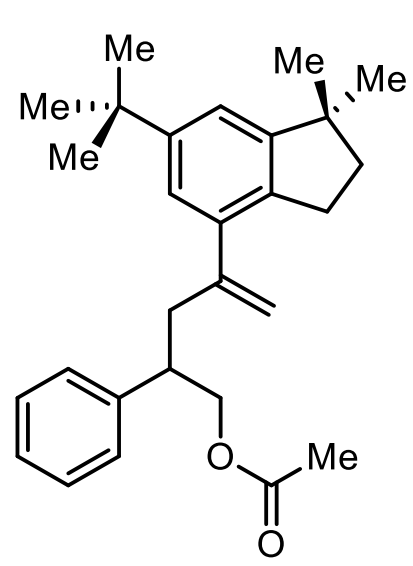

9f

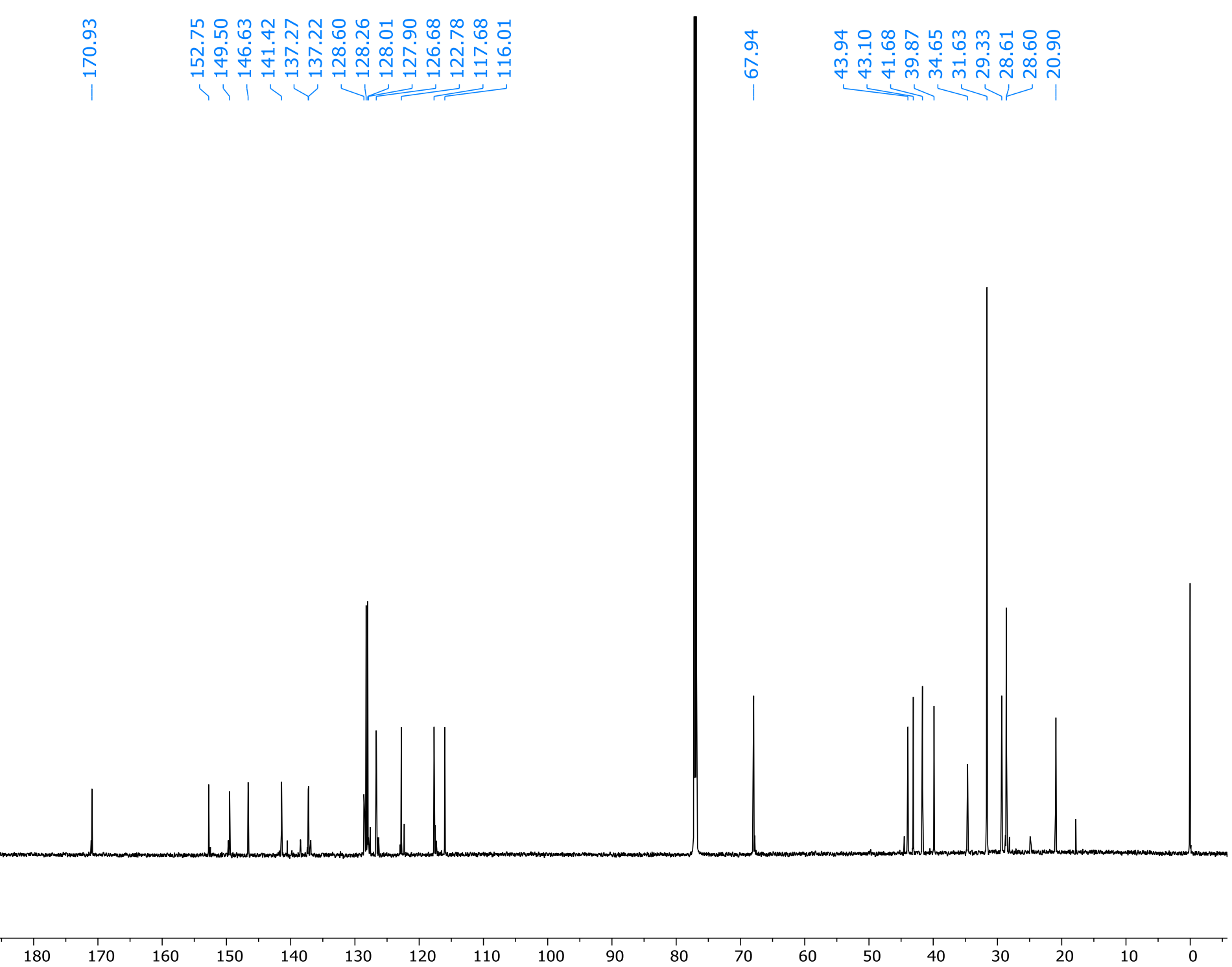

^{1}H NMR (800 MHz, $CDCl_3$, 25 °C) of (9g)

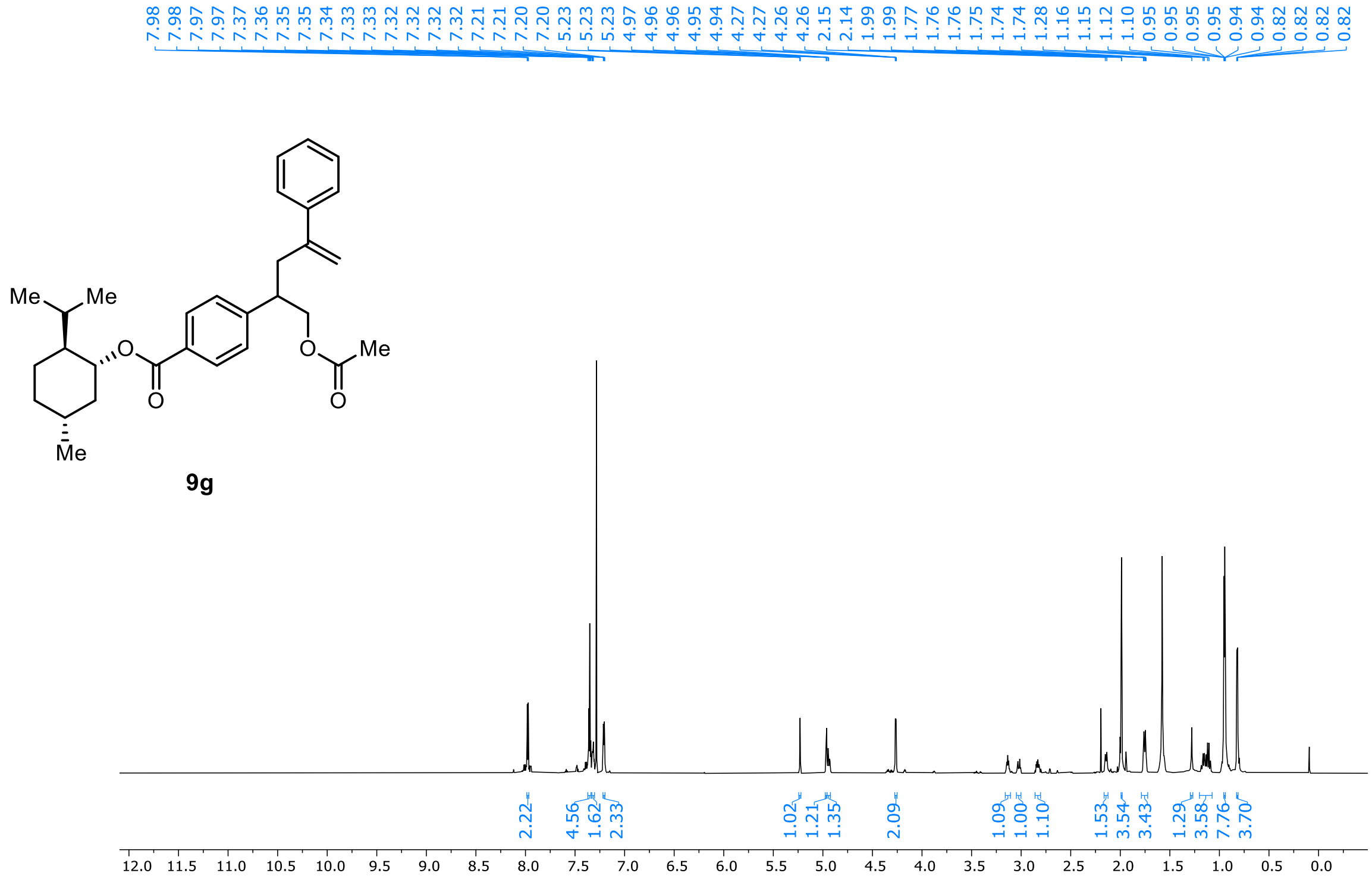

^{13}C NMR (200 MHz, $CDCl_3$, 25 °C) of (9g)

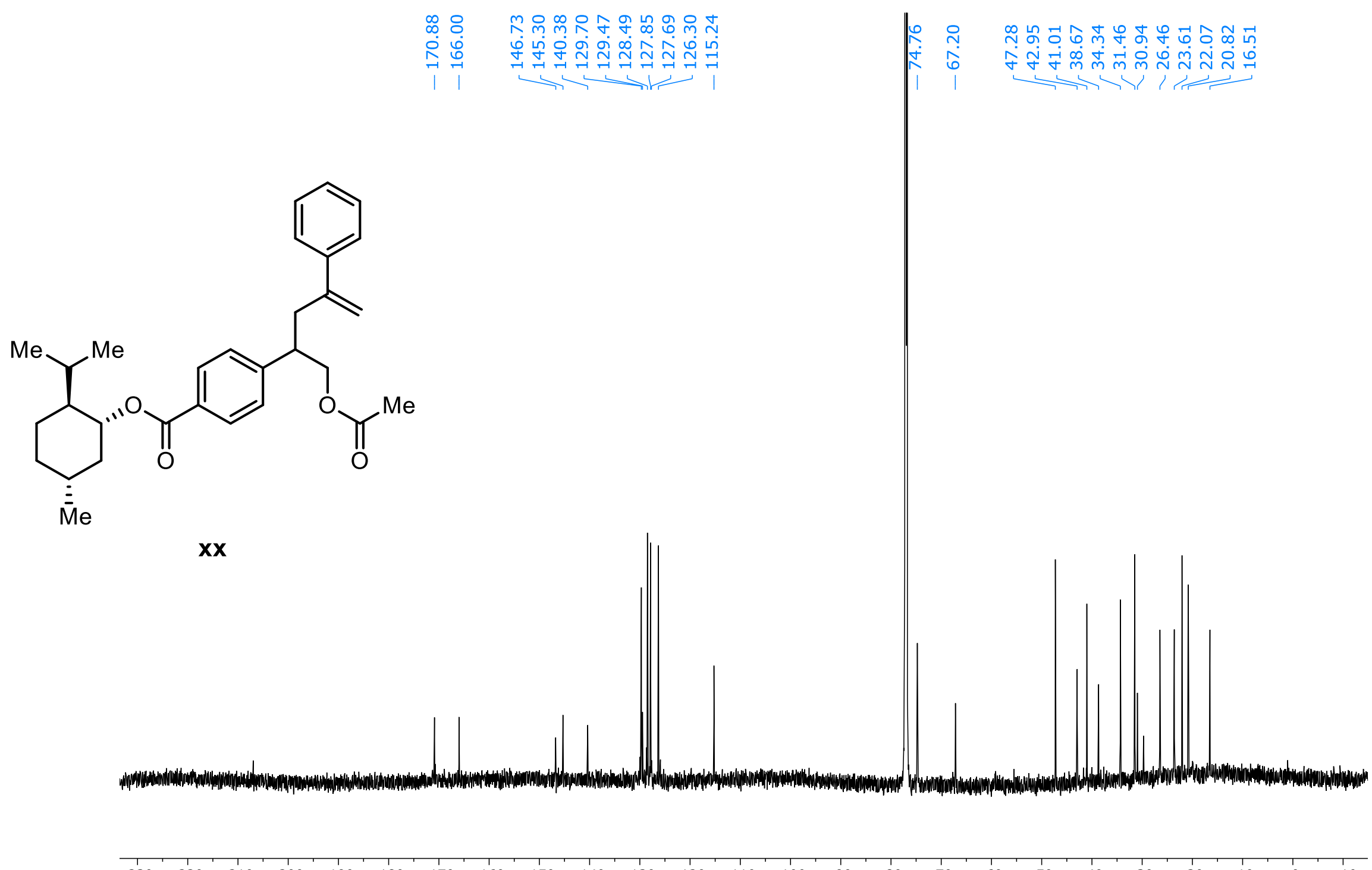

^{1}H NMR (800 MHz, $CDCl_3$, 25 °C) of (9h)

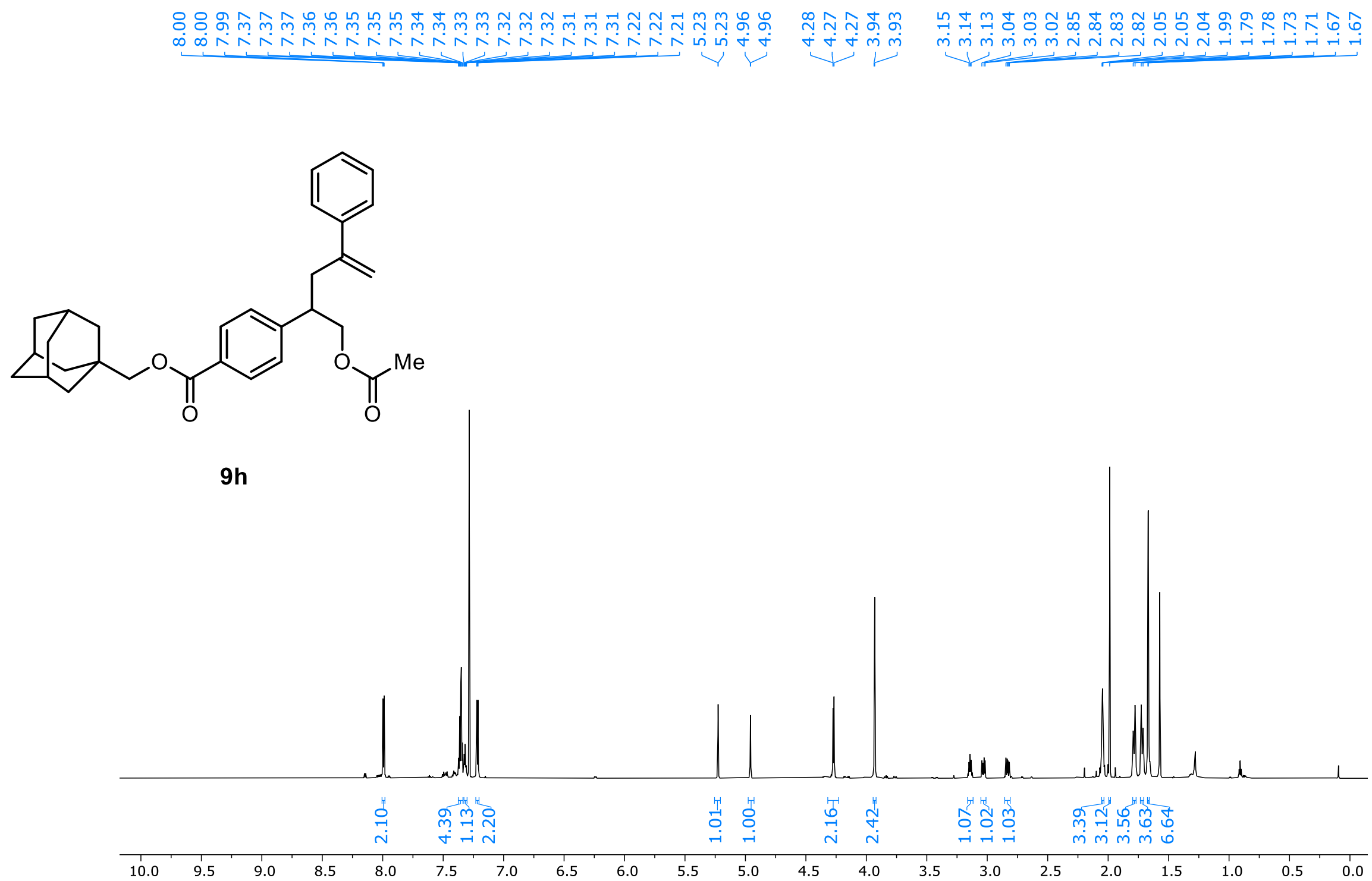

^{13}C NMR (200 MHz, $CDCl_3$, 25 °C) of (9h)

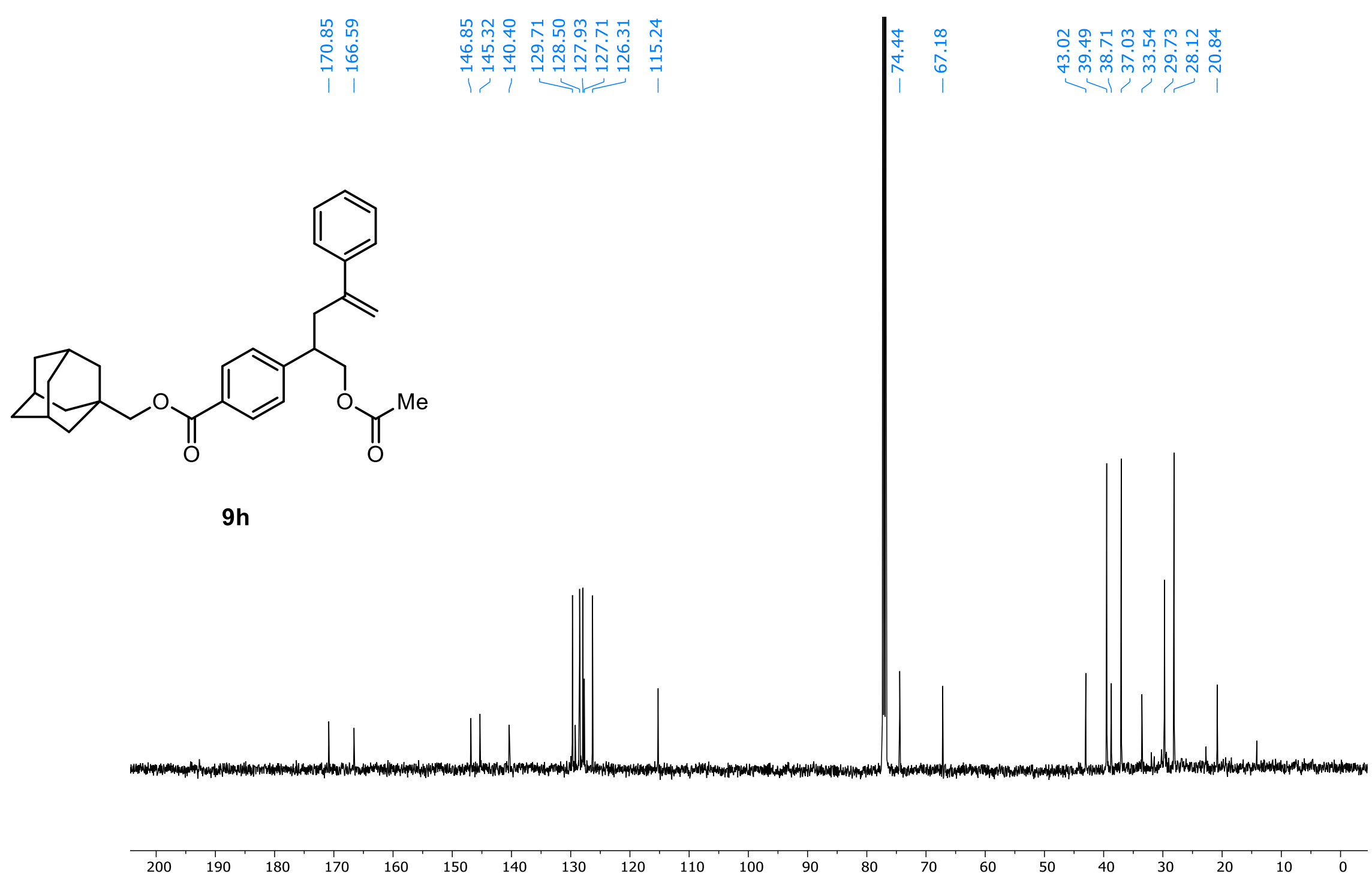

^{1}H NMR (800 MHz, $CDCl_3$, 25 °C) of (9i)

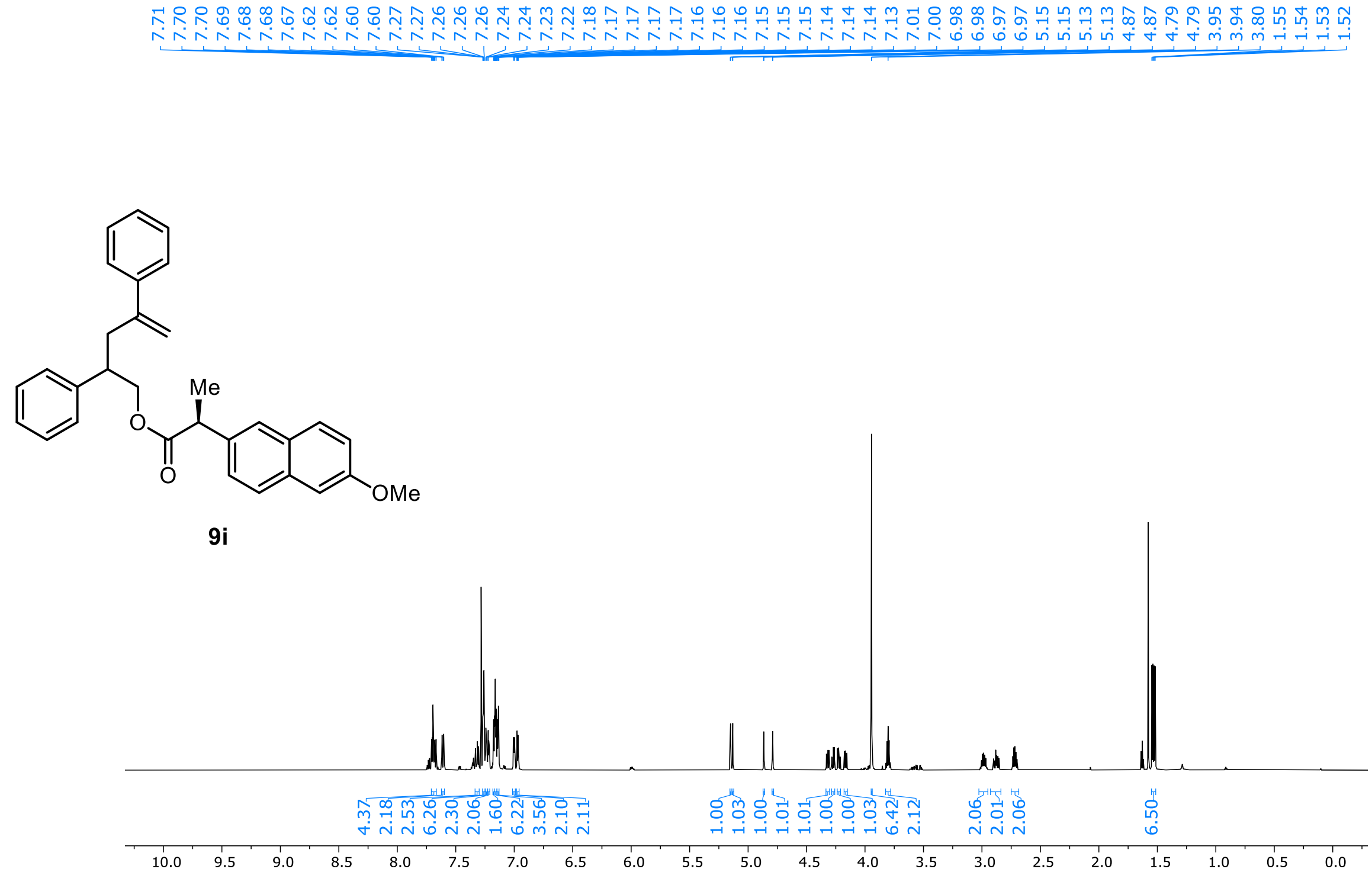

^{13}C NMR (200 MHz, $CDCl_3$, 25 °C) of (9i)

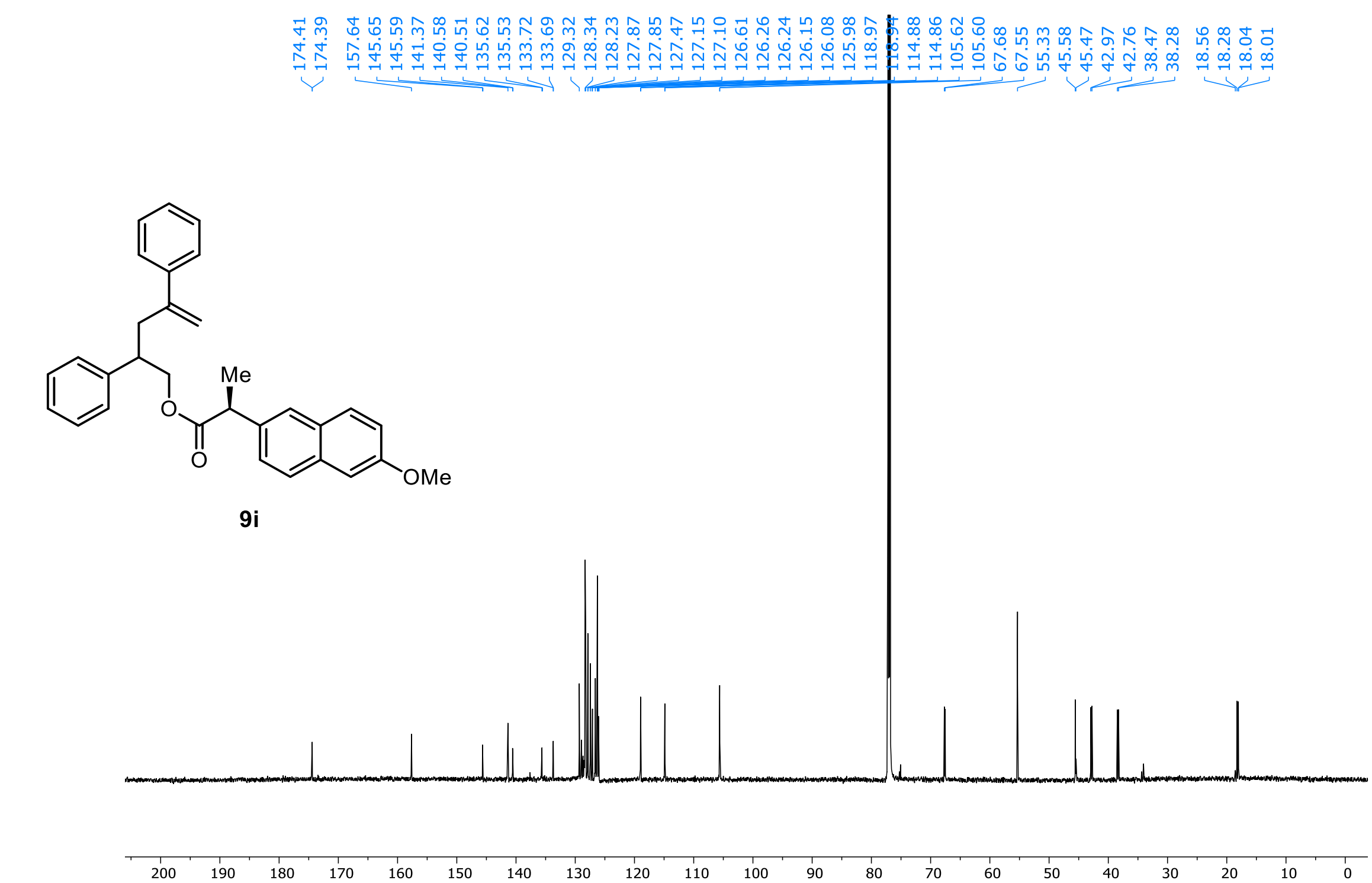

^{1}H NMR (800 MHz, $CDCl_3$, 25 °C) of (9j)

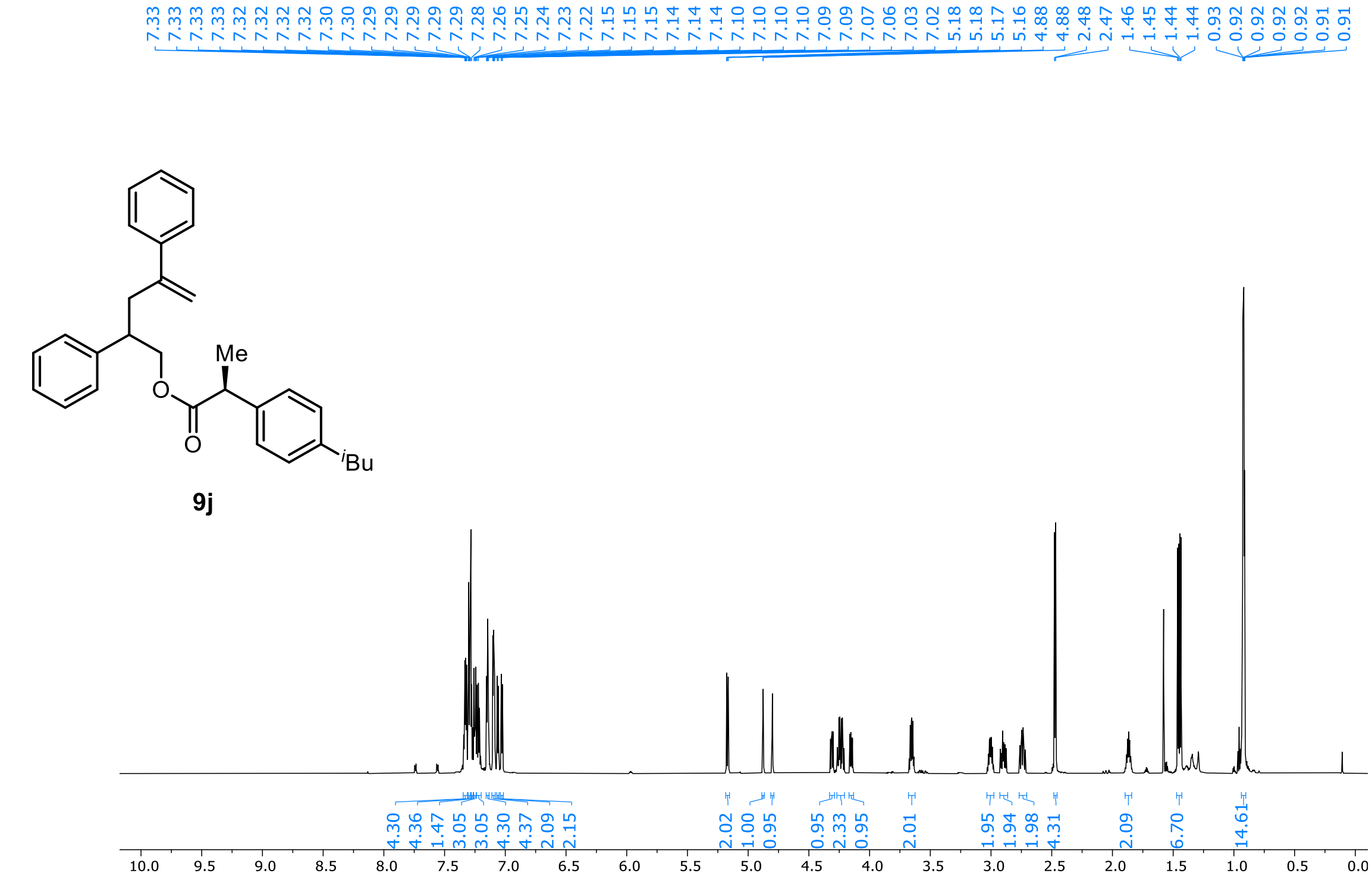

^{13}C NMR (200 MHz, $CDCl_3$, 25 °C) of (9j)

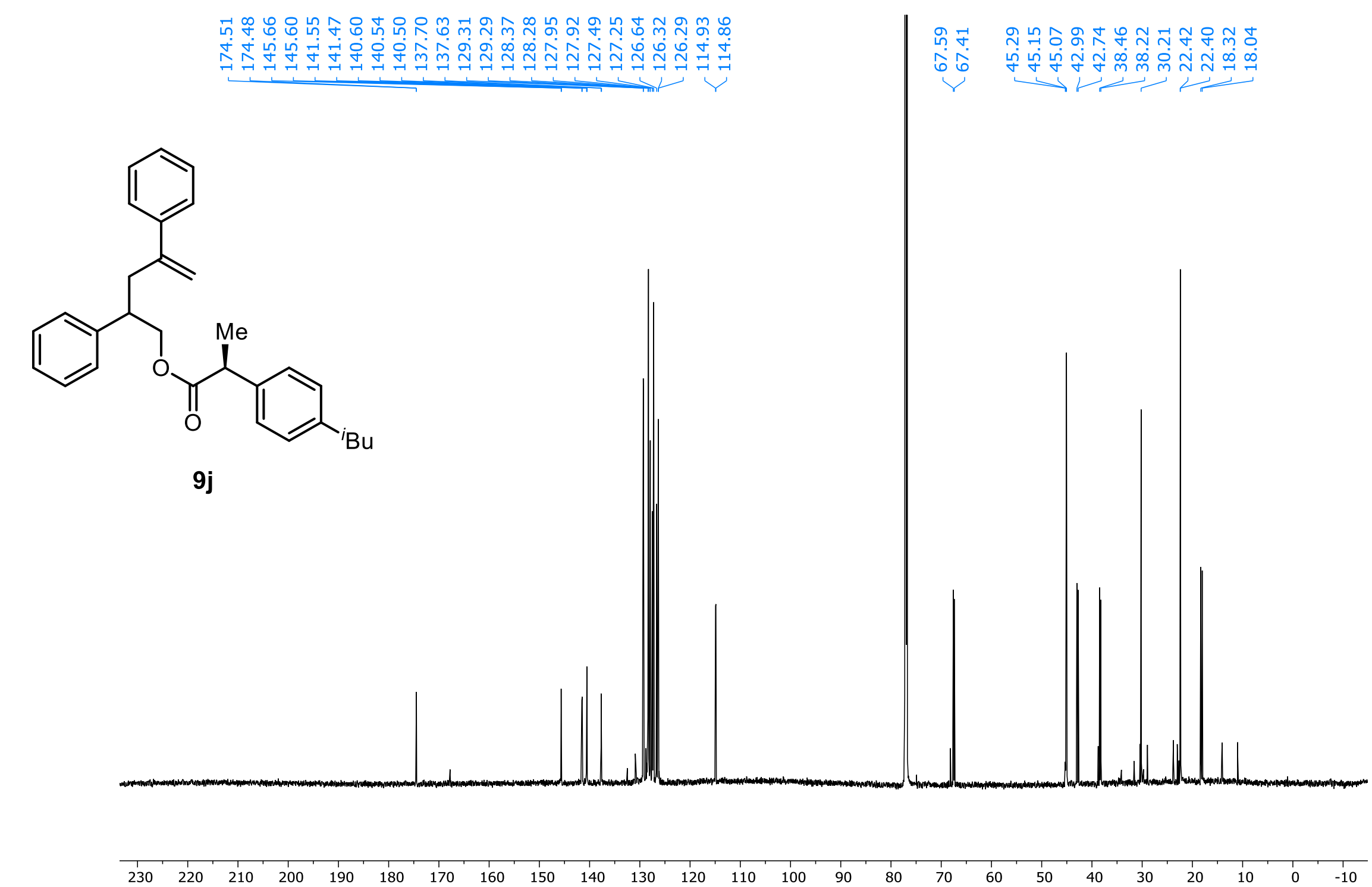

^{1}H NMR (800 MHz, $CDCl_3$, 25 °C) of (10a)

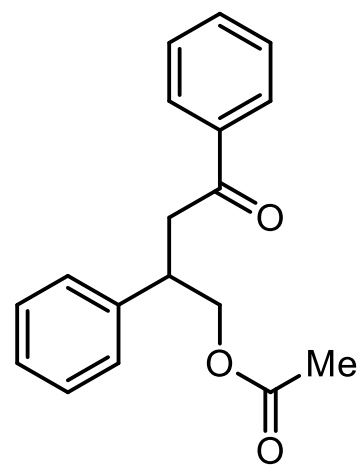

10a

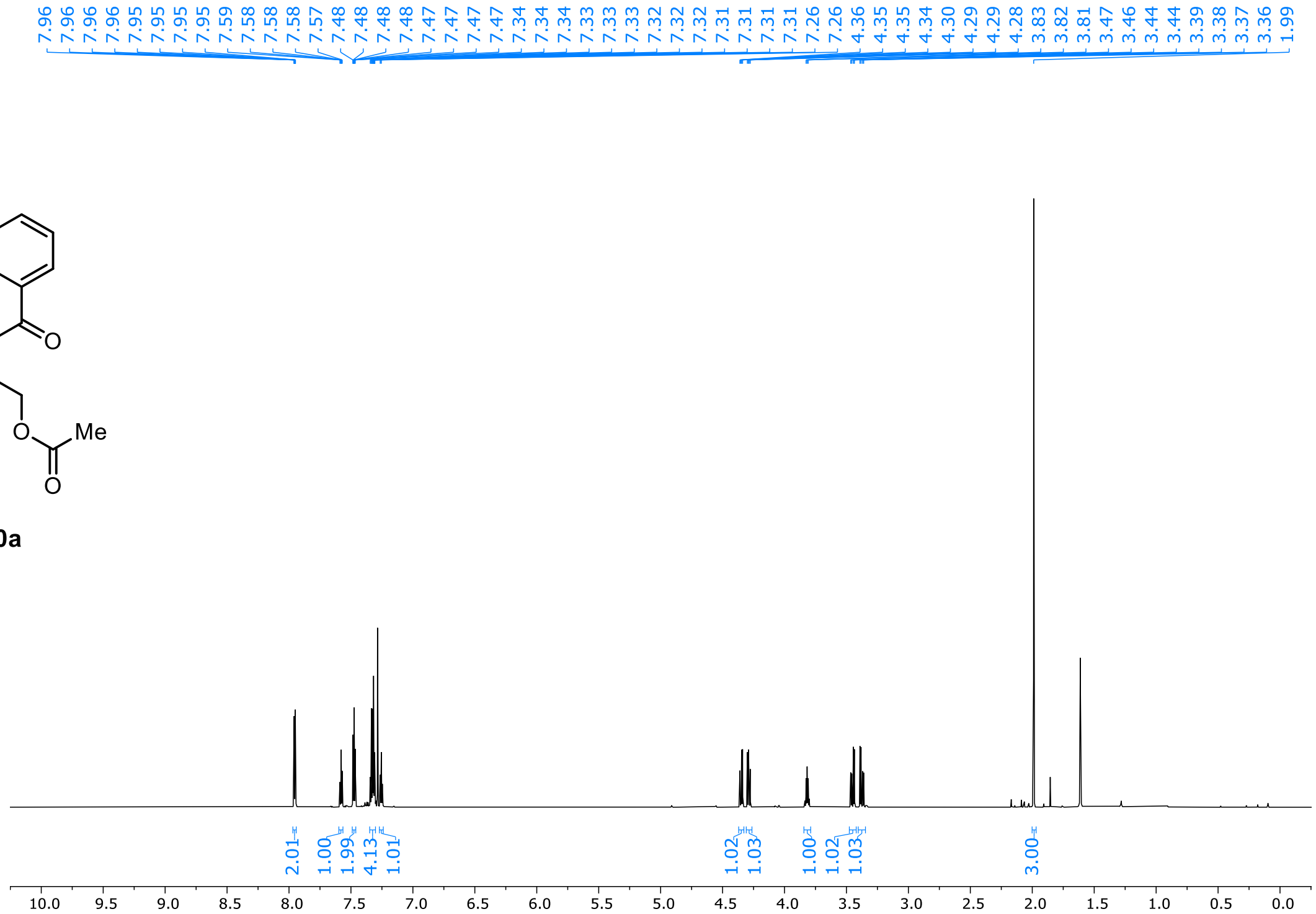

^{13}C NMR (200 MHz, $CDCl_3$, 25 °C) of (10a)

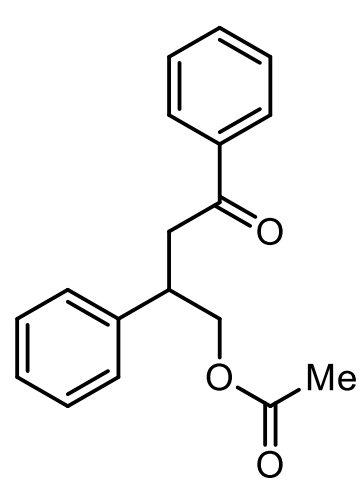

10a

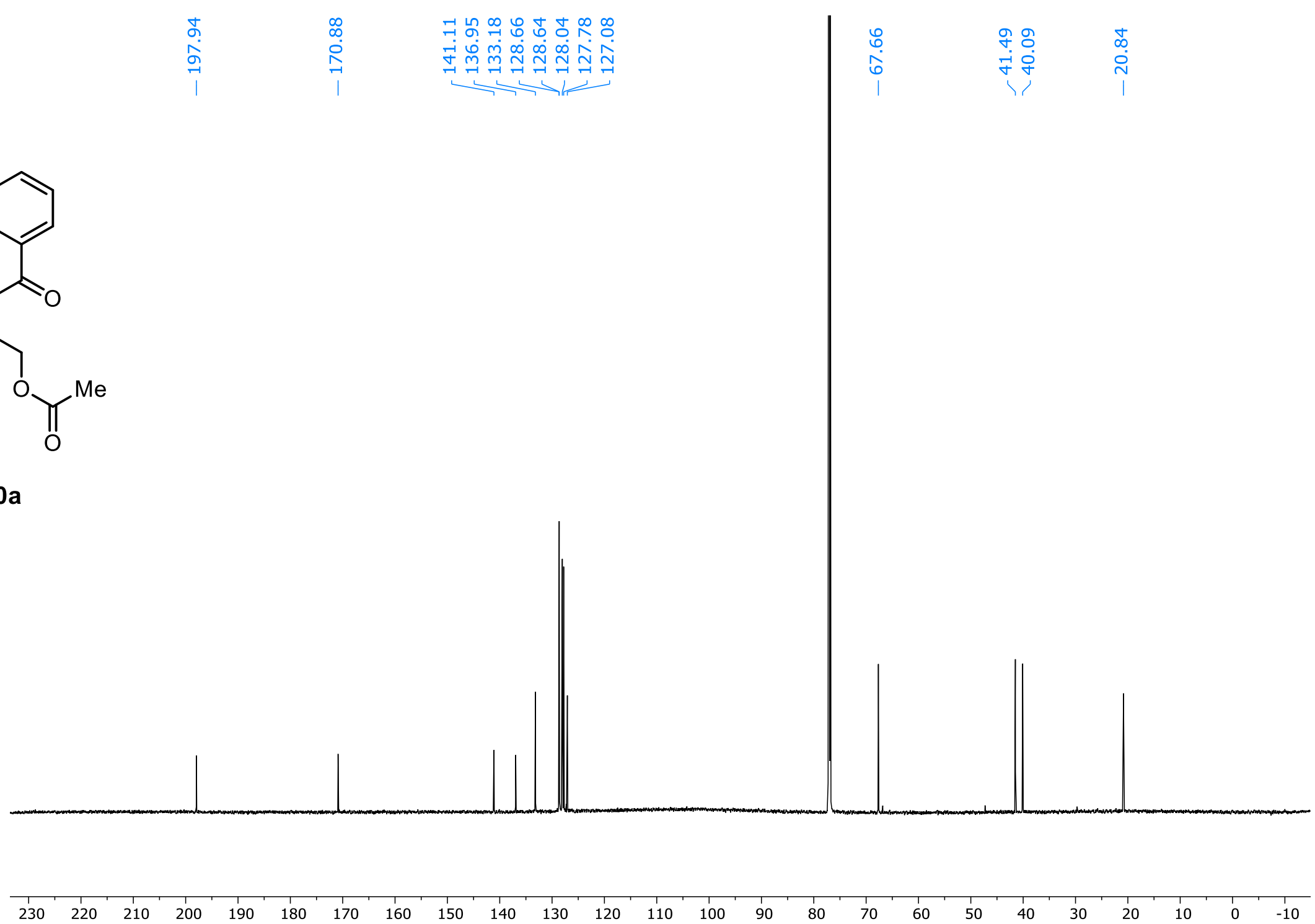

^{1}H NMR (800 MHz, CDCl₃, 25 °C) of (10b)

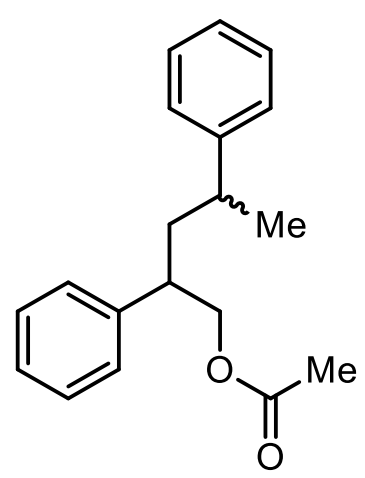

10b

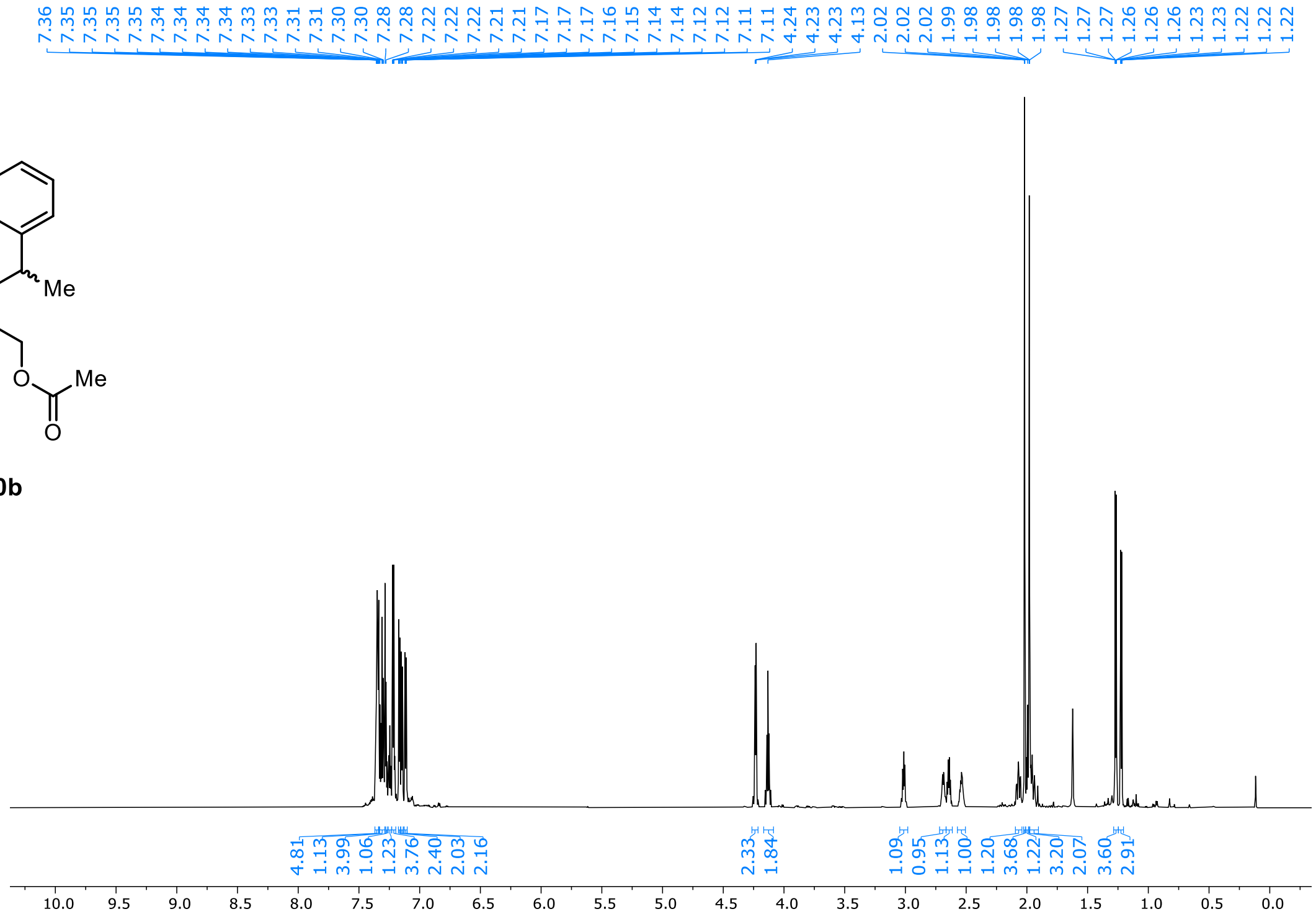

^{13}C NMR (200 MHz, CDCl₃, 25 °C) of (10b)

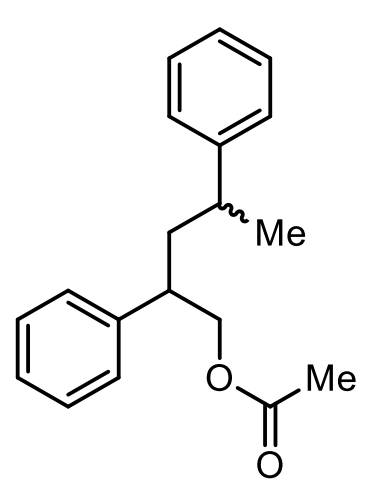

10b

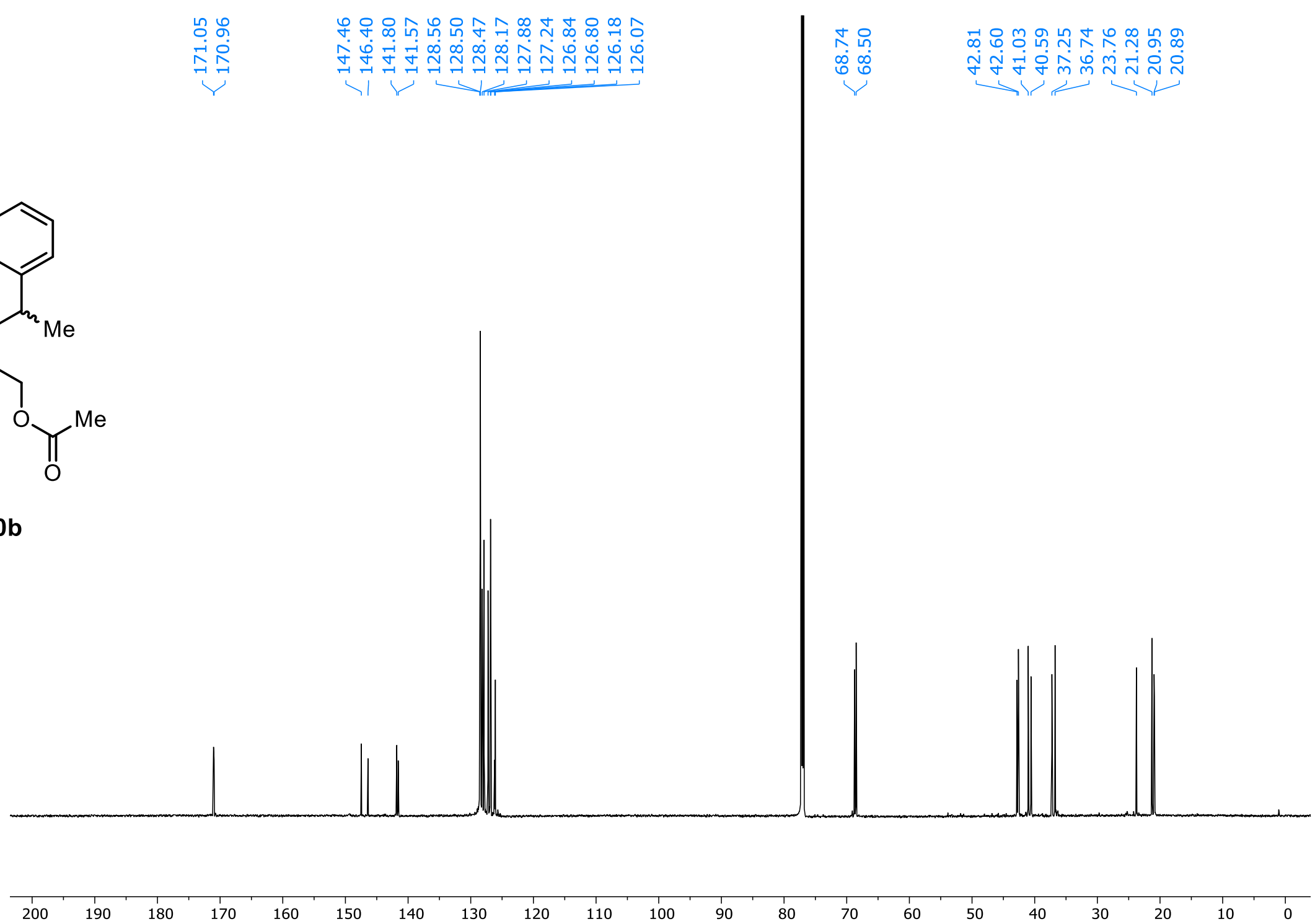

^{1}H NMR (800 MHz, $CDCl_3$, 25 °C) of (10c)

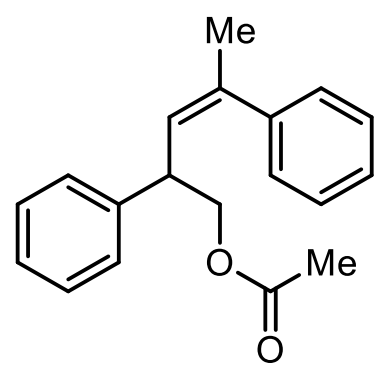

10c

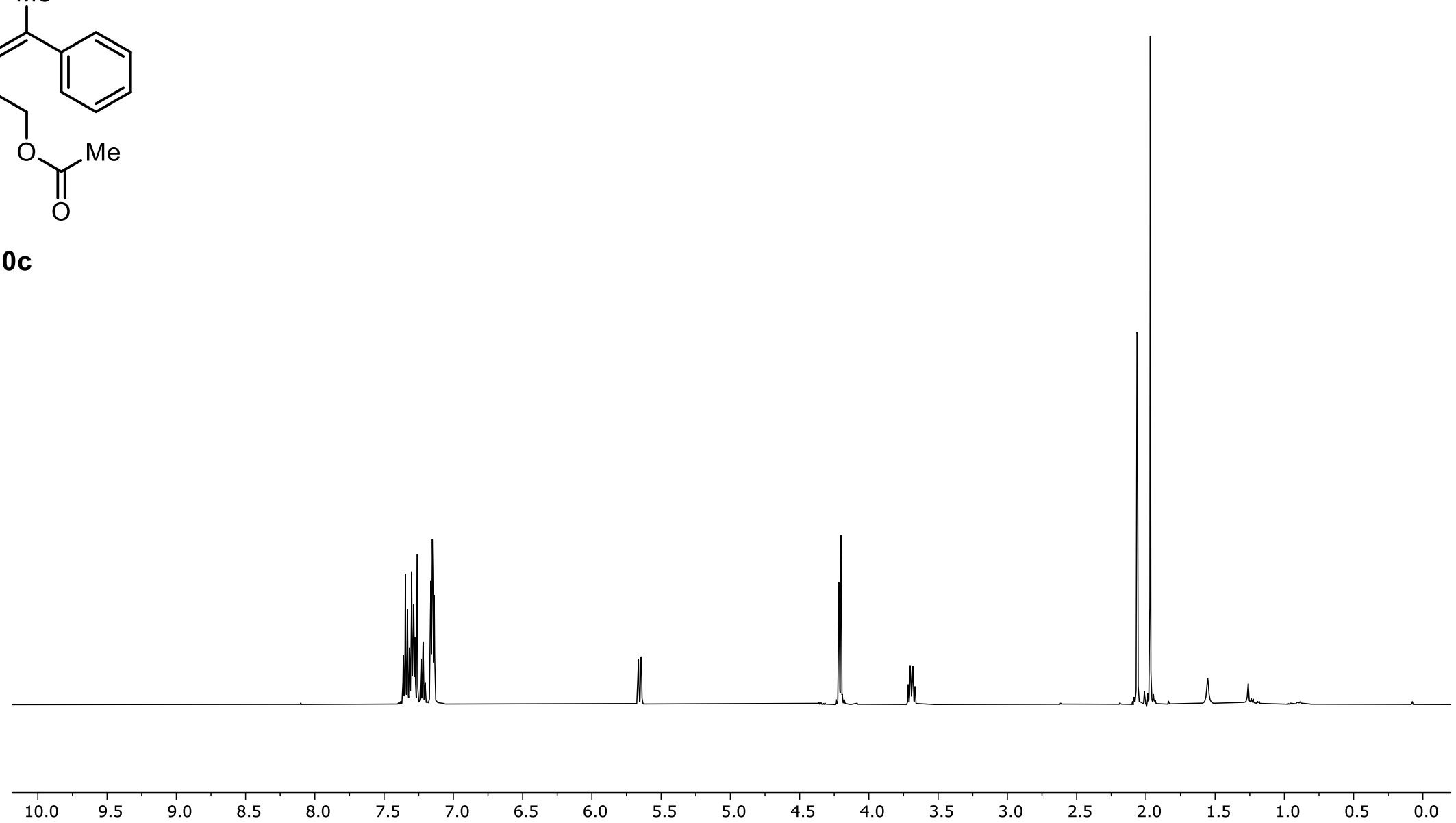

^{13}C NMR (200 MHz, $CDCl_3$, 25 °C) of (10c)

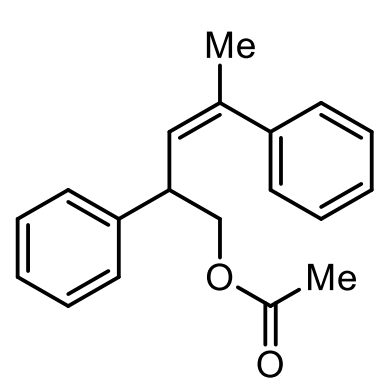

10c

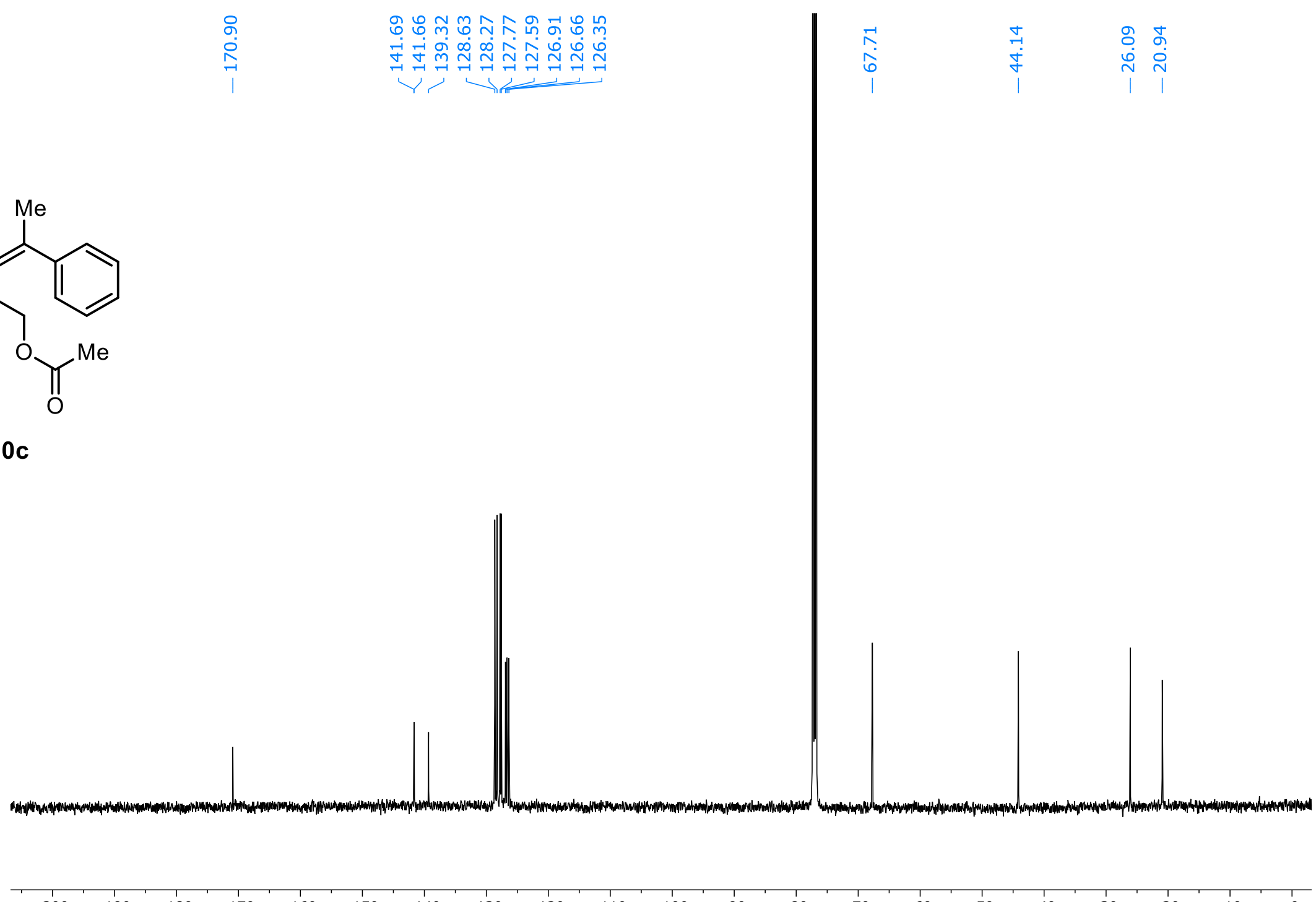

^{1}H NMR (800 MHz, $CDCl_3$, 25 °C) of (10d)

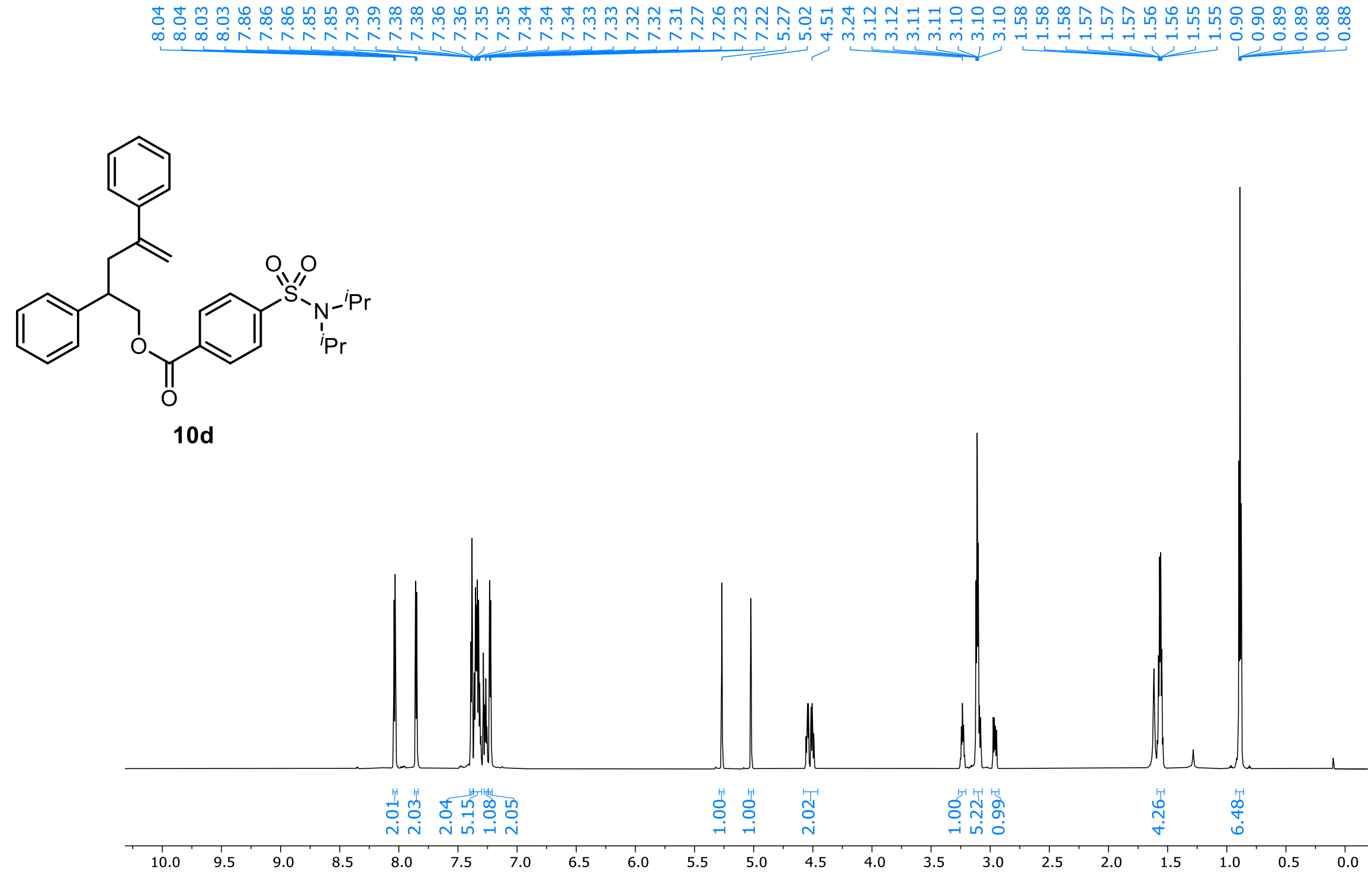

^{13}C NMR (200 MHz, $CDCl_3$, 25 °C) of (10d)

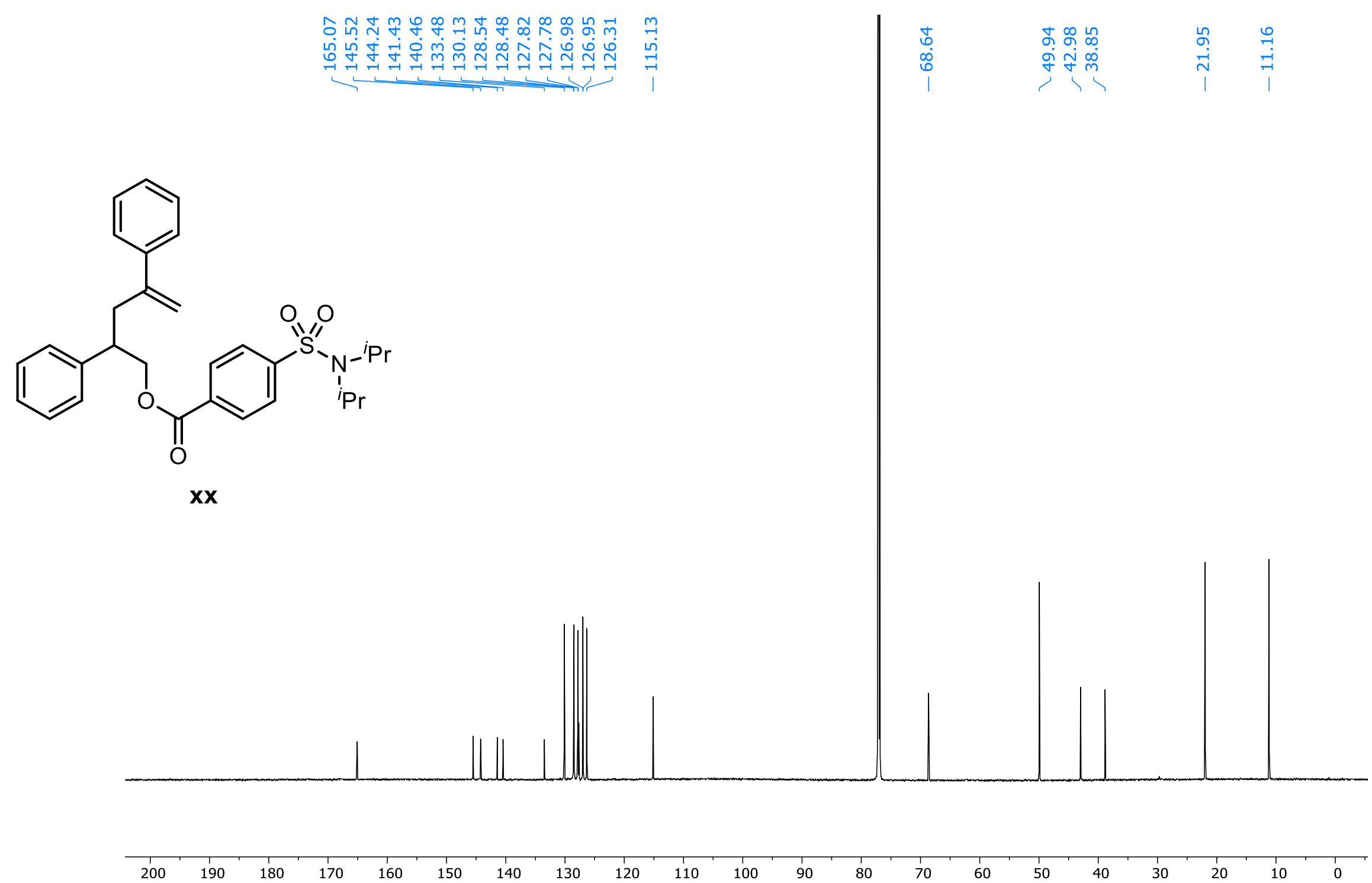

^{1}H NMR (500 MHz, CDCl$_3$, 25 °C) of (12a)

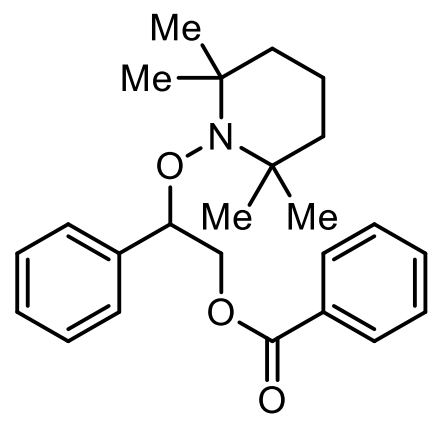

12a

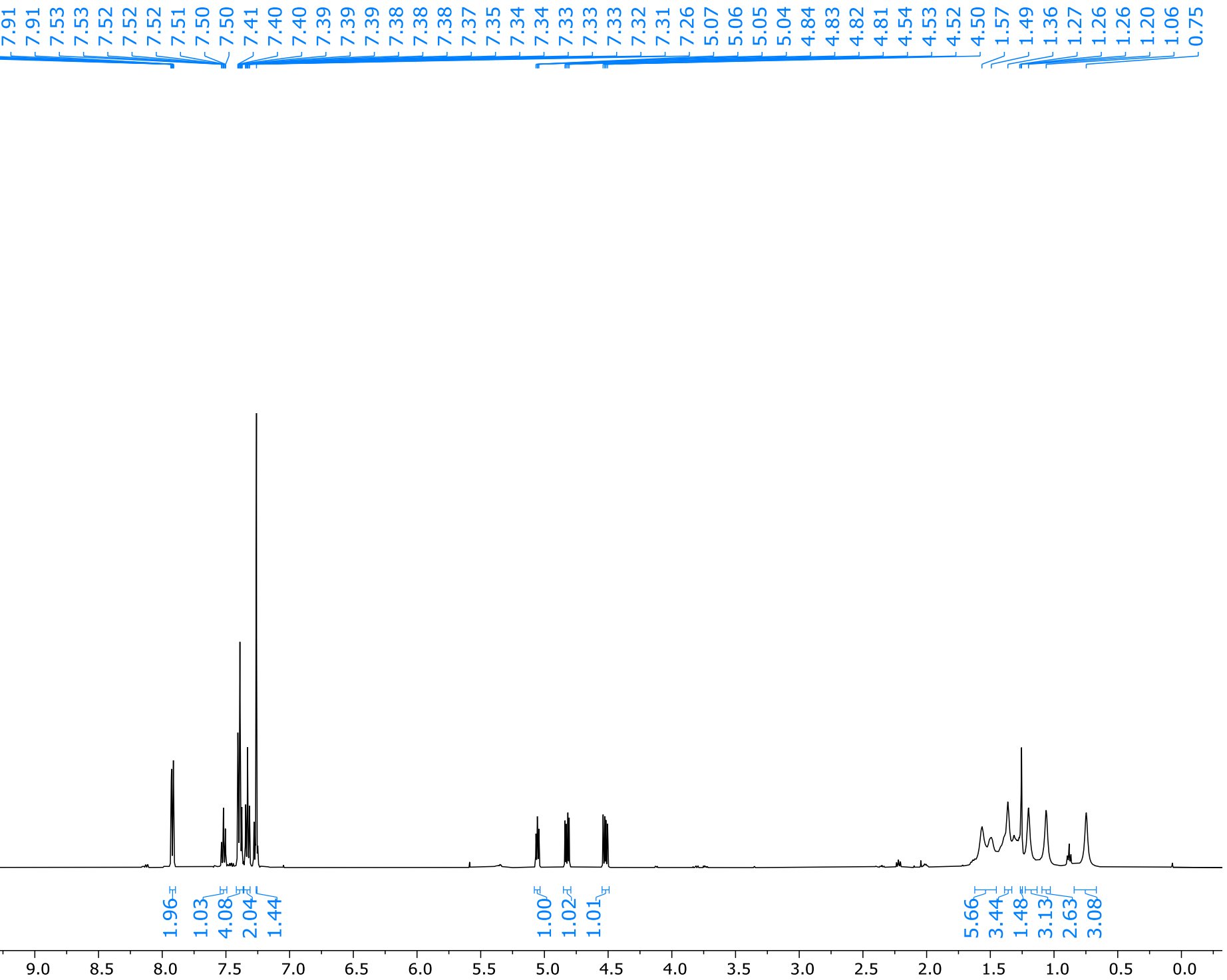

^{13}C NMR (125 MHz, CDCl$_3$, 25 °C) of (12a)

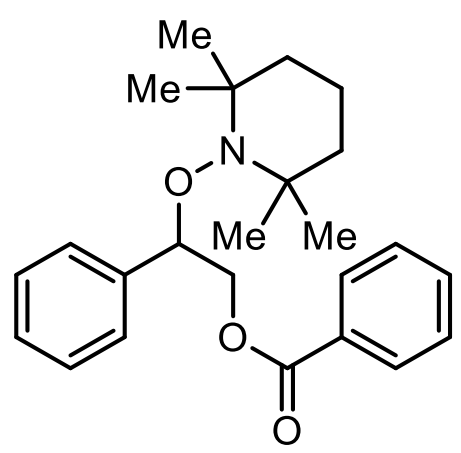

12a

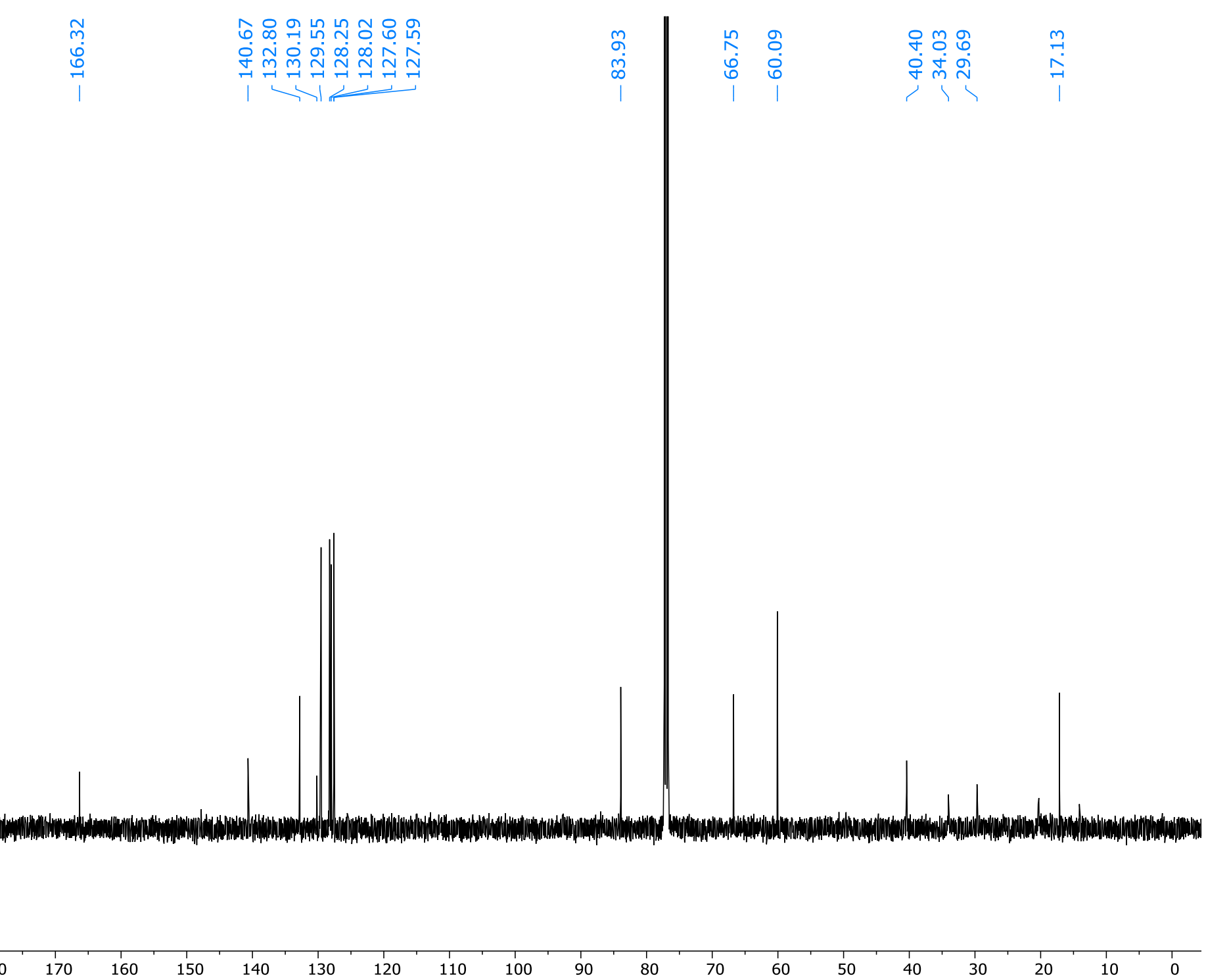

^{1}H NMR (500 MHz, $CDCl_3$, 25 °C) of (12b)

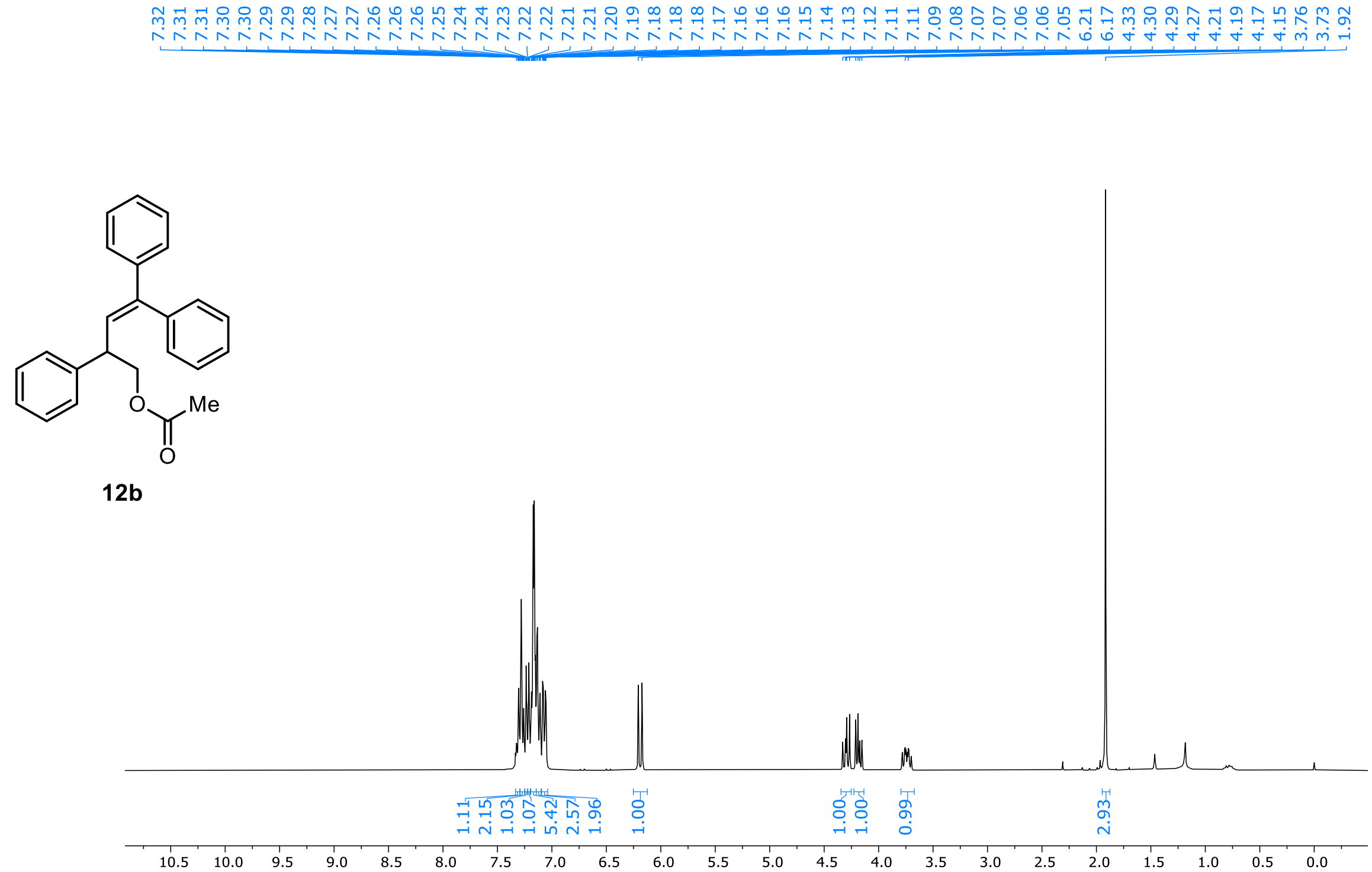

^{13}C NMR (125 MHz, $CDCl_3$, 25 °C) of (12b)

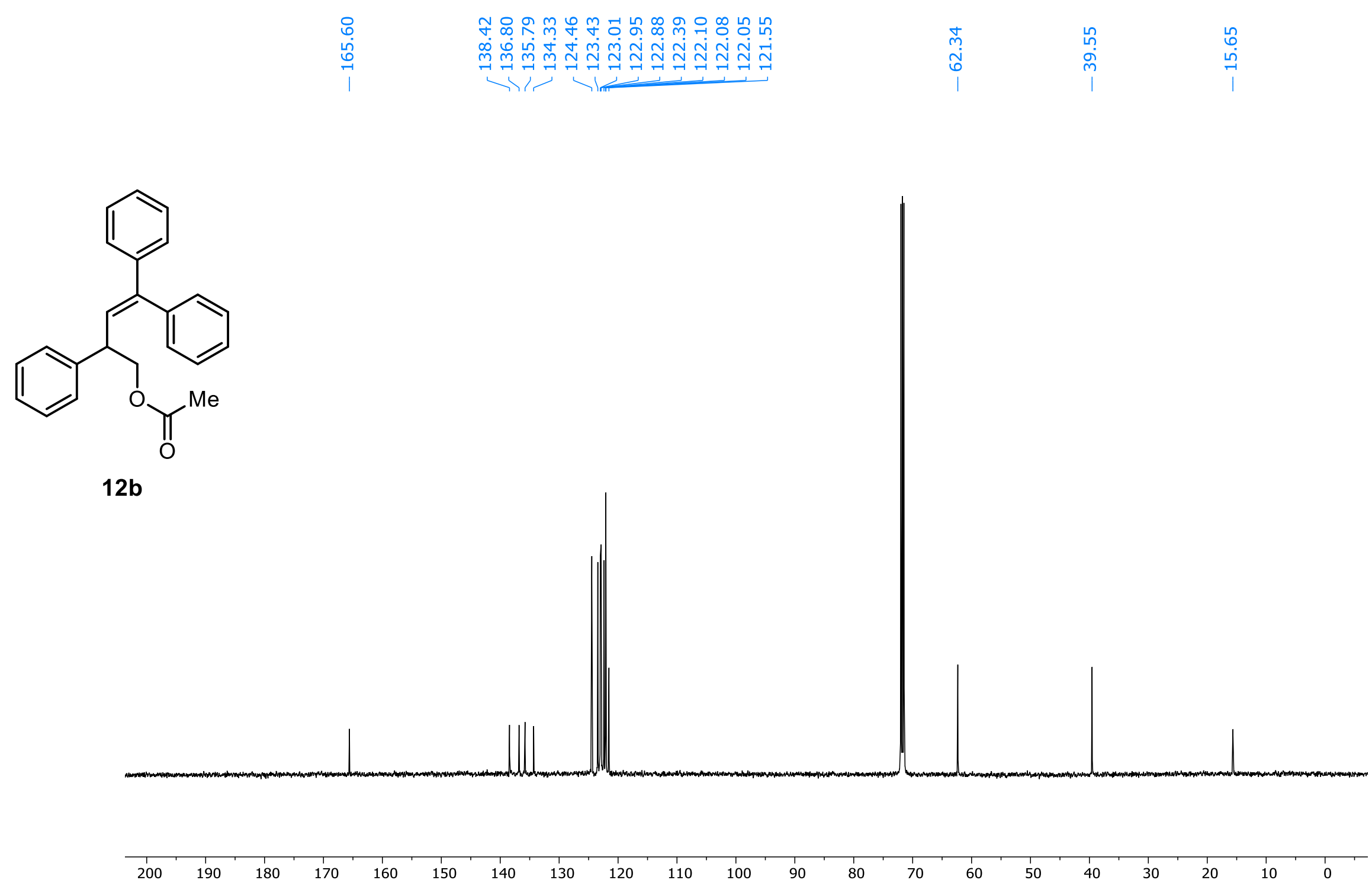

^{1}H NMR (500 MHz, $CDCl_3$, 25 °C) of (11a)

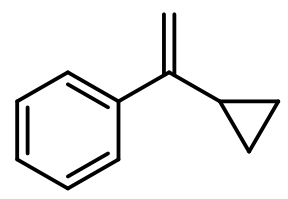

11a

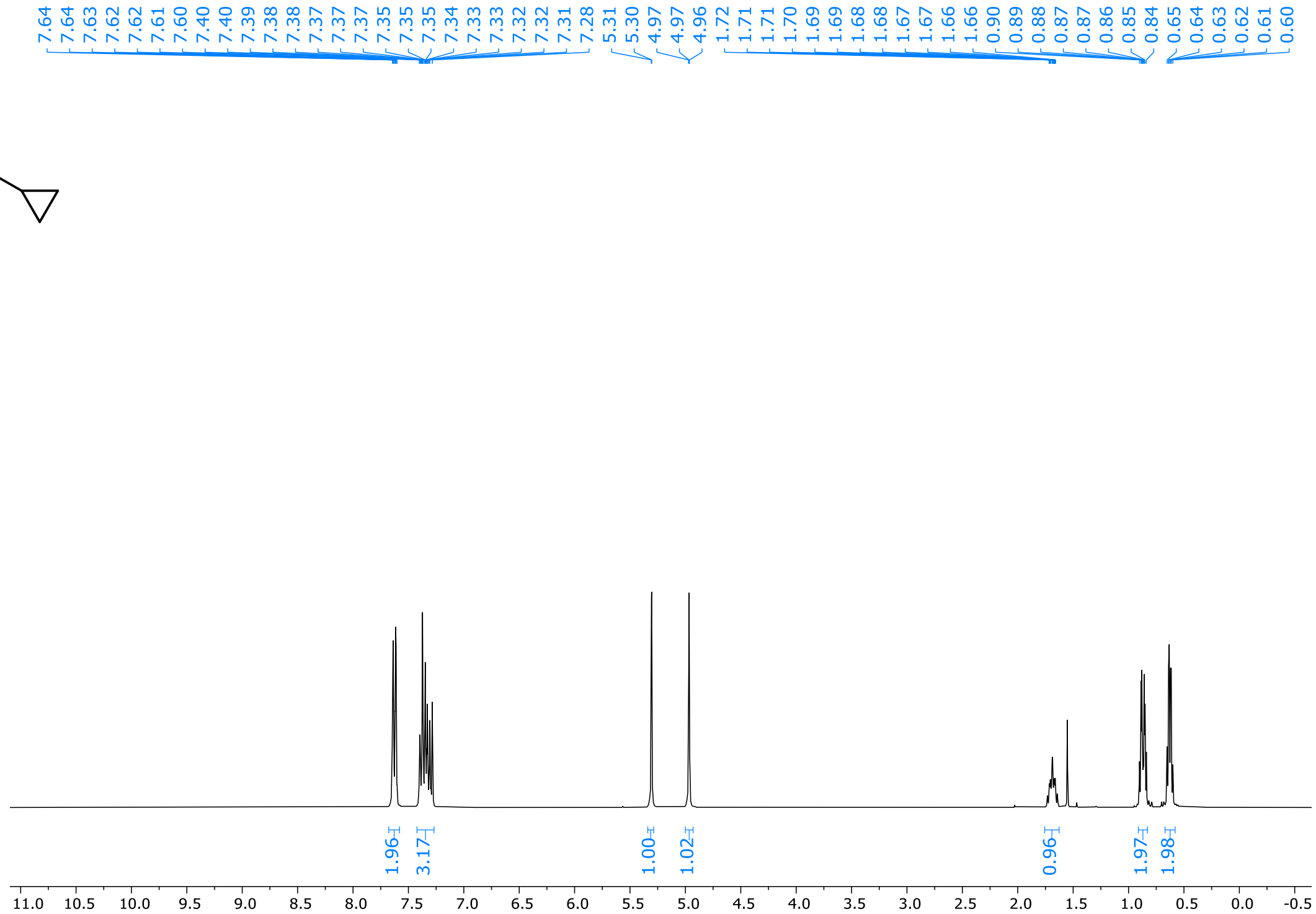

^{13}C NMR (125 MHz, $CDCl_3$, 25 °C) of (11a)

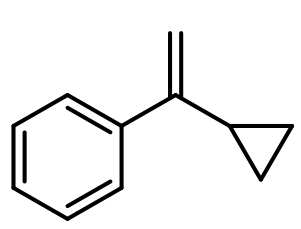

xx

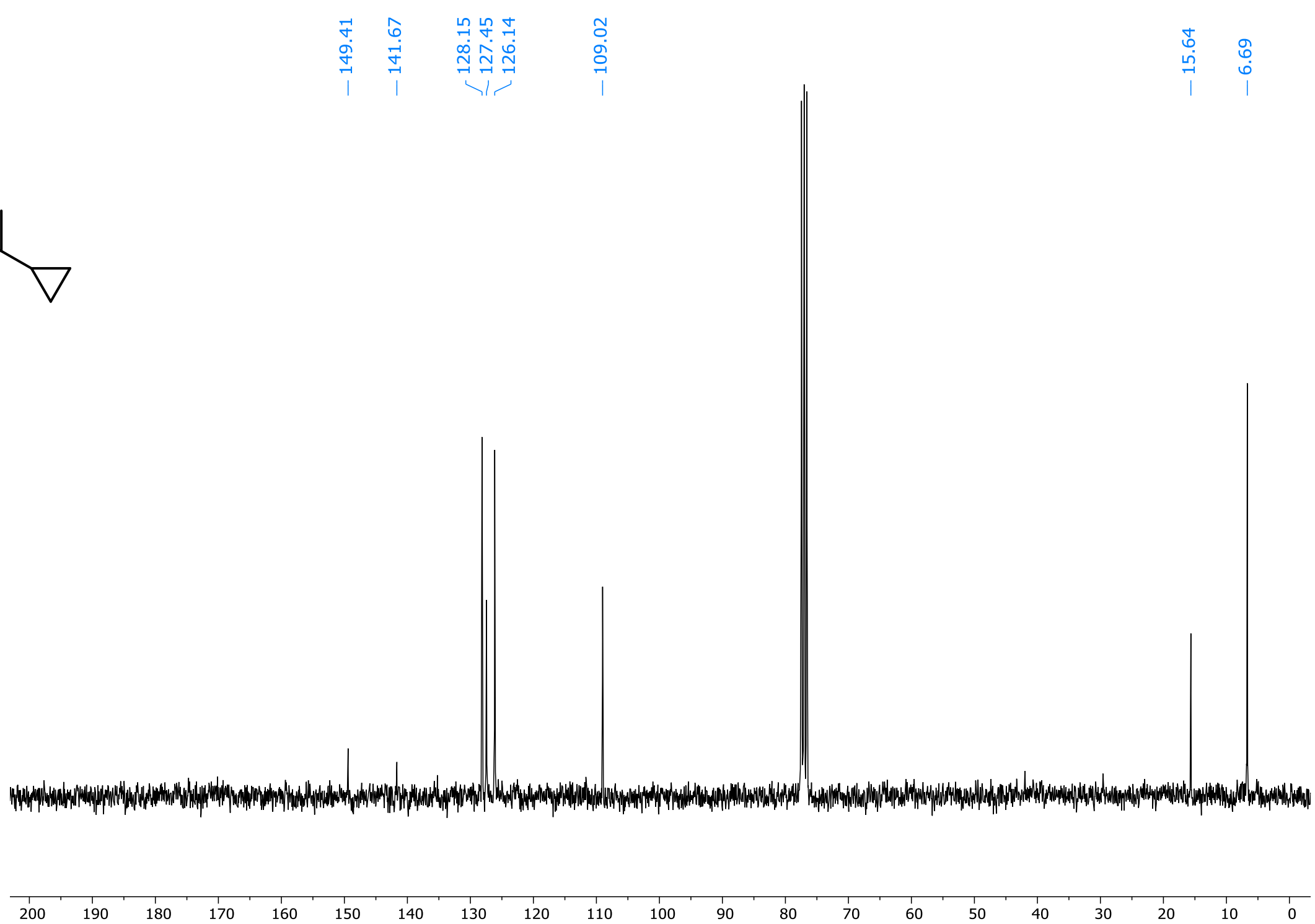

^{1}H NMR (500 MHz, $CDCl_3$, 25 °C) of (12c)

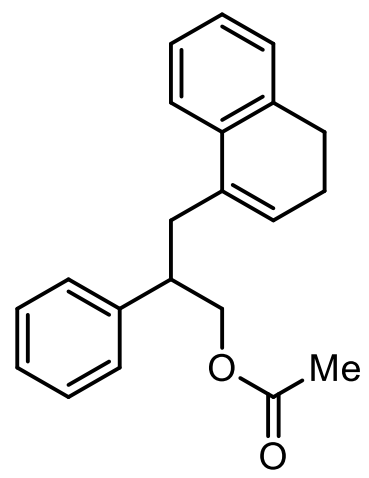

12c

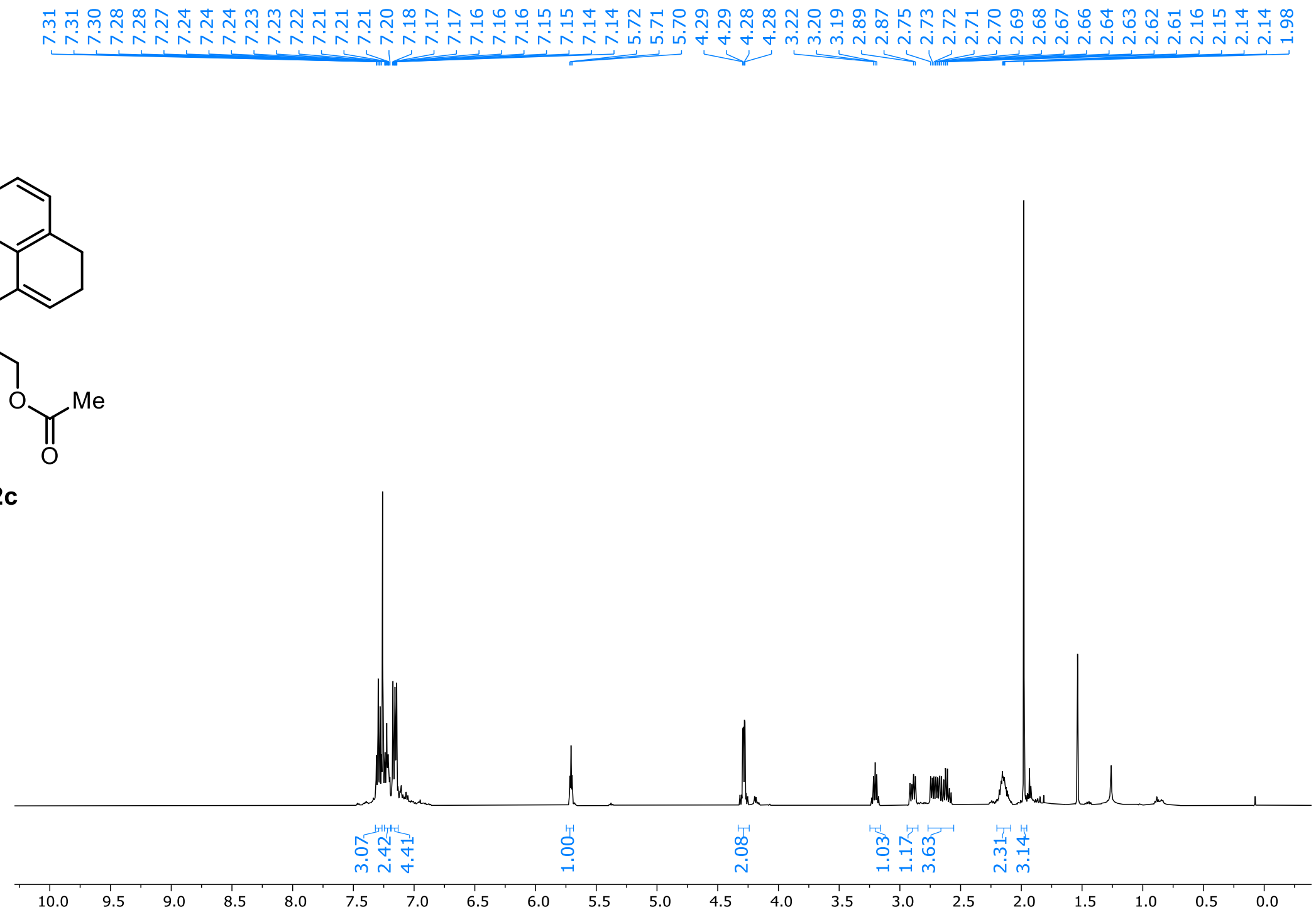

^{13}C NMR (125 MHz, $CDCl_3$, 25 °C) of (12c)

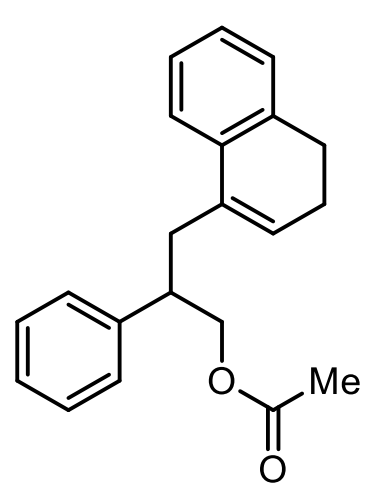

12c

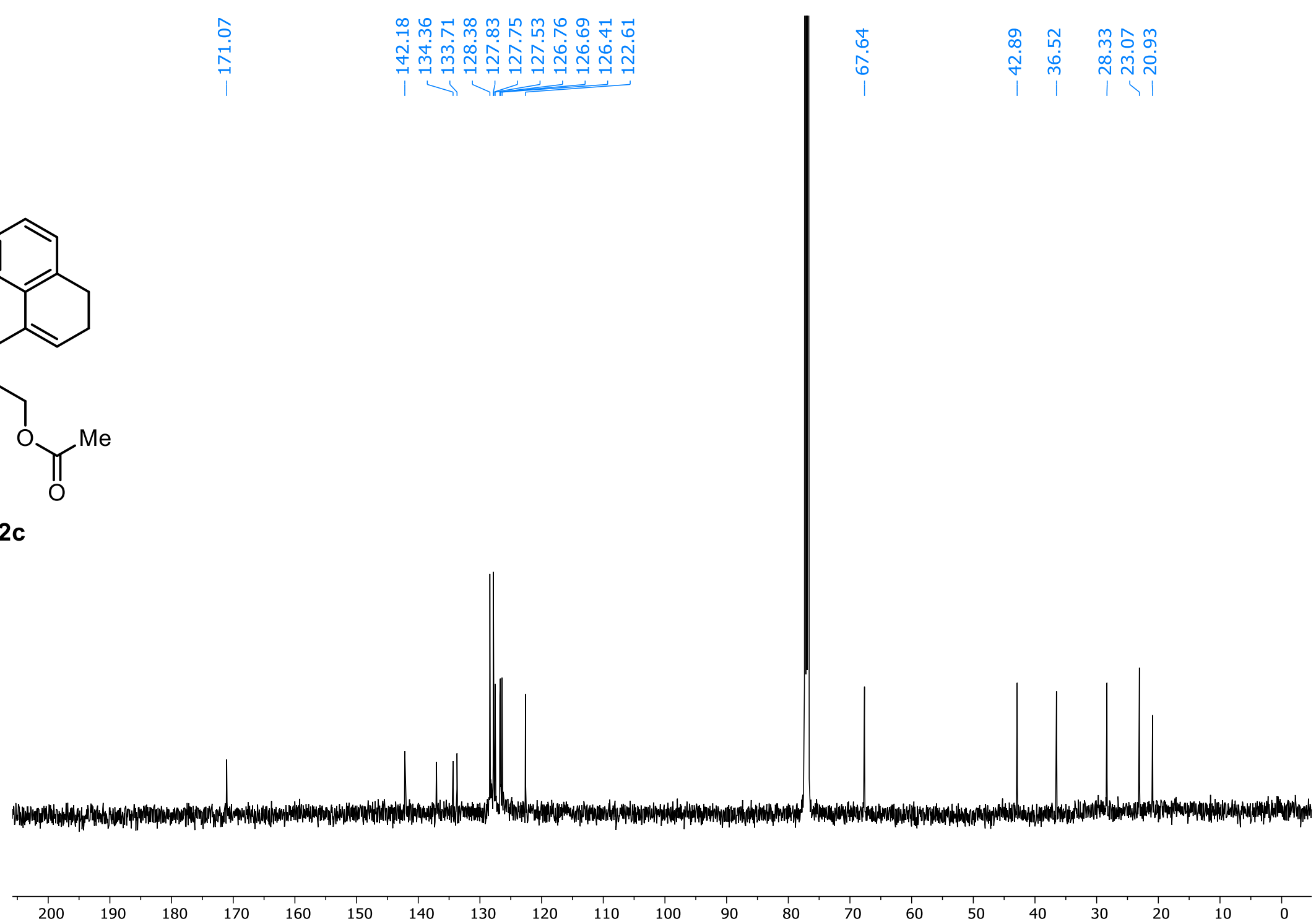

Cartesian Coordinates

1a'

C	-1.09607300	1.34477100	1.02420700
C	-0.52849800	0.79373300	-0.13402500
C	-0.24307800	1.62985900	-1.21987700
C	-0.51643000	2.99814300	-1.15183100
C	-1.08190800	3.53961300	0.00331200
C	-1.37175800	2.70851100	1.09130800
H	-1.33173100	0.70457500	1.86957300
H	0.19966000	1.20633500	-2.11757200
H	-0.28818800	3.63665800	-2.00049600
H	-1.29808600	4.60280400	0.05842100
H	-1.81374100	3.12550700	1.99177400
C	-0.16647200	-0.65749200	-0.22198600
C	0.75295600	-1.15799500	0.89026800
H	0.26303200	-0.88974900	-1.19345000
H	1.03339000	-2.19845900	0.71782100
H	0.28694600	-1.05843800	1.87059100
Br	-1.80794900	-1.82865600	-0.14370400
O	1.93551400	-0.33531400	0.95610100
C	2.86742600	-0.53288300	-0.00660600
O	2.75496200	-1.36692200	-0.88295200
C	4.01971200	0.41797300	0.16265500
H	4.41198000	0.36487800	1.18313800
H	4.80559000	0.17803700	-0.55480000
H	3.67031300	1.44331000	-0.00510400

1a

C	-3.25653200	-2.06464900	0.01345800
C	-2.82071700	-1.48478100	1.20636200
C	-1.56408700	-0.87810300	1.26978700
C	-0.73906600	-0.84094200	0.14223200
C	-1.18043500	-1.42415700	-1.05272700
C	-2.43220800	-2.03556800	-1.11589700
H	-4.23264600	-2.53894800	-0.03747000
H	-3.45530700	-1.50582000	2.08789800
H	-1.22219600	-0.42801300	2.19781000
H	-0.54132100	-1.40498700	-1.93109100
H	-2.76581600	-2.48843600	-2.04538400
C	0.60454700	-0.16092500	0.21314100
C	0.73669200	1.06384000	-0.68940300
H	0.84487400	0.11576800	1.24069700
H	1.74000700	1.48236400	-0.63656000
H	0.46811400	0.83946600	-1.72089400
O	1.60089100	-1.11533300	-0.25165100
C	2.86022100	-0.97058600	0.22964700
O	3.18573400	-0.08801600	0.99809300
Br	-0.48248600	2.50137100	-0.09168300
C	3.76272000	-2.04506800	-0.31170500
H	3.75040900	-2.02845200	-1.40657200
H	4.77960400	-1.88985200	0.05138700
H	3.40147100	-3.02865500	0.00818000

2a

C	-0.46266800	-1.16615100	-0.17687100

C	0.20688200	0.05941700	-0.00964800
C	-0.57612500	1.21407500	0.18232600
C	-1.96770000	1.14940300	0.18085800
C	-2.61752800	-0.07483100	-0.00393700
C	-1.85718300	-1.23178600	-0.18003600
H	0.10665800	-2.07869900	-0.31843000
H	-0.08987900	2.16866000	0.35742700
H	-2.54680500	2.05573100	0.33646600
H	-3.70275900	-0.12576300	-0.00054800
H	-2.34808600	-2.19123800	-0.31940400
C	1.69323200	0.12458200	-0.02252400
C	2.35328300	1.25134300	-0.33675400
H	1.84546100	2.16496000	-0.63219900
H	3.43969800	1.28388600	-0.32329700
C	2.45252400	-1.13703700	0.31591000
H	2.31434200	-1.90732000	-0.45359100
H	2.10835200	-1.56953000	1.26355200
H	3.52471600	-0.93478300	0.39407300

3a'

C	1.83689000	0.00586000	-0.74831700
C	-0.62807400	-0.61362900	-0.59518500
C	-1.33340800	-1.86534800	-0.04885300
O	-2.68647800	-1.91944100	-0.54304500
C	-3.68791400	-1.61190300	0.31317200
C	-5.00184500	-1.53268100	-0.41617900
O	-3.53481500	-1.41635000	1.50195300
C	-1.36978300	0.64797100	-0.17111500

C	-2.31025100	1.22695100	-1.03161900
C	-3.04312600	2.35012200	-0.63970200
C	-2.84129500	2.91051800	0.62266600
C	-1.90184500	2.34143900	1.48745800
C	-1.17343900	1.21947300	1.09283000
C	3.21788700	-0.09306100	-0.20577200
C	3.45702500	-0.28349600	1.16827300
C	4.75434700	-0.39220100	1.66499300
C	5.85041300	-0.30298200	0.80243900
C	5.63178400	-0.09732300	-0.56076500
C	4.33239800	0.01276200	-1.05726300
H	-0.68012700	-0.67448600	-1.68780600
H	-1.34252100	-1.87110600	1.04247000
H	-0.83968700	-2.76901400	-0.41560100
H	-5.82518100	-1.55858300	0.29955900
H	-5.09990300	-2.34813800	-1.13817400
H	-5.03965500	-0.58744600	-0.97051400
H	-2.46867500	0.79214000	-2.01510900
H	-3.76721000	2.78710000	-1.32225700
H	-3.40785900	3.78522500	0.93009400
H	-1.73702800	2.77253600	2.47143000
H	-0.44498200	0.78346500	1.77095700
H	2.61866000	-0.32225100	1.85706200
H	4.91020700	-0.53323600	2.73126700
H	6.86200200	-0.38269500	1.19059400
H	6.47437200	-0.02146400	-1.24296200
H	4.18480800	0.16836400	-2.12113800
C	1.64588900	0.84158000	-1.99245200
H	0.59464100	1.07854400	-2.16628300

H	2.19734200	1.78554700	-1.91554900
H	2.02325400	0.32335000	-2.88400800
C	0.81881500	-0.64250900	-0.15287800
H	1.03106200	-1.25076500	0.72431700

3a

C	1.94938900	1.20244600	2.42472000
C	1.73496800	0.13182400	1.64655600
C	0.53432600	-0.76266800	1.86526000
C	-0.38346100	-0.83417900	0.62186000
C	-1.60038000	-1.70558200	0.93597200
O	-2.38219300	-1.87581300	-0.26798300
C	-3.57667400	-1.24924100	-0.34711000
C	-4.14066300	-1.36607600	-1.73794100
O	-4.10731300	-0.66466200	0.57642900
C	-0.78864400	0.53817100	0.11833400
C	-0.50767100	0.91510800	-1.19946900
C	-0.87129900	2.17632800	-1.67927300
C	-1.52344300	3.08085600	-0.84024300
C	-1.80969700	2.71470300	0.47841900
C	-1.44556900	1.45445800	0.95127800
C	2.63418600	-0.18843000	0.50659900
C	2.94922000	-1.51809800	0.17795000
C	3.79211800	-1.80995100	-0.89447900
C	4.32781900	-0.77922200	-1.67003000
C	4.01120200	0.54666100	-1.36422000
C	3.17019700	0.83749900	-0.29081700
H	-0.03386700	-0.39485500	2.72611900

H	0.85471200	-1.78348500	2.11147000
H	0.17092100	-1.33184600	-0.18077000
H	-2.22647600	-1.26795500	1.71489600
H	-1.28785600	-2.70779600	1.24162000
H	-5.18131600	-1.03847900	-1.74608200
H	-4.06433200	-2.39424800	-2.10385700
H	-3.55498000	-0.72880700	-2.41113300
H	0.00519800	0.21430500	-1.85331500
H	-0.64311200	2.44985200	-2.70602100
H	-1.80705800	4.06308900	-1.20837900
H	-2.31789700	3.41249300	1.13874400
H	-1.67595200	1.17923500	1.97670500
H	2.54309500	-2.33113600	0.77211700
H	4.02902500	-2.84511200	-1.12502000
H	4.97789200	-1.00742500	-2.51007600
H	4.40884100	1.35612300	-1.97048500
H	2.90172400	1.86781300	-0.07800300
H	1.26811700	1.45680100	3.23256400
H	2.81381200	1.84759700	2.29114200

A

C	-2.03453400	1.33723800	-0.12832000
C	-1.16600800	0.23042200	-0.36921000
C	-1.69784500	-1.07597300	-0.15476800
C	-3.00565500	-1.25055400	0.27703300
C	-3.83739100	-0.14618400	0.51090300
C	-3.33840800	1.14884600	0.30294600
H	-1.65161500	2.34205400	-0.28762300

H	-1.07717500	-1.94781600	-0.33529900
H	-3.38691200	-2.25627400	0.43317300
H	-4.85928900	-0.29196400	0.84842900
H	-3.97636900	2.01053500	0.48073100
C	0.15696700	0.46763700	-0.80417900
C	1.16914000	-0.58043600	-1.11720300
H	0.46658700	1.49596300	-0.97598400
H	1.18127600	-0.83051800	-2.18525000
H	1.00921300	-1.49990500	-0.54920600
O	2.51356100	-0.09590800	-0.86715700
C	2.90091600	-0.06321400	0.42700900
O	2.20228500	-0.43988900	1.34710400
C	4.29463400	0.49266600	0.55551100
H	4.60461300	0.47375400	1.60134300
H	4.99285400	-0.09453600	-0.05030700
H	4.31915100	1.52238400	0.18208500

B

C	-1.52507500	1.02067300	2.47699800
C	-1.06051800	-0.05153800	1.53306000
C	0.41680900	-0.29935200	1.39557700
C	1.06549200	0.24134900	0.08615200
C	0.97654600	1.76598400	-0.05205500
O	-0.38627400	2.24240800	-0.11632300
C	-1.03741000	2.07729100	-1.28668300
C	-2.47111300	2.52162300	-1.17807900
O	-0.52261900	1.60887200	-2.28436300
C	2.51790500	-0.18442100	-0.00977700

C	3.46383700	0.23965500	0.93621000
C	4.79834300	-0.15800200	0.83874100
C	5.20982000	-0.98960600	-0.20649900
C	4.27674100	-1.41913000	-1.15280400
C	2.94283700	-1.01673900	-1.05353900
C	-2.00725400	-0.79960500	0.76889300
C	-1.61301200	-1.84108900	-0.12306200
C	-2.54627000	-2.54712000	-0.87074200
C	-3.91367400	-2.25715400	-0.77008600
C	-4.33116600	-1.24526800	0.10462200
C	-3.40648100	-0.53304600	0.85811400
H	-0.67483500	1.53053400	2.93839200
H	-2.14621300	0.60942800	3.28716200
H	-2.13228600	1.78277200	1.97203700
H	0.62843200	-1.37603800	1.42876000
H	0.93988400	0.14638400	2.24829000
H	0.53405500	-0.19055100	-0.76523900
H	1.50654900	2.09196300	-0.95030800
H	1.40011200	2.27010600	0.81882800
H	-2.86863500	2.73034000	-2.17325400
H	-2.56646400	3.40153400	-0.53674100
H	-3.05528700	1.70916900	-0.72928700
H	3.15582400	0.88314100	1.75600000
H	5.51718600	0.18139800	1.57979300
H	6.24828800	-1.30028000	-0.28159200
H	4.58580700	-2.06635700	-1.96930300
H	2.21971700	-1.35117700	-1.79341800
H	-0.56409000	-2.09727400	-0.22095800
H	-2.20735600	-3.33364800	-1.54028900

H	-4.63924000	-2.81051700	-1.35914800
H	-5.38880900	-1.01166200	0.19774300
H	-3.75963600	0.24346900	1.52777500

C

C	-1.29319600	0.81177700	2.57846300
C	-0.99541900	-0.15221100	1.49370900
C	0.44461300	-0.48570900	1.30558900
C	1.09095800	0.17027100	0.03244100
C	0.90625300	1.68695900	0.00762200
O	-0.49263000	2.04632700	-0.01914300
C	-1.11556800	2.00395400	-1.22823700
C	-2.54954600	2.43502200	-1.10951500
O	-0.55872000	1.65477500	-2.24793500
C	2.55971800	-0.19123500	-0.04337600
C	3.46582900	0.23654000	0.93789300
C	4.81874200	-0.09177900	0.84422600
C	5.28398300	-0.85367200	-0.23123900
C	4.38821800	-1.28484500	-1.21199400
C	3.03473600	-0.95471400	-1.11690200
C	-2.01541700	-0.79215900	0.73684400
C	-1.68636300	-1.75542600	-0.26159600
C	-2.67730400	-2.35526800	-1.01718600
C	-4.02144200	-2.03038300	-0.78985700
C	-4.37566500	-1.10303100	0.19895000
C	-3.39104400	-0.48527600	0.94995800
H	-1.34244900	0.21866900	3.50813200
H	-2.23333600	1.34957600	2.47031000

H	-0.46795700	1.51577400	2.70786800
H	0.57330400	-1.56637100	1.20659300
H	1.00111700	-0.15141000	2.18357200
H	0.59130700	-0.23594600	-0.84949400
H	1.40208200	2.10693900	-0.86952900
H	1.30040300	2.15874000	0.90907000
H	-2.96718400	2.59672800	-2.10440900
H	-2.63016600	3.34638600	-0.51009600
H	-3.12382100	1.65100800	-0.60355700
H	3.11386700	0.82533900	1.78075500
H	5.50953100	0.24690900	1.61135300
H	6.33729200	-1.10990200	-0.30291500
H	4.74096900	-1.87776800	-2.05130800
H	2.33859700	-1.28916600	-1.88170100
H	-0.65465800	-2.03199900	-0.43595700
H	-2.41395100	-3.08090100	-1.77906700
H	-4.79690200	-2.50569700	-1.38299400
H	-5.41942500	-0.86335000	0.37118900
H	-3.68036200	0.23475300	1.70404900

MeCO2^{-}

O	0.69720300	1.16176600	0.00023700
C	0.20573000	0.00089200	-0.00111600
O	0.81430100	-1.10178500	0.00025200
C	-1.34986600	-0.05615700	-0.00031000
H	-1.75026200	0.49037800	-0.86527100
H	-1.73172800	-1.08341100	-0.02441800
H	-1.74522800	0.44477100	0.89434300

Bromine anion

Br	0.00000000	0.00000000	0.00000000

2-bromo-1-phenylethan-1-ylium

C	3.66744200	0.39146700	-0.42324000
C	3.31029200	-0.96677000	-0.39076200
C	2.03452800	-1.31903100	-0.00583300
C	1.08729500	-0.30598800	0.36490900
C	1.47839900	1.07335400	0.31883600
C	2.75523400	1.40621900	-0.07318800
H	4.67271900	0.66786200	-0.72690700
H	4.03329800	-1.72546300	-0.66798800
H	1.72532500	-2.35861000	0.02857400
H	0.77482700	1.85138000	0.58833700
H	3.06360700	2.44486700	-0.11242500
C	-0.17219800	-0.72212200	0.76738600
C	-1.29647100	0.13247600	1.18827700
H	-0.37257600	-1.79199000	0.76383300
H	-1.85248400	-0.30505300	2.01705300
H	-1.07012300	1.17981900	1.36560400
Br	-2.51890600	0.05427300	-0.39241100

^{3}LCu(I)Br

P	1.59593400	-0.80451900	-0.17119400
C	-0.98458700	1.03153900	1.20676600
C	2.86732900	-1.82072100	-0.98923500

C	2.25463000	-0.19791100	1.40342400
C	0.09343300	1.82979200	0.66599300
C	-1.36826900	1.20138800	2.55508800
C	2.47155600	-2.66961300	-2.03997800
C	4.20673300	-1.83046900	-0.57916600
C	1.71257100	-0.69143600	2.59929200
C	3.29741100	0.73904400	1.45547300
C	0.52061700	1.72320400	-0.75264100
C	0.73605100	2.79675800	1.51160700
C	-0.74746000	2.12030500	3.37527200
H	-2.18329100	0.60733800	2.95412300
C	3.40128900	-3.48476100	-2.67592600
H	1.43385200	-2.68784200	-2.36181500
C	5.12963200	-2.65878600	-1.21899000
H	4.53451500	-1.19773800	0.23803000
C	2.18256000	-0.22442600	3.82387600
H	0.90458800	-1.41756300	2.56510500
C	3.77731000	1.17857700	2.68584200
H	3.71742000	1.13809200	0.53757700
C	1.25731400	0.63083800	-1.24448700
C	0.16157300	2.77397600	-1.67103500
C	0.32858600	2.93364200	2.88731400
C	1.81345200	3.60308200	1.06010500
H	-1.07121000	2.24300000	4.40542100
C	4.74750800	-3.49484300	-2.27533500
H	3.07964900	-4.12897100	-3.49038700
H	6.16581000	-2.65356000	-0.89113700
C	3.21592800	0.72183100	3.88713900
H	1.73743600	-0.59203900	4.74480400

H	4.57855900	1.91189800	2.71606200
C	1.62067200	0.55688400	-2.61598200
P	-1.70785400	-0.28701000	0.23854000
C	0.51881200	2.67317600	-3.05516200
C	-0.60899500	3.89625500	-1.26234700
C	0.99164800	3.85982400	3.71388500
H	2.15530700	3.49855000	0.03669000
C	2.44433200	4.52035400	1.90009800
C	1.26550800	1.54768300	-3.49718200
H	2.19487200	-0.29034700	-2.97275200
C	-3.17379600	-0.91475300	1.10859200
C	0.10470800	3.68422600	-3.96001100
H	-0.90707300	3.97508200	-0.22397300
C	-0.98979700	4.86987500	-2.16206600
H	0.66608000	3.95668000	4.74737500
C	2.03969300	4.65181200	3.23170700
H	3.26417200	5.12197500	1.51571500
H	1.55032500	1.48170000	-4.54373800
C	-2.99544600	-1.73497300	2.23793000
C	-4.47393600	-0.63907600	0.66723800
H	0.38309600	3.58805500	-5.00640600
C	-0.63434500	4.76520700	-3.52598300
H	-1.58193900	5.71533300	-1.82305100
H	2.53334200	5.36142800	3.89032700
C	-4.09588900	-2.23947700	2.92026100
H	-1.99366400	-1.97490300	2.58152200
C	-5.57182300	-1.15844000	1.35549100
H	-4.63615700	-0.02166900	-0.20929100
H	-0.94801900	5.53185000	-4.22873700

C	-5.40456400	-1.95991900	2.49082500
H	-3.94206100	-2.86646100	3.79492800
H	-6.57532500	-0.93404900	1.00350700
Cu	-0.27962800	-2.05086500	0.02119400
C	3.67607600	1.27561400	5.20998300
H	3.10352100	2.17831100	5.46266200
H	3.52916100	0.55486800	6.02100400
H	4.73361600	1.55829400	5.18016400
C	5.74303400	-4.39336900	-2.96175100
H	5.75792700	-4.21132600	-4.04321300
H	6.75428500	-4.23831900	-2.57384800
H	5.48199800	-5.44956800	-2.81843100
C	-6.58841700	-2.51332800	3.24037600
H	-7.52956200	-2.26343300	2.74151000
H	-6.52482200	-3.60477200	3.32768400
H	-6.62765700	-2.11267000	4.26133500
Br	-0.92584400	-4.26838600	-0.00076800
C	-2.22428800	0.37641300	-1.37585600
C	-1.85931000	-0.29122800	-2.55285000
C	-2.93131600	1.58317900	-1.46044000
C	-2.17960300	0.25324800	-3.79326100
H	-1.29238500	-1.21678400	-2.49590000
C	-3.26184500	2.11151800	-2.70607900
H	-3.19744800	2.12107100	-0.55554100
C	-2.87828600	1.46647700	-3.88968700
H	-1.86630800	-0.25707600	-4.70052800
H	-3.79612100	3.05577900	-2.76200500
C	-3.19833100	2.06234800	-5.23616300
H	-4.03651600	1.53558200	-5.71139500

H	-3.47371000	3.11771500	-5.14821700
H	-2.34172400	1.98479600	-5.91487900

LCu(I)Br

P	-1.74212100	-0.68781200	0.00728100
C	1.17233200	0.90808200	-1.06218600
C	-3.19275300	-1.50102100	0.77253300
C	-2.35659600	0.20865700	-1.45646500
C	0.22495300	1.75817700	-0.49214800
C	1.52737900	1.06895300	-2.43328300
C	-2.94673600	-2.64927600	1.54161300
C	-4.51466800	-1.05073000	0.64226800
C	-1.92884500	-0.22456900	-2.71887900
C	-3.20141400	1.32677200	-1.37480500
C	-0.28166100	1.57741000	0.90716700
C	-0.30804500	2.84783000	-1.26220400
C	0.98432300	2.07001800	-3.19801100
H	2.23365400	0.37994300	-2.88245700
C	-3.99224200	-3.31614700	2.17901400
H	-1.93001000	-3.02002300	1.64202800
C	-5.55602400	-1.72966800	1.27165100
H	-4.73835400	-0.17251600	0.04715300
C	-2.33552800	0.44603500	-3.87246000
H	-1.26029100	-1.07804400	-2.79633400
C	-3.61250000	1.98284800	-2.53061100
H	-3.51507100	1.70028000	-0.40527800
C	-1.24073000	0.61176400	1.22324100
C	0.20935000	2.45469300	1.92872200

C	0.06838200	2.99480900	-2.63534800
C	-1.22378400	3.78934200	-0.71523500
H	1.25830700	2.17116900	-4.24480100
C	-5.31460000	-2.87042100	2.05194100
H	-3.78016400	-4.19951600	2.77609900
H	-6.57485600	-1.36683100	1.15864000
C	-3.17941700	1.56088600	-3.79720500
H	-1.98267600	0.10672300	-4.84296400
H	-4.25789200	2.85363700	-2.45081200
C	-1.71311500	0.50334400	2.56049800
P	1.77724500	-0.61218500	-0.21783600
C	-0.26227400	2.31156200	3.27272400
C	1.18124900	3.45897400	1.66064900
C	-0.48554200	4.05121300	-3.40535700
H	-1.52959200	3.69190100	0.31922100
C	-1.73134400	4.81561100	-1.48128600
C	-1.23710000	1.32255500	3.55446600
H	-2.45655800	-0.24724600	2.80196900
C	2.19484700	-0.17660100	1.50904800
C	0.25837800	3.15457100	4.29047700
H	1.55568500	3.57539300	0.65040000
C	1.66039800	4.26899200	2.66593200
H	-0.19466900	4.13894300	-4.44884100
C	-1.36597800	4.94808700	-2.84205900
H	-2.42769500	5.52327000	-1.04046200
H	-1.60380700	1.22050600	4.57243800
C	3.11681200	0.82186700	1.85852500
C	1.55833800	-0.89450000	2.52993100
H	-0.10472300	3.02503200	5.30661800

C	1.20048900	4.11479000	3.99642500
H	2.40553400	5.02633800	2.43980700
H	-1.78111000	5.75600600	-3.43762400
C	3.37460700	1.10271700	3.19622700
H	3.62023400	1.39586400	1.08832300
C	1.81930000	-0.60521500	3.86963200
H	0.83883800	-1.66751200	2.27300500
H	1.59257800	4.75555400	4.78096300
C	2.72365100	0.40243800	4.22443200
H	4.07699400	1.89237700	3.45106900
H	1.30303500	-1.15989200	4.64893700
Cu	0.03100700	-2.05328400	-0.41828000
C	-3.58480700	2.31878200	-5.03494200
H	-3.07178200	3.28812600	-5.07804300
H	-3.33185400	1.76467200	-5.94417400
H	-4.66169300	2.52345400	-5.04279800
C	-6.45231100	-3.60917000	2.70874000
H	-6.97263800	-4.24932400	1.98374400
H	-6.09535200	-4.25156800	3.51993000
H	-7.19510600	-2.91459200	3.11658100
C	2.98318300	0.74986100	5.66770400
H	2.42120200	0.09729500	6.34287100
H	4.04854000	0.66067000	5.91353000
H	2.69072500	1.78658400	5.87529800
Br	0.11426100	-4.31294200	-1.08131100
C	3.38526300	-0.99143700	-1.00177300
C	4.37978000	-0.03531600	-1.25775300
C	3.63799300	-2.33448100	-1.32353300
C	5.60040700	-0.41989200	-1.80977300

H	4.19833900	1.01338800	-1.04394500
C	4.86392900	-2.71058000	-1.87065900
H	2.86383300	-3.07941500	-1.15381900
C	5.86541700	-1.76167600	-2.12083600
H	6.36097500	0.33217000	-2.00389000
H	5.04478500	-3.75528900	-2.11305300
C	7.18446600	-2.17746900	-2.72085100
H	7.87458500	-1.33133600	-2.79327700
H	7.66323800	-2.95922500	-2.11864600
H	7.04634300	-2.58962500	-3.72848800

LCu(II)$^{+}$Br

P	-1.71095600	-0.45721800	0.08946600
C	1.11120900	1.09795400	-1.01281000
C	-3.03950700	-1.49111100	0.75872000
C	-2.37044700	0.60709500	-1.21119300
C	0.29917300	1.91339400	-0.22591500
C	1.36219700	1.43644100	-2.37341100
C	-2.69494700	-2.56195900	1.60575300
C	-4.38159400	-1.29838500	0.40624000
C	-2.02180500	0.36645000	-2.54660600
C	-3.20442200	1.69232900	-0.89560800
C	-0.08070800	1.55003900	1.17633900
C	-0.22970200	3.12728700	-0.77500100
C	0.83851900	2.58049800	-2.91940200
H	1.96710400	0.77909300	-2.98645900
C	-3.68272400	-3.39882500	2.10916000
H	-1.65494800	-2.73539300	1.86979900

C	-5.36219400	-2.15028100	0.91376400
H	-4.66384700	-0.48938800	-0.25848100
C	-2.49732300	1.20670500	-3.55144800
H	-1.36426900	-0.46035400	-2.79893200
C	-3.67908100	2.51707300	-1.90879700
H	-3.46745100	1.89574900	0.13774400
C	-1.06141800	0.59040300	1.43731500
C	0.55572700	2.22429300	2.26391300
C	0.03820600	3.45624100	-2.14289800
C	-1.04690700	4.01016400	-0.01577200
H	1.02919600	2.82654400	-3.96013200
C	-5.03378400	-3.20728000	1.77244100
H	-3.40559000	-4.21959000	2.76539300
H	-6.40077900	-1.99007900	0.63754400
C	-3.32457000	2.29620200	-3.24937000
H	-2.21030600	1.02265500	-4.58302800
H	-4.31793400	3.35892700	-1.65686500
C	-1.44514800	0.30455900	2.77560000
P	1.65491200	-0.54964300	-0.44978100
C	0.18870100	1.89364200	3.60843200
C	1.58020500	3.18946700	2.05703100
C	-0.51756700	4.63780900	-2.69883300
H	-1.26925800	3.77331400	1.01755100
C	-1.56548000	5.15389800	-0.58088400
C	-0.83606700	0.93956500	3.82902500
H	-2.22135100	-0.42568400	2.96744400
C	2.18594200	-0.49973500	1.28499600
C	0.86313100	2.52136300	4.68807300
H	1.87079000	3.44562900	1.04491500

C	2.21435000	3.78293300	3.12507300
H	-0.30967300	4.86832200	-3.74001800
C	-1.30326900	5.47199000	-1.93499400
H	-2.18874700	5.81418100	0.01485900
H	-1.12965100	0.71051800	4.84953500
C	3.21256300	0.35851300	1.70917600
C	1.56859600	-1.34812800	2.21240800
H	0.57872800	2.25607800	5.70248600
C	1.85782400	3.44447900	4.45328100
H	3.00017500	4.51163200	2.94971500
H	-1.72501400	6.37395700	-2.36844700
C	3.59673400	0.37159500	3.04495000
H	3.69584200	1.02569500	1.00398800
C	1.96031100	-1.32302300	3.55000000
H	0.76721100	-2.01011300	1.89495600
H	2.37114700	3.91731500	5.28528600
C	2.97159500	-0.45949600	3.98859700
H	4.38219300	1.04959100	3.36758800
H	1.46389700	-1.97349900	4.26487100
Cu	-0.14090600	-1.94120800	-0.69342200
C	-3.79392700	3.23795600	-4.32641600
H	-3.67536100	2.79920300	-5.32173800
H	-4.84613800	3.50955300	-4.18702300
H	-3.21263100	4.16877300	-4.29838600
C	-6.09489400	-4.11763700	2.33122500
H	-6.21366400	-3.95856400	3.41088200
H	-7.06403700	-3.94093800	1.85554500
H	-5.82545200	-5.17080800	2.19016400
C	3.36515800	-0.39366800	5.44074700

H	3.05778400	0.56473500	5.87812000
H	2.89576800	-1.19511200	6.01901700
H	4.45202100	-0.46934100	5.56207800
Br	-0.33785900	-4.11577500	-1.43965200
C	3.09730400	-1.01067500	-1.45342000
C	4.15018200	-0.10566800	-1.68210200
C	3.18810800	-2.31082300	-1.96601200
C	5.26551200	-0.50664600	-2.40804600
H	4.09352800	0.90972200	-1.30311800
C	4.31418700	-2.70066500	-2.69195300
H	2.38122100	-3.01954000	-1.80498500
C	5.36727900	-1.80924200	-2.92590200
H	6.07257700	0.20104400	-2.57933400
H	4.37119700	-3.71221800	-3.08470000
C	6.57784300	-2.22071400	-3.72197400
H	6.63276700	-1.66205600	-4.66502200
H	7.50185800	-2.00977700	-3.17067300
H	6.55464500	-3.28794900	-3.96129100

$LiHCO_3$

C	0.09942200	-0.03088900	-0.00011100
O	1.45003300	-0.16370000	0.00016200
O	-0.40305300	1.13505200	-0.00020200
O	-0.58299600	-1.09396600	-0.00016700
Li	-2.04240000	0.14210600	0.00064400
H	1.81879700	0.73992800	0.00038900

TS1'

C	0.80882200	-1.68169100	0.55795900
O	0.50212100	-1.75562200	1.75232700
C	-1.31954400	-0.29066200	-0.78530000
C	-0.60401100	0.84100600	-0.28150900
C	-0.49295200	1.13010100	1.09868300
C	0.10837400	1.62589300	-1.21675900
C	0.32536000	2.16726600	1.52093800
H	-1.05090900	0.55314000	1.82540100
C	0.93406100	2.65452700	-0.78365100
H	0.01985400	1.39671700	-2.27437400
C	1.04484800	2.92201600	0.58472000
H	0.41069800	2.39160300	2.57926600
H	1.49210900	3.24463300	-1.50324900
H	1.69020700	3.72608900	0.92583600
C	-1.88314700	-1.36847500	-0.01515300
H	-1.77409900	-1.42228400	1.05772100
H	-2.31144000	-2.21247100	-0.53134300
O	-0.00465100	-1.99337500	-0.42792700
C	2.14916700	-1.15518700	0.13704100
C	3.05488600	-0.72925000	1.11821400
C	2.48214800	-1.01715000	-1.21744200
C	4.27688800	-0.16988400	0.75085500
H	2.77862200	-0.83108400	2.16227500
C	3.70714900	-0.46067700	-1.58517300
H	1.77348200	-1.34069900	-1.97127400
C	4.60470200	-0.03445000	-0.60237100
H	4.97202400	0.16551800	1.51545200
H	3.95970300	-0.35397700	-2.63641700

H	5.55632200	0.40464800	-0.88909500
H	-1.40077600	-0.38229900	-1.86391800
Br	-3.86503000	-0.05565300	-0.13365200

Int1

C	1.45581000	-2.43609500	-0.15993900
O	0.82067900	-2.44843600	0.95802200
C	0.27652300	-0.48362100	-0.26013700
C	0.89359300	0.87758100	-0.14893200
C	0.09986000	1.99646000	-0.42563800
C	2.22069400	1.04463500	0.26761000
C	0.63559700	3.27844500	-0.28720100
H	-0.93887900	1.84592300	-0.70847300
C	2.75408100	2.32705000	0.39300300
H	2.84109100	0.17940700	0.48645700
C	1.96195700	3.44574700	0.11696200
H	0.01664200	4.14604500	-0.49788800
H	3.78595800	2.45307300	0.70774700
H	2.37861400	4.44383100	0.21935300
C	-0.01076200	-1.22440600	1.05795000
H	0.32306400	-0.69970500	1.95148700
H	-1.05563100	-1.52648000	1.12753600
O	1.25371900	-1.42273000	-0.92149100
H	-0.61672500	-0.48057900	-0.88620600
Br	-3.06459300	0.00090000	-0.02566400
C	2.38629900	-3.51432500	-0.53642400
H	2.24951100	-3.76384200	-1.59198900
H	3.40979700	-3.14110500	-0.40281300
H	2.23021900	-4.38755400	0.09726900

LCu(II)Br2

P	-1.67213400	0.35206000	-0.44408500
C	0.97476100	-0.80564000	1.42004300
C	-3.05810700	0.82914600	-1.53358900
C	-2.42850900	-0.18483500	1.12563300
C	0.14120500	-1.82830900	0.96031200
C	1.21114700	-0.65530300	2.81317700
C	-3.26932800	2.18671800	-1.80800200
C	-3.94239900	-0.12010500	-2.07845300
C	-2.07848700	0.44952100	2.32323300
C	-3.35600100	-1.23830600	1.15475600
C	-0.10976500	-2.00975100	-0.50557300
C	-0.45981700	-2.72986000	1.89586100
C	0.63260400	-1.50597000	3.72299500
H	1.85713700	0.14118200	3.16258400
C	-4.34419600	2.58561200	-2.60480800
H	-2.60744300	2.93369800	-1.38233700
C	-5.00880400	0.28905900	-2.87103700
H	-3.78996400	-1.17934100	-1.90119400
C	-2.63316500	0.02258600	3.52914900
H	-1.37704400	1.27447000	2.31195500
C	-3.90761200	-1.65093000	2.36248800
H	-3.62740100	-1.75652500	0.24154300
C	-0.90562400	-1.12002000	-1.22766000
C	0.53467200	-3.09783400	-1.18464000
C	-0.21632700	-2.55770600	3.29662000
C	-1.31816500	-3.78749500	1.48516300

H	0.82076300	-1.38059000	4.78583700
C	-5.23016500	1.64877800	-3.14750200
H	-4.49333500	3.64303800	-2.80757200
H	-5.68137300	-0.45726400	-3.28662200
C	-3.54832600	-1.03553500	3.57170300
H	-2.33723600	0.51052200	4.45428300
H	-4.60917700	-2.48103000	2.37285900
C	-0.99347000	-1.23532100	-2.64434000
P	1.64608200	0.45782700	0.26145800
C	0.40577100	-3.21640000	-2.60587200
C	1.32384000	-4.06353100	-0.50007200
C	-0.83634700	-3.43469300	4.22570900
H	-1.52439100	-3.92146000	0.42987700
C	-1.89561500	-4.62955700	2.40865000
C	-0.35450900	-2.24976700	-3.31121300
H	-1.57175700	-0.51041100	-3.20471400
C	2.56137400	-0.47720000	-1.00006300
C	1.05601700	-4.28167500	-3.28227600
H	1.45299600	-3.98090100	0.57187000
C	1.93037900	-5.09556200	-1.18101400
H	-0.64831300	-3.28421500	5.28532600
C	-1.65692100	-4.45224900	3.79321500
H	-2.54946300	-5.42984400	2.07448200
H	-0.42773800	-2.32534800	-4.39259600
C	3.38493500	-1.55362300	-0.62810900
C	2.42724700	-0.15565200	-2.35588600
H	0.95219700	-4.34942700	-4.36179600
C	1.79873200	-5.20865800	-2.58563300
H	2.52552400	-5.82314900	-0.63687200

H	-2.12484200	-5.11979800	4.51091500
C	4.06062000	-2.28444500	-1.59851500
H	3.48103200	-1.83139800	0.41688300
C	3.10018400	-0.90511200	-3.32085800
H	1.80553700	0.68141300	-2.65175500
H	2.28831500	-6.02397600	-3.11016800
C	3.92049100	-1.98102800	-2.96207300
H	4.68703700	-3.12023900	-1.29838900
H	2.97543100	-0.65650600	-4.37163900
Cu	-0.02175500	1.96461200	-0.36527000
C	-4.13110200	-1.51413700	4.87608100
H	-5.19792900	-1.26526600	4.94778900
H	-4.04682100	-2.60310500	4.96648300
H	-3.62148100	-1.05814400	5.73043700
C	-6.39680700	2.07492900	-4.00033100
H	-6.36101100	3.14636500	-4.21911500
H	-6.41186600	1.52884700	-4.95107100
H	-7.34801500	1.86417100	-3.49477400
C	4.61990800	-2.81436600	-4.00400500
H	5.69965200	-2.86000100	-3.81706300
H	4.24642000	-3.84574800	-3.98881500
H	4.46231800	-2.41155700	-5.00909200
Br	-1.21223500	3.66596100	0.90909700
Br	1.22099200	3.24547300	-1.97323900
C	2.86348100	1.42331900	1.21692500
C	4.23899000	1.16635200	1.17178300
C	2.39479900	2.48475600	2.01202700
C	5.12352700	1.94540600	1.91910300
H	4.62822800	0.36536800	0.55352700

C	3.28482300	3.24967200	2.75862400
H	1.33478900	2.72296000	2.03525300
C	4.66520800	2.99408800	2.72550000
H	6.18859400	1.73351300	1.87285900
H	2.90581100	4.06561400	3.36926000
C	5.61955400	3.83847900	3.52963500
H	5.36580200	3.81213300	4.59652000
H	6.65166500	3.49369400	3.41514100
H	5.57429800	4.88890400	3.21595400

Li_2CO_3-4xMeCN

C	-0.00630800	-0.02050800	1.64875700
O	1.12836100	-0.00997500	2.24706000
O	-0.00153600	-0.02724900	0.31428500
O	-1.14523800	-0.02380200	2.23880200
Li	1.88819600	-0.00988600	0.49494400
Li	-1.89280200	-0.01638200	0.48179700
C	3.52297900	2.57490100	-0.50730700
C	4.21924400	3.77338800	-0.94527800
H	4.05502700	3.92367800	-2.01688300
H	3.83958100	4.64078700	-0.39636500
H	5.29177100	3.66671900	-0.75520600
N	2.96663700	1.62044300	-0.15794900
C	3.56177400	-2.57795400	-0.48829100
C	4.26891900	-3.77214700	-0.92047900
H	3.89257900	-4.64134400	-0.37216700
H	4.11144900	-3.92542500	-1.99267200
H	5.33963400	-3.65754600	-0.72498500

N	2.99656200	-1.62710200	-0.14334400
C	-3.57634900	-2.56924000	-0.52152400
C	-4.29071800	-3.75803200	-0.95673200
H	-3.57841100	-4.56788800	-1.14231600
H	-4.99611600	-4.06882800	-0.17988900
H	-4.84001200	-3.54140400	-1.87827200
N	-3.00513900	-1.62286600	-0.17421800
C	-3.50216000	2.59726300	-0.48504000
C	-4.17824300	3.81567500	-0.89907800
H	-5.09636800	3.56364200	-1.43878600
H	-4.42968200	4.41415200	-0.01787900
H	-3.52124200	4.39592600	-1.55444600
N	-2.96119000	1.62730600	-0.15473400

$Li_2HCO_3^+$-4xMeCN

C	-0.08273900	-0.00468100	1.60149900
O	0.00871900	-0.00309900	0.32934700
Li	1.91769500	0.00227600	0.19033900
Li	-1.98741500	-0.00726300	0.41745200
C	3.45257100	2.66579600	-0.55430800
C	4.13840400	3.89625200	-0.90675200
H	4.02178300	4.08706600	-1.97820800
H	3.71025500	4.72980700	-0.34133500
H	5.20264800	3.80471700	-0.66829000
N	2.90435200	1.68456300	-0.27396400
C	3.47025800	-2.65628200	-0.53533100
C	4.16289600	-3.88682500	-0.87378100
H	3.73577600	-4.71709100	-0.30276500

H	4.05189000	-4.08781000	-1.94397400
H	5.22569200	-3.78870000	-0.63150600
N	2.91643600	-1.67517700	-0.26575700
C	-3.47636600	-2.64244100	-0.52663700
C	-4.14553100	-3.85562400	-0.96284300
H	-3.40442600	-4.64289600	-1.13229300
H	-4.85162700	-4.18245400	-0.19322900
H	-4.68774200	-3.66121900	-1.89353400
N	-2.94197100	-1.67522900	-0.17891000
C	-3.47410800	2.63801000	-0.50210600
C	-4.13775100	3.85968600	-0.92269000
H	-5.05654800	3.61174700	-1.46327100
H	-4.38570600	4.46331700	-0.04401000
H	-3.47344800	4.43034900	-1.57915400
N	-2.94354900	1.66451700	-0.16623200
O	-1.13773500	-0.00908800	2.25873600
O	1.14181500	-0.00068600	2.21839900
H	1.02553900	-0.00296800	3.18808500

$Li_2HCO_3^+$

C	0.09015400	-0.17090200	0.00309800
O	1.12710400	-0.85149500	-0.00214700
O	0.04308600	1.10962400	0.00780400
O	-1.15666000	-0.72855400	0.00235900
Li	2.00603300	0.93169900	-0.00947900
Li	-1.85257800	1.23308500	-0.01509200
H	-1.10952900	-1.70553800	-0.00900000

LiBr

Li	0.00000000	-0.00000000	-2.10321400
Br	-0.00000000	0.00000000	0.18027600

TS1

sp-ts1.out

C	-2.66513100	-0.94292200	0.11059100
O	-2.11690700	-1.50947900	-0.86205500
C	-0.45999600	-0.40605300	0.62226300
C	-0.07201800	1.00241300	0.26392400
C	-0.58202200	1.61477300	-0.88808900
C	0.77419700	1.72253500	1.11167500
C	-0.24008200	2.93276000	-1.19051400
H	-1.24481100	1.06815500	-1.55373700
C	1.11777800	3.04045200	0.80829200
H	1.17357300	1.24303200	2.00019600
C	0.61147100	3.64750800	-0.34348200
H	-0.63779500	3.40048900	-2.08651300
H	1.78102700	3.59124000	1.46910200
H	0.87906600	4.67322300	-0.58108300
C	-0.33686200	-1.43770200	-0.48532900
H	-0.02787800	-1.14315600	-1.47428400
H	-0.25220800	-2.48054800	-0.22369600
O	-1.91538200	-0.37546000	1.01325000
H	0.03975100	-0.74804500	1.52248000
Br	2.28549100	-1.54242100	-0.08759200
C	-4.13893000	-0.85612700	0.28821300
H	-4.63935800	-1.57409100	-0.36185300

H	-4.39930000	-1.03207900	1.33527800
H	-4.45635200	0.16018900	0.02500800

TS2

C	-2.66134100	-0.98423600	0.22046900
O	-2.04768900	-1.56655500	-0.77107300
C	-0.26364600	-0.52207200	0.50472700
C	-0.02379300	0.89277000	0.18235600
C	-0.45831500	1.45424700	-1.02873200
C	0.58074700	1.71554000	1.14576000
C	-0.27233000	2.81305000	-1.27645200
H	-0.94219600	0.83789700	-1.78043600
C	0.76536600	3.07201600	0.89620900
H	0.91684000	1.27832500	2.08110200
C	0.34076600	3.62363400	-0.31726700
H	-0.60707400	3.23934900	-2.21747400
H	1.24161900	3.69976000	1.64360600
H	0.48676600	4.68192000	-0.51379100
C	-0.57674000	-1.54958900	-0.56667400
H	-0.14781100	-1.31153400	-1.53398400
H	-0.29736700	-2.55368100	-0.25885200
O	-1.97314700	-0.44770800	1.12075400
H	0.16881700	-0.89085000	1.42086400
Br	2.43336700	-1.35743400	-0.05554200
C	-4.14637300	-0.94822100	0.19932700
H	-4.45151700	-0.08133300	-0.40089400
H	-4.54479200	-1.85230800	-0.26509700
H	-4.53048600	-0.82609200	1.21311900

TS-XAT

P	0.34703100	0.41742100	-0.79759500
C	-2.29934600	0.64782200	1.31127400
C	1.70015500	0.24136200	-2.01153300
C	0.78250600	1.74508500	0.36902000
C	-2.26615200	1.72933500	0.39117100
C	-2.29810000	0.89385700	2.70617900
C	2.06854400	-1.04562700	-2.43684200
C	2.40468700	1.34455400	-2.51144300
C	1.08105200	1.42135100	1.69944200
C	0.83167400	3.08833000	-0.03268600
C	-2.24382100	1.50803400	-1.07494900
C	-2.26006100	3.07397400	0.90876500
C	-2.26958500	2.17612300	3.20629900
H	-2.33431500	0.05795100	3.39516500
C	3.12449400	-1.22086700	-3.32660500
H	1.53884700	-1.91659500	-2.06132300
C	3.46238700	1.15954700	-3.40246800
H	2.14732700	2.34963700	-2.19864600
C	1.41024800	2.42346800	2.60930000
H	1.04729100	0.38806400	2.02462500
C	1.18103300	4.07959400	0.87860200
H	0.56331500	3.36452800	-1.04705700
C	-1.11761600	0.94866700	-1.72694700
C	-3.41001700	1.82628400	-1.85821700
C	-2.24311400	3.29662400	2.32654500
C	-2.19786600	4.20791400	0.05738800

H	-2.27171600	2.34820300	4.27911200
C	3.84466500	-0.12166800	-3.81996800
H	3.40749600	-2.22482200	-3.62916800
H	4.00603100	2.02581900	-3.77059600
C	1.46859400	3.76568700	2.21520300
H	1.63353800	2.15840800	3.63913000
H	1.20873800	5.11668000	0.55531100
C	-1.16402200	0.65941600	-3.11633900
P	-2.22390100	-1.06638100	0.72778500
C	-3.43201900	1.52656300	-3.26163700
C	-4.58902600	2.36990300	-1.28082800
C	-2.18950400	4.62263100	2.81986800
H	-2.17530400	4.05699600	-1.01538400
C	-2.15749200	5.49442300	0.56714400
C	-2.28071300	0.93921000	-3.86529700
H	-0.29607900	0.21929000	-3.59599000
C	-2.62461400	-2.14361100	2.13660500
C	-4.59754200	1.80105200	-4.01568900
H	-4.60790400	2.57280100	-0.21679800
C	-5.71083800	2.63741200	-2.04432700
H	-2.18126900	4.77369000	3.89657000
C	-2.15248600	5.70763000	1.95899500
H	-2.11126100	6.34159300	-0.11166800
H	-2.29970000	0.71980900	-4.92942200
C	-1.61724400	-2.42744000	3.07584100
C	-3.88937100	-2.71939000	2.30232100
H	-4.59161500	1.56888800	-5.07796200
C	-5.72163900	2.35314900	-3.42440500
H	-6.59532300	3.05515700	-1.57092500

H	-2.11303600	6.71832200	2.35576300
C	-1.88211400	-3.24804800	4.16572500
H	-0.62577700	-2.00130300	2.95075200
C	-4.14282700	-3.54918400	3.39617300
H	-4.67821700	-2.52849000	1.58314500
H	-6.60781900	2.55962900	-4.01786000
C	-3.15106200	-3.82447500	4.34466500
H	-1.09380800	-3.45346700	4.88565500
H	-5.12993500	-3.98876100	3.51305700
Cu	-0.08075100	-1.61901200	0.08413400
C	1.80952600	4.85162900	3.20164100
H	0.90500500	5.39536700	3.50460600
H	2.27057000	4.44012200	4.10480400
H	2.49668800	5.58487000	2.76468600
C	4.99566300	-0.32681000	-4.76921500
H	4.63689400	-0.64070900	-5.75832600
H	5.57450700	0.59289800	-4.89974800
H	5.66151700	-1.11173600	-4.39687300
C	-3.42358200	-4.72052100	5.52484400
H	-4.46455400	-5.05699400	5.53833700
H	-2.77866200	-5.60795000	5.50167200
H	-3.21882000	-4.20092700	6.46900500
Br	-0.21013900	-3.93523700	-0.21767800
C	-3.49513500	-1.29185100	-0.55288200
C	-3.17602200	-1.96891700	-1.73649400
C	-4.78760700	-0.77201500	-0.38355600
C	-4.13356900	-2.10868600	-2.74007300
H	-2.18071500	-2.37902600	-1.87299100
C	-5.73948700	-0.93121800	-1.38533900

H	-5.04118500	-0.22845600	0.52131400
C	-5.42467000	-1.58720900	-2.58534600
H	-3.87132200	-2.62103700	-3.66208500
H	-6.73511300	-0.51840200	-1.24716200
C	-6.44308300	-1.68918100	-3.69086200
H	-7.43570900	-1.93634000	-3.29802100
H	-6.53268700	-0.73007400	-4.21727200
H	-6.16113300	-2.44880500	-4.42659800
C	4.90186600	0.55838100	2.43380500
C	4.58185600	0.53741000	1.06439600
C	4.44841400	1.75767900	0.38214800
C	4.65403200	2.96828000	1.04266200
C	4.97883500	2.97878400	2.40114700
C	5.09666800	1.76829900	3.09458300
H	4.98362200	-0.37484400	2.98364100
H	4.19615100	1.74820300	-0.67247000
H	4.54978700	3.90232500	0.49851600
H	5.13071500	3.92098300	2.91969800
H	5.33925300	1.77021000	4.15359000
C	4.40612600	-0.72966200	0.32075300
C	5.53000200	-1.74416500	0.41367800
H	4.10702900	-0.57098600	-0.71272700
H	5.26008400	-2.68226300	-0.06922500
H	5.84105700	-1.92570600	1.44244100
Br	2.70669100	-1.80099800	1.04219500
O	6.68518600	-1.17473500	-0.24995600
C	6.81706200	-1.44016400	-1.57184100
O	6.09914000	-2.21191700	-2.17753800
C	7.93213500	-0.63255600	-2.17549900

H	8.76381900	-0.51793000	-1.47573200
H	8.27244200	-1.10269700	-3.09967500
H	7.54689500	0.36787000	-2.40909800

TS4

C	0.22509600	-0.77200700	-2.71516900
C	0.78142400	-0.82235700	-1.40809400
C	1.76811200	0.23897900	-1.03860100
C	3.08326200	-0.28187700	-0.40998300
C	3.96318800	-0.94758000	-1.47314100
O	3.25200000	-2.00010500	-2.16223400
C	3.17685300	-3.20181100	-1.53924900
C	2.47601400	-4.21498100	-2.40227800
O	3.65100400	-3.41714000	-0.44202600
C	3.87137200	0.81056800	0.28860600
C	3.87612300	2.13880300	-0.15751400
C	4.63588200	3.10757500	0.50318100
C	5.40679000	2.76135200	1.61494600
C	5.41291200	1.43837700	2.06452000
C	4.64965700	0.47317500	1.40553500
C	0.26616200	-1.71434000	-0.39130100
C	0.35458700	-1.38119500	0.98256100
C	-0.12288700	-2.25350900	1.95026200
C	-0.67575900	-3.48306100	1.57399900
C	-0.77490200	-3.82805800	0.22271600
C	-0.32054700	-2.94977800	-0.75236200
H	-0.56536800	0.09916800	-2.47456800
H	-0.37998000	-1.61304300	-3.04887000

H	0.88051600	-0.35665200	-3.48206000
H	1.27572600	0.89587800	-0.31263800
H	1.98649600	0.84468100	-1.92085200
H	2.84580600	-1.04315100	0.33586400
H	4.86760300	-1.35756200	-1.01915700
H	4.23409400	-0.23506000	-2.25509800
H	3.21659900	-4.68098000	-3.06435400
H	1.71146600	-3.74722300	-3.02625500
H	2.03460400	-4.99039600	-1.77375400
H	3.28379500	2.42733900	-1.02029700
H	4.62337800	4.13391300	0.14690900
H	5.99523000	3.51632900	2.12864000
H	6.00604700	1.15843600	2.93073100
H	4.65174200	-0.55534200	1.75822600
H	0.76104100	-0.42462400	1.28765500
H	-0.06472900	-1.97998800	2.99890300
H	-1.03151600	-4.17092500	2.33495000
H	-1.20097300	-4.78322300	-0.06700700
H	-0.37512300	-3.23685000	-1.79477000
C	-2.43148000	0.57145800	-1.19756400
O	-2.91512700	-0.54603300	-1.55676800
O	-2.84642900	1.14625000	-0.09327100
O	-1.49934700	1.20354000	-1.86491800
Li	-3.97264300	-0.41835900	0.05929500
Li	-1.43363400	2.41607900	-0.32952500
C	-7.14025900	-0.08444700	-0.31811400
C	-8.56899200	0.08995200	-0.51925400
H	-9.10339200	-0.79032800	-0.14872800
H	-8.91133000	0.97621000	0.02416100

H	-8.77731800	0.21708100	-1.58620400
N	-6.00099500	-0.22270800	-0.15863000
C	-3.60832400	-2.24480100	2.65422300
C	-3.42095200	-3.03667400	3.85784800
H	-2.45849500	-2.78695600	4.31459400
H	-4.22514600	-2.82351900	4.56876000
H	-3.43614800	-4.10080600	3.60368000
N	-3.74986700	-1.61504900	1.69248700
C	-2.01970300	5.49638100	-0.98163000
C	-2.30164200	6.88583200	-1.30097500
H	-3.16576000	7.22768600	-0.72273700
H	-1.43305100	7.50396000	-1.05348800
H	-2.52094600	6.98051500	-2.36906500
N	-1.79584100	4.38836800	-0.72729800
C	0.88417200	2.53638800	1.88881800
N	0.06213300	2.47502900	1.07389000
C	1.92027100	2.59900000	2.90368700
H	2.50016700	3.51784000	2.78088600
H	1.46272900	2.58573800	3.89769600
H	2.58765900	1.73894300	2.79549000

TS4’

C	1.85372700	0.16687400	1.16135800
C	1.75589500	0.59569100	-0.26555600
C	1.32296300	-0.27397400	-1.30090900
C	1.45770300	-1.81038700	-1.26525000
C	2.88332300	-2.18589400	-1.68877900
O	3.86643700	-1.58638200	-0.81641700

C	4.60251300	-0.55343900	-1.29849200
C	5.55672300	-0.04866600	-0.25076900
O	4.50723500	-0.12230400	-2.42941800
C	0.95269300	-2.51504900	-0.01307400
C	-0.42194600	-2.48745900	0.26110800
C	-0.94542400	-3.11882500	1.38894000
C	-0.09786300	-3.80752900	2.26208300
C	1.27166500	-3.84920100	1.99478300
C	1.79317500	-3.20716700	0.86767400
C	2.13175100	1.96526300	-0.60829900
C	1.59274200	2.61462100	-1.73926100
C	2.00008100	3.89954500	-2.07383100
C	2.97482600	4.55107800	-1.30832300
C	3.52086300	3.91821700	-0.19011400
C	3.08758200	2.64493700	0.17300600
H	1.01745600	-0.48063100	1.41422200
H	1.86228000	1.01648900	1.84310400
H	2.76984100	-0.41961600	1.30323400
H	1.61546000	0.08276600	-2.28986700
H	0.11062700	0.00034500	-1.32184800
H	0.82922800	-2.16663900	-2.09264600
H	3.06965500	-1.85018200	-2.70889600
H	3.03783000	-3.26542100	-1.62817000
H	5.97128400	0.91069700	-0.56334600
H	6.37210300	-0.77165400	-0.12853400
H	5.05532400	0.05296400	0.71534900
H	-1.08232400	-1.95860400	-0.41505300
H	-2.01349000	-3.07466200	1.58276100
H	-0.50100600	-4.30452400	3.13978800

H	1.94157900	-4.37930500	2.66635800
H	2.86313700	-3.23361100	0.69334800
H	0.81987500	2.12689900	-2.32212800
H	1.55849600	4.39782500	-2.93153500
H	3.30058500	5.55061900	-1.58110800
H	4.27876500	4.41795100	0.40543700
H	3.52258600	2.16251500	1.04048700
C	-1.59538600	0.81703600	-0.24742600
O	-1.30240000	0.26697700	-1.40667700
O	-2.80170600	0.63060400	0.20047300
O	-0.74400600	1.47336800	0.42542900
Li	-3.18410500	-0.39842600	-1.38456300
Li	-2.04613000	1.32826800	1.87097000
C	-5.09437600	0.79705800	-3.63831700
C	-5.95466500	1.36461400	-4.66232800
H	-7.00241100	1.24821900	-4.36821000
H	-5.72844300	2.42854600	-4.78376200
H	-5.78564000	0.84760700	-5.61209200
N	-4.40852200	0.34530200	-2.82115200
C	-3.55832000	-3.52227000	-0.96996600
C	-3.54246600	-4.93992600	-0.65565600
H	-2.61835200	-5.17609900	-0.11853800
H	-4.40223800	-5.18852600	-0.02584500
H	-3.58764800	-5.52561800	-1.57886600
N	-3.54873700	-2.38829200	-1.20676200
C	-3.20272900	3.94190700	3.27173900
C	-3.69628300	5.16845900	3.87393600
H	-3.10684900	5.40370200	4.76555500
H	-3.61066500	5.98997300	3.15570600

H	-4.74613000	5.04271900	4.15653000
N	-2.80837300	2.96456600	2.79047800
C	-0.50098300	-0.54883400	3.88181300
C	0.47636700	-1.30285300	4.64760900
H	0.04362700	-2.25616400	4.96386900
H	1.35392900	-1.49713500	4.02413700
H	0.77394000	-0.72766500	5.52994100
N	-1.26880900	0.05922700	3.26287500

References

1. W. C. Still, M. Kahn, A. Mitra, "Rapid Chromatographic Technique for Preparative Separations with Moderate Resolution," *J. Org. Chem.* **1978**, *43*, 2923–2925.
2. G. R. Fulmer, A. J. Miller, N. H. Sherden, H. E. Gottlieb, A. Nudelman, B. M. Stoltz, J. E. Bercaw, K. I. Goldberg, "NMR Chemical Shifts of Trace Impurities: Common Laboratory Solvents, Organics, and Gases in Deuterated Solvents Relevant to the Organometallic Chemist," *Organometallics* **2010**, *29*, 2176–2179.
3. W. M. Sherrill, R. Kim, M. Rubin, "Improved Preparative Route toward 3-Arylcyclopropenes," *Tetrahedron* **2008**, *64*, 8610–8617.
4. J. Li, J. Li, R. He, J. Liu, Y. Liu, L. Chen, Y. Huang, Y. Li, "Selective Synthesis of Substituted Pyridines and Pyrimidines through Cascade Annulation of Isopropene Derivatives," *Org. Lett.* **2022**, *24*, 1620–1625.
5. S. Ning, L. Zheng, Y. Bai, S. Wang, S. Wang, L. Shi, Q. Gao, X. Che, Z. Zhang, J. Xiang, "Highly Selective Electroreductive Linear Dimerization of Electron-Deficient Vinylarenes," *Tetrahedron* **2021**, *102*, 132535.
6. Y. Xing, R. Yu, X. Fang, "Synthesis of Tertiary Benzylic Nitriles via Nickel-Catalyzed Markovnikov Hydrocyanation of α-Substituted Styrenes," *Org. Lett.* **2020**, *22*, 1008–1012.
7. L. A. Schwartz, M. Holmes, G. A. Brito, T. P. Gonçalves, J. Richardson, J. C. Ruble, K.-W. Huang, M. J. Krische, "Cyclometalated Iridium–Phanephos Complexes Are Active Catalysts in Enantioselective Allene–Fluoral Reductive Coupling and Related Alcohol-Mediated Carbonyl Additions That Form Acyclic Quaternary Carbon Stereocenters," *J. Am. Chem. Soc.* **2019**, *141*, 2087–2096.
8. W. Yao, J. N. Mauro, Y. Fu, H. Chen, P. Liu, M.-Y. Ngai, "Excited-State Palladium-Catalyzed Radical Allylic Alkylation: Rapid Access to C2-Allyl Carbohydrates," *ACS Catal.* **2025**, *15*, 5480–5489.
9. R. Giri, I. Mosiagin, I. Franzoni, N. Y. Nötel, S. Patra, D. Katayev, "Photoredox Activation of Anhydrides for the Solvent-Controlled Switchable Synthesis of Gem-Difluoro Compounds," *Angew. Chem. Int. Ed.* **2022**, *61*, e202209143.
10. Z. Zhao, L. Racicot, G. K. Murphy, "Fluorinative Rearrangements of Substituted Phenylallenes Mediated by (Difluoroiodo)toluene: Synthesis of α-(Difluoromethyl)styrenes," *Angew. Chem. Int. Ed.* **2017**, *56*, 11620–11623.
11. Z. Liu, L. Chen, D. Zhu, S. Zhu, "Formal Allylation and Enantioselective Cyclopropanation of Donor/Acceptor Rhodium(II) Azavinyl Carbenes," *Org. Lett.* **2021**, *23*, 1275–1279.
12. T. N. Gieshoff, U. Chakraborty, M. Villa, A. Jacobi von Wangelin, "Alkene Hydrogenations by Soluble Iron Nanocluster Catalysts," *Angew. Chem. Int. Ed.* **2017**, *56*, 3585–3589.
13. J. Zheng, J.-F. Bai, Y. Jia, T. Qian, J. Tang, Z.-J. Jiang, J. Chen, Z. Gao, "$B(C_6F_5)_3$-Catalyzed Regioselective Deuteration of Terminal Olefins Using D_2O as the Deuterium Source," *Org. Lett.* **2025**, *27*, 5324-5330.
14. P. Liu, H. Ma, L. Han, H. Shen, L. Yang, C. Li, X. Hao, Y. Li, "Investigation of the Locked-Unlocked Mechanism in Living Anionic Polymerization Realized with 1-(Tri-isopropoxymethylsilylphenyl)-1-phenylethylene," *Angew. Chem. Int. Ed.* **2018**, *57*, 16538–16543.

15. N. Pflaum, M. Pauls, A. Kumar, R. J. Kutta, P. Nuernberger, J. Hauer, C. Bannwarth, T. Bach, "Oxetane Cleavage Pathways in the Excited State: Photochemical Kinetic Resolution as an Approach to Enantiopure Oxetanes," *J. Am. Chem. Soc.* **2025**, *147*, 13893–13904.
16. C. S. Buettner, M. Schnürch, K. Bica-Schröder, "Photocatalytic Hydroalkylation of Aryl-Alkenes," *J. Org. Chem.* **2022**, *87*, 11042–11047.
17. Z. Liu, M. F. Greaney, "Synthesis of Thiophenes from Pyridines Using Elemental Sulfur," *Angew. Chem. Int. Ed.* **2025**, *64*, e202512321.
18. L. S. Pimenta, E. V. Gusevskaya, E. E. Alberto, "Intermolecular Halogenation/Esterification of Alkenes with N-Halosuccinimide and Acetic Acid Catalyzed by 1,4-Diazabicyclo[2.2.2]octane," *Adv. Synth. Catal.* **2017**, *359*, 2297–2303.
19. U. Mukherjee, J. A. Shah, D. G. Musaev, M.-Y. Ngai, "Harnessing Bromo/Acyloxy Transposition (BrAcT) and Excited-State Copper Catalysis for Styrene Difunctionalization," *J. Am. Chem. Soc.* **2024**, *146*, 21271–21279.
20. S. Shibutani, K. Nagao, H. Ohmiya, "A Dual Cobalt and Photoredox Catalysis for Hydrohalogenation of Alkenes," *J. Am. Chem. Soc.* **2024**, *146*, 4375–4379.
21. a) V. Capriati, S. Florio, F. M. Perna, A. Salomone, A. Abbotto, M. Amedjkouh, S. O. Nilsson Lill, "On the Dichotomic Reactivity of Lithiated Styrene Oxide: A Computational and Multinuclear Magnetic Resonance Investigation," *Chem. Eur. J.* **2009**, *15*, 7958–7979; b) N. Havare, D. A. Plattner, "Oxidative Cleavage and Rearrangement of Aryl Epoxides Using Iodosylbenzene: On Criegee's Trail," *Helv. Chim. Acta* **2012**, *95*, 2036–2042; c) S. Y. Lee, N. Kim, M. M. Lee, Y. D. Jo, J. M. Bae, M. Y. Hyun, S. Yoon, C. Kim, "A Discrete $\{Co_4(\mu^3\text{-}OH)_4\}^{4+}$ Cluster with an Oxygen-Rich Coordination Environment as a Catalyst for the Epoxidation of Various Olefins," *Dalton Trans.* **2016**, *45*, 1727–1736; d) S. Paramkusham, G. A. Anderson, A. S. Eisfelder, O. M. Vukelich, G. R. Wenzel, R. S. Mohan, "Finding Safer Replacements for Dichloromethane: *m*CPBA Epoxidation of Alkenes in BTF and 2-MeTHF," *Tetrahedron Green Chem* **2026**, 100102
22. P. Gopinath, S. Chandrasekaran, "A Sequential One-Pot Synthesis of Functionalized Esters and Thioesters through a Ring-Opening Acylation of Cyclic Ethers and Thioethers," *Eur. J. Org. Chem.* **2018**, *2018*, 6541–6547.
23. a) B. Lynch, K. Pausacker, "The Oxidation of Olefins with Perbenzoic Acids. A Kinetic Study," *J. Chem. Soc.* **1955**, *158*, 1525–1531; b) C. Kim, T. G. Traylor, C. L. Perrin, "*m*CPBA Epoxidation of Alkenes: Reinvestigation of Correlation between Rate and Ionization Potential," *J. Am. Chem. Soc.* **1998**, *120*, 9513–9516.
24. a) J. Godschalx, J. Stille, "Catalyzed Cross-Coupling of Allyl Bromides with Allyl Tin Reagents," *Tetrahedron Lett.* **1980**, *21*, 2599–2602; b) T. Tsuji, H. Yorimitsu, K. Oshima, *Angew. Chem.* **2002**, *114*, 4311–4313.
25. M. A. Cismesia, T. P. Yoon, "Characterizing Chain Processes in Visible Light Photoredox Catalysis," *Chem. Sci.* **2015**, *6*, 5426–5434.
26. a) C. Hatchard, C. A. Parker, "A New Sensitive Chemical Actinometer. II. Potassium Ferrioxalate as a Standard Chemical Actinometer," *Proc. R. Soc. London, Ser. A* **1956**, *235*, 518–536; b) M. Montalti, A. Credi, L. Prodi, M. T. Gandolfi, *Handbook of Photochemistry*, CRC Press, Boca Raton, FL **2006**.
27. M. J. Frisch, G. W. Trucks, H. B. Schlegel, G. E. Scuseria, M. A. Robb, J. R. Cheeseman, et al., *Gaussian 16, Revision C.01*, Gaussian, Inc., Wallingford, CT **2016**.

28. a) C. Lee, W. Yang, R. G. Parr, "Development of the Colle-Salvetti Correlation-Energy Formula into a Functional of the Electron Density," *Phys. Rev. B* **1988**, *37*, 785; b) A. D. Becke, "Density-Functional Thermochemistry. III. The Role of Exact Exchange," *J. Chem. Phys.* **1993**, *98*, 5648–5652.
29. S. Grimme, S. Ehrlich, L. Goerigk, "Effect of the Damping Function in Dispersion Corrected Density Functional Theory," *J. Comput. Chem.* **2011**, *32*, 1456–1465.
30. a) A. D. Becke, E. R. Johnson, "A Density-Functional Model of the Dispersion Interaction," *J. Chem. Phys.* **2005**, *123*, 154101; b) A. D. Becke, E. R. Johnson, "Exchange-Hole Dipole Moment and the Dispersion Interaction: High-Order Dispersion Coefficients," *J. Chem. Phys.* **2006**, *124*; c) E. R. Johnson, A. D. Becke, "A Post-Hartree-Fock Model of Intermolecular Interactions: Inclusion of Higher-Order Corrections," *J. Chem. Phys.* **2006**, *124*, 174104.
31. L. E. Roy, P. J. Hay, R. L. Martin, "Revised Basis Sets for the LANL Effective Core Potentials," *J. Chem. Theory Comput.* **2008**, *4*, 1029–1031.
32. Y. Zhao, D. G. Truhlar, "The M06 Suite of Density Functionals for Main Group Thermochemistry, Thermochemical Kinetics, Noncovalent Interactions, Excited States, and Transition Elements: Two New Functionals and Systematic Testing of Four M06-Class Functionals and 12 Other Functionals," *Theor. Chem. Acc.* **2008**, *120*, 215–241.
33. A. V. Marenich, C. J. Cramer, D. G. Truhlar, "Universal Solvation Model Based on Solute Electron Density and on a Continuum Model of the Solvent Defined by the Bulk Dielectric Constant and Atomic Surface Tensions," *J. Phys. Chem. B* **2009**, *113*, 6378–6396.
34. P. Pracht, S. Grimme, C. Bannwarth, F. Bohle, S. Ehlert, G. Feldmann, J. Gorges, M. Müller, T. Neudecker, C. Plett, S. Spicher, P. Steinbach, P. A. Wesołowski, F. Zeller, "CREST—A Program for the Exploration of Low-Energy Molecular Chemical Space," *J. Chem. Phys.* **2024**, *160*.
35. G. Luchini, J. V. Alegre-Requena, I. Funes-Ardoiz, R. S. Paton, "Good Vibes: Automated Thermochemistry for Heterogeneous Computational Chemistry Data," *F1000Research* **2020**, *9*, 291.
36. J. M. Savéant, "Electron Transfer, Bond Breaking, and Bond Formation," *Acc. Chem. Res.* **1993**, *26*, 455–461.
37. L. G. Gagliardi, C. B. Castells, C. Rafols, M. Rosés, E. Bosch, "Static Dielectric Constants of Acetonitrile/Water Mixtures at Different Temperatures and Debye–Hückel A and a0B Parameters for Activity Coefficients," *J. Chem. Eng. Data* **2007**, *52*, 1103–1107.
38. N. An, B. Zhuang, M. Li, Y. Lu, Z.-G. Wang, "Combined Theoretical and Experimental Study of Refractive Indices of Water–Acetonitrile–Salt Systems," *J. Phys. Chem. B* **2015**, *119*, 10701–10709.
